# Genetically predicted metabolites mediate the association between lipidome and malignant melanoma of skin

**DOI:** 10.3389/fonc.2024.1430533

**Published:** 2024-09-10

**Authors:** Yuzhou Wu, Hang Ma, Zhenyu Liu

**Affiliations:** ^1^ The First Clinical College of Chongqing Medical University, Chongqing, China; ^2^ Rheumatology and Immunology Department, The First Affiliated Hospital of Zhengzhou University, Zhengzhou, Henan, China; ^3^ Department of Urology, The First Affiliated Hospital of Chongqing Medical University, Chongqing, China

**Keywords:** malignant melanoma of skin, lipidome, Mendelian randomization, metabolites, mediator

## Abstract

**Objective:**

To investigate the causal relationship between lipidome and malignant melanoma of skin (MMOS), while identifying and quantifying the role of metabolites as potential mediators.

**Methods:**

A two-sample Mendelian randomization (MR) analysis of lipid species (n=7174) and MMOS was performed using pooled data from genome-wide association studies (GWAS). In addition, we quantified the proportion of metabolite-mediated lipidome effects on MMOS by two-step MR.

**Results:**

This study identified potential causal relationships between 11 lipids and MMOS, and 40 metabolites and MMOS, respectively. Phosphatidylethanolamine (18:0_18:2) levels mined from 179 lipids by MR Analysis increased the risk of MMOS (OR: 1.962; 95%CI:1.298,2.964; P=0.001). There is no strong evidence for a relationship between genetically predicted MMOS and phosphatidylethanolamine (18:0_18:2) levels (P=0.628). The proportion of gene predictions for phosphatidylethanolamine (18:0_18:2) levels mediated by 1-stearoyl-(glycosylphosphatidylinositol) GPI (18:0) levels was 12.40%.

**Conclusion:**

This study identifies 1-stearoyl-GPI (18:0) levels as a potential mediator that may mediate the causal relationship between phosphatidylethanolamine (18:0_18:2) levels and MMOS, This provides direction for the investigation of MMOS, but further research of other possible potential mediators is still needed.

## 1 Introduction

Cutaneous melanoma (CM) is a malignant tumor that originates from melanocytes in the skin. CM usually presents as a black or brown mole or nevus-like lesion that may extend into deeper tissues and other organs. Melanoma of the skin is usually caused by exposure to ultraviolet radiation from natural light and indoor tanning, and its incidence is increasing annually, especially in Caucasian populations (1). Epidemiological estimates based on global cancer data indicate that there were 325,000 new cases of CM and 57,000 melanoma-related deaths in 2020. Based on 2020 incidence estimates, the number of new melanoma cases and melanoma-related deaths worldwide will increase to 510,000 and 96,000, respectively, in 2040 (2). Currently, the main markers that are used for the diagnosis and detection of melanoma are S100, HMB-45, Melan A, MITF, SOX 10, microRNA, exosomes, and Melanoma-Inhibiting Activity (MIA) (3). Surgical excision is the treatment of choice for patients with newly diagnosed early-stage cutaneous melanoma, while immunotherapy, kinase inhibitors, and chemotherapy may be considered for patients with advanced disease (4).

It has been shown that adipocytes increase the invasion and proliferation of melanoma cells *in vivo* and *in vitro* and that adipocyte-derived lipids are transferred to melanoma cells via the FATP/SLC27A family of lipid transporter proteins that are expressed on the surface of tumor cells. Pharmacological blockade of the FATP protein with the small molecule lipofermata prevents lipid translocation into melanoma cells and reduces melanoma growth and invasion (5). Via quantitative lipid profiling of plasma samples from 151 patients with melanoma, a recent study revealed that the levels of free fatty acids (FFAs) and lactosylceramides (LCERs) were significantly decreased in patients with metastatic melanoma, demonstrating that plasma lipid profiles may be an important predictor of clinical outcome in patients with melanoma and may also be an indicator of patient survival (6). Hye-Youn Kim et al. identified potential biomarkers in melanoma cells with different metastatic abilities by metabolic and lipidomic analyses; the results revealed that aminomalonic acid may be a novel biomarker and that the enhanced metastatic potential of melanoma is accompanied by an increase in phosphatidylinositol (PI) species (7). These findings indicate that lipids and metabolites may be strongly associated with skin melanoma.

Some estimates indicate that there are hundreds of thousands of species of lipids (8). The 179 lipids that were included in this study were chosen based on a recent study by Ottensmann, L. et al. (9). Via univariate and multivariate genome-wide analyses, these authors revealed genetic links between diseases and lipids. Metabolites are products of metabolic reactions, and they are influenced by a variety of factors, including genetics, diet, gut microbes and a variety of diseases (10, 11). The 1400 metabolites that were included in this study were chosen based on Yiheng Chen et al. (12). Through a series of large-scale GWASs, these authors inferred causal relationships between metabolite levels and multiple traits or diseases. In this article, we explored the possible causal relationships among the lipidome, metabolites and malignant melanoma of skin using Mendelian randomization analysis, and we identified potential metabolites that may be useful for early diagnosis and therapeutic targeting based on previous studies.

## 2 Methods

### 2.1 Data sources

In this study, the GWAS participants were all of European ancestry. We obtained GWAS data on lipids from a recent study by Ottensmann L et al. (9) that included 771 Finnish individuals. The data can be obtained from http://ftp.ebi.ac.uk/pub/databases/gwas/summary_statistics/GCST90277001-GCST90278000/. Data of malignant melanoma of skin (MMOS) were obtained from FinnGen and included 98 patients and 218694 controls; these data can be obtained from https://gwas.mrcieu.ac.uk/. Genetic associations of plasma metabolites were obtained from the study by Yiheng Chen and colleagues; these data were from European populations and are available at http://ftp.ebi.ac.uk/pub/databases/gwas/summary_statistics/GCST90199001-GCST90200000/ (12).

### 2.2 Selection of instrumental variables and data harmonization

First, we selected SNPs as potential candidate IVs following the criterion of P<1×10^-5^ in a 10000 kb window. Additionally, independent SNPs needed to have a low correlation with other SNPs in the region (R^2^<0.001). Second, effect SNPs were extracted from the outcome GWAS dataset with the filtering criterion of MAF>0.01. Next, valid IVs were identified after harmonizing exposure and outcome effects and removing SNPs with F < 10 or failing to harmonize. To calculate the F value, we use the latest and most accurate method: *F* = R^2^(N-K-1)/K(1-R^2^). R^2^ is the cumulative variance of exposure, K is the total number of IVs, and N is the total number of samples. It is generally assumed that with an F-statistic >10, the correlation is strong enough to prevent weak instrument bias (13–15). In addition, we used PhenoScanner (http://www.phenoscanner.medschl.cam.ac.uk/) to search for relationships between IVs and phenotypes, and then removed IVs associated with confounders.

### 2.3 Statistical analysis

Effective IVs were assessed by MR using inverse variance weighted (IVW), weighted mode, simple mode (SM), weighted median, and MR−Egger regression, heterogeneity was assessed using Cochran’s Q and funnel plots, and pleiotropy was assessed using MR−Egger intercept analysis while visualizing the MR results (16, 17). All the statistical analyses for this study were performed in R v4.2.3 using the “TwoSampleMR”, “VariantAnnotation”, “gwasglue”, “ieugwasr”, “grid”, “readr”, “forestploter”, and “p.value” R packages.

### 2.4 Primary analysis and mediation analysis

**Figure 1:**
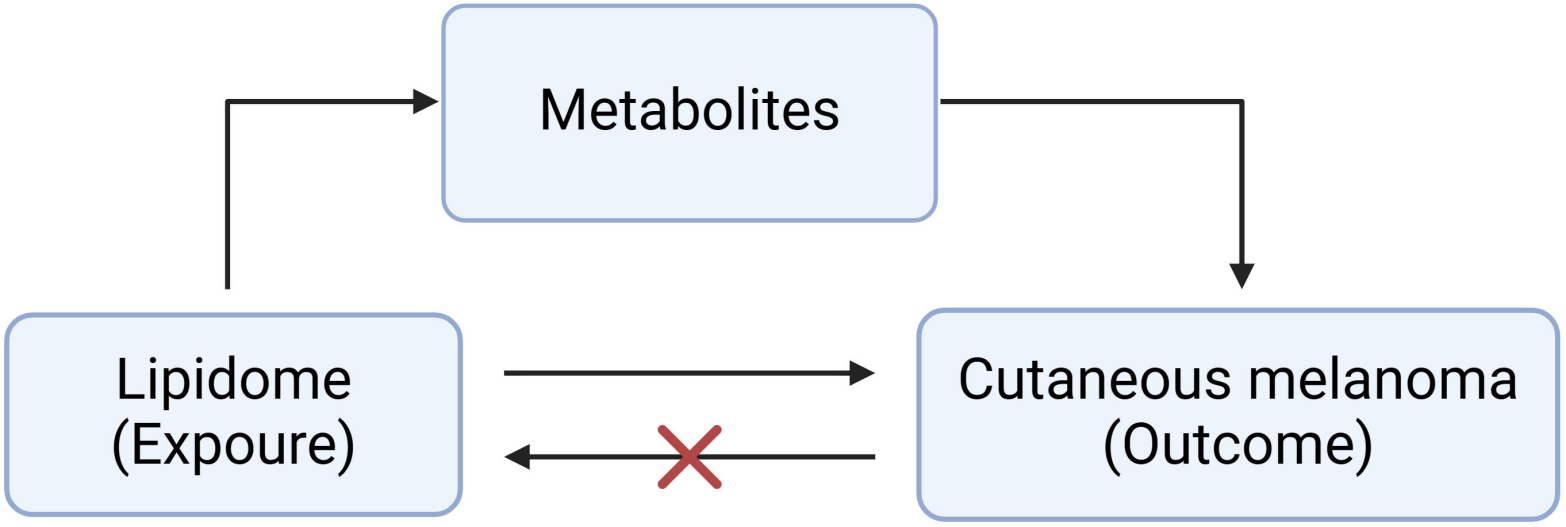
The basic principles of mediated Mendelian analysis.

**Figure 1** shows the basic principles of mediated Mendelian analysis. This study used one of the mediated Mendelian randomization methods: two-step MR. Two-step MR is also known as network MR, which is similar to the coefficient product method. It calculates two estimates of MR i) the causality of exposure on mediator and ii) the causality of mediator on outcome (18). Two-step MR also requires that there is no causal relationship of the mediator on exposure. Finally, multiplying these two estimates gives the indirect effect, and subtracting the indirect effect from the total effect gives the direct effect (19). We first explored the causal role of the 179 lipids on MMOS (**Supplementary Material 1**). Lipidomes associated with MMOS were screened based on a P value of the IVW method <0.01, a P value of pleiotropy >0.05, and the consistent direction of the ORs of the five methods. Finally, one eligible lipidome was obtained (**Supplementary Material 2**). Reverse MR was then performed with this lipidome as the outcome and the skin malignant melanoma as the exposure, and the results of the reverse MR showed that there was no causal relationship between MMOS and this lipid species; thus, it could be used for subsequent analysis (**Supplementary Material 3**). We then explored the causal relationships of 1400 metabolites with MMOS (**Supplementary Material 4**) and screened for metabolites that were associated with the disease based on a P value of the IVW method <0.001, a P value of pleiotropy >0.05, and the consistent direction of the ORs of the five methods (**Supplementary Material 5**).

We further explored whether metabolites mediate the causal pathway from the lipidome to MMOS by two-step MR. The beta value for the causality of lipidome on metabolites was defined as *beta 1*. The beta value for the causality of metabolites on MMOS was defined as *beta 2*. The beta value for the causality of lipidome on MMOS was defined as the *beta ALL*. The mediating effect is equal to beta1*beta2. The direct effect is equal to the total effect minus the mediating effect (20).

## 3 Results

We performed a two-sample Mendelian randomization analysis between 179 lipids and MMOS and found a causal relationship between 11 lipids species and MMOS. The results of the Mendelian randomization analysis of the 179 lipids and MMOS are presented in **Supplementary Material 1**, their heterogeneity and pleiotropy results are presented in **Supplementary Materials 6** and **7**, respectively, and the forest plots, scatter plots, funnel plots, and leave-one-out analysis plots of the MR results are presented in **Supplementary Materials 8–11**, respectively, all of which indicate that the 11 lipids and MMOS have a causality is robust. Then, we analyzed 1 lipidome to assess its causal effects on MMOS based on the conditions mentioned above (**Supplementary Material 2**). Subsequent reverse MR analysis was performed, and the lipidome was preserved for subsequent analysis (**Supplementary Material 3**).

We also performed a two-sample Mendelian randomization analysis between 1400 metabolites and MMOS and found a potential causal relationship between 40 metabolites and MMOS. The results of the Mendelian randomization analysis of the 1400 metabolites and MMOS are presented in **Supplementary Material 4**, and their heterogeneity and pleiotropy results are presented in **Supplementary Materials 12** and **13**, suggesting that the causal relationship between these 40 metabolites and MMOS is robust. We also screened 10 metabolites that were associated with MMOS for subsequent analysis based on a P value of the IVW method <0.001, a P value of pleiotropy >0.05, and the consistent direction of the ORs of the five methods (**Supplementary Material 5**).

### 3.1 Association of the lipidome with metabolites

**Figure 2:**
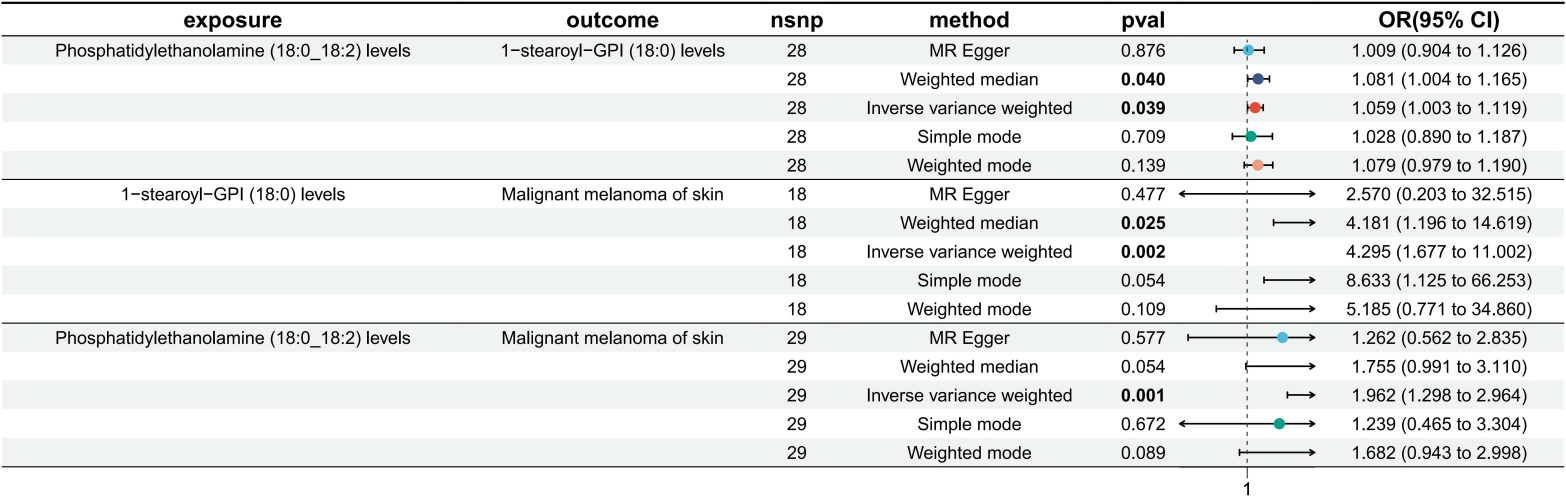
MR results of phosphatidylethanolamine (18:0_18:2) levels on 1-stearoyl-GPI (18:0) levels, 1-stearoyl-GPI (18:0) levels on MMOS and phosphatidylethanolamine (18:0_18:2) levels on MMOS.

Phosphatidylethanolamine (18:0_18:2) levels were causally associated with only 1 metabolite (1-stearoyl-GPI (18:0) levels) in 10 metabolites. A total of 28 SNPs were included in the MR analysis: IVW OR: 1.059, 95% CI: [1.003, 1.119], *P* = 0.039; MR−Egger OR: 1.001, 95% CI: [0.904, 1.126], P = 0.876; weighted mean OR: 1.081, 95% CI: [1.004, 1.165], *P* = 0.040; SM OR: 1.028, 95% CI: [0.890, 1.187], P=0.709; weighted mode OR: 1.079, 95% CI: [0.979, 1.190], P=0.139 (**Figure 2**; **Supplementary Material 14**). Although the three methods other than IVW and weighted median did not show statistical significance, their ORs were in the same direction, while neither heterogeneity nor pleiotropy was statistically significant; thus, it can be concluded that there is a causal relationship between phosphatidylethanolamine (18:0_18:2) levels and 1-stearoyl-GPI (18:0) levels. **Supplementary Material 15** shows the scatterplot, forest plot, funnel plot, and leave-one-out analysis of this MR analysis to demonstrate the stability of the results.

### 3.2 Association of metabolites with MMOS

Among 1400 metabolites, we analyzed whether there was a causal relationship between 1-stearoyl-GPI (18:0) levels and MMOS (**Supplementary Material 5**). A total of 18 SNPs were included in the study, and the IVW method was used as the primary method of analysis. The OR for IVW was 4.295 (95% CI [1.677, 11.002], P=0.002). The results of the remaining four methods were as follows: MR−Egger OR: 2.570, 95% CI: [0.203, 32.515], P = 0.477; weighted mean OR: 4.181, 95% CI: [1.196, 14.619], P = 0.025; SM OR: 8.633, 95% CI: [1.125, 66.253], P=0.054; weighted mode OR: 5.185, 95% CI: [0.771, 34.860], P=0.109 (**Figure 2**; **Supplementary Material 16**). The directions of the ORs for all five methods were consistent, and the Pvalue for IVW was <0.05. Moreover, neither heterogeneity nor multiplicity showed statistical significance (**Supplementary Material 16**). The visualization of the MR analysis of the effect of 1-stearoyl-GPI (18:0) levels on MMOS is shown in **Supplementary Material 17**, and the results once again demonstrate the robustness of the results. Additionally, we believe that the level of 1-stearoyl-GPI (18:0) is a potential risk factor for MMOS.

### 3.3 Association of the lipidome with MMOS

Based on the screening criteria mentioned above, we identified 1 lipidome with a potential causal relationship with MMOS. Therefore, we again performed an MR analysis of phosphatidylethanolamine (18:0_18:2) levels on MMOS to determine the total effect. MR analysis revealed that the phosphatidylethanolamine (18:0_18:2) level is a risk factor for MMOS, with an OR of 1.962 for the IVW approach (95% CI: 1.298, 2.964; P=0.001). The results of the remaining four methods did not show statistical significance (**Figure 2**; **Supplementary Material 18**). Neither heterogeneity nor pleiotropy was statistically significant (**Supplementary Material 18**). Scatterplots, forest plots, funnel plots, and leave-one-out analyses all showed that the results were stable (**Supplementary Material 11**).

### 3.4 Proportion of the association between the lipidome and MMOS mediated by metabolites

**Figure 3:**
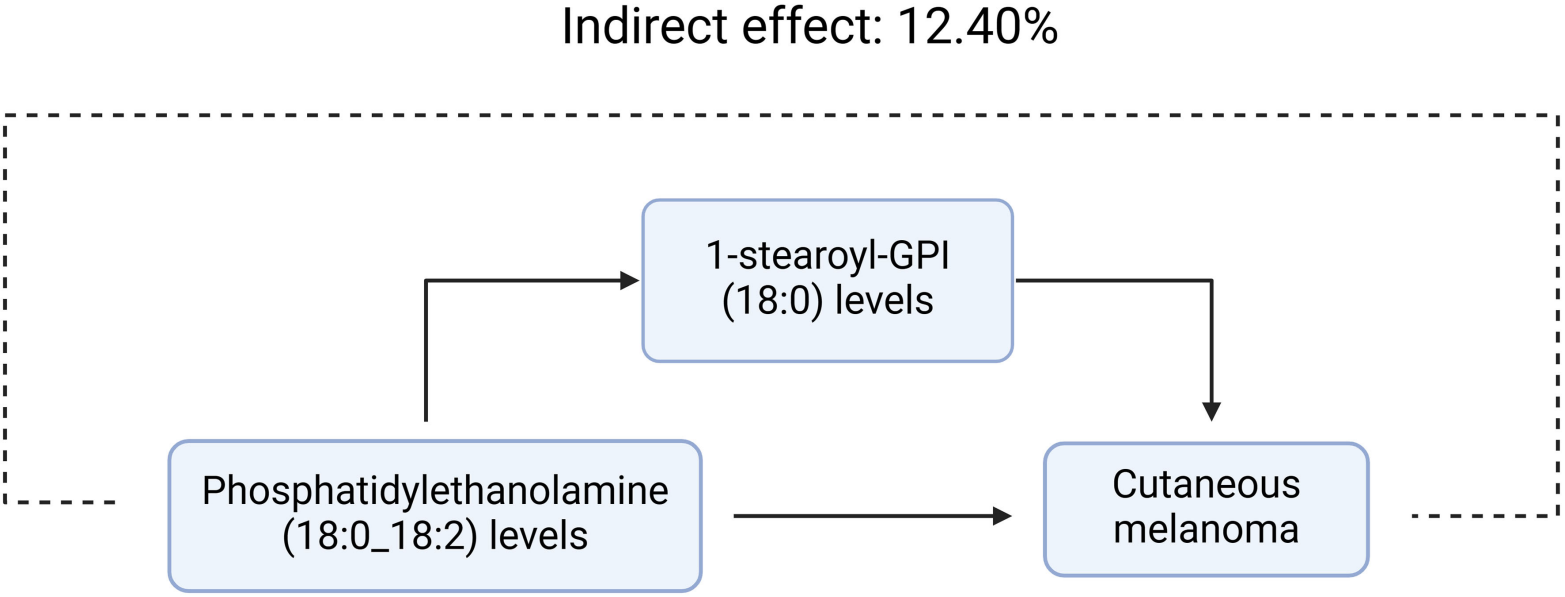
1-stearoyl-GPI (18:0) levels accounted for 12.40% of the elevated risk of MMOS associated with phosphatidylethanolamine (18:0_18:2)

We analyzed the levels of 1-stearoyl-GPI (18:0), which is a mediator of the pathway from phosphatidylethanolamine (18:0_18:2) to MMOS. We found that increased phosphatidylethanolamine (18:0_18:2) levels were associated with elevated 1-stearoyl-GPI (18:0) levels, which in turn were associated with an increased risk of melanoma. As shown in **Figure 3**, our study demonstrated that 1-stearoyl-GPI (18:0) levels accounted for 12.40% of the elevated risk of MMOS associated with phosphatidylethanolamine (18:0_18:2) (P<0.05).

## 4 Discussion

CM is one of the most aggressive and deadly types of skin cancer, and its global incidence rates continue to rise, placing a heavy burden on healthcare systems worldwide (21). It has been shown that there is a relationship between lipids and melanoma (5, 22, 23). Additionally, a Mendelian randomization study showed no association between polyunsaturated fatty acids (PUFAs) and increased or decreased risk of melanoma (24). However, these studies on lipids and melanoma are insufficient because they did not consider the large number of lipids that exist. Therefore, the aim of this study was to use a new statistical method, namely, Mendelian randomization, in combination with the current up-to-date lipid GWAS database to illustrate the causal relationship between 179 lipids and CM and to demonstrate whether the relationship is mediated through a certain metabolite. Our findings revealed a potential association between phosphatidylethanolamine (18:0_18:2) levels and an elevated risk of CM, 12.40% of which was mediated through 1-stearoyl-GPI (18:0) levels.

To date, we are the first to explore the causal relationship between metabolite-mediated lipids and CM through a mediated Mendelian approach. It was also demonstrated that 1-stearoyl-GPI (18:0) levels mediate this effect. A study by John S Fletcher et al. revealed that increased phosphatidylethanolamine in the membranes of cancer cells, possibly due to structural changes in the cytoskeleton of proteins, may also have effects on cancer, such as by increasing membrane fluidity and increasing motility (25). In addition, the cholesterol/phospholipid ratio, arachidonic acid content, and polyunsaturated fatty acid content of the membranes of highly metastatic B16-F10 melanoma cells were lower than those of B16-F1 melanoma cells, and the phosphatidylcholine/phosphatidylethanolamine ratio was higher than that of B16-F1 melanoma cell membranes; these results indicate that the membrane lipid composition of B16-F10 melanoma cells is different from that of B16-F1 melanoma cells, and this may help to explain the molecular basis for the different metastatic properties of these cell lines *in vivo* (26). CM can colonize and metastasize remotely, and these processes are largely based on lipid-based cell membrane scaffolds. Thus, Arantza Perez-Valle and his colleagues identified 32 lipid biomarkers of melanoma that are associated with benign-malignant transformation by UHPLC-mass spectrometry of human melanocytic cells; these biomarkers included phosphatidyl ethanolamine, phosphatidylcholine, phosphatidylinositol, and phosphatidylglycerol (23).

Raf kinase inhibitor protein (RKIP) is a member of the phosphatidylethanolamine-binding protein (PEBP) family and is an inhibitor of the Raf/MEK/ERK signaling pathway (27, 28). PEBP has sites for binding phospholipids, morphine and nucleotides, and it has been demonstrated that phosphatidylethanolamine is a specific ligand for PEBP, but the affinity between the two is low (29–32). RKIP expression has been associated with a variety of cancers, such as prostate cancer (33), hepatocellular carcinoma (34), breast cancer (35), and melanoma (36). Downregulation of RKIP leads to activation of the Ras/Raf/ERK/MRK signaling pathway, causing malignant transformation of melanocytes and contributing to migration and invasion of melanoma cells (37).

In this study, we found that 1-stearoyl-GPI (18:0) levels mediated the causal relationship between phosphatidylethanolamine (18:0_18:2) levels and MMOS. However, our study has several limitations. First, we included only European populations in our study, making this study much less generalizable. Second, we used looser thresholds and p values, which increases the risk of false positives. Third, we used summary-level statistics rather than individual-level data in our study. As a result, we were unable to further explore causal relationships between subgroups such as sex, race, etc. Fourth, our findings suggest that 1-stearoyl-GPI (18:0) levels mediated a genetic prediction rate of 12.40%, although this difference was statistically significant and relatively low. Therefore, additional subsequent studies are needed to quantify other mediators. Fifth, the types of lipids in our chosen dataset remain small compared to those that were identified. Finally, we only explored the causal relationship between lipids and MMOS, and we did not categorize the disease according to skin location or further explore other melanoma subtypes.

## 5 Conclusion

In conclusion, this study identified a causal relationship between phosphatidylethanolamine (18:0_18:2) levels and MMOS and identified the 1-stearoyl-GPI (18:0) level as a potential mediator that may mediate this relationship. This study provides a direction for researchers to explore mechanisms underlying melanoma; however, most of the effects of phosphatidylethanolamine (18:0_18:2) levels on melanoma remain unclear, and further research into other possible potential mediators is needed.

## Data Availability

The original contributions presented in the study are included in the article/**Supplementary Material**. Further inquiries can be directed to the corresponding author/s.
