# Supplementary figures and images for: Genetically predicted metabolites mediate the association between lipidome and malignant melanoma of skin
Source: Front Oncol. 2024 Sep 10;14:1430533. doi: 10.3389/fonc.2024.1430533 (PMC11419955; doi:10.3389/fonc.2024.1430533)

Supplementary  
material 8

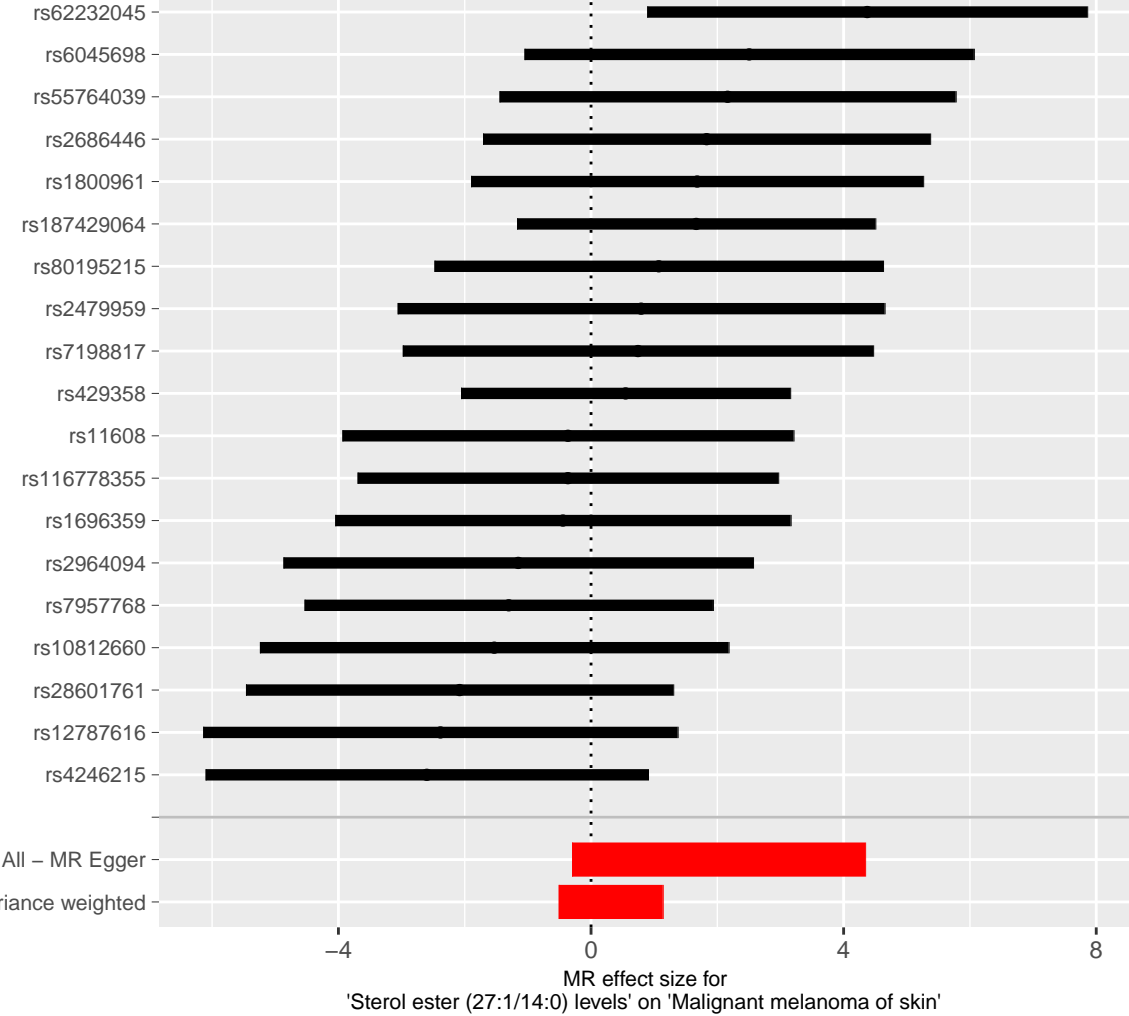

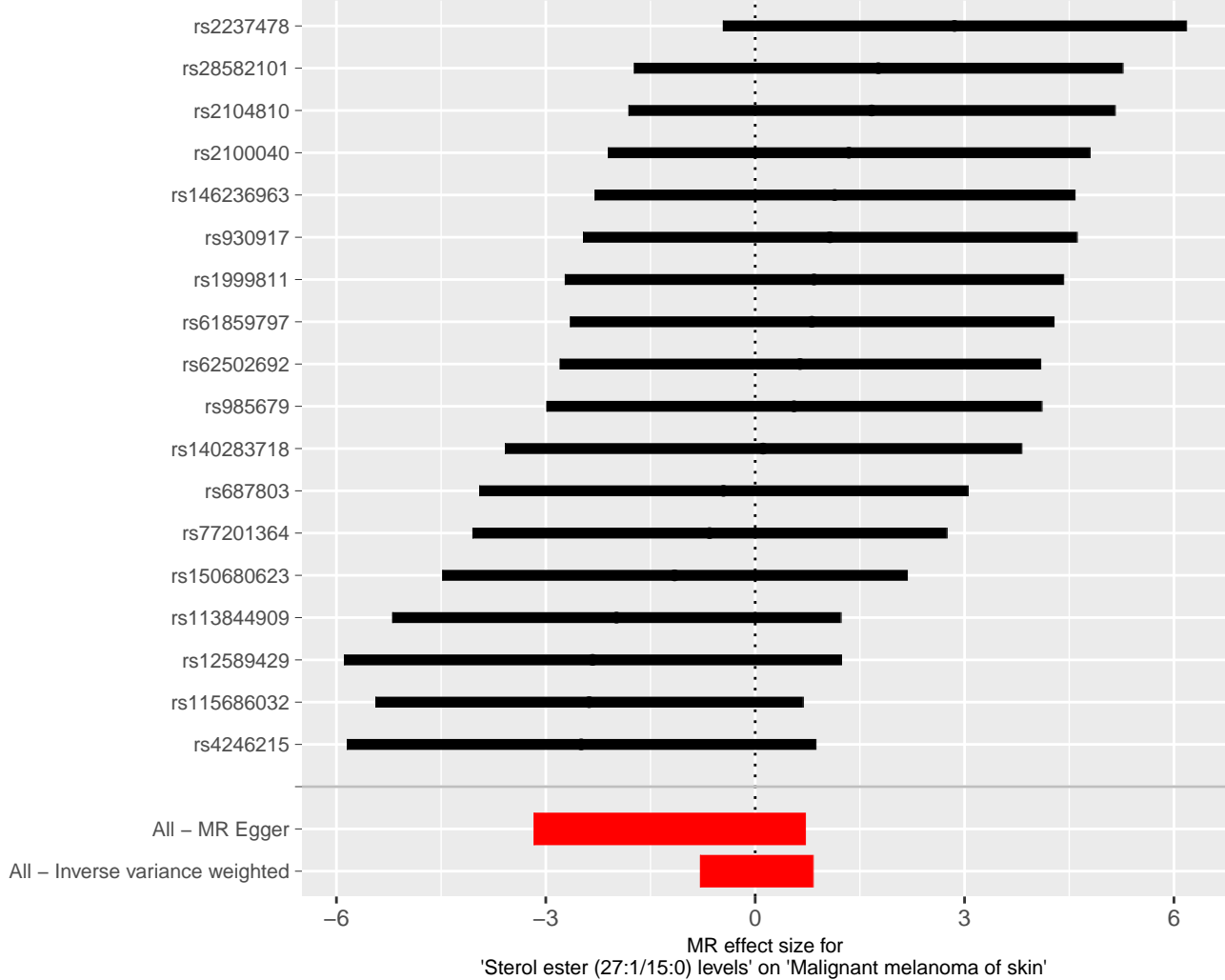

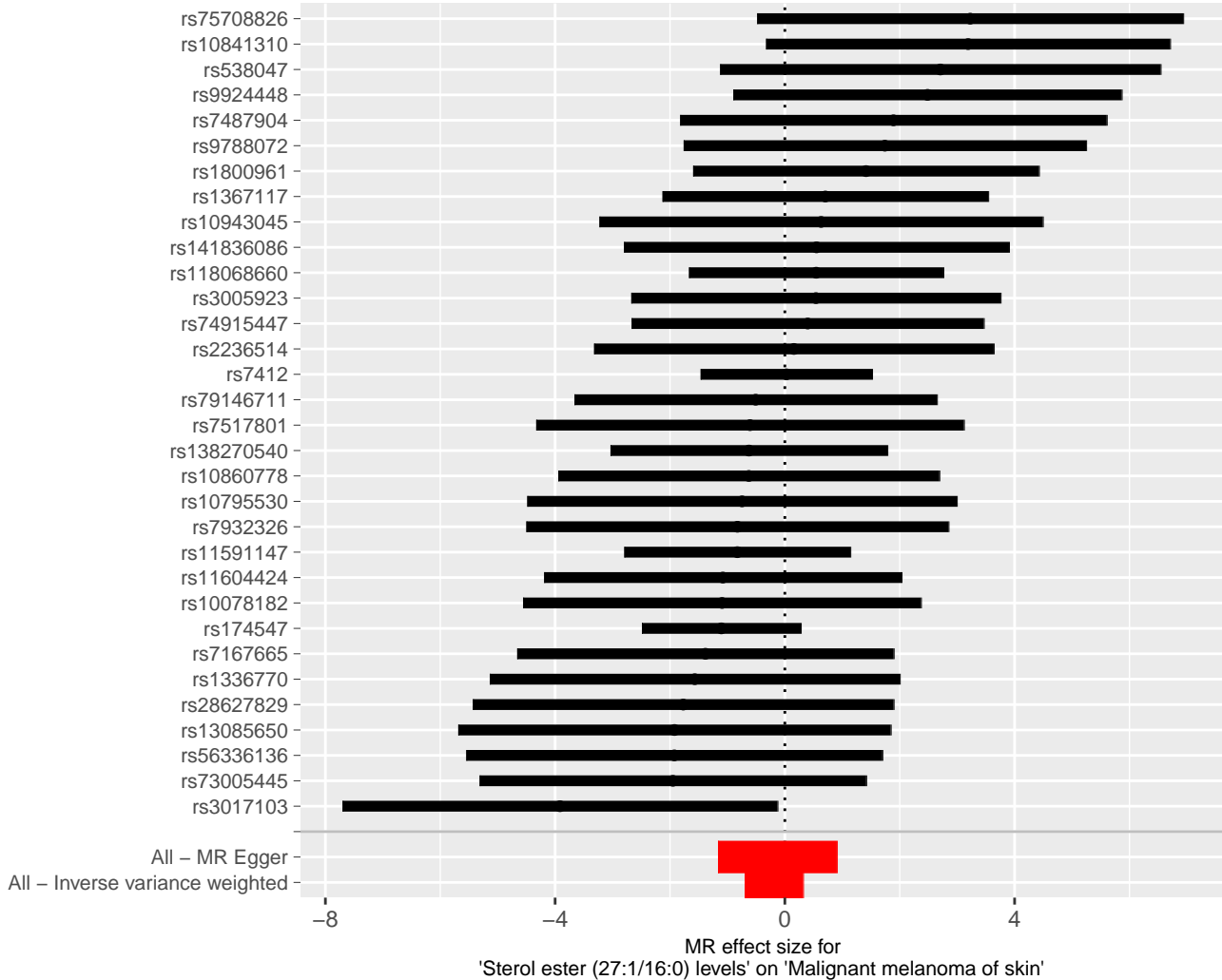

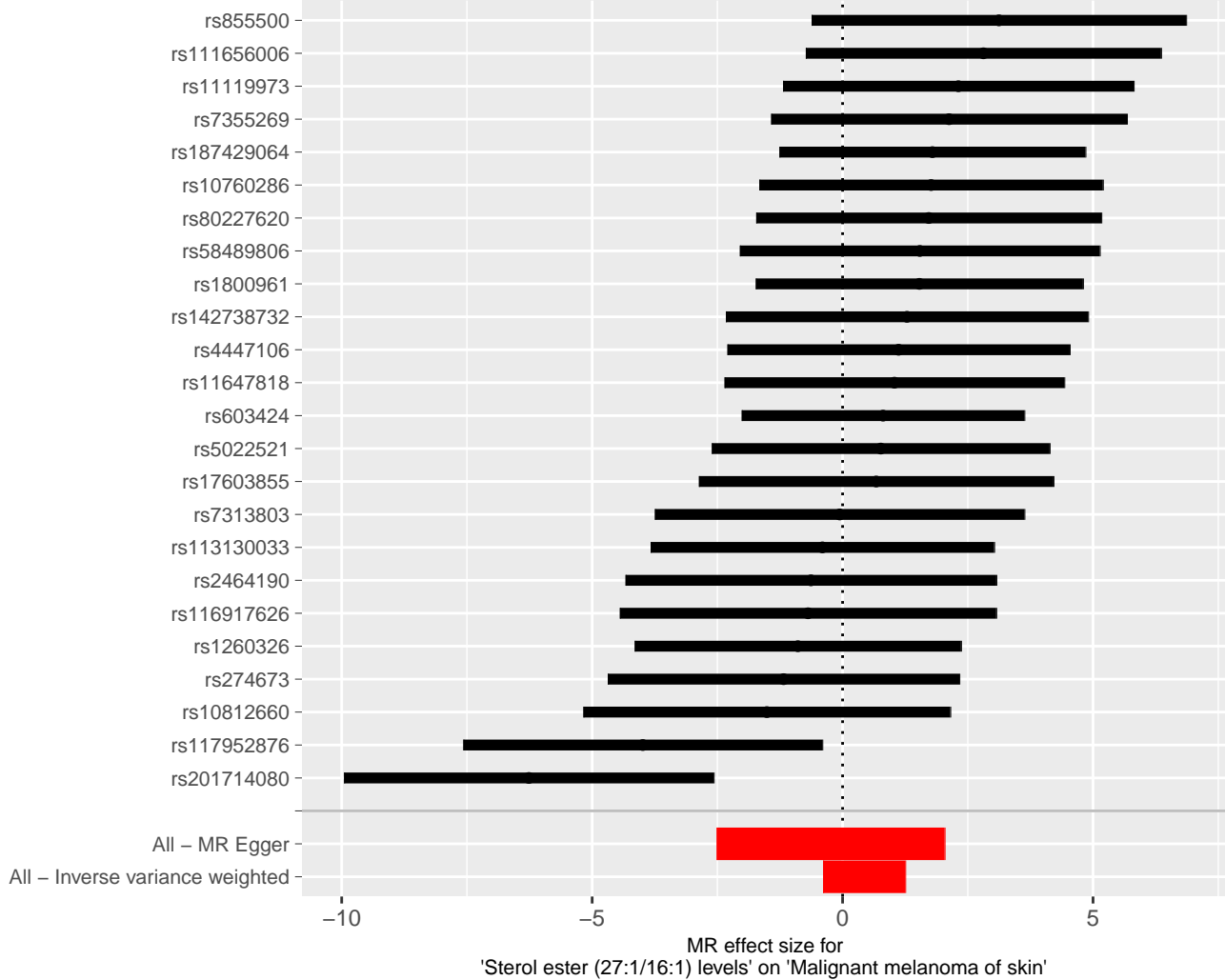

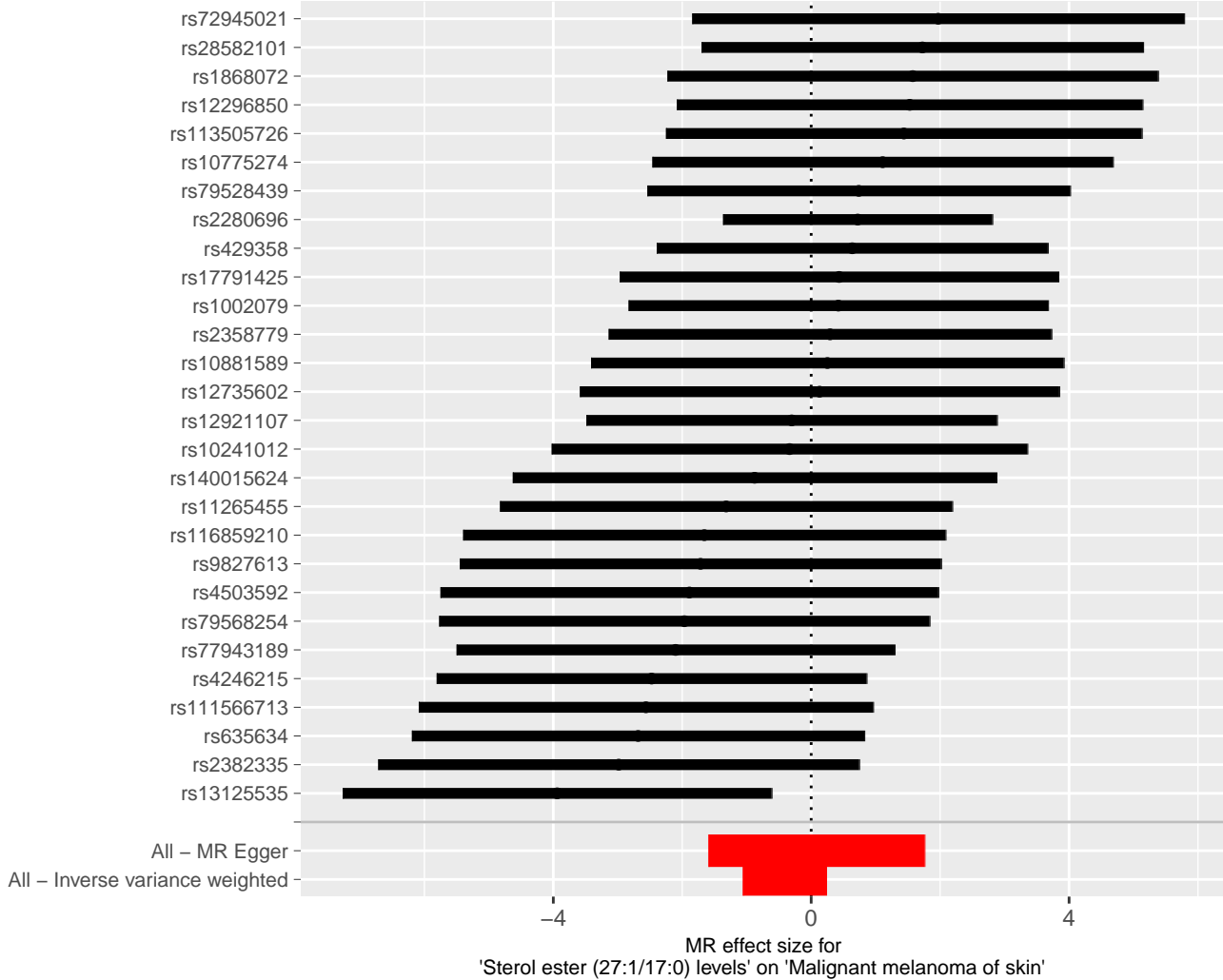

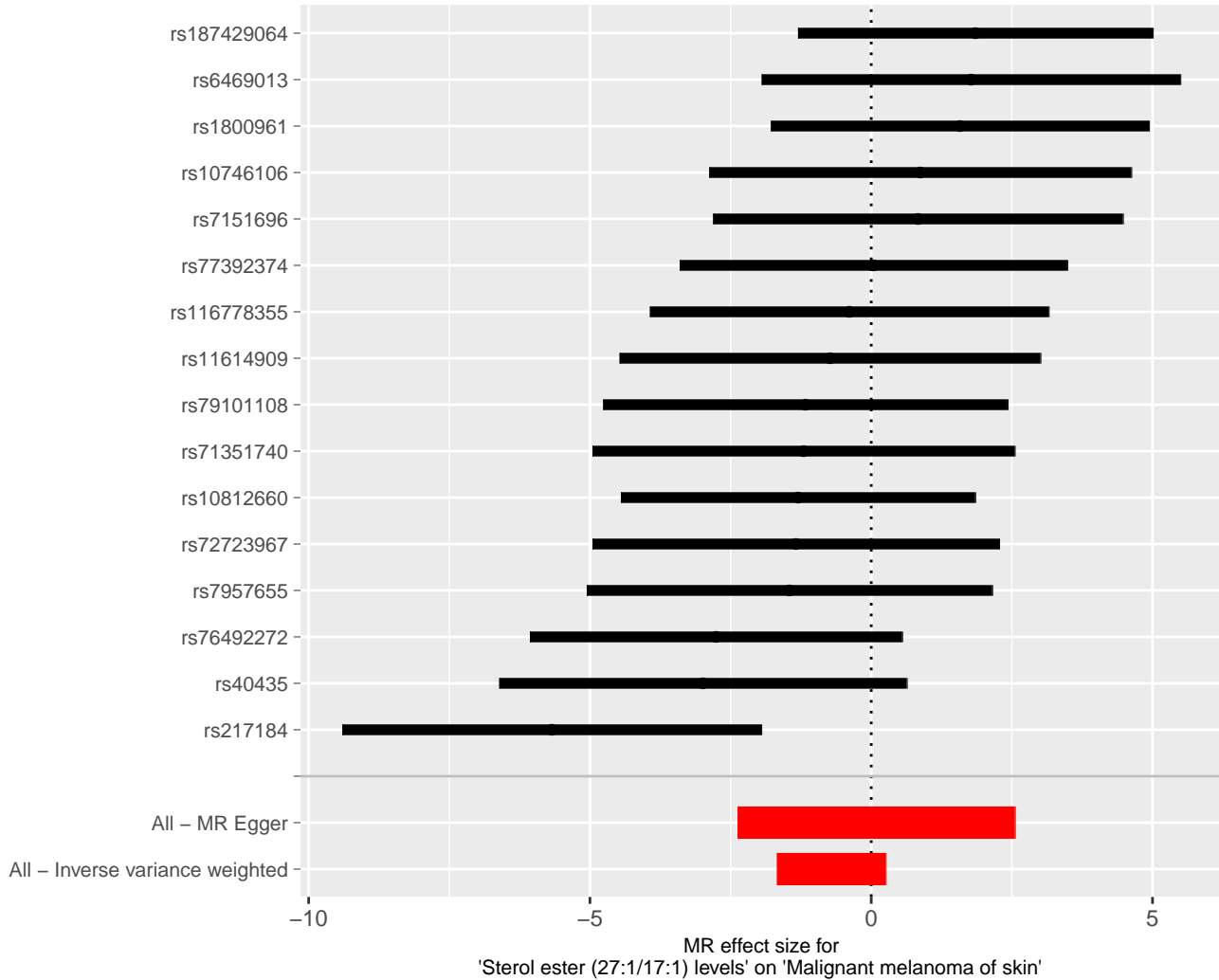

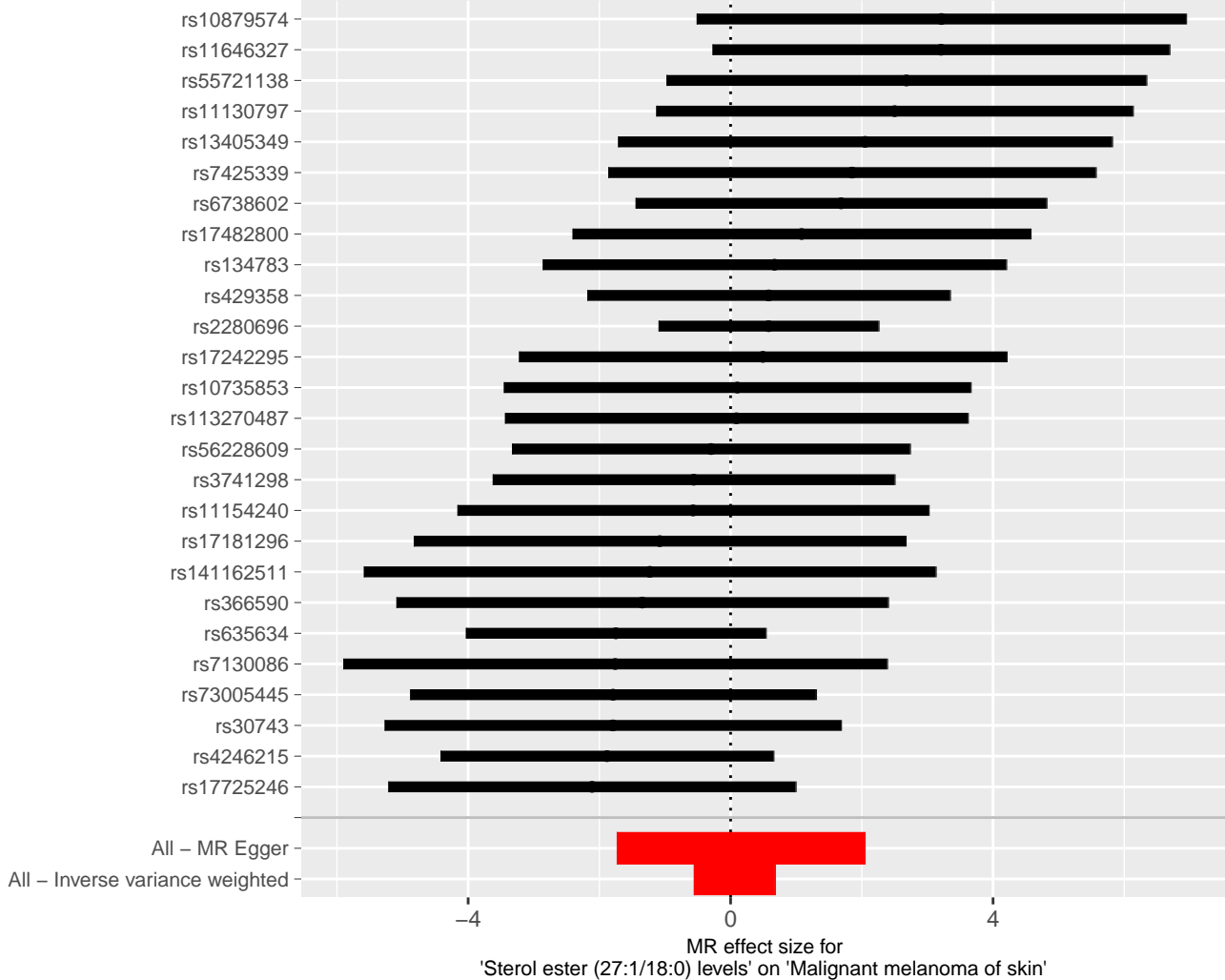

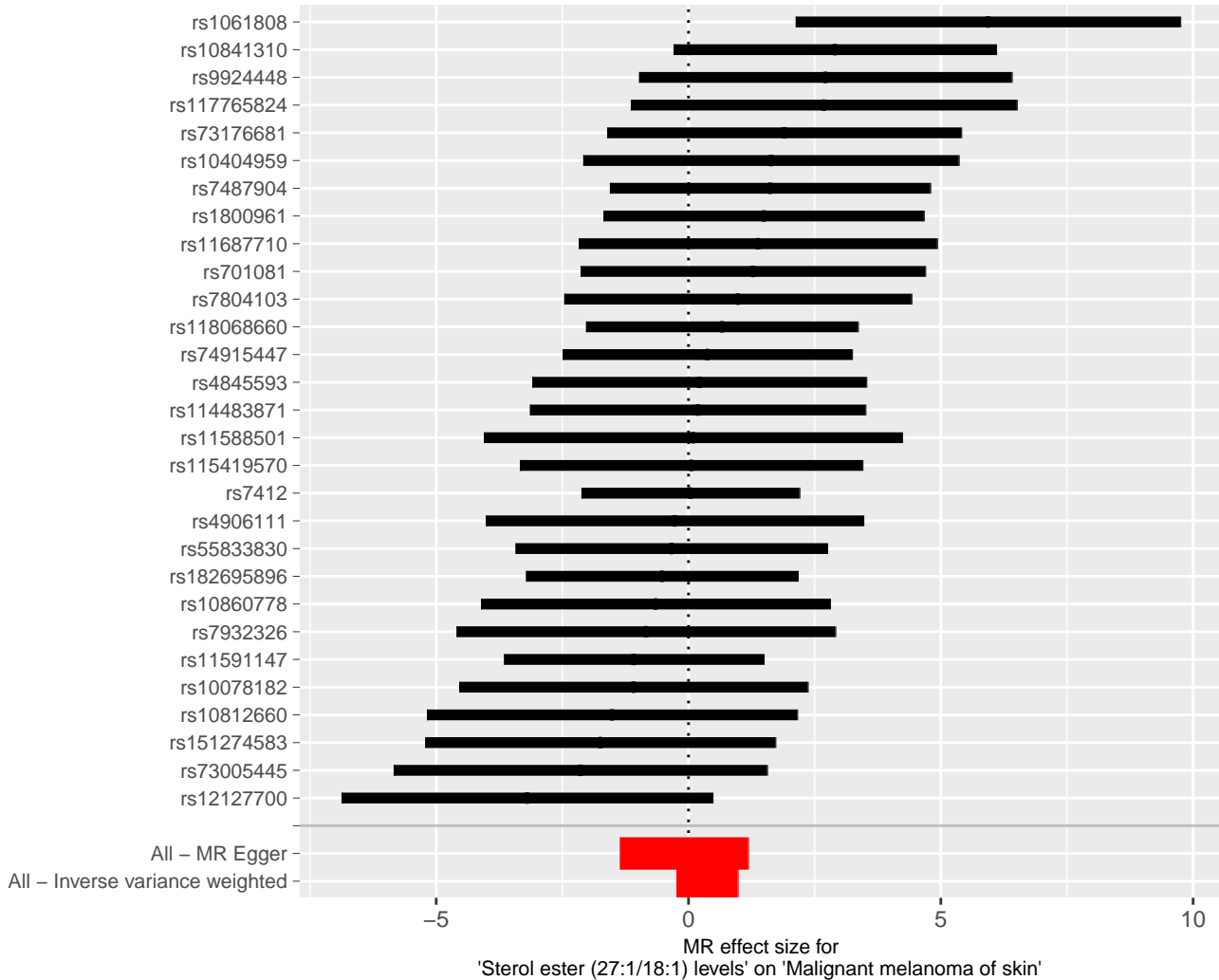

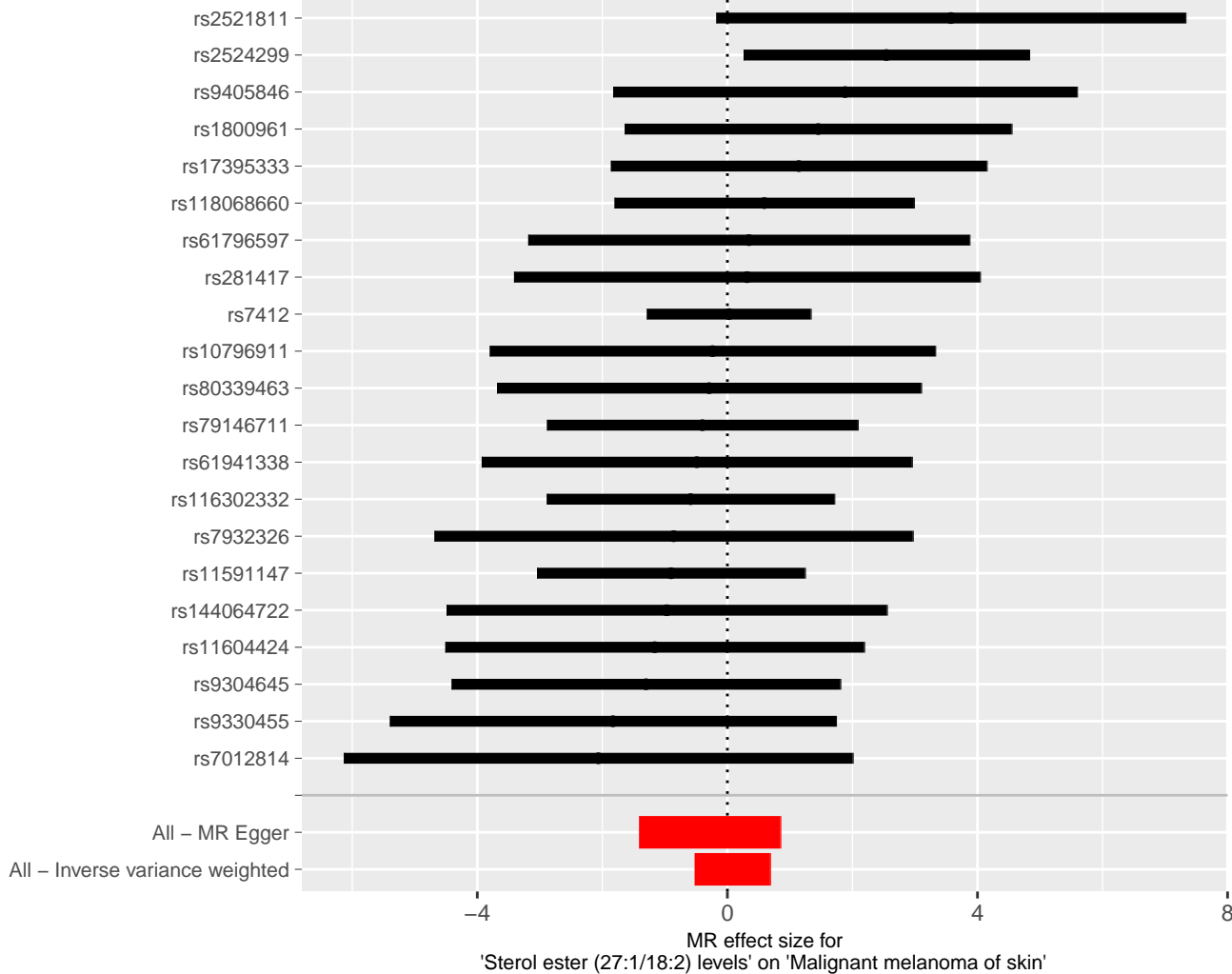

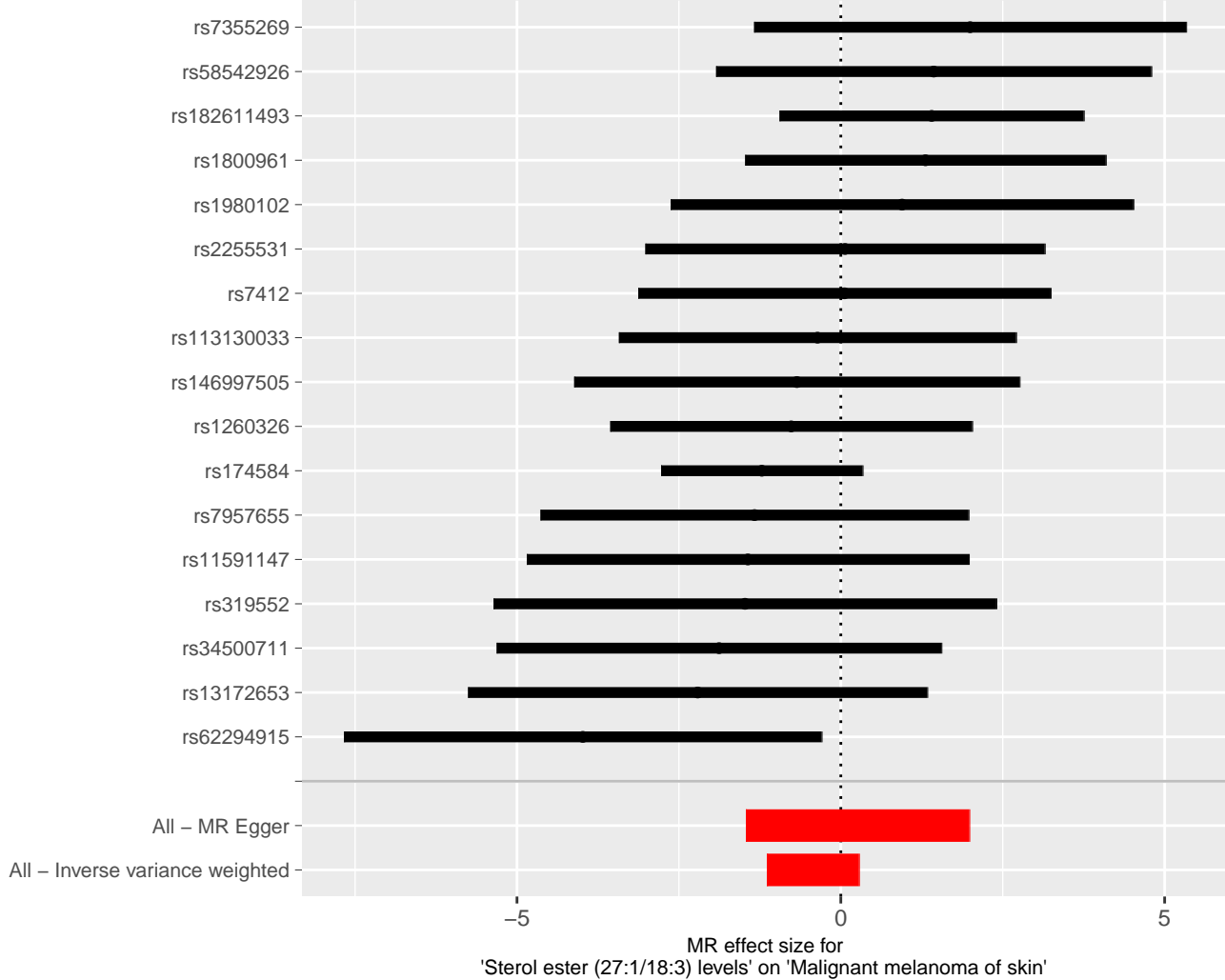

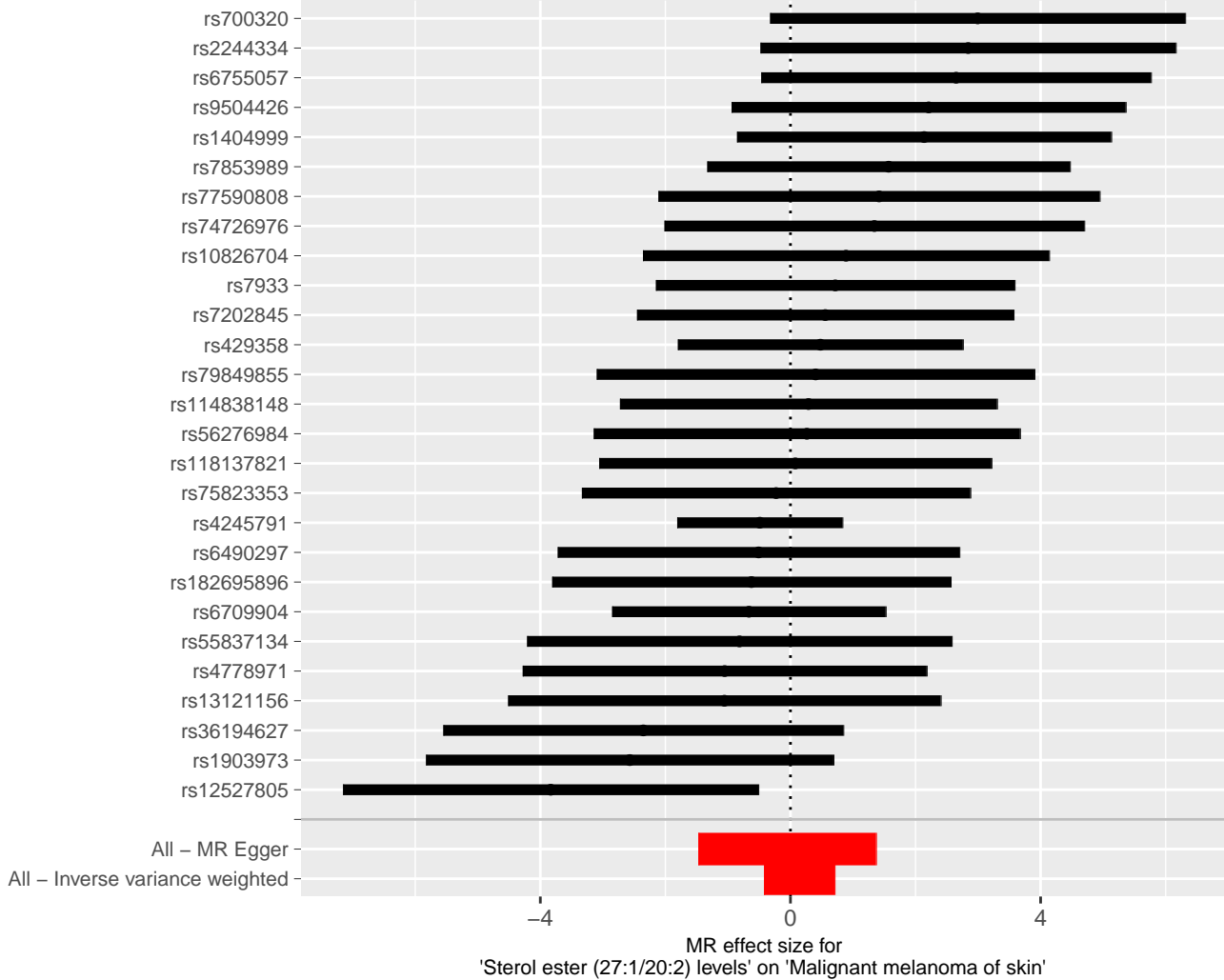

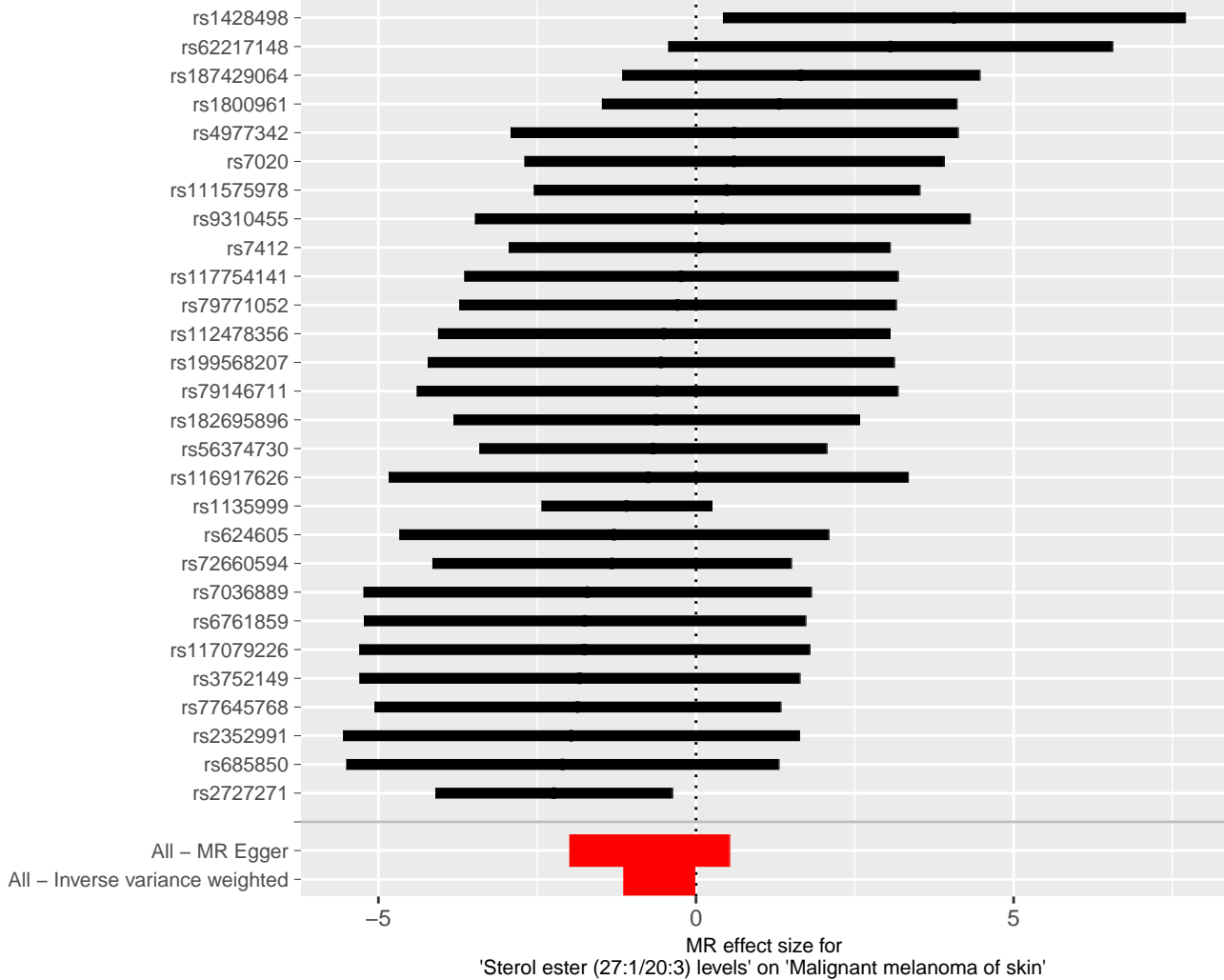

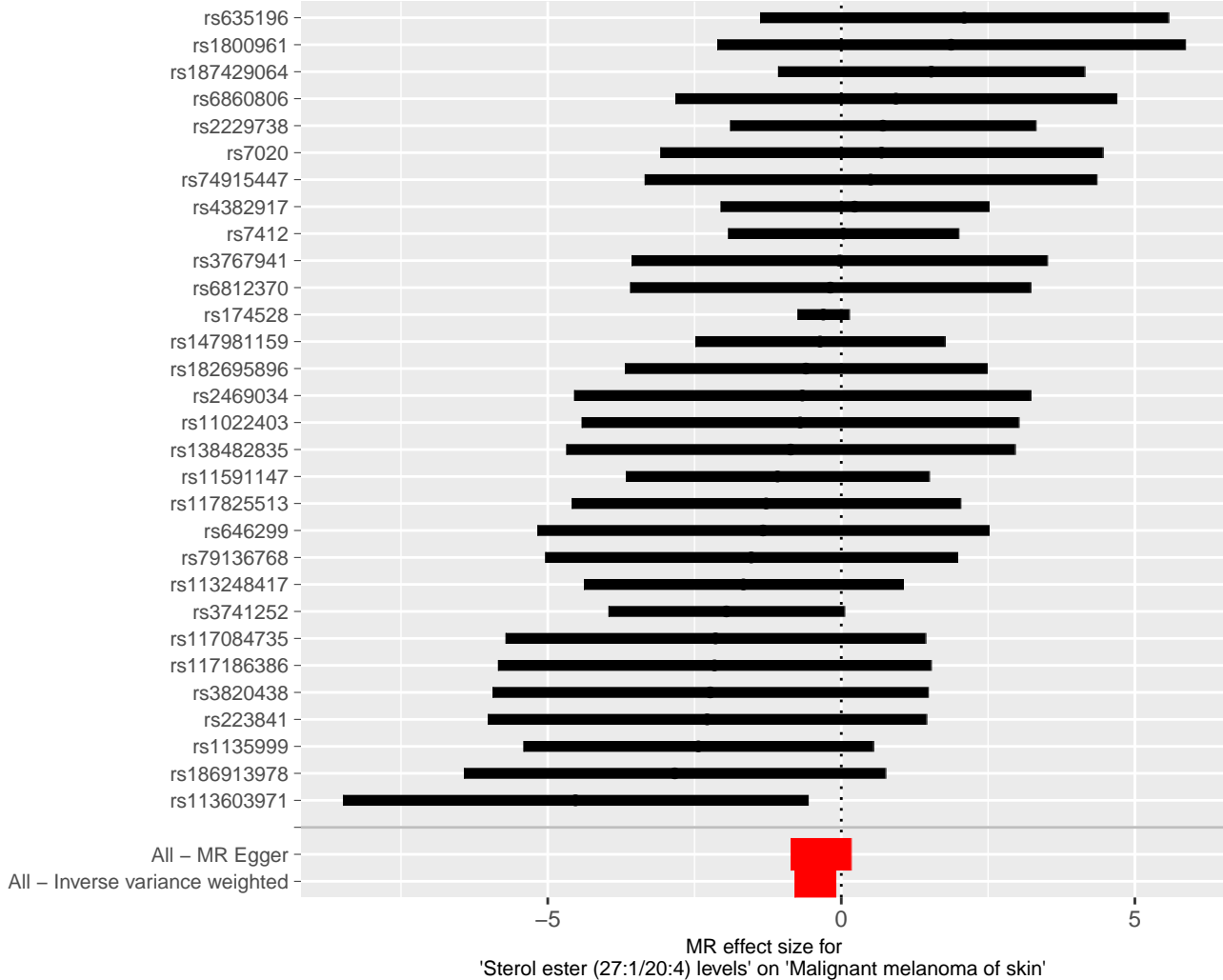

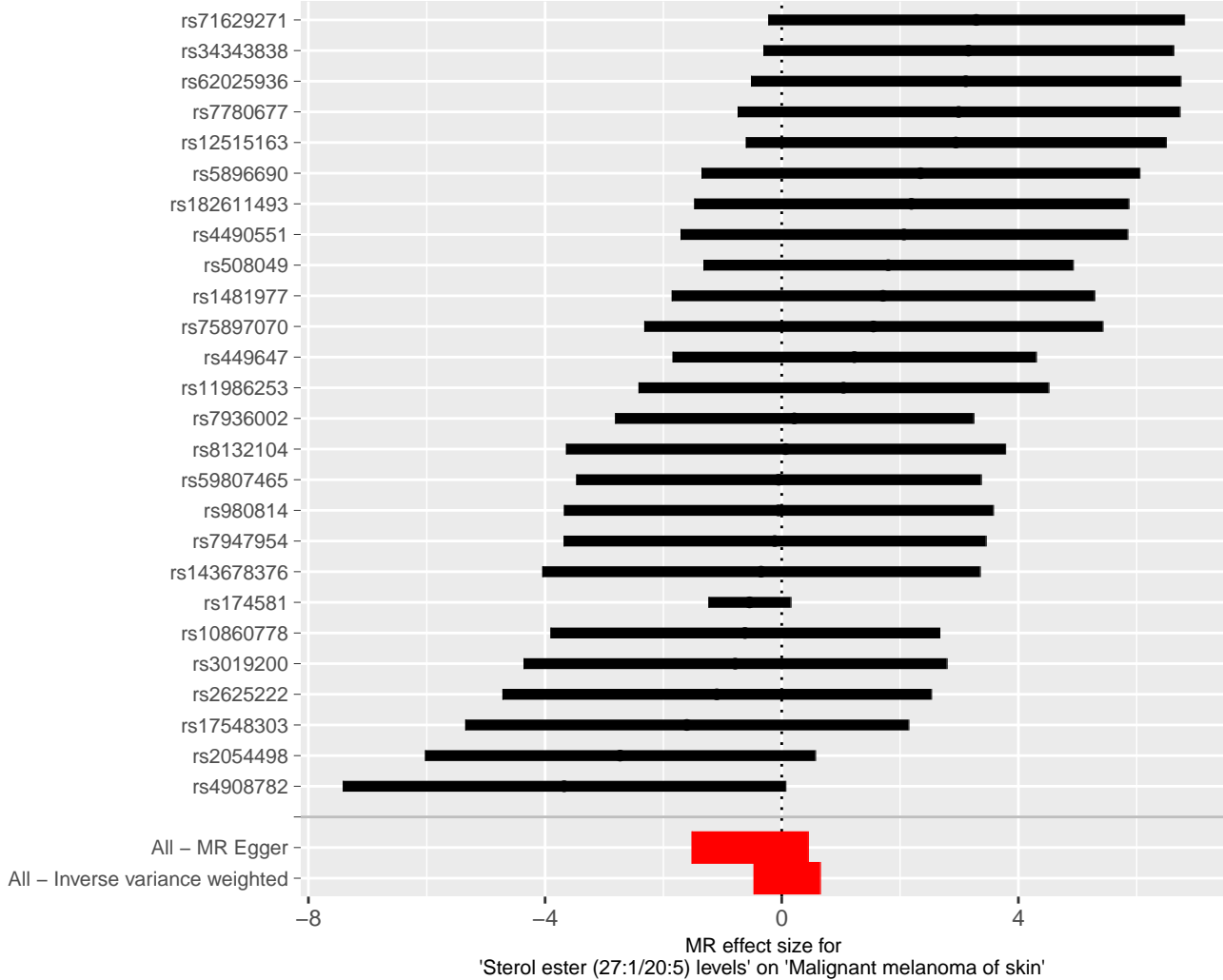

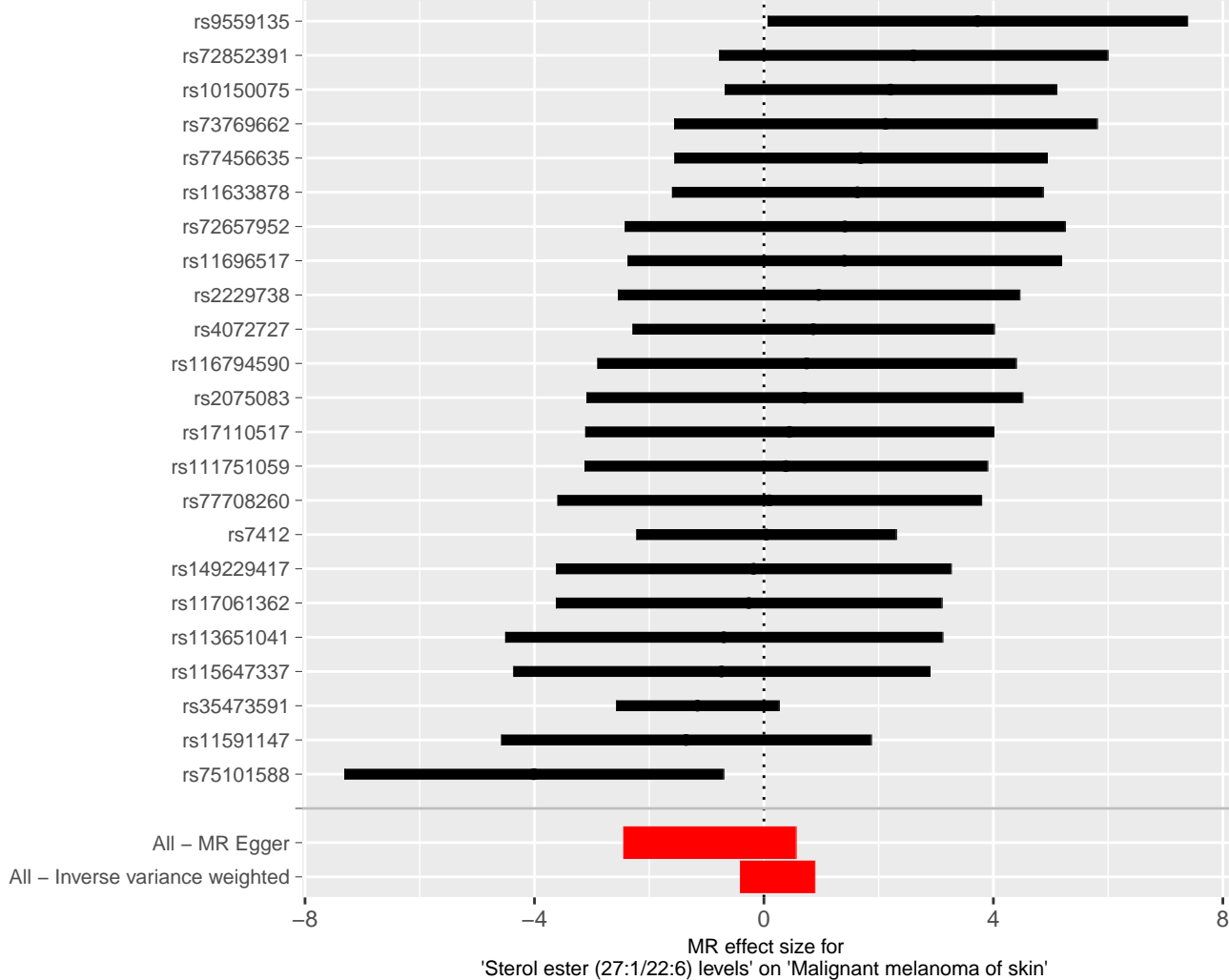

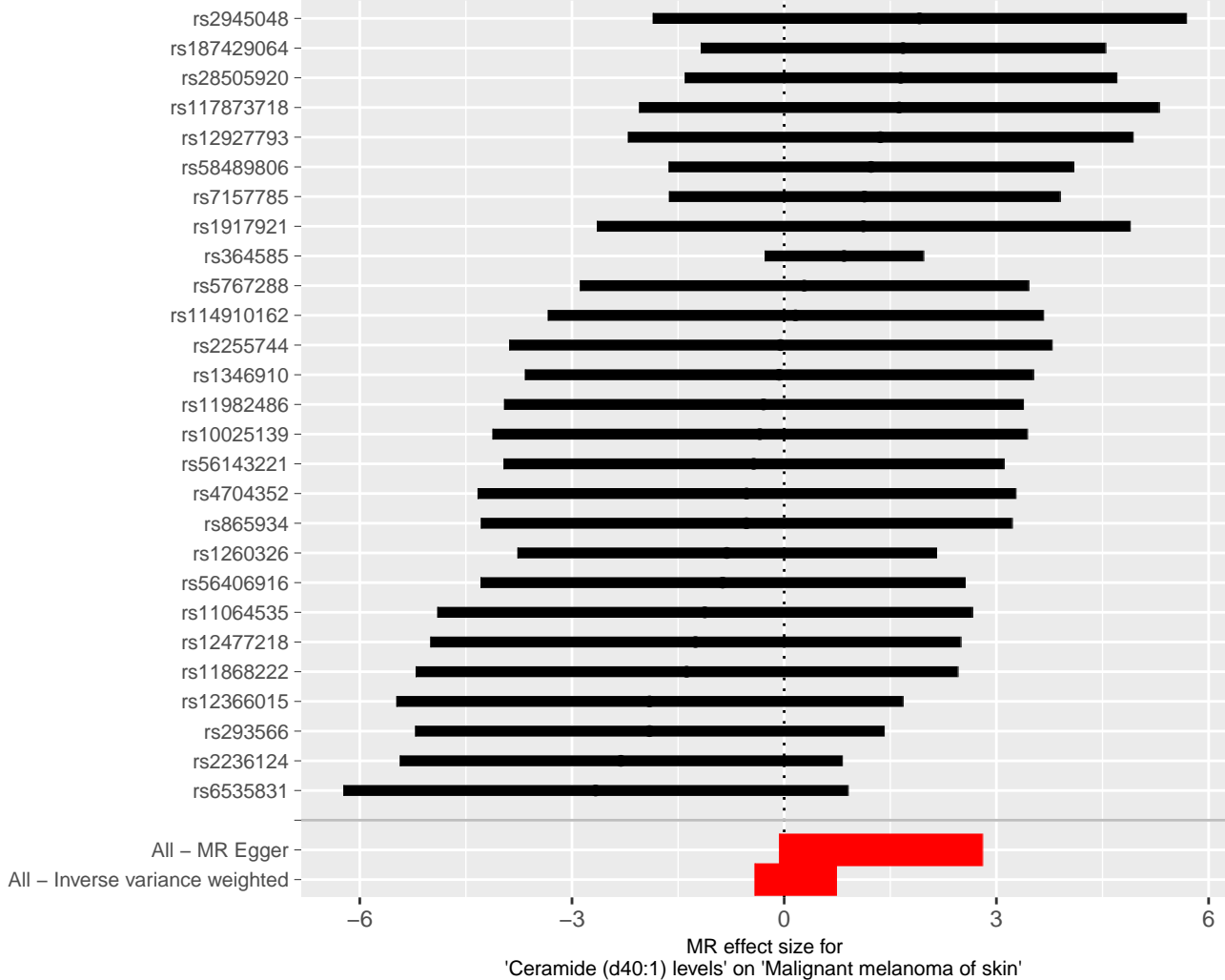

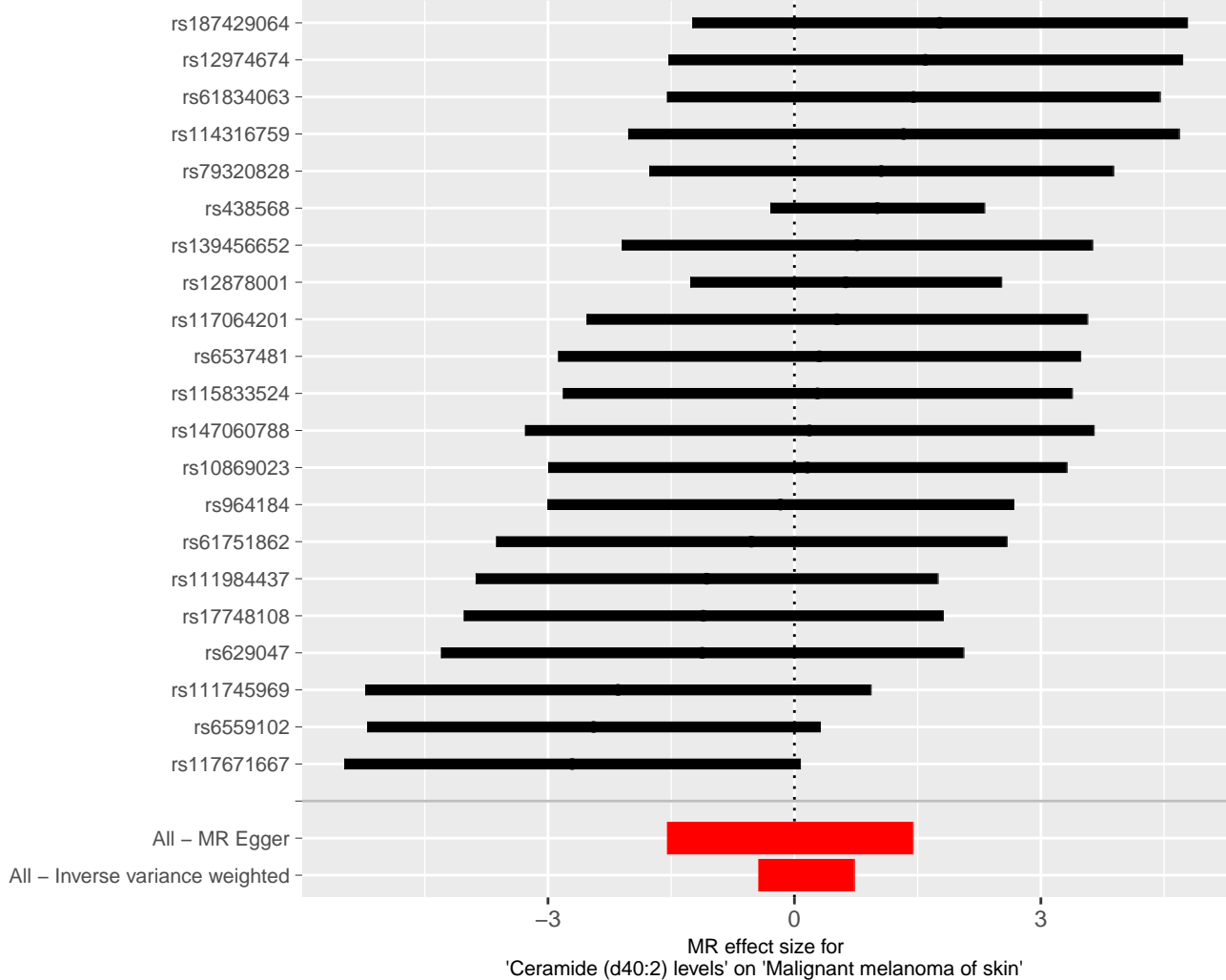

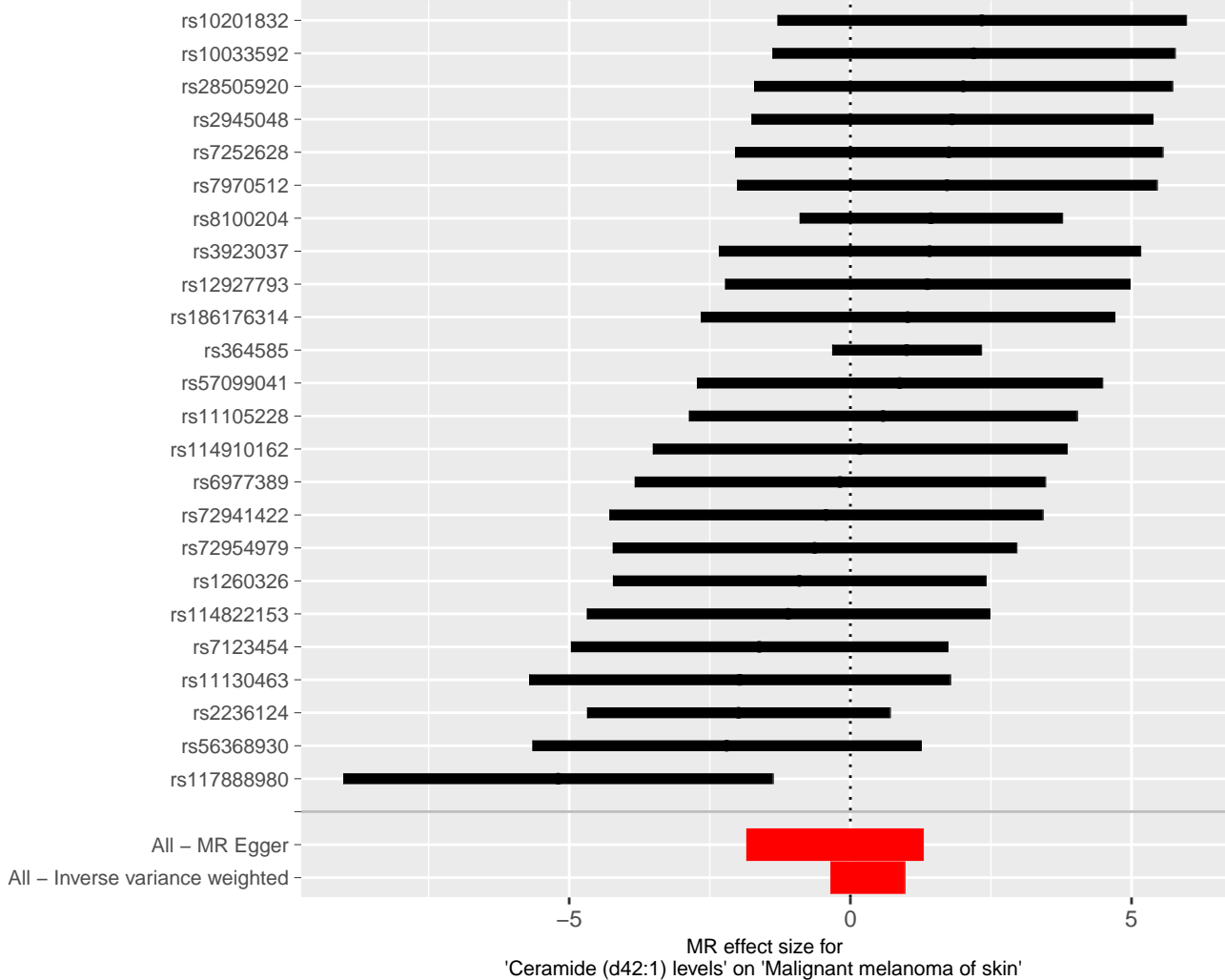

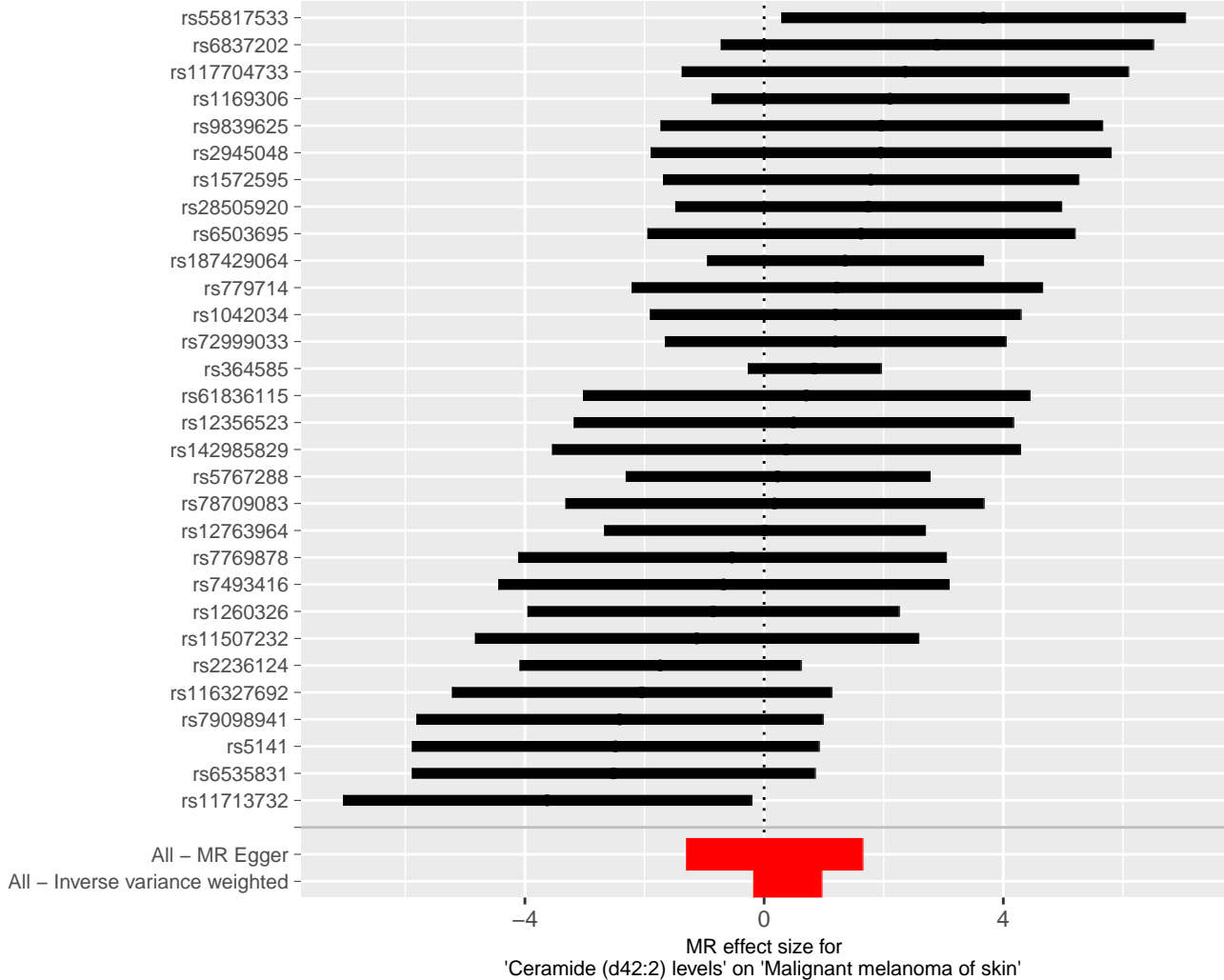

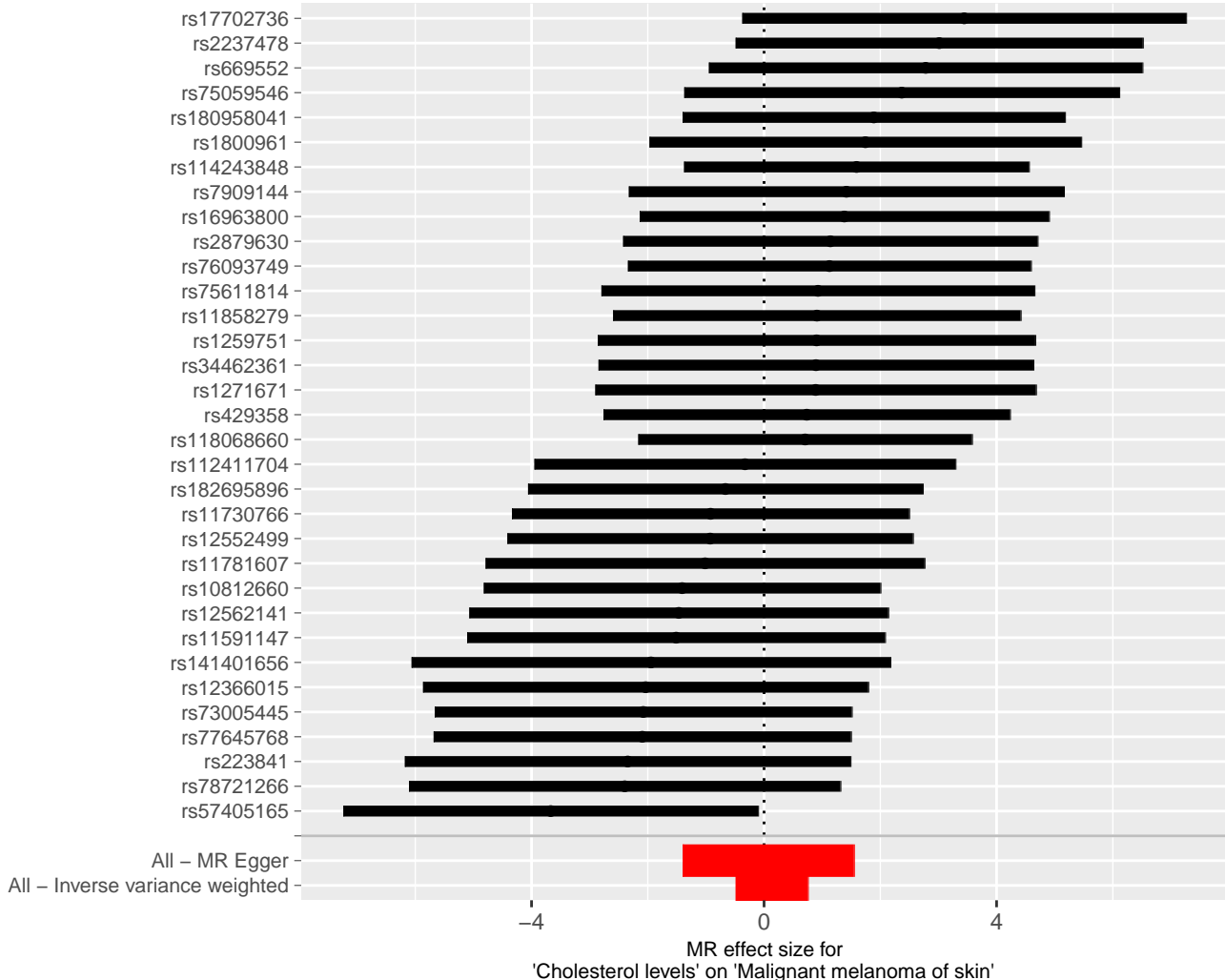

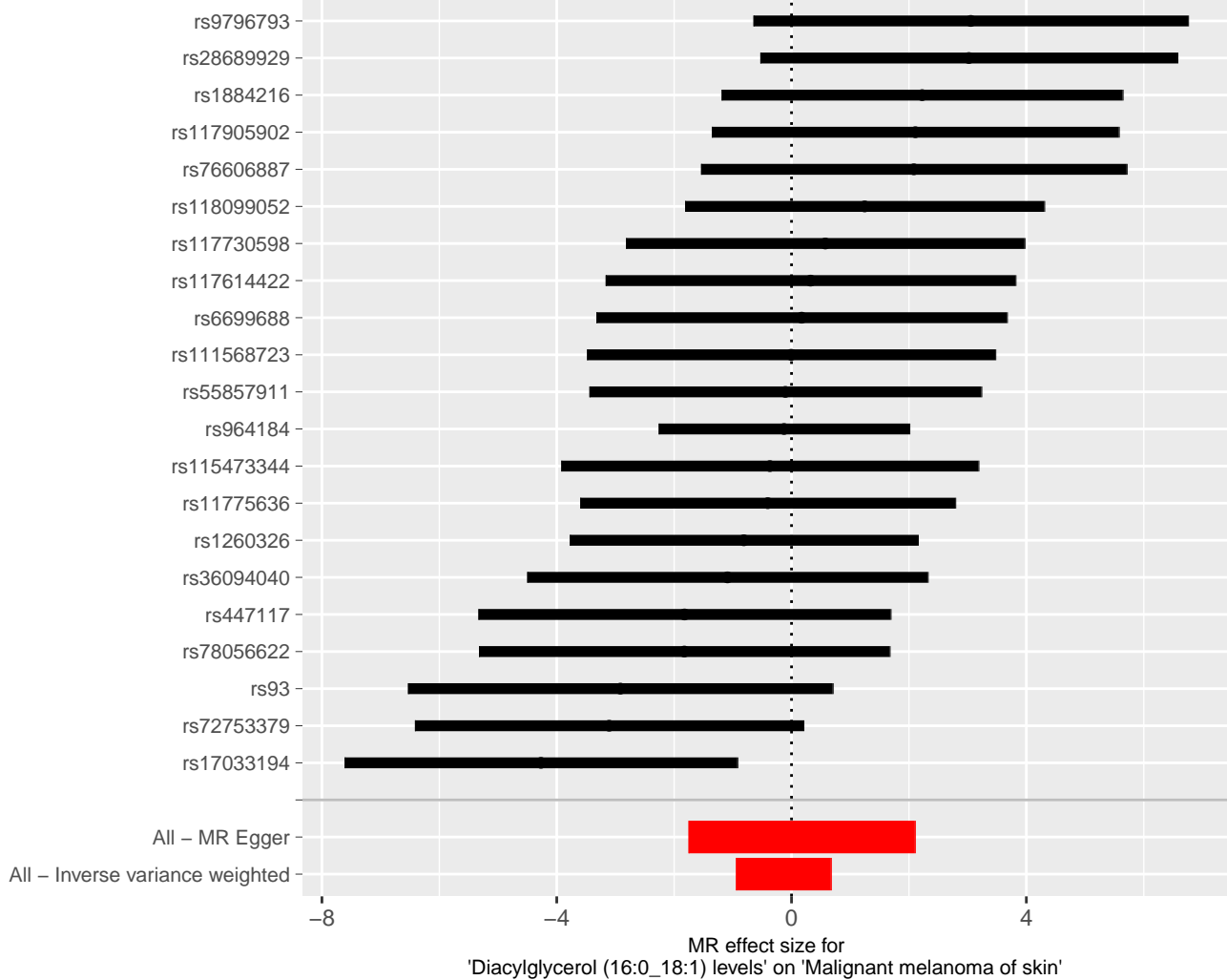

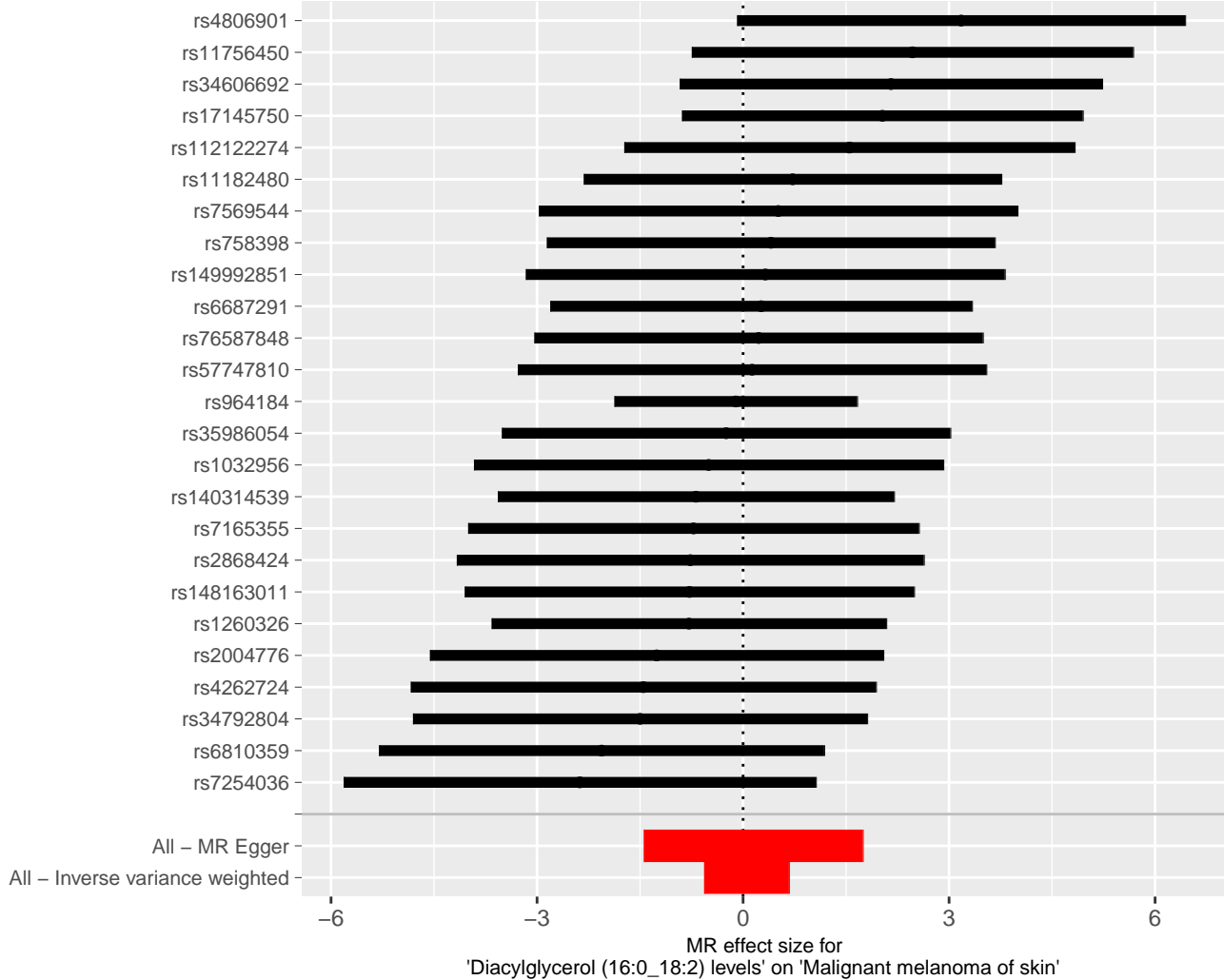

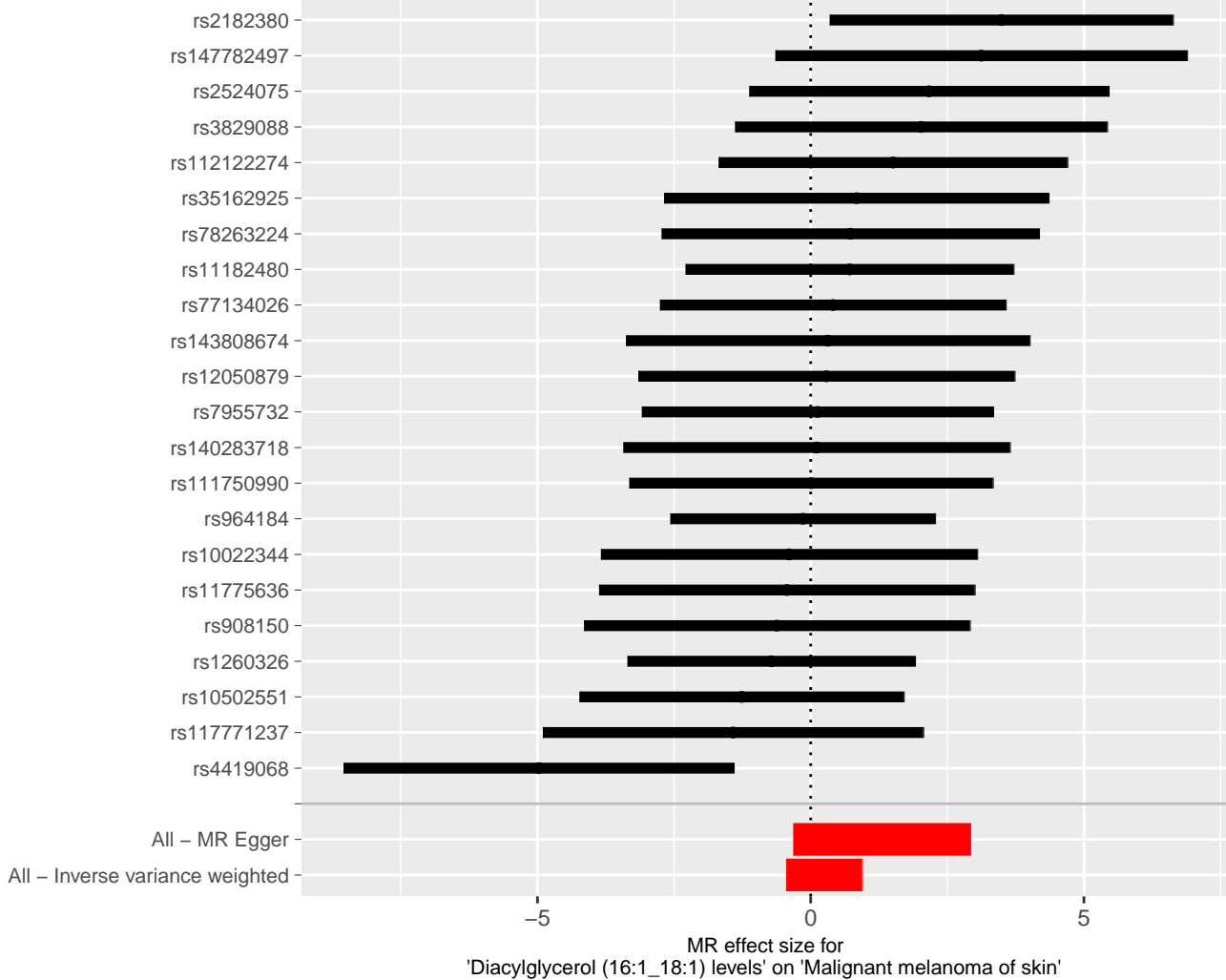

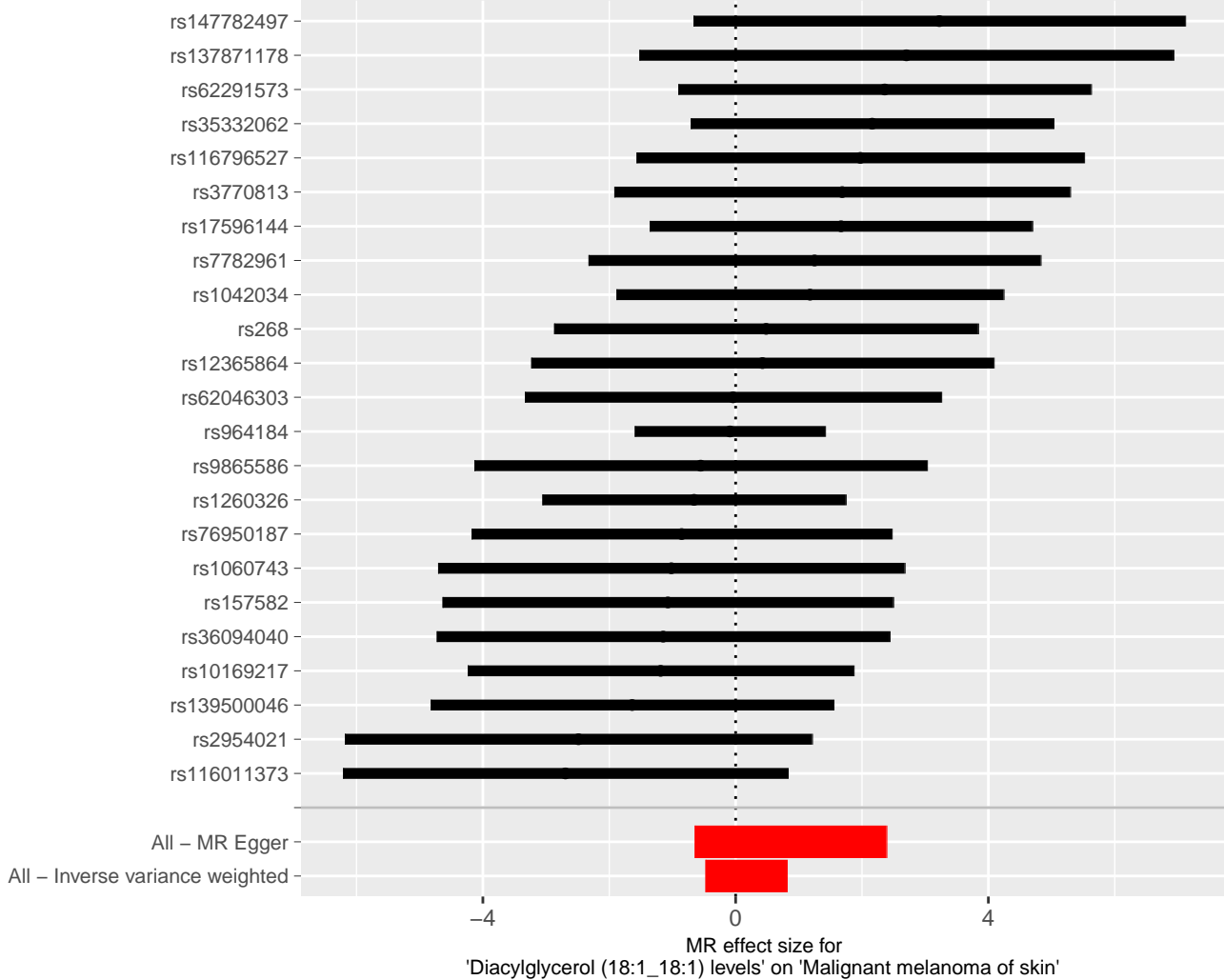

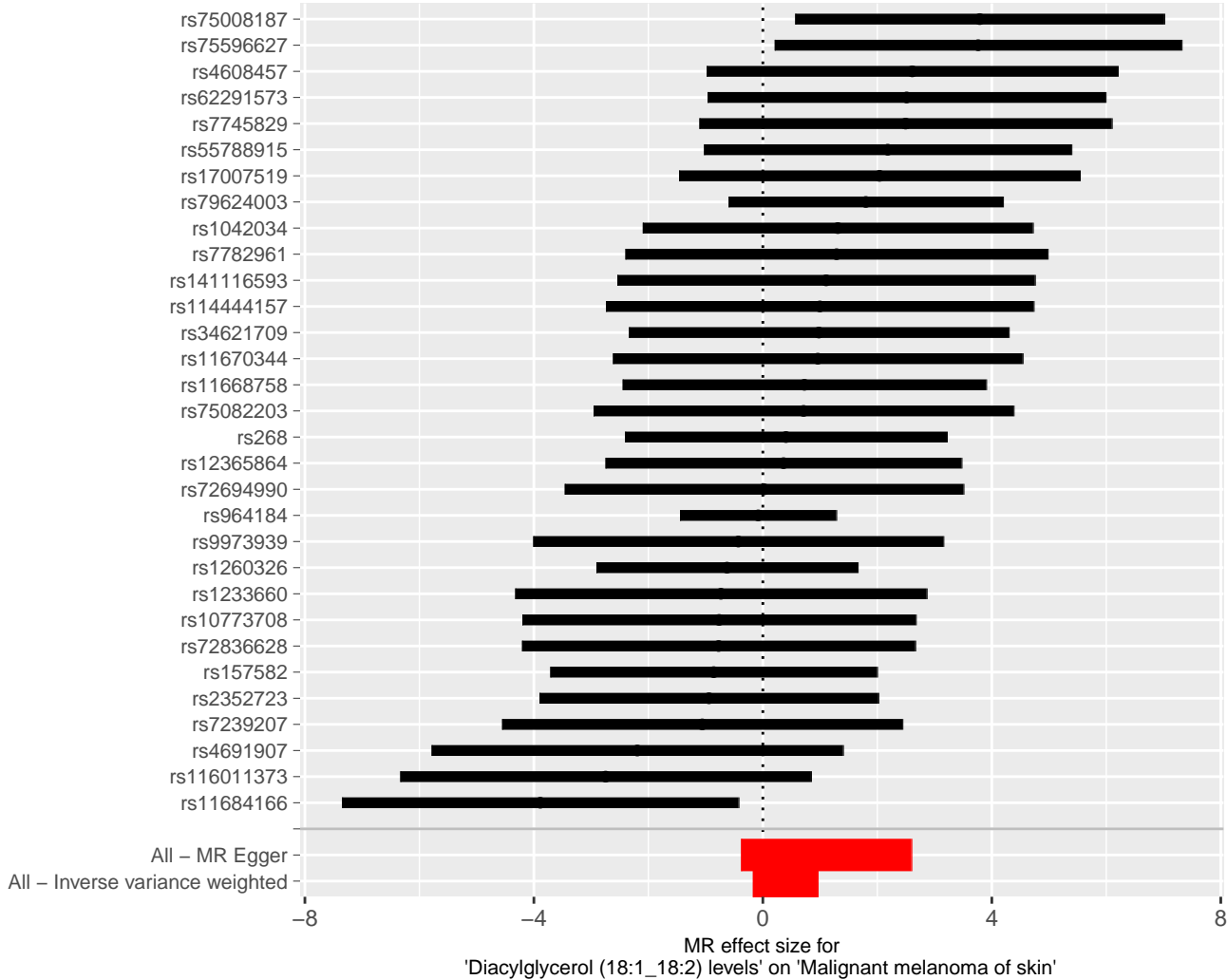

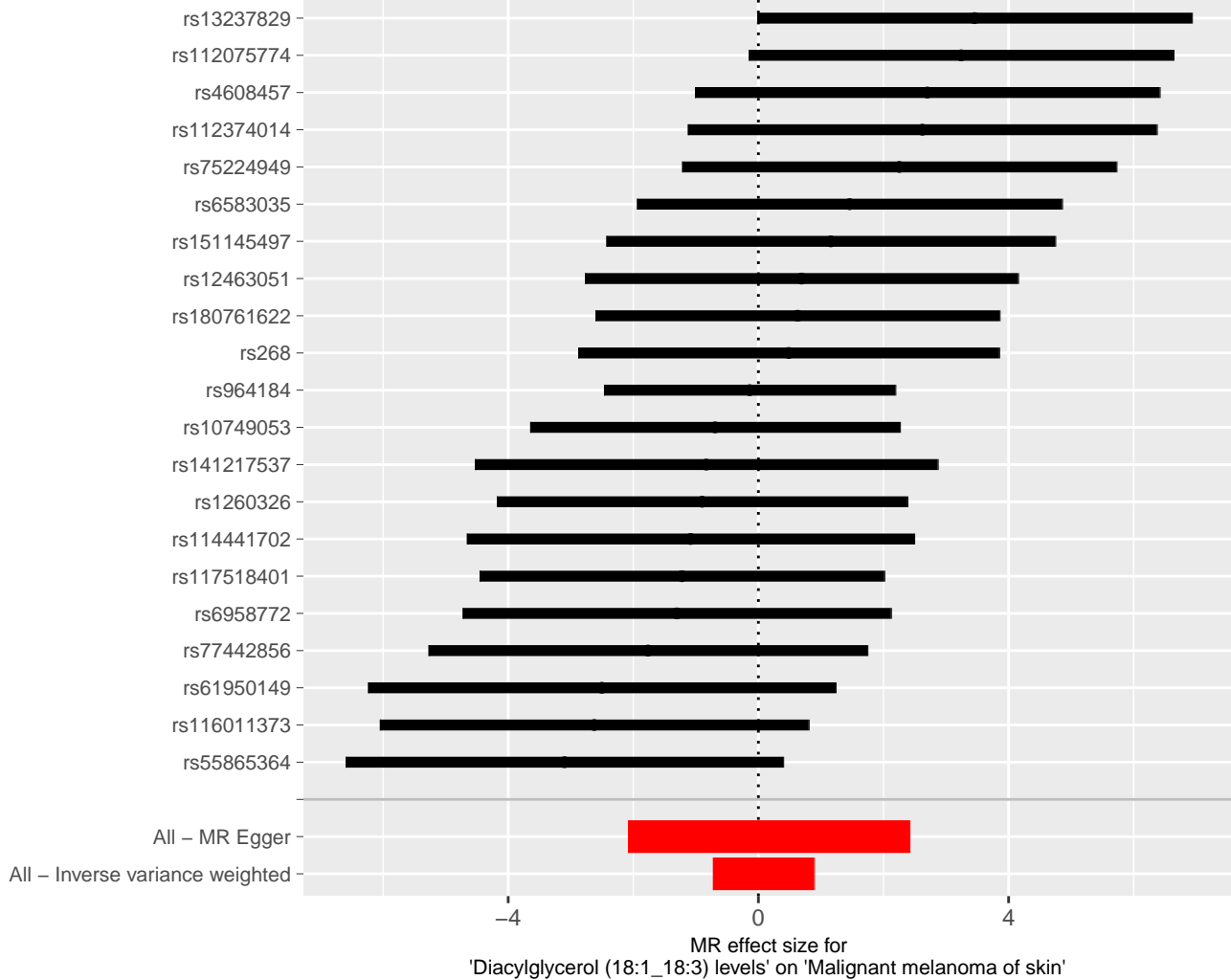

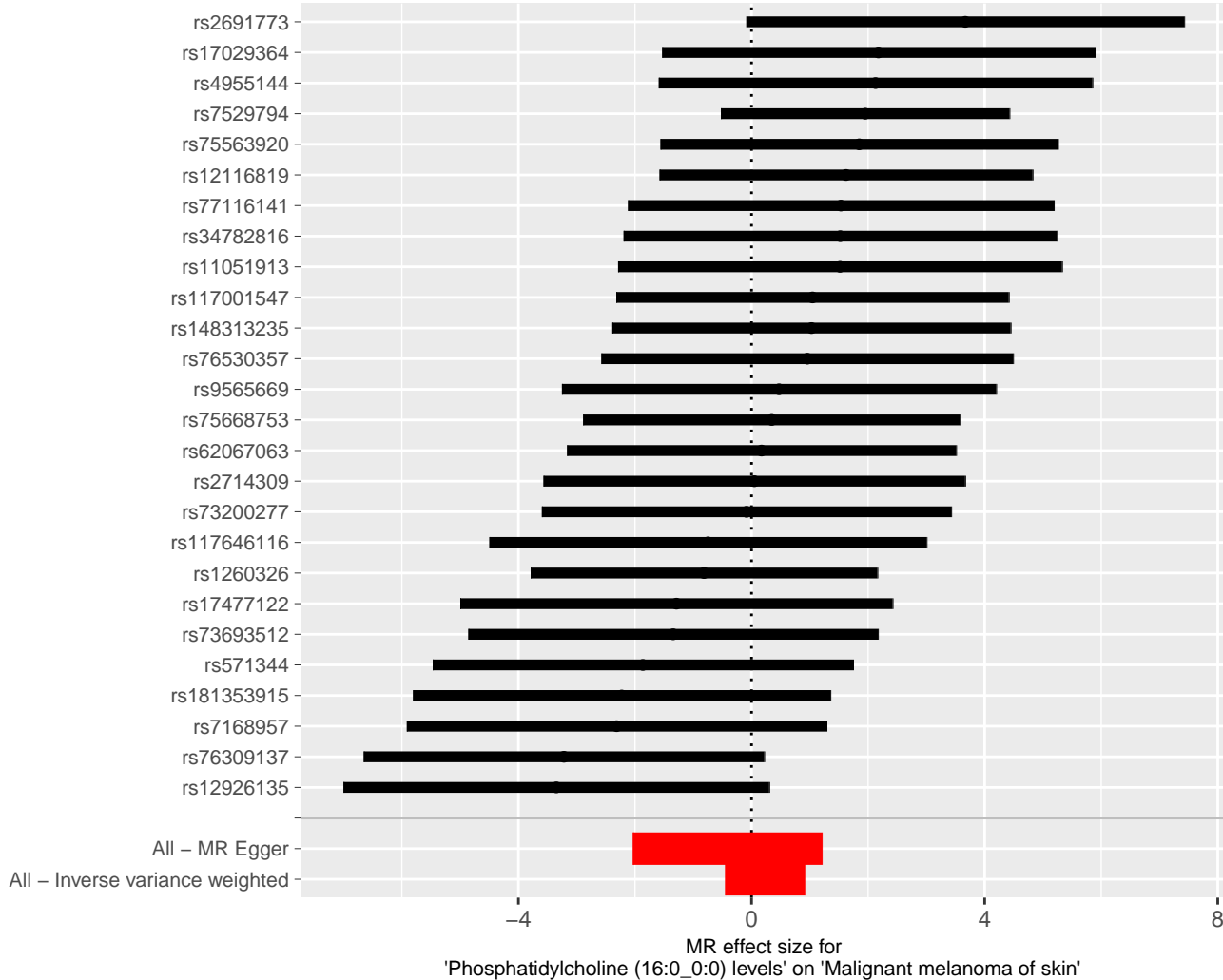

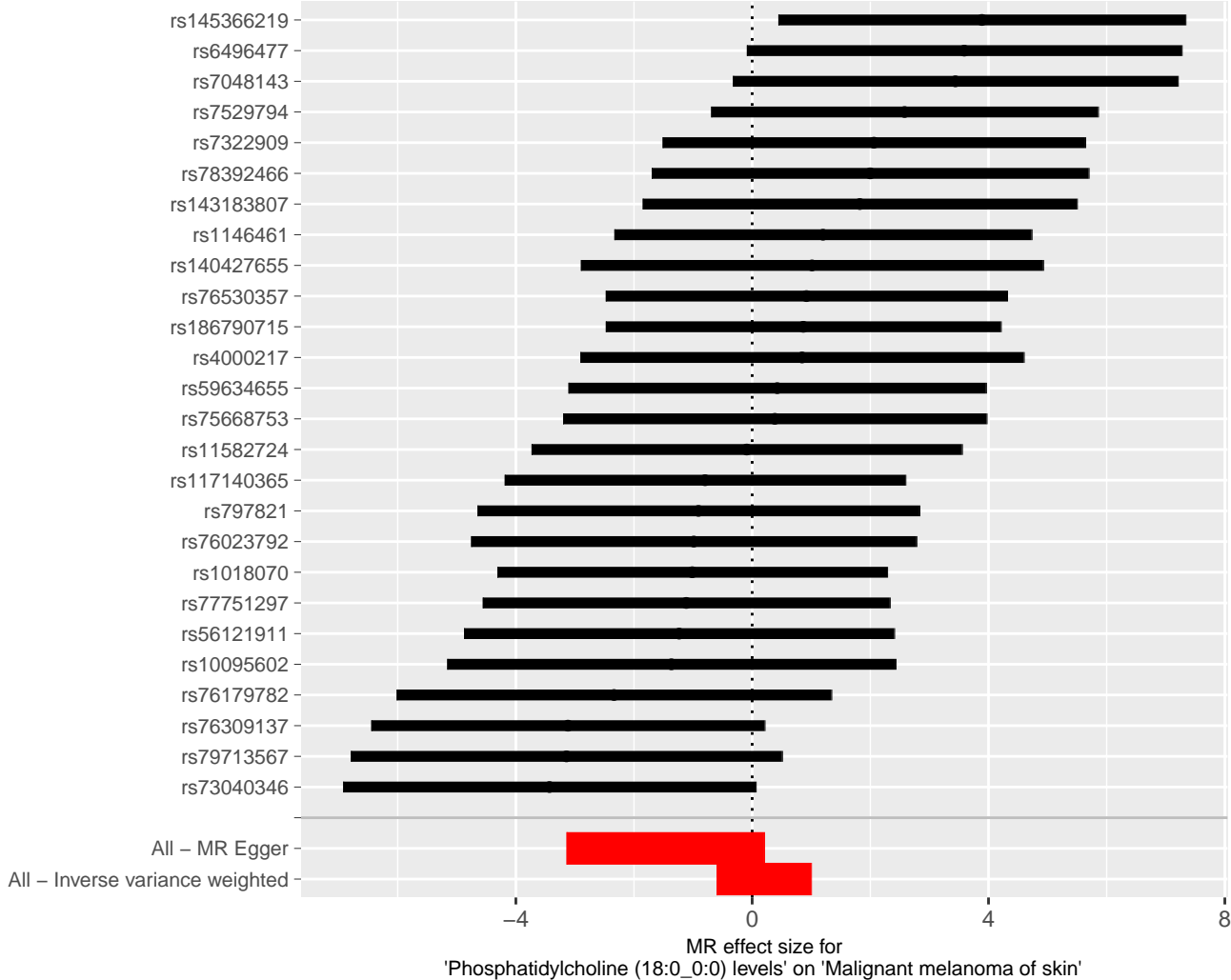

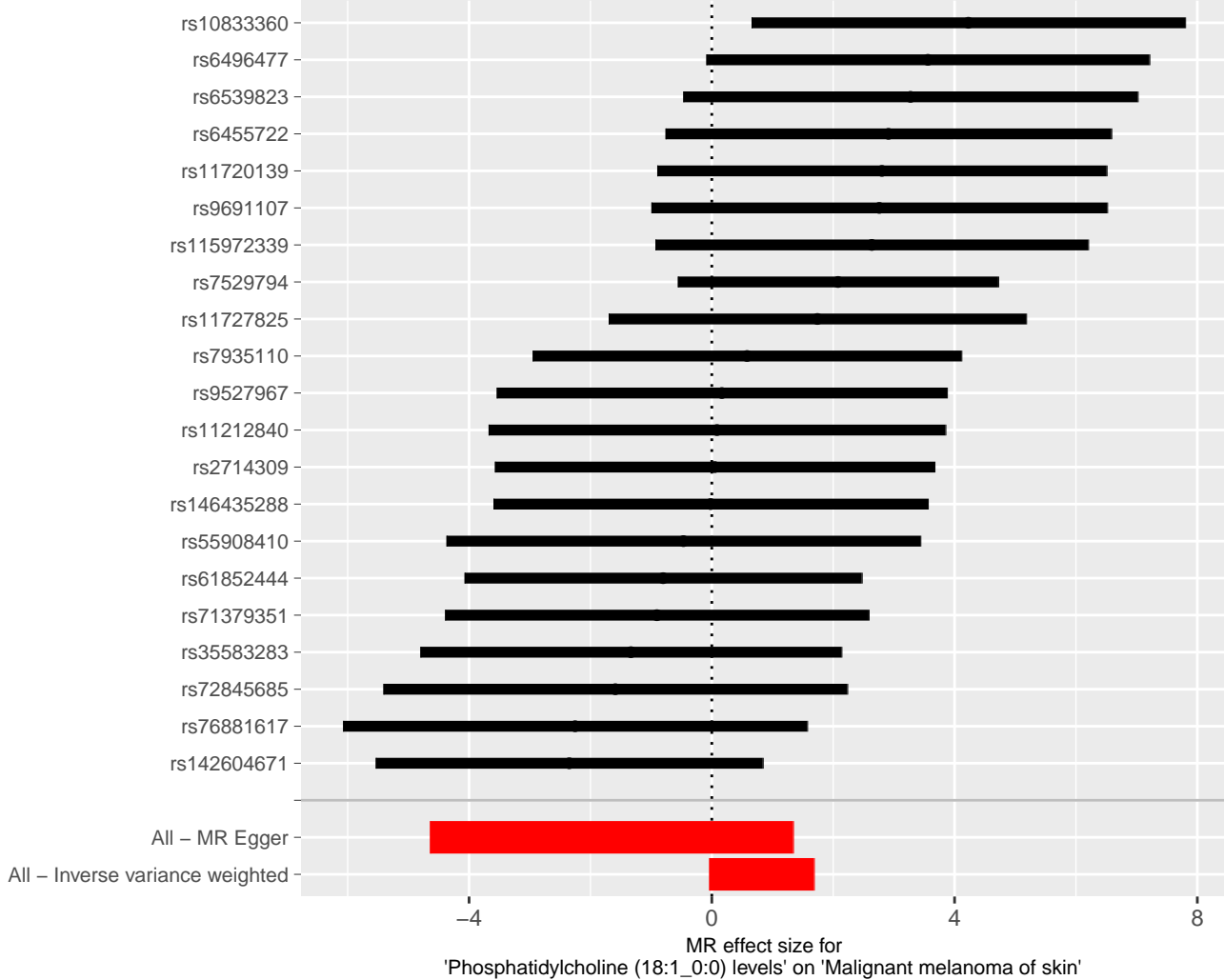

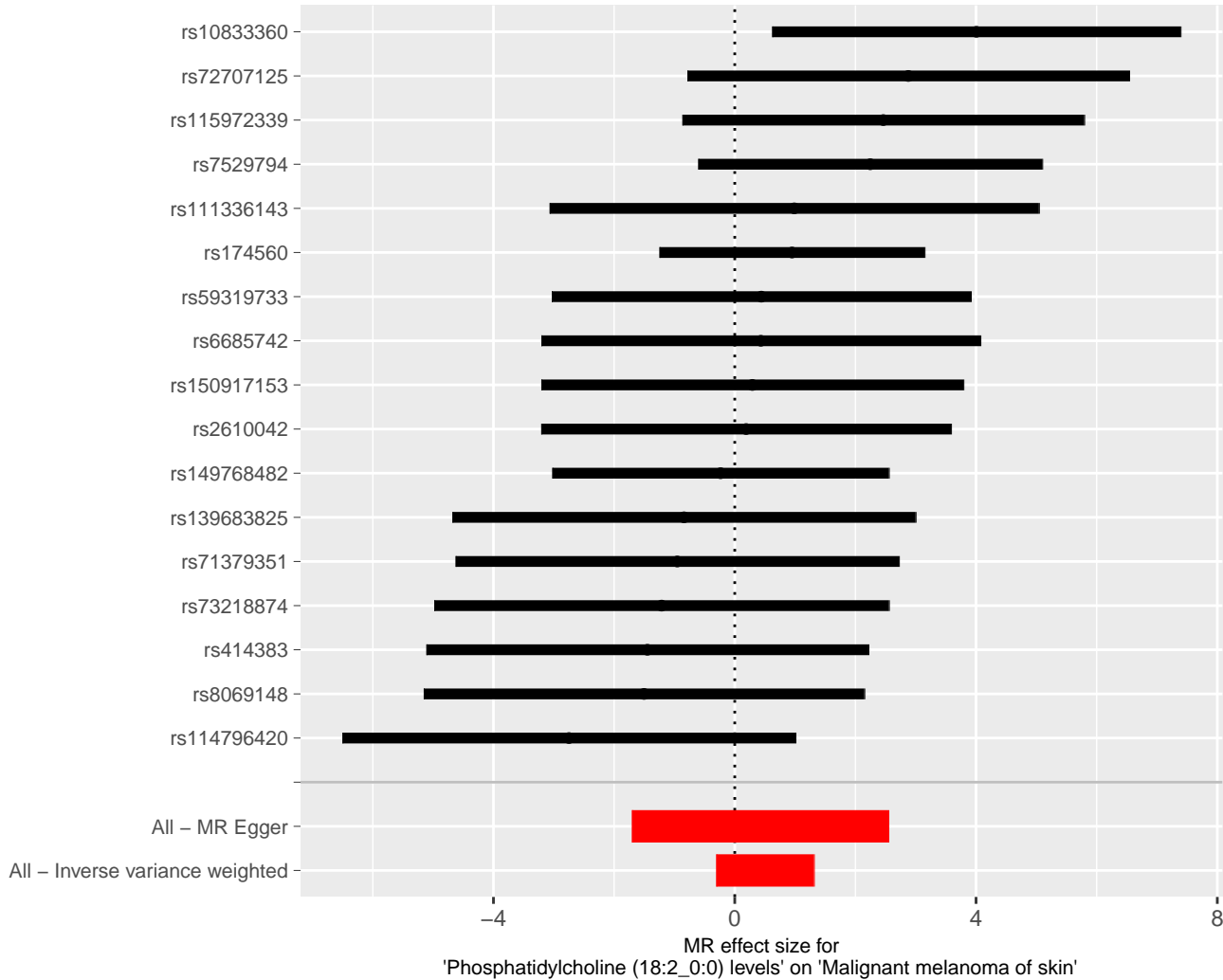

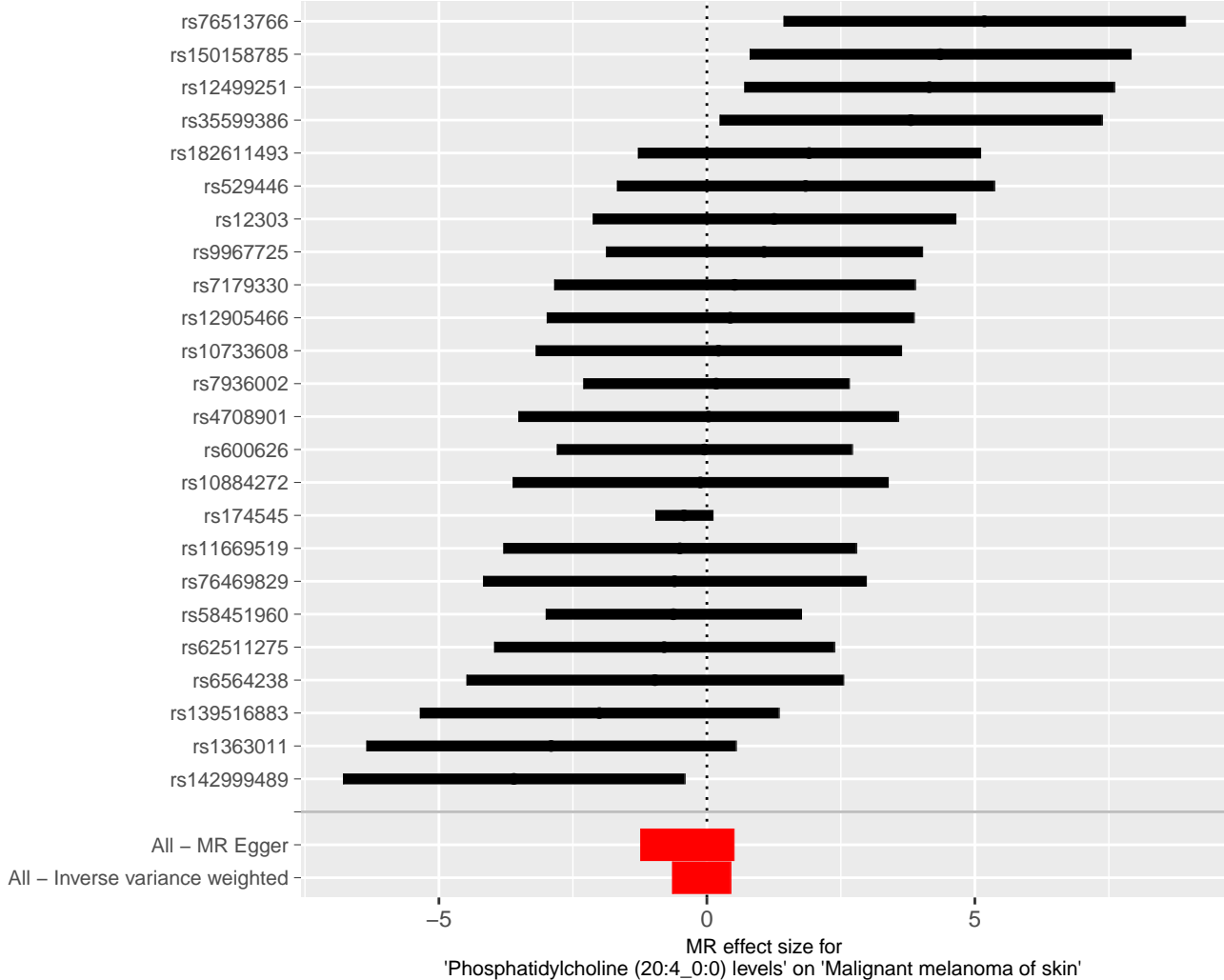

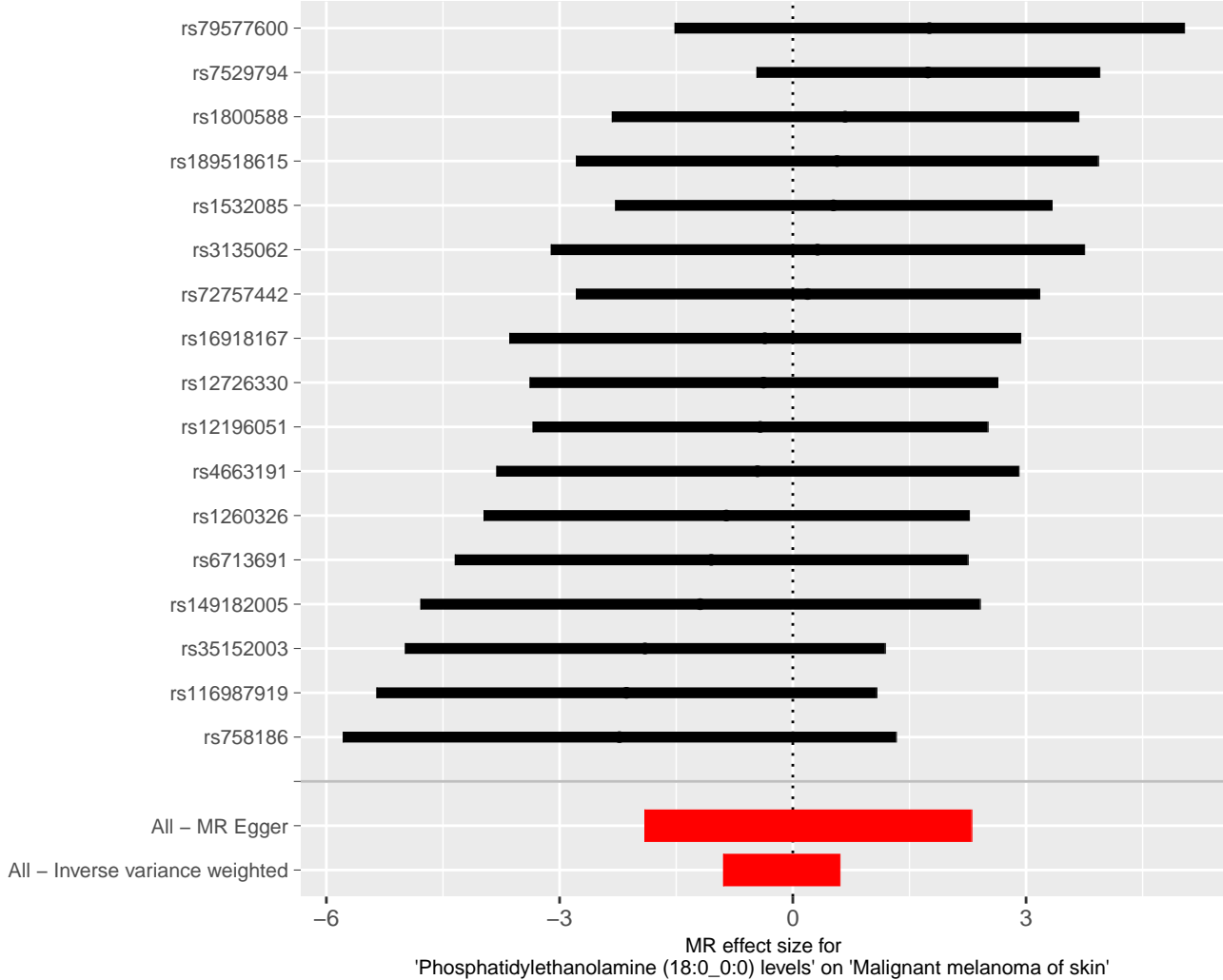

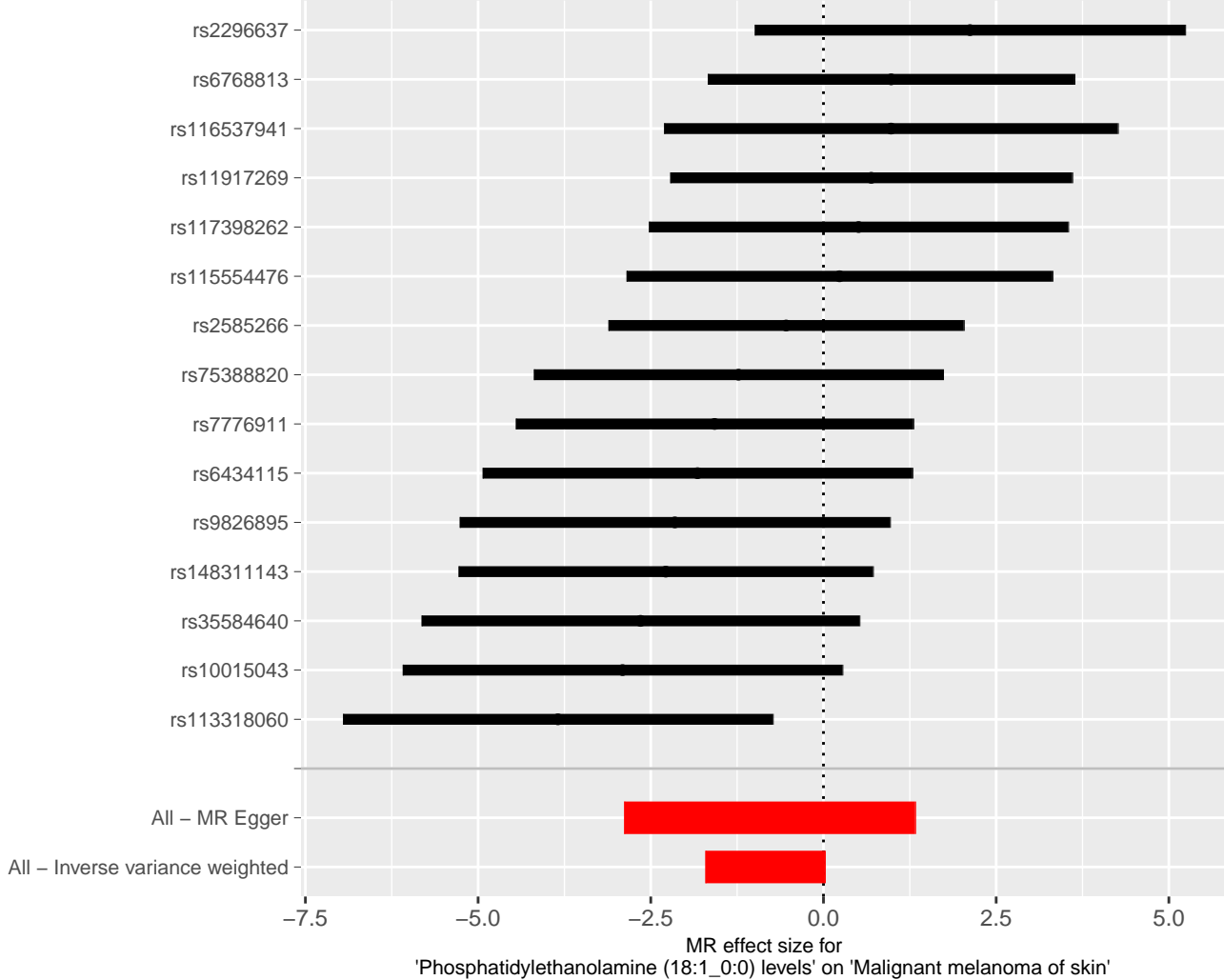

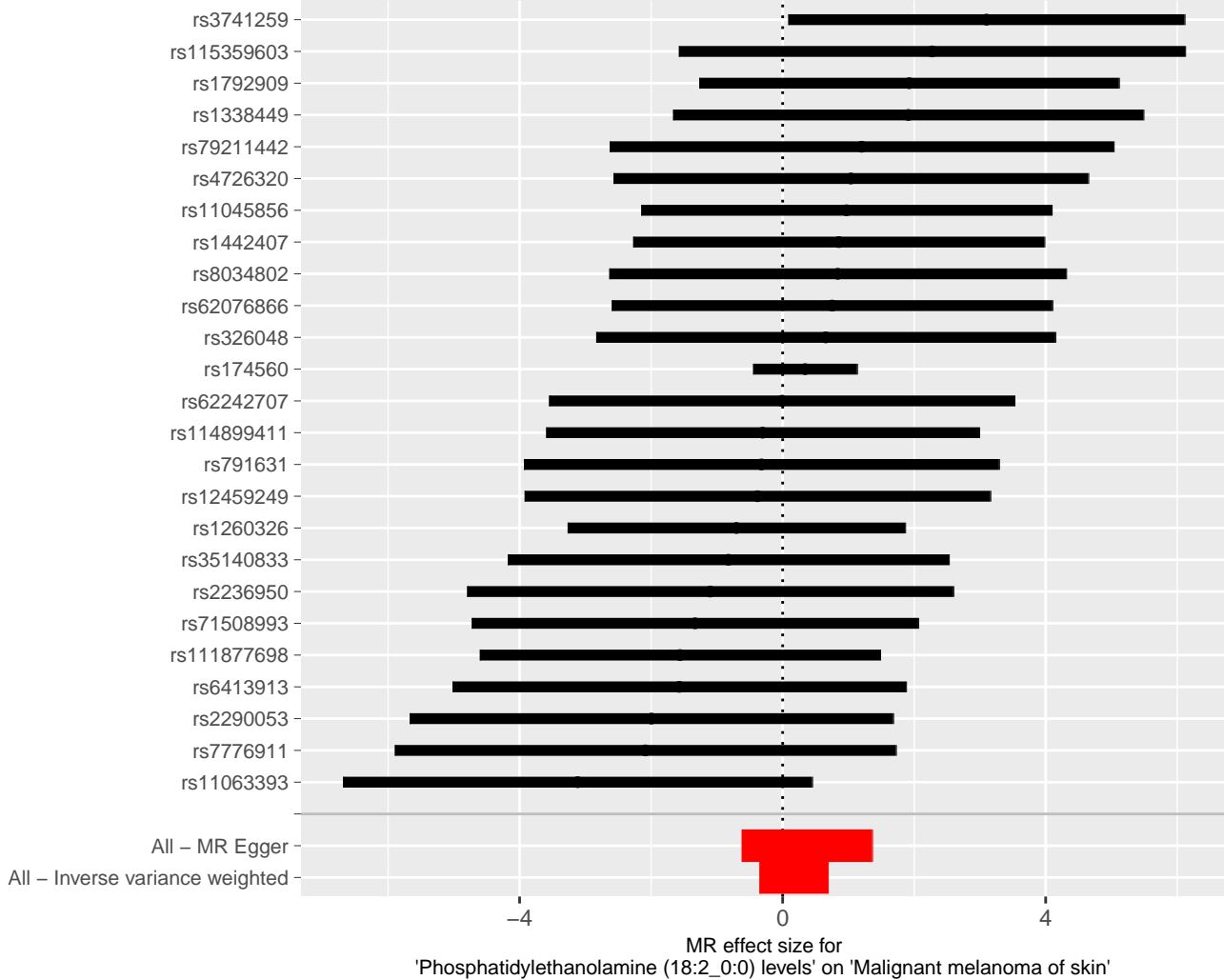

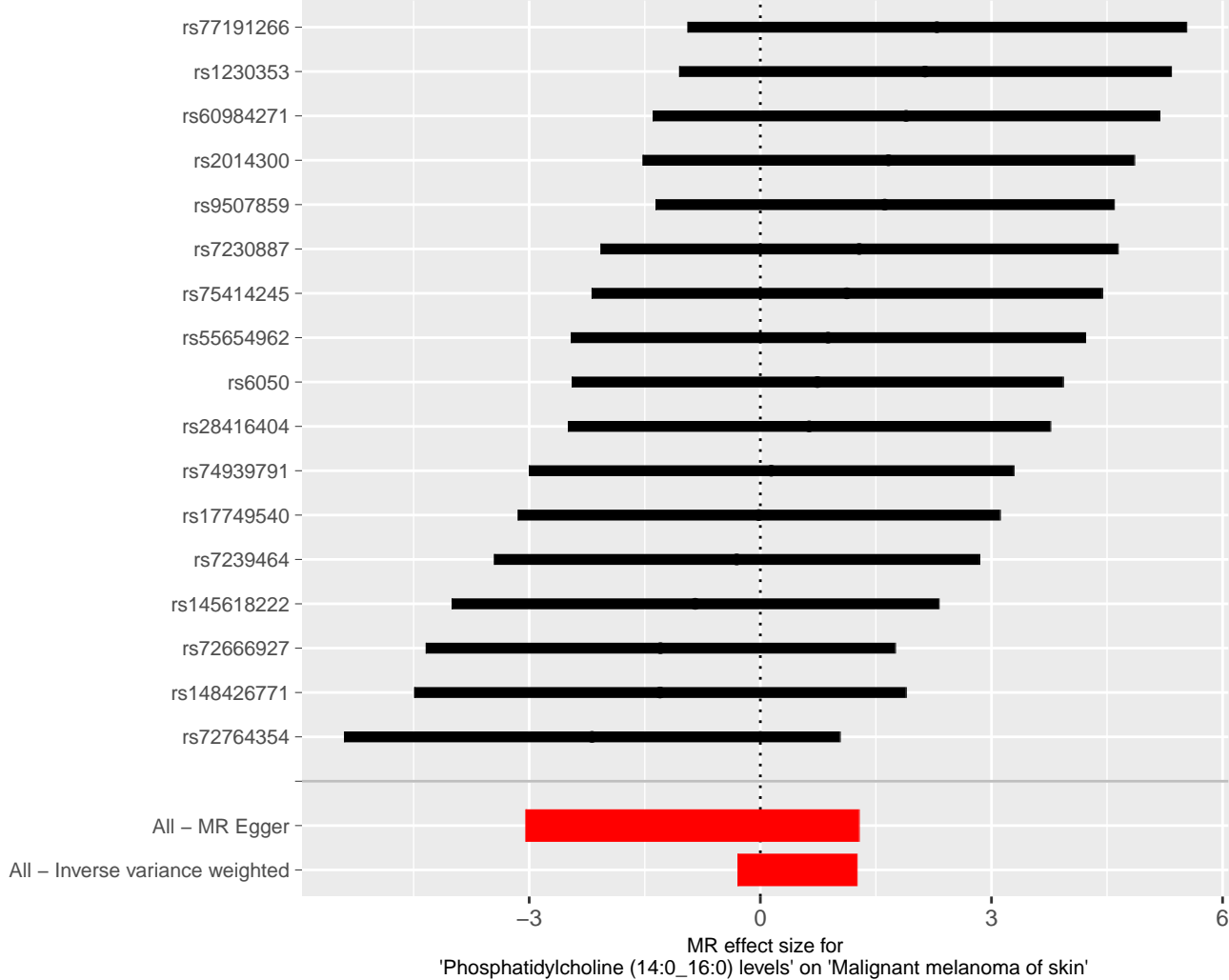

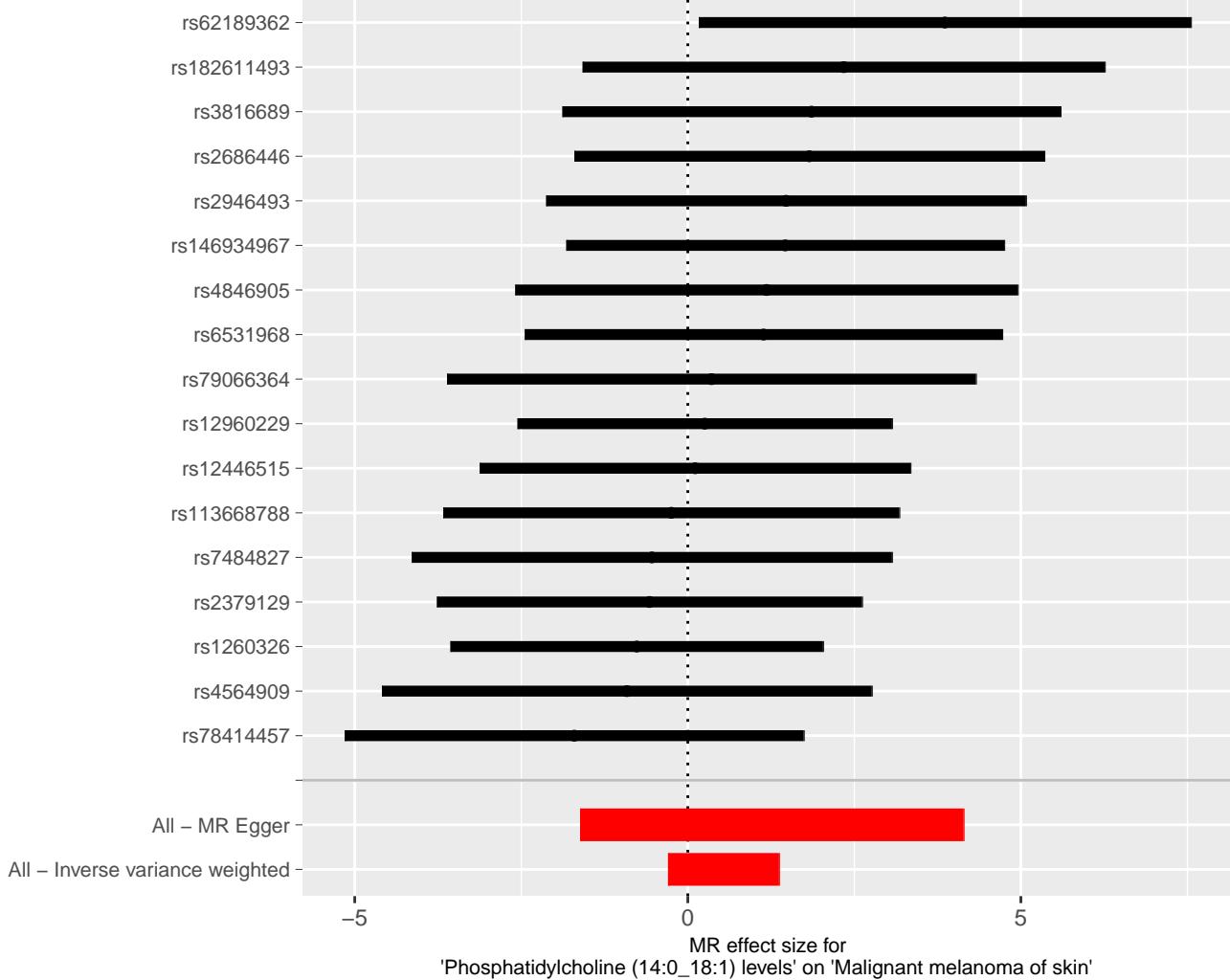

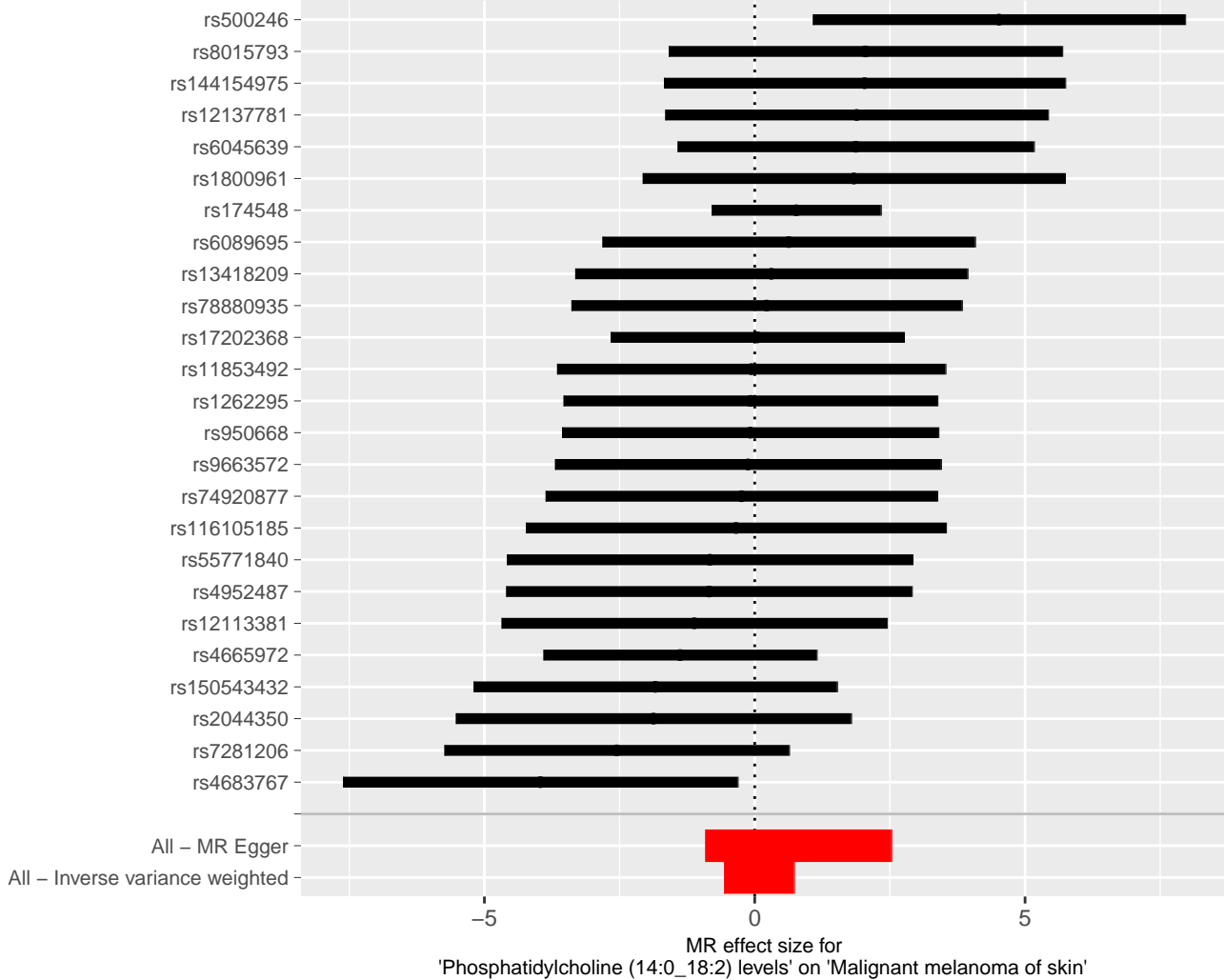

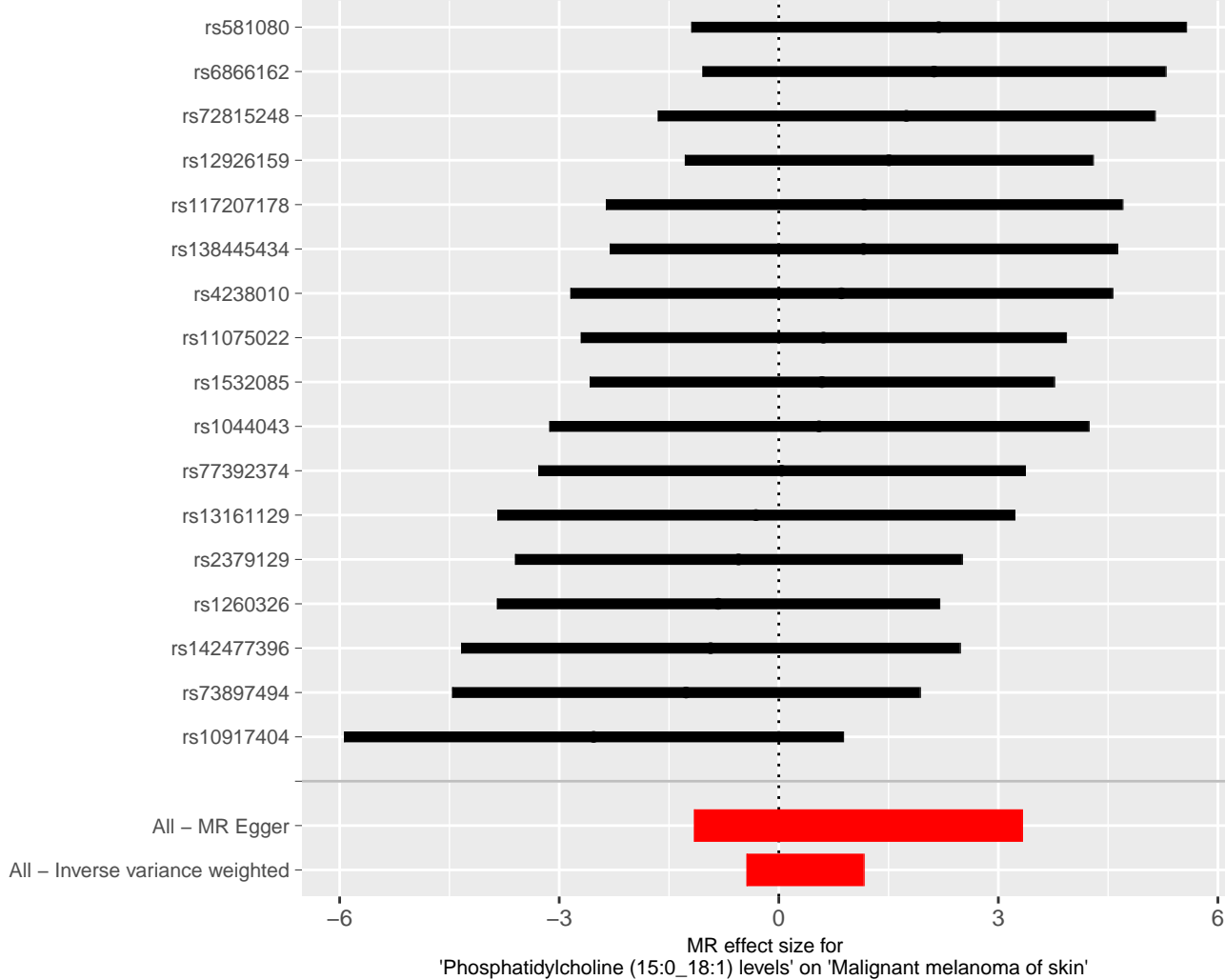

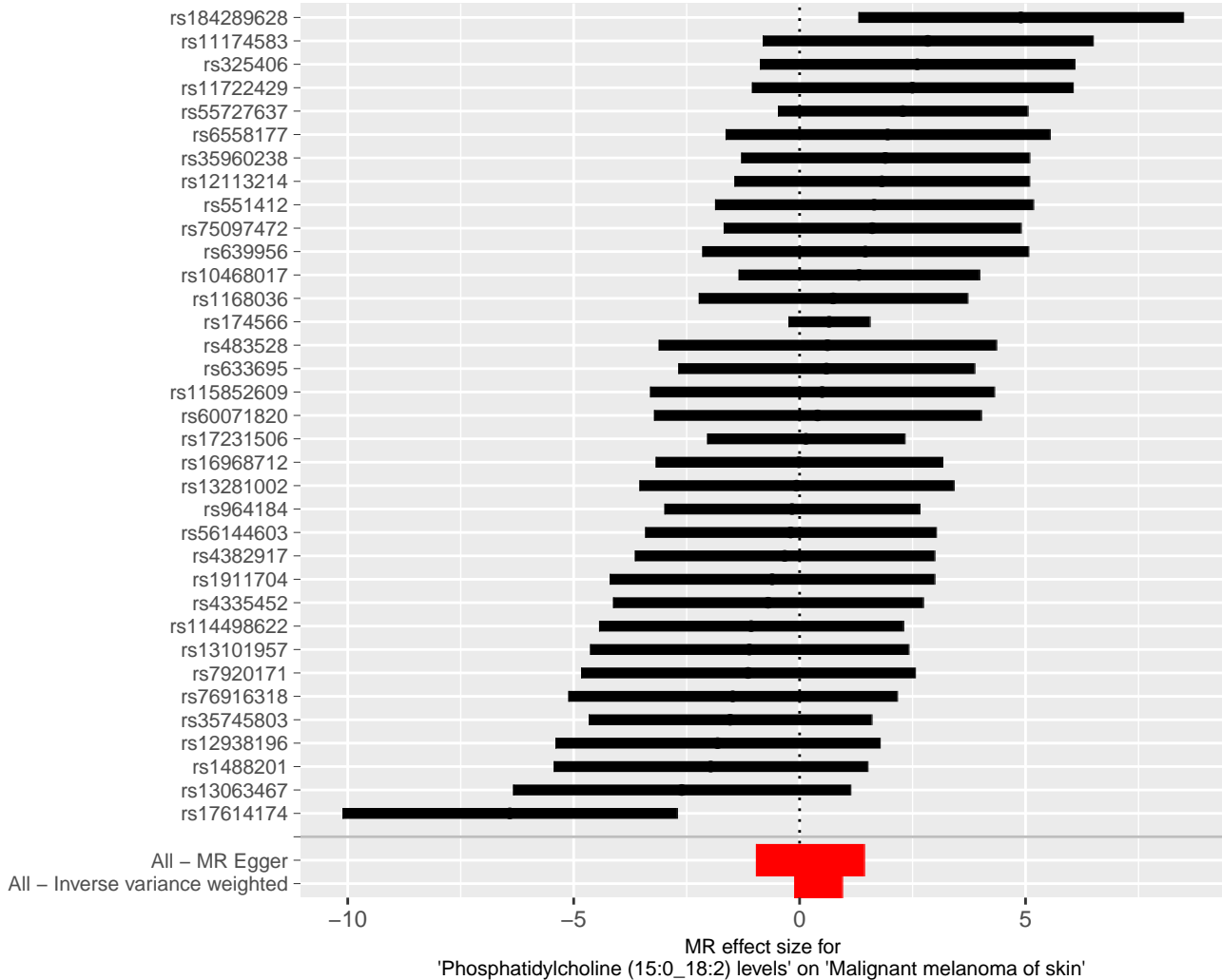

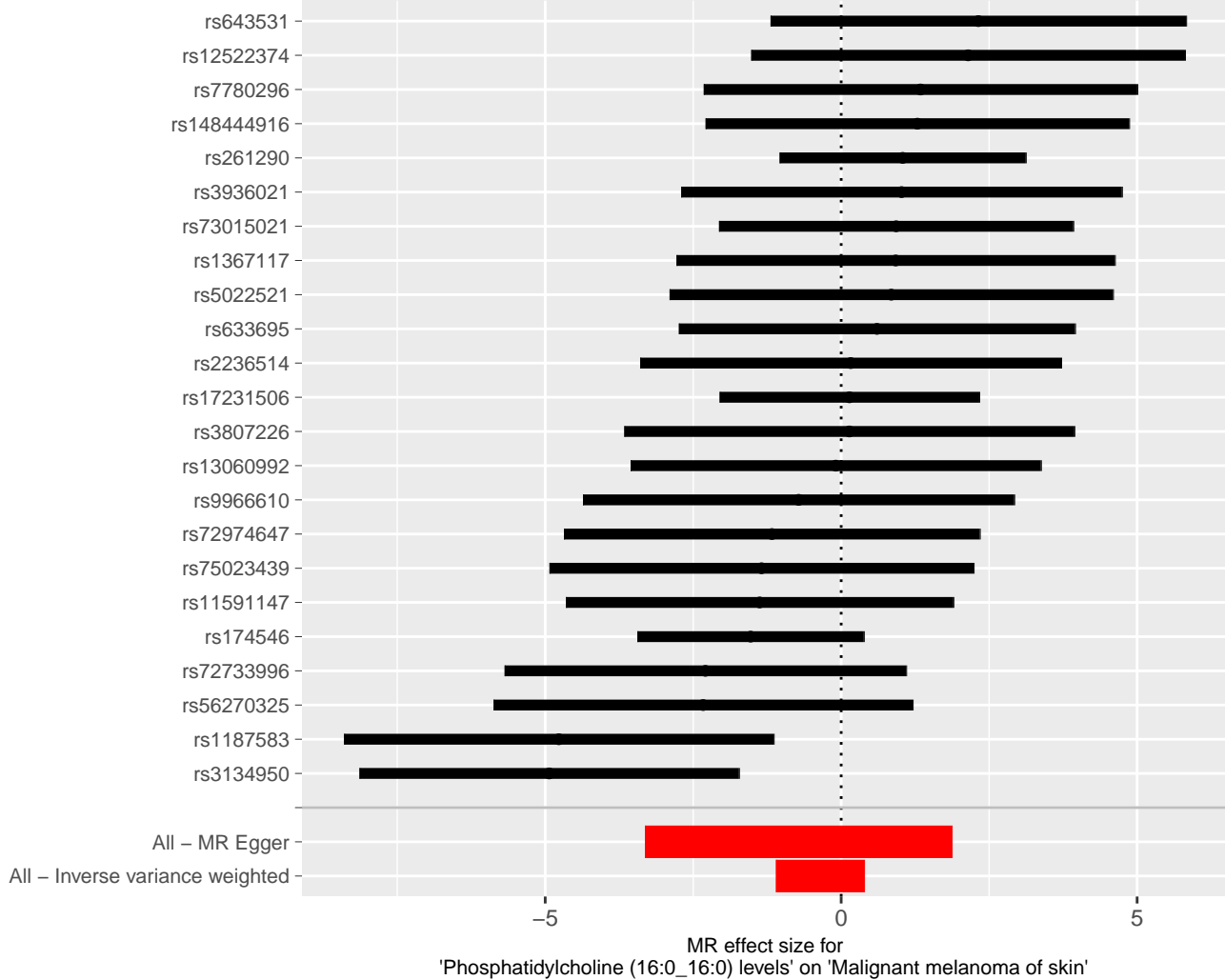

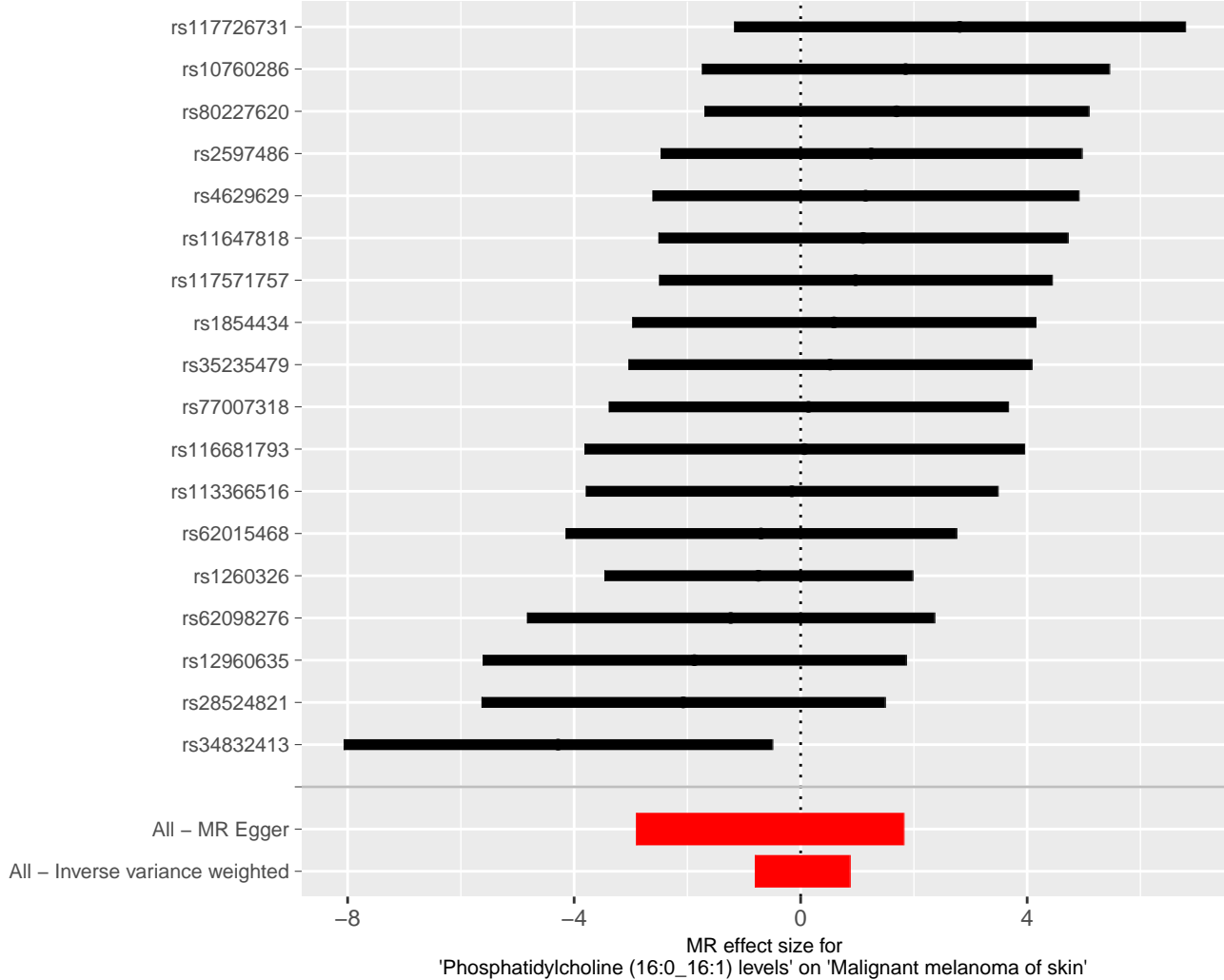

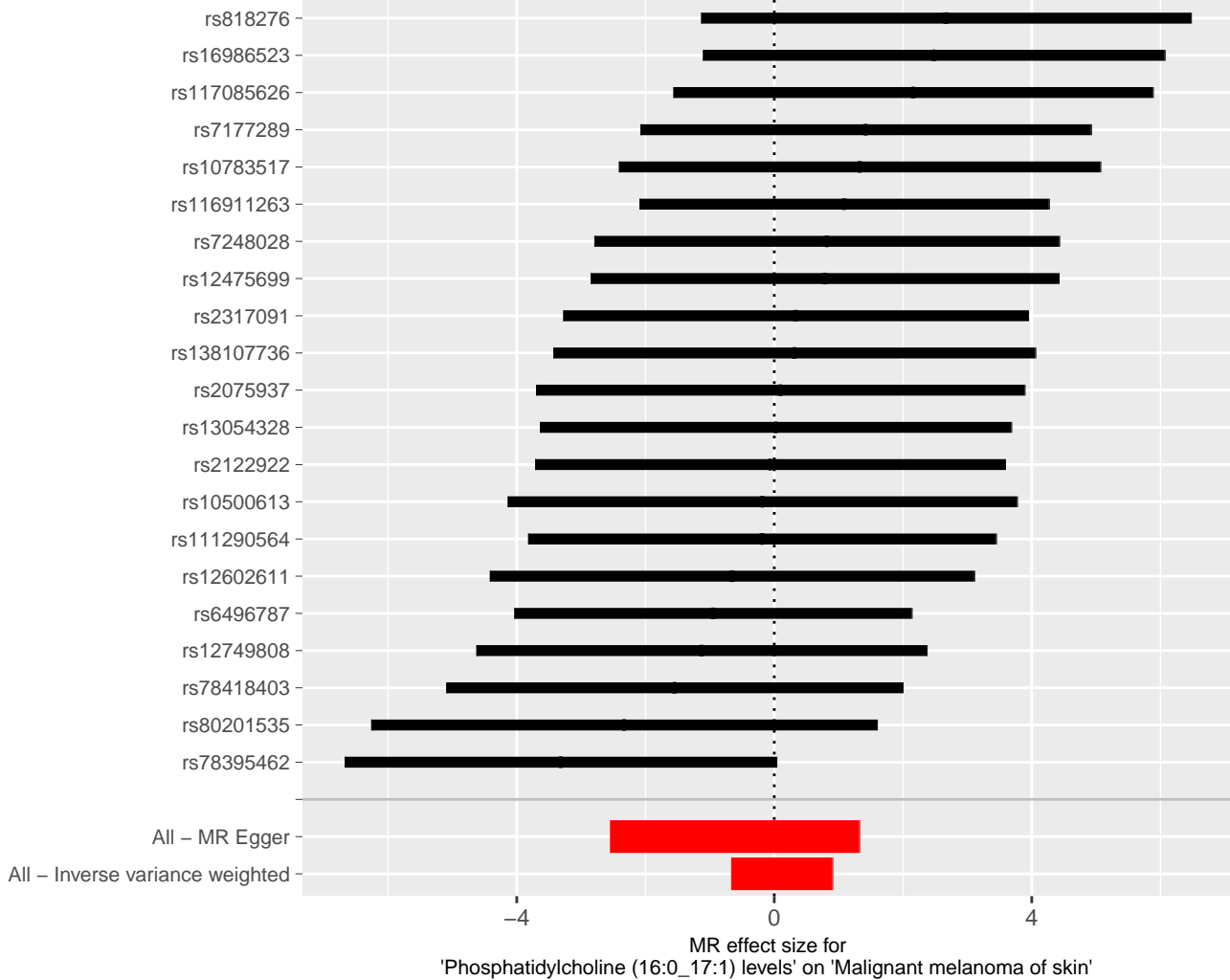

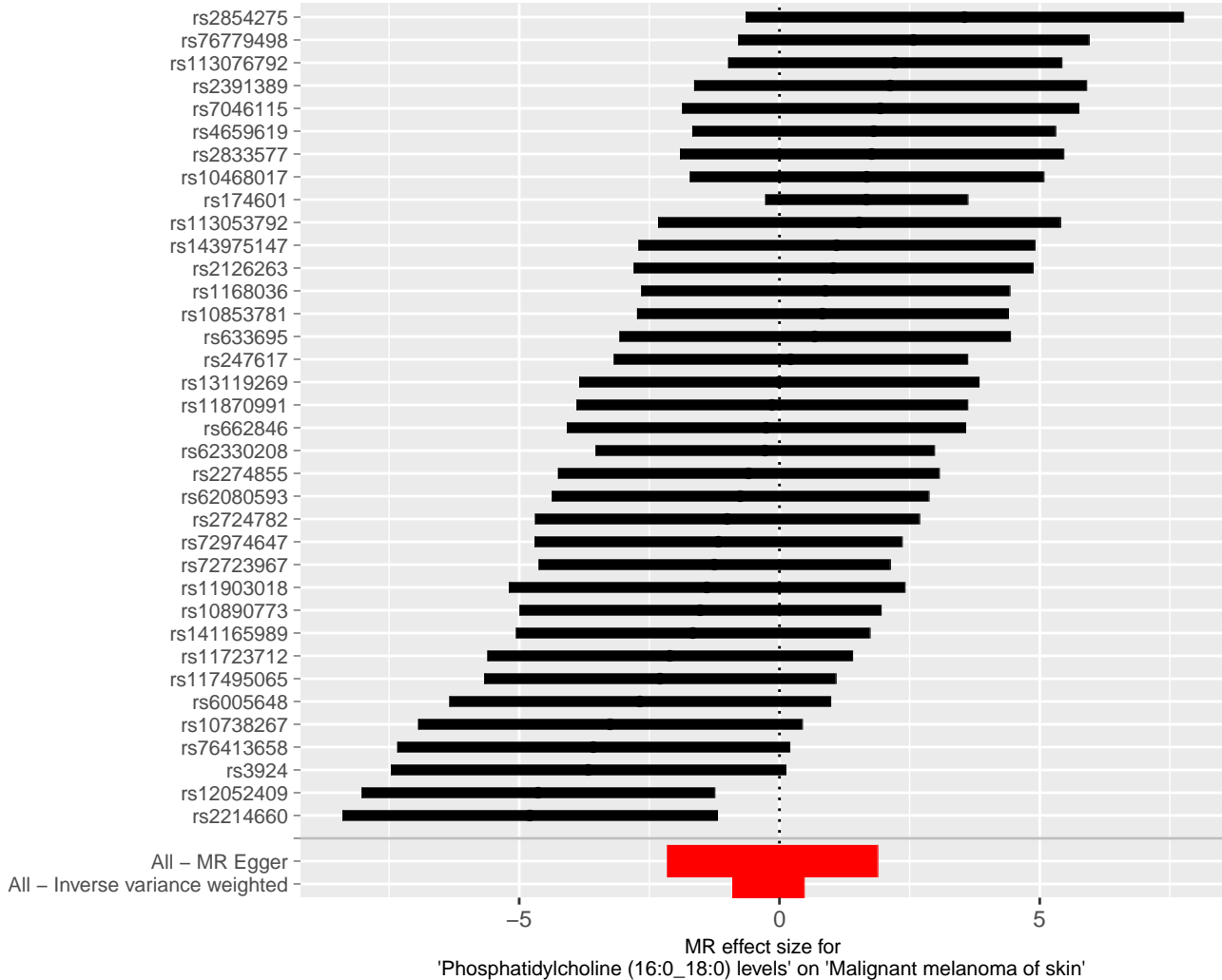

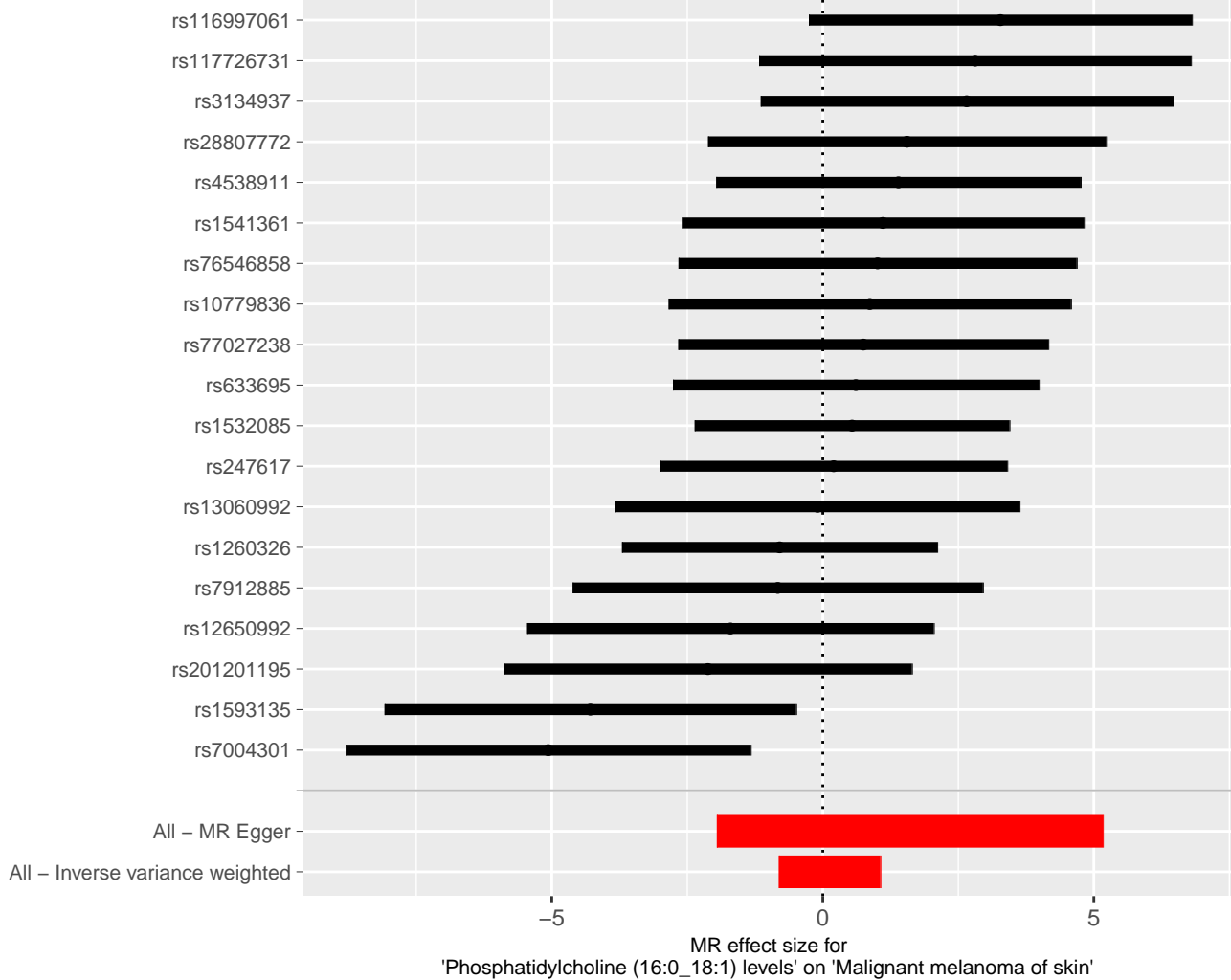

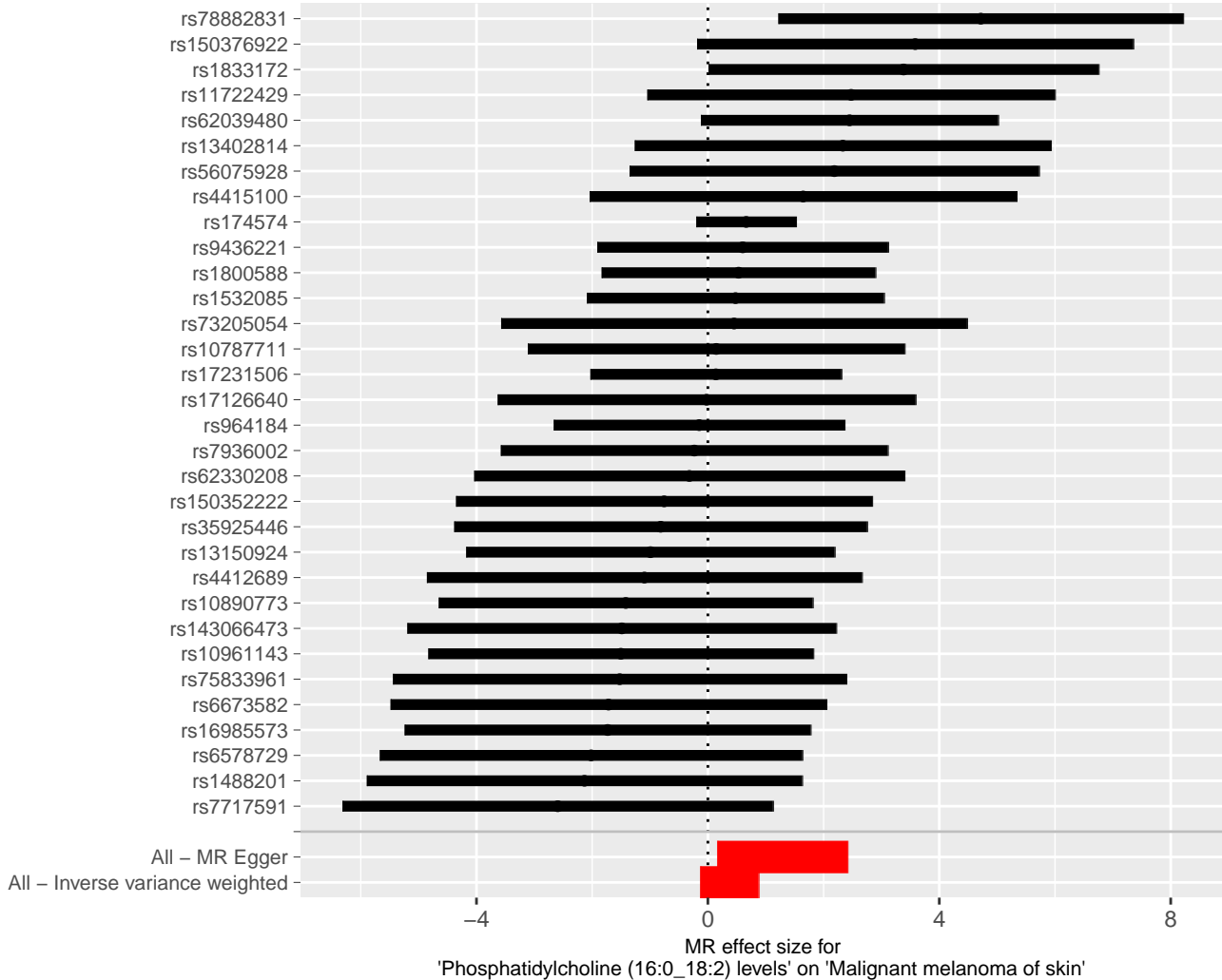

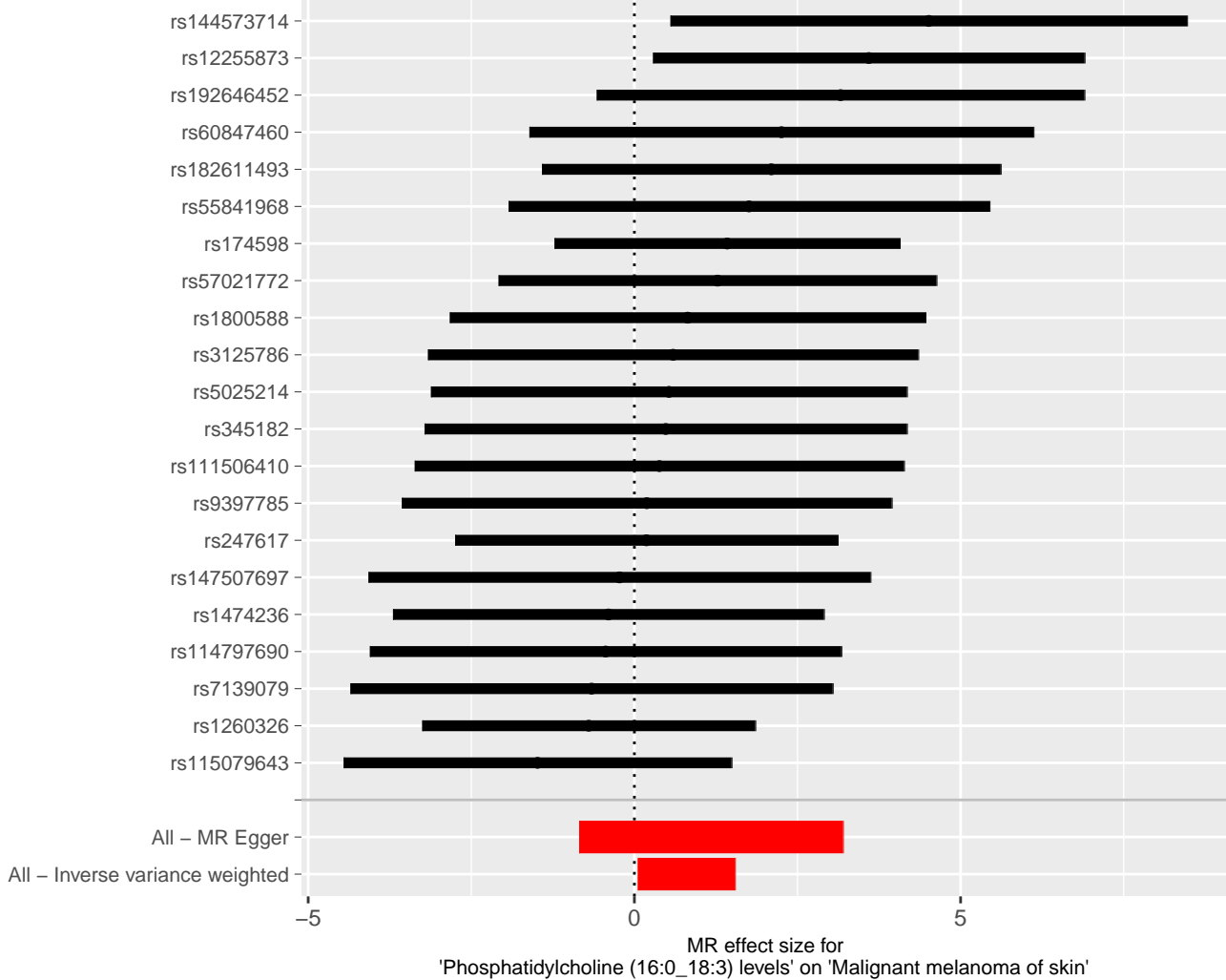

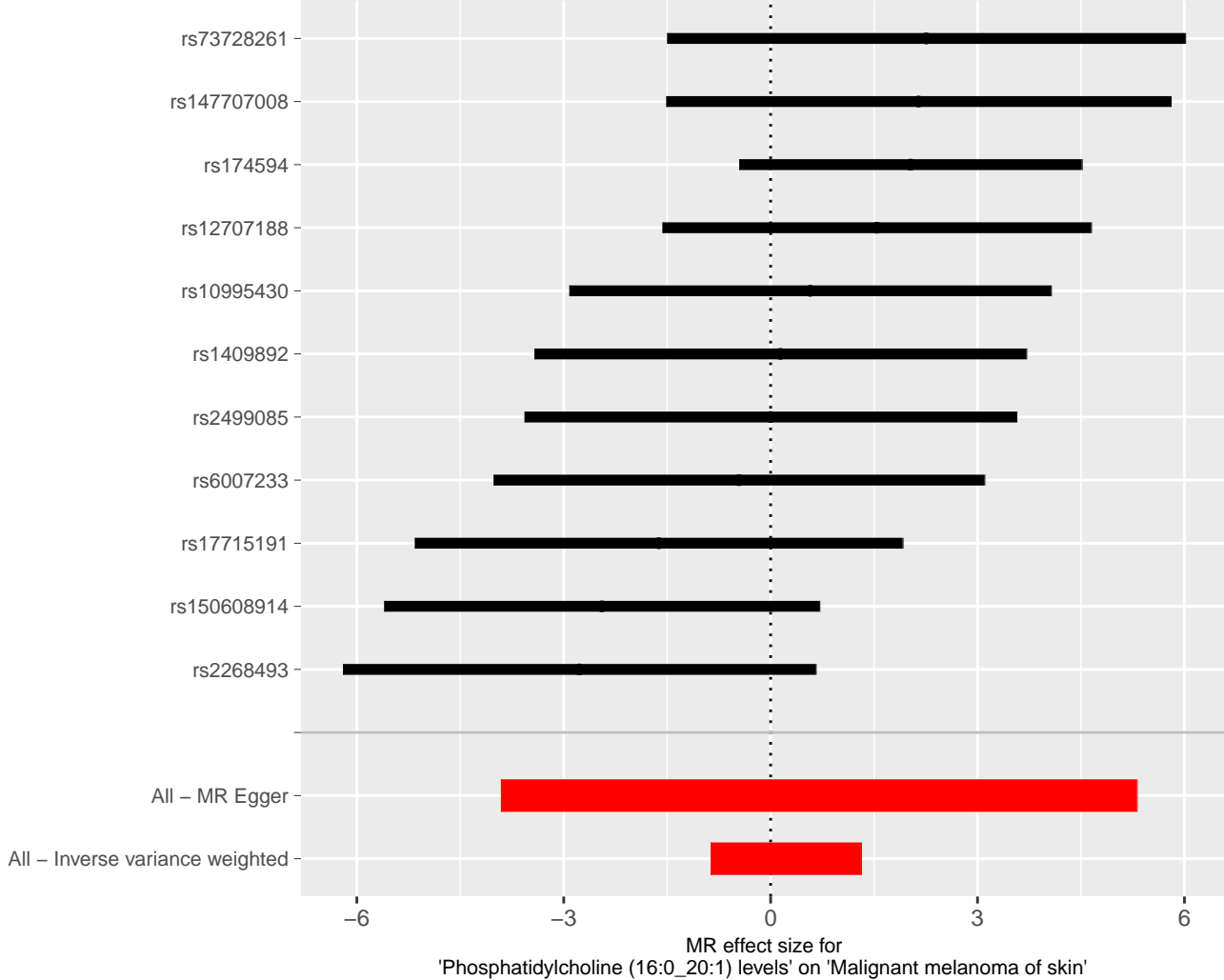

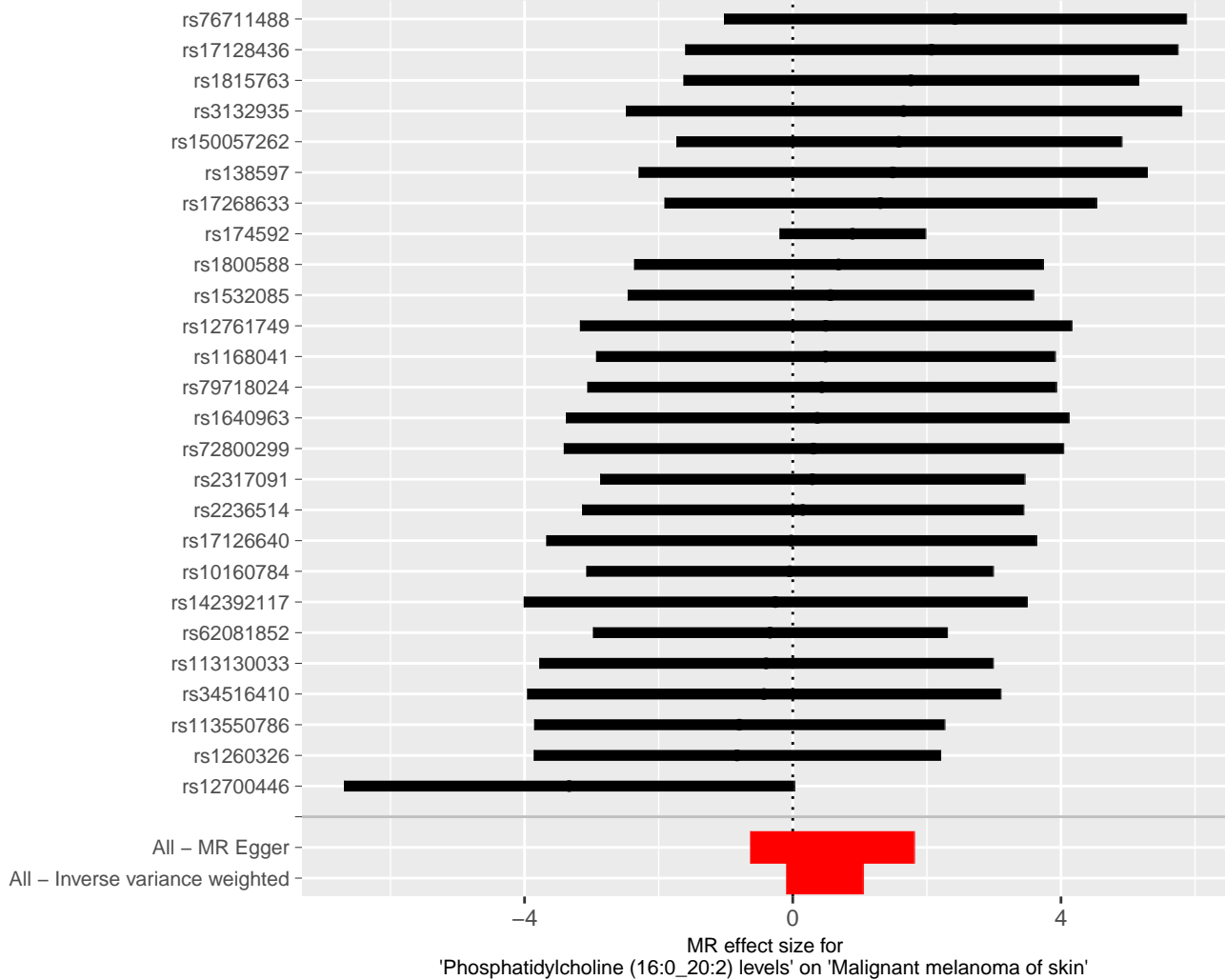

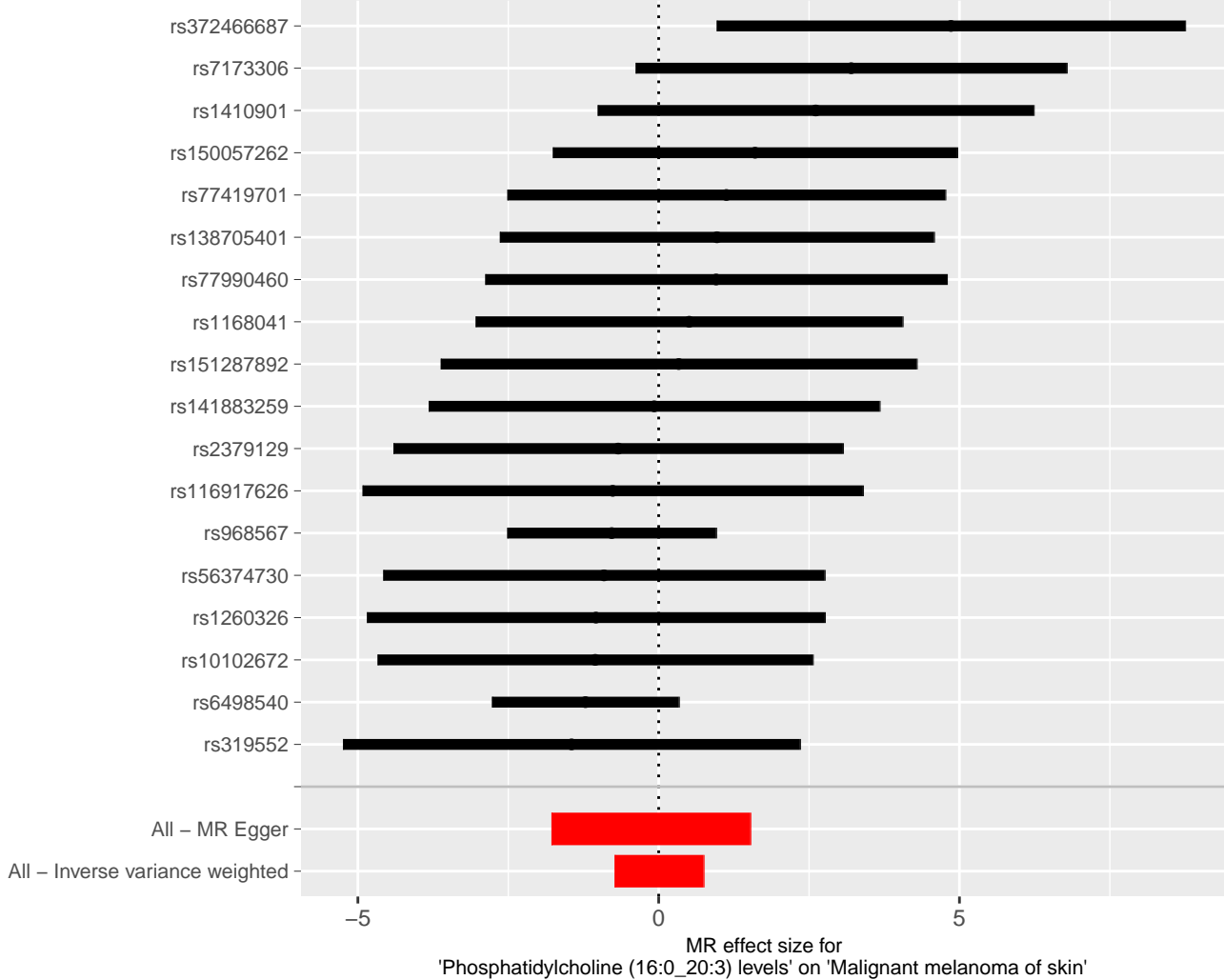

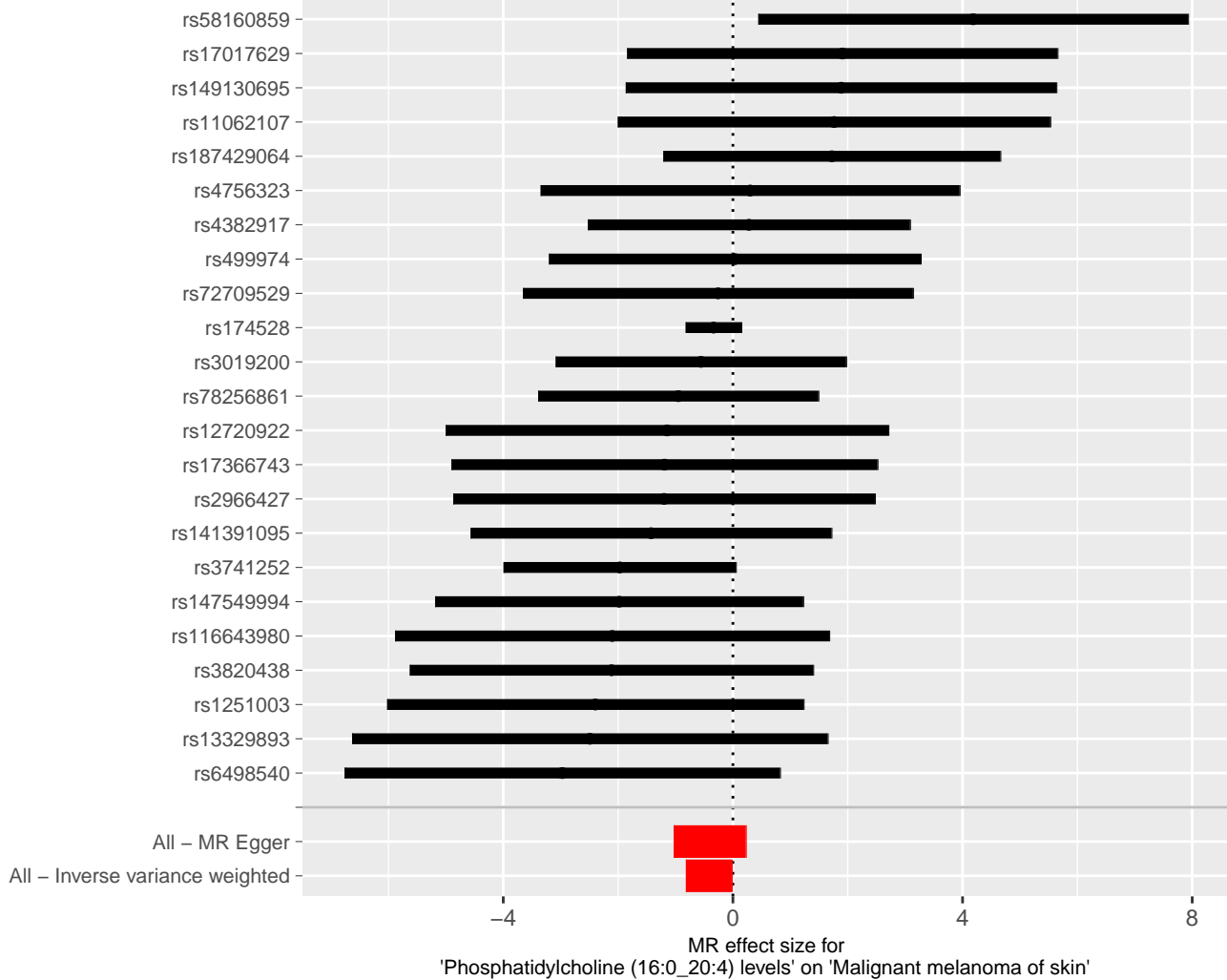

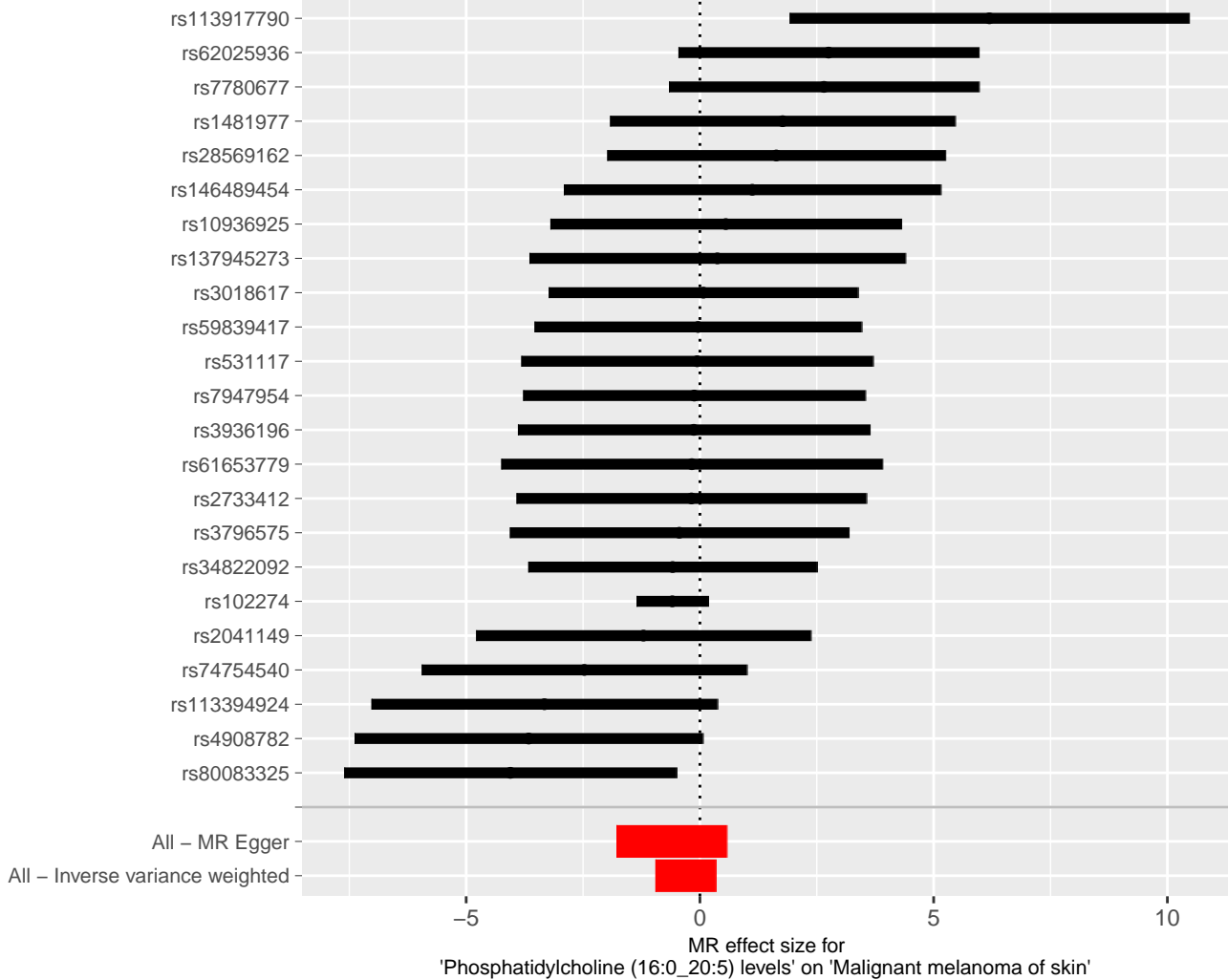

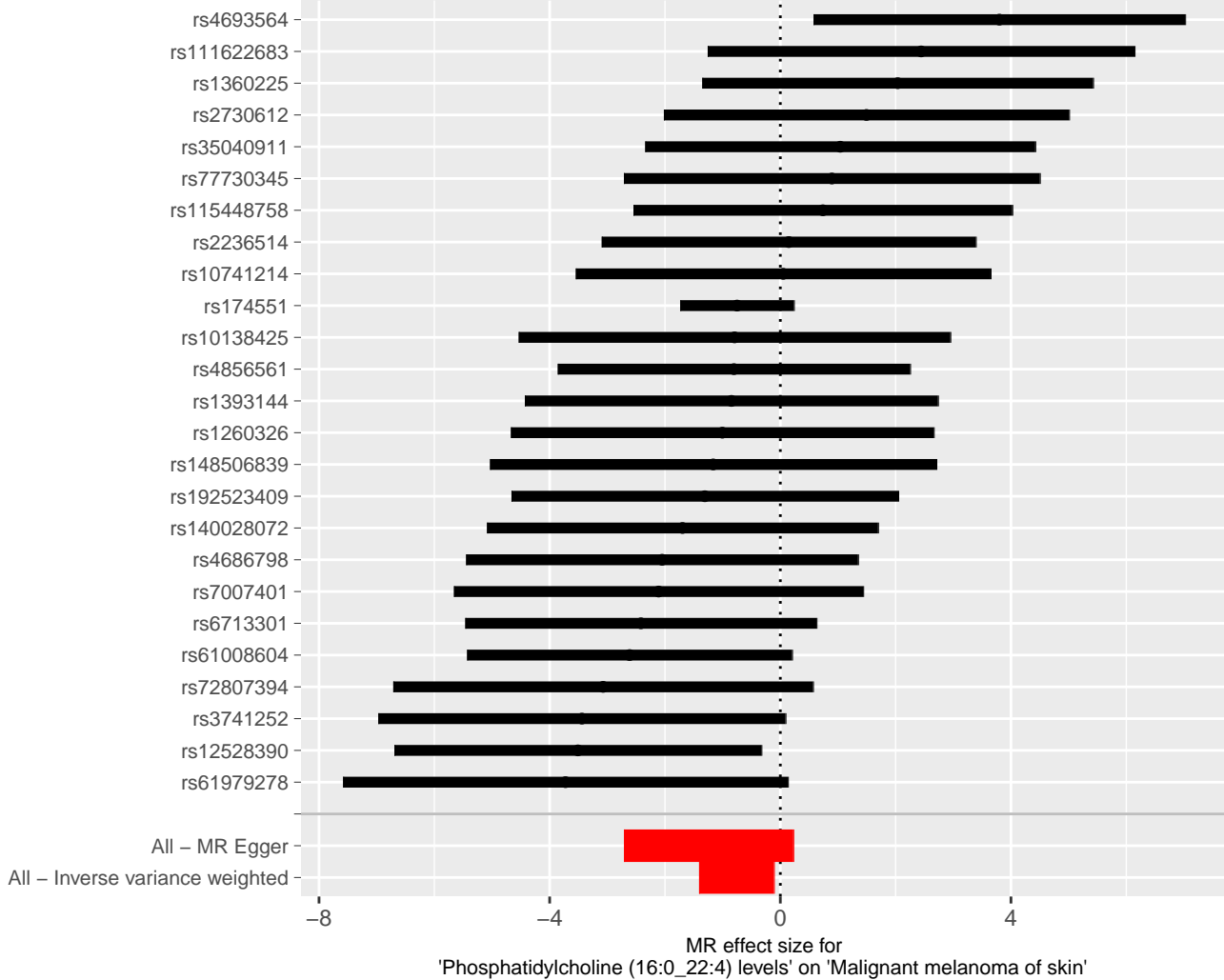

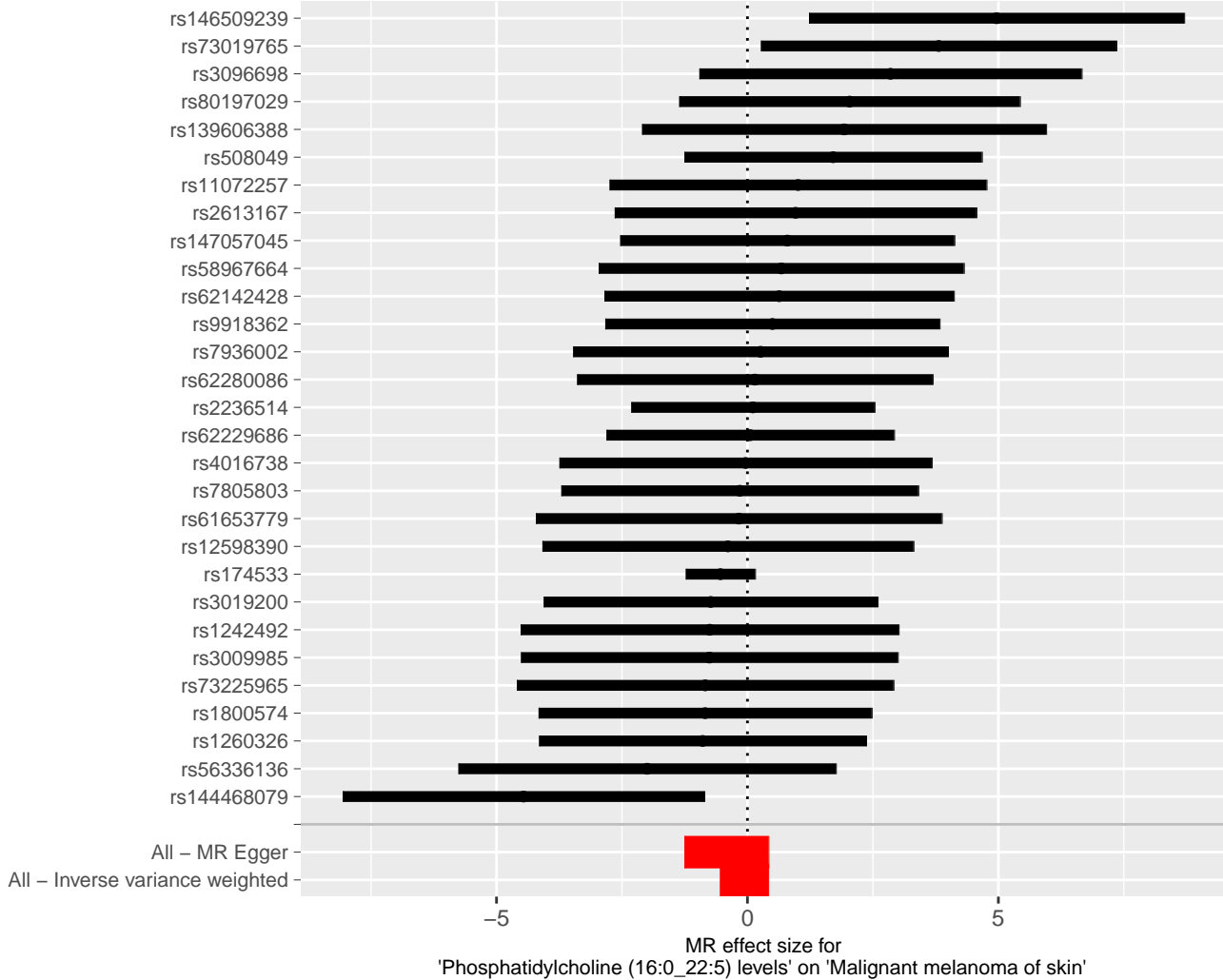

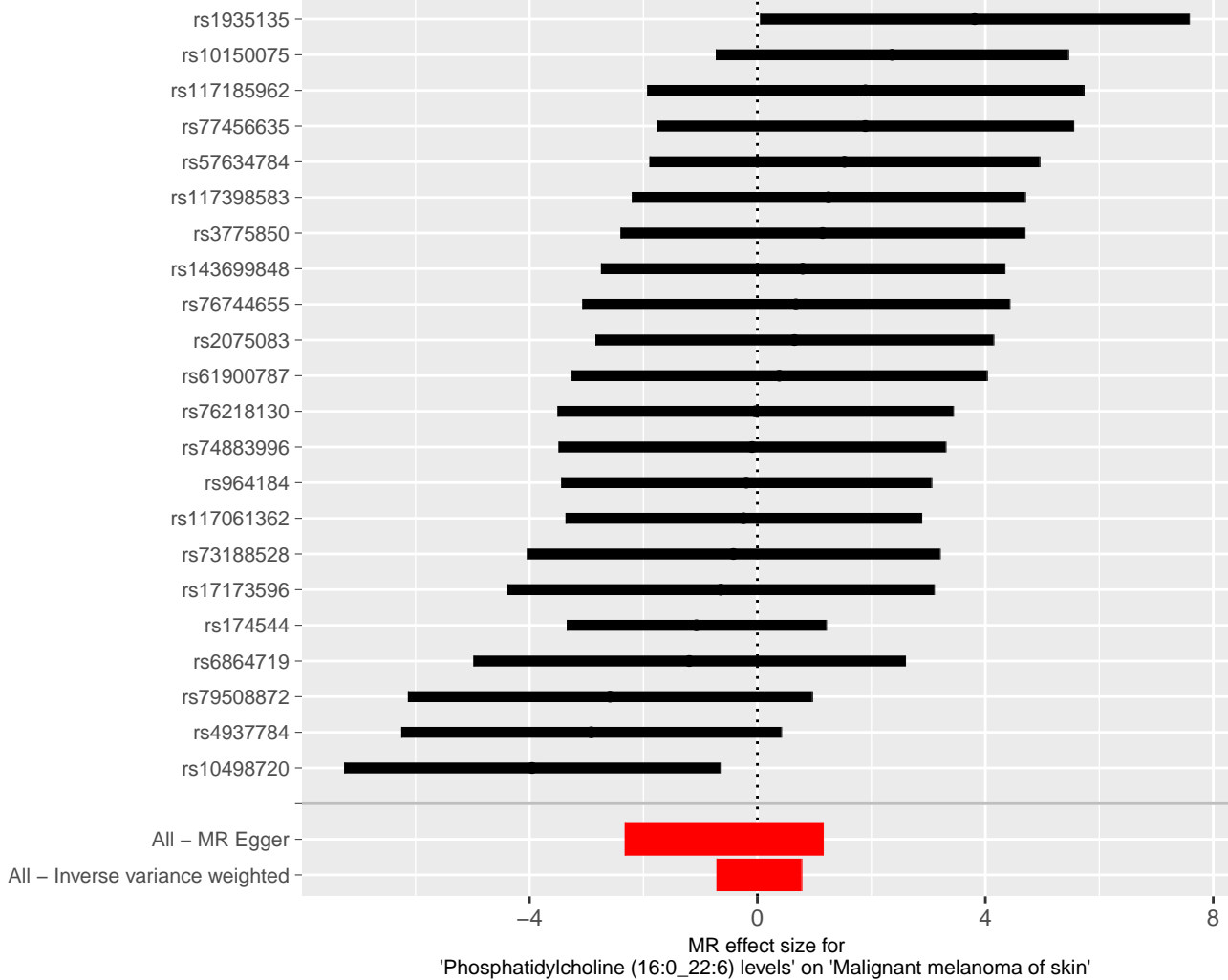

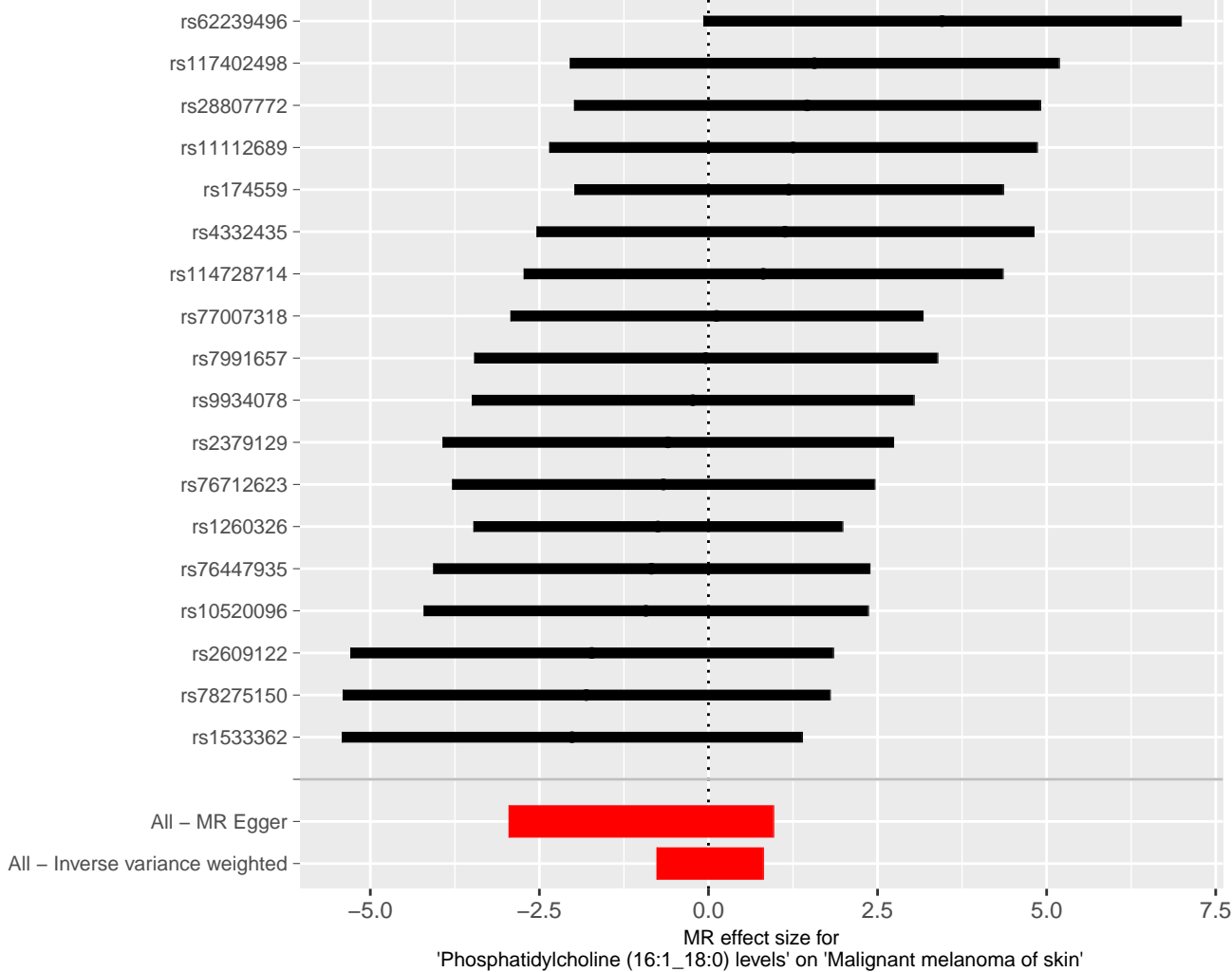

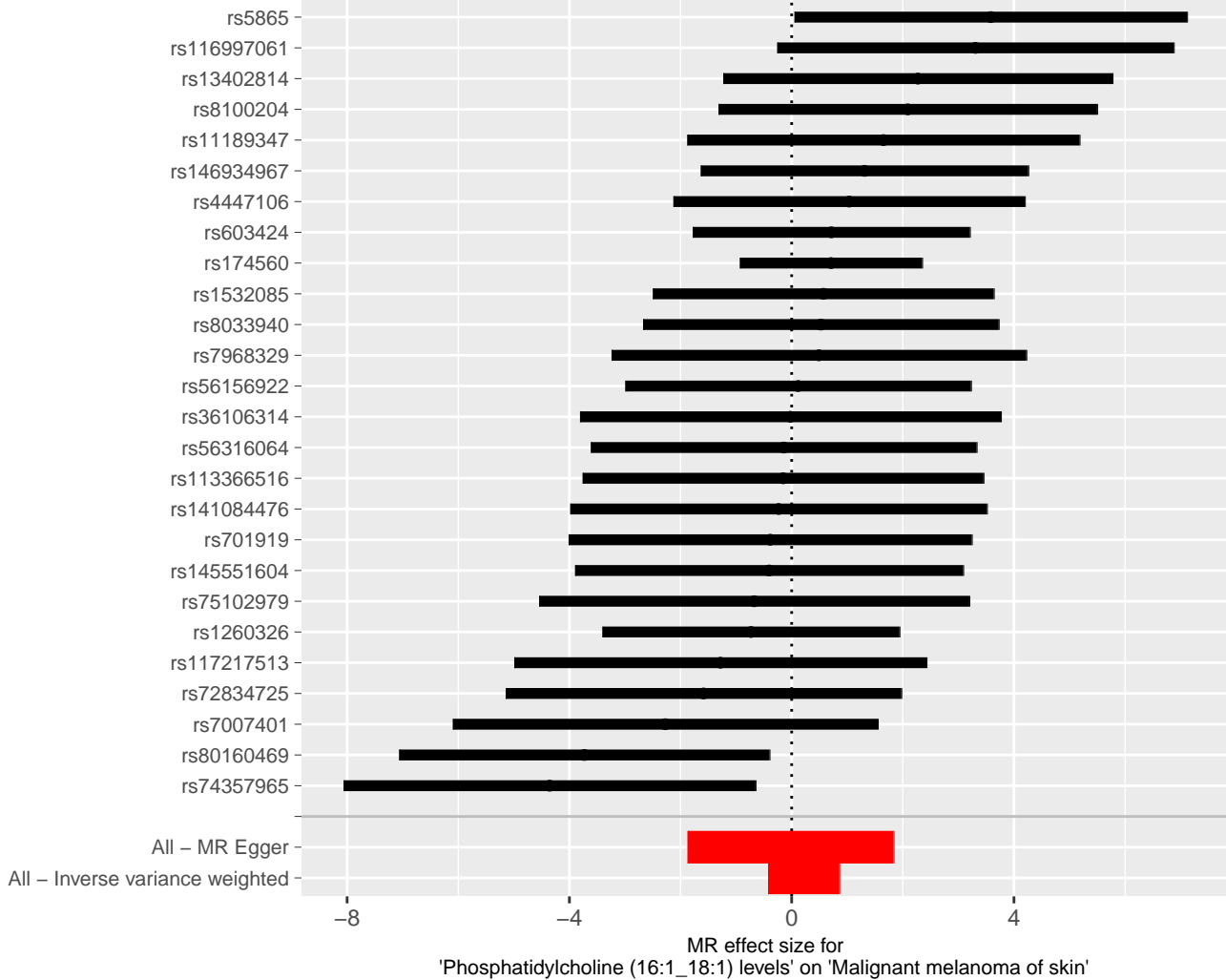

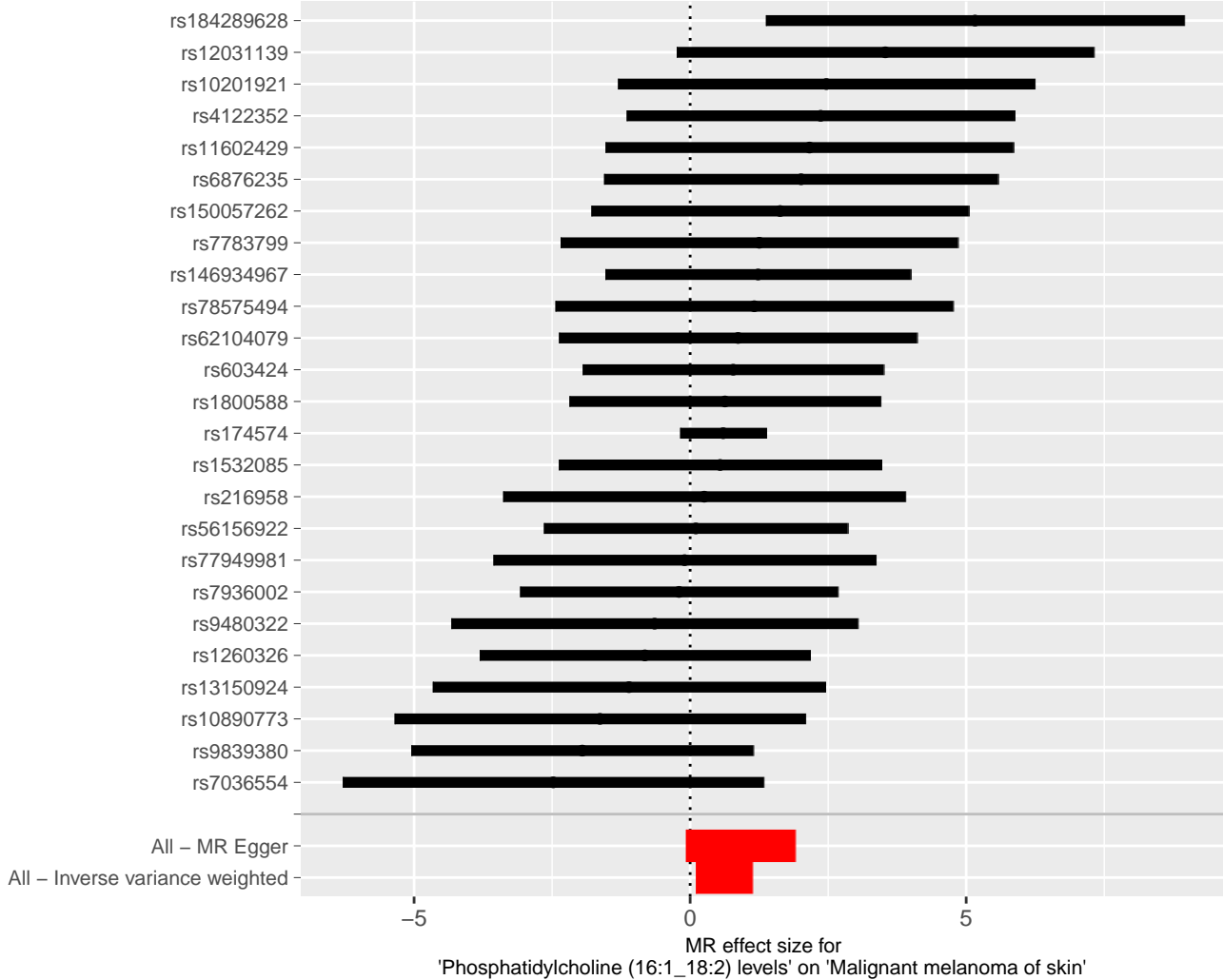

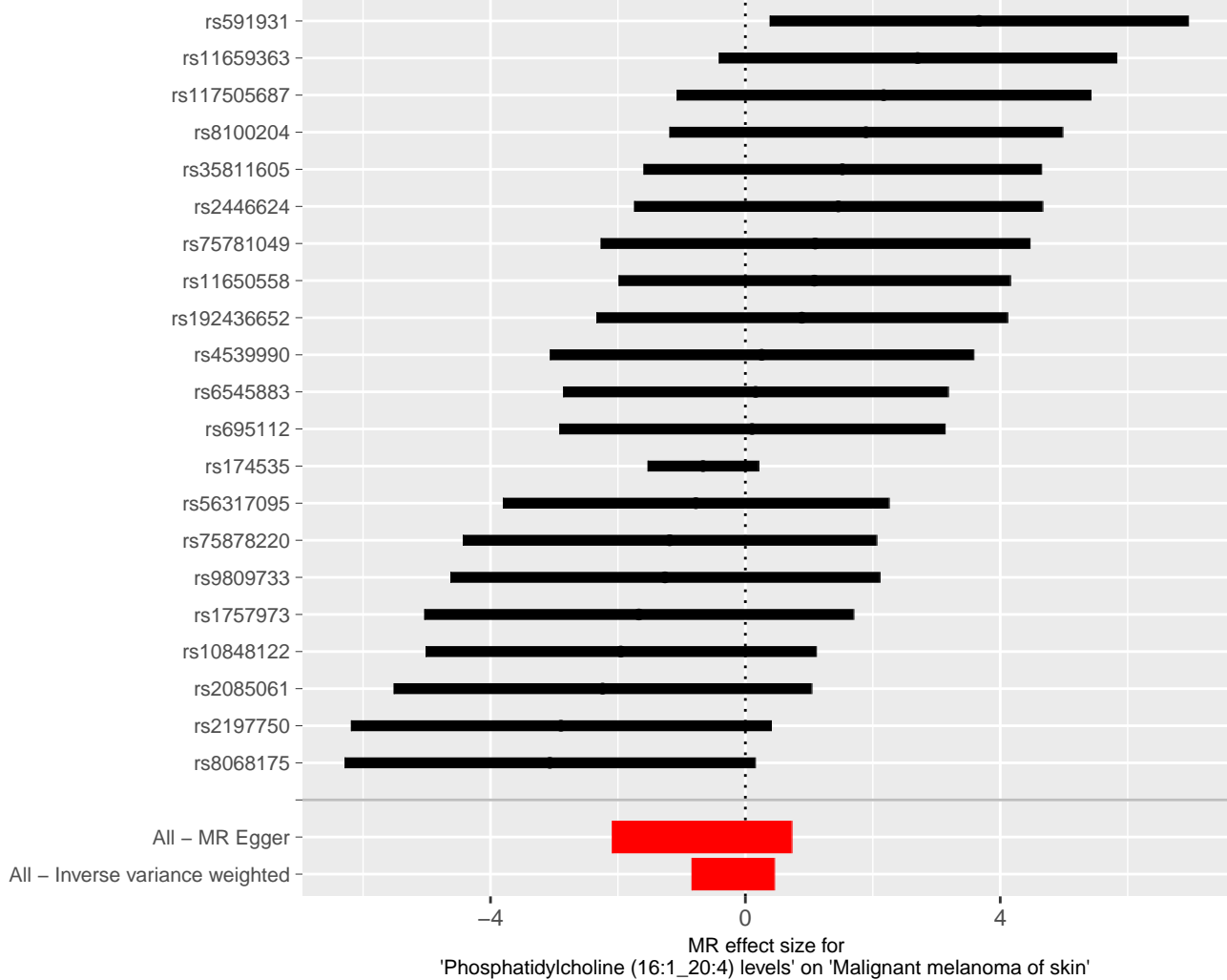

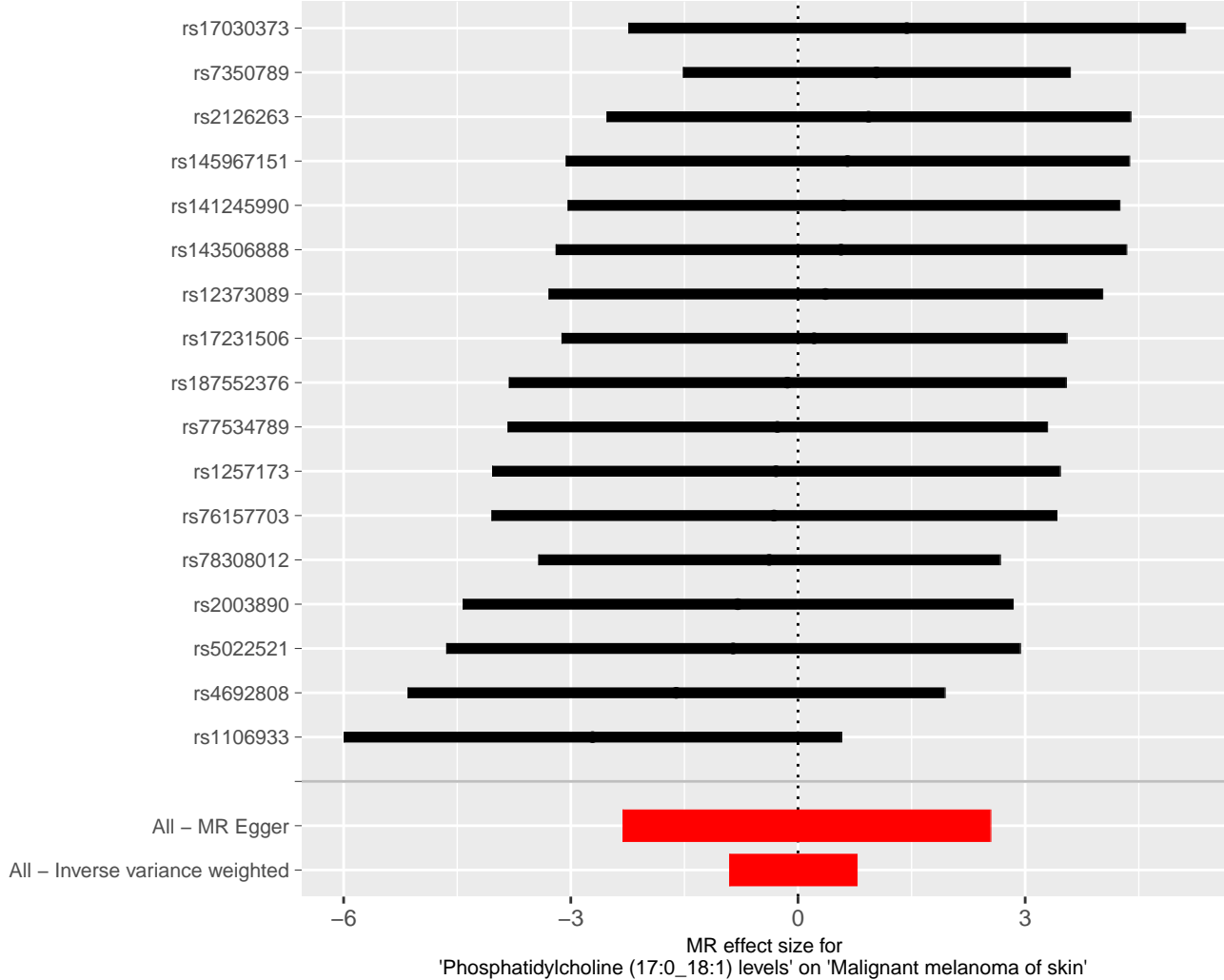

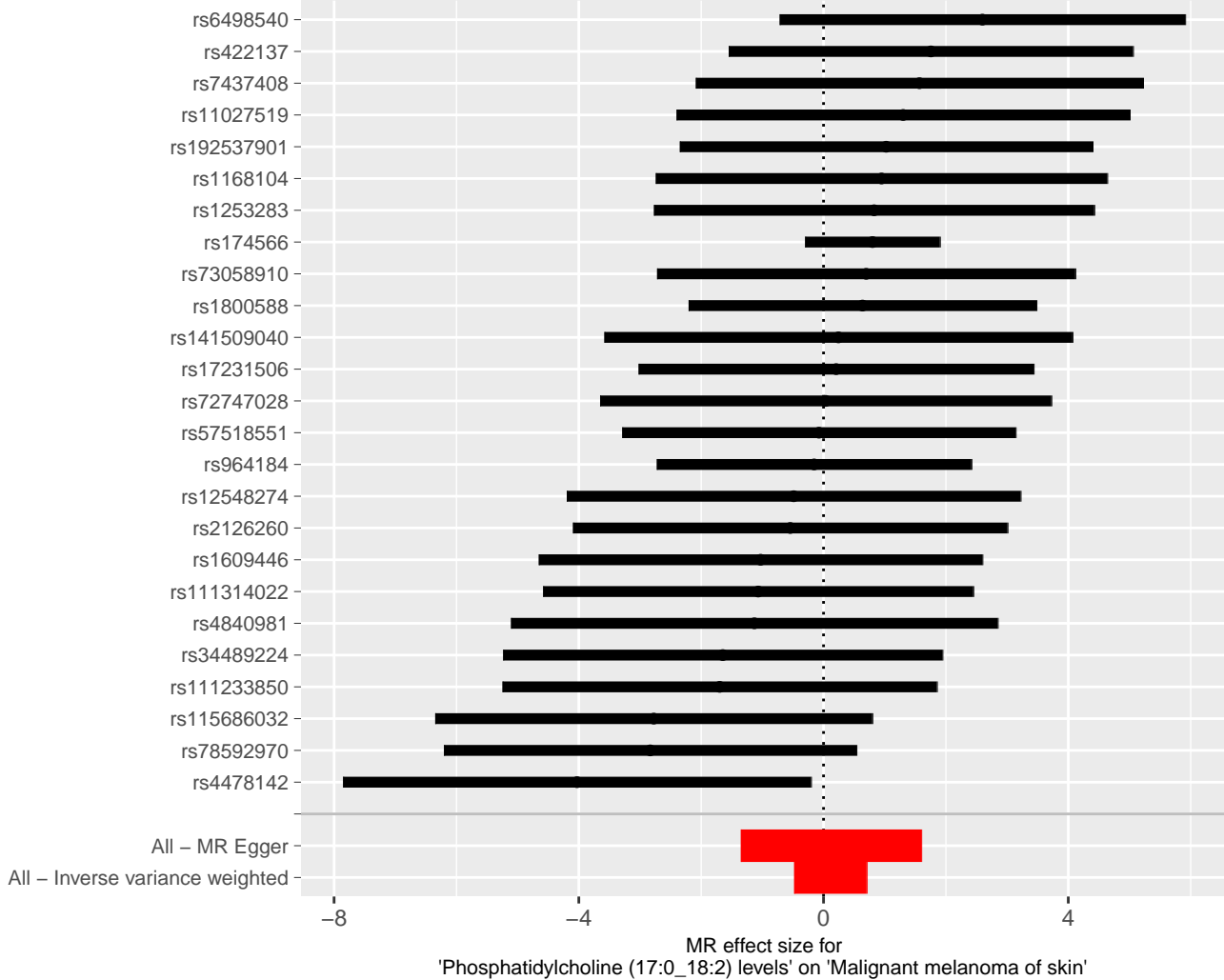

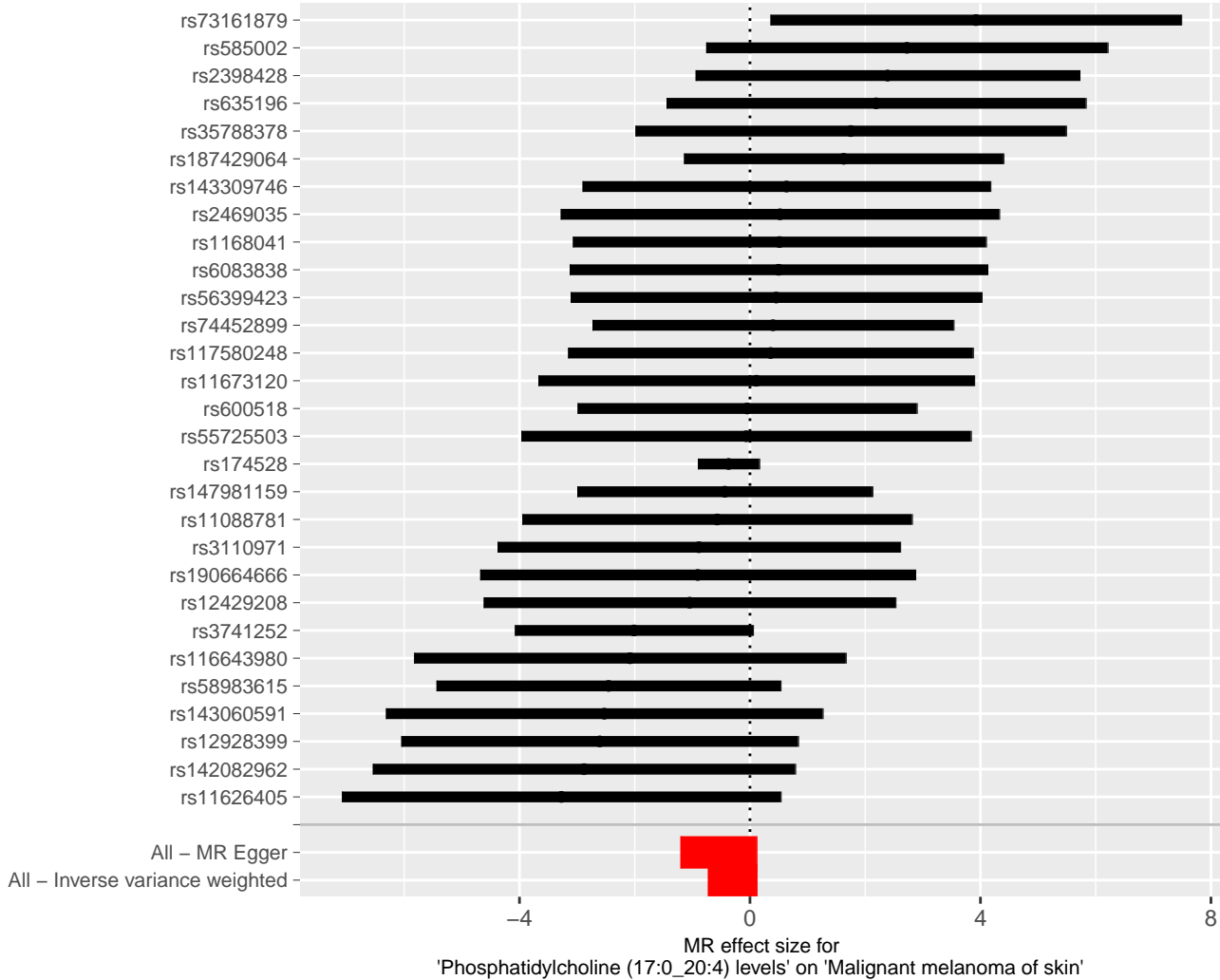

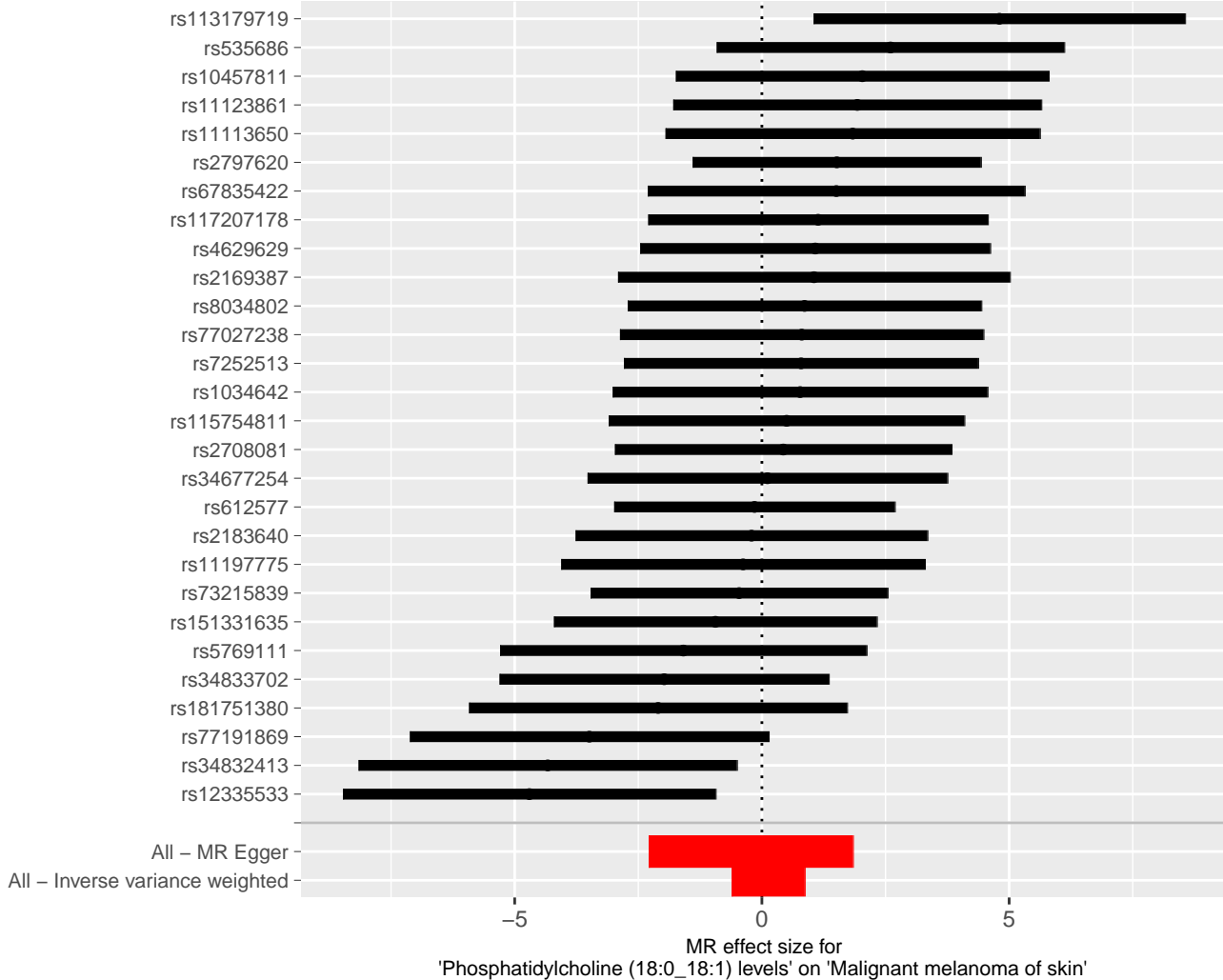

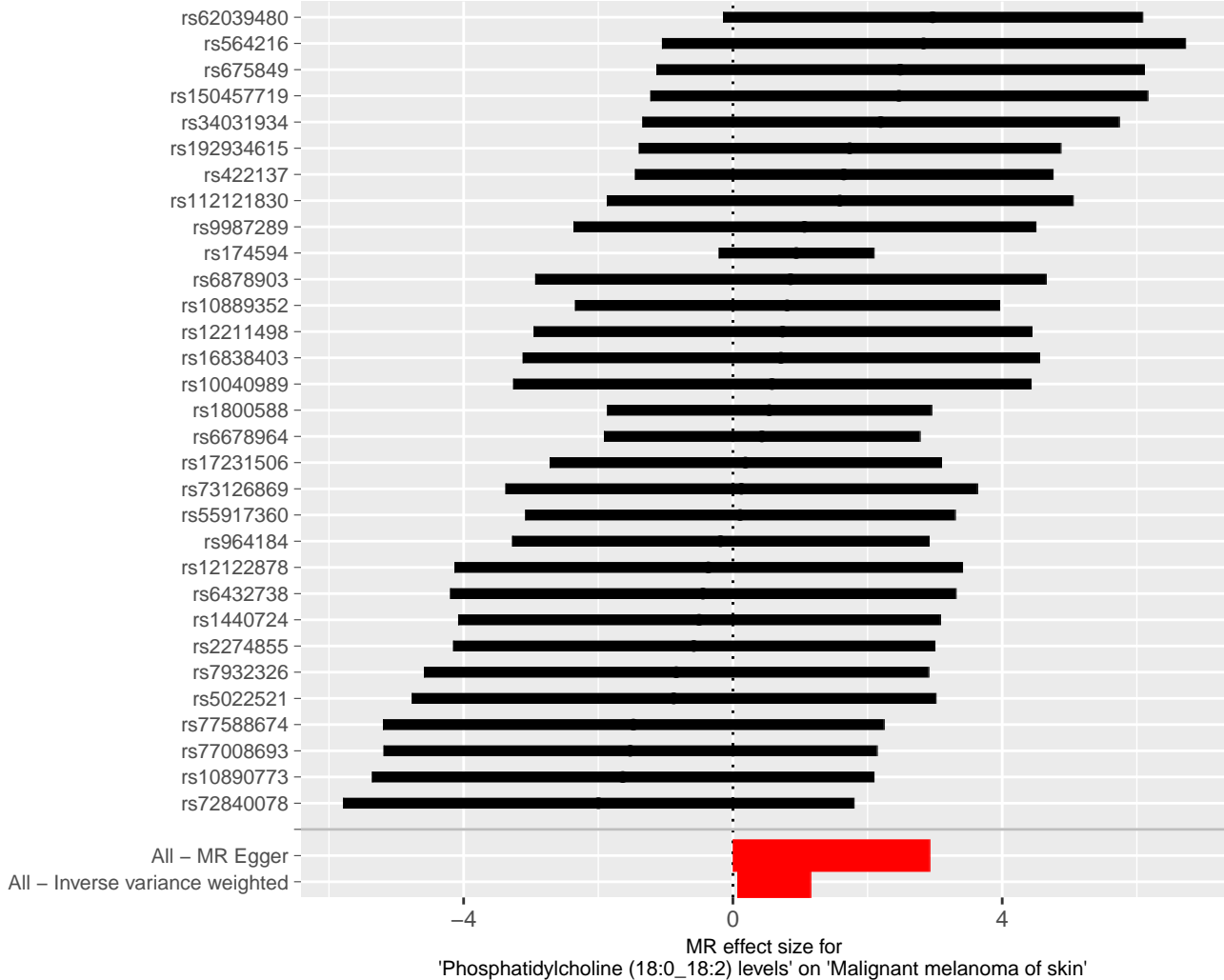

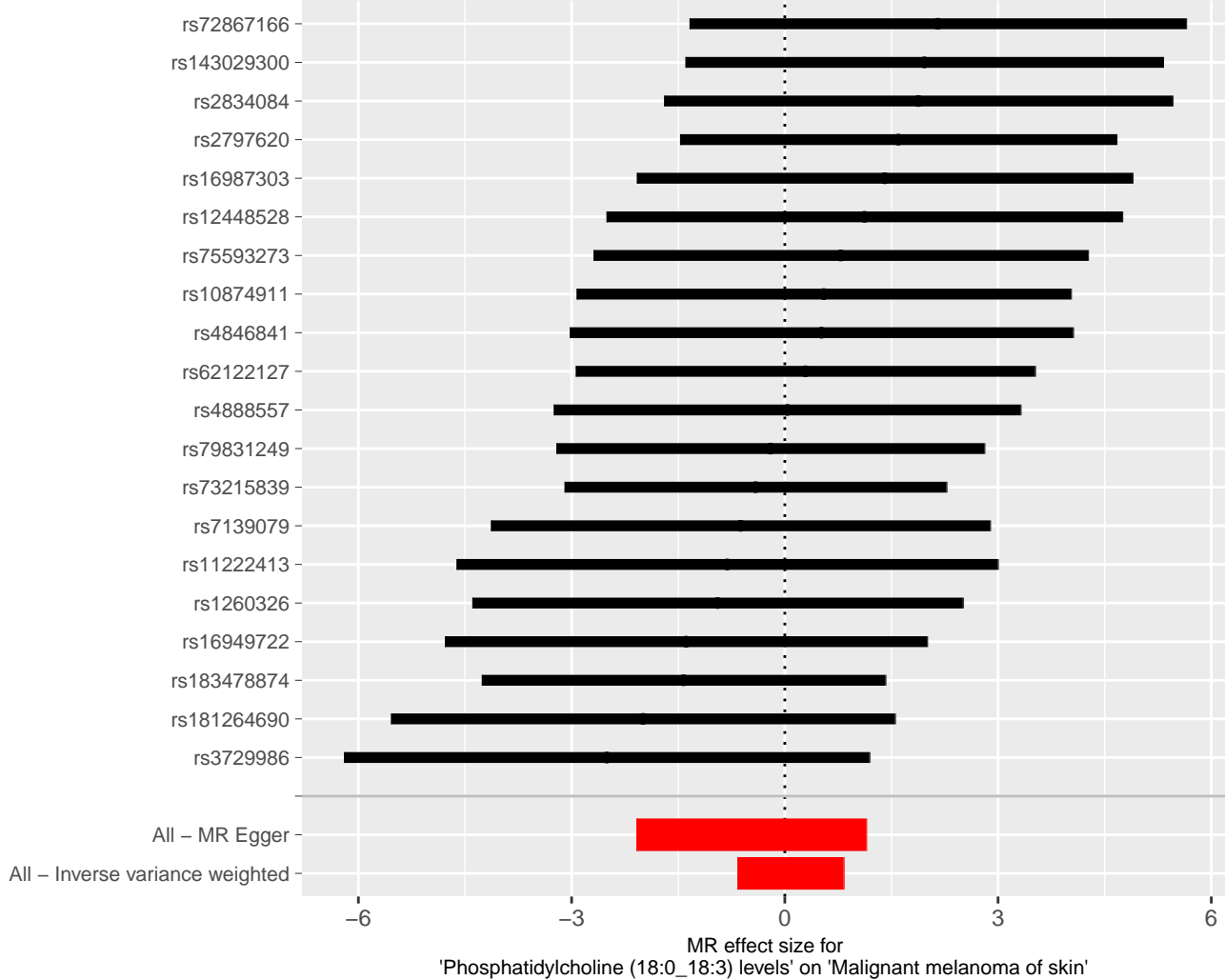

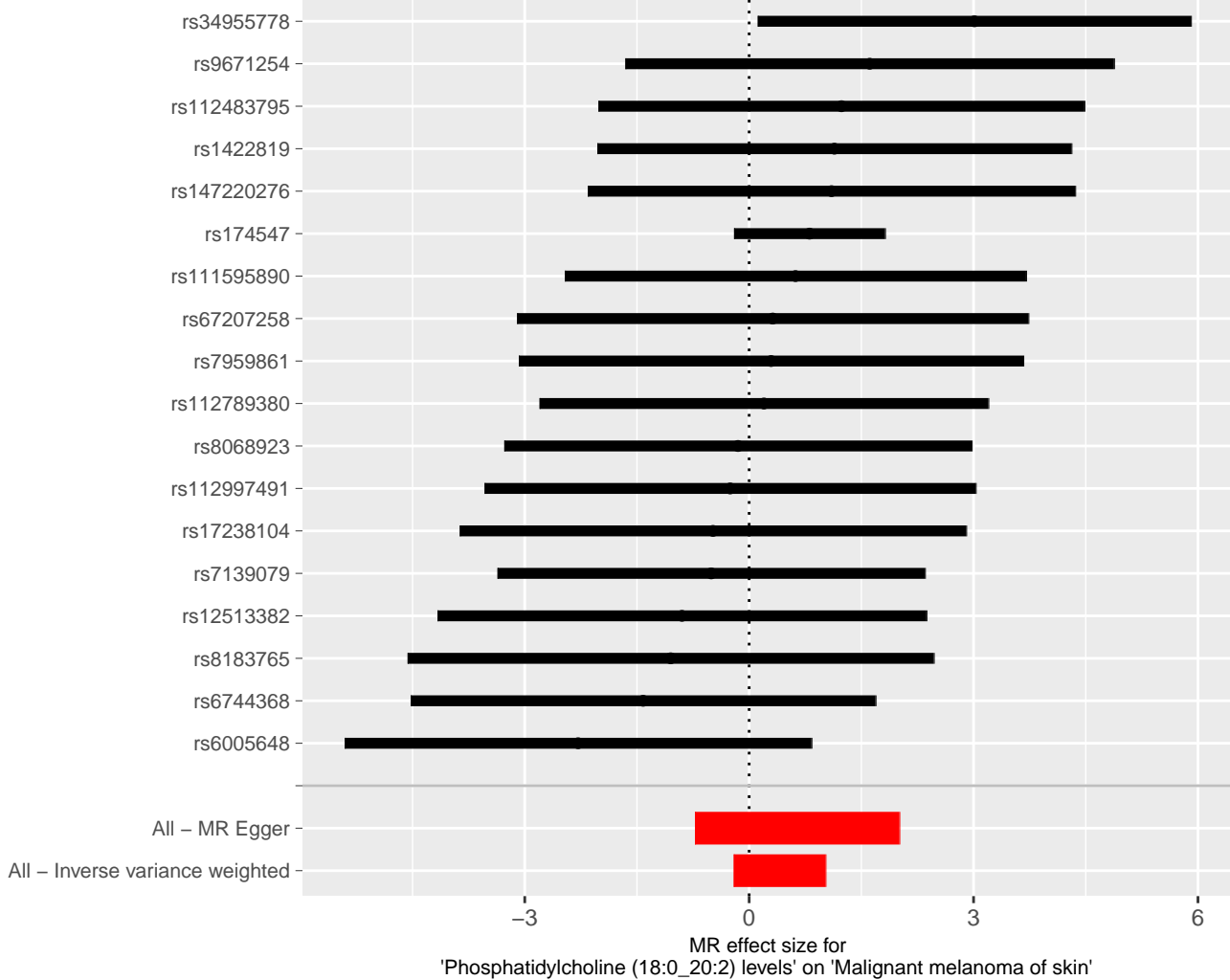

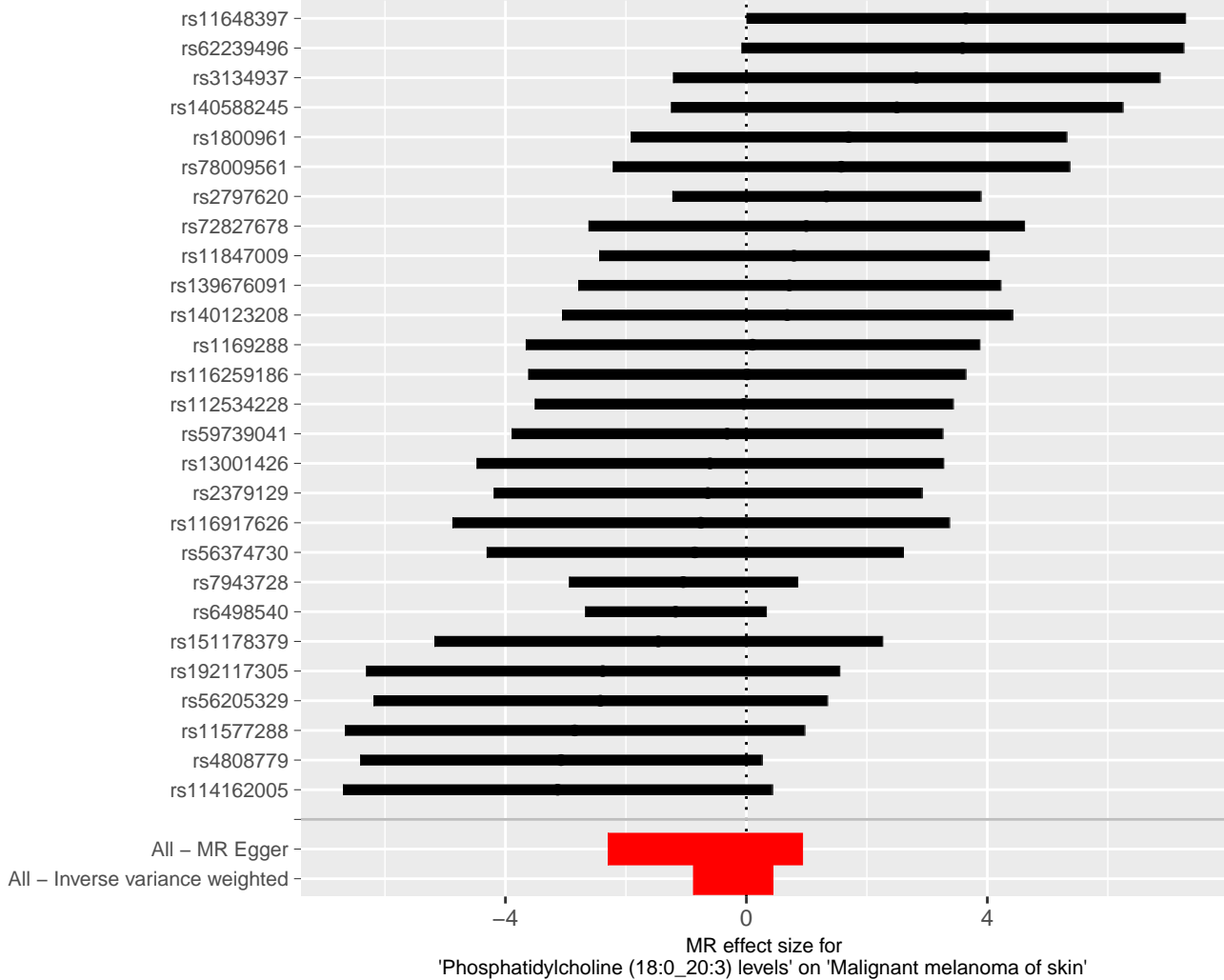

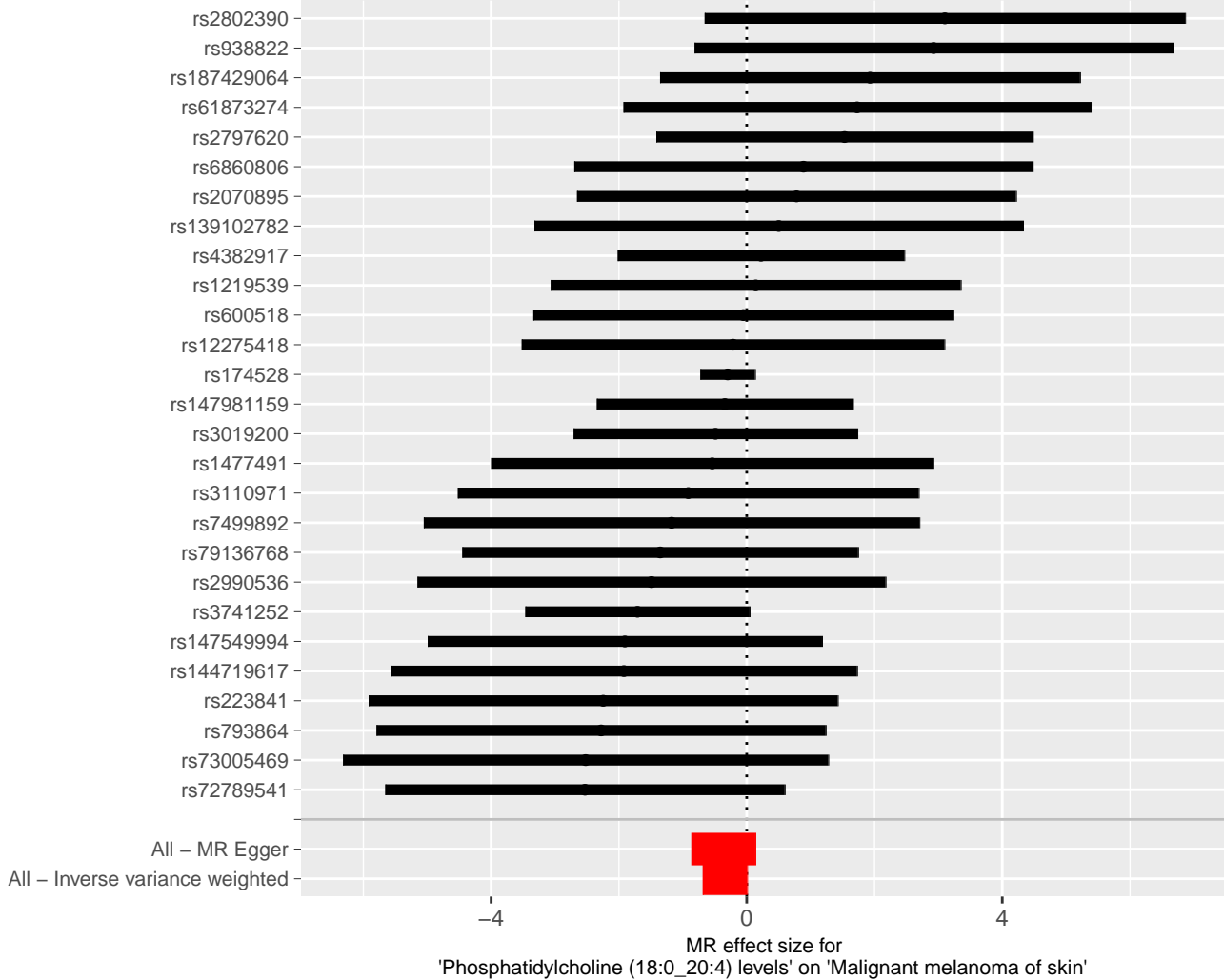

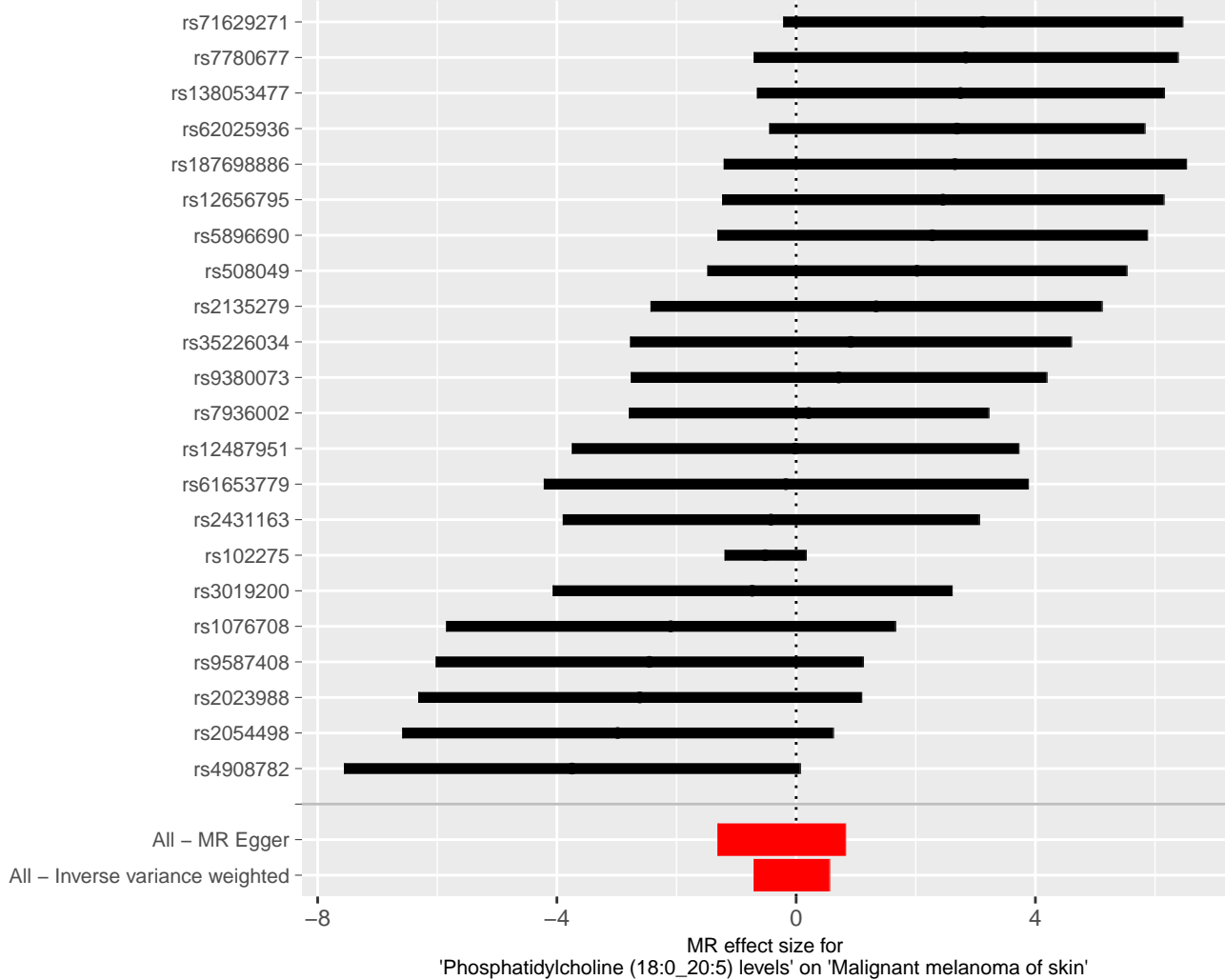

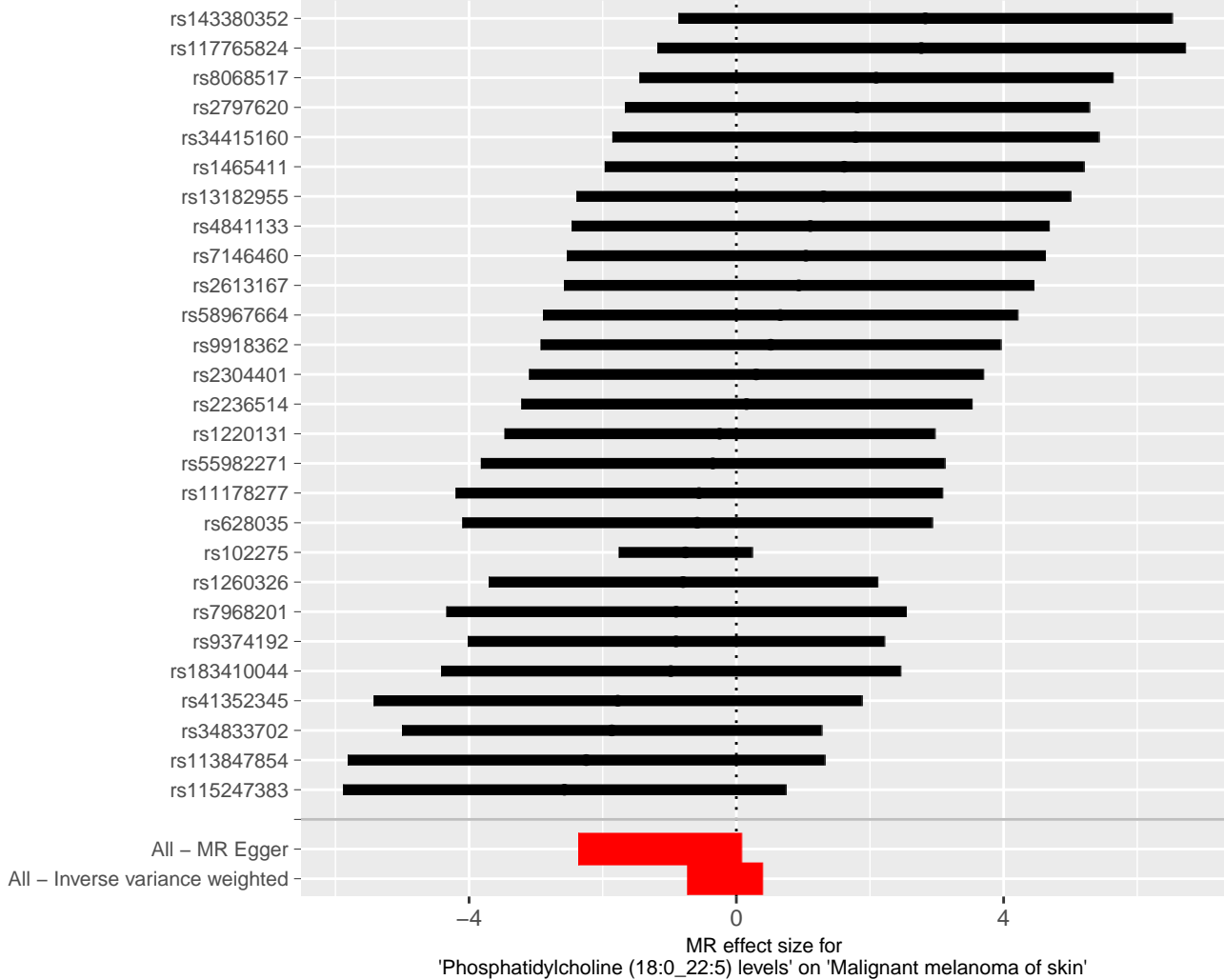

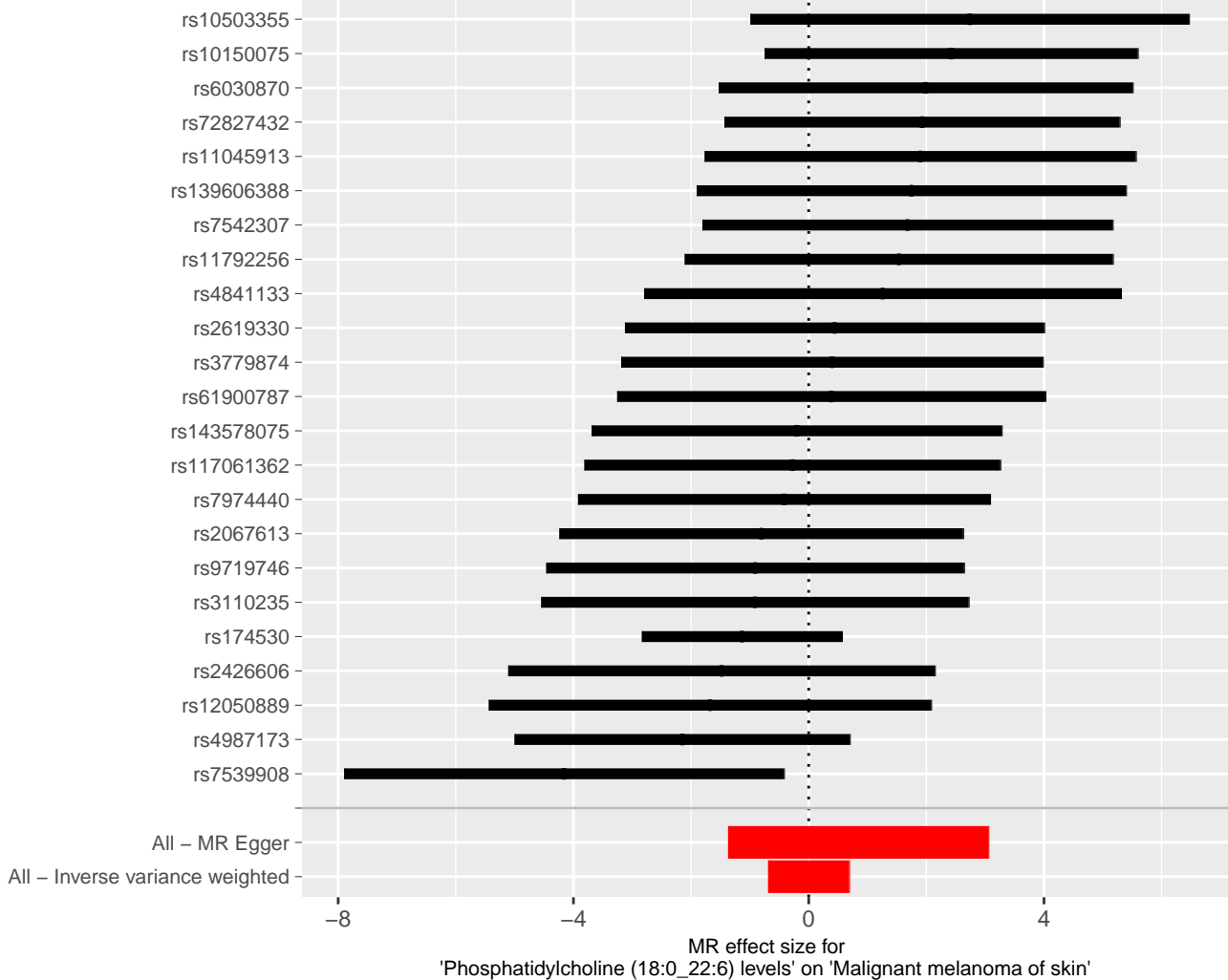

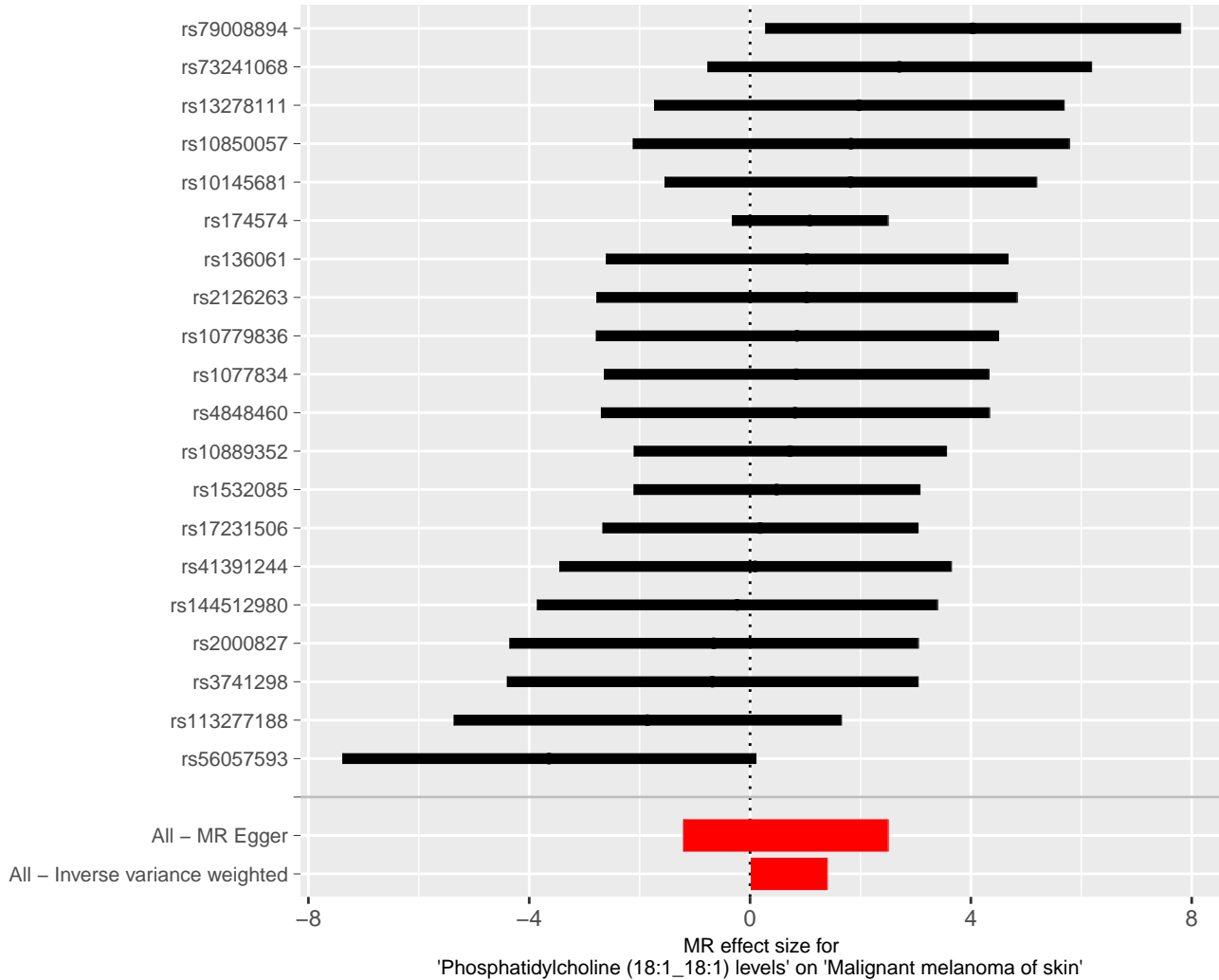

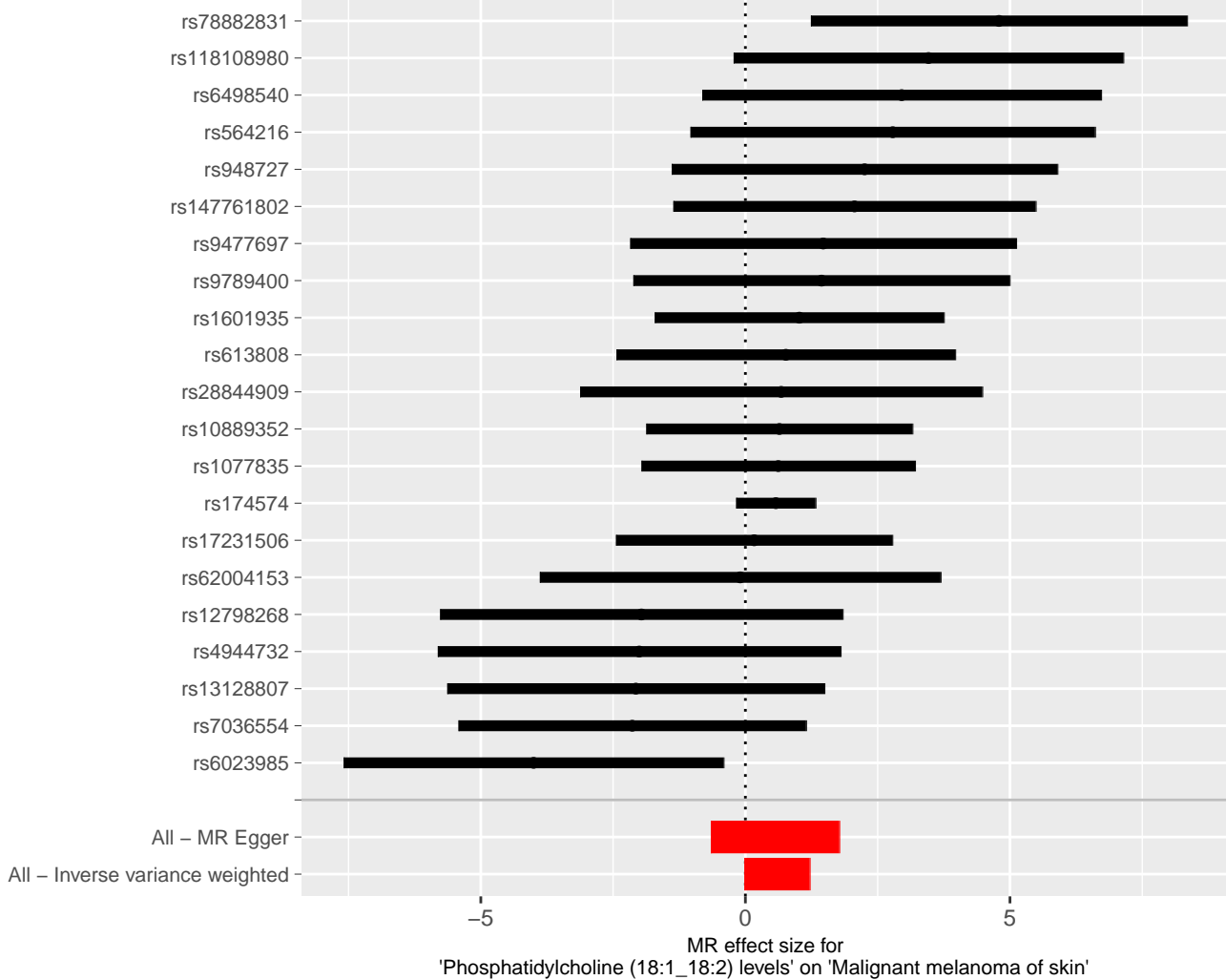

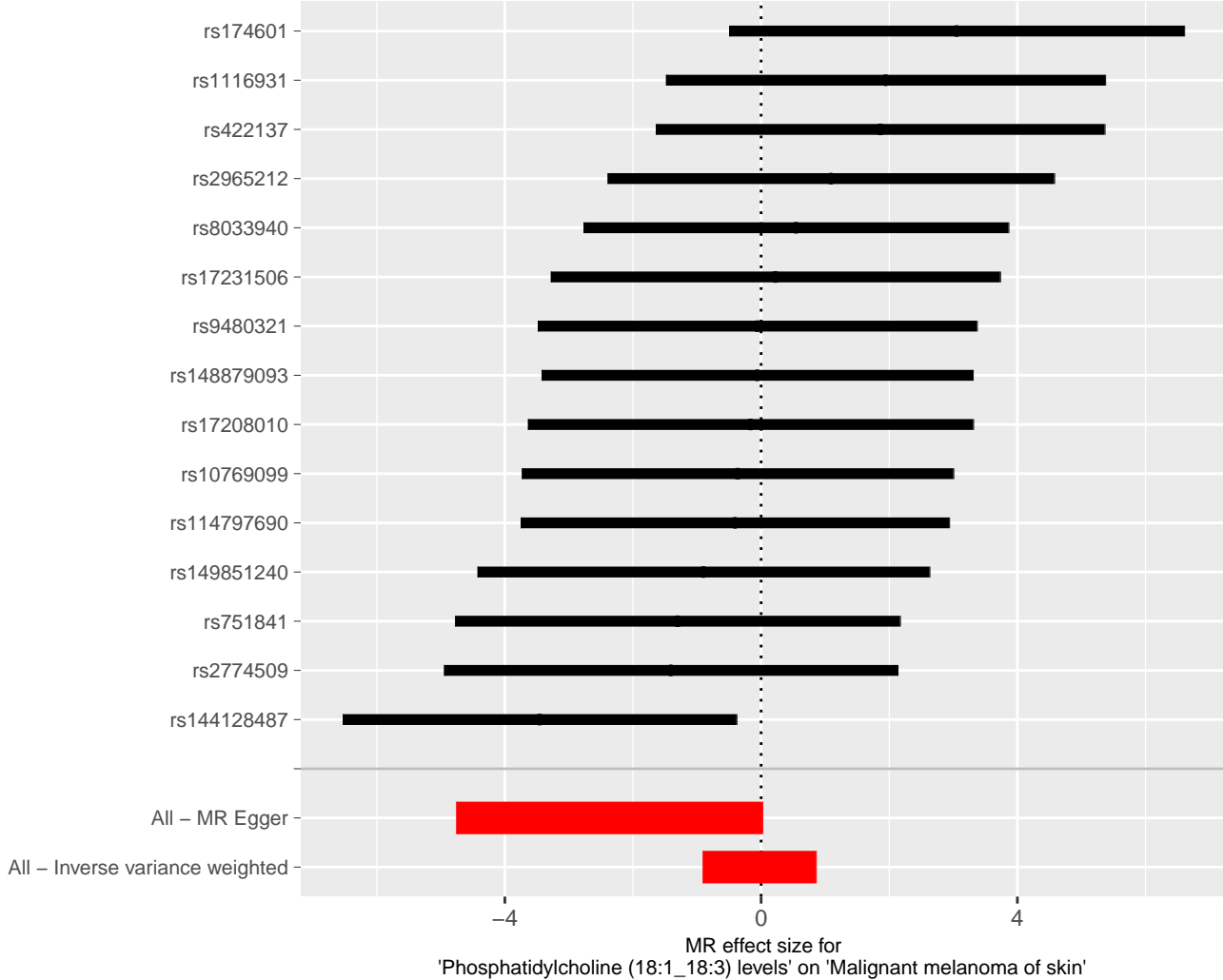

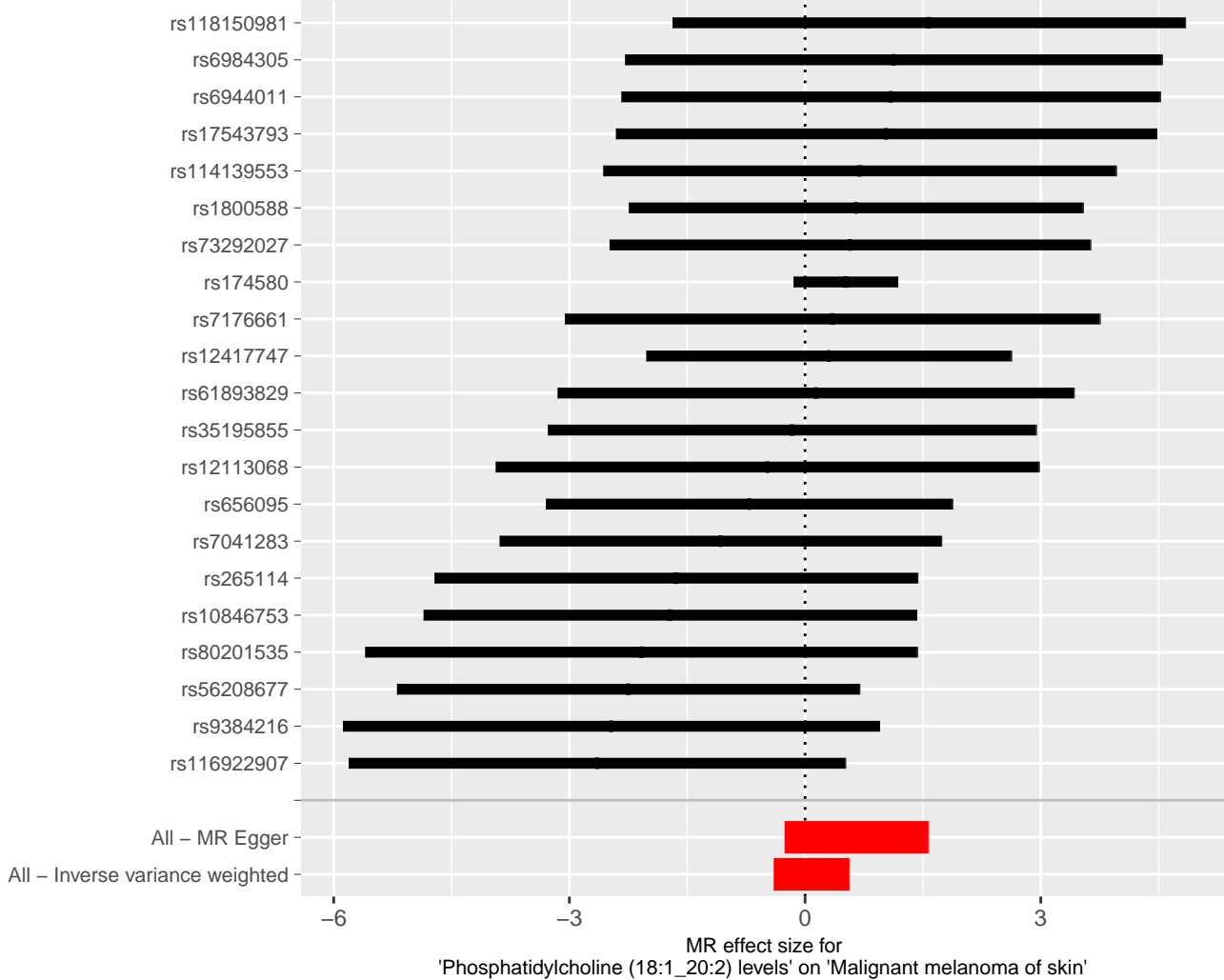

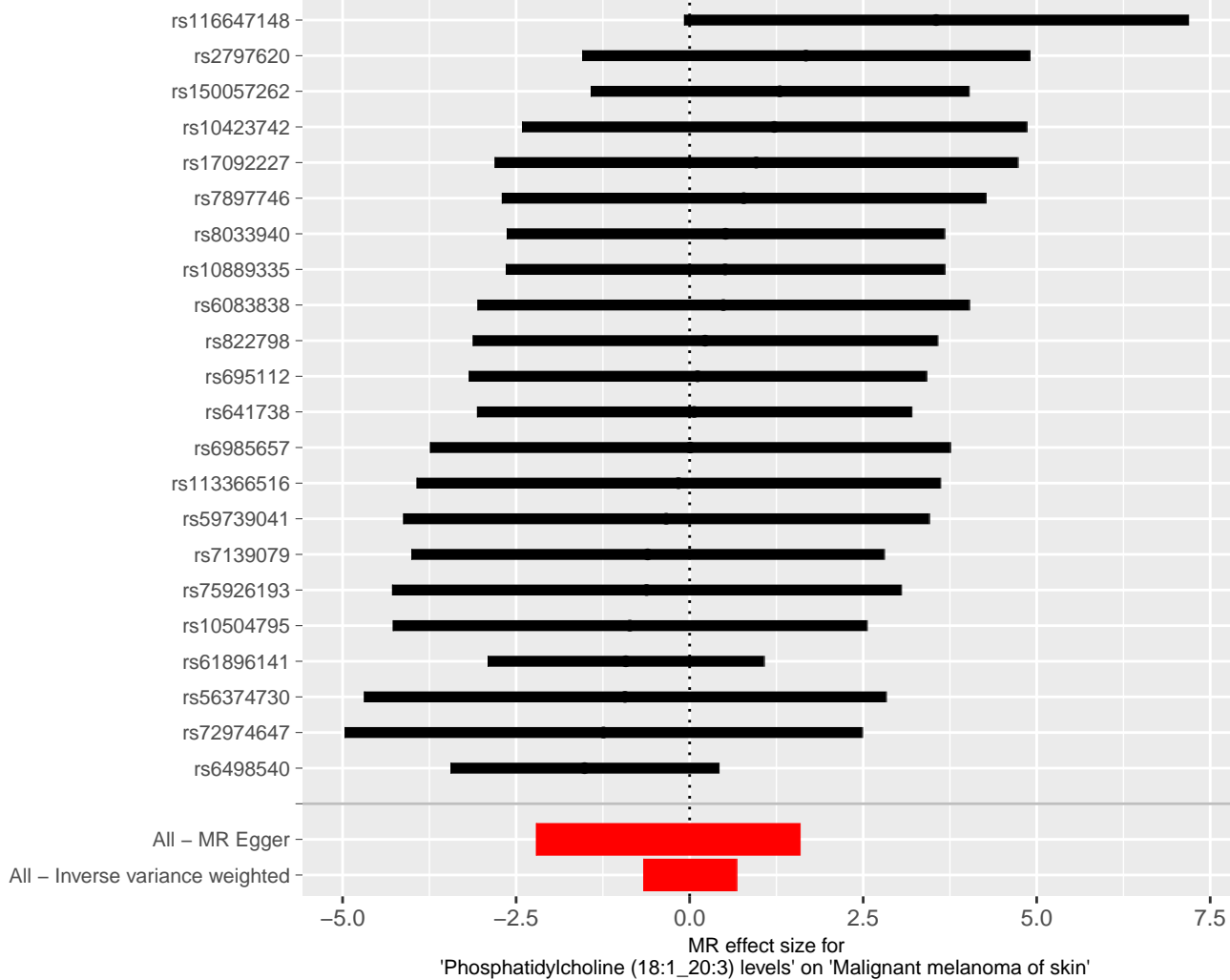

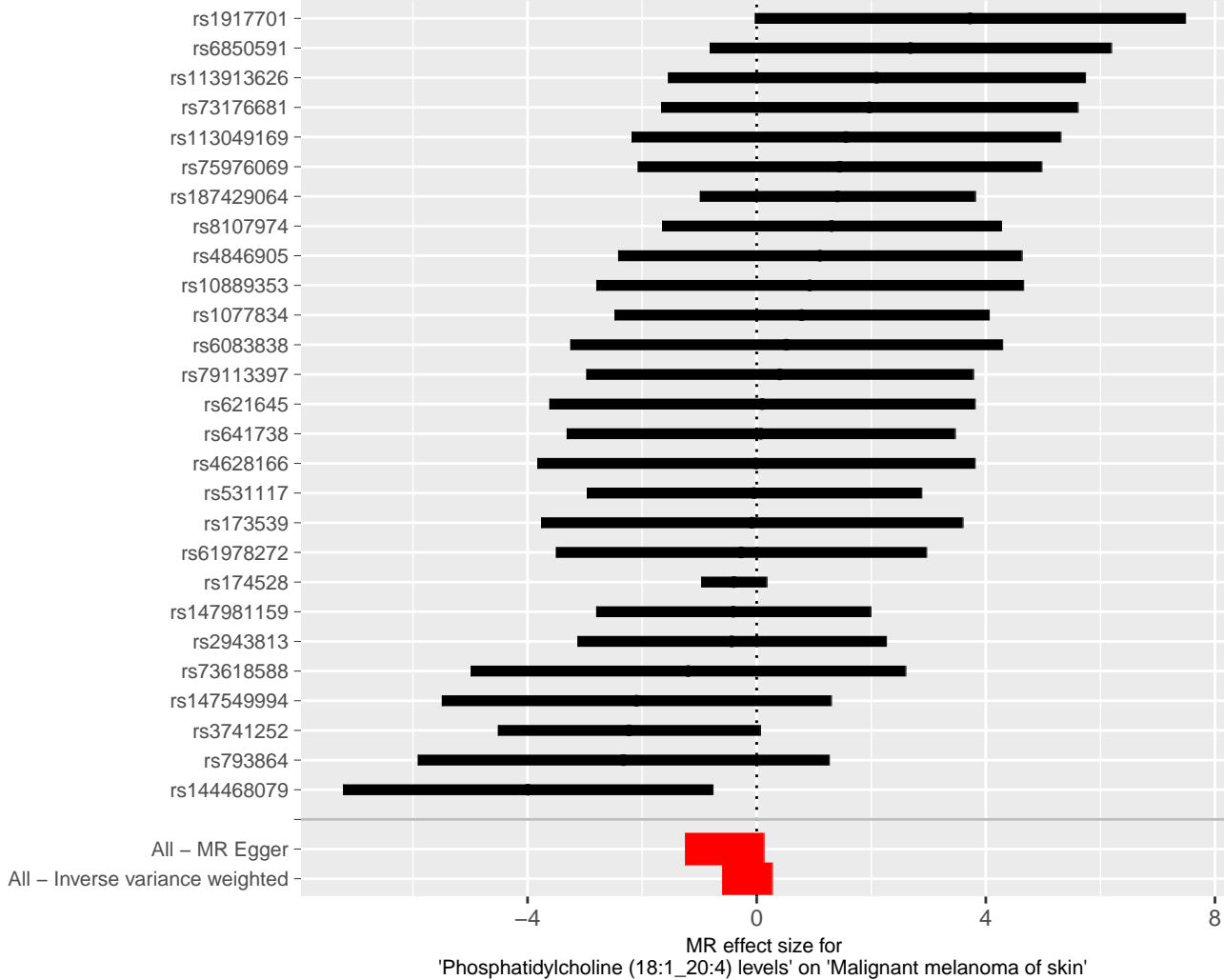

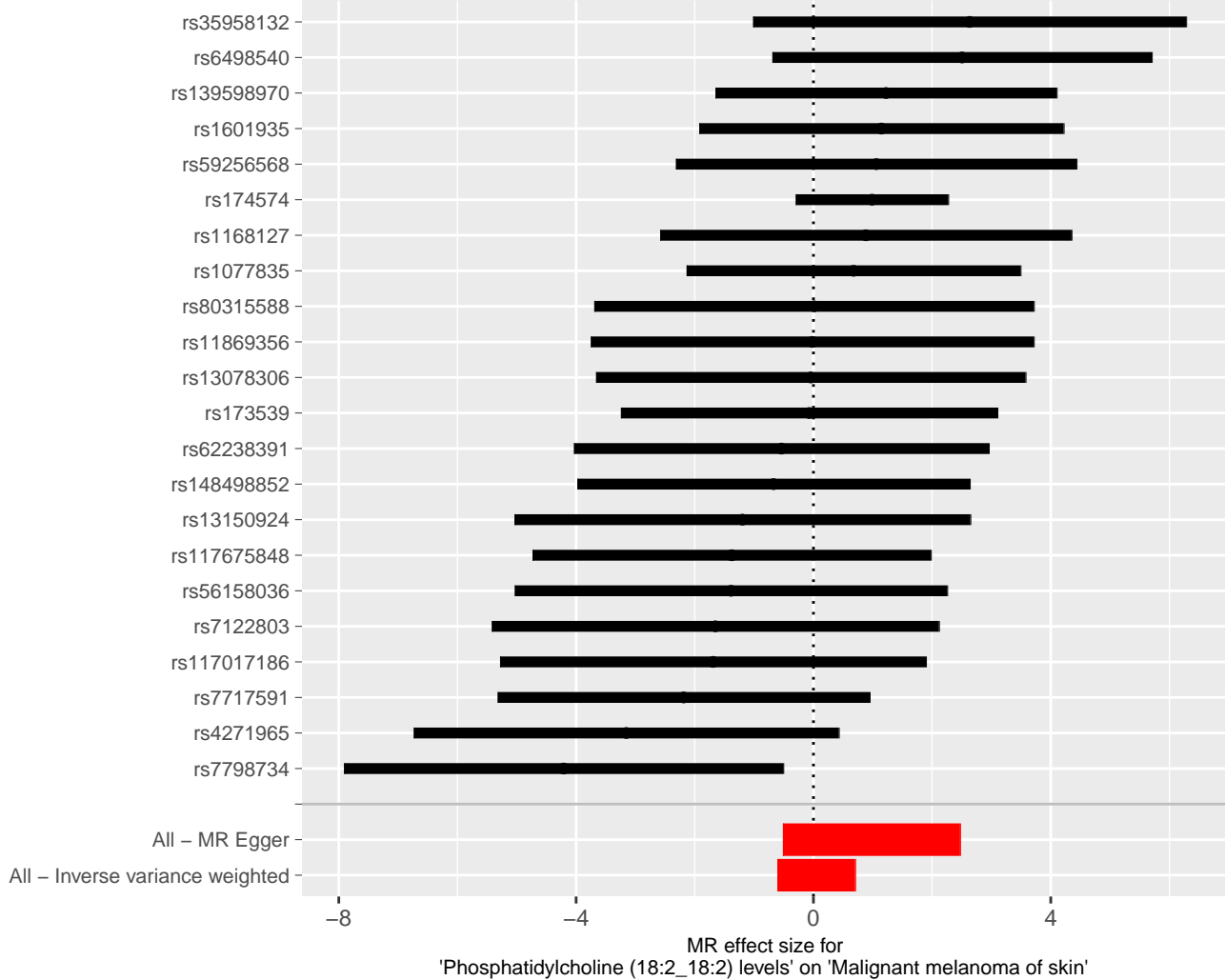

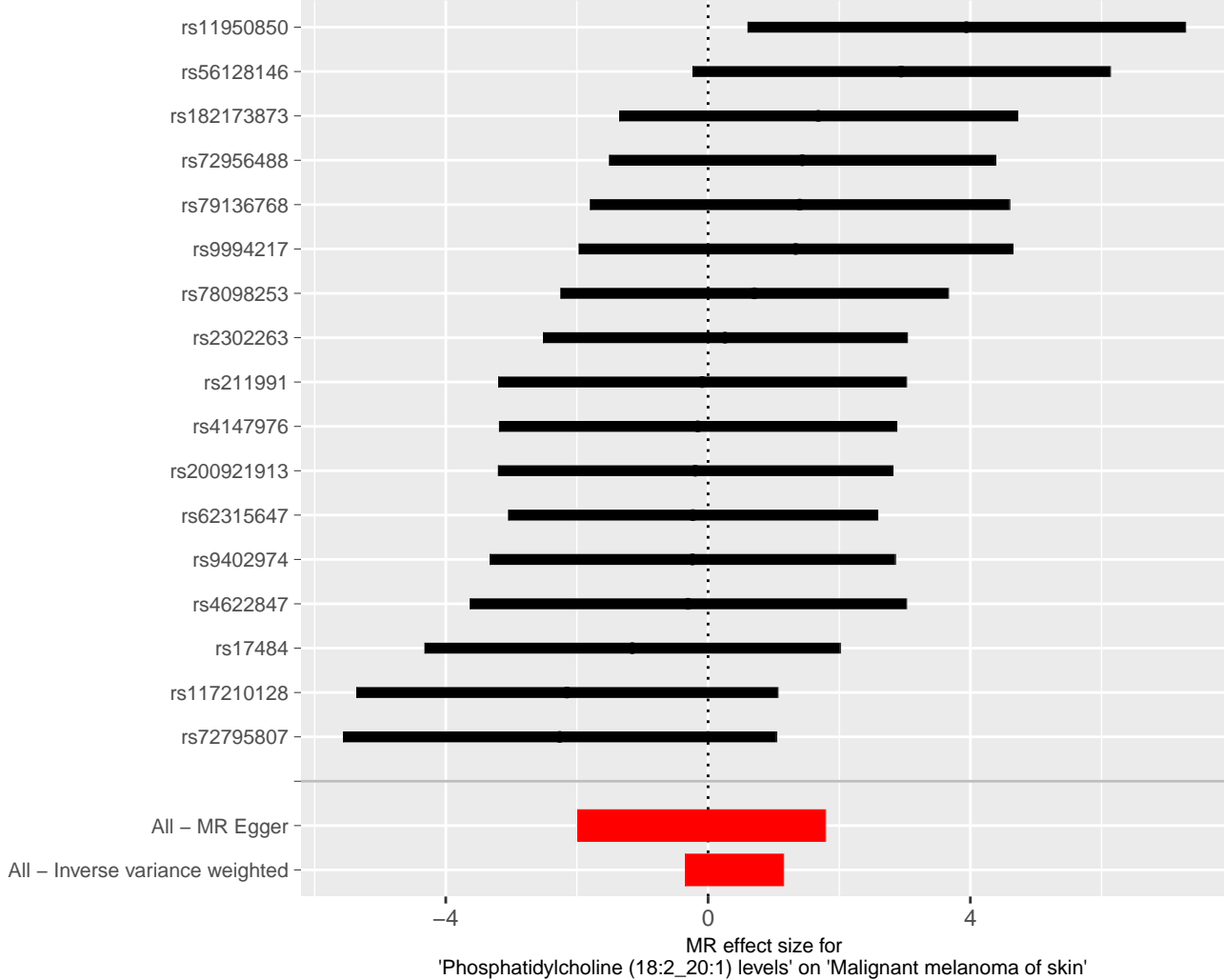

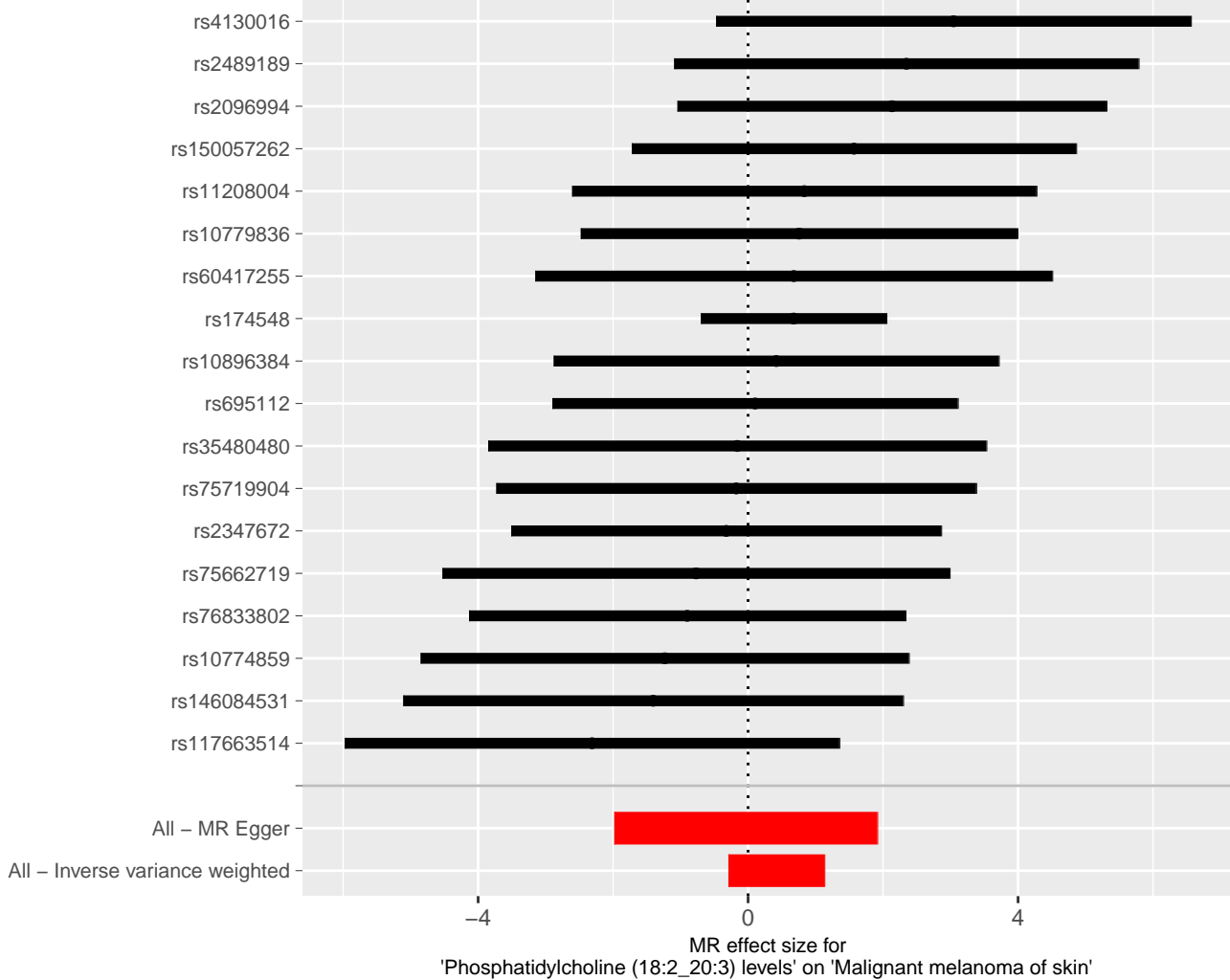

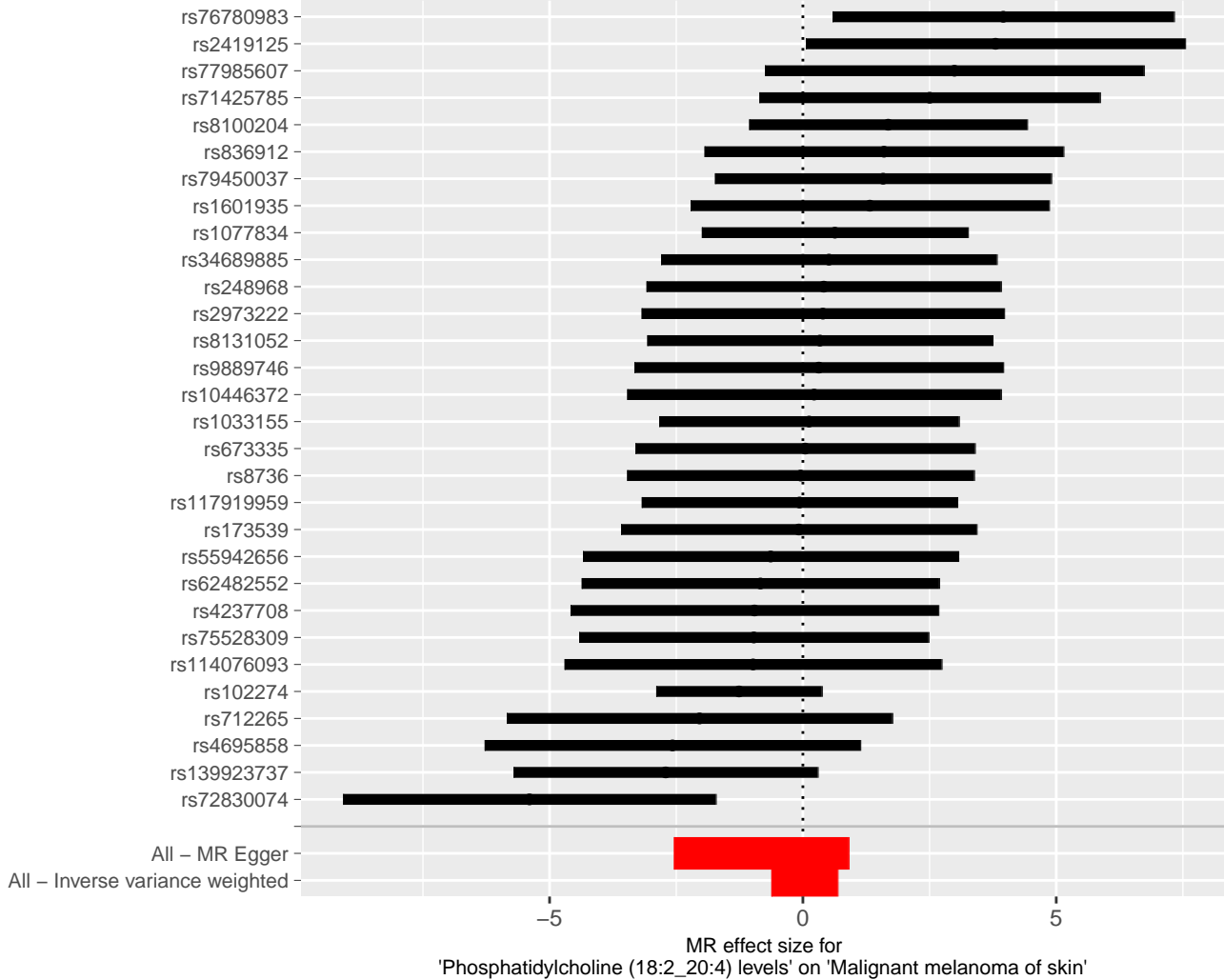

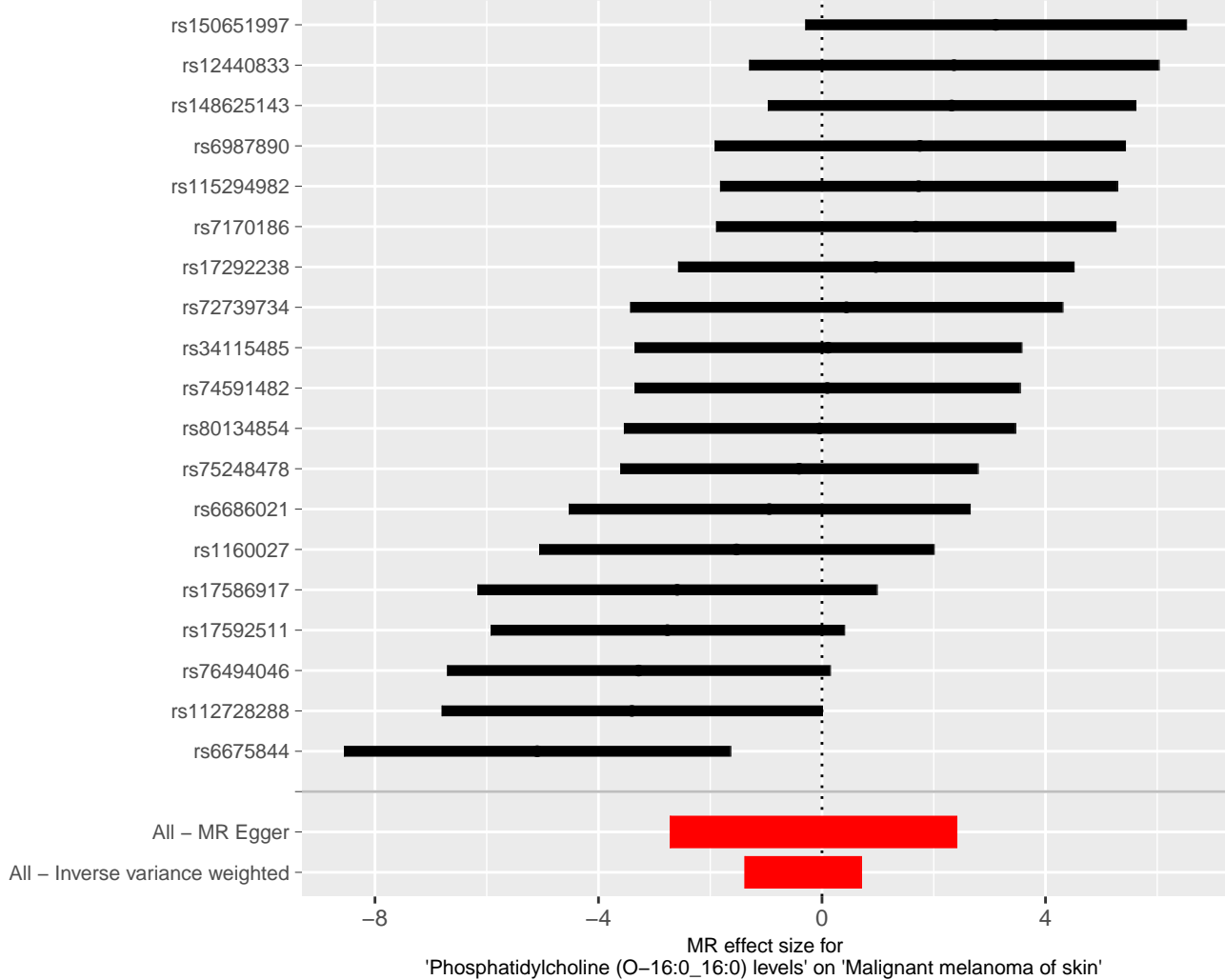

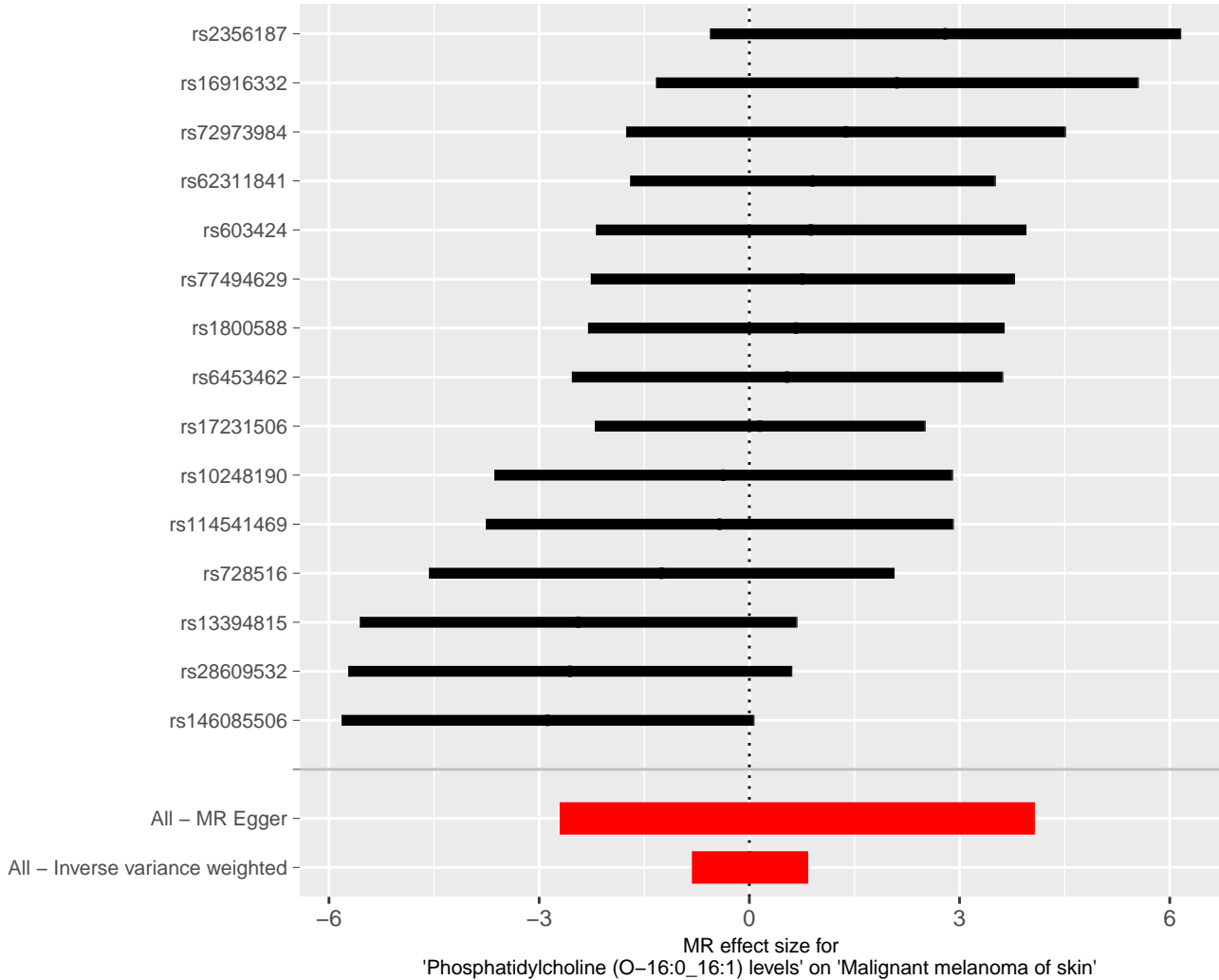

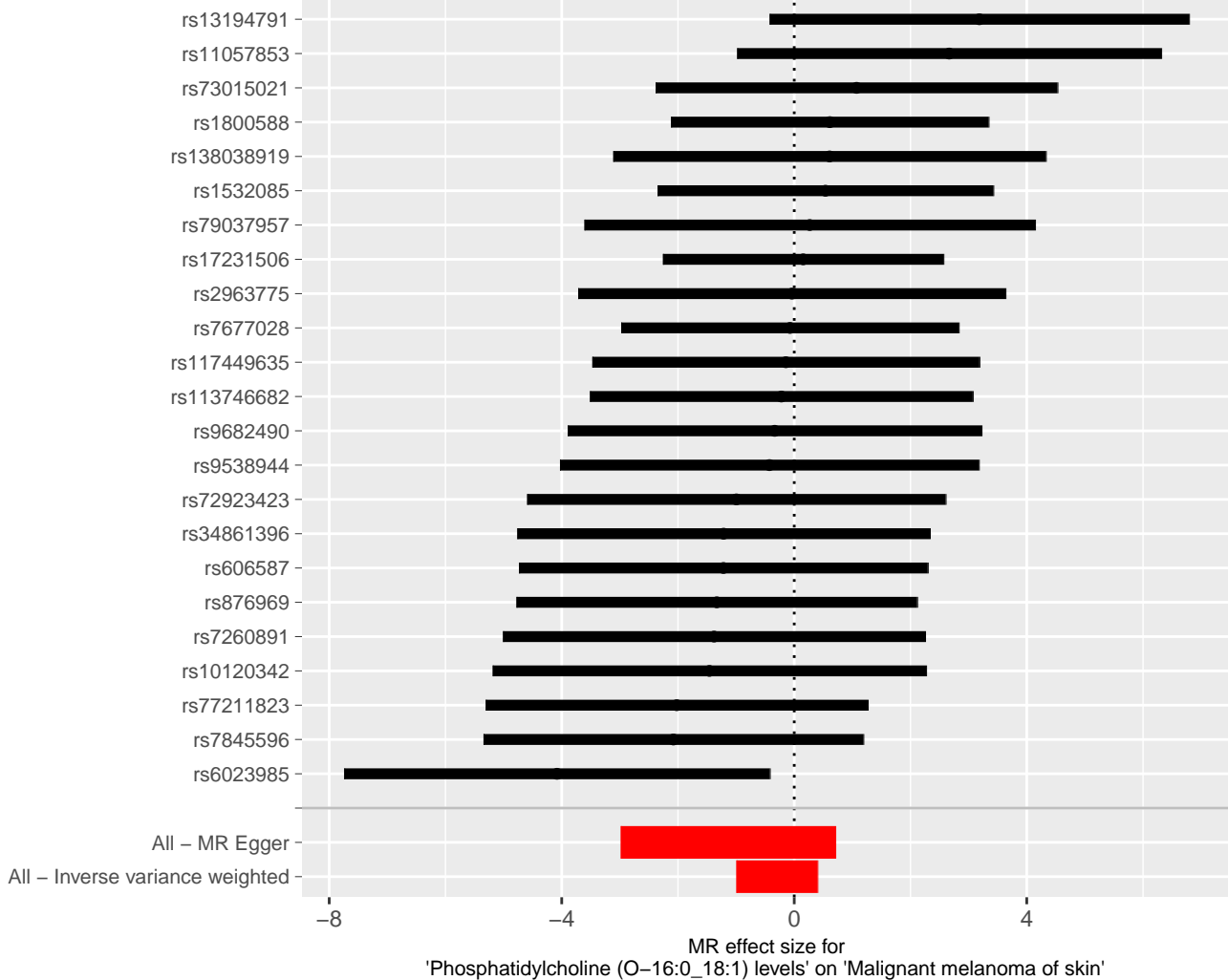

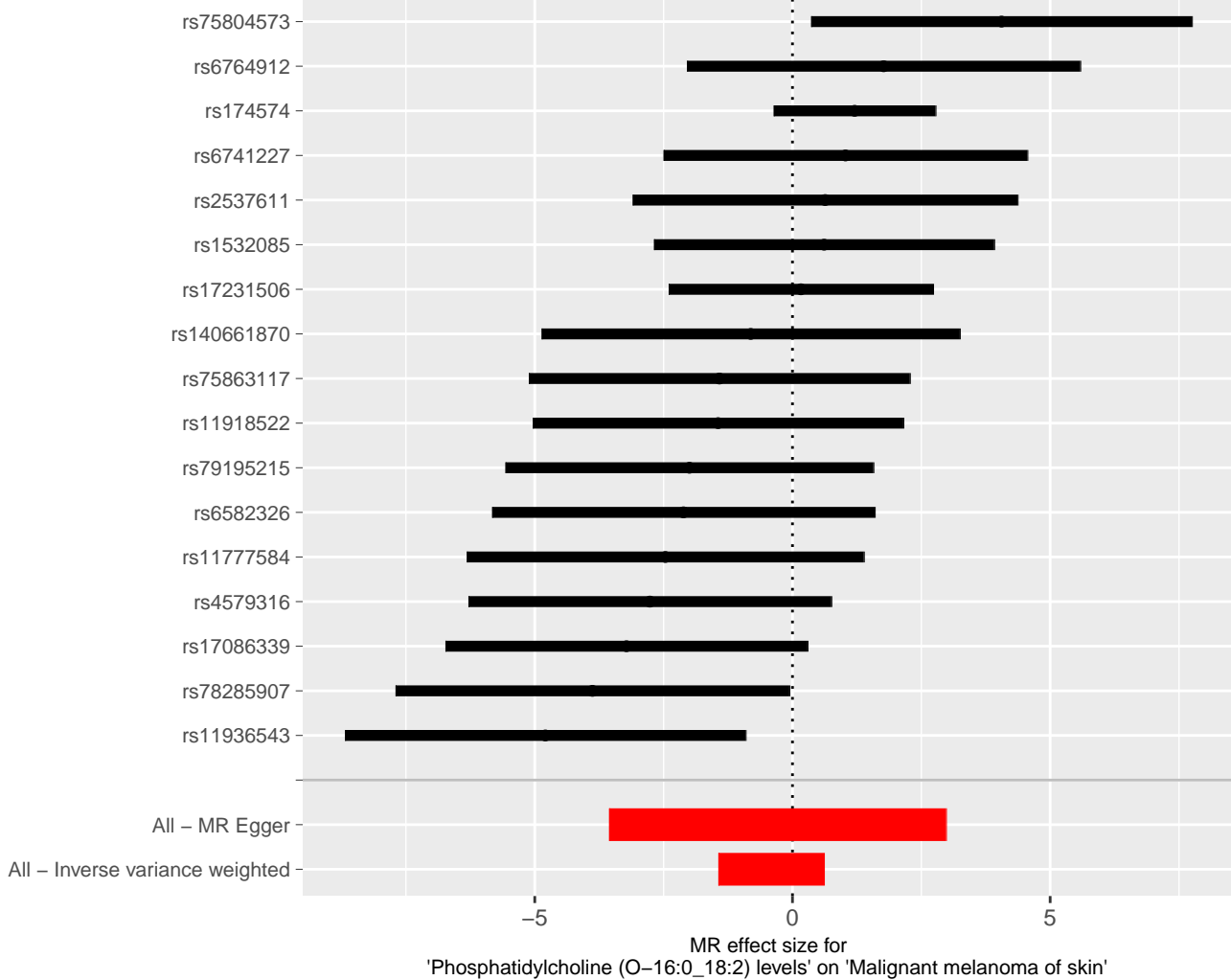

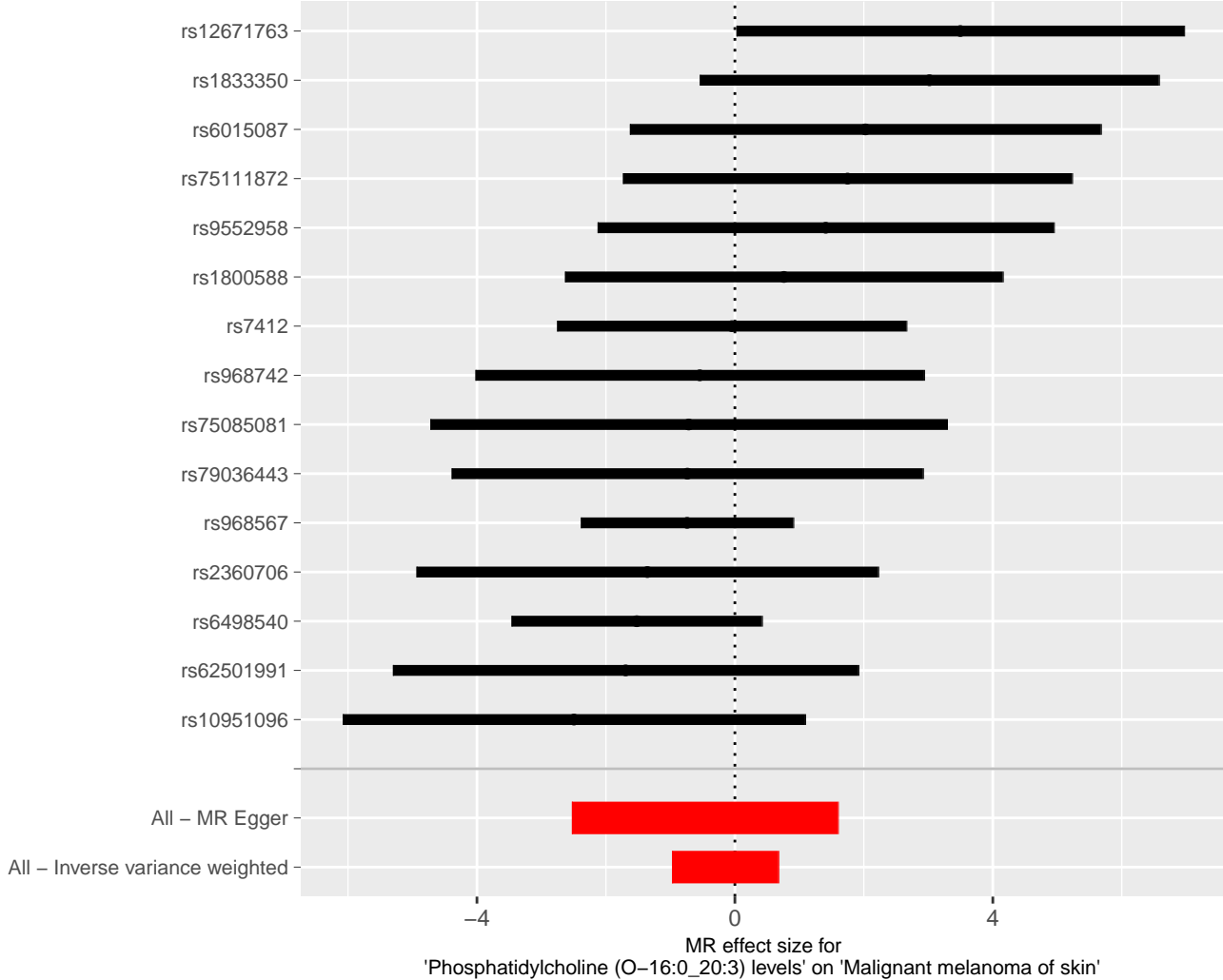

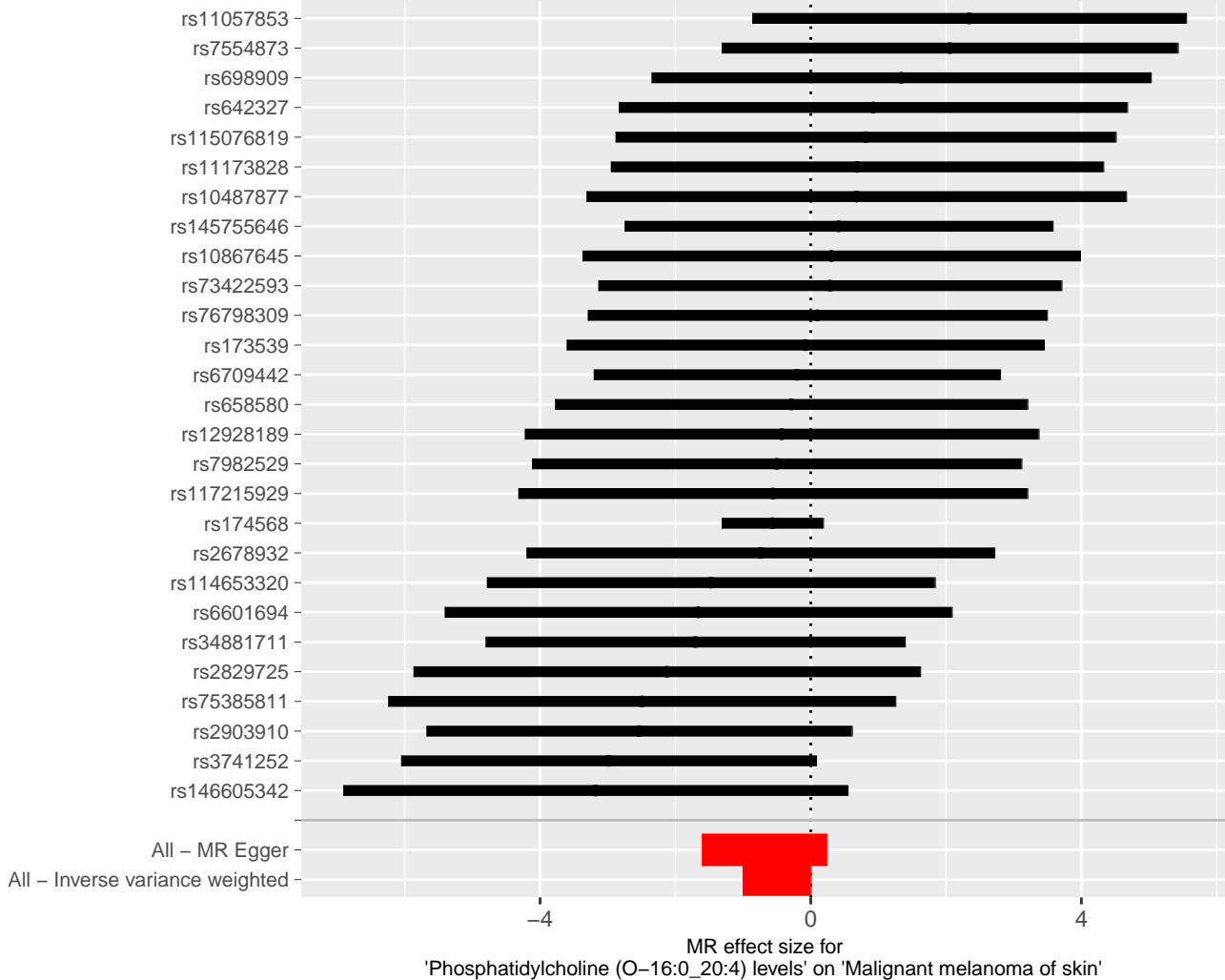

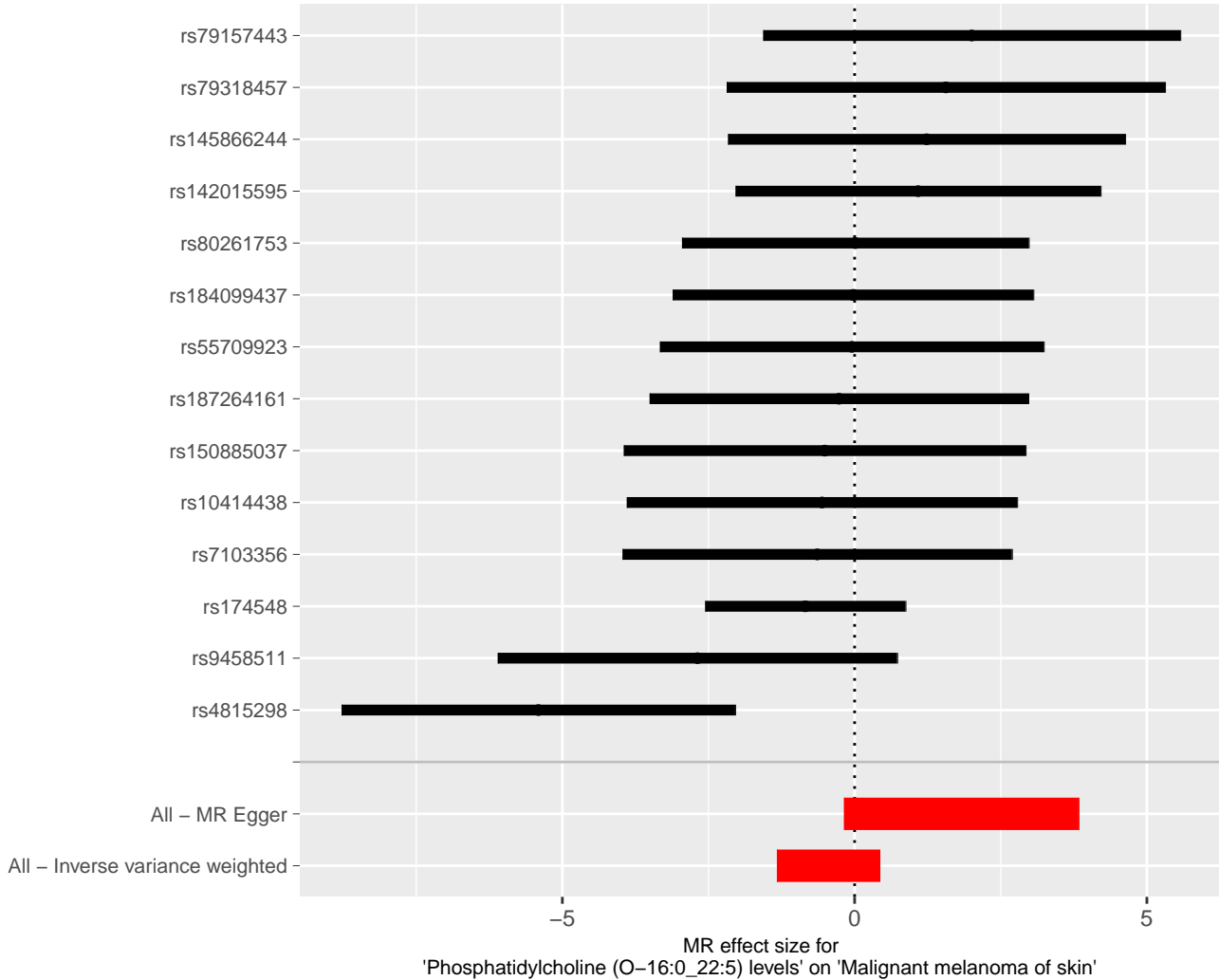

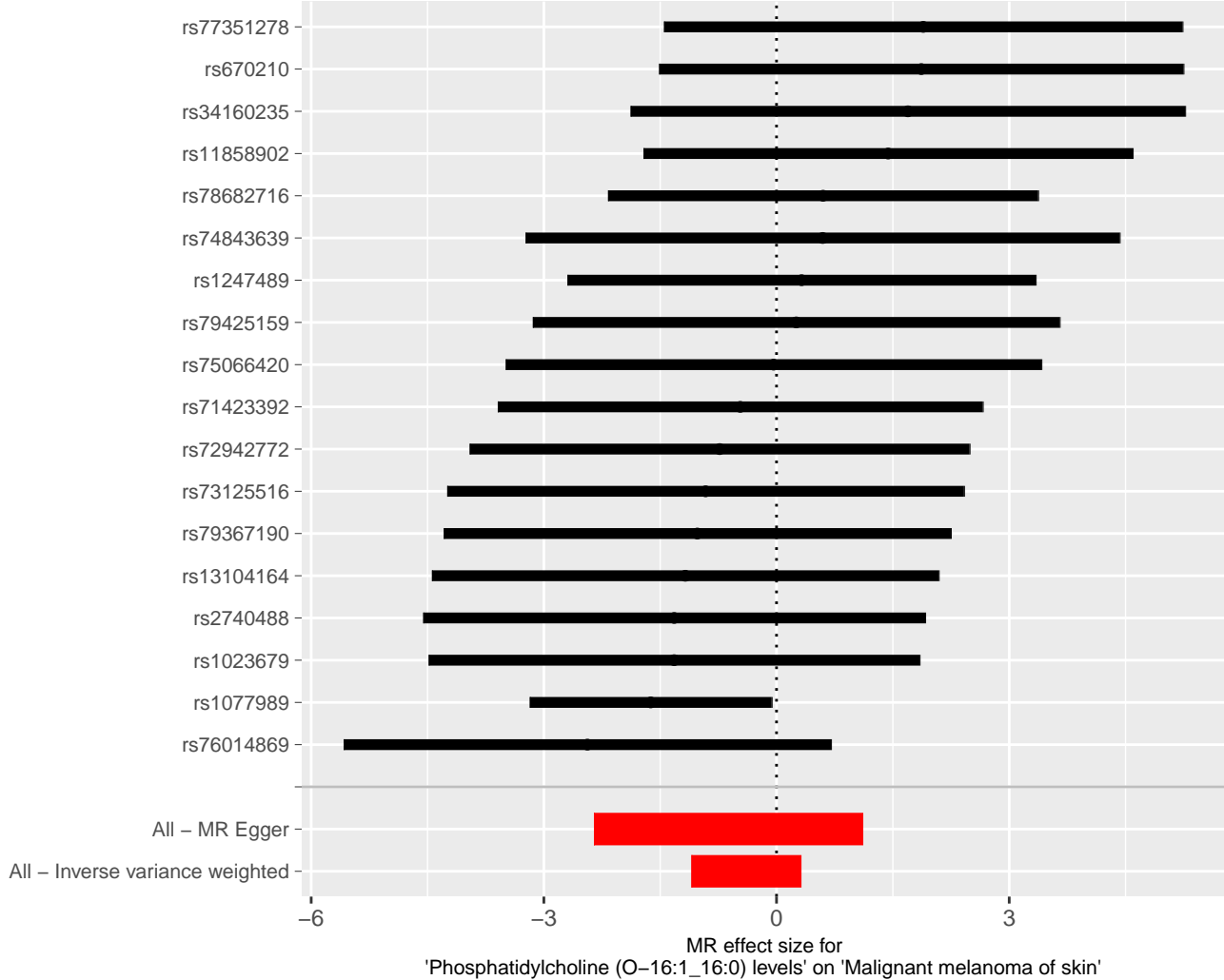

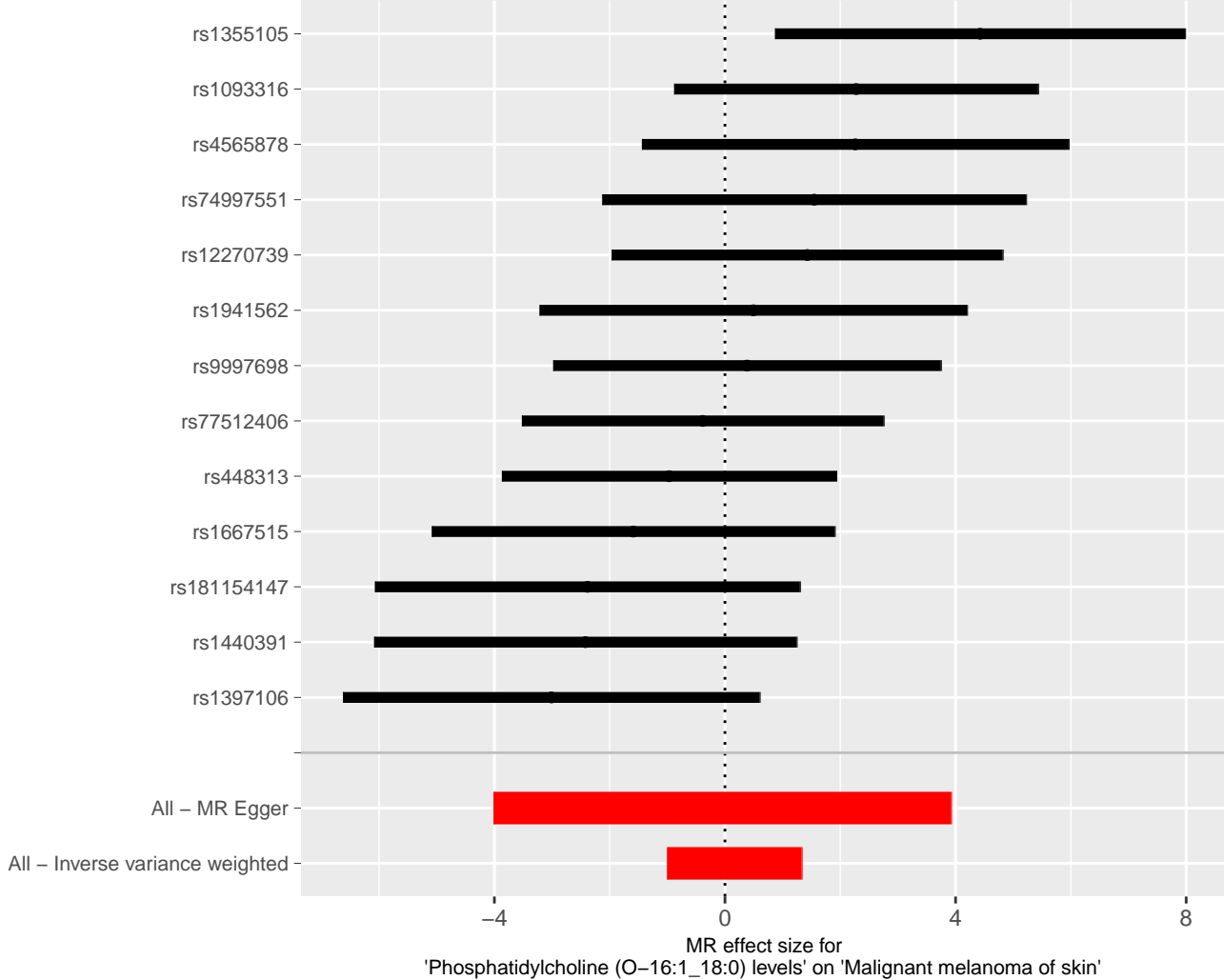

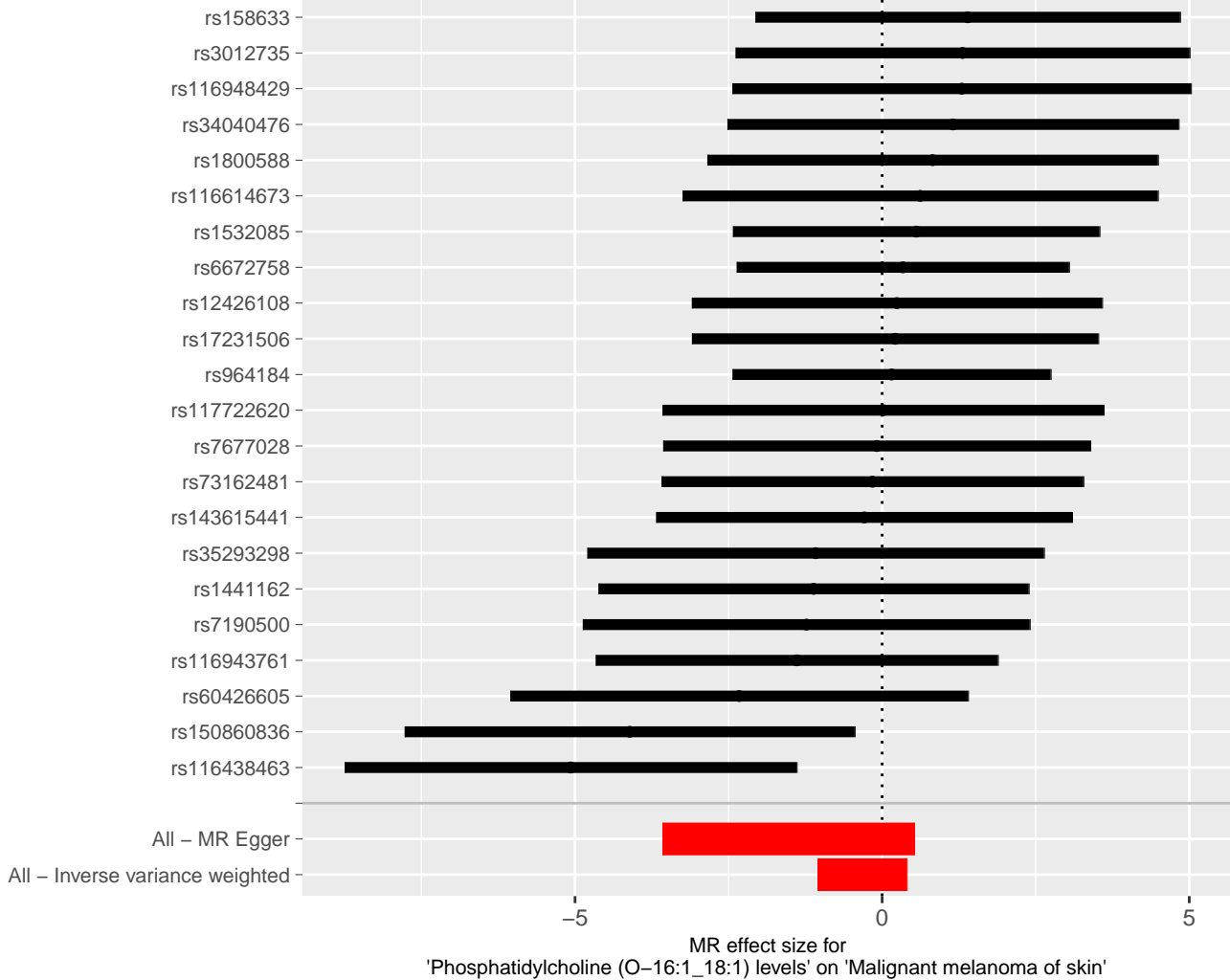

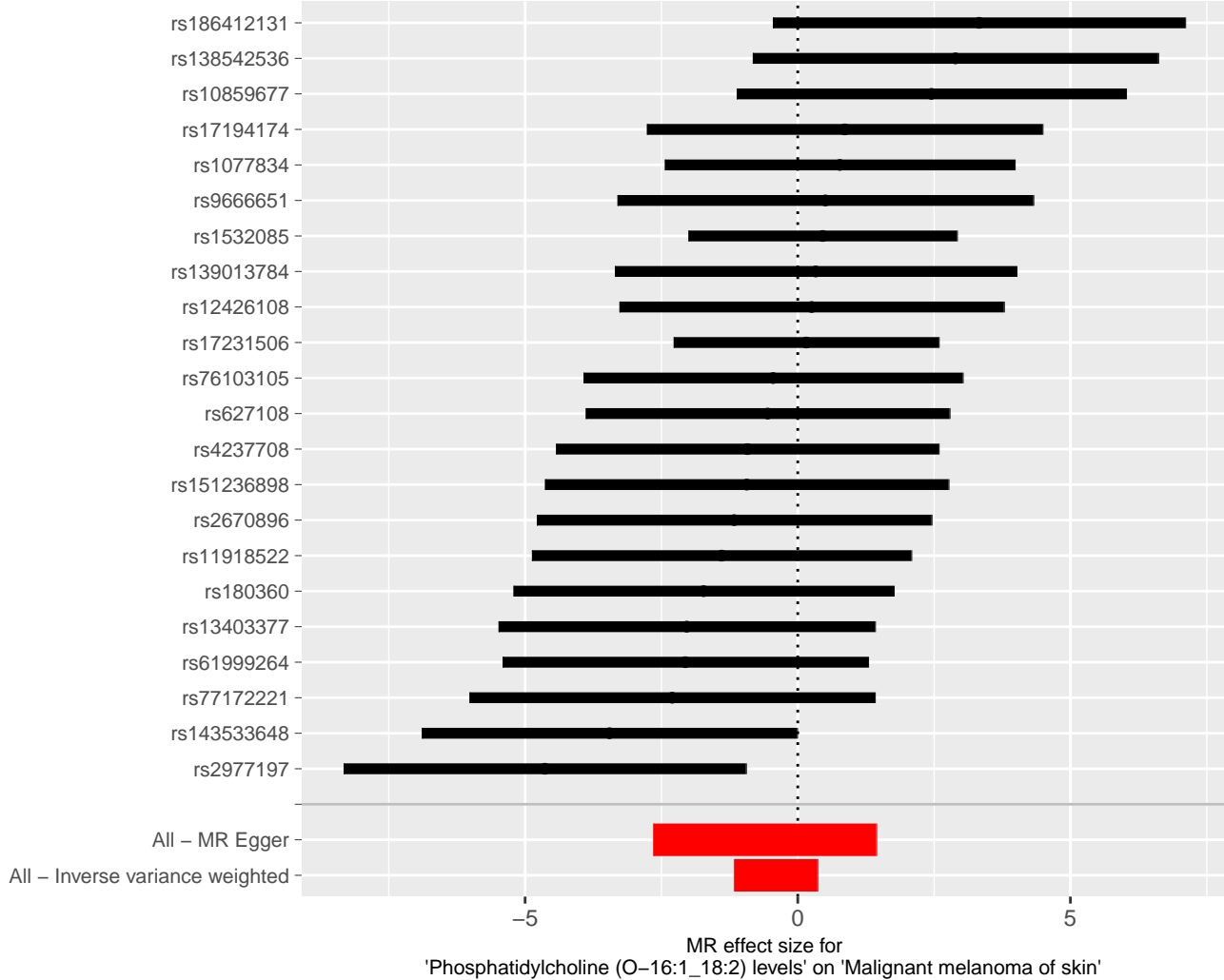

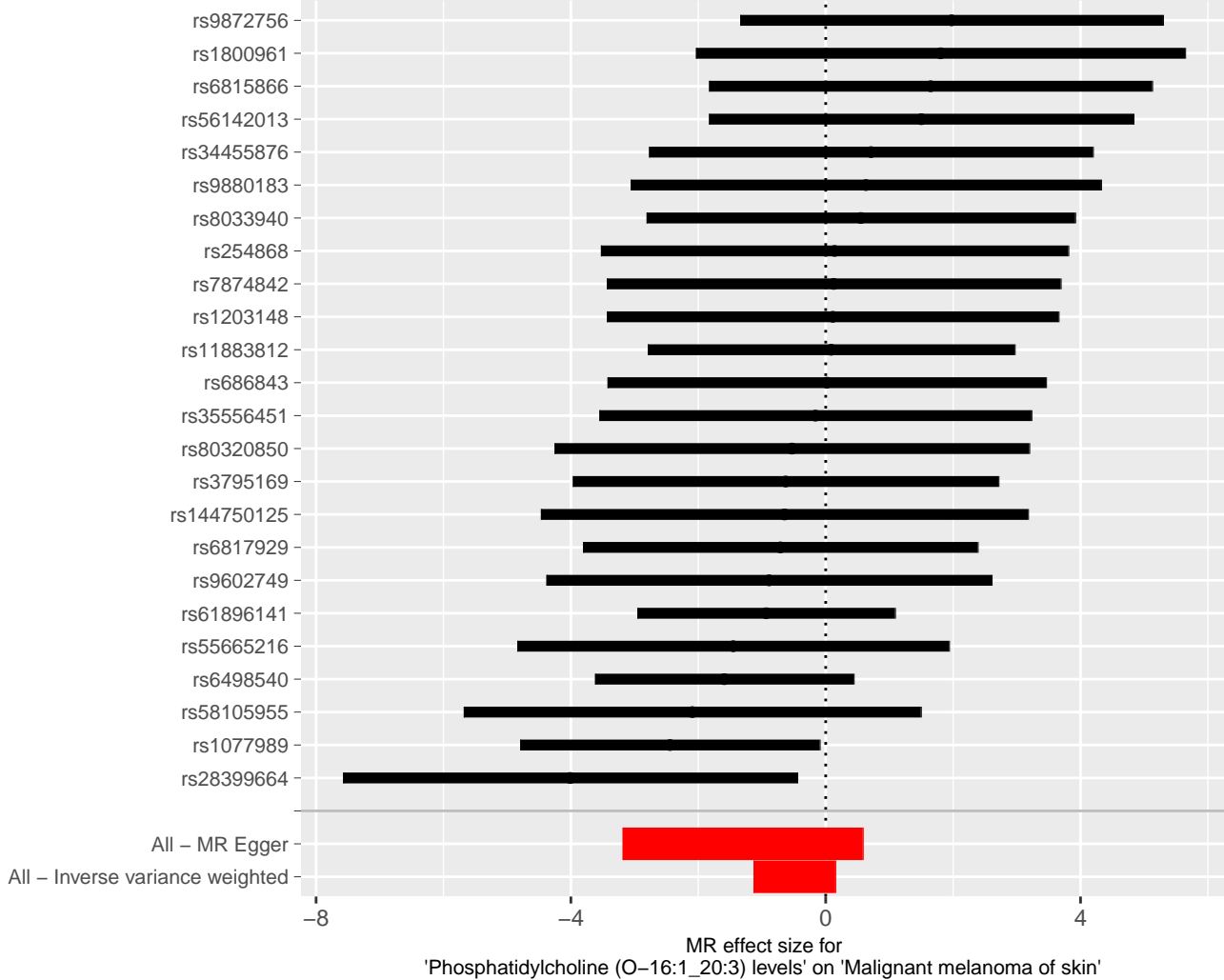

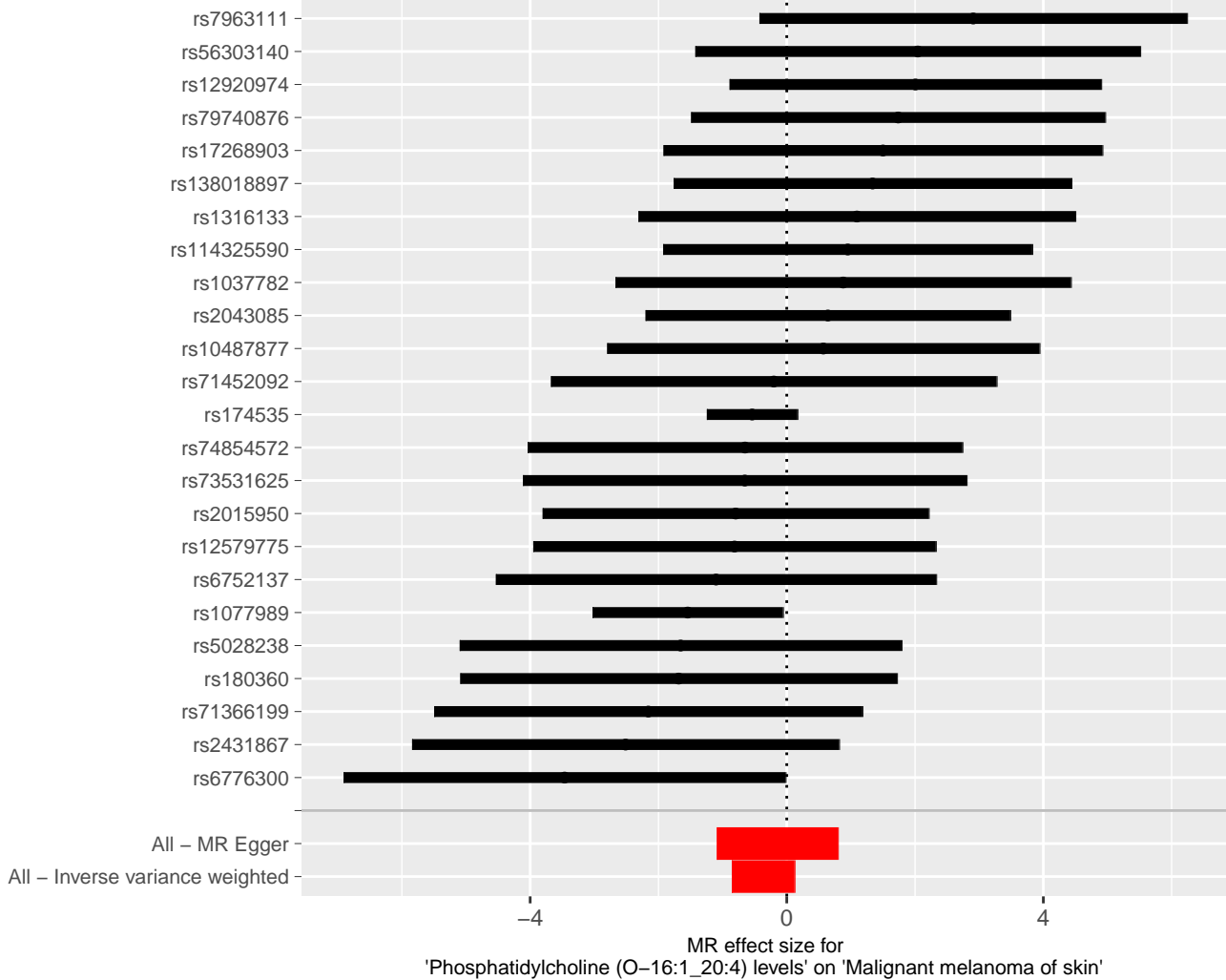

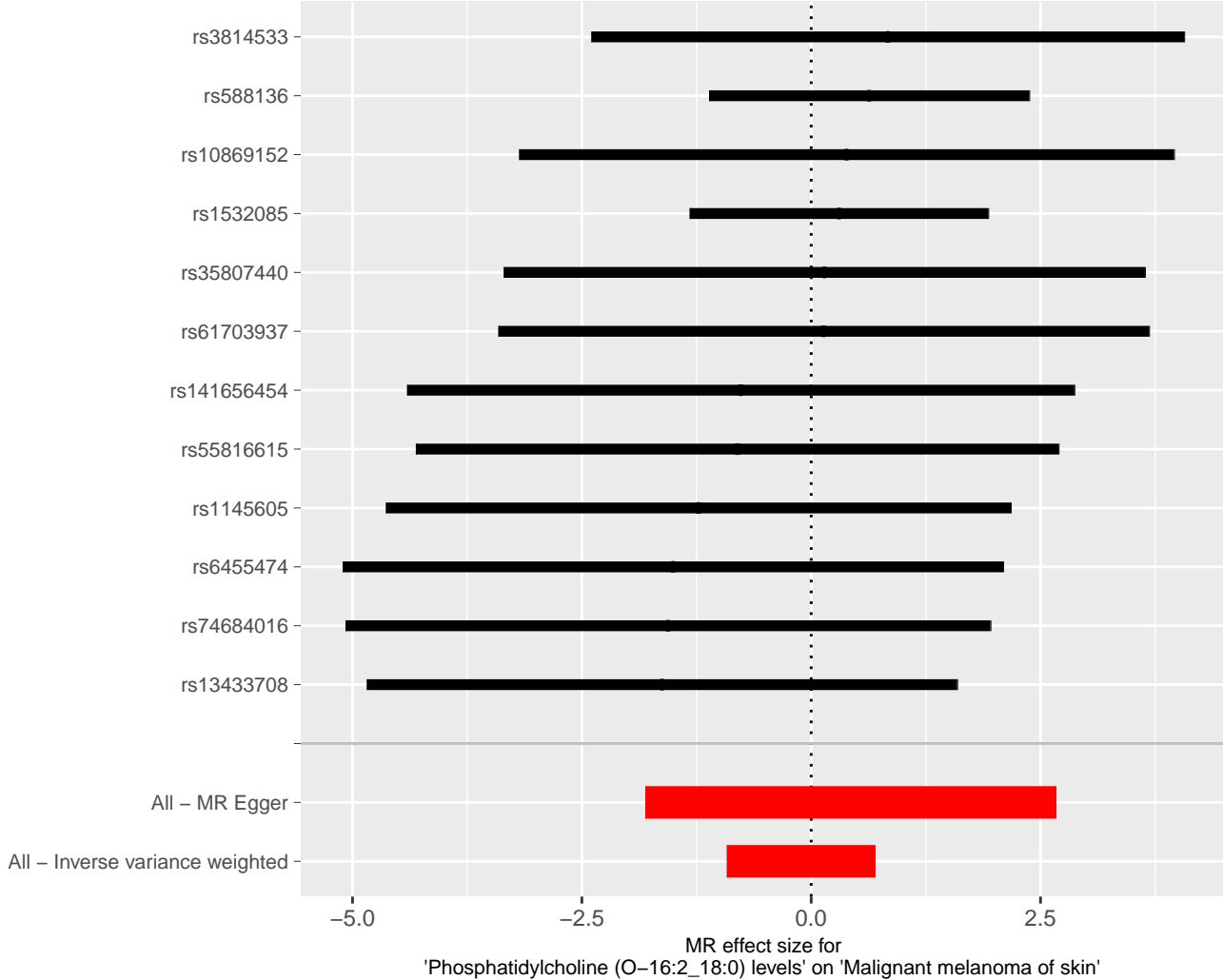

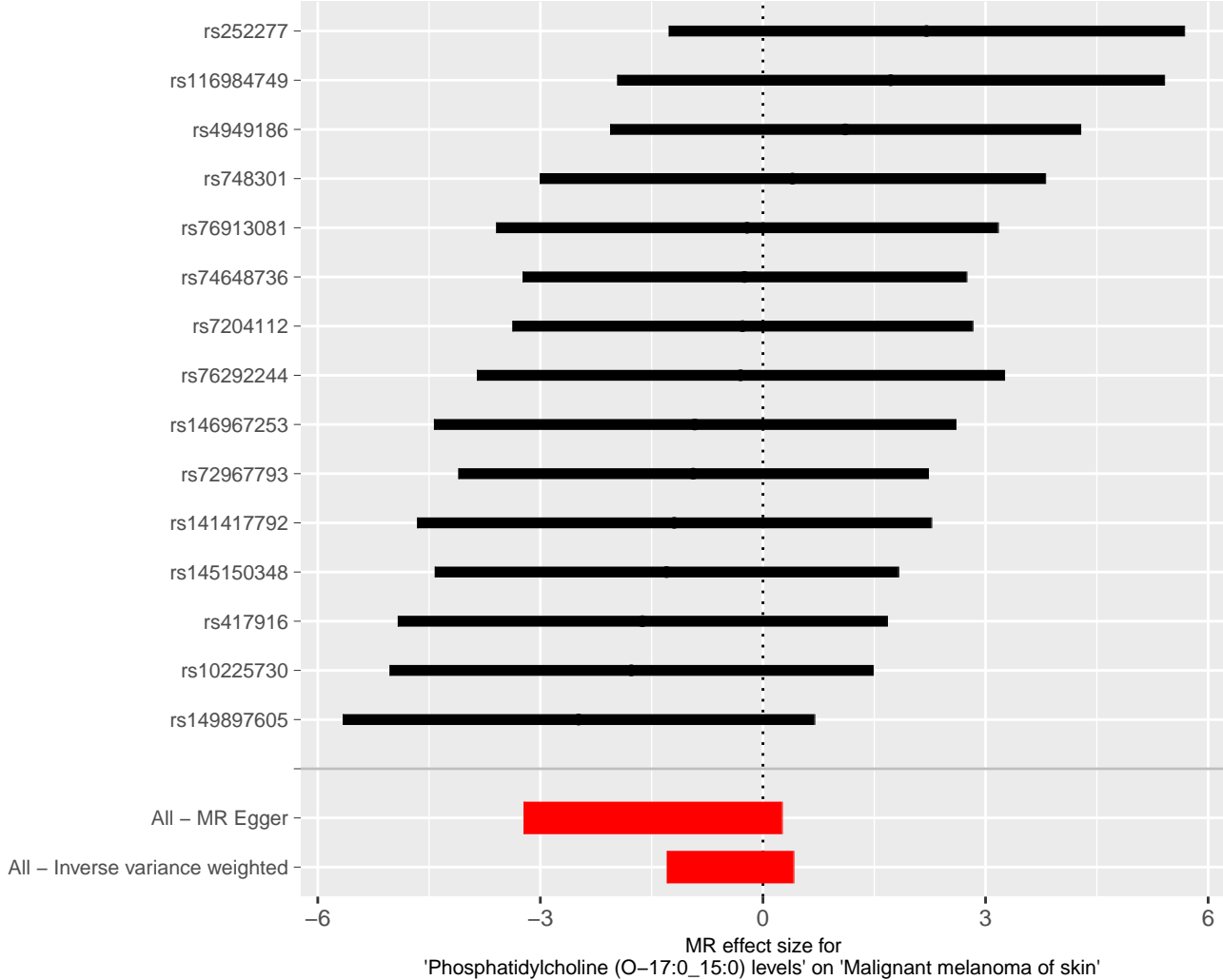

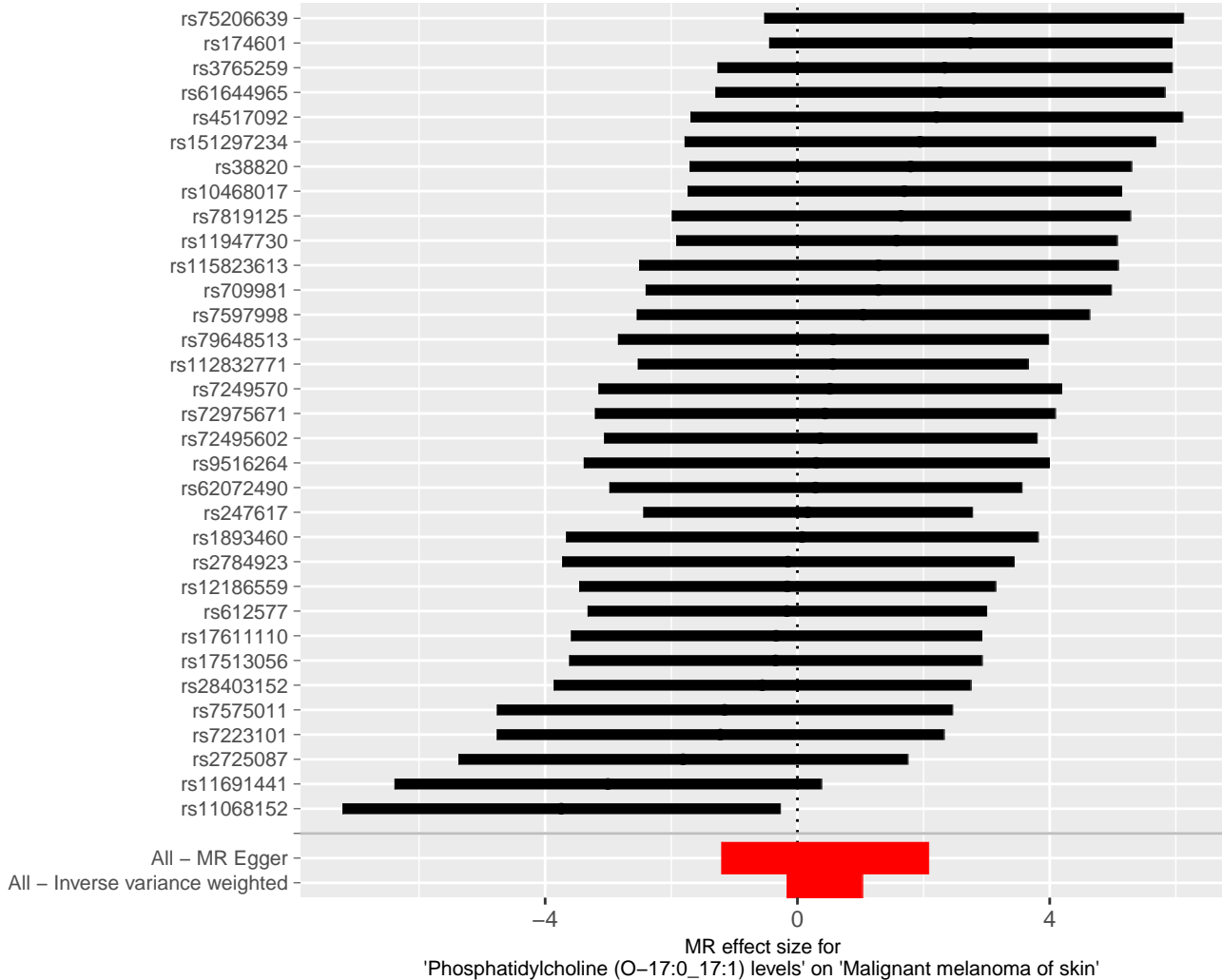

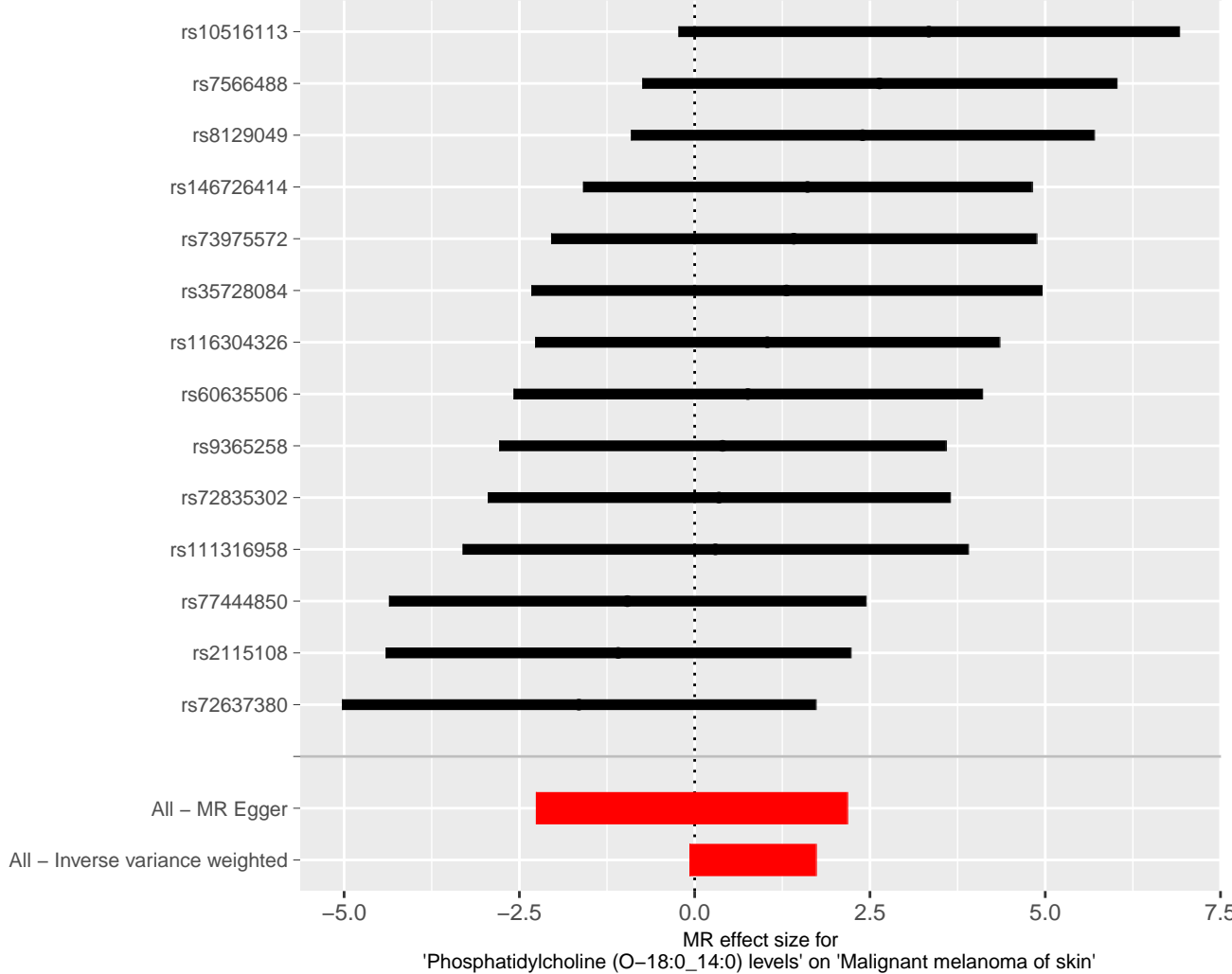

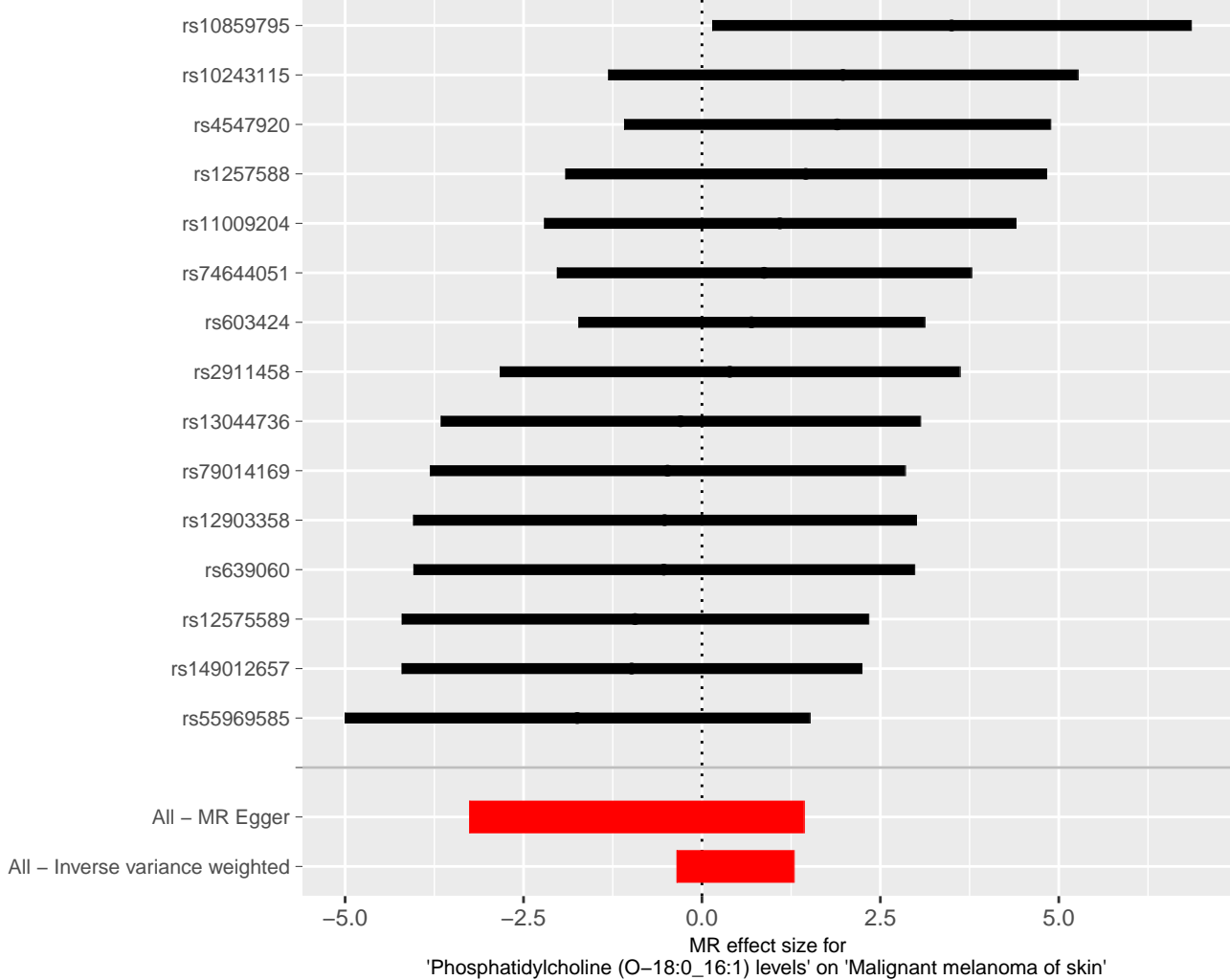

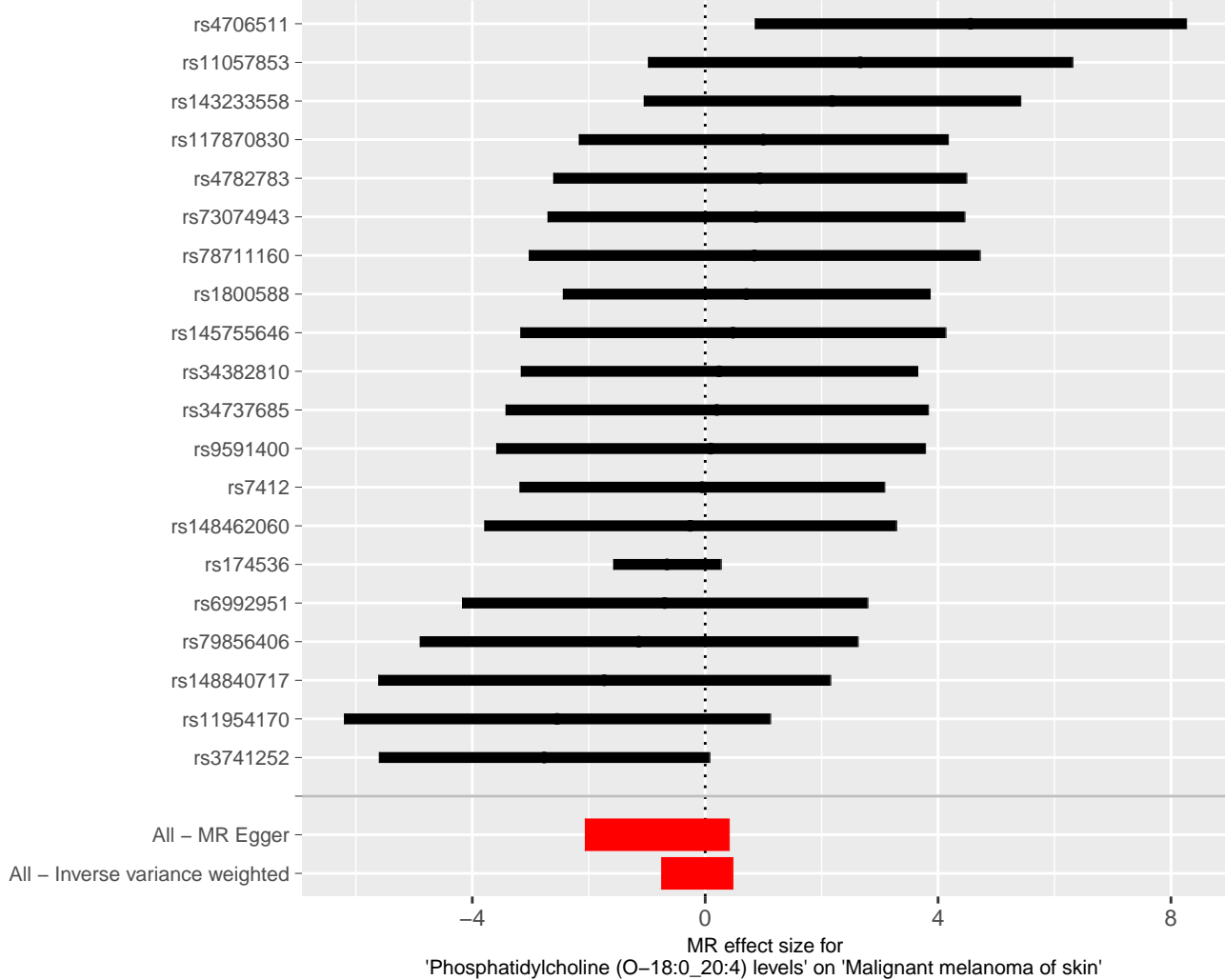

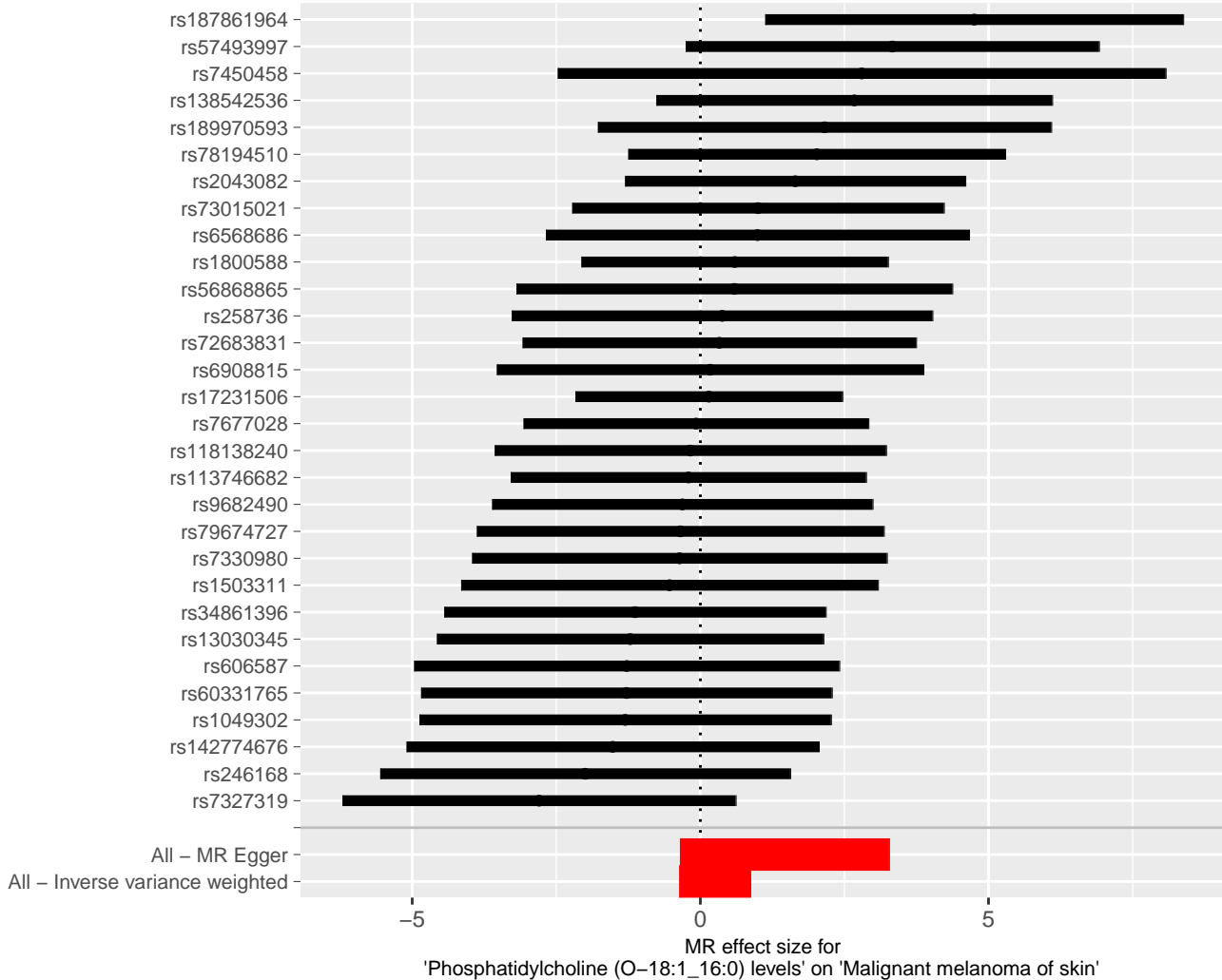

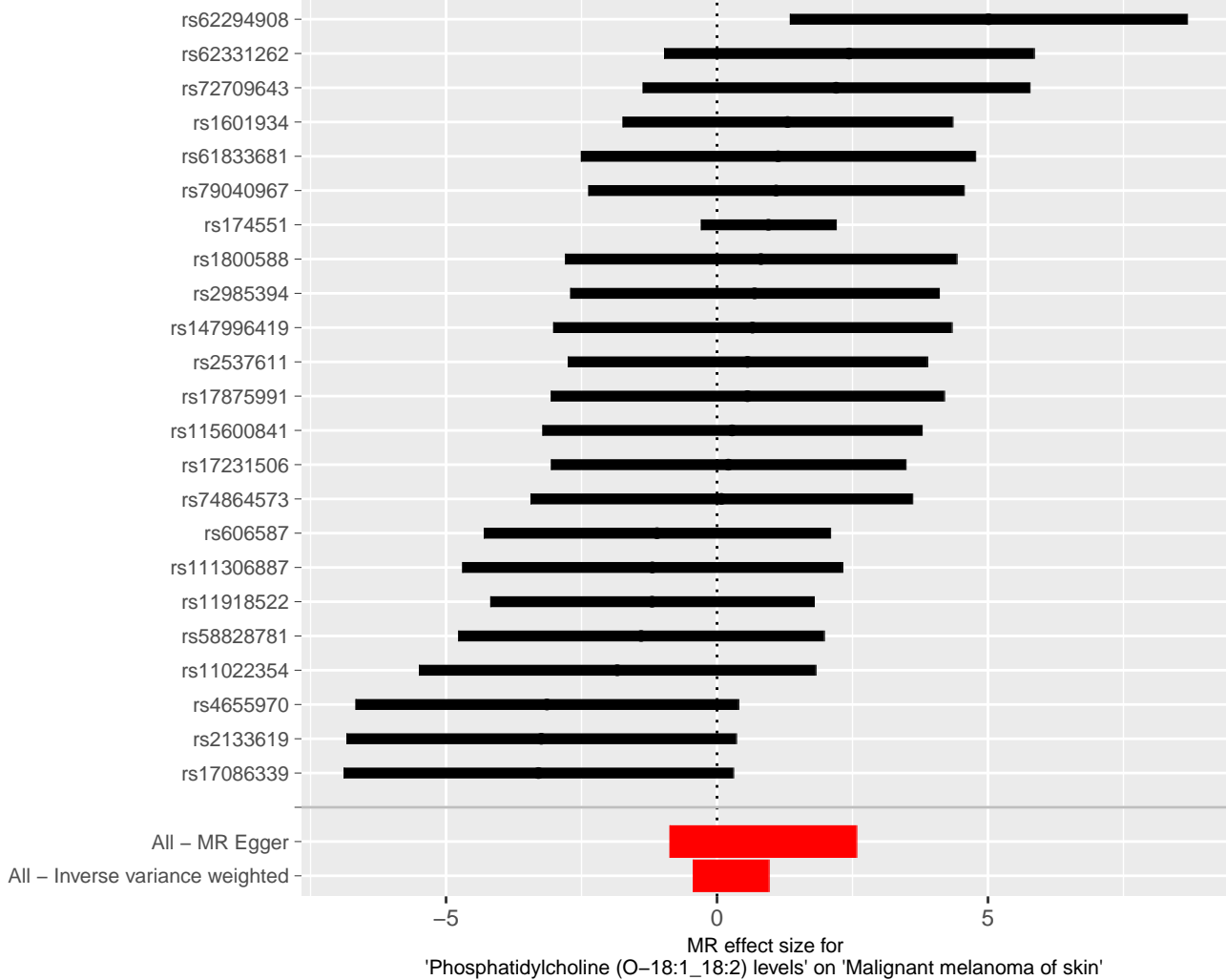

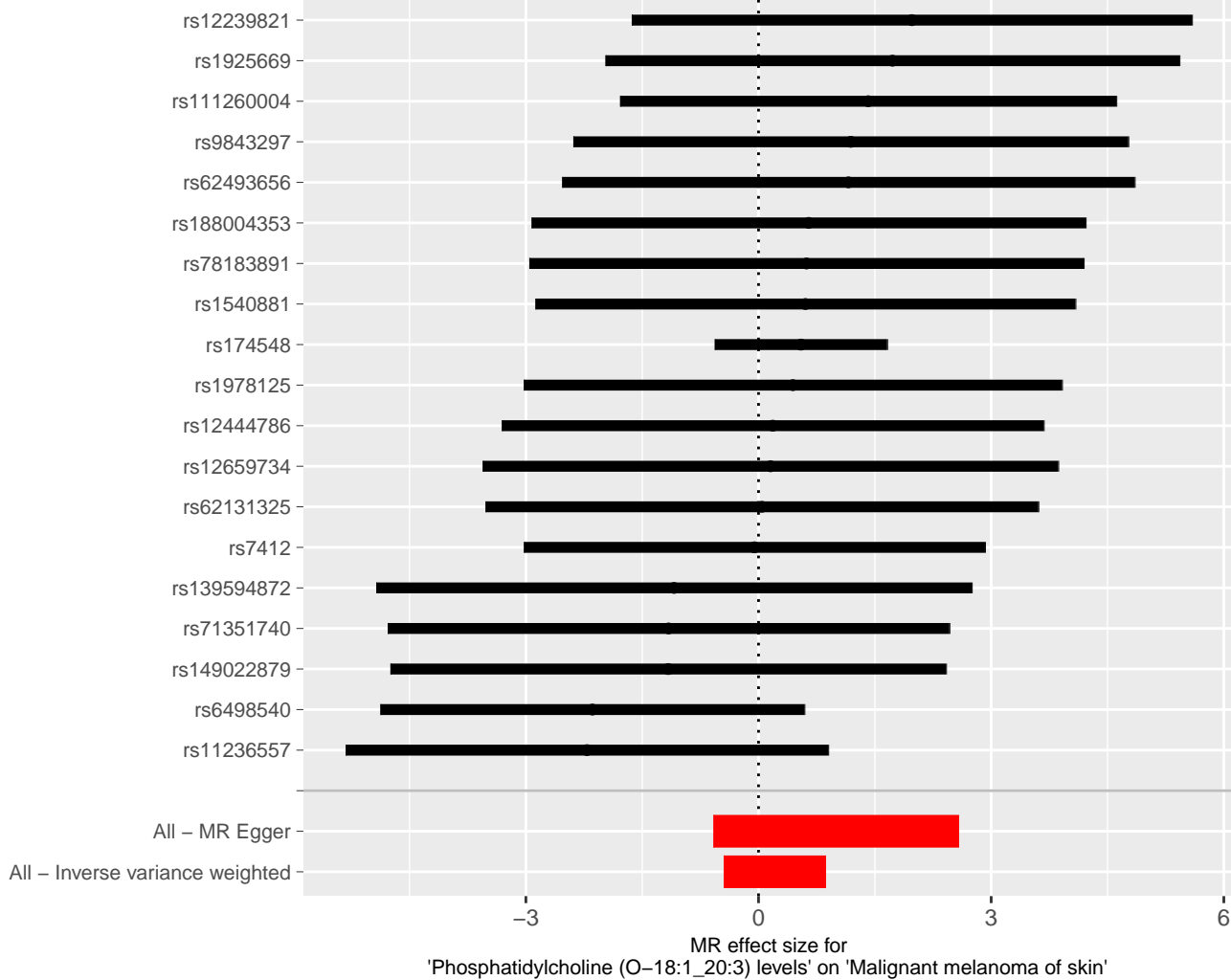

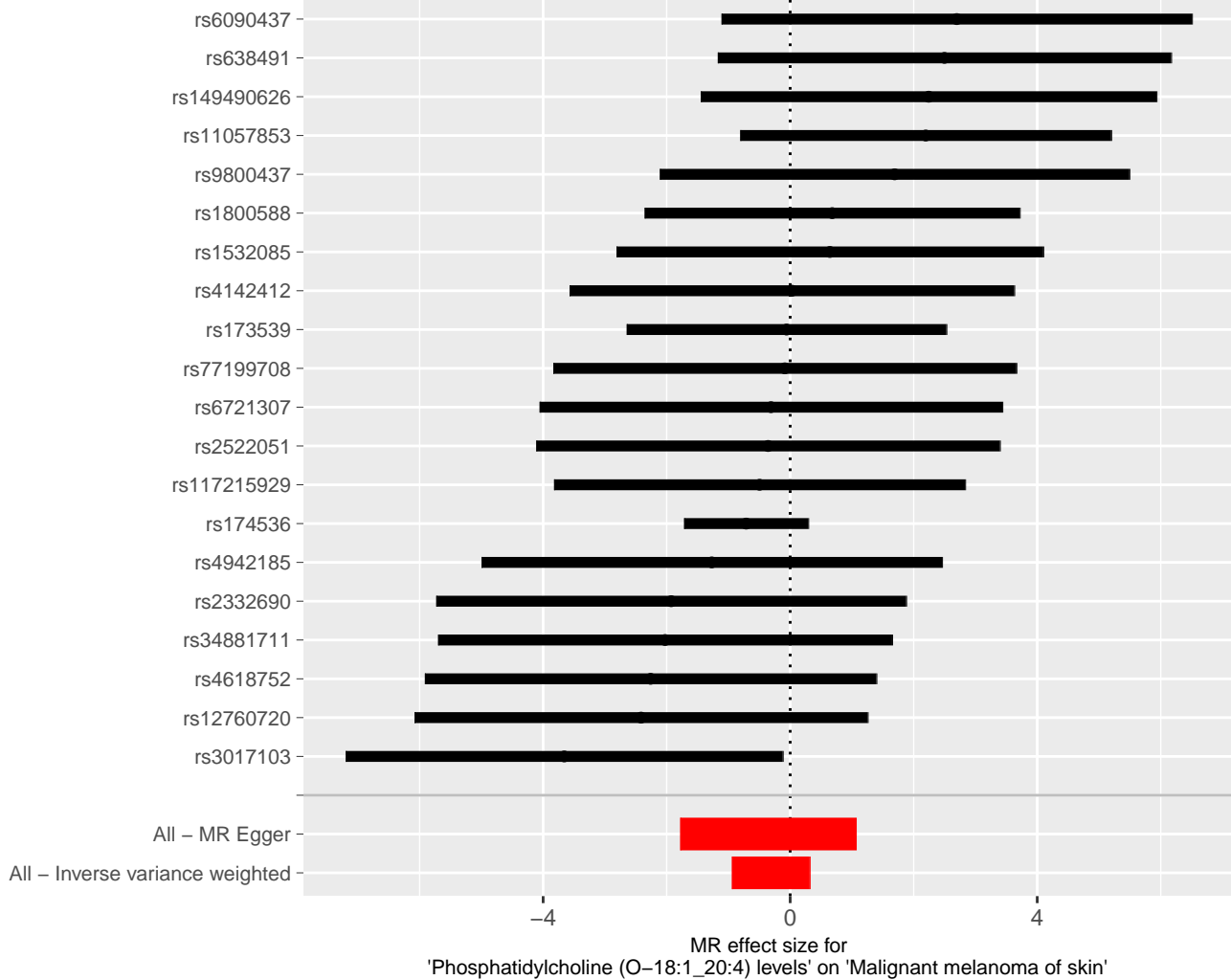

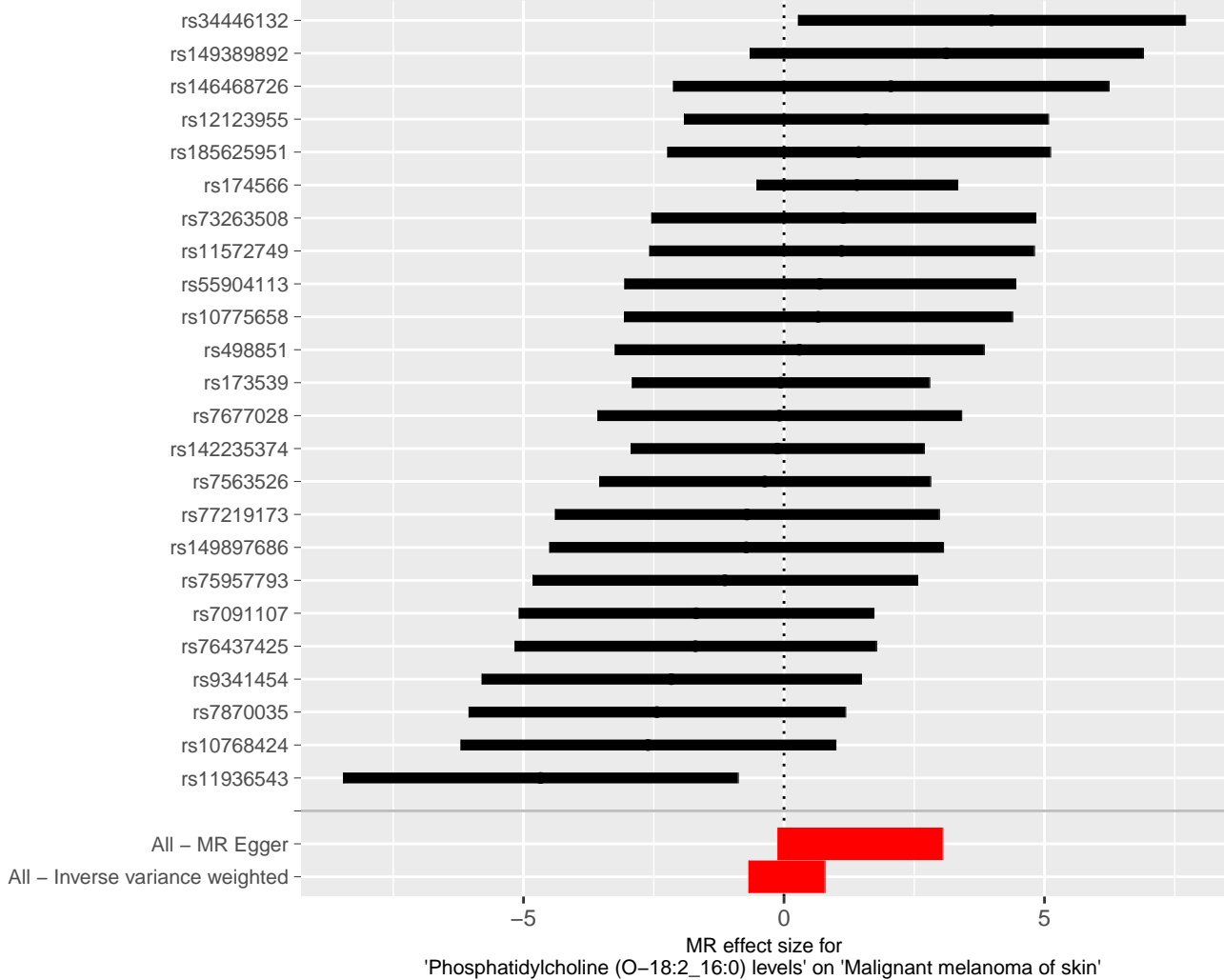

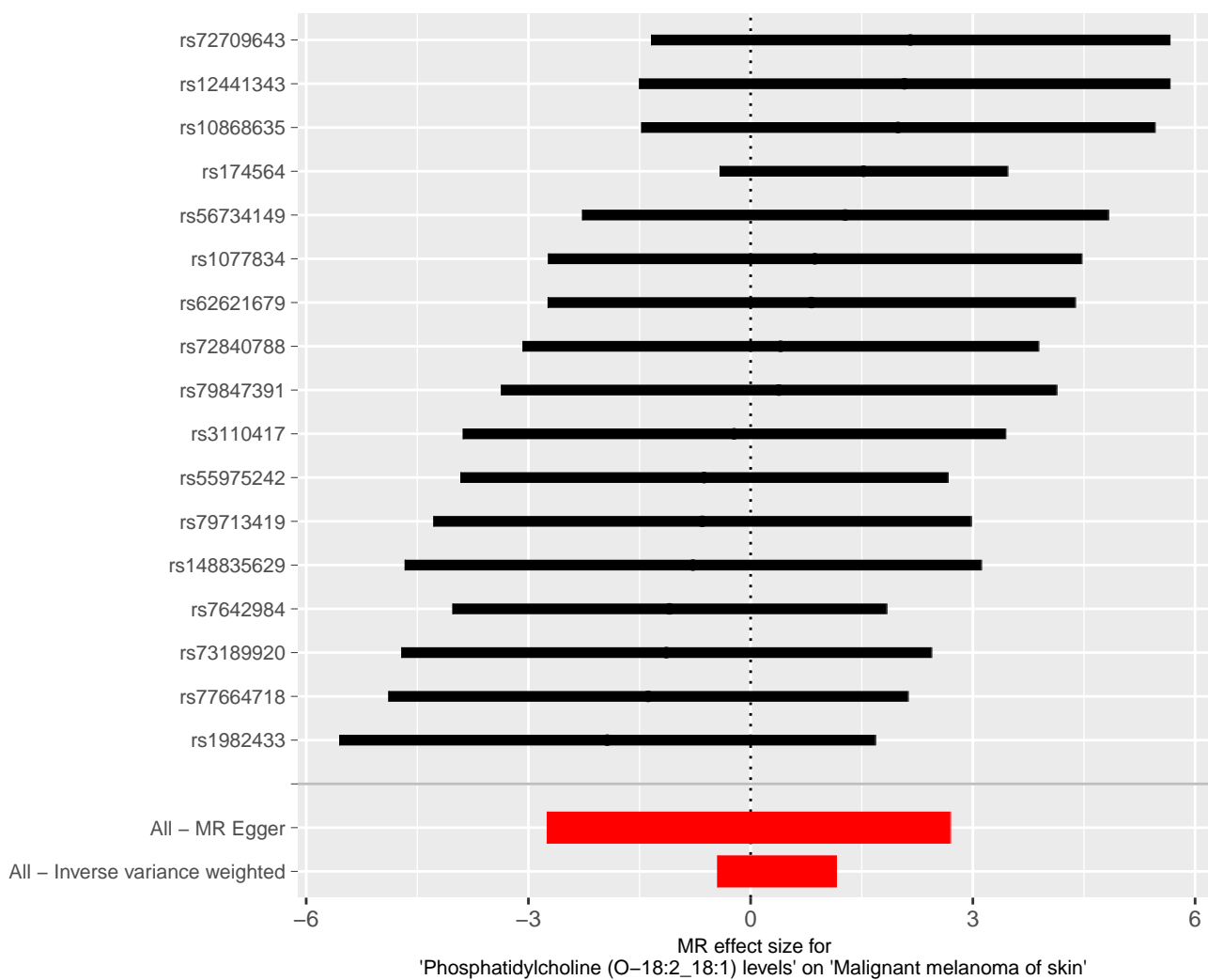

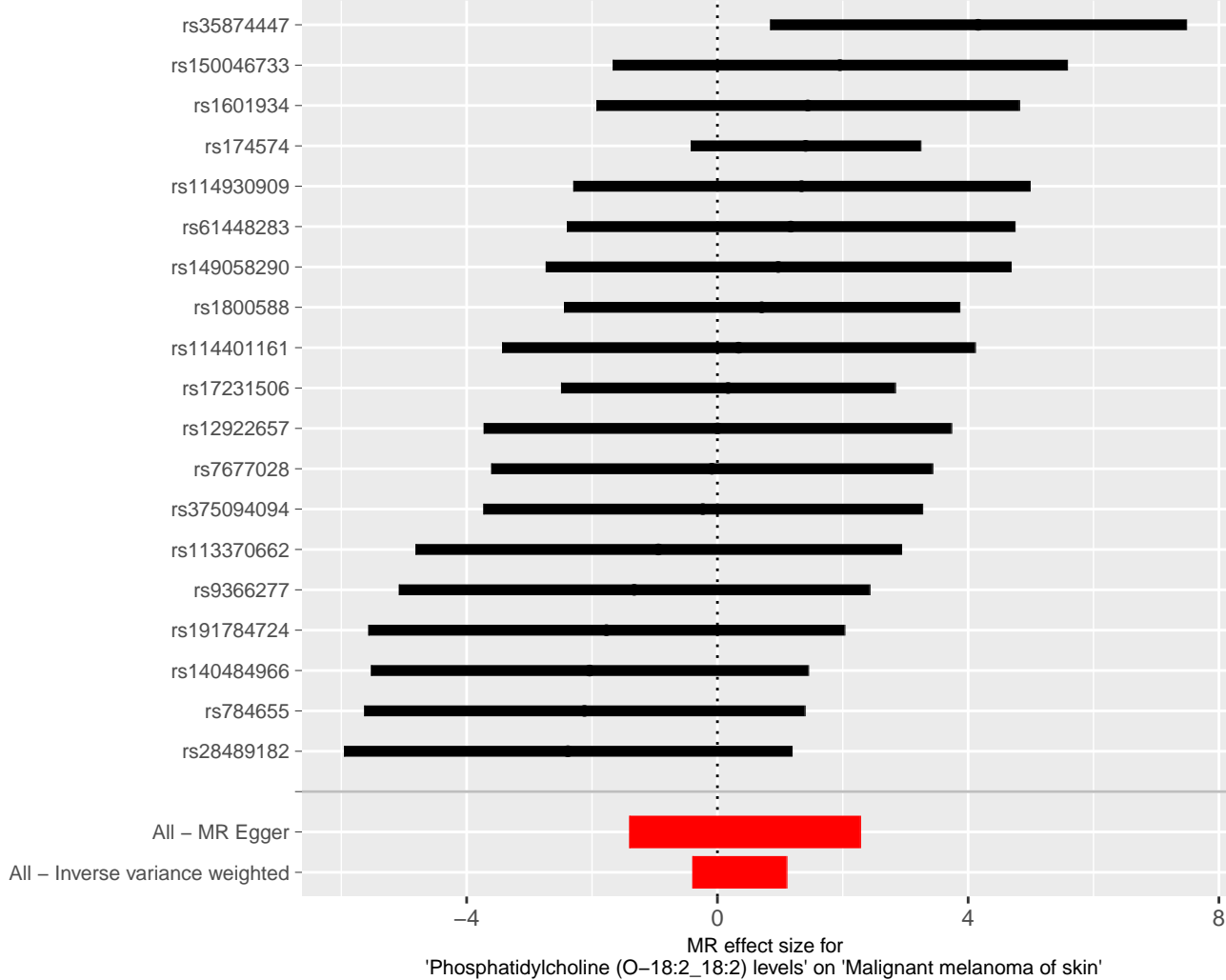

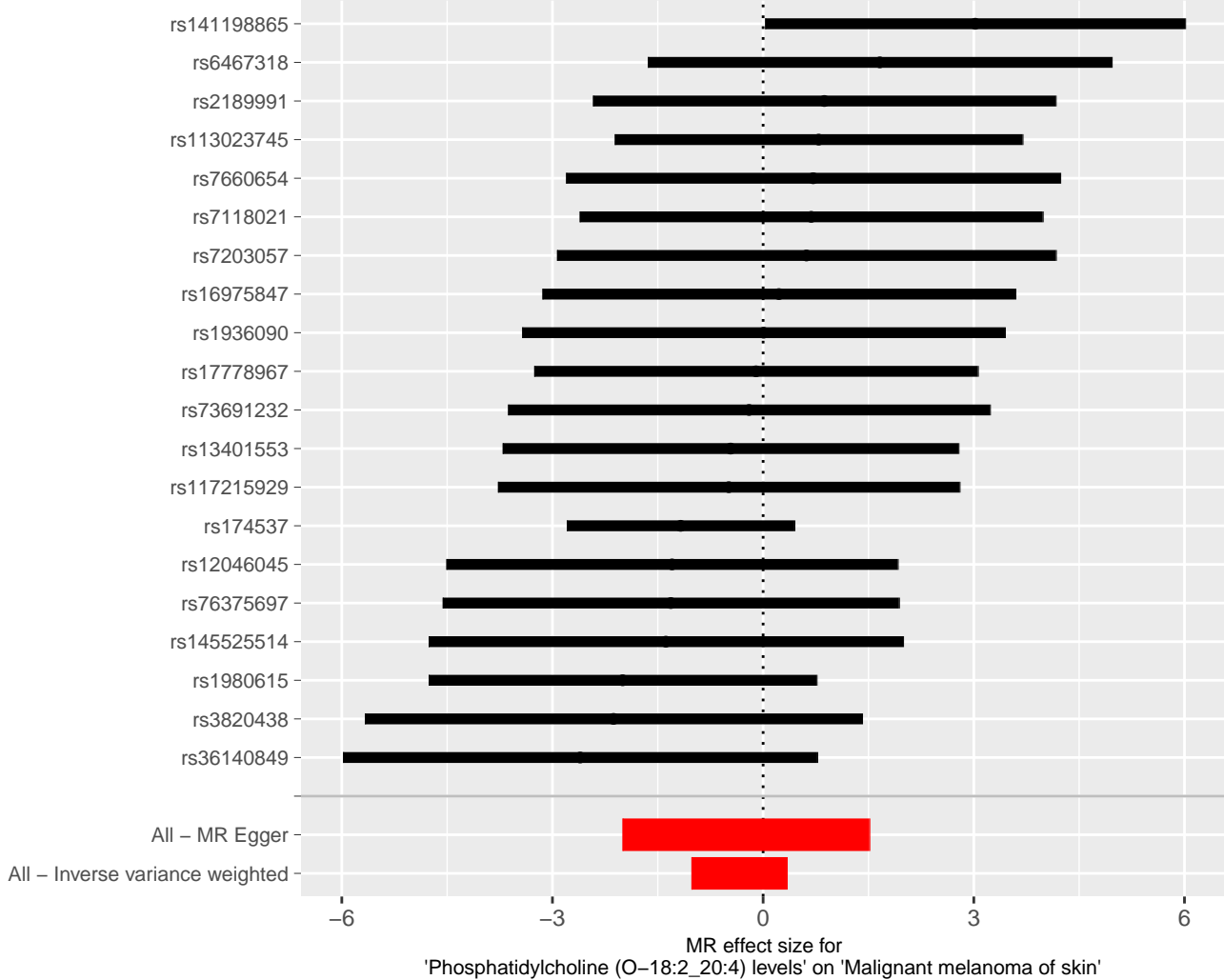

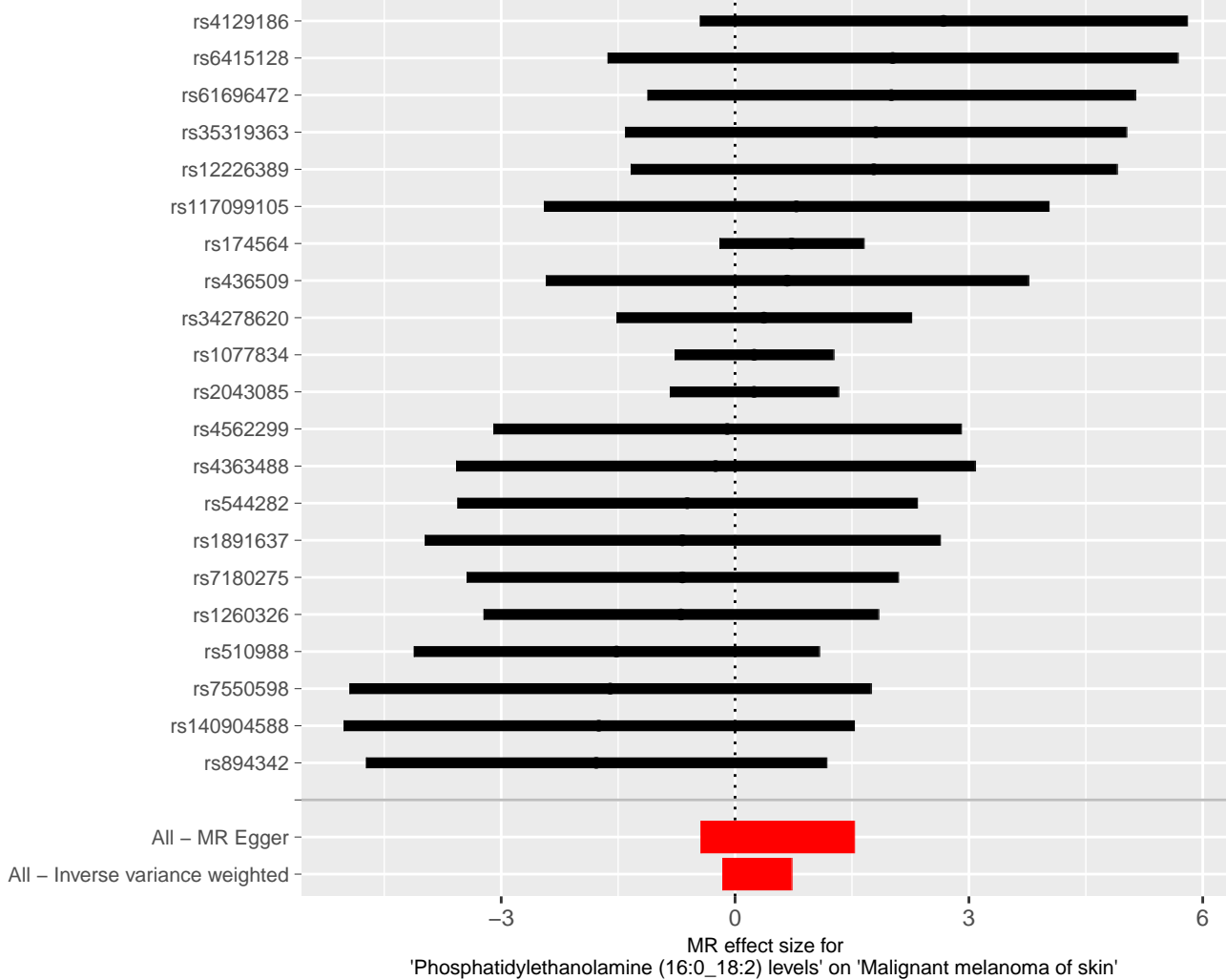

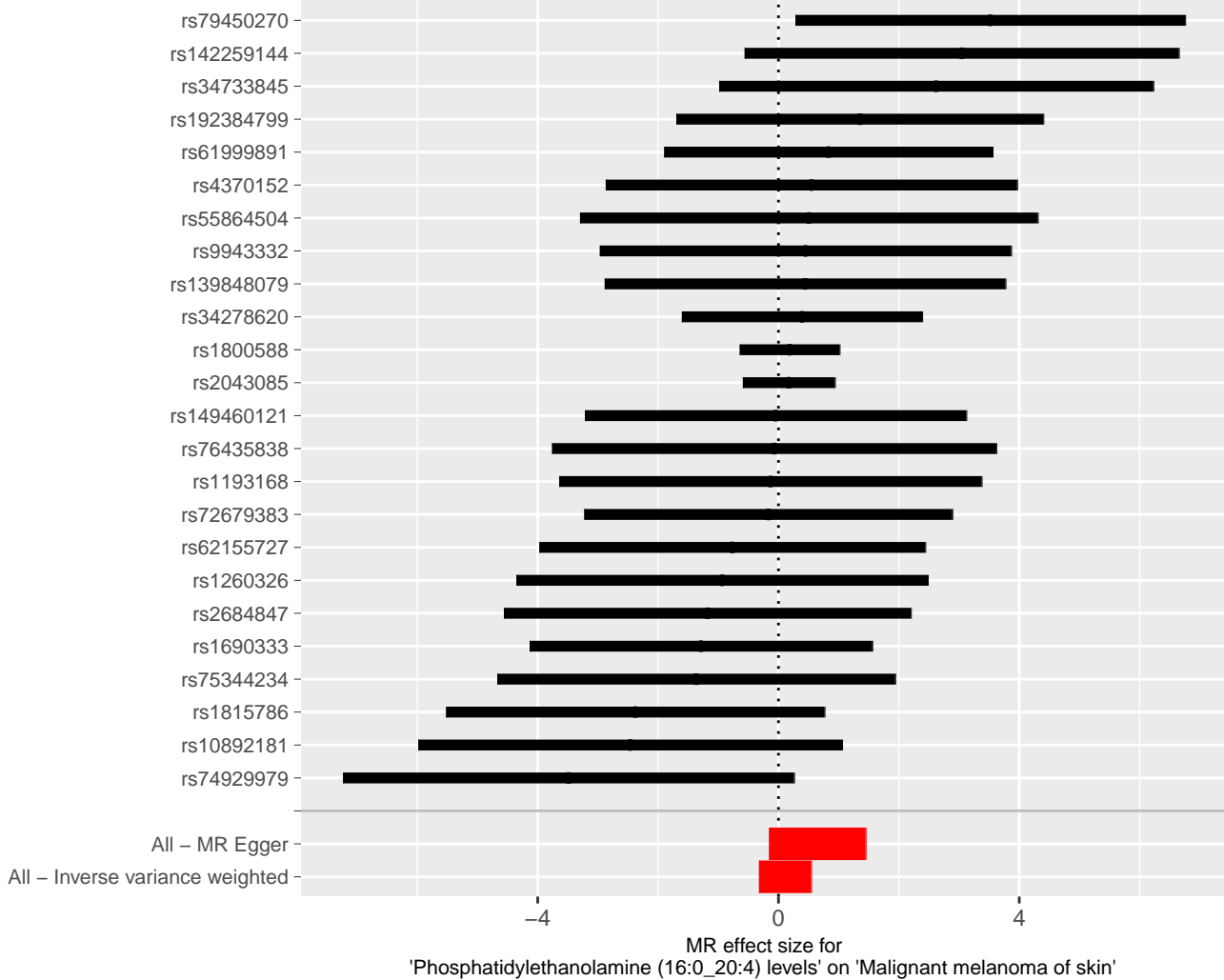

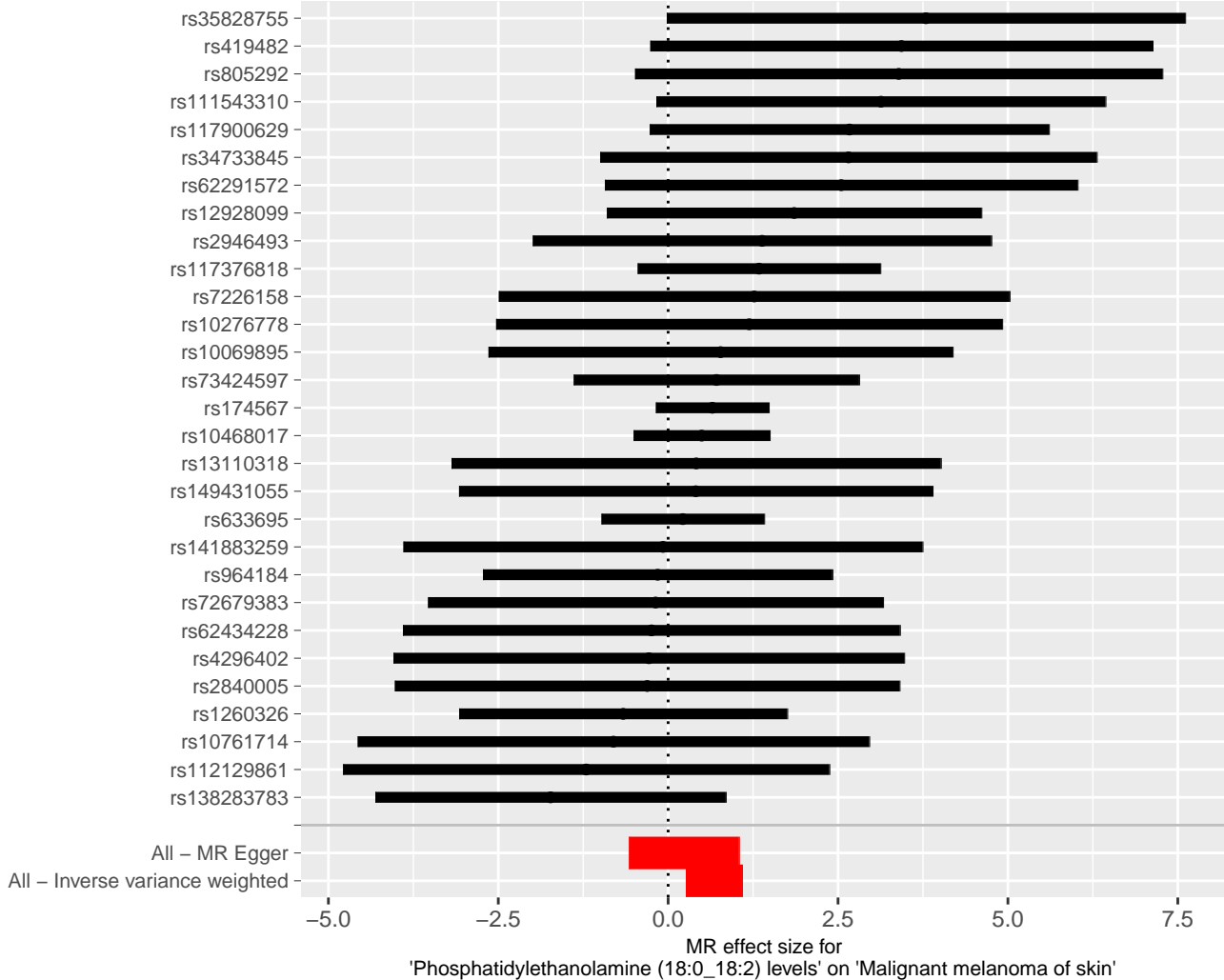

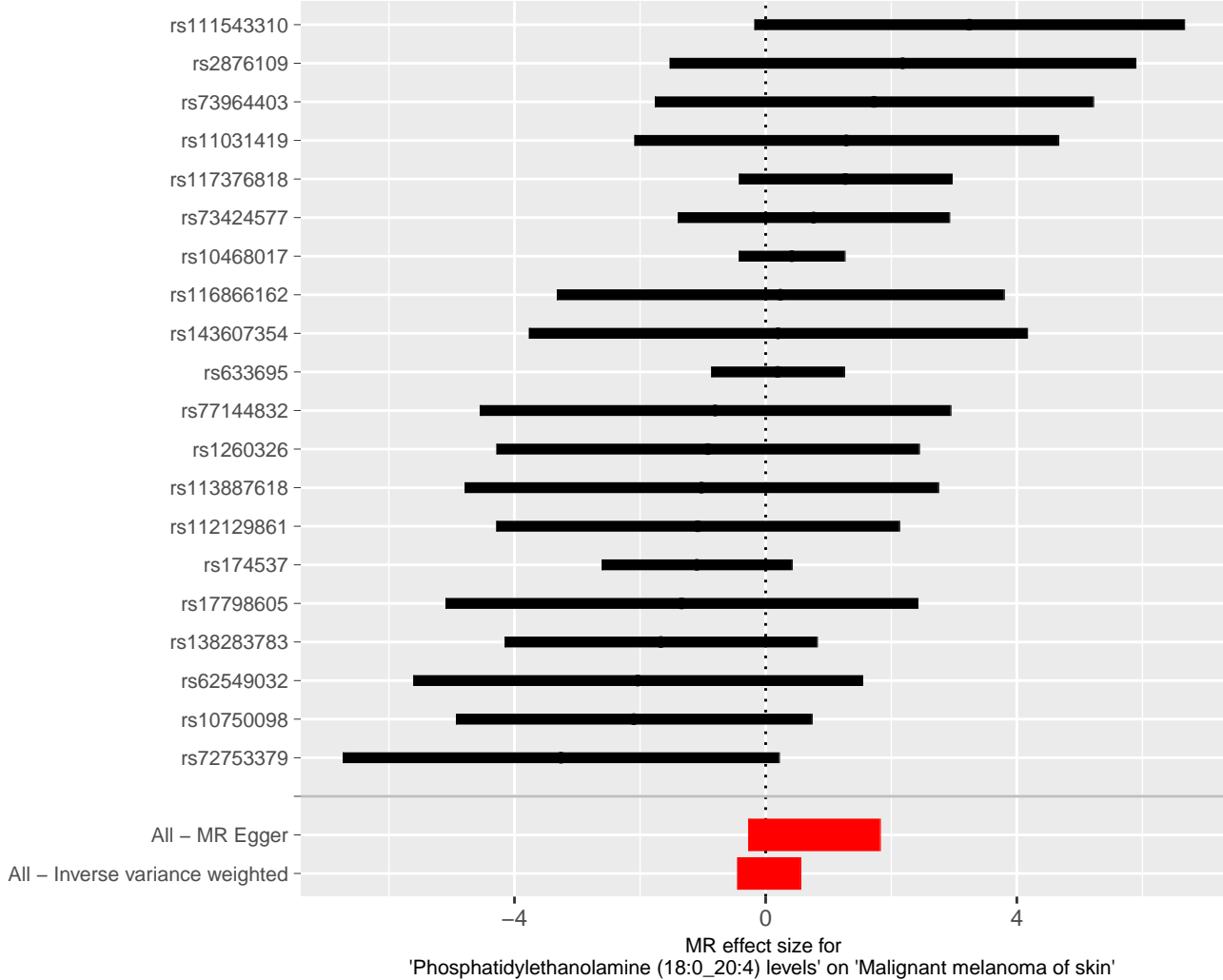

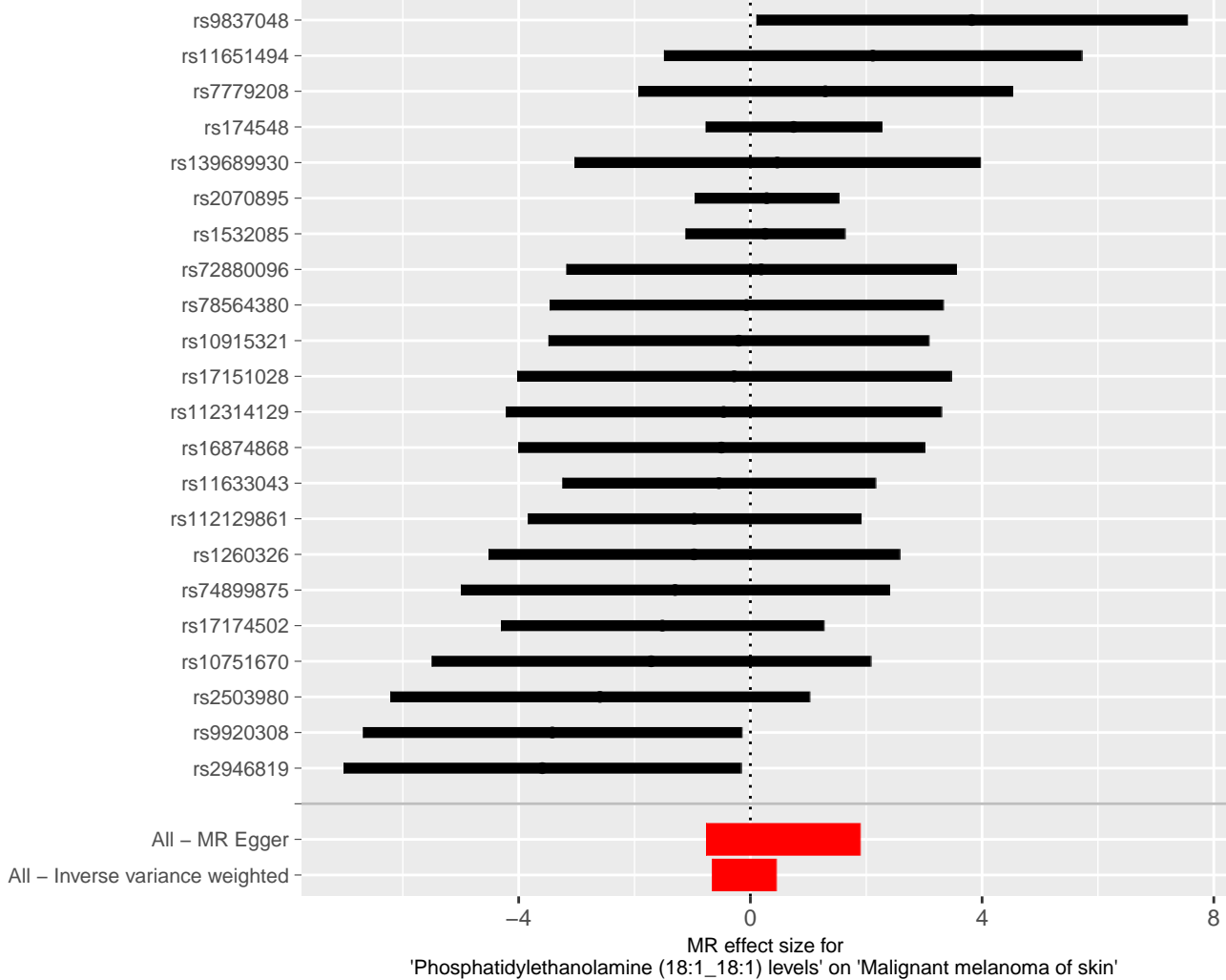

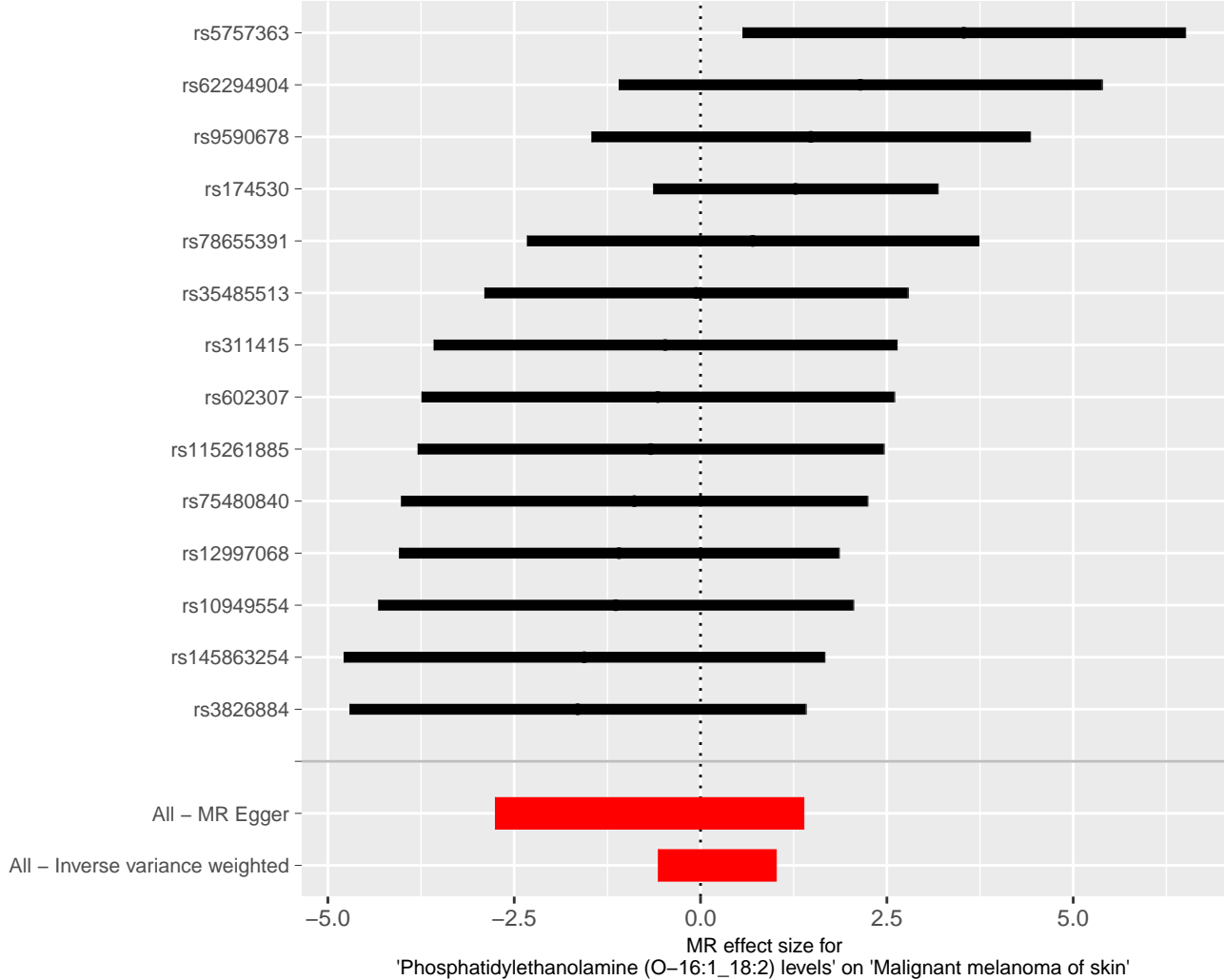

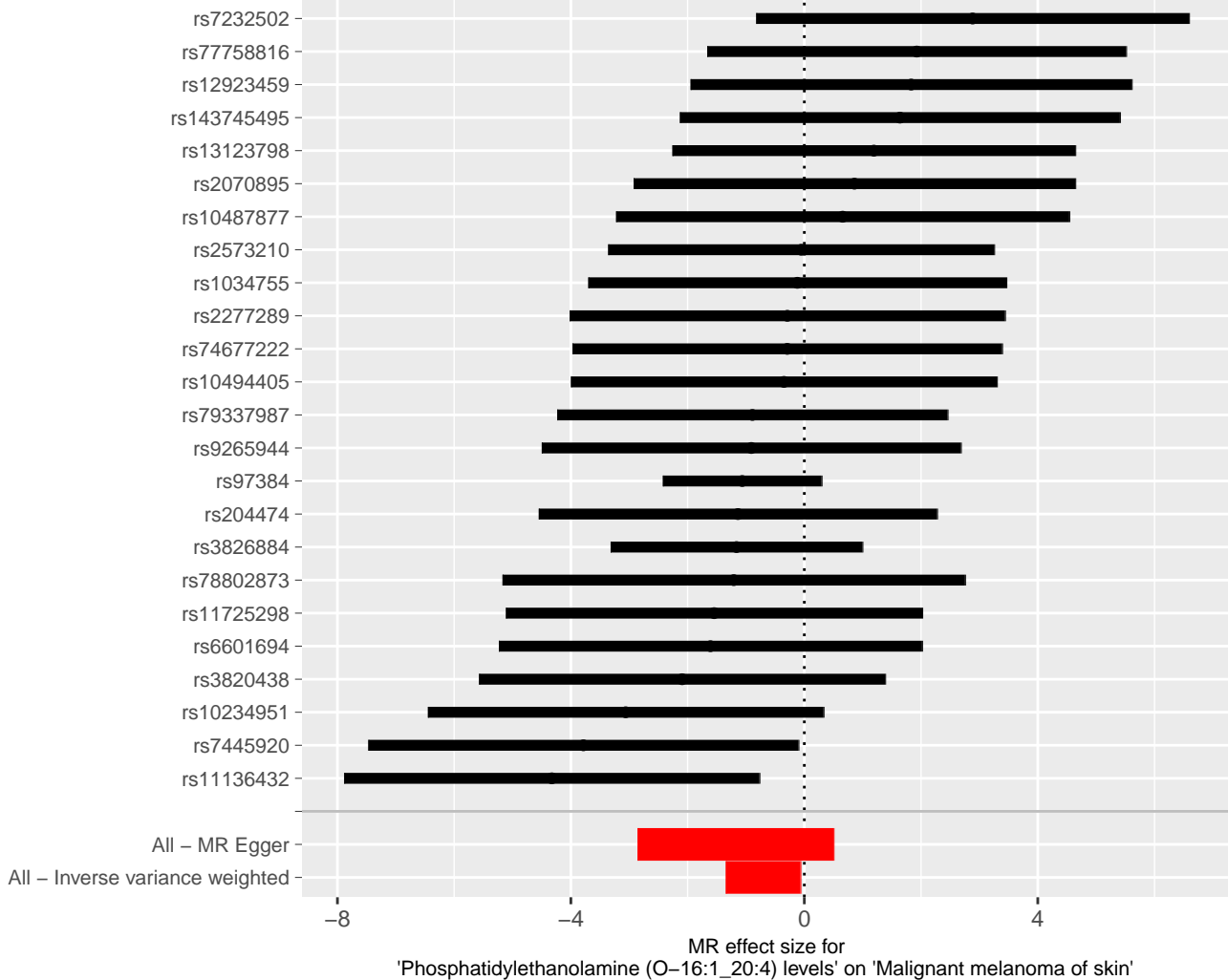

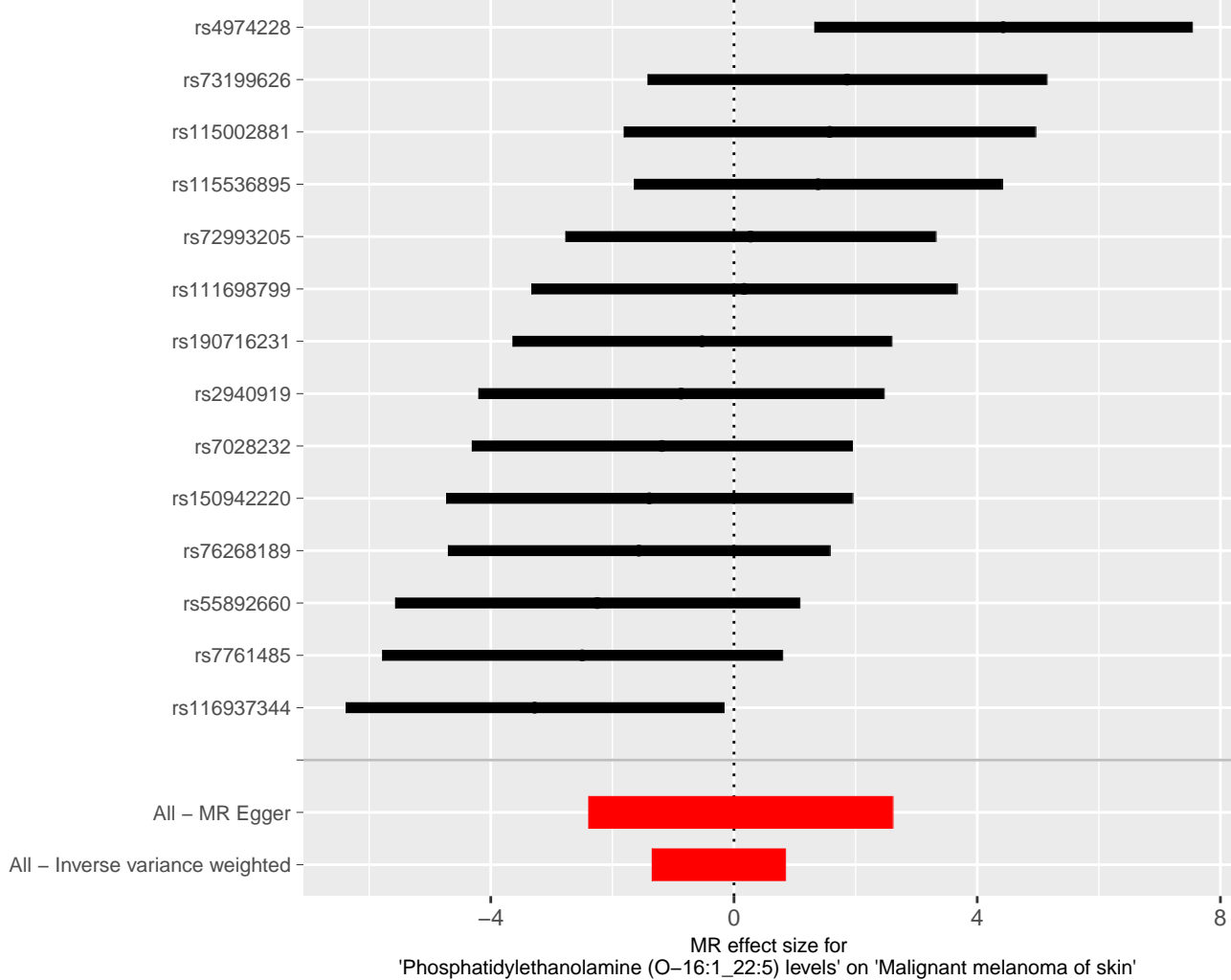

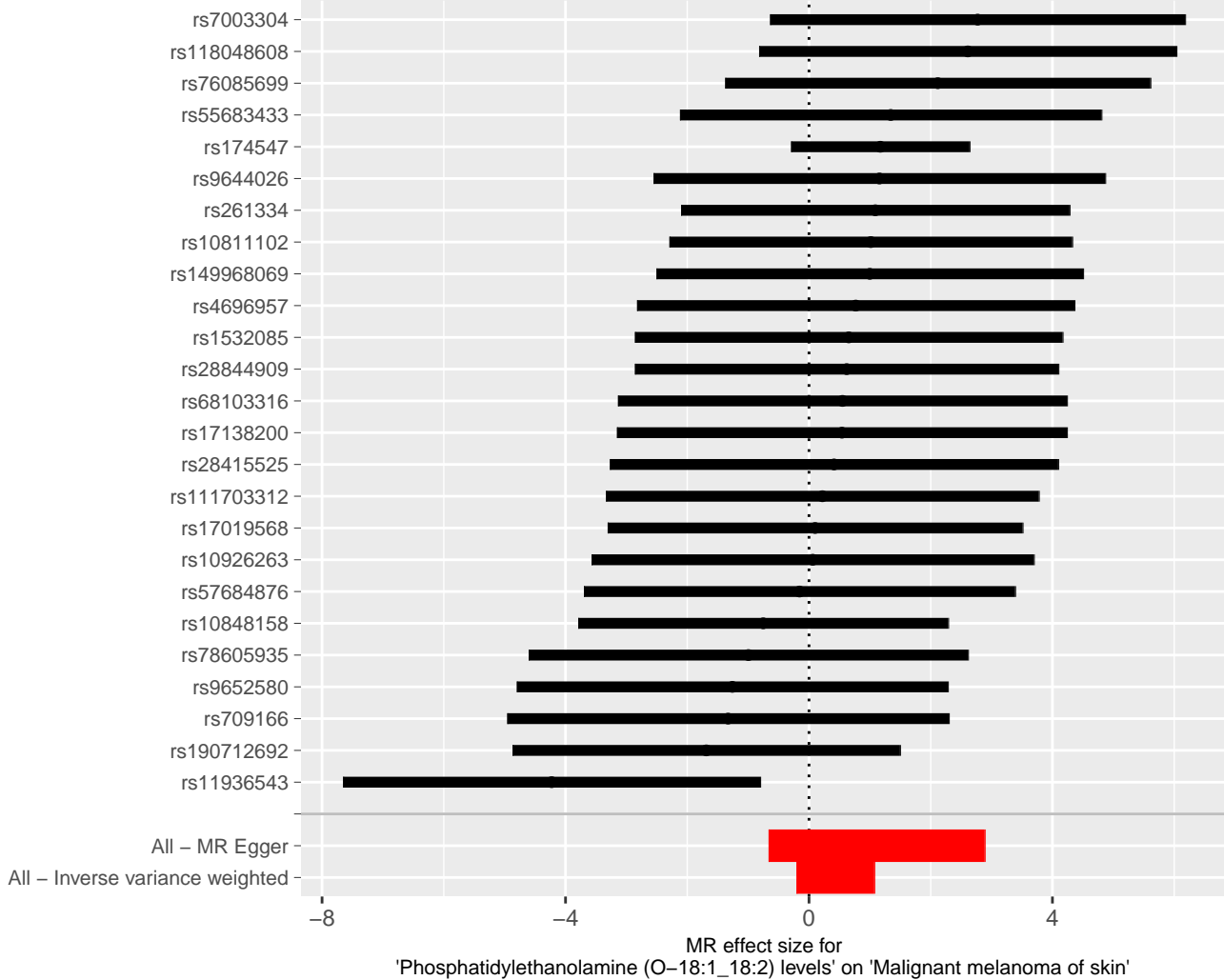

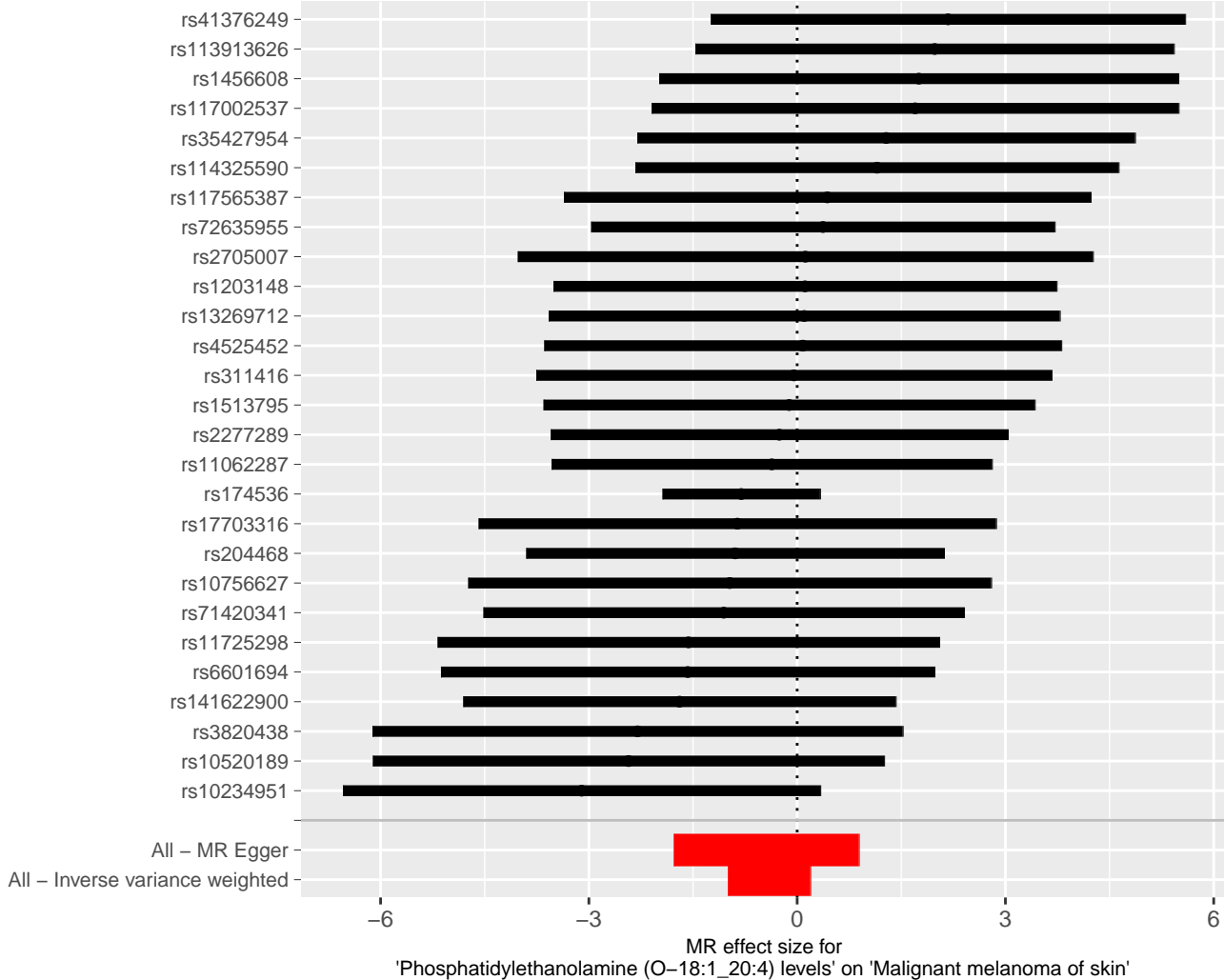

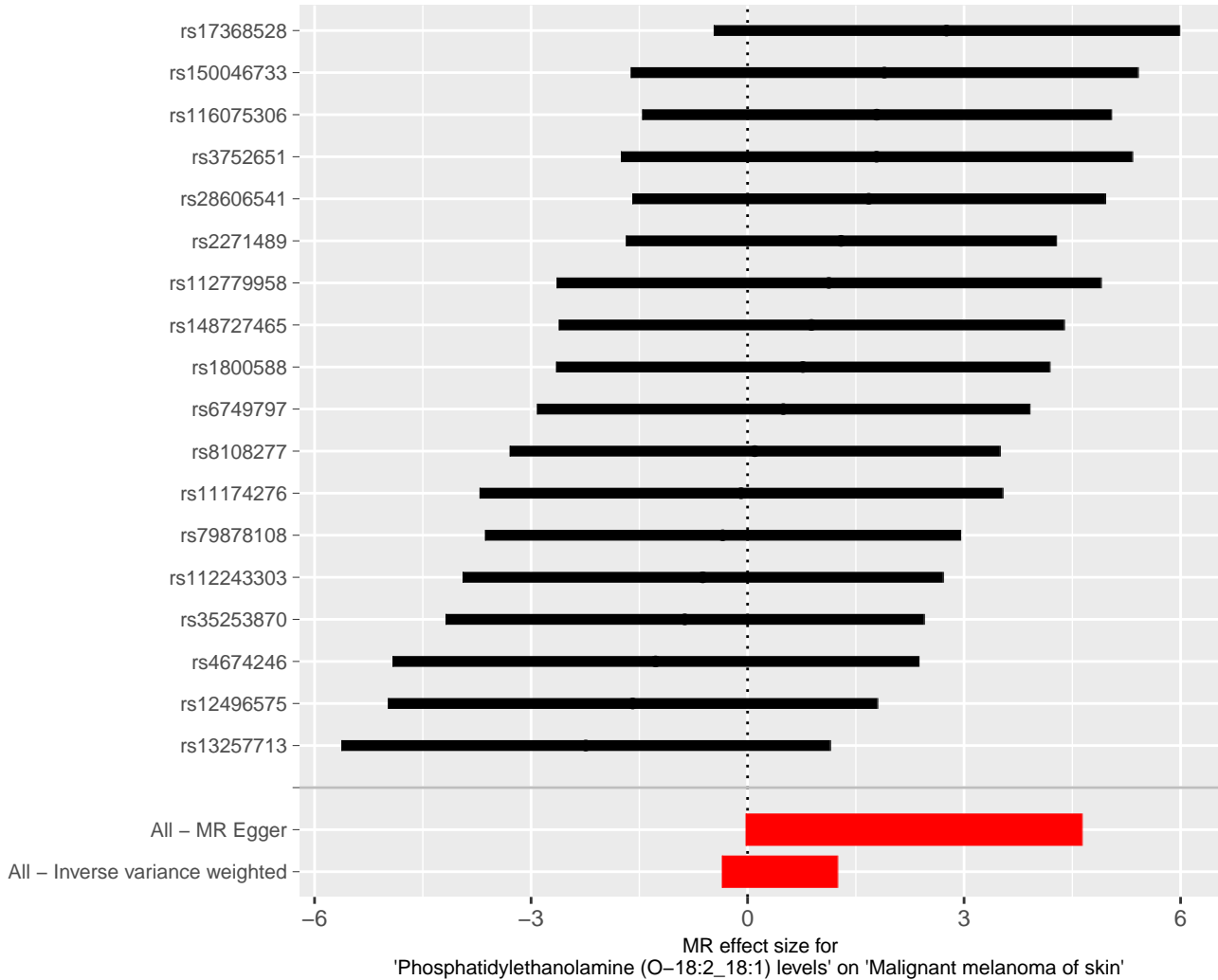

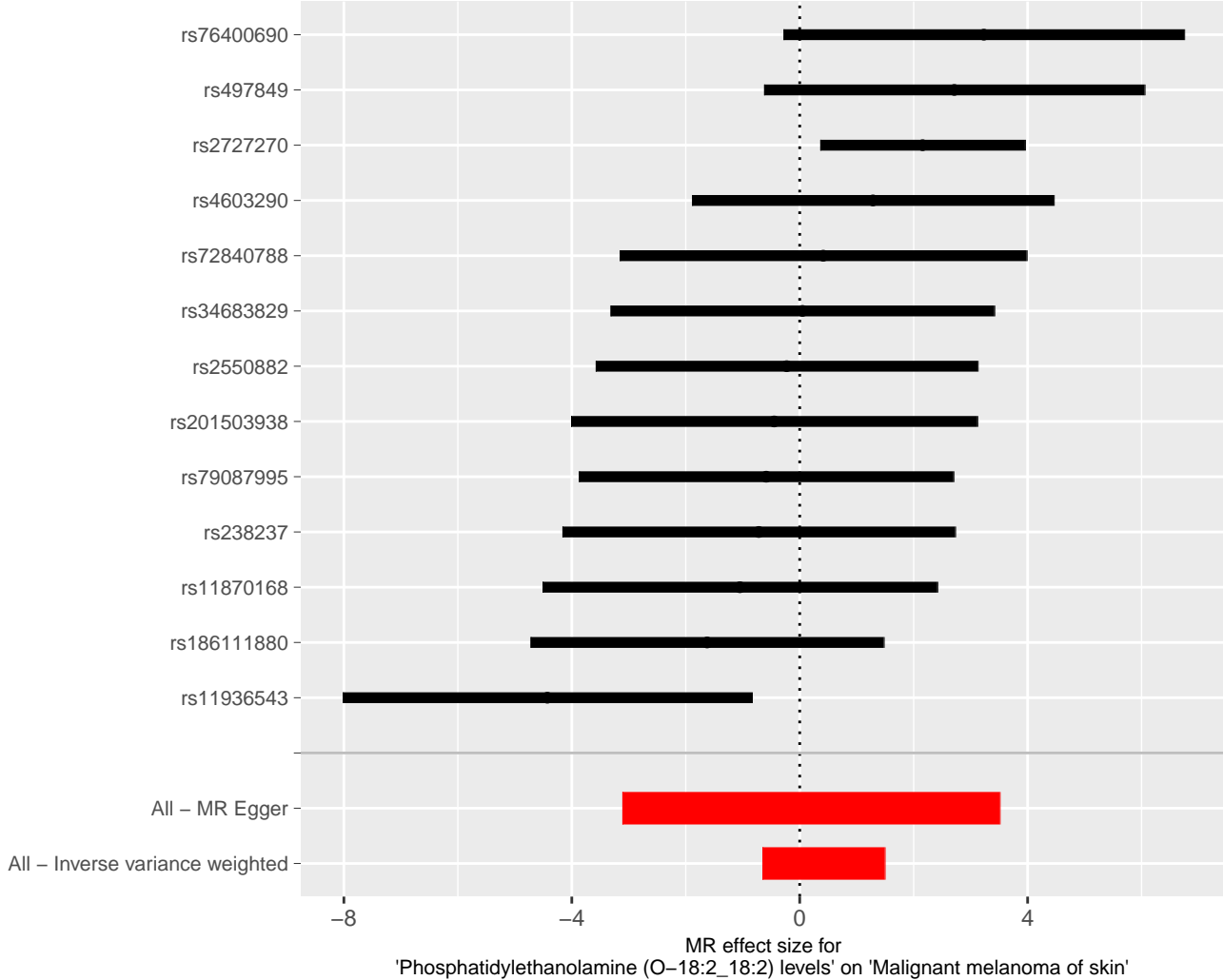

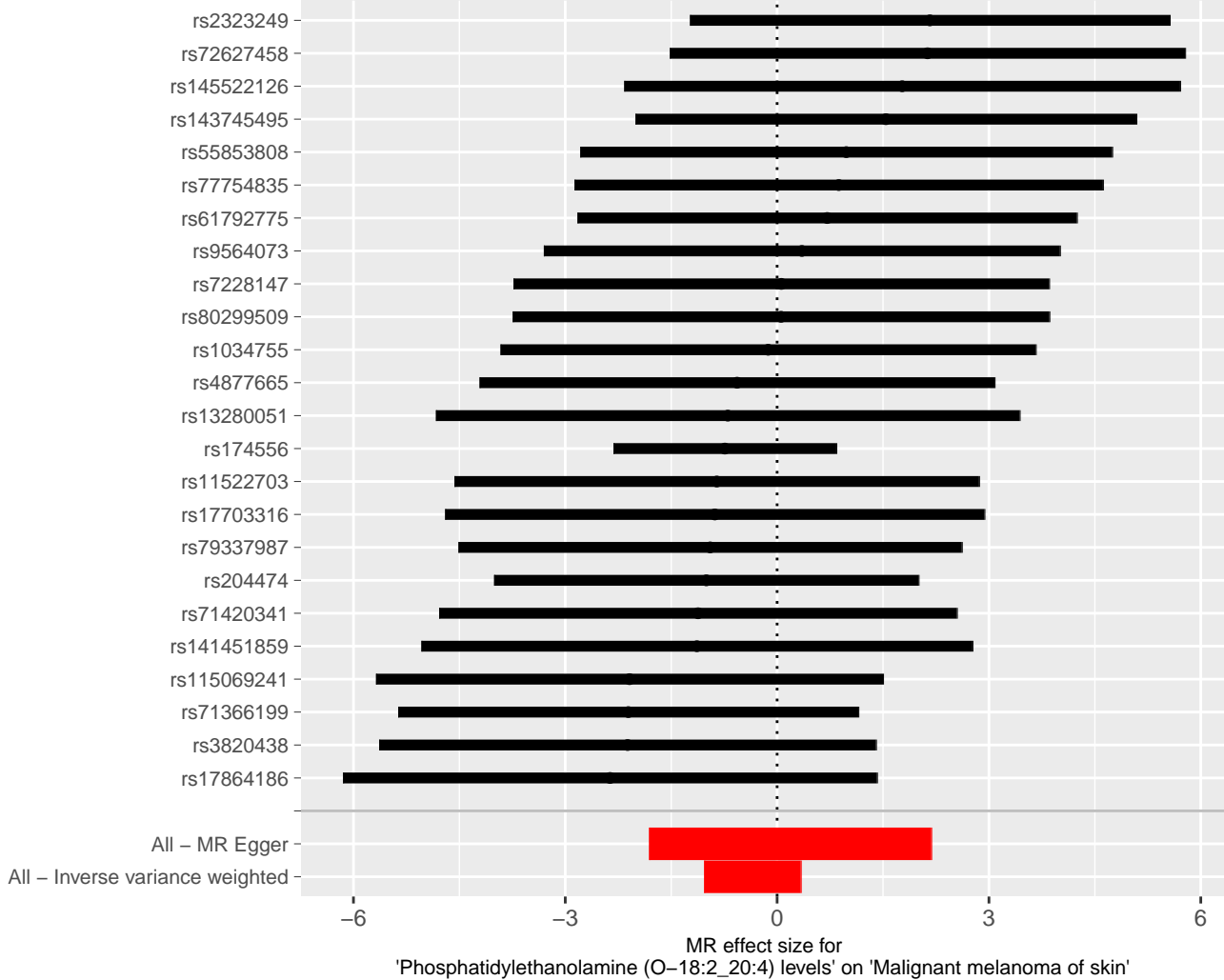

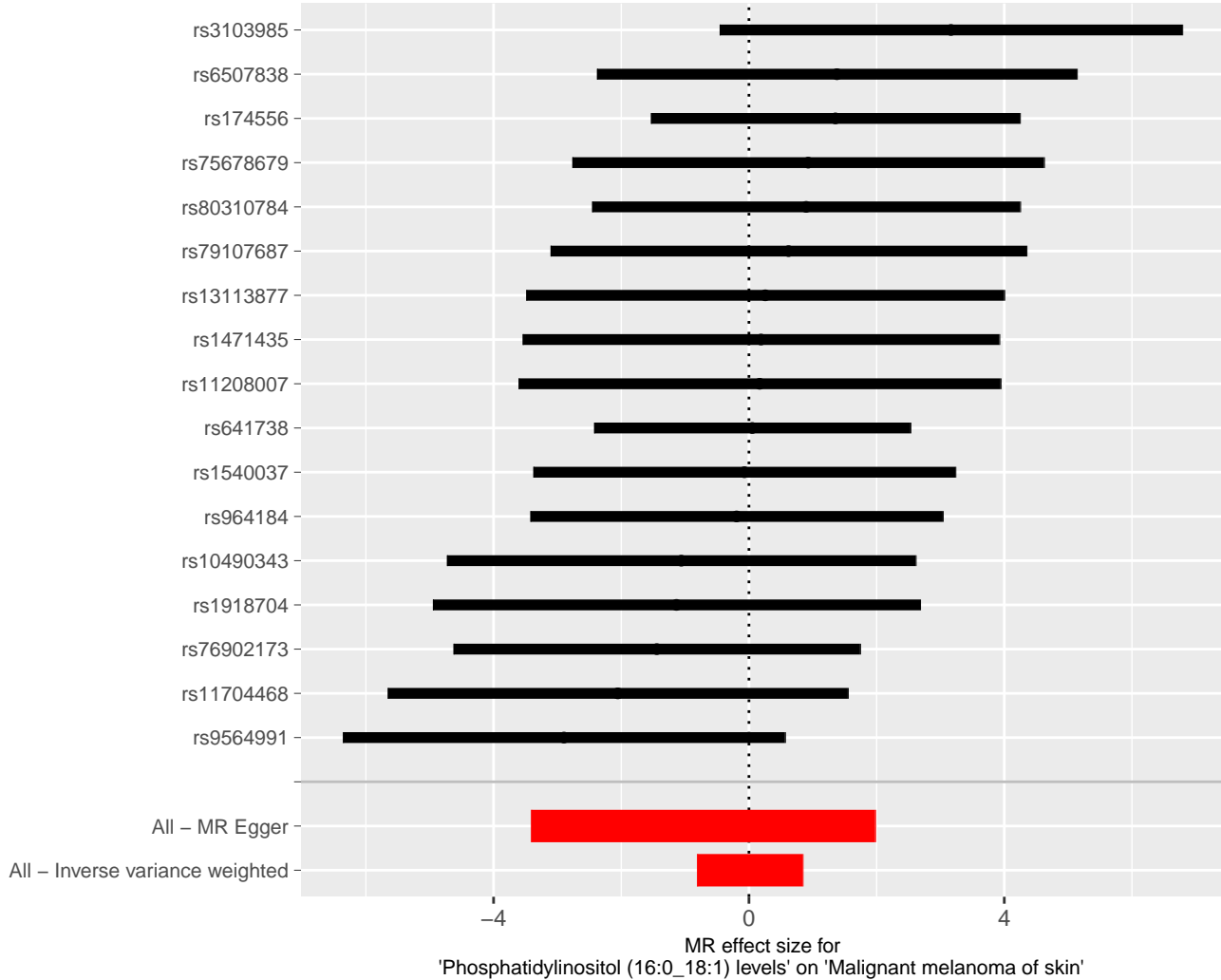

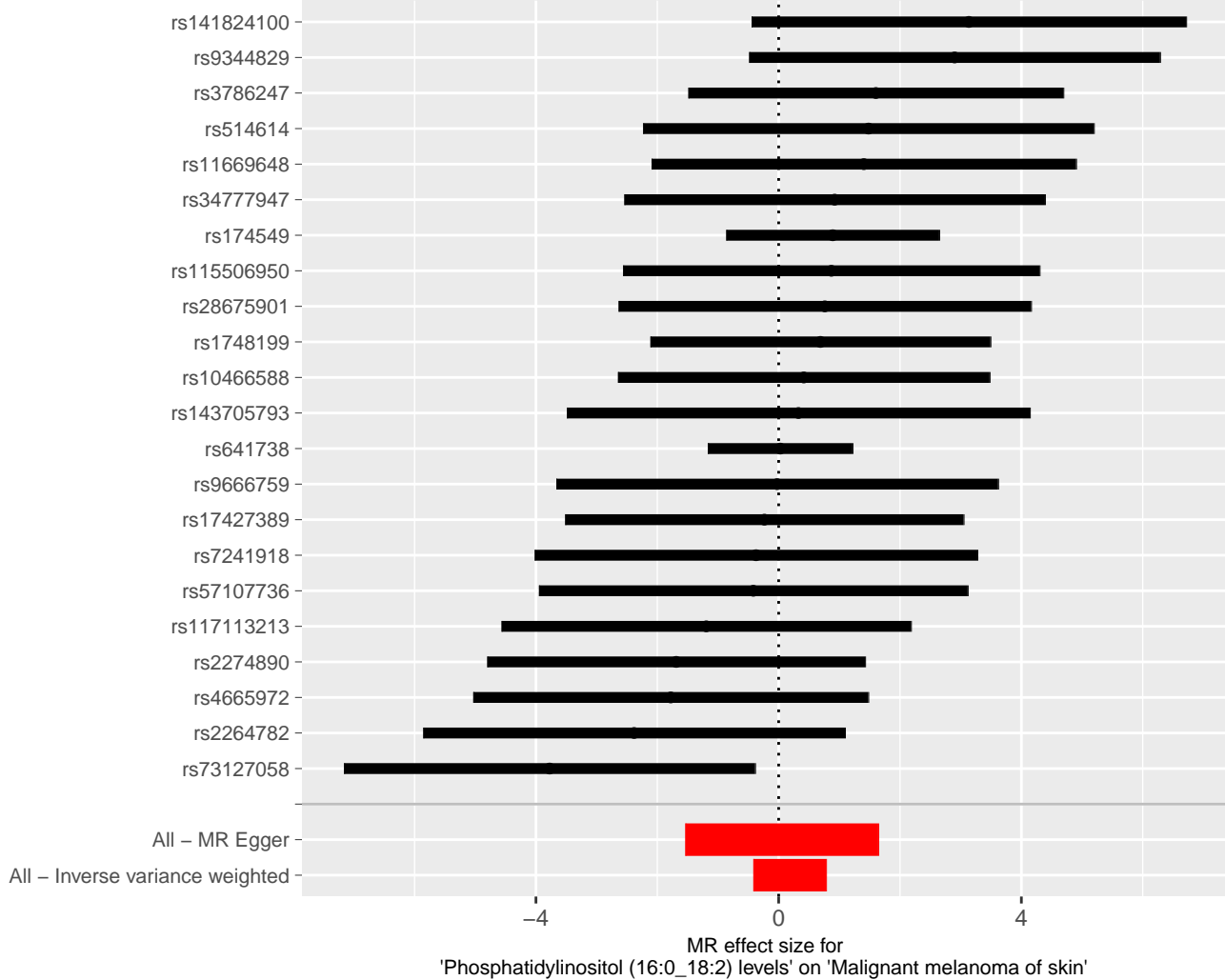

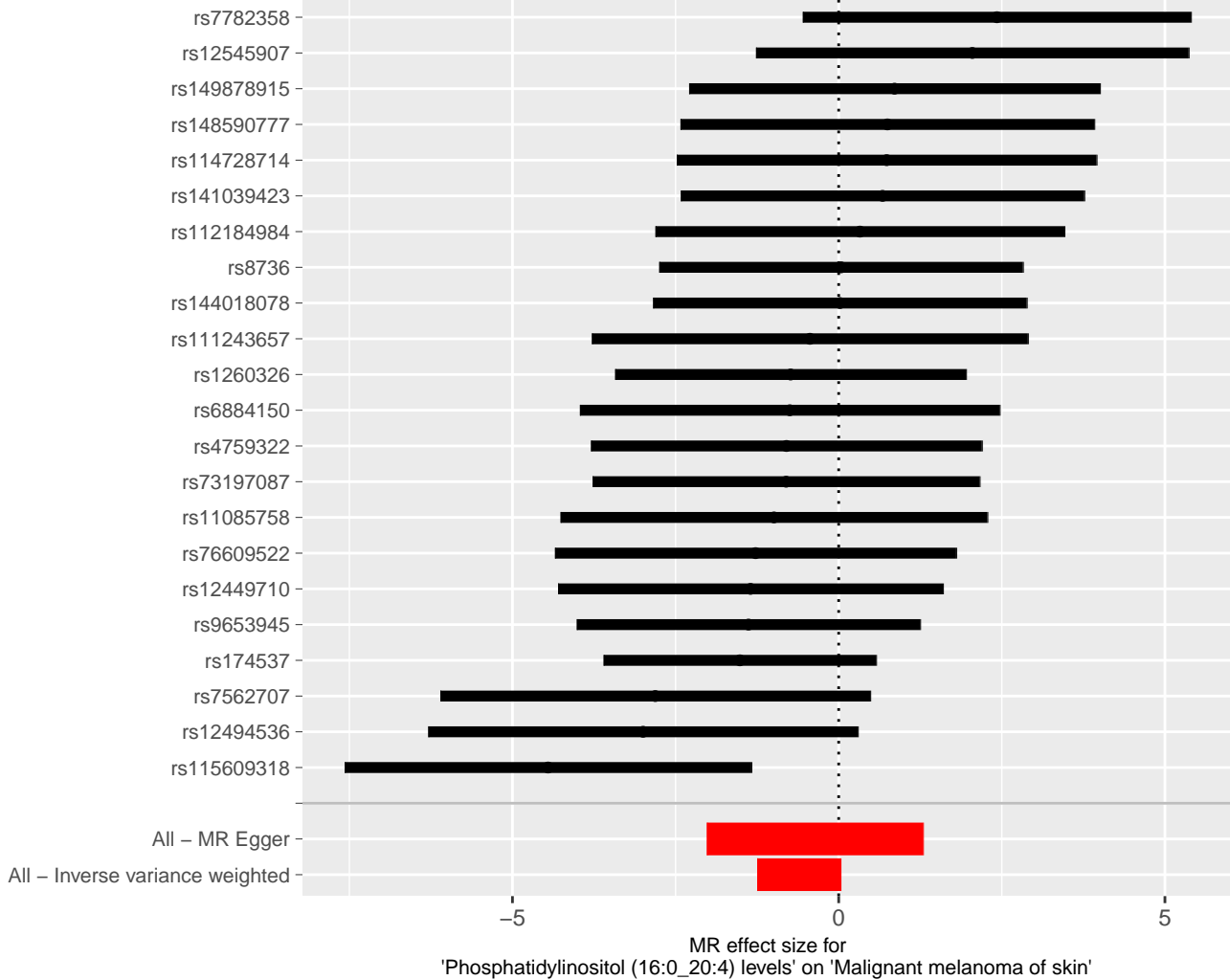

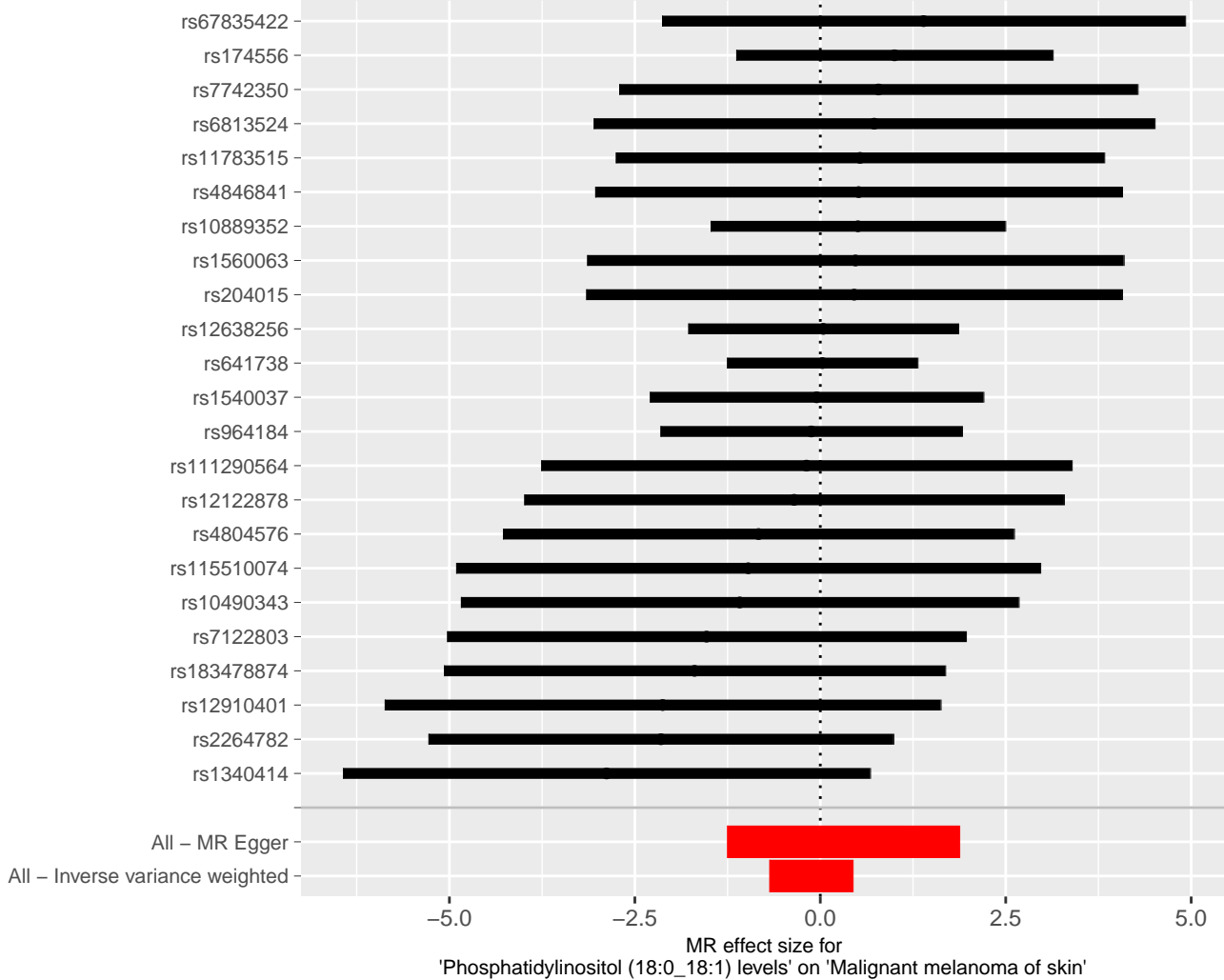

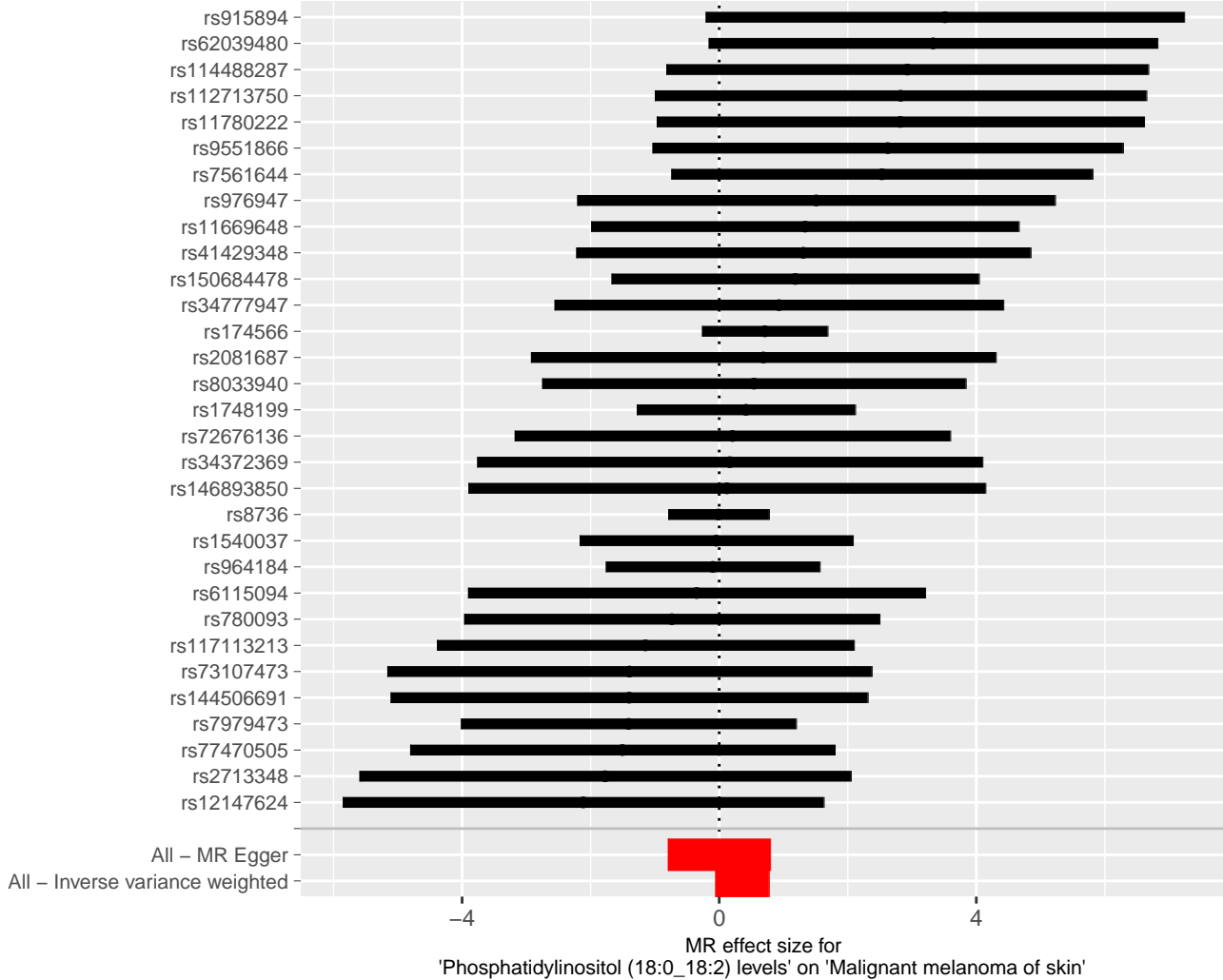

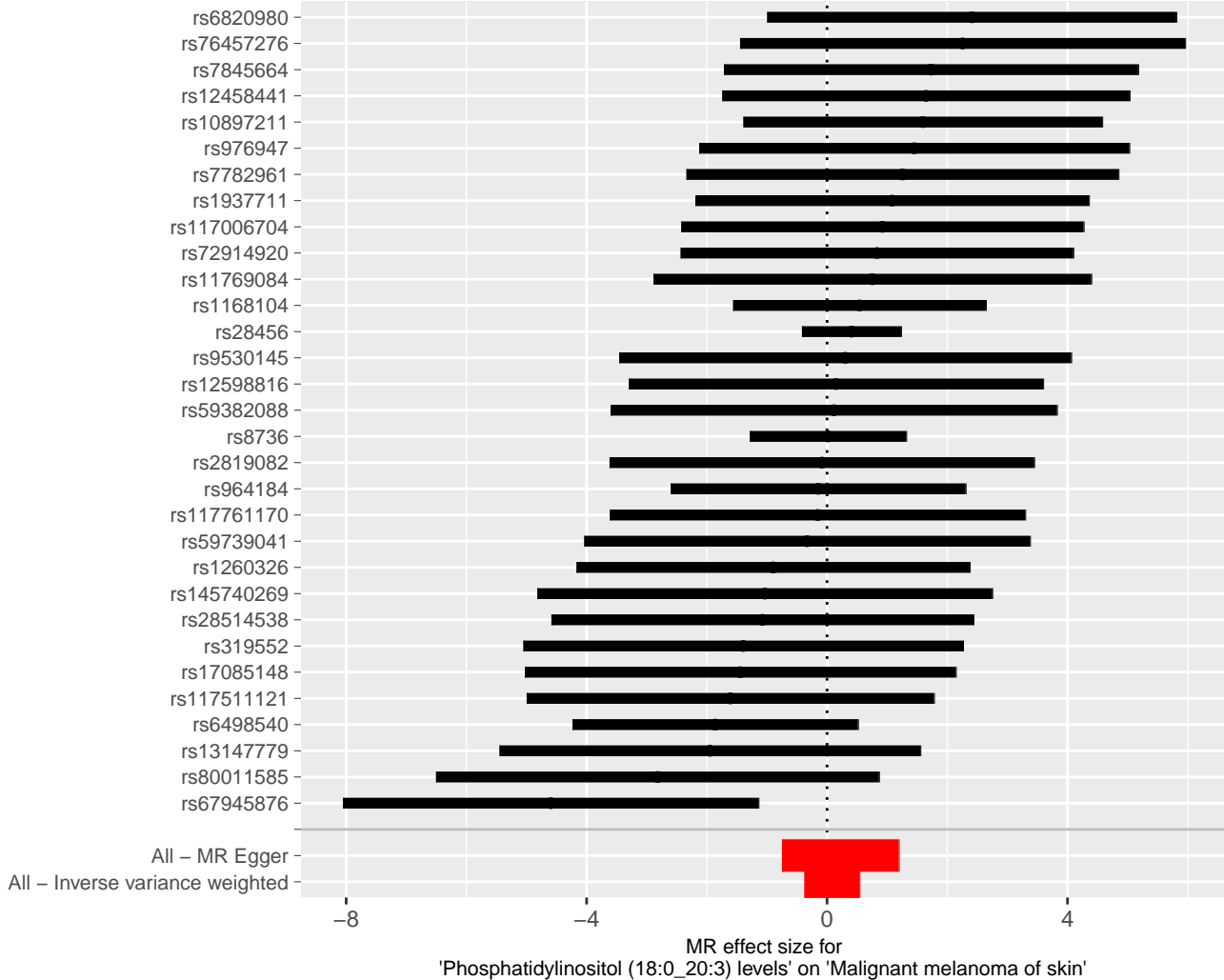

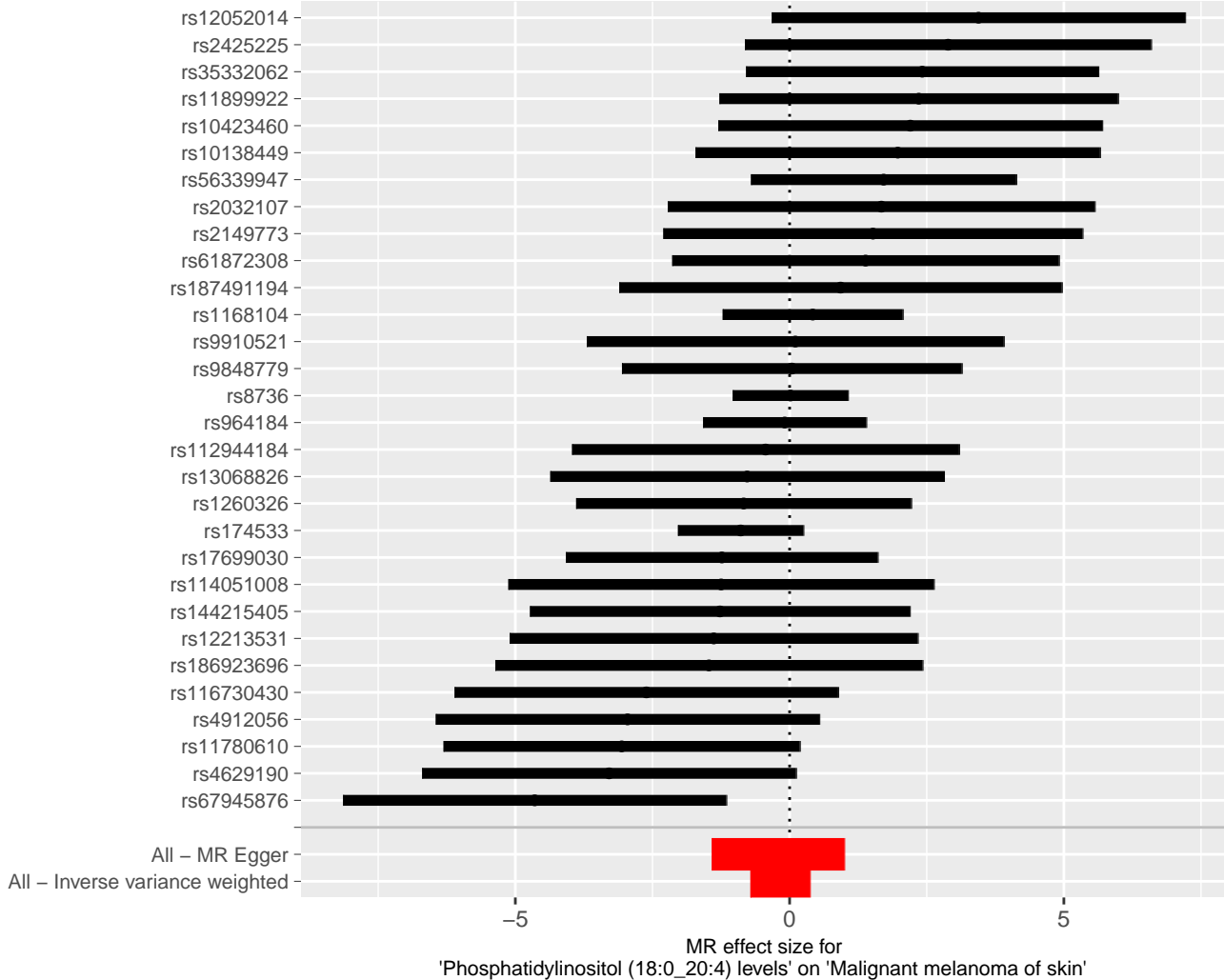

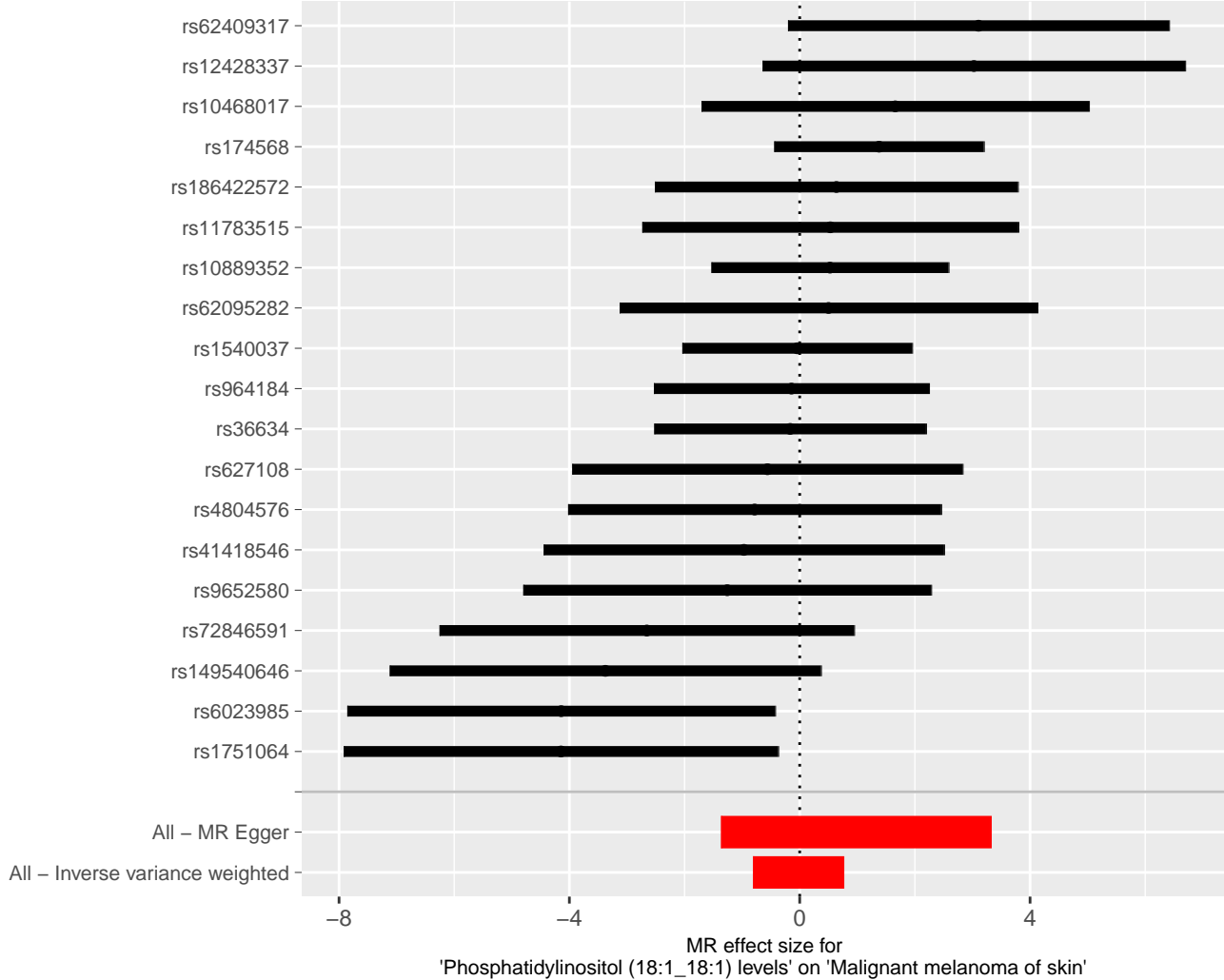

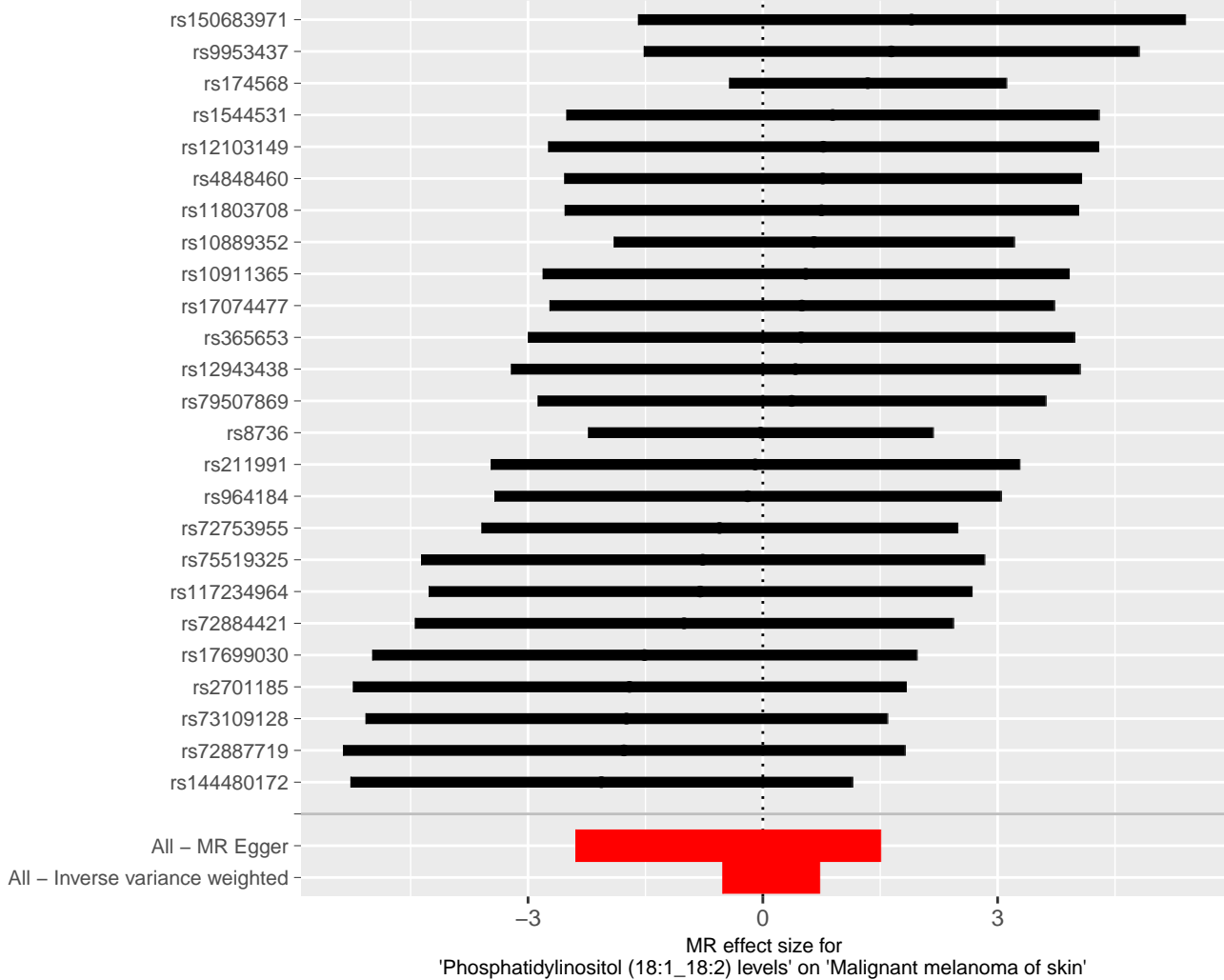

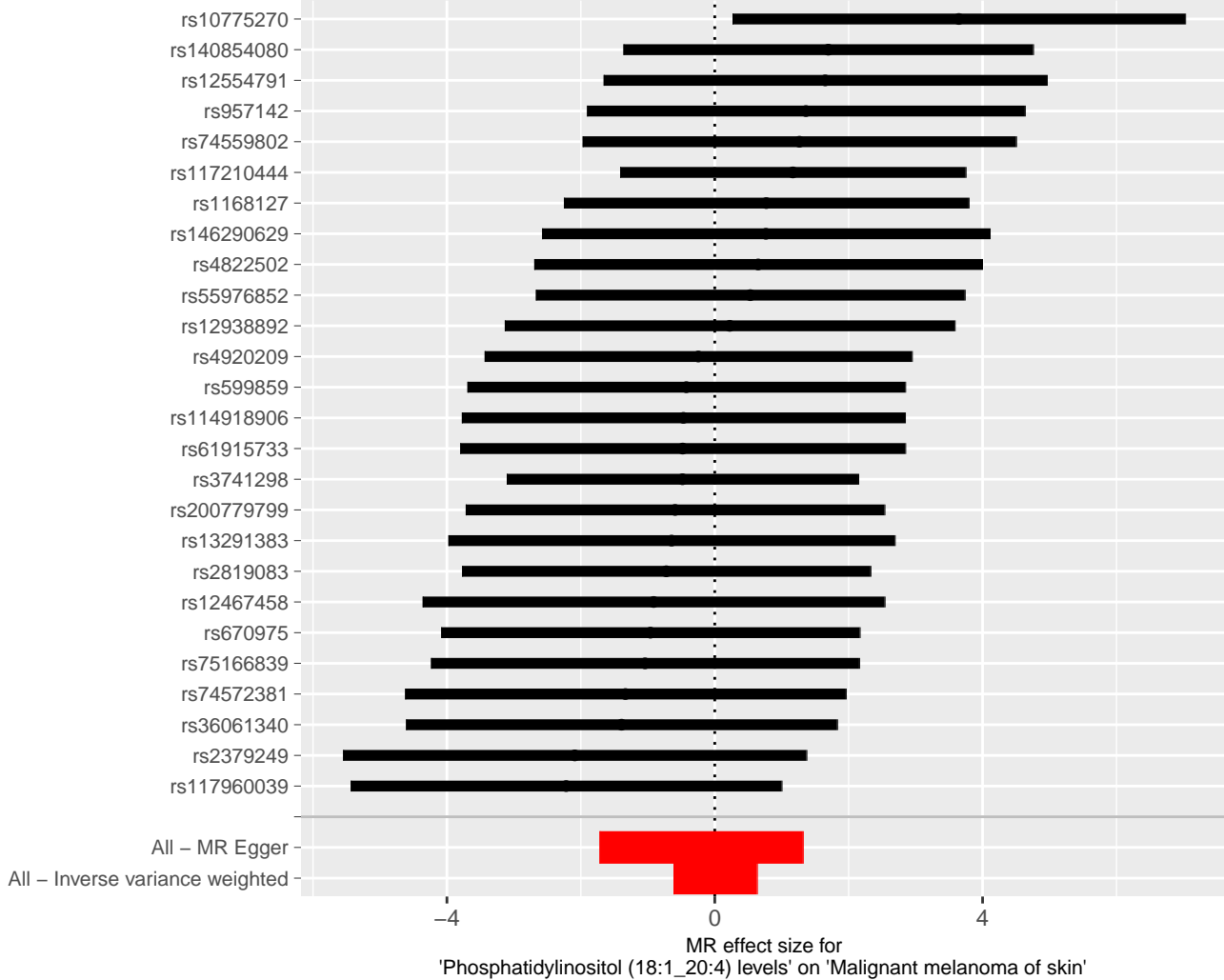

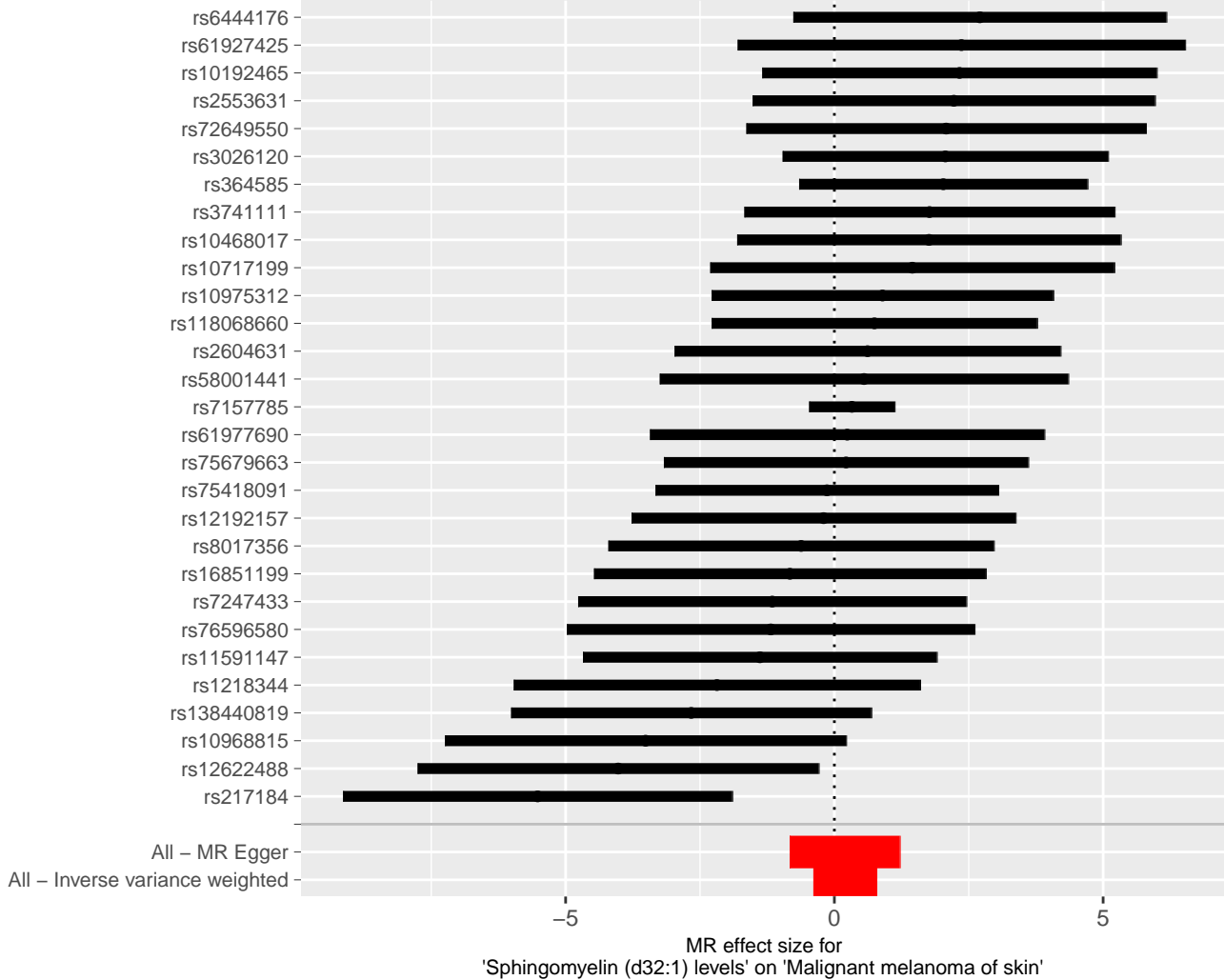

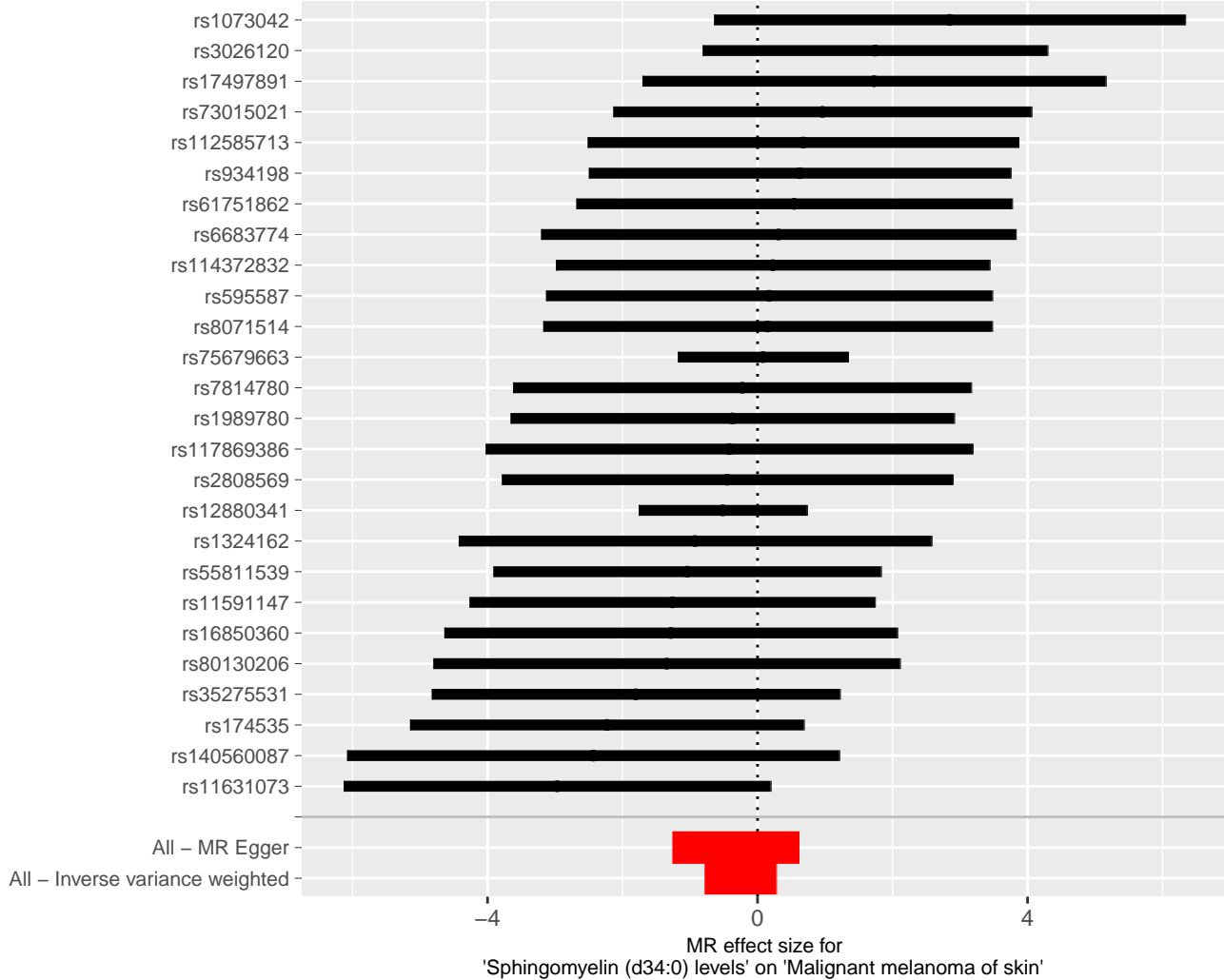

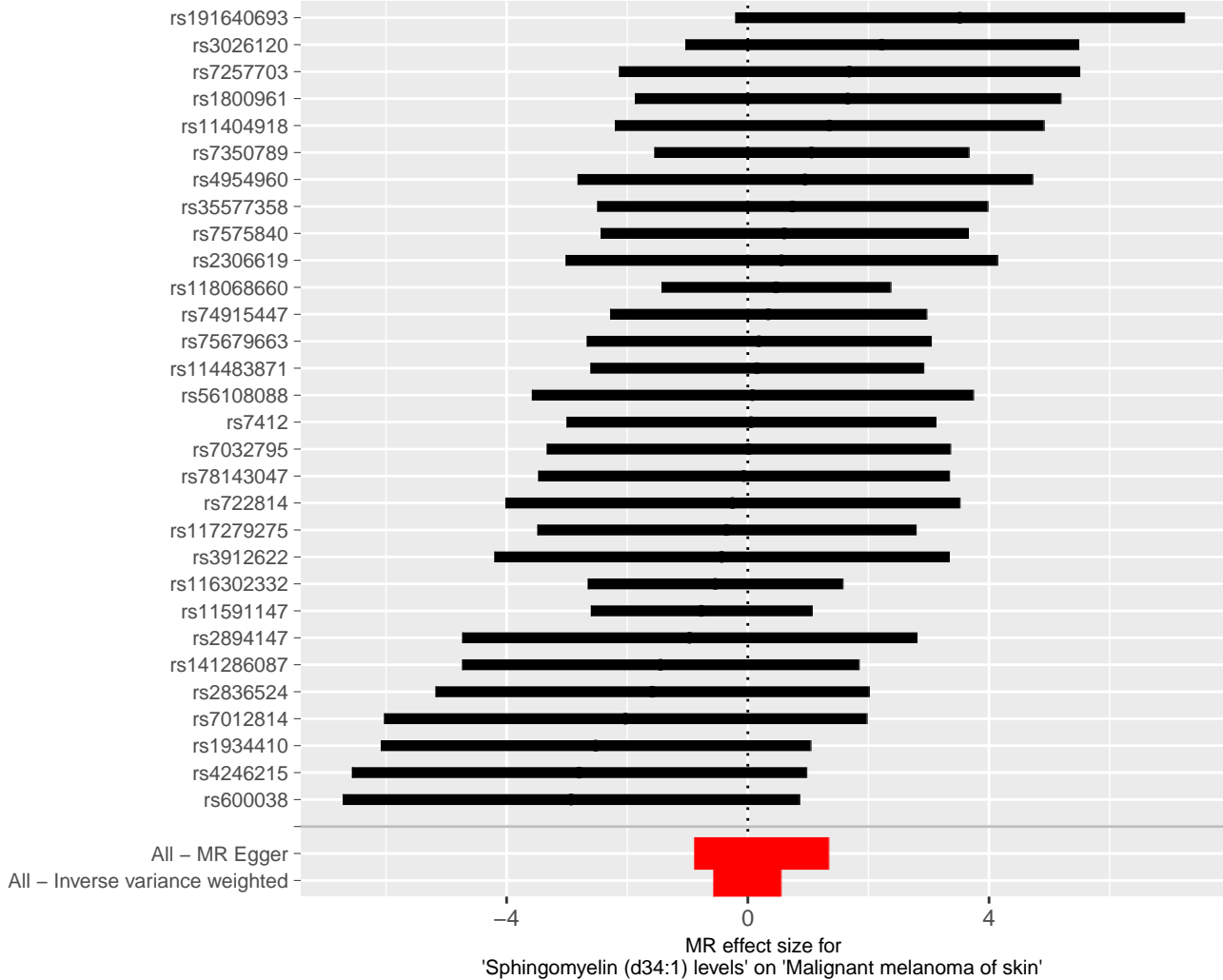

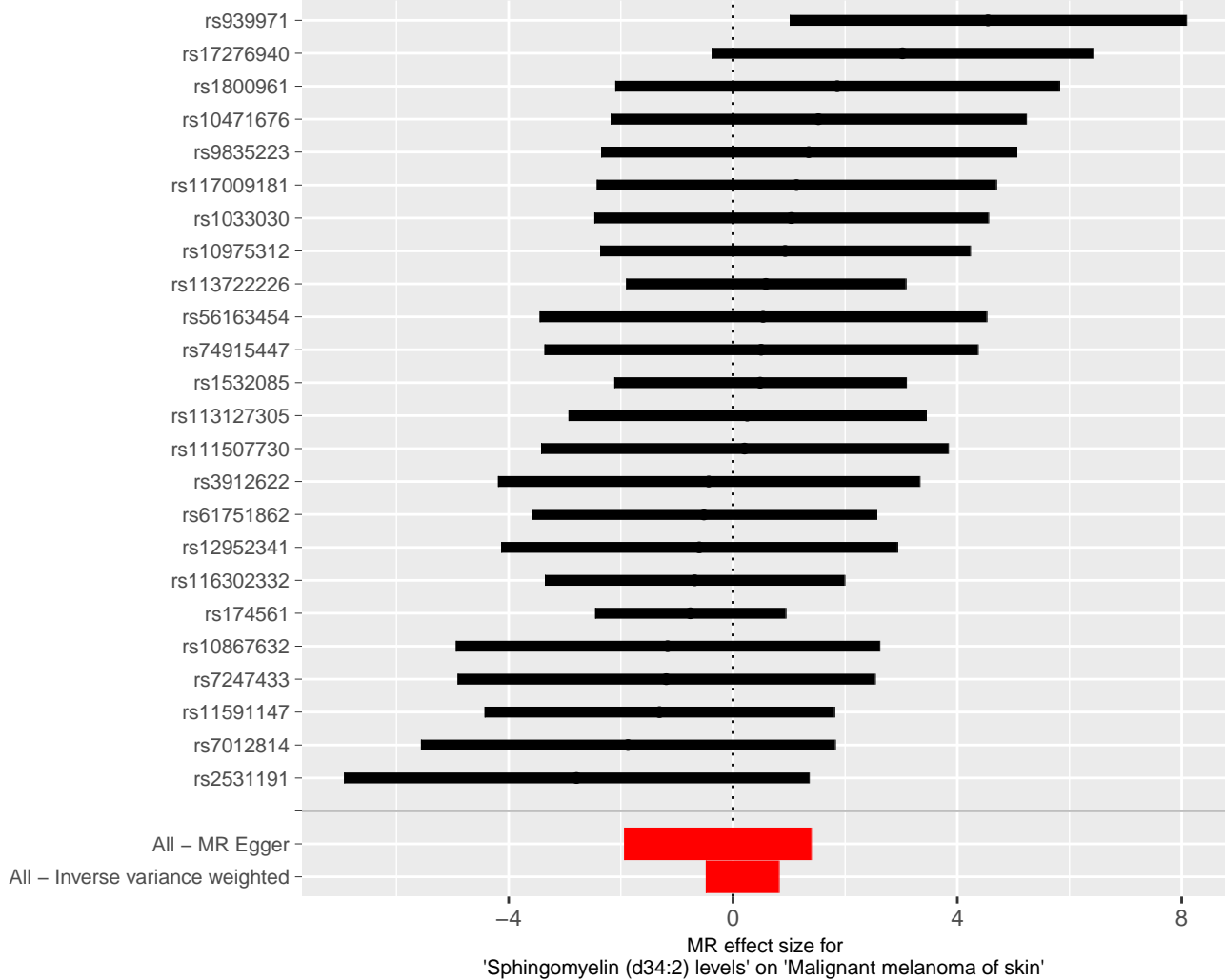

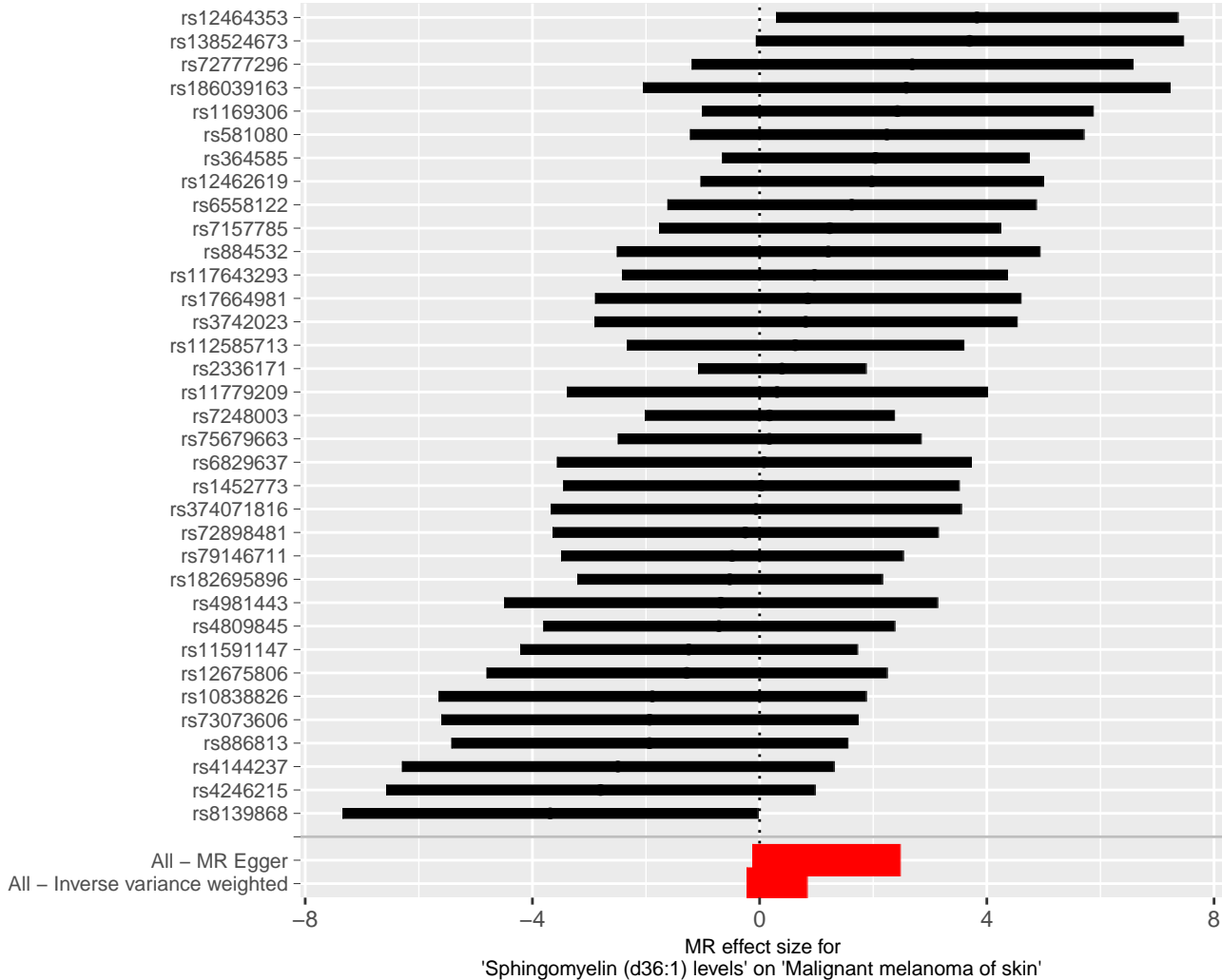

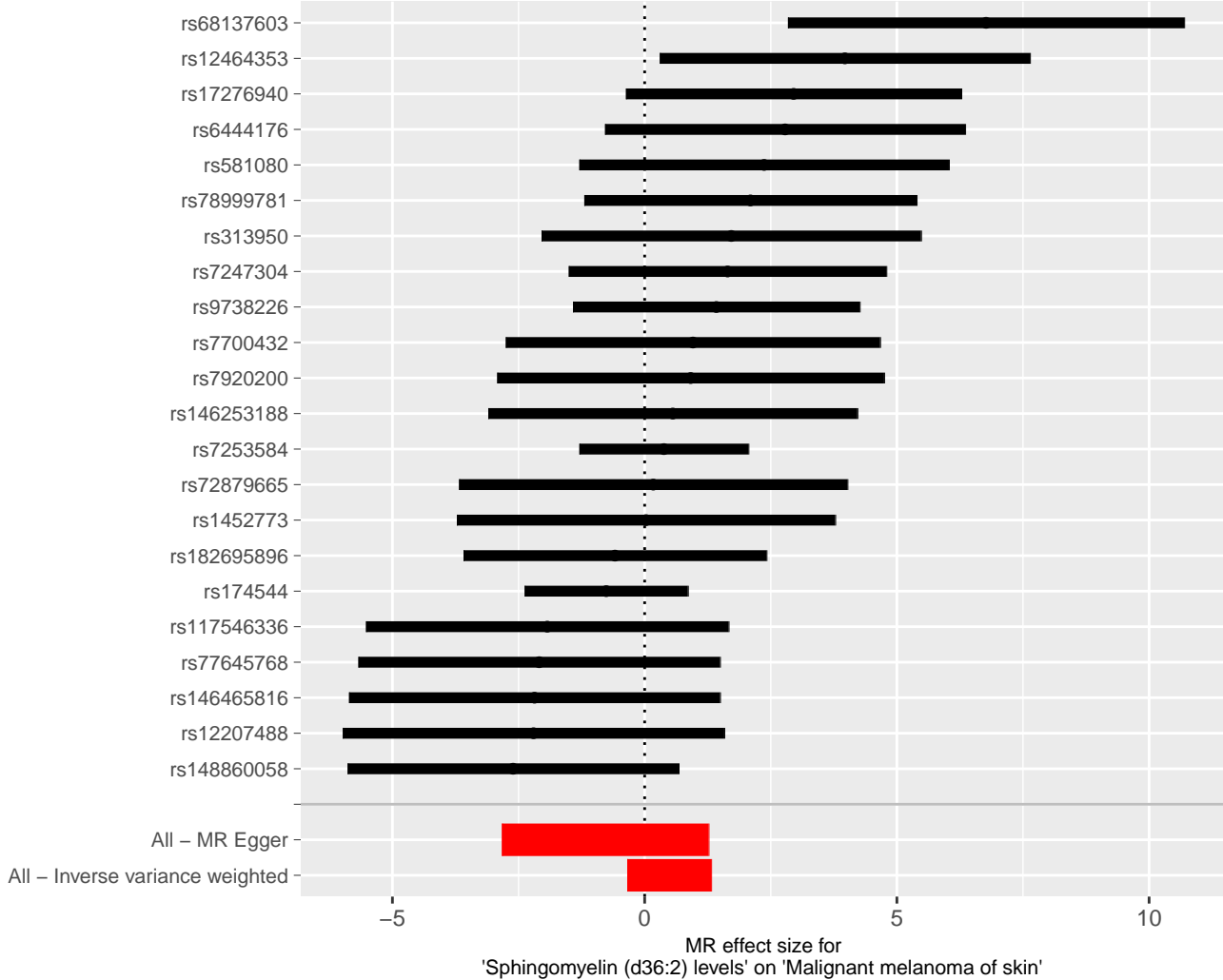

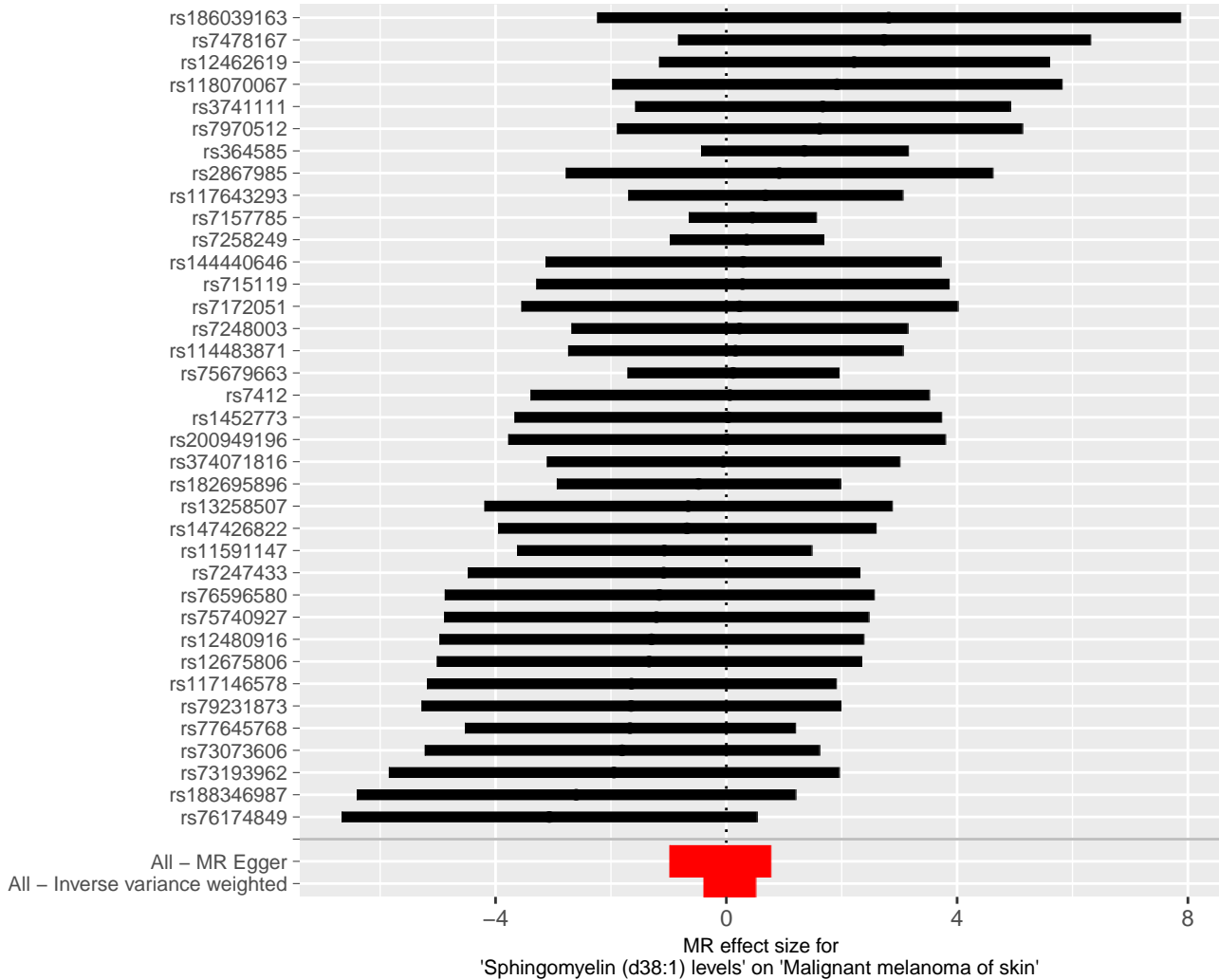

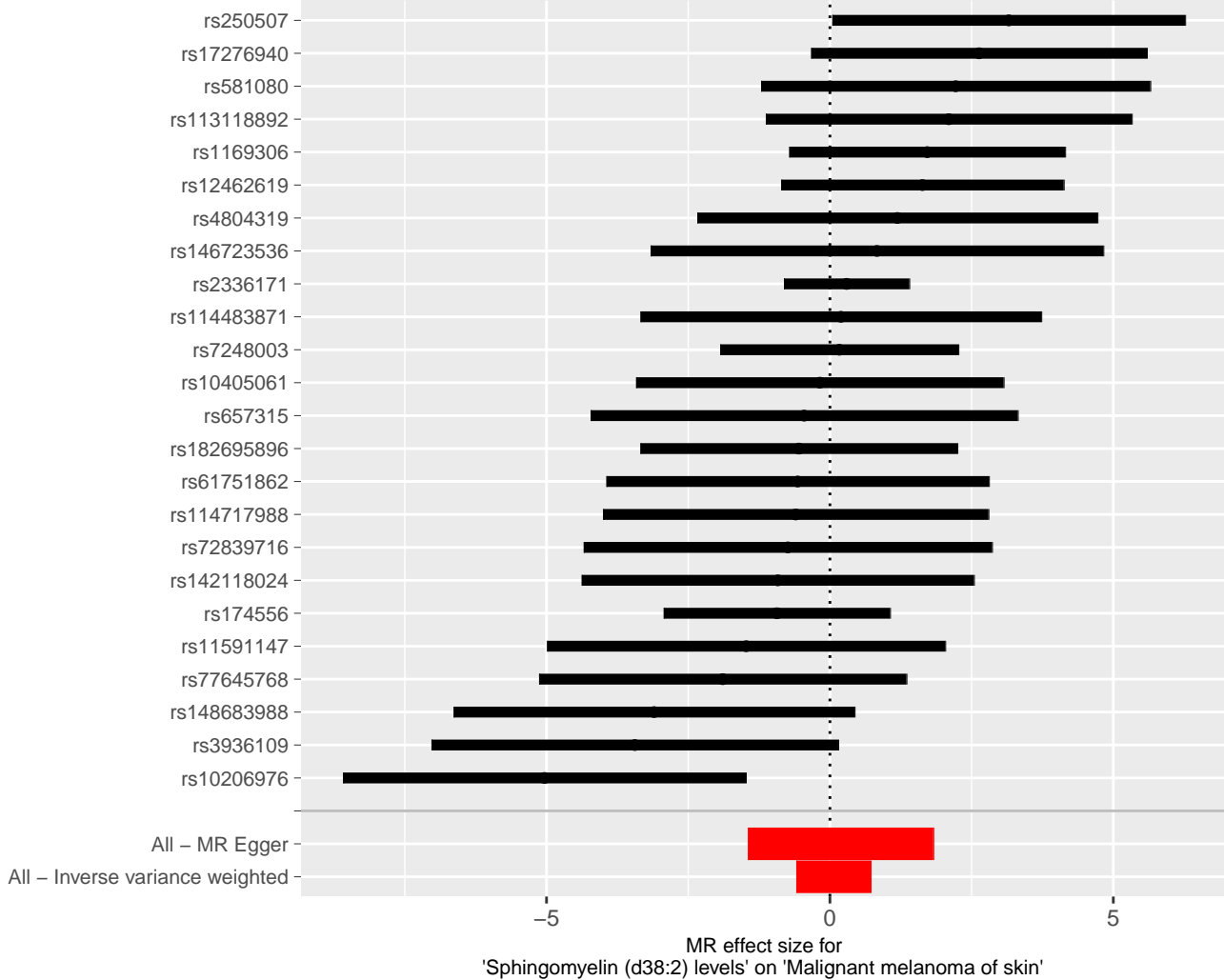

All – MR Egger  
All – Inverse variance weighted

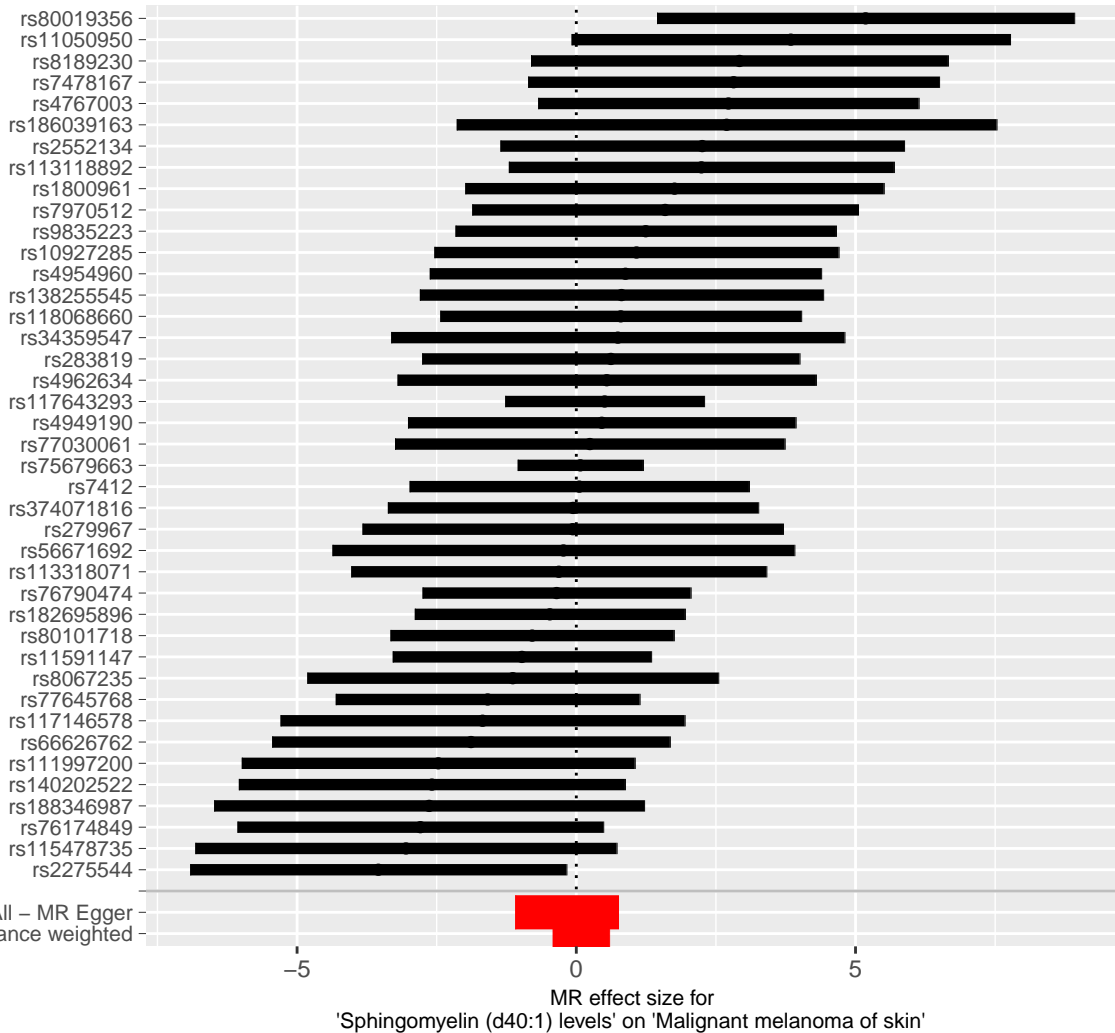

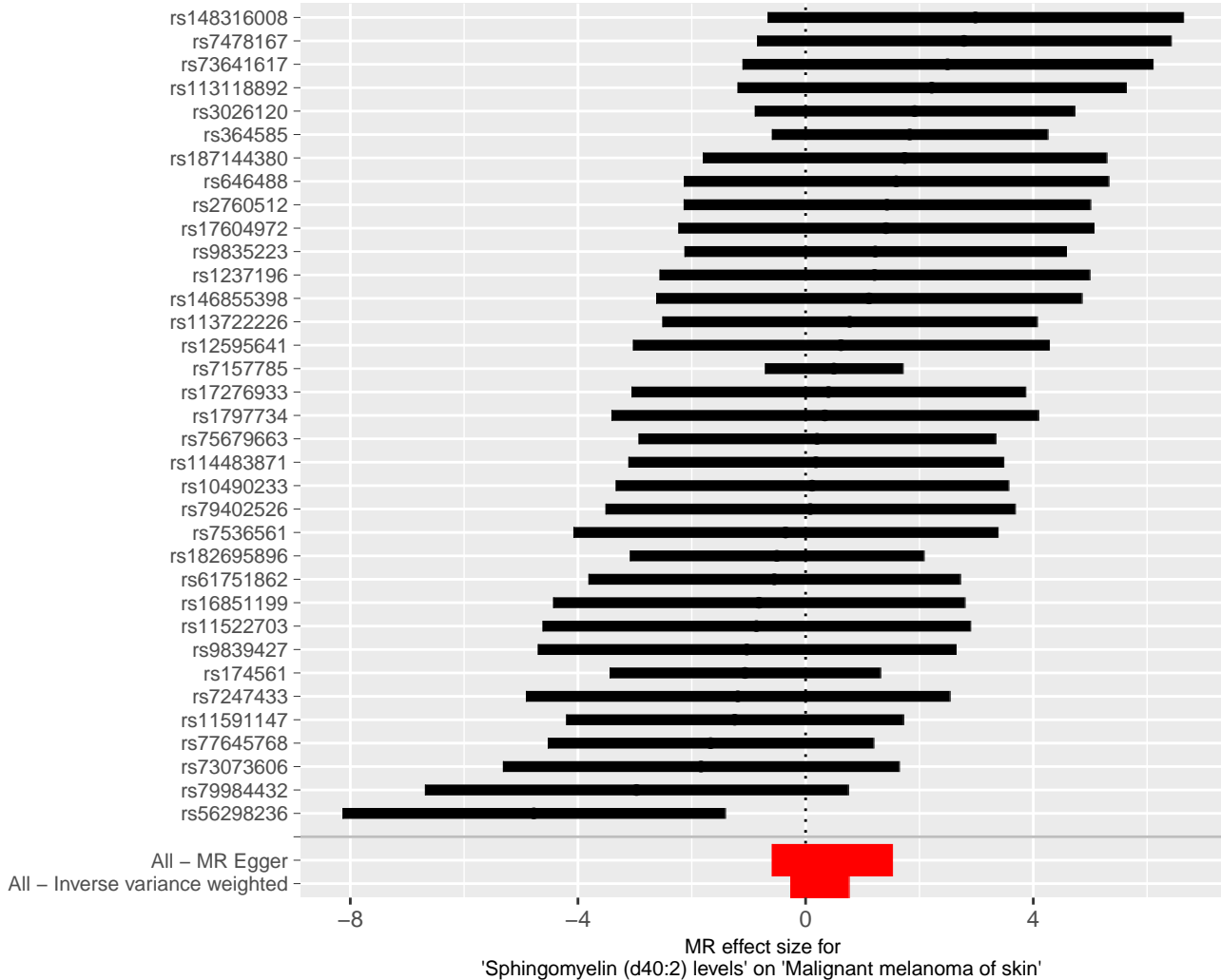

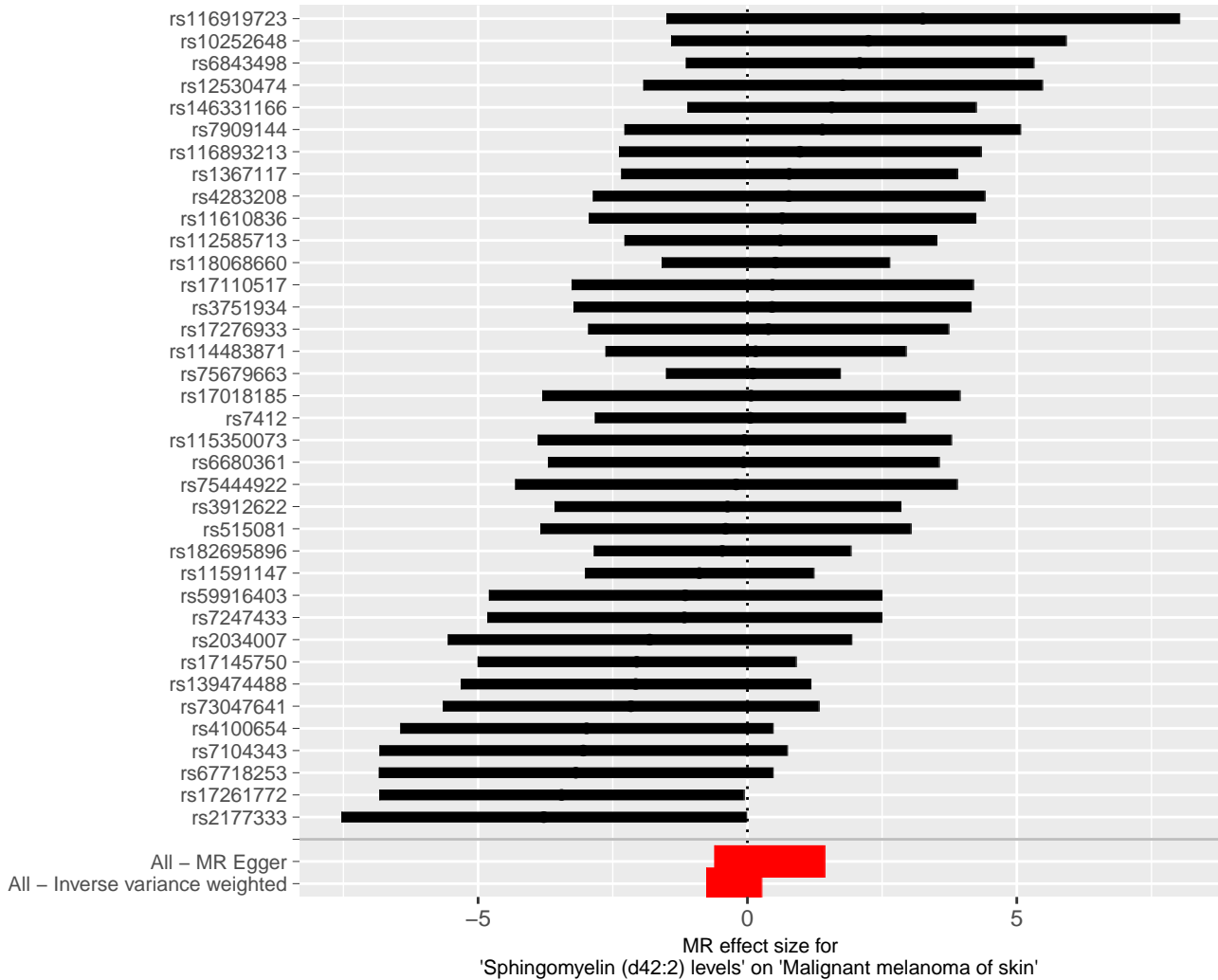

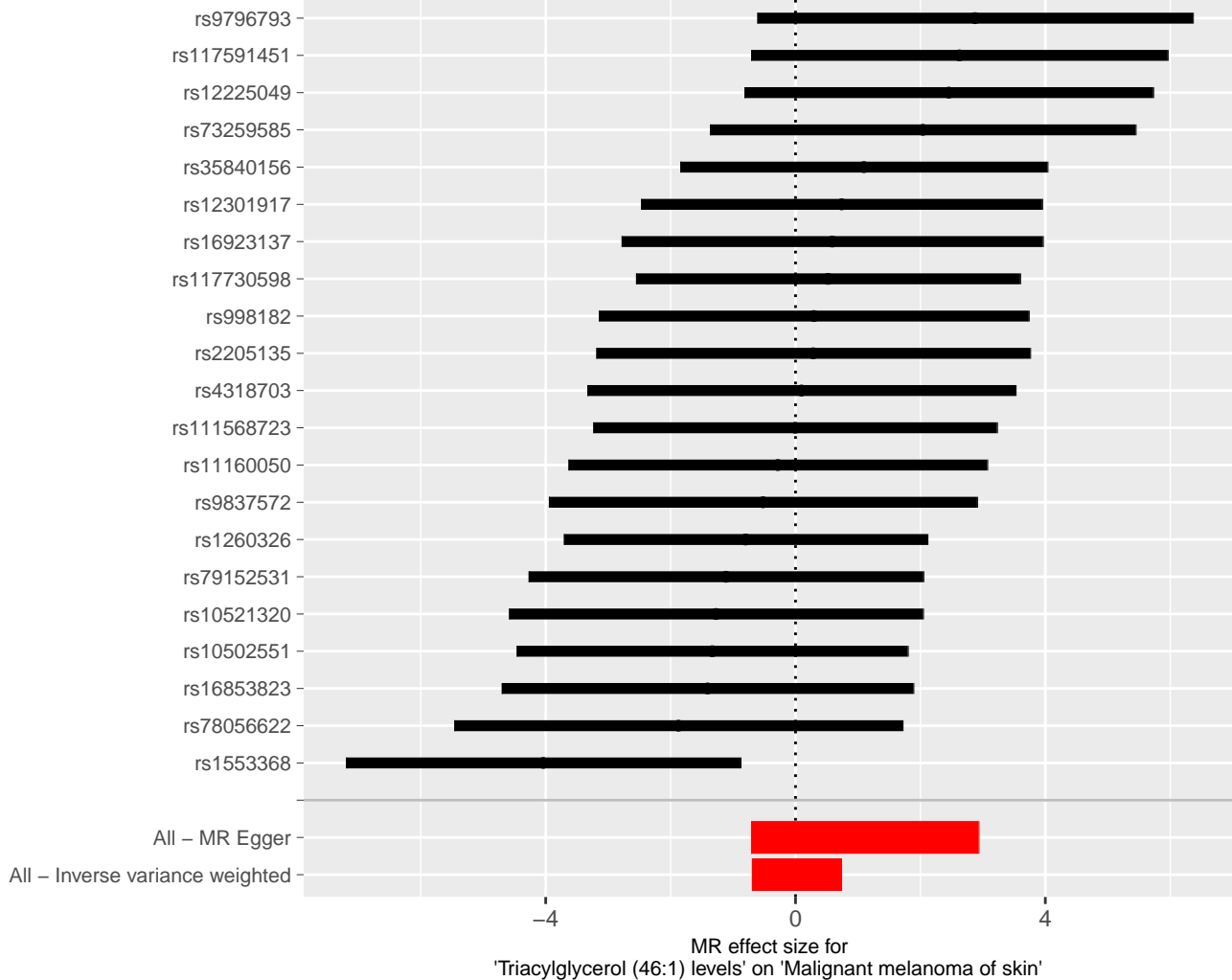

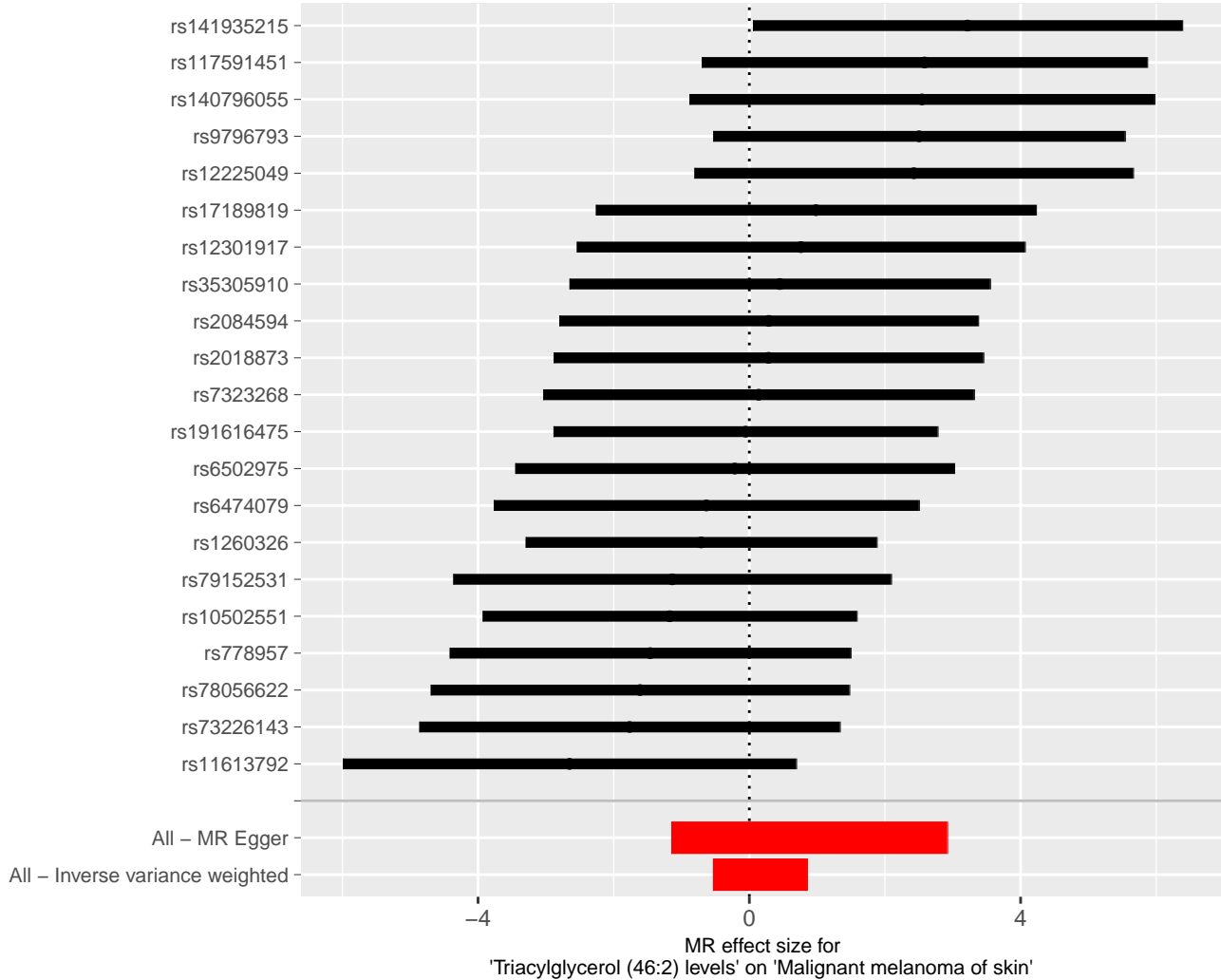

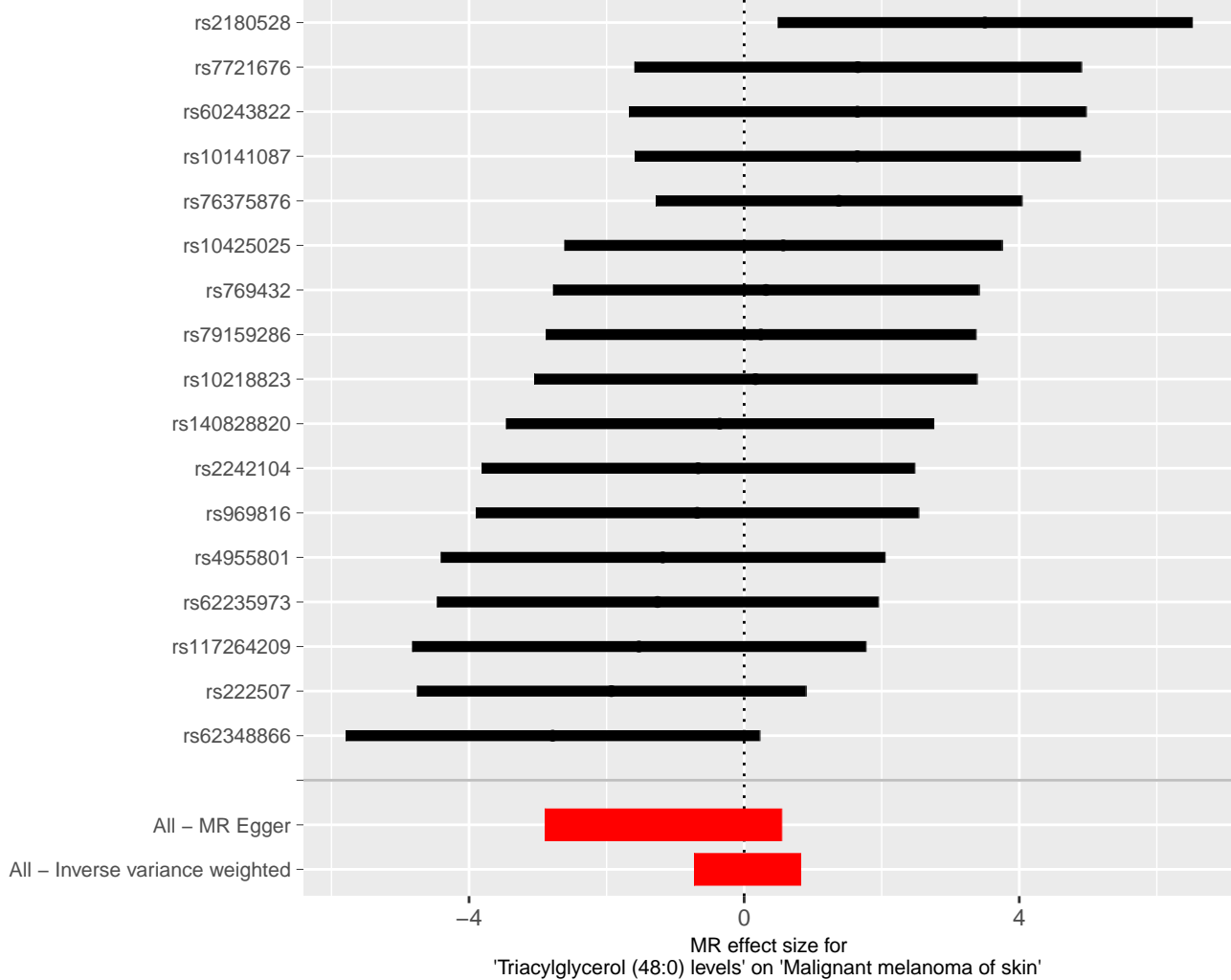

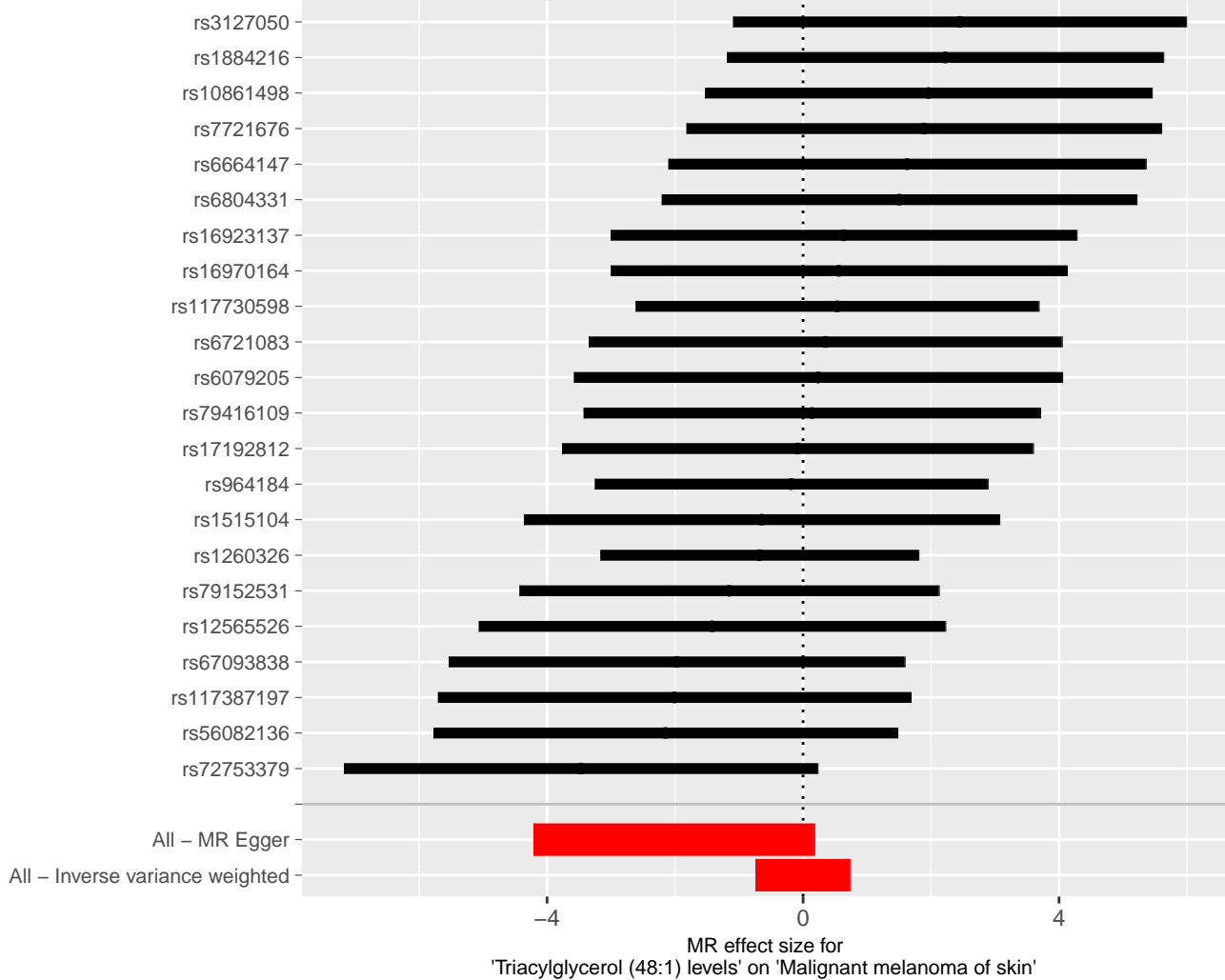

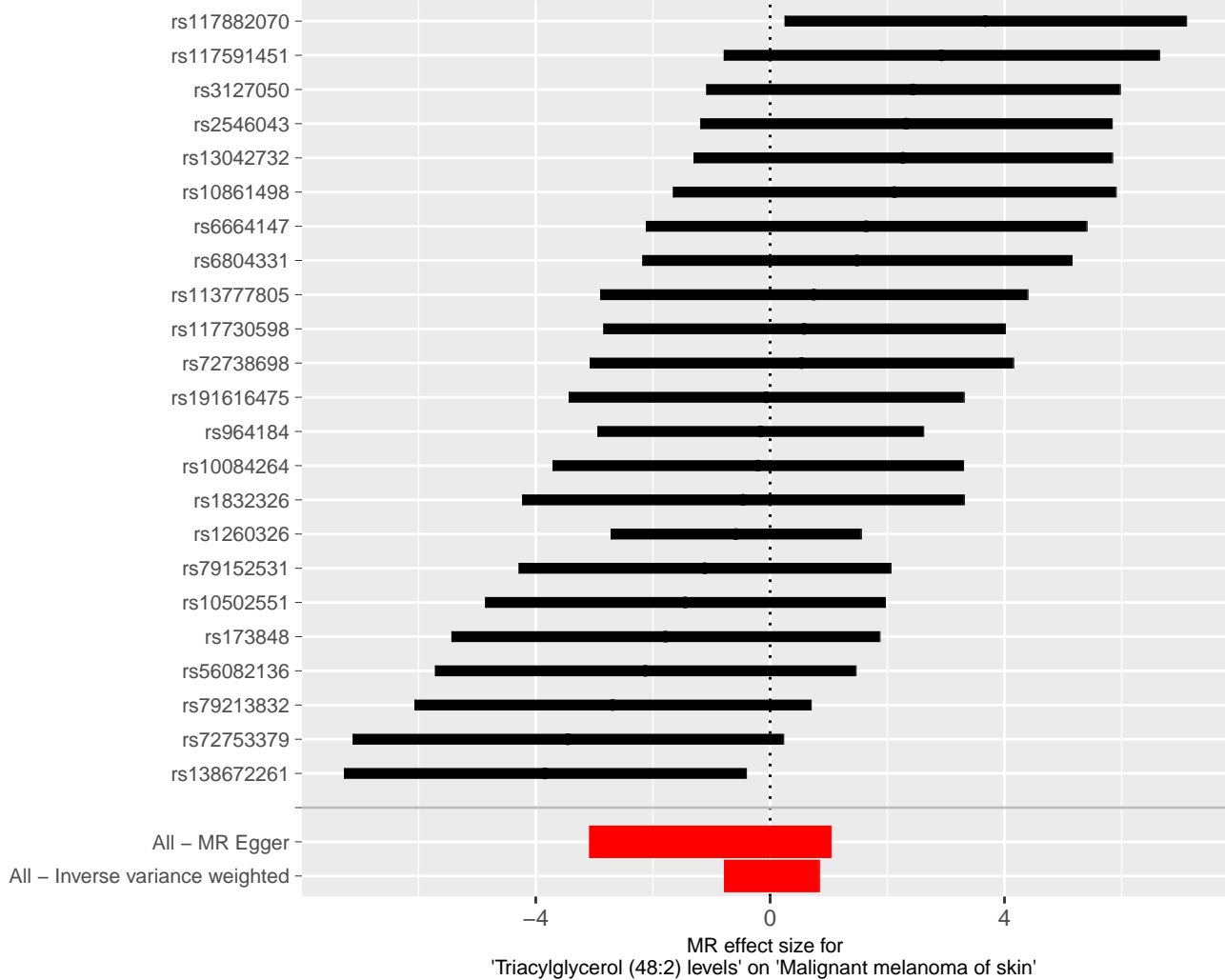

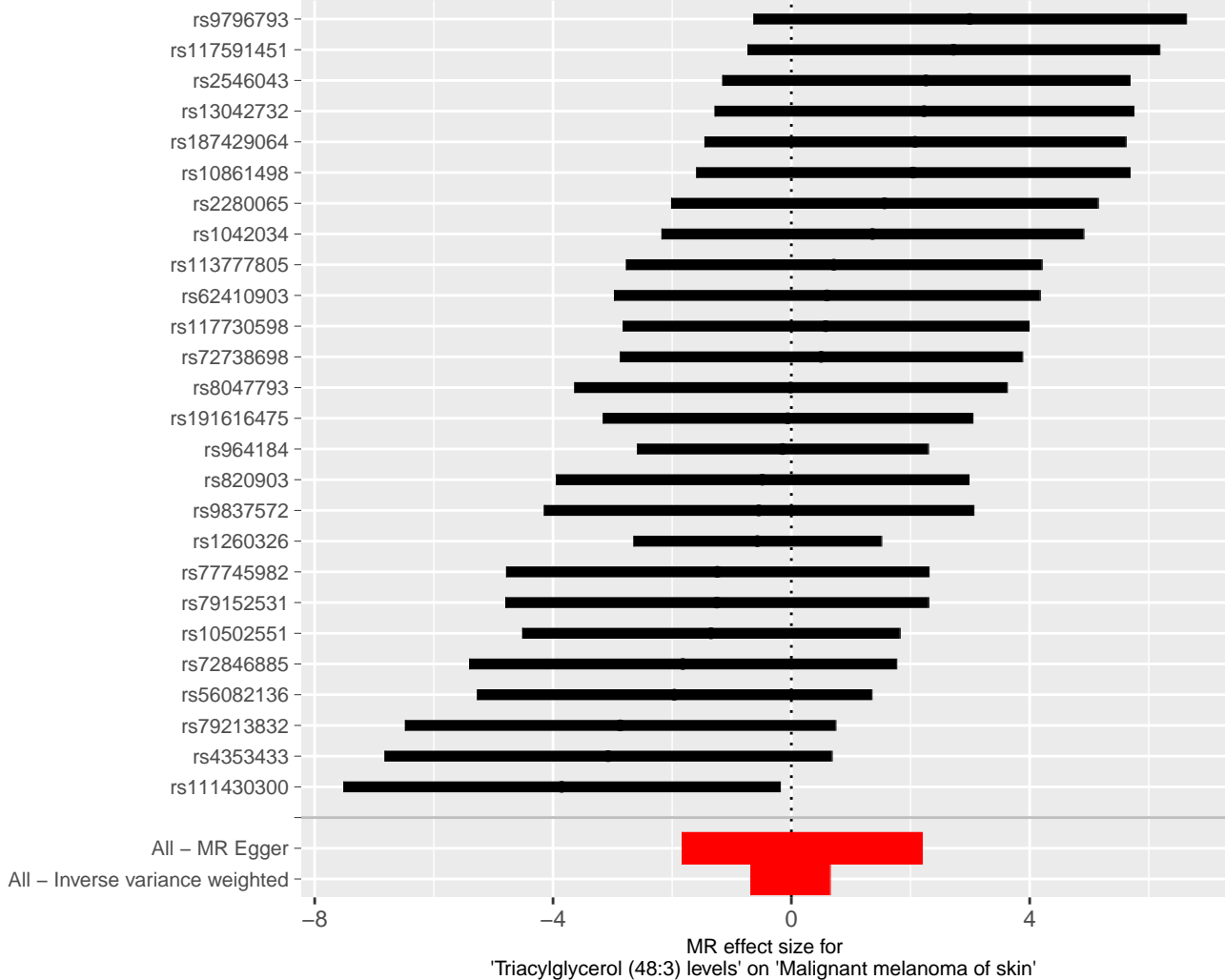

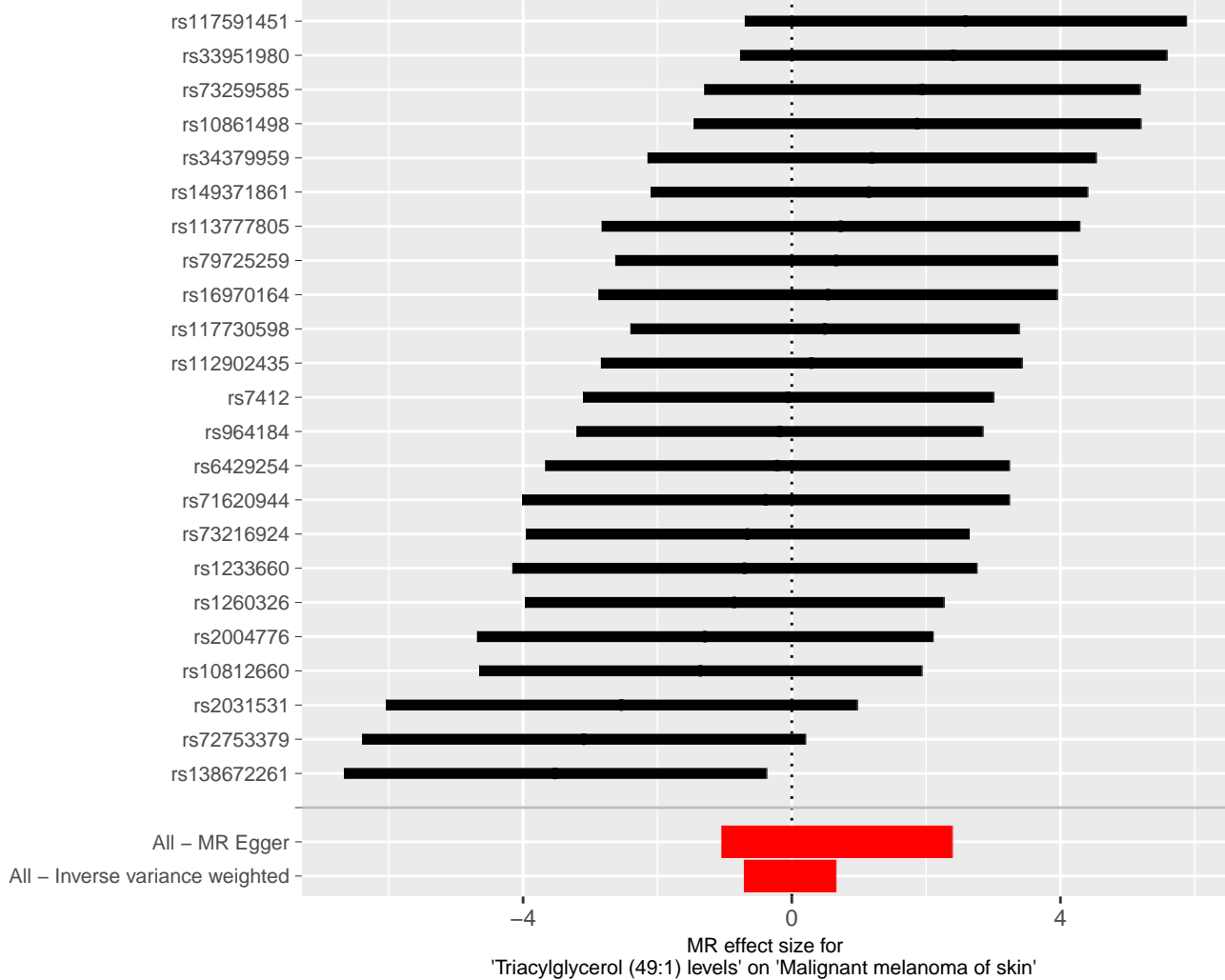

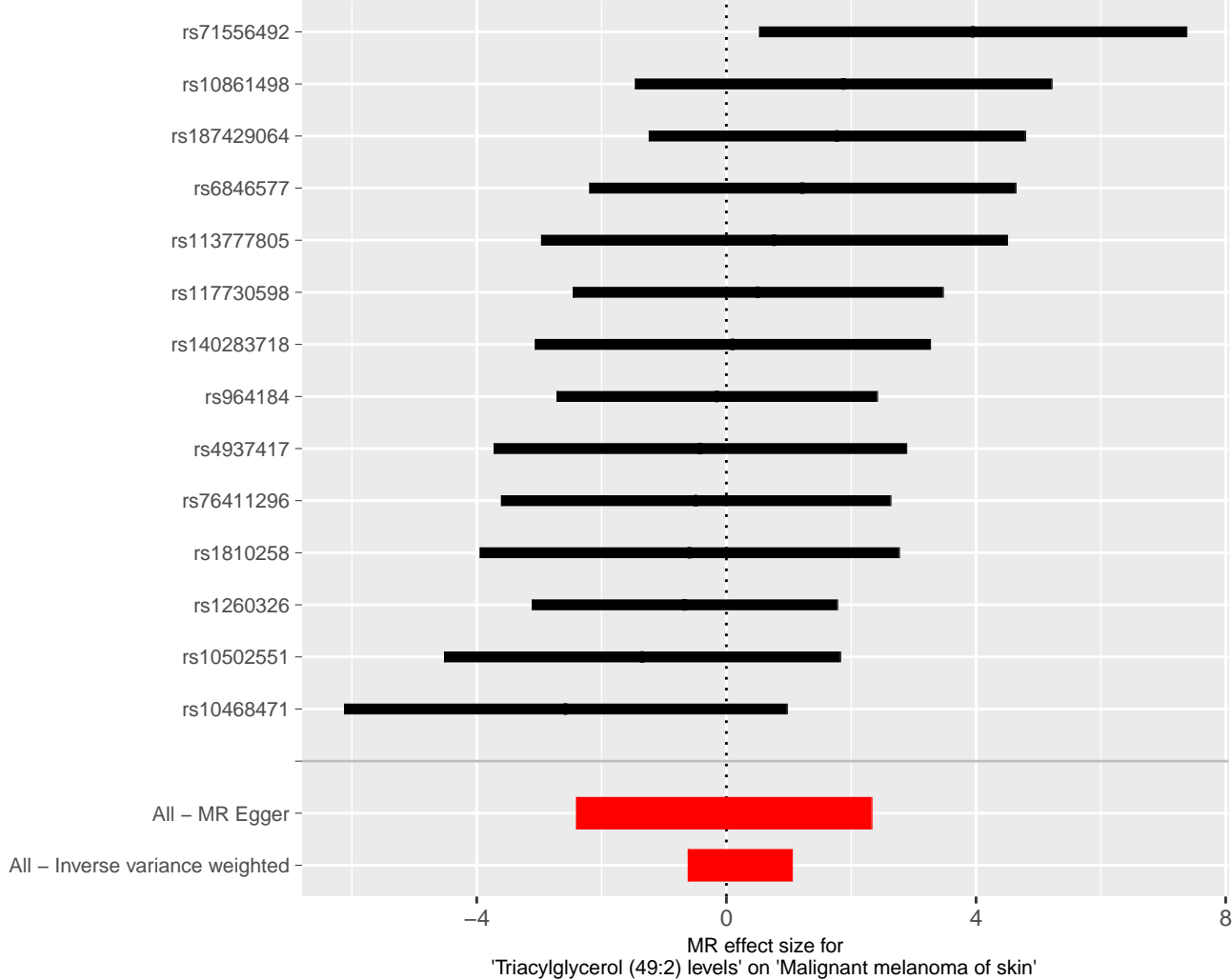

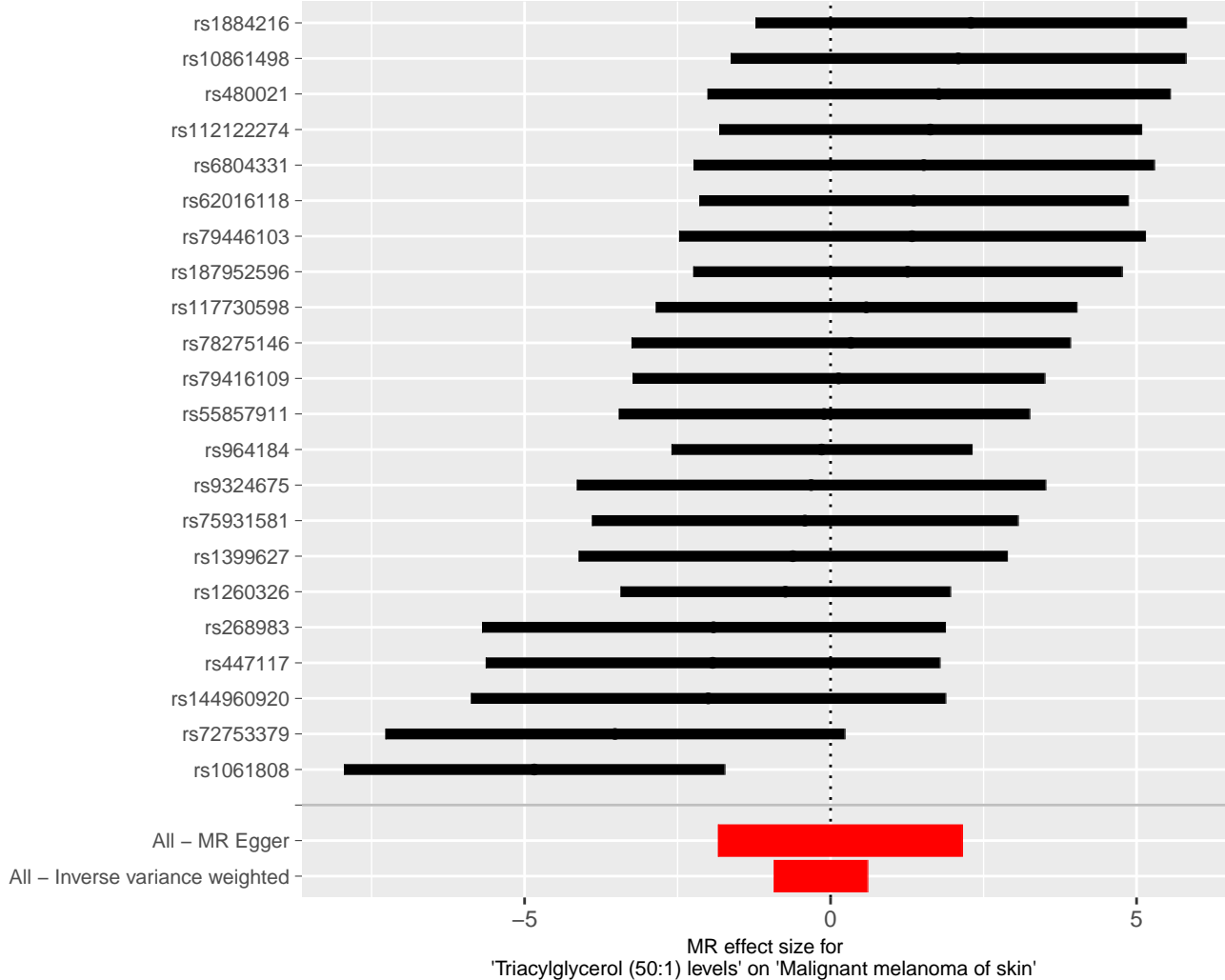

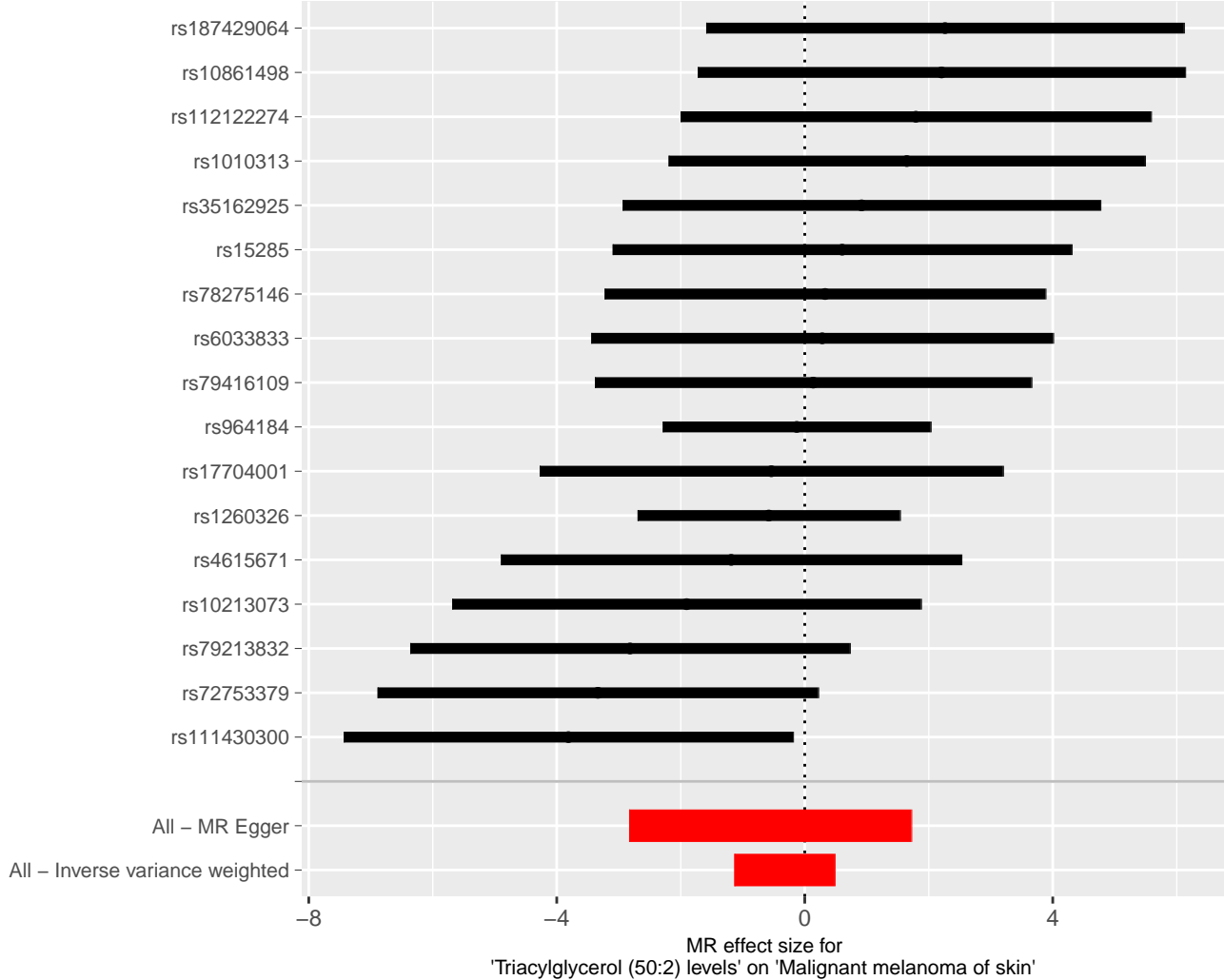

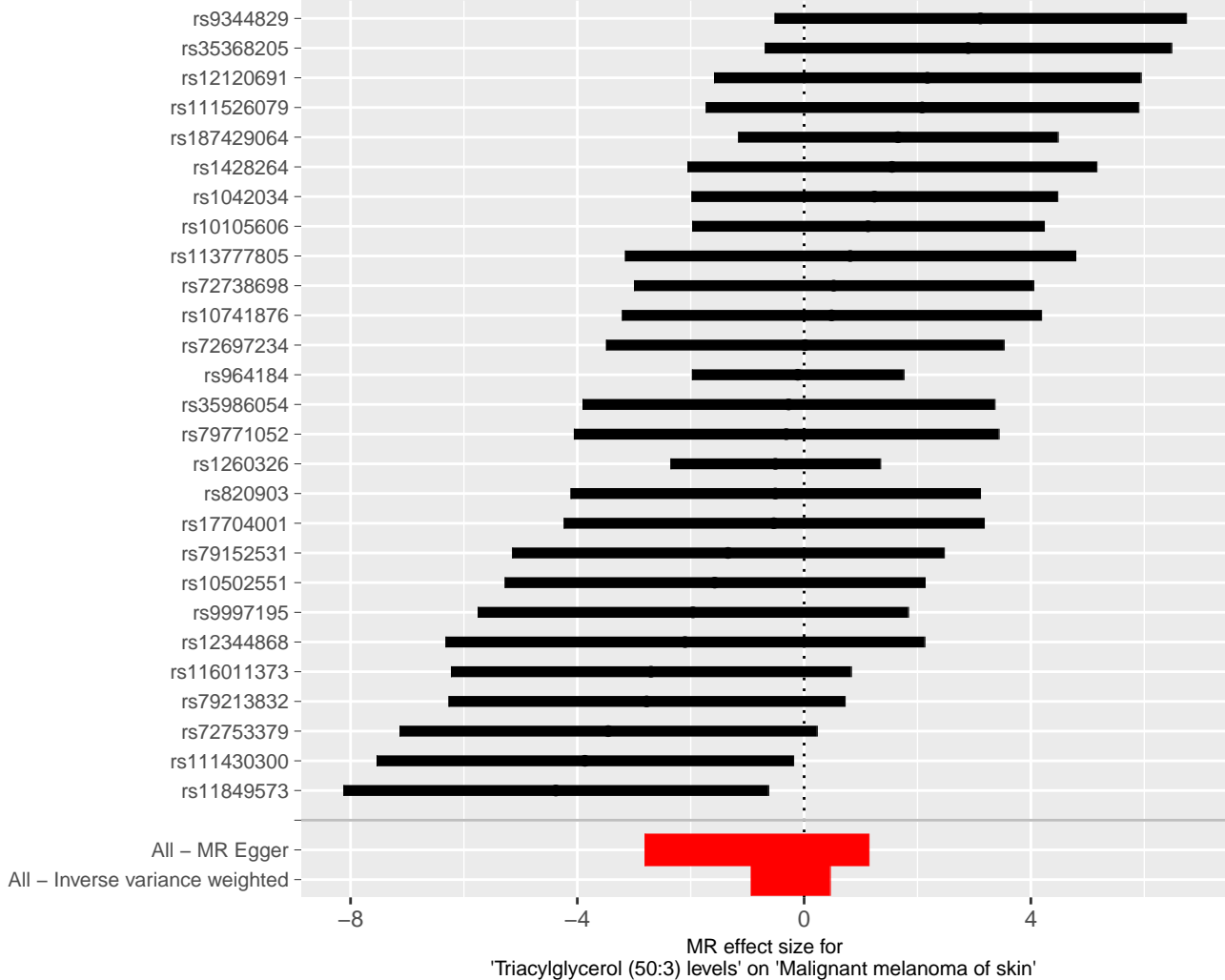

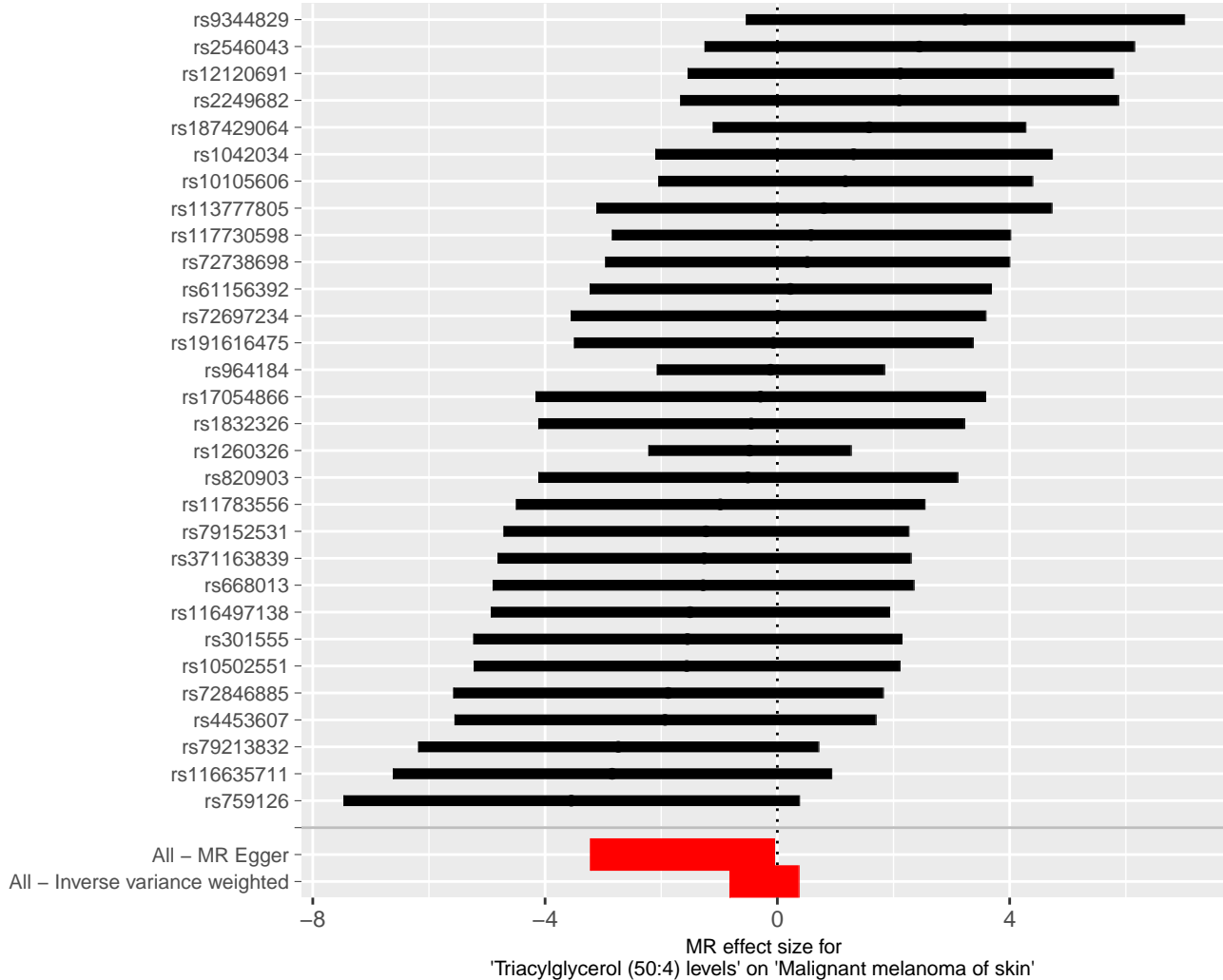

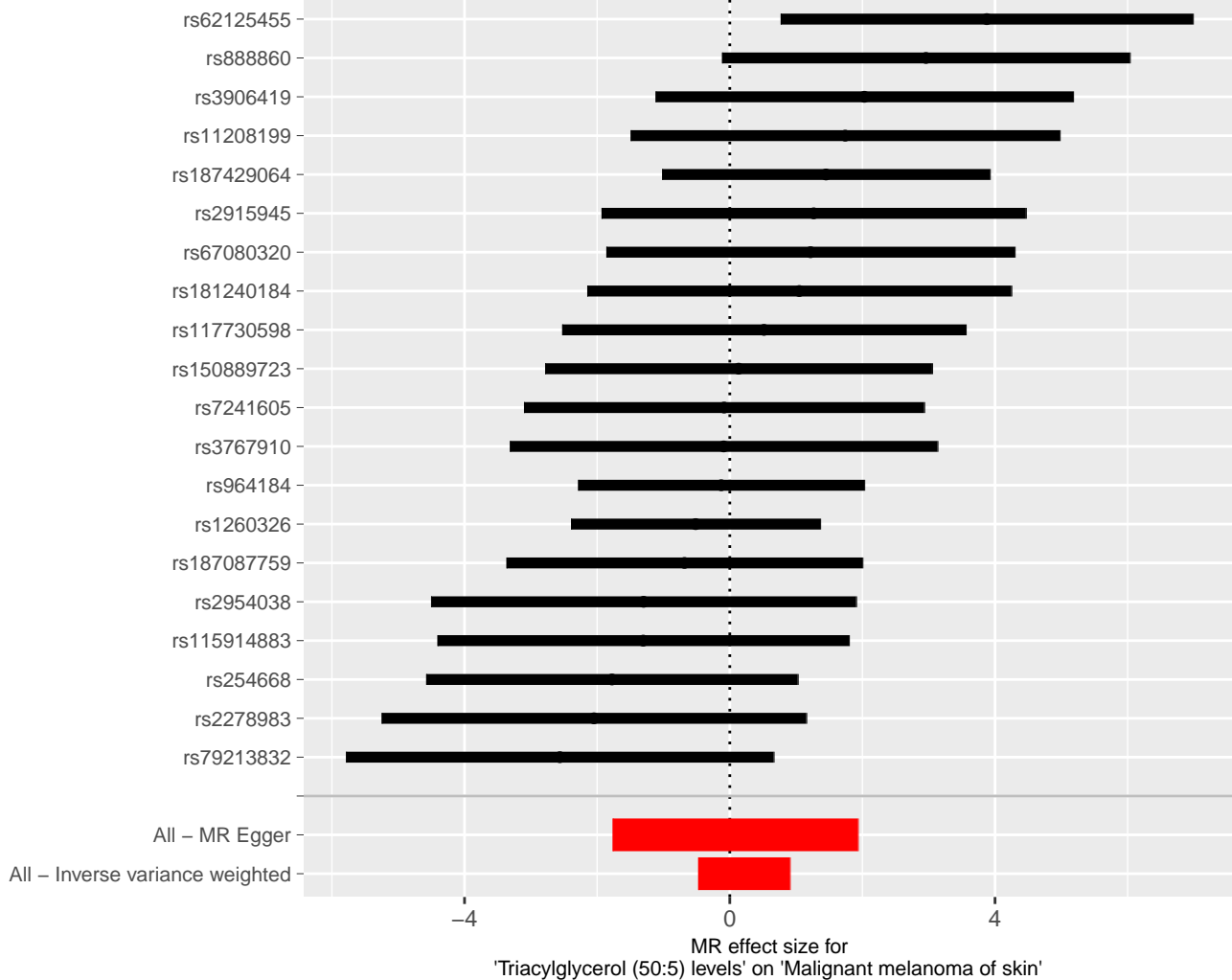

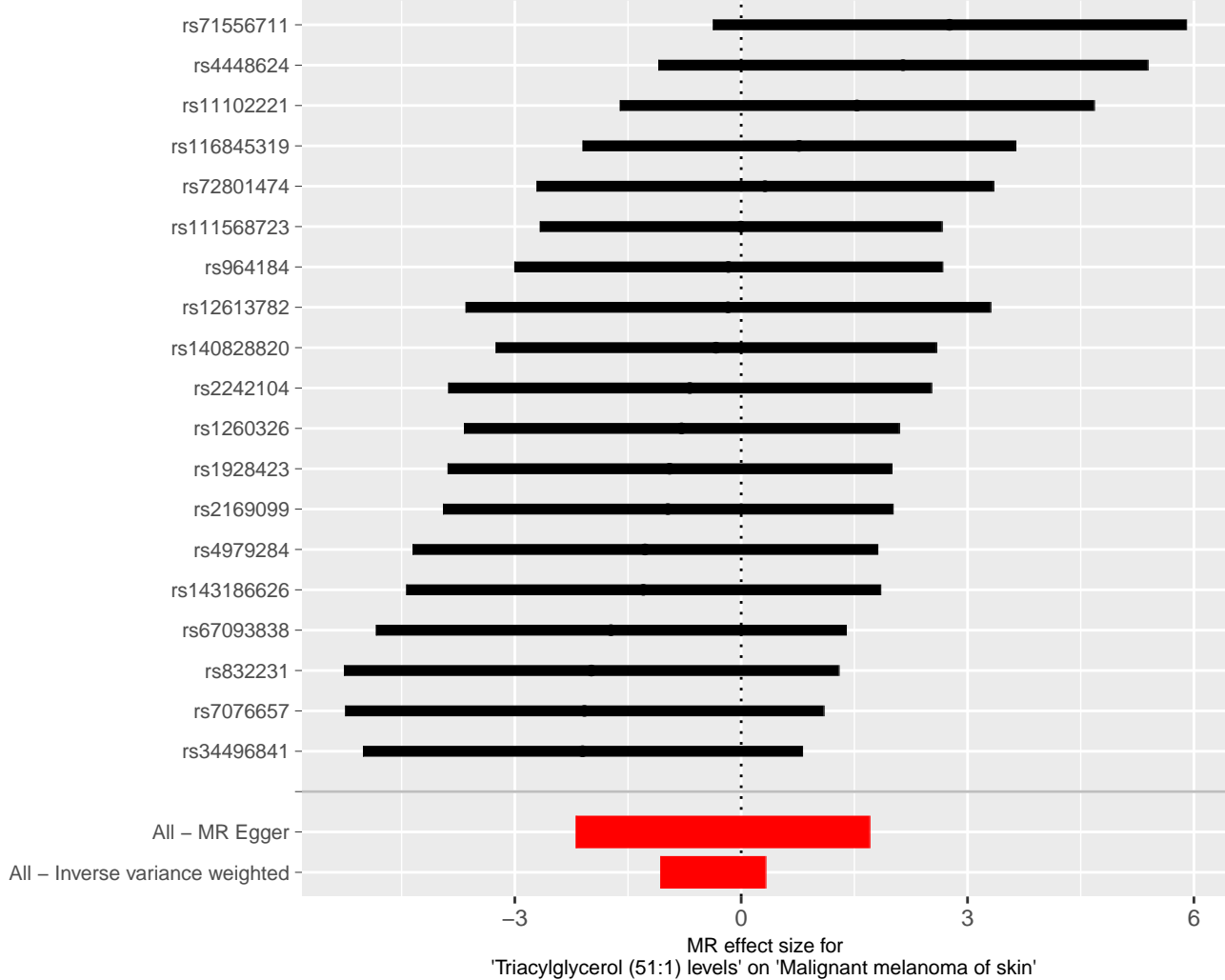

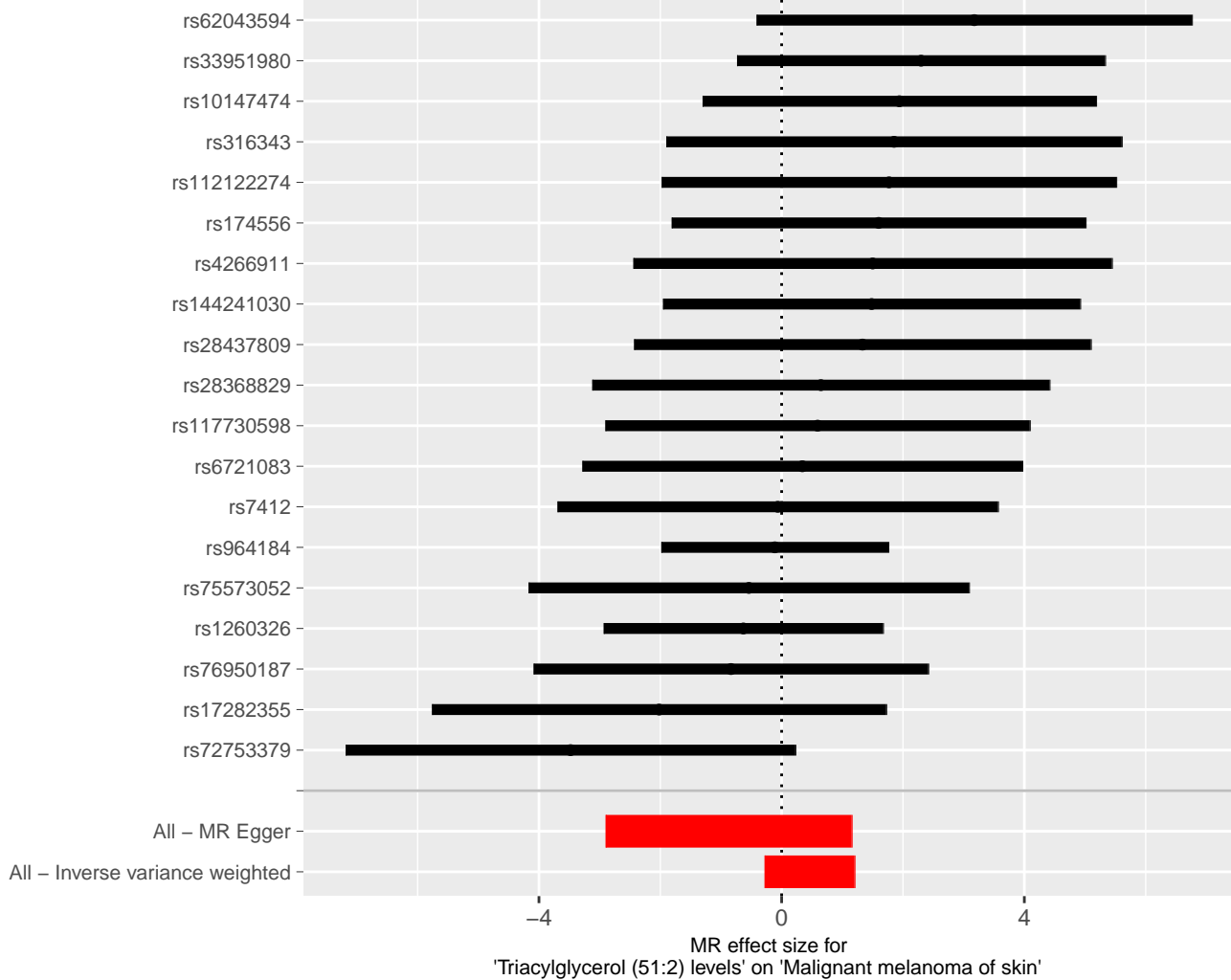

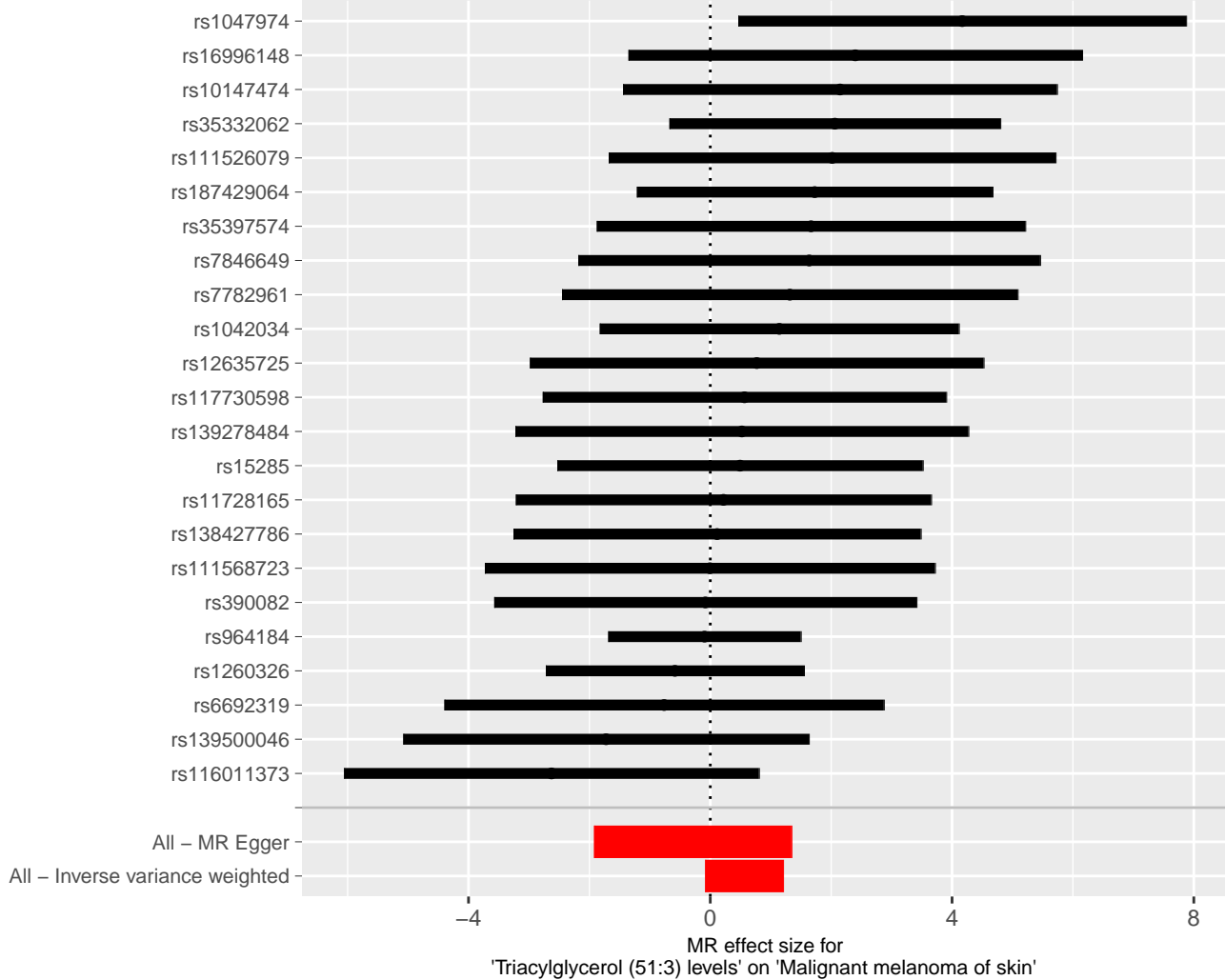

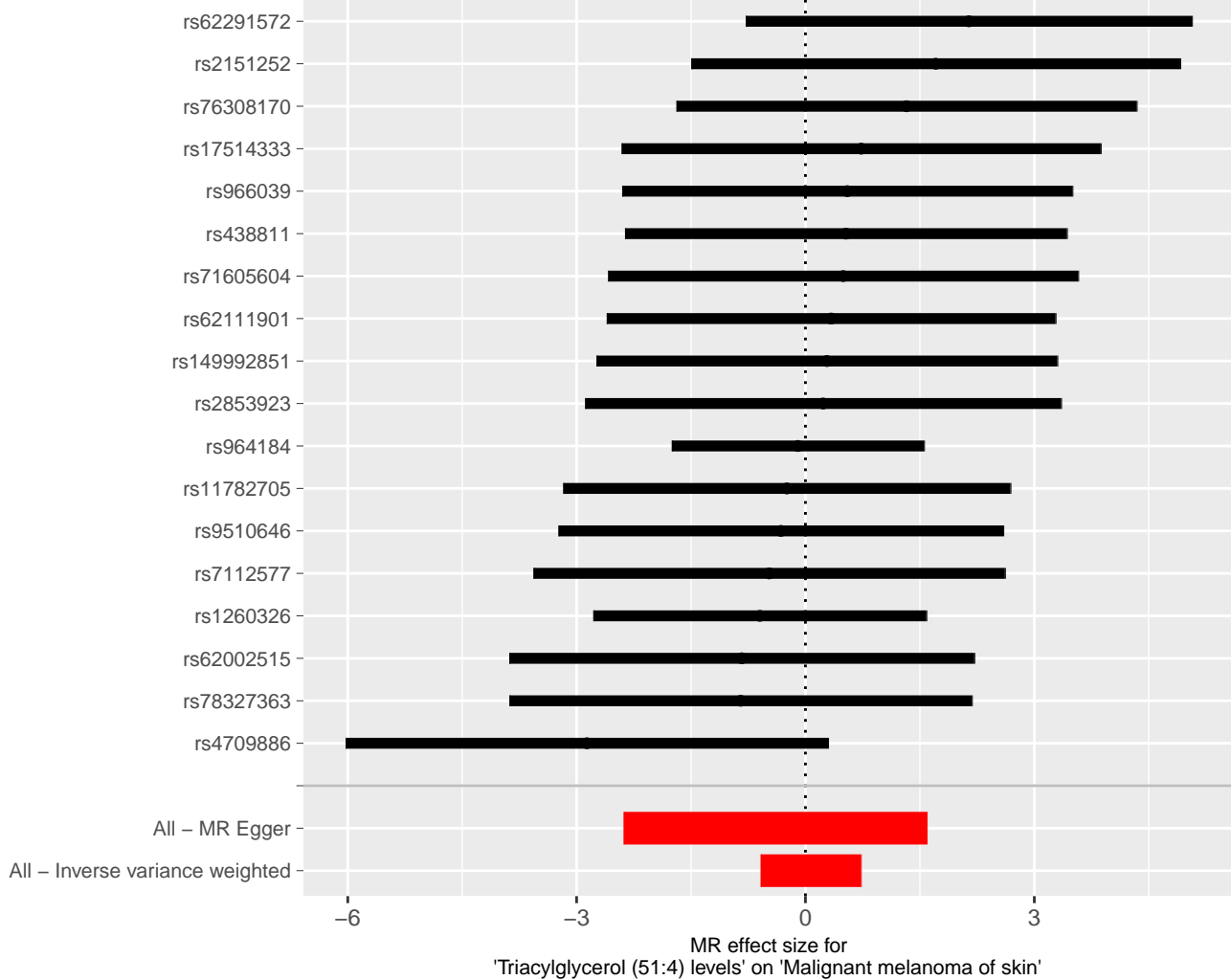

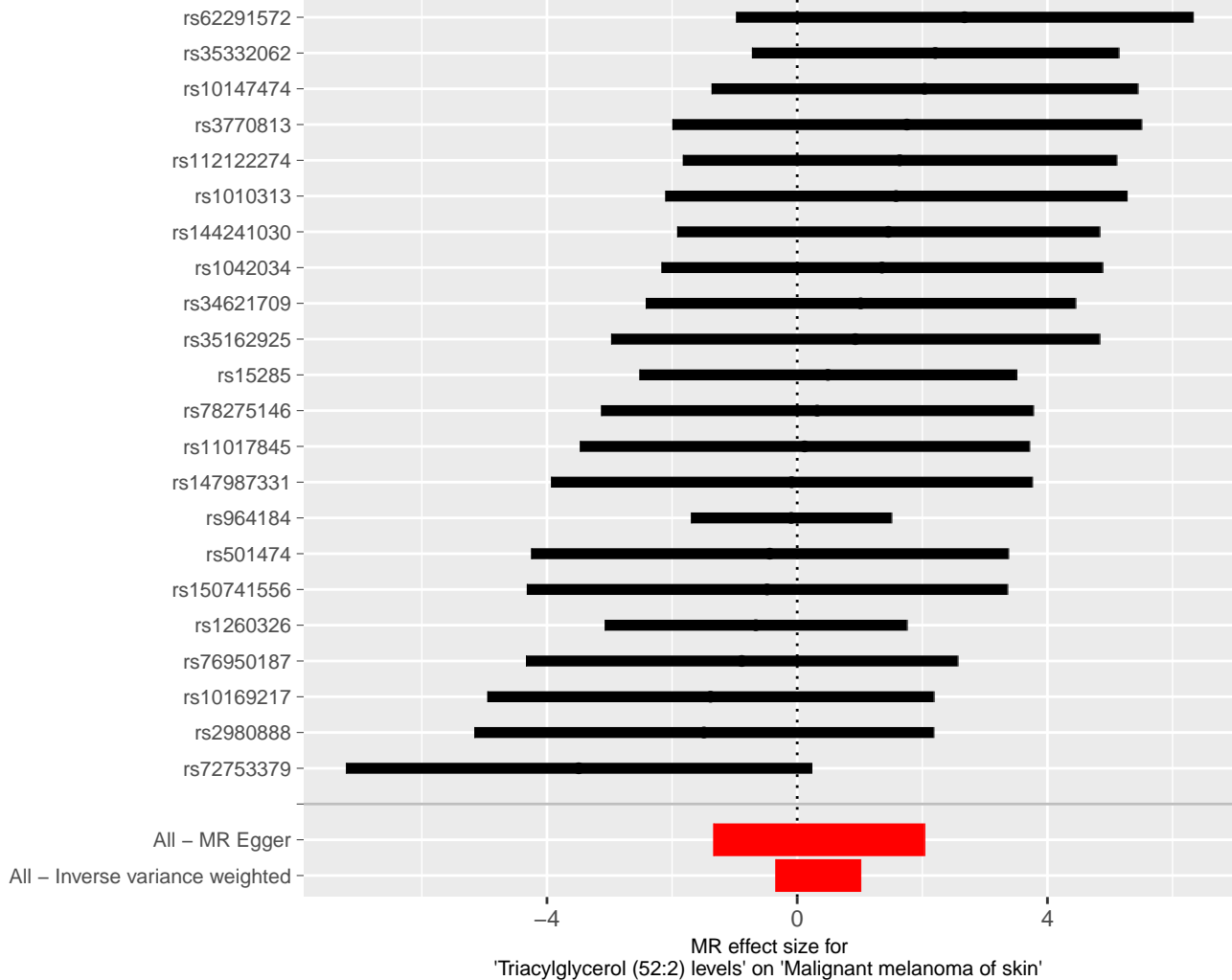

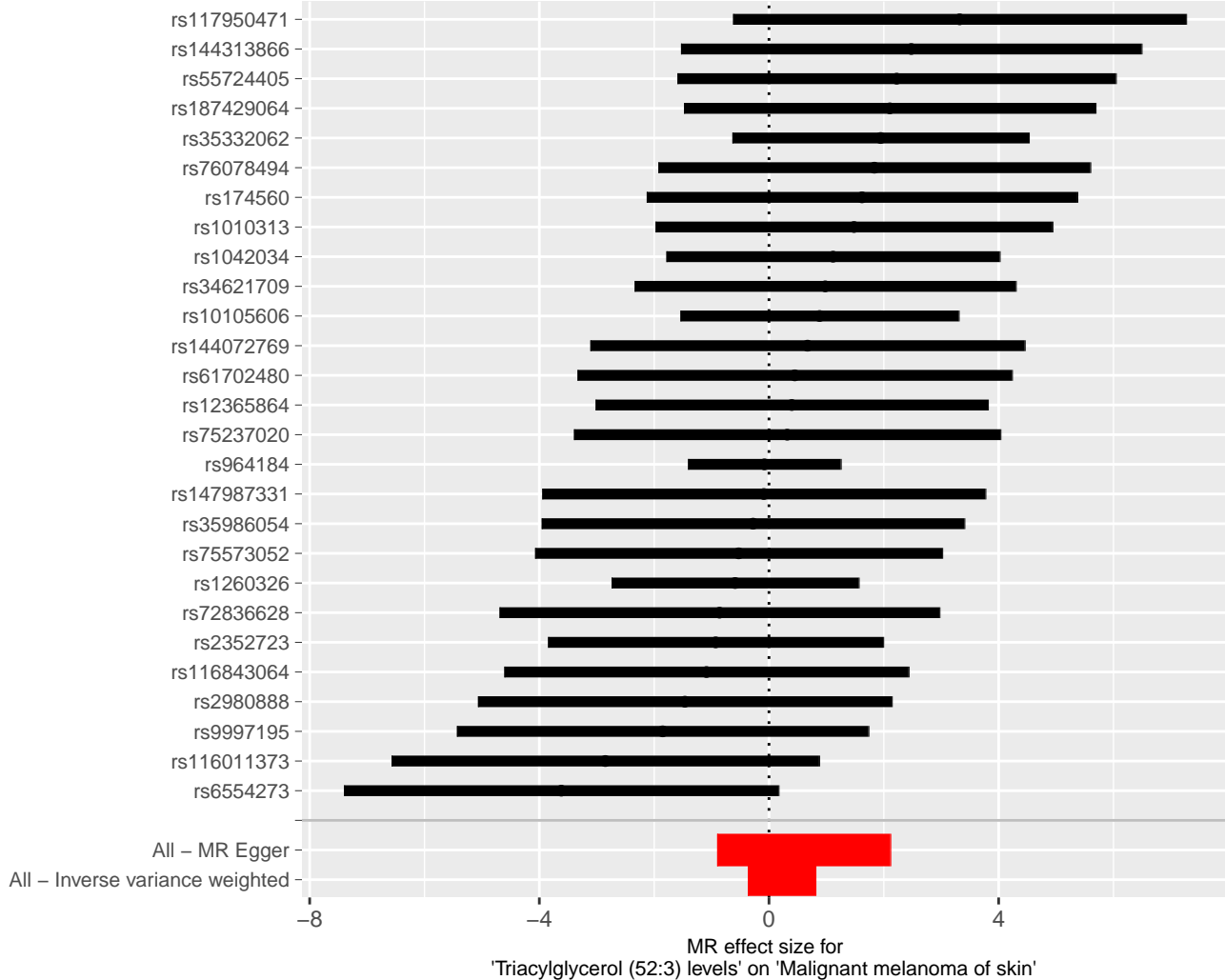

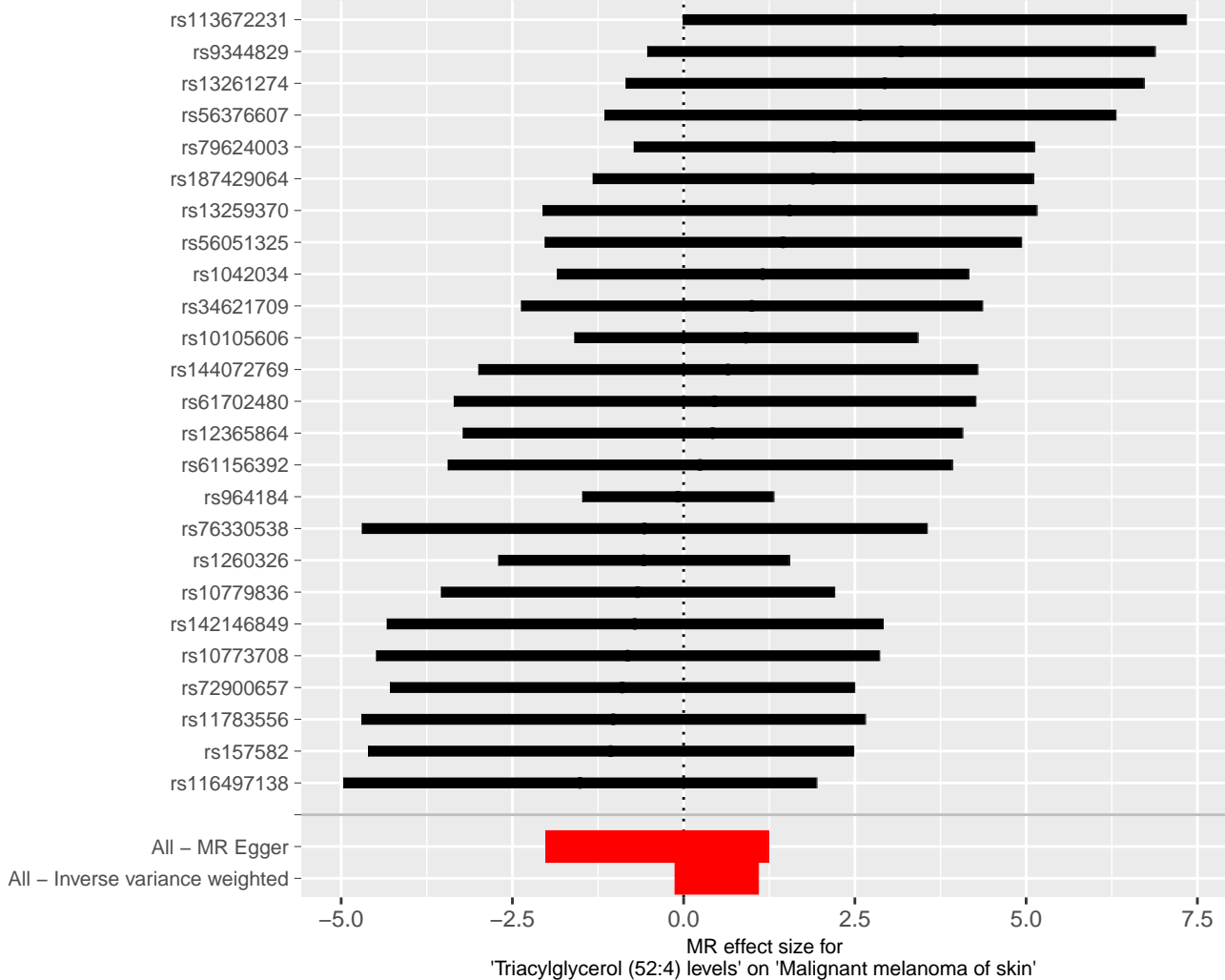

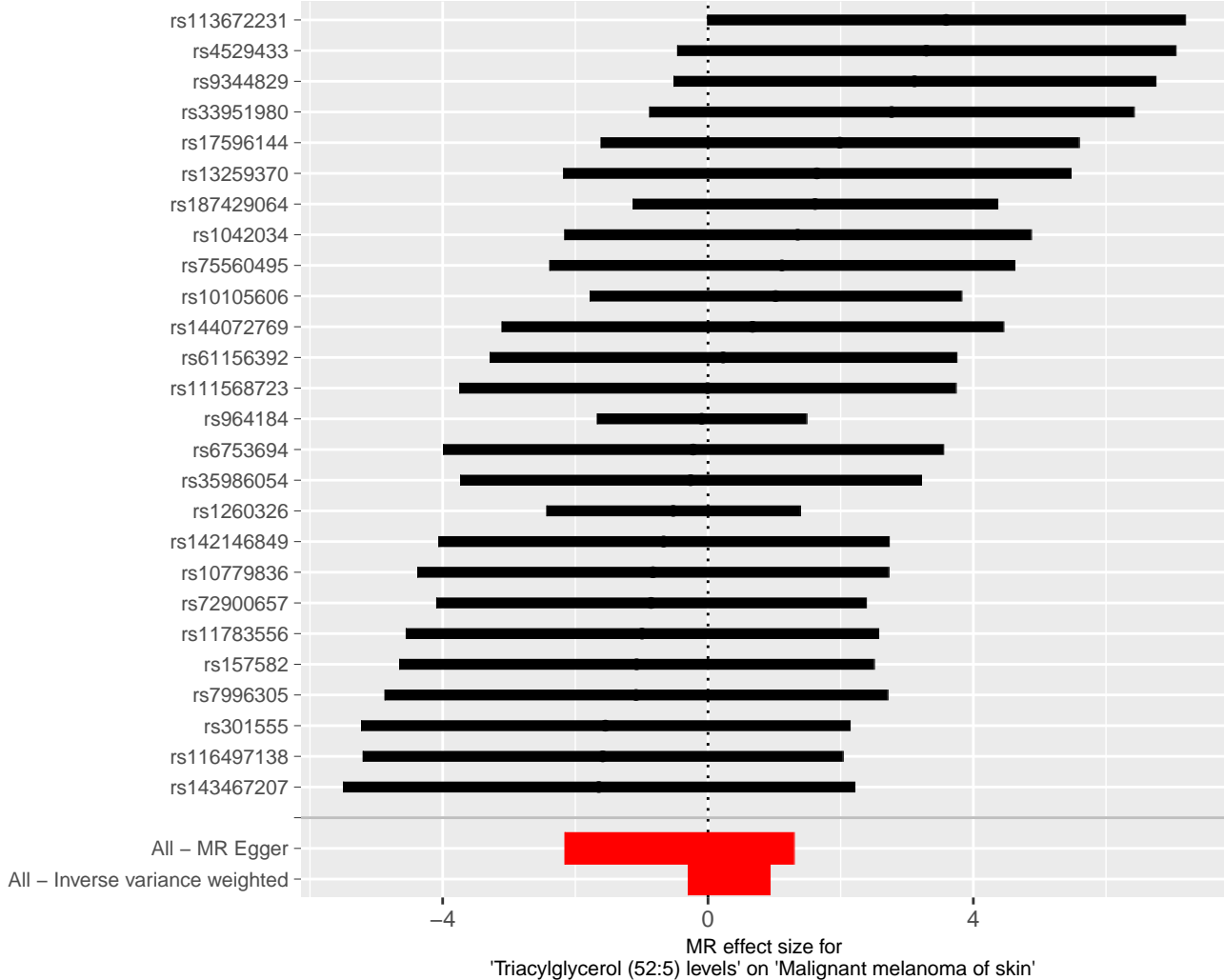

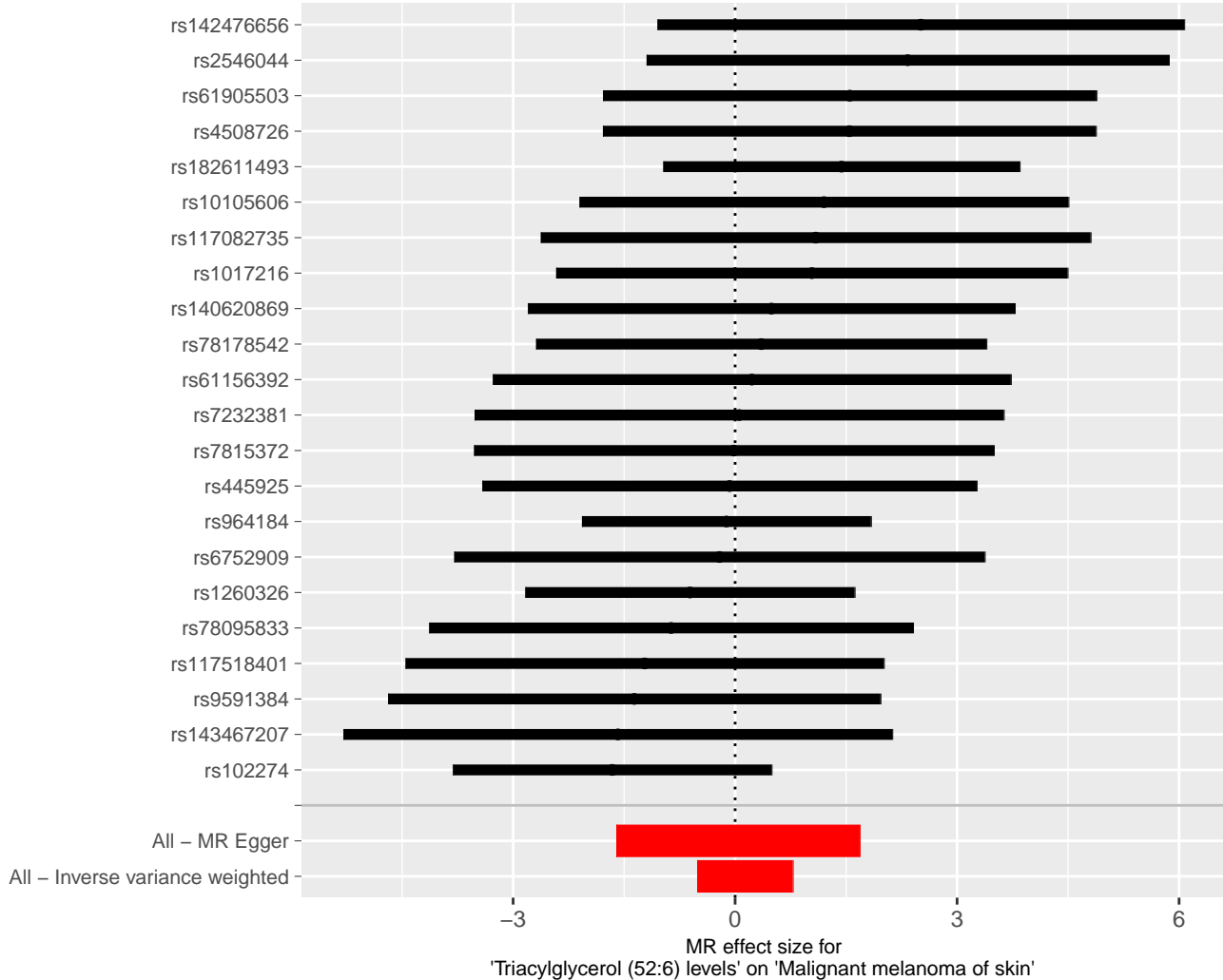

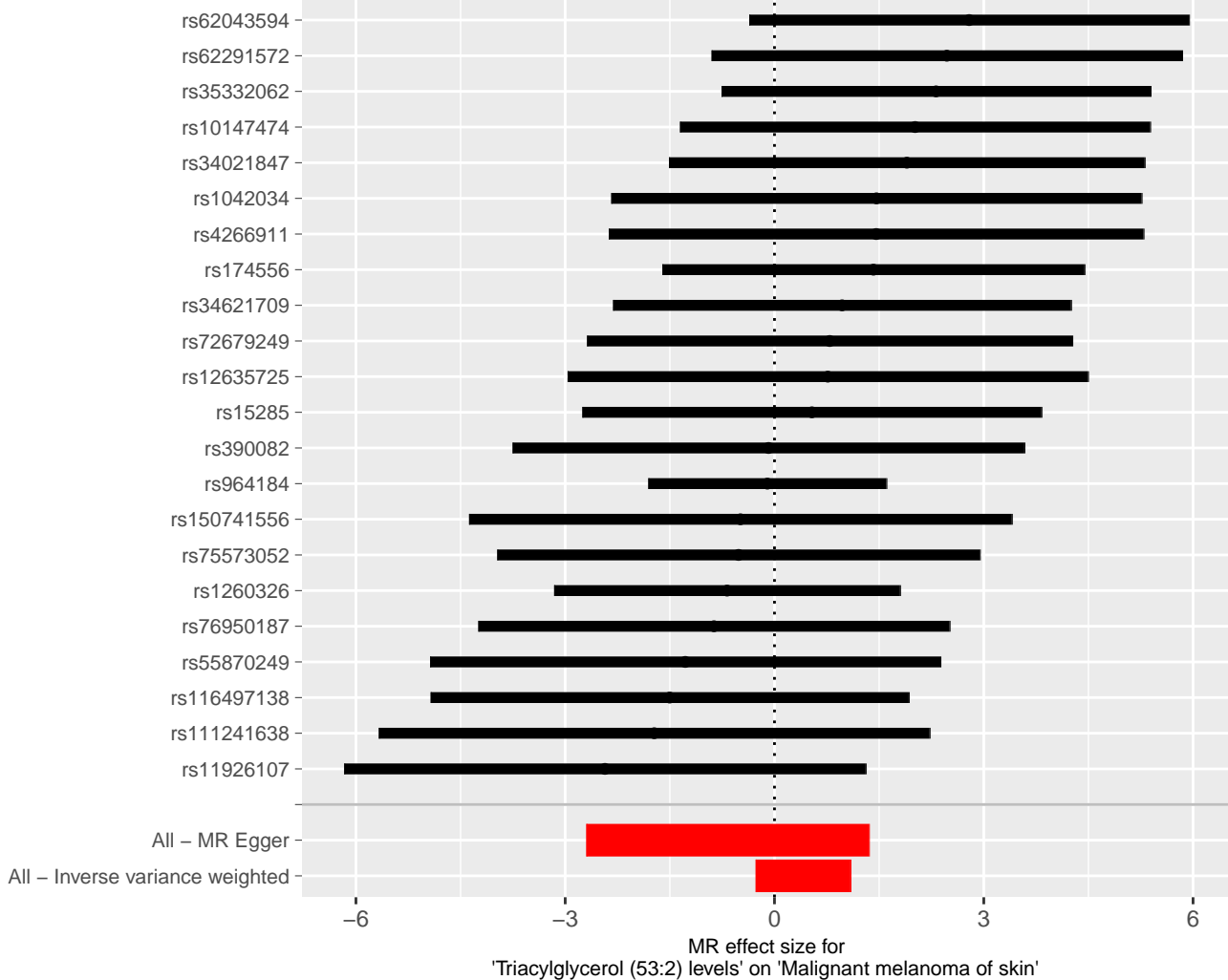

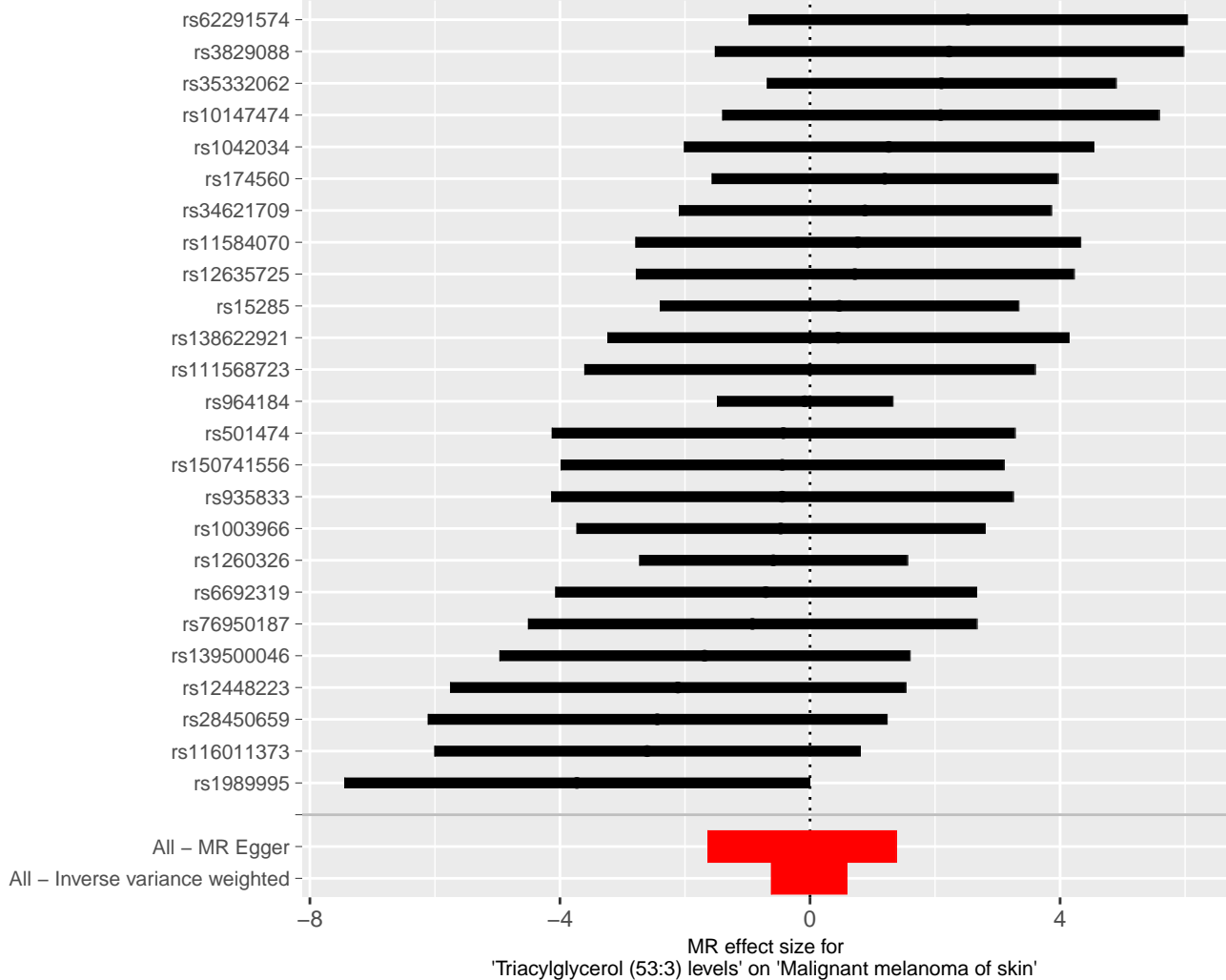

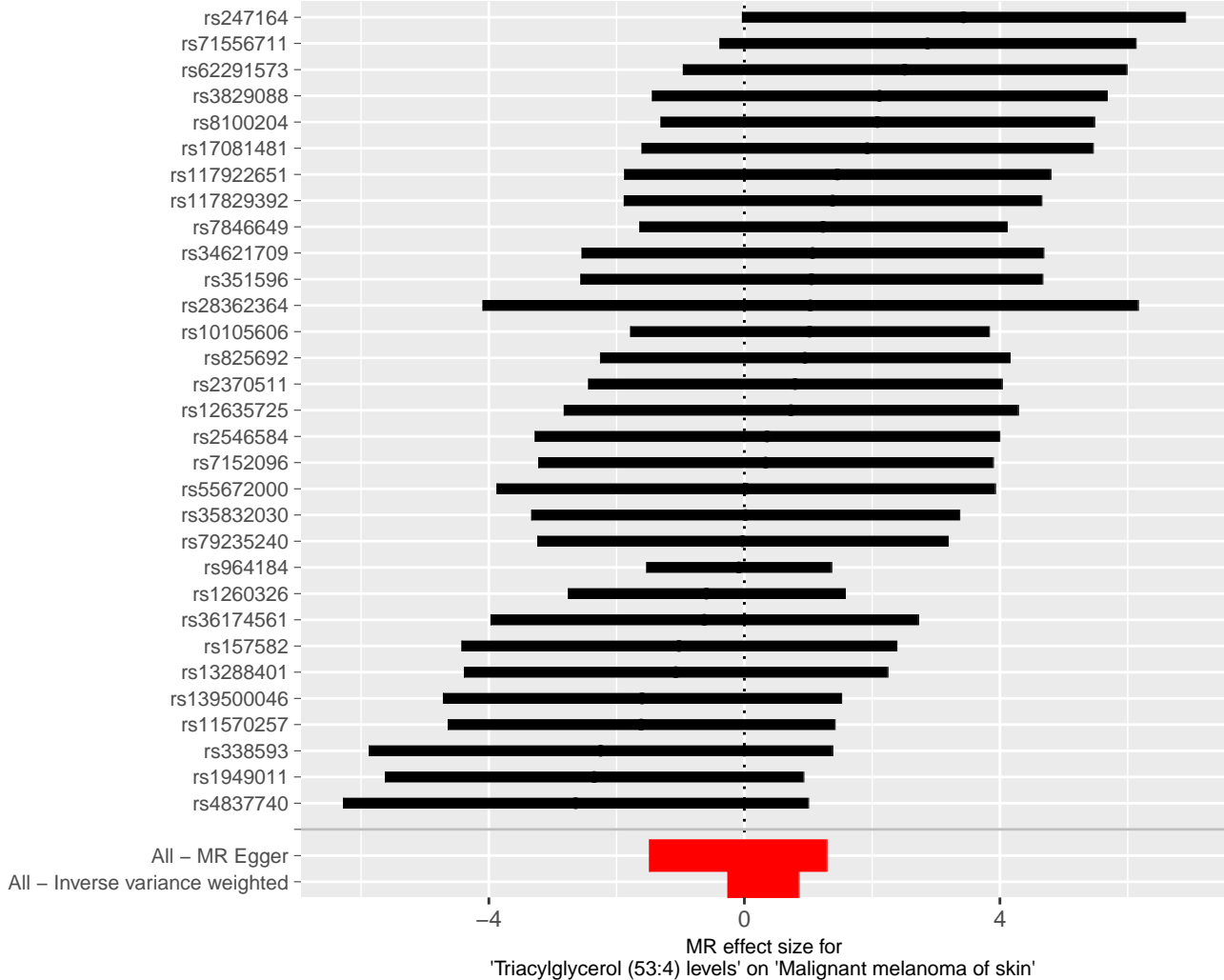

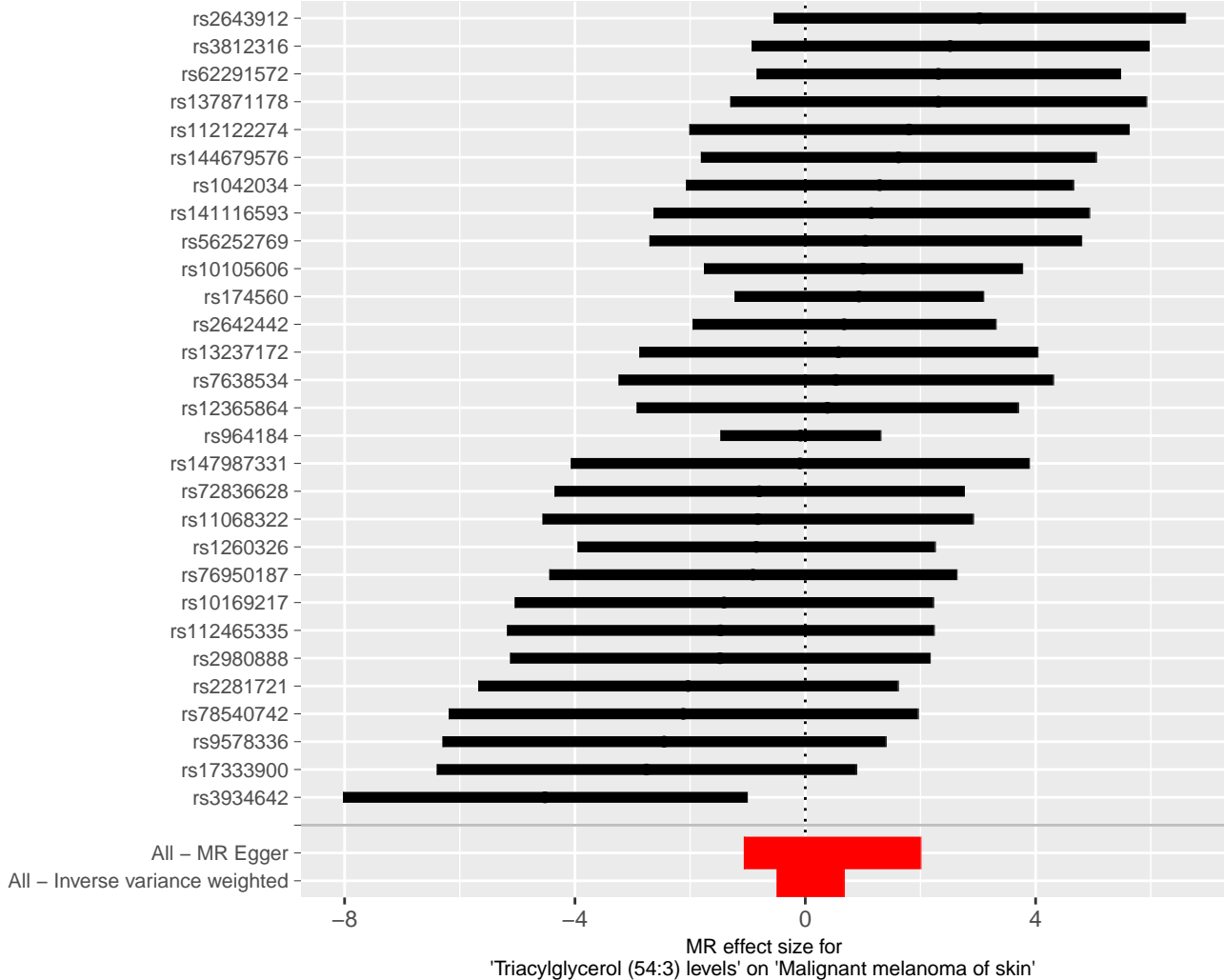

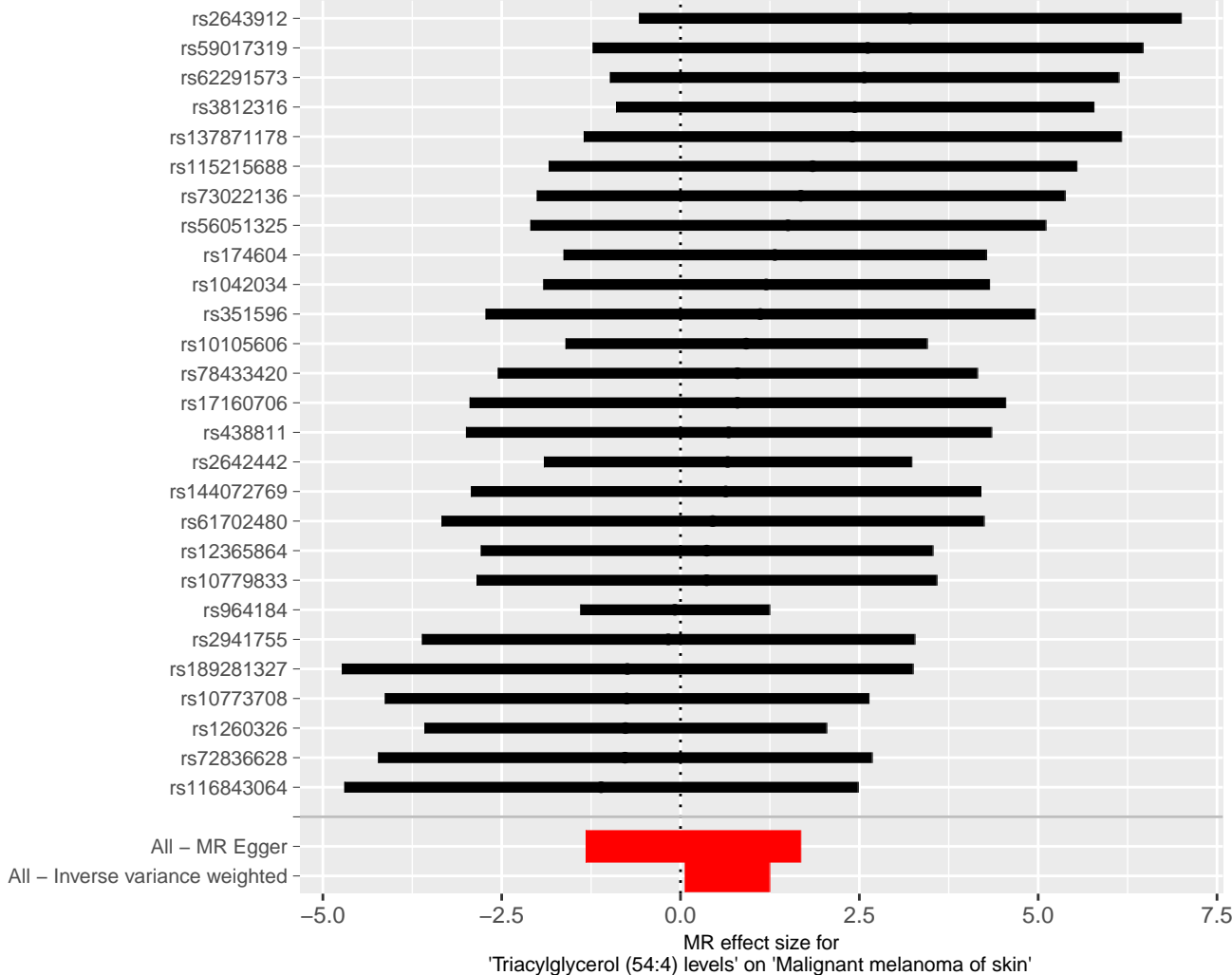

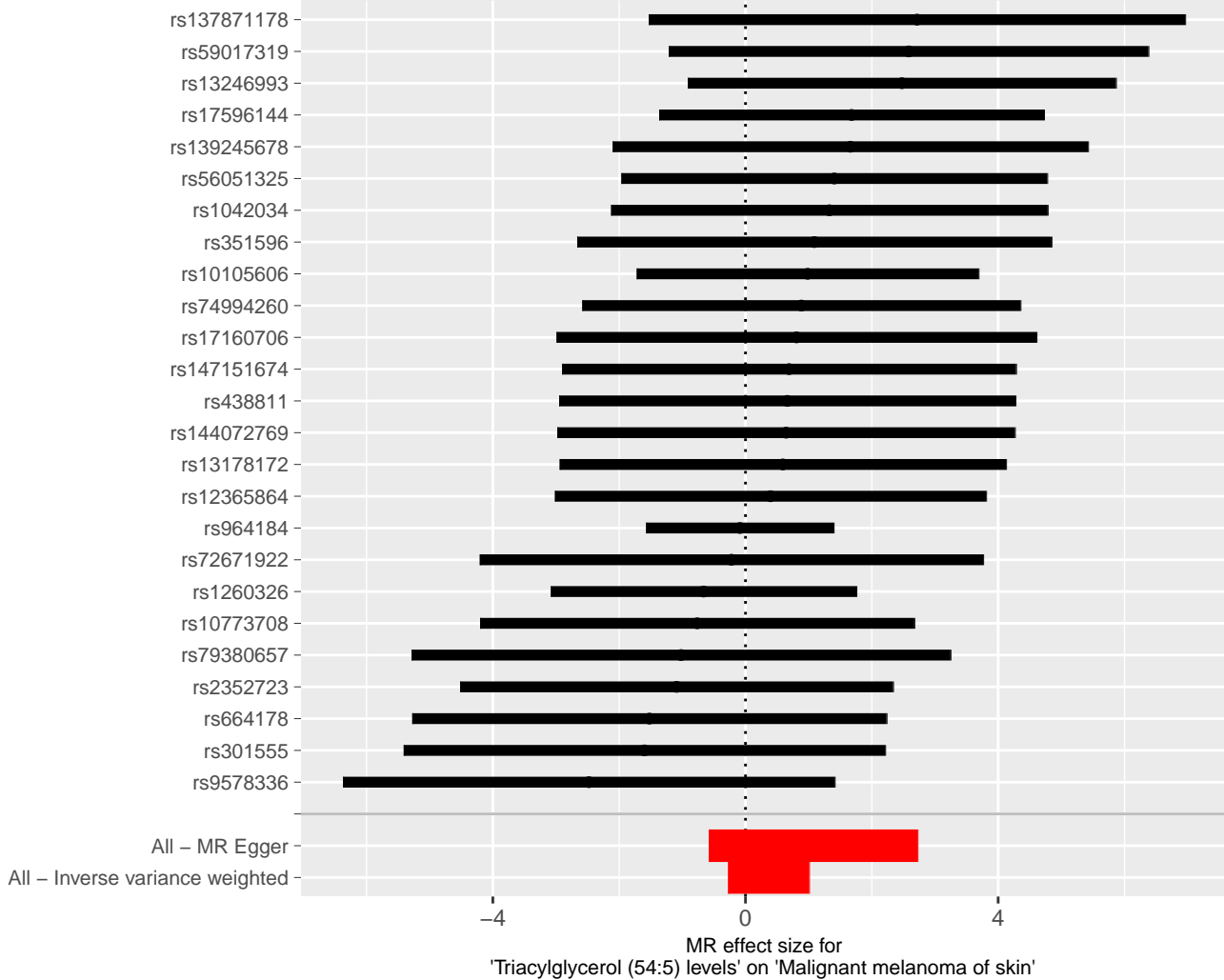

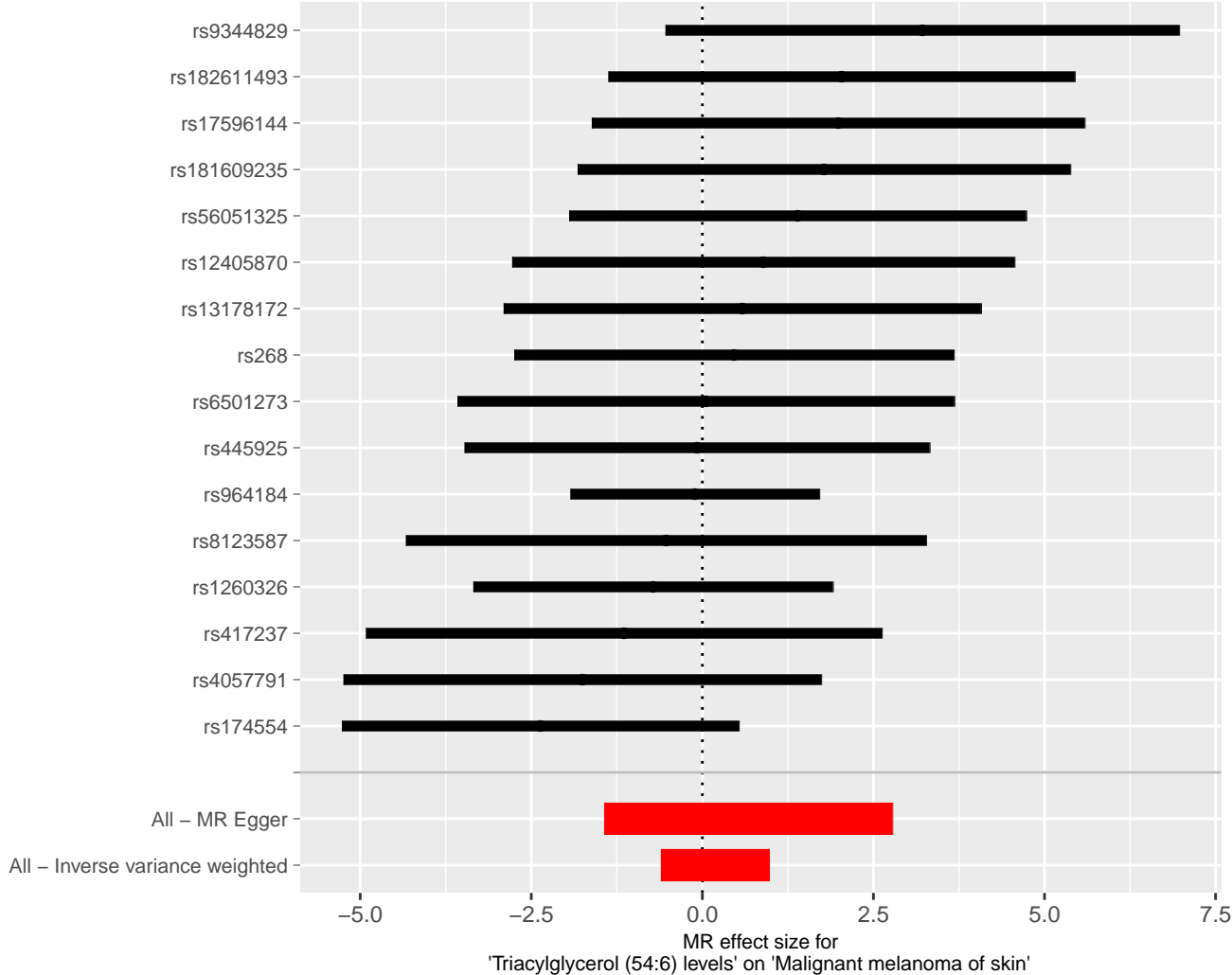

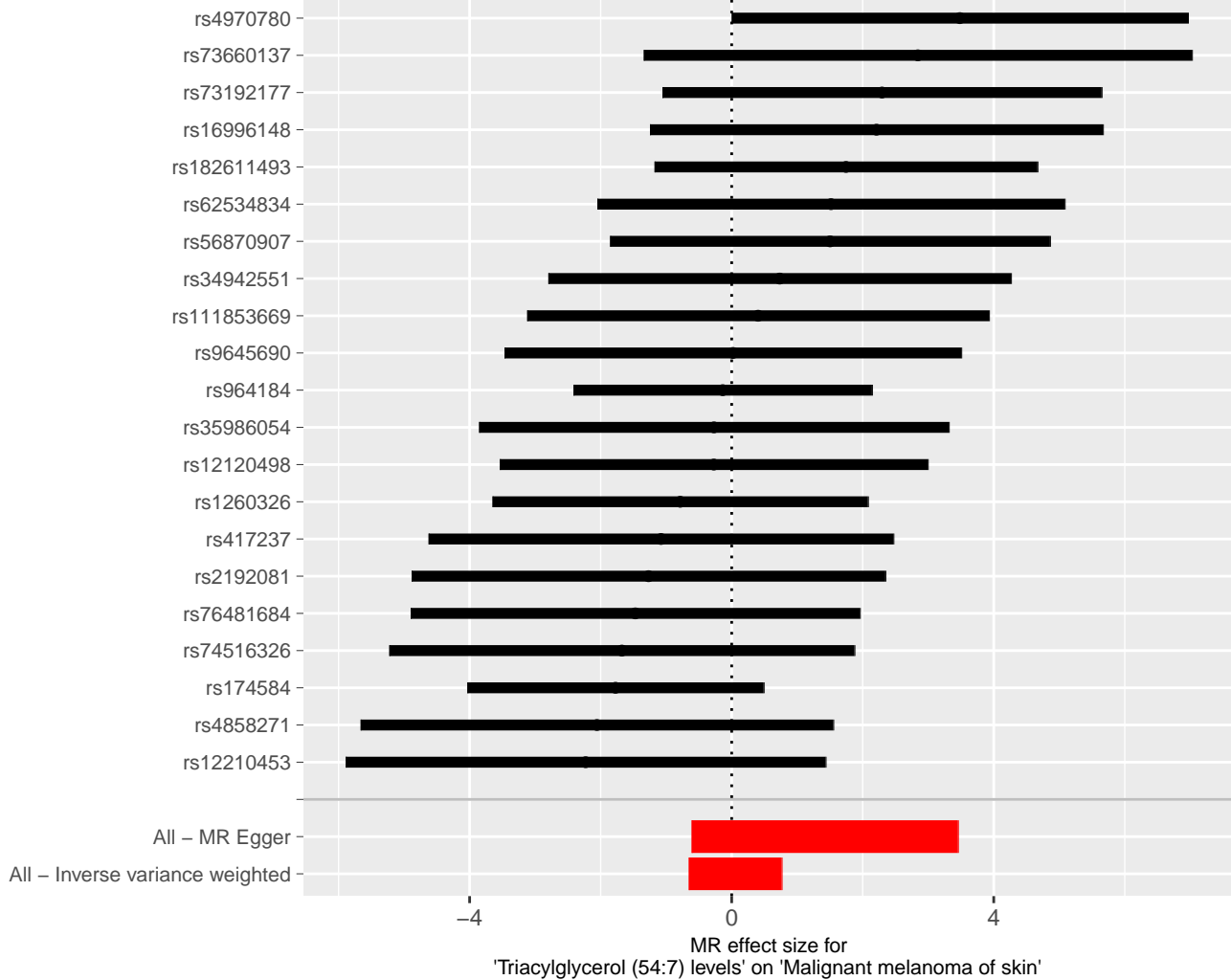

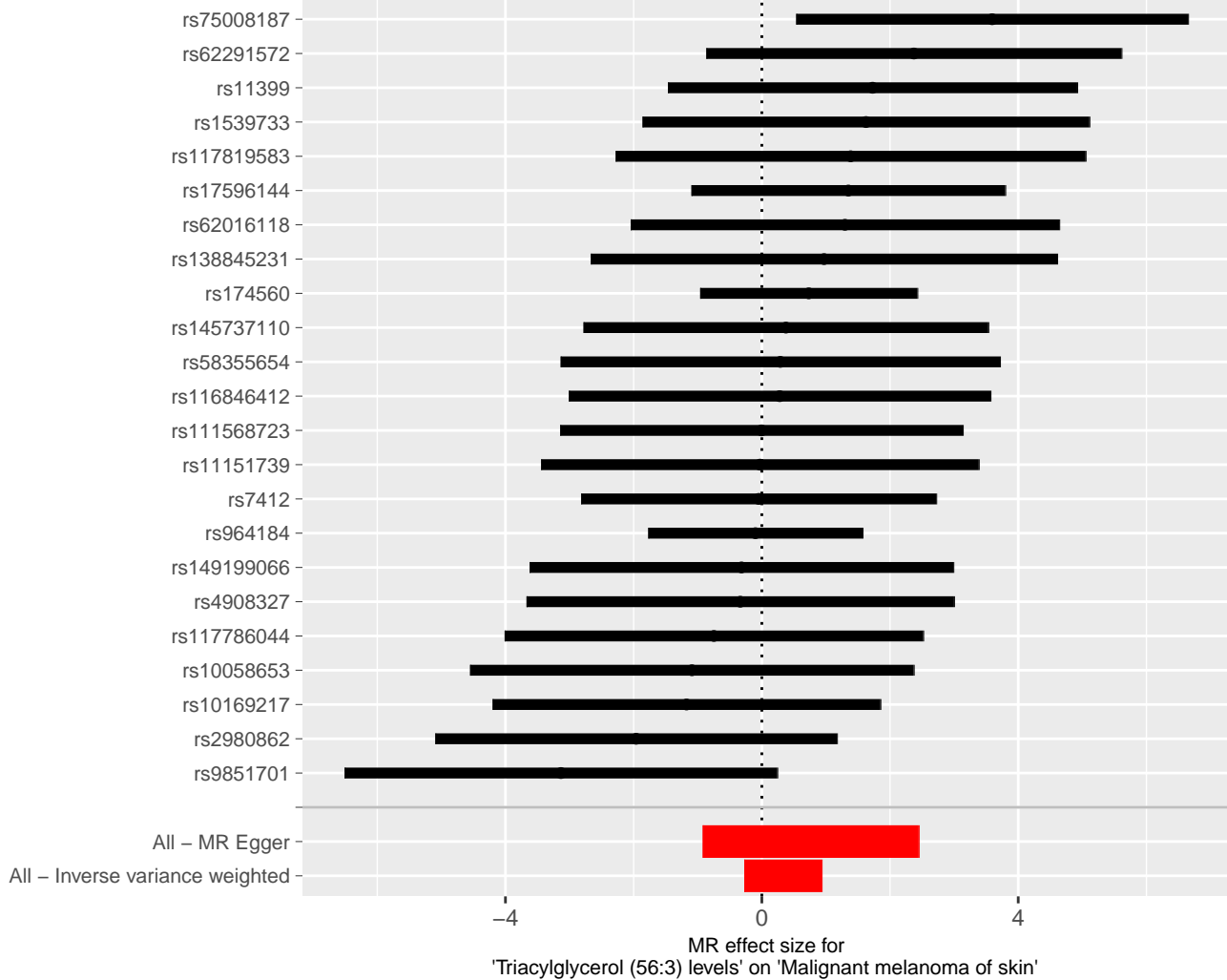

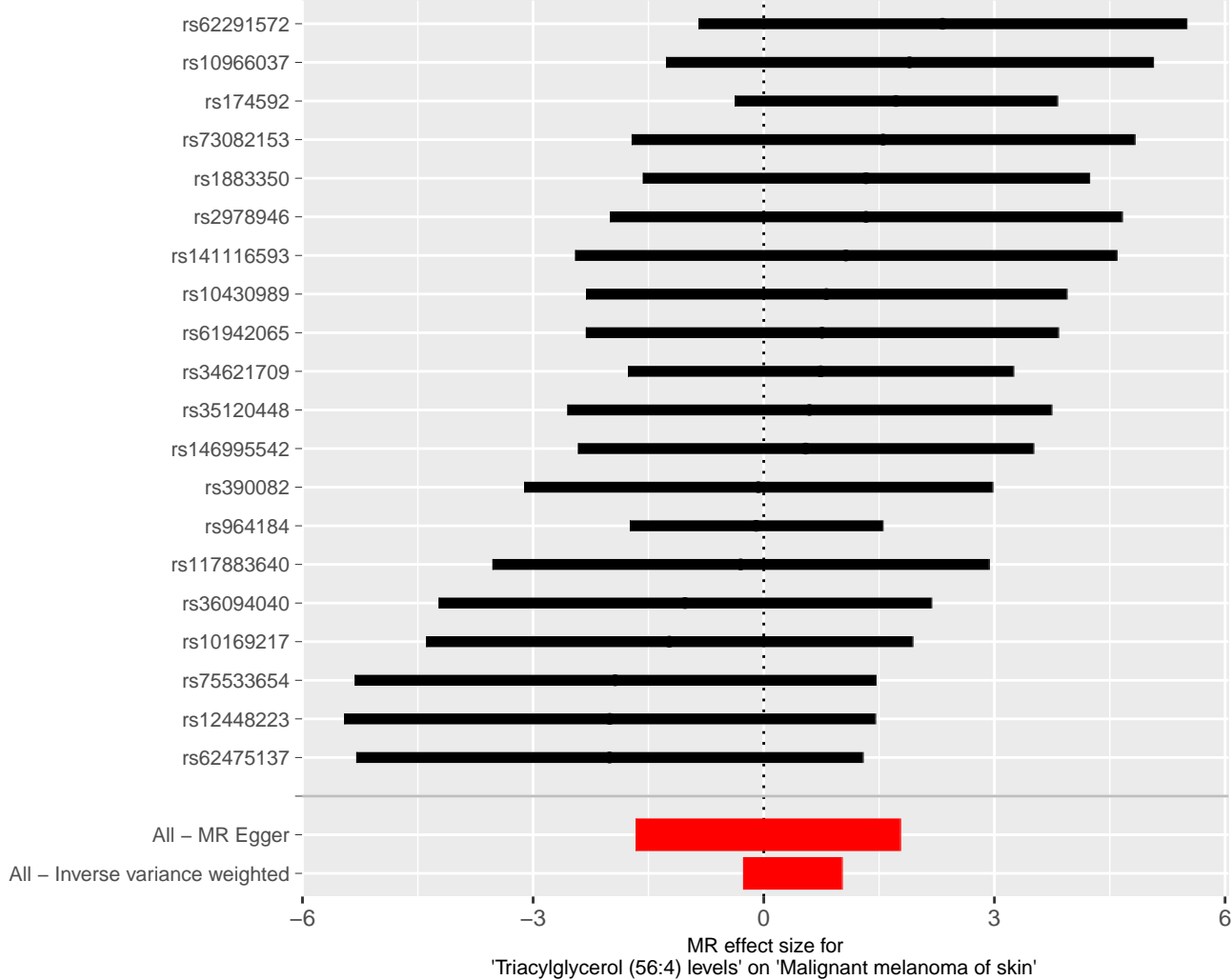

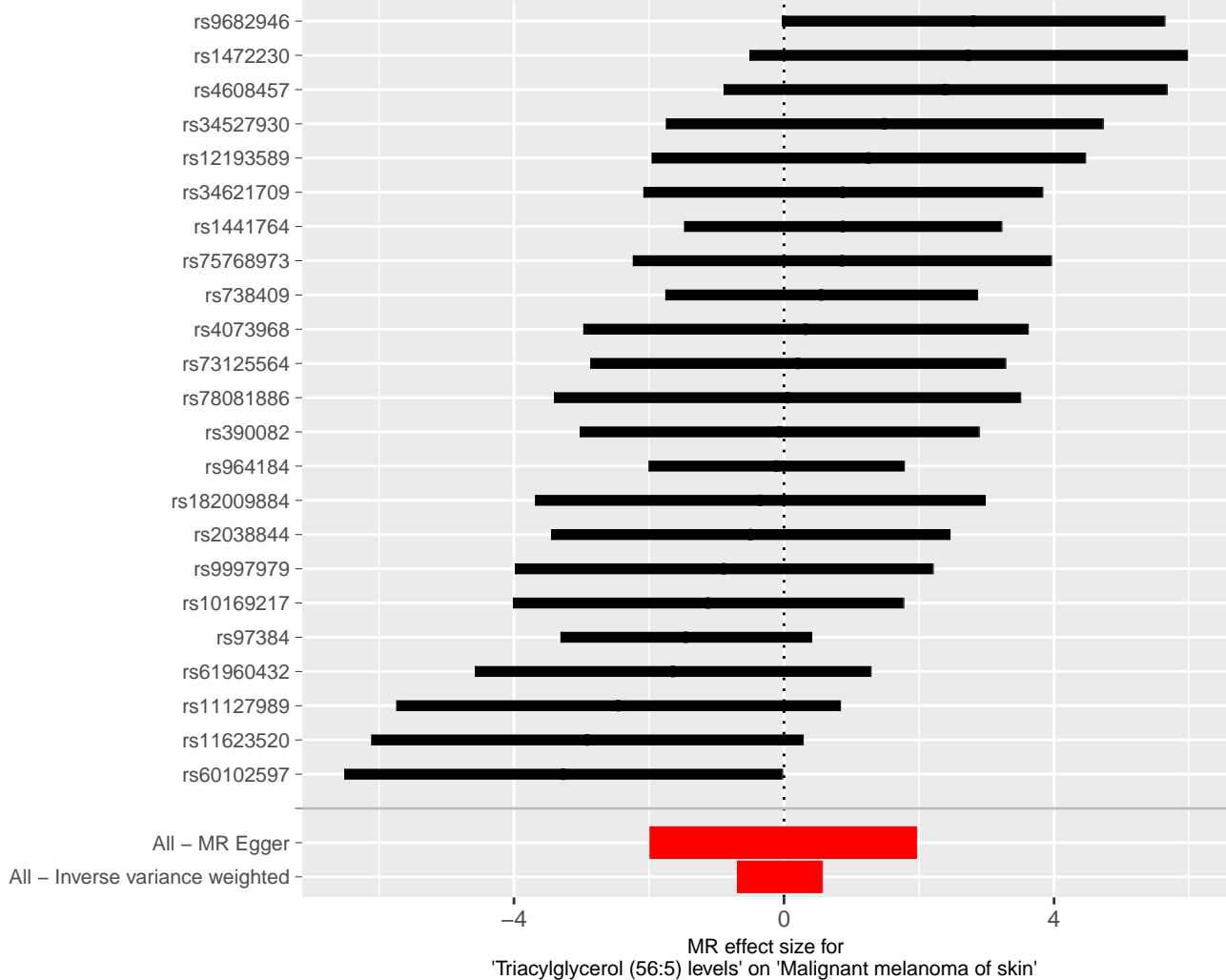

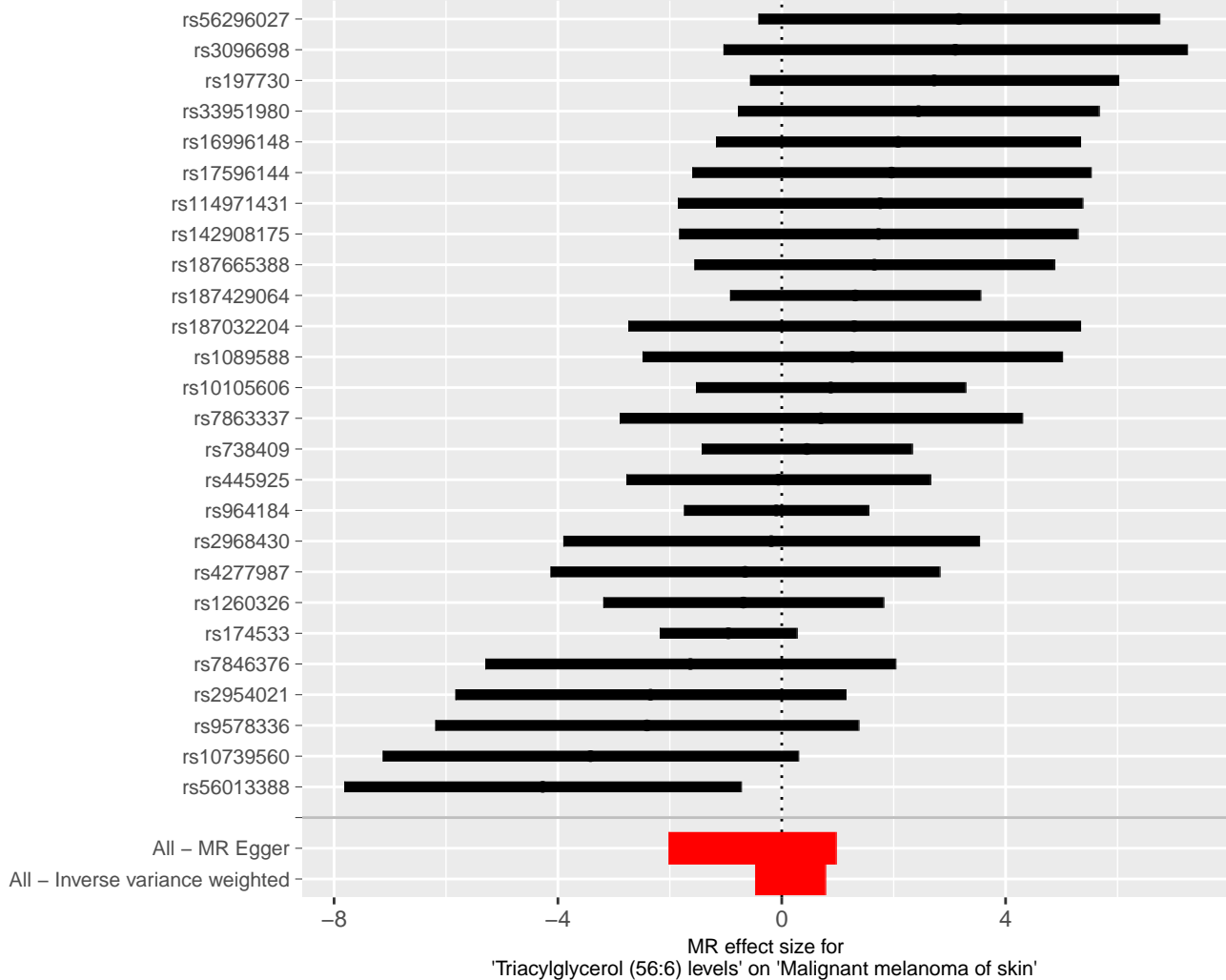

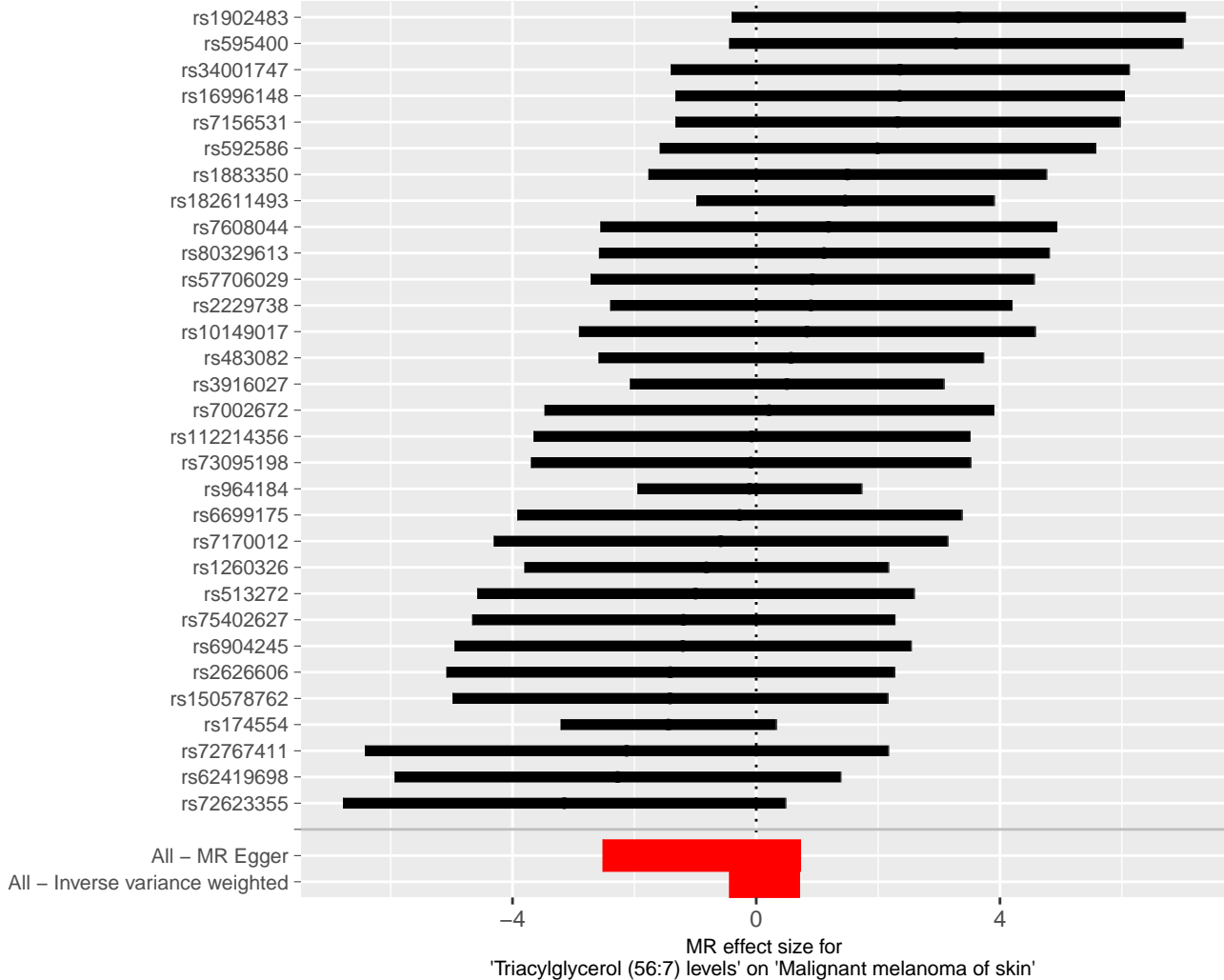

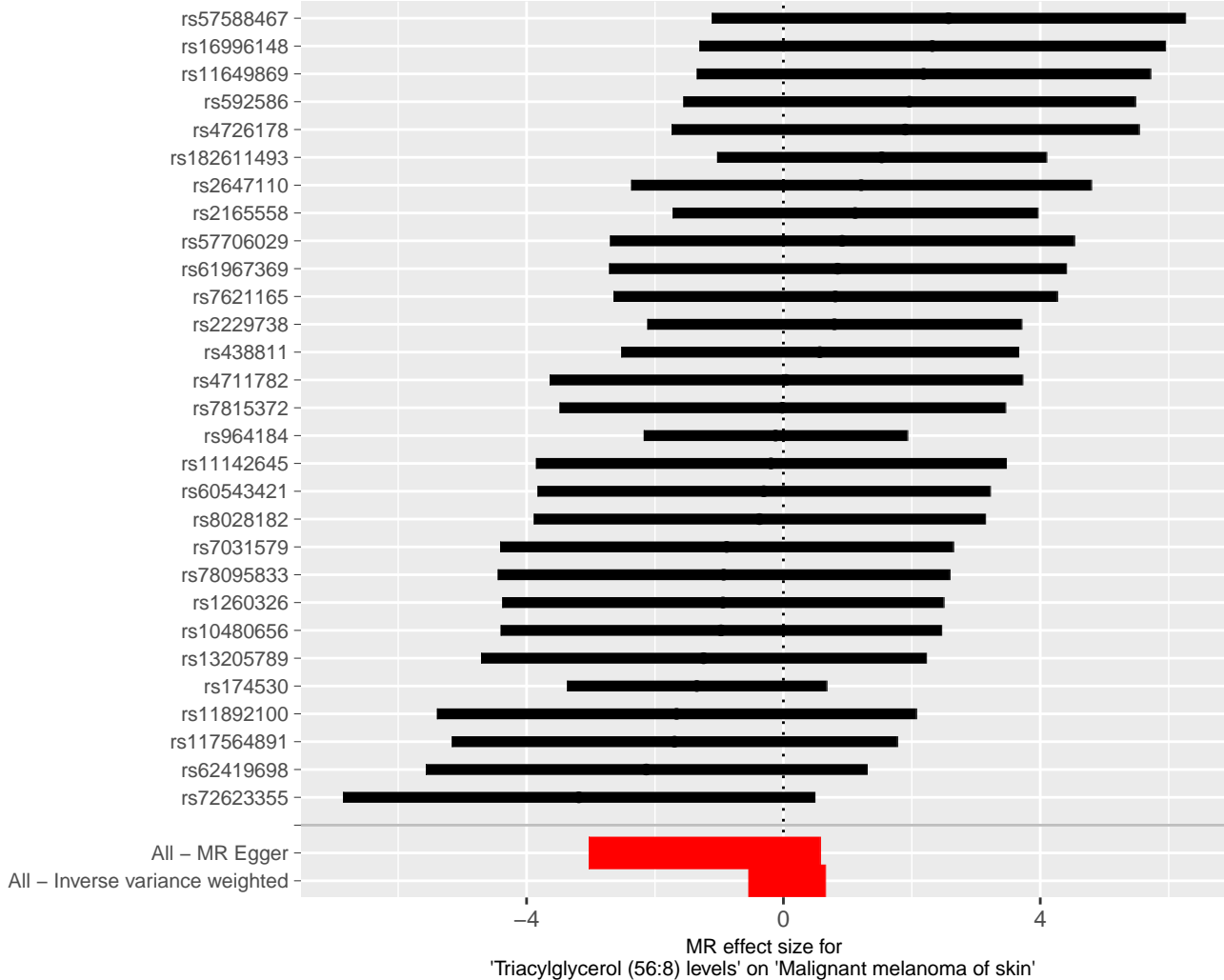

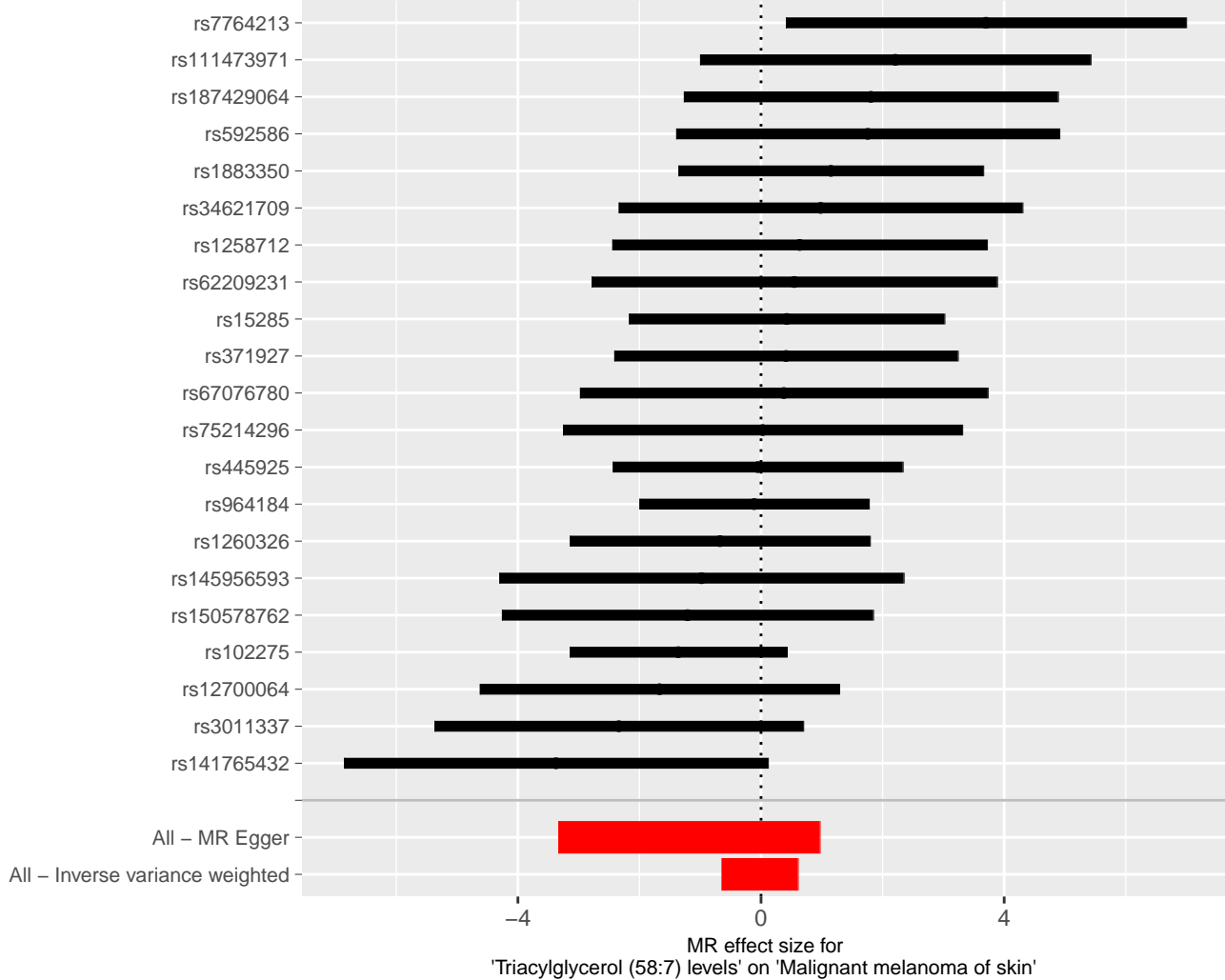

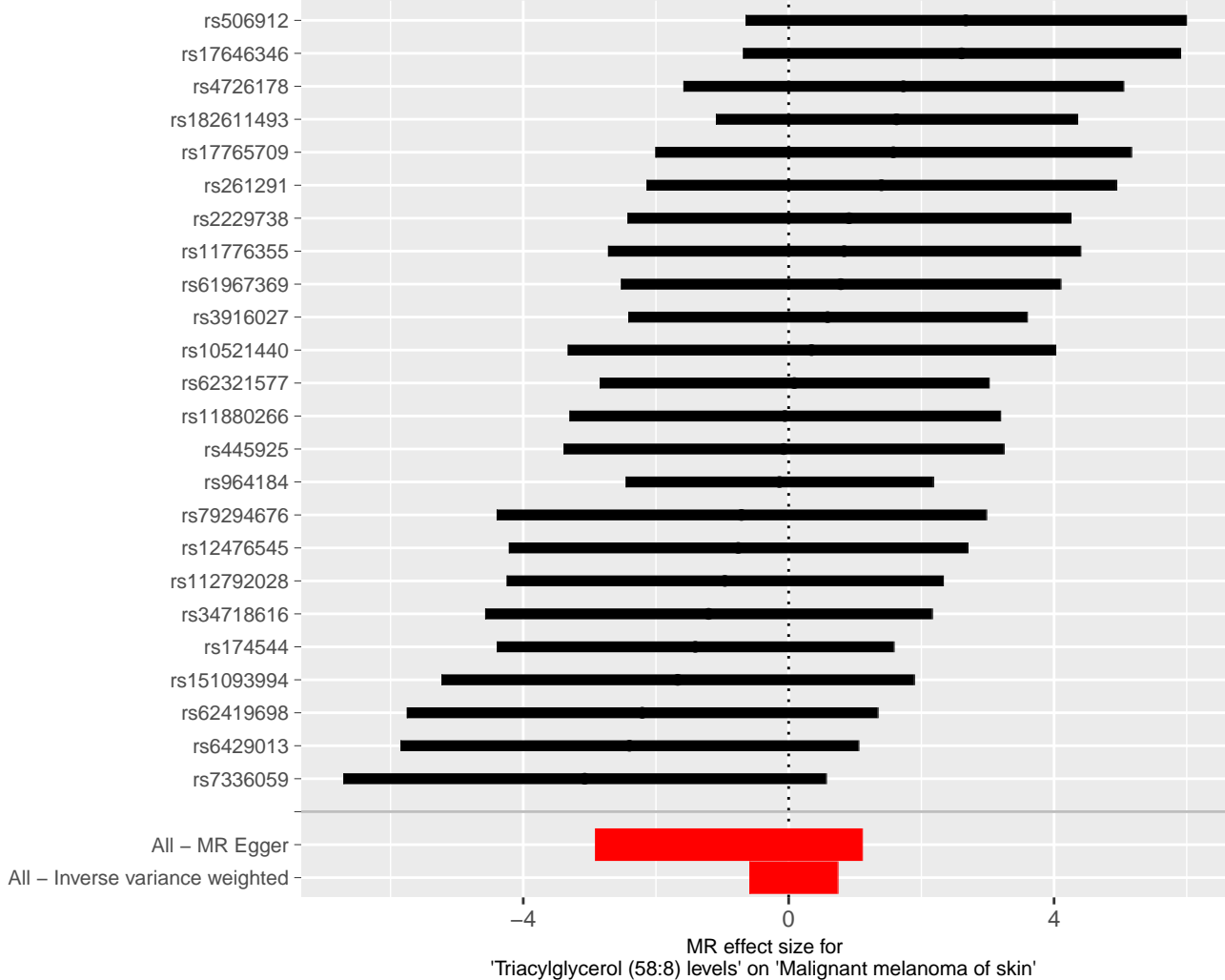

Supplement: Supplementary file 9 [file SupplementaryFile8.pdf]
