# Supplementary material for: Genetically predicted metabolites mediate the association between lipidome and malignant melanoma of skin
Source: Front Oncol. 2024 Sep 10;14:1430533. doi: 10.3389/fonc.2024.1430533 (PMC11419955; doi:10.3389/fonc.2024.1430533)
Supplement: Supplementary file 10 [file SupplementaryFile9.pdf]

Supplementary material 9

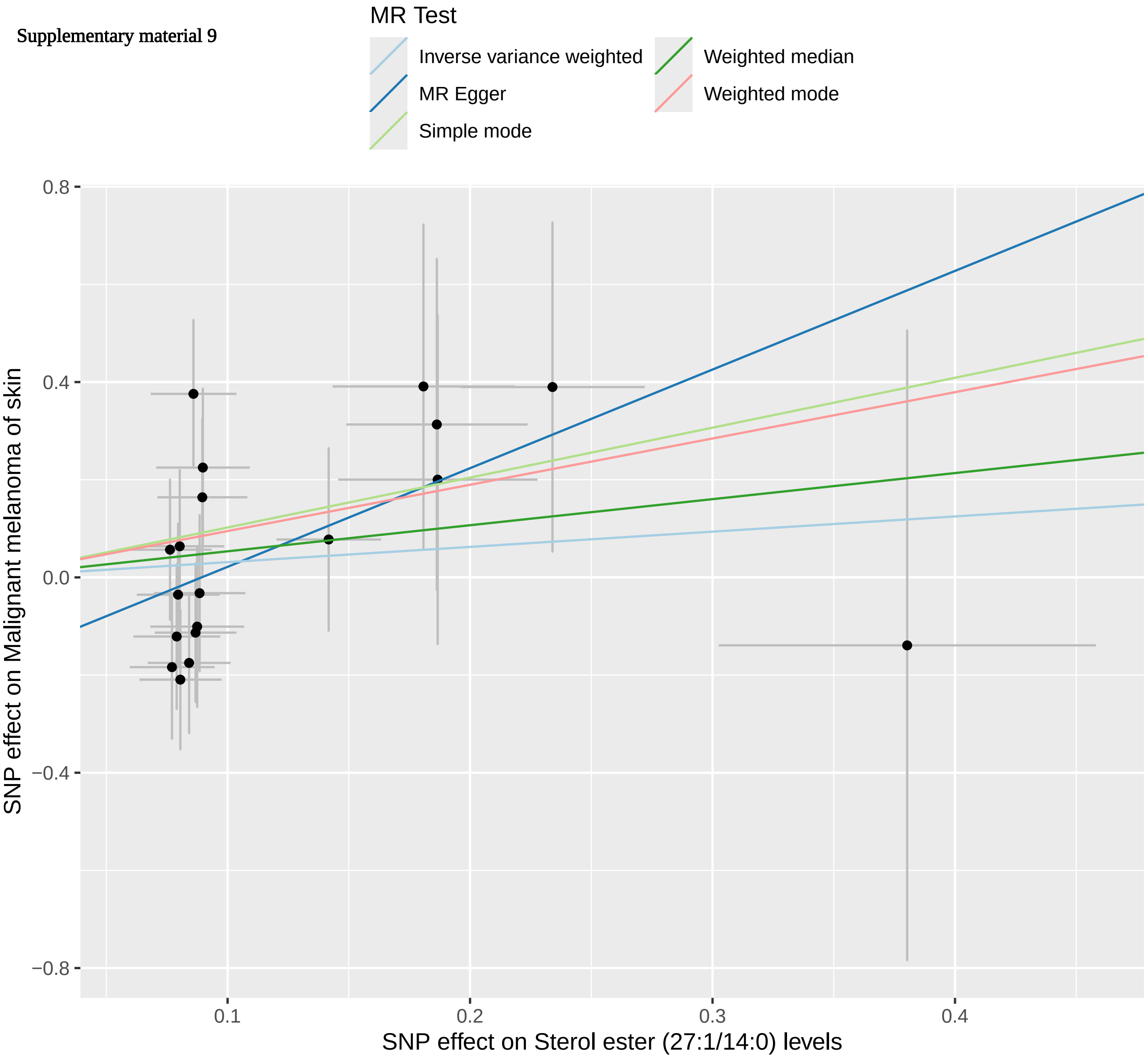

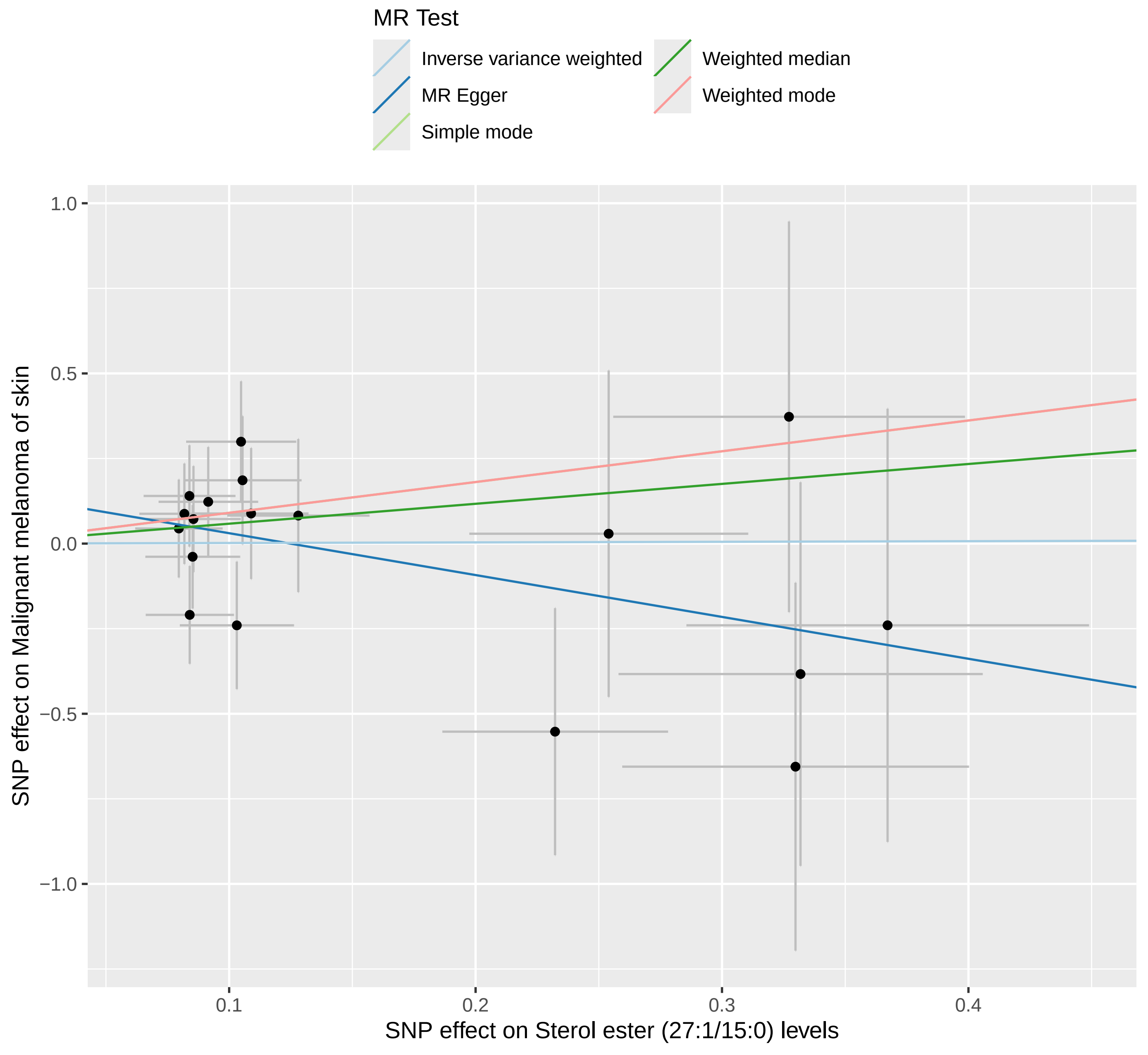

MR Test
Inverse variance weighted
MR Egger
Simple mode
Weighted median
Weighted mode
1.0
0.5
0.0
−0.5
−1.0
0.1
0.2
0.3
0.4
SNP effect on Malignant melanoma of skin
SNP effect on Sterol ester (27:1/15:0) levels

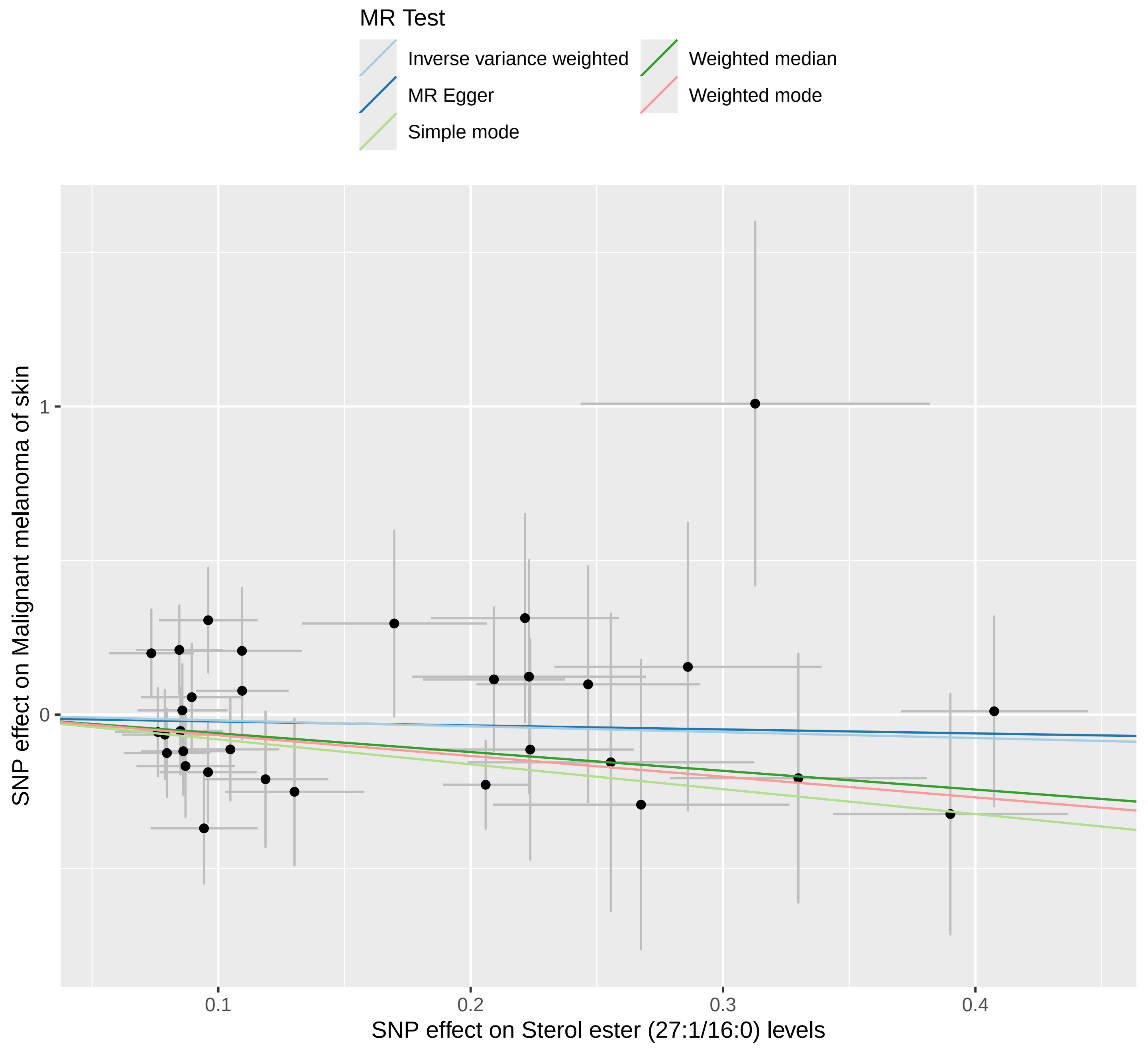
MR Test
Inverse variance weighted
MR Egger
Simple mode
Weighted median
Weighted mode
1
0
0.1
0.2
0.3
0.4
SNP effect on Sterol ester (27:1/16:0) levels
SNP effect on Malignant melanoma of skin

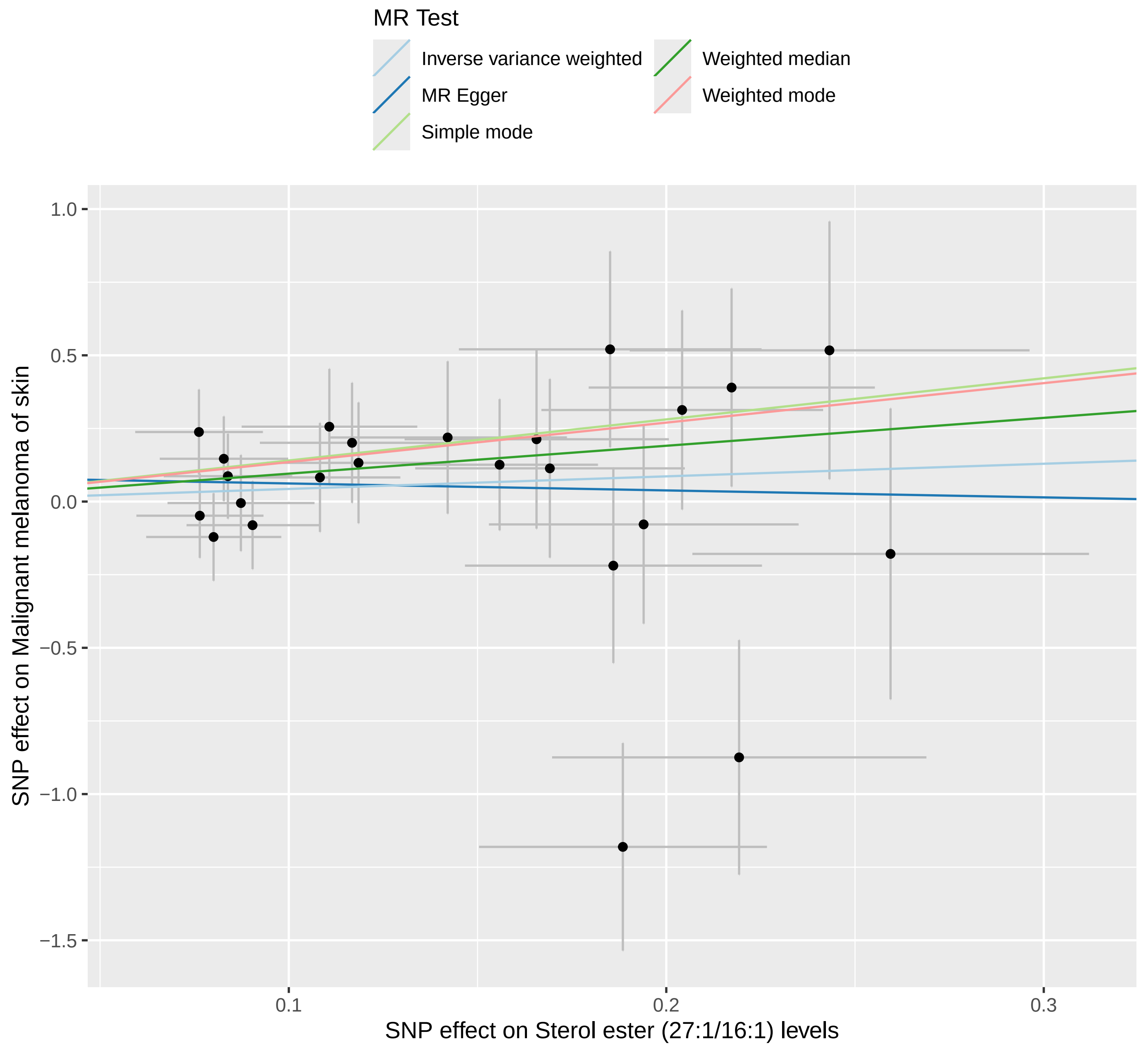
MR Test
Inverse variance weighted
MR Egger
Simple mode
Weighted median
Weighted mode
1.0
0.5
0.0
−0.5
−1.0
−1.5
SNP effect on Malignant melanoma of skin
0.1
0.2
0.3
SNP effect on Sterol ester (27:1/16:1) levels

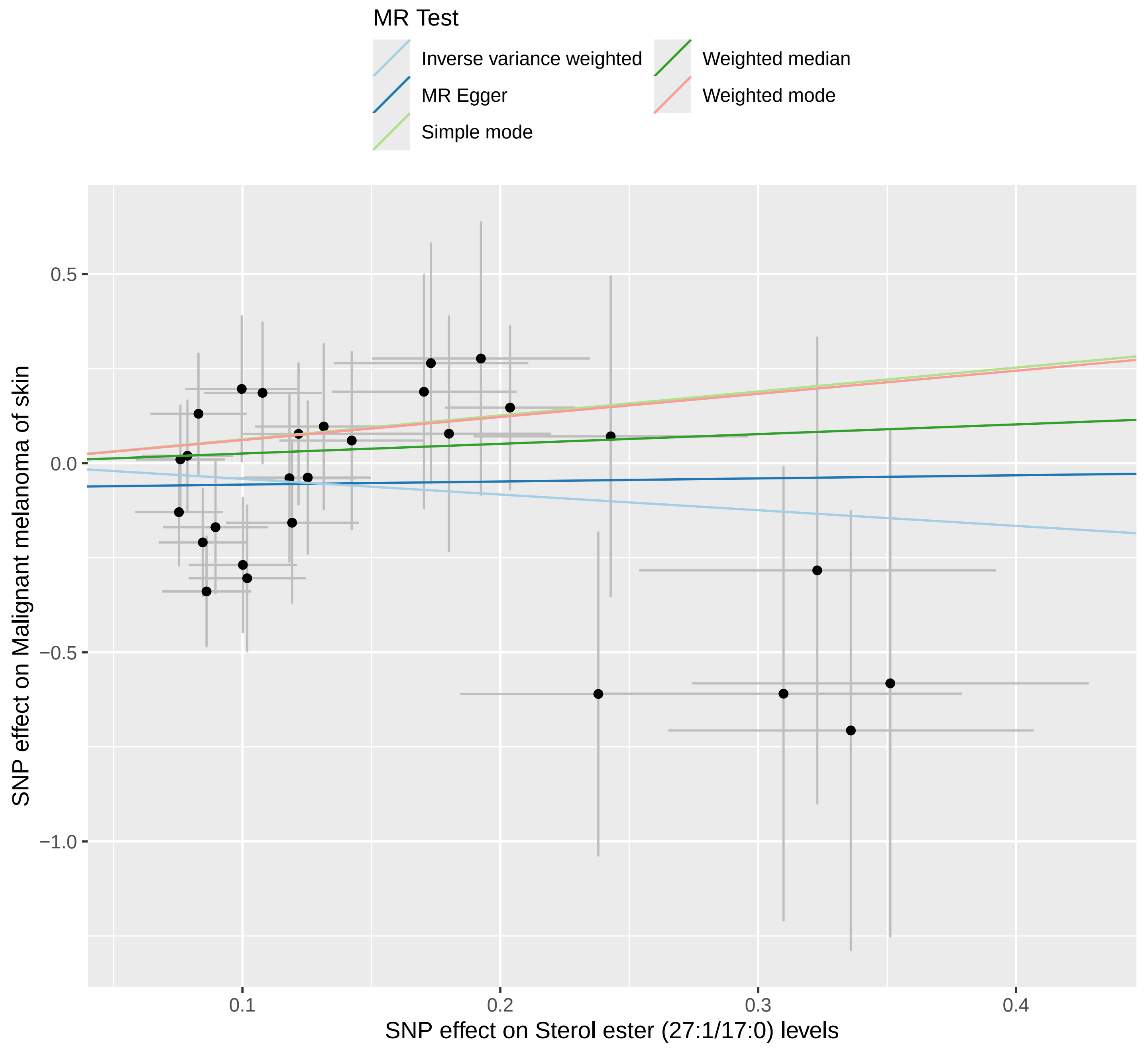

MR Test
Inverse variance weighted
MR Egger
Simple mode
Weighted median
Weighted mode
0.5
0.0
−0.5
−1.0
0.1
0.2
0.3
0.4
SNP effect on Malignant melanoma of skin
SNP effect on Sterol ester (27:1/17:0) levels

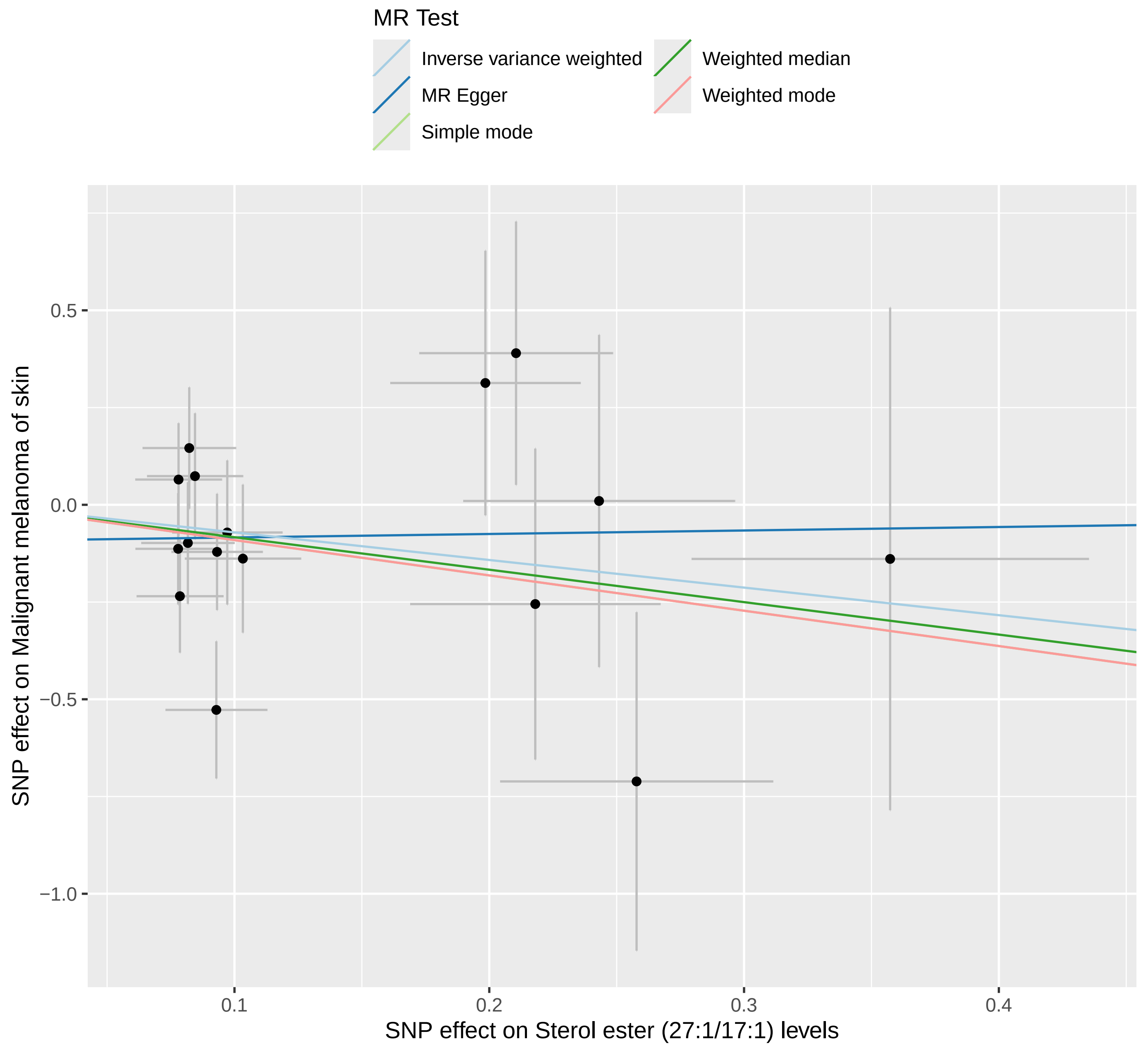
MR Test
Inverse variance weighted
MR Egger
Simple mode
Weighted median
Weighted mode
0.5
0.0
−0.5
−1.0
0.1
0.2
0.3
0.4
SNP effect on Malignant melanoma of skin
SNP effect on Sterol ester (27:1/17:1) levels

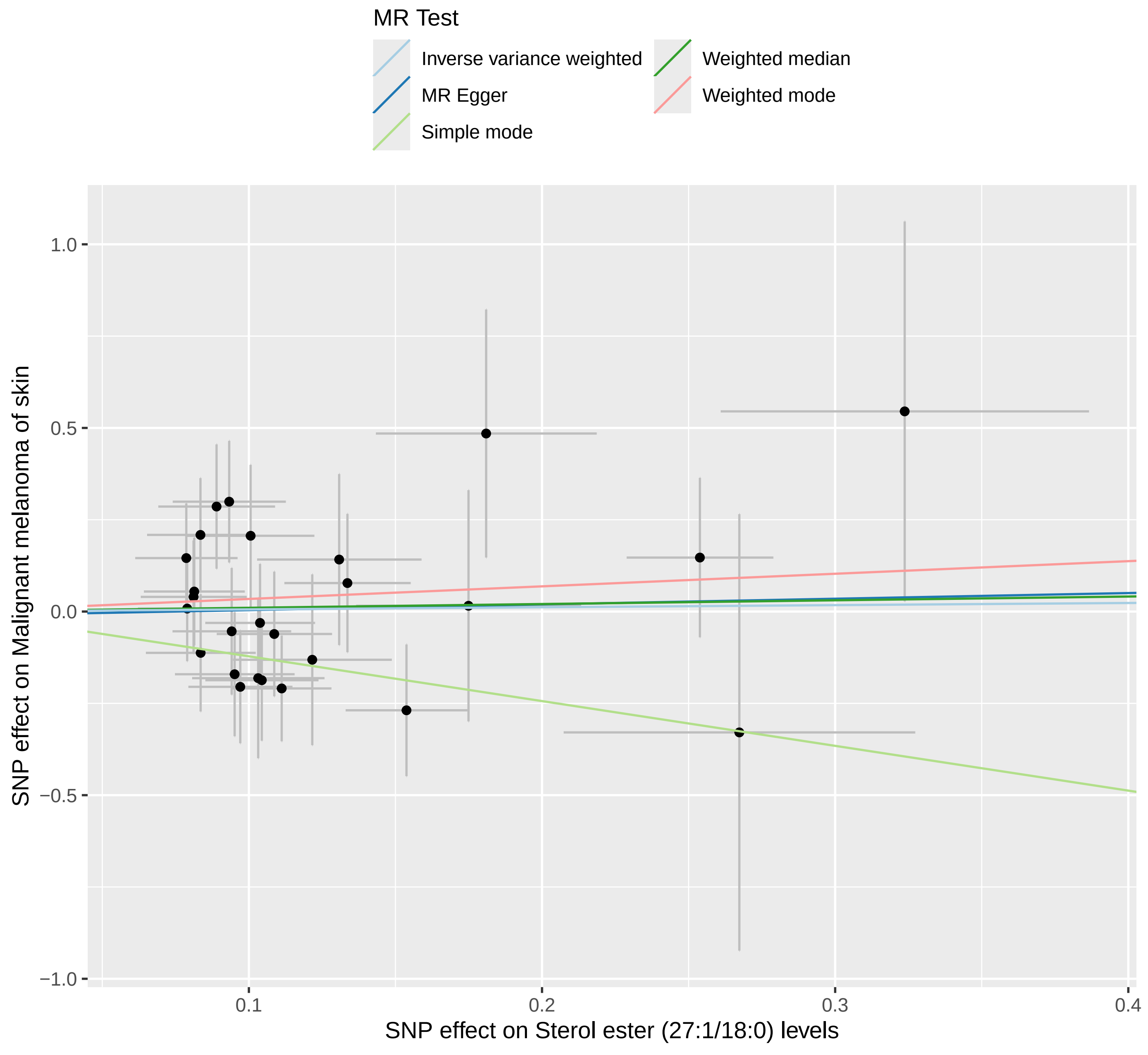

MR Test
Inverse variance weighted
MR Egger
Simple mode
Weighted median
Weighted mode
1.0
0.5
0.0
−0.5
−1.0
0.1
0.2
0.3
0.4
SNP effect on Malignant melanoma of skin
SNP effect on Sterol ester (27:1/18:0) levels

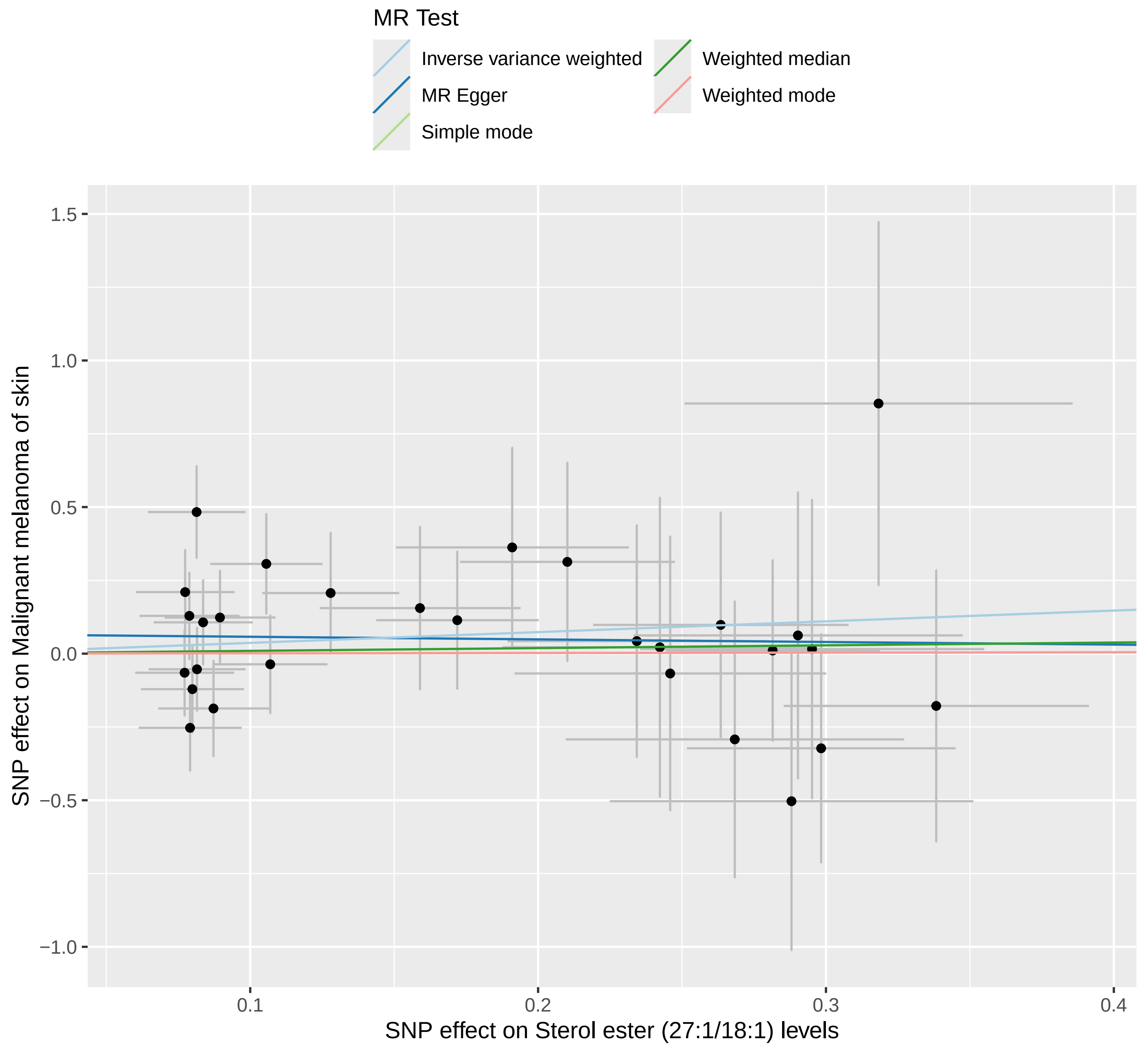
MR Test
Inverse variance weighted
MR Egger
Simple mode
Weighted median
Weighted mode
1.5
1.0
0.5
0.0
−0.5
−1.0
0.1
0.2
0.3
0.4
SNP effect on Malignant melanoma of skin
SNP effect on Sterol ester (27:1/18:1) levels

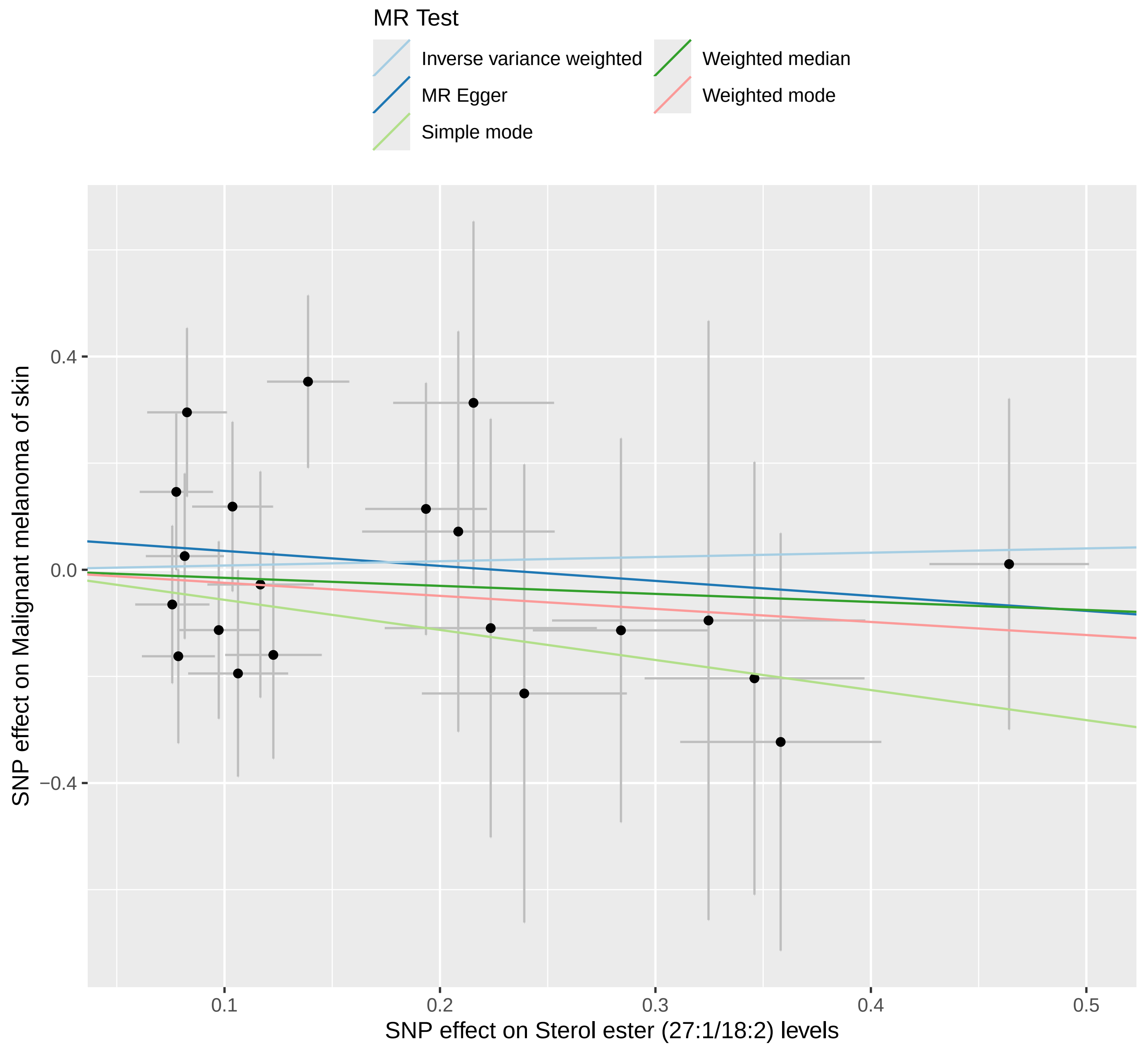
MR Test
Inverse variance weighted
MR Egger
Simple mode
Weighted median
Weighted mode
0.4
0.0
−0.4
0.1
0.2
0.3
0.4
0.5
SNP effect on Malignant melanoma of skin
SNP effect on Sterol ester (27:1/18:2) levels

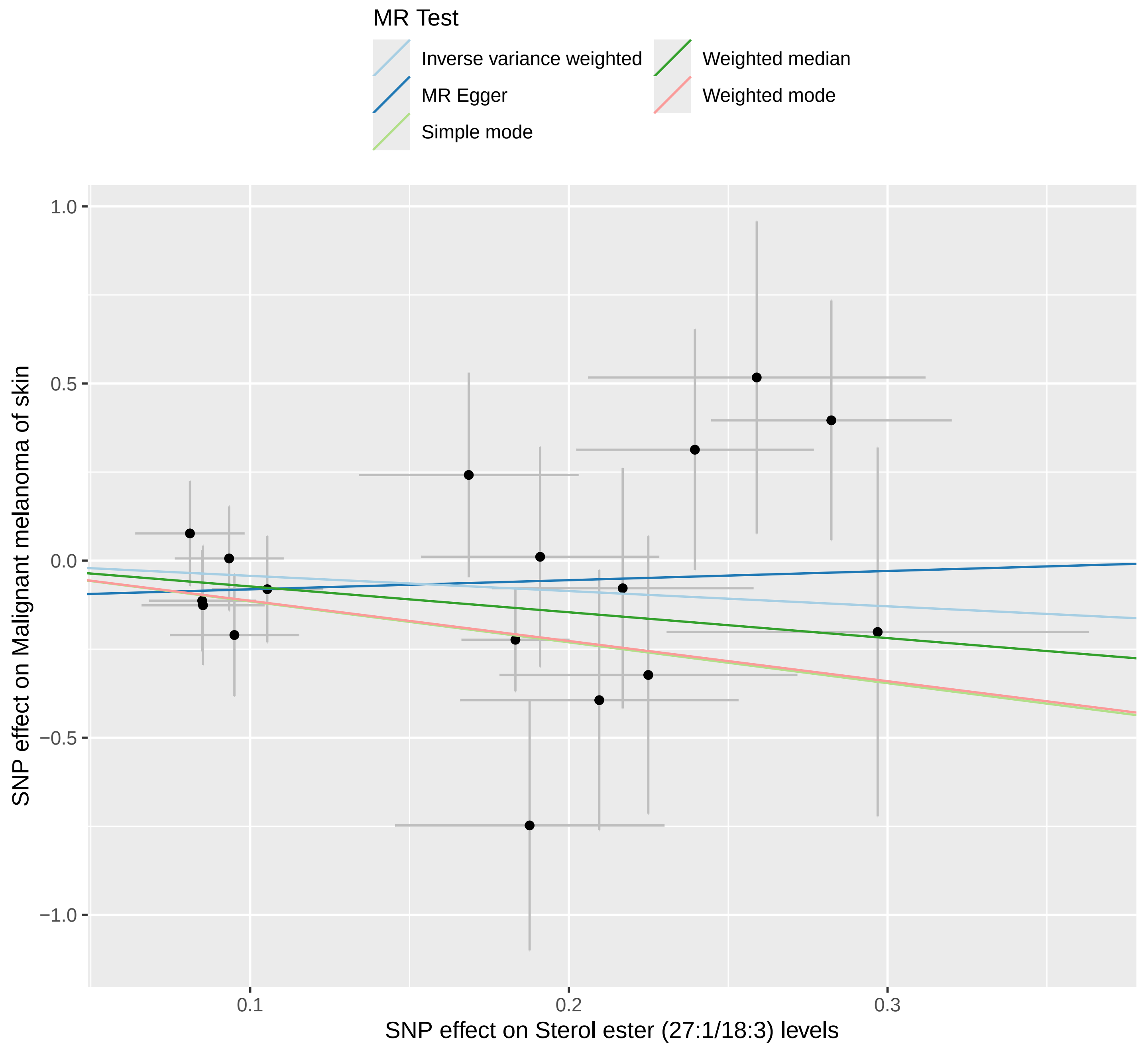
MR Test
Inverse variance weighted
MR Egger
Simple mode
Weighted median
Weighted mode
1.0
0.5
0.0
−0.5
−1.0
0.1
0.2
0.3
SNP effect on Malignant melanoma of skin
SNP effect on Sterol ester (27:1/18:3) levels

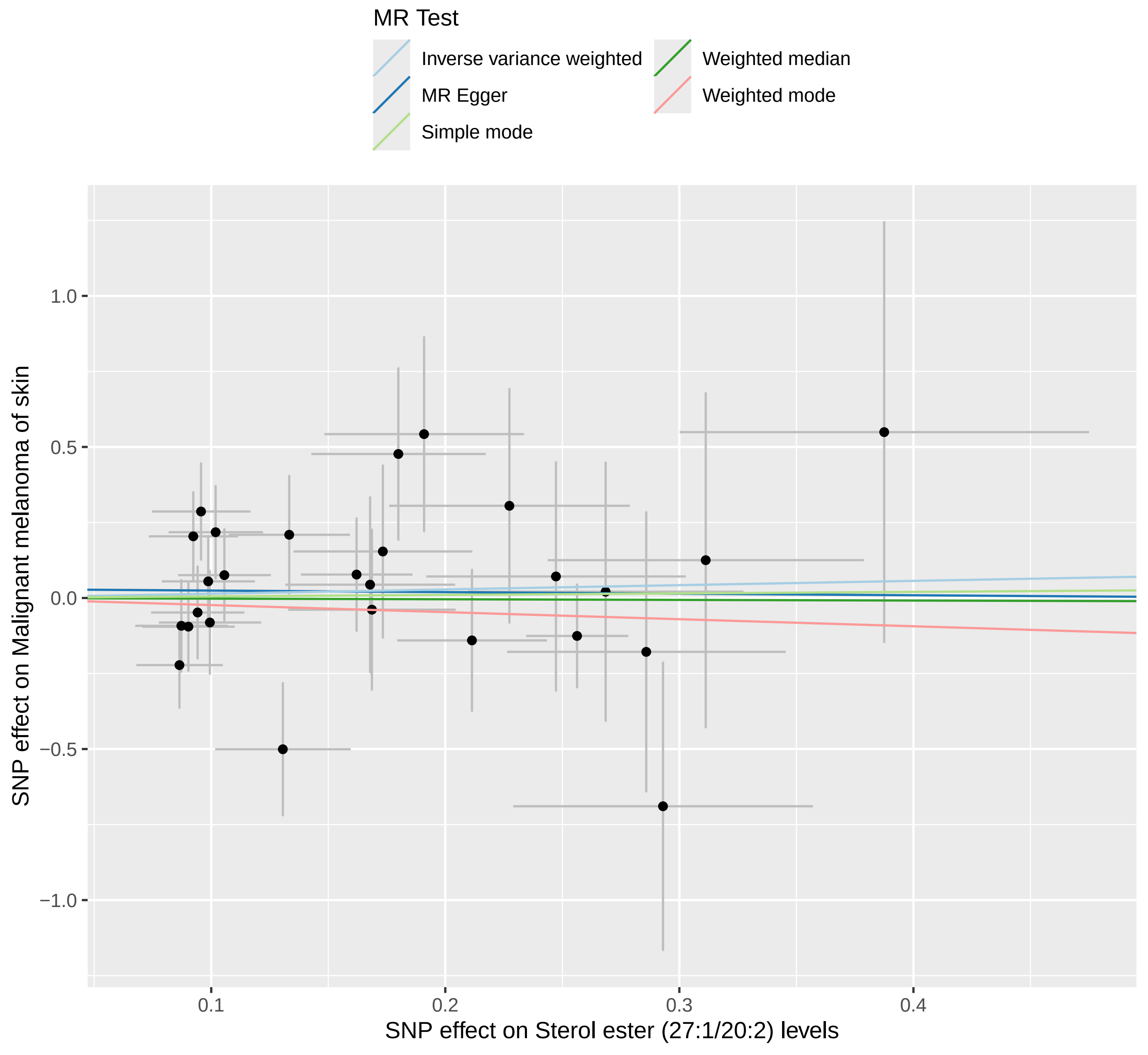

MR Test
Inverse variance weighted
MR Egger
Simple mode
Weighted median
Weighted mode
1.0
0.5
0.0
−0.5
−1.0
0.1
0.2
0.3
0.4
SNP effect on Malignant melanoma of skin
SNP effect on Sterol ester (27:1/20:2) levels

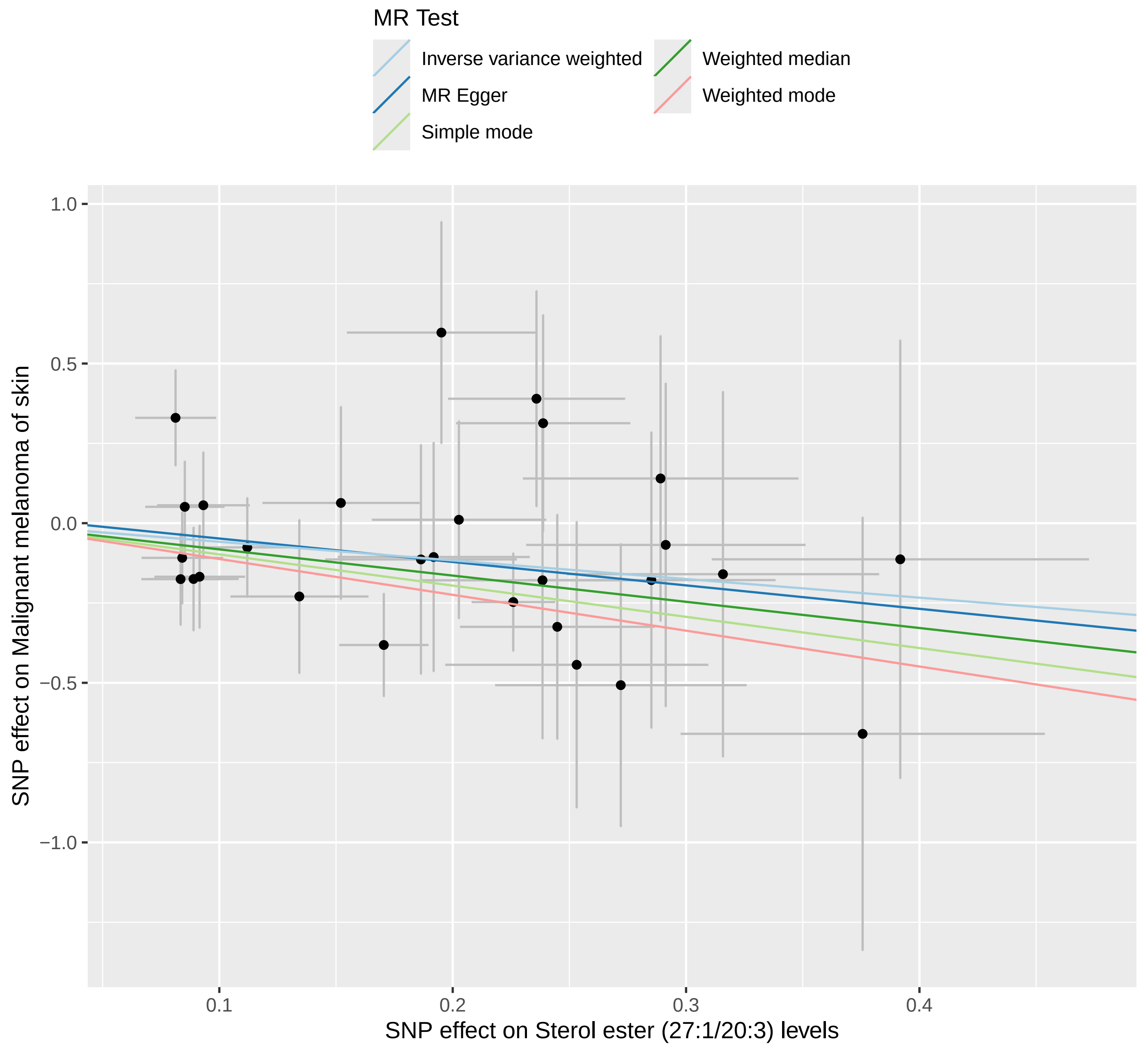

MR Test
Inverse variance weighted
MR Egger
Simple mode
Weighted median
Weighted mode
1.0
0.5
0.0
−0.5
−1.0
0.1
0.2
0.3
0.4
SNP effect on Malignant melanoma of skin
SNP effect on Sterol ester (27:1/20:3) levels

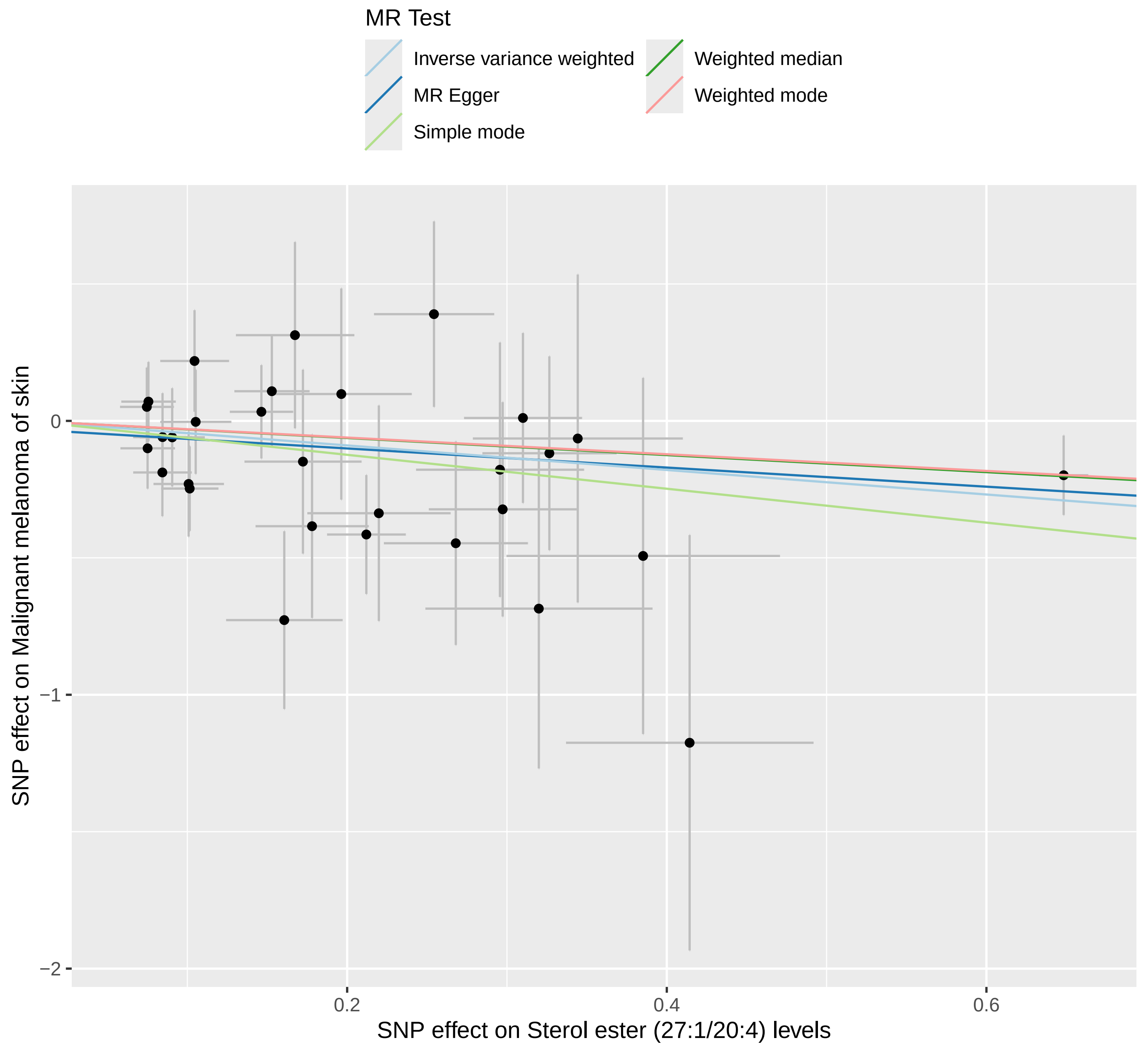
MR Test
Inverse variance weighted
MR Egger
Simple mode
Weighted median
Weighted mode
SNP effect on Malignant melanoma of skin
0
−1
−2
0.2
0.4
0.6
SNP effect on Sterol ester (27:1/20:4) levels

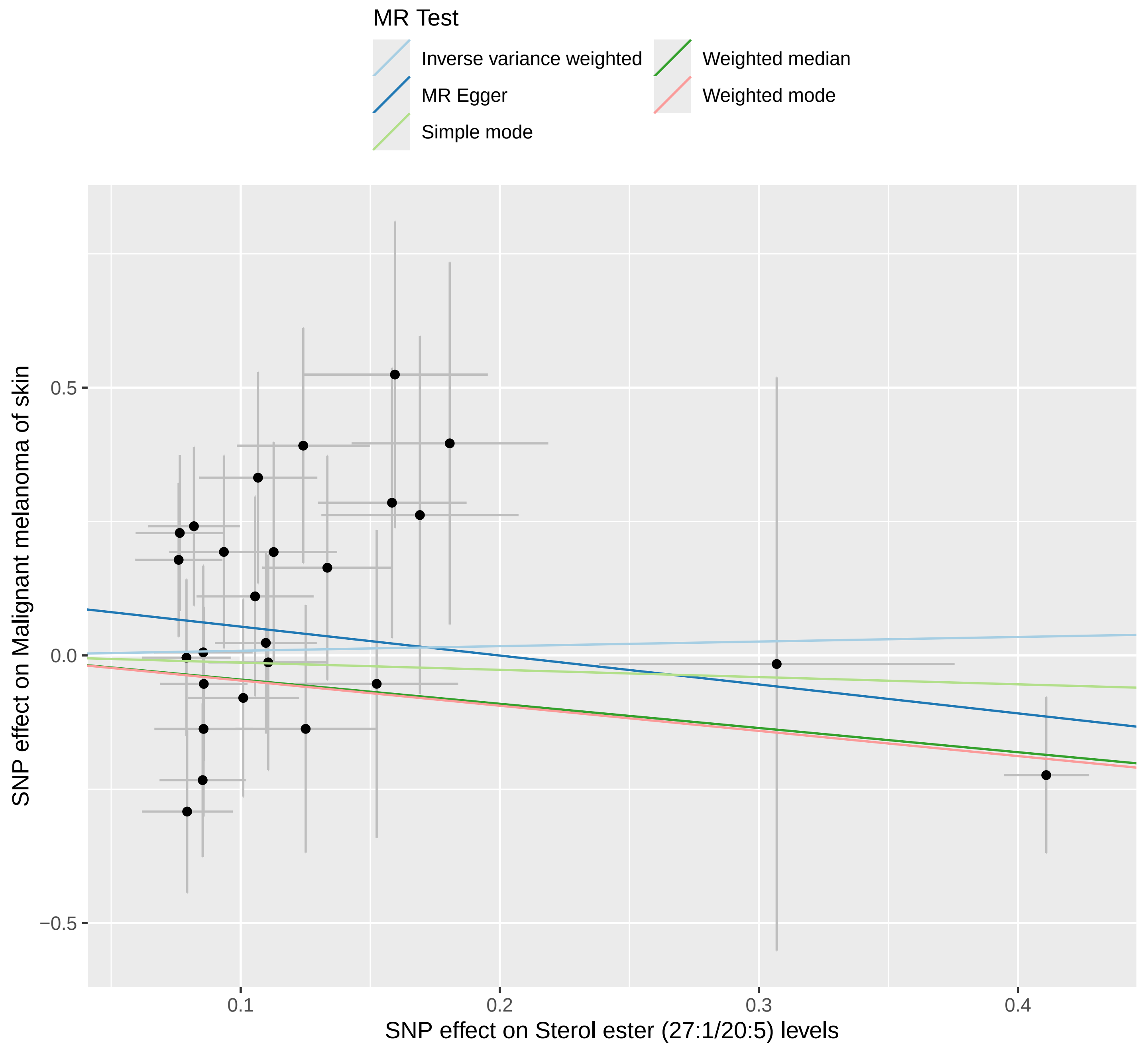
MR Test
Inverse variance weighted
MR Egger
Simple mode
Weighted median
Weighted mode
0.5
0.0
−0.5
0.1
0.2
0.3
0.4
SNP effect on Malignant melanoma of skin
SNP effect on Sterol ester (27:1/20:5) levels

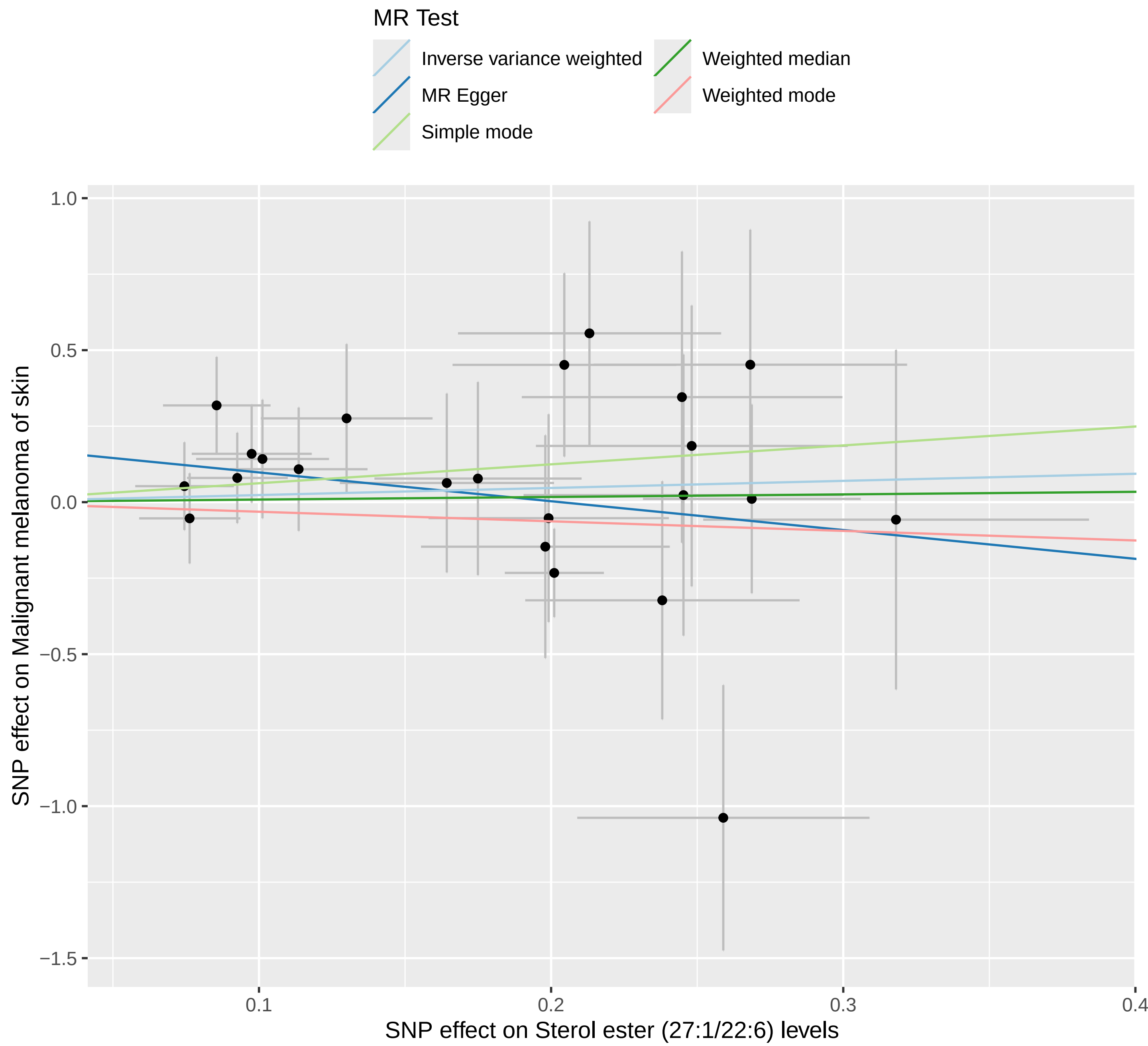
MR Test
Inverse variance weighted
MR Egger
Simple mode
Weighted median
Weighted mode
1.0
0.5
0.0
−0.5
−1.0
−1.5
0.1
0.2
0.3
0.4
SNP effect on Malignant melanoma of skin
SNP effect on Sterol ester (27:1/22:6) levels

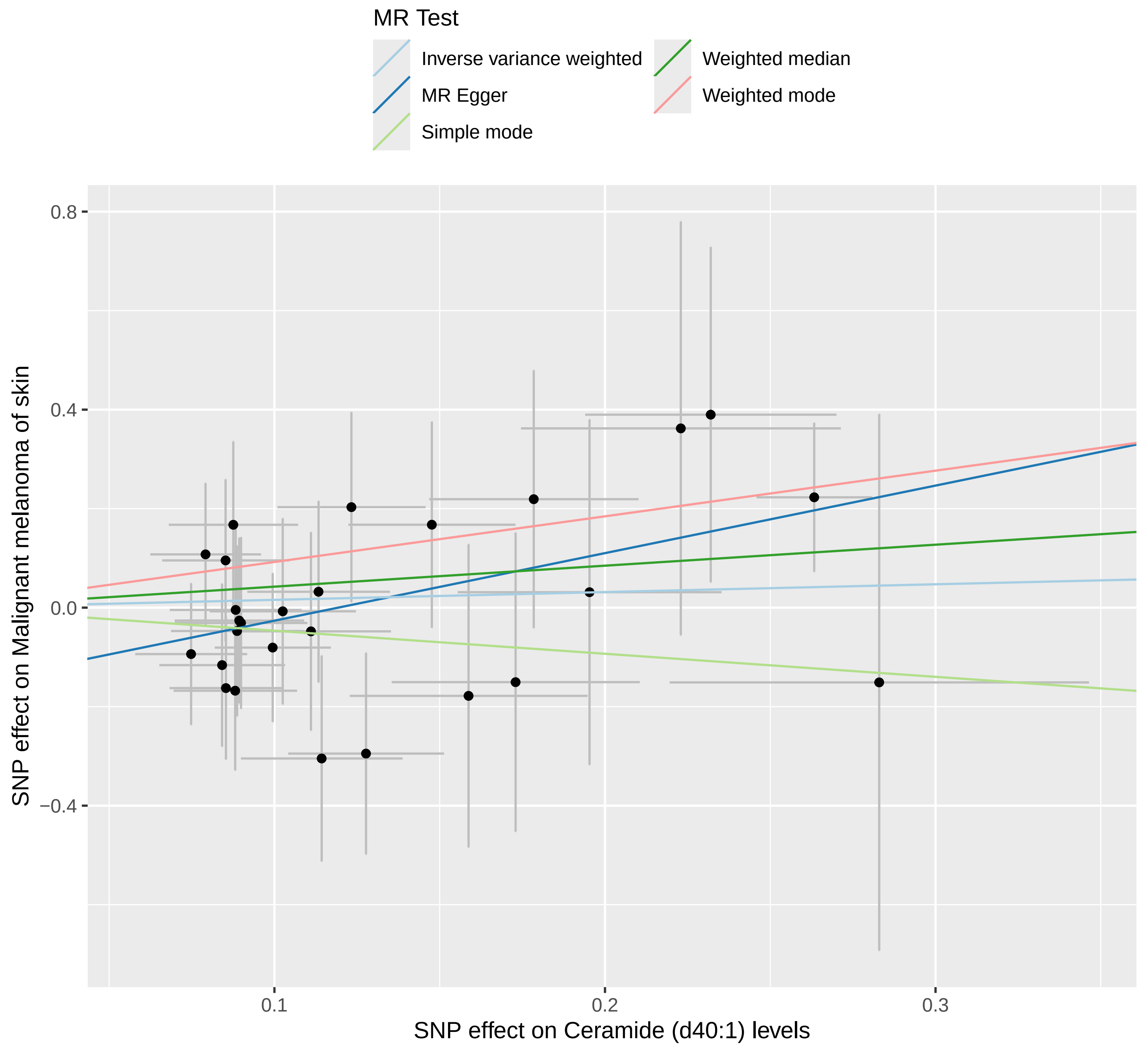
MR Test
Inverse variance weighted
MR Egger
Simple mode
Weighted median
Weighted mode
0.8
0.4
0.0
−0.4
0.1
0.2
0.3
SNP effect on Malignant melanoma of skin
SNP effect on Ceramide (d40:1) levels

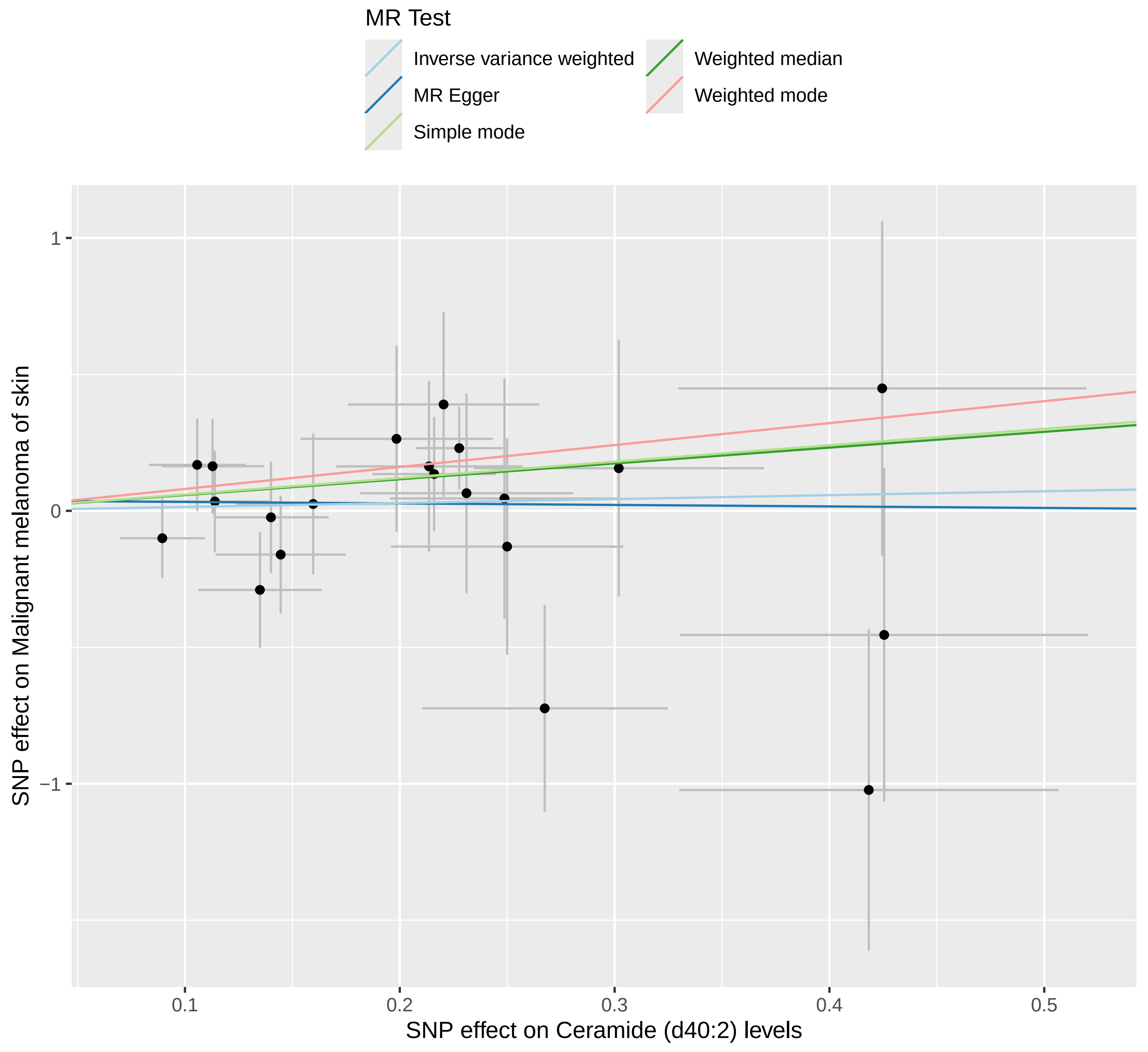
MR Test
Inverse variance weighted
MR Egger
Simple mode
Weighted median
Weighted mode
SNP effect on Malignant melanoma of skin
1
0
−1
0.1
0.2
0.3
0.4
0.5
SNP effect on Ceramide (d40:2) levels

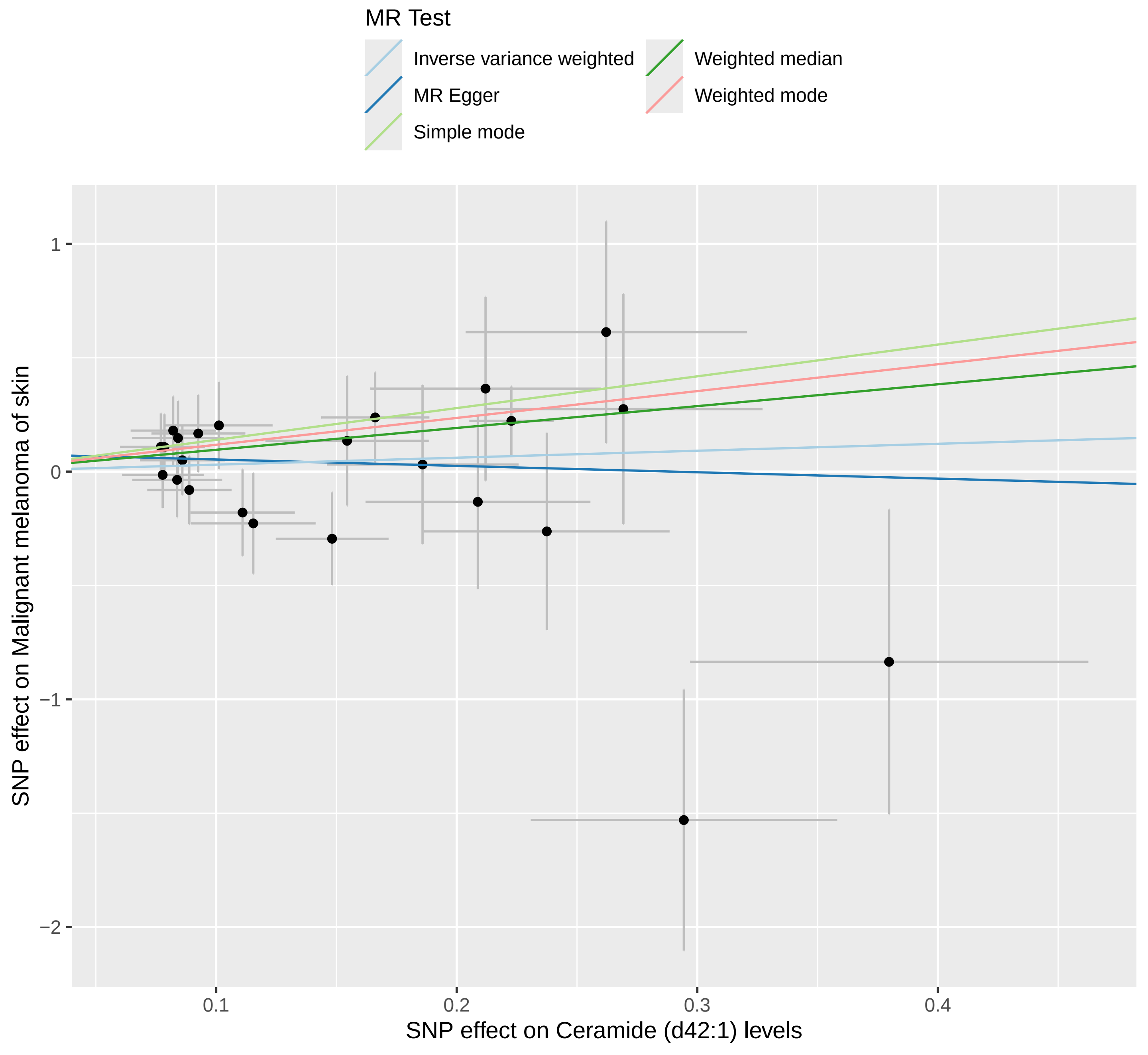
MR Test
Inverse variance weighted
MR Egger
Simple mode
Weighted median
Weighted mode
1
0
−1
−2
0.1
0.2
0.3
0.4
SNP effect on Malignant melanoma of skin
SNP effect on Ceramide (d42:1) levels

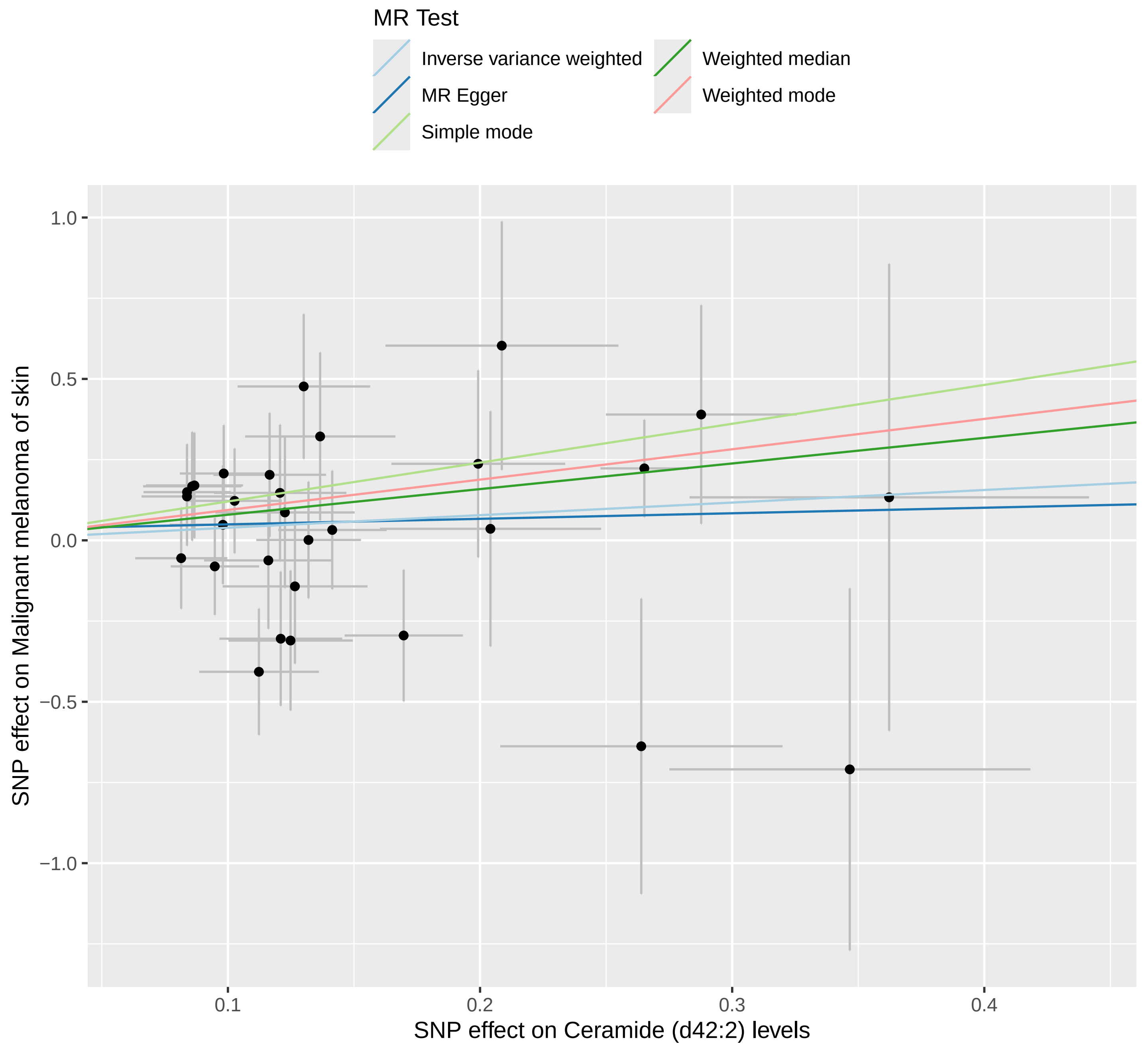

MR Test
Inverse variance weighted
MR Egger
Simple mode
Weighted median
Weighted mode
1.0
0.5
0.0
−0.5
−1.0
SNP effect on Malignant melanoma of skin
0.1
0.2
0.3
0.4
SNP effect on Ceramide (d42:2) levels

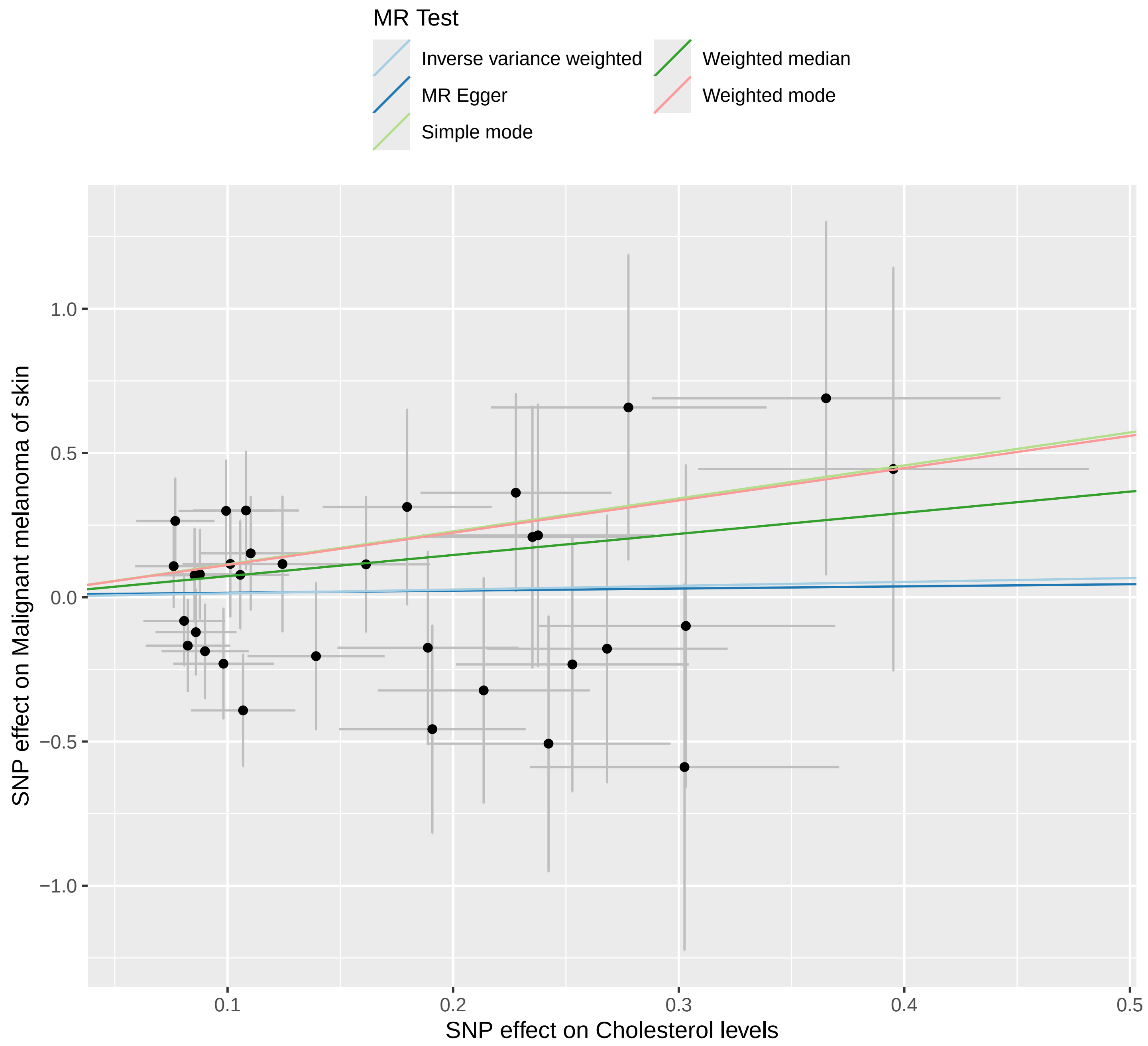

MR Test
Inverse variance weighted
MR Egger
Simple mode
Weighted median
Weighted mode
1.0
0.5
0.0
−0.5
−1.0
0.1
0.2
0.3
0.4
0.5
SNP effect on Cholesterol levels
SNP effect on Malignant melanoma of skin

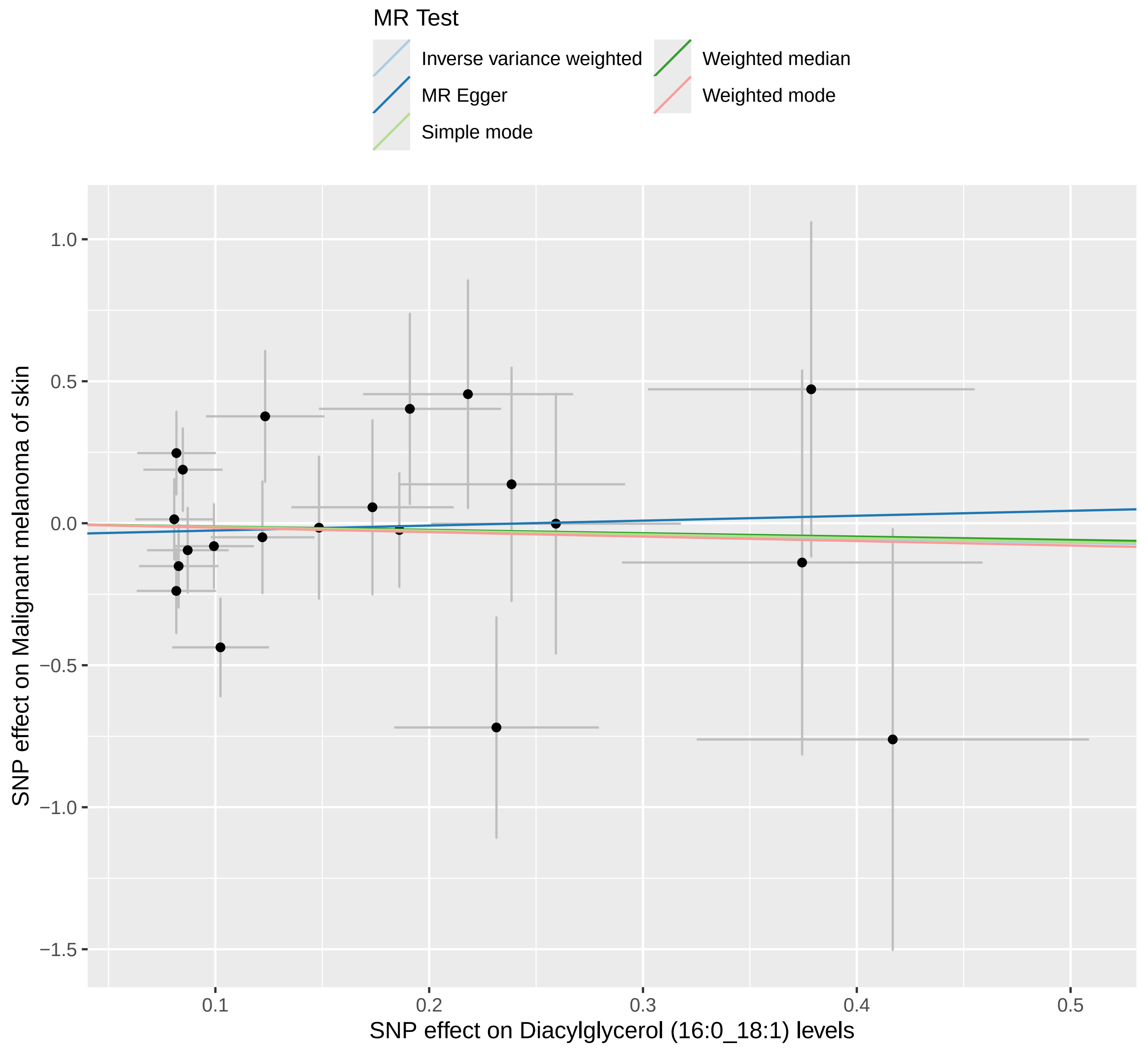
MR Test
Inverse variance weighted
MR Egger
Simple mode
Weighted median
Weighted mode
1.0
0.5
0.0
−0.5
−1.0
−1.5
0.1
0.2
0.3
0.4
0.5
SNP effect on Malignant melanoma of skin
SNP effect on Diacylglycerol (16:0_18:1) levels

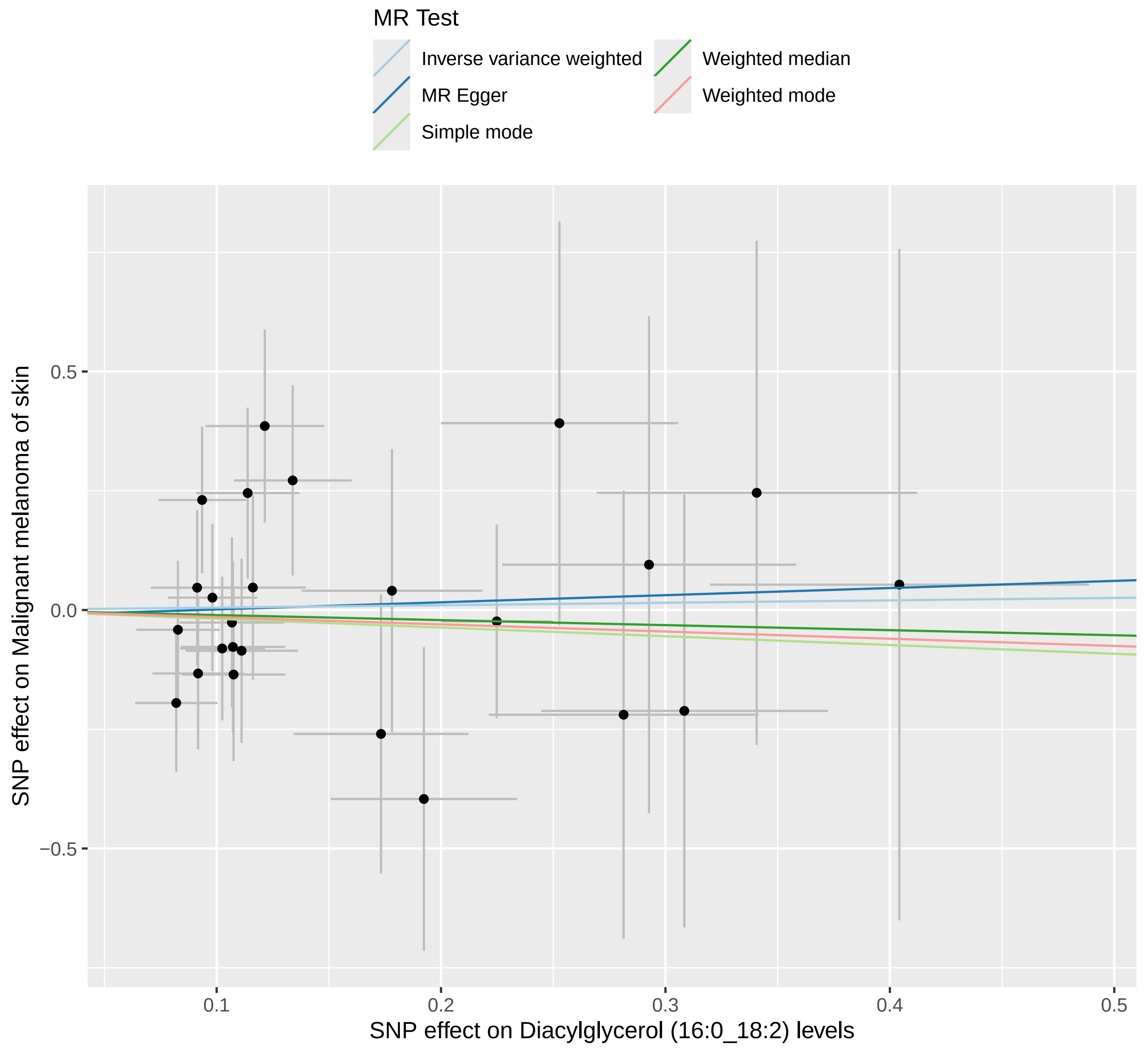
MR Test
Inverse variance weighted
MR Egger
Simple mode
Weighted median
Weighted mode
0.5
0.0
−0.5
SNP effect on Malignant melanoma of skin
0.1
0.2
0.3
0.4
0.5
SNP effect on Diacylglycerol (16:0_18:2) levels

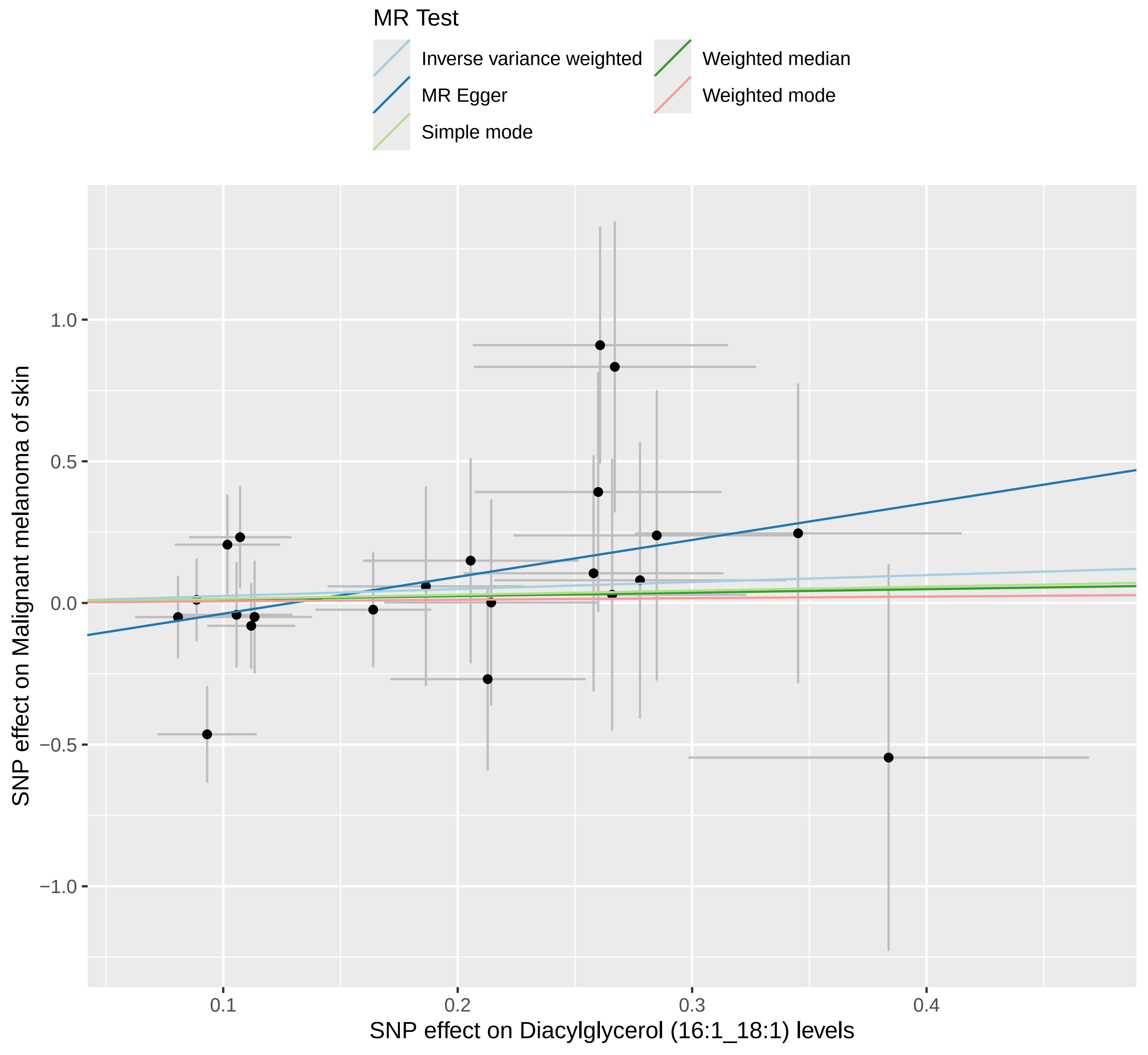
MR Test
Inverse variance weighted
MR Egger
Simple mode
Weighted median
Weighted mode
SNP effect on Malignant melanoma of skin
1.0
0.5
0.0
−0.5
−1.0
0.1
0.2
0.3
0.4
SNP effect on Diacylglycerol (16:1_18:1) levels

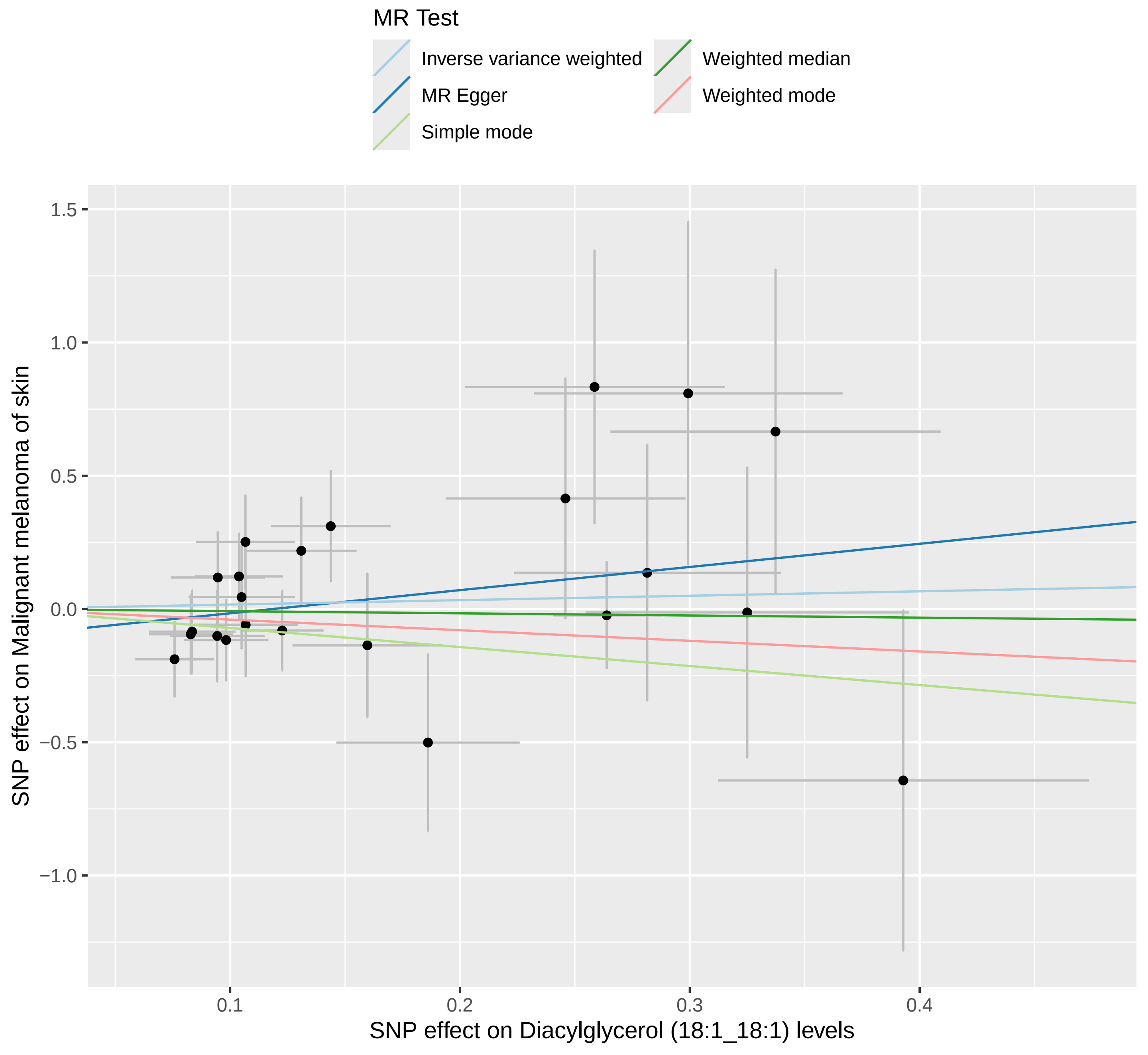
MR Test
Inverse variance weighted
MR Egger
Simple mode
Weighted median
Weighted mode
SNP effect on Malignant melanoma of skin
SNP effect on Diacylglycerol (18:1_18:1) levels
1.5
1.0
0.5
0.0
−0.5
−1.0
0.1
0.2
0.3
0.4

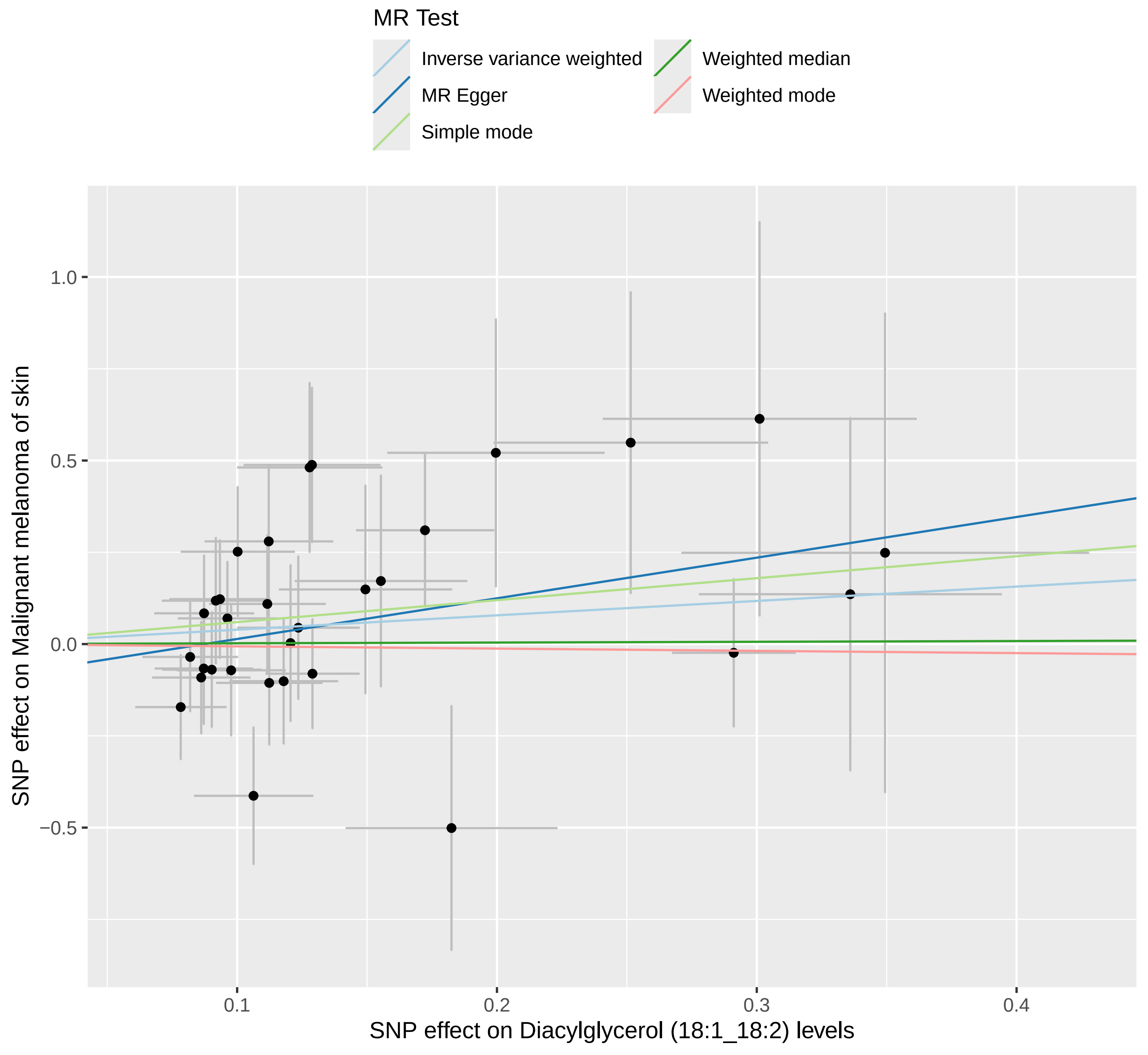
MR Test
Inverse variance weighted
Weighted median
MR Egger
Weighted mode
Simple mode
1.0
0.5
0.0
−0.5
0.1
0.2
0.3
0.4
SNP effect on Diacylglycerol (18:1_18:2) levels
SNP effect on Malignant melanoma of skin

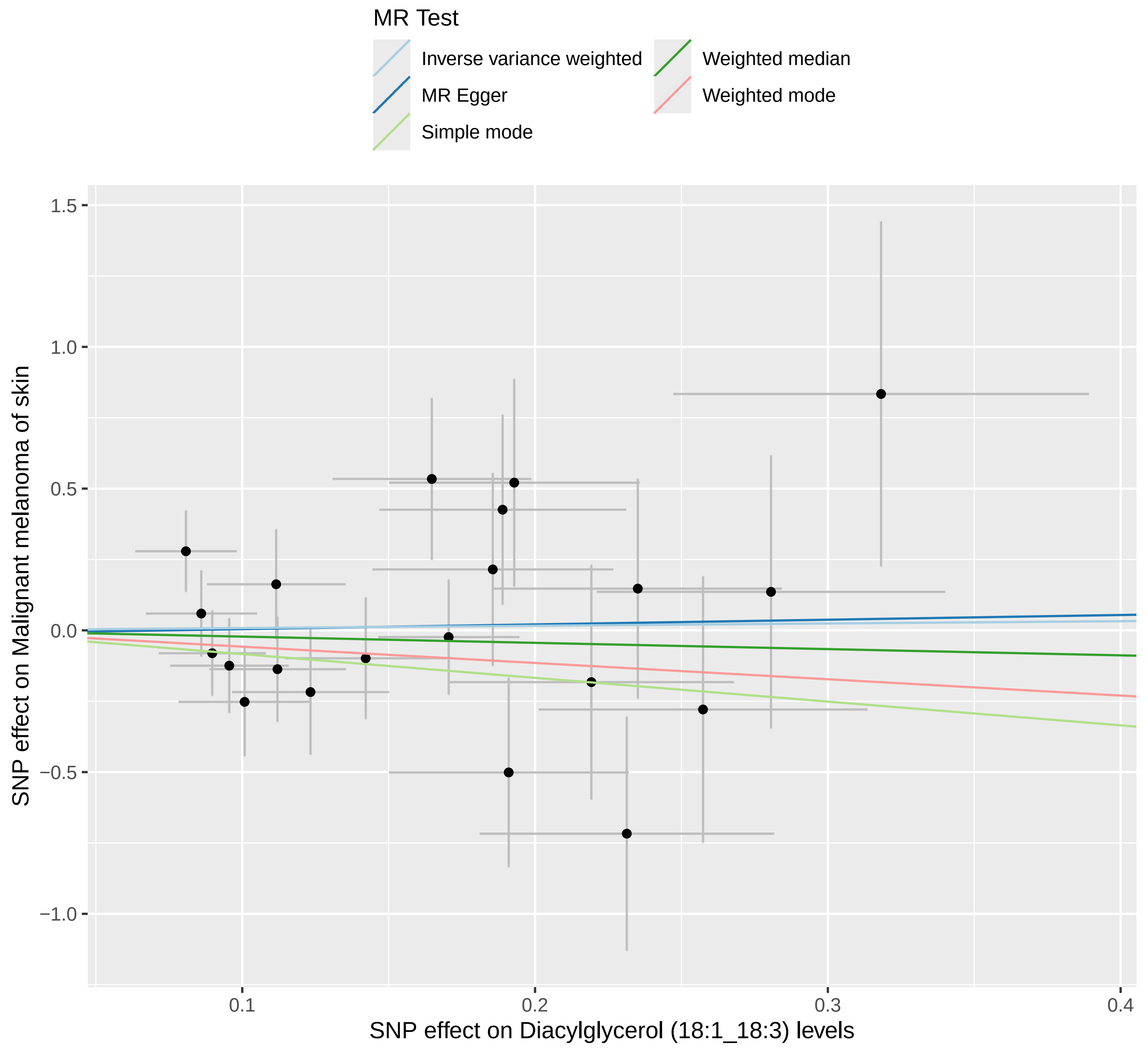
MR Test
Inverse variance weighted
MR Egger
Simple mode
Weighted median
Weighted mode
1.5
1.0
0.5
0.0
−0.5
−1.0
SNP effect on Malignant melanoma of skin
0.1
0.2
0.3
0.4
SNP effect on Diacylglycerol (18:1_18:3) levels

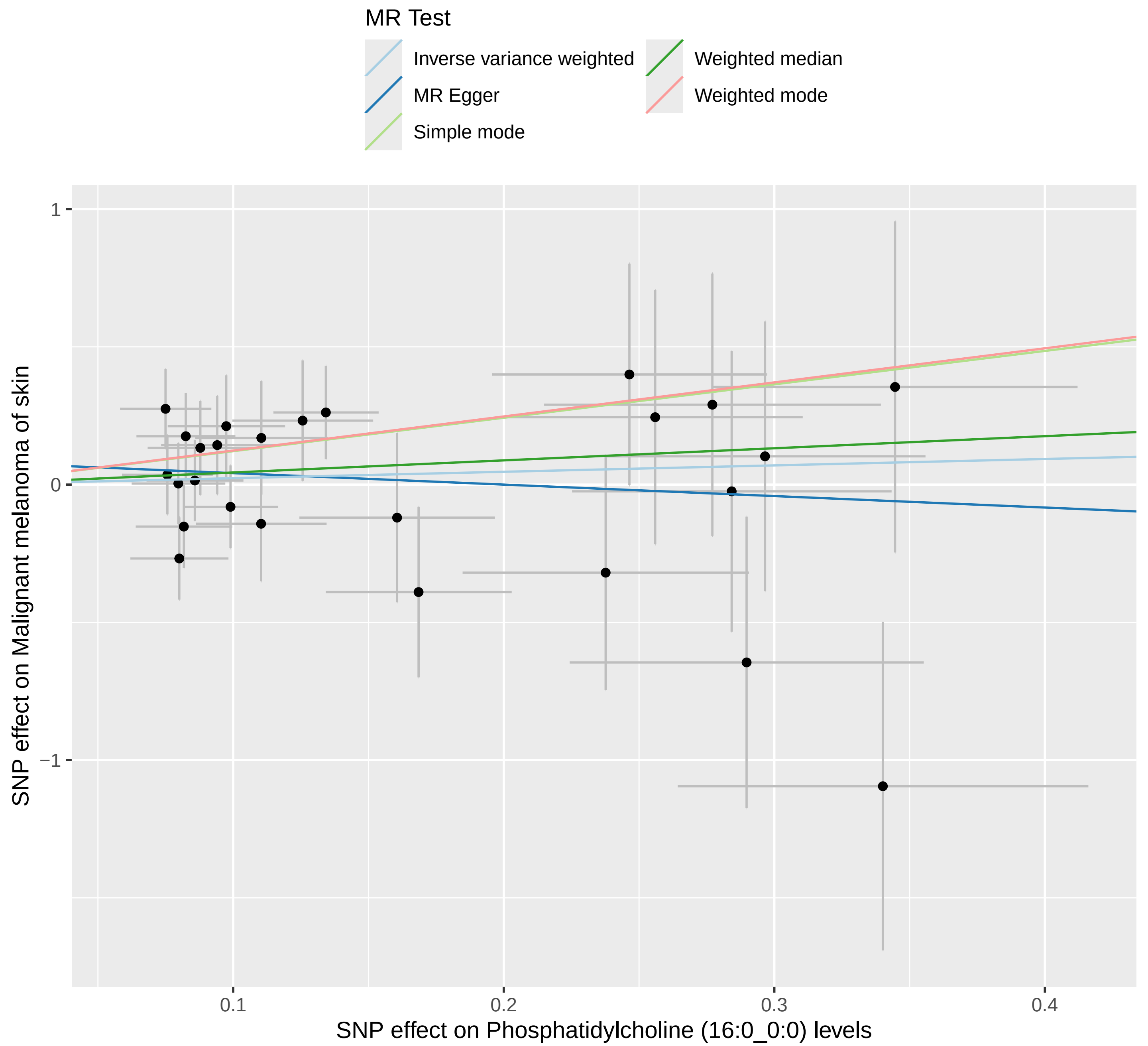

MR Test
Inverse variance weighted
MR Egger
Simple mode
Weighted median
Weighted mode
1
0
−1
0.1
0.2
0.3
0.4
SNP effect on Malignant melanoma of skin
SNP effect on Phosphatidylcholine (16:0_0:0) levels

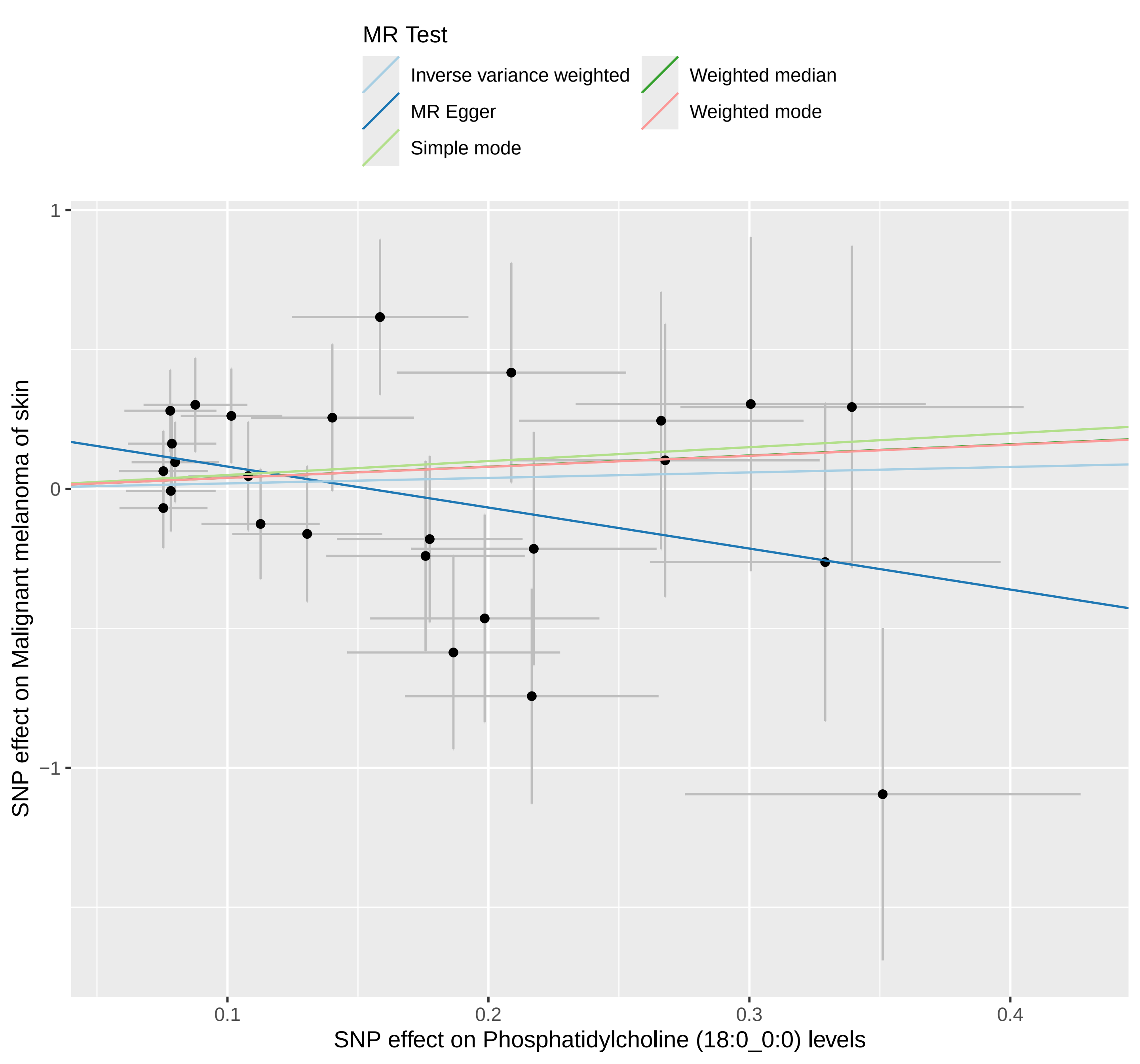

MR Test
Inverse variance weighted
MR Egger
Simple mode
Weighted median
Weighted mode
1
0
−1
0.1
0.2
0.3
0.4
SNP effect on Malignant melanoma of skin
SNP effect on Phosphatidylcholine (18:0_0:0) levels

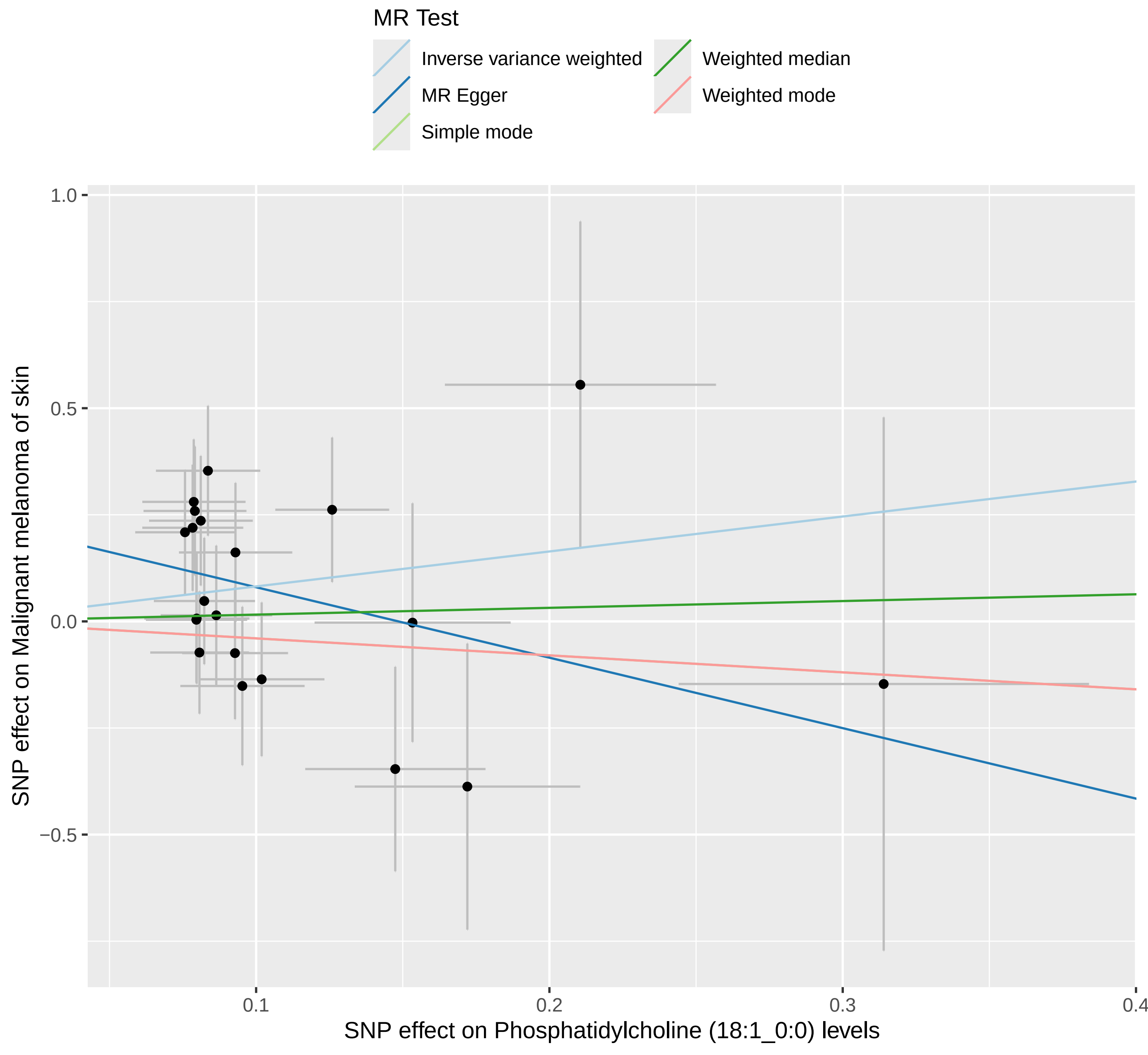
MR Test
Inverse variance weighted
MR Egger
Simple mode
Weighted median
Weighted mode
1.0
0.5
0.0
−0.5
0.1
0.2
0.3
0.4
SNP effect on Malignant melanoma of skin
SNP effect on Phosphatidylcholine (18:1_0:0) levels

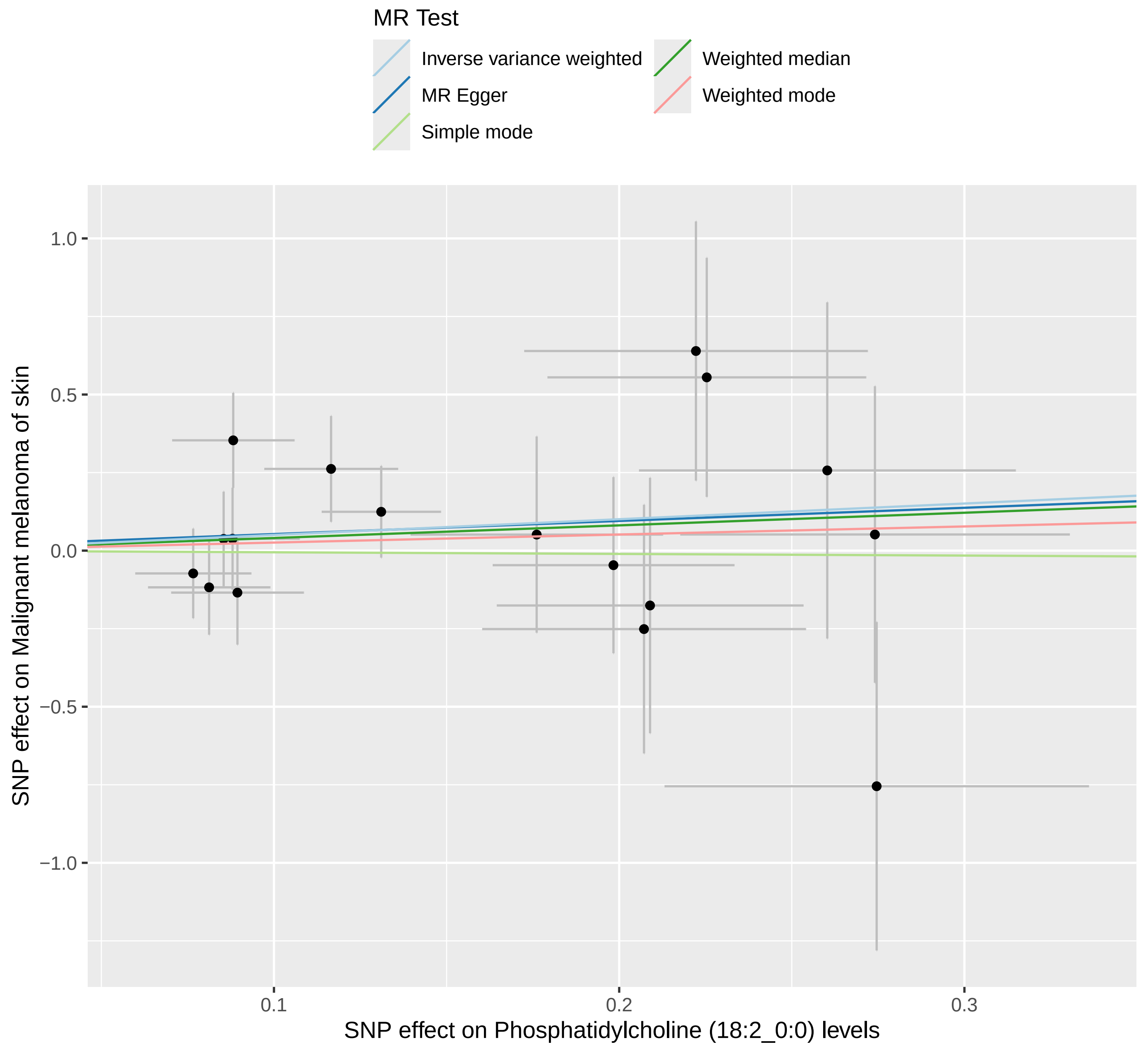
MR Test
Inverse variance weighted
MR Egger
Simple mode
Weighted median
Weighted mode
1.0
0.5
0.0
−0.5
−1.0
0.1
0.2
0.3
SNP effect on Malignant melanoma of skin
SNP effect on Phosphatidylcholine (18:2_0:0) levels

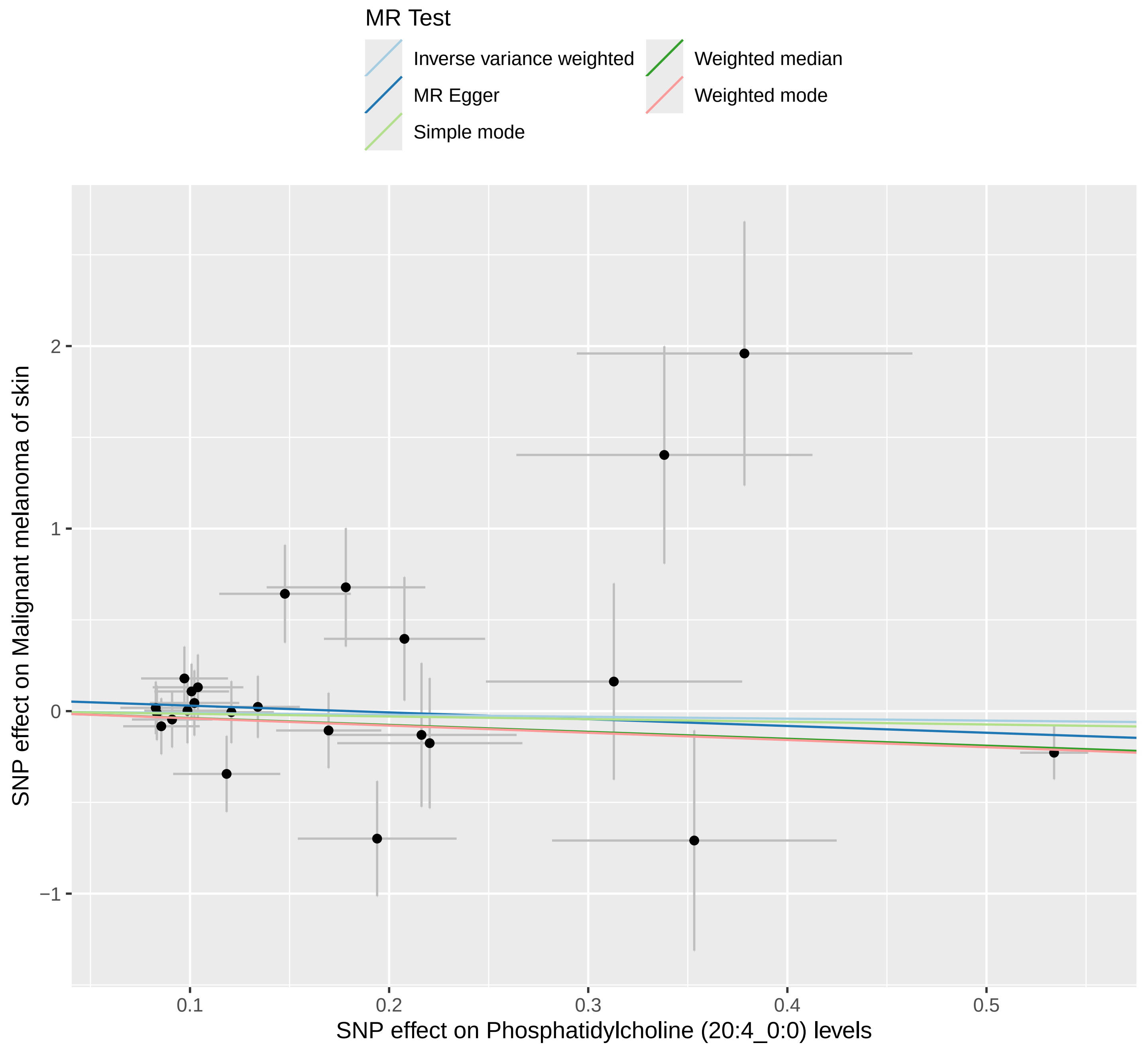
MR Test
Inverse variance weighted
MR Egger
Simple mode
Weighted median
Weighted mode
SNP effect on Malignant melanoma of skin
2
1
0
−1
0.1
0.2
0.3
0.4
0.5
SNP effect on Phosphatidylcholine (20:4_0:0) levels

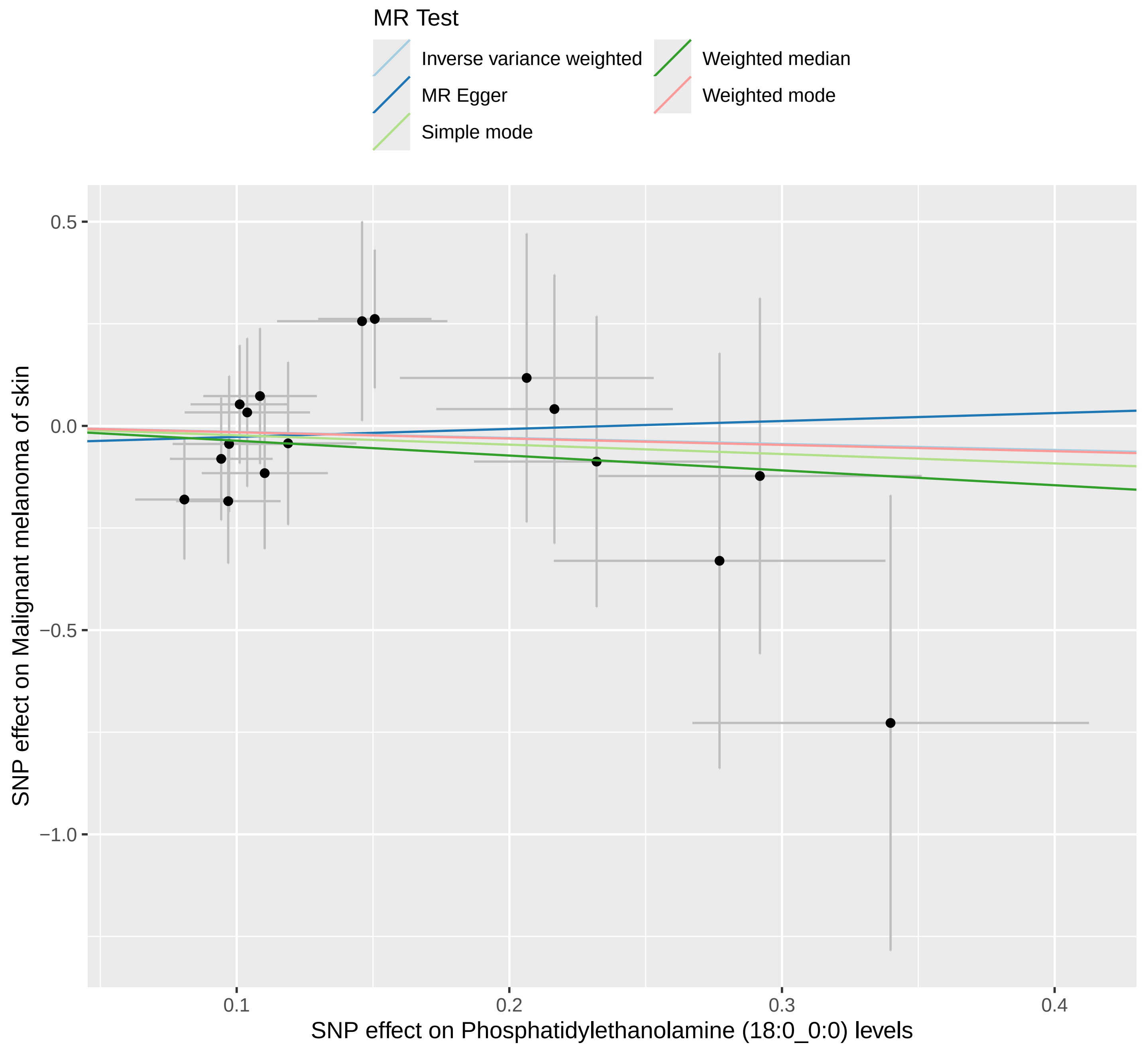
MR Test
Inverse variance weighted
MR Egger
Simple mode
Weighted median
Weighted mode
0.5
0.0
−0.5
−1.0
0.1
0.2
0.3
0.4
SNP effect on Malignant melanoma of skin
SNP effect on Phosphatidylethanolamine (18:0_0:0) levels

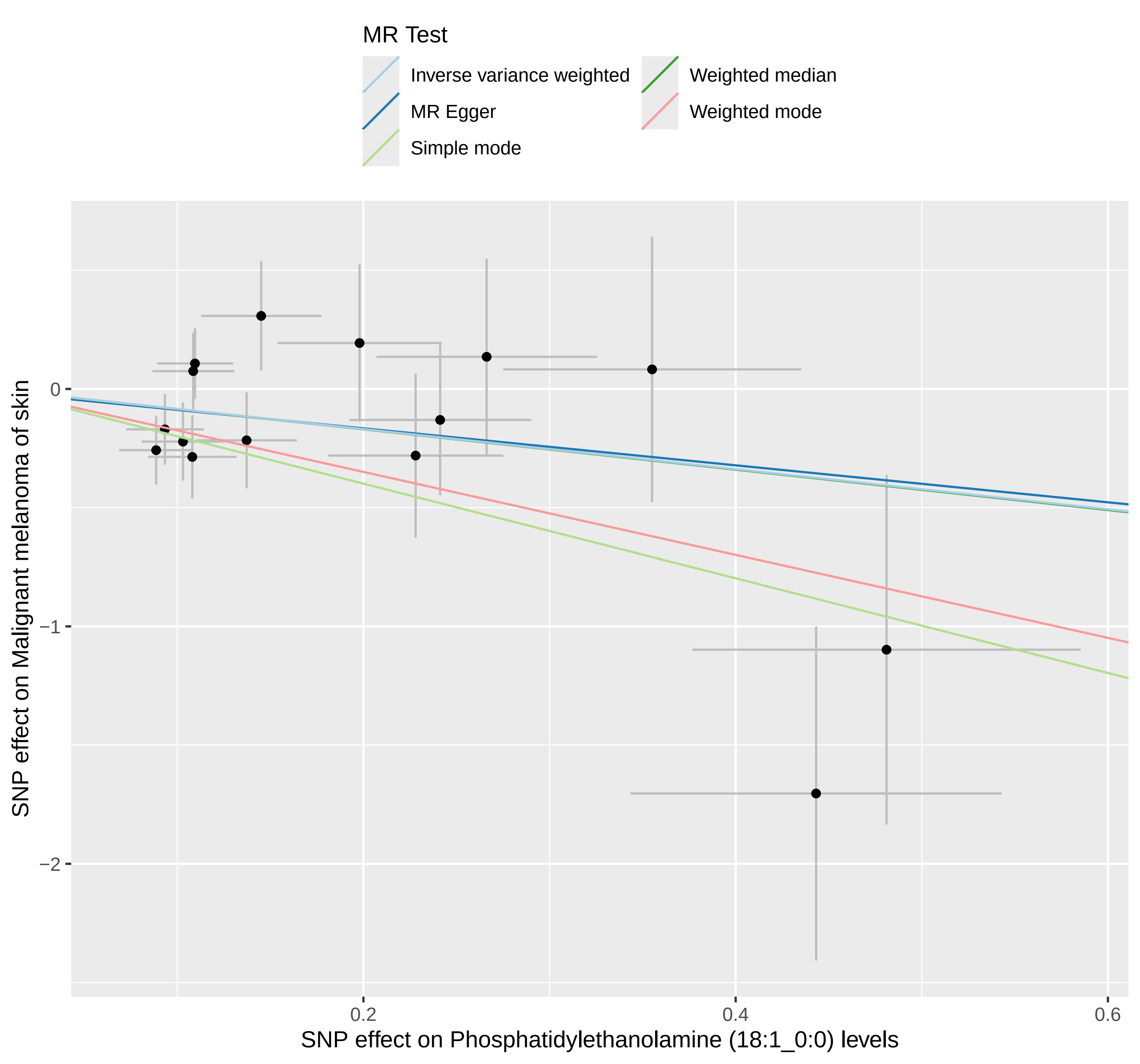

MR Test
Inverse variance weighted
MR Egger
Simple mode
Weighted median
Weighted mode
0
−1
−2
0.2
0.4
0.6
SNP effect on Malignant melanoma of skin
SNP effect on Phosphatidylethanolamine (18:1_0:0) levels

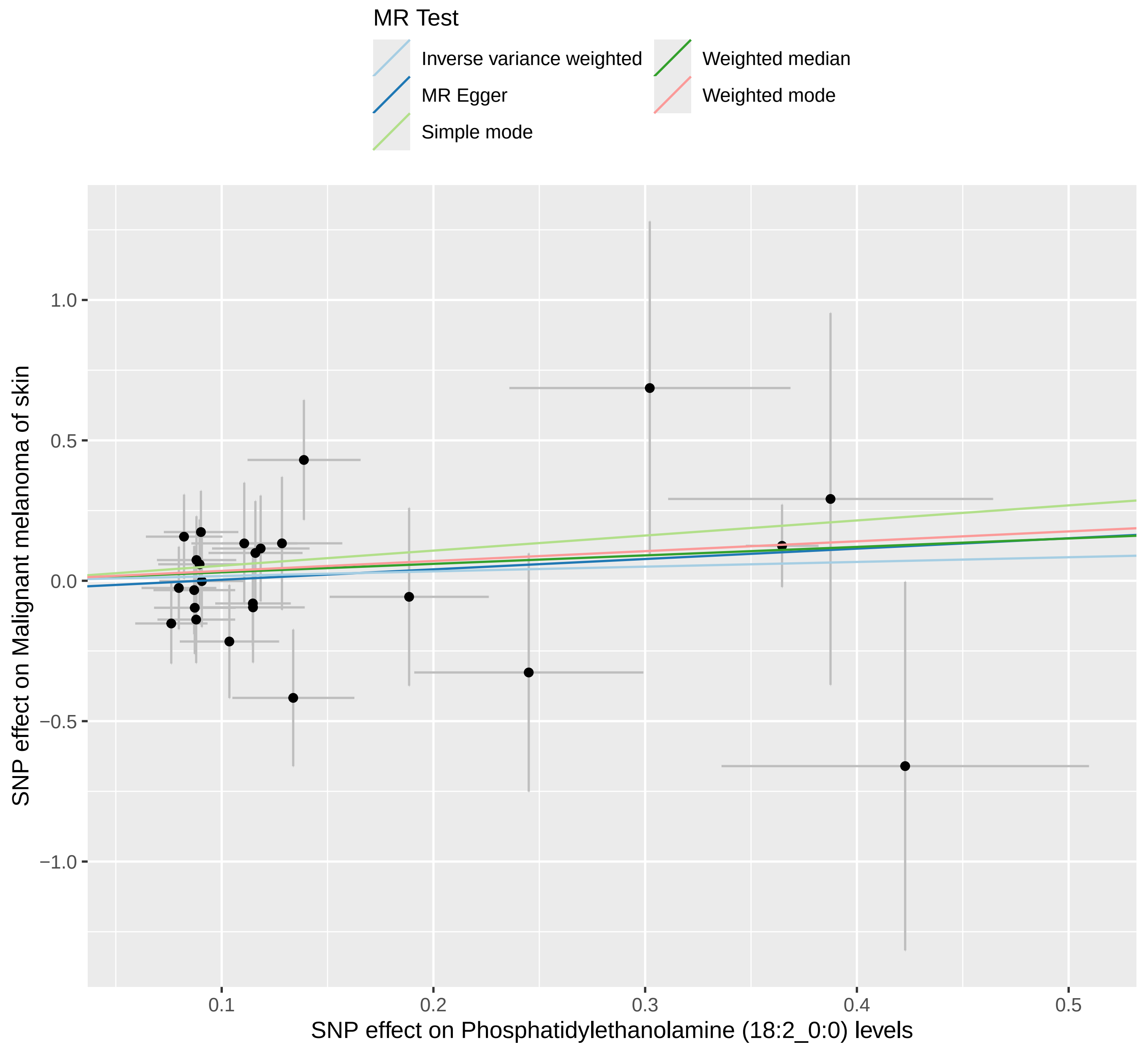

MR Test
Inverse variance weighted
MR Egger
Simple mode
Weighted median
Weighted mode
SNP effect on Malignant melanoma of skin
1.0
0.5
0.0
−0.5
−1.0
0.1
0.2
0.3
0.4
0.5
SNP effect on Phosphatidylethanolamine (18:2_0:0) levels

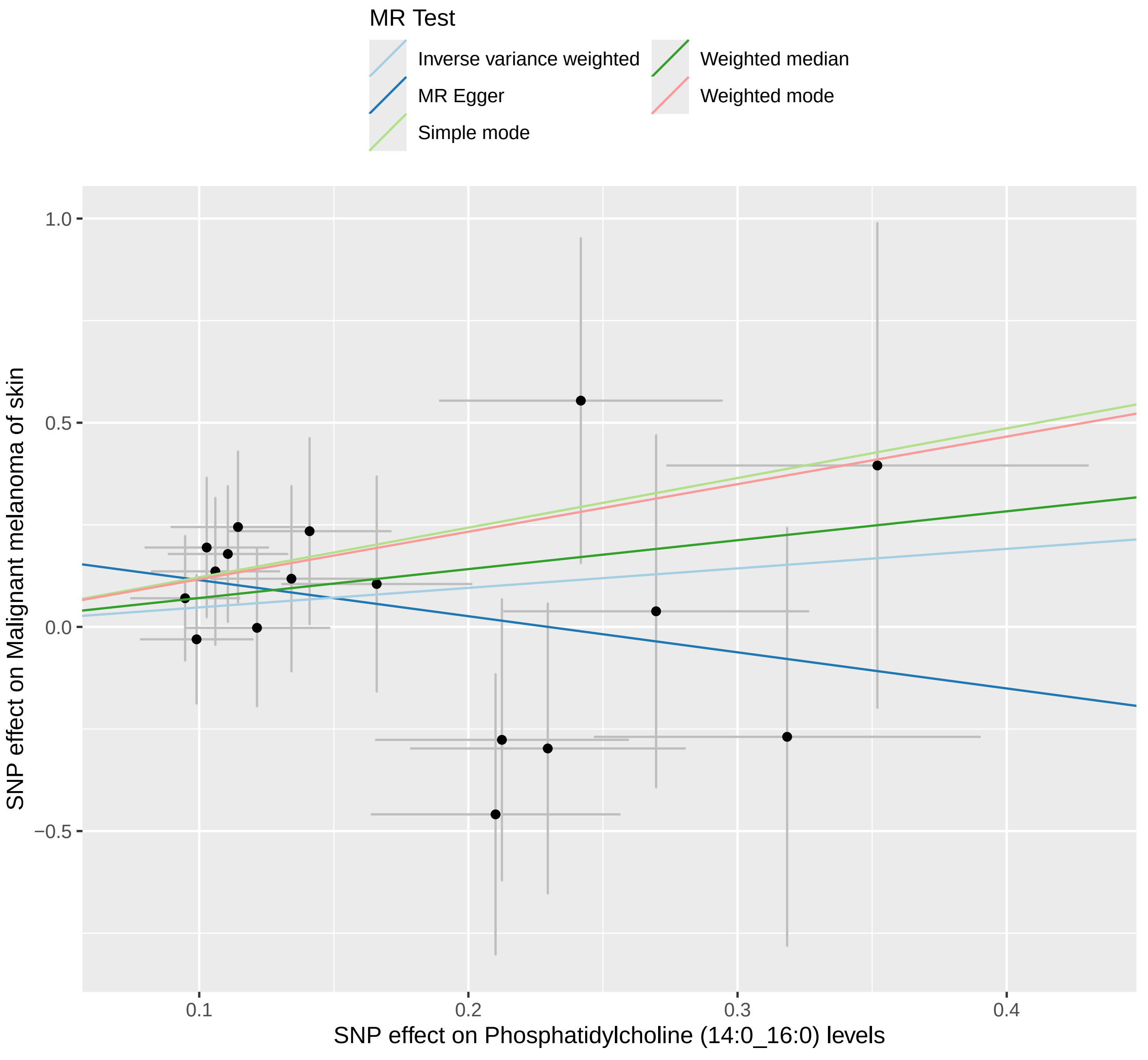
MR Test
Inverse variance weighted
MR Egger
Simple mode
Weighted median
Weighted mode
1.0
0.5
0.0
−0.5
SNP effect on Malignant melanoma of skin
0.1
0.2
0.3
0.4
SNP effect on Phosphatidylcholine (14:0_16:0) levels

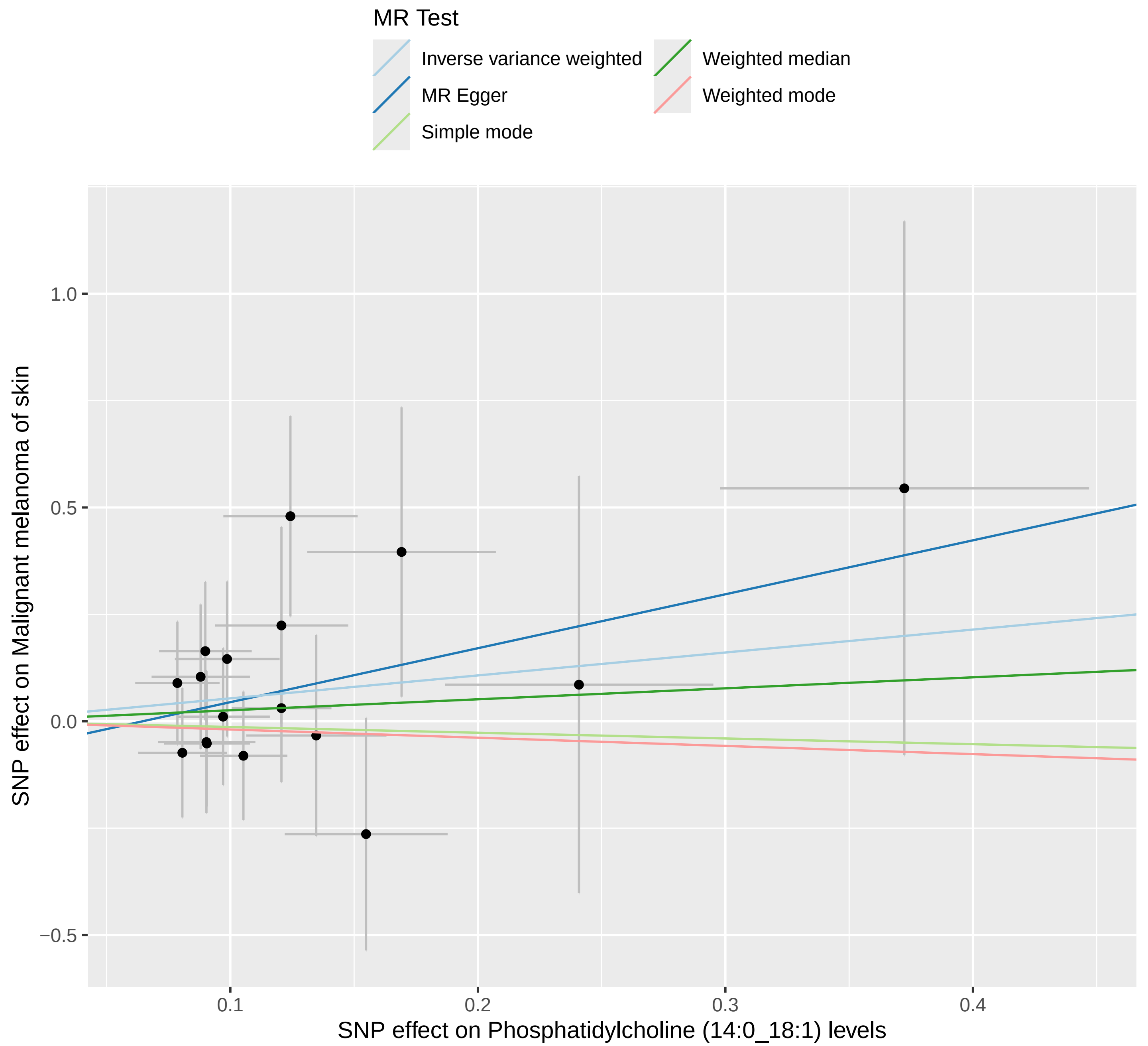

MR Test
Inverse variance weighted
MR Egger
Simple mode
Weighted median
Weighted mode
1.0
0.5
0.0
−0.5
0.1
0.2
0.3
0.4
SNP effect on Malignant melanoma of skin
SNP effect on Phosphatidylcholine (14:0_18:1) levels

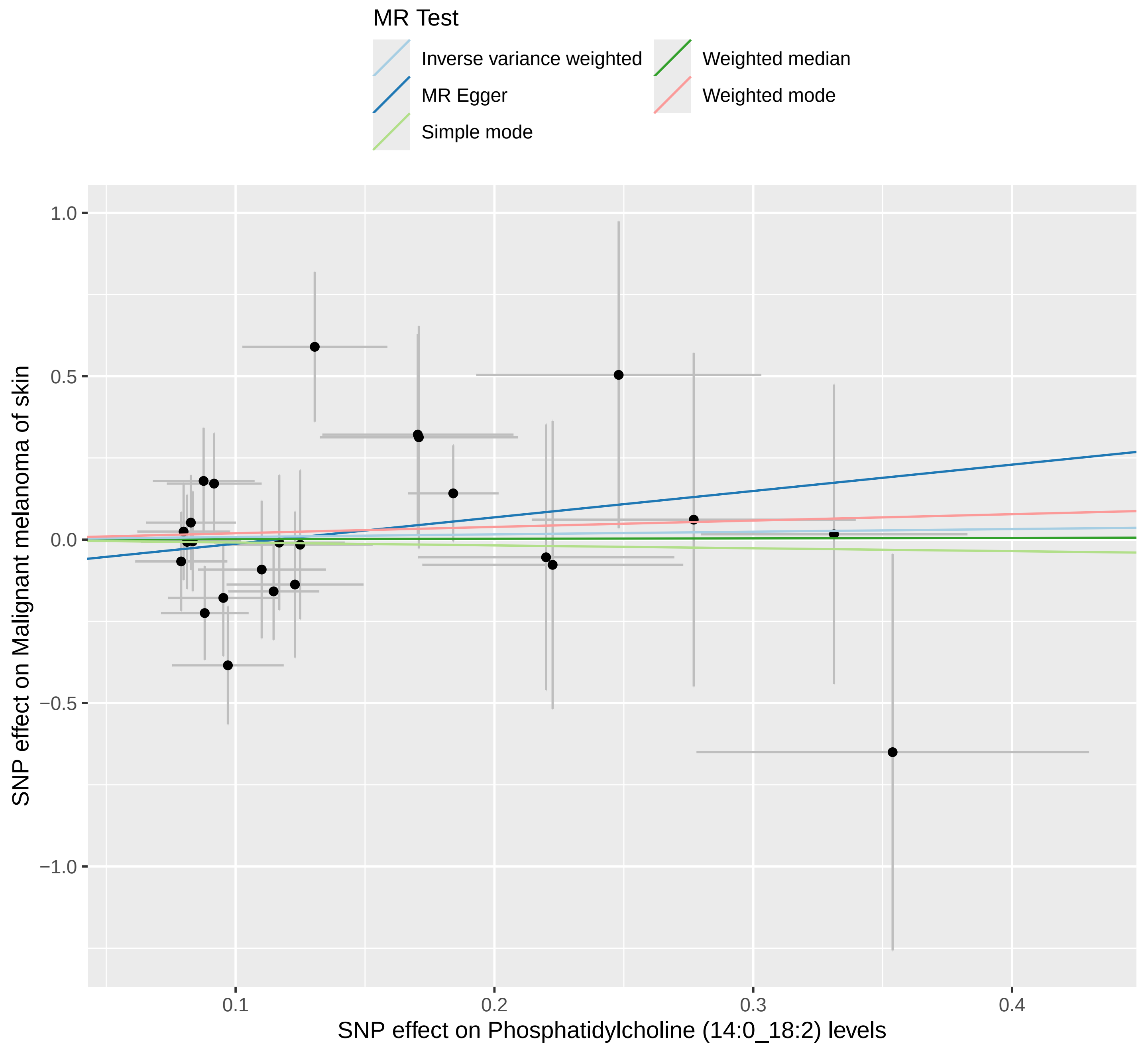

MR Test
Inverse variance weighted
MR Egger
Simple mode
Weighted median
Weighted mode
1.0
0.5
0.0
−0.5
−1.0
0.1
0.2
0.3
0.4
SNP effect on Malignant melanoma of skin
SNP effect on Phosphatidylcholine (14:0_18:2) levels

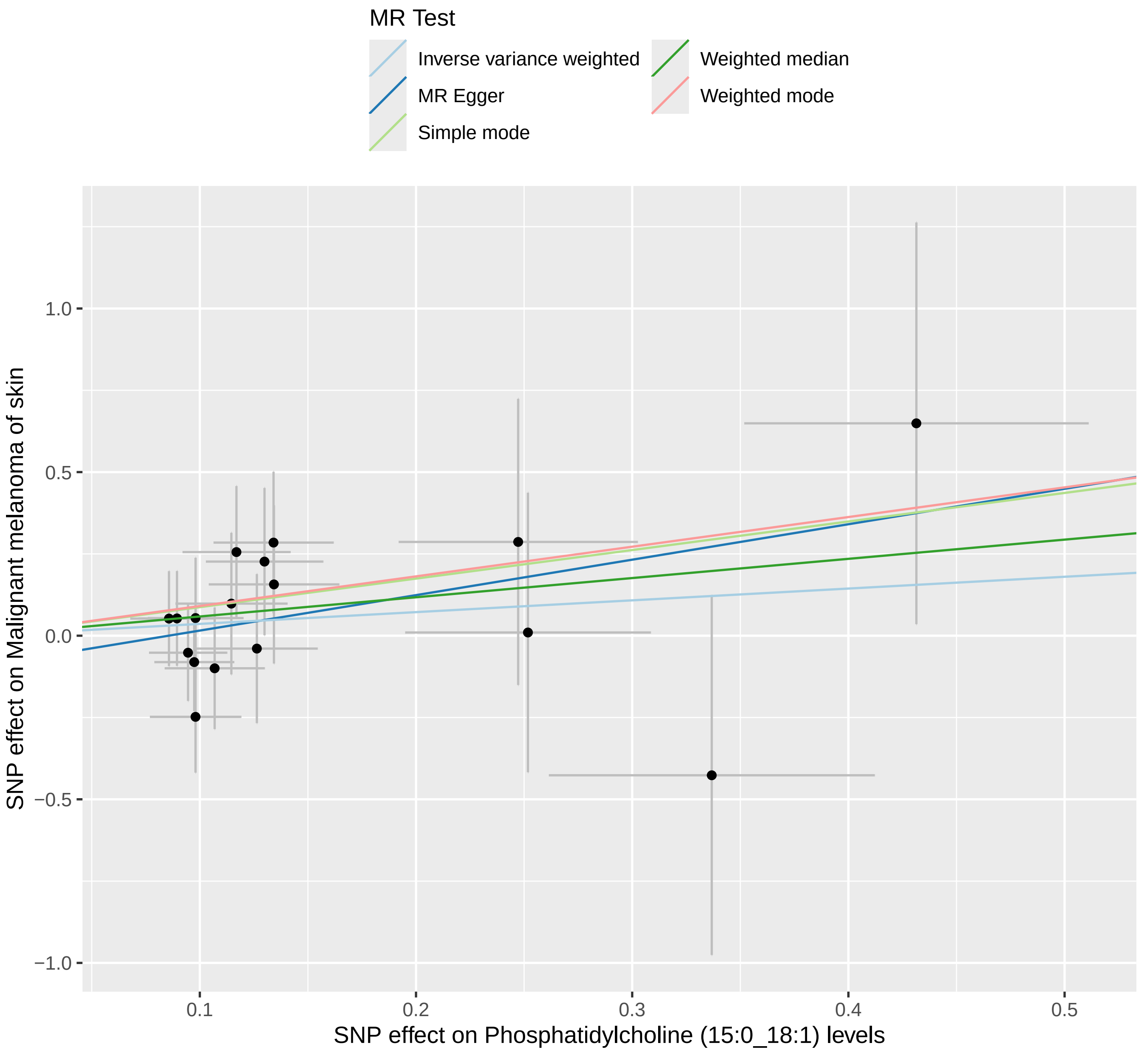

MR Test
Inverse variance weighted
MR Egger
Simple mode
Weighted median
Weighted mode
1.0
0.5
0.0
−0.5
−1.0
0.1
0.2
0.3
0.4
0.5
SNP effect on Malignant melanoma of skin
SNP effect on Phosphatidylcholine (15:0_18:1) levels

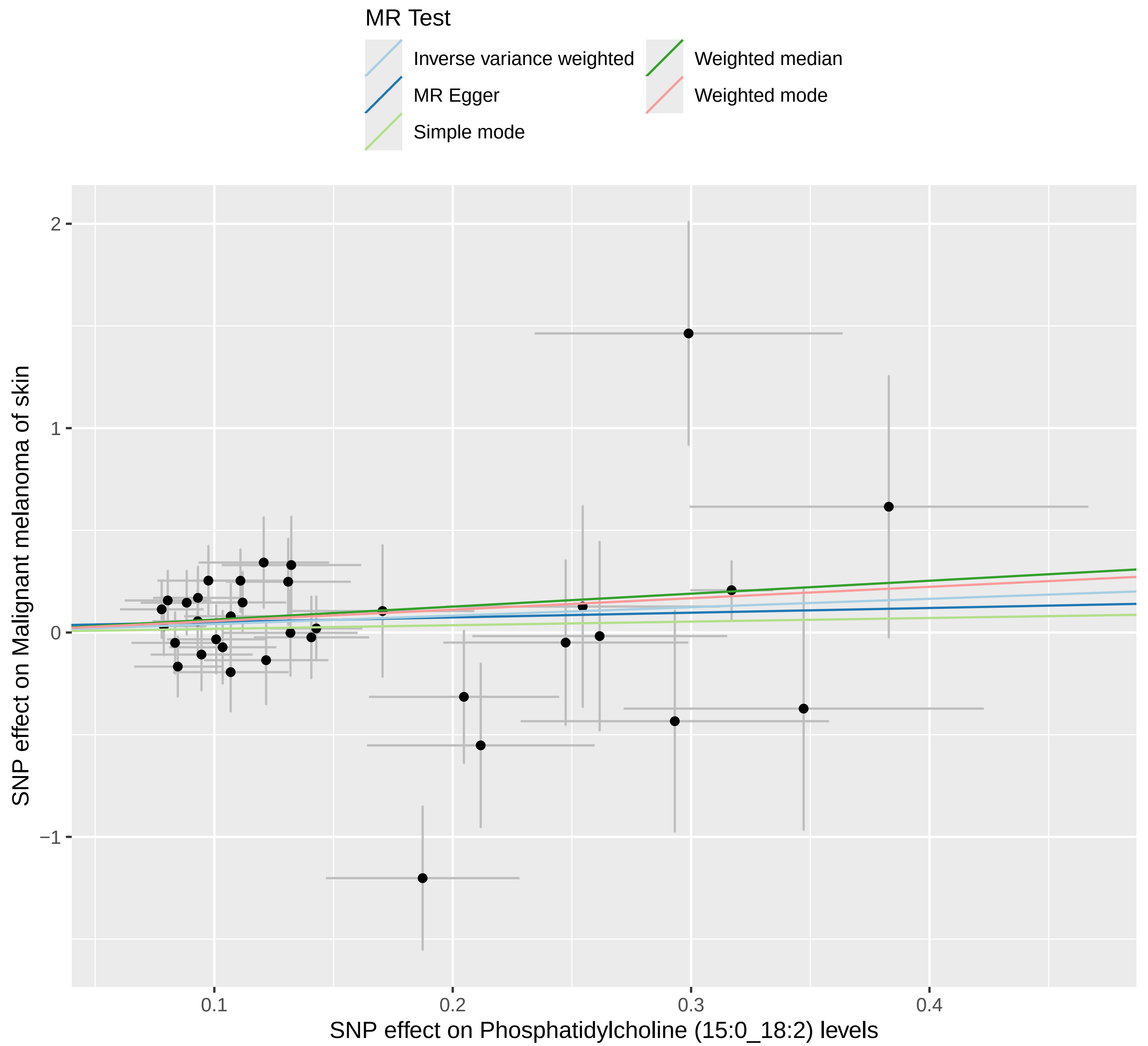
MR Test
Inverse variance weighted
MR Egger
Simple mode
Weighted median
Weighted mode
2
1
0
−1
0.1
0.2
0.3
0.4
SNP effect on Malignant melanoma of skin
SNP effect on Phosphatidylcholine (15:0_18:2) levels

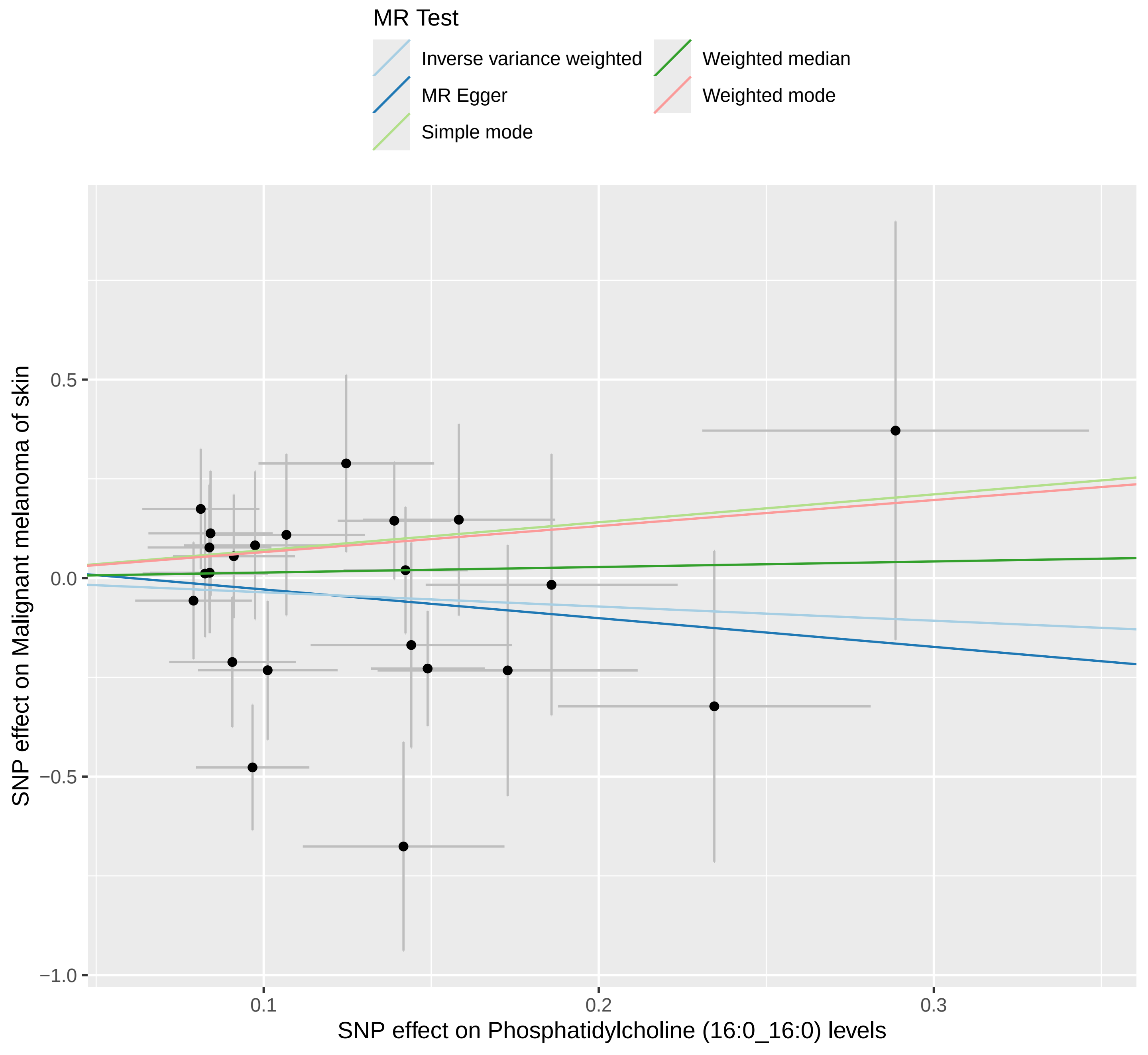

MR Test
Inverse variance weighted
MR Egger
Simple mode
Weighted median
Weighted mode
0.5
0.0
−0.5
−1.0
0.1
0.2
0.3
SNP effect on Malignant melanoma of skin
SNP effect on Phosphatidylcholine (16:0_16:0) levels

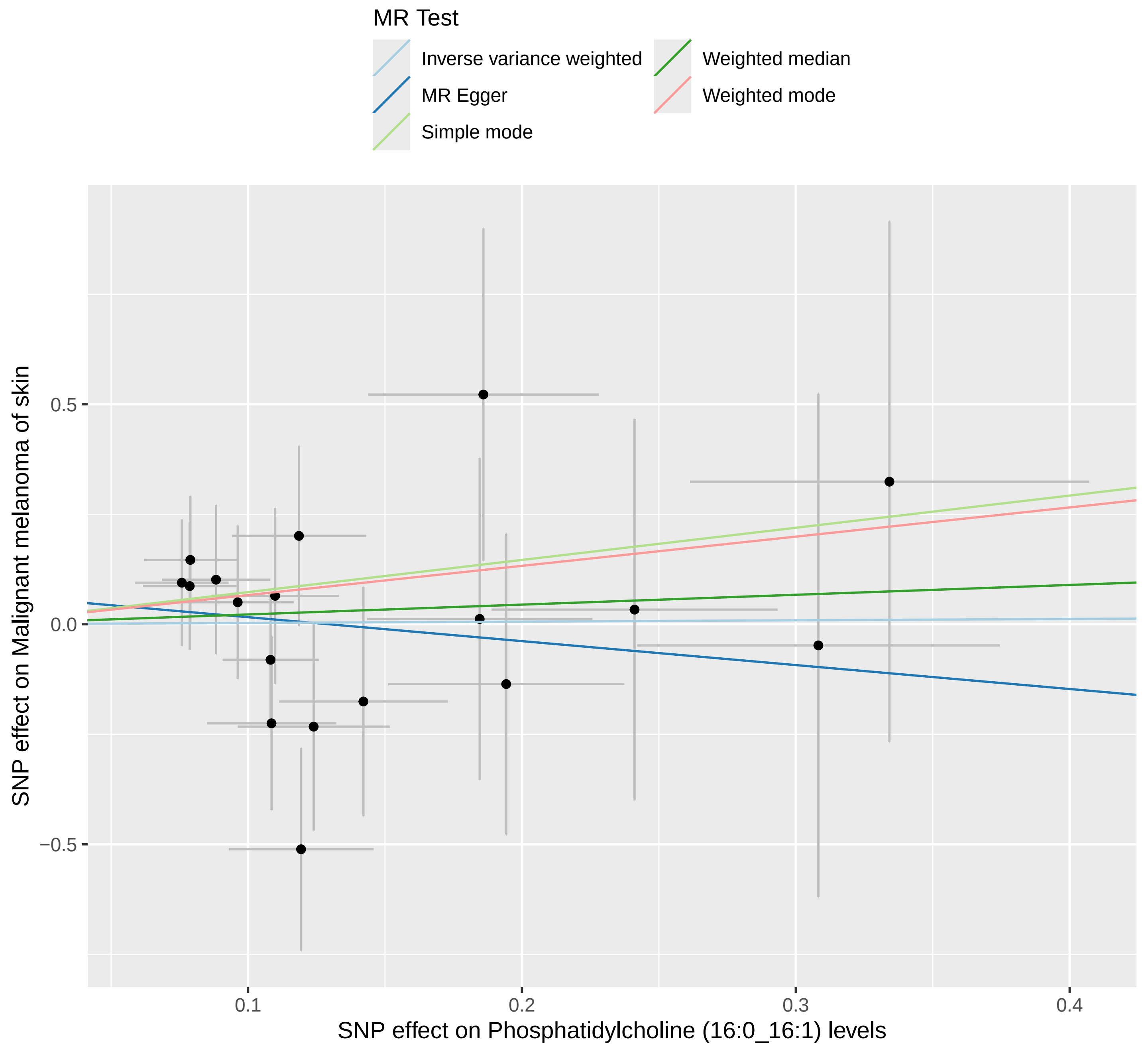
MR Test
Inverse variance weighted
MR Egger
Simple mode
Weighted median
Weighted mode
SNP effect on Malignant melanoma of skin
0.5
0.0
−0.5
0.1
0.2
0.3
0.4
SNP effect on Phosphatidylcholine (16:0_16:1) levels

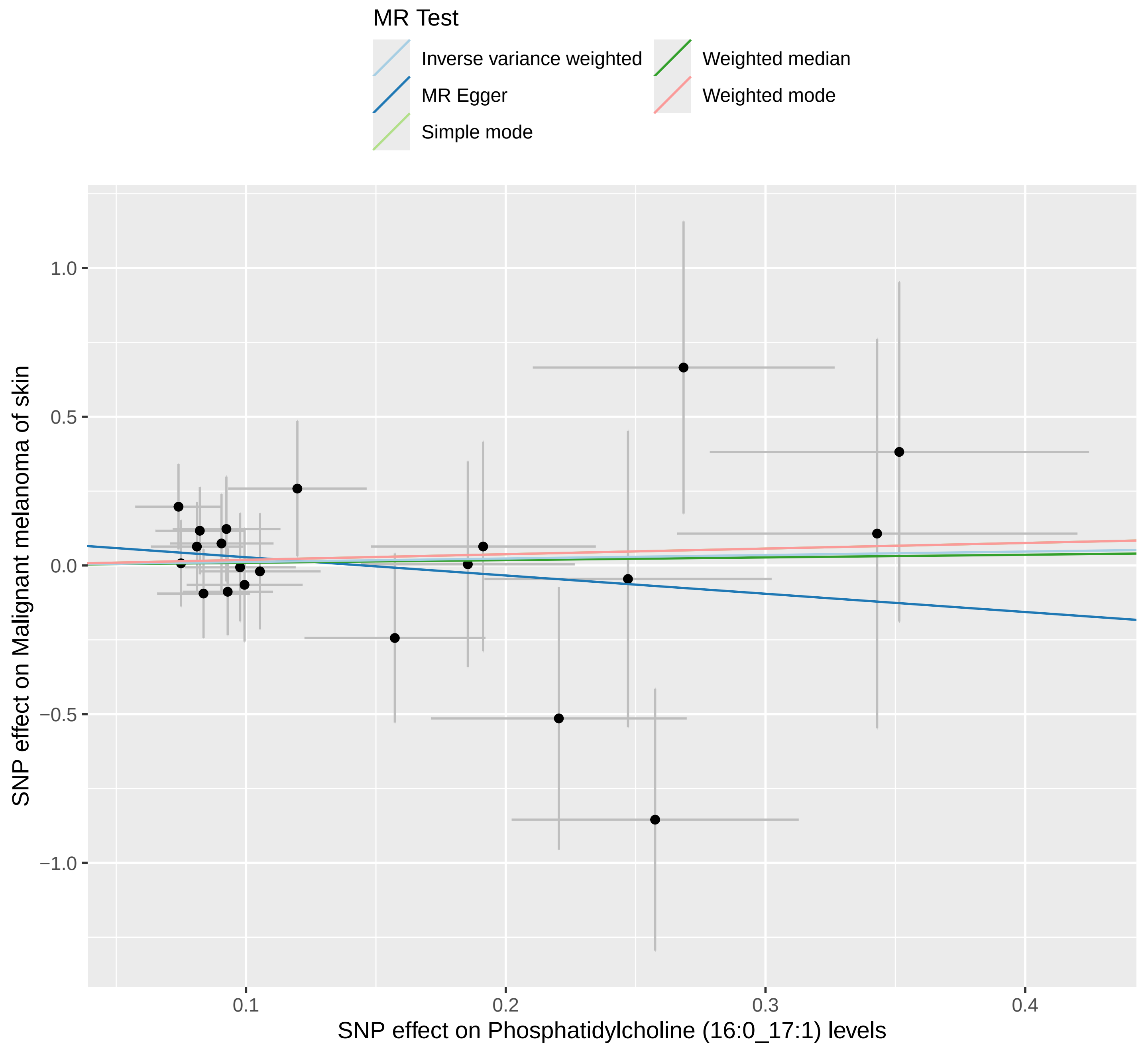
MR Test
Inverse variance weighted
MR Egger
Simple mode
Weighted median
Weighted mode
1.0
0.5
0.0
−0.5
−1.0
0.1
0.2
0.3
0.4
SNP effect on Malignant melanoma of skin
SNP effect on Phosphatidylcholine (16:0_17:1) levels

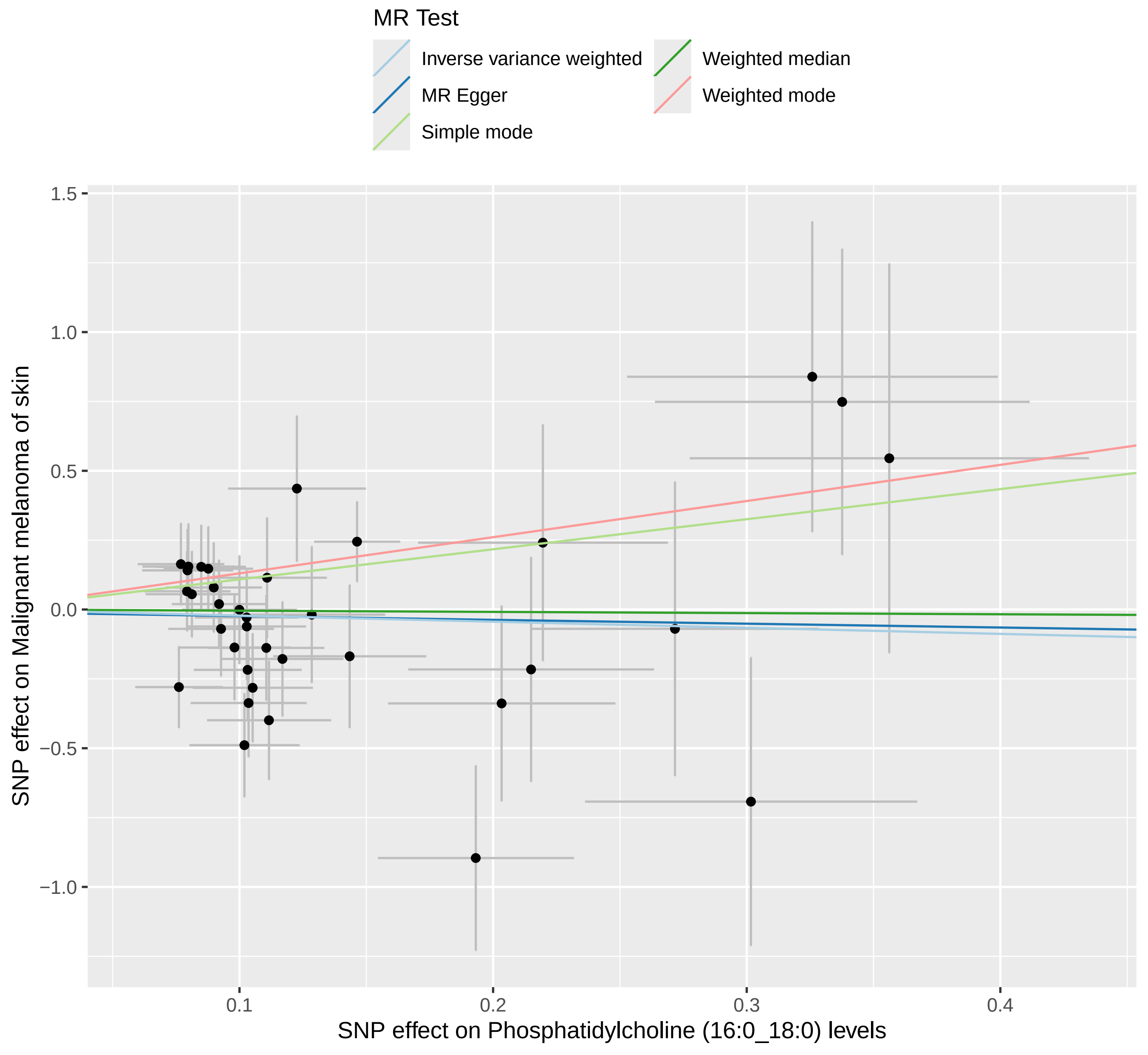
MR Test
Inverse variance weighted
MR Egger
Simple mode
Weighted median
Weighted mode
1.5
1.0
0.5
0.0
−0.5
−1.0
SNP effect on Malignant melanoma of skin
0.1
0.2
0.3
0.4
SNP effect on Phosphatidylcholine (16:0_18:0) levels

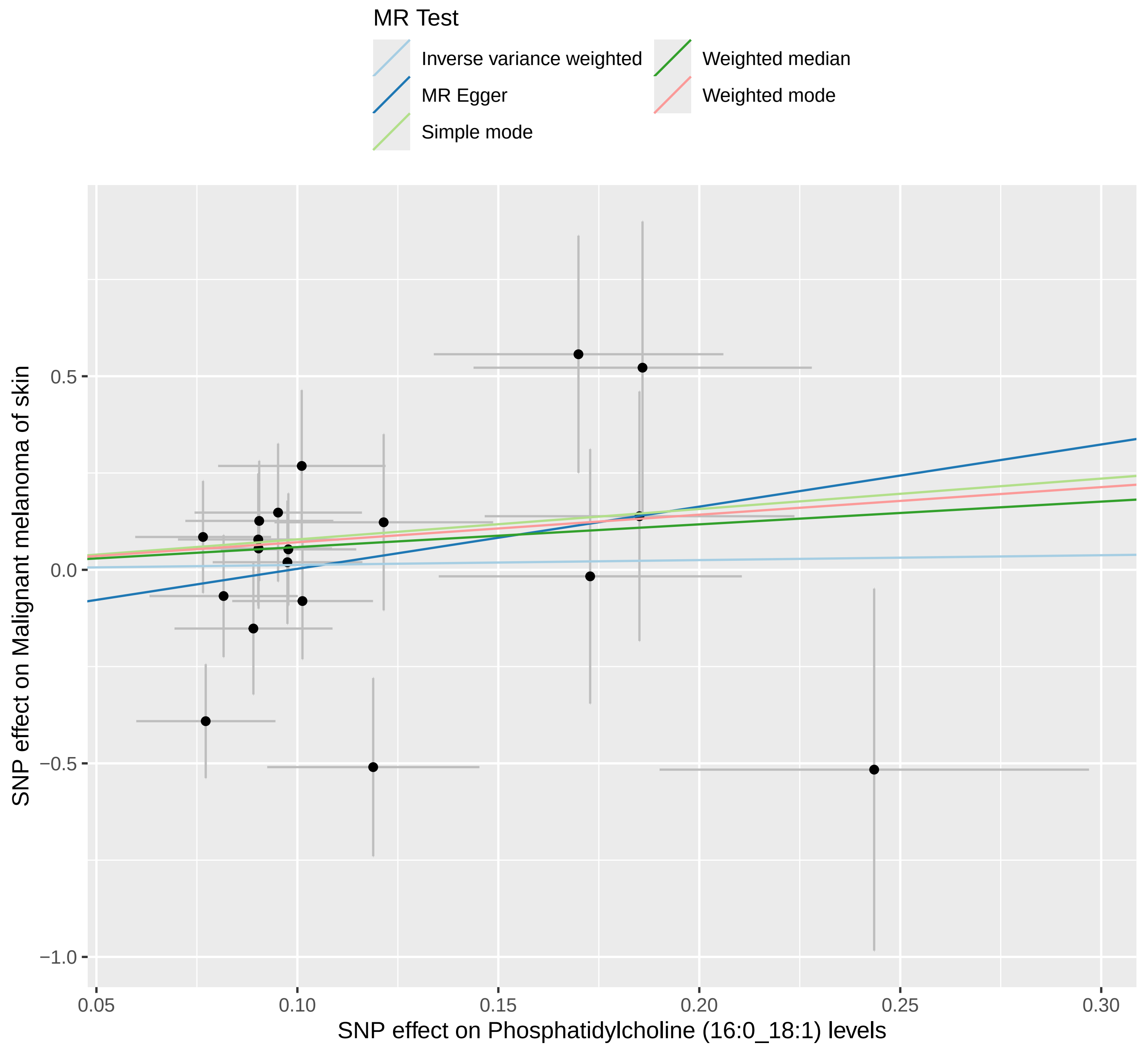
MR Test
Inverse variance weighted
MR Egger
Simple mode
Weighted median
Weighted mode
0.5
0.0
−0.5
−1.0
SNP effect on Malignant melanoma of skin
0.05
0.10
0.15
0.20
0.25
0.30
SNP effect on Phosphatidylcholine (16:0_18:1) levels

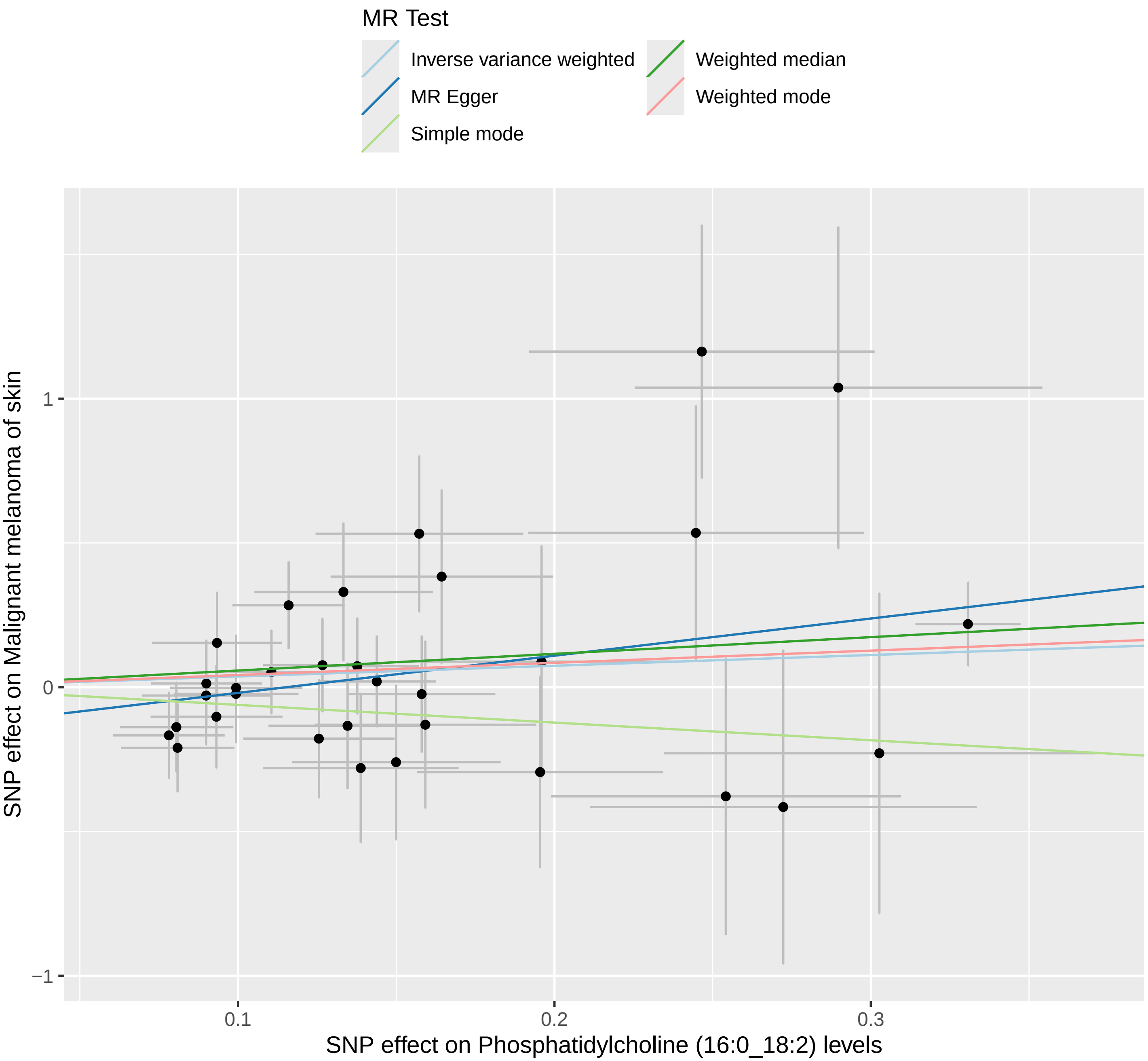

MR Test
Inverse variance weighted
MR Egger
Simple mode
Weighted median
Weighted mode
SNP effect on Malignant melanoma of skin
1
0
−1
0.1
0.2
0.3
SNP effect on Phosphatidylcholine (16:0_18:2) levels

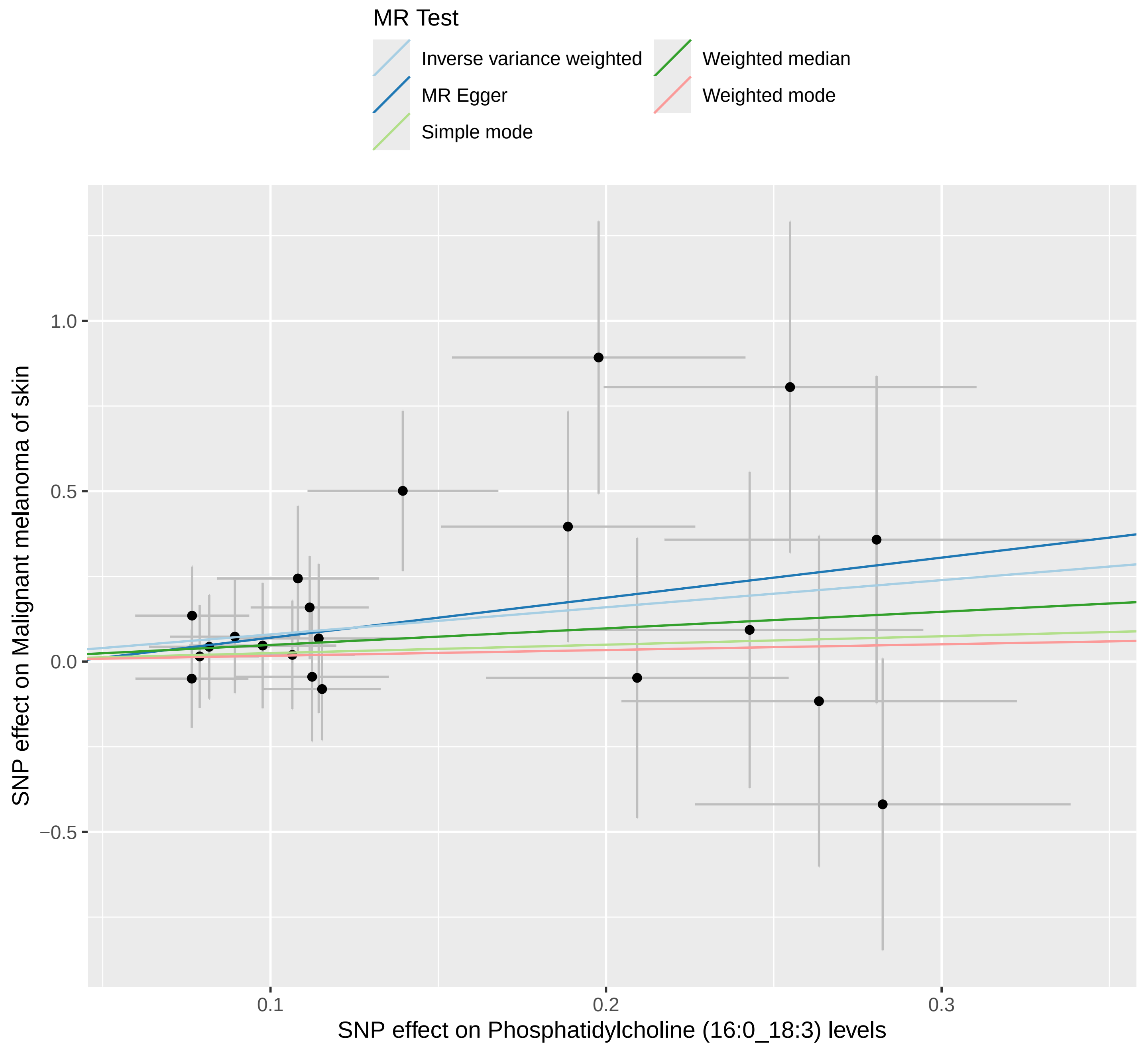

MR Test
Inverse variance weighted
MR Egger
Simple mode
Weighted median
Weighted mode
1.0
0.5
0.0
−0.5
0.1
0.2
0.3
SNP effect on Malignant melanoma of skin
SNP effect on Phosphatidylcholine (16:0_18:3) levels

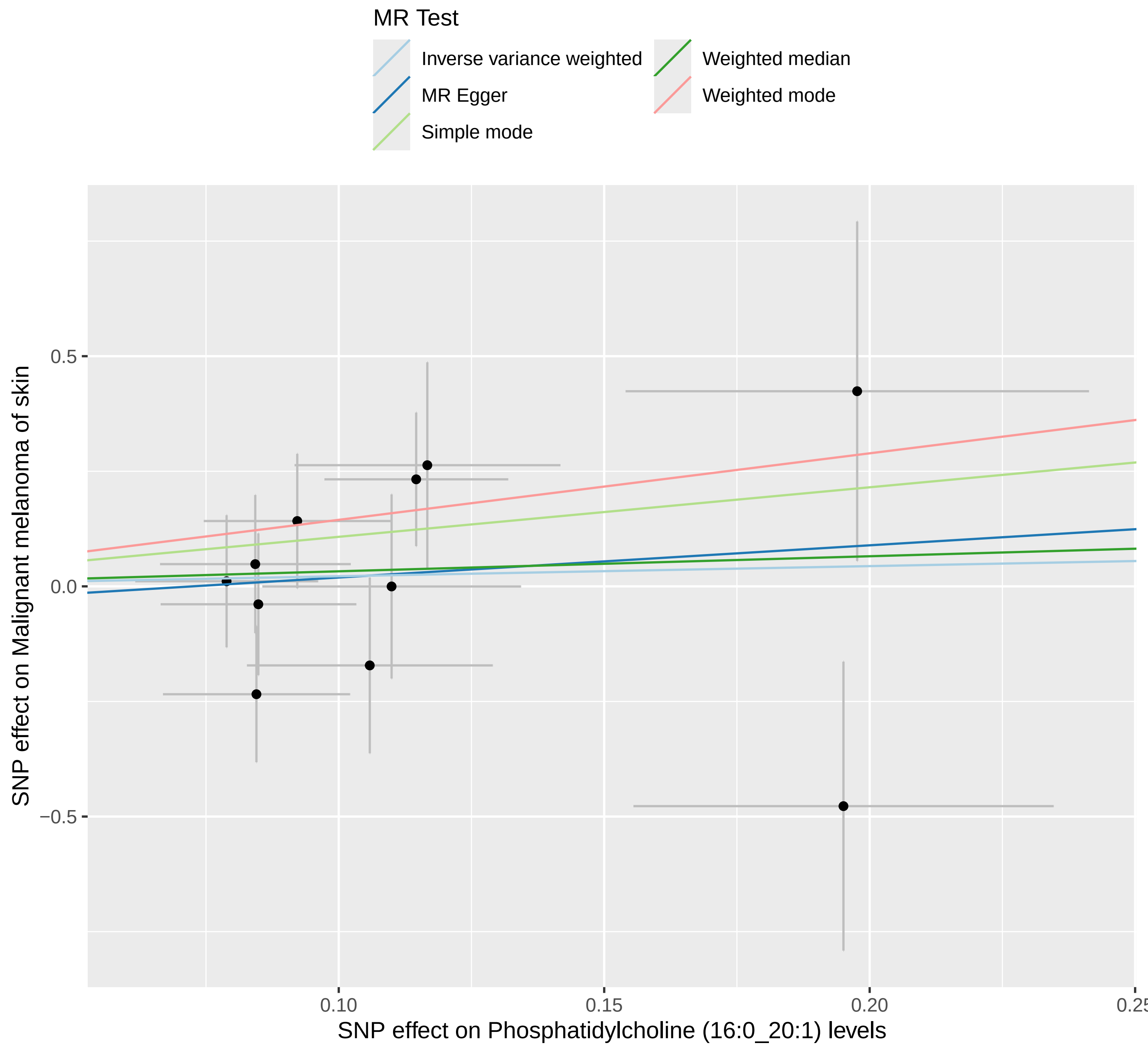
MR Test
Inverse variance weighted
MR Egger
Simple mode
Weighted median
Weighted mode
0.5
0.0
−0.5
SNP effect on Malignant melanoma of skin
0.10
0.15
0.20
0.25
SNP effect on Phosphatidylcholine (16:0_20:1) levels

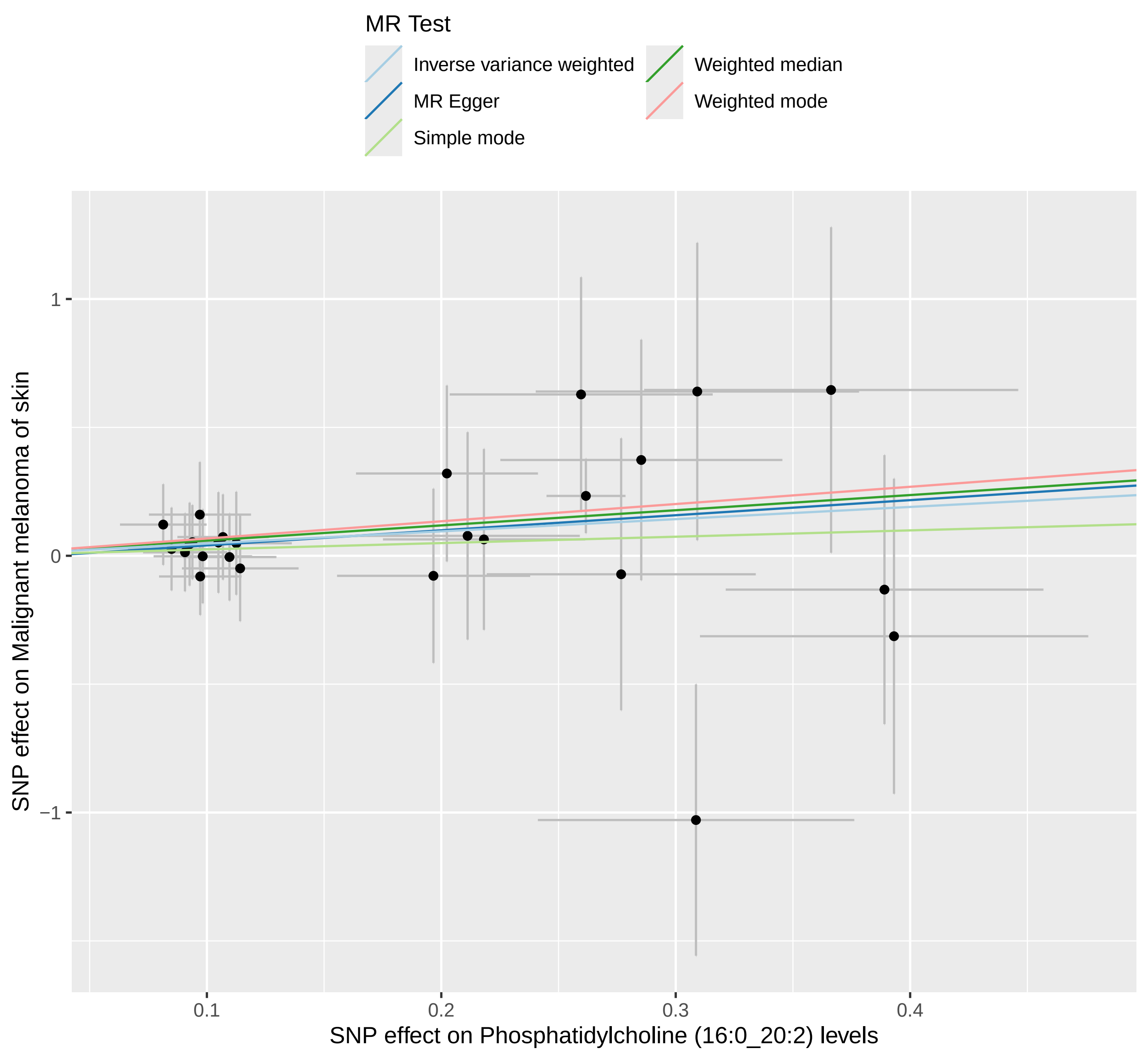
MR Test
Inverse variance weighted
MR Egger
Simple mode
Weighted median
Weighted mode
1
0
−1
0.1
0.2
0.3
0.4
SNP effect on Malignant melanoma of skin
SNP effect on Phosphatidylcholine (16:0_20:2) levels

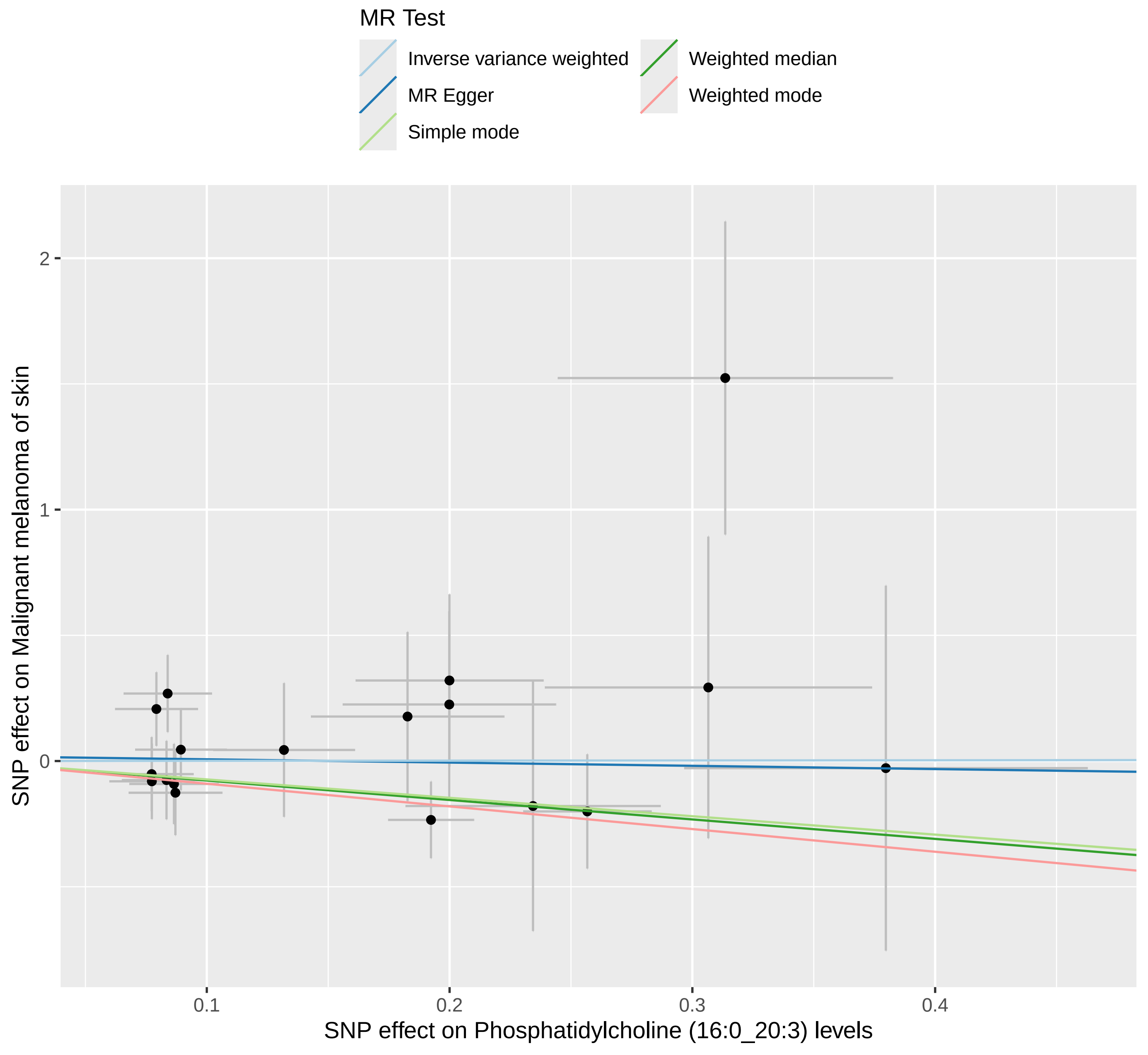
MR Test
Inverse variance weighted
MR Egger
Simple mode
Weighted median
Weighted mode
2
1
0
0.1
0.2
0.3
0.4
SNP effect on Malignant melanoma of skin
SNP effect on Phosphatidylcholine (16:0_20:3) levels

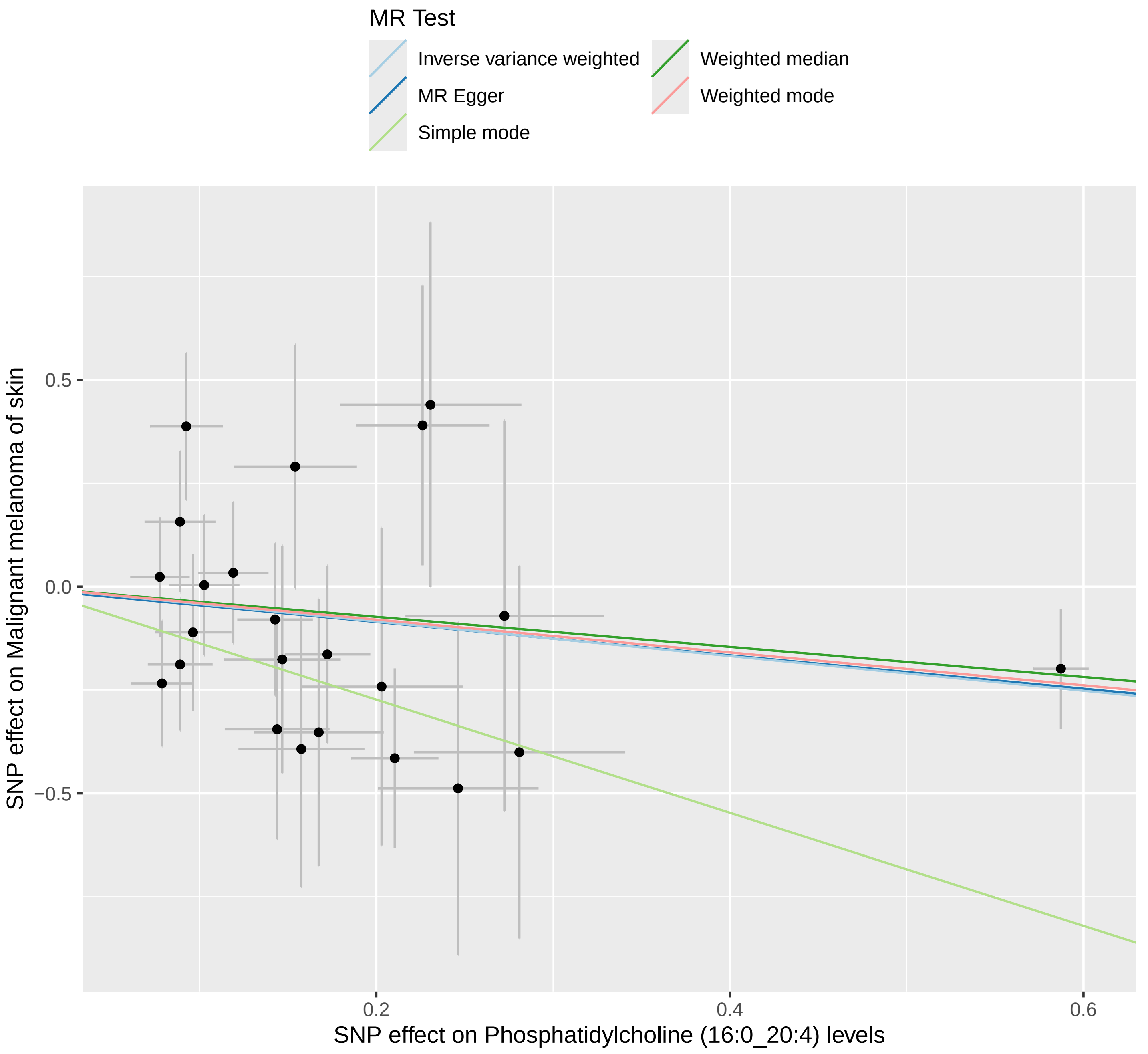
MR Test
Inverse variance weighted
MR Egger
Simple mode
Weighted median
Weighted mode
0.5
0.0
−0.5
0.2
0.4
0.6
SNP effect on Malignant melanoma of skin
SNP effect on Phosphatidylcholine (16:0_20:4) levels

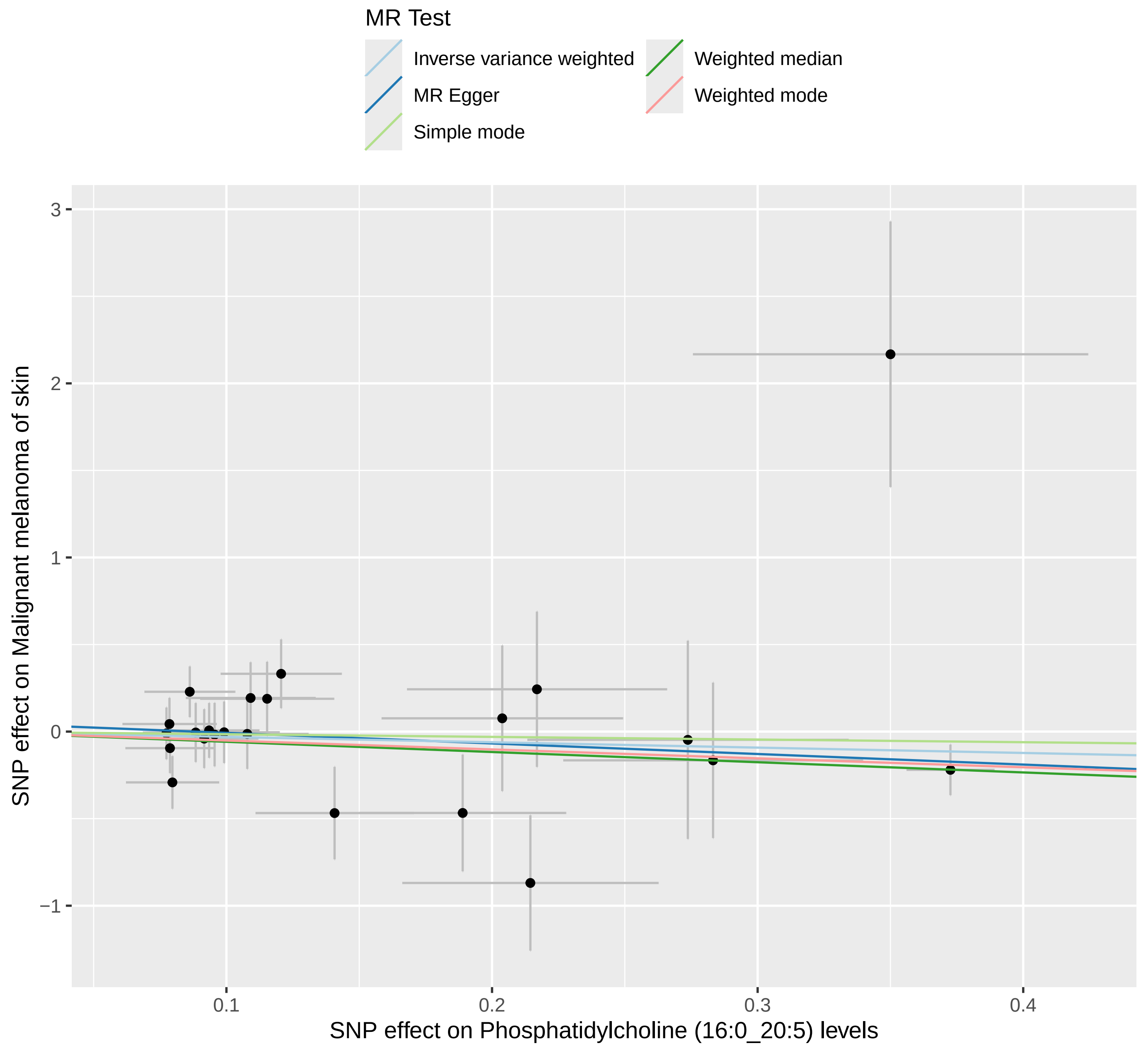

MR Test
Inverse variance weighted
MR Egger
Simple mode
Weighted median
Weighted mode
3
2
1
0
−1
0.1
0.2
0.3
0.4
SNP effect on Malignant melanoma of skin
SNP effect on Phosphatidylcholine (16:0_20:5) levels

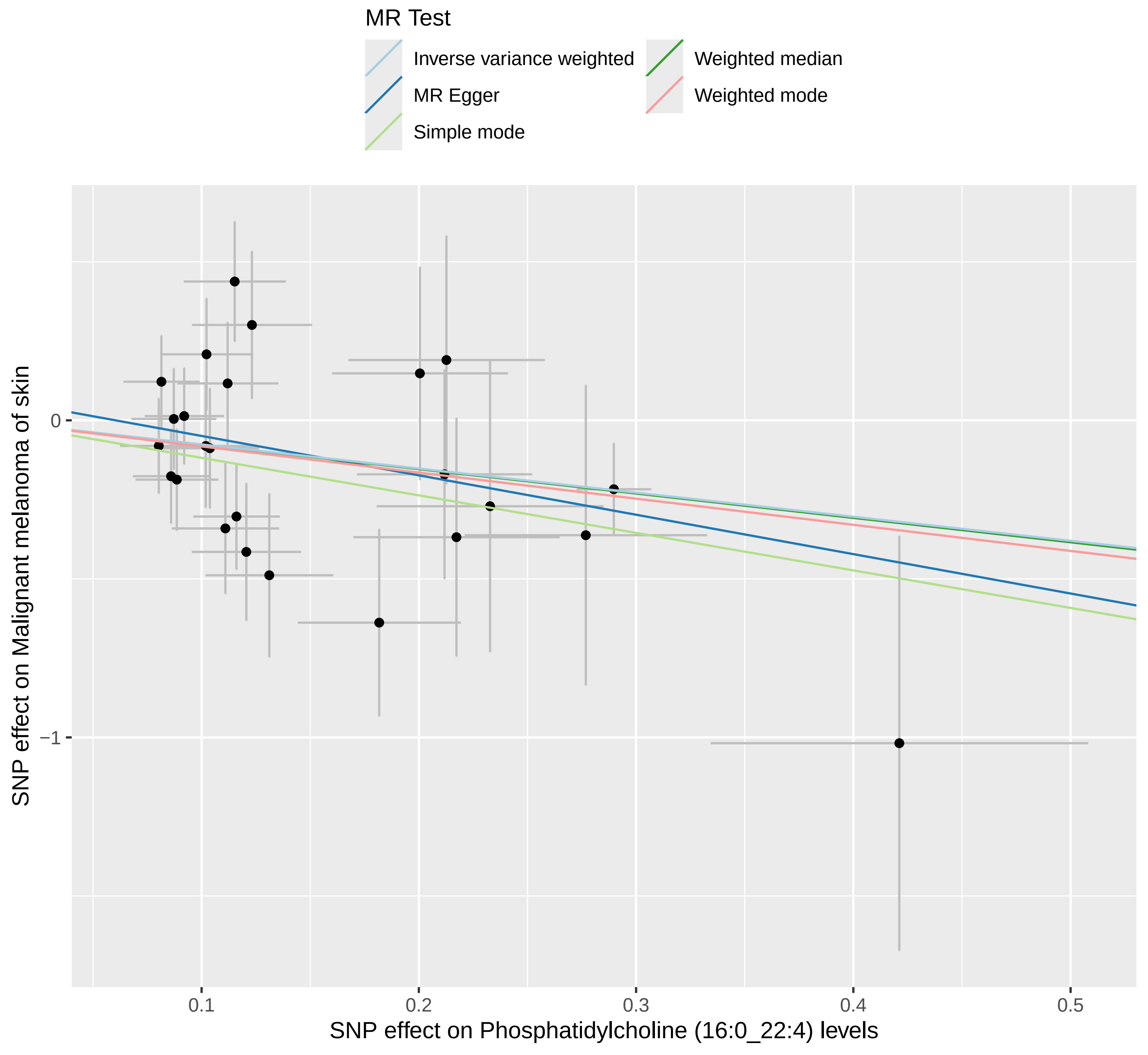
MR Test
Inverse variance weighted
MR Egger
Simple mode
Weighted median
Weighted mode
0
−1
0.1
0.2
0.3
0.4
0.5
SNP effect on Malignant melanoma of skin
SNP effect on Phosphatidylcholine (16:0_22:4) levels

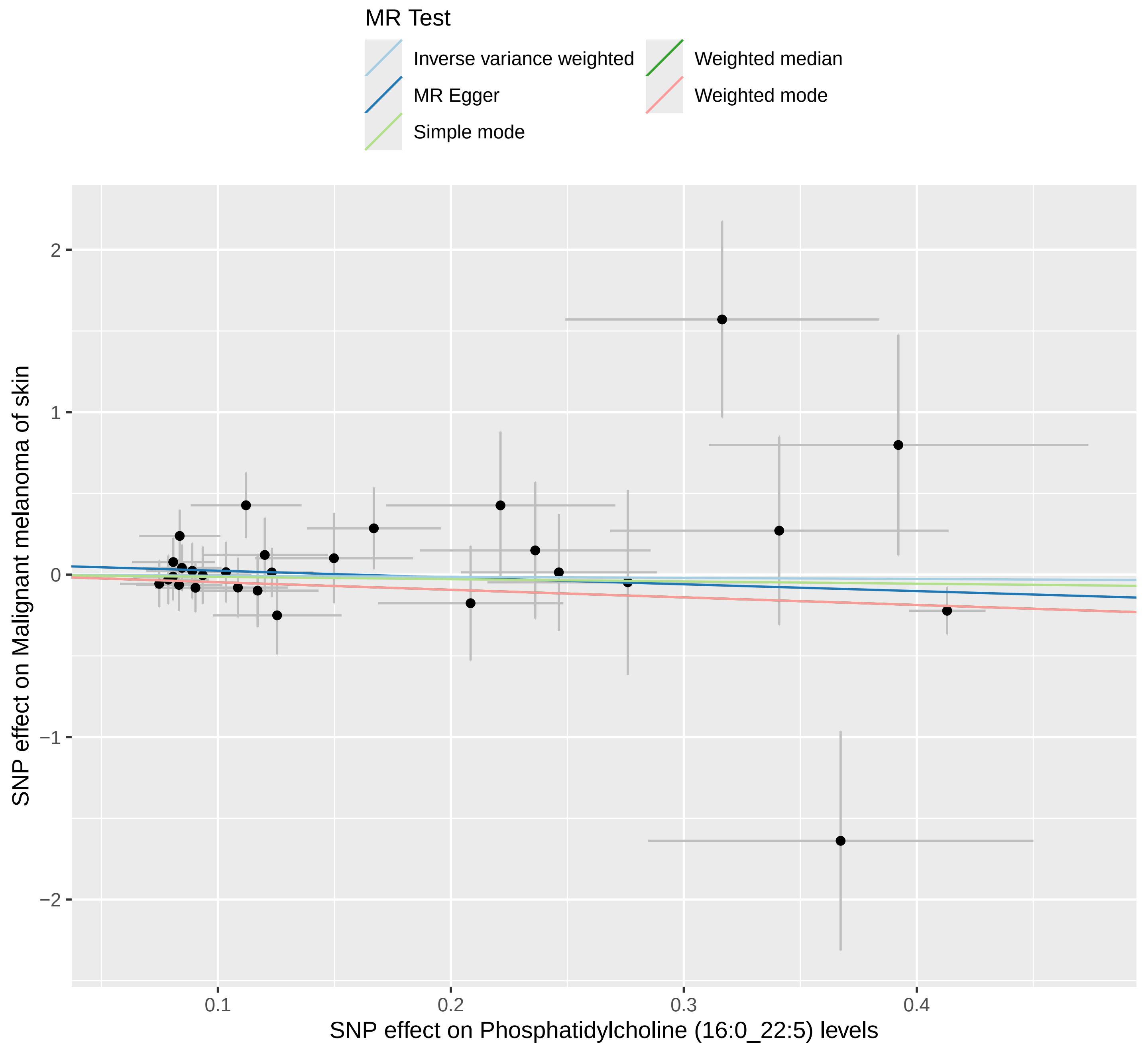
MR Test
Inverse variance weighted
MR Egger
Simple mode
Weighted median
Weighted mode
SNP effect on Malignant melanoma of skin
2
1
0
−1
−2
0.1
0.2
0.3
0.4
SNP effect on Phosphatidylcholine (16:0_22:5) levels

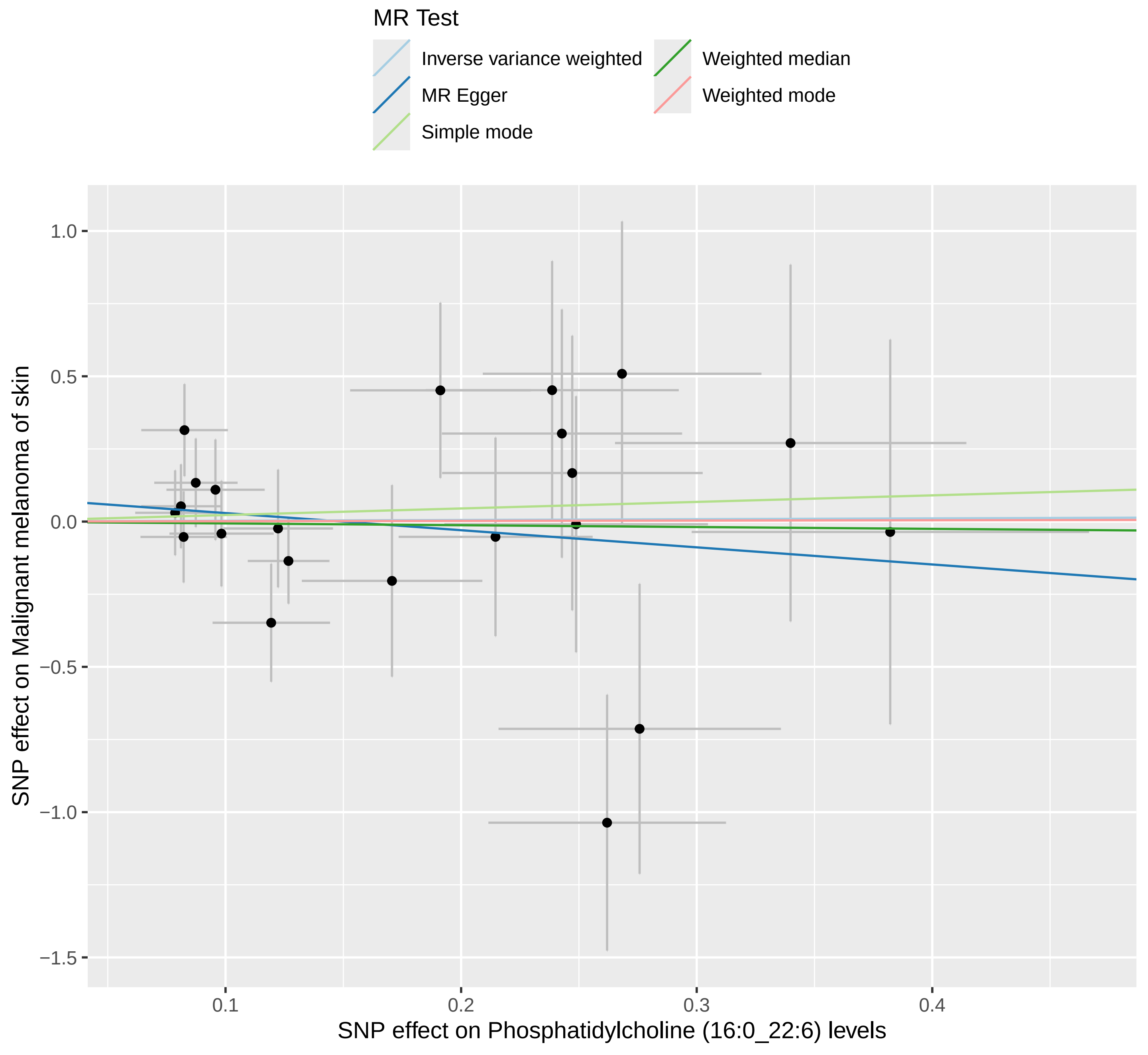

MR Test
Inverse variance weighted
MR Egger
Simple mode
Weighted median
Weighted mode
1.0
0.5
0.0
−0.5
−1.0
−1.5
0.1
0.2
0.3
0.4
SNP effect on Malignant melanoma of skin
SNP effect on Phosphatidylcholine (16:0_22:6) levels

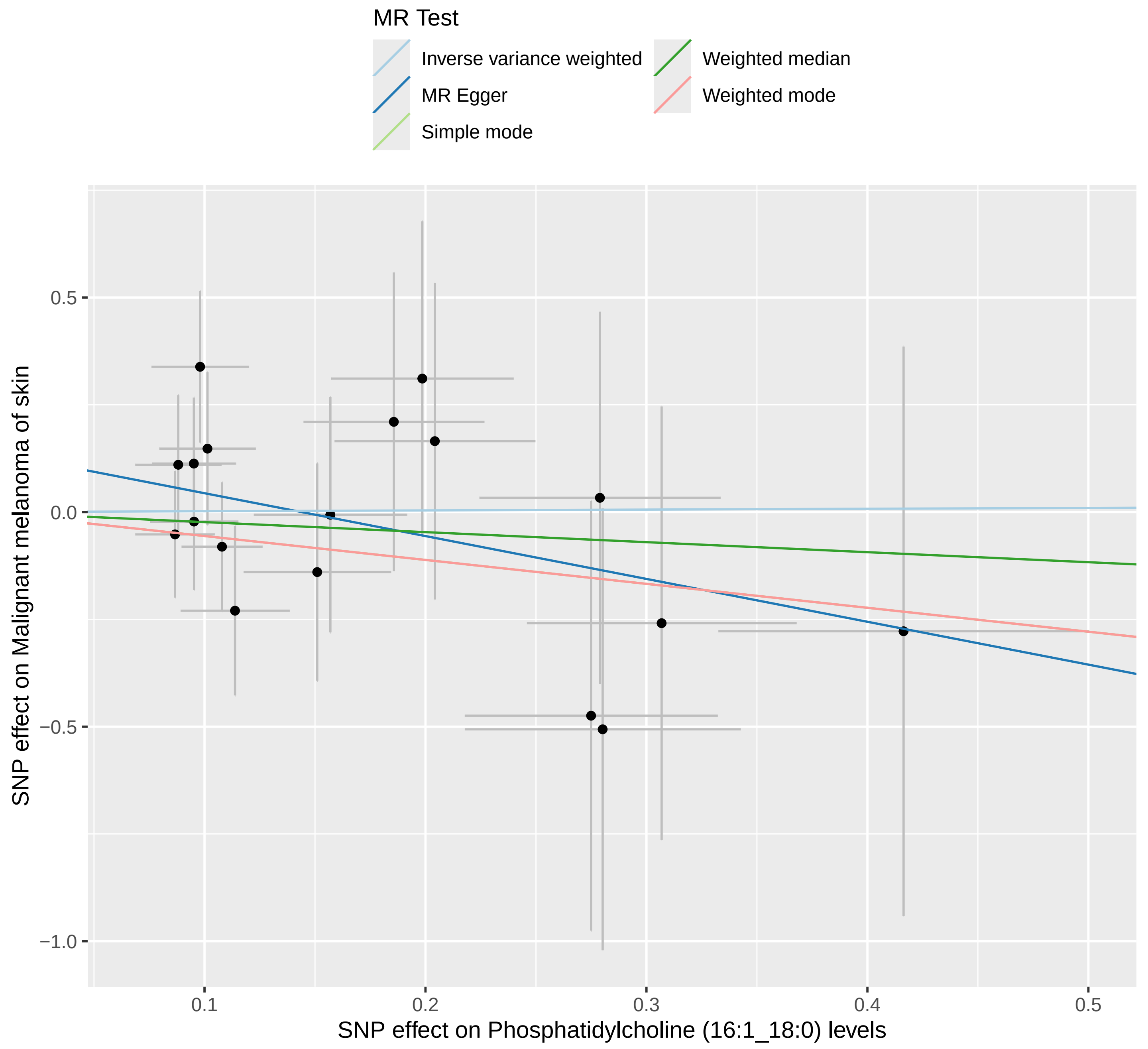
MR Test
Inverse variance weighted
MR Egger
Simple mode
Weighted median
Weighted mode
0.5
0.0
−0.5
−1.0
0.1
0.2
0.3
0.4
0.5
SNP effect on Malignant melanoma of skin
SNP effect on Phosphatidylcholine (16:1_18:0) levels

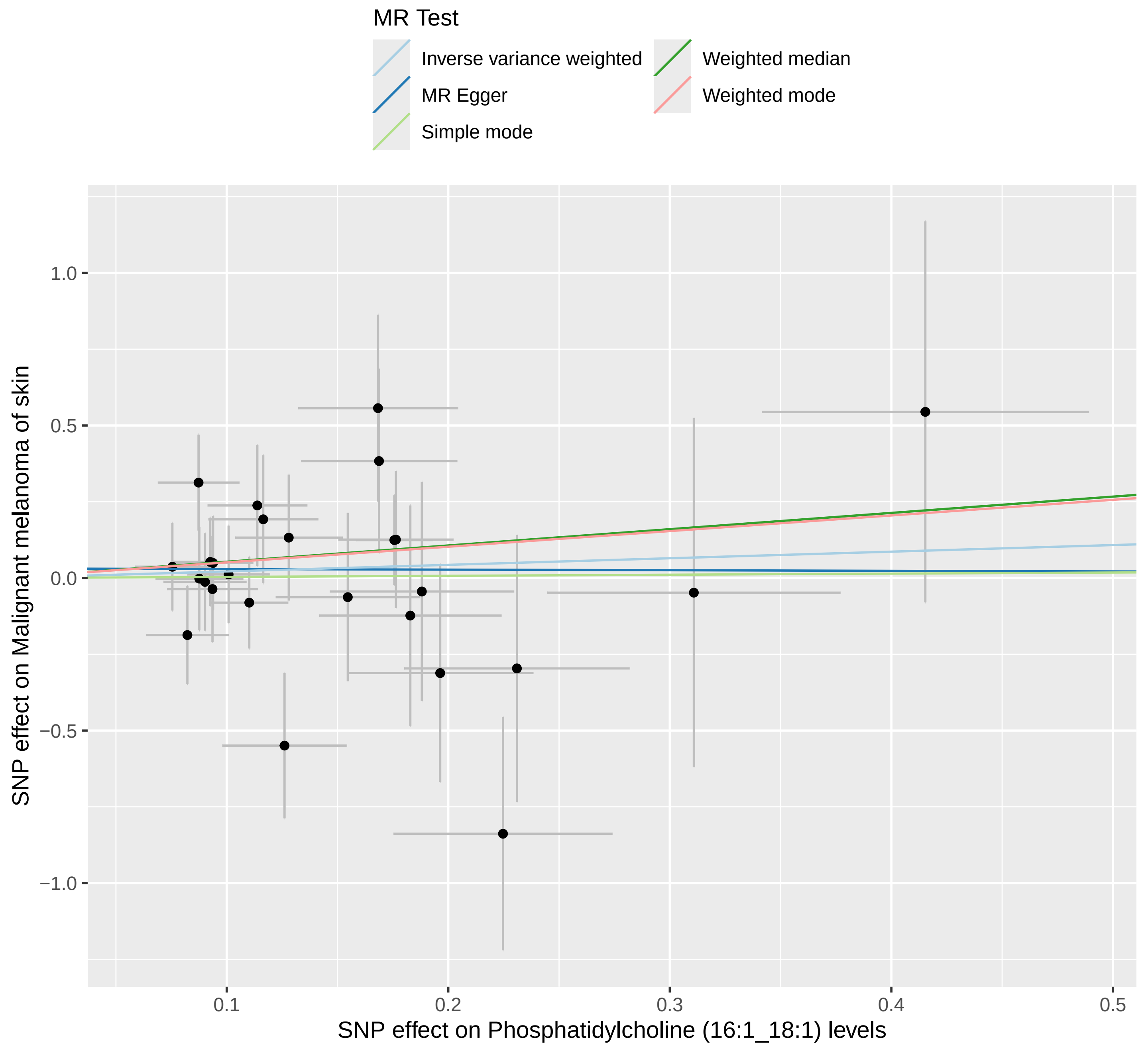
MR Test
Inverse variance weighted
MR Egger
Simple mode
Weighted median
Weighted mode
1.0
0.5
0.0
−0.5
−1.0
0.1
0.2
0.3
0.4
0.5
SNP effect on Malignant melanoma of skin
SNP effect on Phosphatidylcholine (16:1_18:1) levels

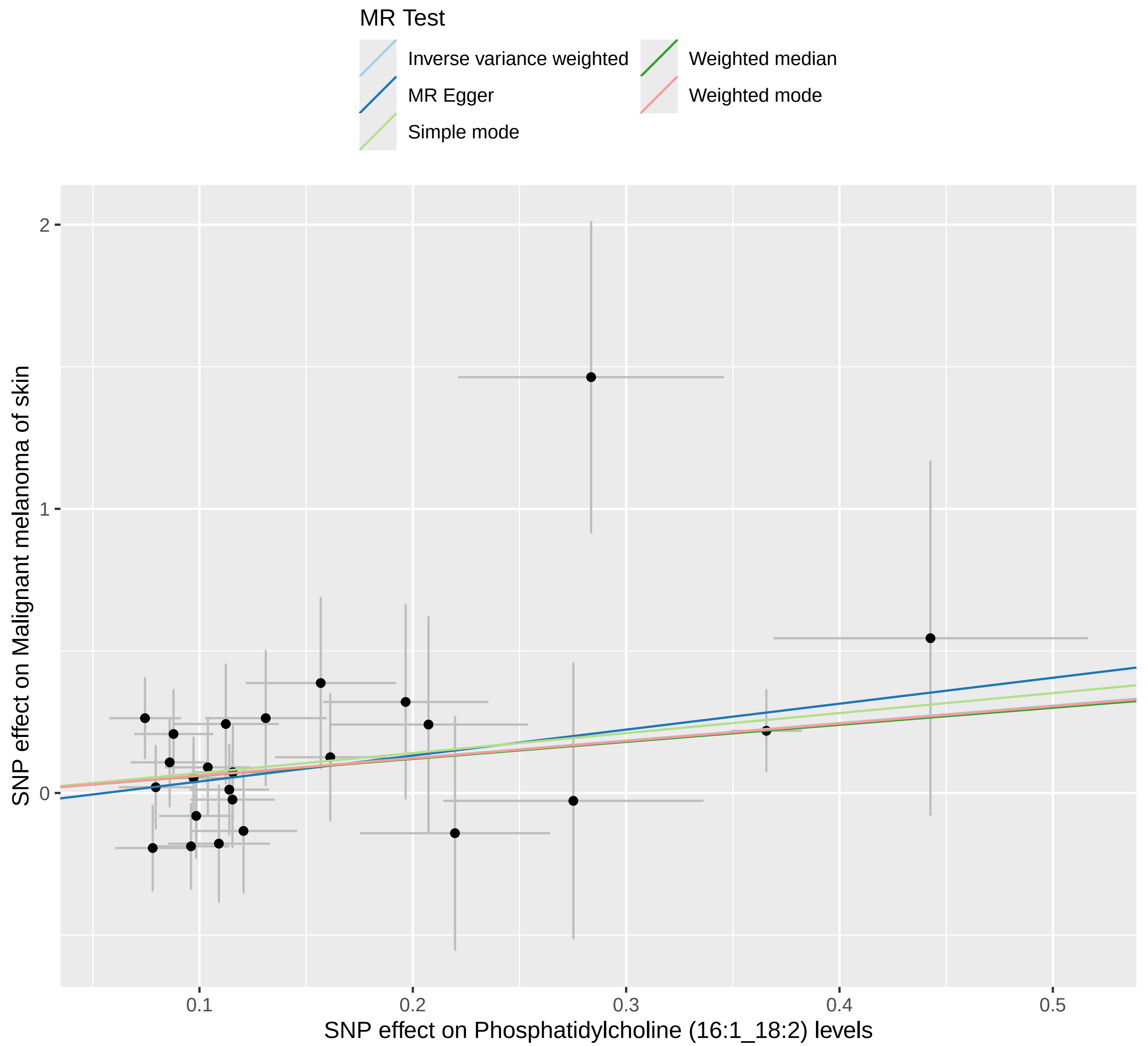
MR Test
Inverse variance weighted
MR Egger
Simple mode
Weighted median
Weighted mode
2
1
0
0.1
0.2
0.3
0.4
0.5
SNP effect on Malignant melanoma of skin
SNP effect on Phosphatidylcholine (16:1_18:2) levels

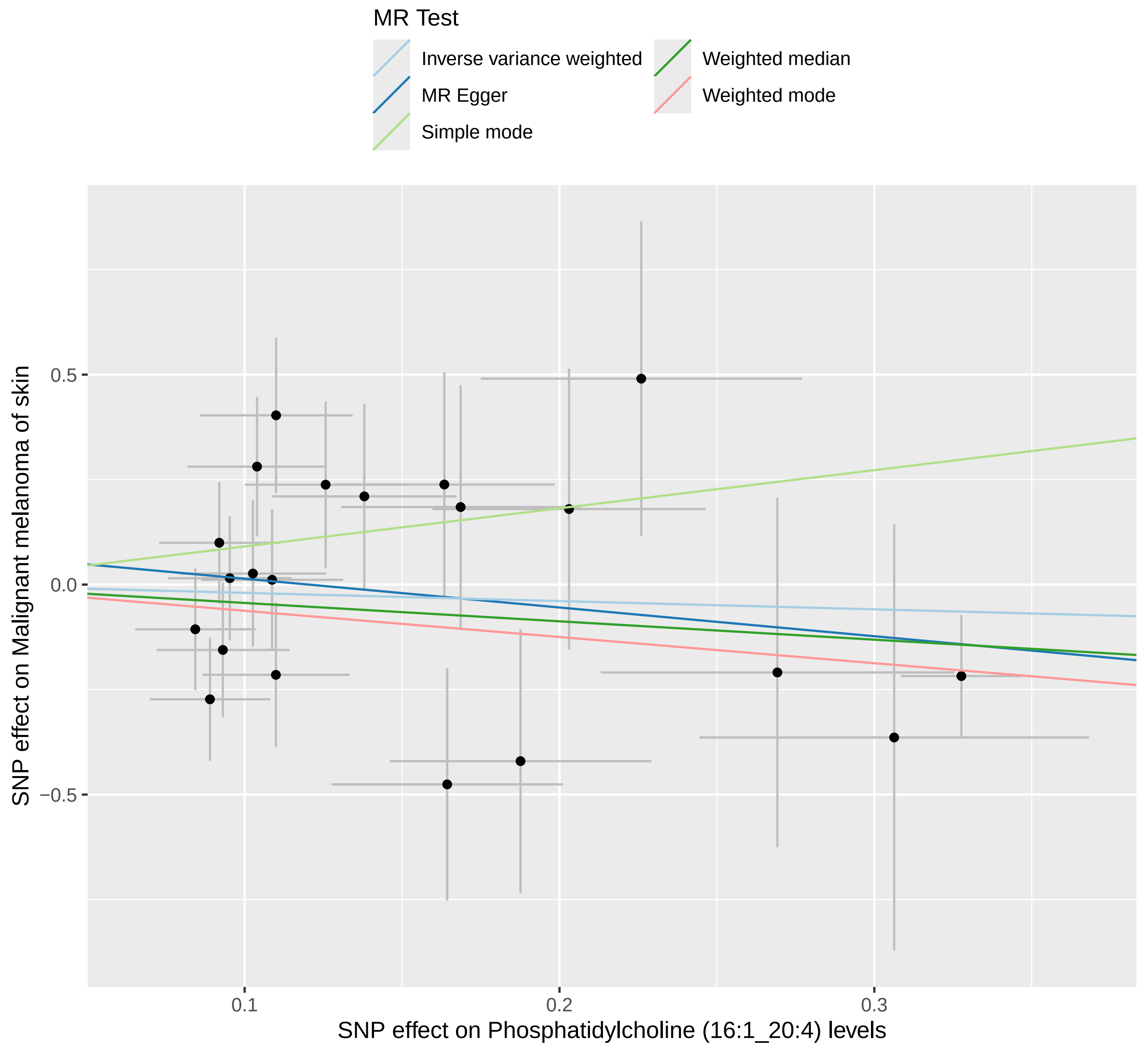
MR Test
Inverse variance weighted
MR Egger
Simple mode
Weighted median
Weighted mode
0.5
0.0
−0.5
0.1
0.2
0.3
SNP effect on Malignant melanoma of skin
SNP effect on Phosphatidylcholine (16:1_20:4) levels

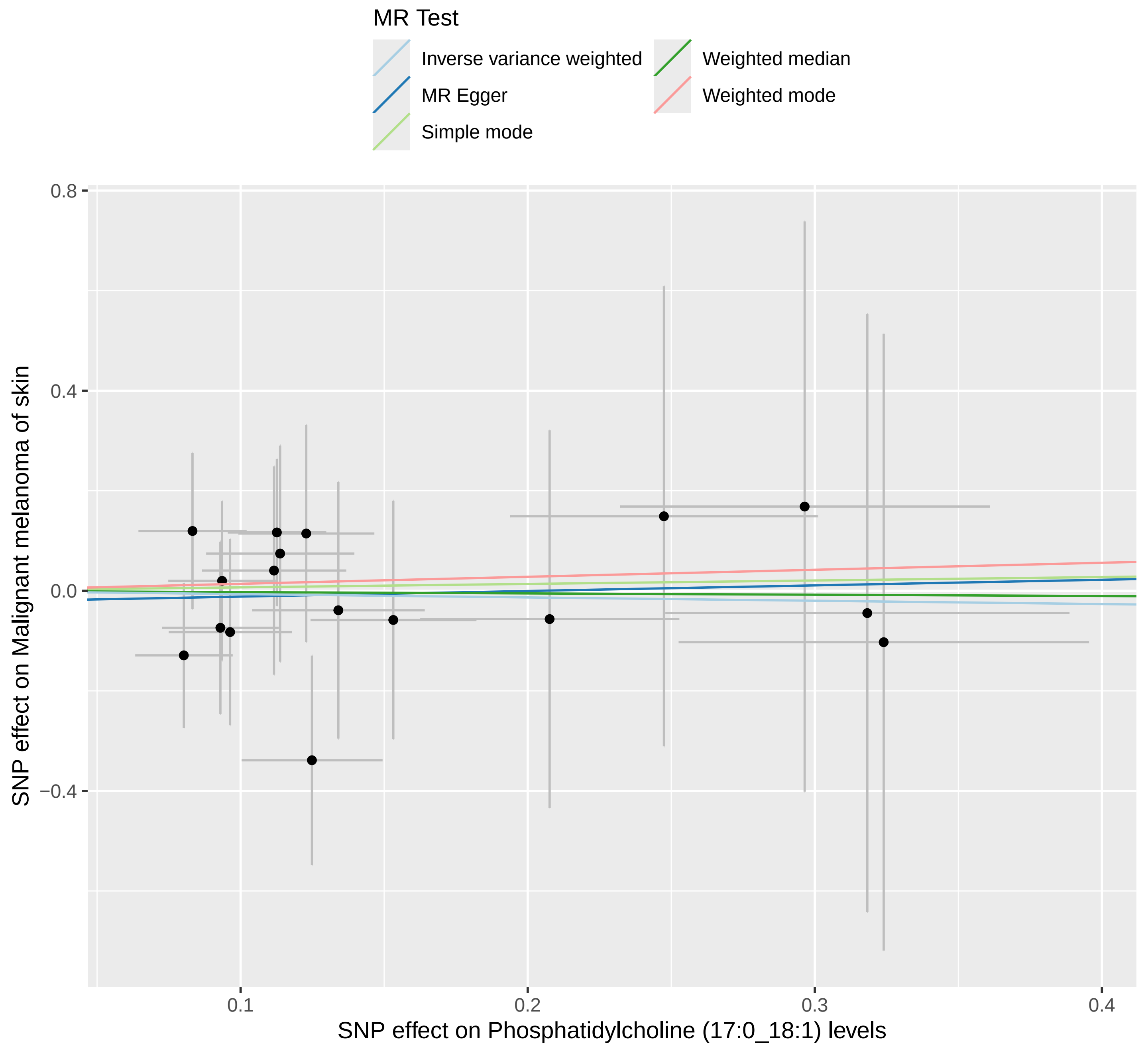

MR Test
Inverse variance weighted
MR Egger
Simple mode
Weighted median
Weighted mode
0.8
0.4
0.0
−0.4
0.1
0.2
0.3
0.4
SNP effect on Malignant melanoma of skin
SNP effect on Phosphatidylcholine (17:0_18:1) levels

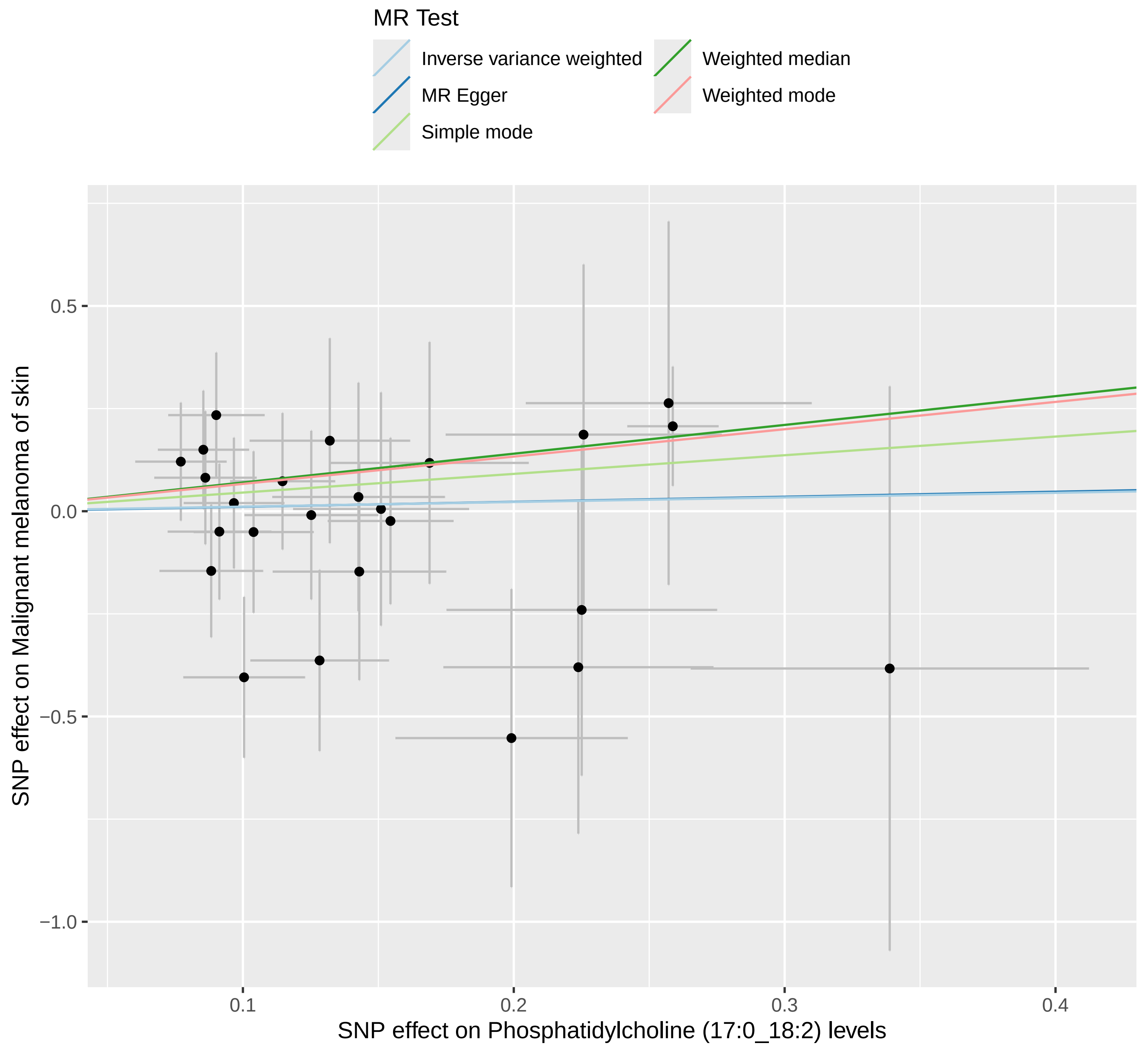

MR Test
Inverse variance weighted
MR Egger
Simple mode
Weighted median
Weighted mode
0.5
0.0
−0.5
−1.0
0.1
0.2
0.3
0.4
SNP effect on Malignant melanoma of skin
SNP effect on Phosphatidylcholine (17:0_18:2) levels

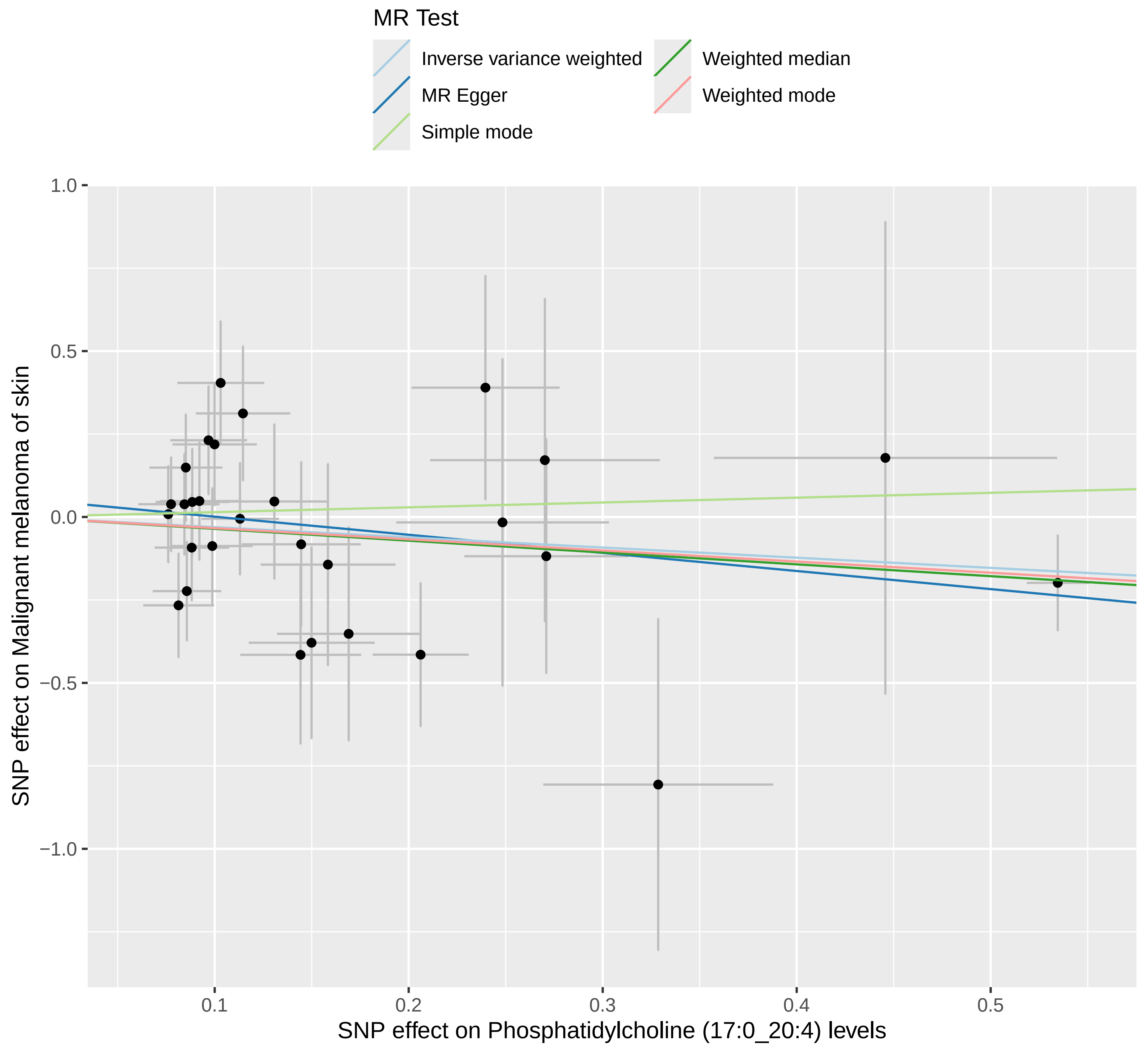

MR Test
Inverse variance weighted
MR Egger
Simple mode
Weighted median
Weighted mode
1.0
0.5
0.0
−0.5
−1.0
0.1
0.2
0.3
0.4
0.5
SNP effect on Malignant melanoma of skin
SNP effect on Phosphatidylcholine (17:0_20:4) levels

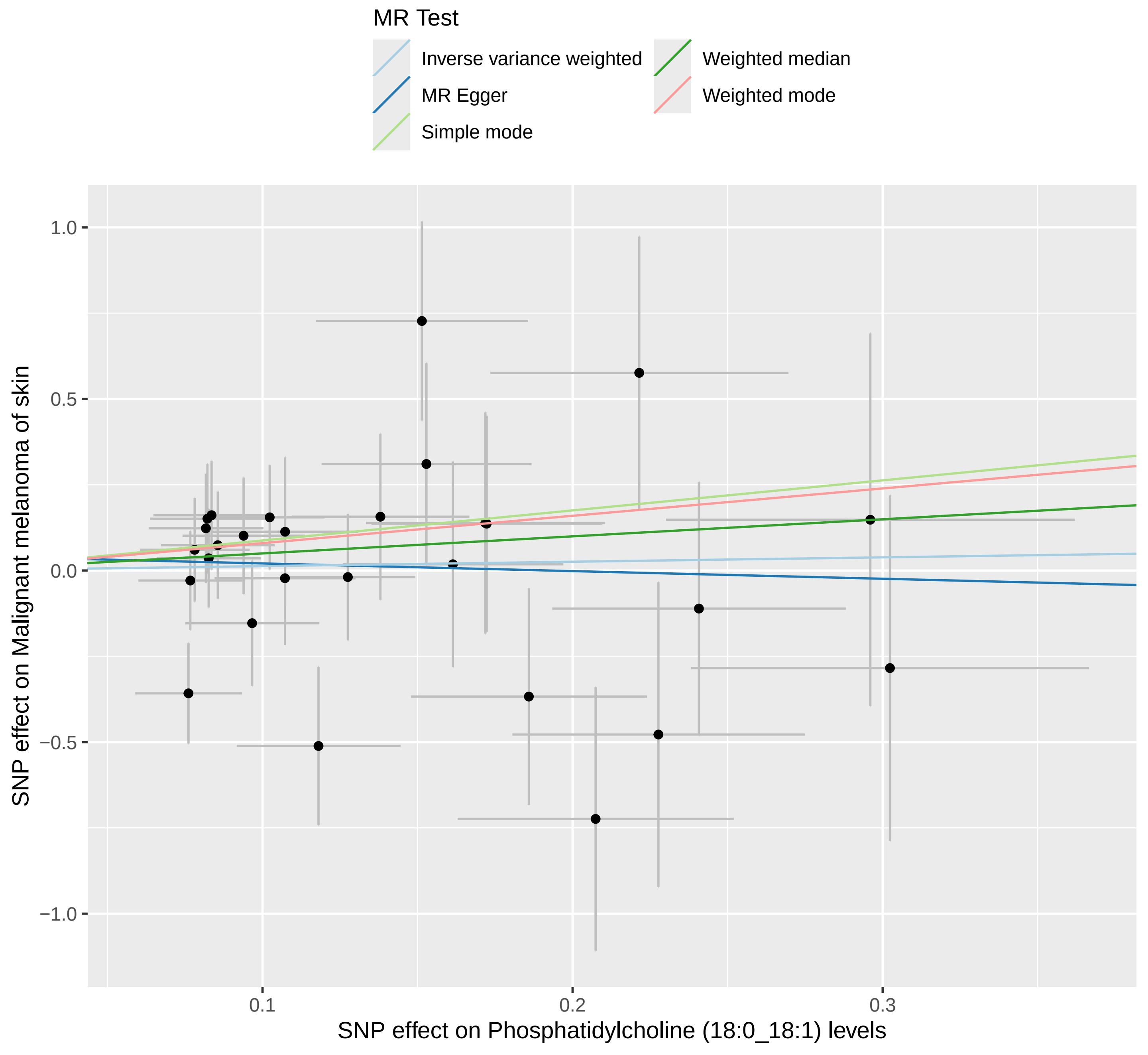

MR Test
Inverse variance weighted
MR Egger
Simple mode
Weighted median
Weighted mode
1.0
0.5
0.0
−0.5
−1.0
0.1
0.2
0.3
SNP effect on Malignant melanoma of skin
SNP effect on Phosphatidylcholine (18:0_18:1) levels

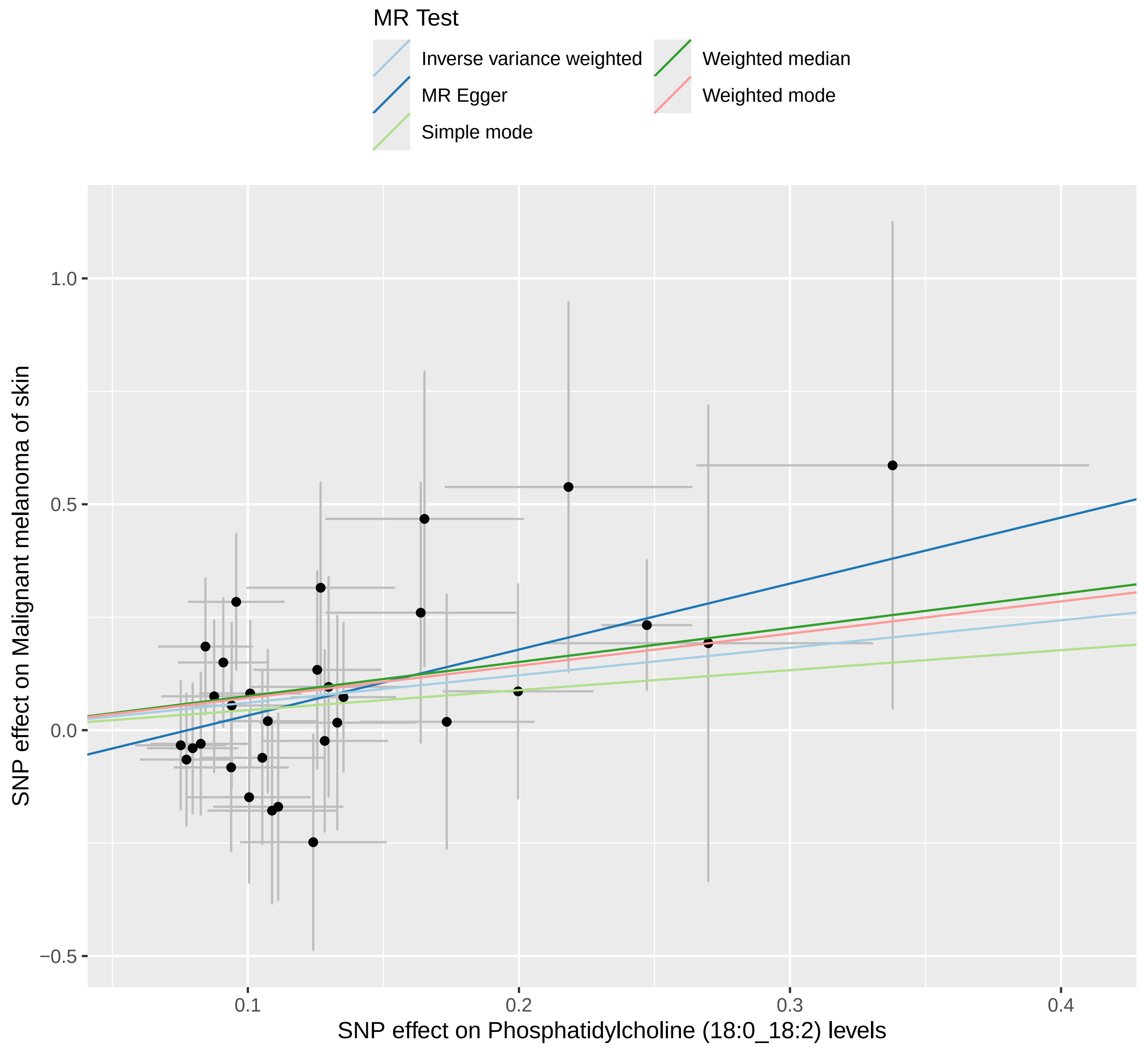

MR Test
Inverse variance weighted
MR Egger
Simple mode
Weighted median
Weighted mode
1.0
0.5
0.0
−0.5
0.1
0.2
0.3
0.4
SNP effect on Malignant melanoma of skin
SNP effect on Phosphatidylcholine (18:0_18:2) levels

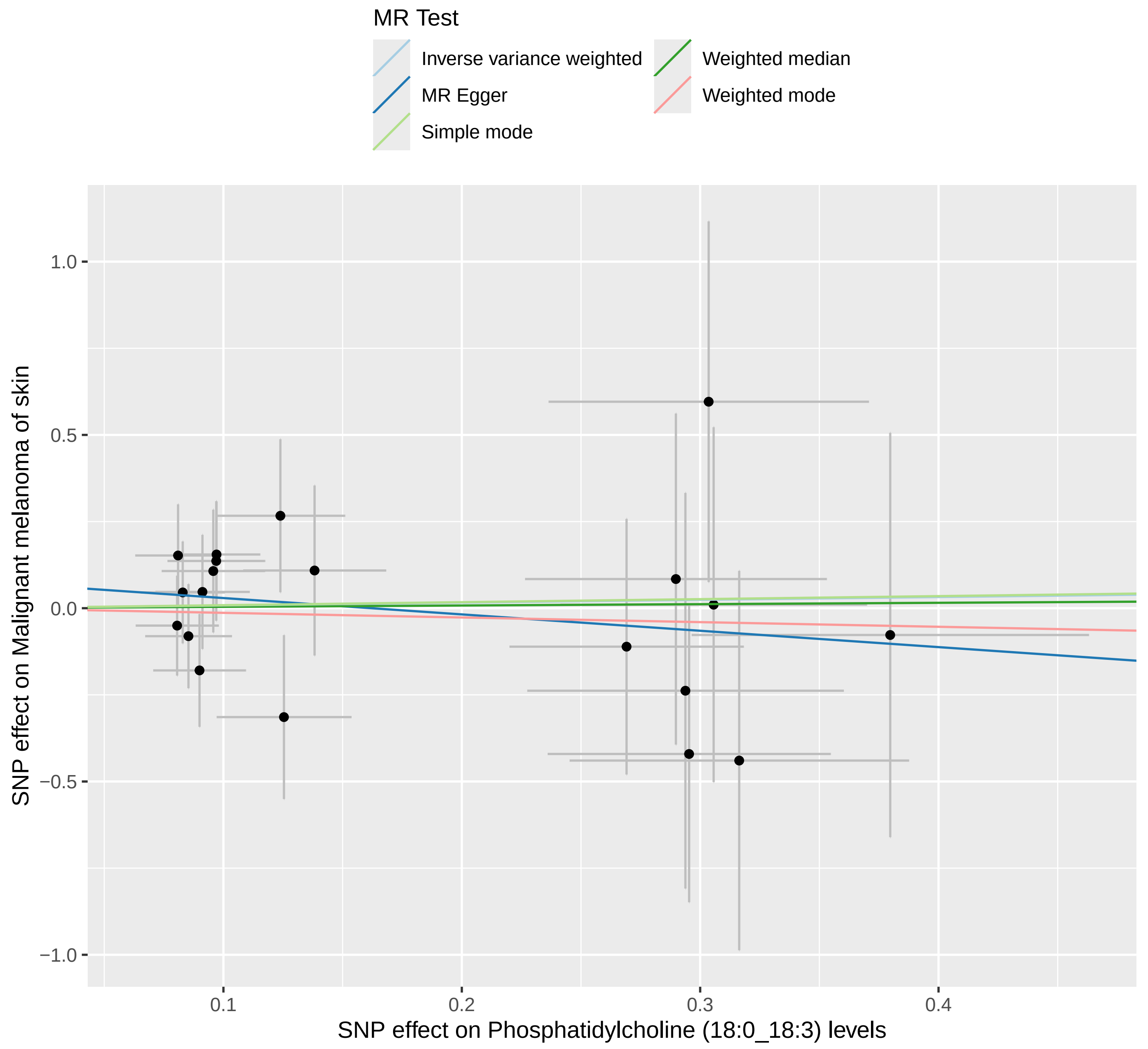

MR Test
Inverse variance weighted
MR Egger
Simple mode
Weighted median
Weighted mode
1.0
0.5
0.0
−0.5
−1.0
0.1
0.2
0.3
0.4
SNP effect on Malignant melanoma of skin
SNP effect on Phosphatidylcholine (18:0_18:3) levels

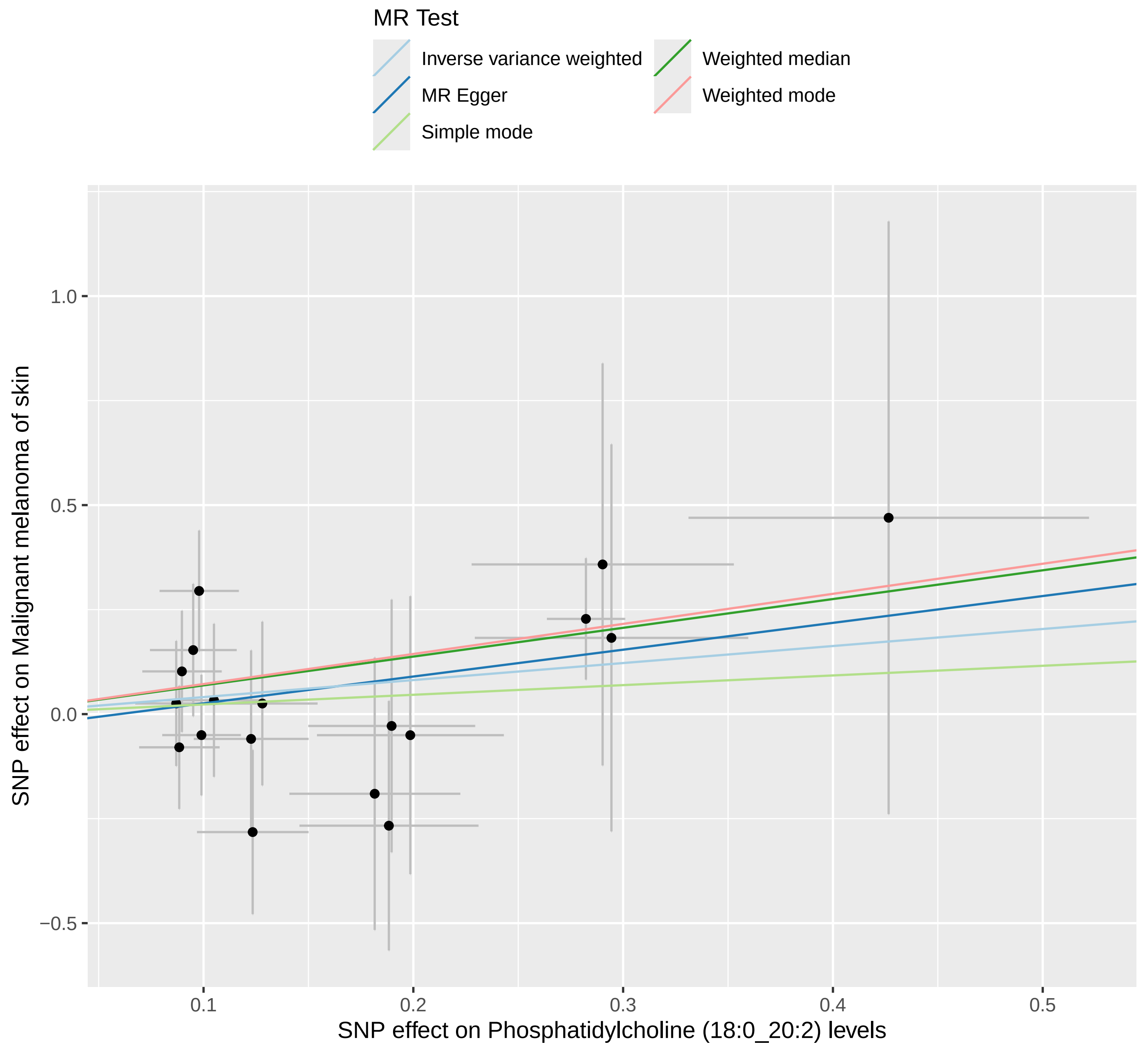
MR Test
Inverse variance weighted
MR Egger
Simple mode
Weighted median
Weighted mode
1.0
0.5
0.0
−0.5
0.1
0.2
0.3
0.4
0.5
SNP effect on Malignant melanoma of skin
SNP effect on Phosphatidylcholine (18:0_20:2) levels

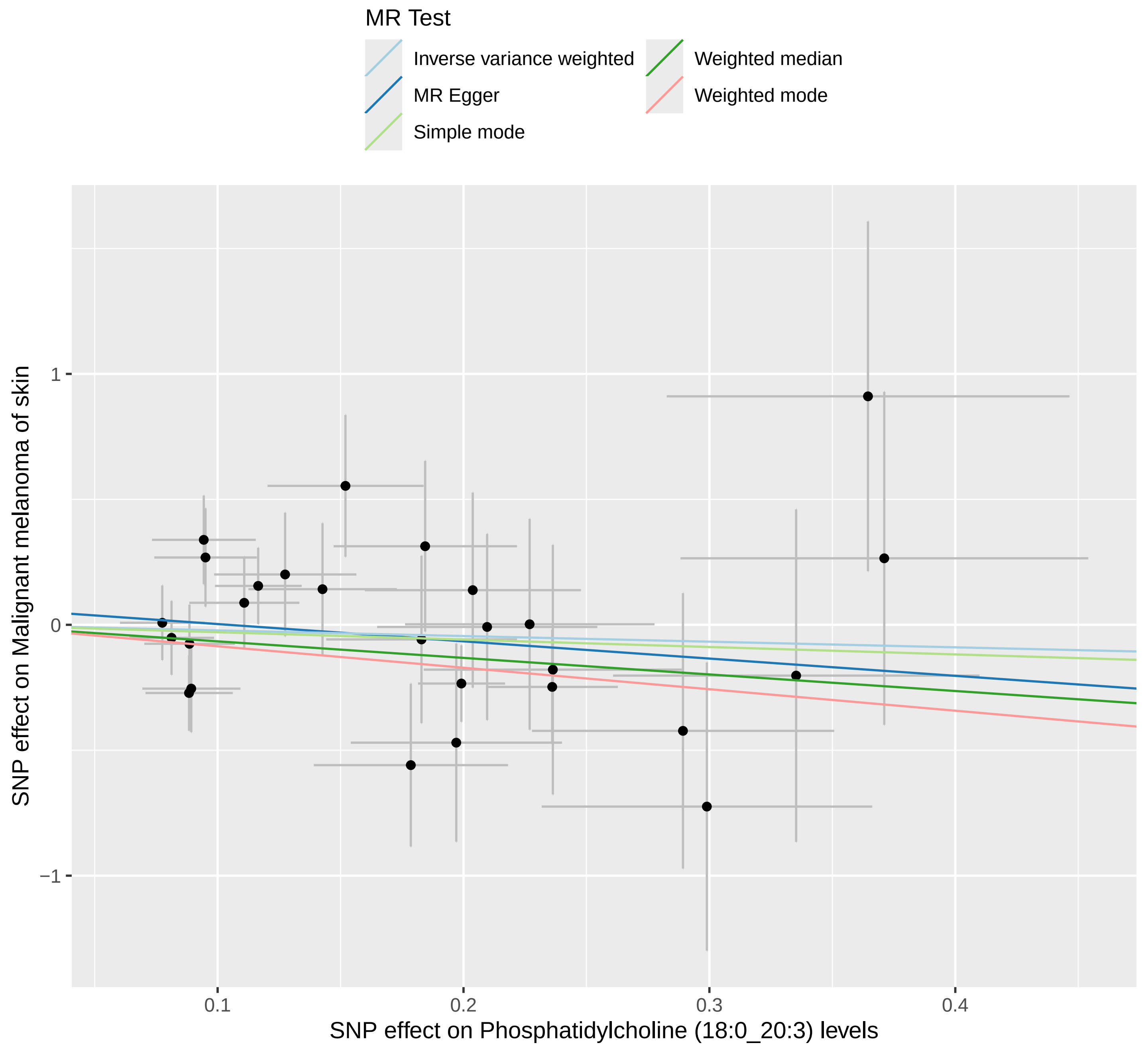
MR Test
Inverse variance weighted
MR Egger
Simple mode
Weighted median
Weighted mode
1
0
−1
0.1
0.2
0.3
0.4
SNP effect on Malignant melanoma of skin
SNP effect on Phosphatidylcholine (18:0_20:3) levels

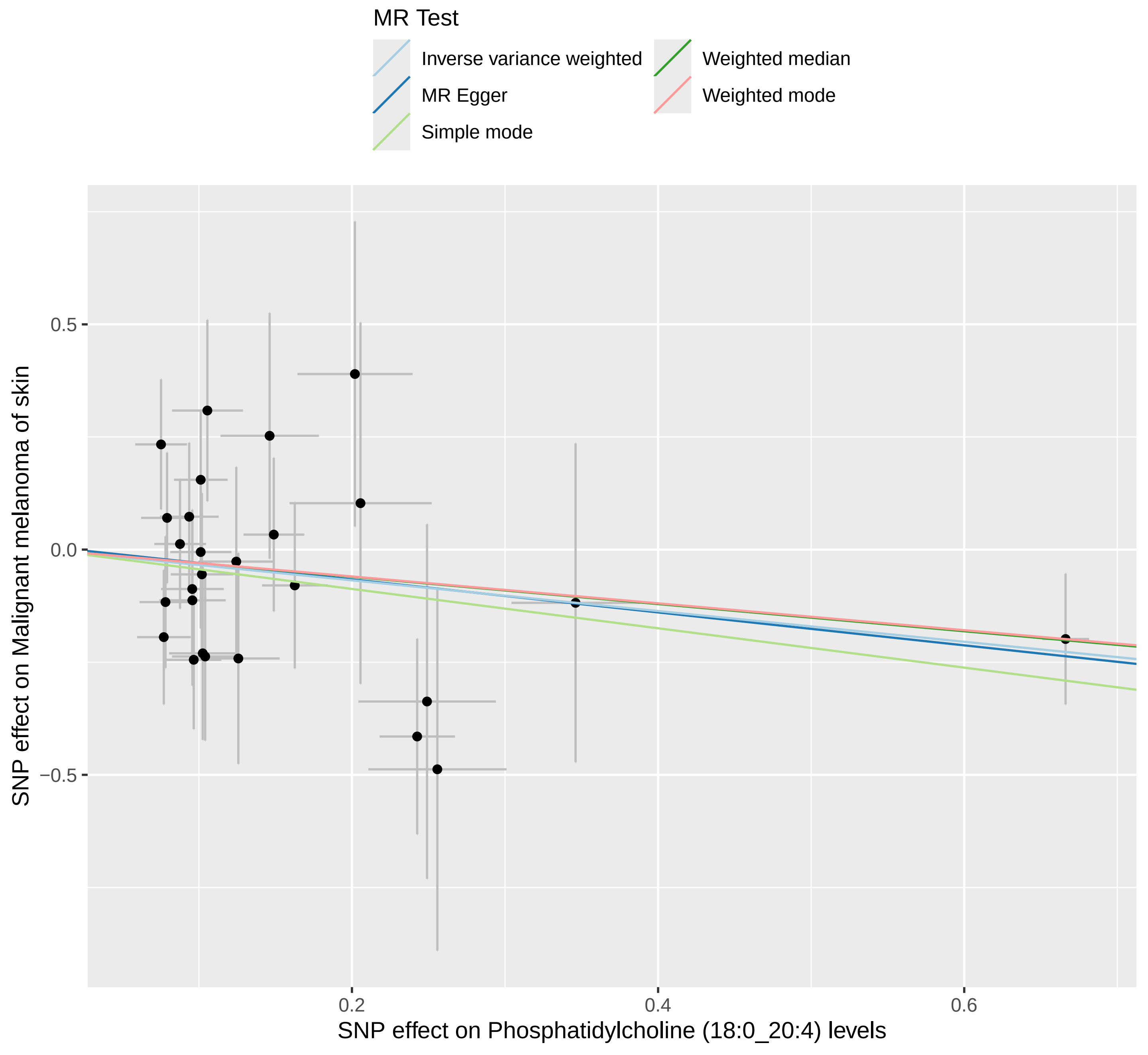
MR Test
Inverse variance weighted
MR Egger
Simple mode
Weighted median
Weighted mode
0.5
0.0
−0.5
0.2
0.4
0.6
SNP effect on Malignant melanoma of skin
SNP effect on Phosphatidylcholine (18:0_20:4) levels

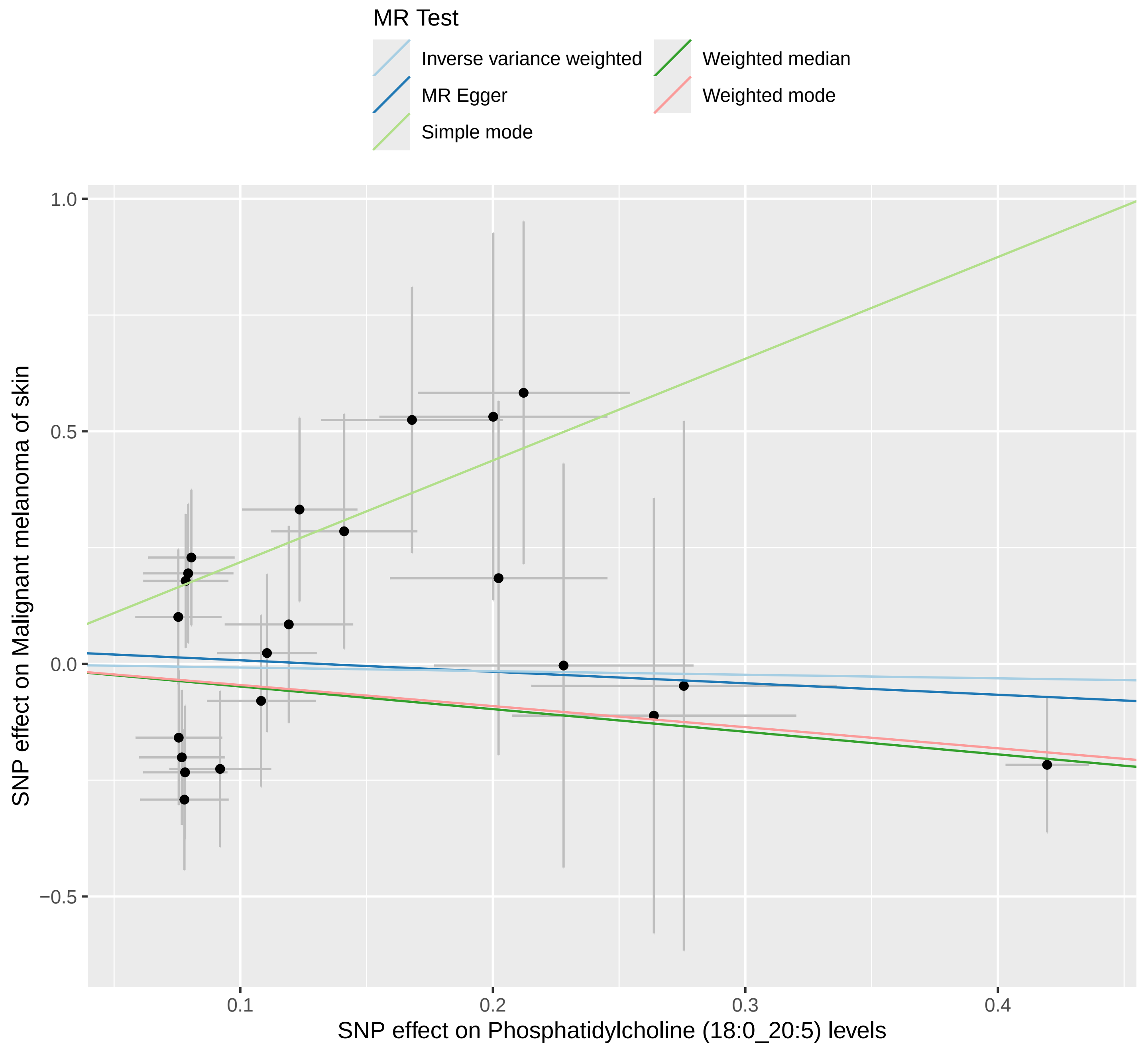
MR Test
Inverse variance weighted
MR Egger
Simple mode
Weighted median
Weighted mode
1.0
0.5
0.0
−0.5
SNP effect on Malignant melanoma of skin
0.1
0.2
0.3
0.4
SNP effect on Phosphatidylcholine (18:0_20:5) levels

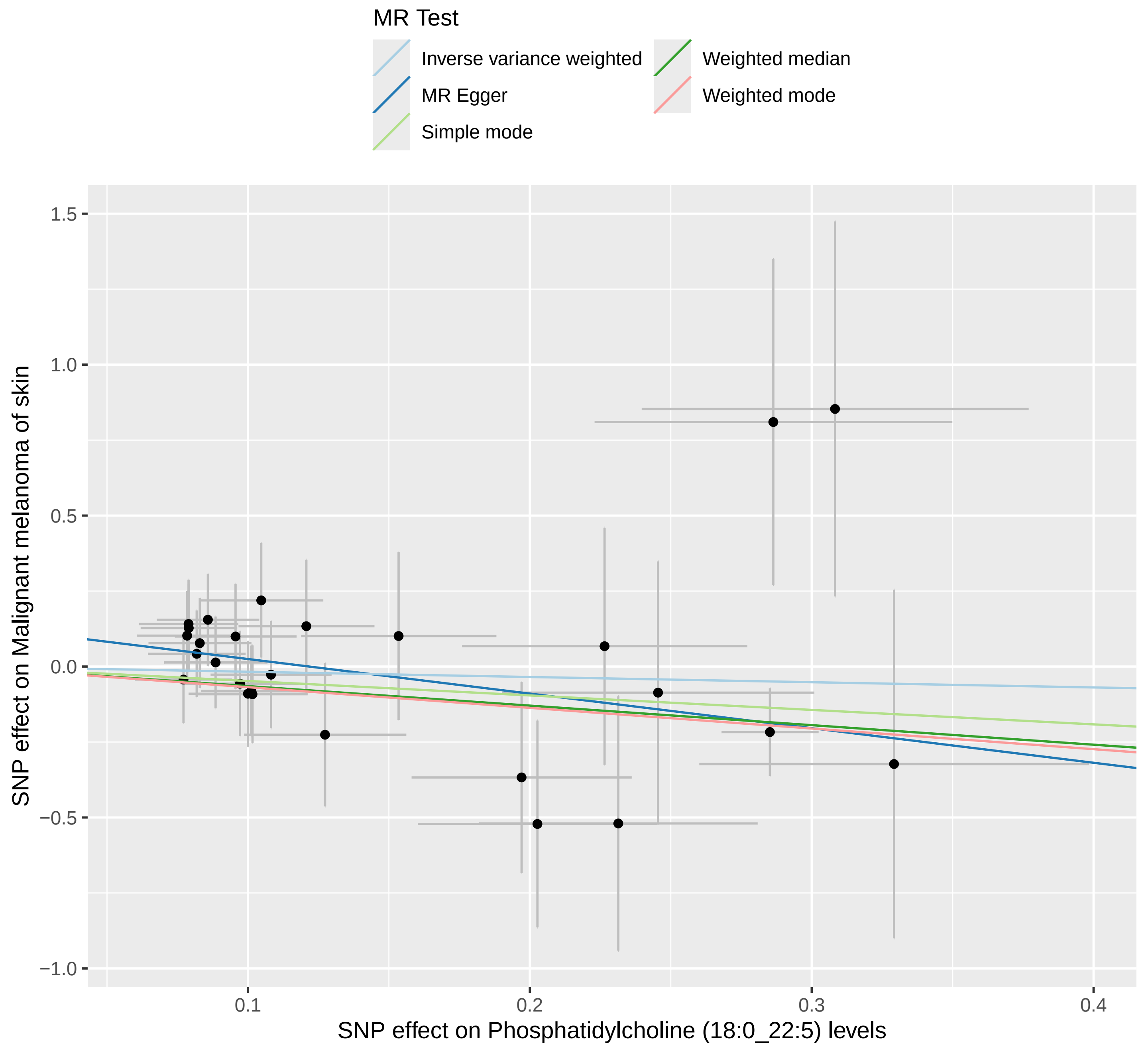

MR Test
Inverse variance weighted
MR Egger
Simple mode
Weighted median
Weighted mode
1.5
1.0
0.5
0.0
−0.5
−1.0
SNP effect on Malignant melanoma of skin
0.1
0.2
0.3
0.4
SNP effect on Phosphatidylcholine (18:0_22:5) levels

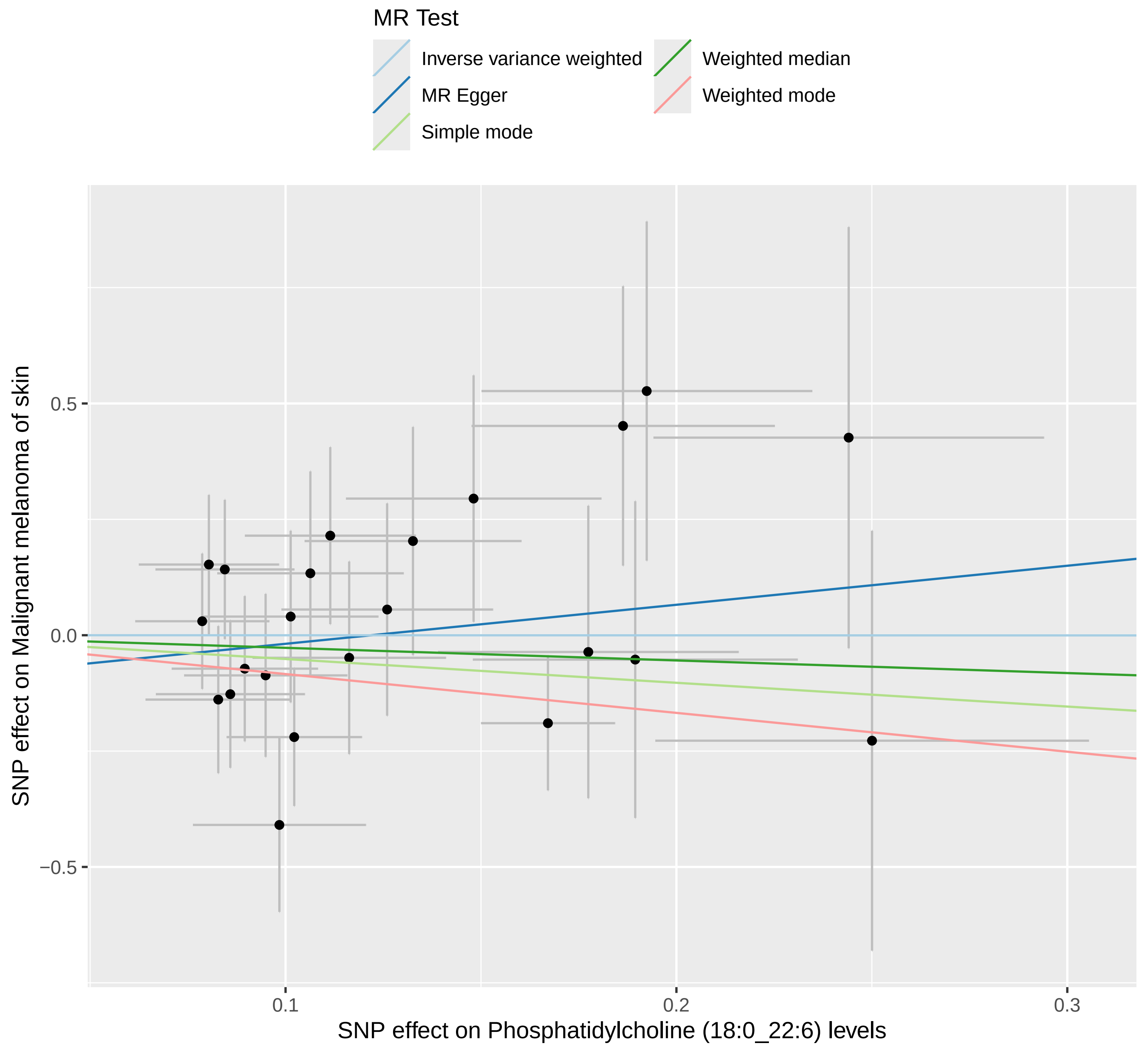
MR Test
Inverse variance weighted
MR Egger
Simple mode
Weighted median
Weighted mode
SNP effect on Malignant melanoma of skin
SNP effect on Phosphatidylcholine (18:0_22:6) levels
0.5
0.0
−0.5
0.1
0.2
0.3

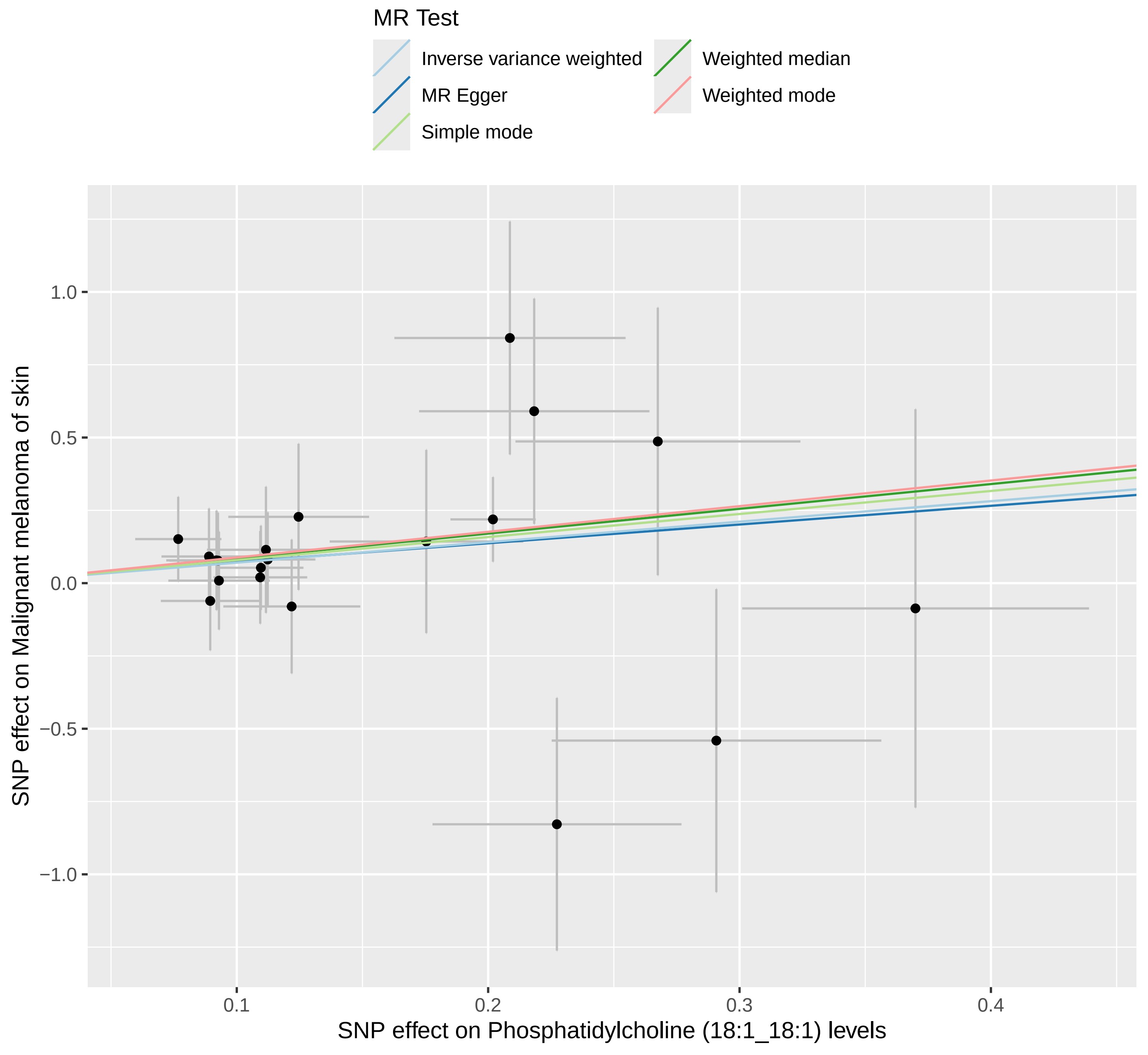

MR Test
Inverse variance weighted
MR Egger
Simple mode
Weighted median
Weighted mode
1.0
0.5
0.0
−0.5
−1.0
0.1
0.2
0.3
0.4
SNP effect on Malignant melanoma of skin
SNP effect on Phosphatidylcholine (18:1_18:1) levels

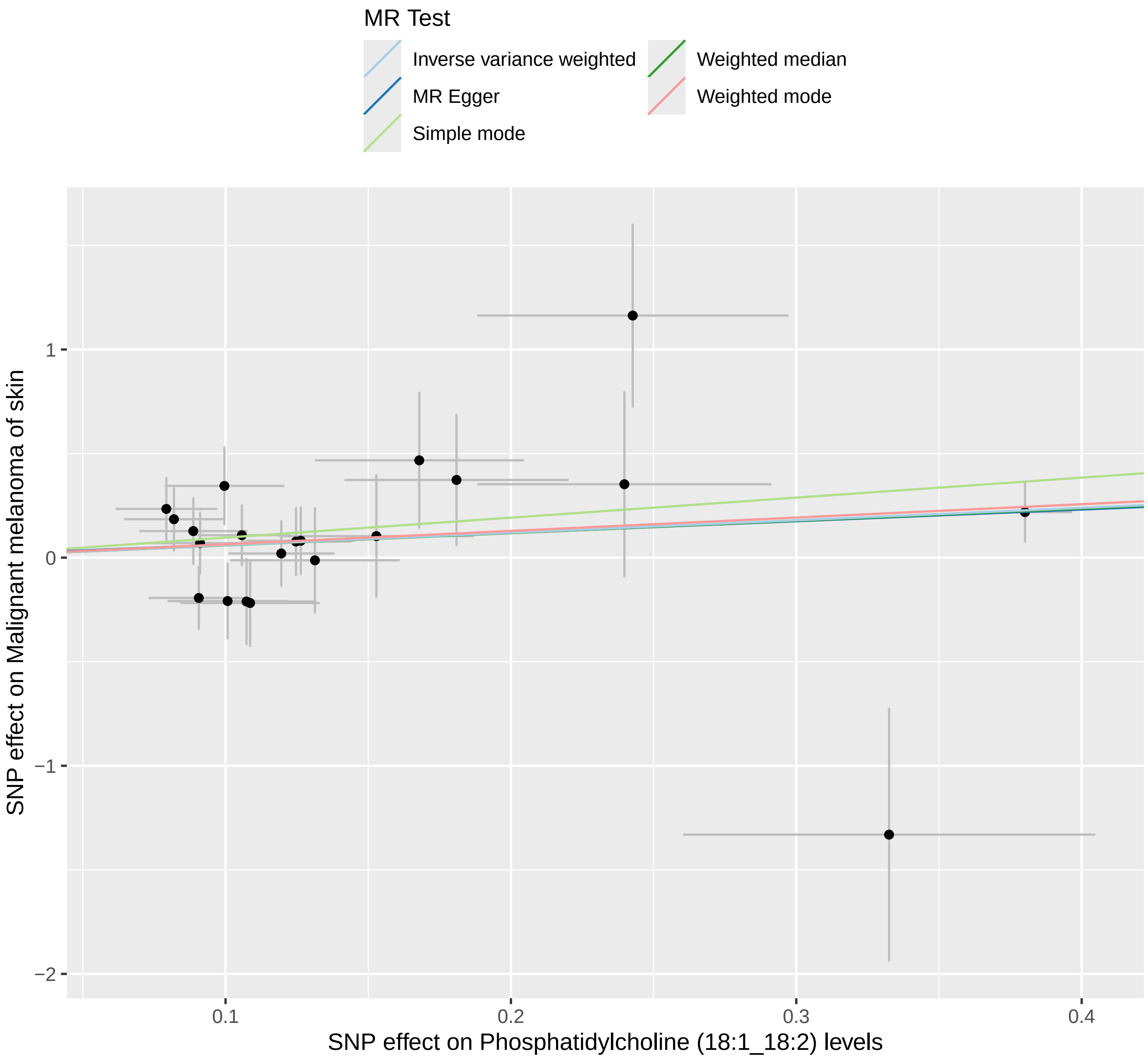
MR Test
Inverse variance weighted
MR Egger
Simple mode
Weighted median
Weighted mode
SNP effect on Malignant melanoma of skin
1
0
−1
−2
0.1
0.2
0.3
0.4
SNP effect on Phosphatidylcholine (18:1_18:2) levels

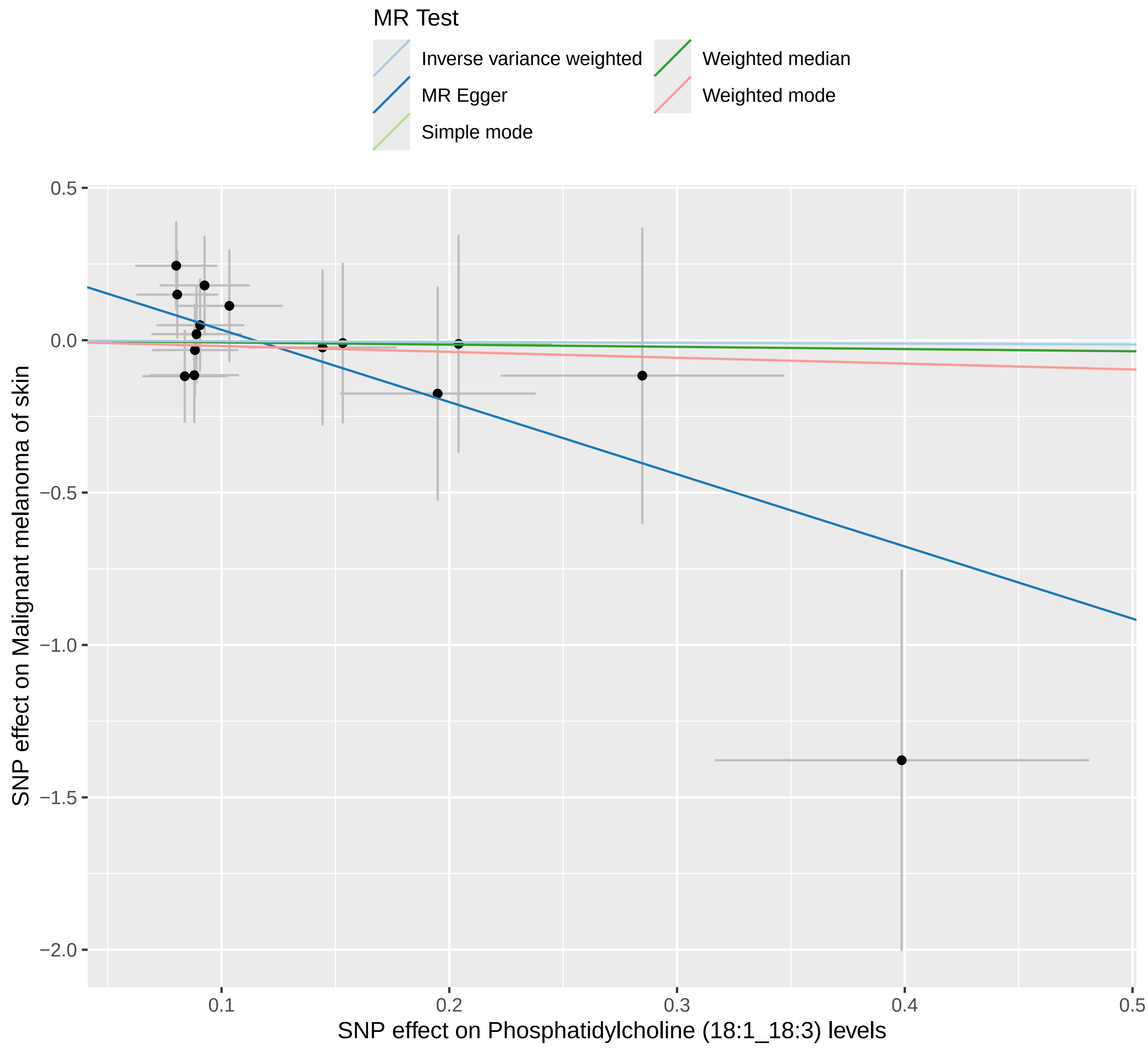
MR Test
Inverse variance weighted
MR Egger
Simple mode
Weighted median
Weighted mode
0.5
0.0
−0.5
−1.0
−1.5
−2.0
0.1
0.2
0.3
0.4
0.5
SNP effect on Phosphatidylcholine (18:1_18:3) levels
SNP effect on Malignant melanoma of skin

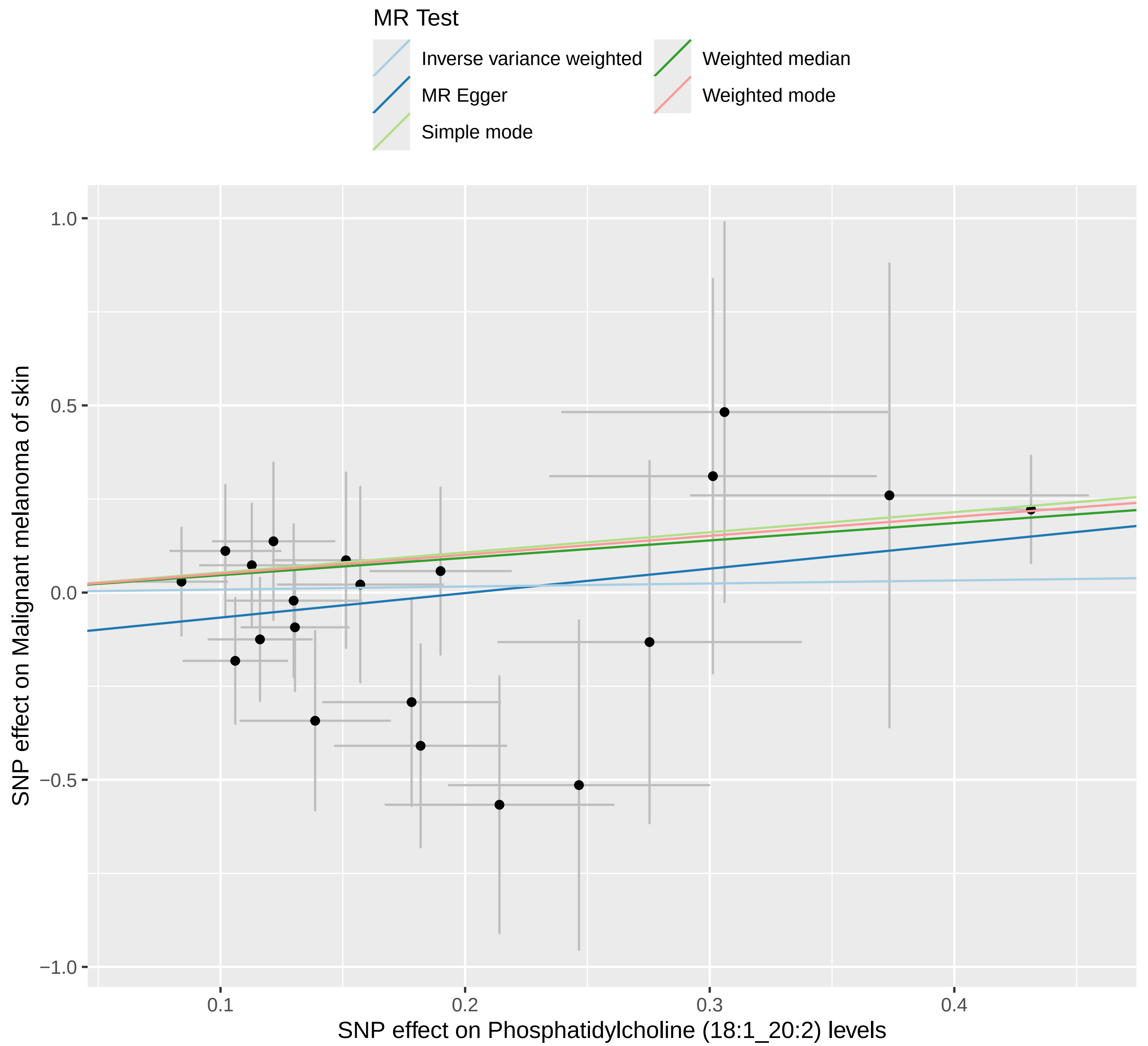
MR Test
Inverse variance weighted
MR Egger
Simple mode
Weighted median
Weighted mode
SNP effect on Malignant melanoma of skin
SNP effect on Phosphatidylcholine (18:1_20:2) levels
1.0
0.5
0.0
−0.5
−1.0
0.1
0.2
0.3
0.4

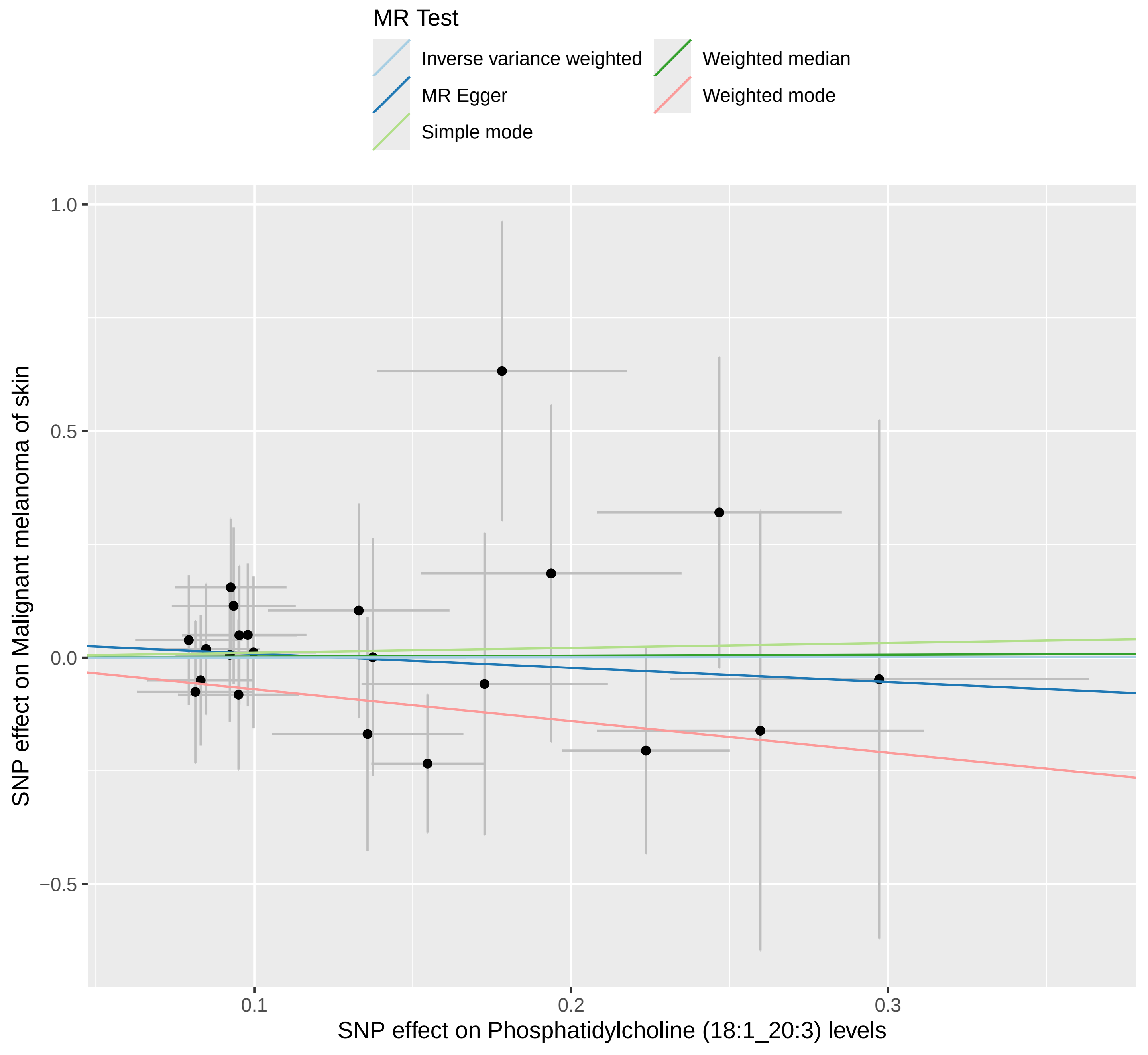
MR Test
Inverse variance weighted
MR Egger
Simple mode
Weighted median
Weighted mode
1.0
0.5
0.0
−0.5
0.1
0.2
0.3
SNP effect on Malignant melanoma of skin
SNP effect on Phosphatidylcholine (18:1_20:3) levels

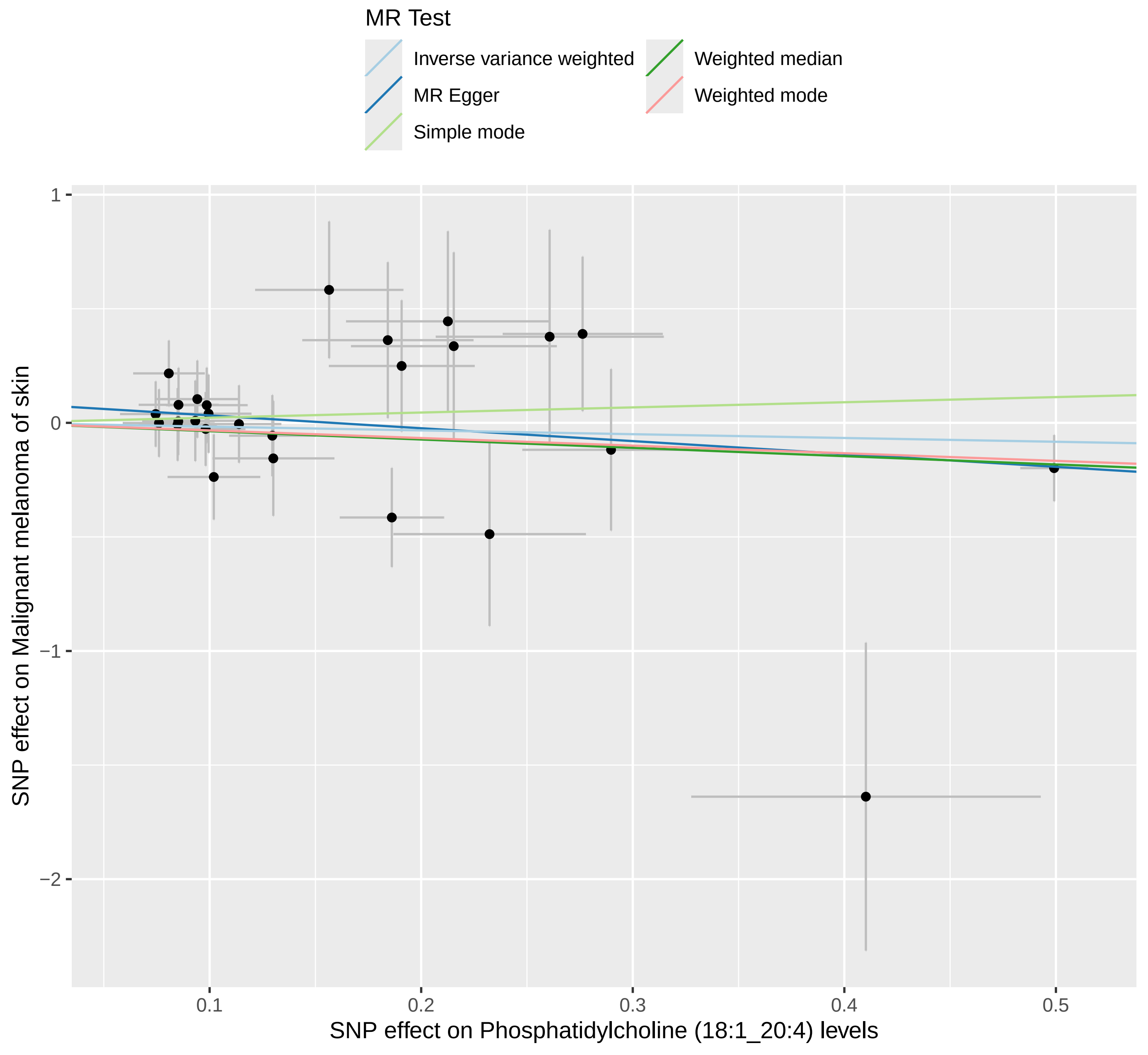
MR Test
Inverse variance weighted
MR Egger
Simple mode
Weighted median
Weighted mode
1
0
−1
−2
0.1
0.2
0.3
0.4
0.5
SNP effect on Malignant melanoma of skin
SNP effect on Phosphatidylcholine (18:1_20:4) levels

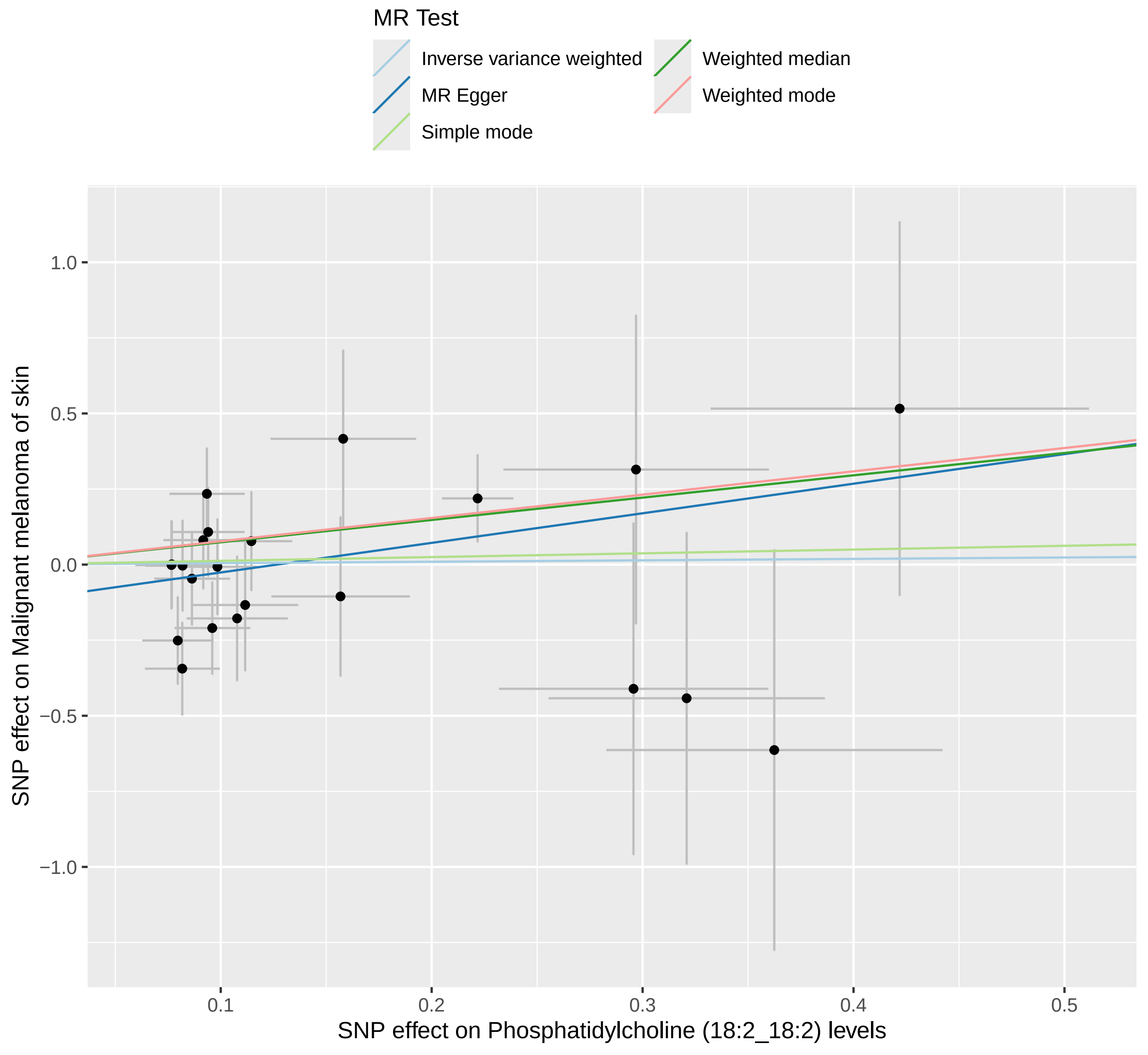

MR Test
Inverse variance weighted
MR Egger
Simple mode
Weighted median
Weighted mode
1.0
0.5
0.0
−0.5
−1.0
0.1
0.2
0.3
0.4
0.5
SNP effect on Malignant melanoma of skin
SNP effect on Phosphatidylcholine (18:2_18:2) levels

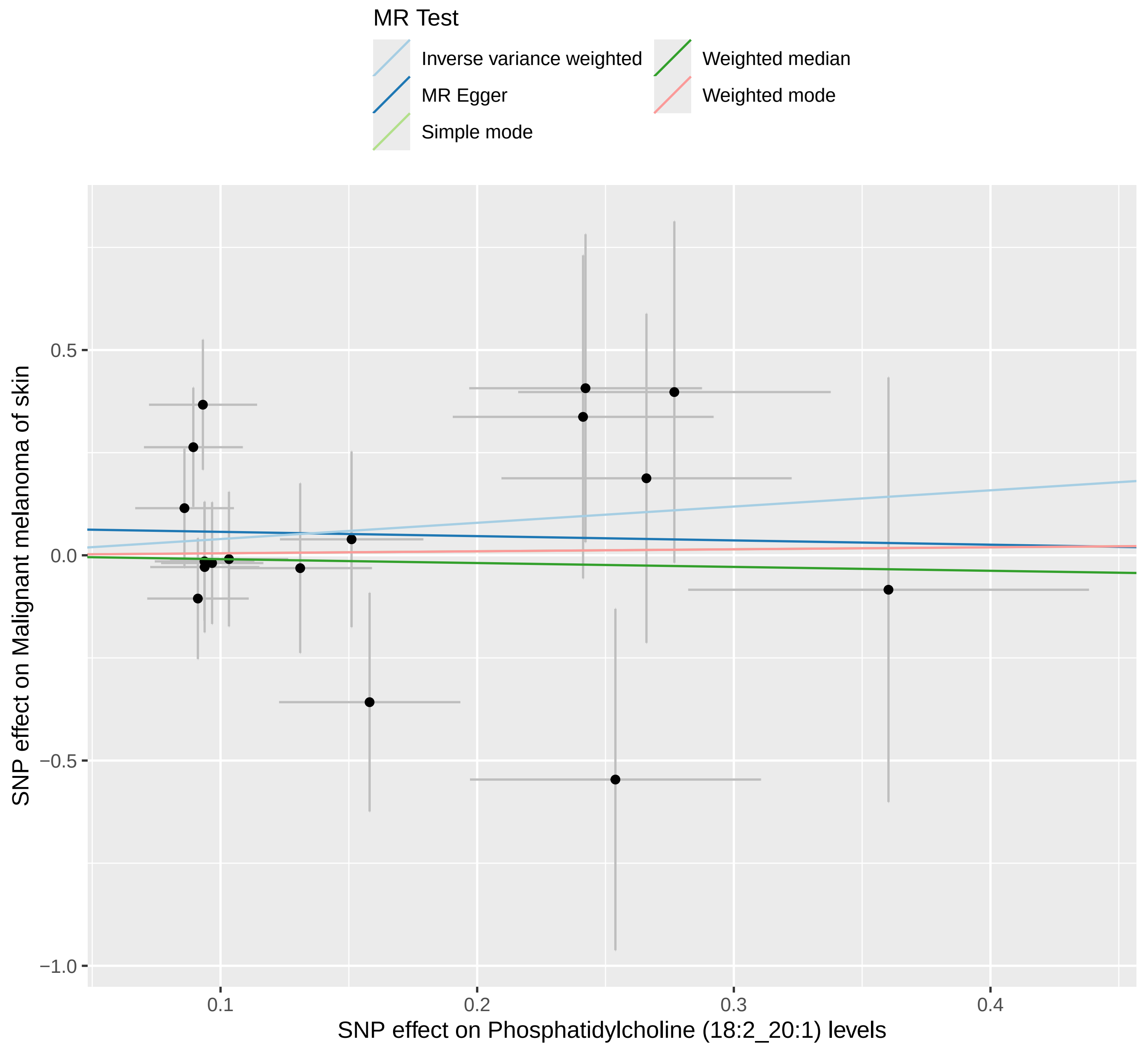

MR Test
Inverse variance weighted
MR Egger
Simple mode
Weighted median
Weighted mode
0.5
0.0
−0.5
−1.0
0.1
0.2
0.3
0.4
SNP effect on Malignant melanoma of skin
SNP effect on Phosphatidylcholine (18:2_20:1) levels

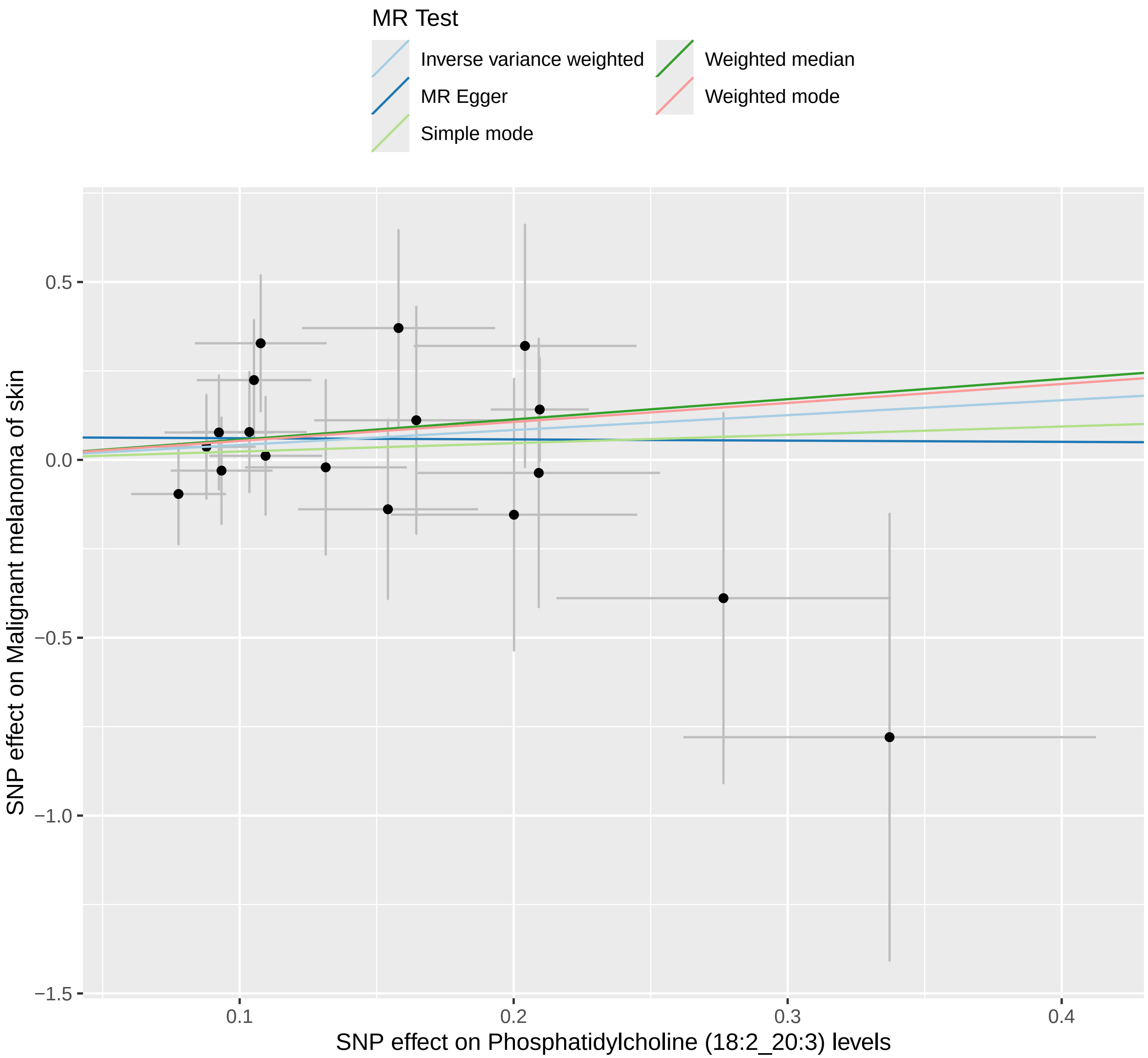
MR Test
Inverse variance weighted
MR Egger
Simple mode
Weighted median
Weighted mode
0.5
0.0
−0.5
−1.0
−1.5
0.1
0.2
0.3
0.4
SNP effect on Malignant melanoma of skin
SNP effect on Phosphatidylcholine (18:2_20:3) levels

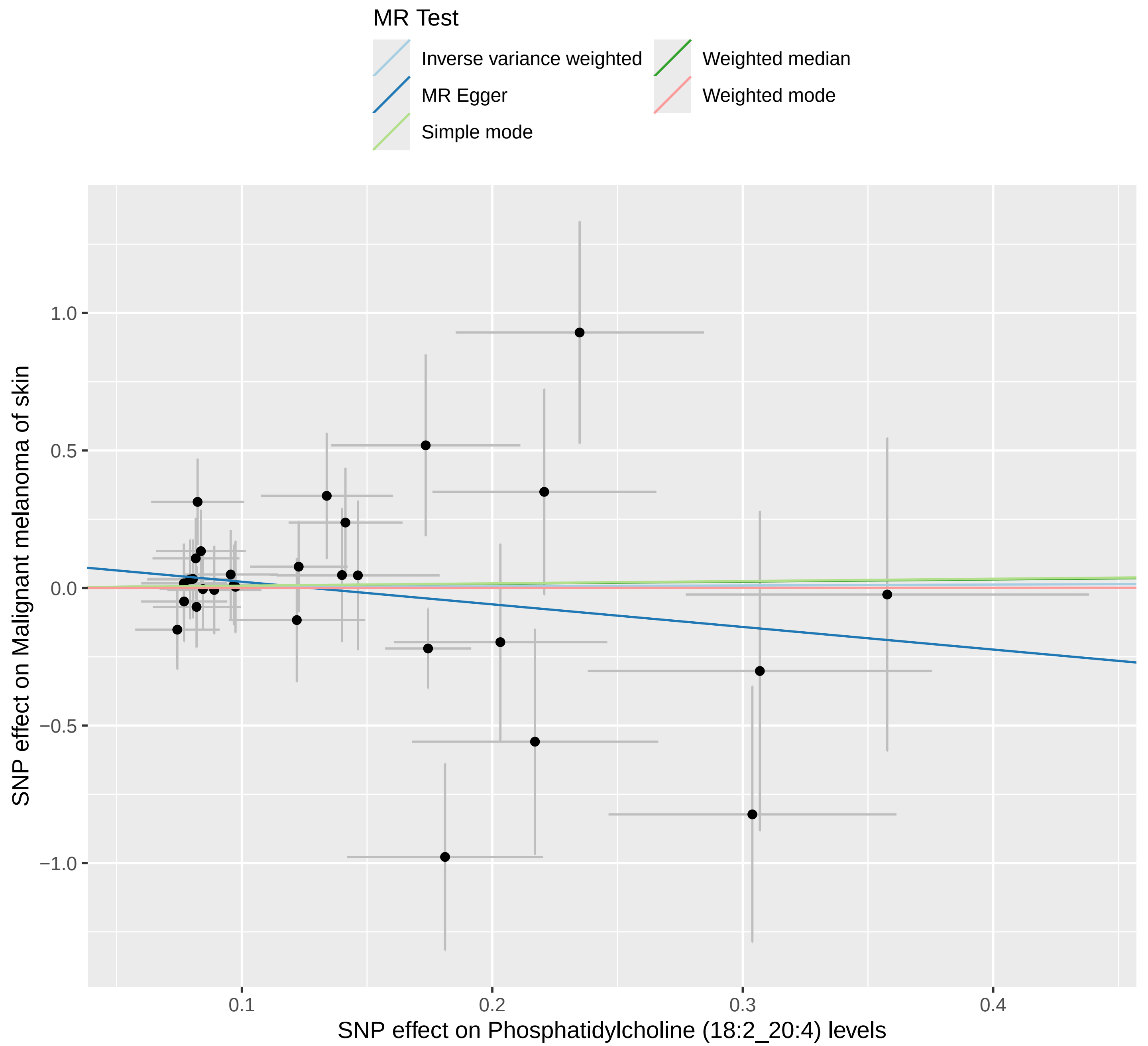

MR Test
Inverse variance weighted
MR Egger
Simple mode
Weighted median
Weighted mode
1.0
0.5
0.0
−0.5
−1.0
SNP effect on Malignant melanoma of skin
0.1
0.2
0.3
0.4
SNP effect on Phosphatidylcholine (18:2_20:4) levels

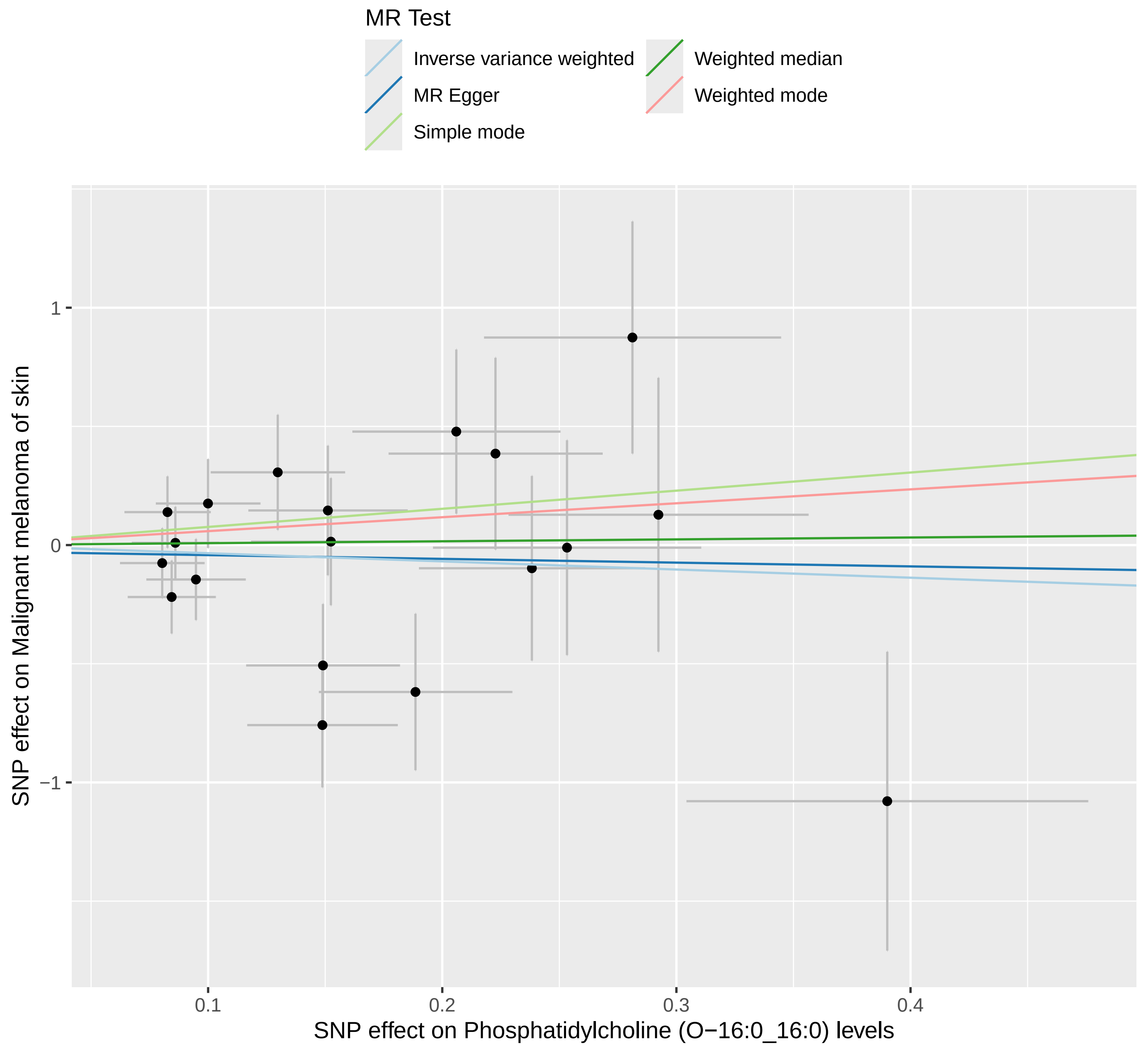

MR Test
Inverse variance weighted
MR Egger
Simple mode
Weighted median
Weighted mode
1
0
−1
0.1
0.2
0.3
0.4
SNP effect on Malignant melanoma of skin
SNP effect on Phosphatidylcholine (O−16:0_16:0) levels

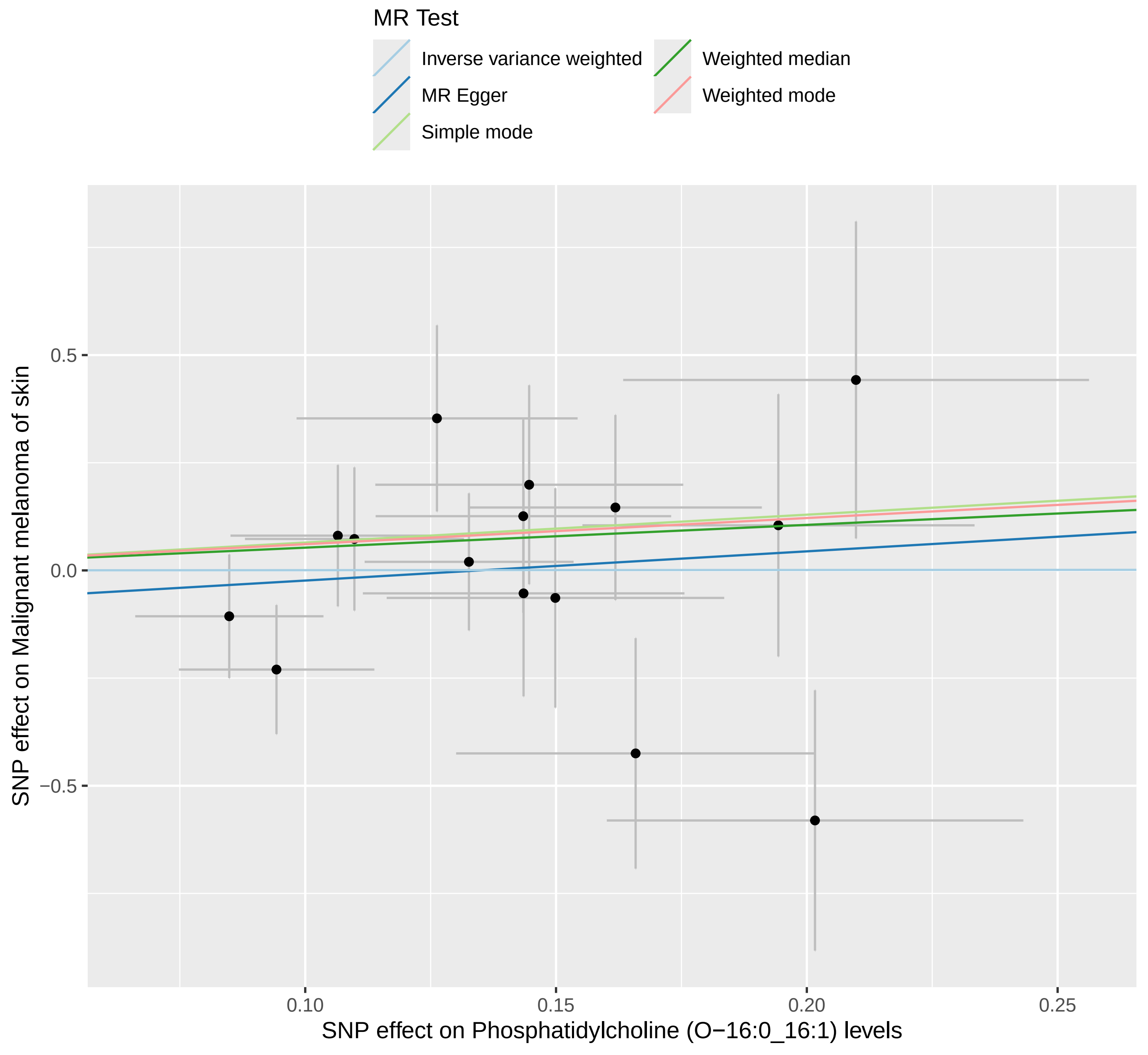
MR Test
Inverse variance weighted
MR Egger
Simple mode
Weighted median
Weighted mode
0.5
0.0
−0.5
0.10
0.15
0.20
0.25
SNP effect on Malignant melanoma of skin
SNP effect on Phosphatidylcholine (O−16:0_16:1) levels

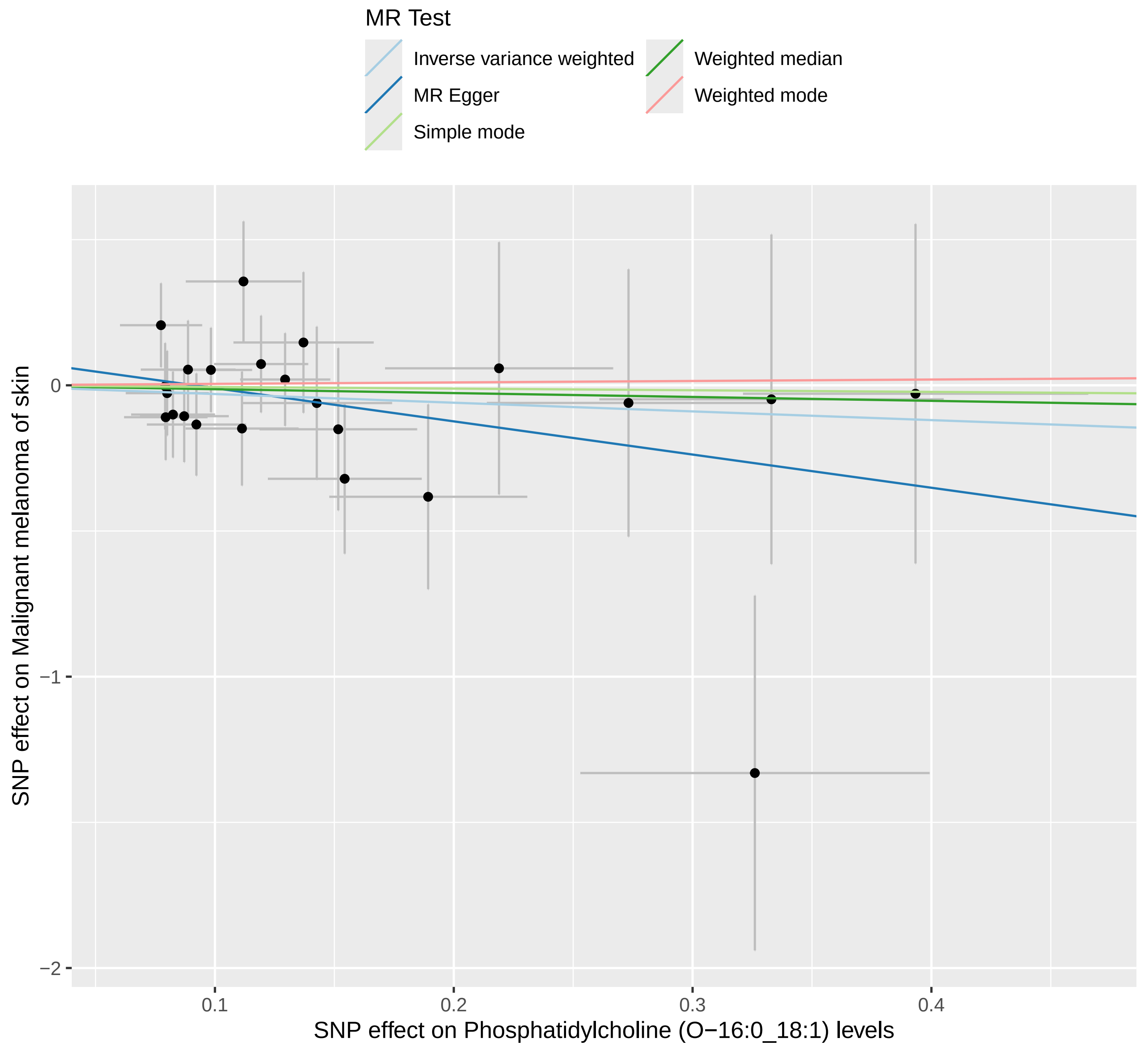
MR Test
Inverse variance weighted
MR Egger
Simple mode
Weighted median
Weighted mode
0
−1
−2
0.1
0.2
0.3
0.4
SNP effect on Malignant melanoma of skin
SNP effect on Phosphatidylcholine (O−16:0_18:1) levels

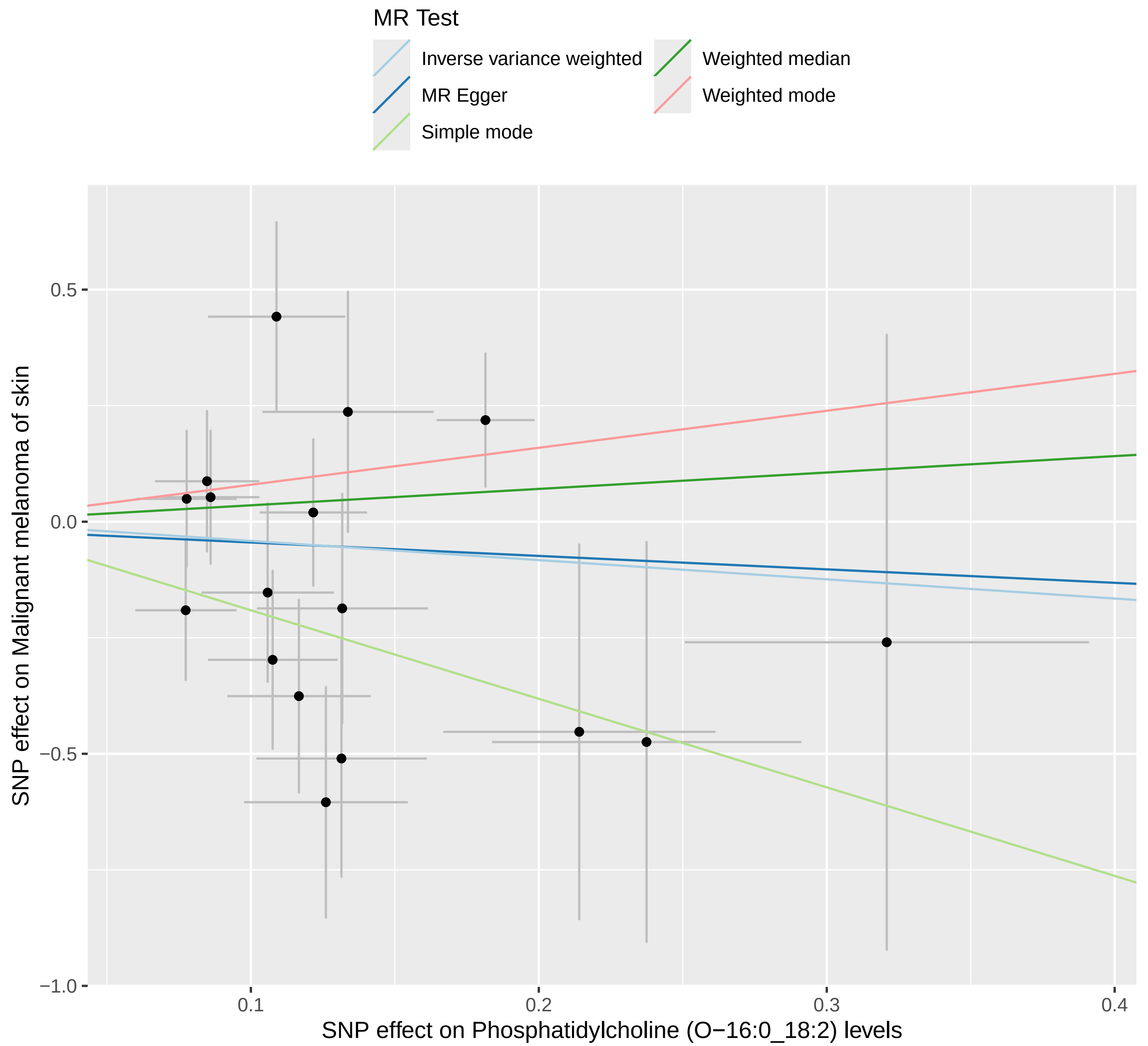
MR Test
Inverse variance weighted
MR Egger
Simple mode
Weighted median
Weighted mode
0.5
0.0
−0.5
−1.0
0.1
0.2
0.3
0.4
SNP effect on Malignant melanoma of skin
SNP effect on Phosphatidylcholine (O−16:0_18:2) levels

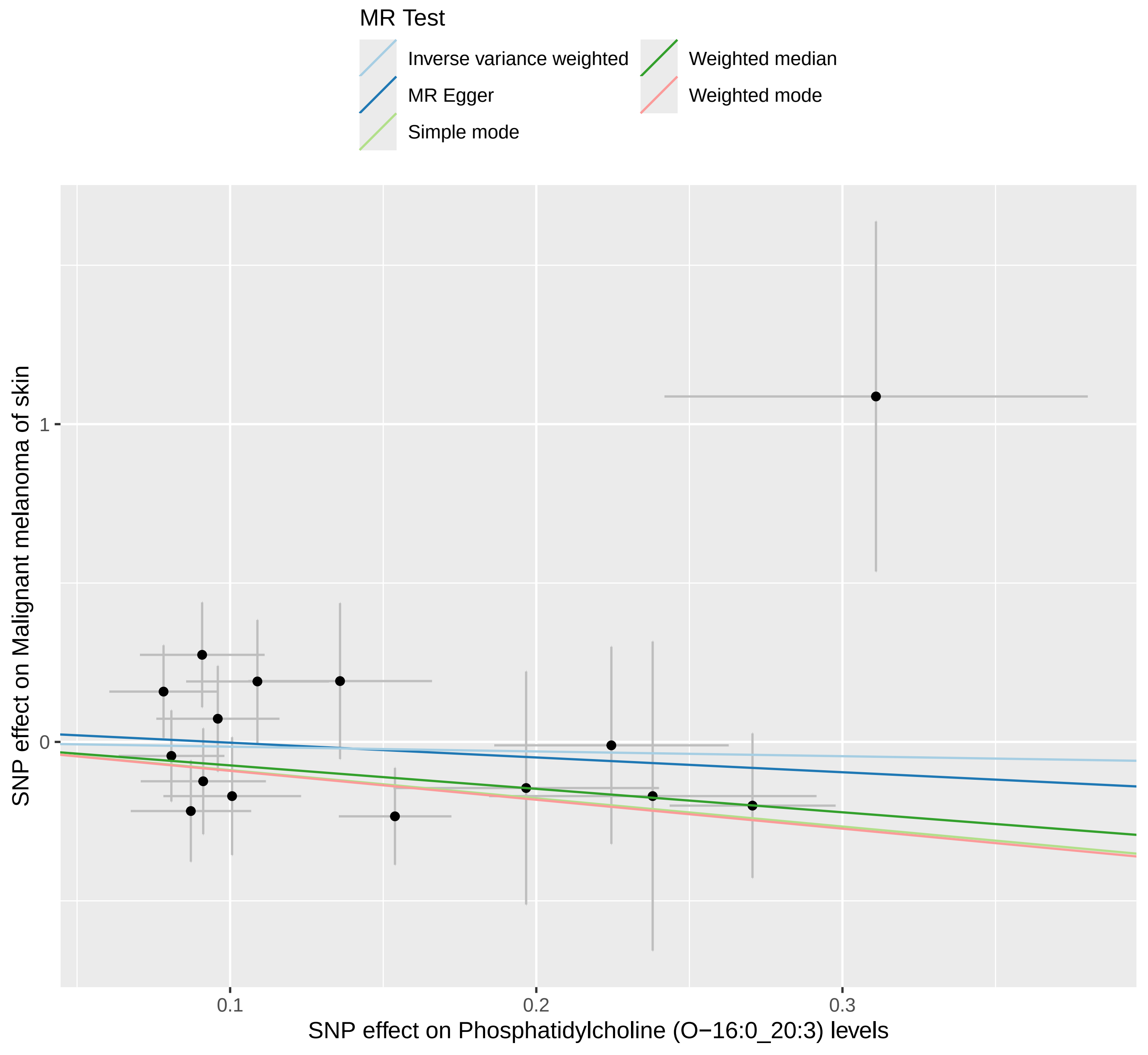
MR Test
Inverse variance weighted
MR Egger
Simple mode
Weighted median
Weighted mode
SNP effect on Malignant melanoma of skin
1
0
0.1
0.2
0.3
SNP effect on Phosphatidylcholine (O−16:0_20:3) levels

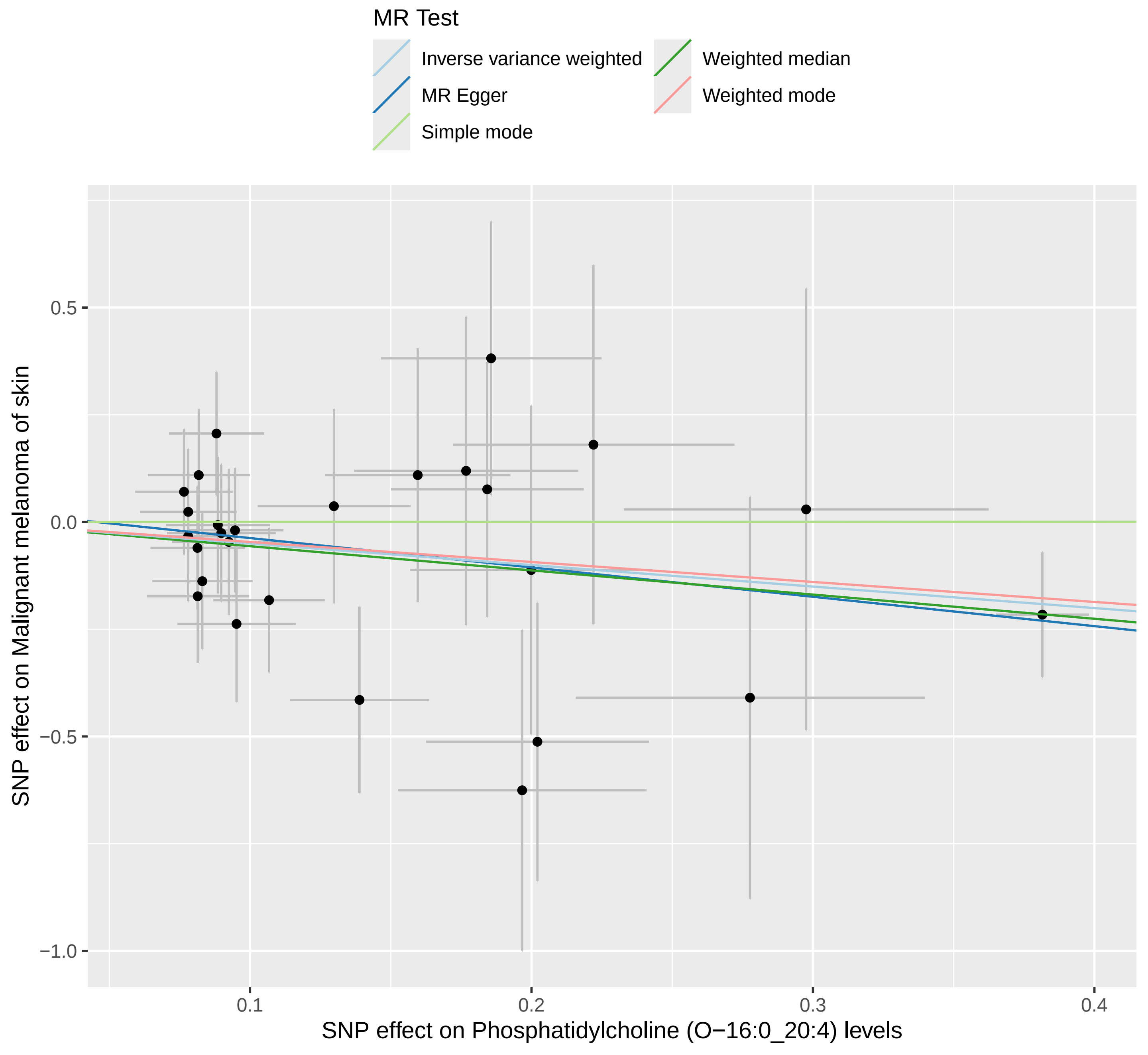
MR Test
Inverse variance weighted
MR Egger
Simple mode
Weighted median
Weighted mode
0.5
0.0
−0.5
−1.0
0.1
0.2
0.3
0.4
SNP effect on Malignant melanoma of skin
SNP effect on Phosphatidylcholine (O−16:0_20:4) levels

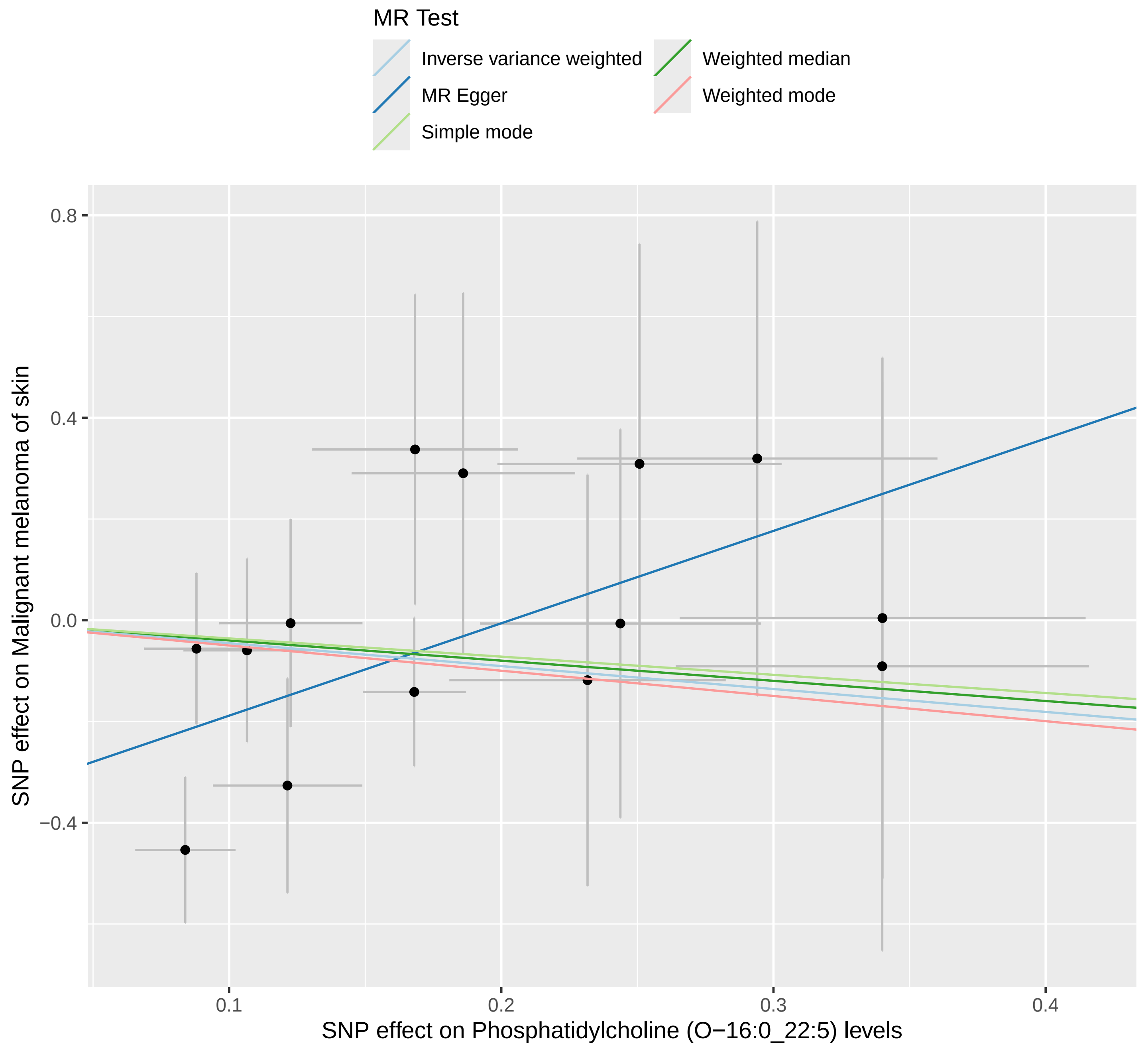
MR Test
Inverse variance weighted
MR Egger
Simple mode
Weighted median
Weighted mode
0.8
0.4
0.0
−0.4
0.1
0.2
0.3
0.4
SNP effect on Malignant melanoma of skin
SNP effect on Phosphatidylcholine (O−16:0_22:5) levels

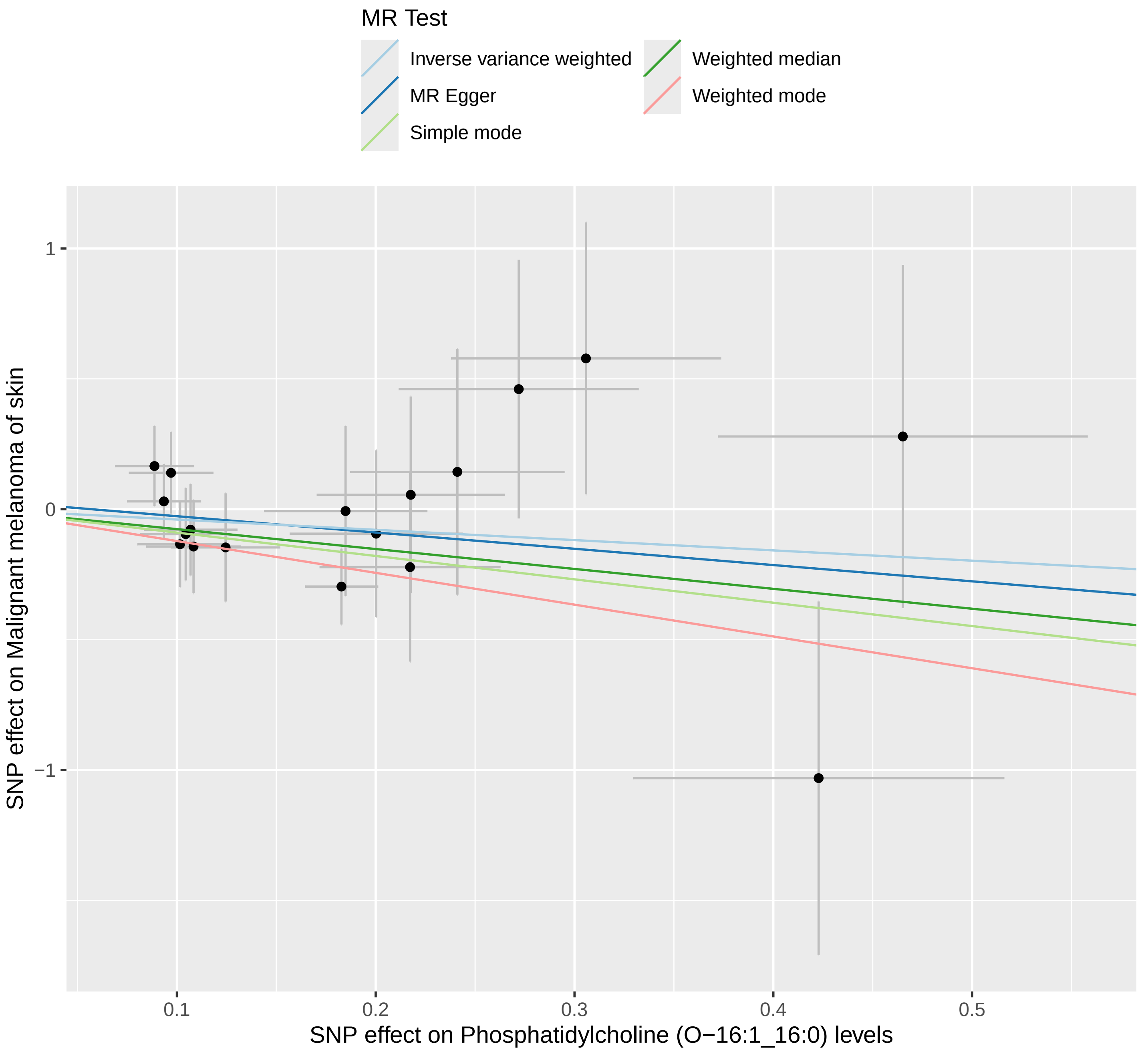
MR Test
Inverse variance weighted
MR Egger
Simple mode
Weighted median
Weighted mode
1
0
−1
0.1
0.2
0.3
0.4
0.5
SNP effect on Malignant melanoma of skin
SNP effect on Phosphatidylcholine (O−16:1_16:0) levels

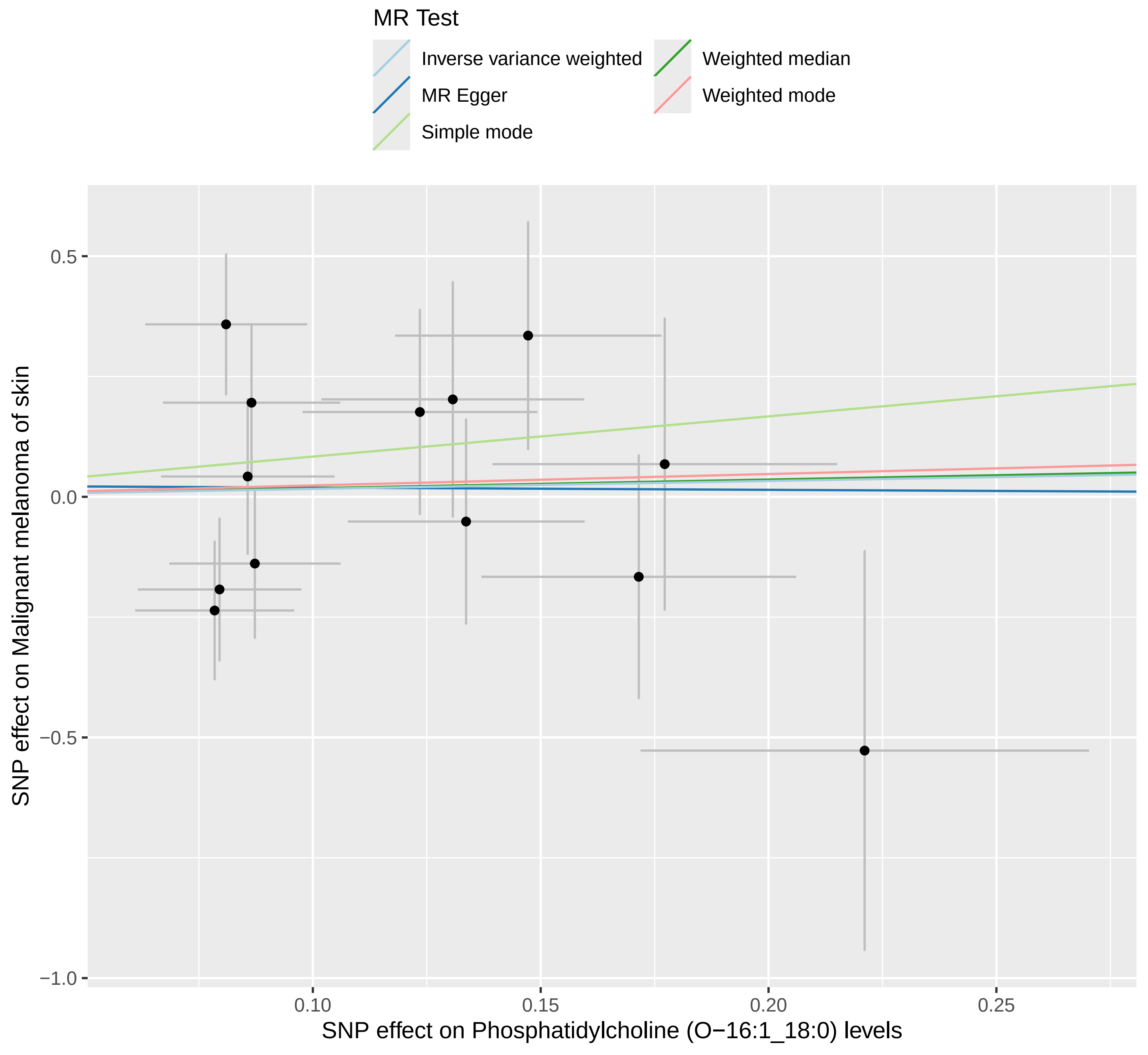
MR Test
Inverse variance weighted
MR Egger
Simple mode
Weighted median
Weighted mode
0.5
0.0
−0.5
−1.0
0.10
0.15
0.20
0.25
SNP effect on Phosphatidylcholine (O−16:1_18:0) levels
SNP effect on Malignant melanoma of skin

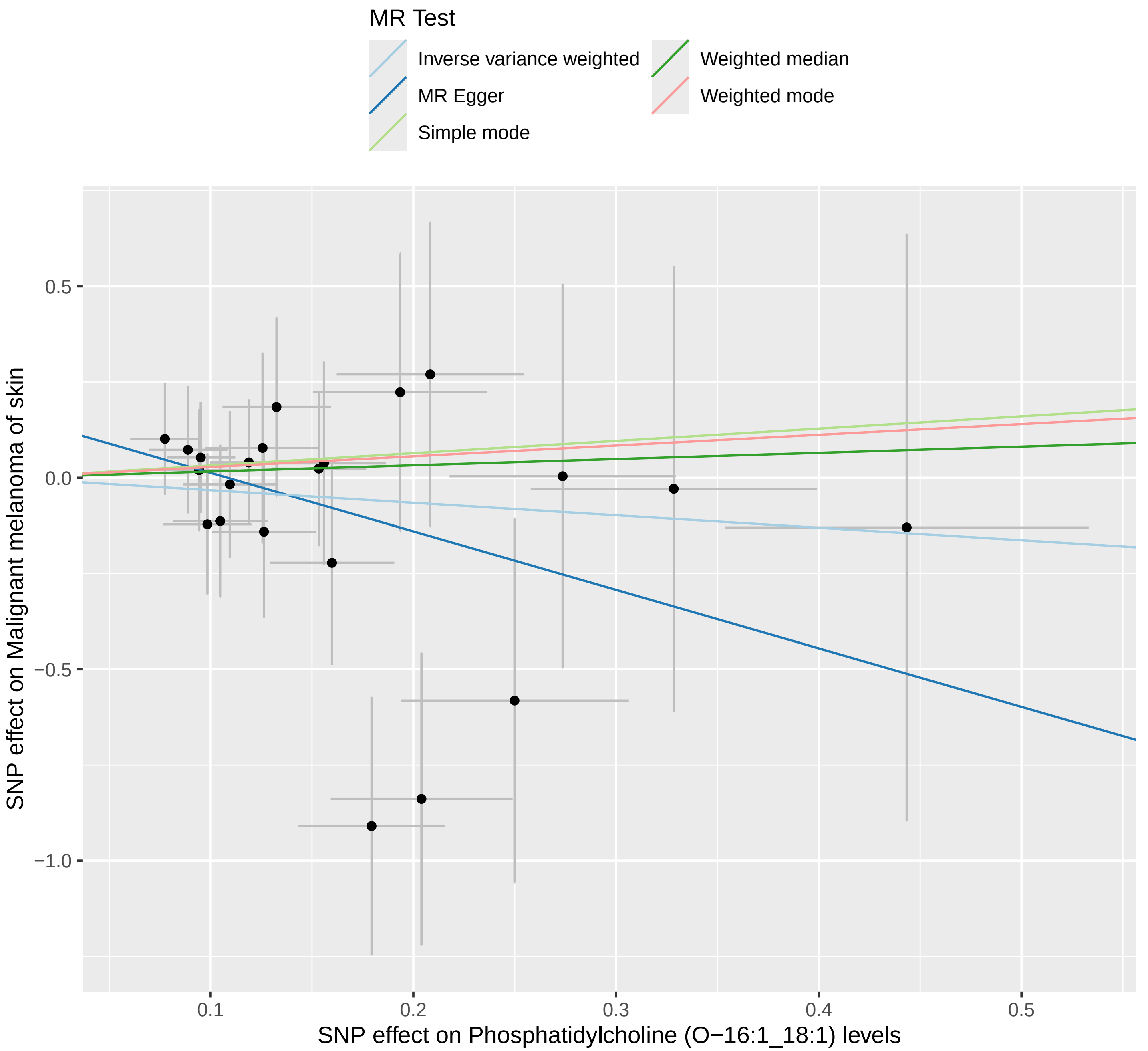

MR Test
Inverse variance weighted
MR Egger
Simple mode
Weighted median
Weighted mode
0.5
0.0
−0.5
−1.0
0.1
0.2
0.3
0.4
0.5
SNP effect on Phosphatidylcholine (O−16:1_18:1) levels
SNP effect on Malignant melanoma of skin

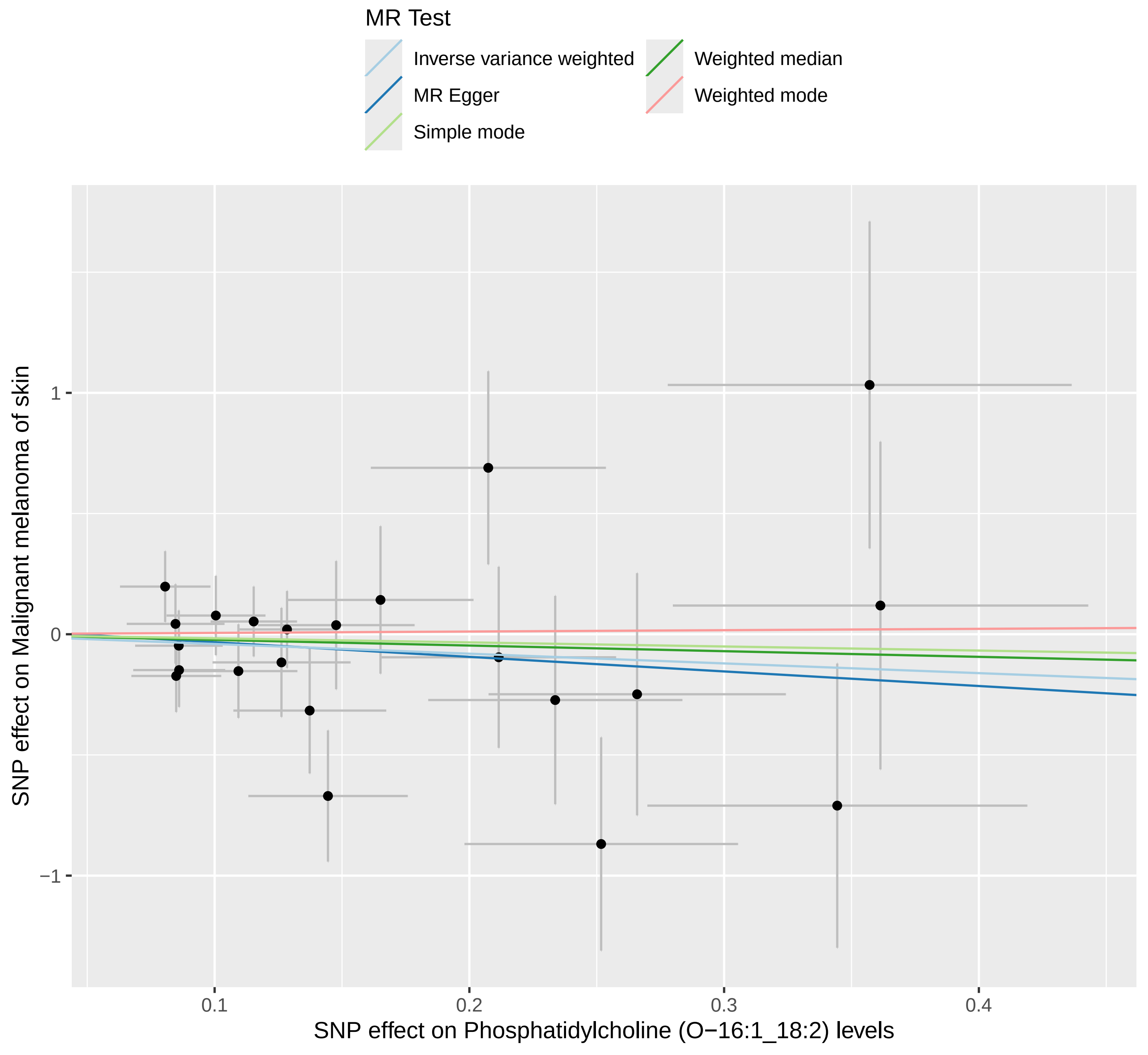
MR Test
Inverse variance weighted
MR Egger
Simple mode
Weighted median
Weighted mode
SNP effect on Malignant melanoma of skin
1
0
−1
0.1
0.2
0.3
0.4
SNP effect on Phosphatidylcholine (O−16:1_18:2) levels

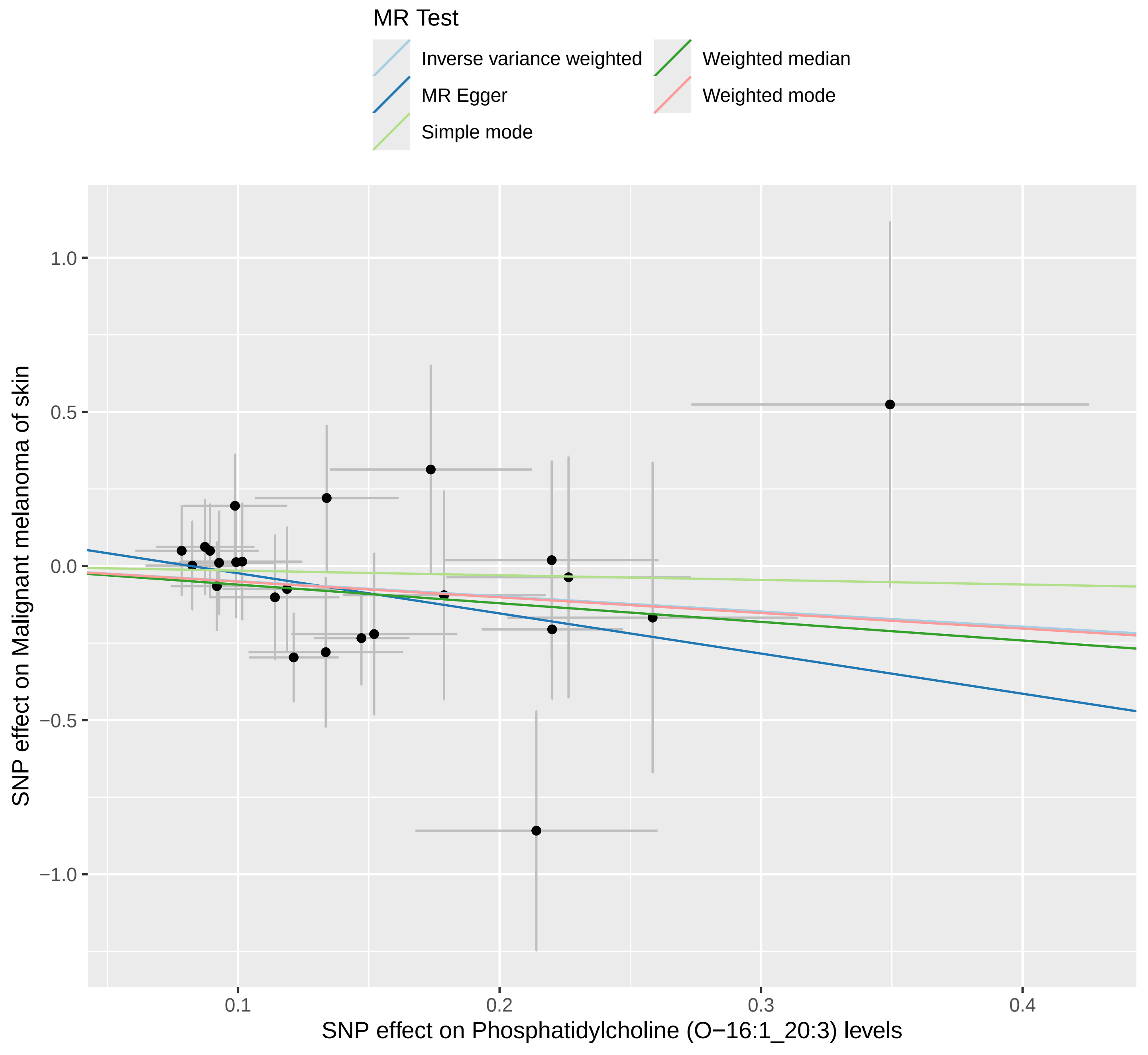

MR Test
Inverse variance weighted
MR Egger
Simple mode
Weighted median
Weighted mode
SNP effect on Malignant melanoma of skin
1.0
0.5
0.0
−0.5
−1.0
0.1
0.2
0.3
0.4
SNP effect on Phosphatidylcholine (O−16:1_20:3) levels

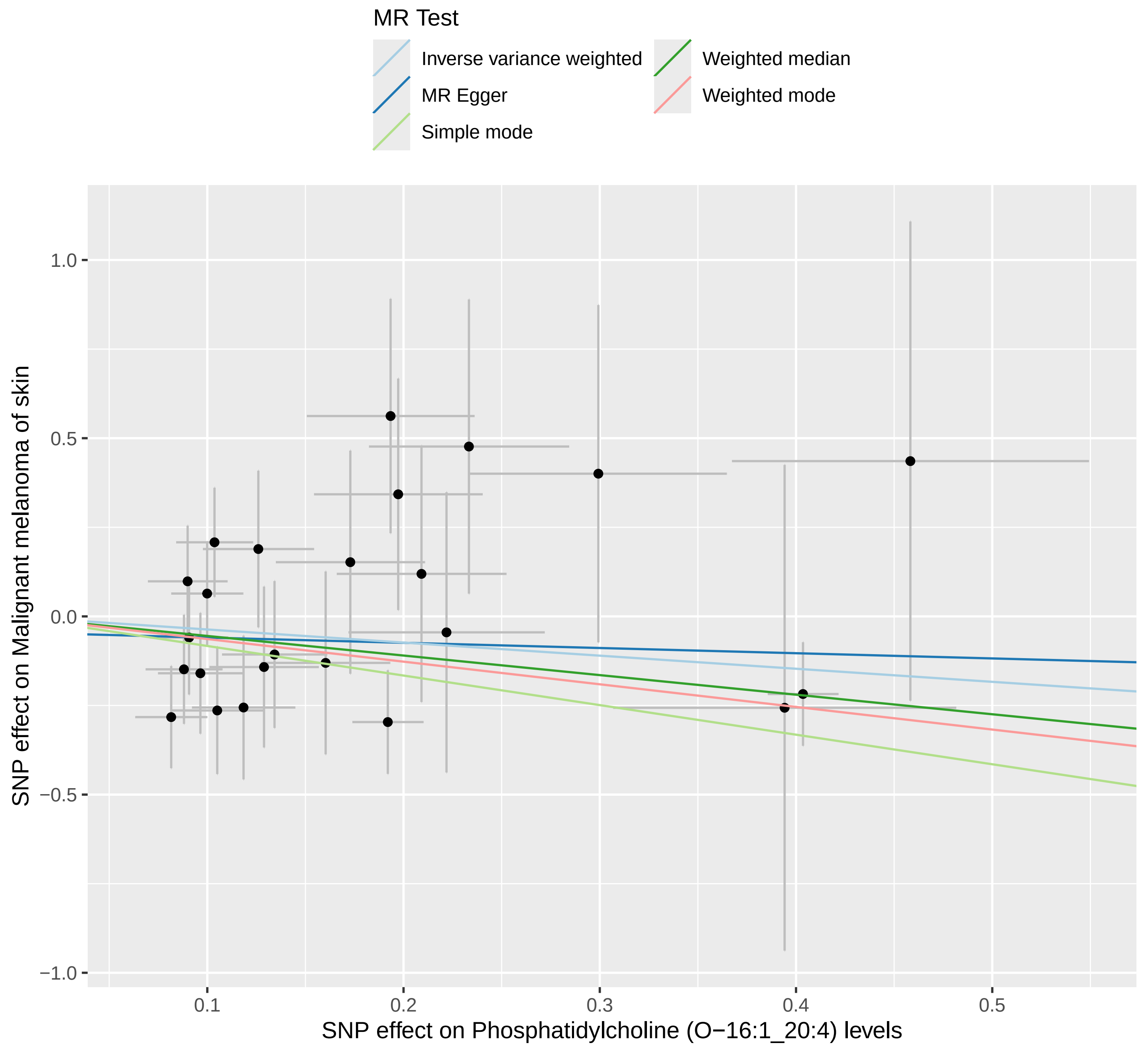

MR Test
Inverse variance weighted
MR Egger
Simple mode
Weighted median
Weighted mode
1.0
0.5
0.0
−0.5
−1.0
0.1
0.2
0.3
0.4
0.5
SNP effect on Malignant melanoma of skin
SNP effect on Phosphatidylcholine (O−16:1_20:4) levels

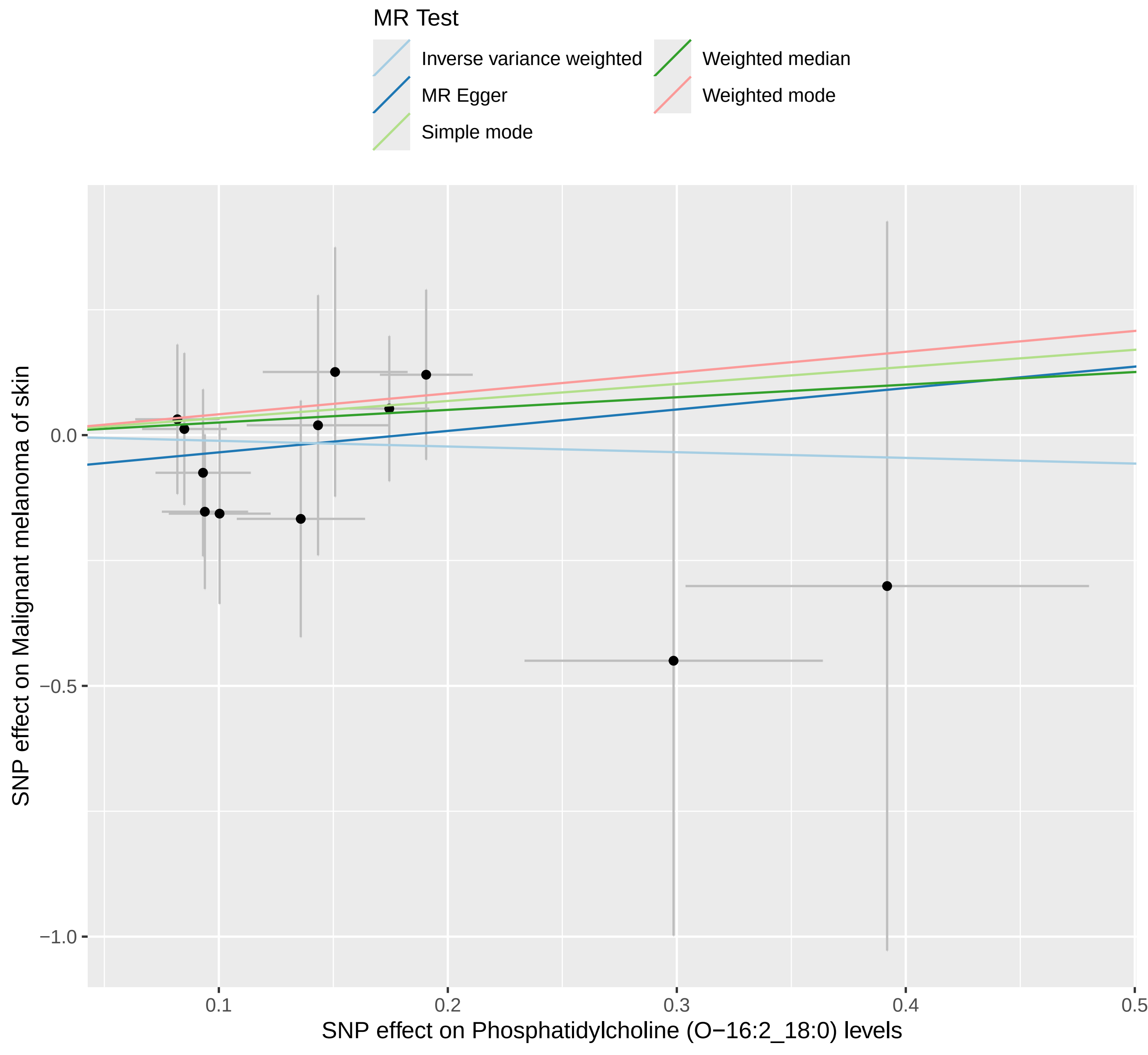
MR Test
Inverse variance weighted
MR Egger
Simple mode
Weighted median
Weighted mode
0.0
−0.5
−1.0
0.1
0.2
0.3
0.4
0.5
SNP effect on Phosphatidylcholine (O−16:2_18:0) levels
SNP effect on Malignant melanoma of skin

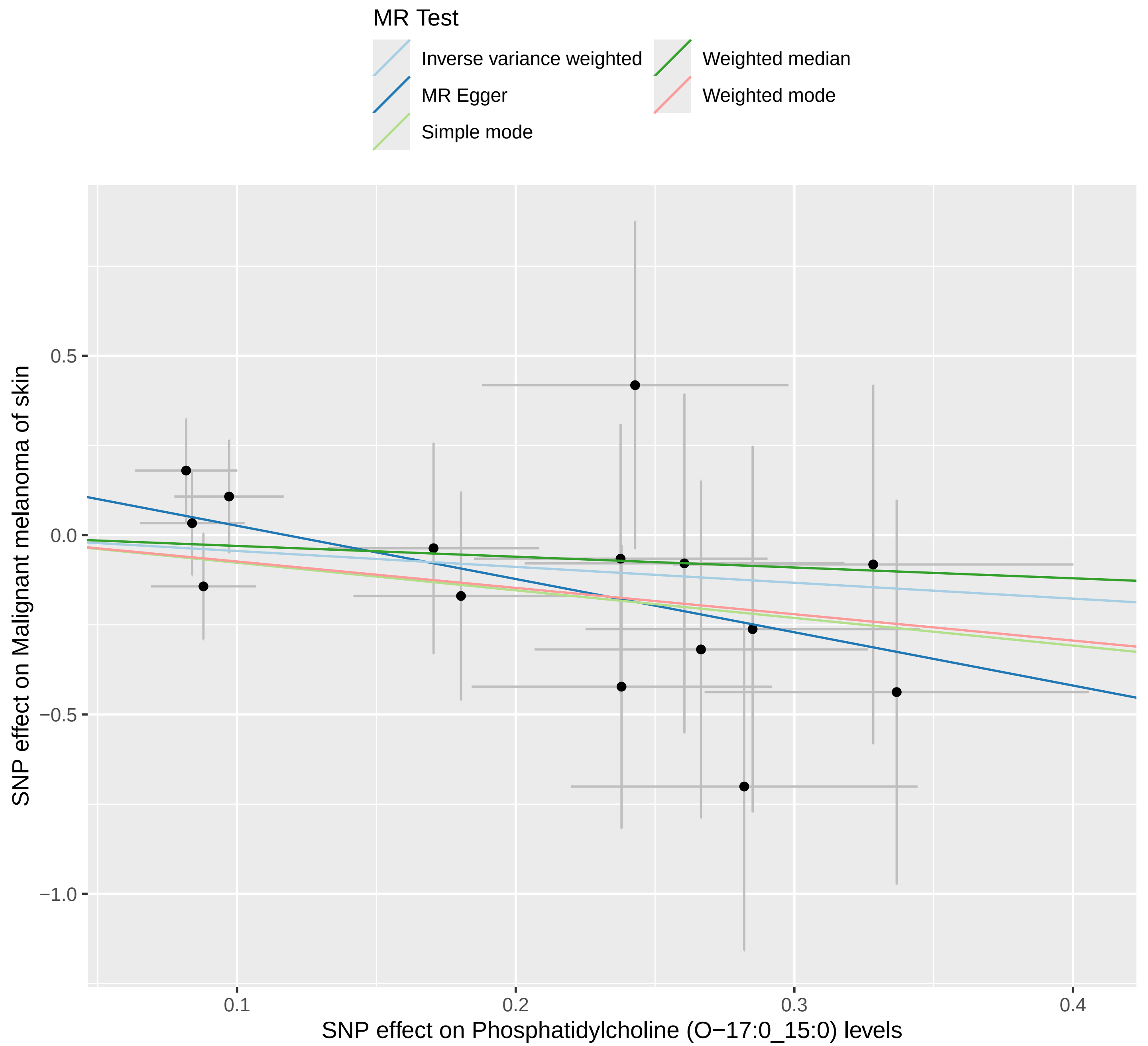
MR Test
Inverse variance weighted
MR Egger
Simple mode
Weighted median
Weighted mode
0.5
0.0
−0.5
−1.0
0.1
0.2
0.3
0.4
SNP effect on Malignant melanoma of skin
SNP effect on Phosphatidylcholine (O−17:0_15:0) levels

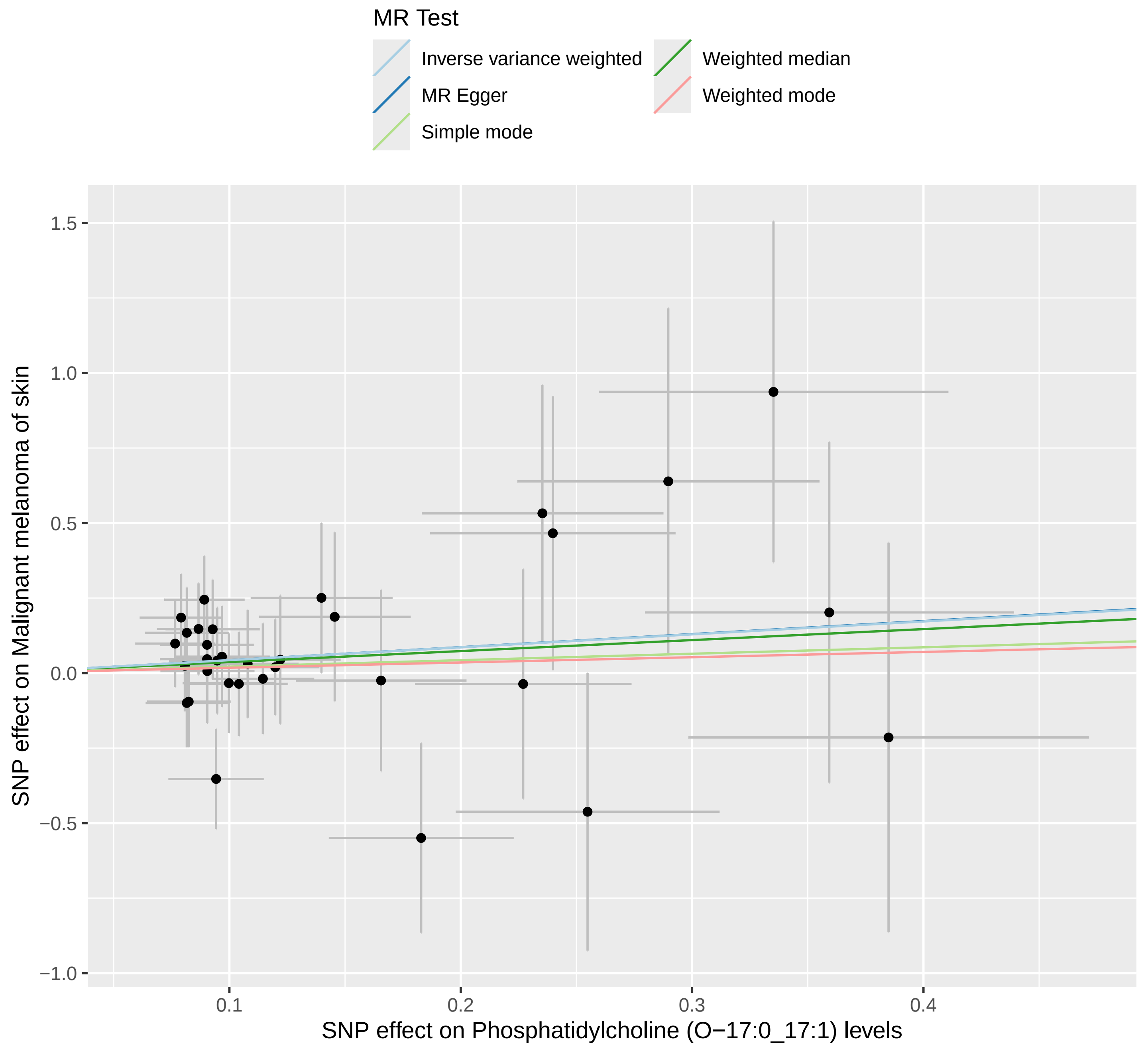

MR Test
Inverse variance weighted
MR Egger
Simple mode
Weighted median
Weighted mode
1.5
1.0
0.5
0.0
−0.5
−1.0
0.1
0.2
0.3
0.4
SNP effect on Malignant melanoma of skin
SNP effect on Phosphatidylcholine (O−17:0_17:1) levels

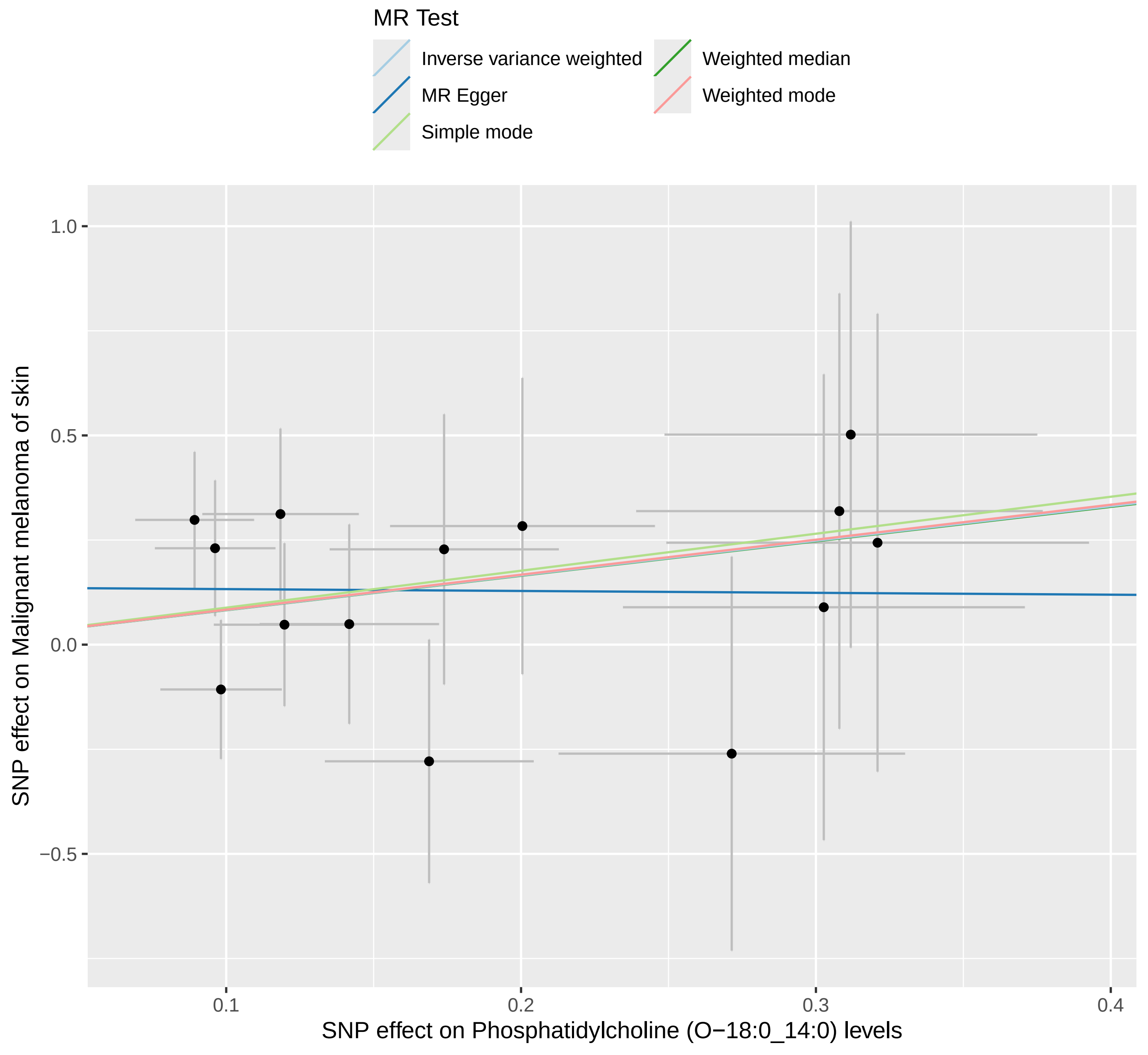
MR Test
Inverse variance weighted
MR Egger
Simple mode
Weighted median
Weighted mode
1.0
0.5
0.0
−0.5
0.1
0.2
0.3
0.4
SNP effect on Malignant melanoma of skin
SNP effect on Phosphatidylcholine (O−18:0_14:0) levels

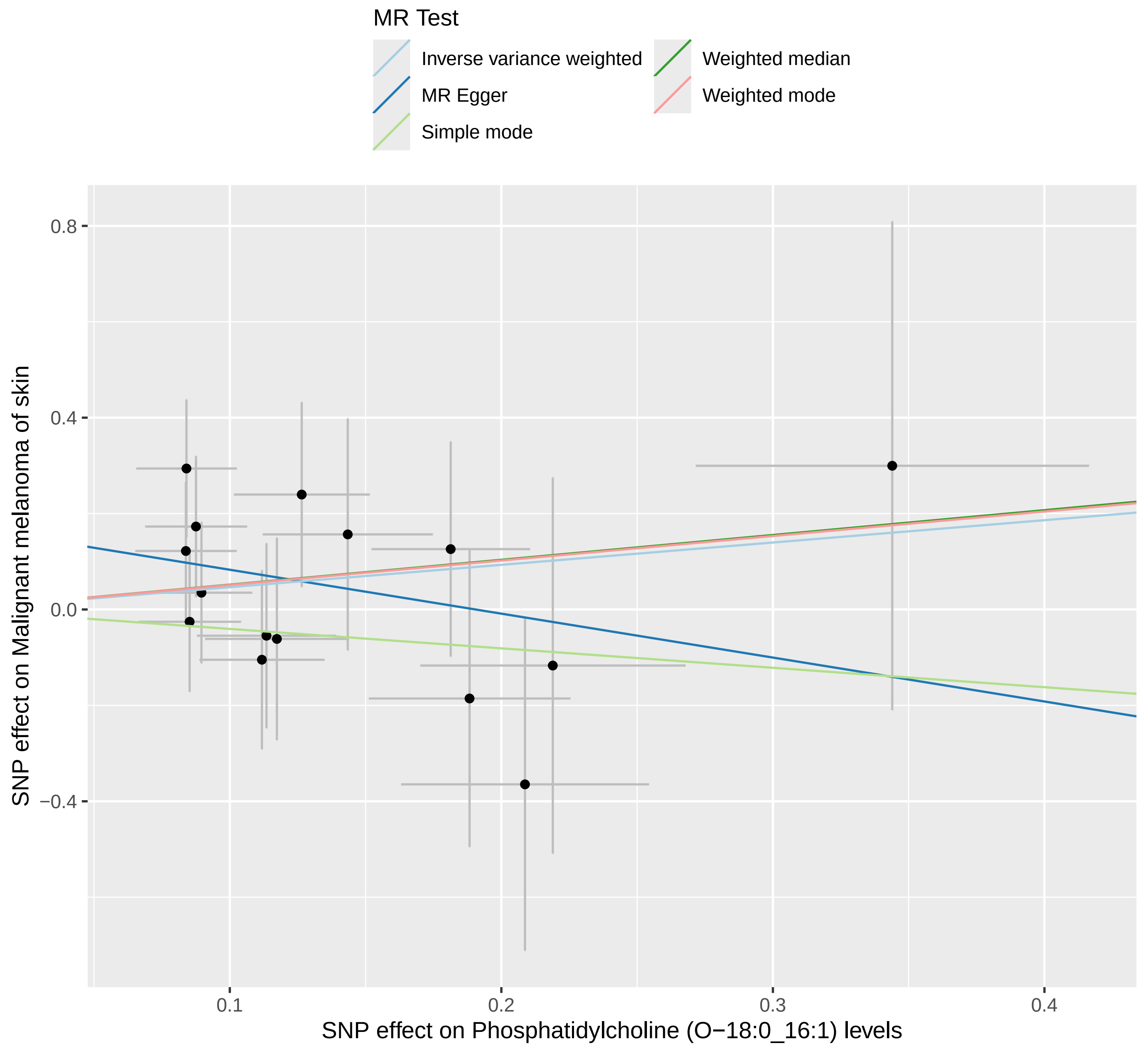
MR Test
Inverse variance weighted
MR Egger
Simple mode
Weighted median
Weighted mode
0.8
0.4
0.0
−0.4
0.1
0.2
0.3
0.4
SNP effect on Malignant melanoma of skin
SNP effect on Phosphatidylcholine (O−18:0_16:1) levels

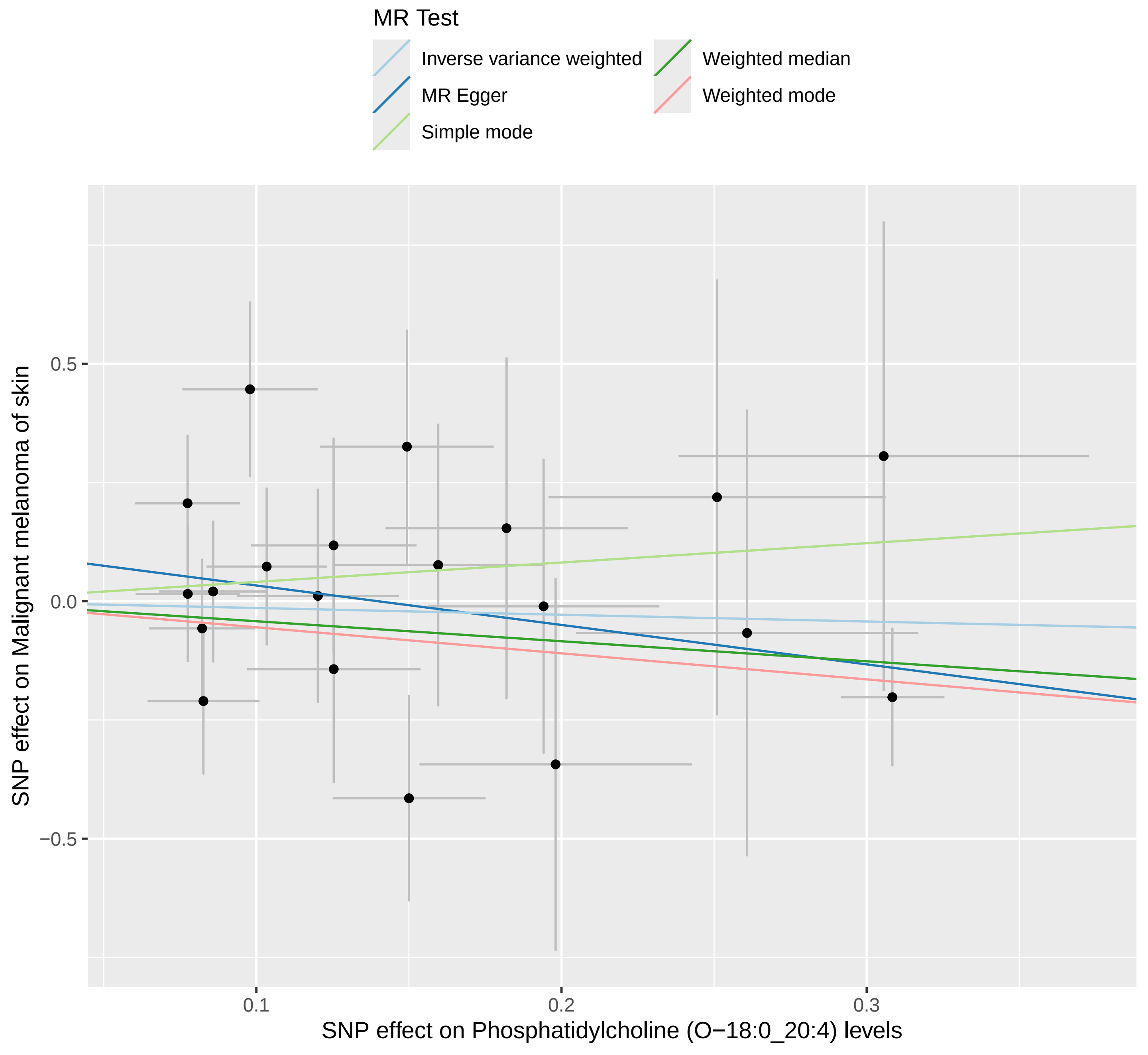
MR Test
Inverse variance weighted
MR Egger
Simple mode
Weighted median
Weighted mode
0.5
0.0
−0.5
0.1
0.2
0.3
SNP effect on Malignant melanoma of skin
SNP effect on Phosphatidylcholine (O−18:0_20:4) levels

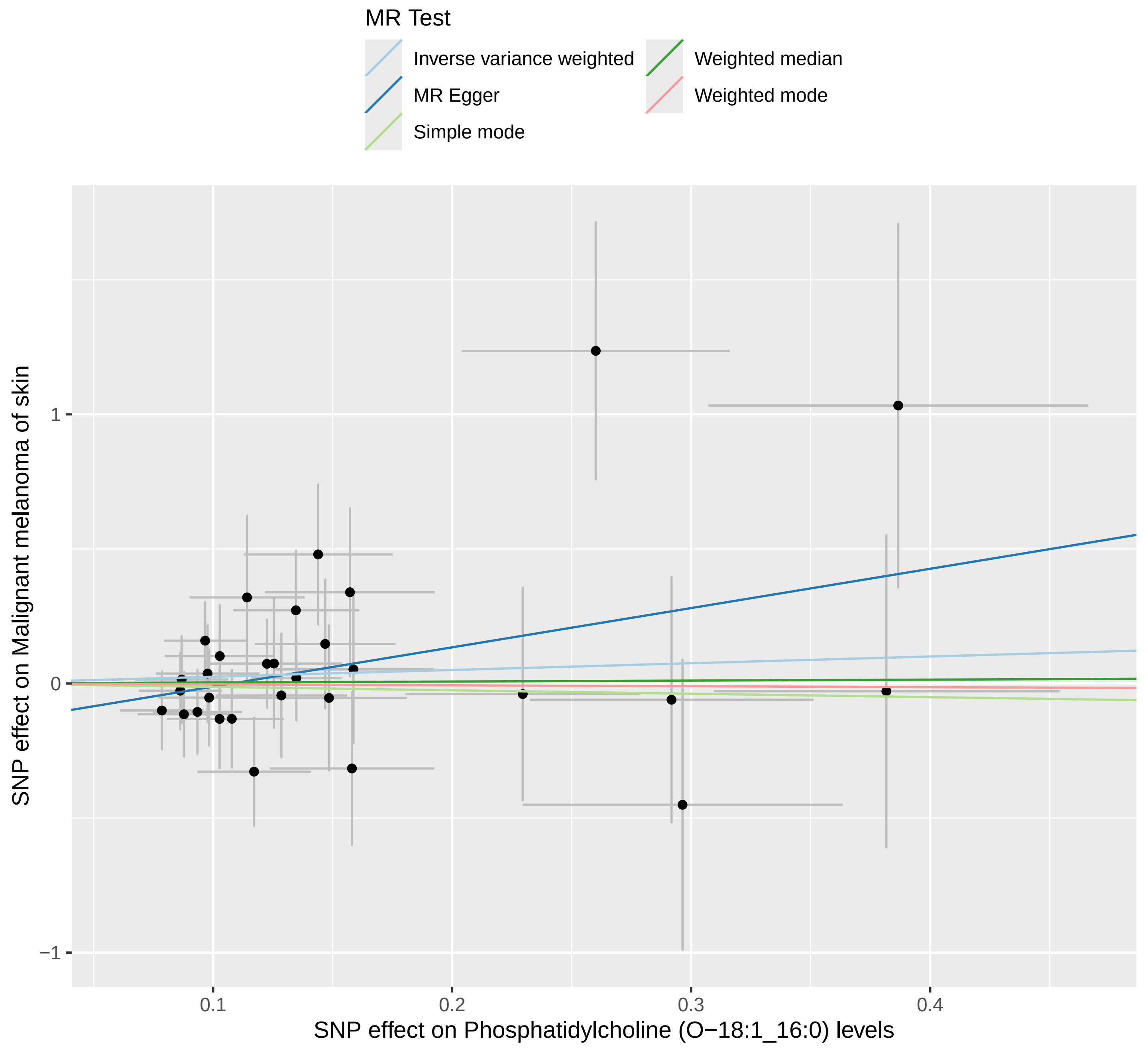

MR Test
Inverse variance weighted
MR Egger
Simple mode
Weighted median
Weighted mode
SNP effect on Malignant melanoma of skin
1
0
−1
0.1
0.2
0.3
0.4
SNP effect on Phosphatidylcholine (O−18:1_16:0) levels

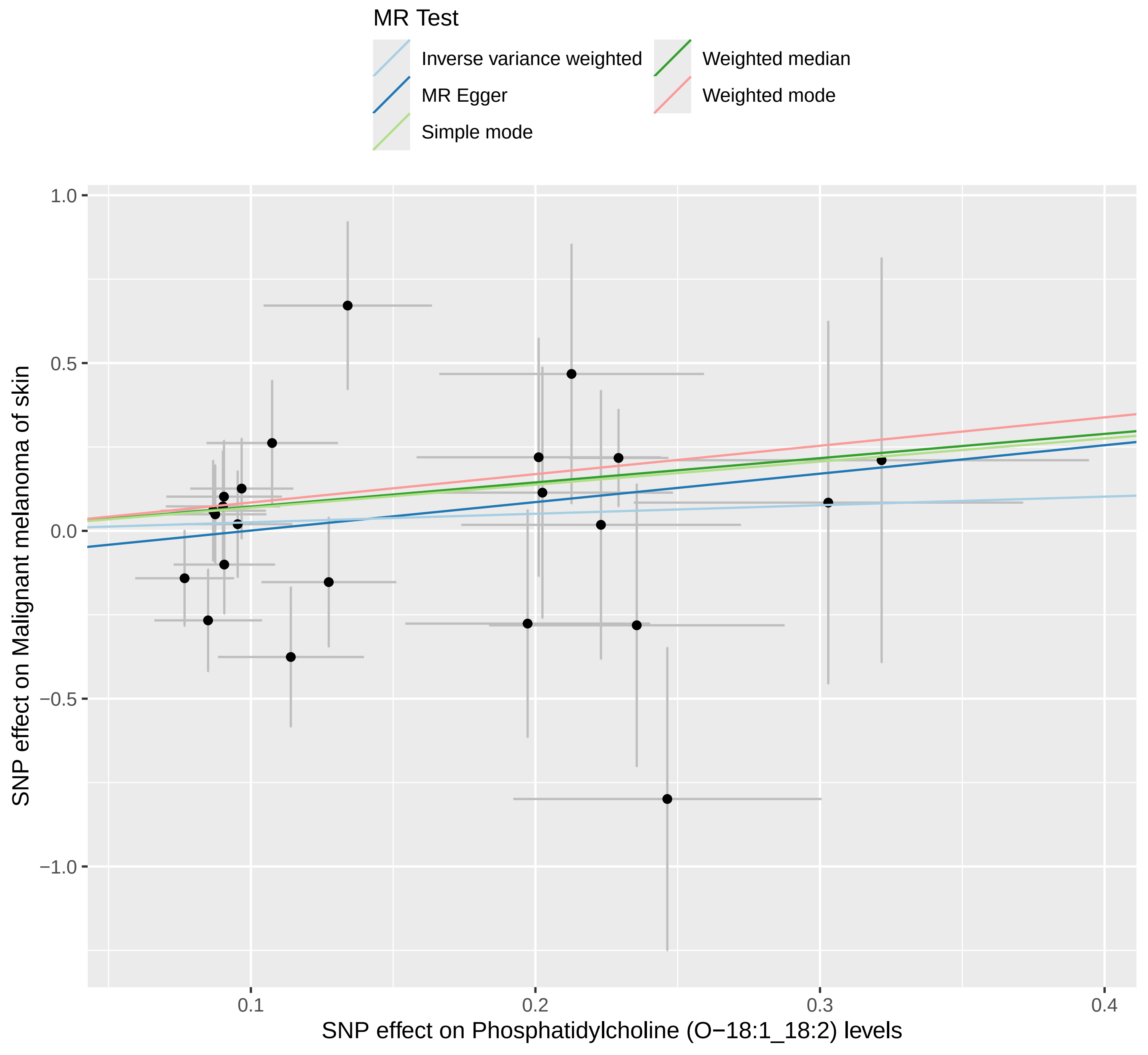
MR Test
Inverse variance weighted
MR Egger
Simple mode
Weighted median
Weighted mode
1.0
0.5
0.0
−0.5
−1.0
SNP effect on Malignant melanoma of skin
0.1
0.2
0.3
0.4
SNP effect on Phosphatidylcholine (O−18:1_18:2) levels

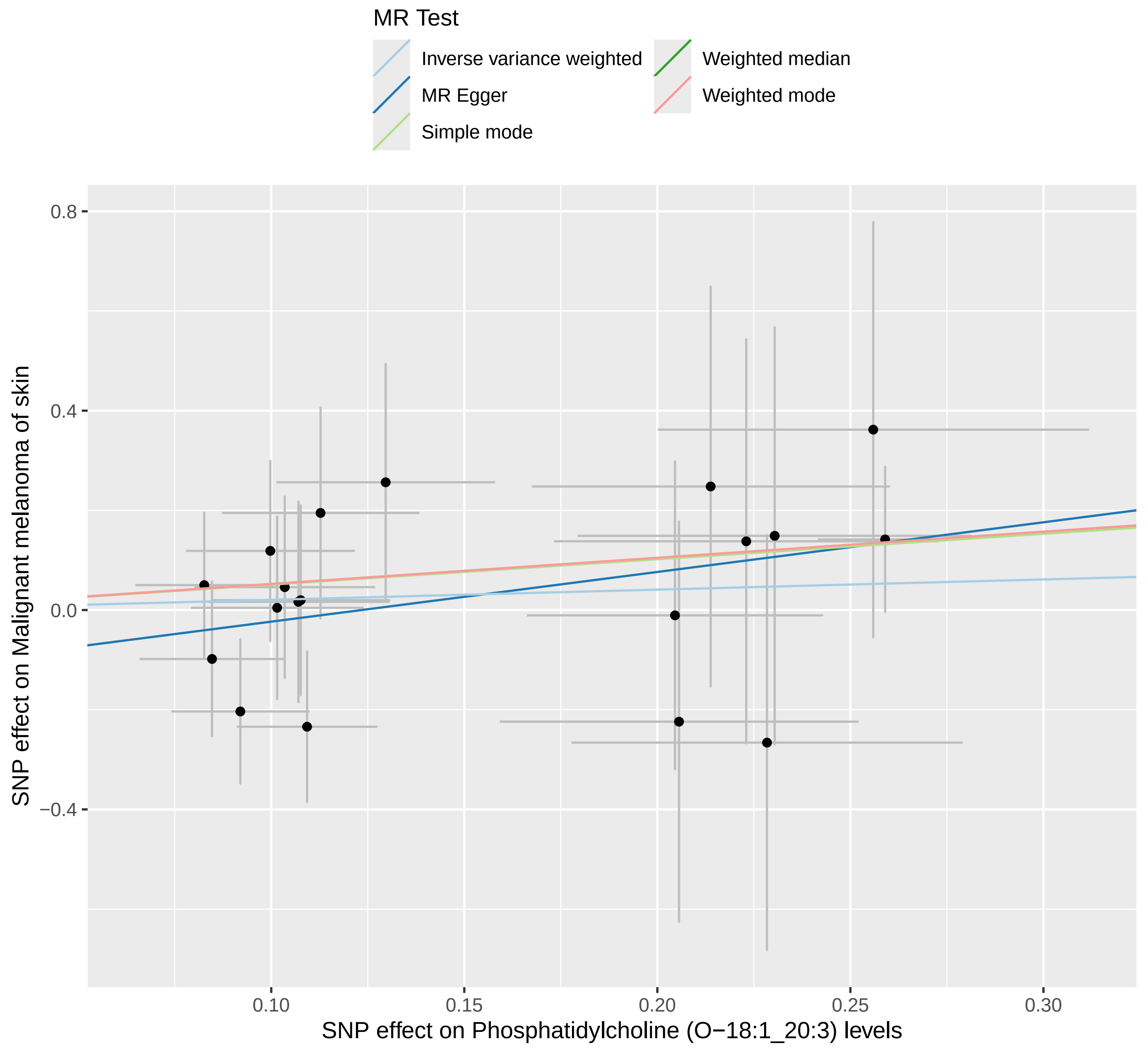

MR Test
Inverse variance weighted
MR Egger
Simple mode
Weighted median
Weighted mode
0.8
0.4
0.0
−0.4
0.10
0.15
0.20
0.25
0.30
SNP effect on Phosphatidylcholine (O−18:1_20:3) levels
SNP effect on Malignant melanoma of skin

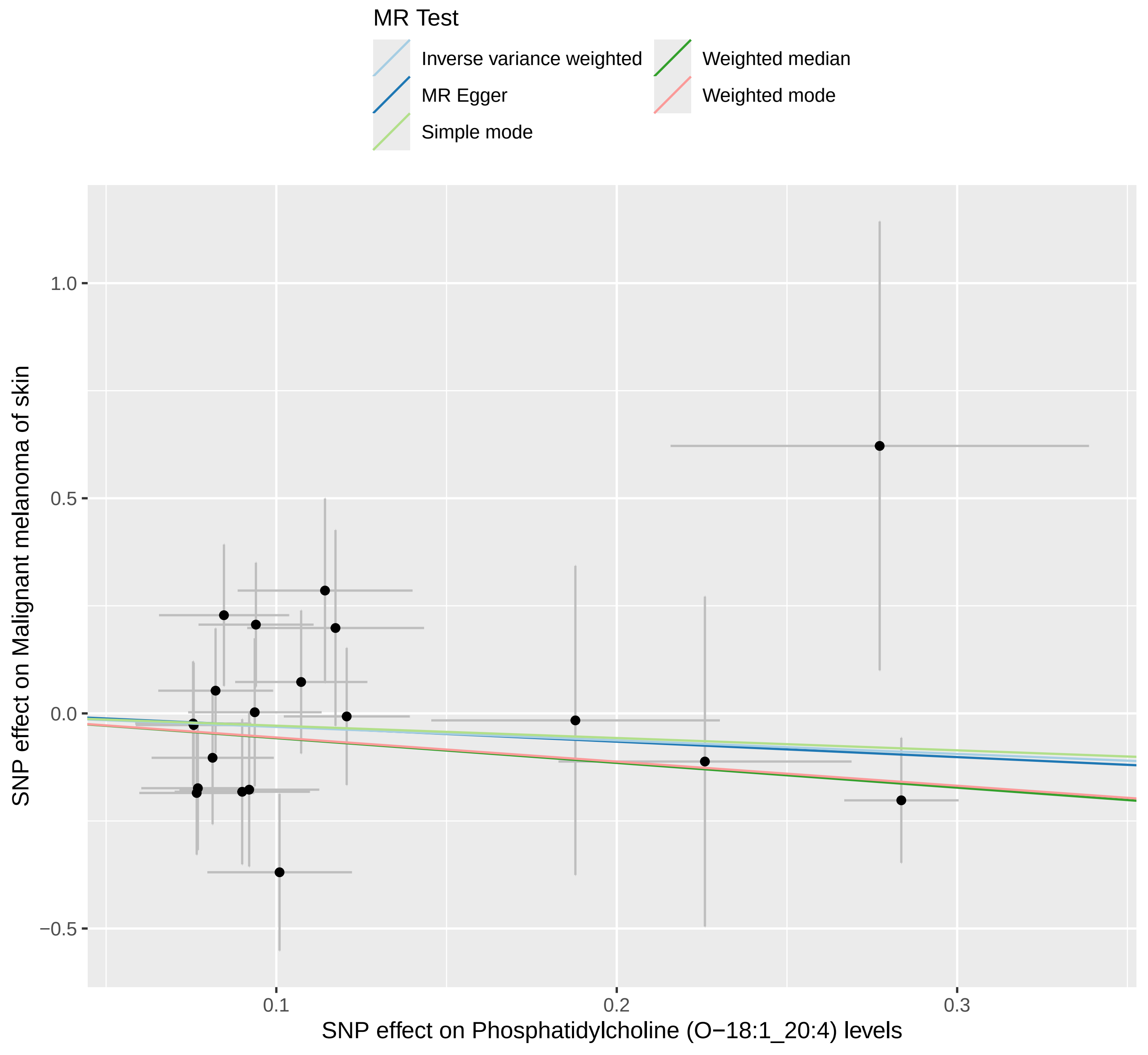
MR Test
Inverse variance weighted
MR Egger
Simple mode
Weighted median
Weighted mode
1.0
0.5
0.0
−0.5
0.1
0.2
0.3
SNP effect on Malignant melanoma of skin
SNP effect on Phosphatidylcholine (O−18:1_20:4) levels

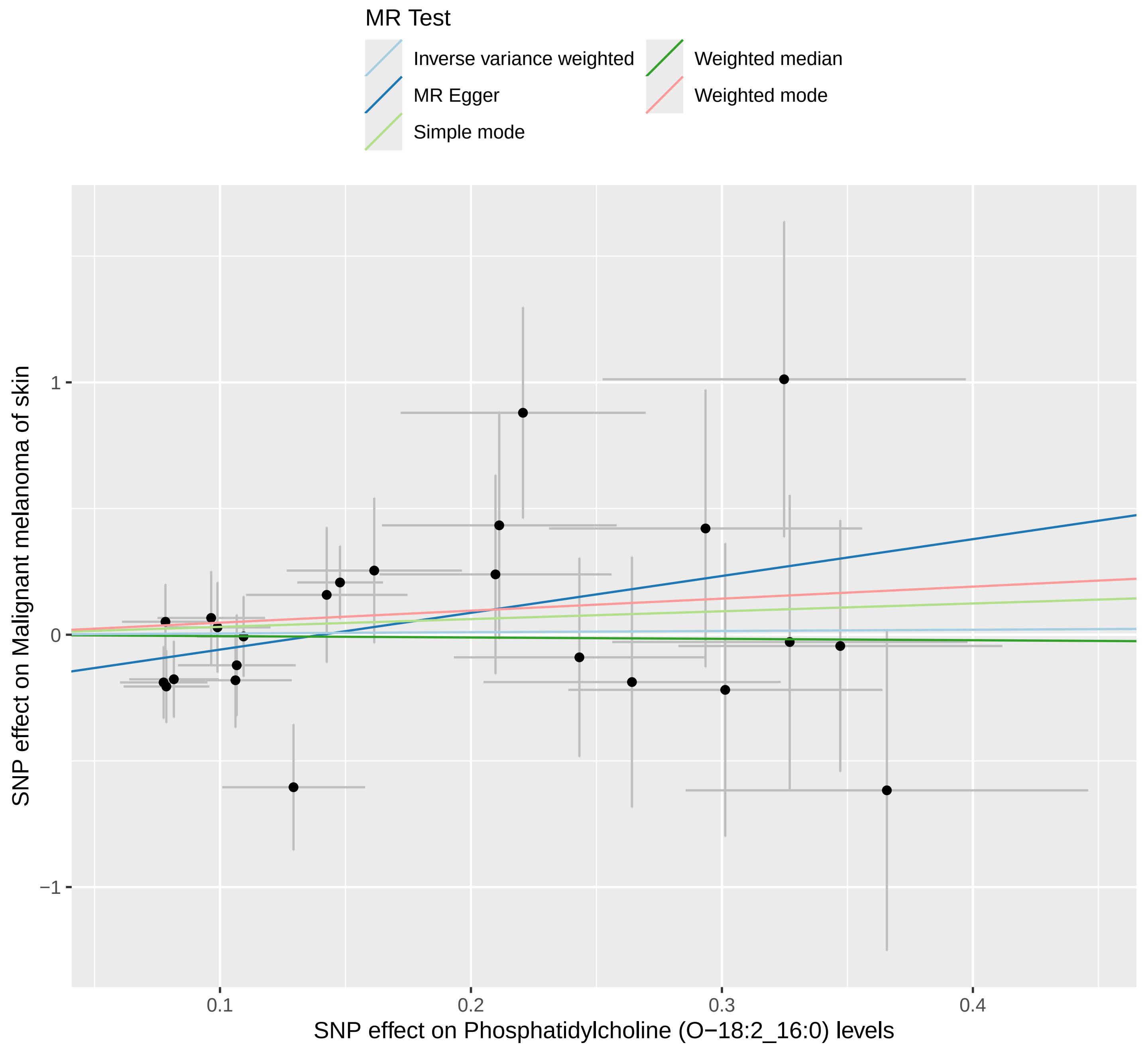
MR Test
Inverse variance weighted
MR Egger
Simple mode
Weighted median
Weighted mode
1
0
−1
0.1
0.2
0.3
0.4
SNP effect on Malignant melanoma of skin
SNP effect on Phosphatidylcholine (O−18:2_16:0) levels

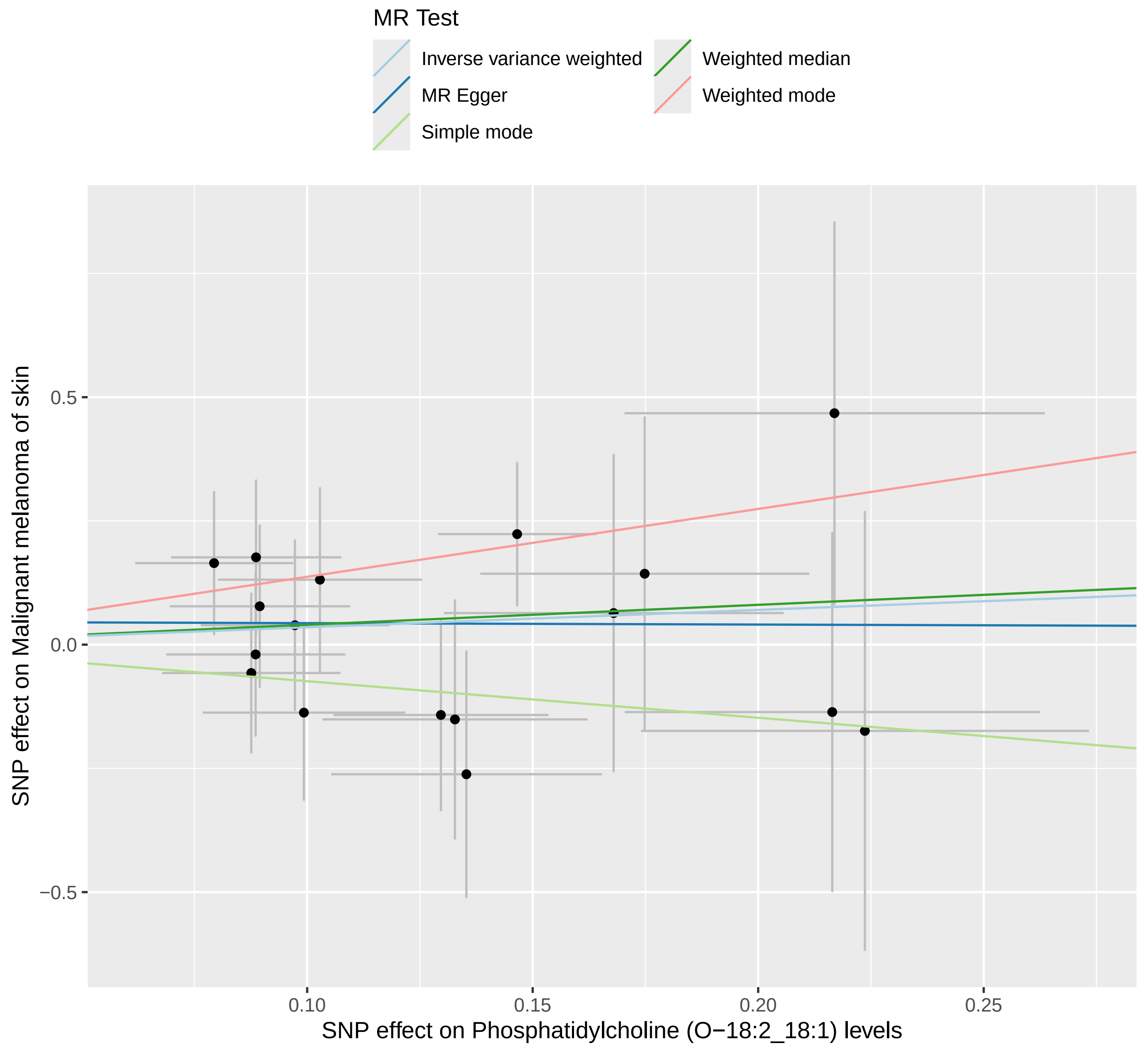
MR Test
Inverse variance weighted
MR Egger
Simple mode
Weighted median
Weighted mode
0.5
0.0
−0.5
0.10
0.15
0.20
0.25
SNP effect on Malignant melanoma of skin
SNP effect on Phosphatidylcholine (O−18:2_18:1) levels

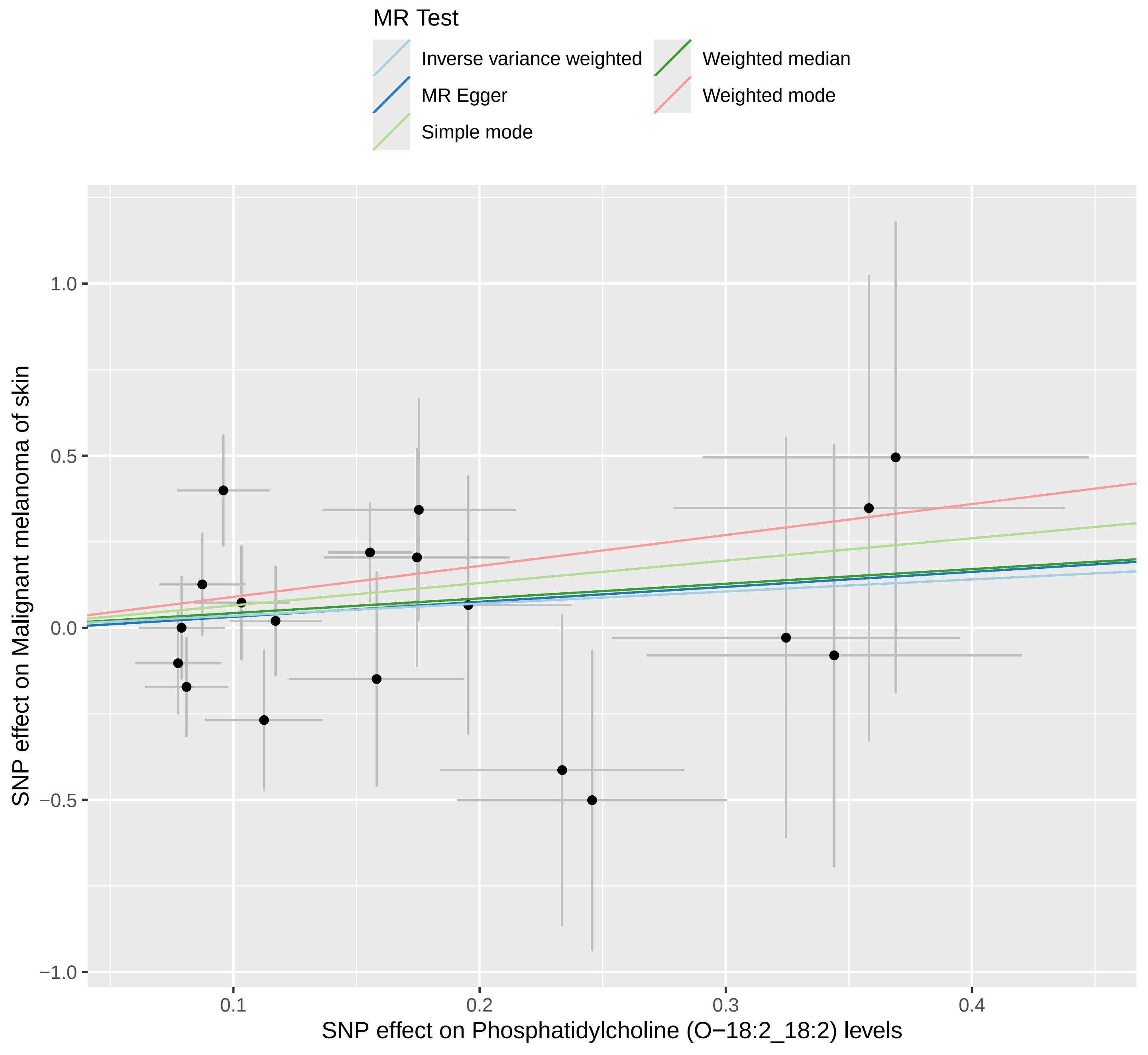

MR Test
Inverse variance weighted
MR Egger
Simple mode
Weighted median
Weighted mode
1.0
0.5
0.0
−0.5
−1.0
0.1
0.2
0.3
0.4
SNP effect on Malignant melanoma of skin
SNP effect on Phosphatidylcholine (O−18:2_18:2) levels

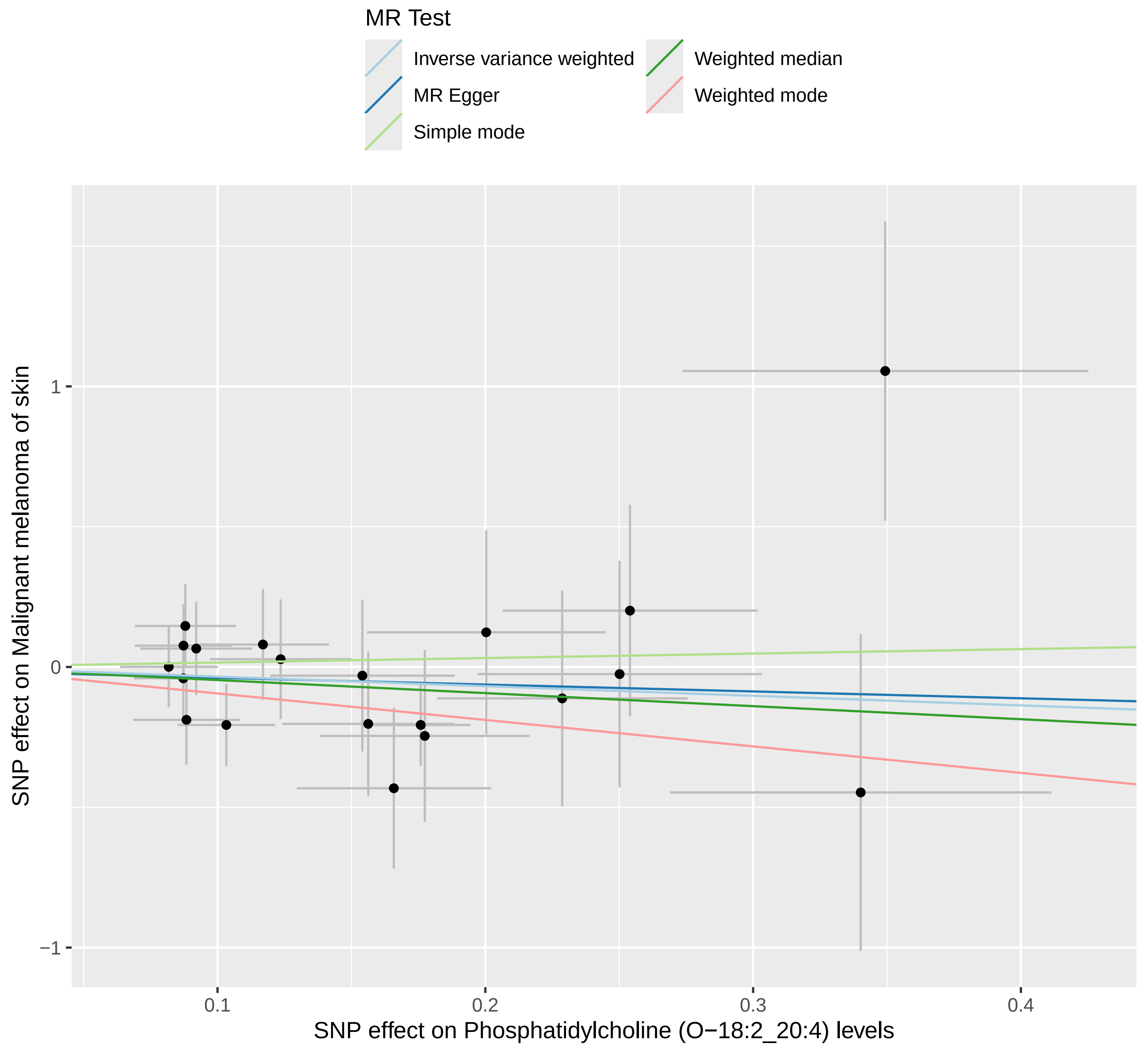
MR Test
Inverse variance weighted
MR Egger
Simple mode
Weighted median
Weighted mode
1
0
−1
0.1
0.2
0.3
0.4
SNP effect on Malignant melanoma of skin
SNP effect on Phosphatidylcholine (O−18:2_20:4) levels

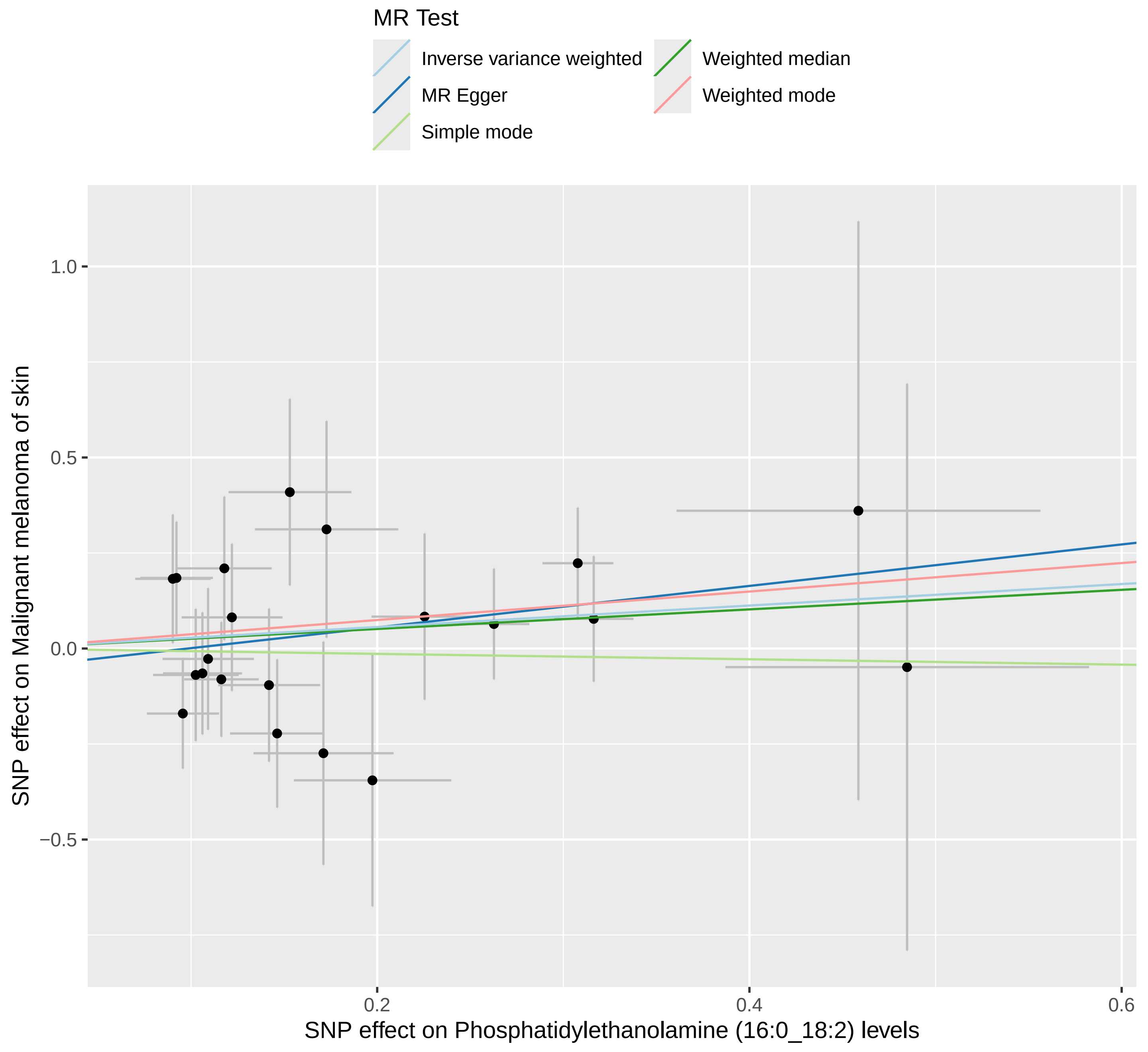
MR Test
Inverse variance weighted
MR Egger
Simple mode
Weighted median
Weighted mode
1.0
0.5
0.0
−0.5
0.2
0.4
0.6
SNP effect on Malignant melanoma of skin
SNP effect on Phosphatidylethanolamine (16:0_18:2) levels

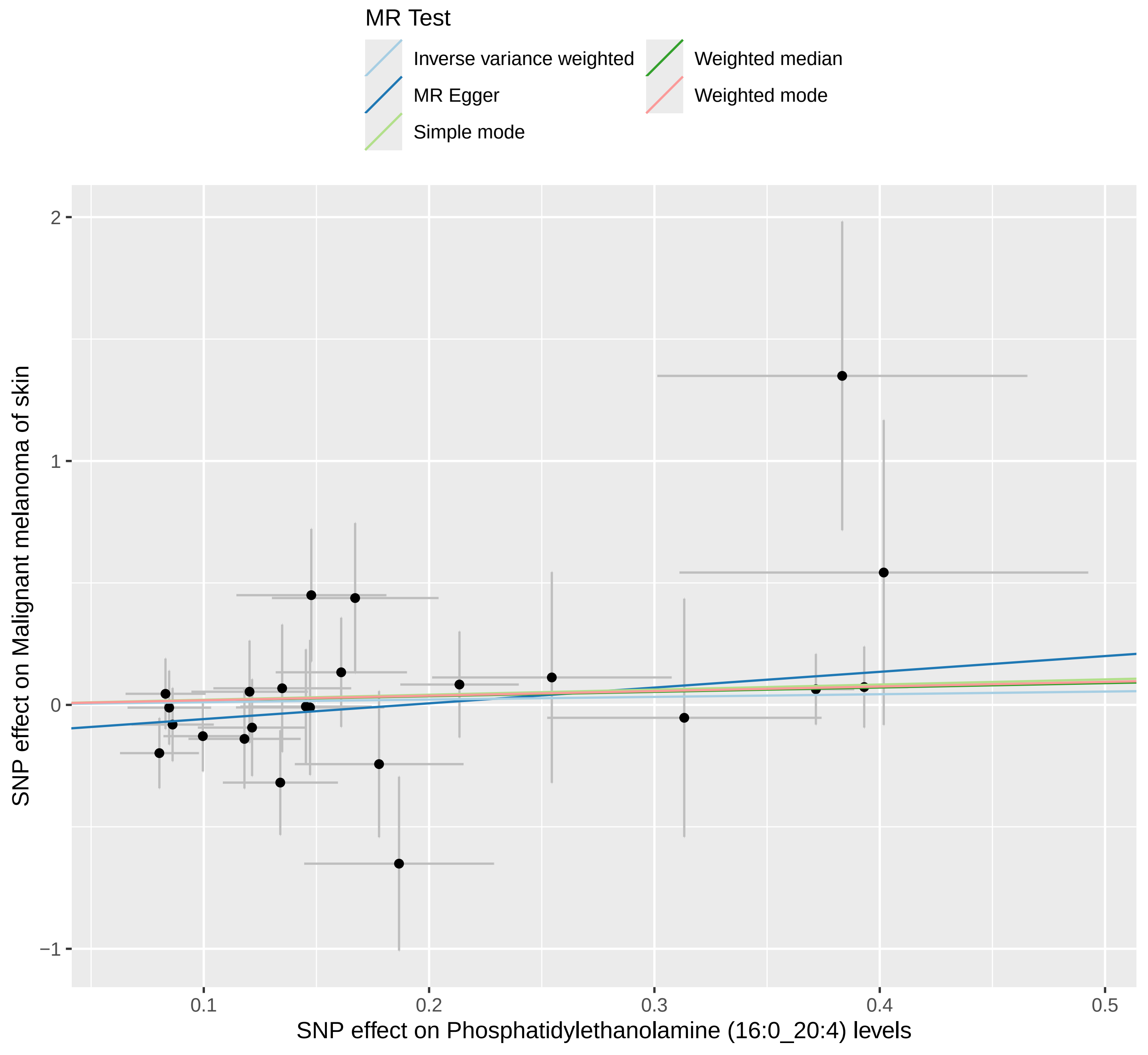

MR Test
Inverse variance weighted
MR Egger
Simple mode
Weighted median
Weighted mode
SNP effect on Malignant melanoma of skin
SNP effect on Phosphatidylethanolamine (16:0_20:4) levels
2
1
0
−1
0.1
0.2
0.3
0.4
0.5

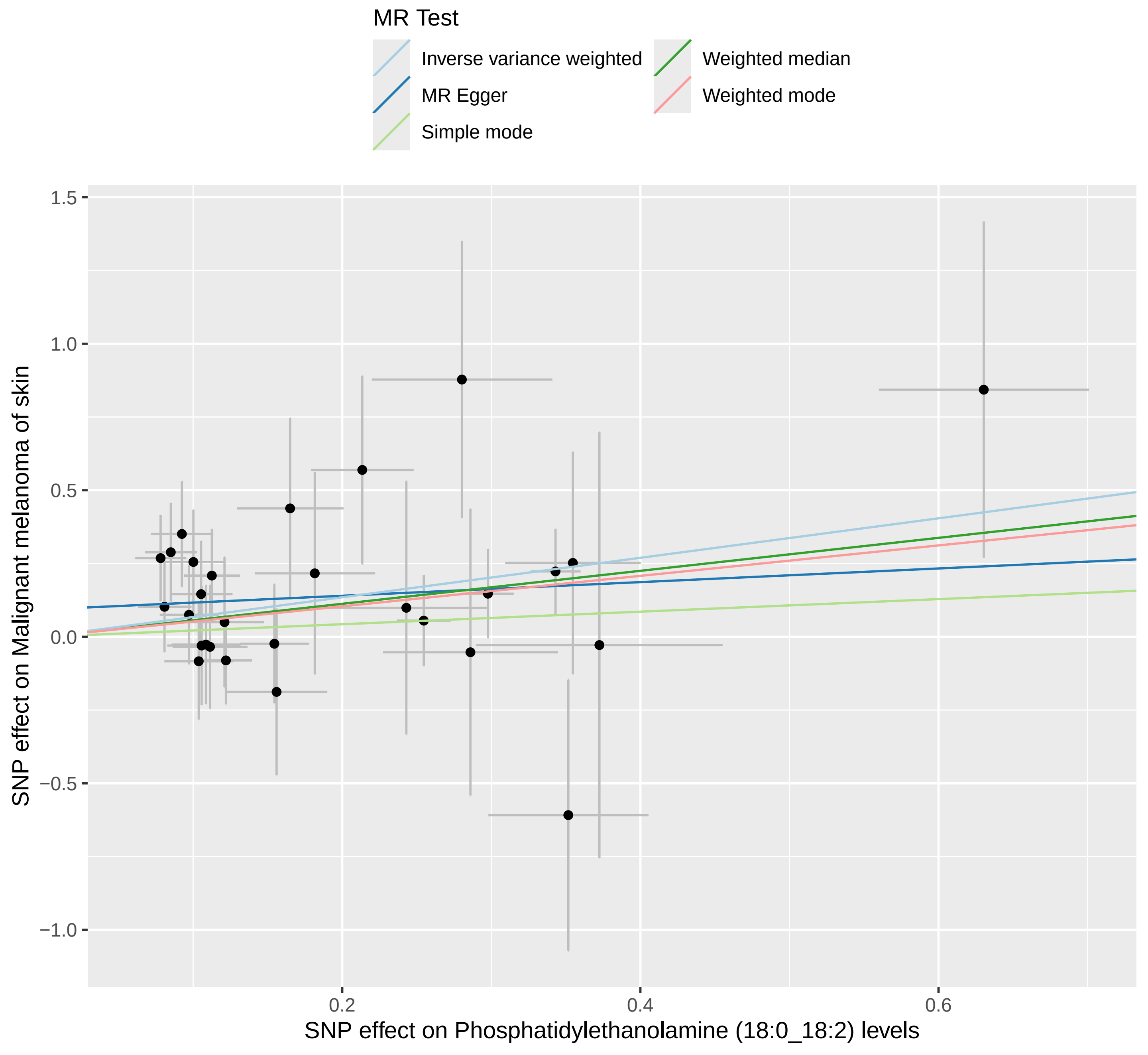

MR Test
Inverse variance weighted
MR Egger
Simple mode
Weighted median
Weighted mode
1.5
1.0
0.5
0.0
−0.5
−1.0
0.2
0.4
0.6
SNP effect on Malignant melanoma of skin
SNP effect on Phosphatidylethanolamine (18:0_18:2) levels

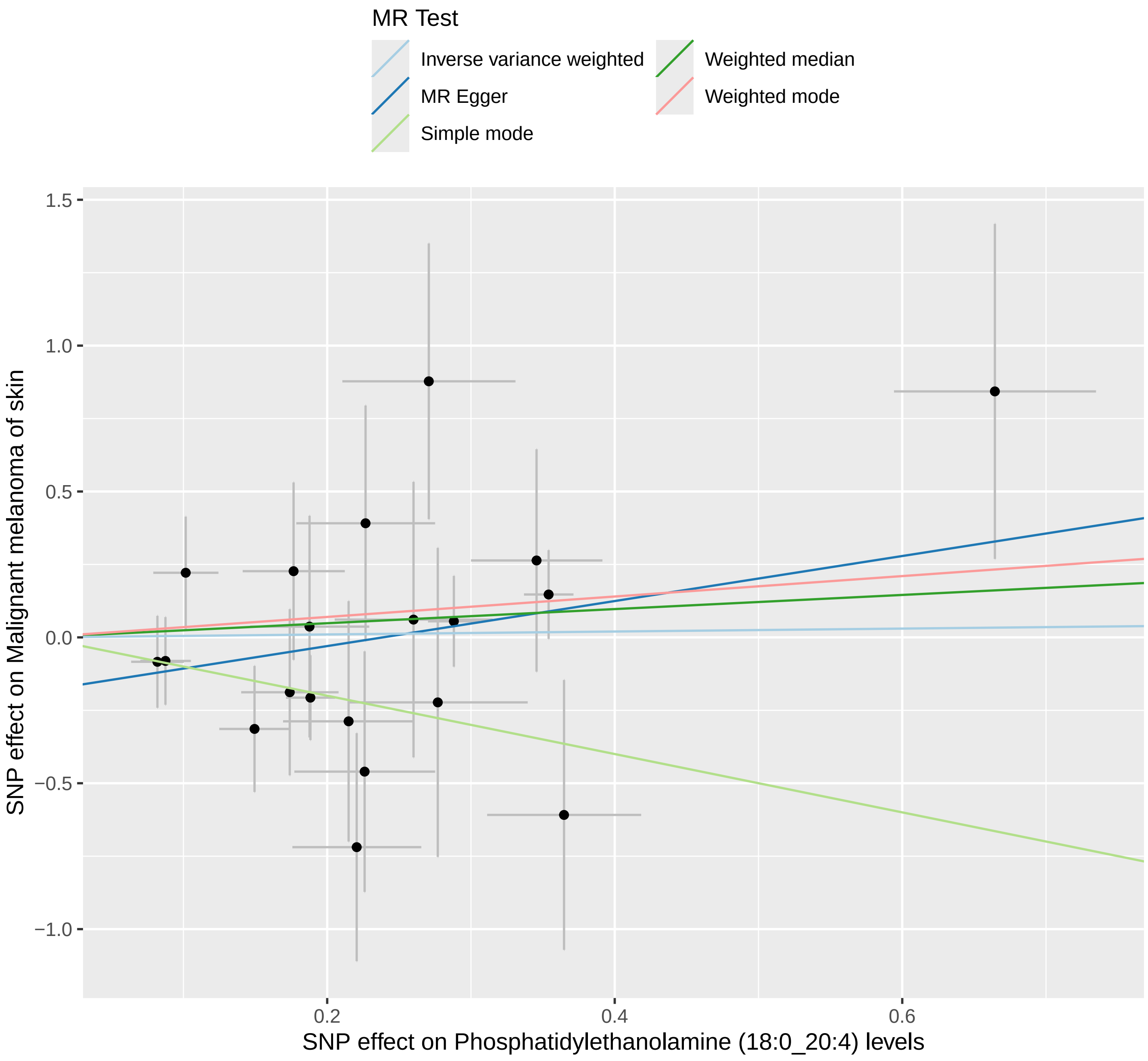
MR Test
Inverse variance weighted
MR Egger
Simple mode
Weighted median
Weighted mode
1.5
1.0
0.5
0.0
−0.5
−1.0
SNP effect on Malignant melanoma of skin
0.2
0.4
0.6
SNP effect on Phosphatidylethanolamine (18:0_20:4) levels

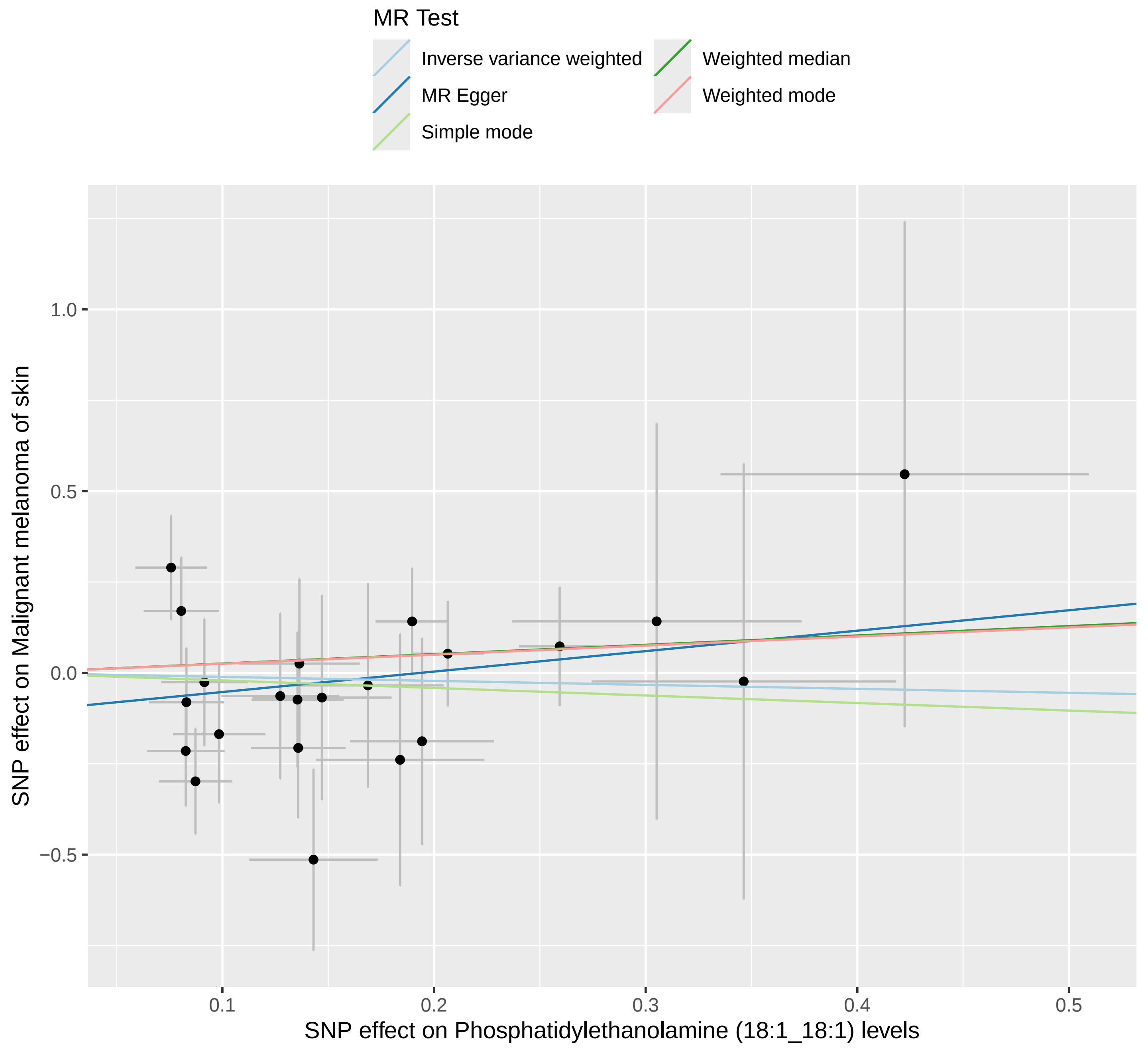

MR Test
Inverse variance weighted
MR Egger
Simple mode
Weighted median
Weighted mode
1.0
0.5
0.0
−0.5
0.1
0.2
0.3
0.4
0.5
SNP effect on Malignant melanoma of skin
SNP effect on Phosphatidylethanolamine (18:1_18:1) levels

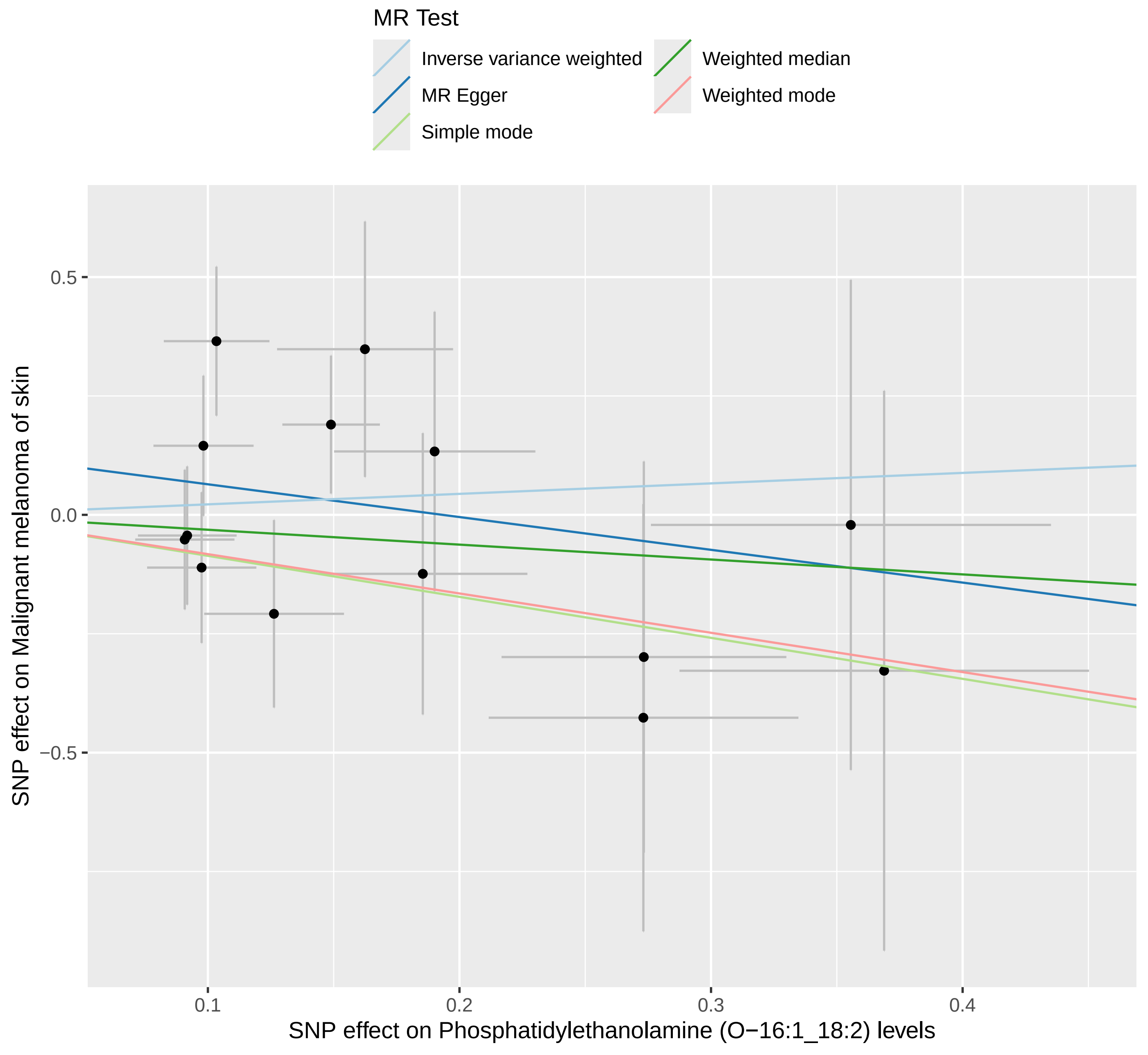

MR Test
Inverse variance weighted
MR Egger
Simple mode
Weighted median
Weighted mode
0.5
0.0
−0.5
0.1
0.2
0.3
0.4
SNP effect on Malignant melanoma of skin
SNP effect on Phosphatidylethanolamine (O−16:1_18:2) levels

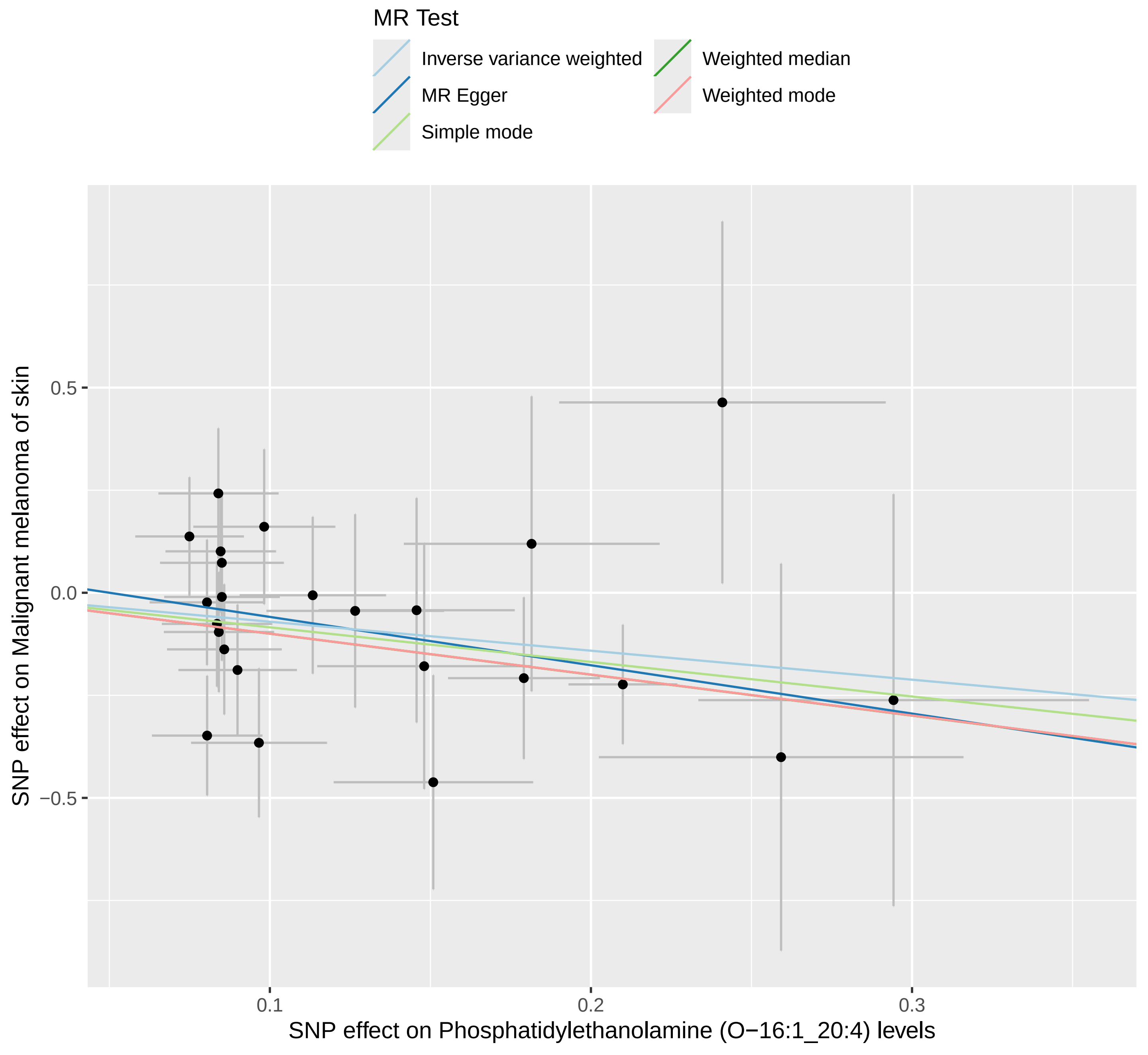

MR Test
Inverse variance weighted
MR Egger
Simple mode
Weighted median
Weighted mode
0.5
0.0
−0.5
0.1
0.2
0.3
SNP effect on Malignant melanoma of skin
SNP effect on Phosphatidylethanolamine (O−16:1_20:4) levels

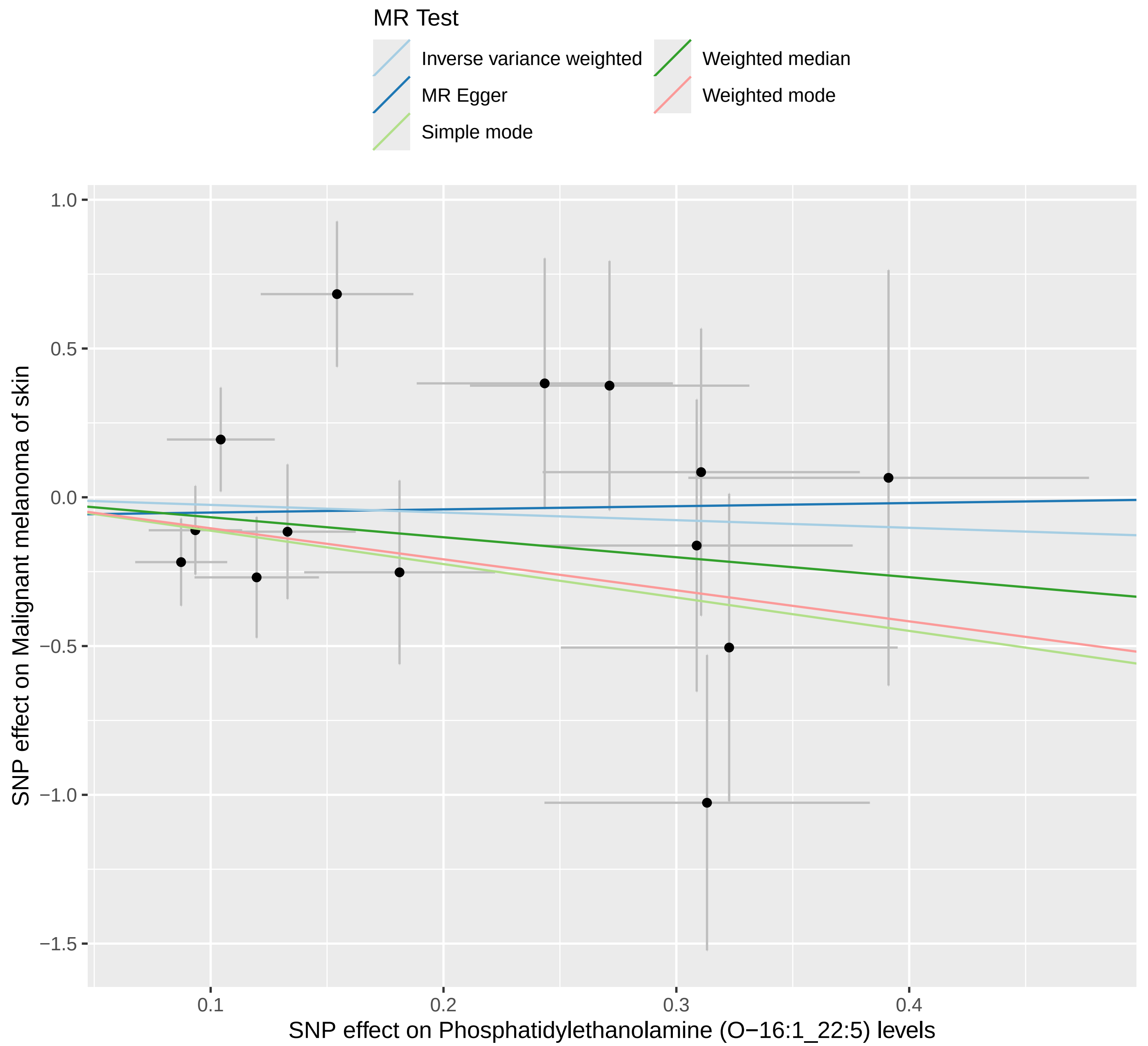
MR Test
Inverse variance weighted
MR Egger
Simple mode
Weighted median
Weighted mode
1.0
0.5
0.0
−0.5
−1.0
−1.5
0.1
0.2
0.3
0.4
SNP effect on Malignant melanoma of skin
SNP effect on Phosphatidylethanolamine (O−16:1_22:5) levels

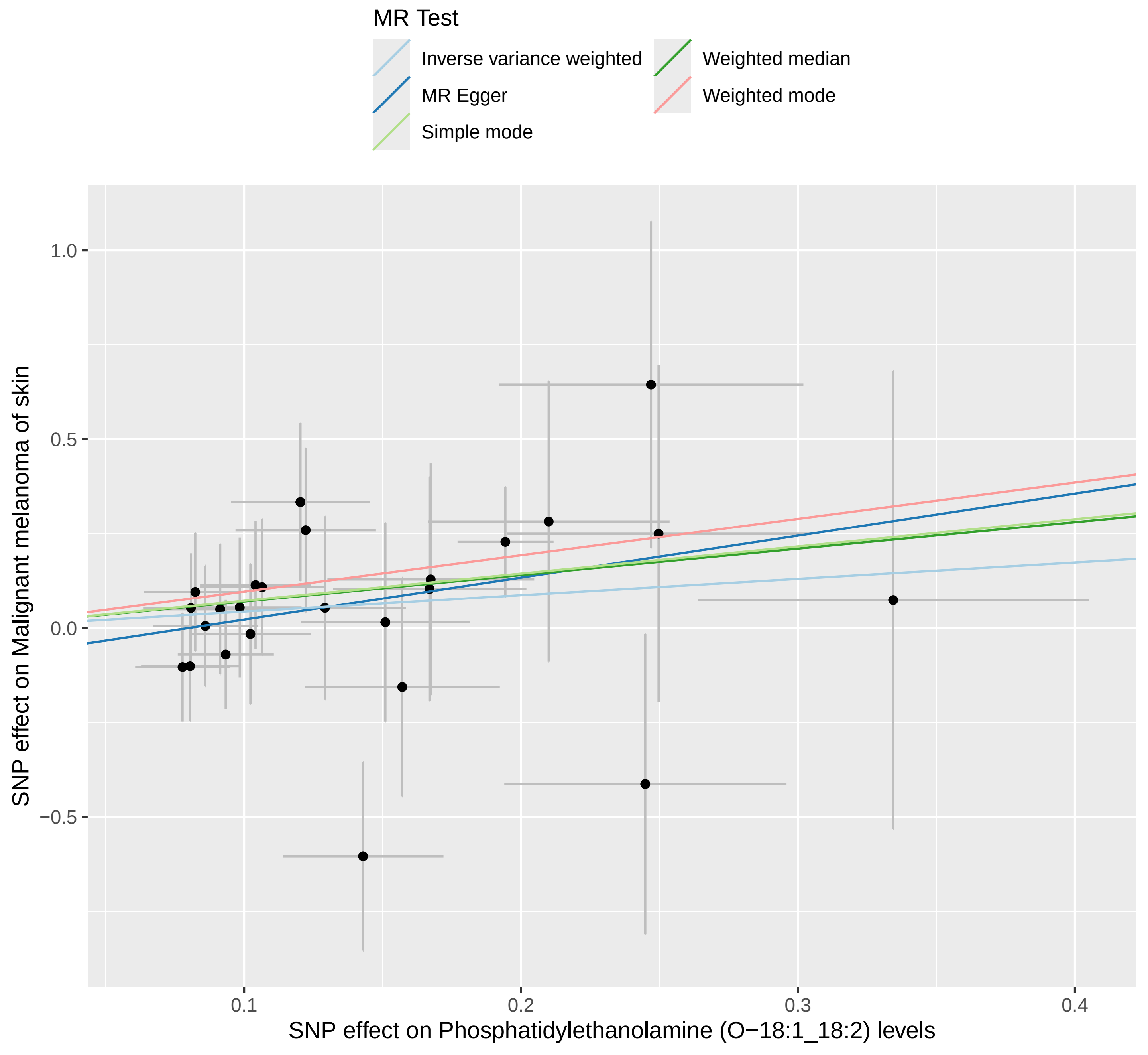

MR Test
Inverse variance weighted
MR Egger
Simple mode
Weighted median
Weighted mode
1.0
0.5
0.0
−0.5
0.1
0.2
0.3
0.4
SNP effect on Malignant melanoma of skin
SNP effect on Phosphatidylethanolamine (O−18:1_18:2) levels

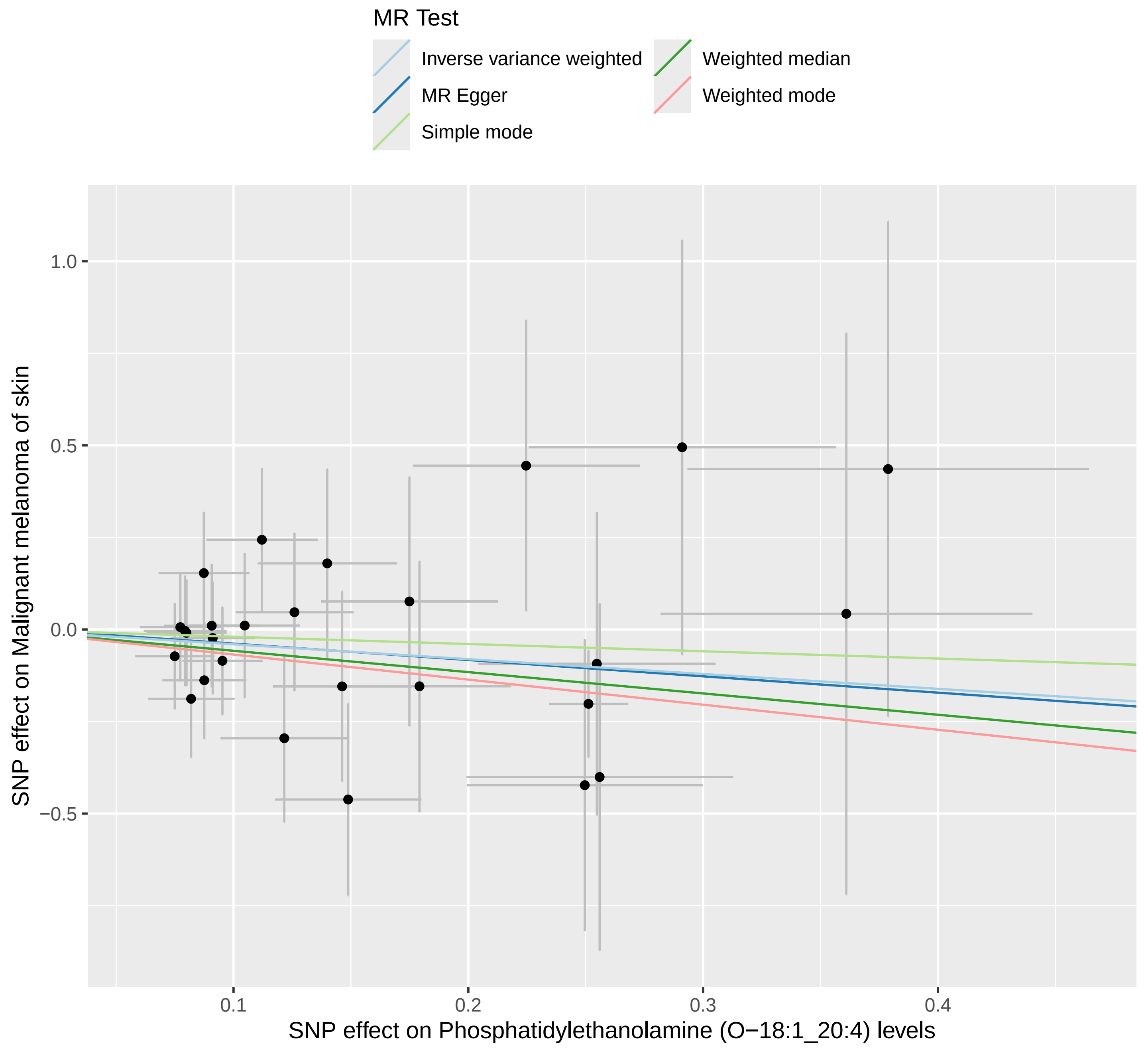
MR Test
Inverse variance weighted
MR Egger
Simple mode
Weighted median
Weighted mode
1.0
0.5
0.0
−0.5
0.1
0.2
0.3
0.4
SNP effect on Malignant melanoma of skin
SNP effect on Phosphatidylethanolamine (O−18:1_20:4) levels

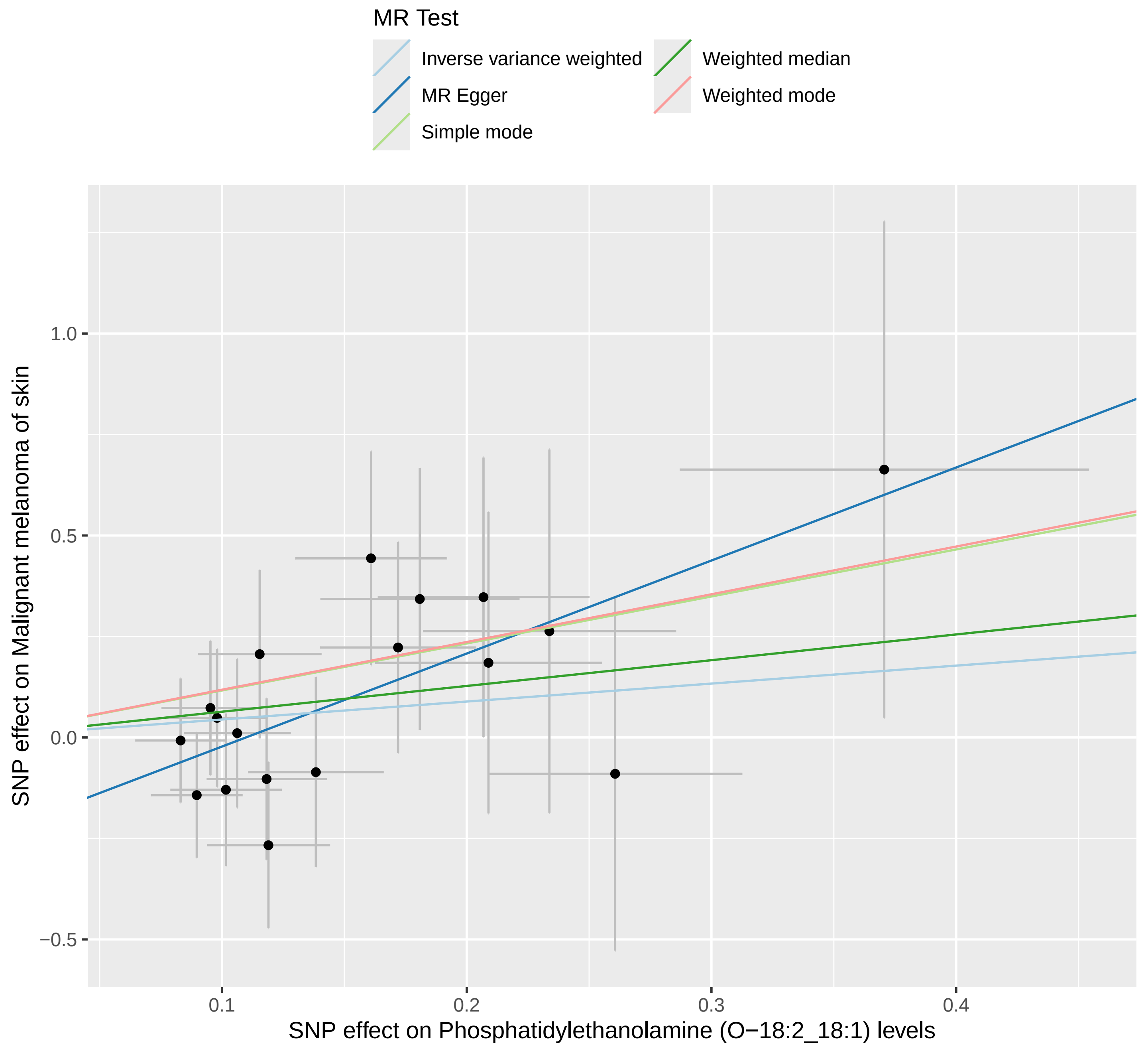

MR Test
Inverse variance weighted
MR Egger
Simple mode
Weighted median
Weighted mode
1.0
0.5
0.0
−0.5
0.1
0.2
0.3
0.4
SNP effect on Malignant melanoma of skin
SNP effect on Phosphatidylethanolamine (O−18:2_18:1) levels

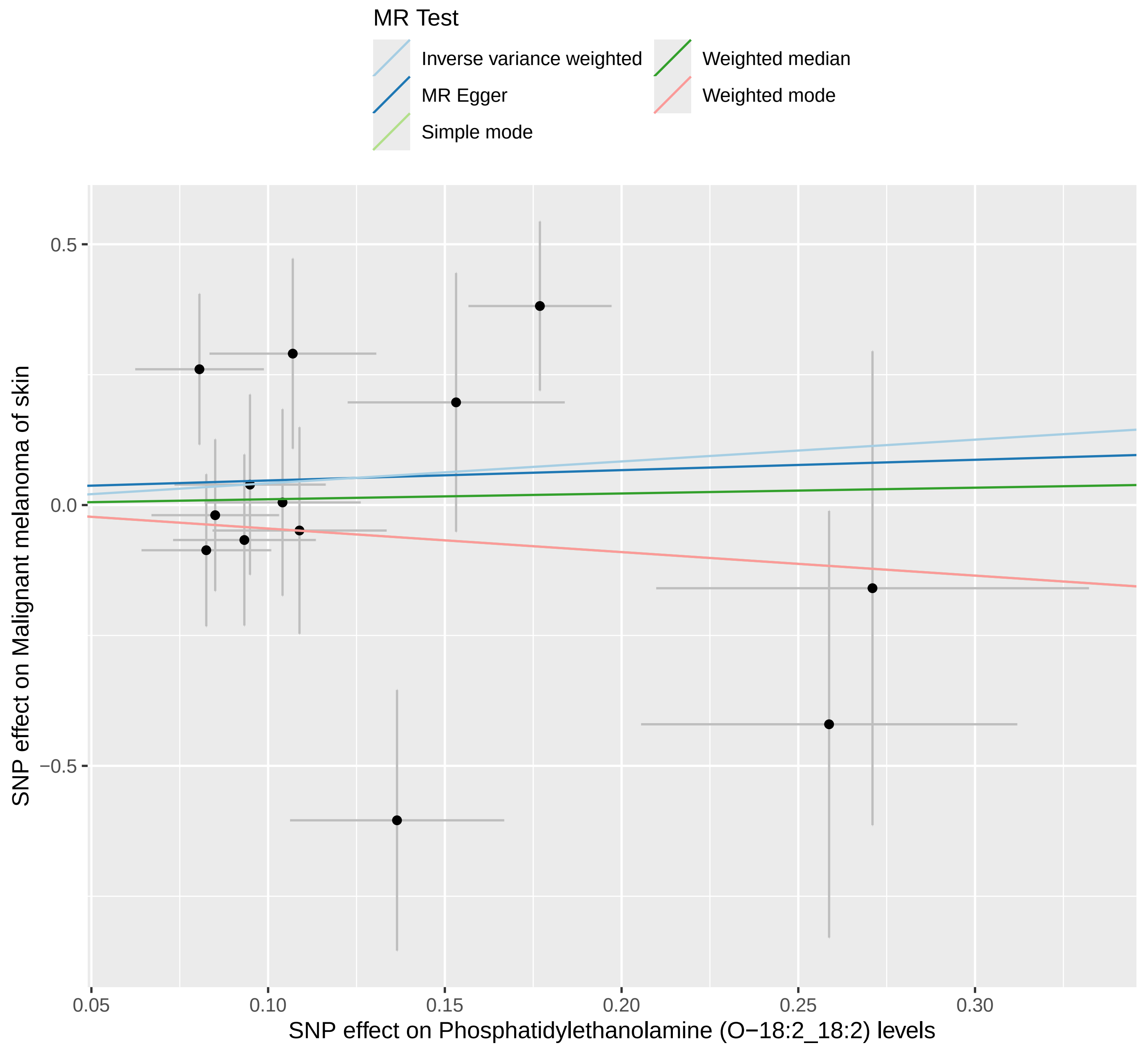

MR Test
Inverse variance weighted
MR Egger
Simple mode
Weighted median
Weighted mode
0.5
0.0
−0.5
0.05
0.10
0.15
0.20
0.25
0.30
SNP effect on Malignant melanoma of skin
SNP effect on Phosphatidylethanolamine (O−18:2_18:2) levels

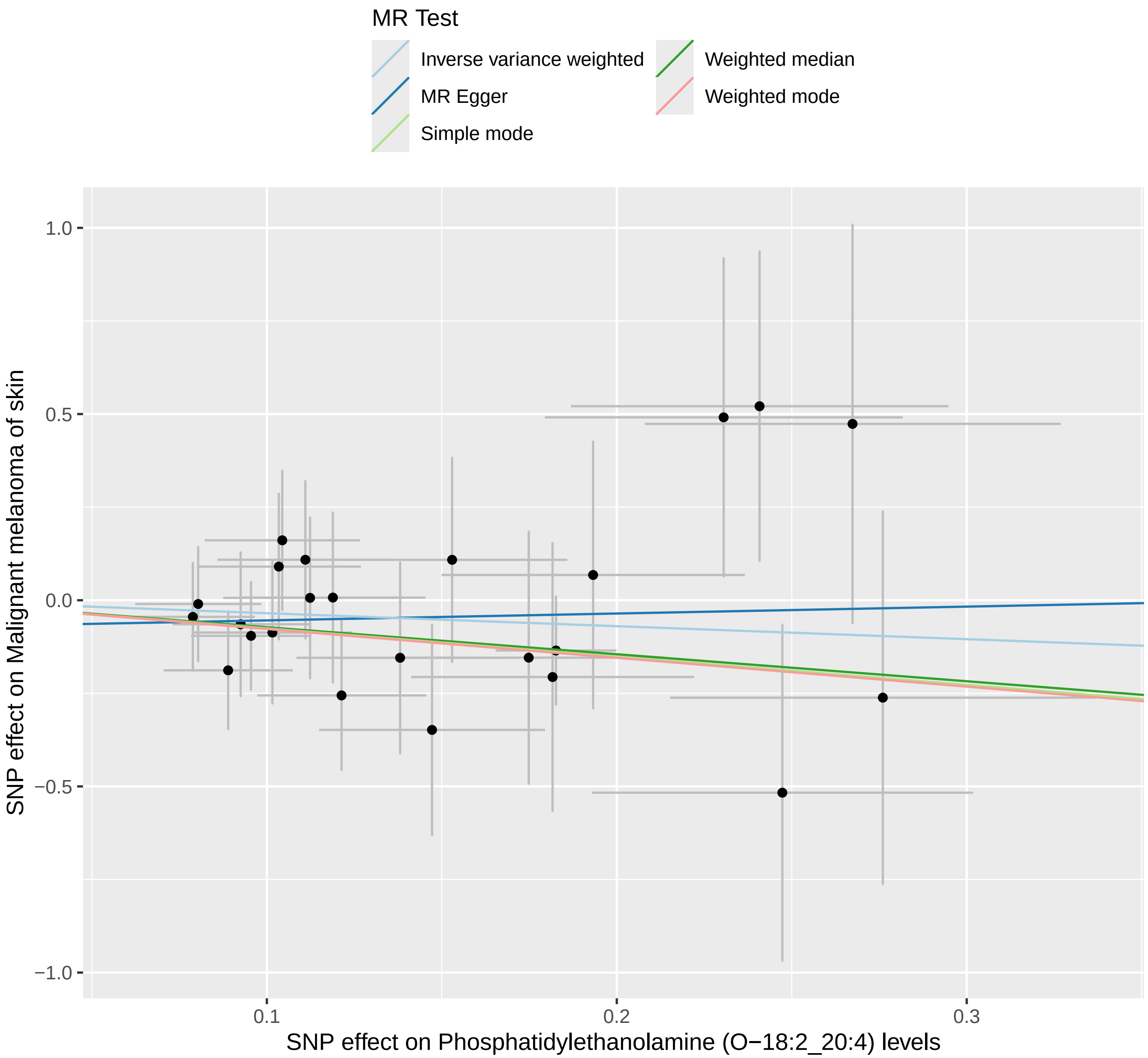

MR Test
Inverse variance weighted
MR Egger
Simple mode
Weighted median
Weighted mode
1.0
0.5
0.0
−0.5
−1.0
0.1
0.2
0.3
SNP effect on Malignant melanoma of skin
SNP effect on Phosphatidylethanolamine (O−18:2_20:4) levels

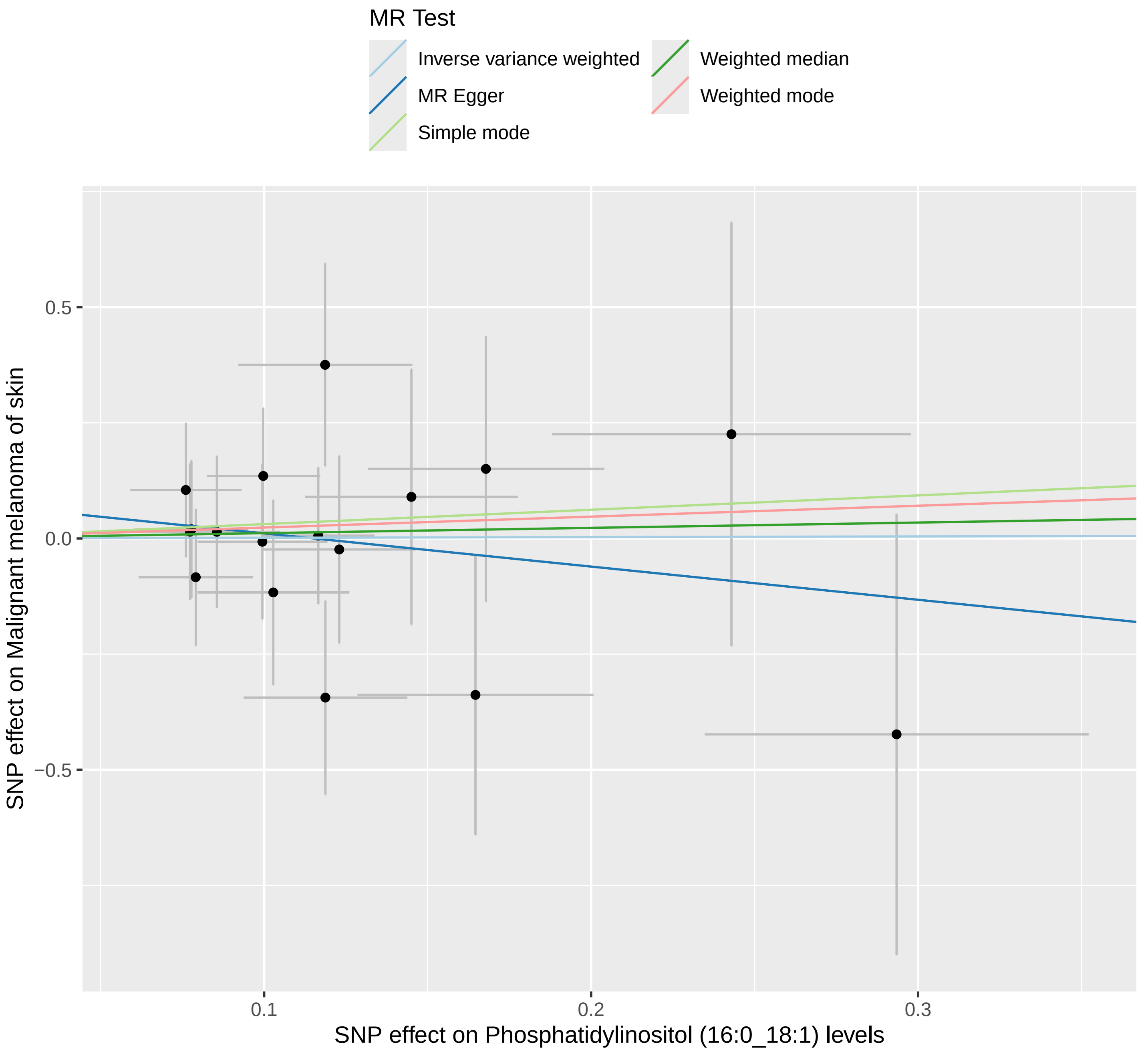
MR Test
Inverse variance weighted
MR Egger
Simple mode
Weighted median
Weighted mode
0.5
0.0
−0.5
0.1
0.2
0.3
SNP effect on Malignant melanoma of skin
SNP effect on Phosphatidylinositol (16:0_18:1) levels

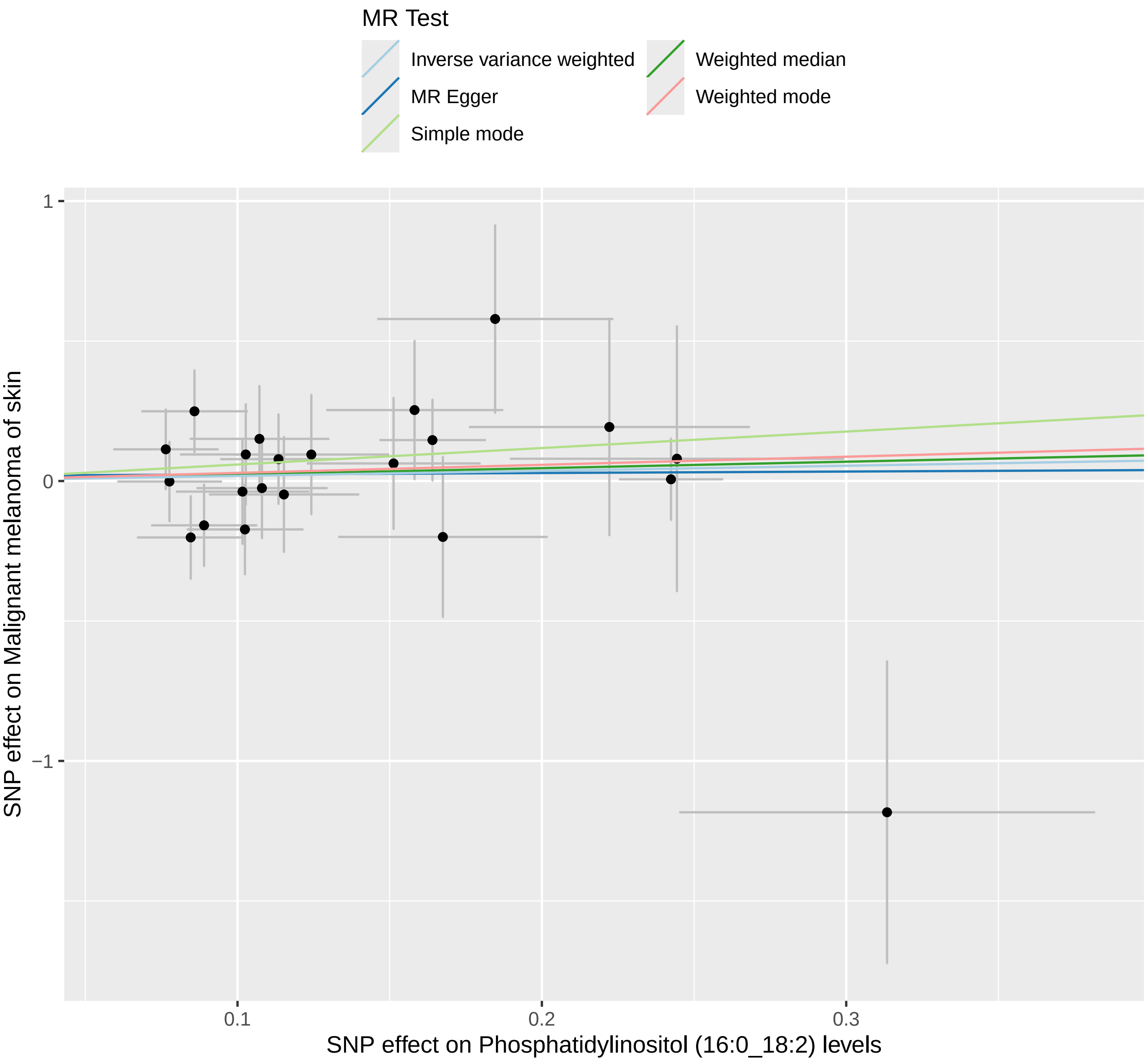

MR Test
Inverse variance weighted
MR Egger
Simple mode
Weighted median
Weighted mode
1
0
−1
0.1
0.2
0.3
SNP effect on Malignant melanoma of skin
SNP effect on Phosphatidylinositol (16:0_18:2) levels

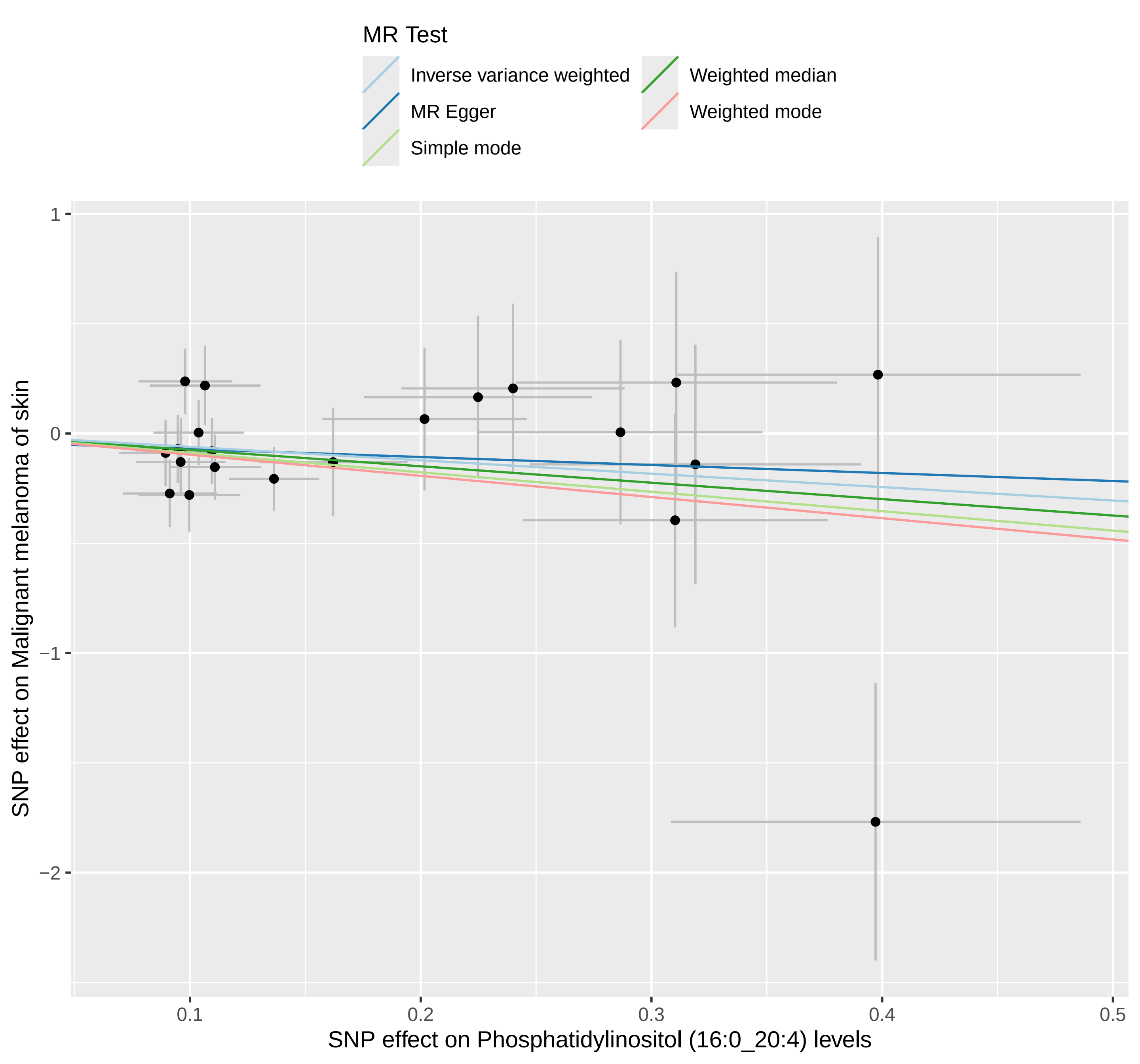

MR Test
Inverse variance weighted
MR Egger
Simple mode
Weighted median
Weighted mode
SNP effect on Malignant melanoma of skin
1
0
−1
−2
0.1
0.2
0.3
0.4
0.5
SNP effect on Phosphatidylinositol (16:0_20:4) levels

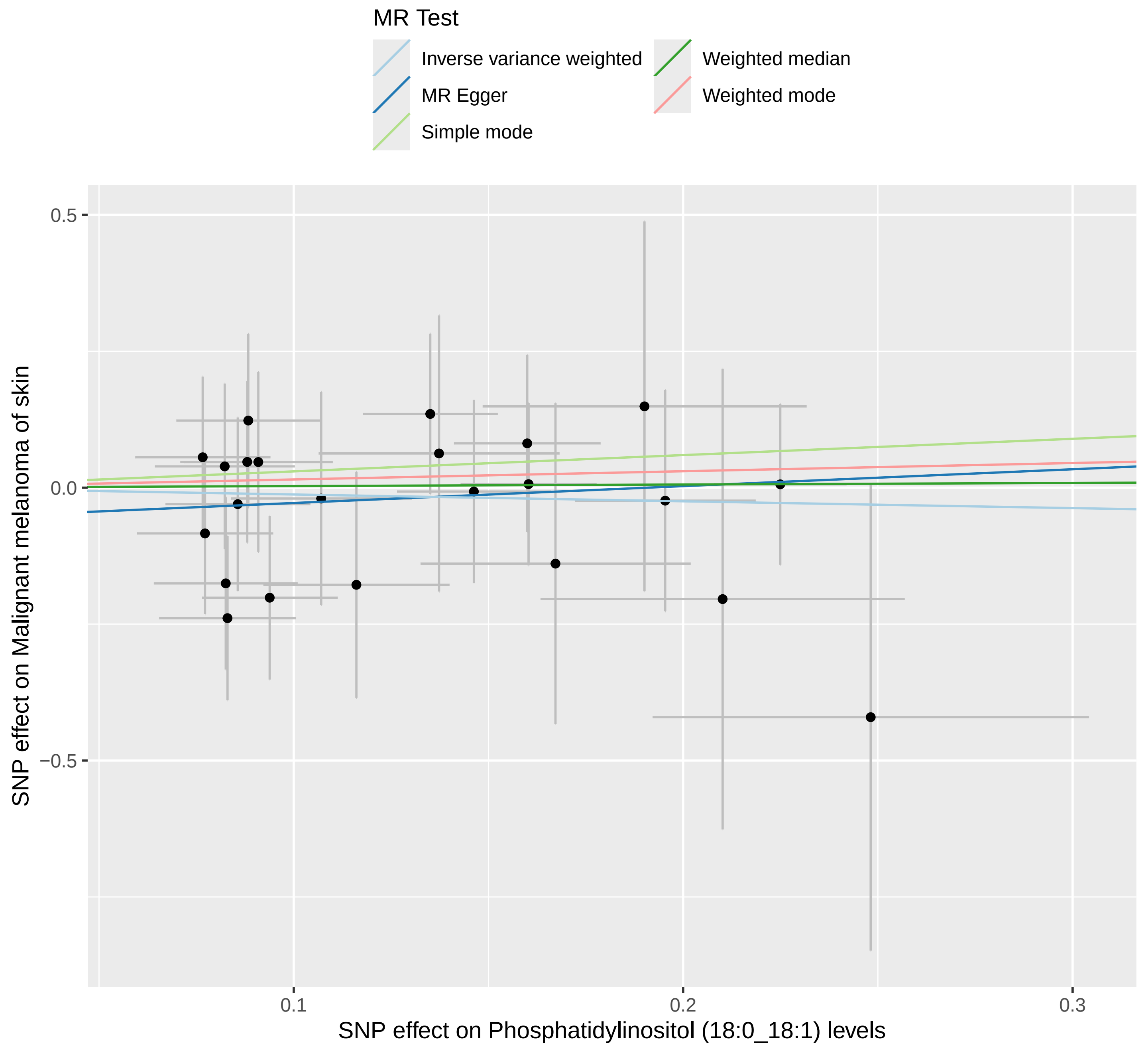
MR Test
Inverse variance weighted
MR Egger
Simple mode
Weighted median
Weighted mode
0.5
0.0
−0.5
0.1
0.2
0.3
SNP effect on Malignant melanoma of skin
SNP effect on Phosphatidylinositol (18:0_18:1) levels

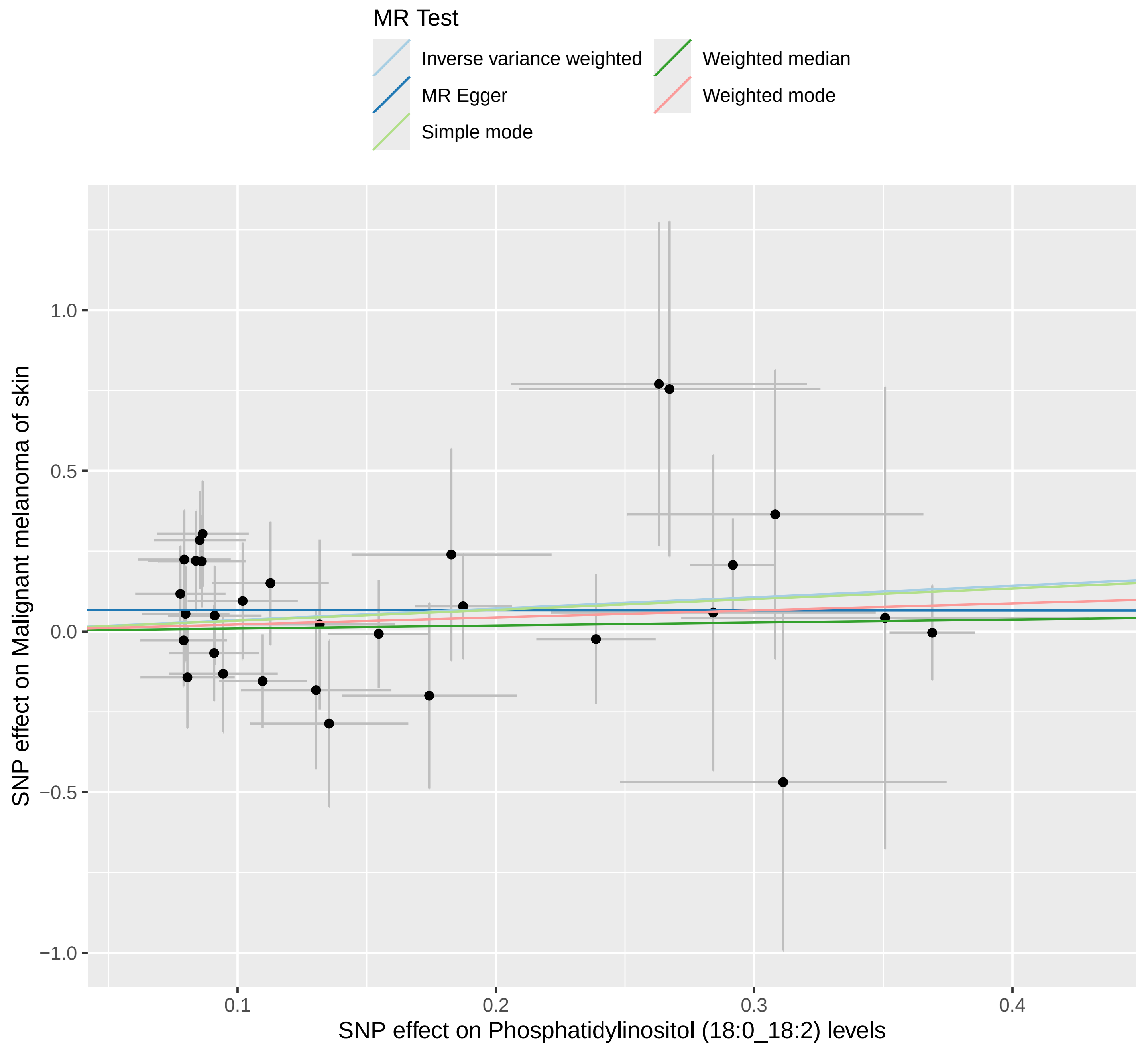

MR Test
Inverse variance weighted
MR Egger
Simple mode
Weighted median
Weighted mode
1.0
0.5
0.0
−0.5
−1.0
0.1
0.2
0.3
0.4
SNP effect on Malignant melanoma of skin
SNP effect on Phosphatidylinositol (18:0_18:2) levels

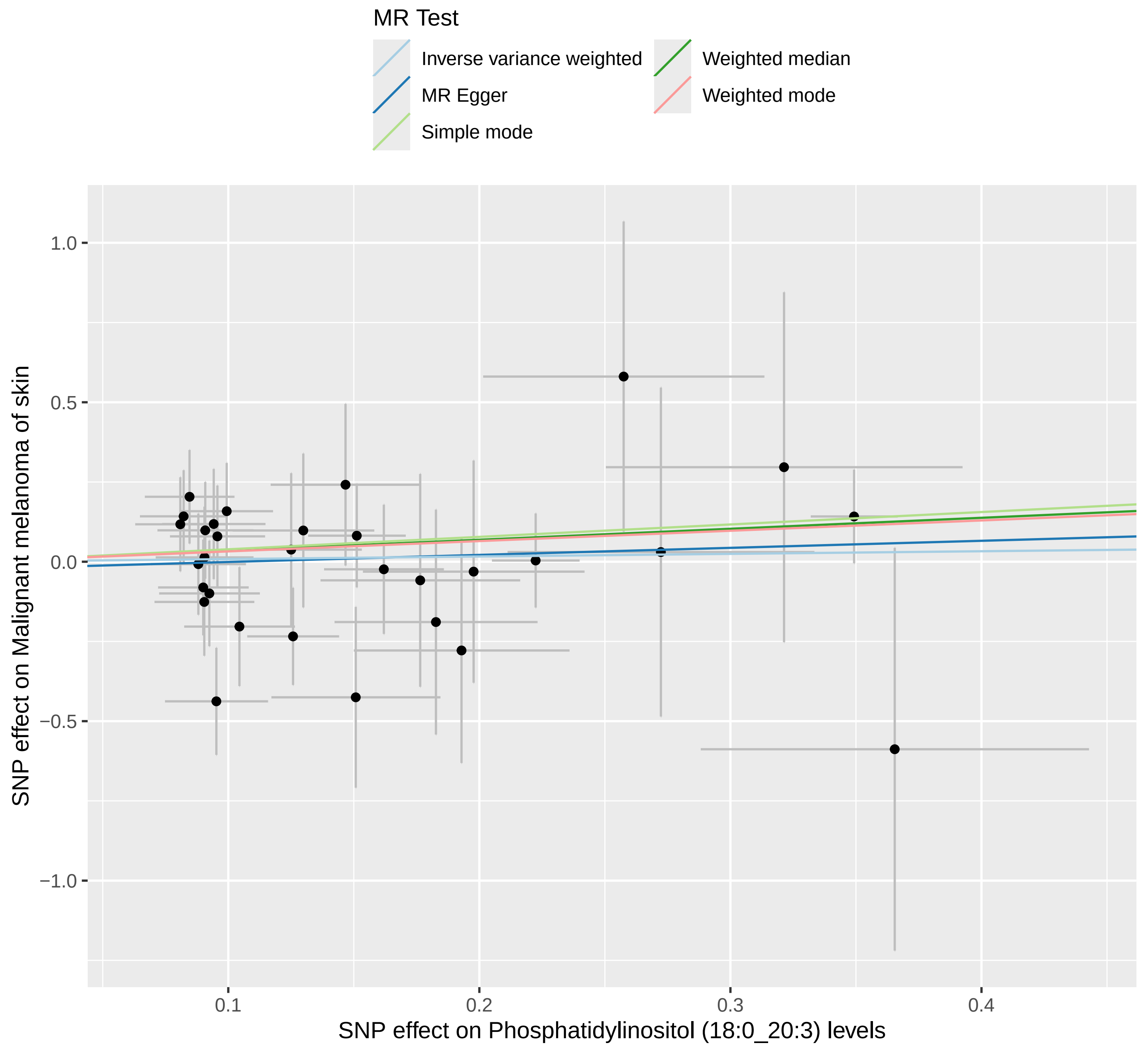

MR Test
Inverse variance weighted
MR Egger
Simple mode
Weighted median
Weighted mode
1.0
0.5
0.0
−0.5
−1.0
SNP effect on Malignant melanoma of skin
0.1
0.2
0.3
0.4
SNP effect on Phosphatidylinositol (18:0_20:3) levels

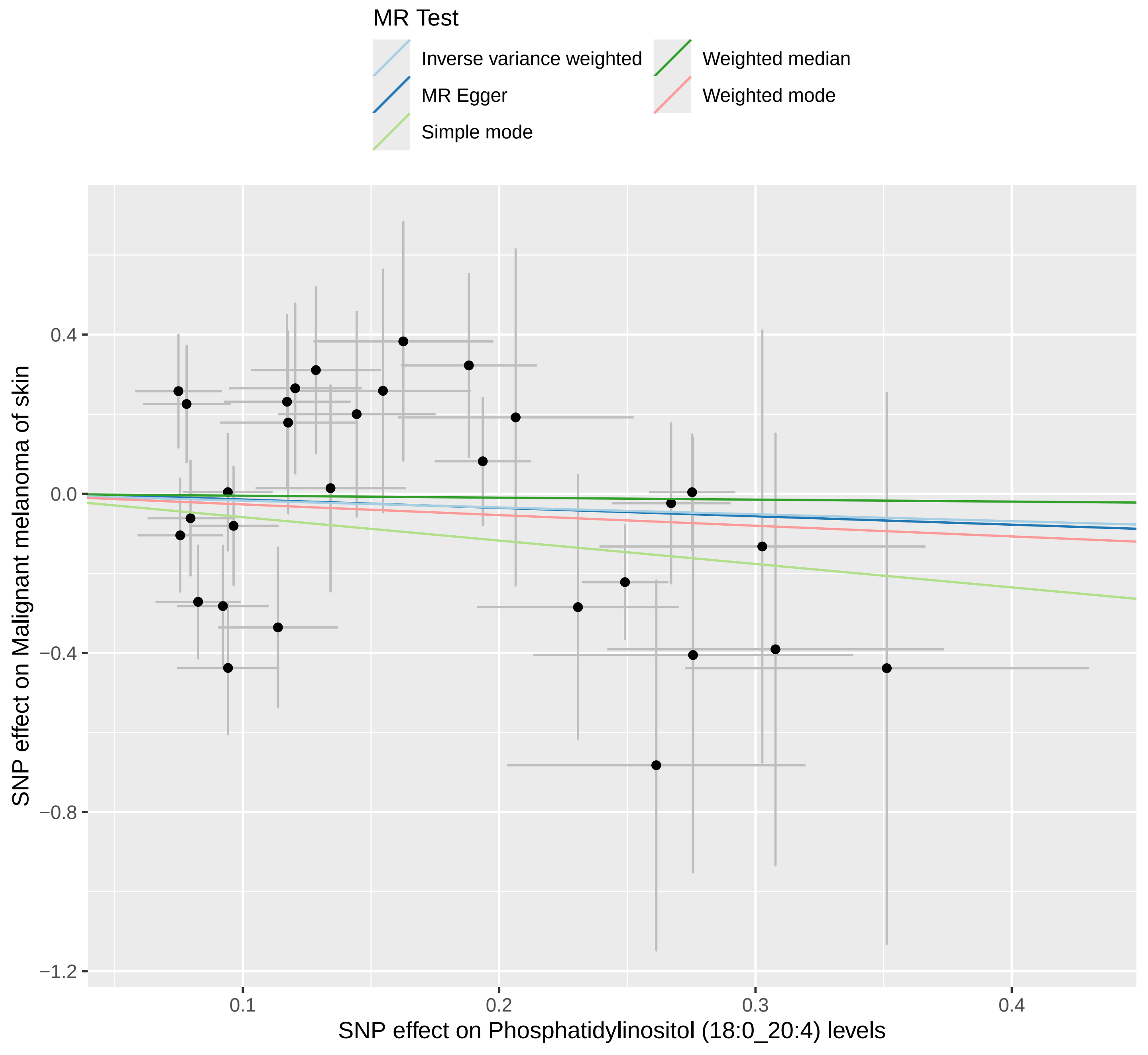
MR Test
Inverse variance weighted
MR Egger
Simple mode
Weighted median
Weighted mode
0.4
0.0
−0.4
−0.8
−1.2
0.1
0.2
0.3
0.4
SNP effect on Malignant melanoma of skin
SNP effect on Phosphatidylinositol (18:0_20:4) levels

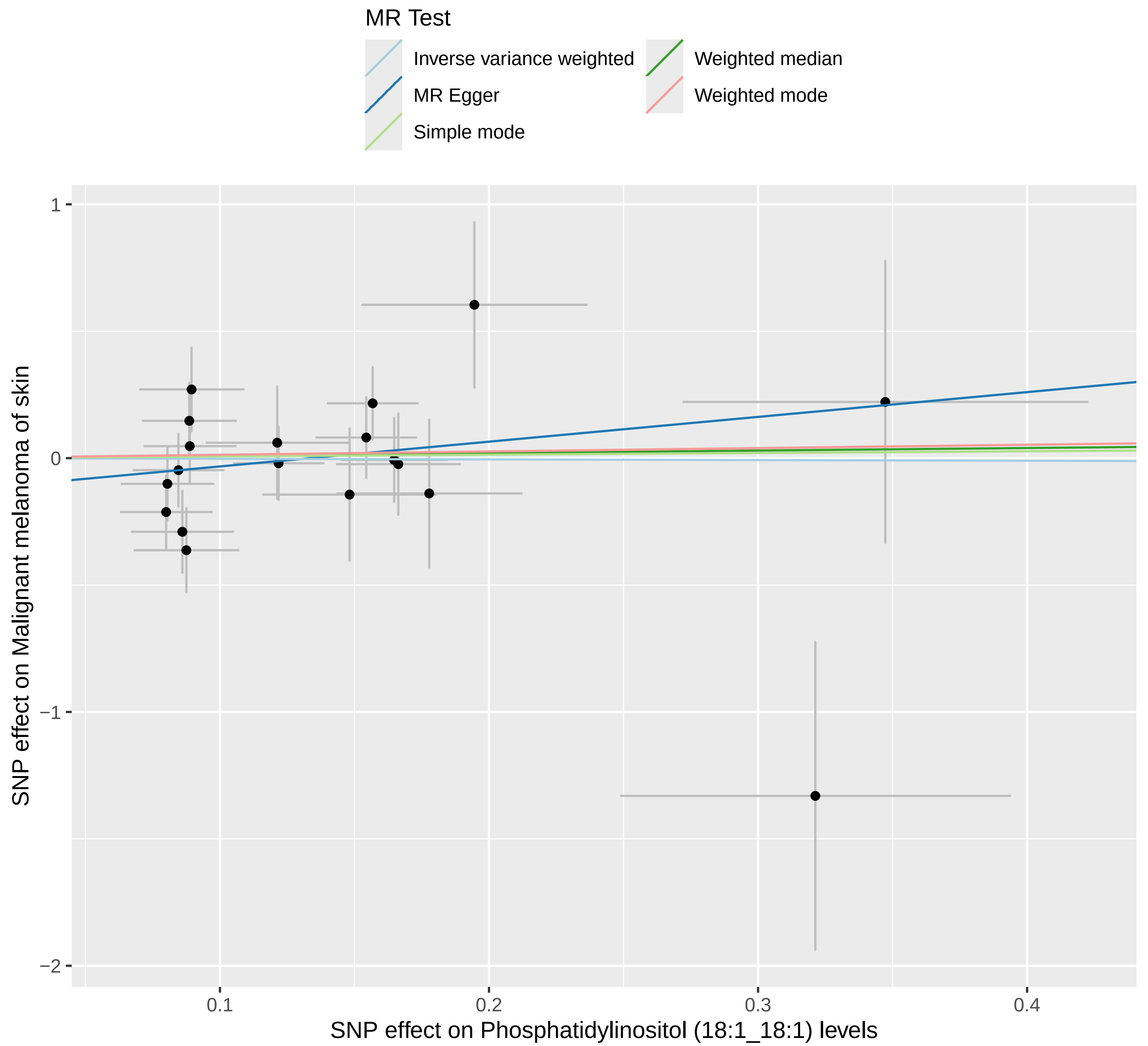
MR Test
Inverse variance weighted
MR Egger
Simple mode
Weighted median
Weighted mode
1
0
−1
−2
0.1
0.2
0.3
0.4
SNP effect on Malignant melanoma of skin
SNP effect on Phosphatidylinositol (18:1_18:1) levels

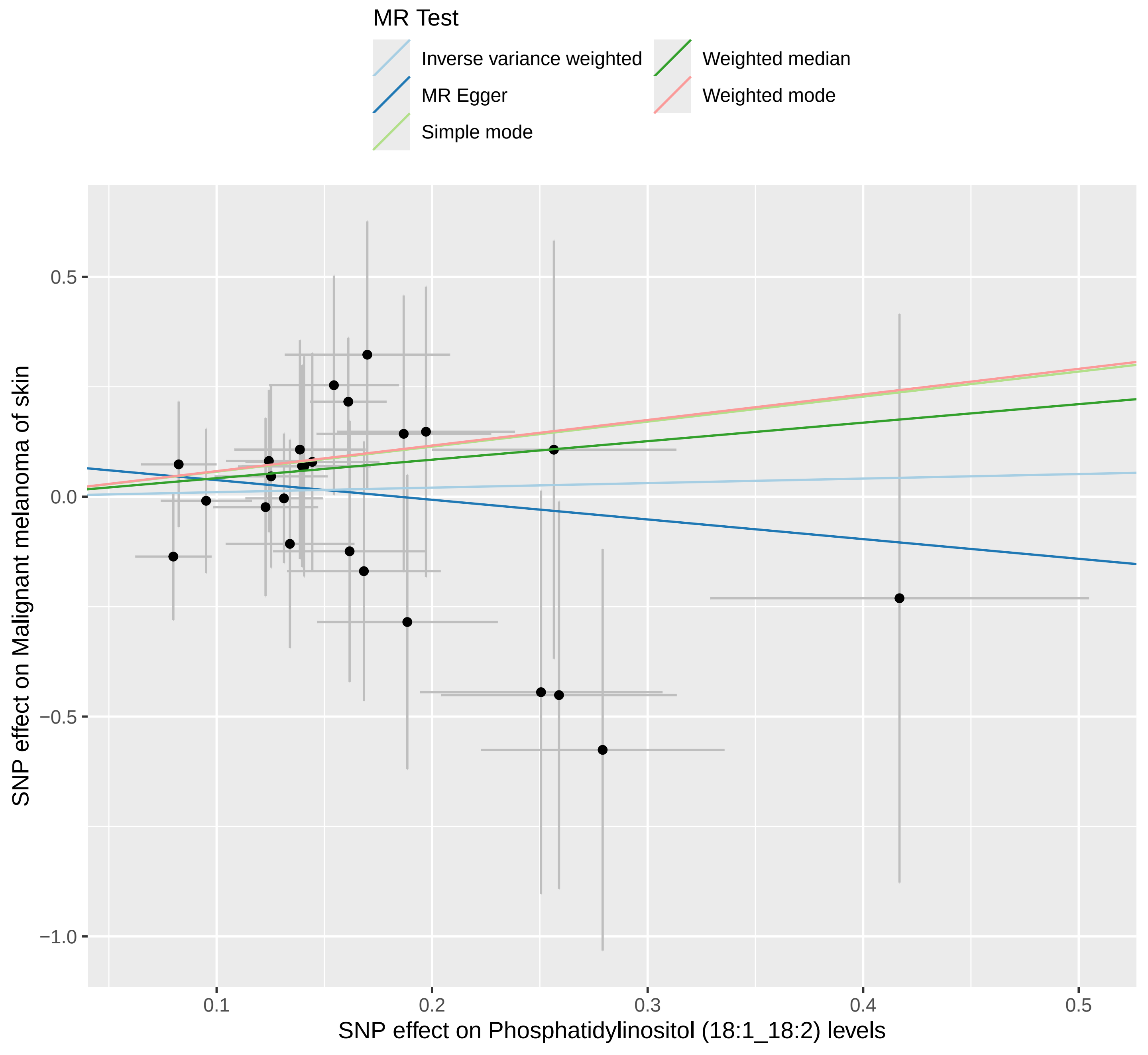
MR Test
Inverse variance weighted
MR Egger
Simple mode
Weighted median
Weighted mode
0.5
0.0
−0.5
−1.0
0.1
0.2
0.3
0.4
0.5
SNP effect on Malignant melanoma of skin
SNP effect on Phosphatidylinositol (18:1_18:2) levels

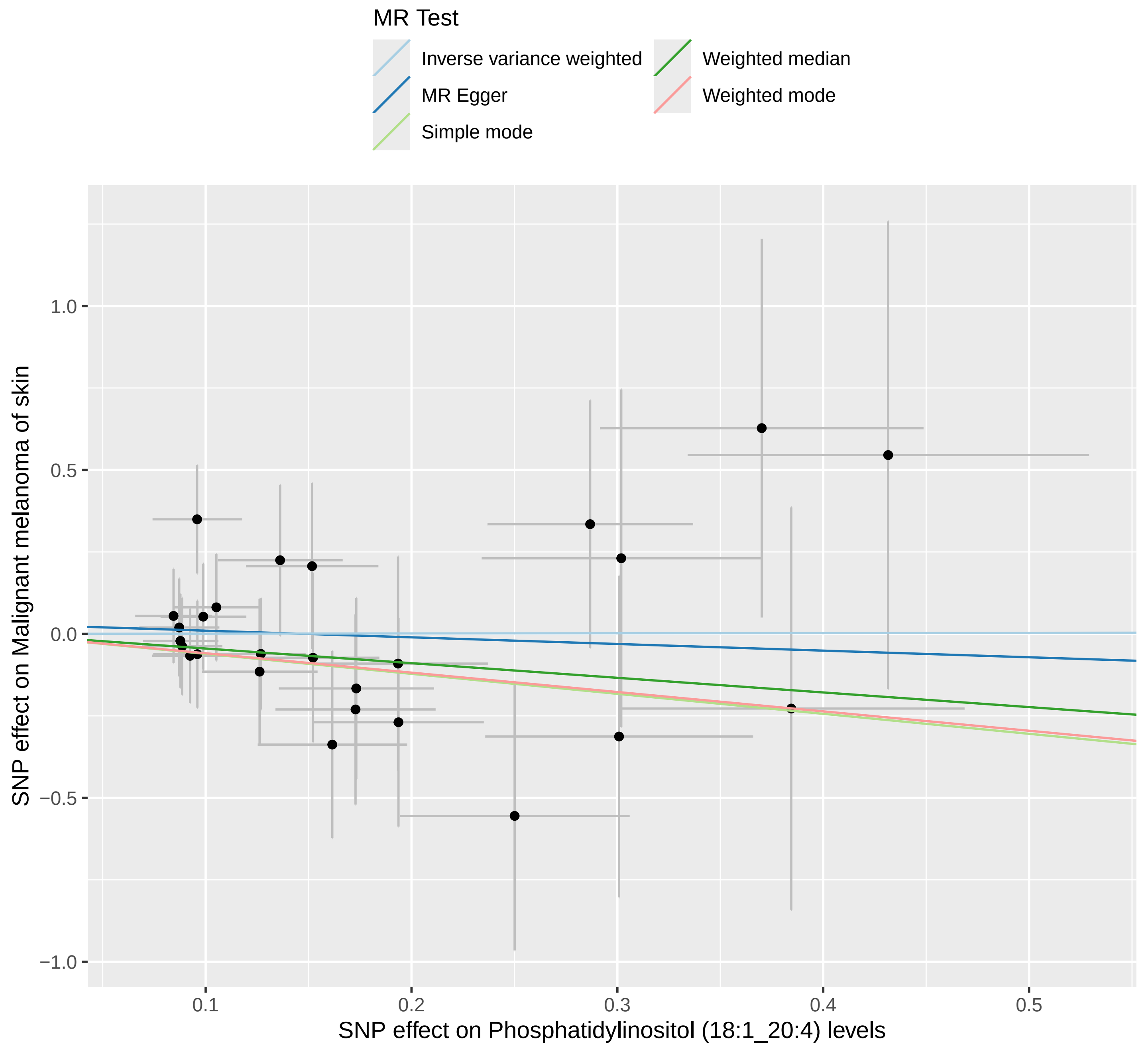
MR Test
Inverse variance weighted
MR Egger
Simple mode
Weighted median
Weighted mode
SNP effect on Malignant melanoma of skin
1.0
0.5
0.0
−0.5
−1.0
0.1
0.2
0.3
0.4
0.5
SNP effect on Phosphatidylinositol (18:1_20:4) levels

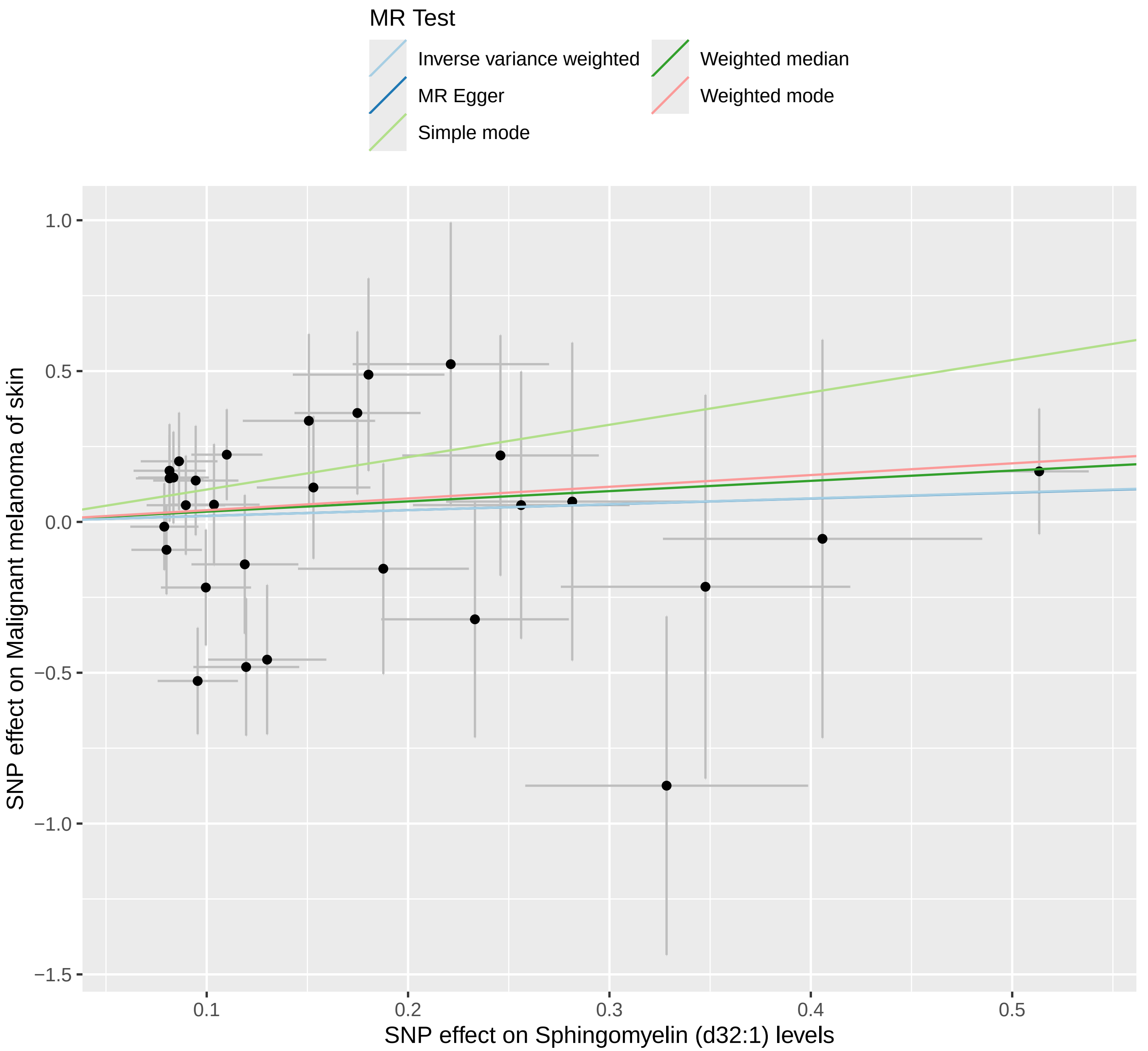
MR Test
Inverse variance weighted
MR Egger
Simple mode
Weighted median
Weighted mode
SNP effect on Malignant melanoma of skin
1.0
0.5
0.0
−0.5
−1.0
−1.5
0.1
0.2
0.3
0.4
0.5
SNP effect on Sphingomyelin (d32:1) levels

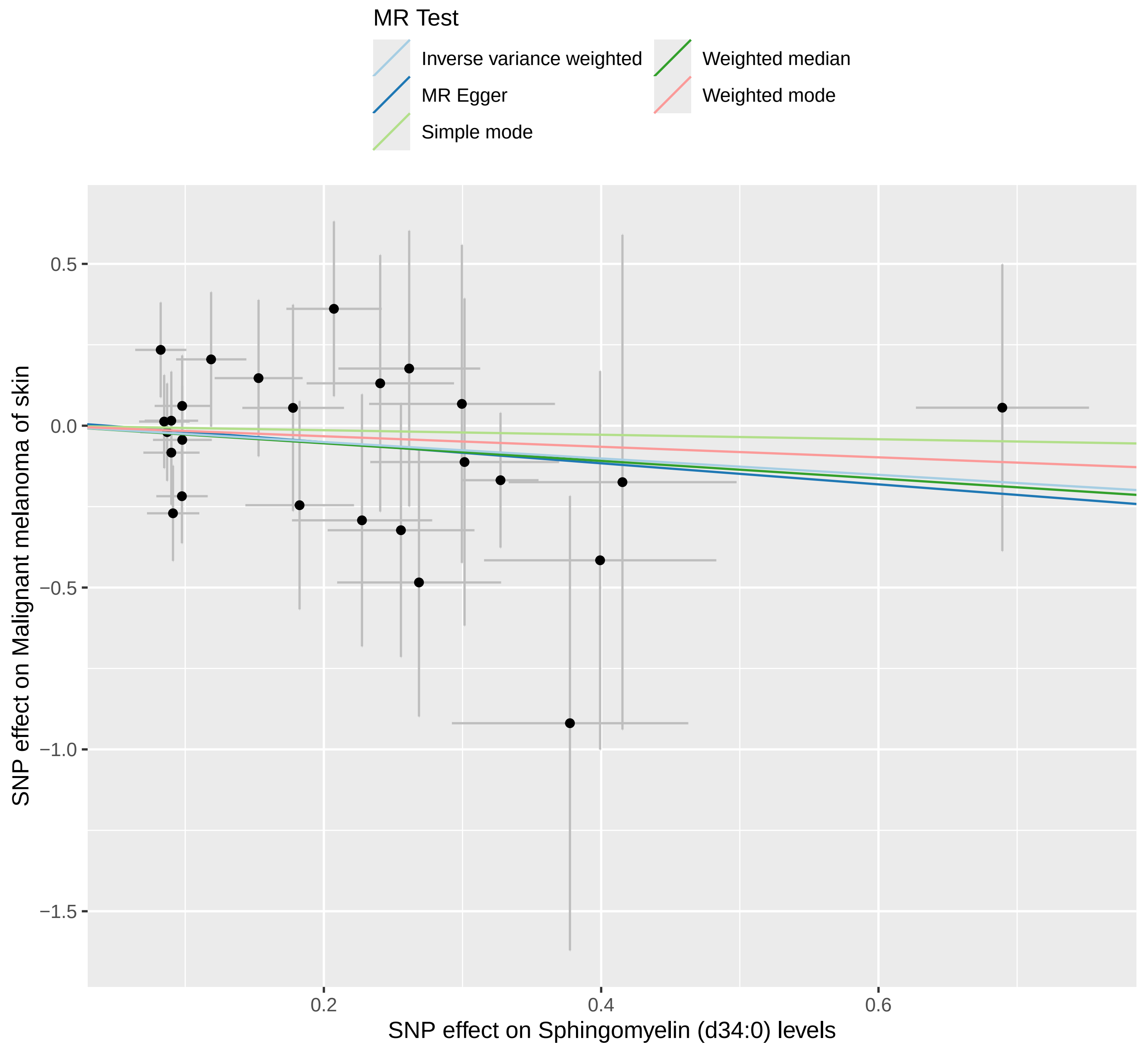
MR Test
Inverse variance weighted
MR Egger
Simple mode
Weighted median
Weighted mode
0.5
0.0
−0.5
−1.0
−1.5
0.2
0.4
0.6
SNP effect on Malignant melanoma of skin
SNP effect on Sphingomyelin (d34:0) levels

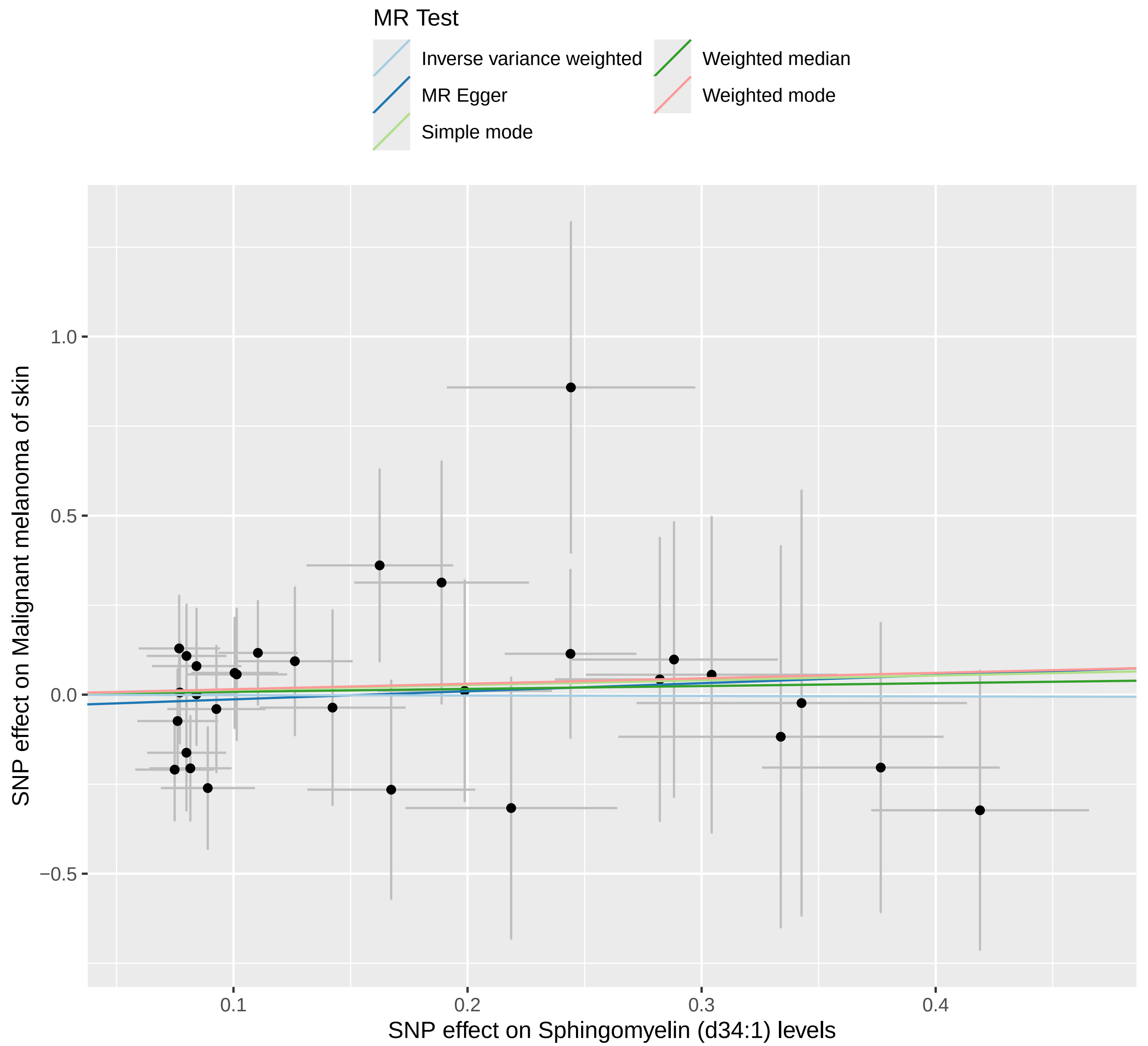
MR Test
Inverse variance weighted
MR Egger
Simple mode
Weighted median
Weighted mode
1.0
0.5
0.0
−0.5
0.1
0.2
0.3
0.4
SNP effect on Malignant melanoma of skin
SNP effect on Sphingomyelin (d34:1) levels

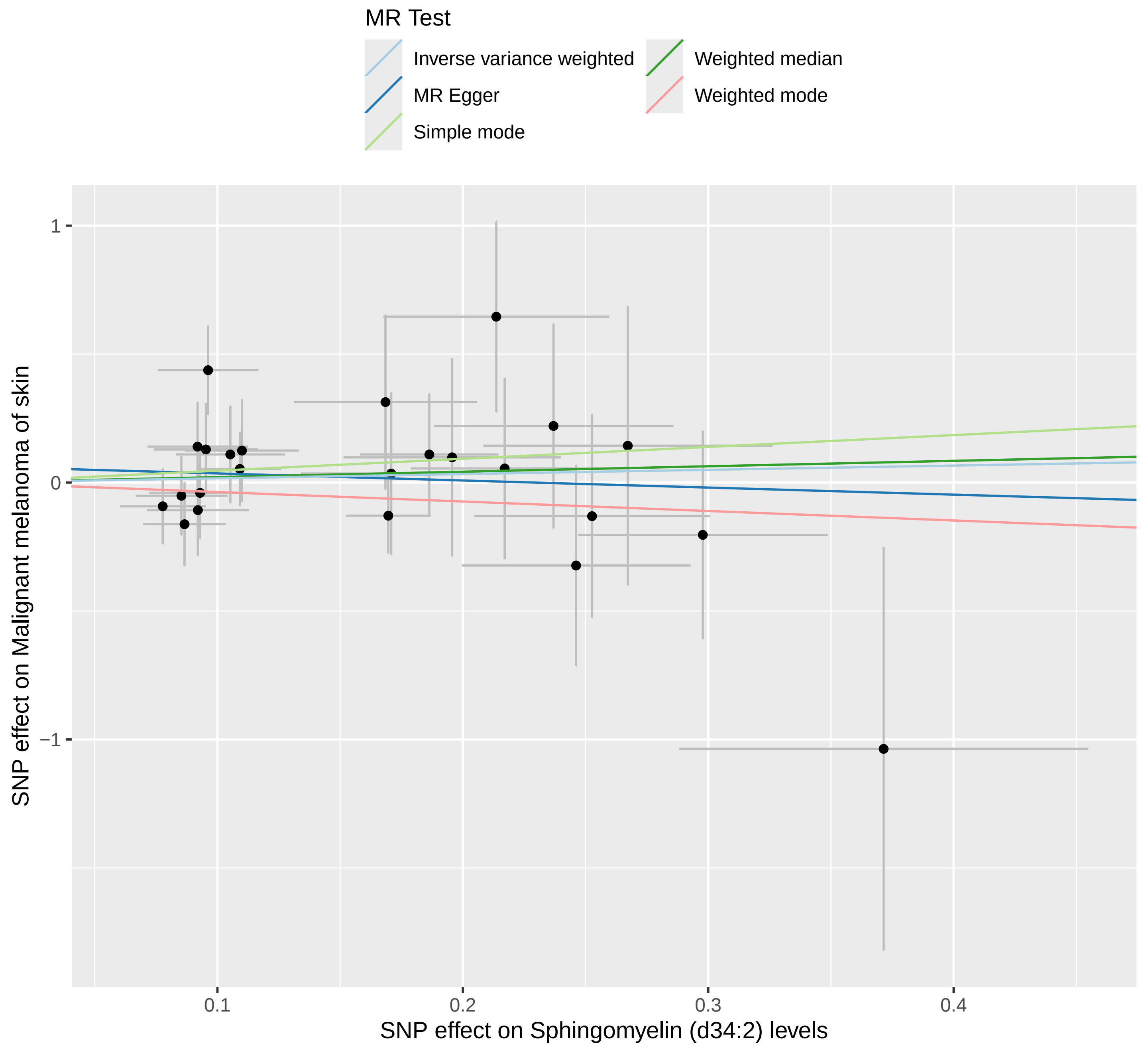

MR Test
Inverse variance weighted
MR Egger
Simple mode
Weighted median
Weighted mode
1
0
−1
0.1
0.2
0.3
0.4
SNP effect on Malignant melanoma of skin
SNP effect on Sphingomyelin (d34:2) levels

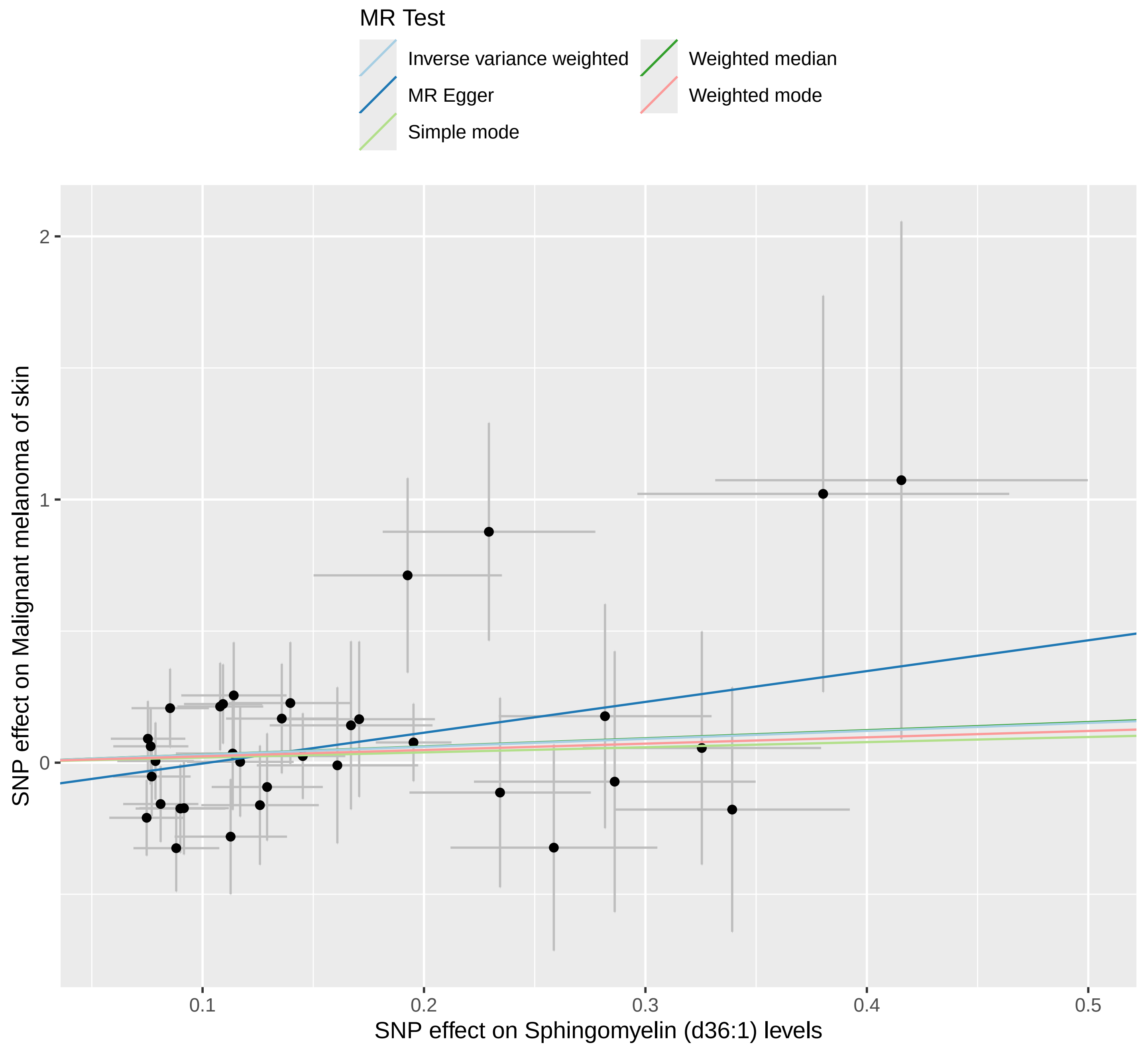

MR Test
Inverse variance weighted
MR Egger
Simple mode
Weighted median
Weighted mode
SNP effect on Malignant melanoma of skin
SNP effect on Sphingomyelin (d36:1) levels
0.1
0.2
0.3
0.4
0.5
0
1
2

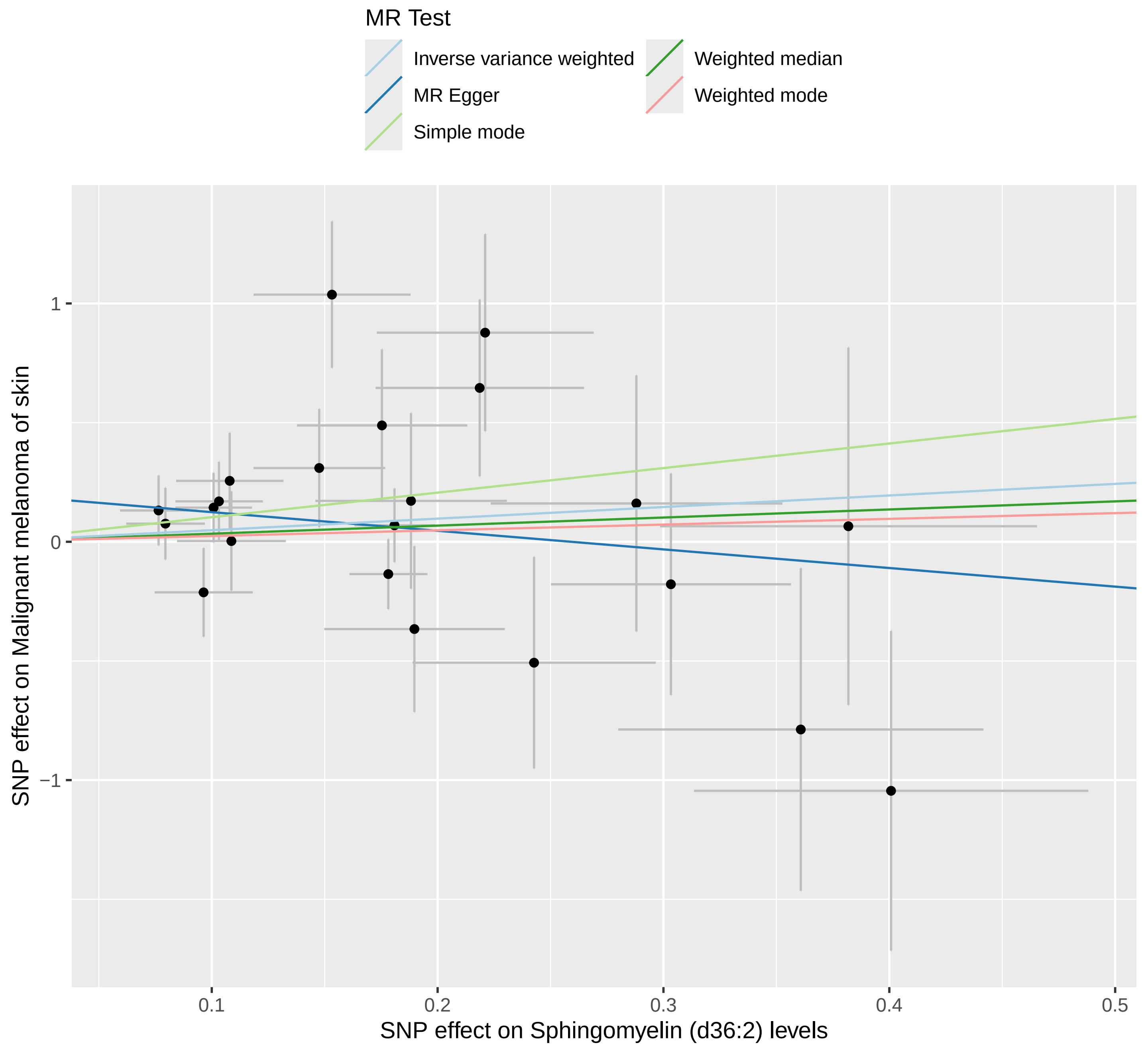
MR Test
Inverse variance weighted
MR Egger
Simple mode
Weighted median
Weighted mode
1
0
−1
0.1
0.2
0.3
0.4
0.5
SNP effect on Sphingomyelin (d36:2) levels
SNP effect on Malignant melanoma of skin

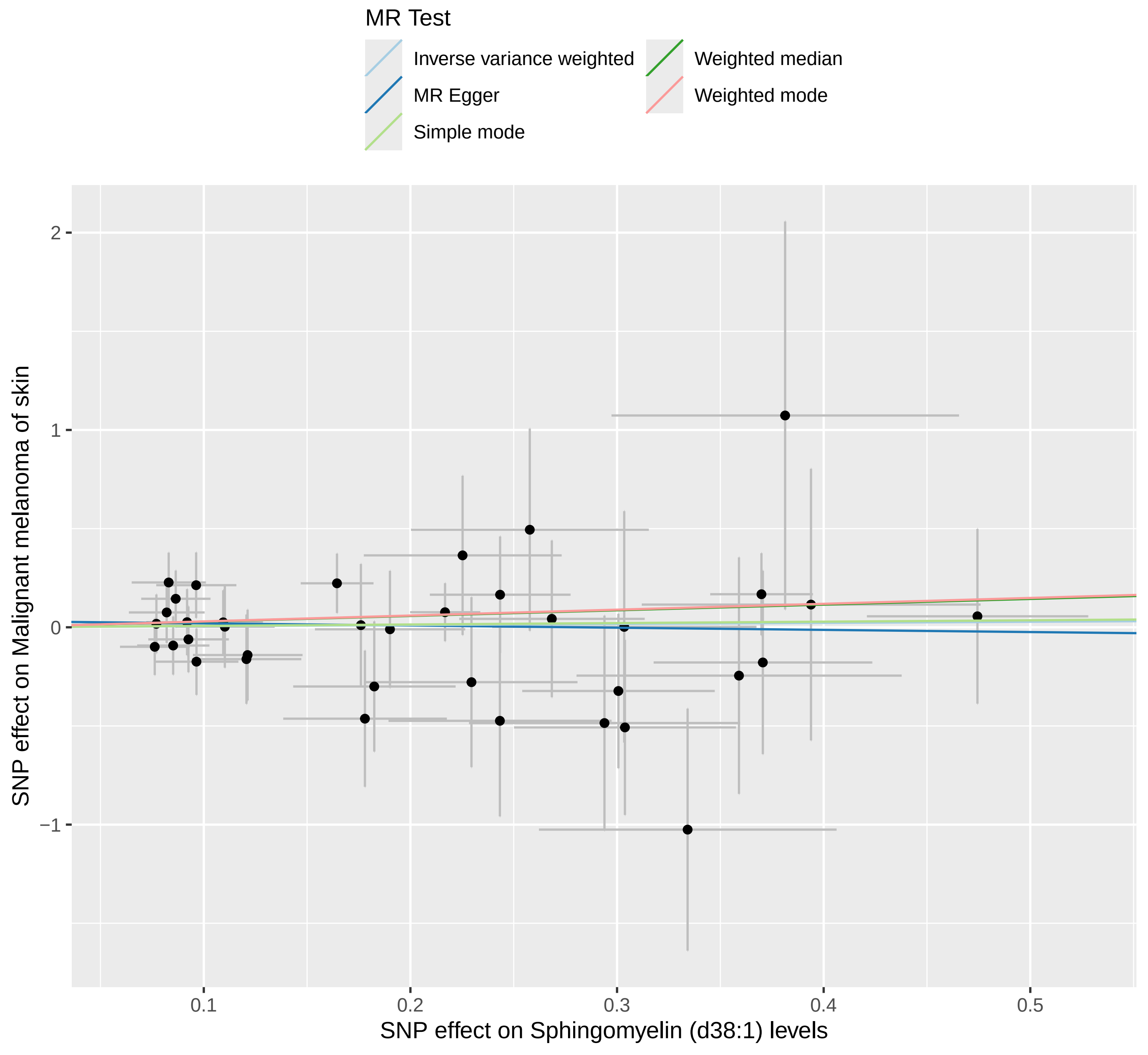

MR Test
Inverse variance weighted
MR Egger
Simple mode
Weighted median
Weighted mode
2
1
0
−1
0.1
0.2
0.3
0.4
0.5
SNP effect on Malignant melanoma of skin
SNP effect on Sphingomyelin (d38:1) levels

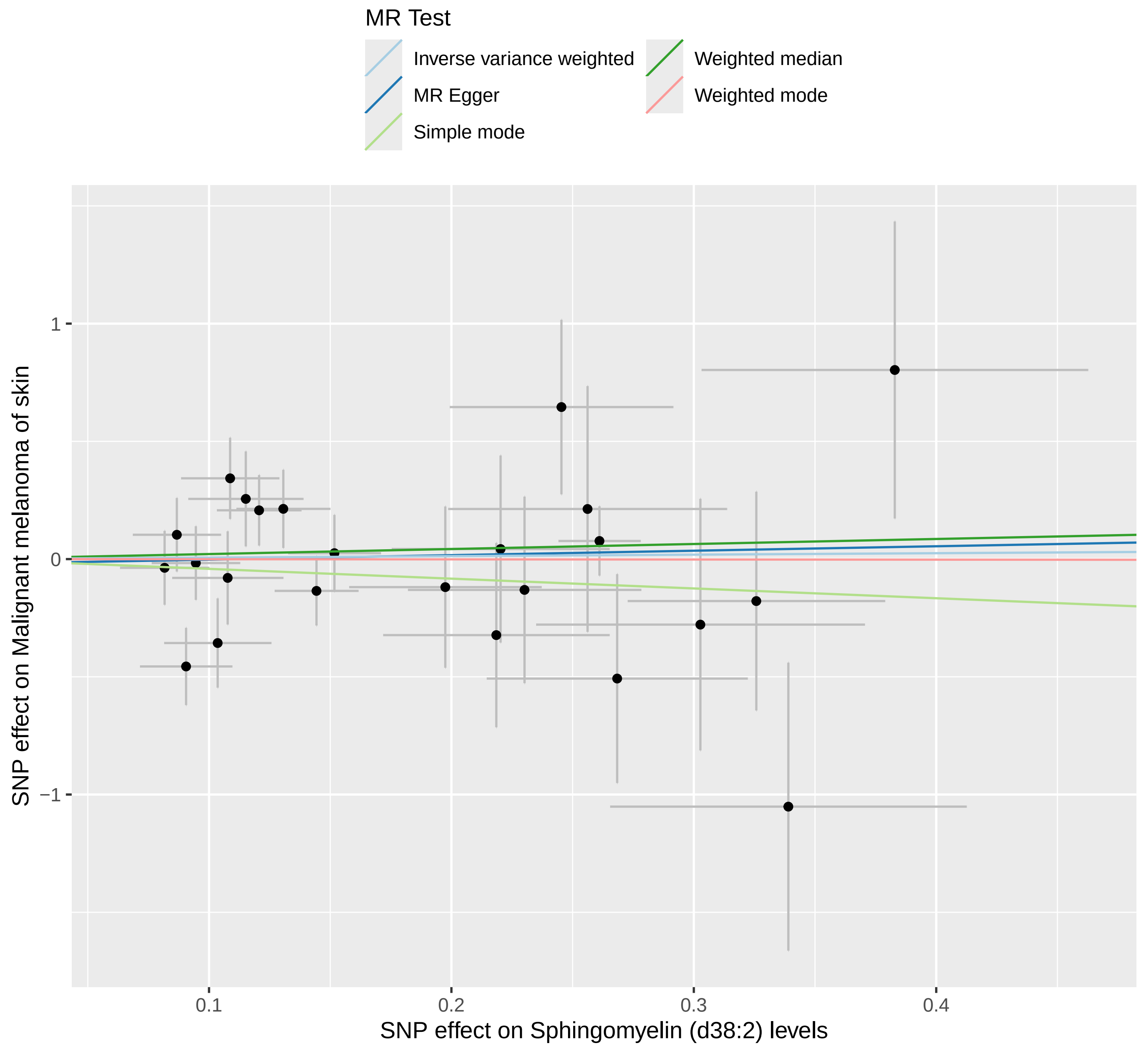

MR Test
Inverse variance weighted
MR Egger
Simple mode
Weighted median
Weighted mode
1
0
−1
0.1
0.2
0.3
0.4
SNP effect on Malignant melanoma of skin
SNP effect on Sphingomyelin (d38:2) levels

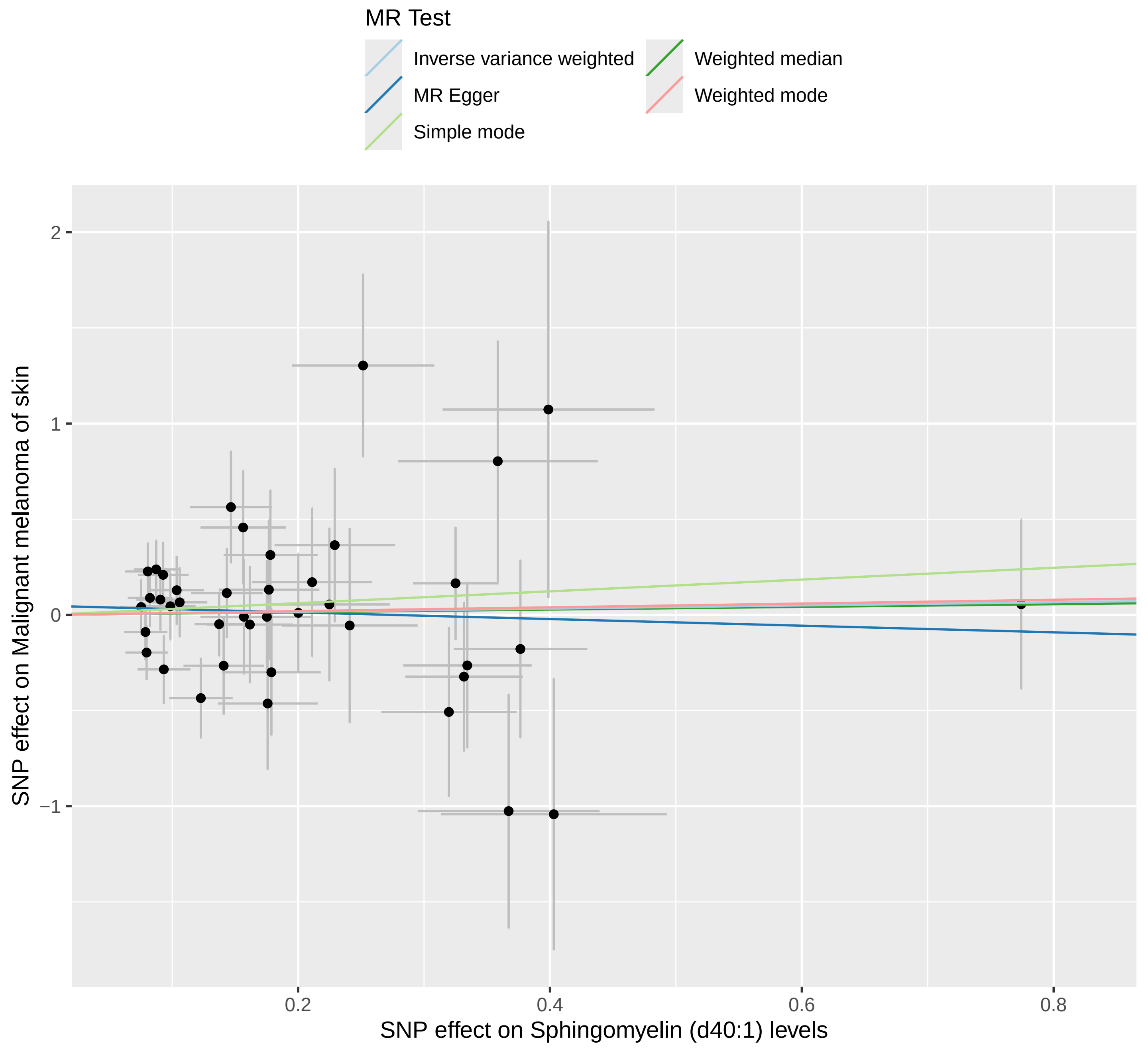
MR Test
Inverse variance weighted
MR Egger
Simple mode
Weighted median
Weighted mode
SNP effect on Malignant melanoma of skin
2
1
0
−1
0.2
0.4
0.6
0.8
SNP effect on Sphingomyelin (d40:1) levels

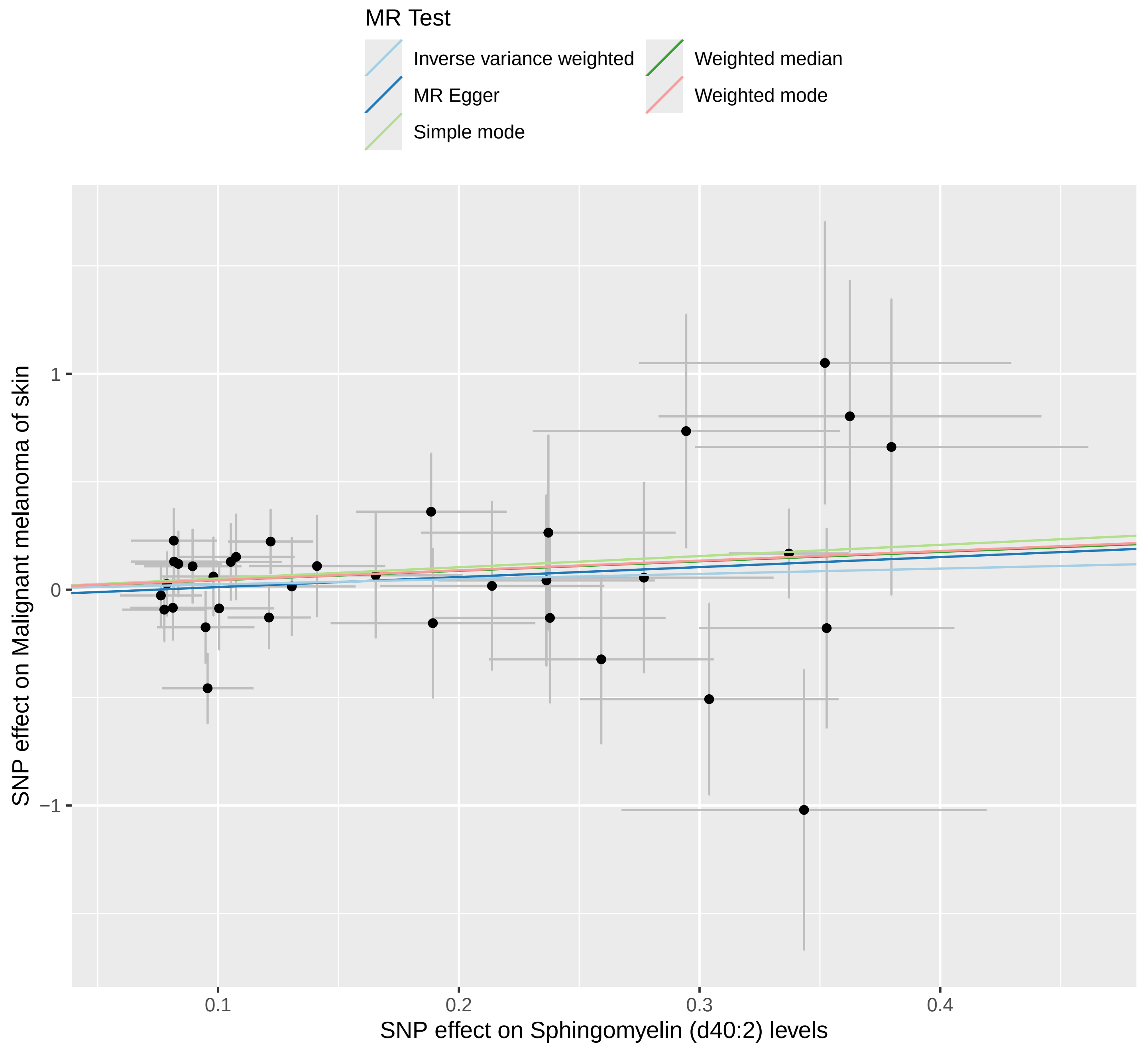

MR Test
Inverse variance weighted
MR Egger
Simple mode
Weighted median
Weighted mode
SNP effect on Malignant melanoma of skin
1
0
−1
0.1
0.2
0.3
0.4
SNP effect on Sphingomyelin (d40:2) levels

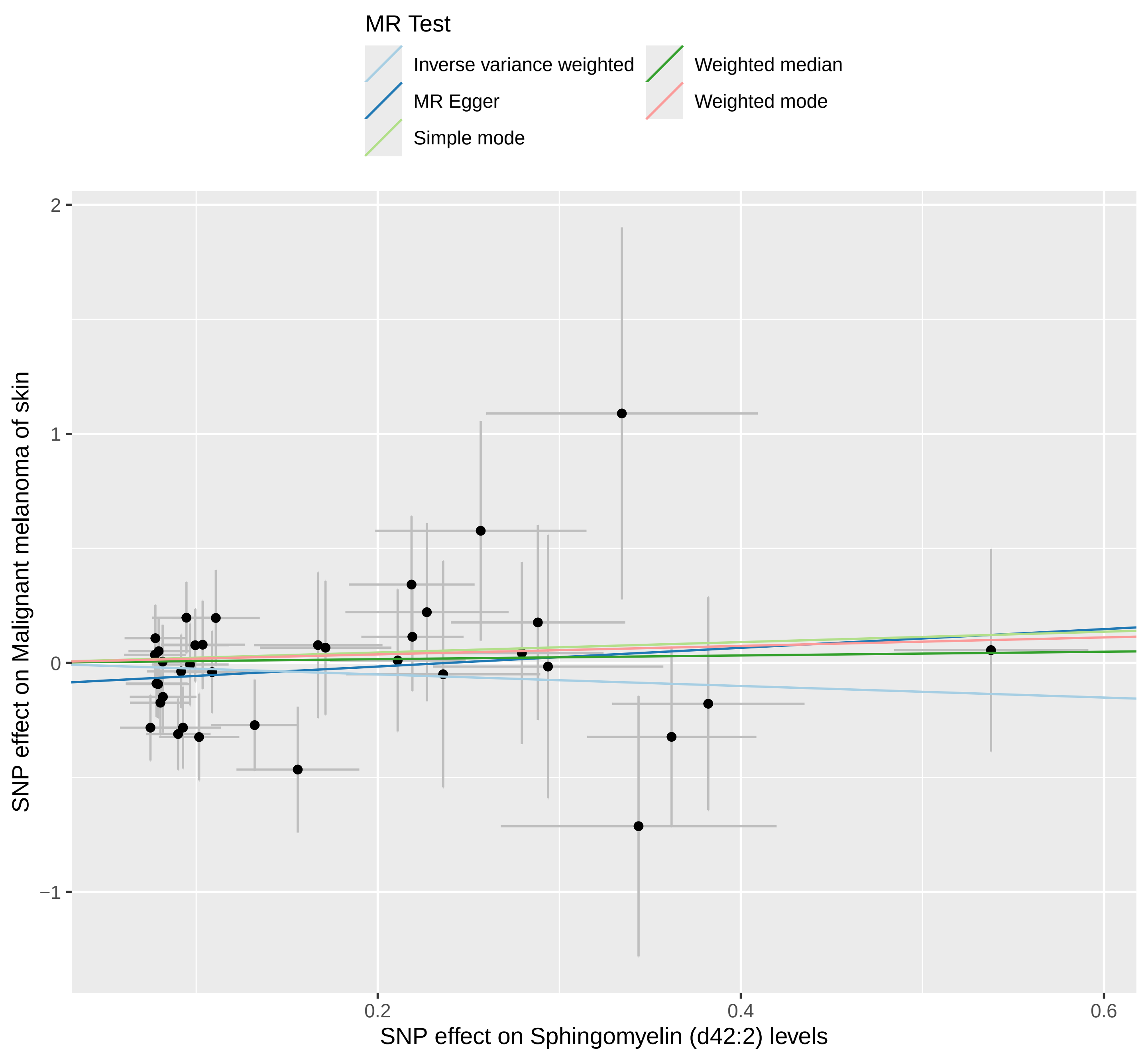

MR Test
Inverse variance weighted
MR Egger
Simple mode
Weighted median
Weighted mode
2
1
0
−1
0.2
0.4
0.6
SNP effect on Malignant melanoma of skin
SNP effect on Sphingomyelin (d42:2) levels

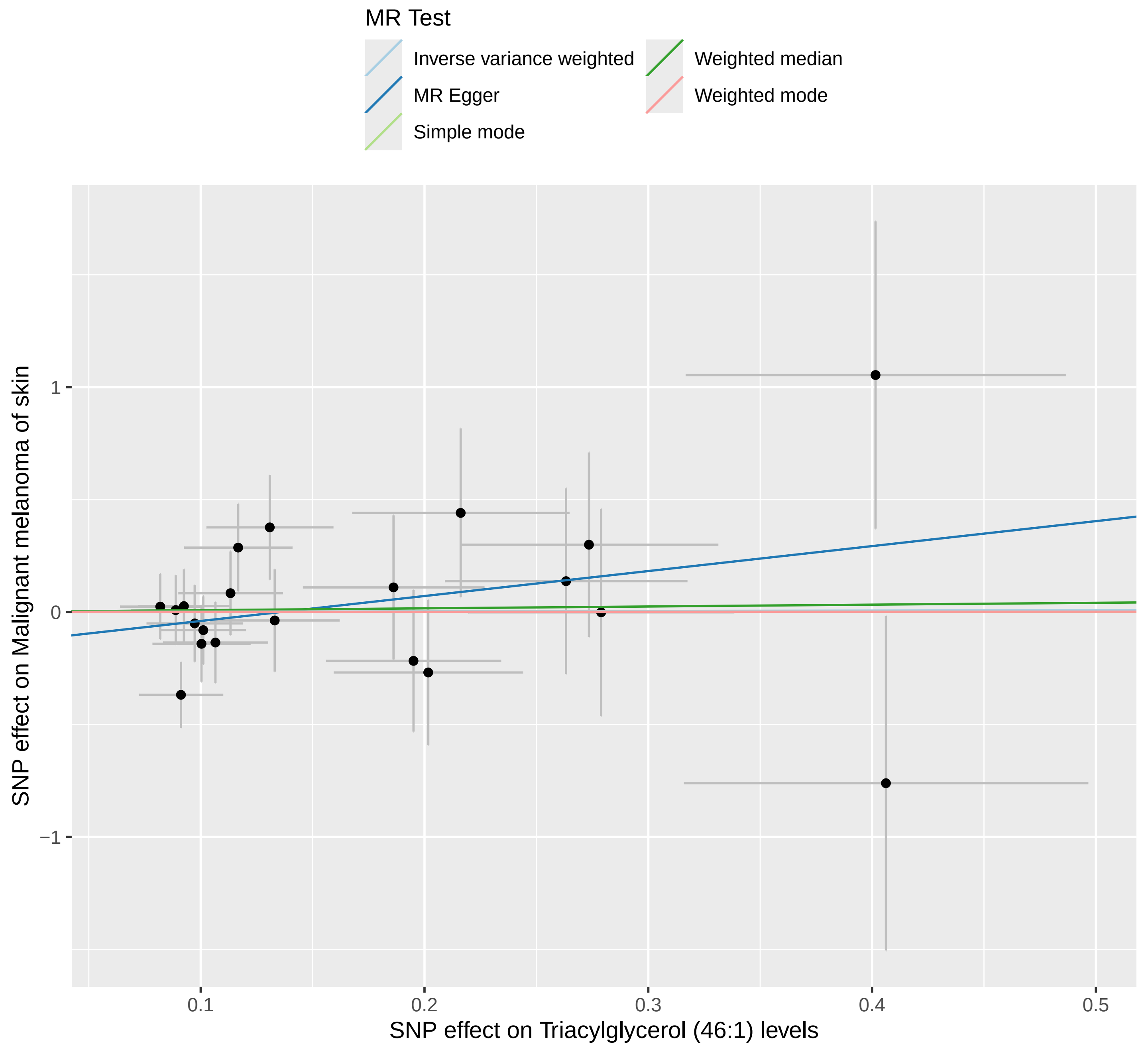
MR Test
Inverse variance weighted
MR Egger
Simple mode
Weighted median
Weighted mode
SNP effect on Malignant melanoma of skin
1
0
−1
0.1
0.2
0.3
0.4
0.5
SNP effect on Triacylglycerol (46:1) levels

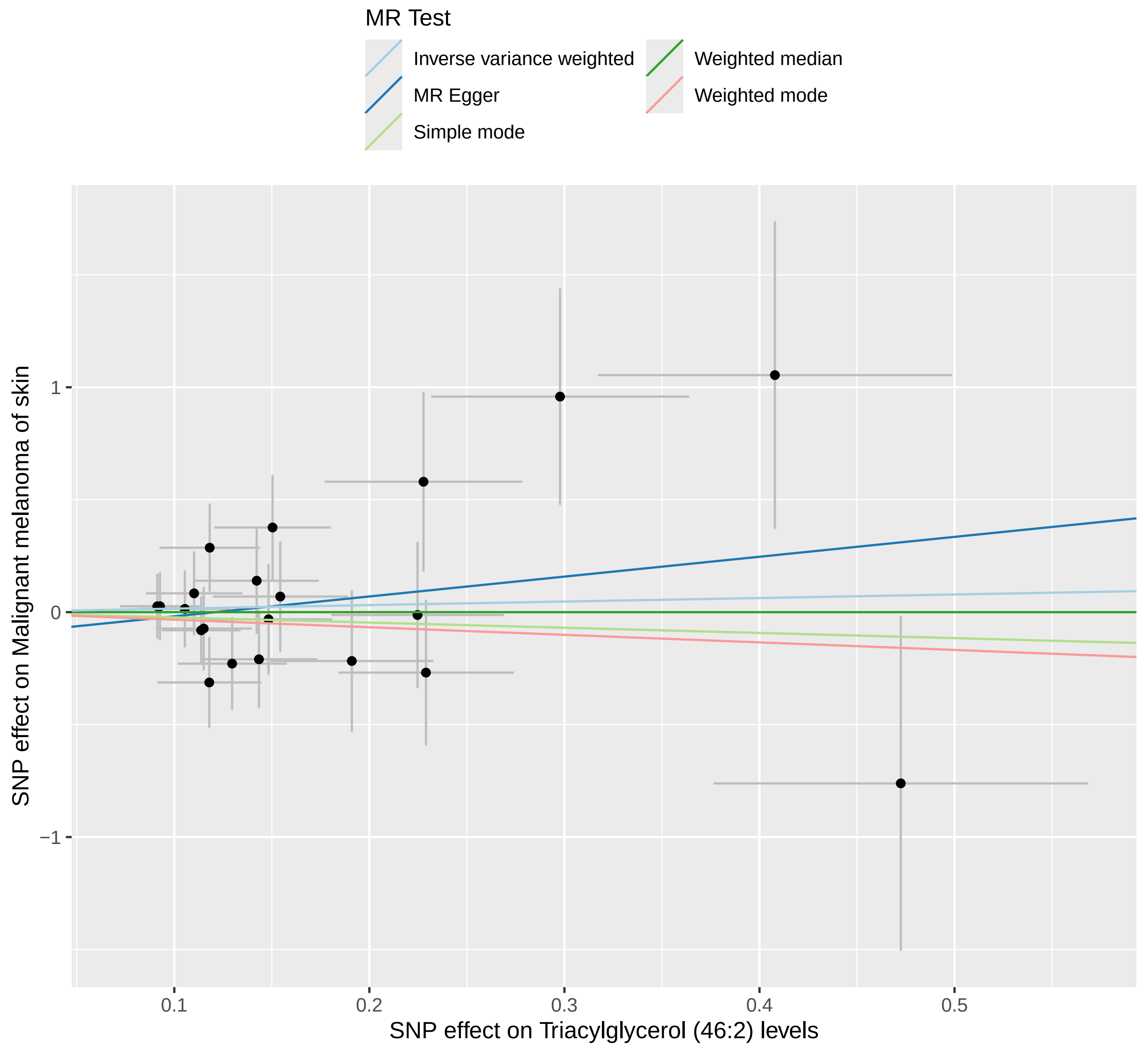
MR Test
Inverse variance weighted
MR Egger
Simple mode
Weighted median
Weighted mode
SNP effect on Malignant melanoma of skin
1
0
−1
0.1
0.2
0.3
0.4
0.5
SNP effect on Triacylglycerol (46:2) levels

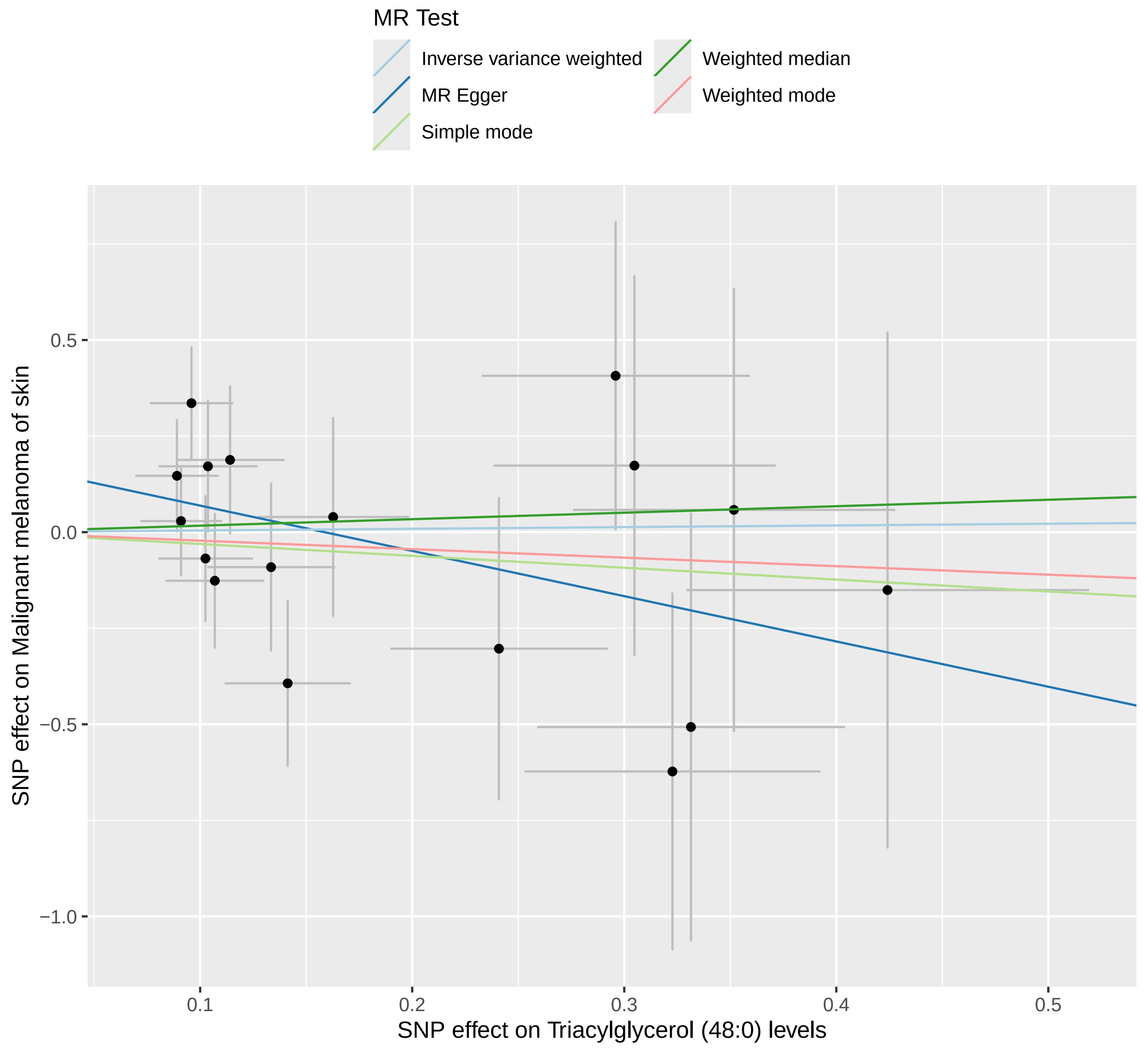
MR Test
Inverse variance weighted
MR Egger
Simple mode
Weighted median
Weighted mode
0.5
0.0
−0.5
−1.0
SNP effect on Malignant melanoma of skin
0.1
0.2
0.3
0.4
0.5
SNP effect on Triacylglycerol (48:0) levels

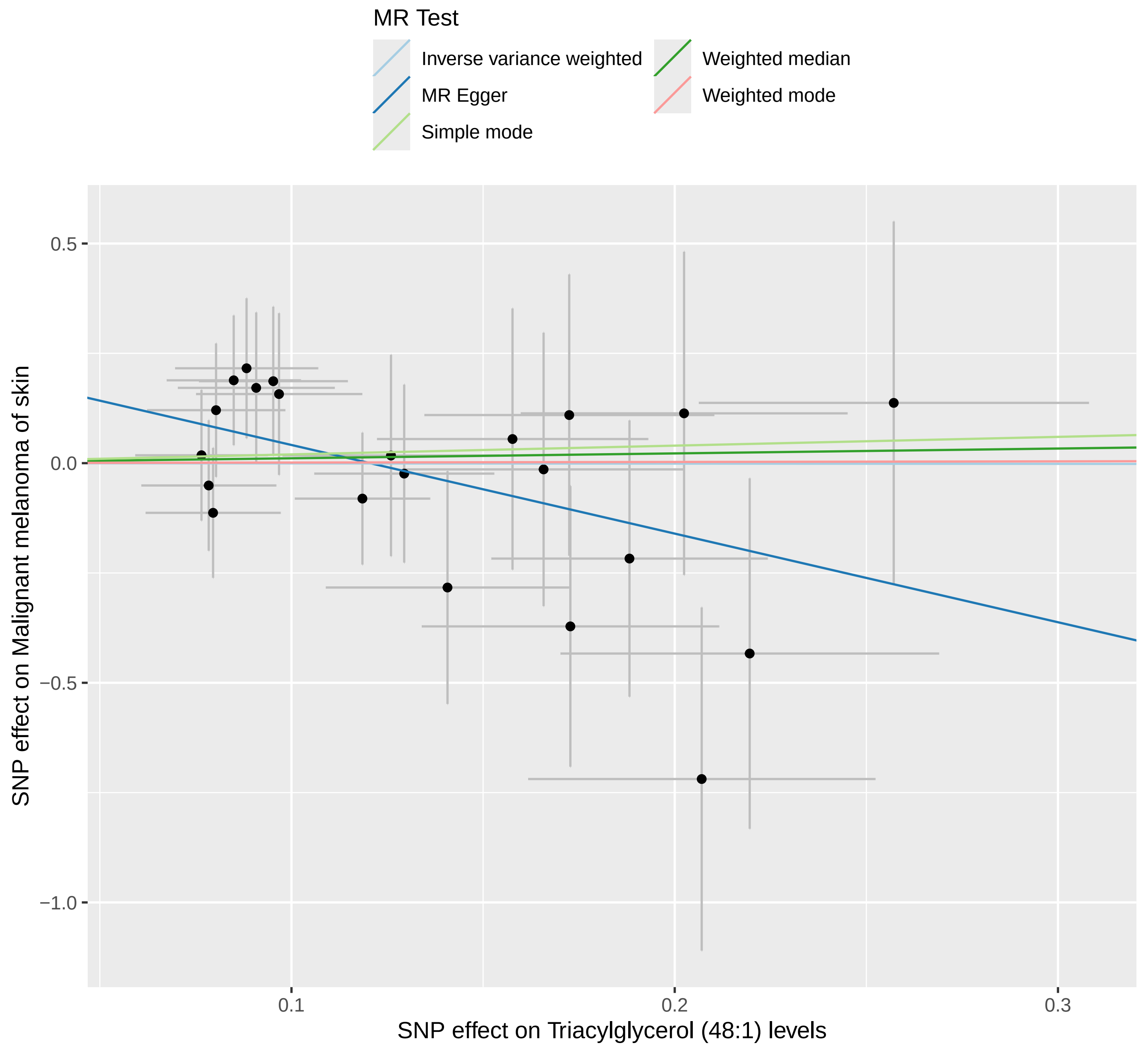
MR Test
Inverse variance weighted
MR Egger
Simple mode
Weighted median
Weighted mode
0.5
0.0
−0.5
−1.0
0.1
0.2
0.3
SNP effect on Malignant melanoma of skin
SNP effect on Triacylglycerol (48:1) levels

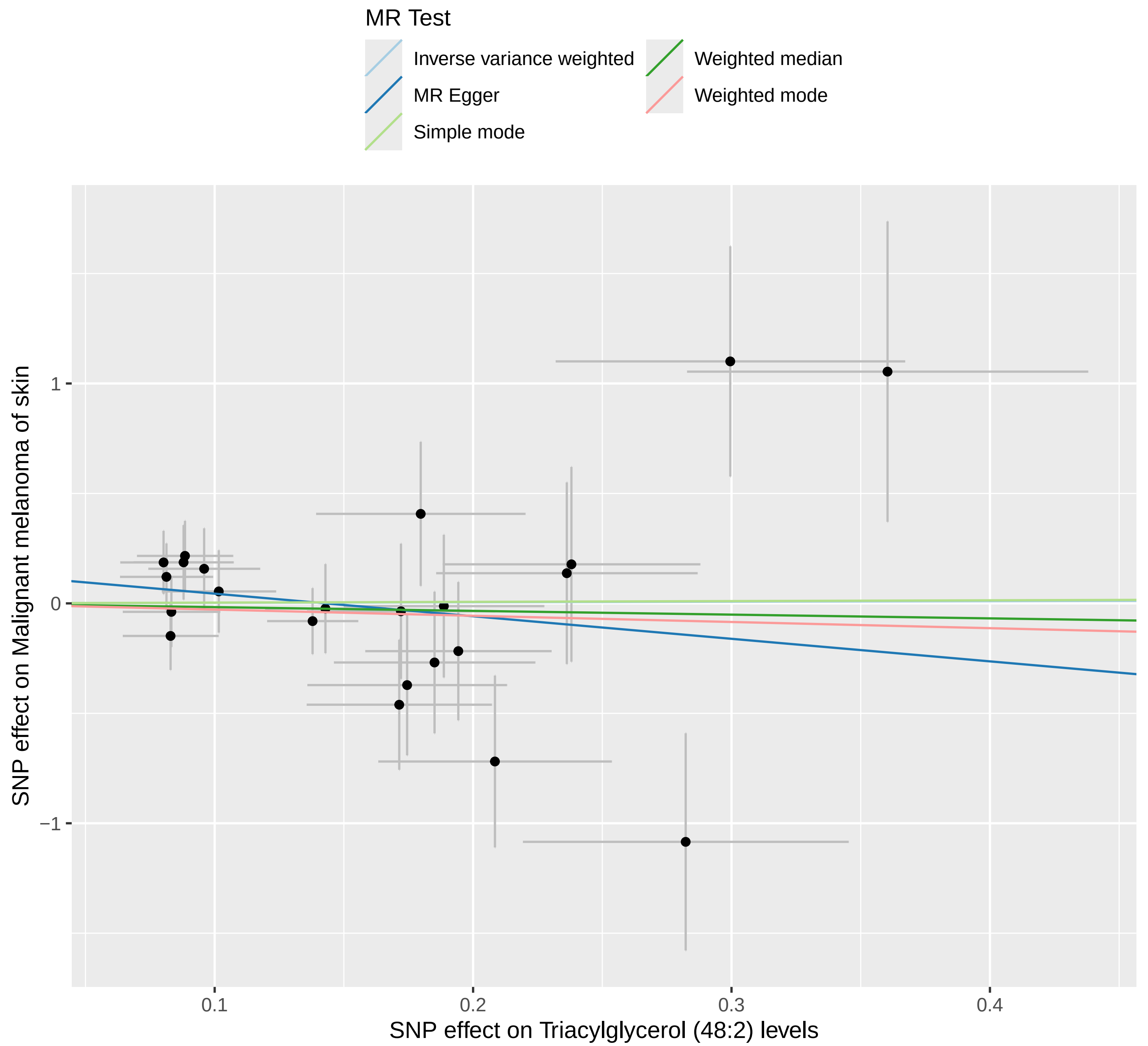
MR Test
Inverse variance weighted
MR Egger
Simple mode
Weighted median
Weighted mode
1
0
−1
0.1
0.2
0.3
0.4
SNP effect on Malignant melanoma of skin
SNP effect on Triacylglycerol (48:2) levels

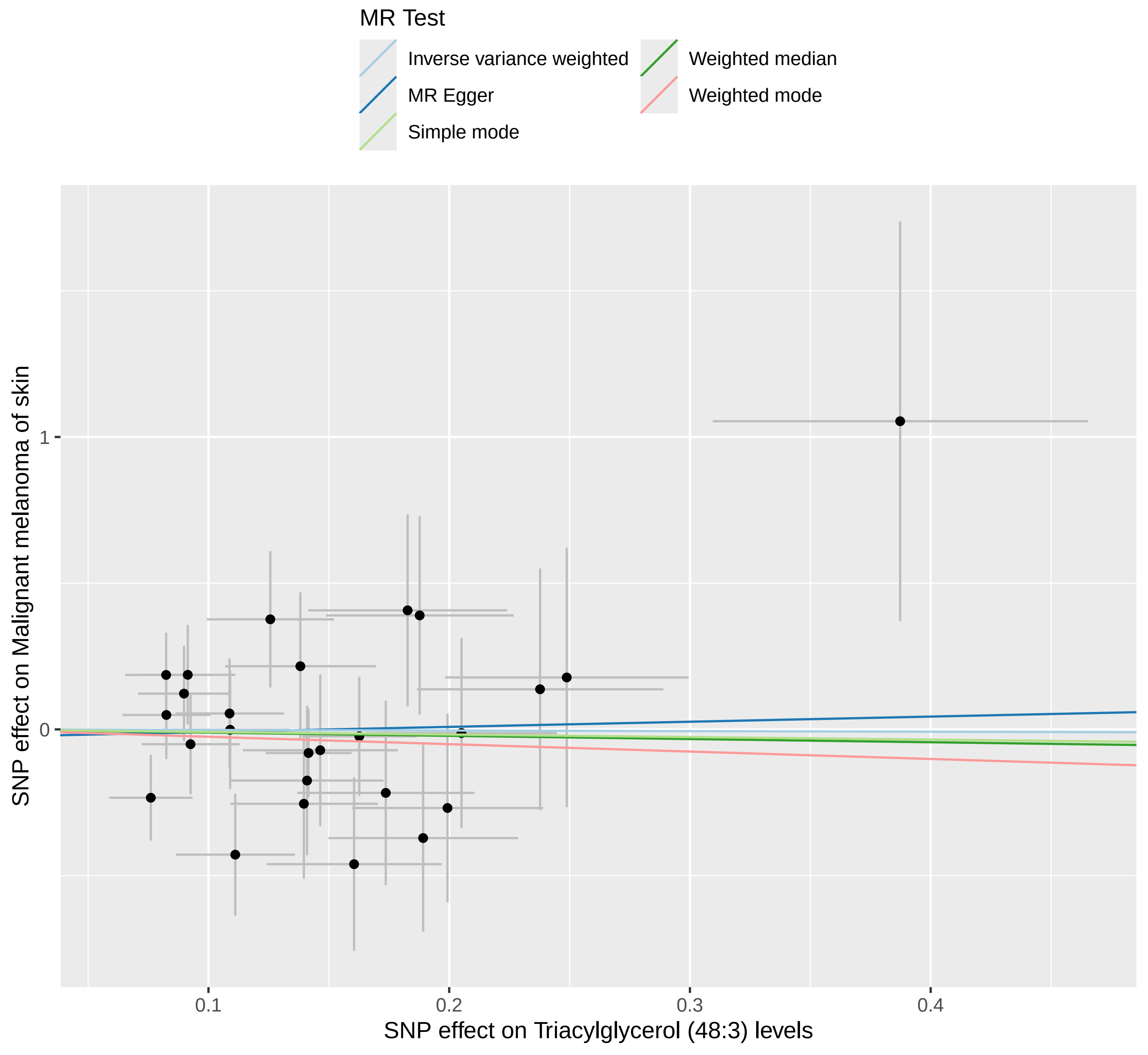
MR Test
Inverse variance weighted
MR Egger
Simple mode
Weighted median
Weighted mode
1
0
0.1
0.2
0.3
0.4
SNP effect on Triacylglycerol (48:3) levels
SNP effect on Malignant melanoma of skin

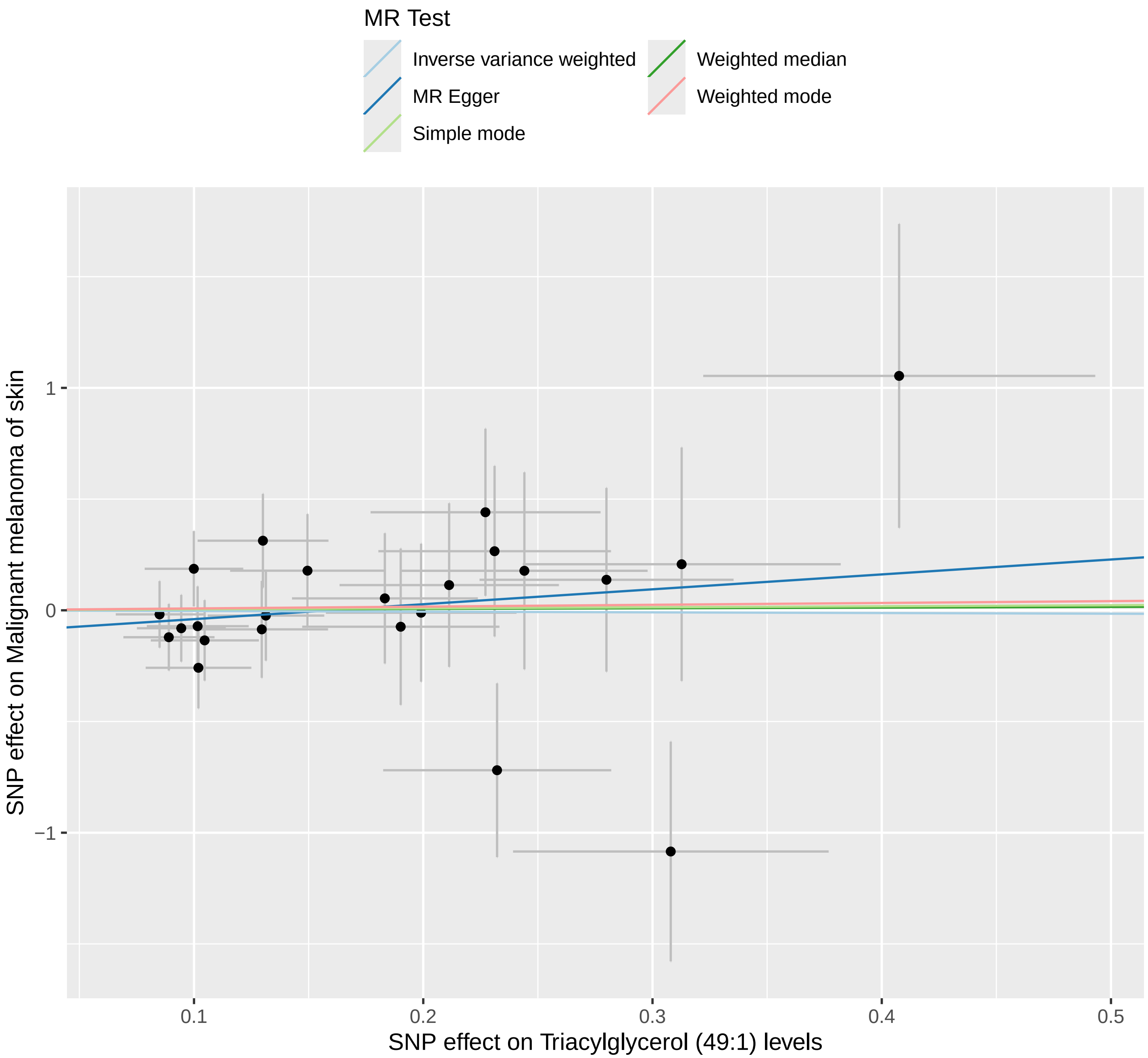

MR Test
Inverse variance weighted
MR Egger
Simple mode
Weighted median
Weighted mode
SNP effect on Malignant melanoma of skin
1
0
−1
0.1
0.2
0.3
0.4
0.5
SNP effect on Triacylglycerol (49:1) levels

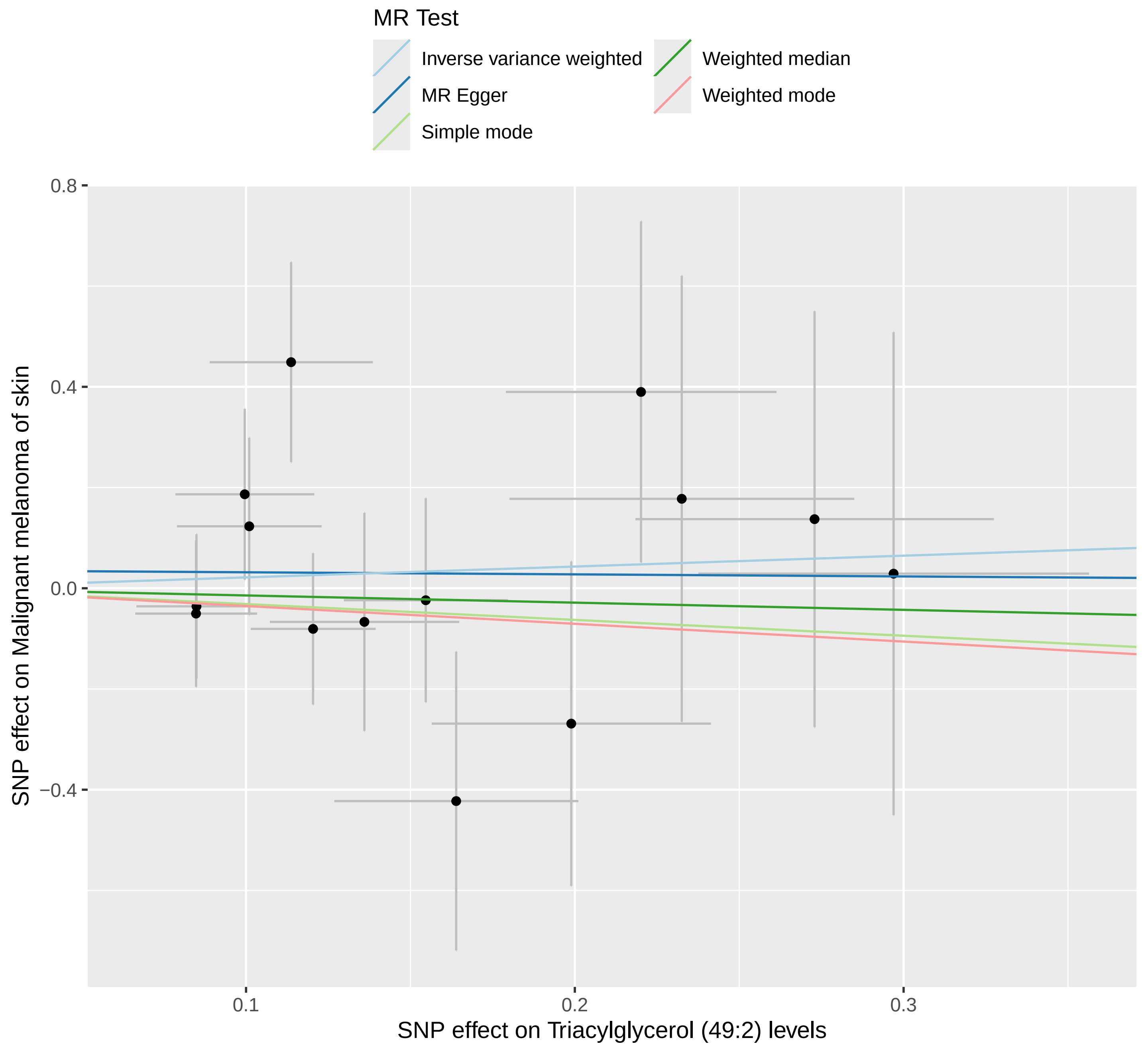

MR Test
Inverse variance weighted
MR Egger
Simple mode
Weighted median
Weighted mode
0.8
0.4
0.0
−0.4
0.1
0.2
0.3
SNP effect on Malignant melanoma of skin
SNP effect on Triacylglycerol (49:2) levels

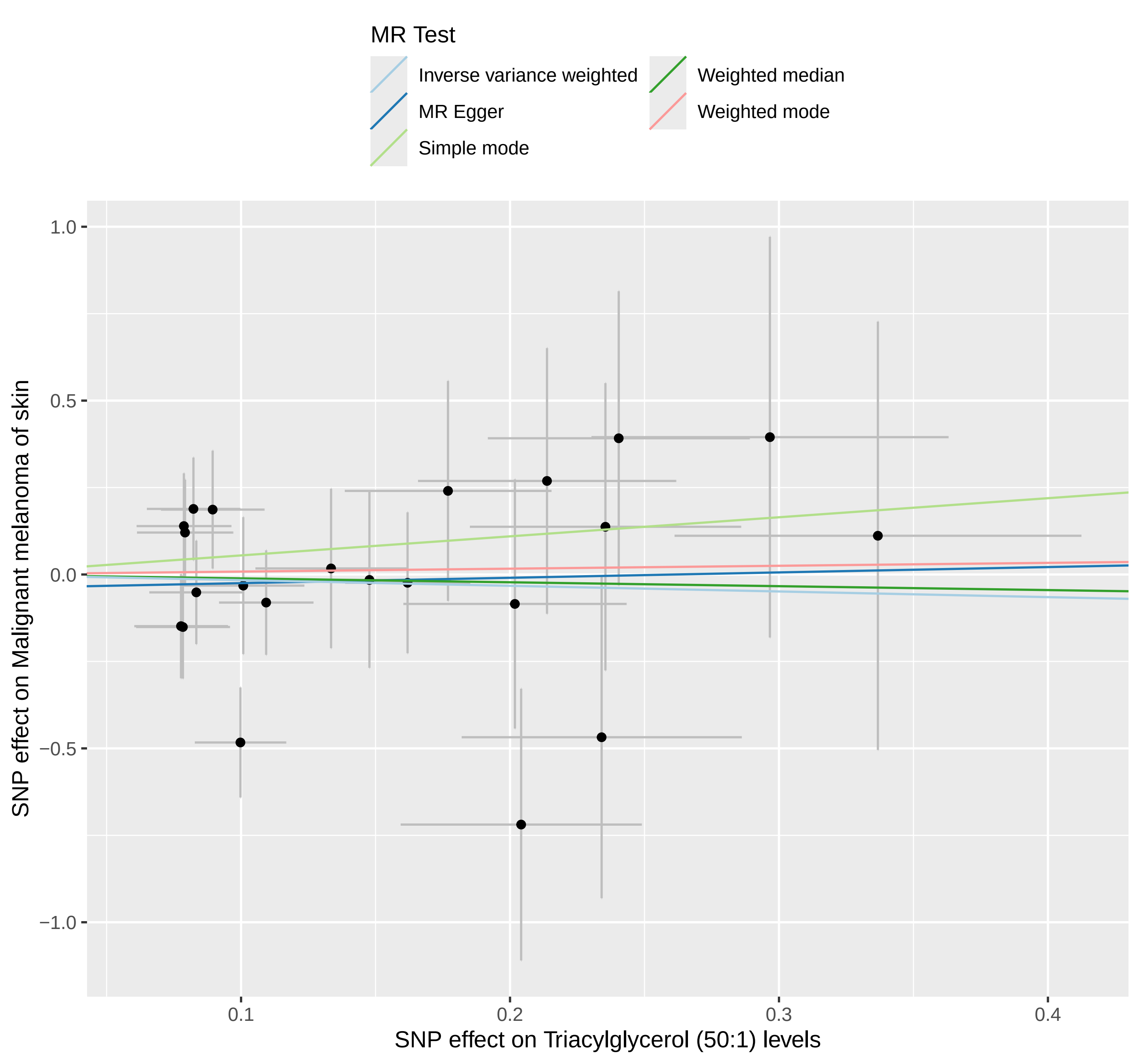

MR Test
Inverse variance weighted
MR Egger
Simple mode
Weighted median
Weighted mode
1.0
0.5
0.0
−0.5
−1.0
0.1
0.2
0.3
0.4
SNP effect on Malignant melanoma of skin
SNP effect on Triacylglycerol (50:1) levels

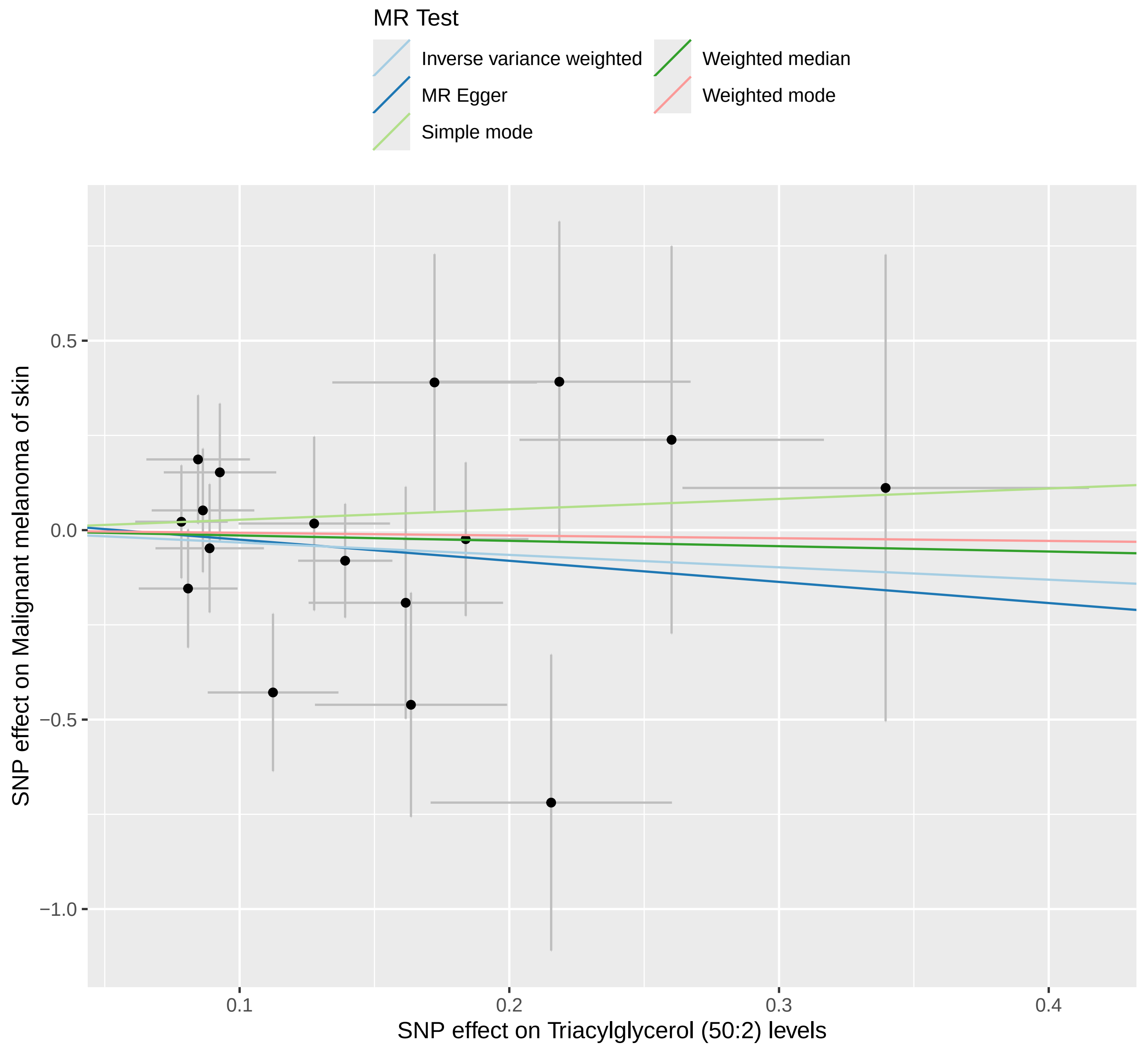

MR Test
Inverse variance weighted
MR Egger
Simple mode
Weighted median
Weighted mode
0.5
0.0
−0.5
−1.0
0.1
0.2
0.3
0.4
SNP effect on Malignant melanoma of skin
SNP effect on Triacylglycerol (50:2) levels

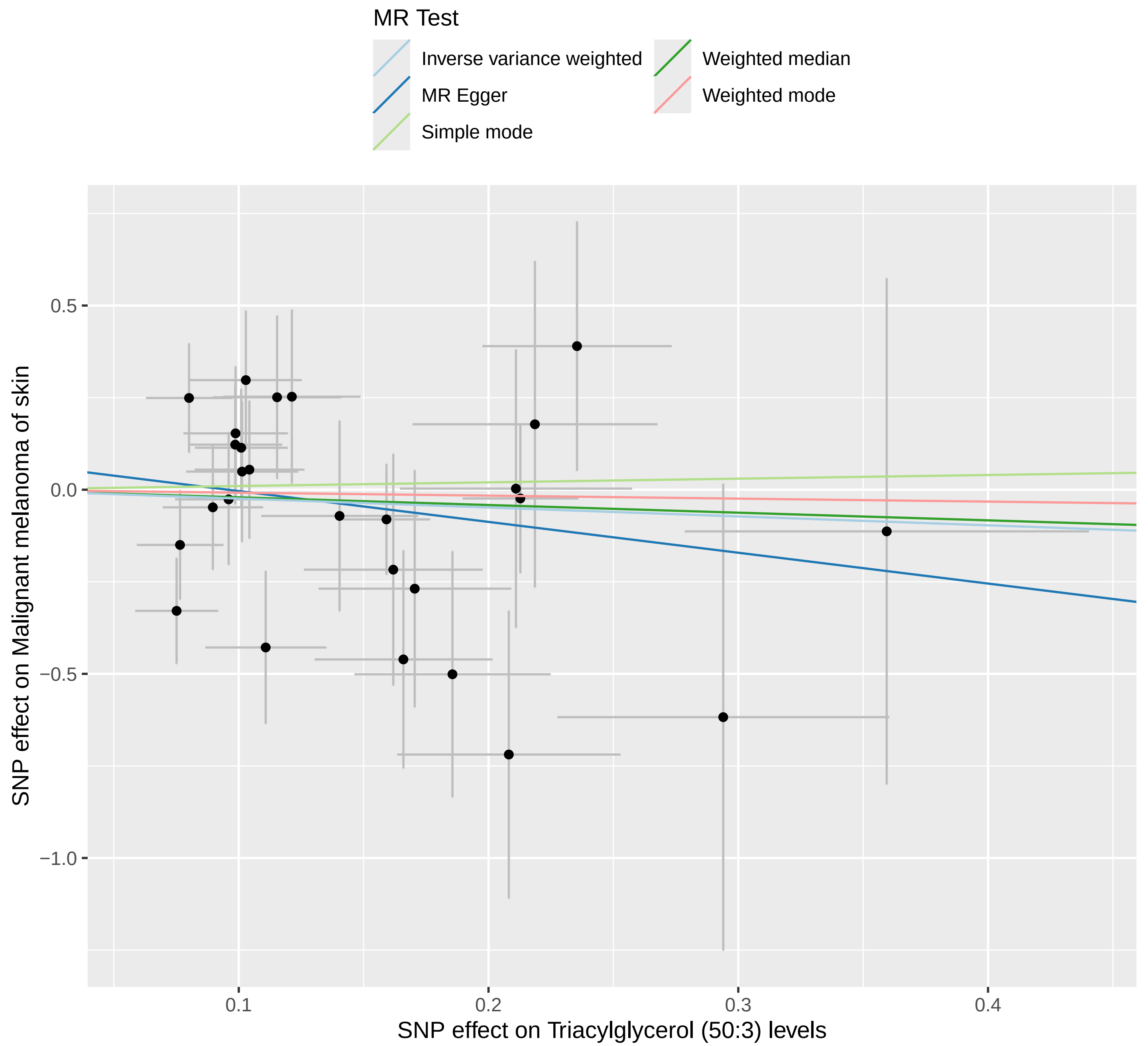
MR Test
Inverse variance weighted
MR Egger
Simple mode
Weighted median
Weighted mode
0.5
0.0
−0.5
−1.0
SNP effect on Malignant melanoma of skin
0.1
0.2
0.3
0.4
SNP effect on Triacylglycerol (50:3) levels

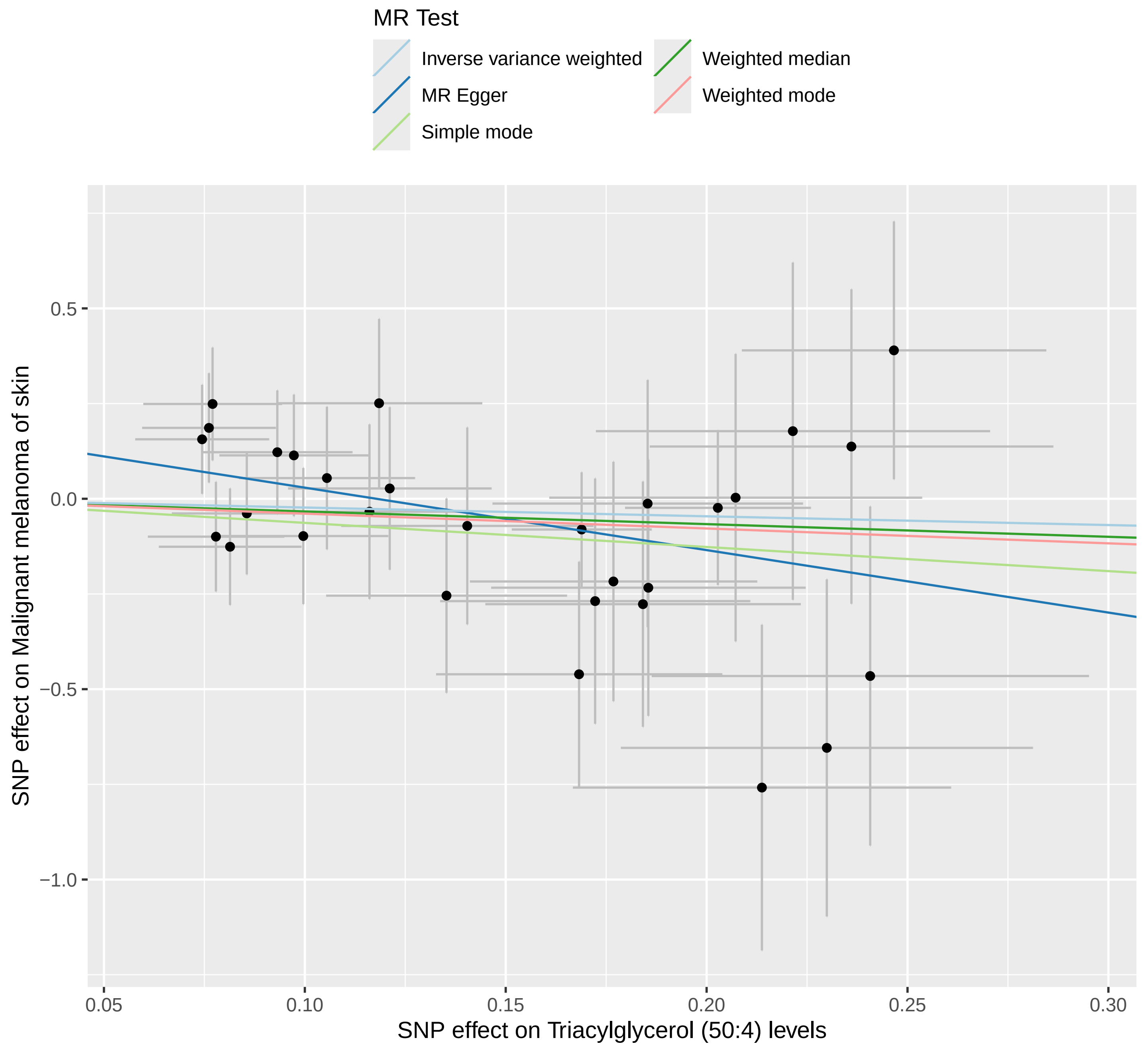

MR Test
Inverse variance weighted
MR Egger
Simple mode
Weighted median
Weighted mode
0.5
0.0
−0.5
−1.0
0.05
0.10
0.15
0.20
0.25
0.30
SNP effect on Malignant melanoma of skin
SNP effect on Triacylglycerol (50:4) levels

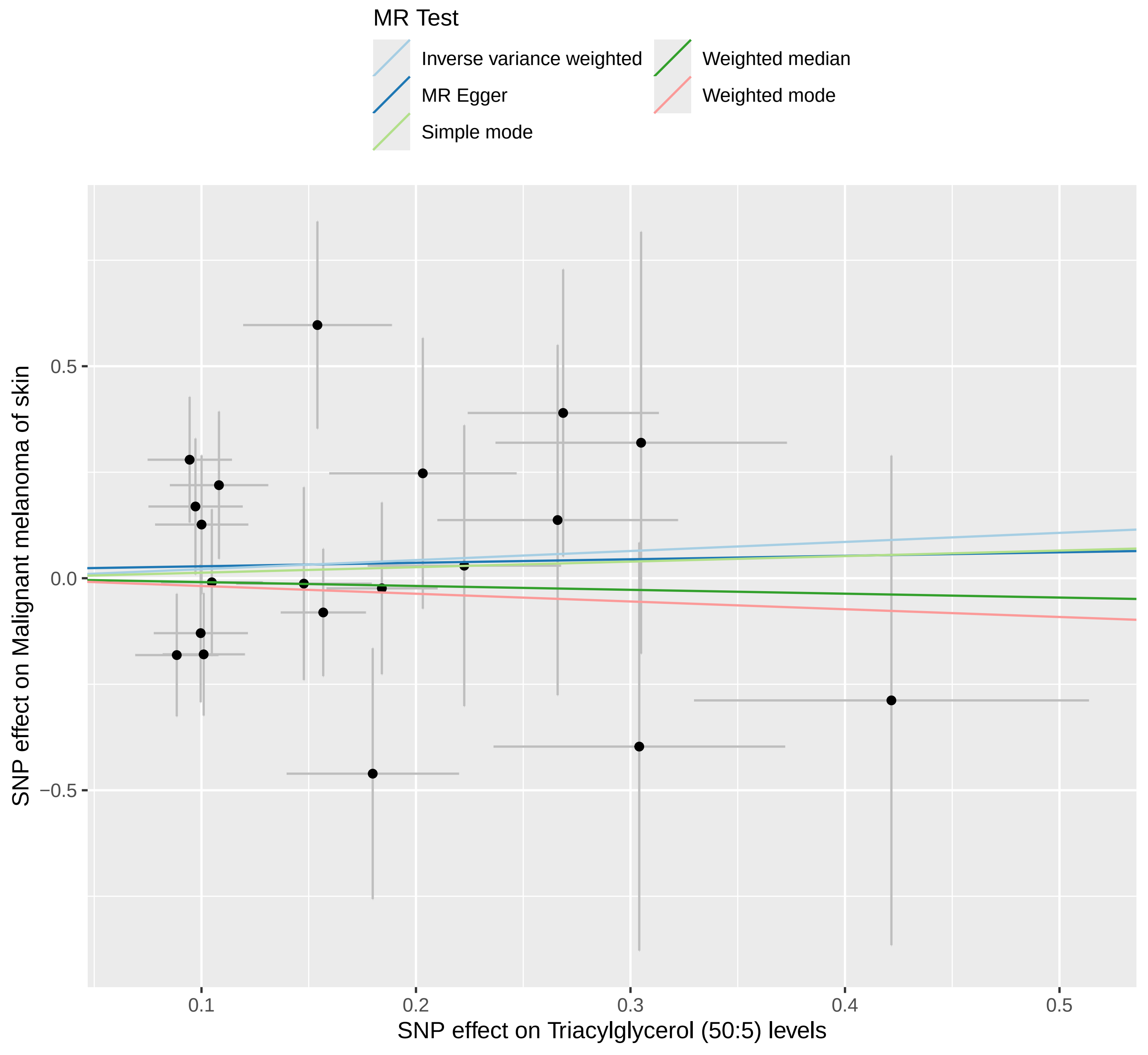
MR Test
Inverse variance weighted
MR Egger
Simple mode
Weighted median
Weighted mode
0.5
0.0
−0.5
0.1
0.2
0.3
0.4
0.5
SNP effect on Malignant melanoma of skin
SNP effect on Triacylglycerol (50:5) levels

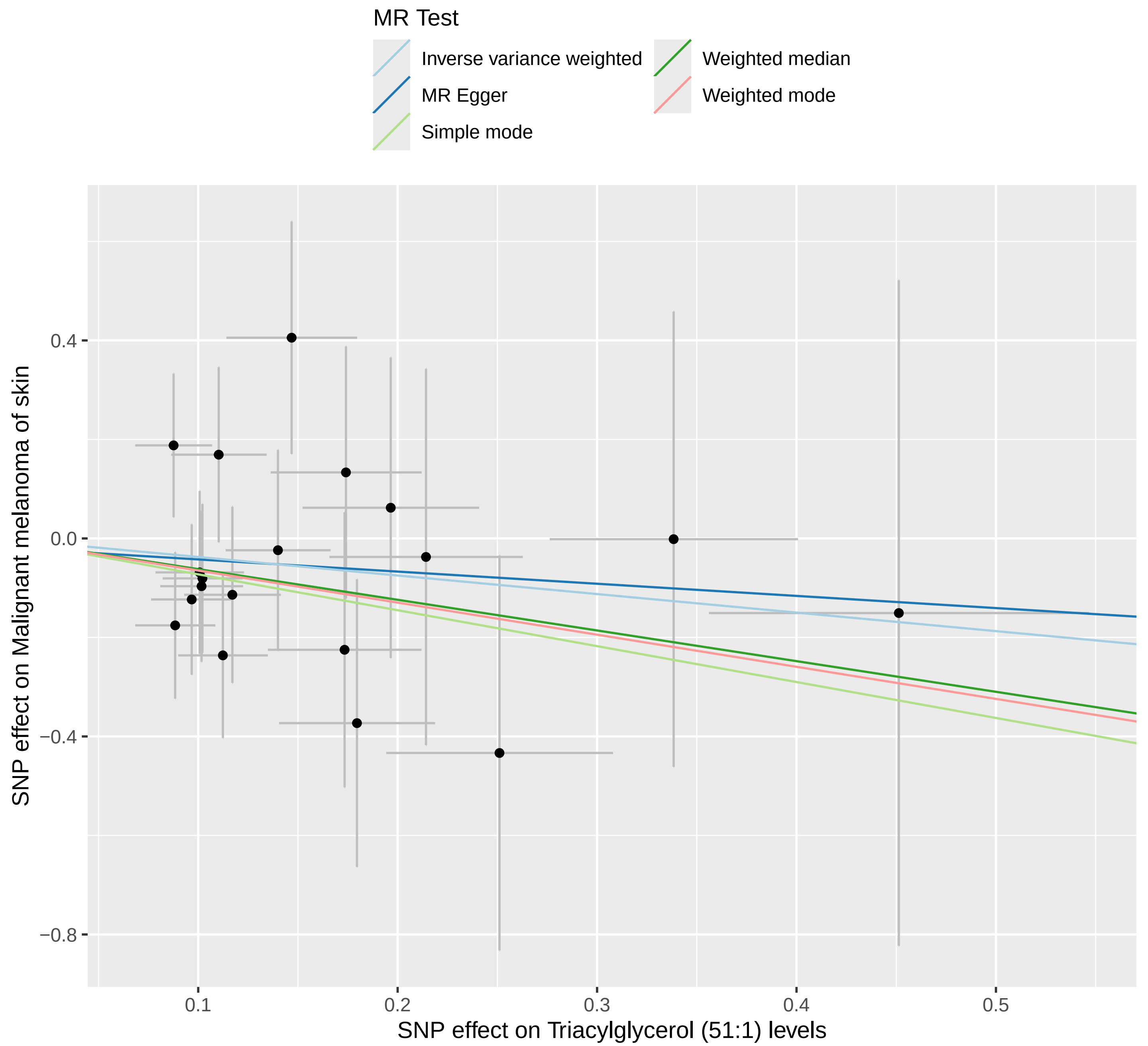
MR Test
Inverse variance weighted
MR Egger
Simple mode
Weighted median
Weighted mode
0.4
0.0
−0.4
−0.8
0.1
0.2
0.3
0.4
0.5
SNP effect on Malignant melanoma of skin
SNP effect on Triacylglycerol (51:1) levels

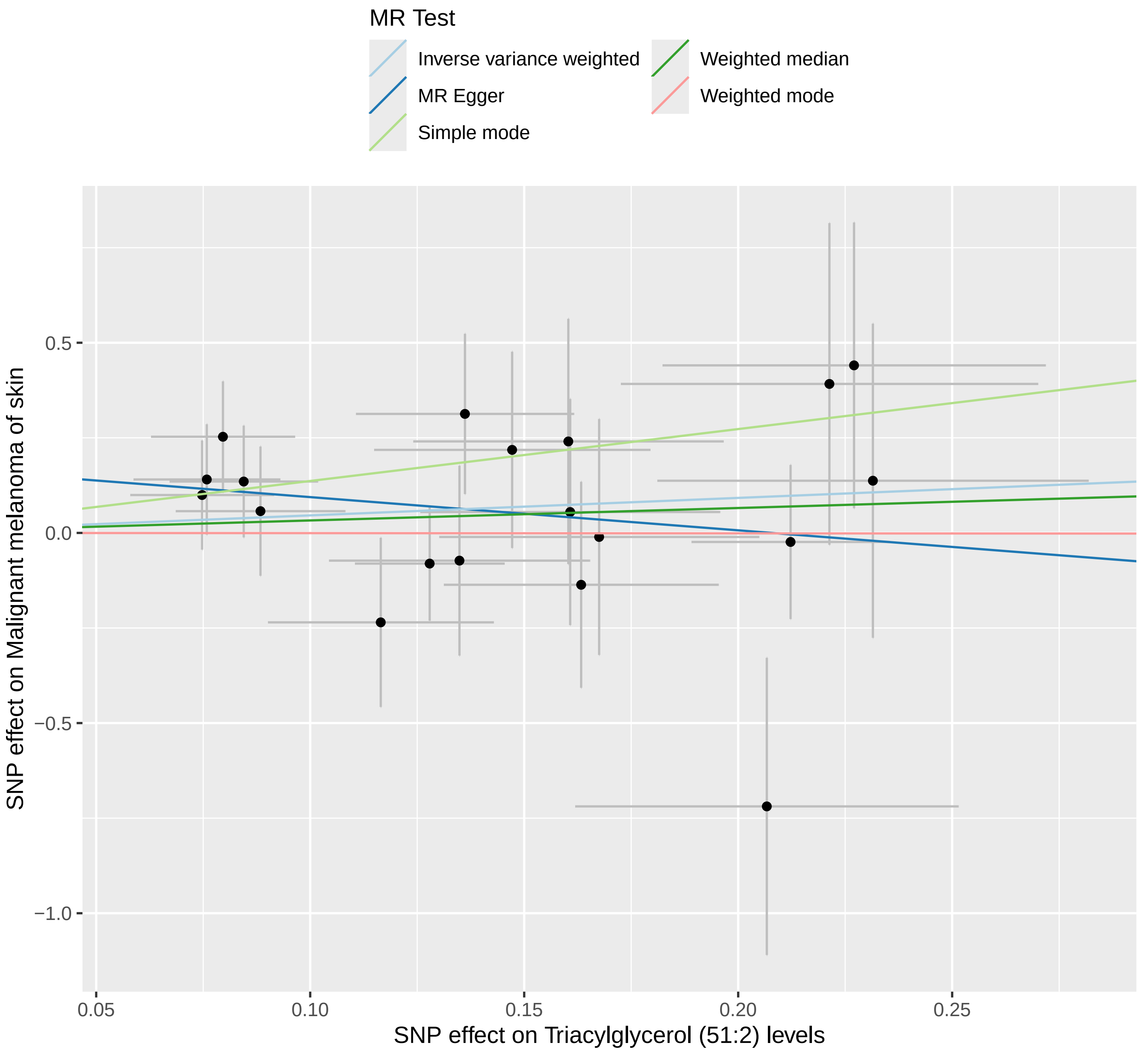
MR Test
Inverse variance weighted
MR Egger
Simple mode
Weighted median
Weighted mode
0.5
0.0
−0.5
−1.0
0.05
0.10
0.15
0.20
0.25
SNP effect on Triacylglycerol (51:2) levels
SNP effect on Malignant melanoma of skin

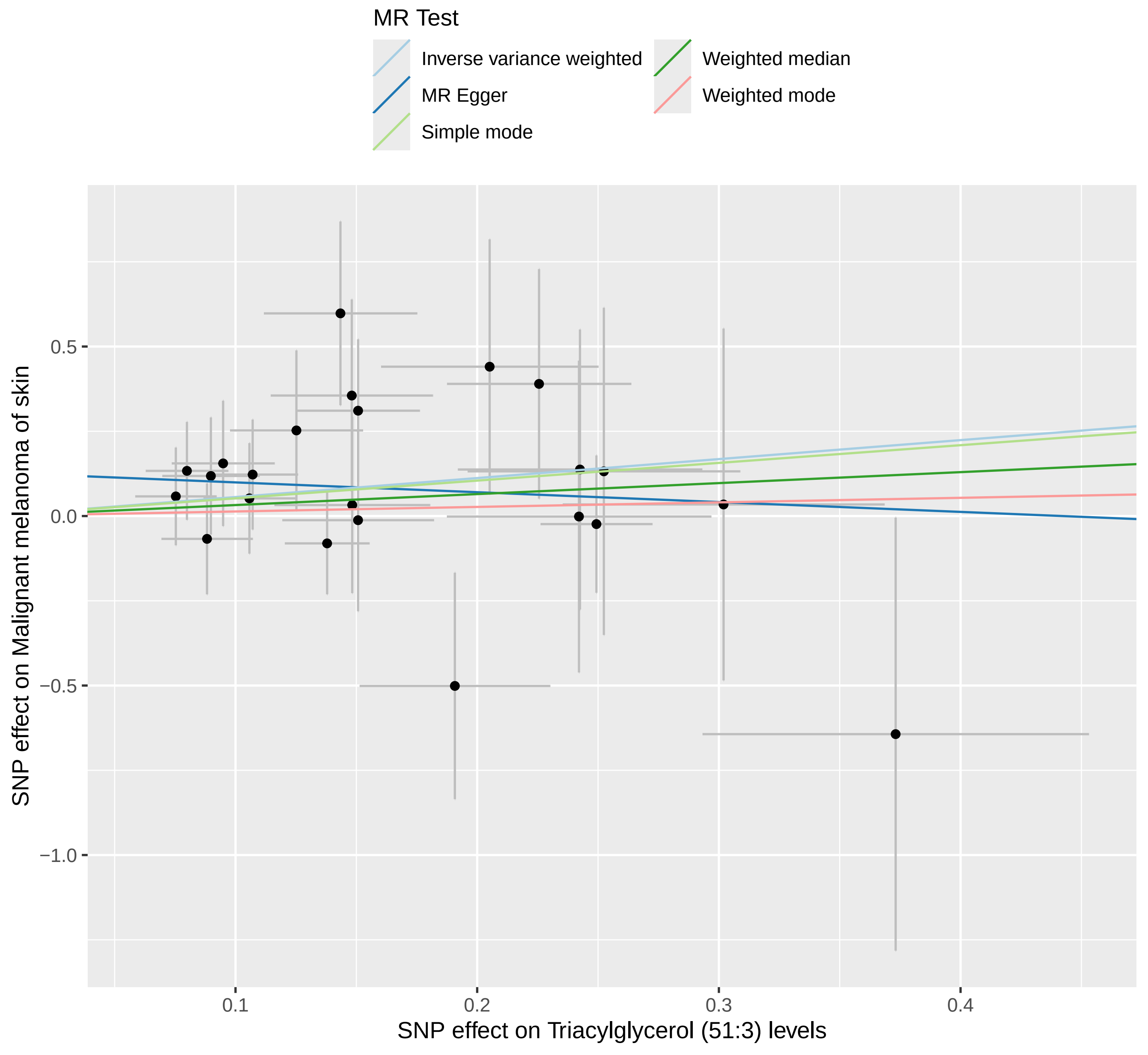

MR Test
Inverse variance weighted
MR Egger
Simple mode
Weighted median
Weighted mode
0.5
0.0
−0.5
−1.0
0.1
0.2
0.3
0.4
SNP effect on Malignant melanoma of skin
SNP effect on Triacylglycerol (51:3) levels

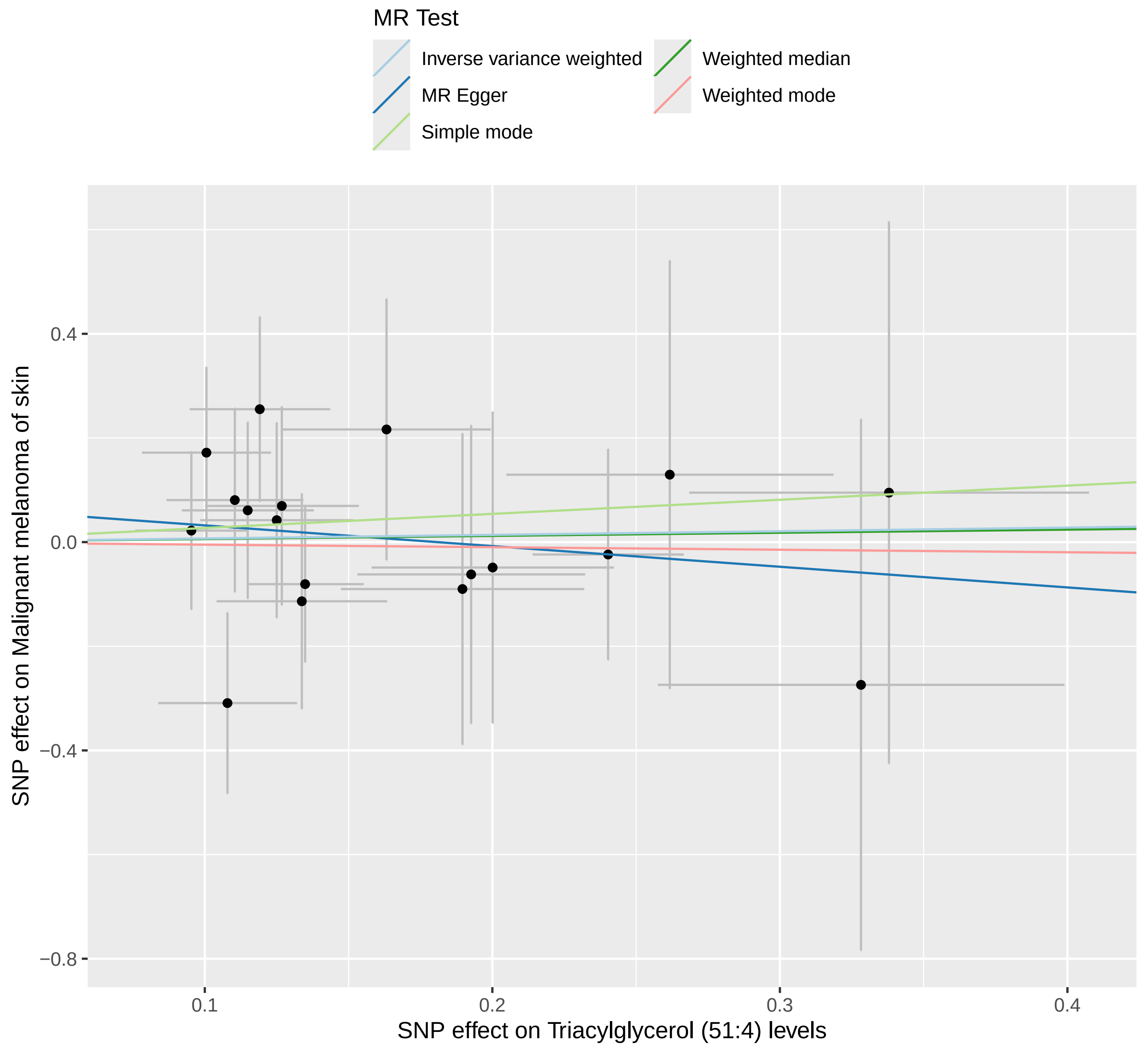

MR Test
Inverse variance weighted
MR Egger
Simple mode
Weighted median
Weighted mode
SNP effect on Malignant melanoma of skin
0.4
0.0
−0.4
−0.8
0.1
0.2
0.3
0.4
SNP effect on Triacylglycerol (51:4) levels

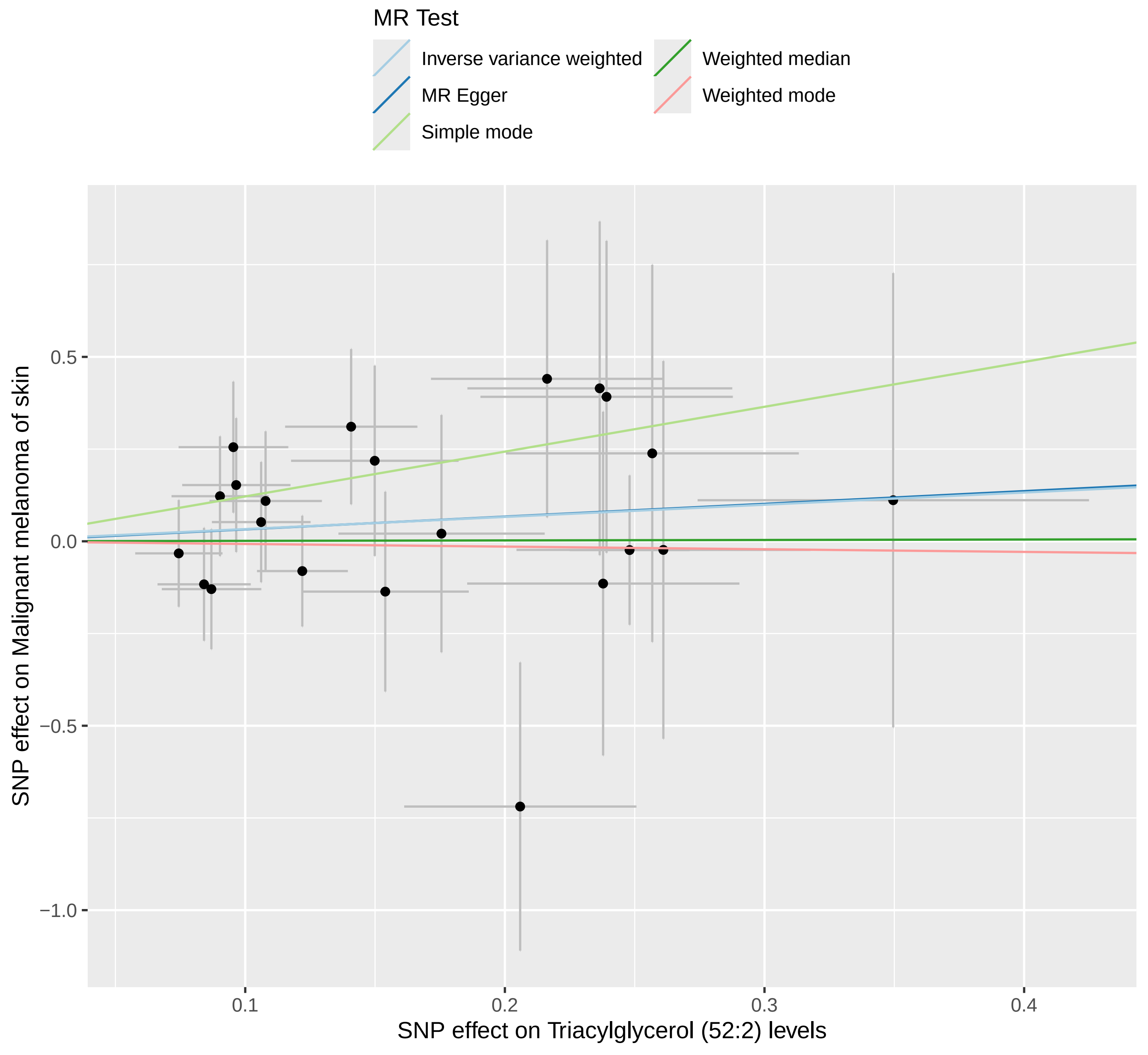
MR Test
Inverse variance weighted
MR Egger
Simple mode
Weighted median
Weighted mode
SNP effect on Malignant melanoma of skin
0.5
0.0
−0.5
−1.0
0.1
0.2
0.3
0.4
SNP effect on Triacylglycerol (52:2) levels

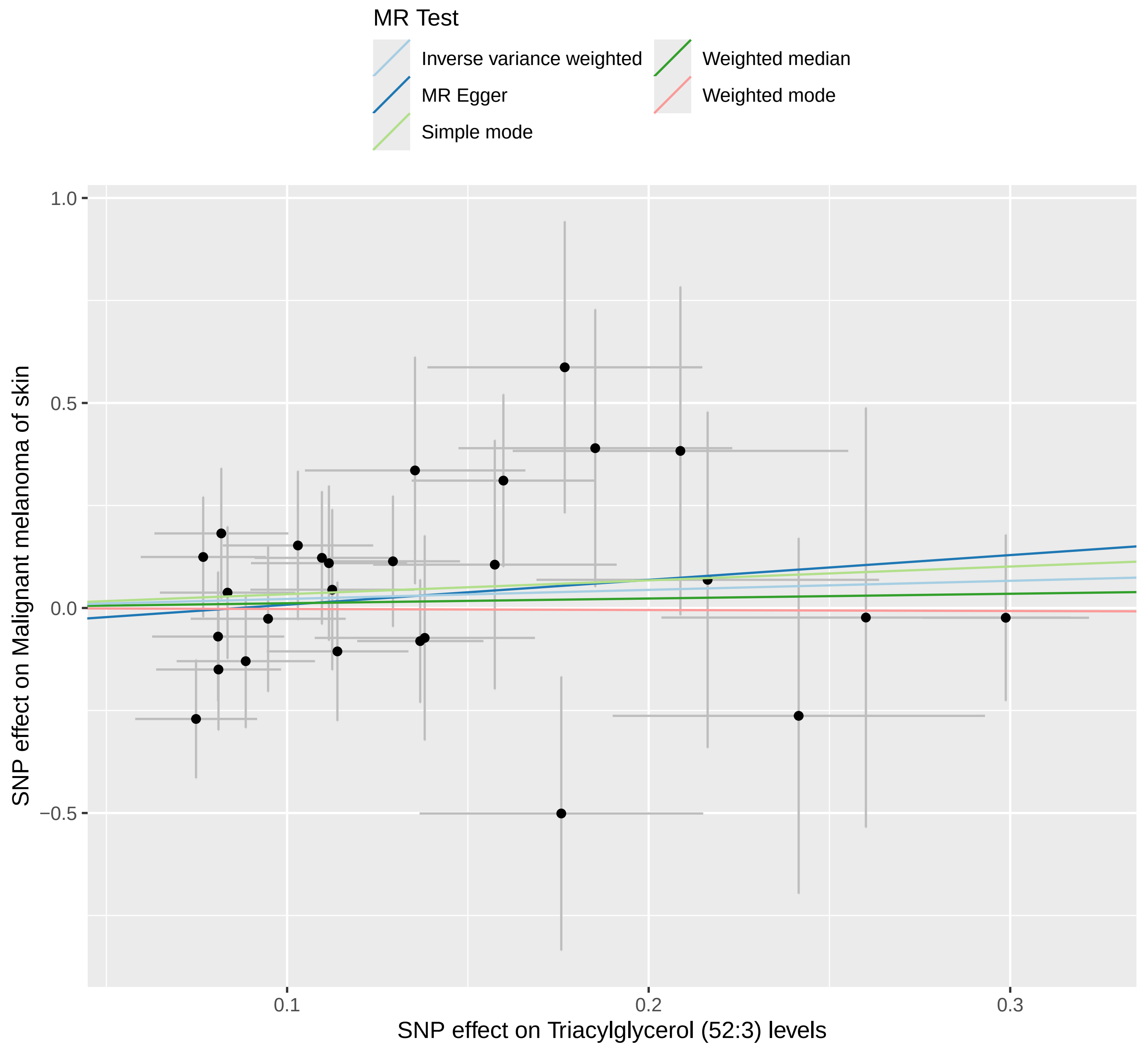
MR Test
Inverse variance weighted
MR Egger
Simple mode
Weighted median
Weighted mode
1.0
0.5
0.0
−0.5
0.1
0.2
0.3
SNP effect on Malignant melanoma of skin
SNP effect on Triacylglycerol (52:3) levels

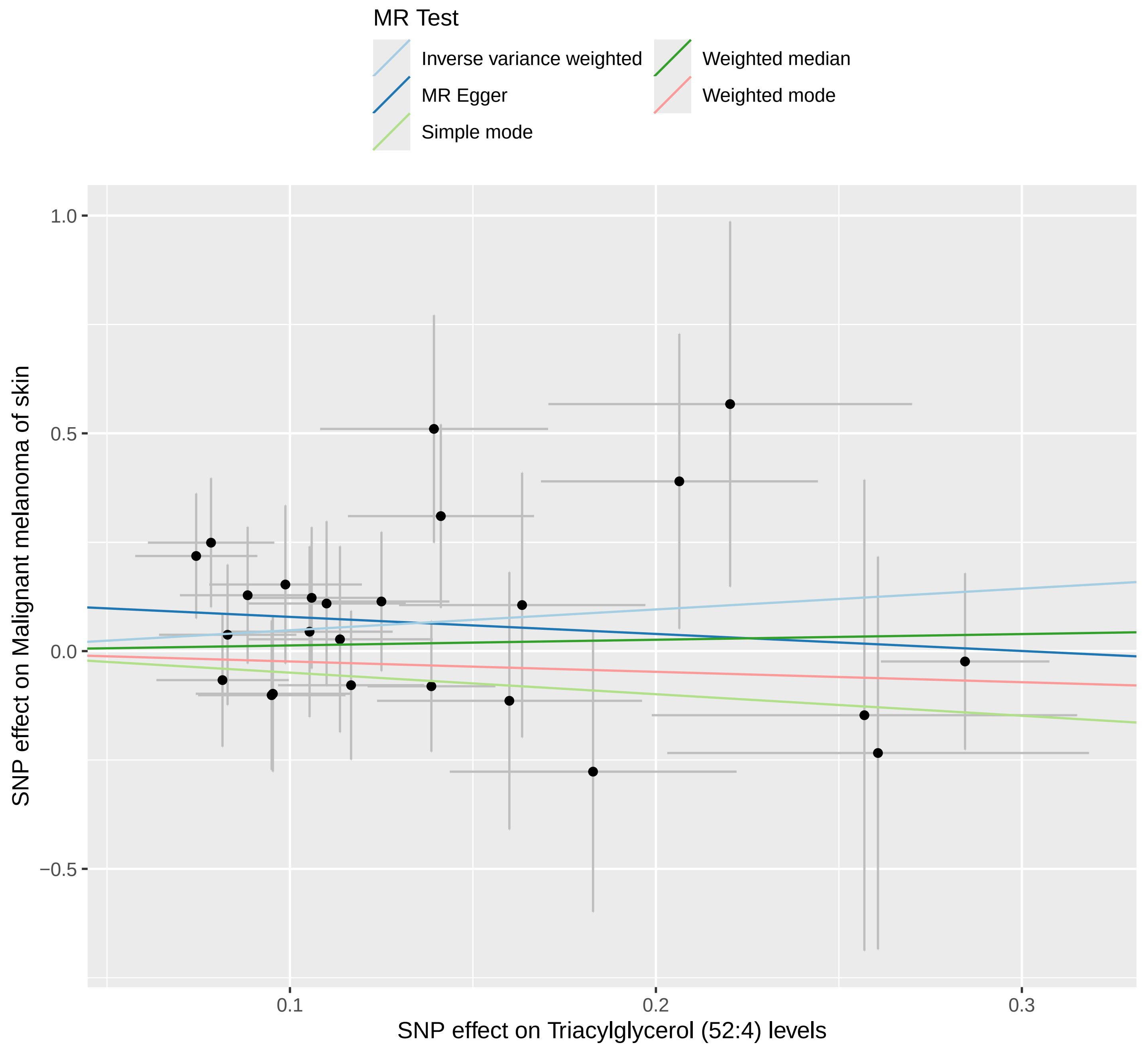
MR Test
Inverse variance weighted
MR Egger
Simple mode
Weighted median
Weighted mode
1.0
0.5
0.0
−0.5
0.1
0.2
0.3
SNP effect on Malignant melanoma of skin
SNP effect on Triacylglycerol (52:4) levels

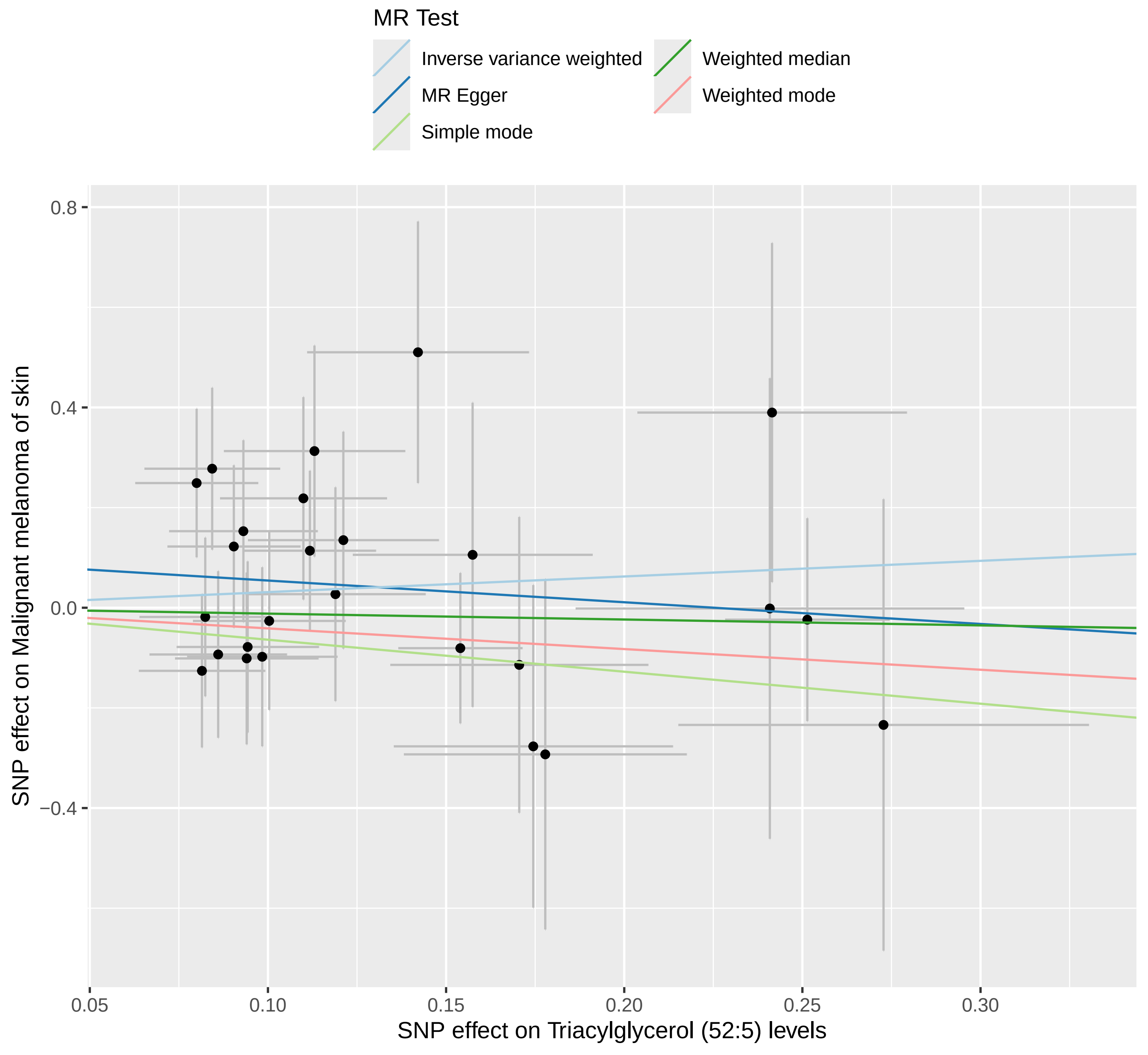

MR Test
Inverse variance weighted
MR Egger
Simple mode
Weighted median
Weighted mode
0.8
0.4
0.0
−0.4
0.05
0.10
0.15
0.20
0.25
0.30
SNP effect on Triacylglycerol (52:5) levels
SNP effect on Malignant melanoma of skin

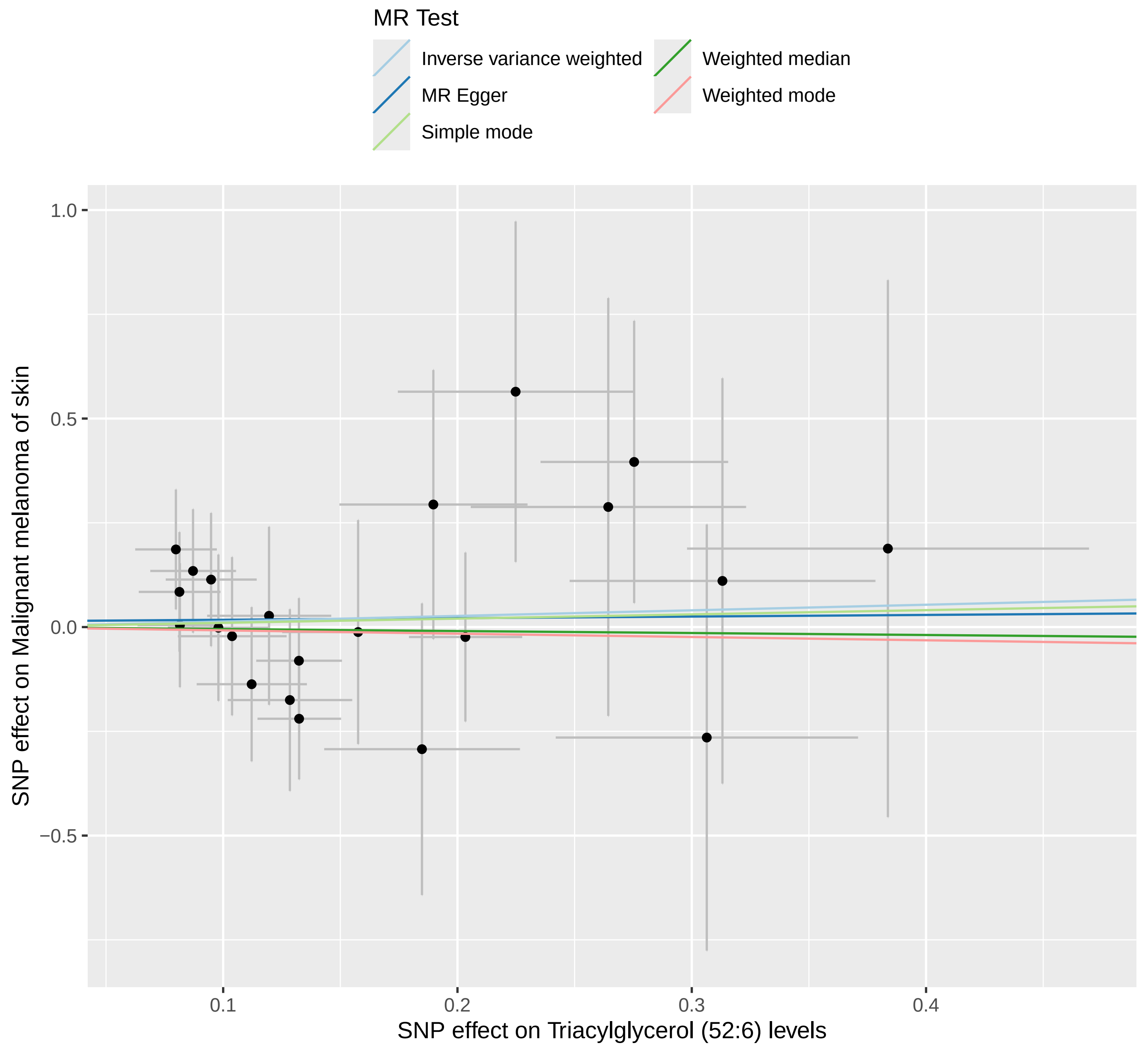

MR Test
Inverse variance weighted
MR Egger
Simple mode
Weighted median
Weighted mode
1.0
0.5
0.0
−0.5
SNP effect on Malignant melanoma of skin
0.1
0.2
0.3
0.4
SNP effect on Triacylglycerol (52:6) levels

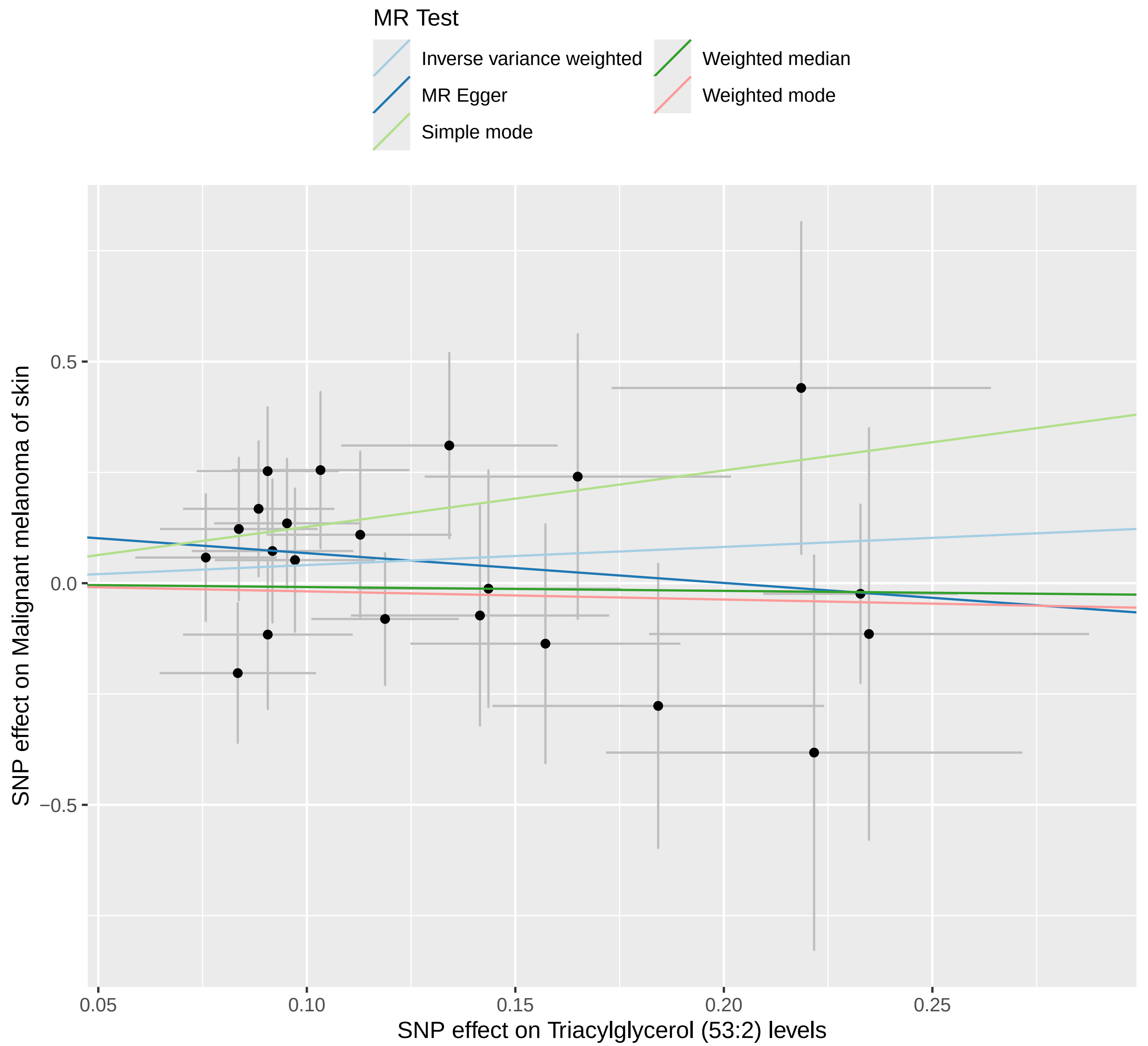
MR Test
Inverse variance weighted
MR Egger
Simple mode
Weighted median
Weighted mode
SNP effect on Malignant melanoma of skin
0.5
0.0
−0.5
0.05
0.10
0.15
0.20
0.25
SNP effect on Triacylglycerol (53:2) levels

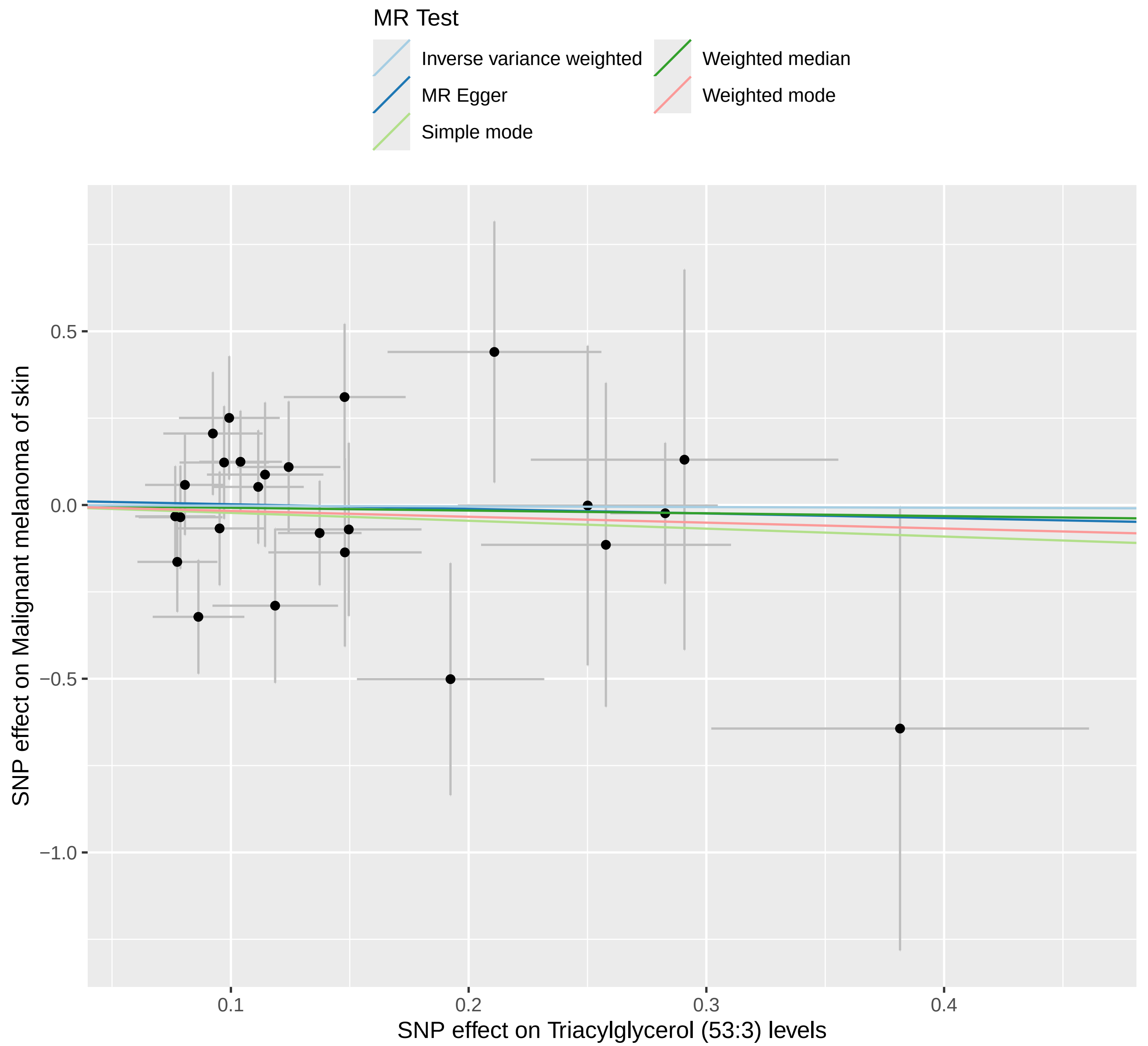

MR Test
Inverse variance weighted
MR Egger
Simple mode
Weighted median
Weighted mode
SNP effect on Malignant melanoma of skin
0.5
0.0
−0.5
−1.0
0.1
0.2
0.3
0.4
SNP effect on Triacylglycerol (53:3) levels

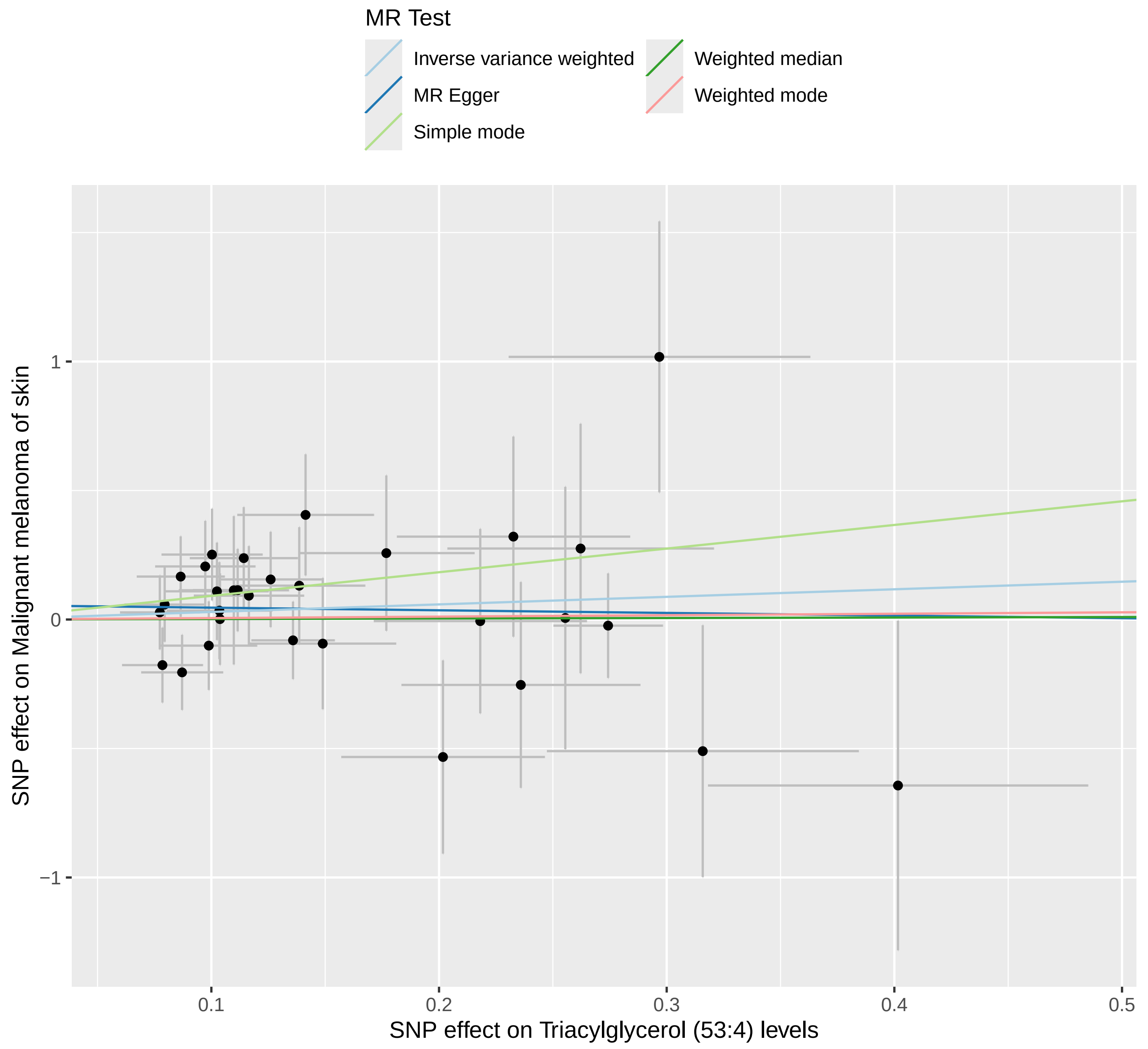

MR Test
Inverse variance weighted
MR Egger
Simple mode
Weighted median
Weighted mode
SNP effect on Malignant melanoma of skin
1
0
−1
0.1
0.2
0.3
0.4
0.5
SNP effect on Triacylglycerol (53:4) levels

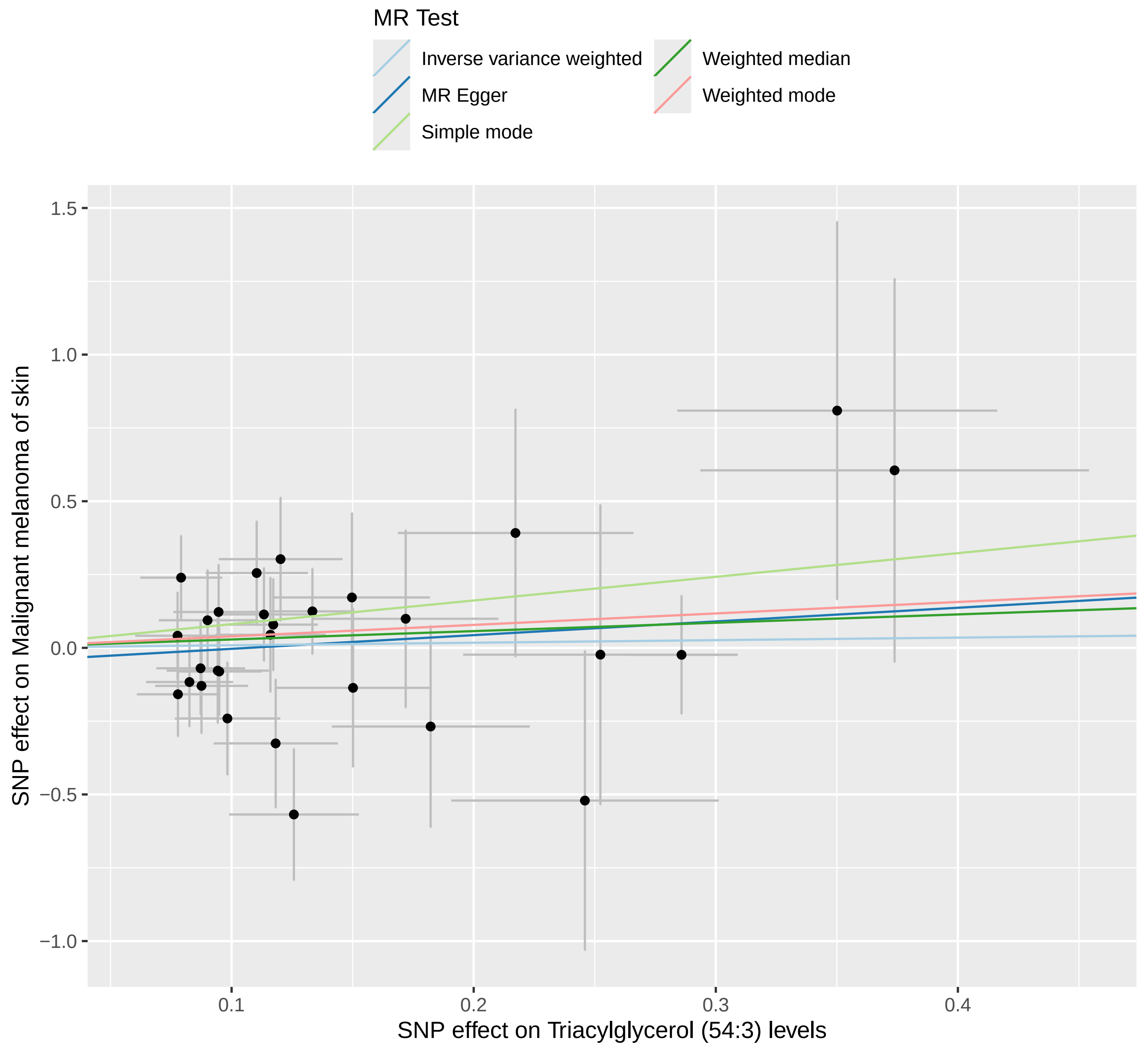
MR Test
Inverse variance weighted
MR Egger
Simple mode
Weighted median
Weighted mode
1.5
1.0
0.5
0.0
−0.5
−1.0
0.1
0.2
0.3
0.4
SNP effect on Malignant melanoma of skin
SNP effect on Triacylglycerol (54:3) levels

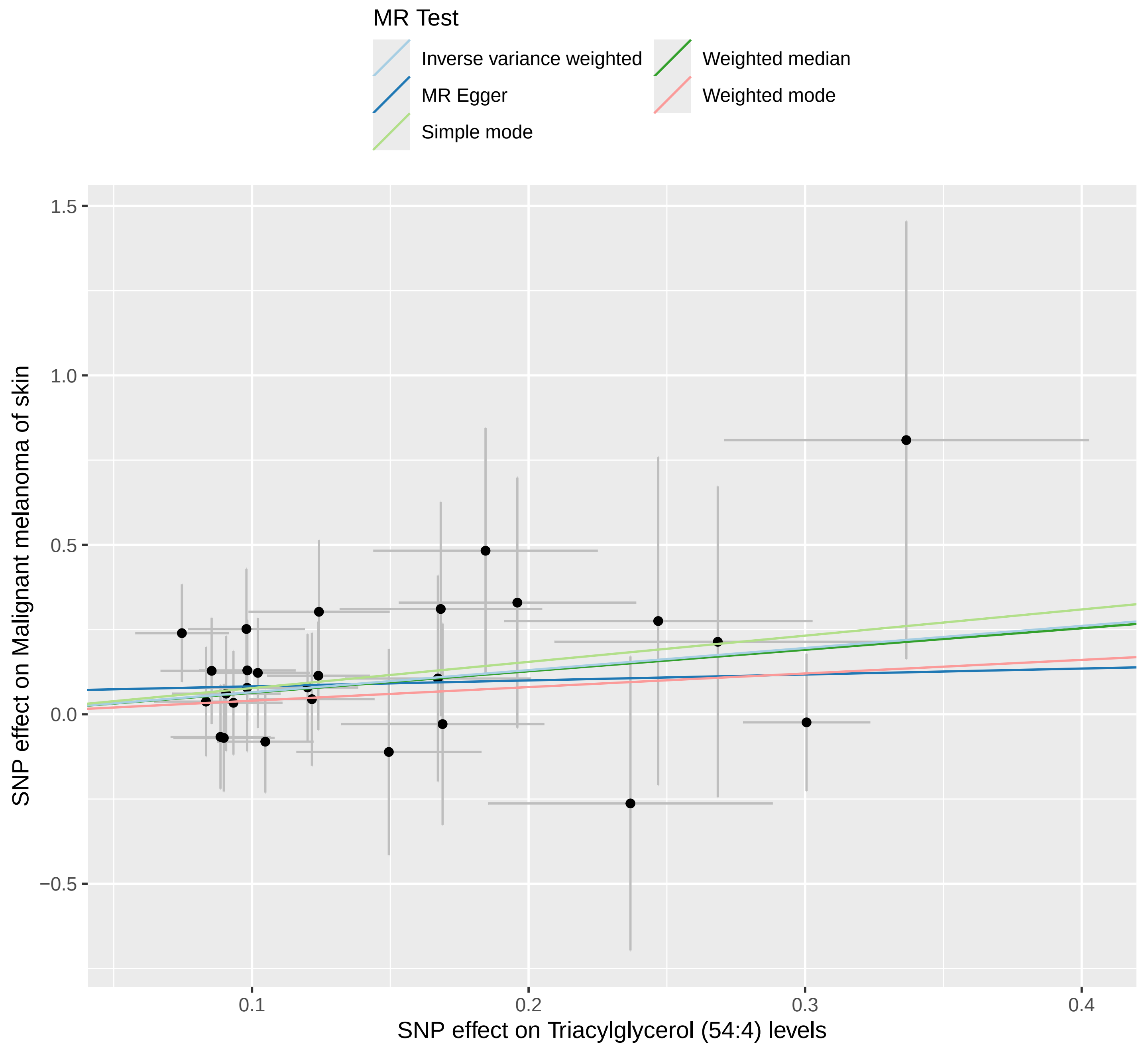
MR Test
Inverse variance weighted
MR Egger
Simple mode
Weighted median
Weighted mode
1.5
1.0
0.5
0.0
−0.5
0.1
0.2
0.3
0.4
SNP effect on Malignant melanoma of skin
SNP effect on Triacylglycerol (54:4) levels

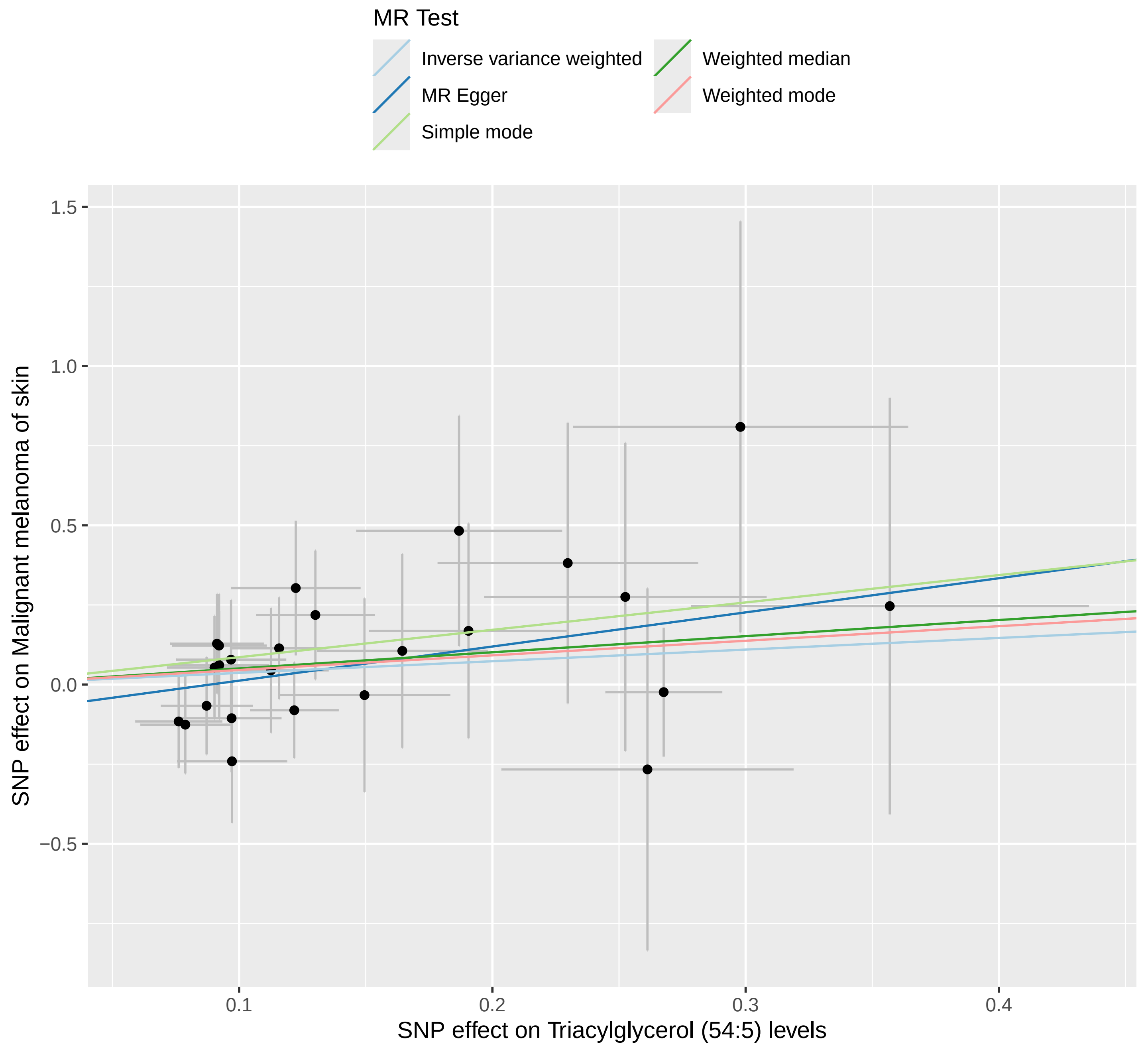

MR Test
Inverse variance weighted
MR Egger
Simple mode
Weighted median
Weighted mode
1.5
1.0
0.5
0.0
−0.5
0.1
0.2
0.3
0.4
SNP effect on Malignant melanoma of skin
SNP effect on Triacylglycerol (54:5) levels

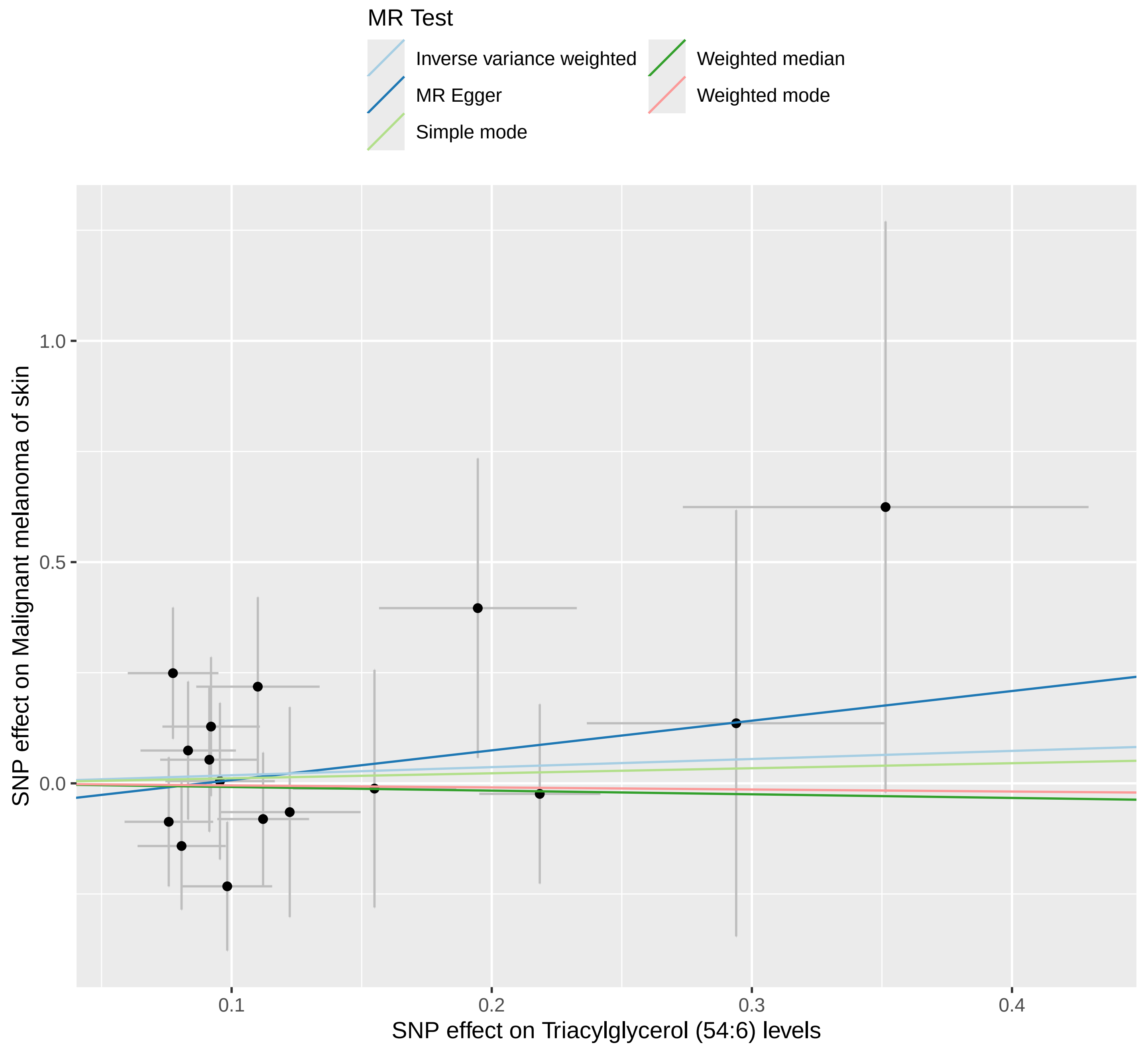

MR Test
Inverse variance weighted
MR Egger
Simple mode
Weighted median
Weighted mode
1.0
0.5
0.0
0.1
0.2
0.3
0.4
SNP effect on Malignant melanoma of skin
SNP effect on Triacylglycerol (54:6) levels

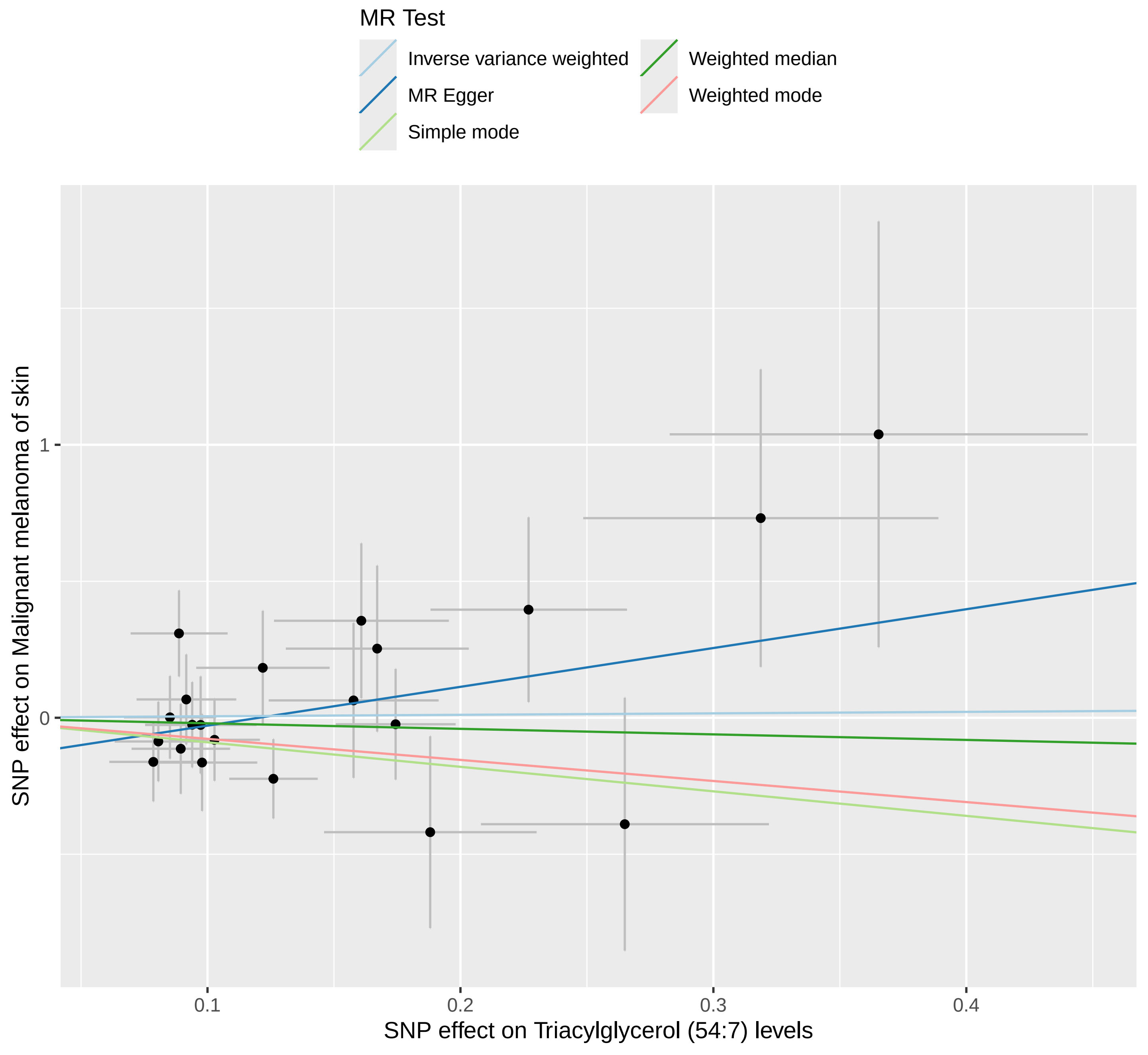
MR Test
Inverse variance weighted
MR Egger
Simple mode
Weighted median
Weighted mode
SNP effect on Malignant melanoma of skin
1
0
0.1
0.2
0.3
0.4
SNP effect on Triacylglycerol (54:7) levels

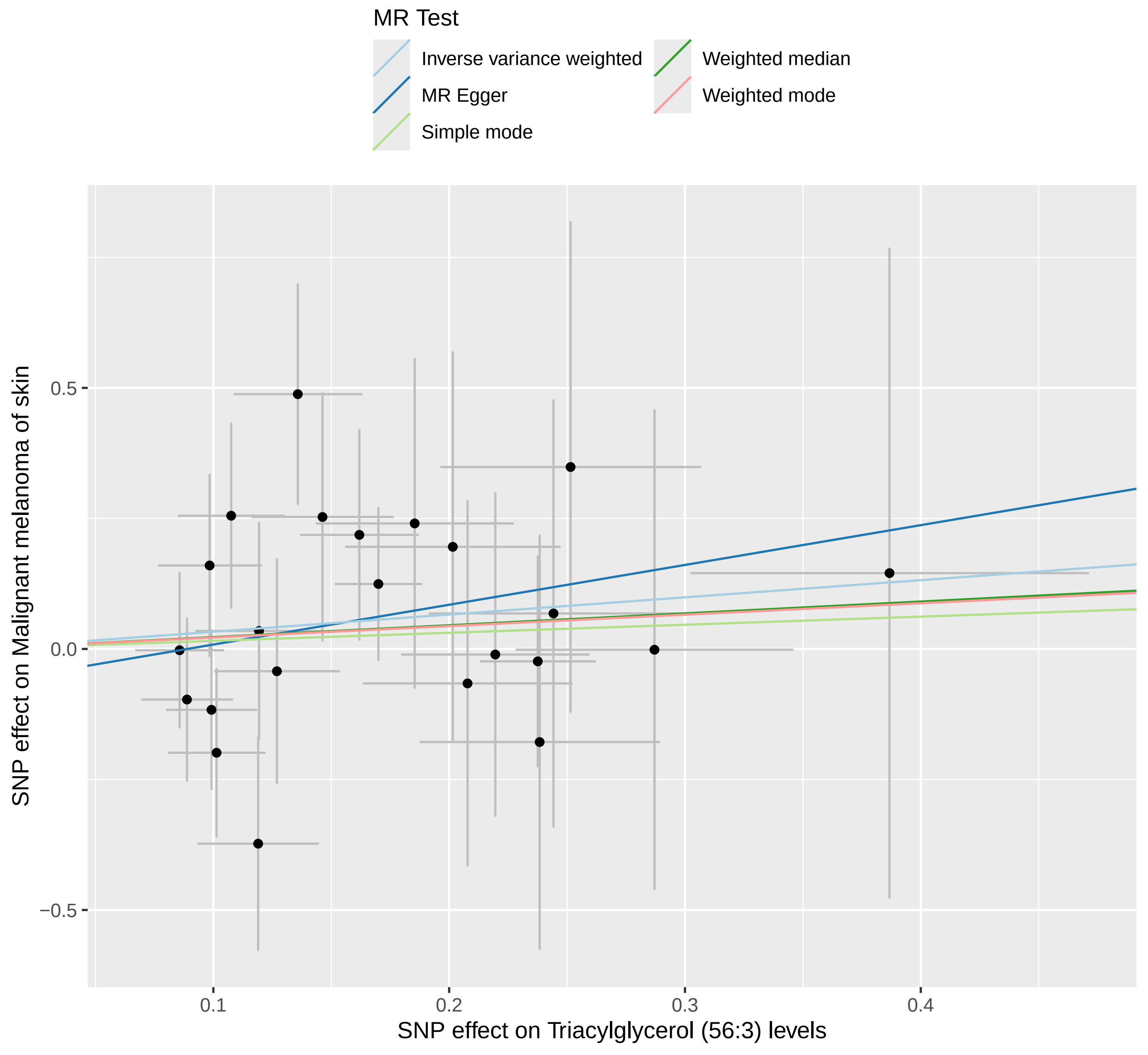

MR Test
Inverse variance weighted
MR Egger
Simple mode
Weighted median
Weighted mode
SNP effect on Malignant melanoma of skin
0.5
0.0
−0.5
0.1
0.2
0.3
0.4
SNP effect on Triacylglycerol (56:3) levels

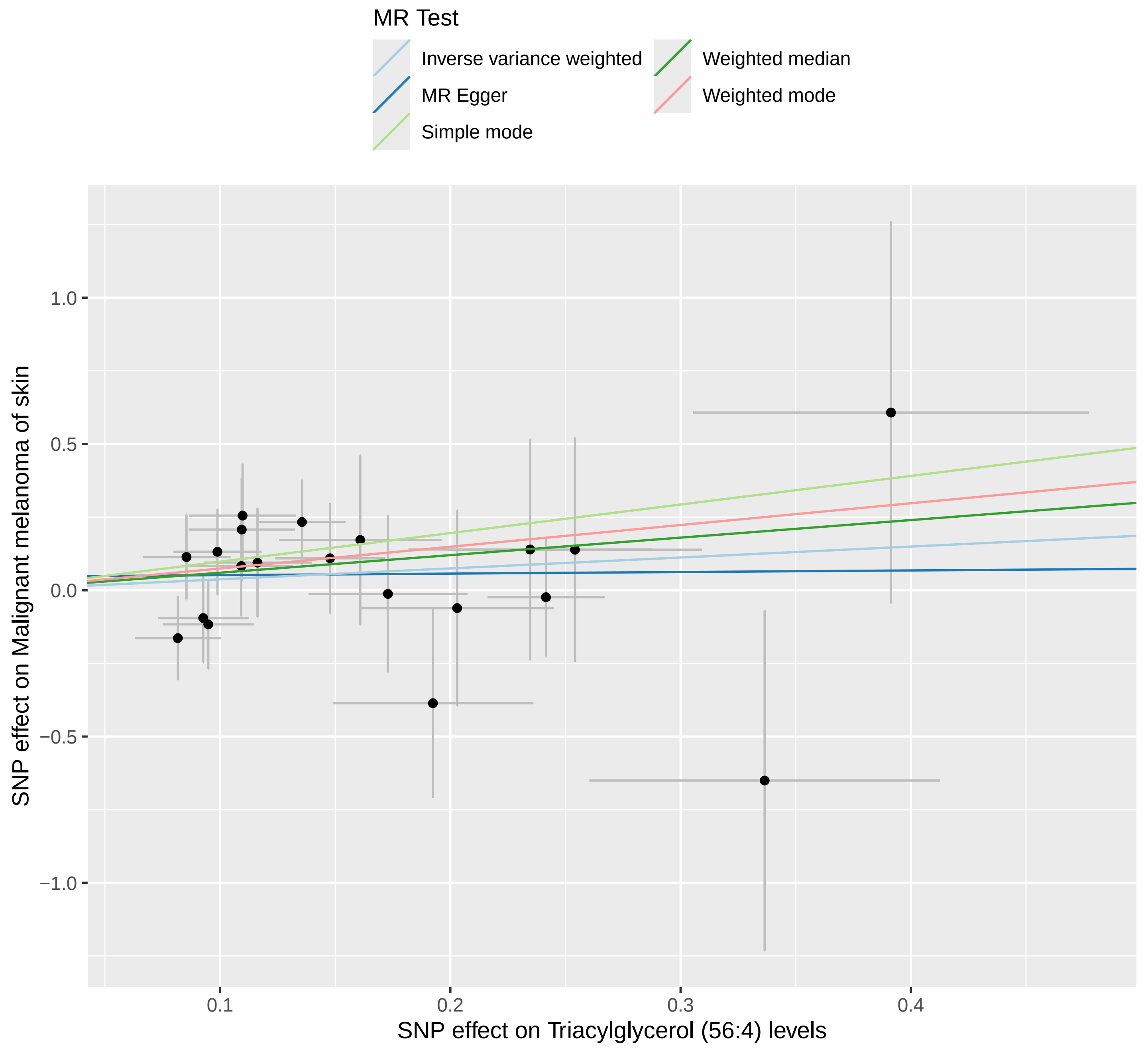
MR Test
Inverse variance weighted
MR Egger
Simple mode
Weighted median
Weighted mode
1.0
0.5
0.0
−0.5
−1.0
0.1
0.2
0.3
0.4
SNP effect on Malignant melanoma of skin
SNP effect on Triacylglycerol (56:4) levels

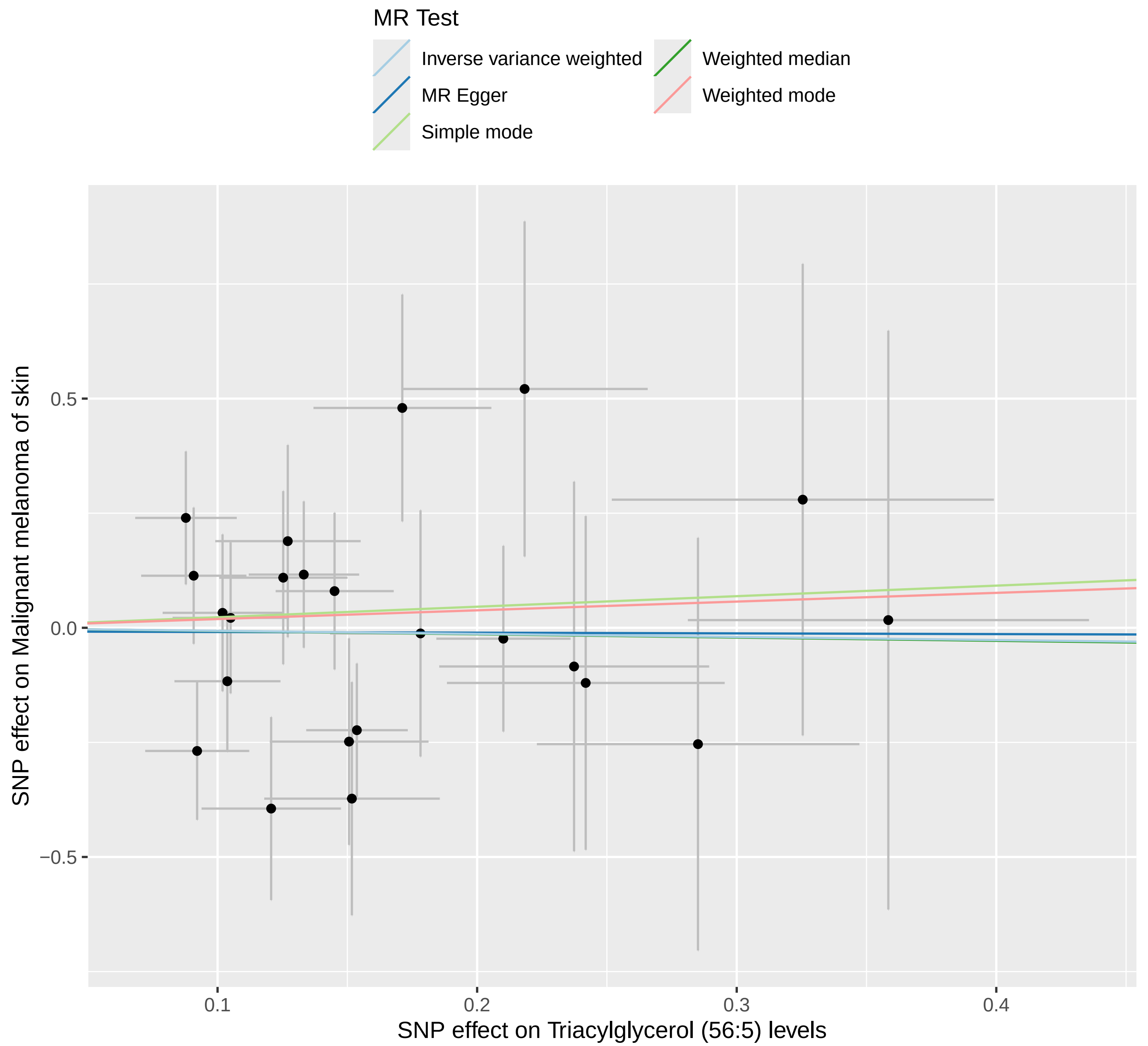

MR Test
Inverse variance weighted
MR Egger
Simple mode
Weighted median
Weighted mode
SNP effect on Malignant melanoma of skin
0.5
0.0
−0.5
0.1
0.2
0.3
0.4
SNP effect on Triacylglycerol (56:5) levels

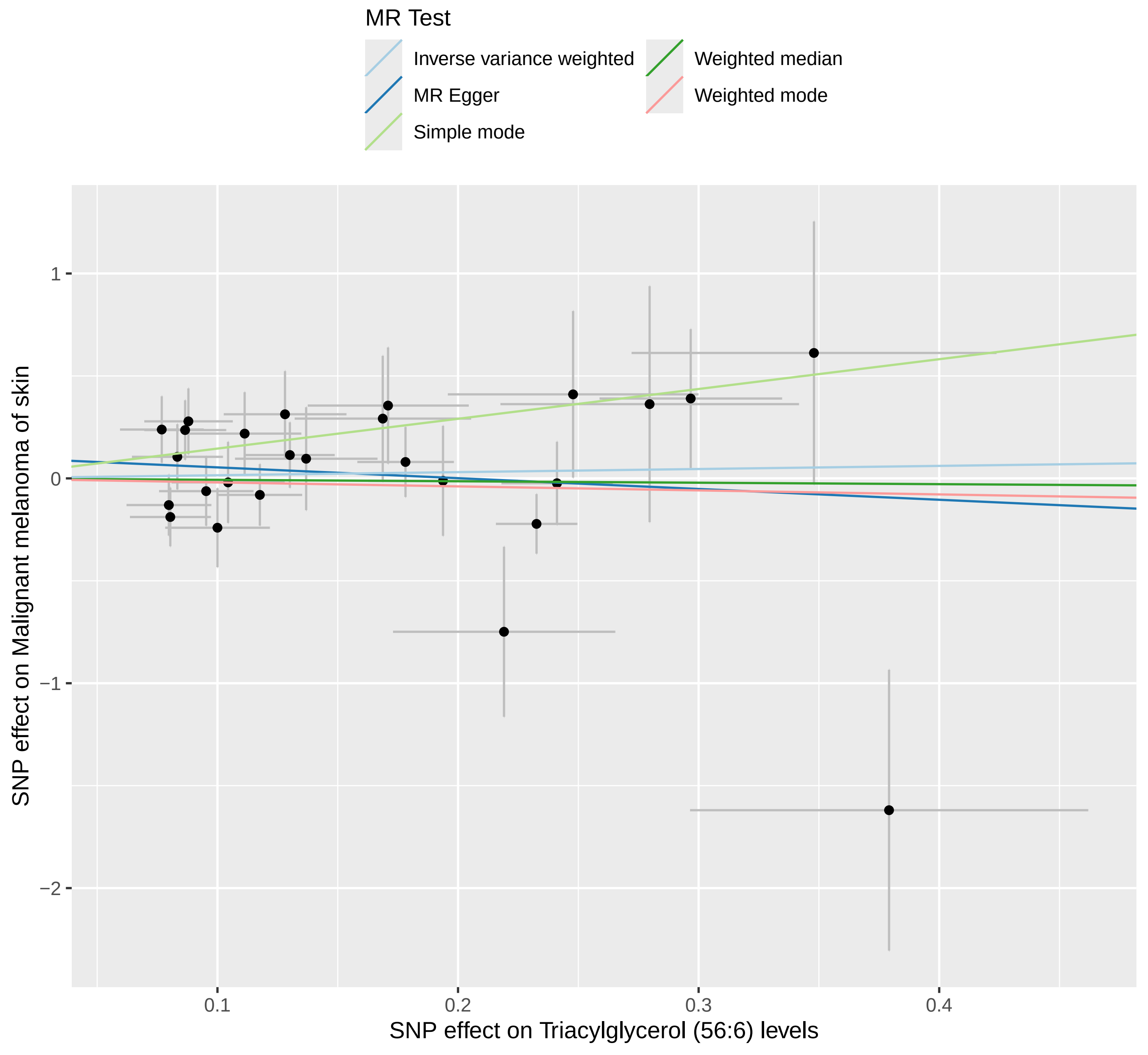
MR Test
Inverse variance weighted
MR Egger
Simple mode
Weighted median
Weighted mode
1
0
−1
−2
0.1
0.2
0.3
0.4
SNP effect on Malignant melanoma of skin
SNP effect on Triacylglycerol (56:6) levels

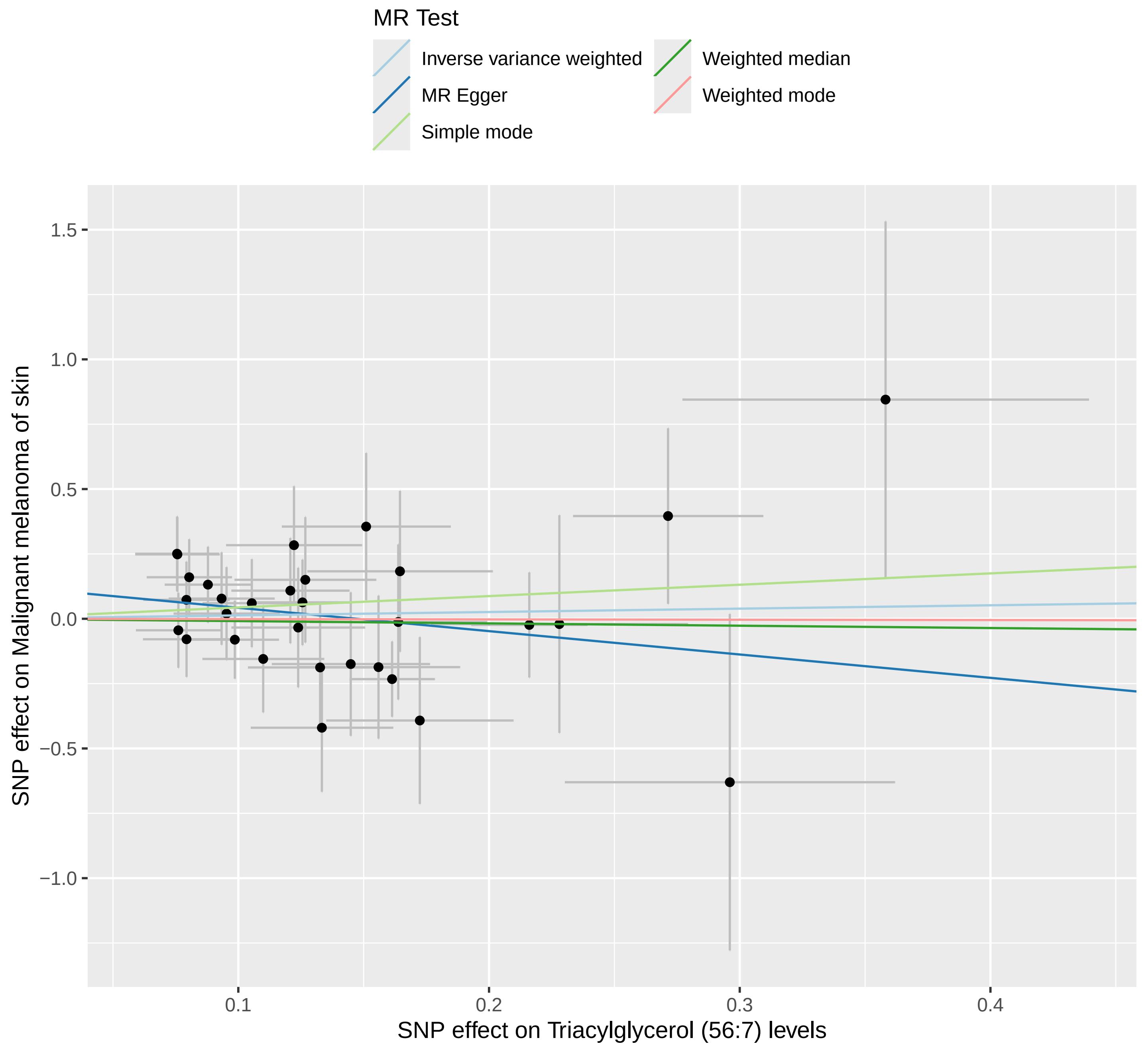
MR Test
Inverse variance weighted
MR Egger
Simple mode
Weighted median
Weighted mode
1.5
1.0
0.5
0.0
−0.5
−1.0
SNP effect on Malignant melanoma of skin
0.1
0.2
0.3
0.4
SNP effect on Triacylglycerol (56:7) levels

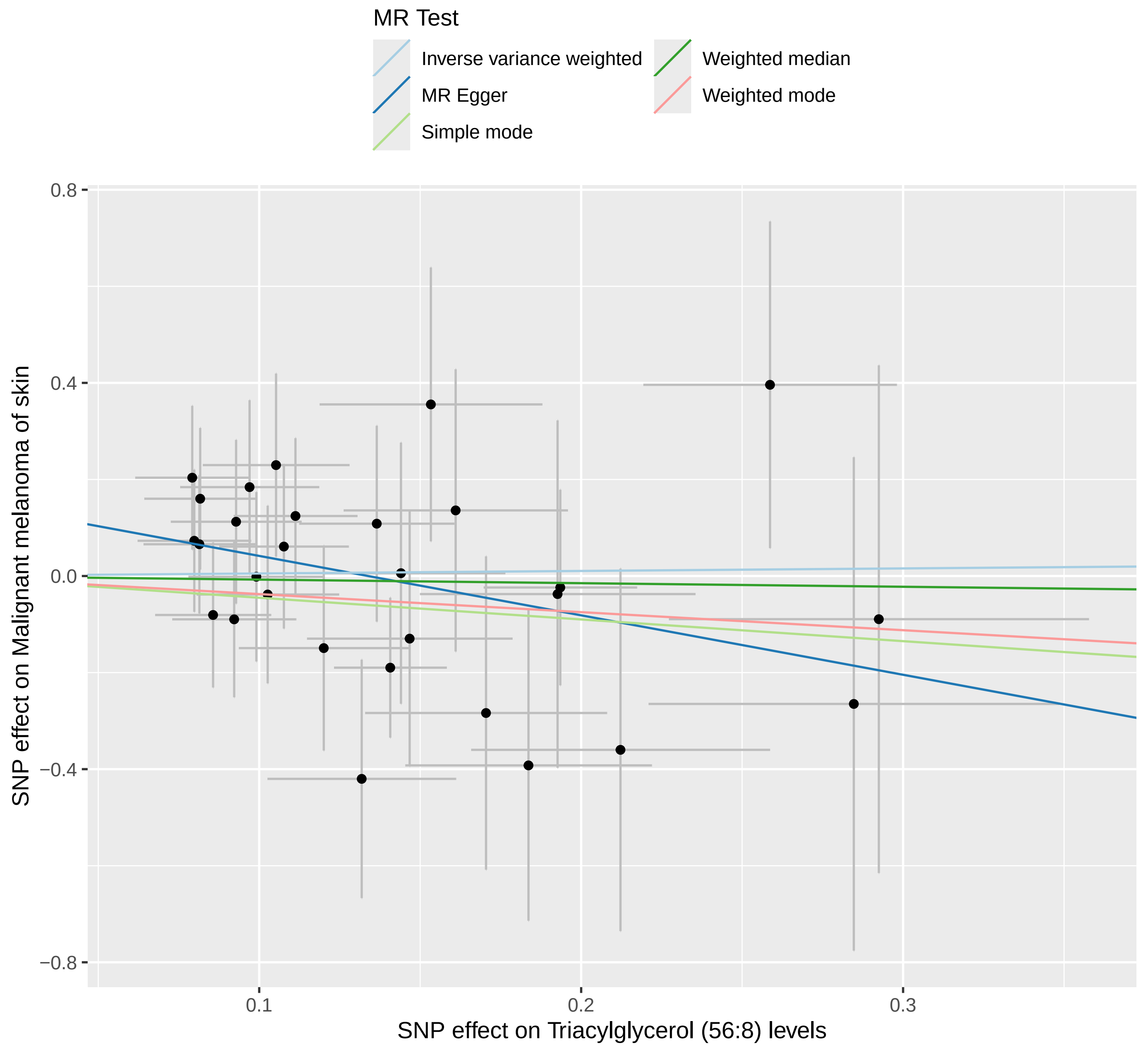

MR Test
Inverse variance weighted
MR Egger
Simple mode
Weighted median
Weighted mode
0.8
0.4
0.0
−0.4
−0.8
SNP effect on Malignant melanoma of skin
0.1
0.2
0.3
SNP effect on Triacylglycerol (56:8) levels

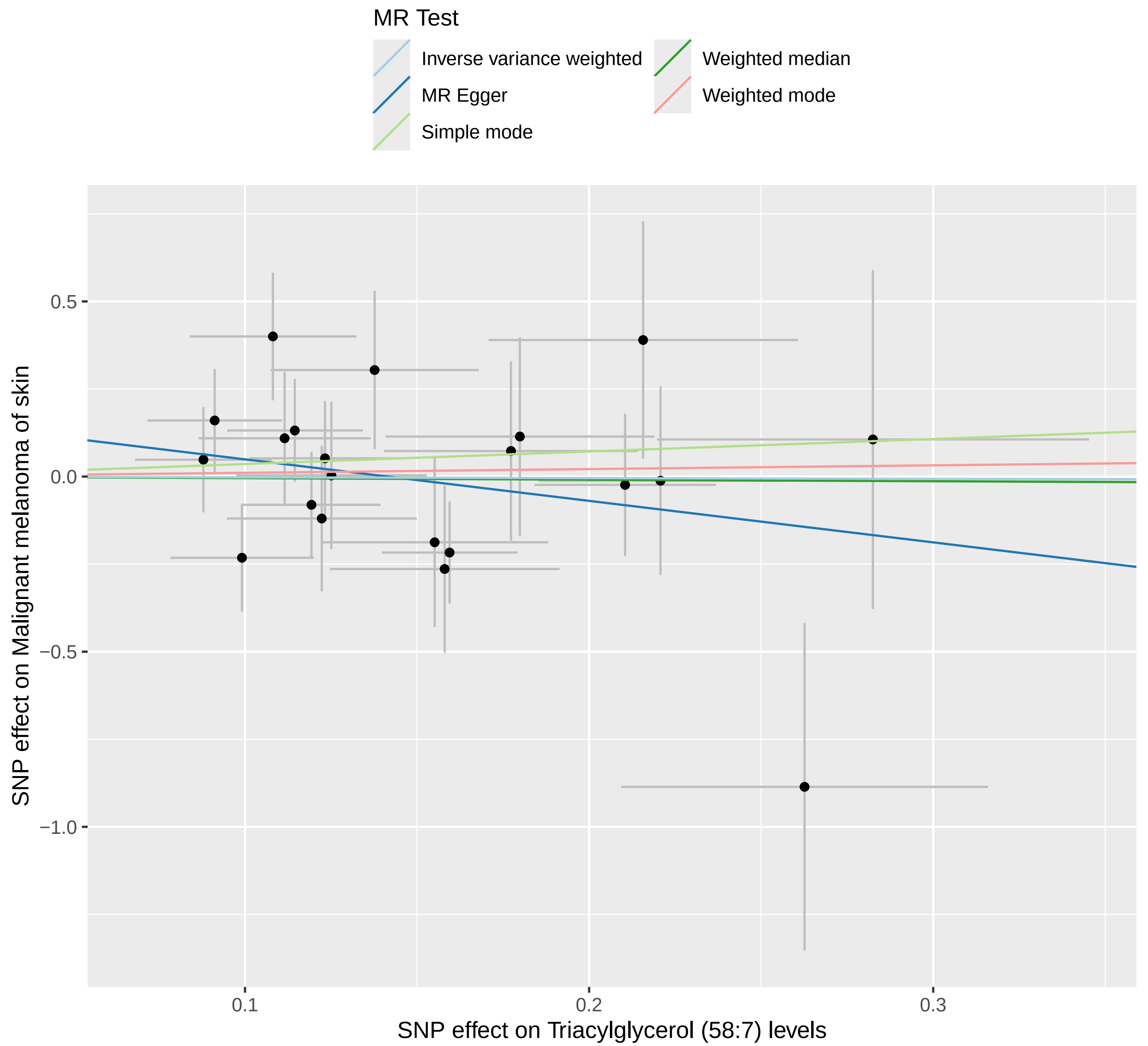

MR Test
Inverse variance weighted
MR Egger
Simple mode
Weighted median
Weighted mode
0.5
0.0
−0.5
−1.0
0.1
0.2
0.3
SNP effect on Malignant melanoma of skin
SNP effect on Triacylglycerol (58:7) levels

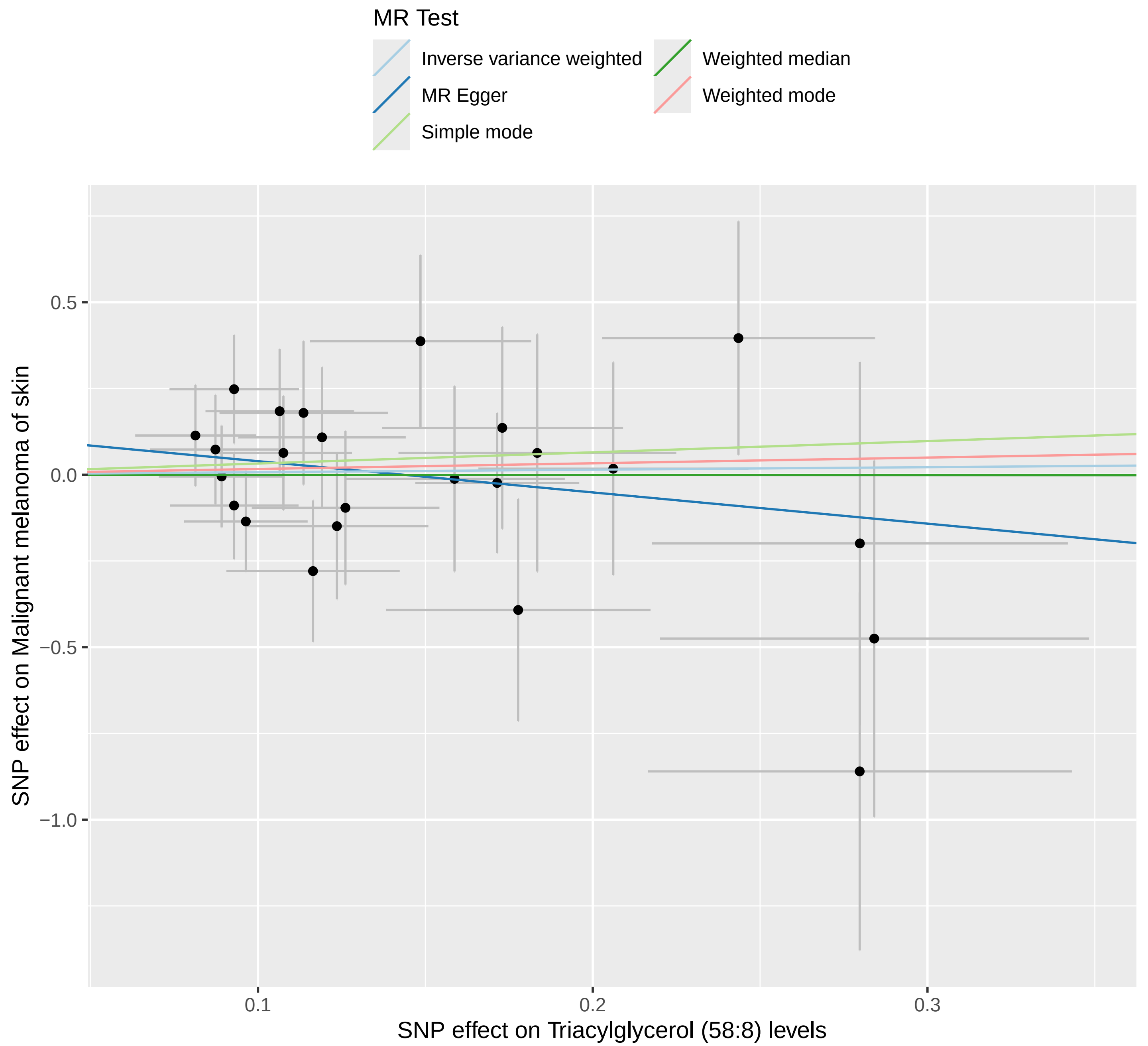

MR Test
Inverse variance weighted
MR Egger
Simple mode
Weighted median
Weighted mode
0.5
0.0
−0.5
−1.0
0.1
0.2
0.3
SNP effect on Malignant melanoma of skin
SNP effect on Triacylglycerol (58:8) levels
