# Supplementary material for: Genetically predicted metabolites mediate the association between lipidome and malignant melanoma of skin
Source: Front Oncol. 2024 Sep 10;14:1430533. doi: 10.3389/fonc.2024.1430533 (PMC11419955; doi:10.3389/fonc.2024.1430533)
Supplement: Supplementary file 11 [file SupplementaryFile10.pdf]

Supplementary material 10

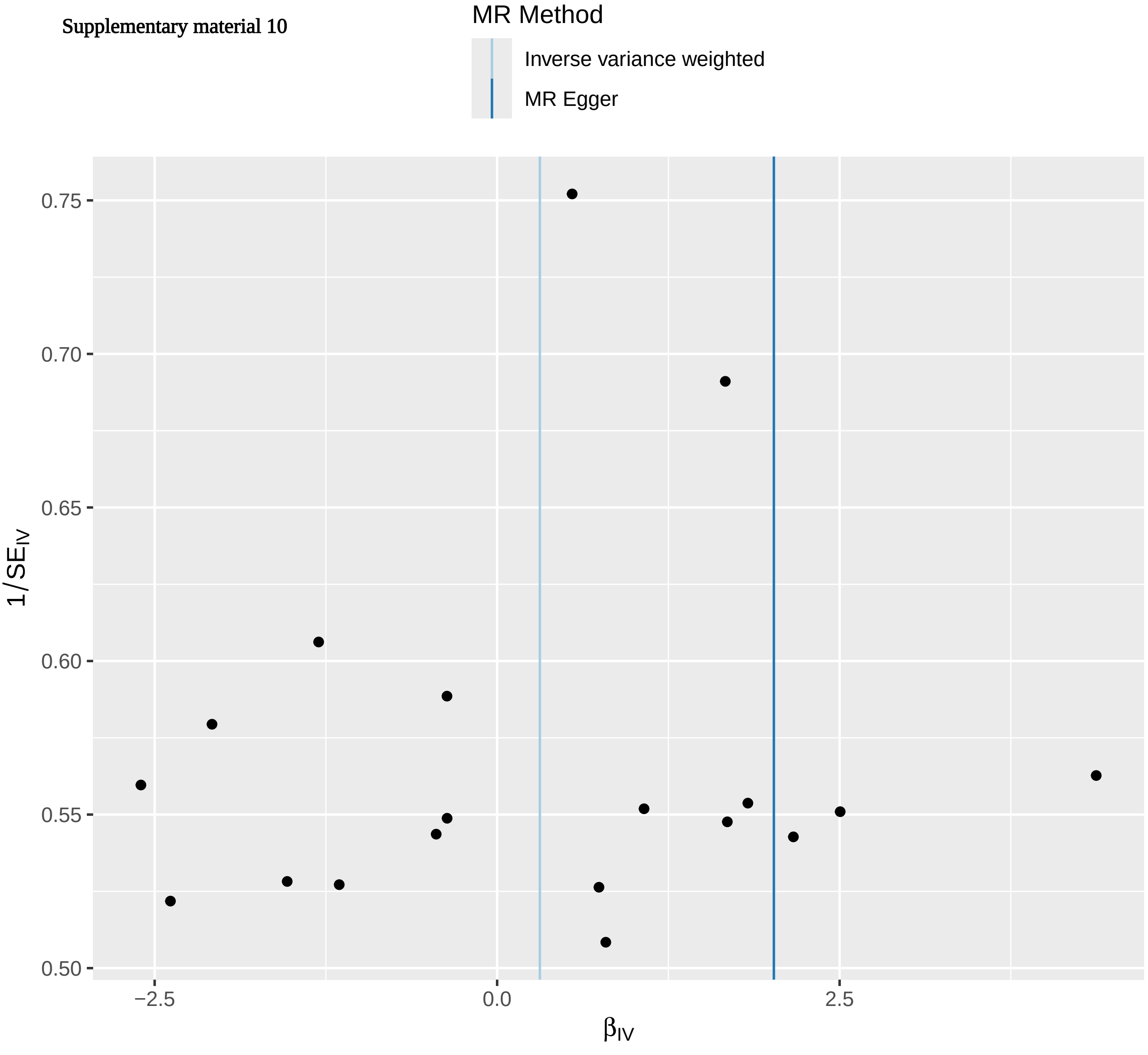

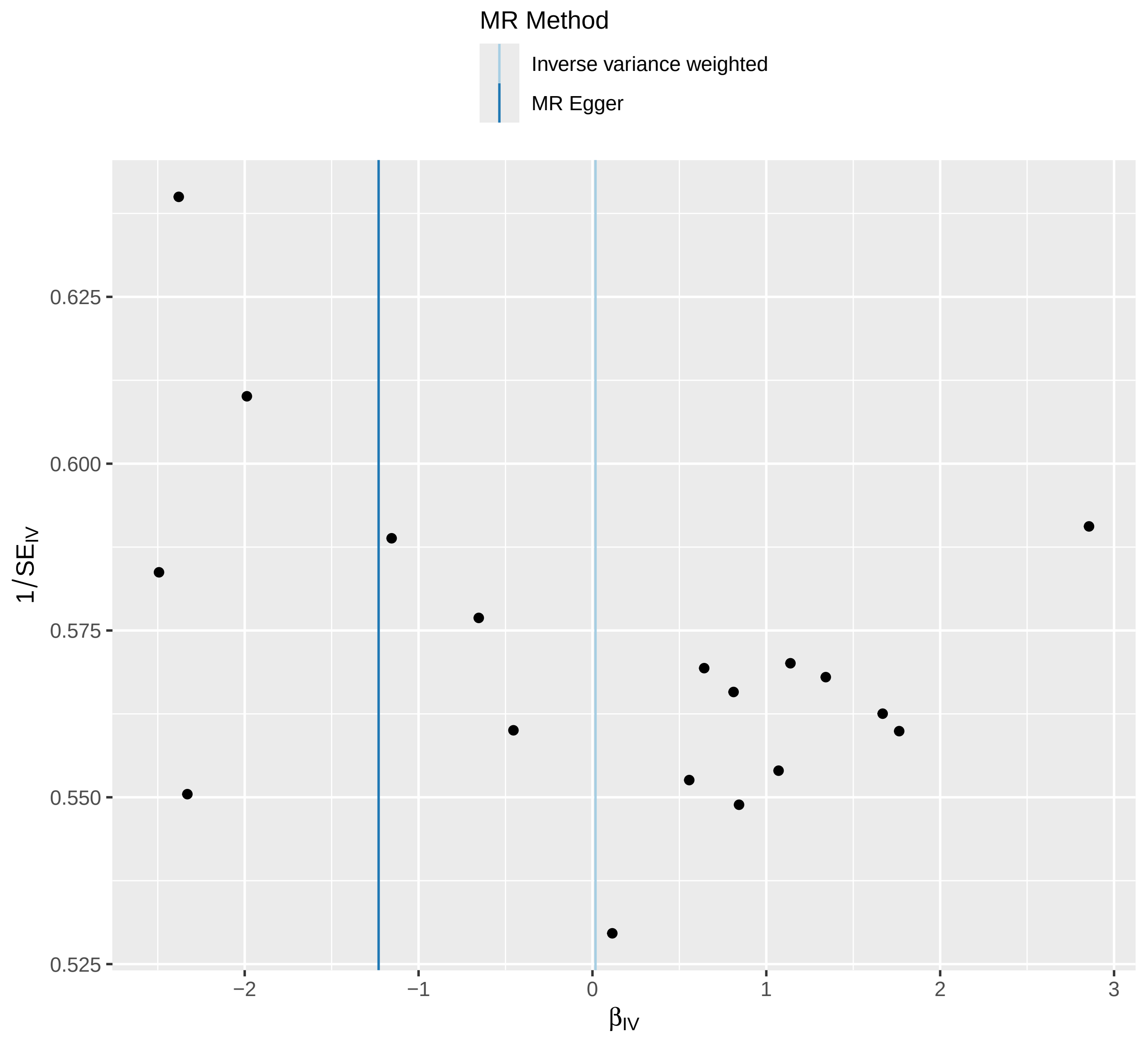
MR Method
Inverse variance weighted
MR Egger
0.625
0.600
0.575
0.550
0.525
1/SE_IV
−2
−1
0
1
2
3
β_IV

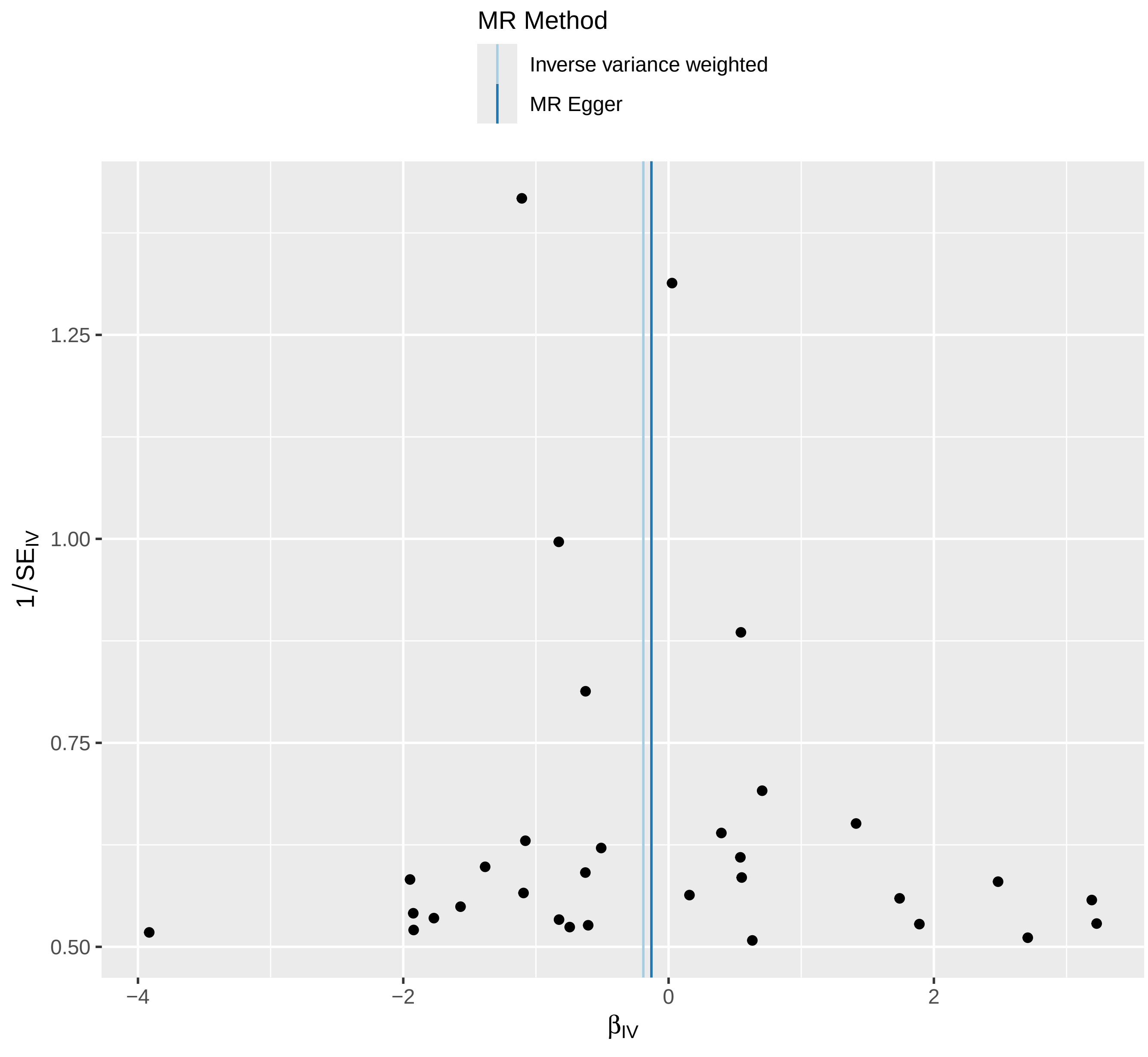
MR Method
Inverse variance weighted
MR Egger
1/SE$_{IV}$
1.25
1.00
0.75
0.50
−4
−2
0
2
β$_{IV}$

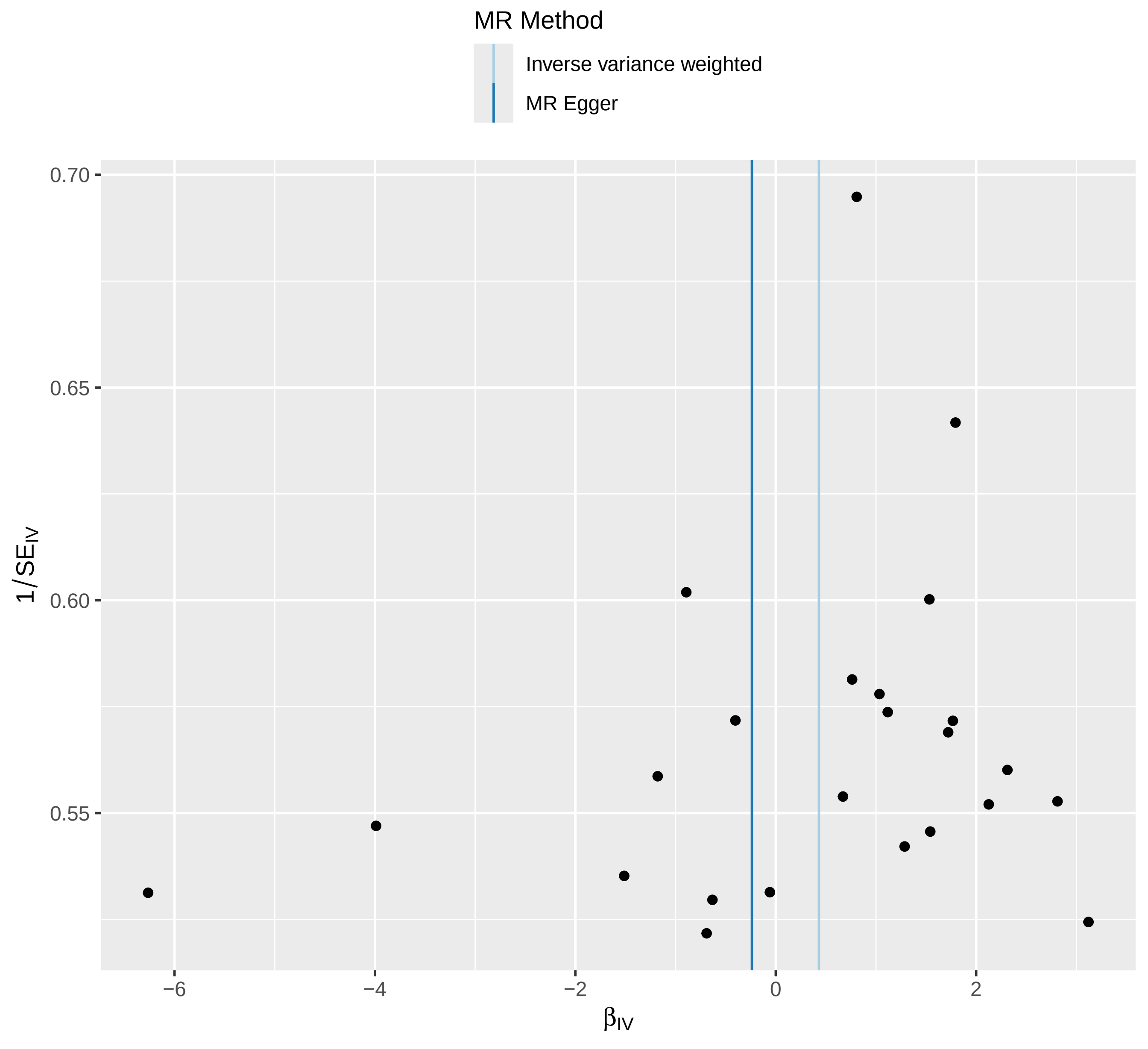
MR Method
Inverse variance weighted
MR Egger
0.70
0.65
0.60
0.55
1/SE_IV
−6
−4
−2
0
2
β_IV

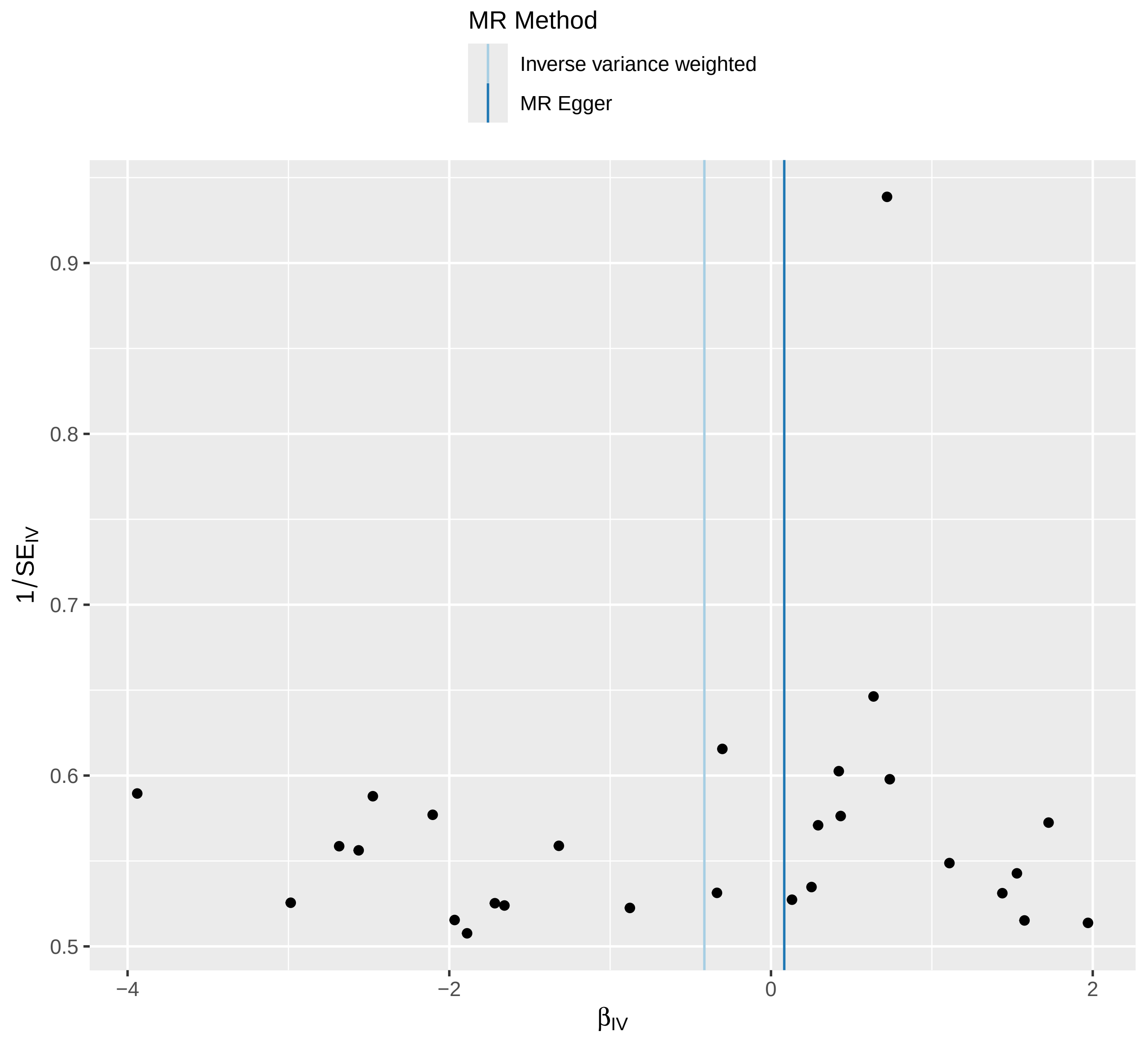
MR Method
Inverse variance weighted
MR Egger
0.9
0.8
0.7
0.6
0.5
$1/SE_{IV}$
−4
−2
0
2
β_{IV}

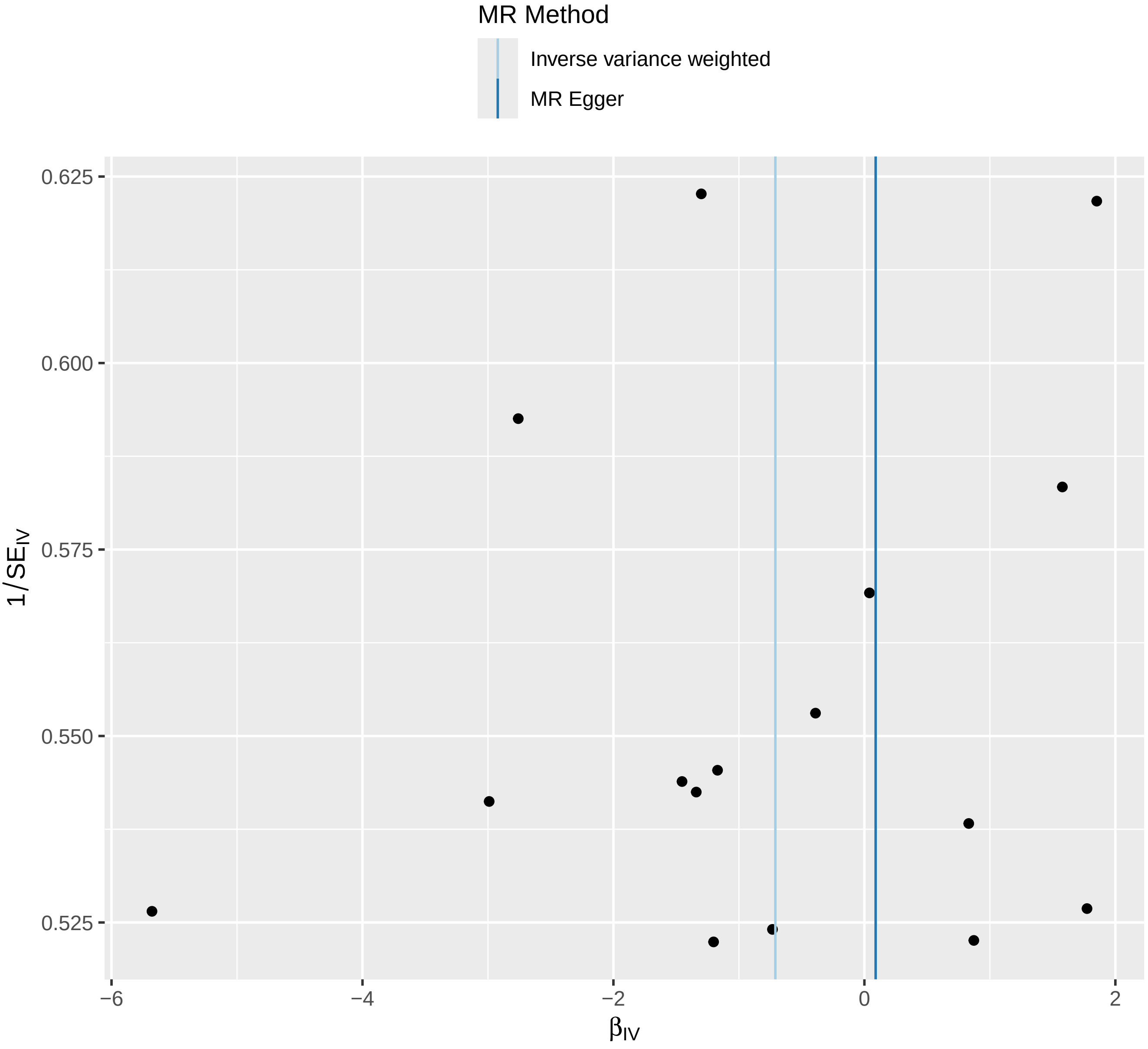

MR Method
Inverse variance weighted
MR Egger
0.625
0.600
0.575
0.550
0.525
$1/SE_{IV}$
−6
−4
−2
0
2
β_{IV}

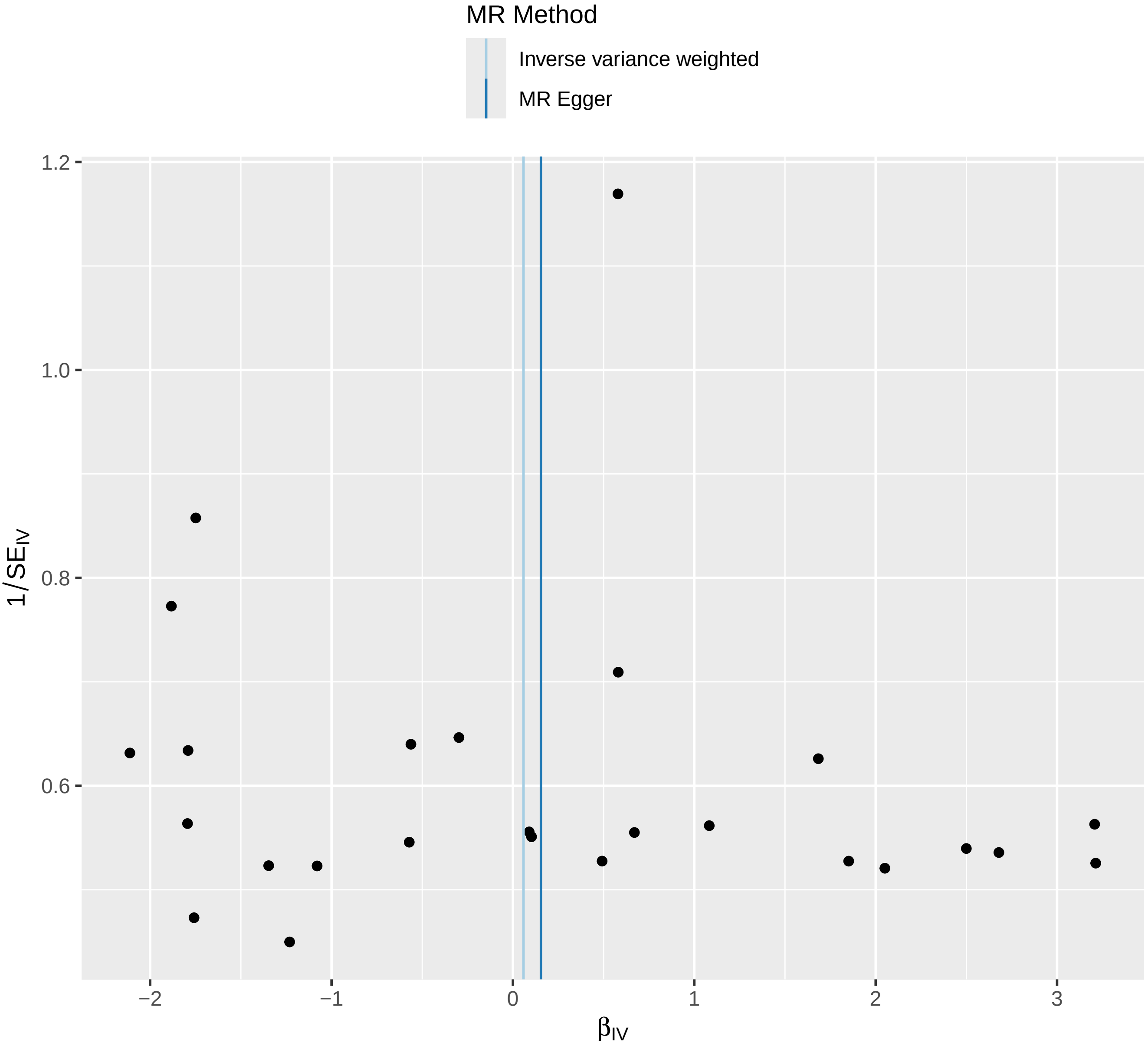
MR Method
Inverse variance weighted
MR Egger
1.2
1.0
0.8
0.6
1/SE_{IV}
−2
−1
0
1
2
3
β_{IV}

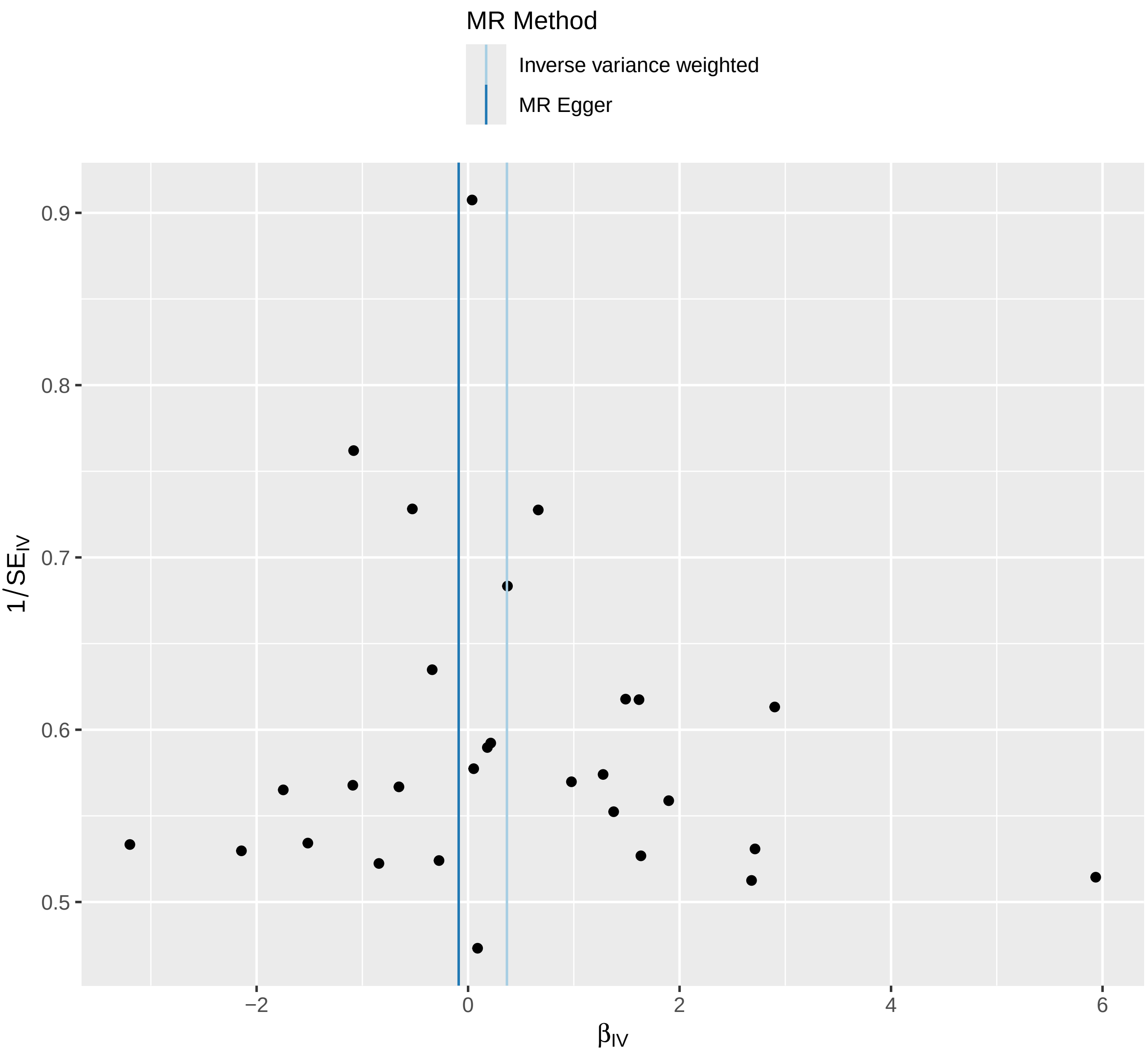

MR Method
Inverse variance weighted
MR Egger
1/SE_IV
0.9
0.8
0.7
0.6
0.5
−2
0
2
4
6
β_IV

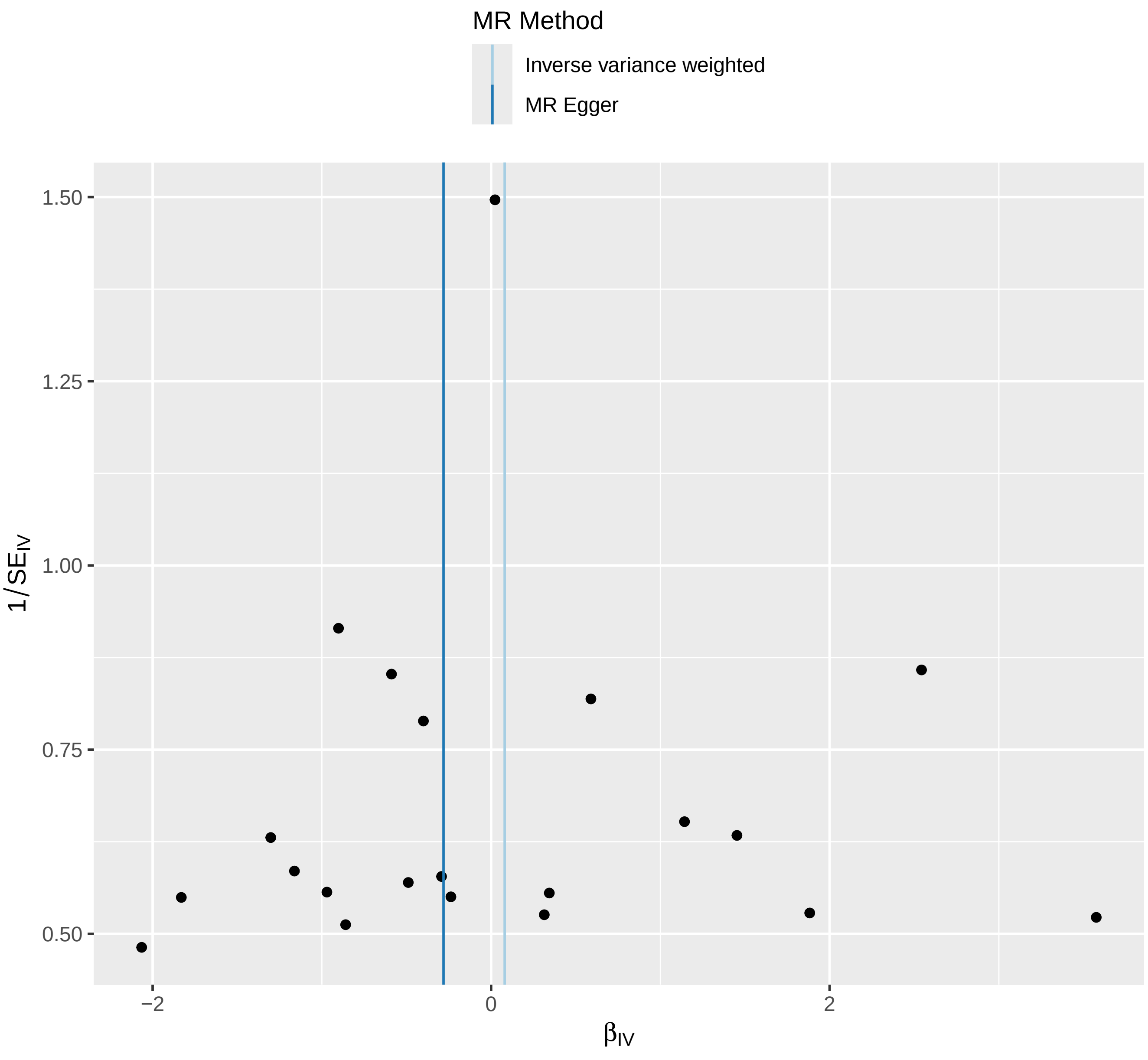
MR Method
Inverse variance weighted
MR Egger
1.50
1.25
1.00
0.75
0.50
1/SE_IV
−2
0
2
β_IV

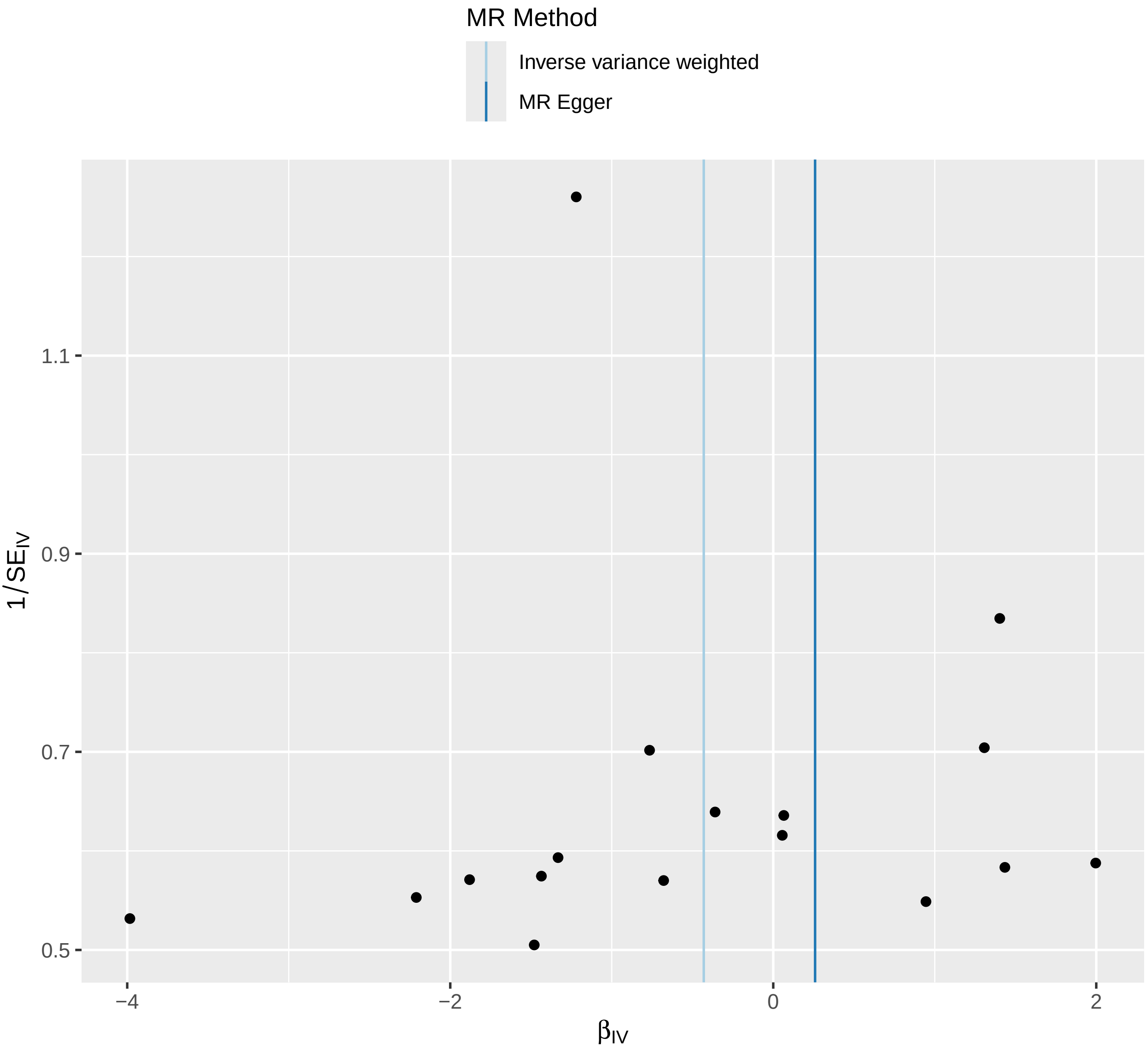
MR Method
Inverse variance weighted
MR Egger
1.1
0.9
0.7
0.5
1/SE$_{IV}$
−4
−2
0
2
β$_{IV}$

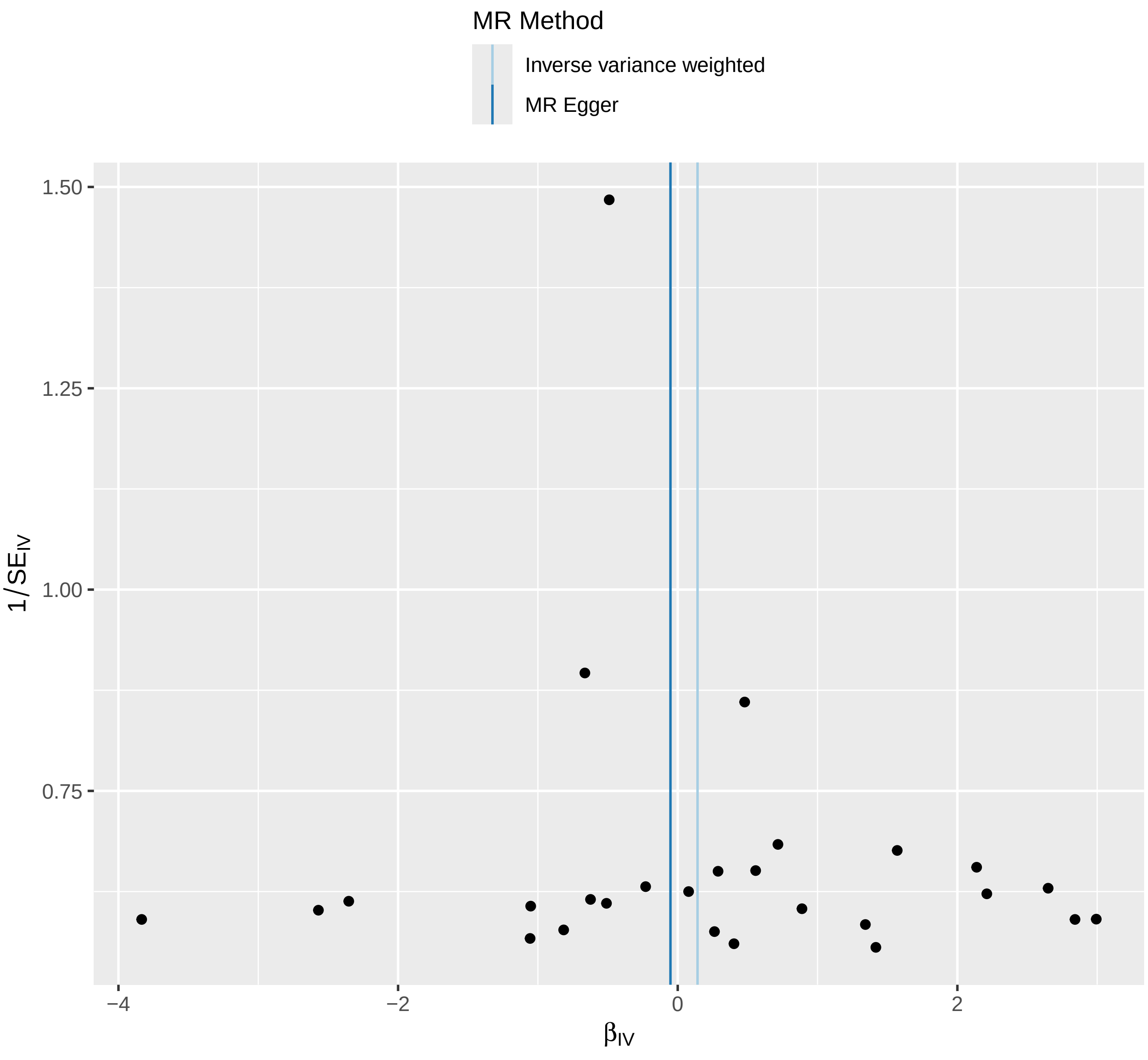
MR Method
Inverse variance weighted
MR Egger
1.50
1.25
1.00
0.75
1/SE_IV
−4
−2
0
2
β_IV

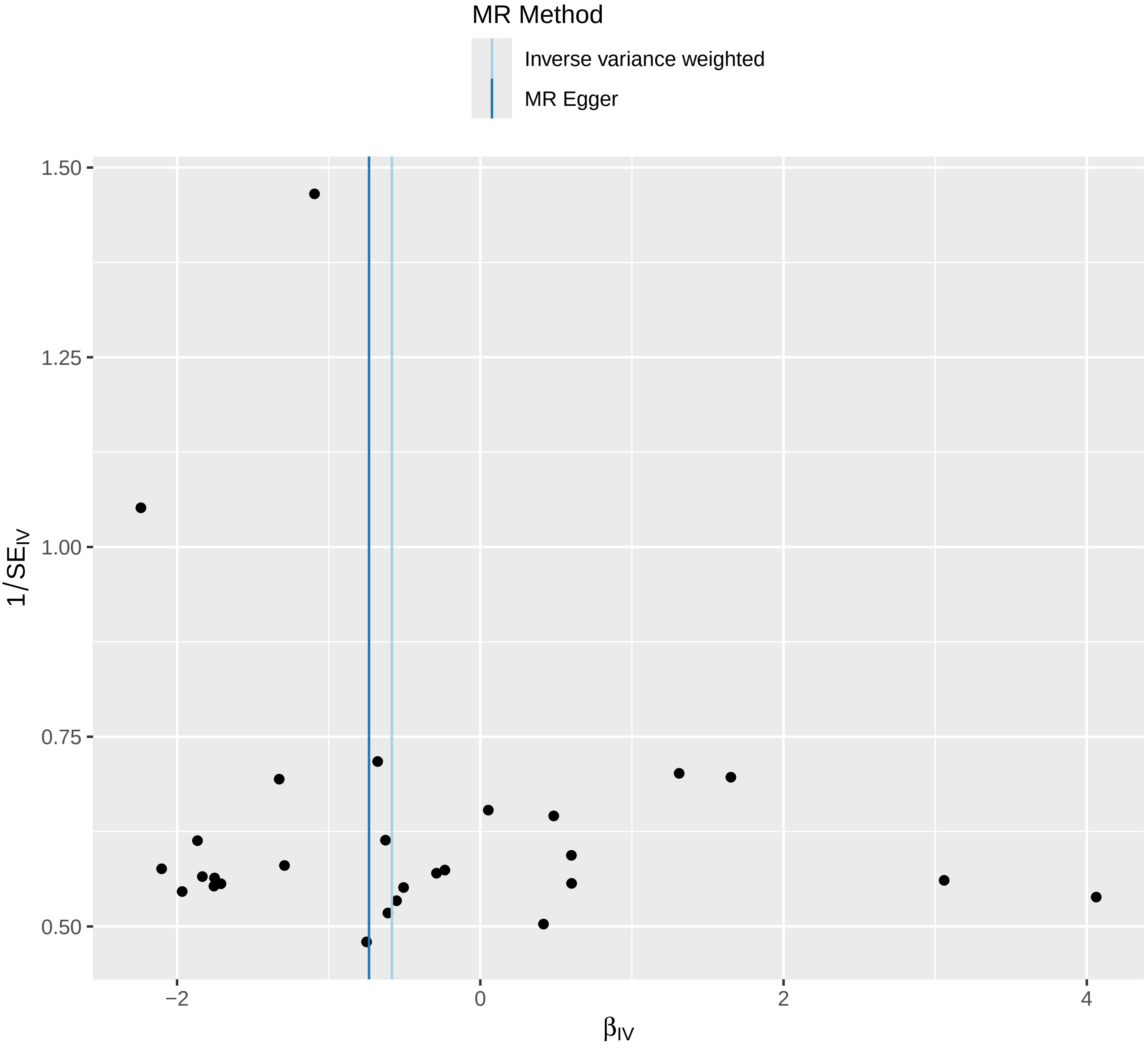

MR Method
Inverse variance weighted
MR Egger
1.50
1.25
1.00
0.75
0.50
$1/SE_{IV}$
−2
0
2
4
β_{IV}

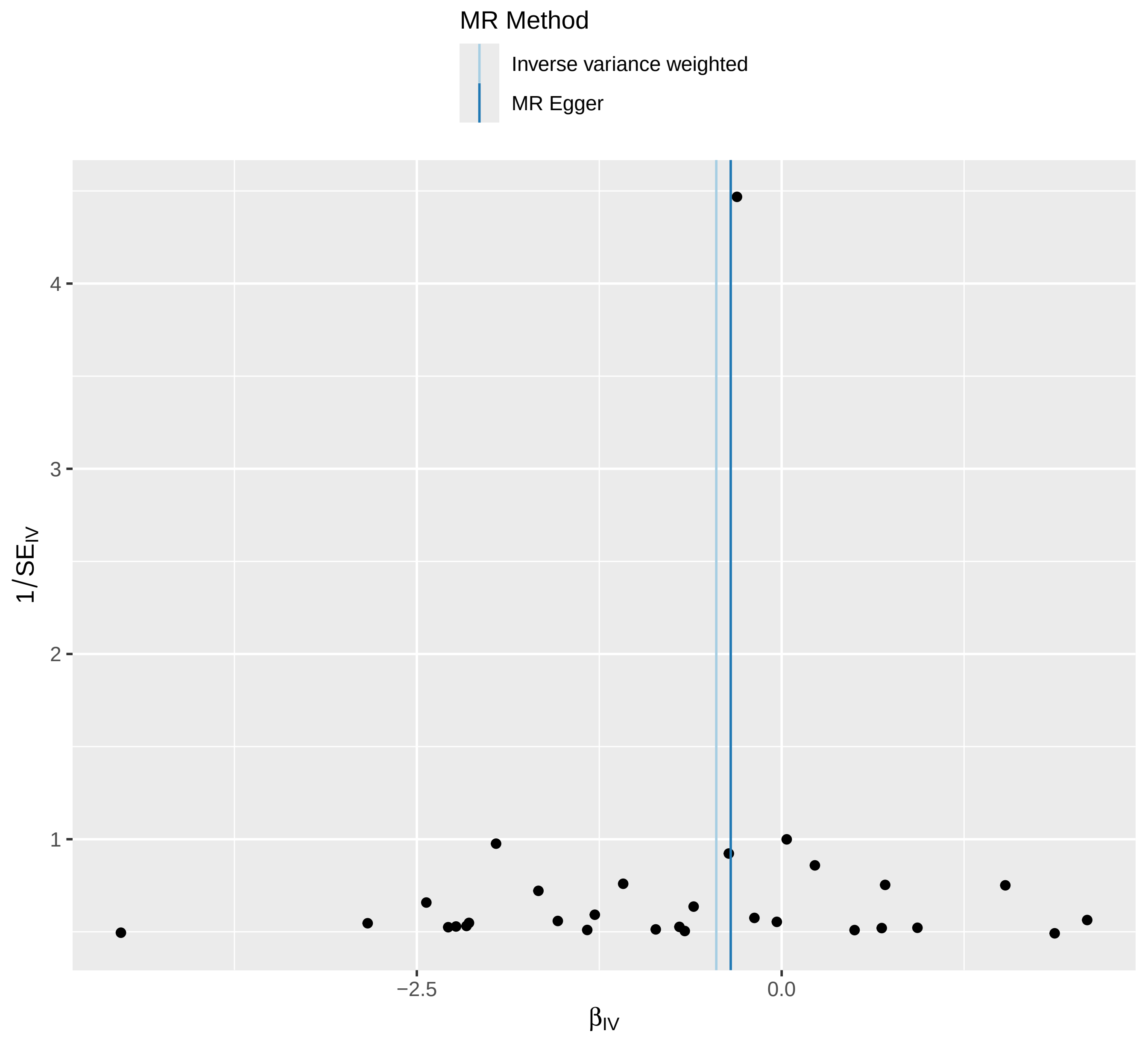
MR Method
Inverse variance weighted
MR Egger
1/SE_IV
4
3
2
1
β_IV
−2.5
0.0

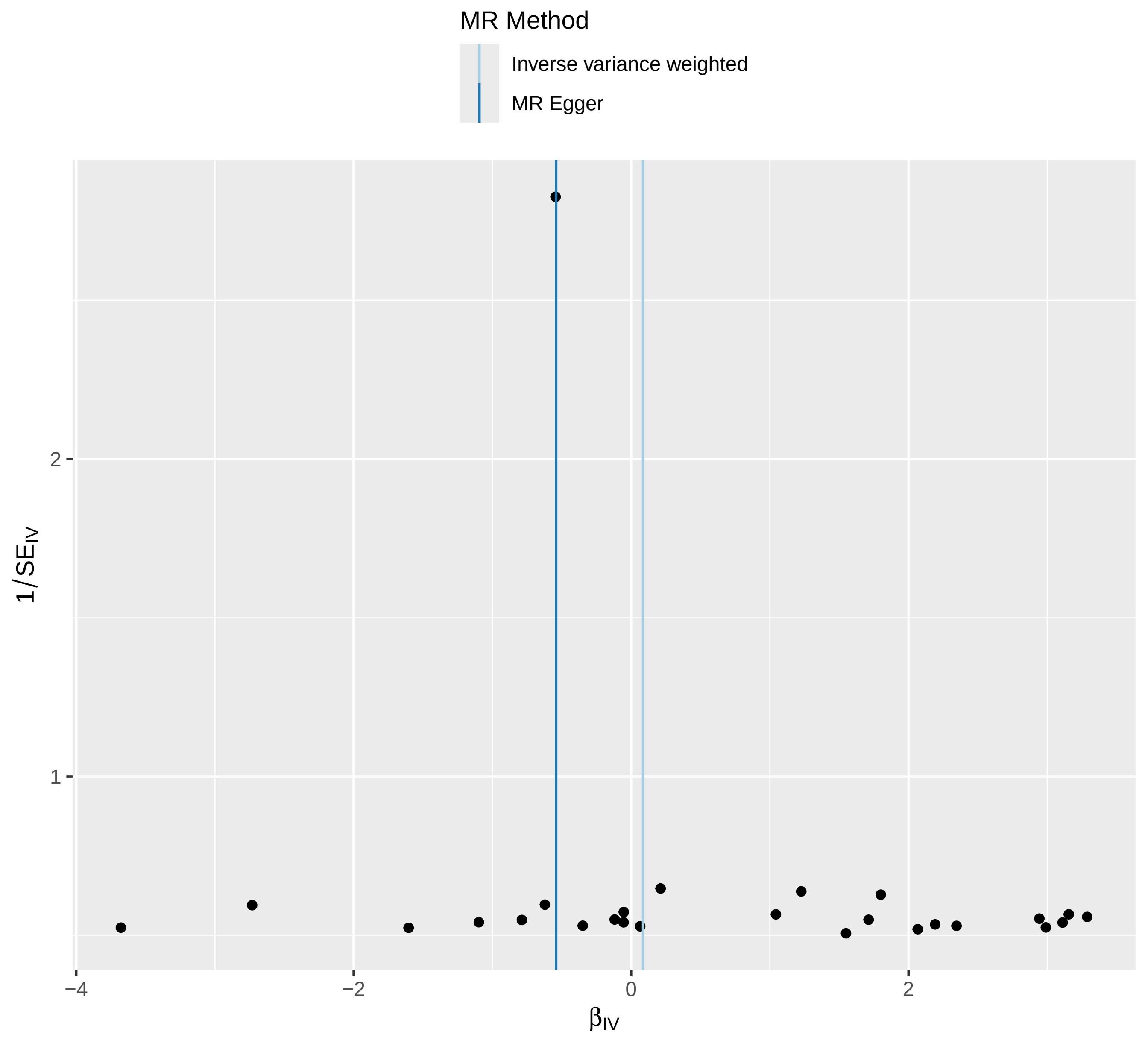

MR Method
Inverse variance weighted
MR Egger
2
1
1/SE$_{IV}$
−4
−2
0
2
β$_{IV}$

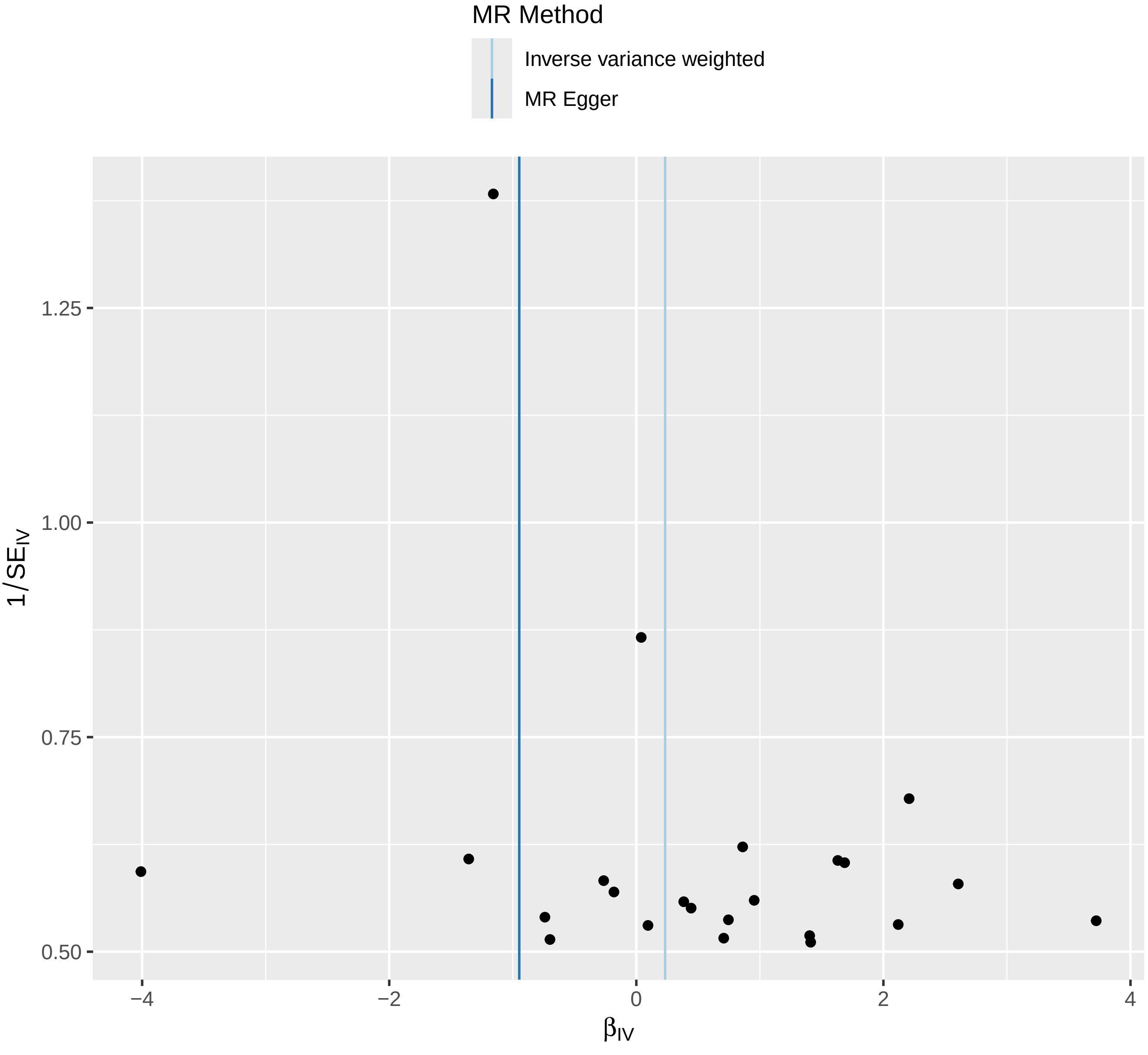
MR Method
Inverse variance weighted
MR Egger
1.25
1.00
0.75
0.50
1/SE_IV
−4
−2
0
2
4
β_IV

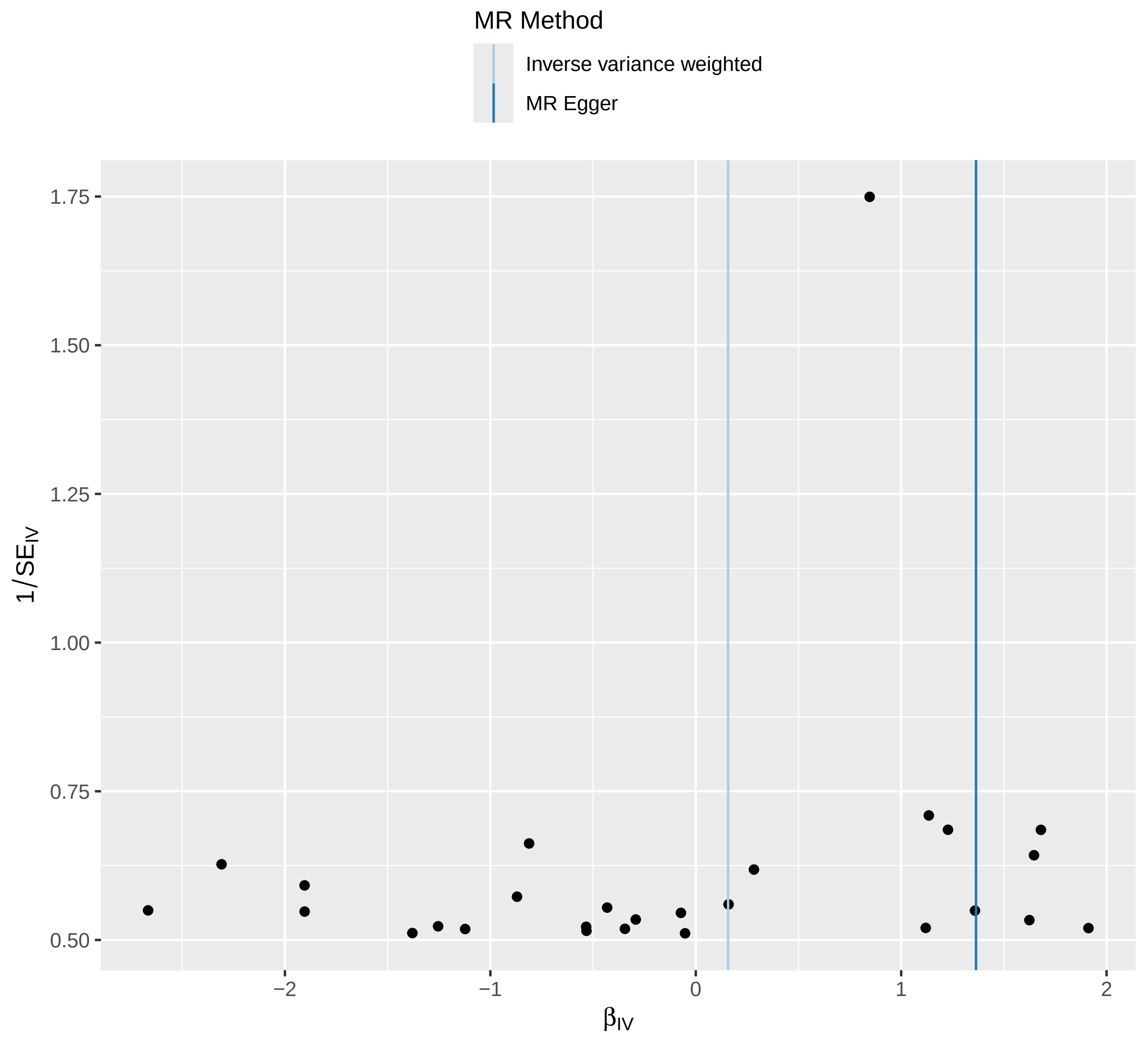
MR Method
Inverse variance weighted
MR Egger
1.75
1.50
1.25
1.00
0.75
0.50
1/SE_IV
−2
−1
0
1
2
β_IV

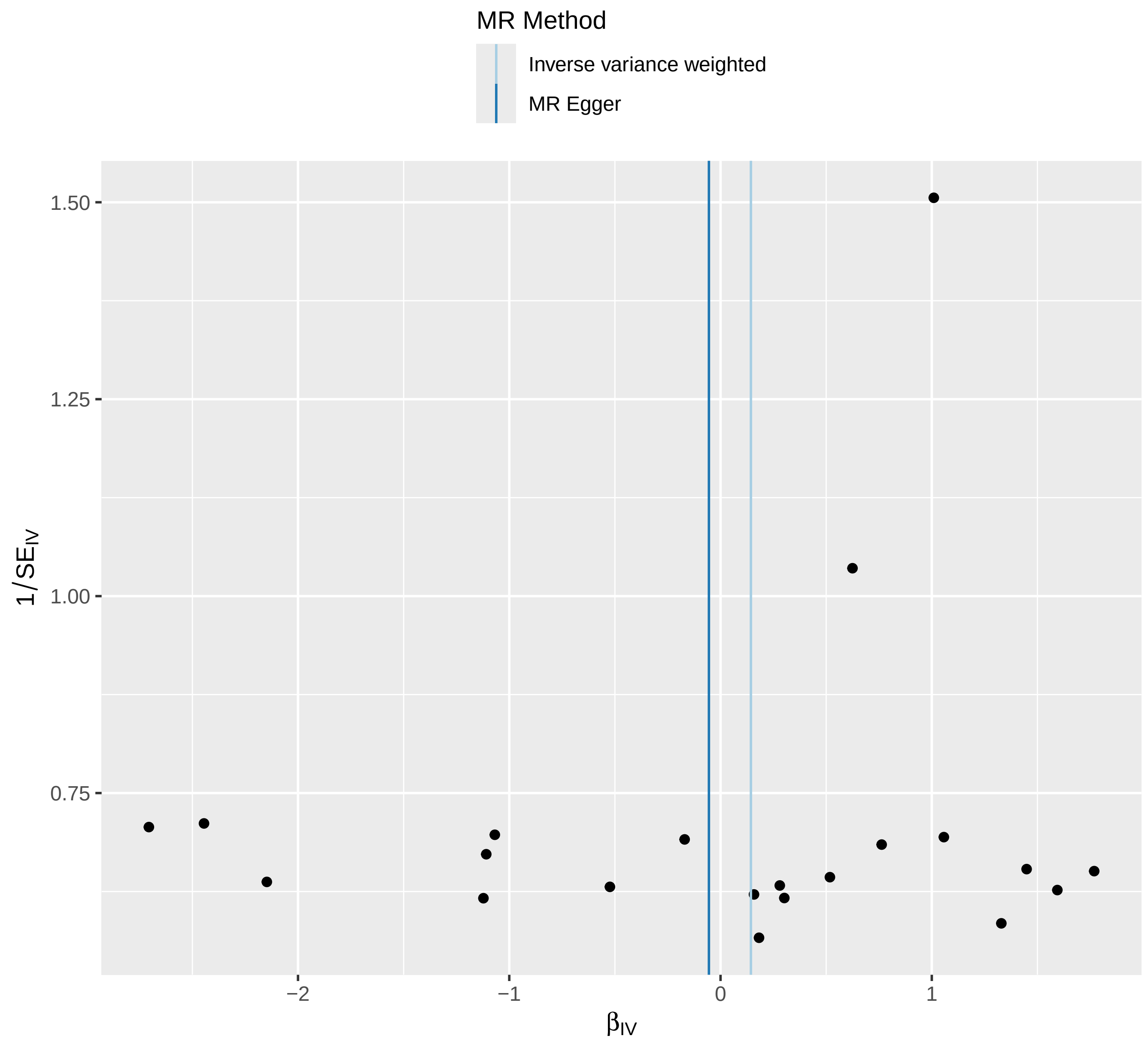
MR Method
Inverse variance weighted
MR Egger
1.50
1.25
1.00
0.75
1/SE$_{IV}$
−2
−1
0
1
β$_{IV}$

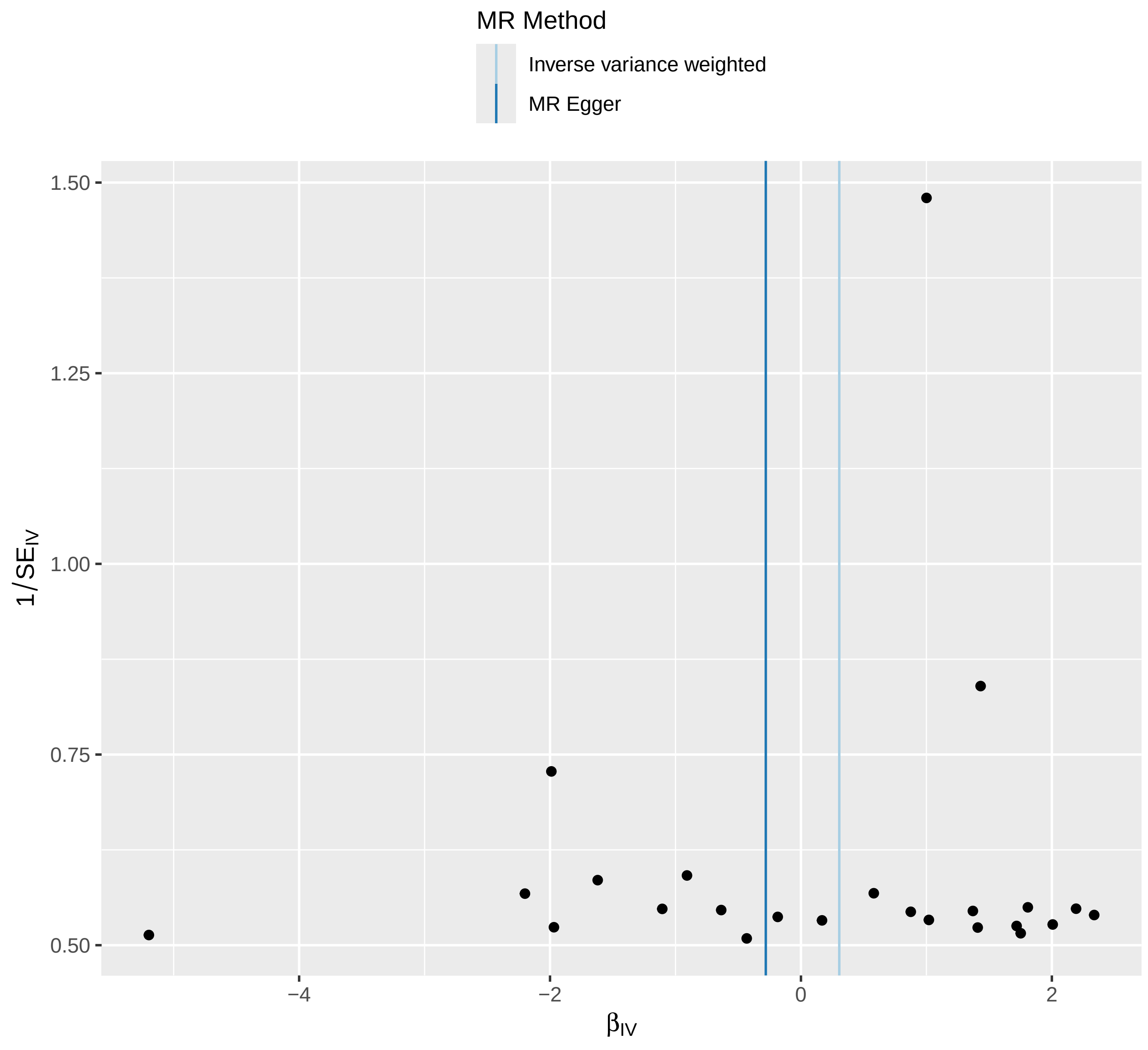

MR Method
Inverse variance weighted
MR Egger
1.50
1.25
1.00
0.75
0.50
1/SE_IV
−4
−2
0
2
β_IV

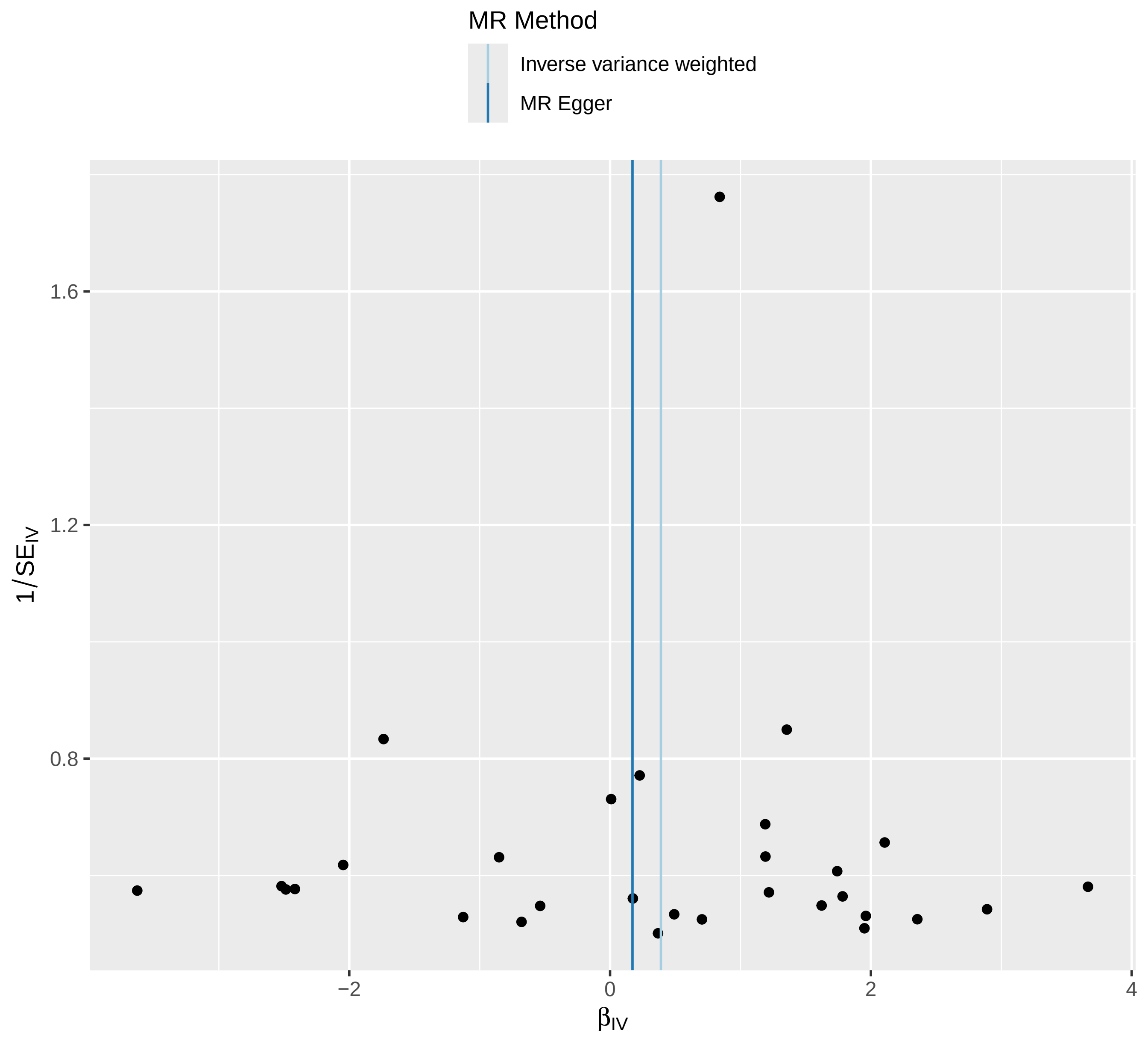
MR Method
Inverse variance weighted
MR Egger
1.6
1.2
0.8
1/SE_IV
−2
0
2
4
β_IV

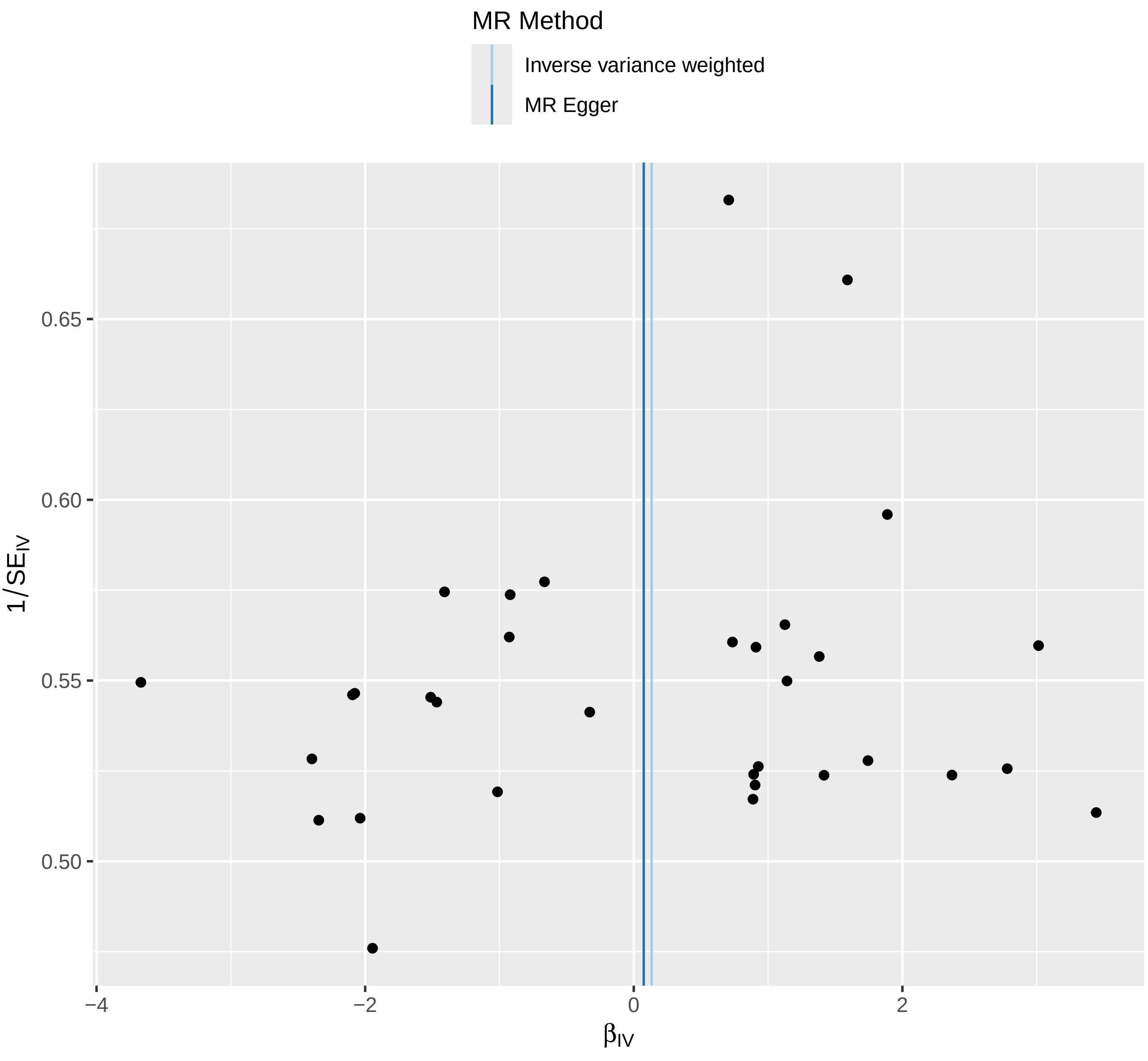
MR Method
Inverse variance weighted
MR Egger
0.65
0.60
0.55
0.50
1/SE_IV
−4
−2
0
2
β_IV

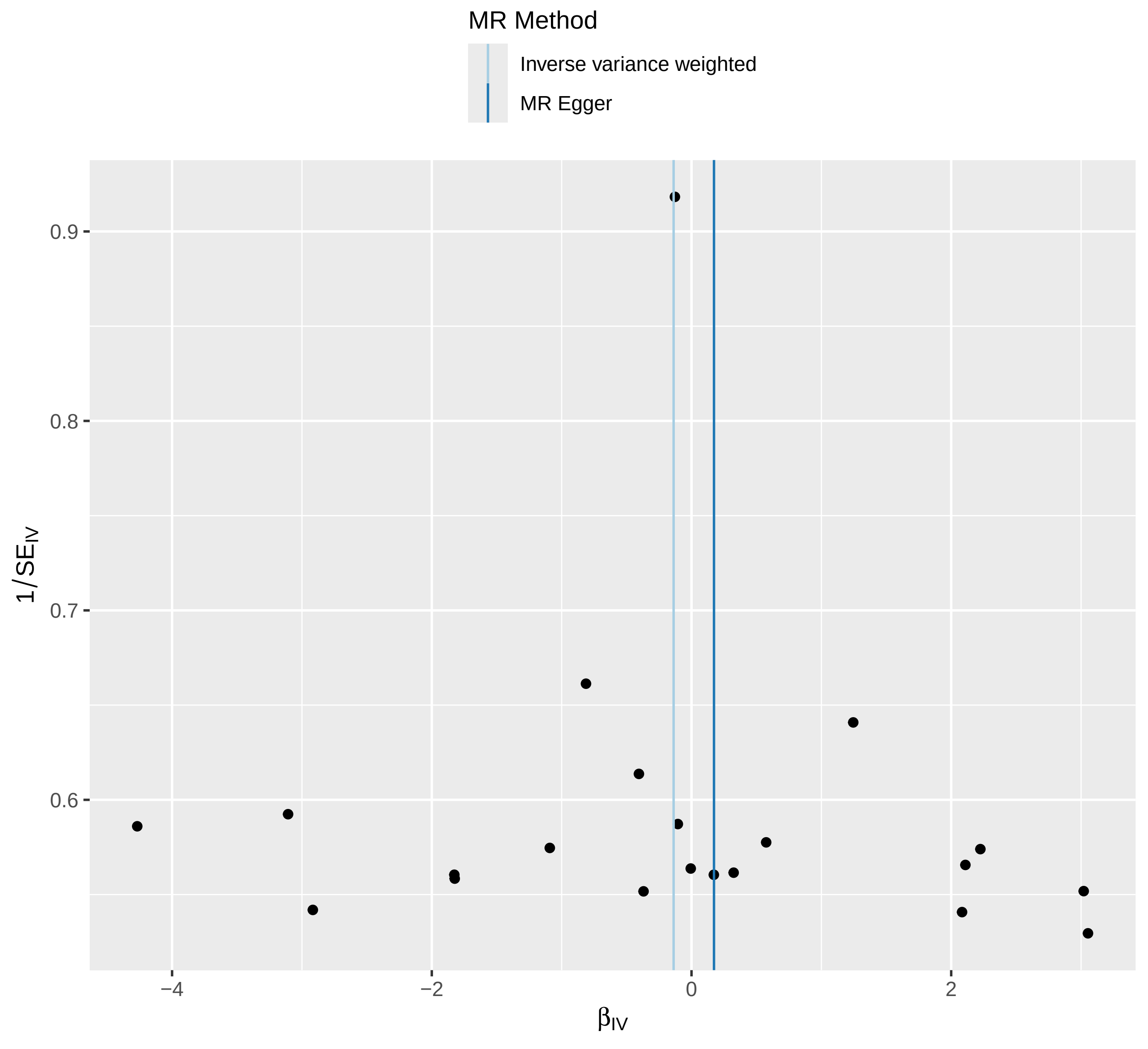
MR Method
Inverse variance weighted
MR Egger
1/SE$_{IV}$
0.9
0.8
0.7
0.6
−4
−2
0
2
β$_{IV}$

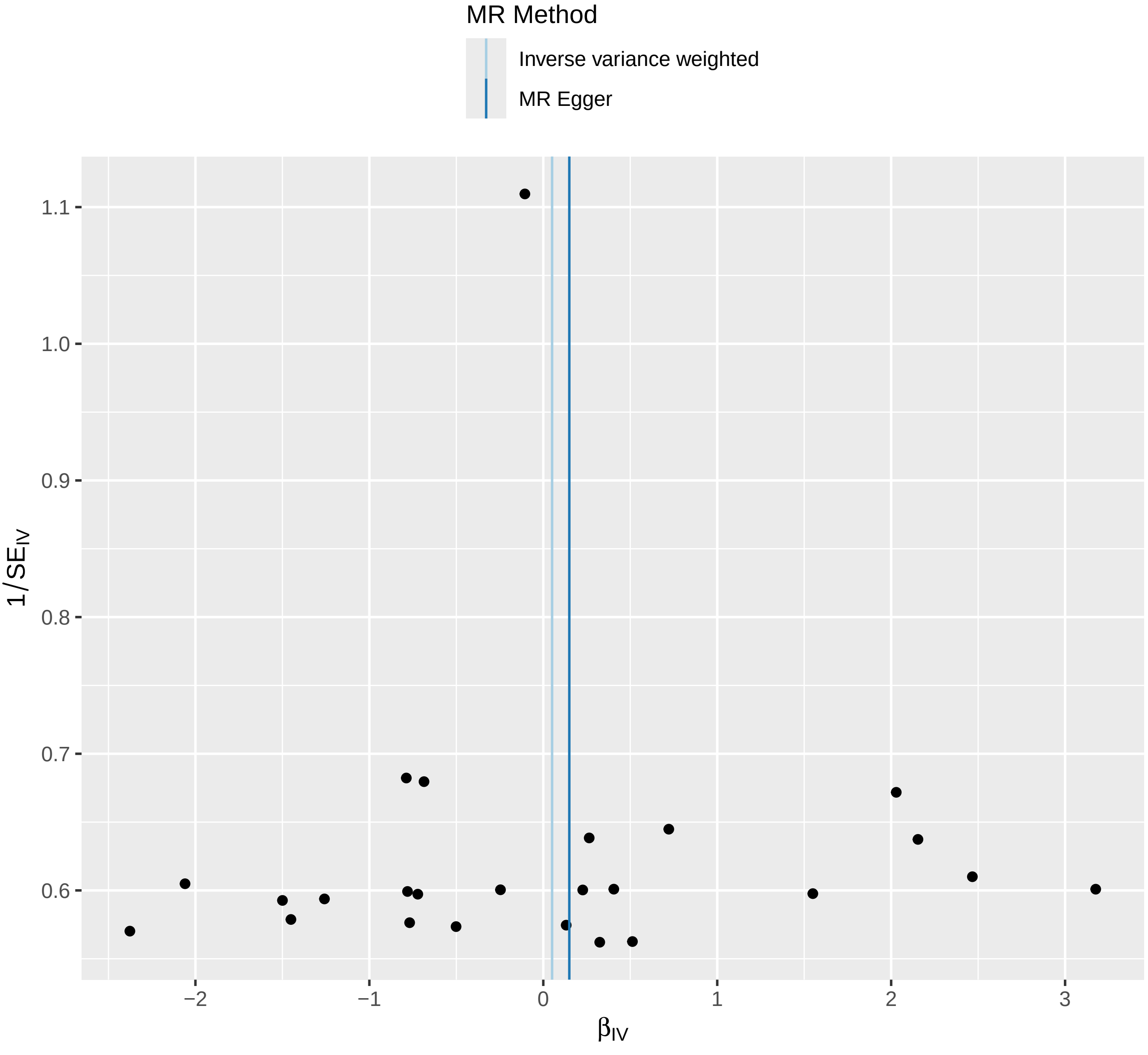

MR Method
Inverse variance weighted
MR Egger
1.1
1.0
0.9
0.8
0.7
0.6
1/SE_IV
−2
−1
0
1
2
3
β_IV

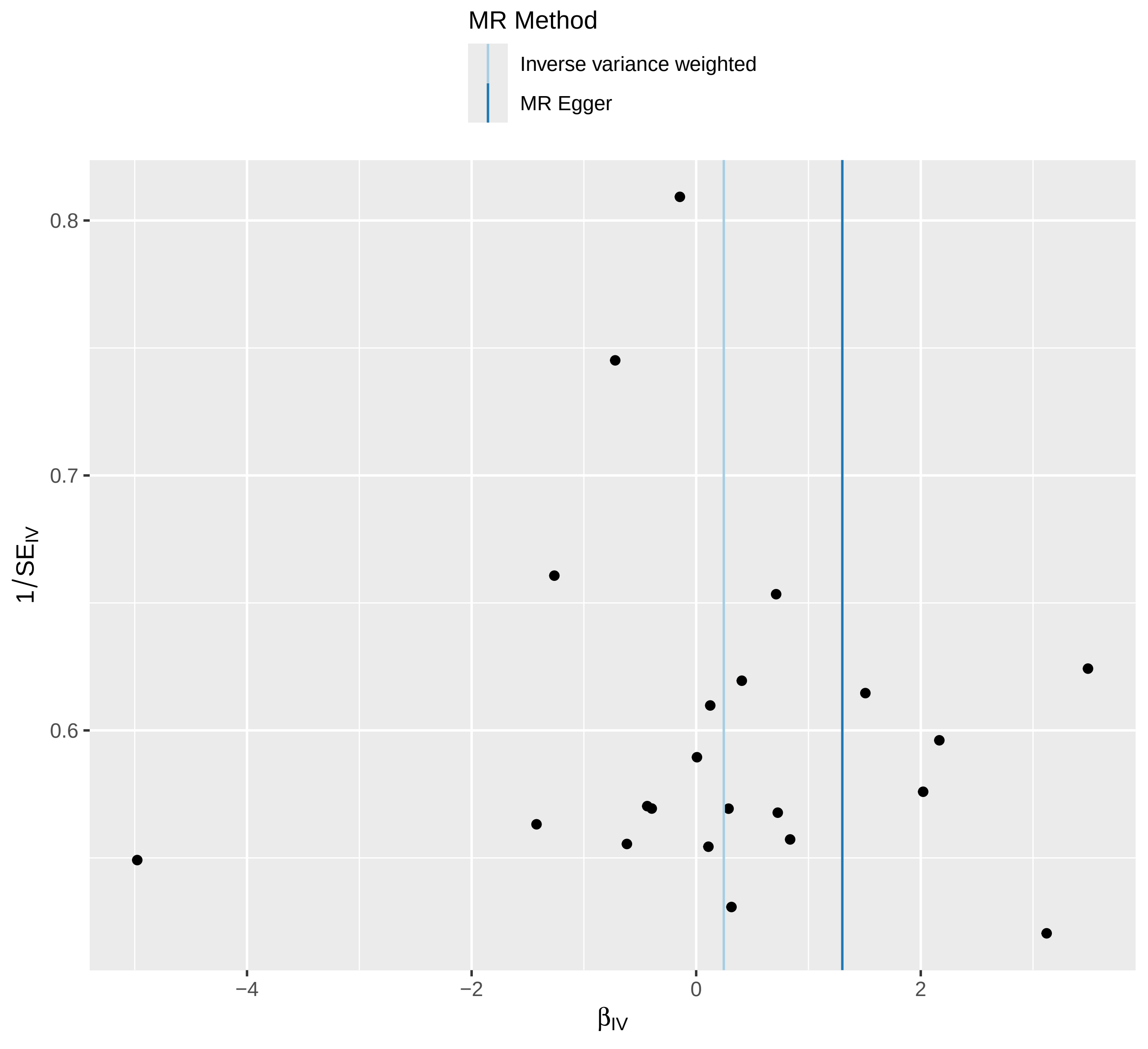
MR Method
Inverse variance weighted
MR Egger
0.8
0.7
0.6
1/SE_IV
−4
−2
0
2
β_IV

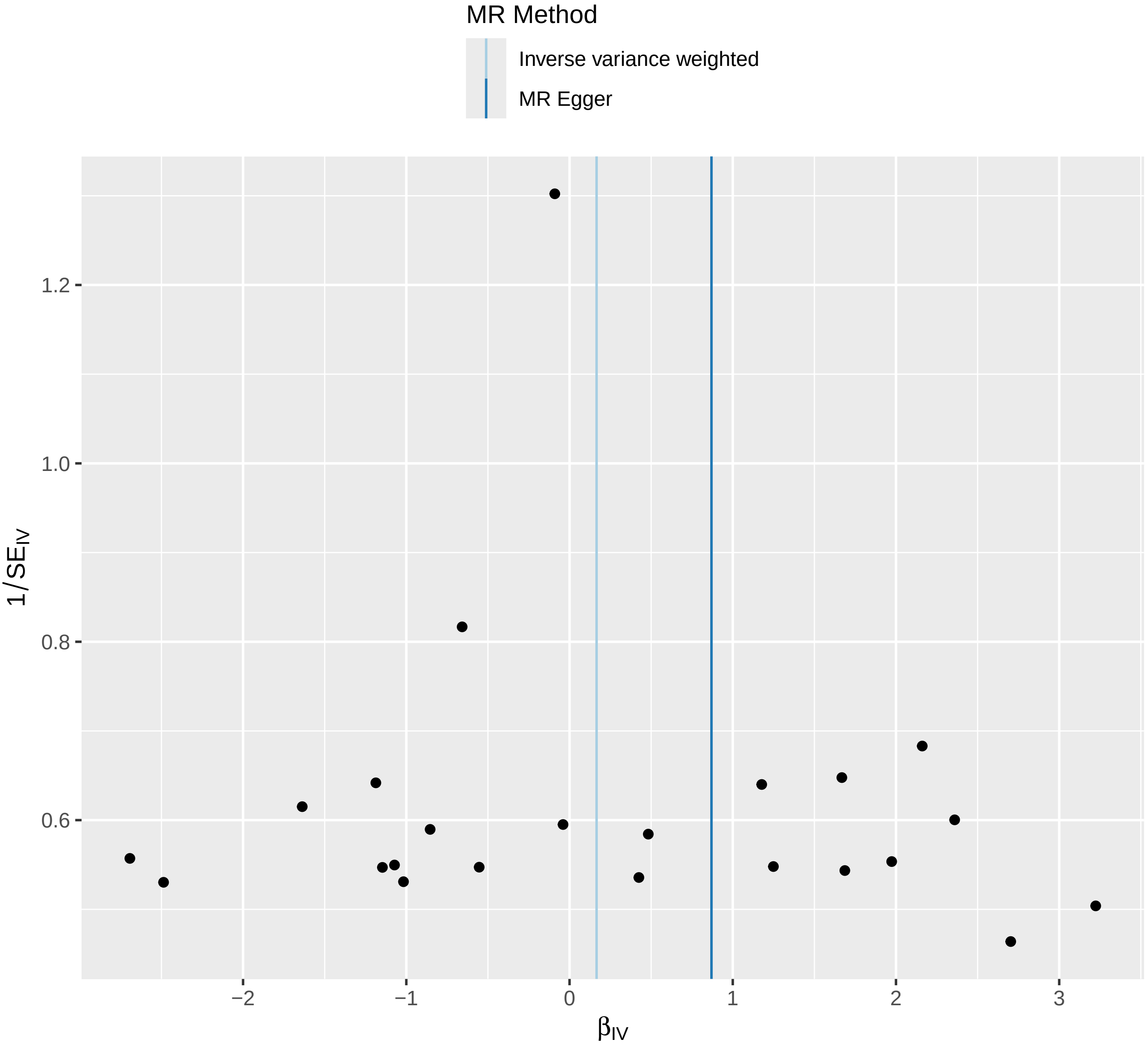
MR Method
Inverse variance weighted
MR Egger
1.2
1.0
0.8
0.6
1/SE_IV
−2
−1
0
1
2
3
β_IV

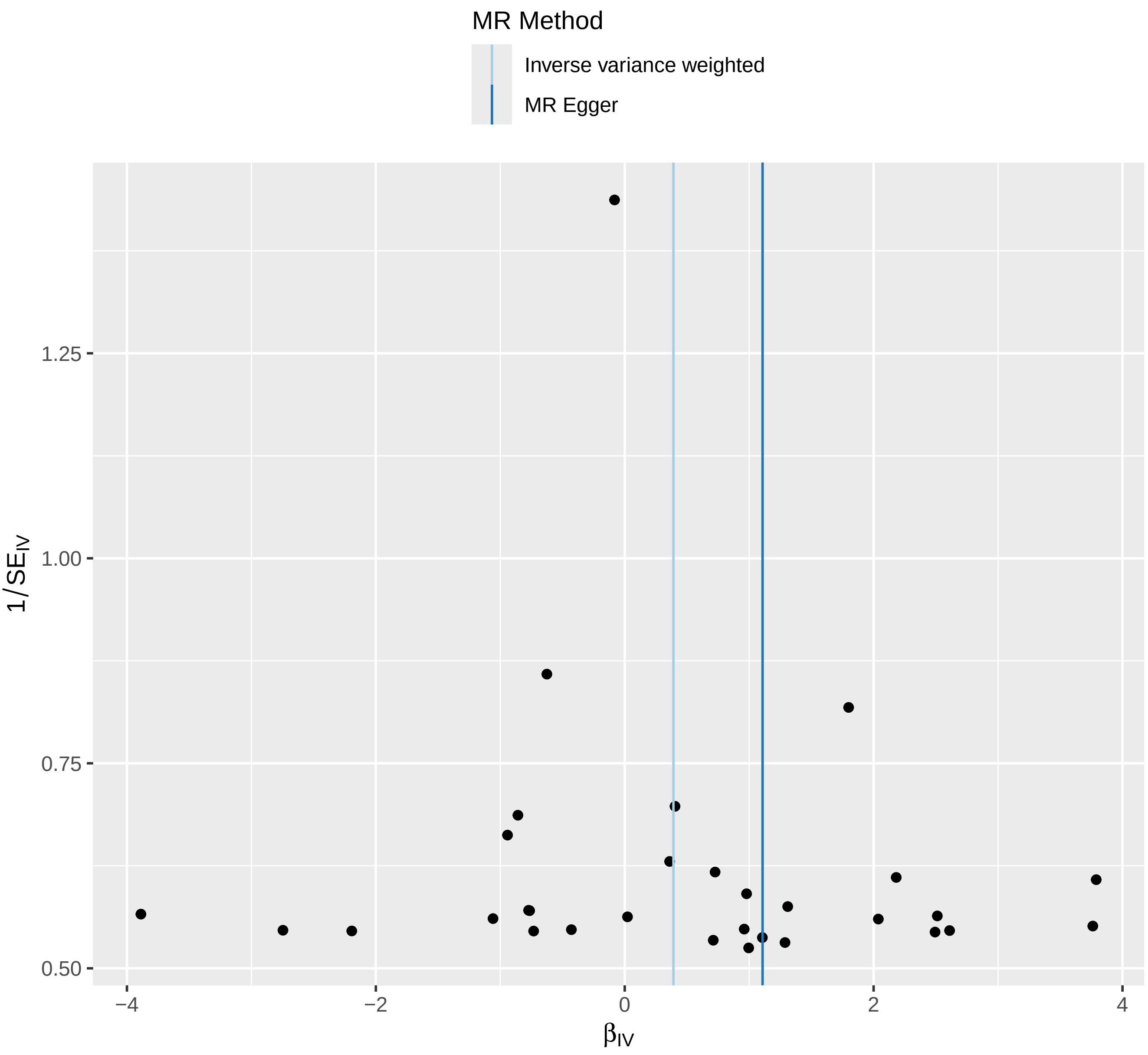
MR Method
Inverse variance weighted
MR Egger
1.25
1.00
0.75
0.50
$1/SE_{IV}$
−4
−2
0
2
4
β_{IV}

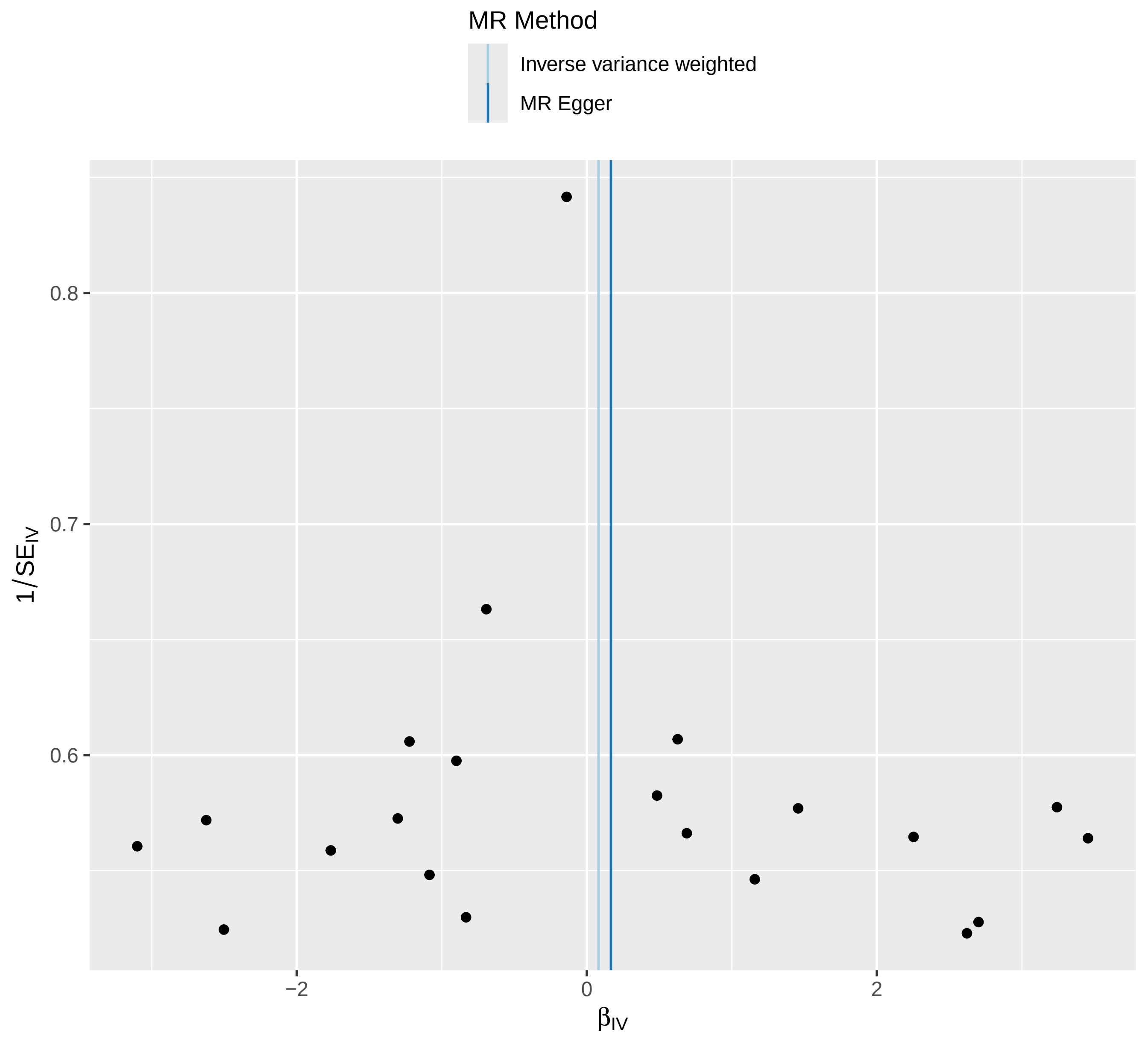
MR Method
Inverse variance weighted
MR Egger
0.8
0.7
0.6
1/SE_IV
−2
0
2
β_IV

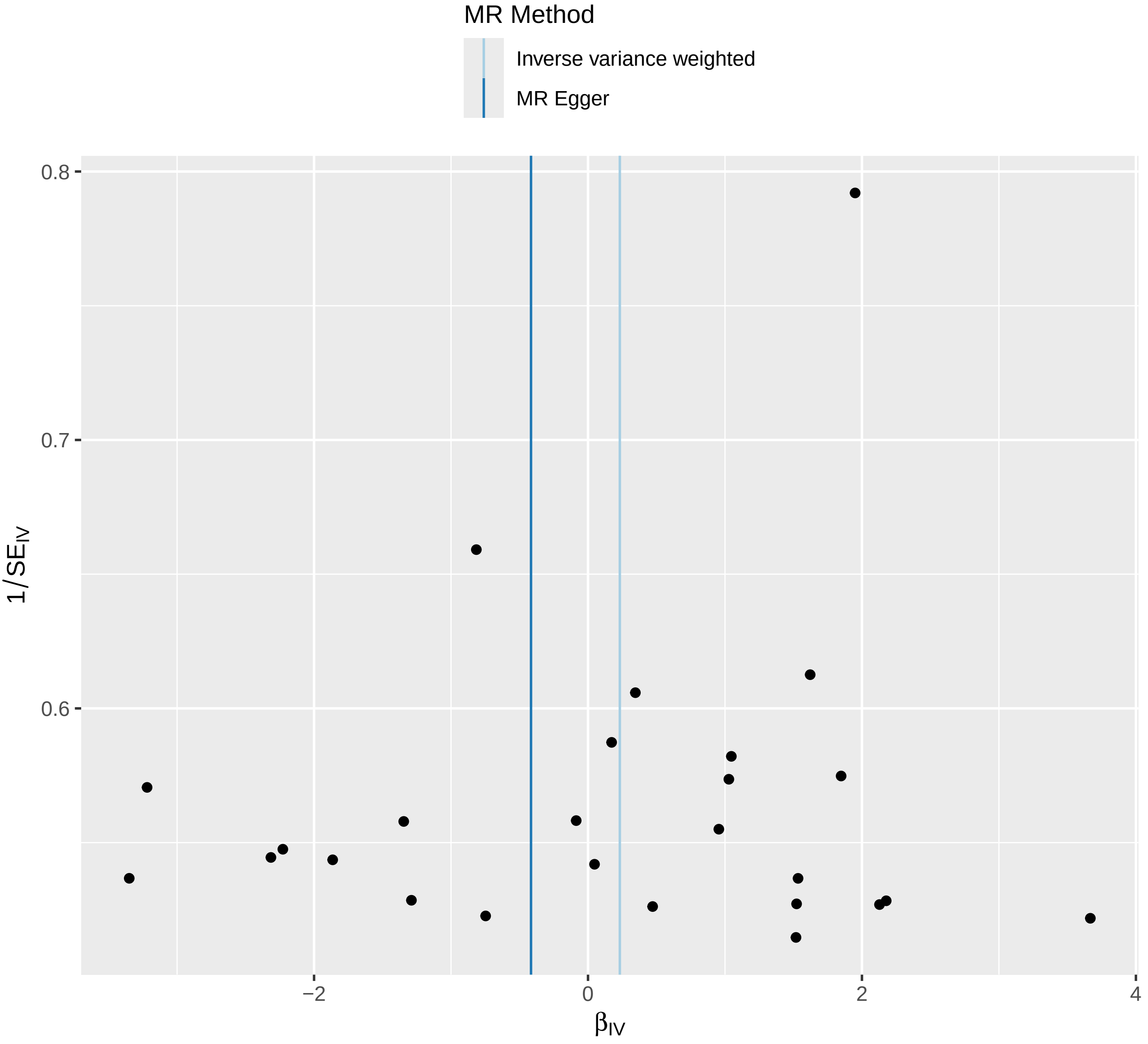

MR Method
Inverse variance weighted
MR Egger
0.8
0.7
0.6
1/SE_IV
−2
0
2
4
β_IV

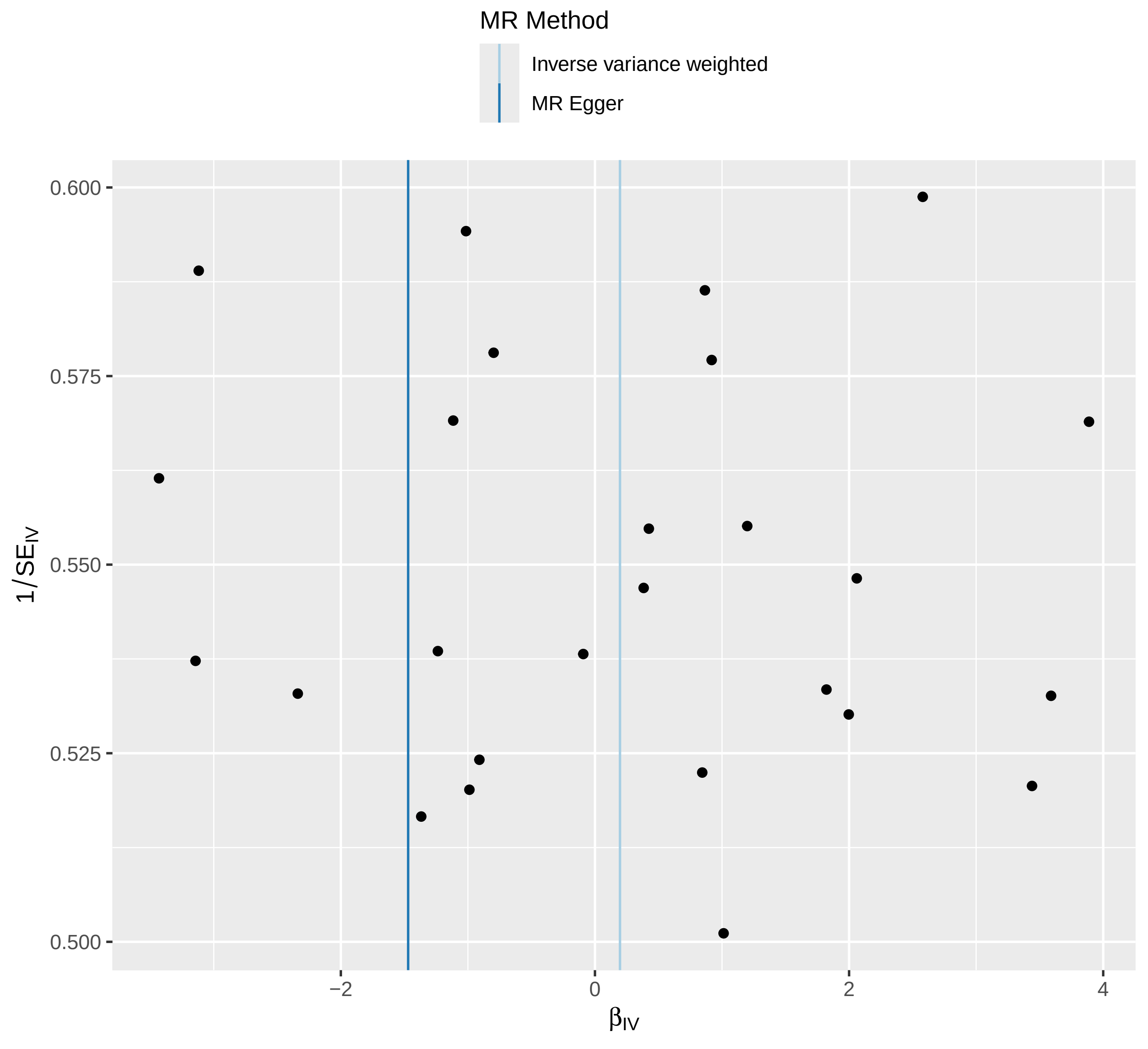
MR Method
Inverse variance weighted
MR Egger
0.600
0.575
0.550
0.525
0.500
$1/SE_{IV}$
−2
0
2
4
β_{IV}

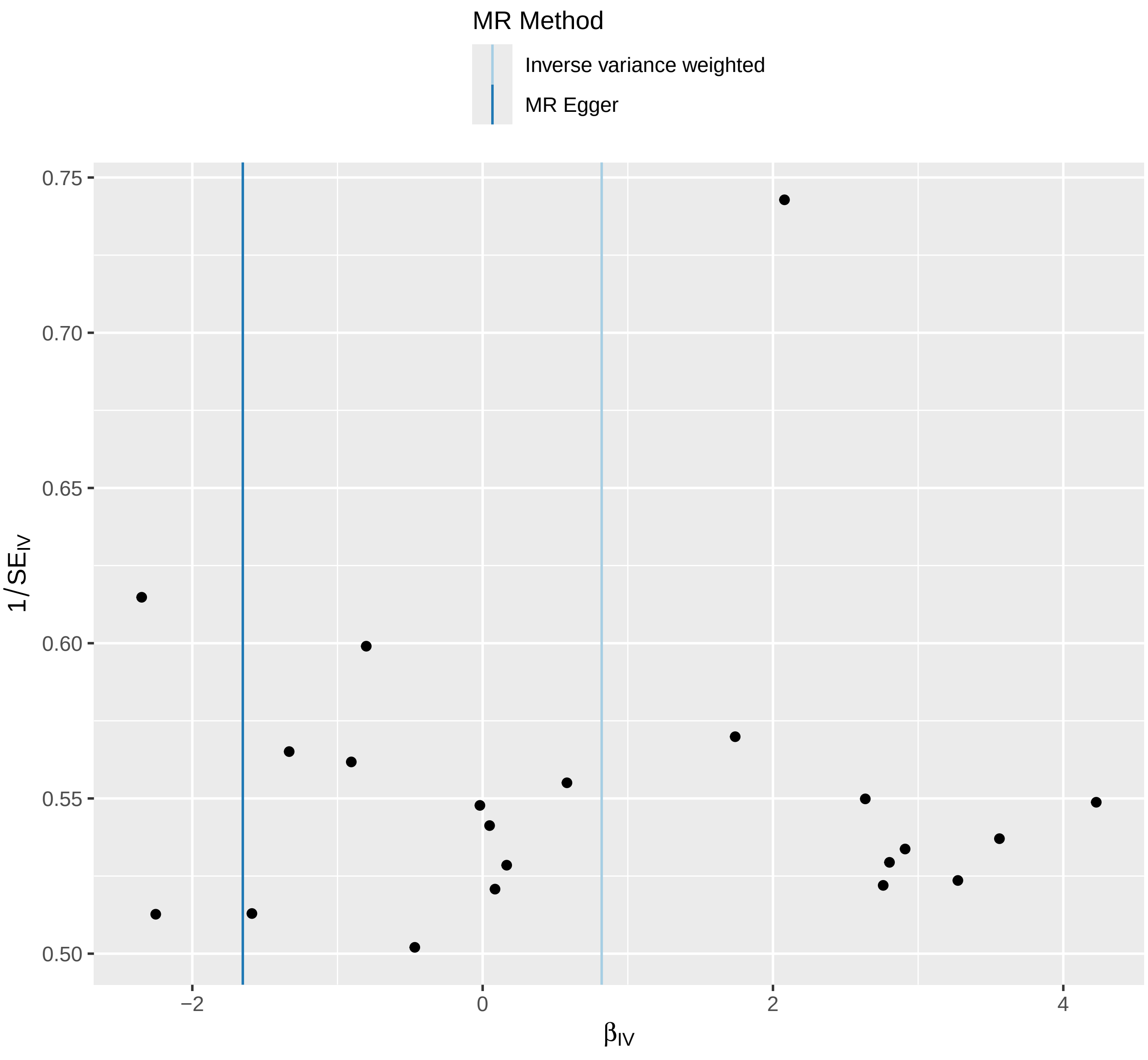

MR Method
Inverse variance weighted
MR Egger
0.75
0.70
0.65
0.60
0.55
0.50
1/SE_IV
−2
0
2
4
β_IV

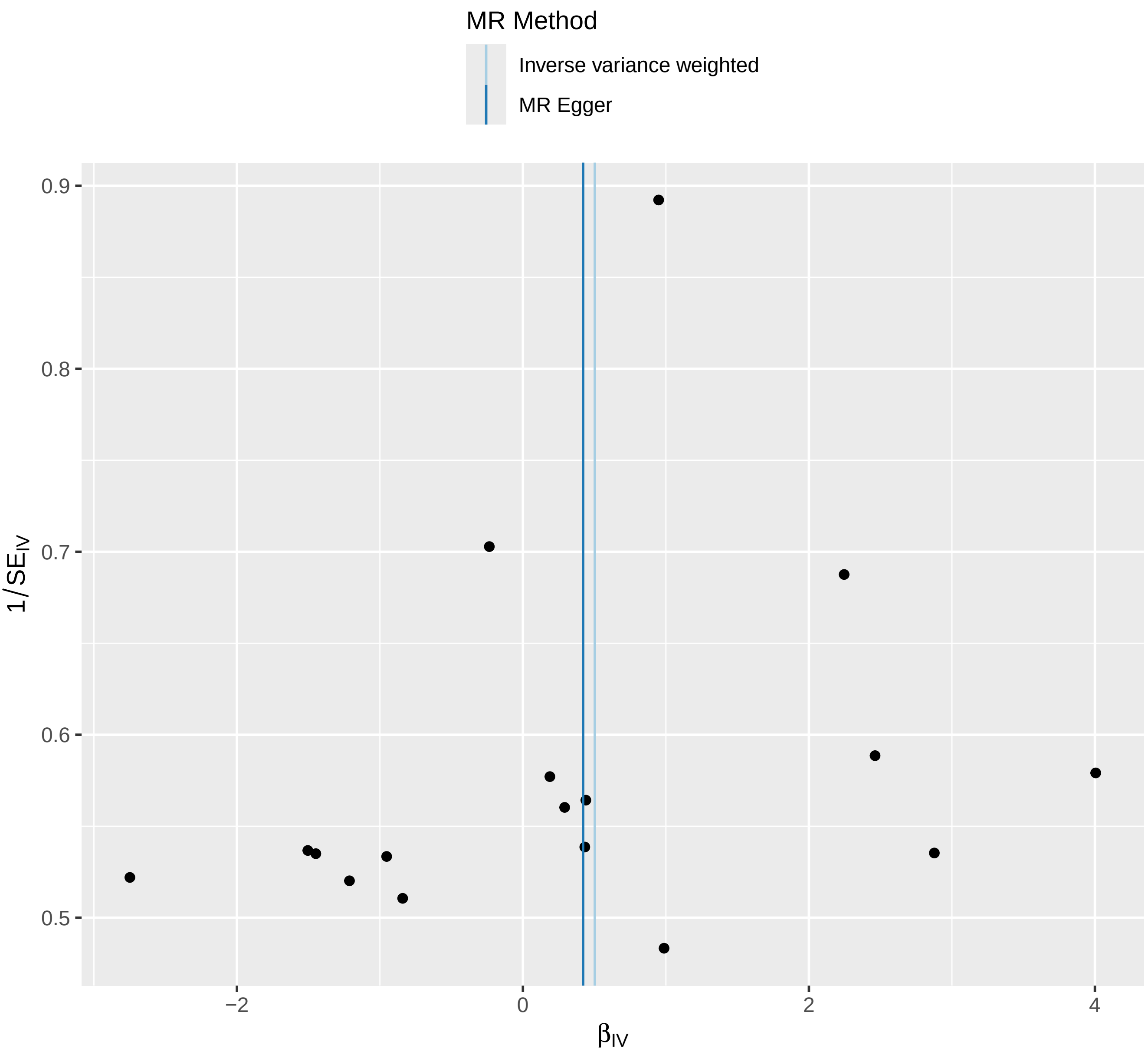
MR Method
Inverse variance weighted
MR Egger
0.9
0.8
0.7
0.6
0.5
1/SE_IV
−2
0
2
4
β_IV

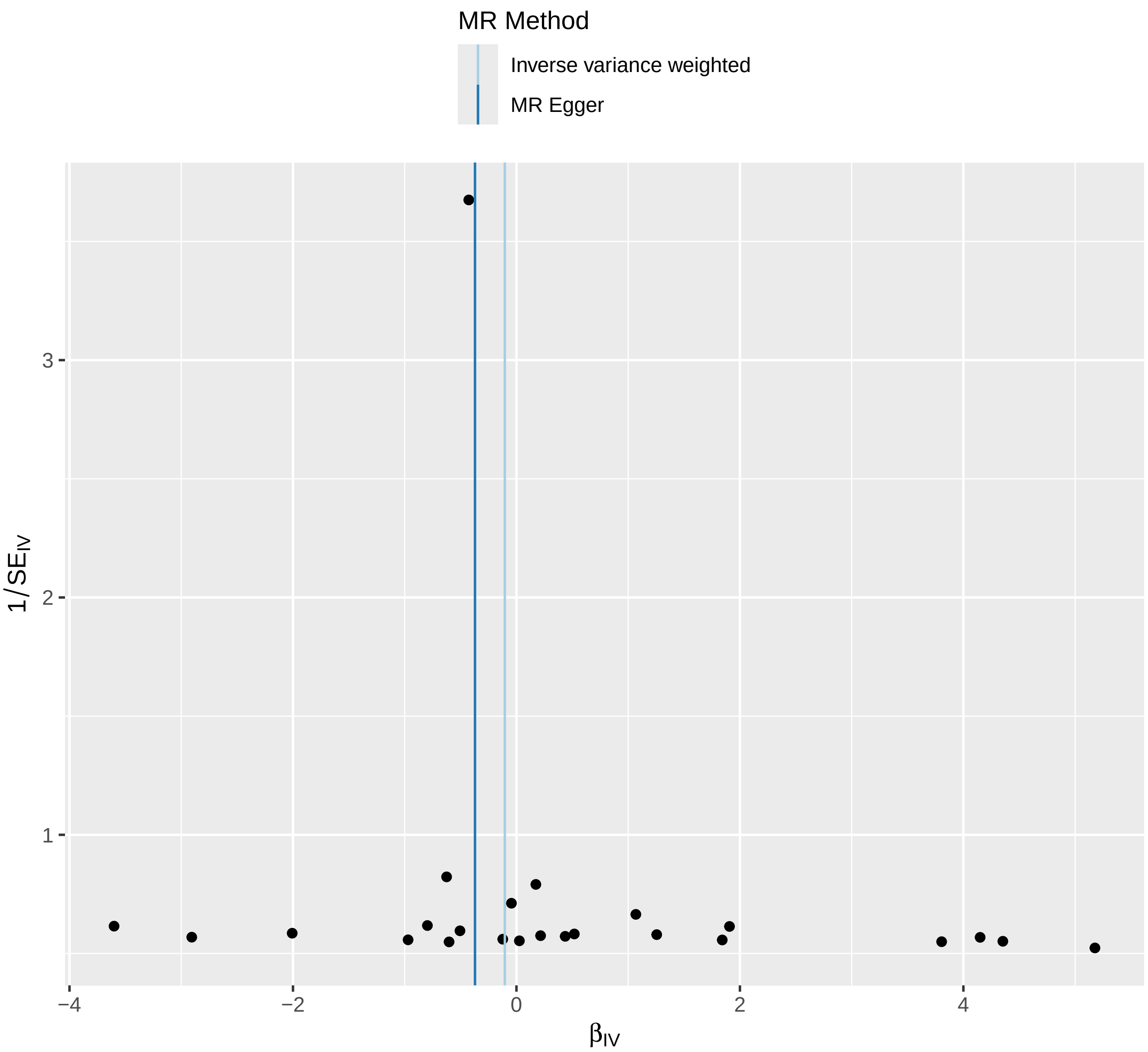

MR Method
Inverse variance weighted
MR Egger
3
2
1
1/SE_IV
−4
−2
0
2
4
β_IV

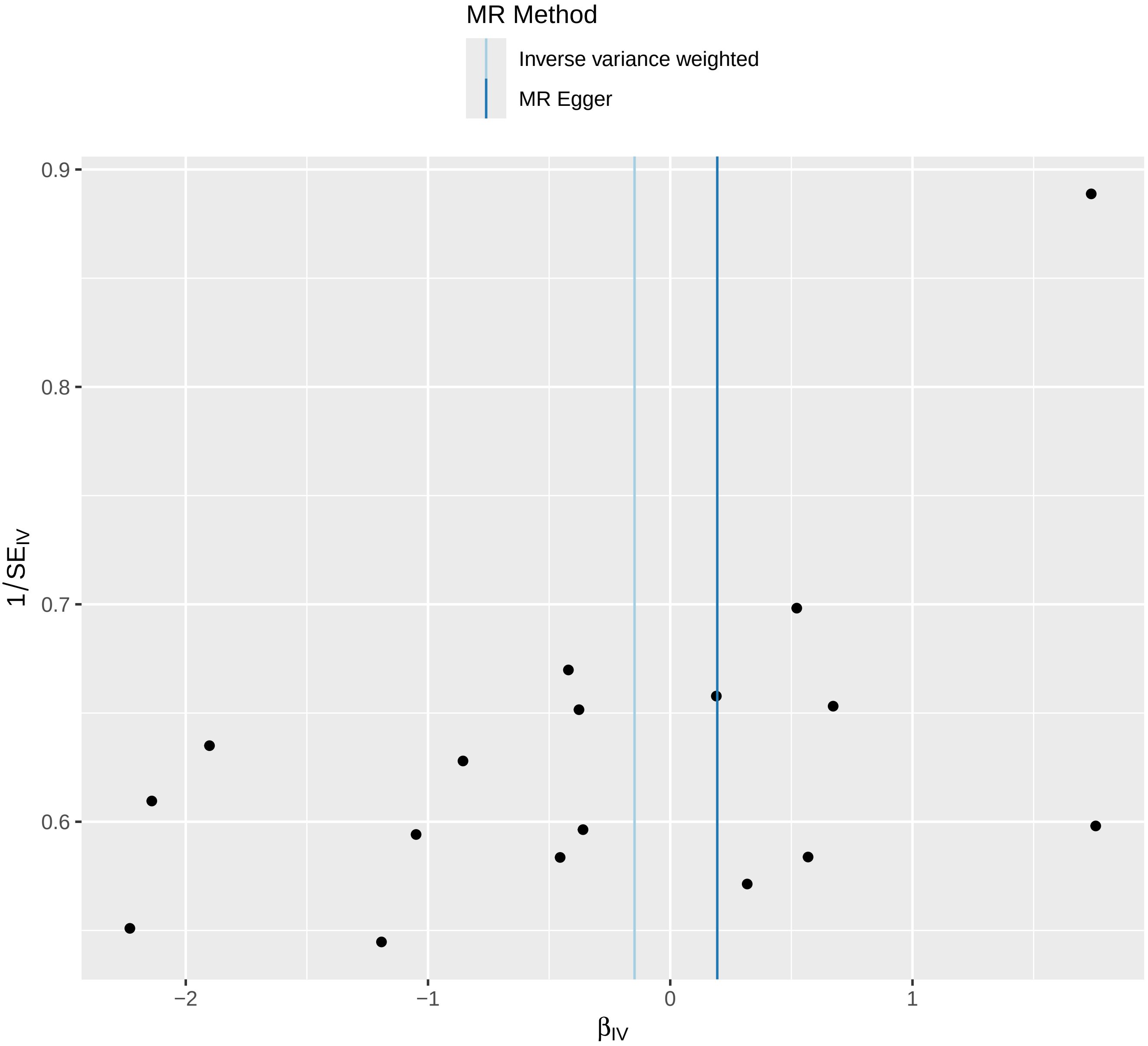
MR Method
Inverse variance weighted
MR Egger
0.9
0.8
0.7
0.6
1/SE$_{IV}$
−2
−1
0
1
β$_{IV}$

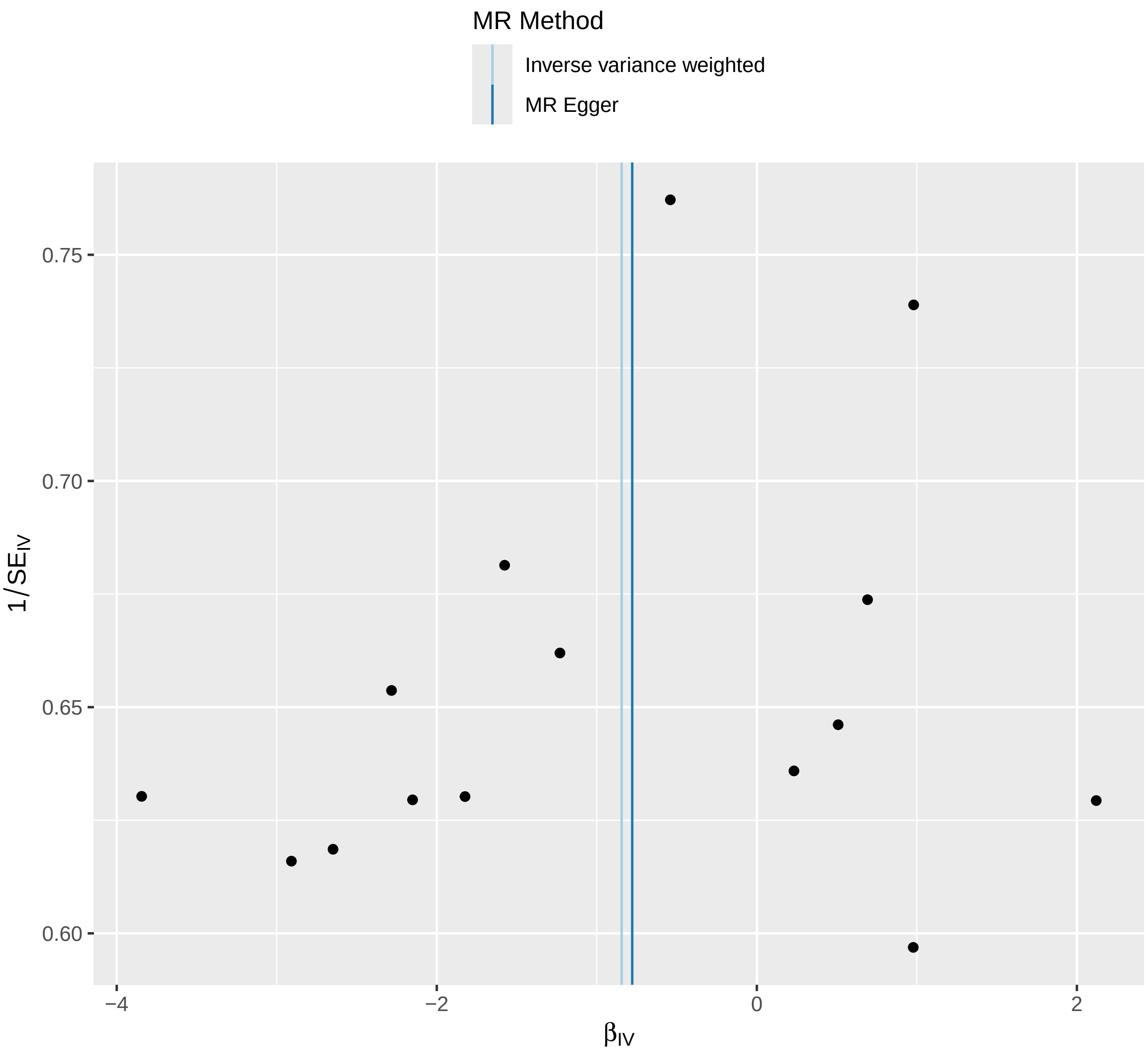
MR Method
Inverse variance weighted
MR Egger
0.75
0.70
0.65
0.60
1/SE_IV
−4
−2
0
2
β_IV

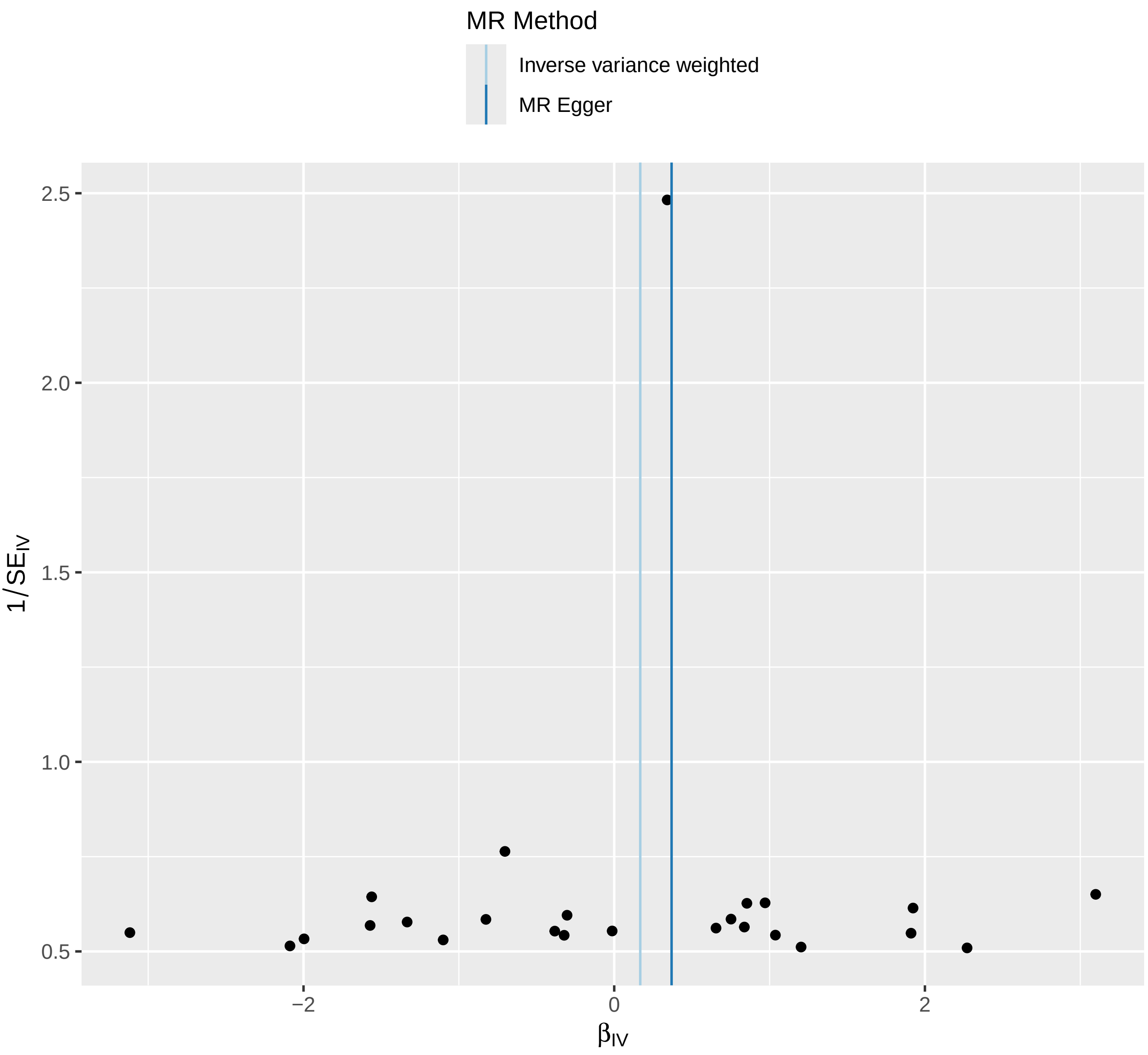

MR Method
Inverse variance weighted
MR Egger
2.5
2.0
1.5
1.0
0.5
1/SE$_{IV}$
−2
0
2
β$_{IV}$

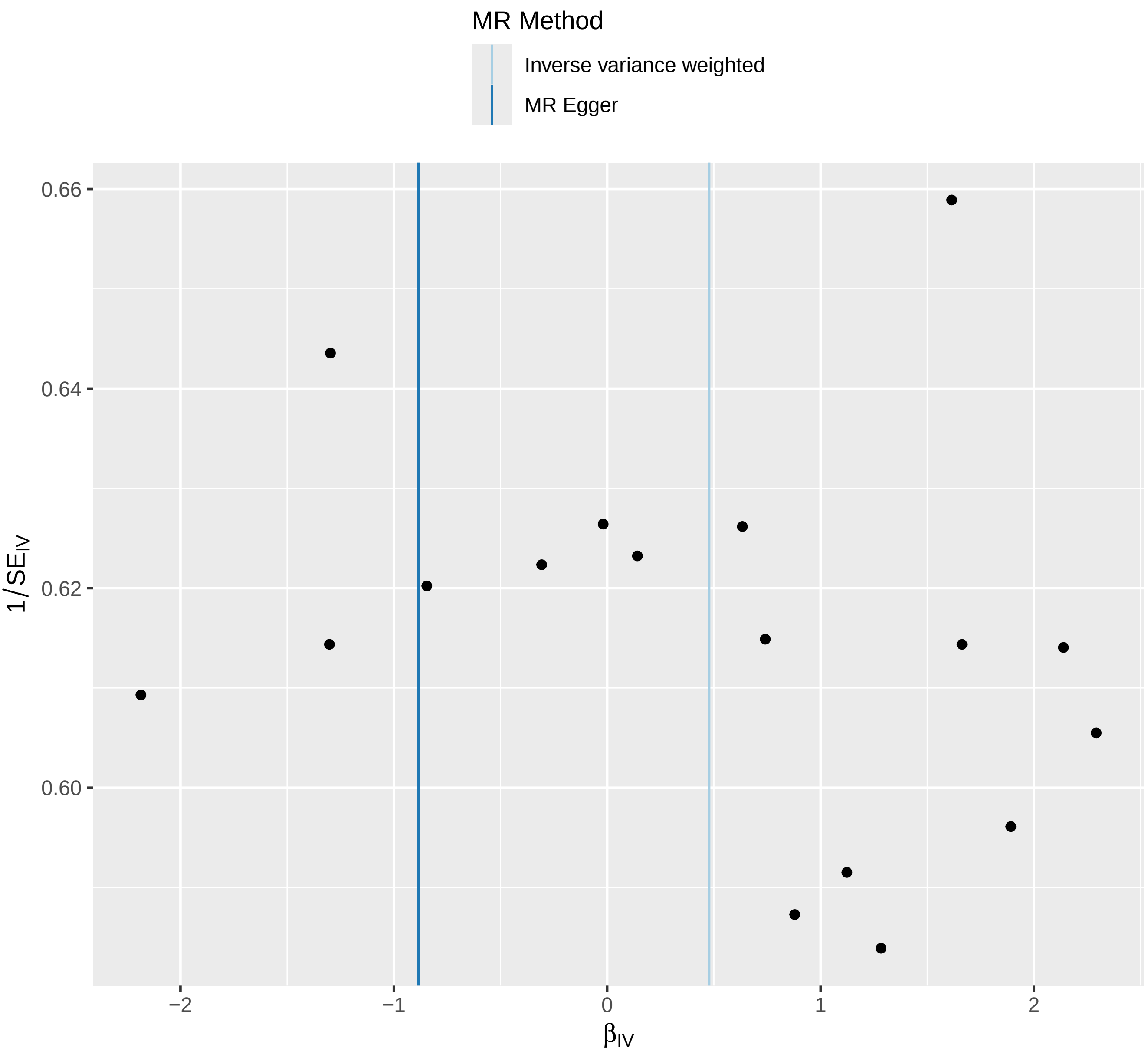
MR Method
Inverse variance weighted
MR Egger
0.66
0.64
0.62
0.60
1/SE_IV
−2
−1
0
1
2
β_IV

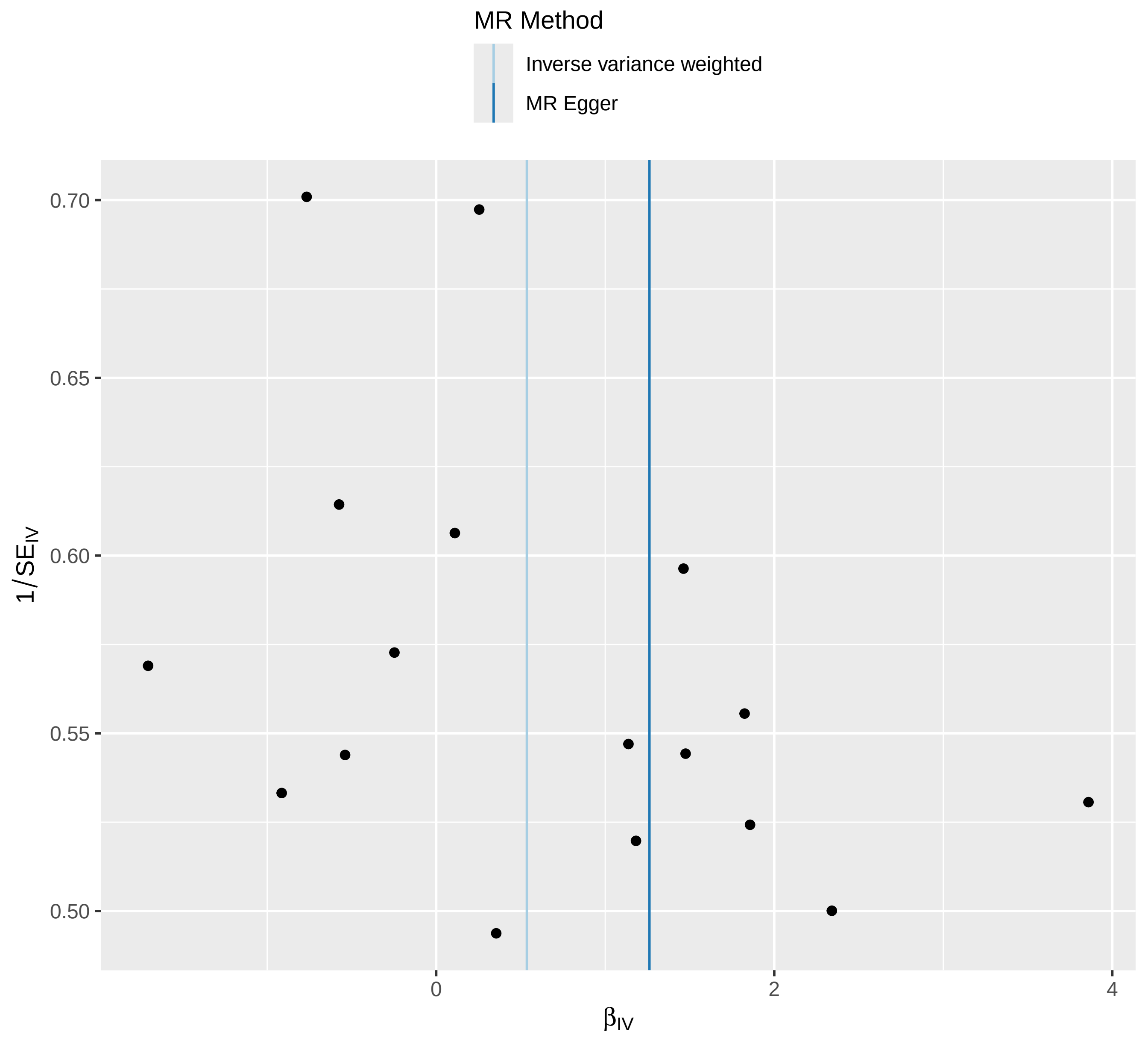
MR Method
Inverse variance weighted
MR Egger
0.70
0.65
0.60
0.55
0.50
1/SE_IV
0
2
4
β_IV

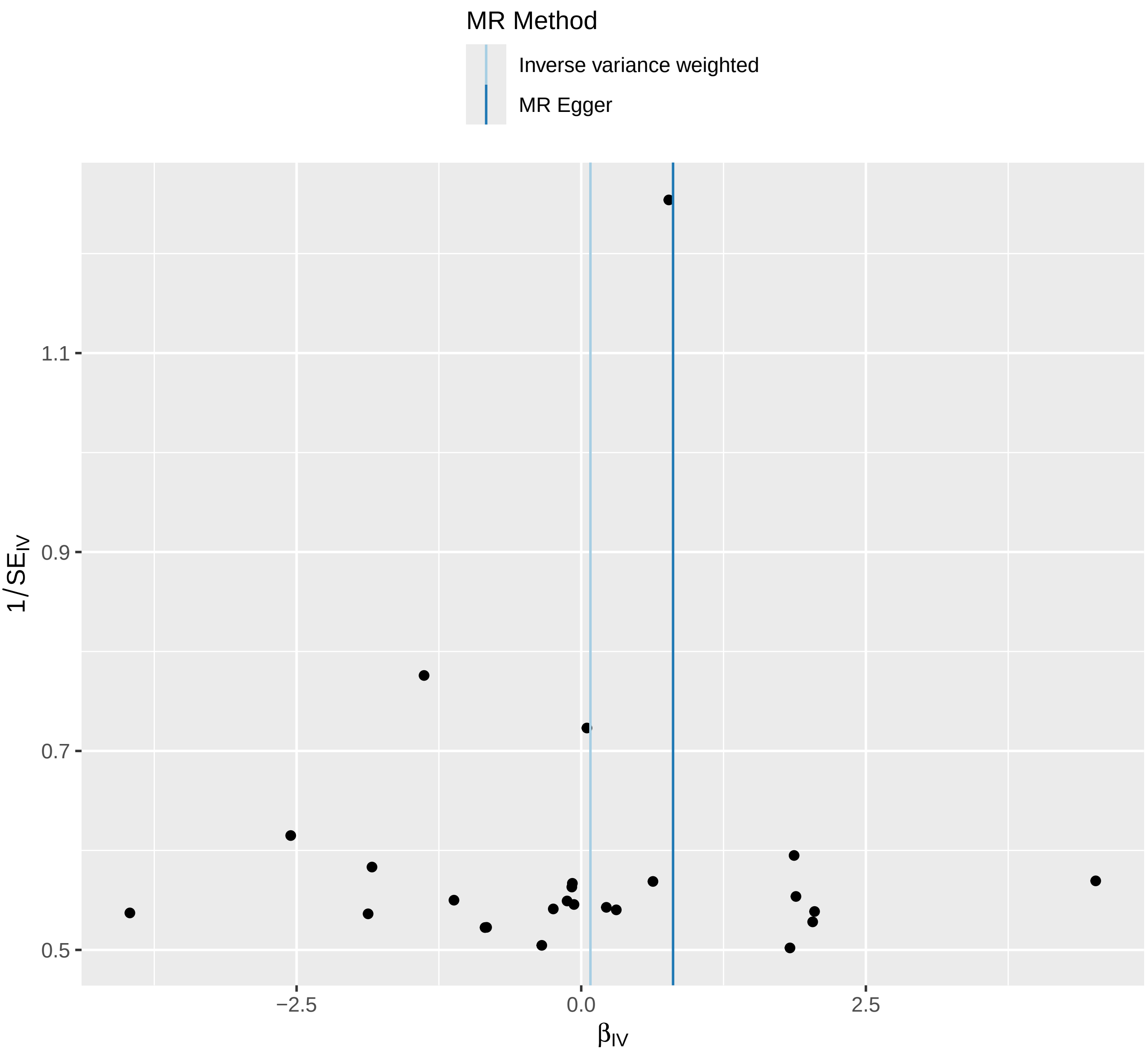
MR Method
Inverse variance weighted
MR Egger
1.1
0.9
0.7
0.5
1/SE$_{IV}$
−2.5
0.0
2.5
β$_{IV}$

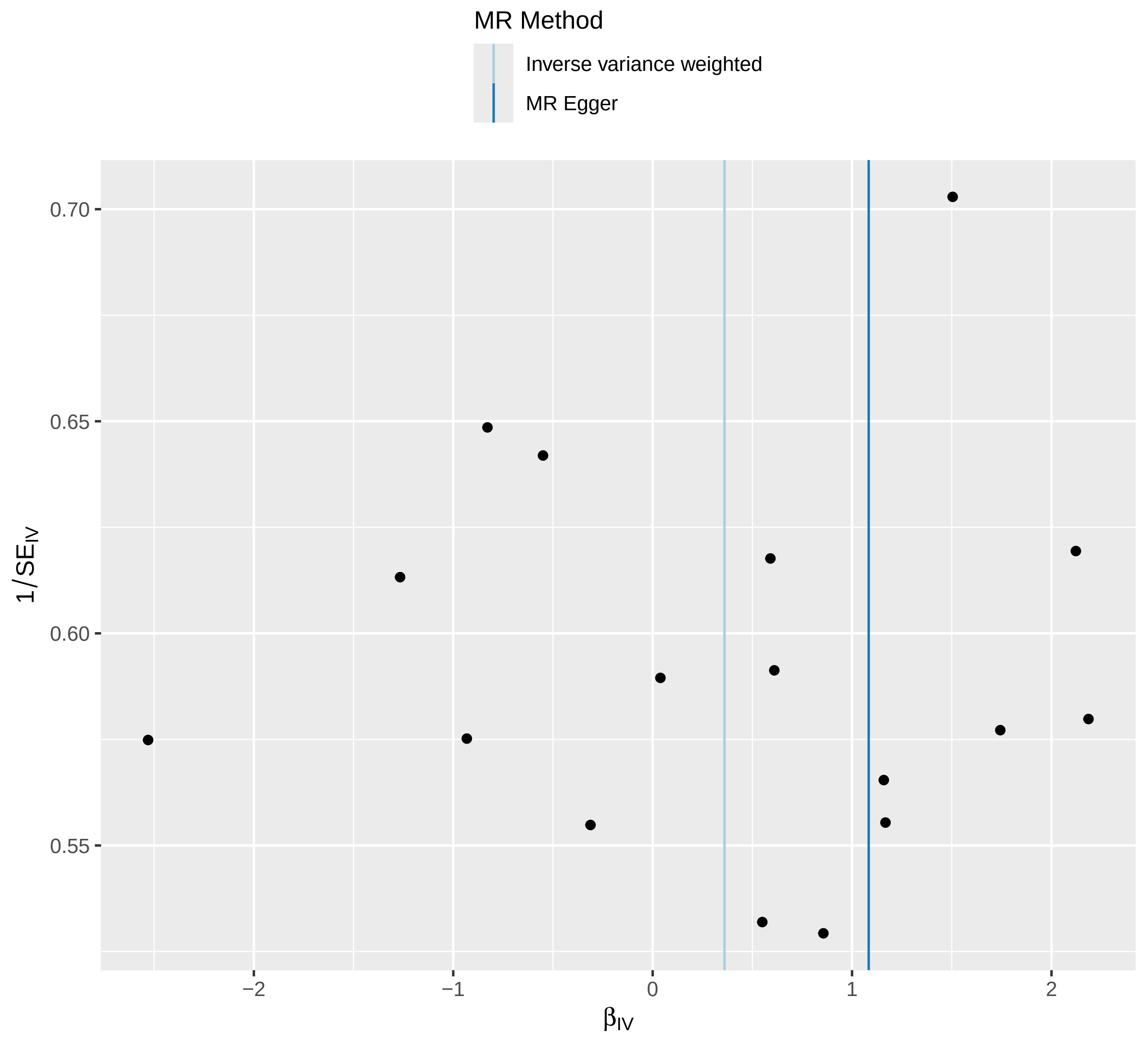
MR Method
Inverse variance weighted
MR Egger
0.70
0.65
0.60
0.55
$1/SE_{IV}$
−2
−1
0
1
2
β_{IV}

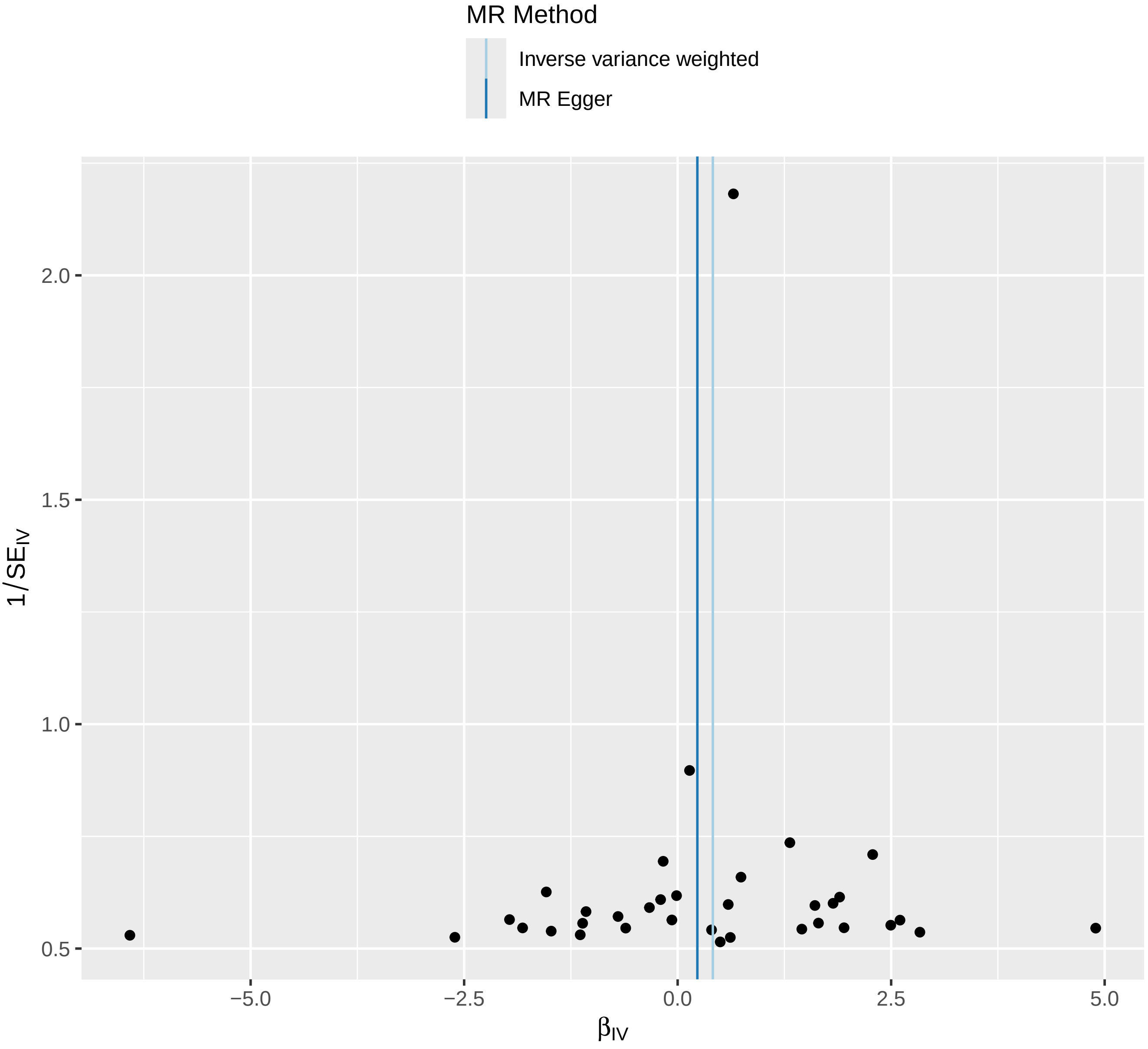

MR Method
Inverse variance weighted
MR Egger
2.0
1.5
1.0
0.5
1/SE_IV
−5.0
−2.5
0.0
2.5
5.0
β_IV

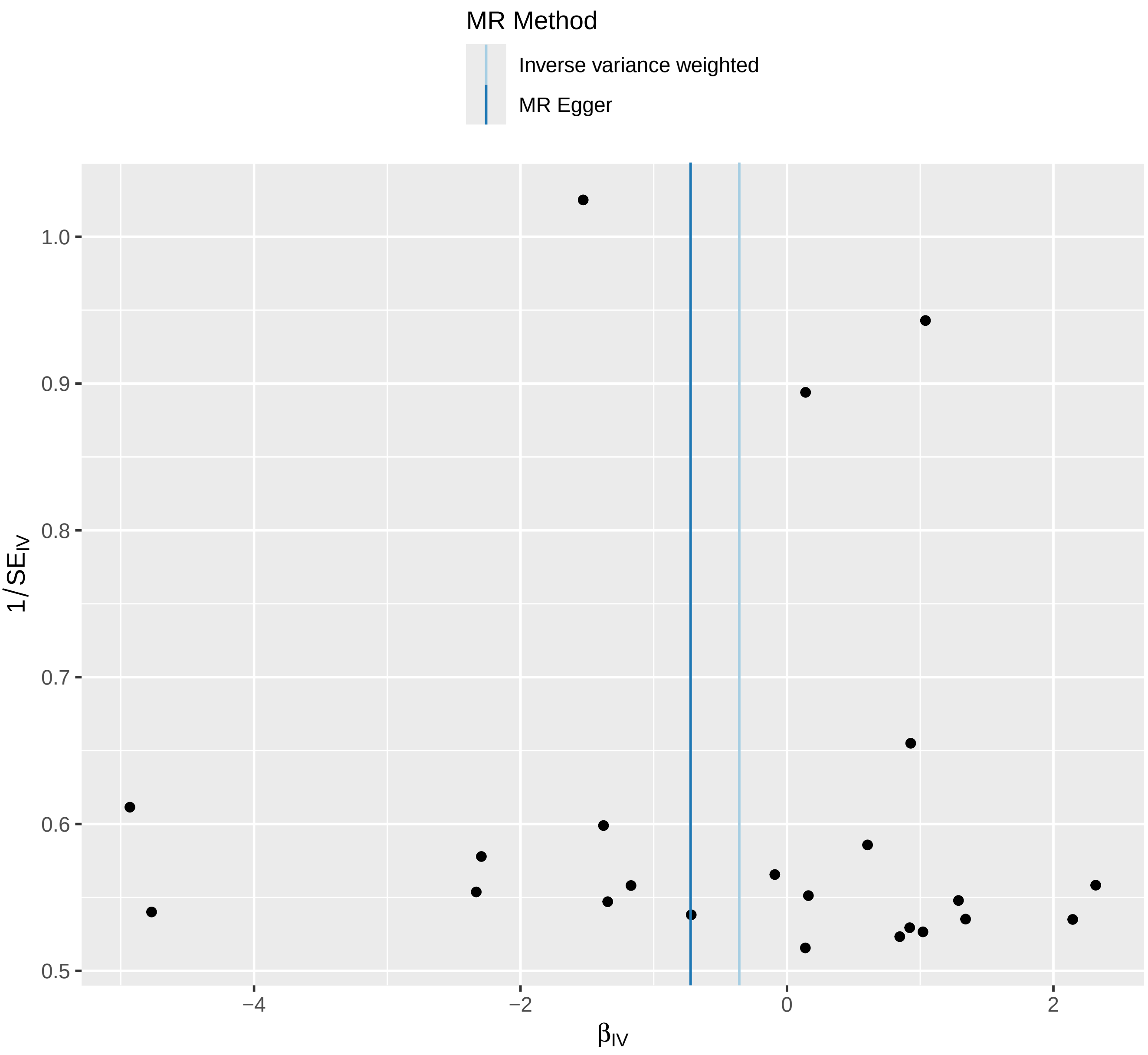
MR Method
Inverse variance weighted
MR Egger
1/SE$_{IV}$
1.0
0.9
0.8
0.7
0.6
0.5
−4
−2
0
2
β$_{IV}$

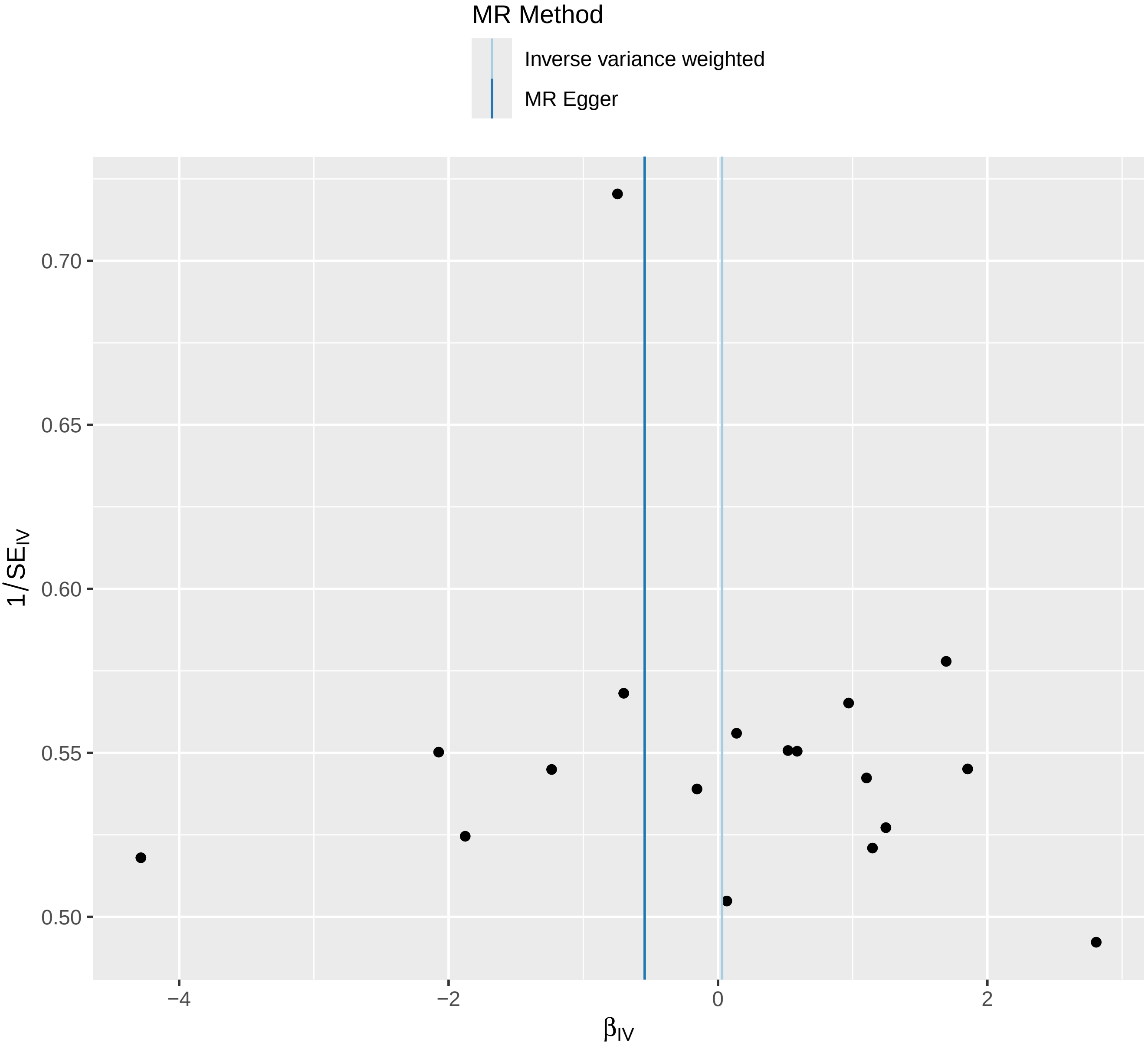
MR Method
Inverse variance weighted
MR Egger
0.70
0.65
0.60
0.55
0.50
1/SE_IV
−4
−2
0
2
β_IV

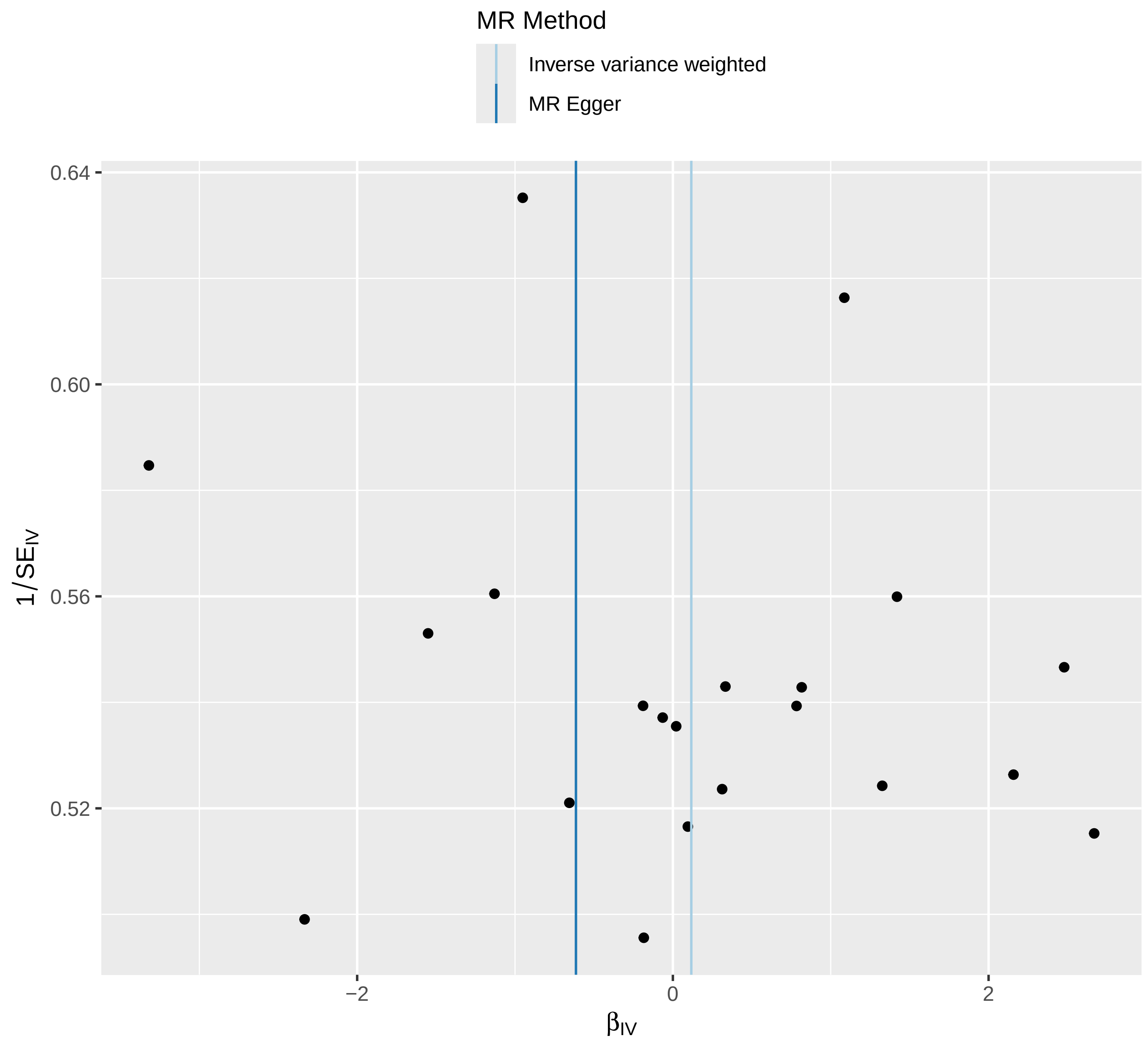

MR Method
Inverse variance weighted
MR Egger
0.64
0.60
0.56
0.52
1/SE_IV
−2
0
2
β_IV

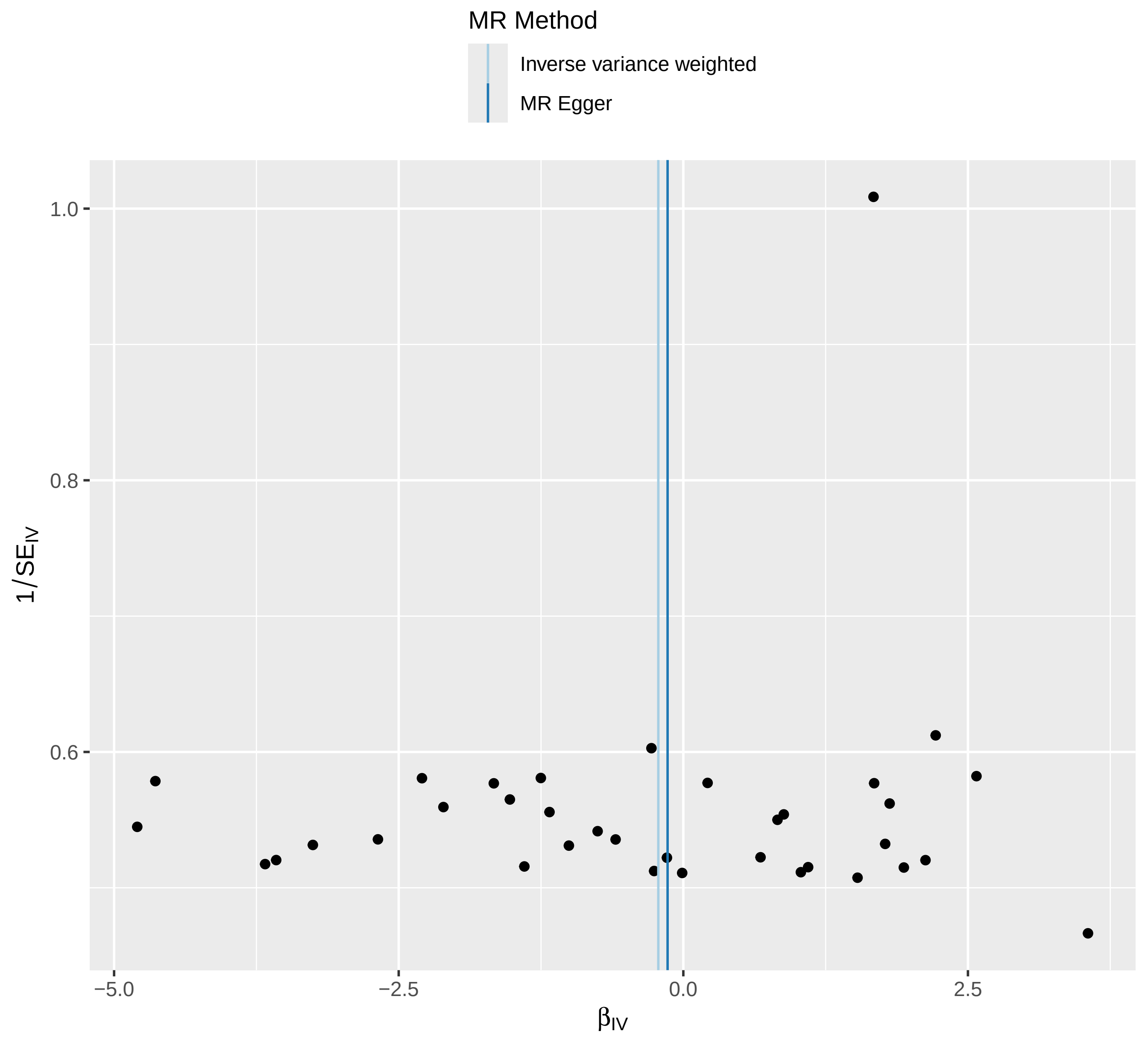

MR Method
Inverse variance weighted
MR Egger
1.0
0.8
0.6
1/SE_IV
−5.0
−2.5
0.0
2.5
β_IV

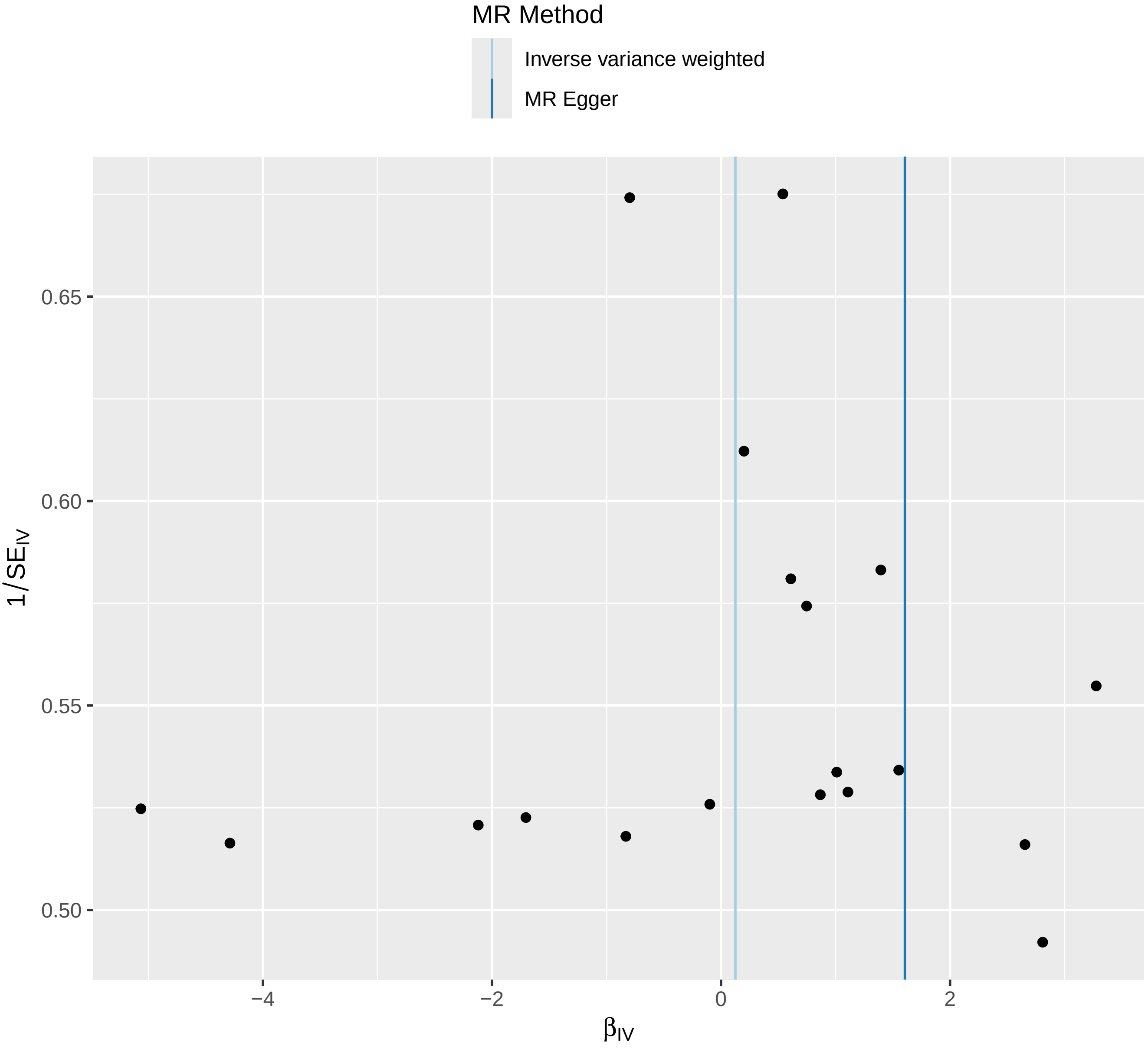
MR Method
Inverse variance weighted
MR Egger
0.65
0.60
0.55
0.50
1/SE_IV
−4
−2
0
2
β_IV

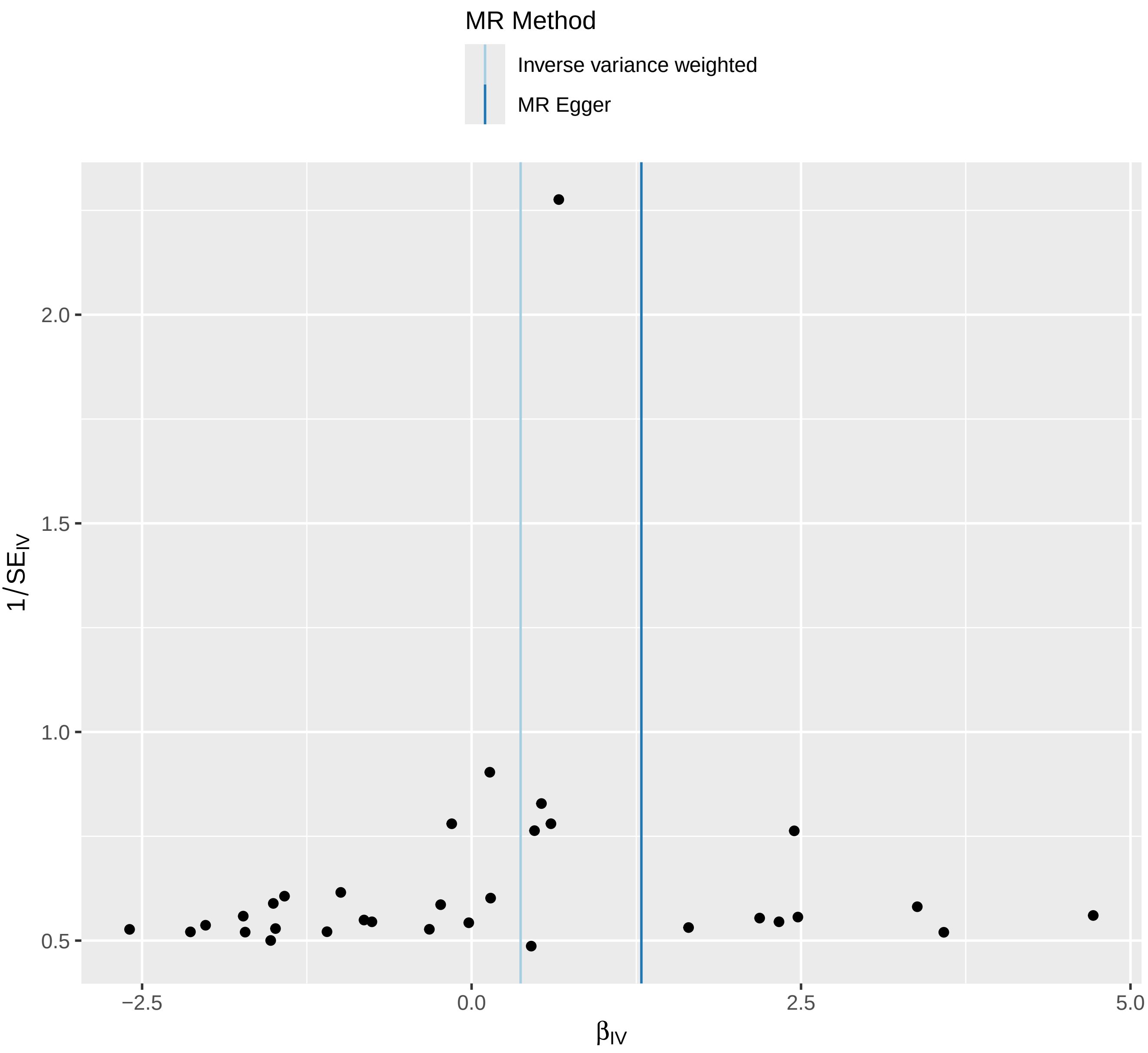
MR Method
Inverse variance weighted
MR Egger
1/SE_{IV}
β_{IV}
2.0
1.5
1.0
0.5
−2.5
0.0
2.5
5.0

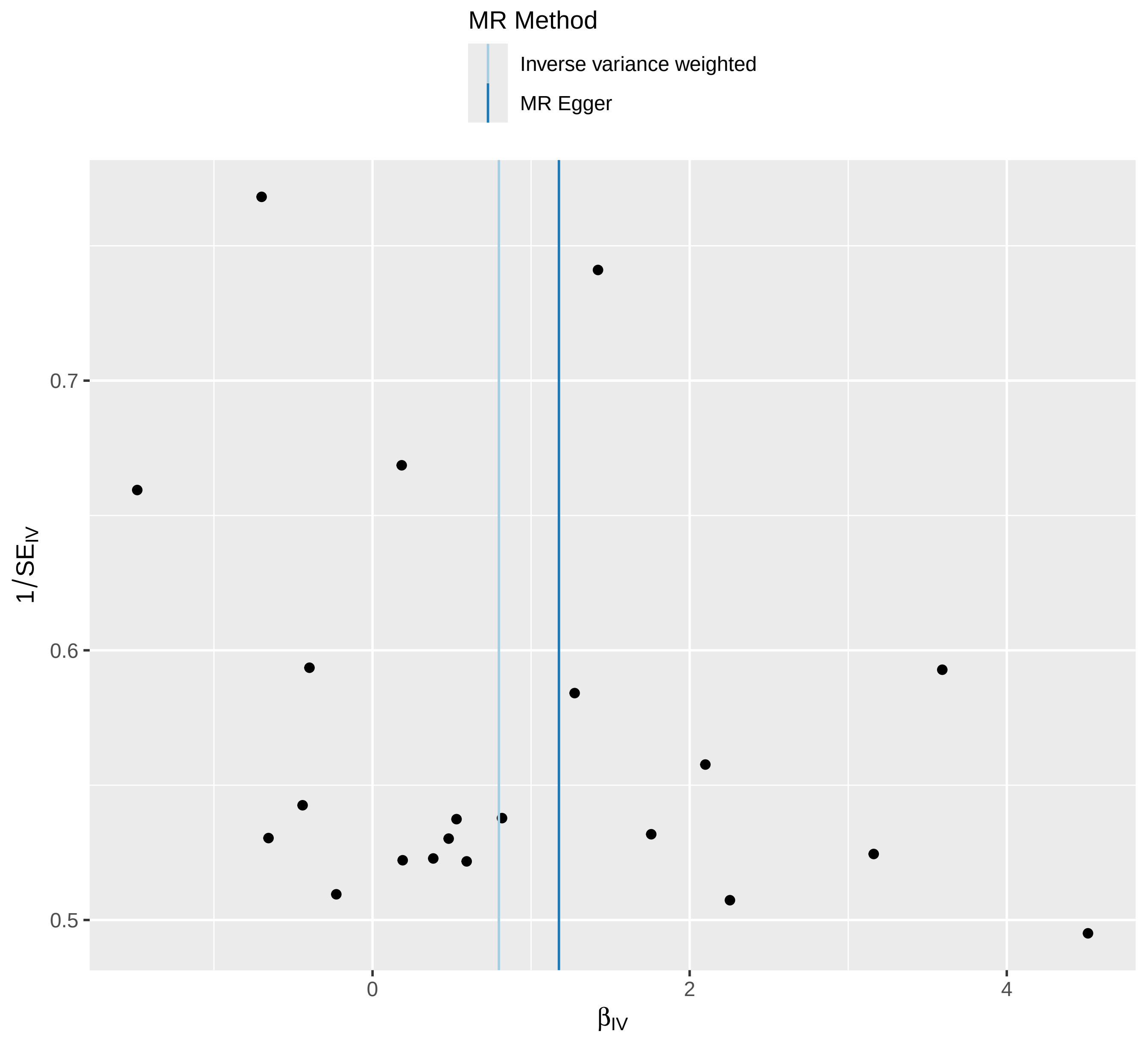
MR Method
Inverse variance weighted
MR Egger
0.7
0.6
0.5
1/SE_IV
0
2
4
β_IV

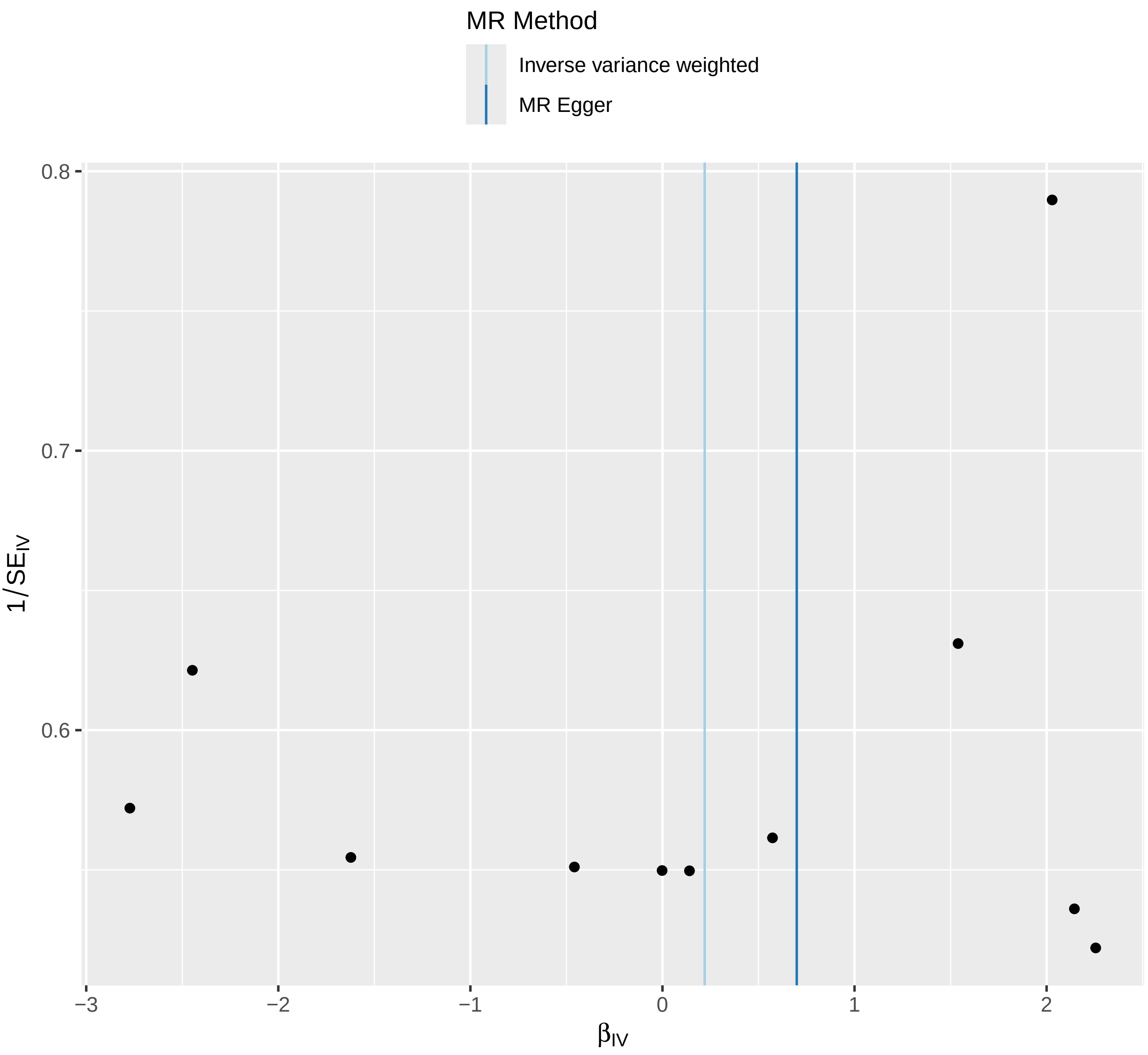
MR Method
Inverse variance weighted
MR Egger
0.8
0.7
0.6
1/SE$_{IV}$
−3
−2
−1
0
1
2
β_{IV}

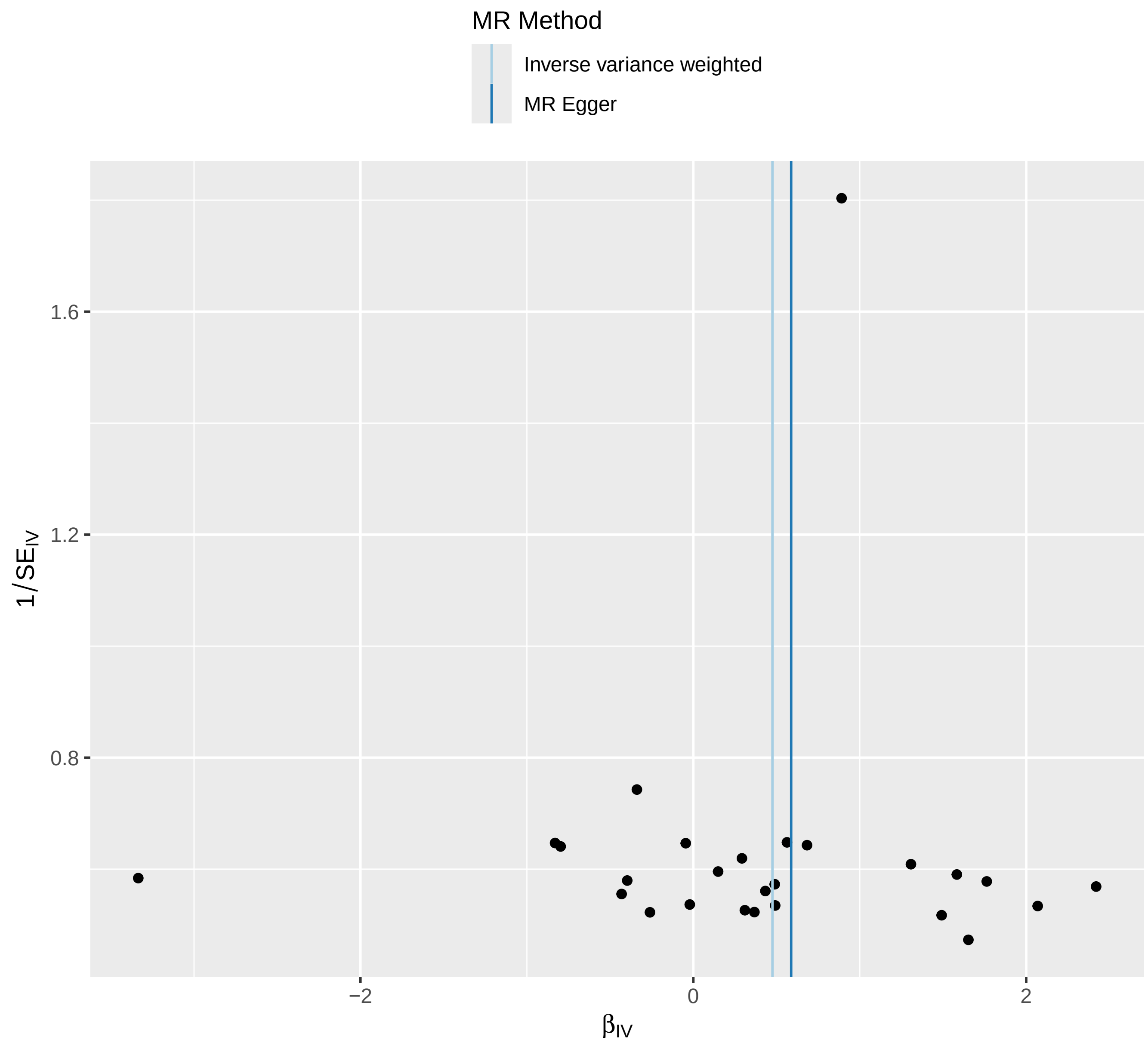
MR Method
Inverse variance weighted
MR Egger
1.6
1.2
0.8
1/SE_IV
−2
0
2
β_IV

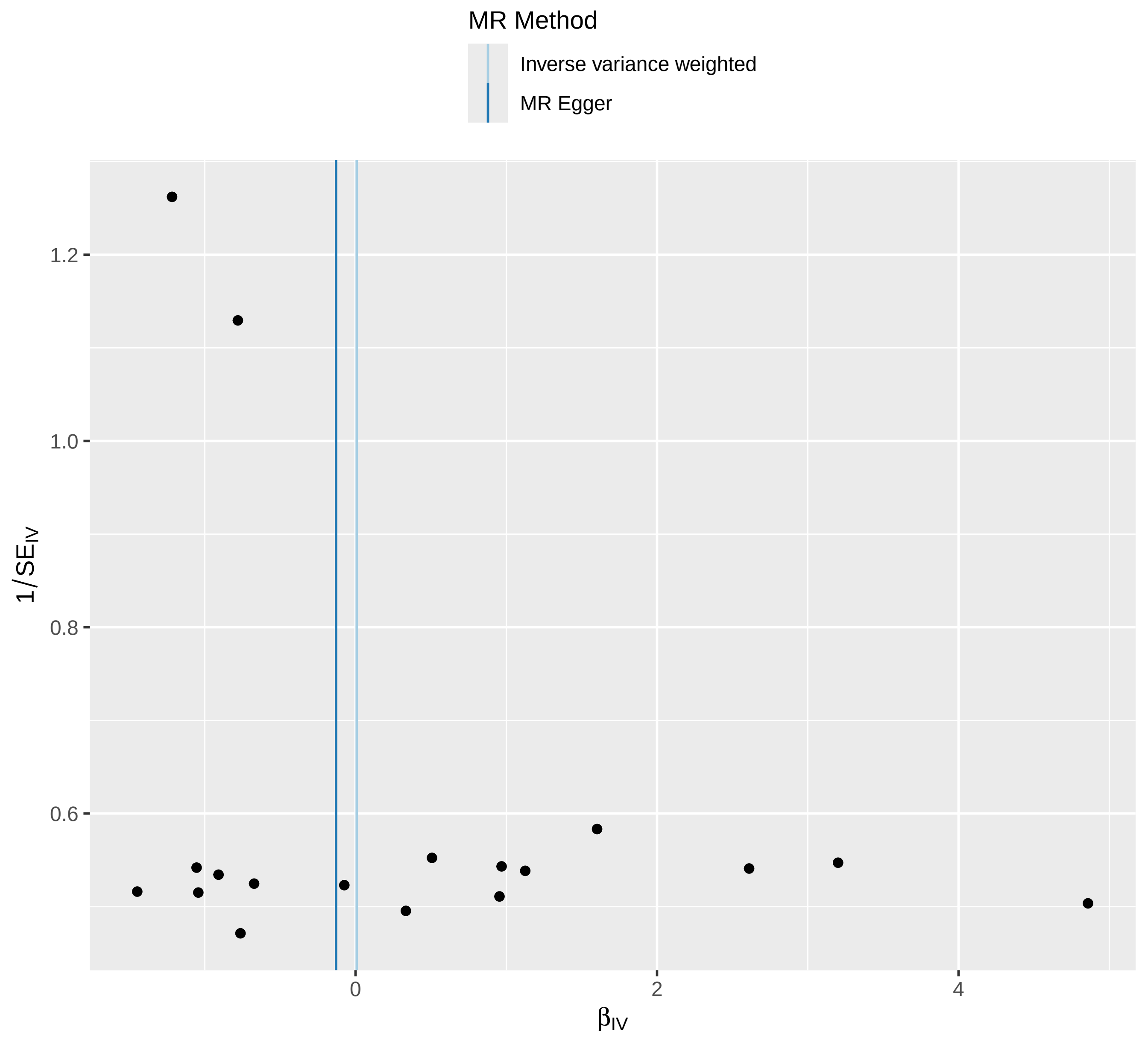
MR Method
Inverse variance weighted
MR Egger
1.2
1.0
0.8
0.6
1/SE_IV
0
2
4
β_IV

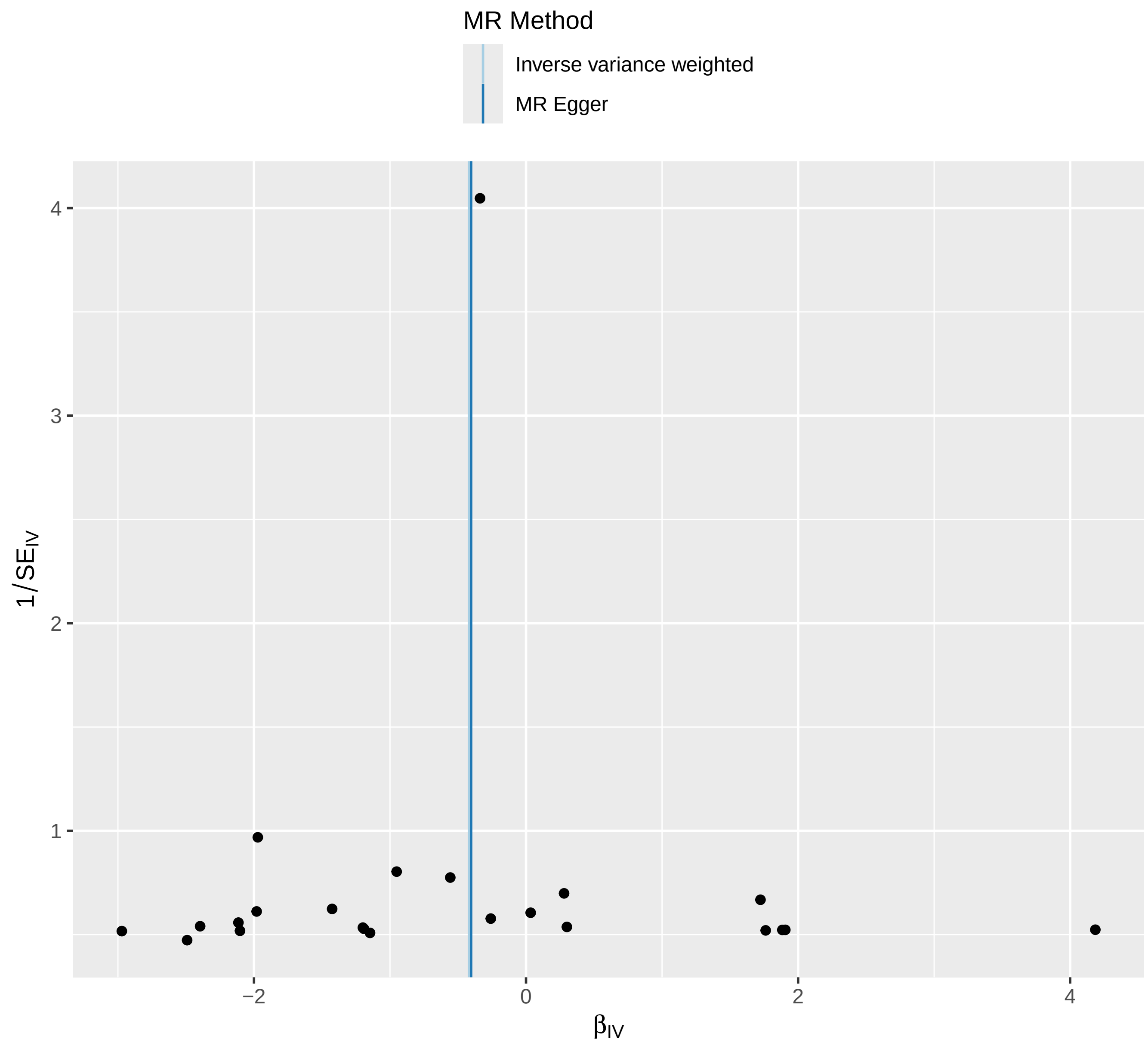
MR Method
Inverse variance weighted
MR Egger
4
3
2
1
1/SE$_{IV}$
−2
0
2
4
β$_{IV}$

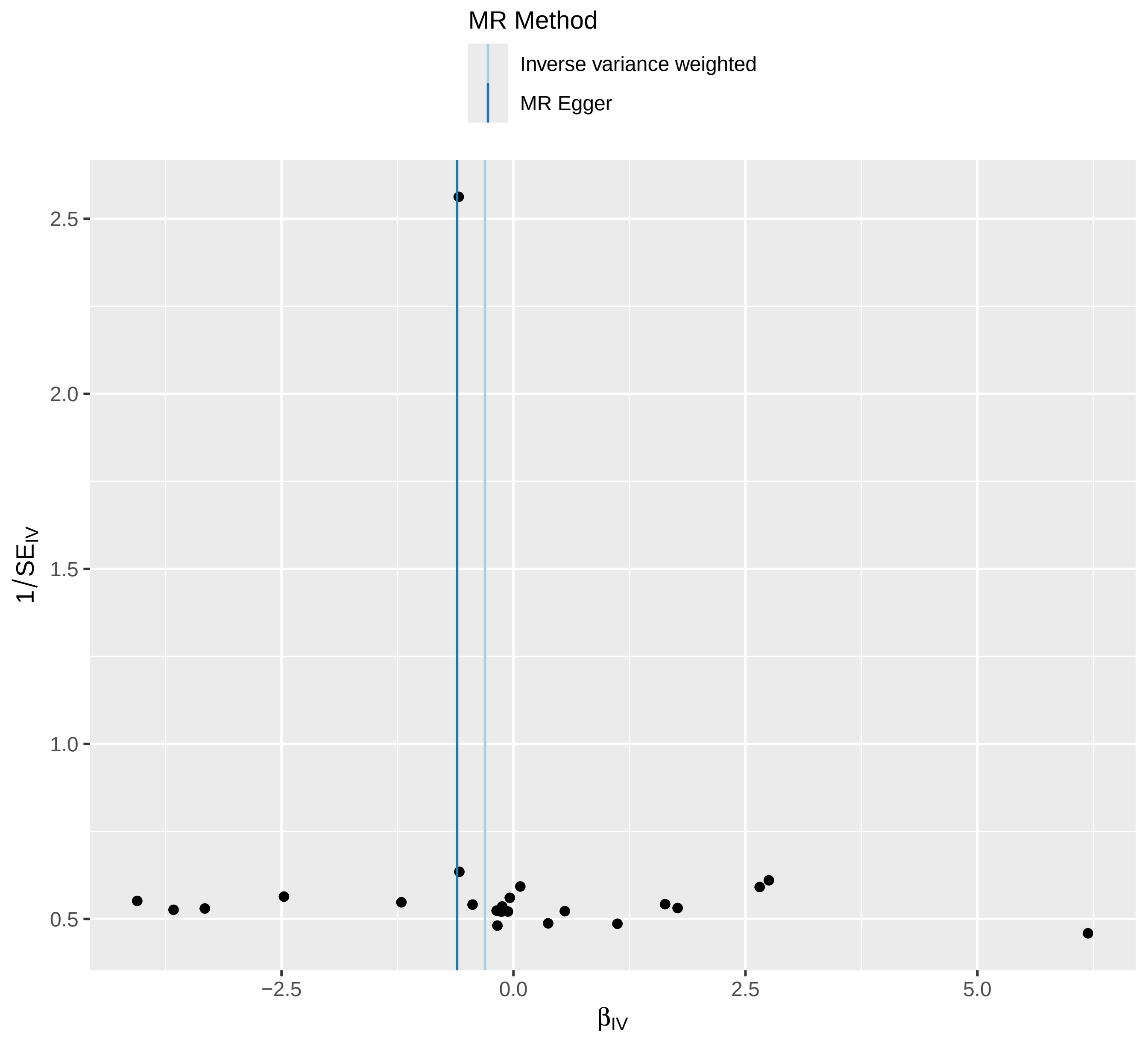

MR Method
Inverse variance weighted
MR Egger
2.5
2.0
1.5
1.0
0.5
−2.5
0.0
2.5
5.0
$1/SE_{IV}$
β_{IV}

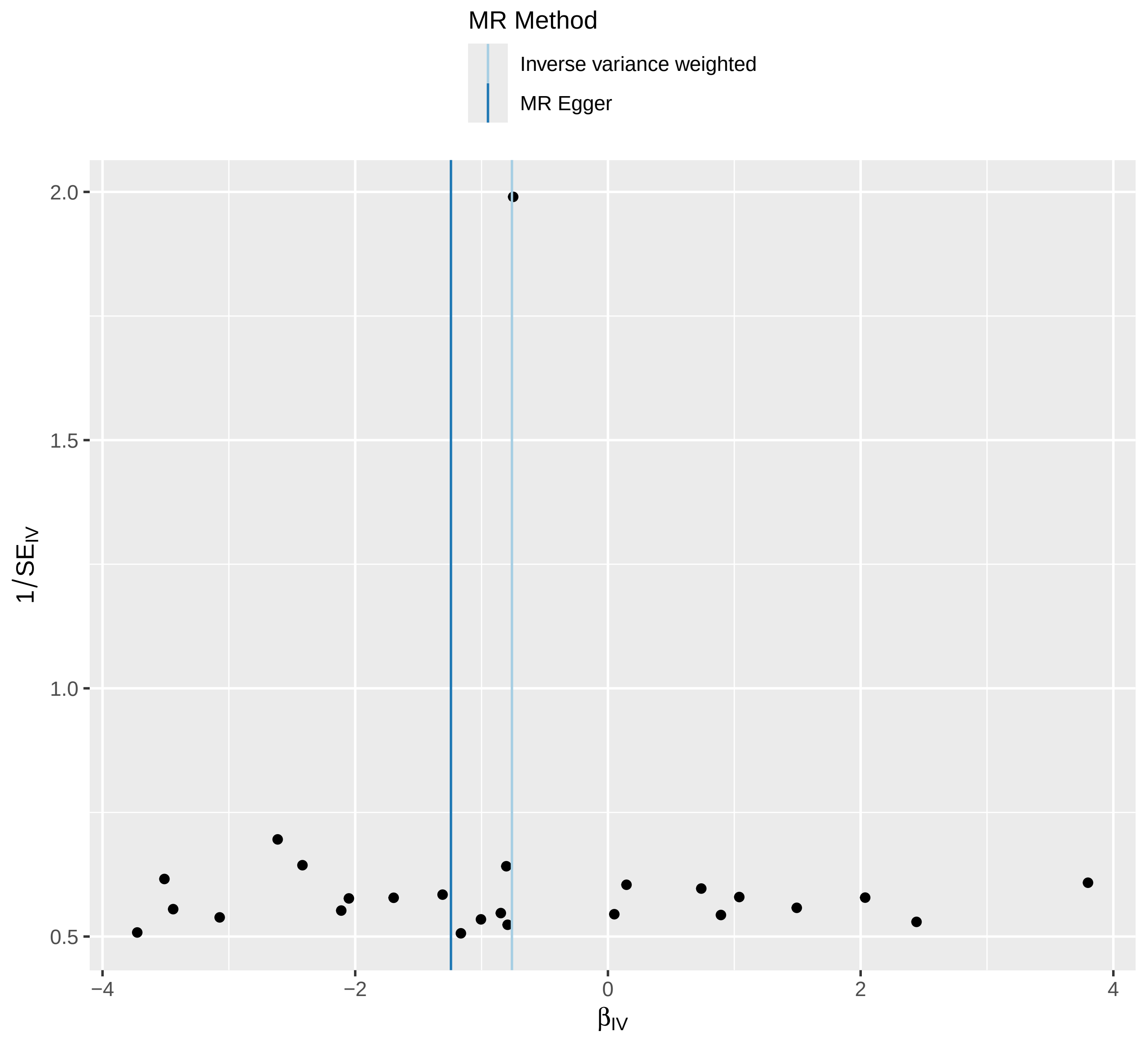

MR Method
Inverse variance weighted
MR Egger
2.0
1.5
1.0
0.5
$1/SE_{IV}$
−4
−2
0
2
4
β_{IV}

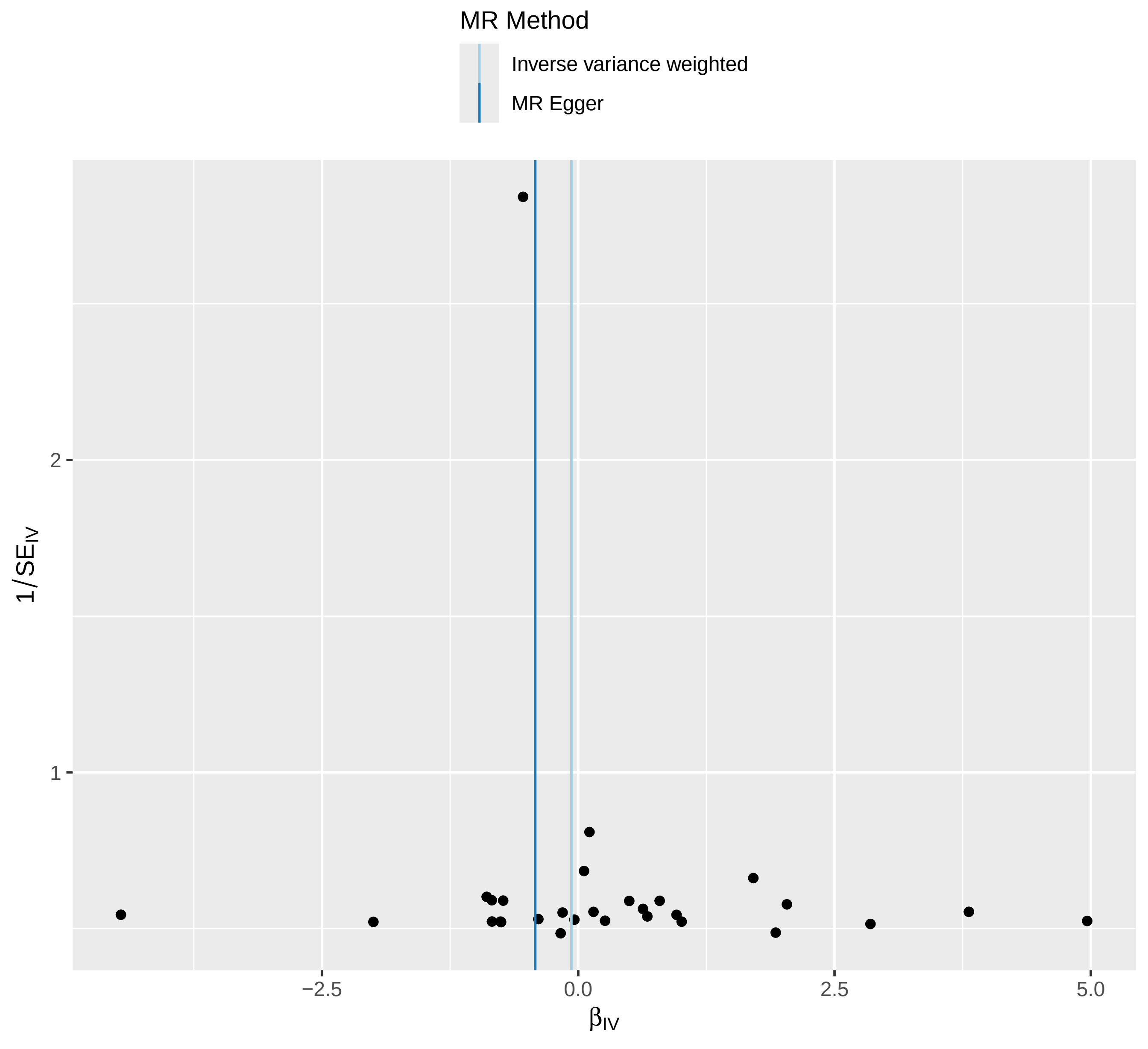

MR Method
Inverse variance weighted
MR Egger
2
1
1/SE_IV
−2.5
0.0
2.5
5.0
β_IV

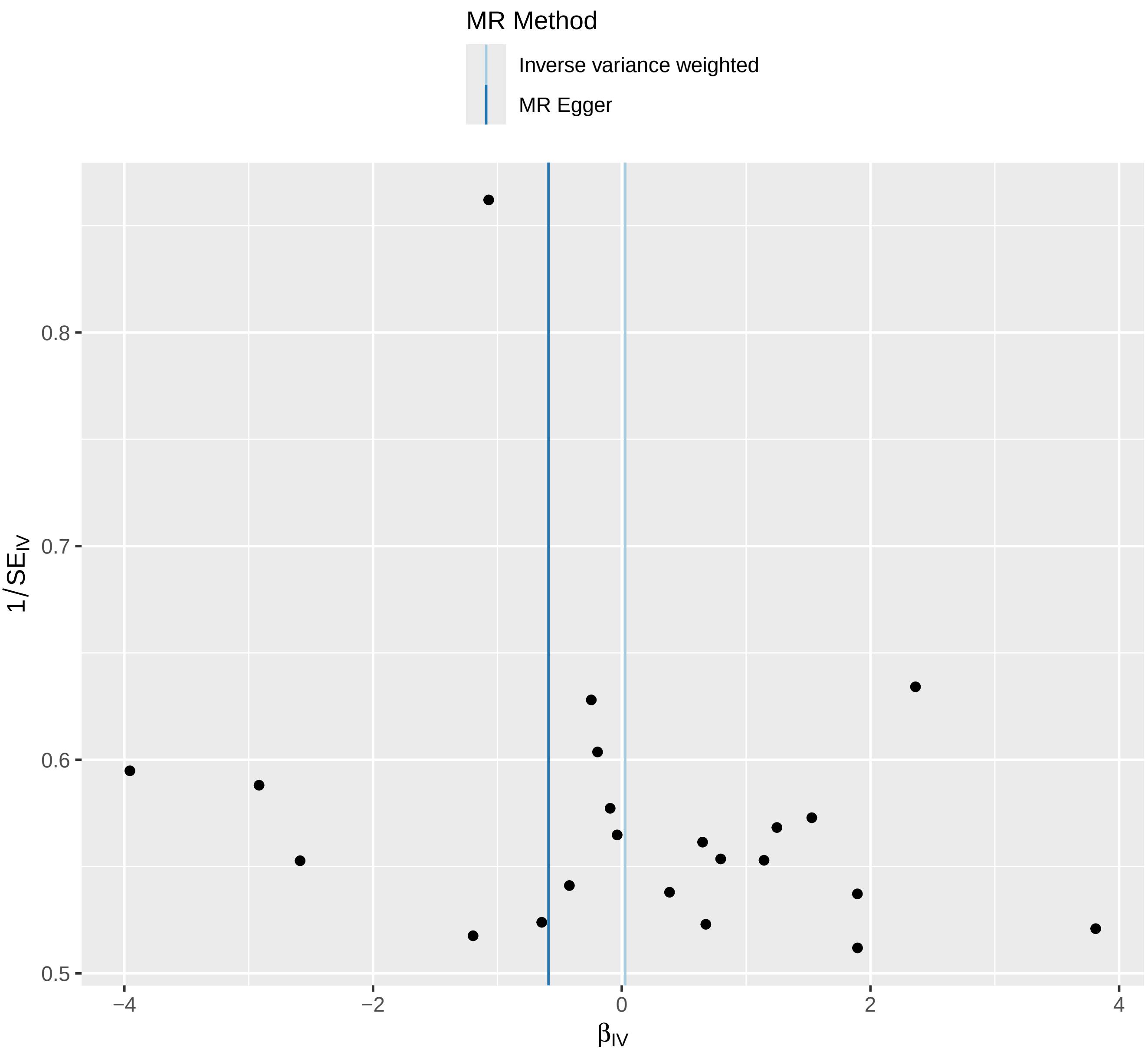
MR Method
Inverse variance weighted
MR Egger
0.8
0.7
0.6
0.5
$1/SE_{IV}$
−4
−2
0
2
4
β_{IV}

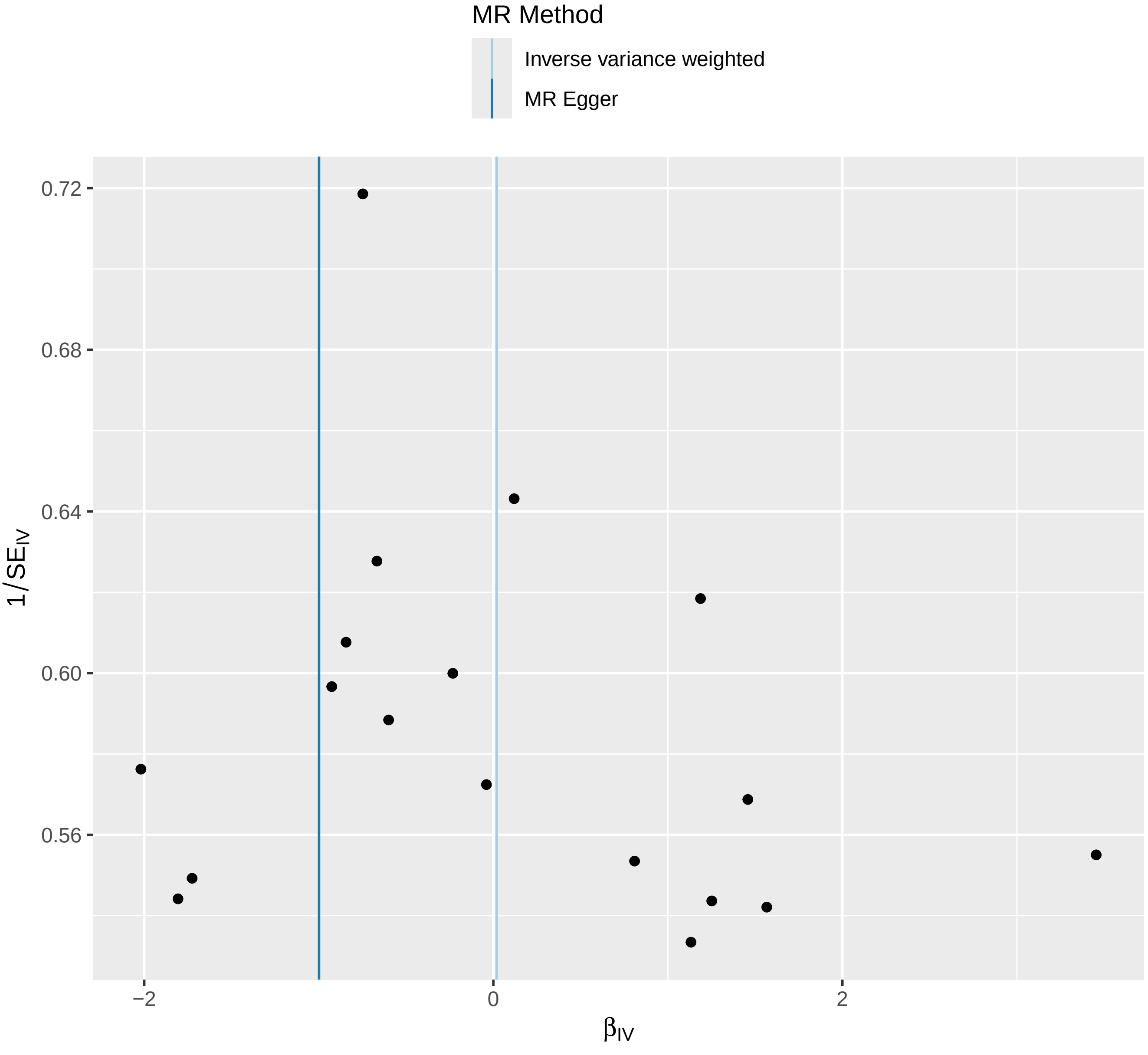
MR Method
Inverse variance weighted
MR Egger
0.72
0.68
0.64
0.60
0.56
$1/SE_{IV}$
−2
0
2
β_{IV}

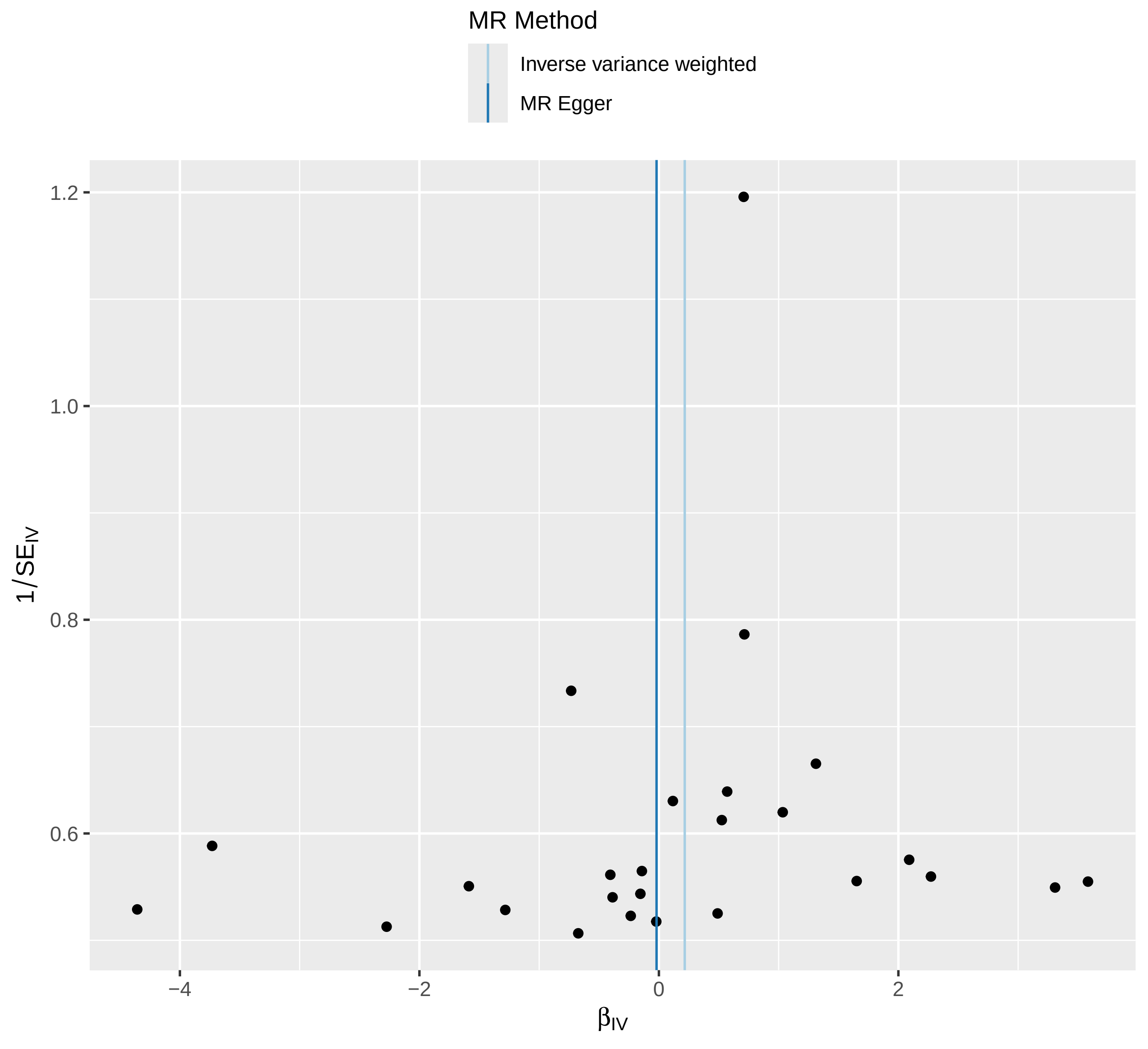
MR Method
Inverse variance weighted
MR Egger
1/SE$_{IV}$
1.2
1.0
0.8
0.6
−4
−2
0
2
β$_{IV}$

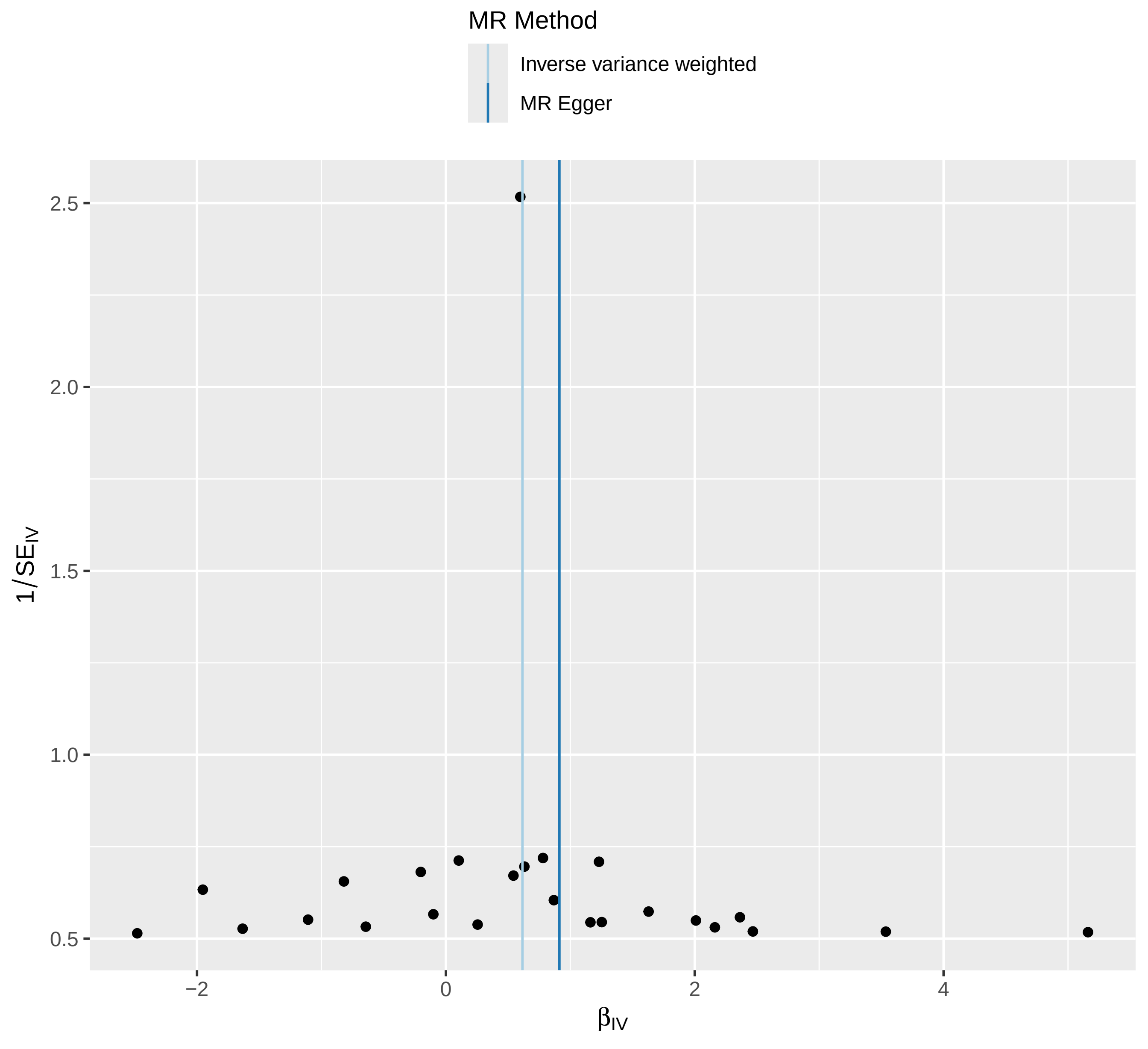

MR Method
Inverse variance weighted
MR Egger
2.5
2.0
1.5
1.0
0.5
−2
0
2
4
$1/SE_{IV}$
β_{IV}

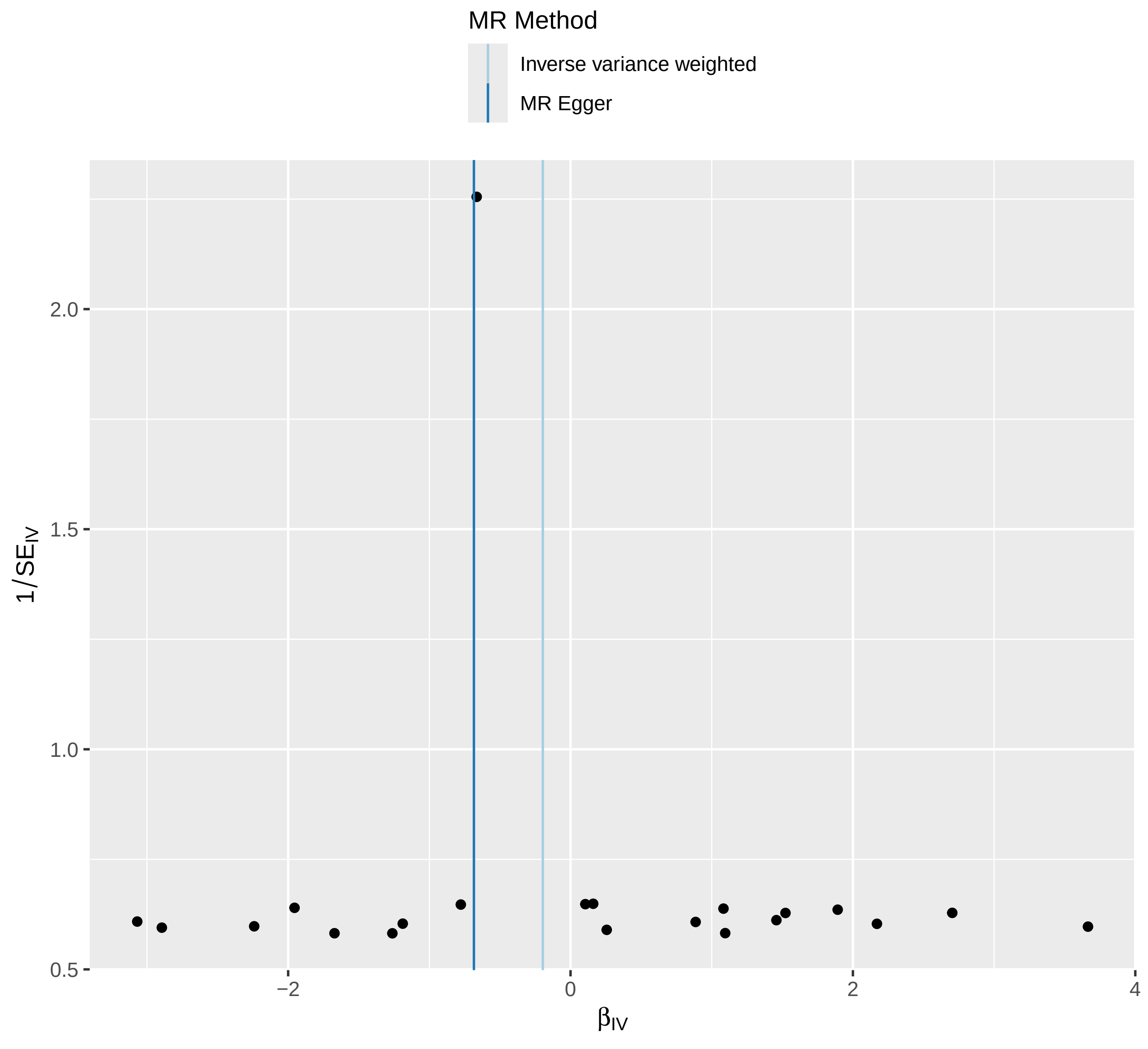
MR Method
Inverse variance weighted
MR Egger
2.0
1.5
1.0
0.5
1/SE_IV
−2
0
2
4
β_IV

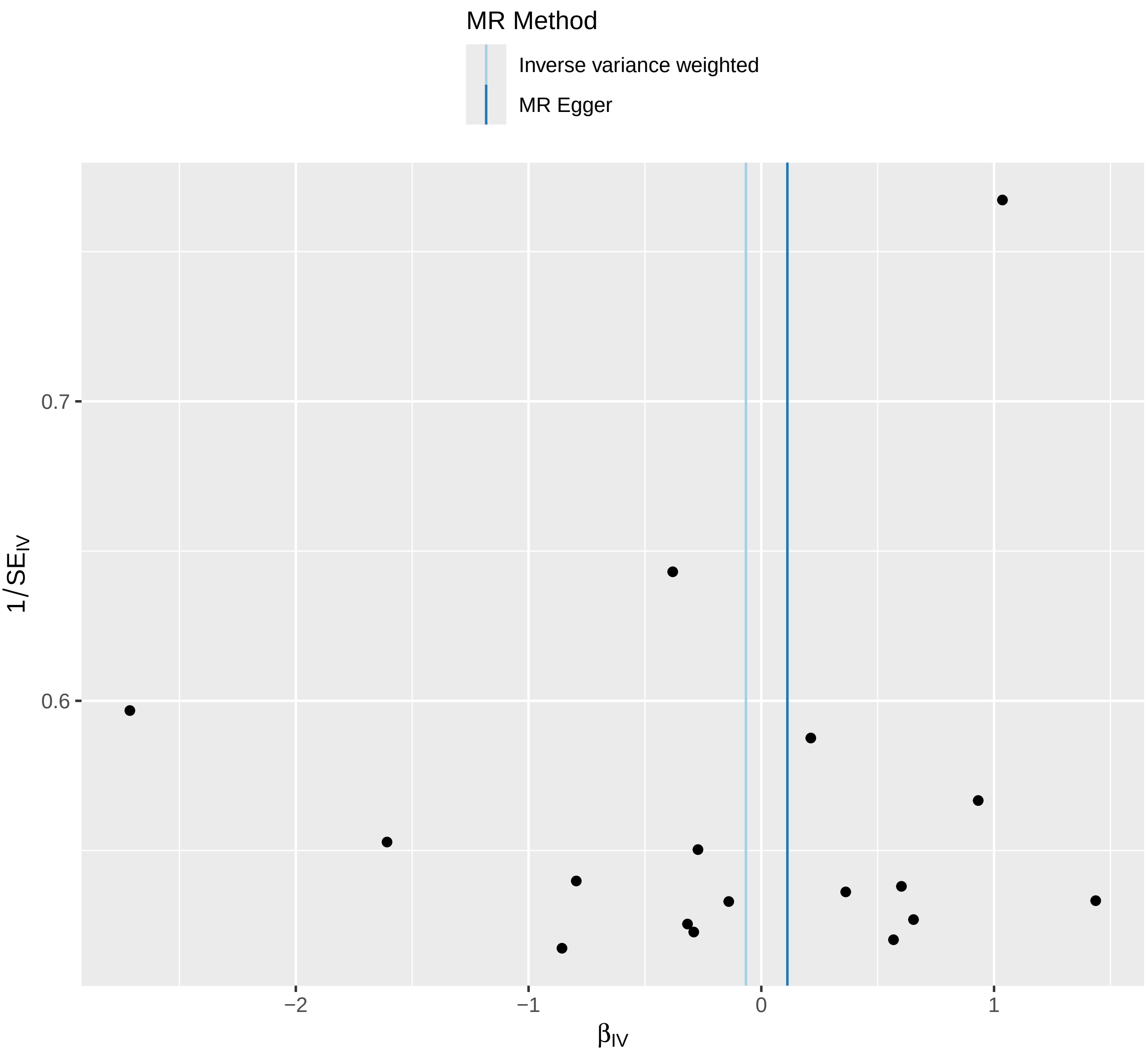

MR Method
Inverse variance weighted
MR Egger
0.7
0.6
1/SE_IV
−2
−1
0
1
β_IV

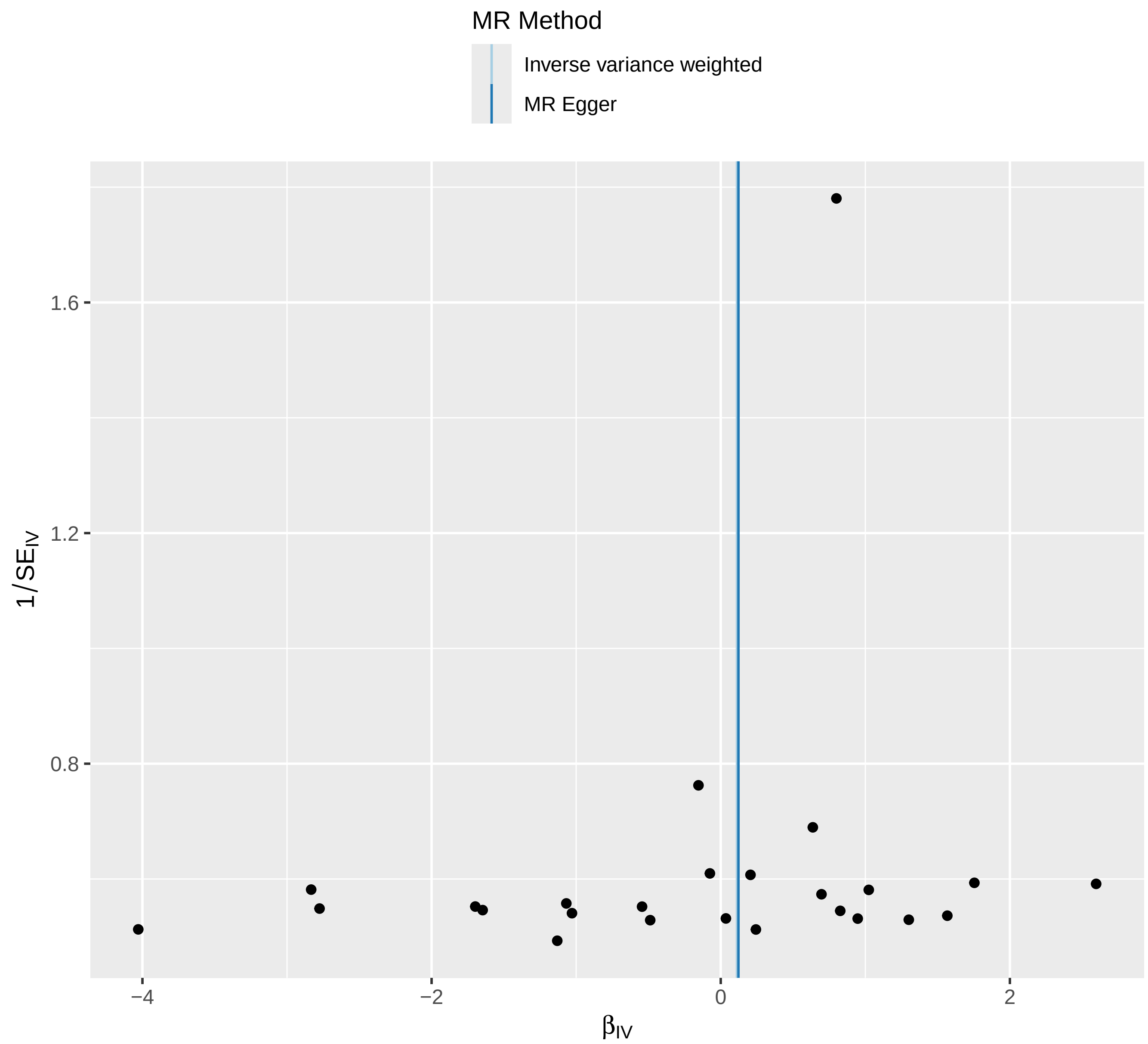
MR Method
Inverse variance weighted
MR Egger
1.6
1.2
0.8
−4
−2
0
2
$1/SE_{IV}$
β_{IV}

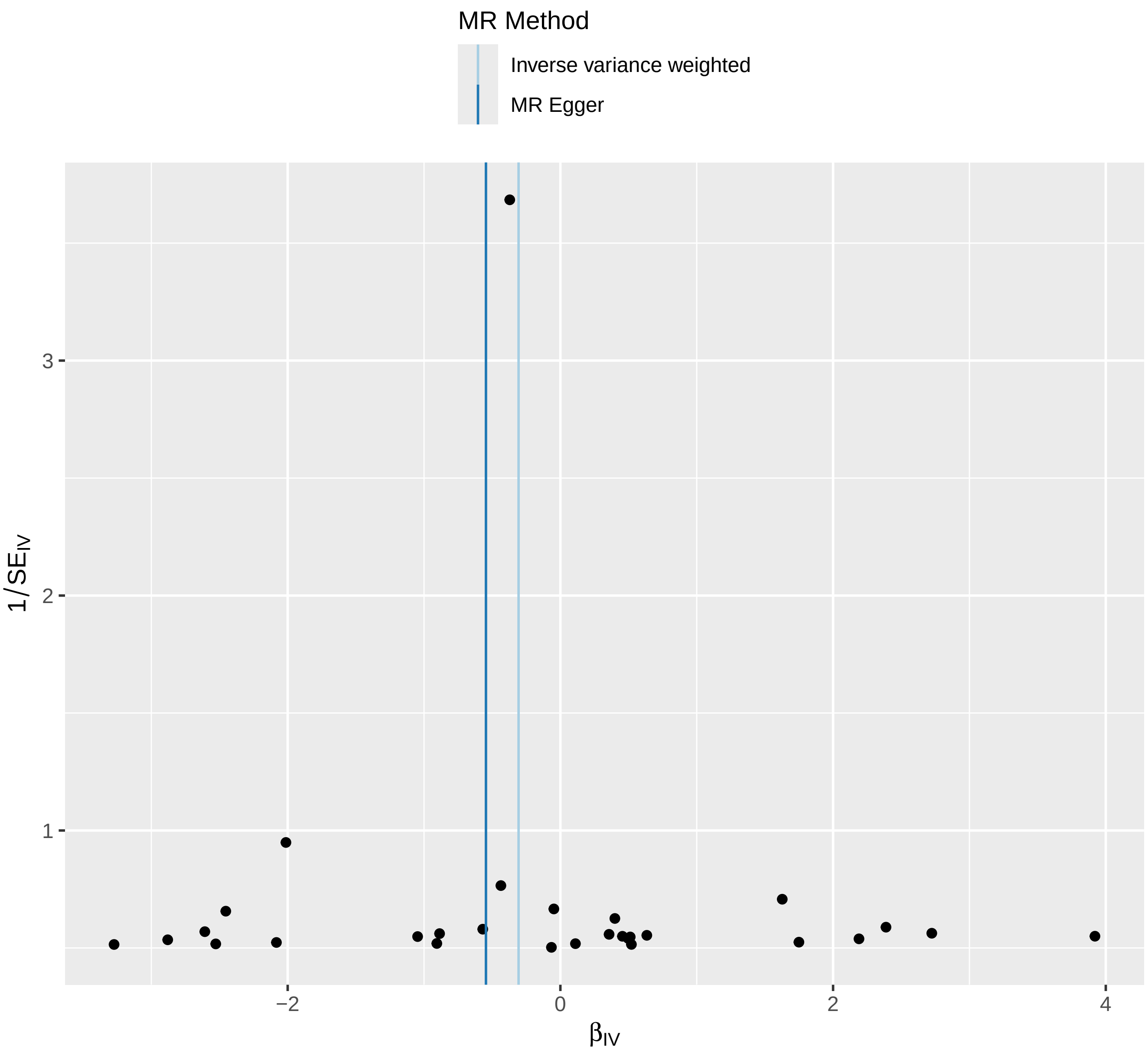
MR Method
Inverse variance weighted
MR Egger
1/SE_IV
3
2
1
−2
0
2
4
β_IV

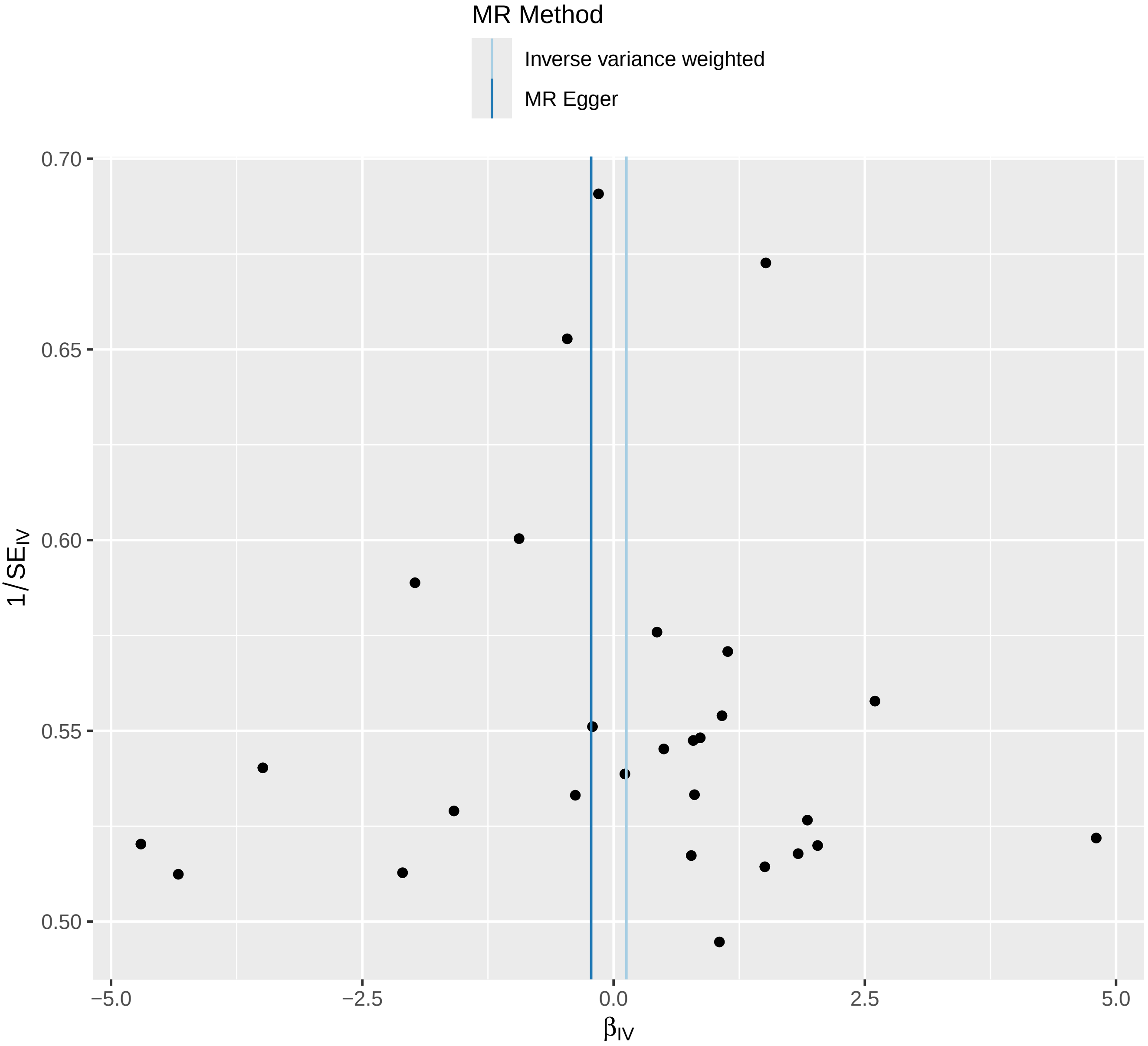
MR Method
Inverse variance weighted
MR Egger
0.70
0.65
0.60
0.55
0.50
1/SE_IV
−5.0
−2.5
0.0
2.5
5.0
β_IV

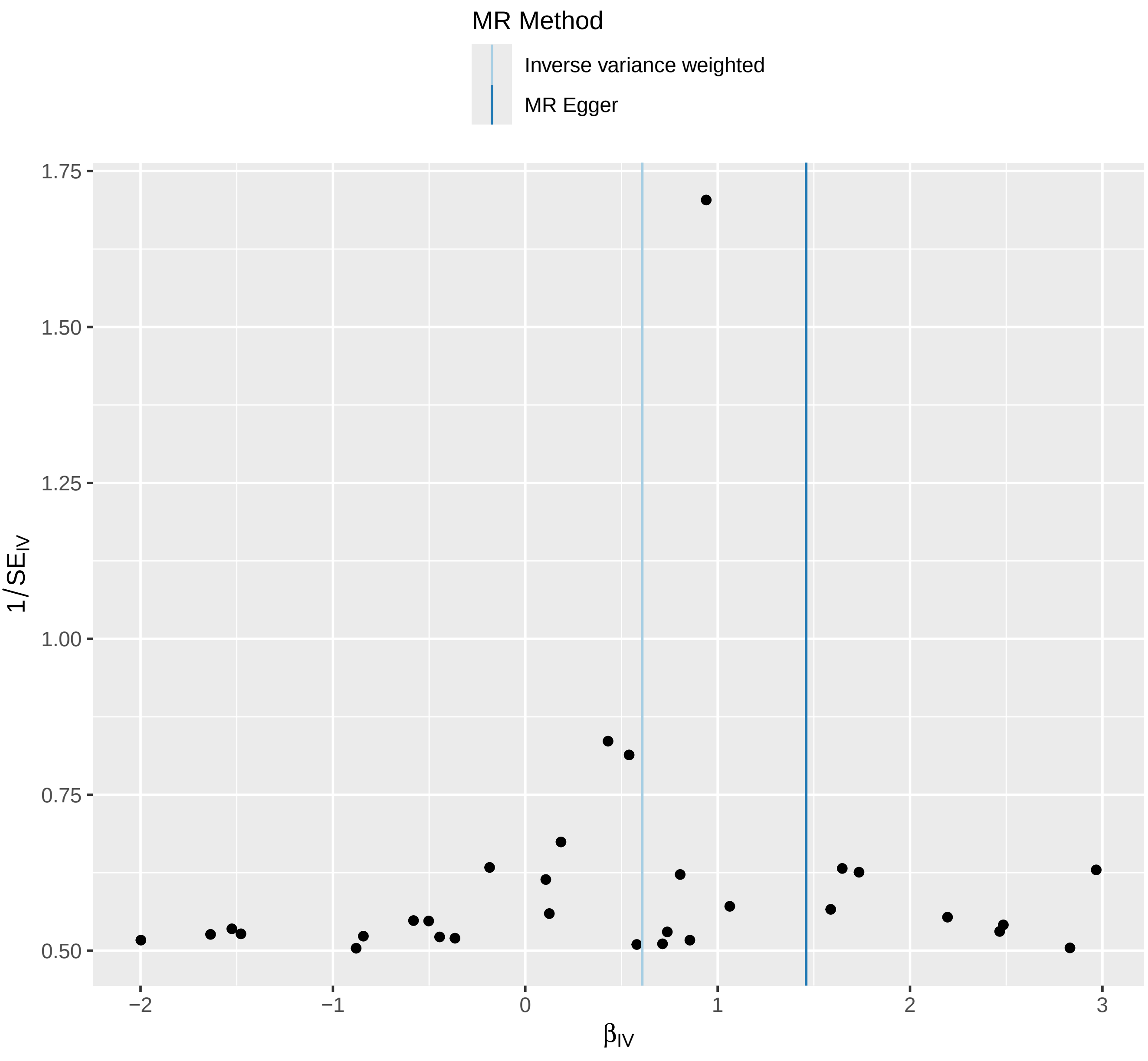
MR Method
Inverse variance weighted
MR Egger
1.75
1.50
1.25
1.00
0.75
0.50
1/SE_IV
−2
−1
0
1
2
3
β_IV

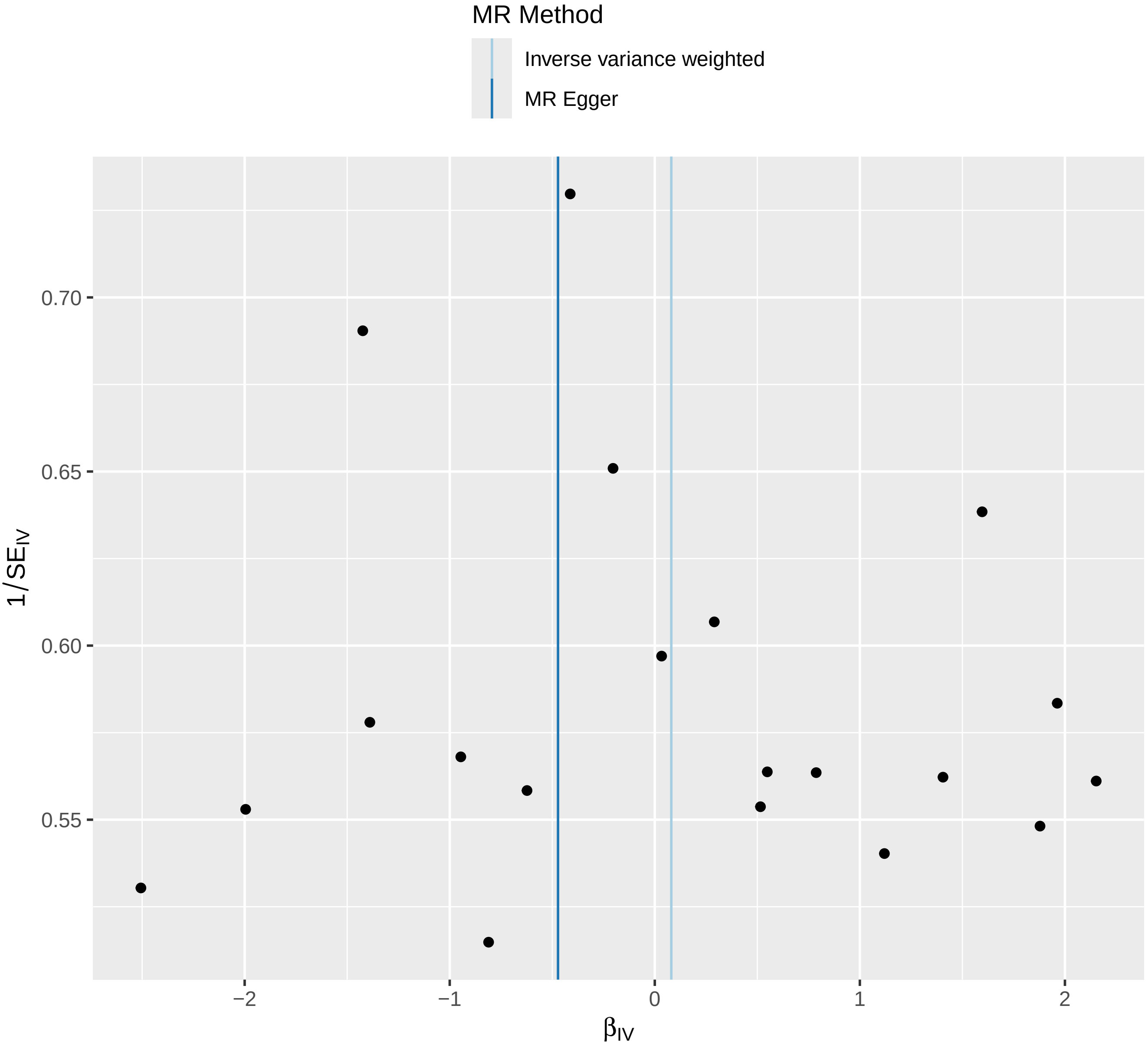
MR Method
Inverse variance weighted
MR Egger
0.70
0.65
0.60
0.55
1/SE_IV
−2
−1
0
1
2
β_IV

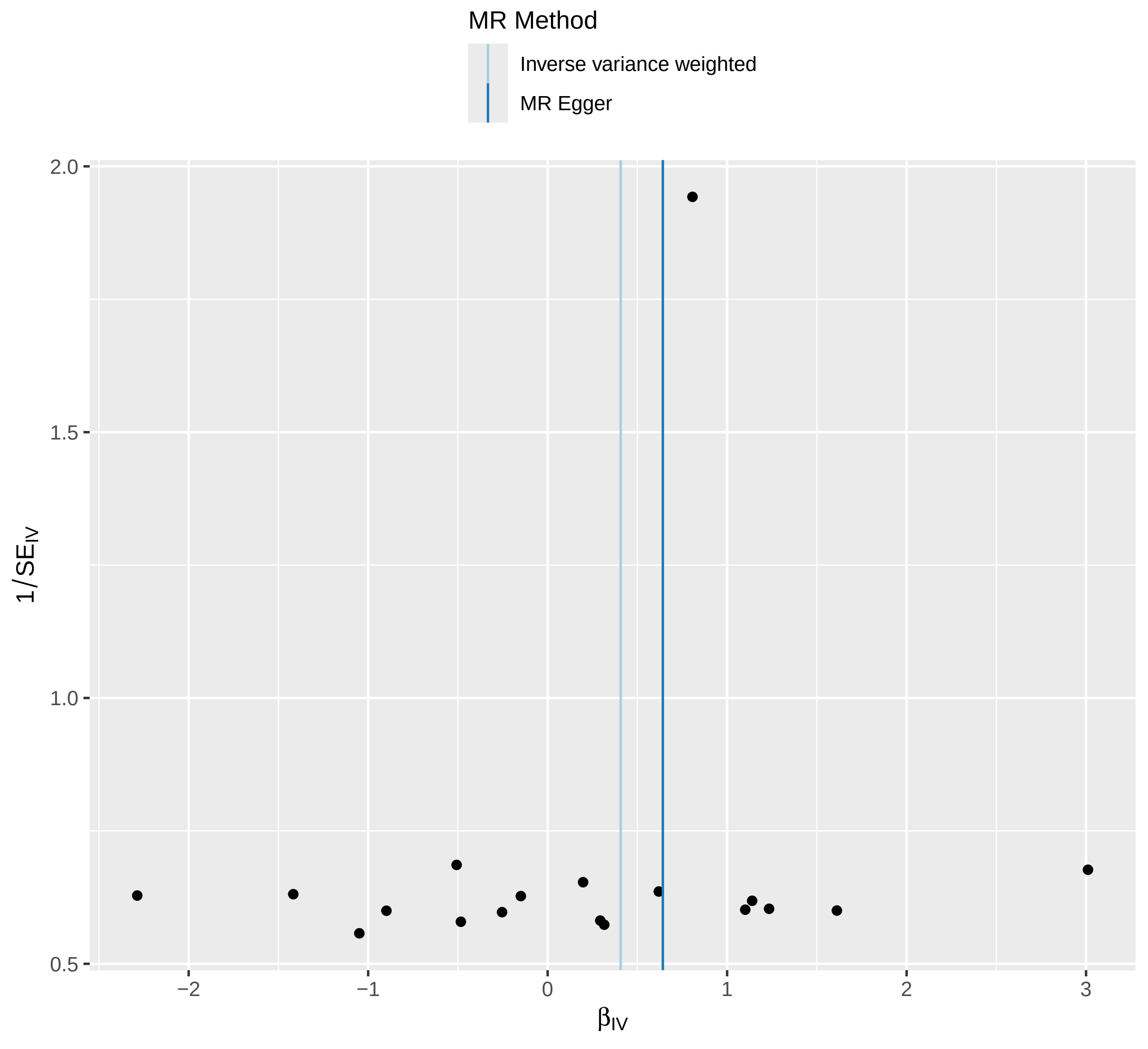
MR Method
Inverse variance weighted
MR Egger
2.0
1.5
1.0
0.5
1/SE_IV
−2
−1
0
1
2
3
β_IV

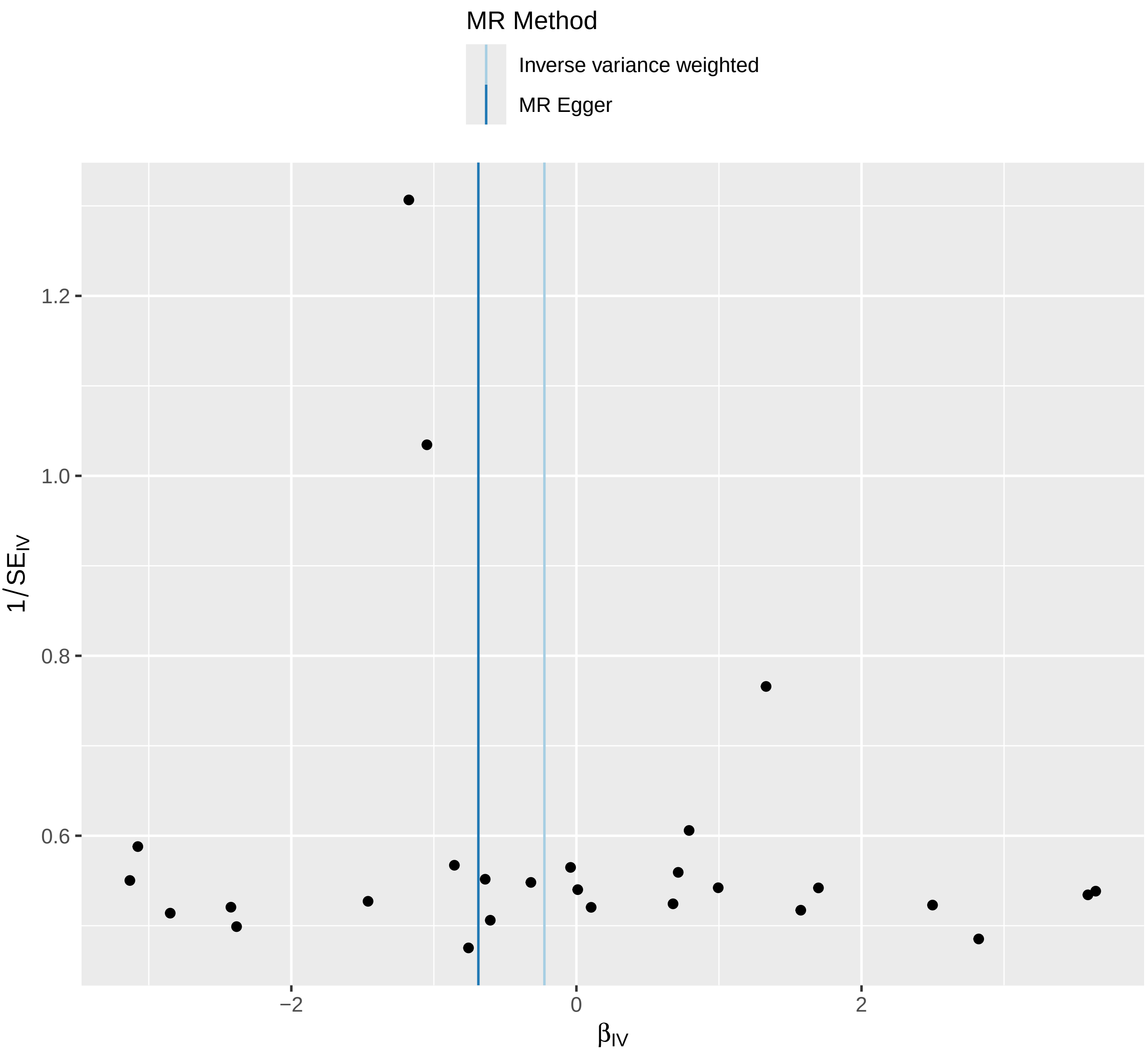
MR Method
Inverse variance weighted
MR Egger
1.2
1.0
0.8
0.6
$1/SE_{IV}$
−2
0
2
β_{IV}

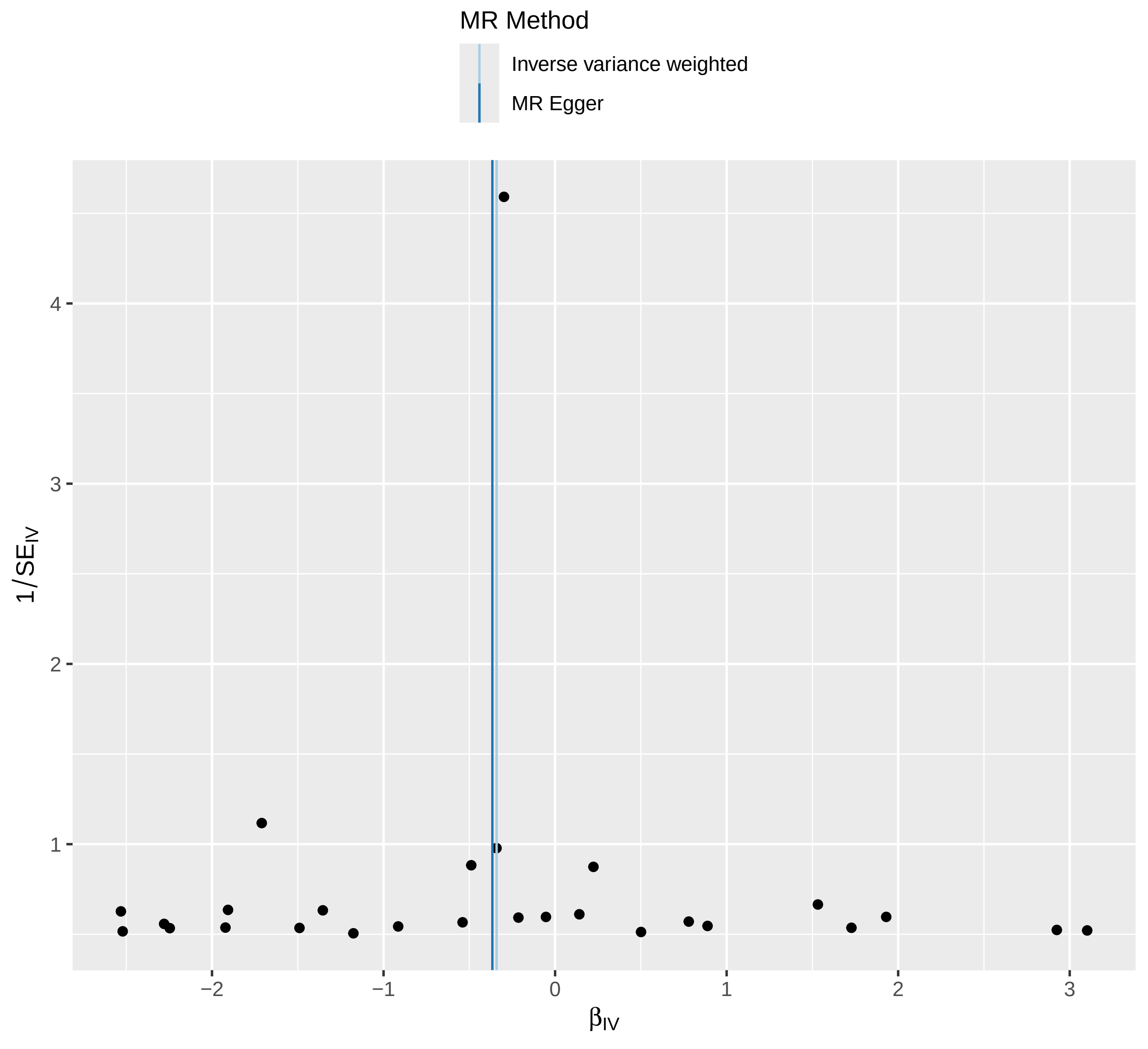
MR Method
Inverse variance weighted
MR Egger
1/SE_IV
β_IV
4
3
2
1
−2
−1
0
1
2
3

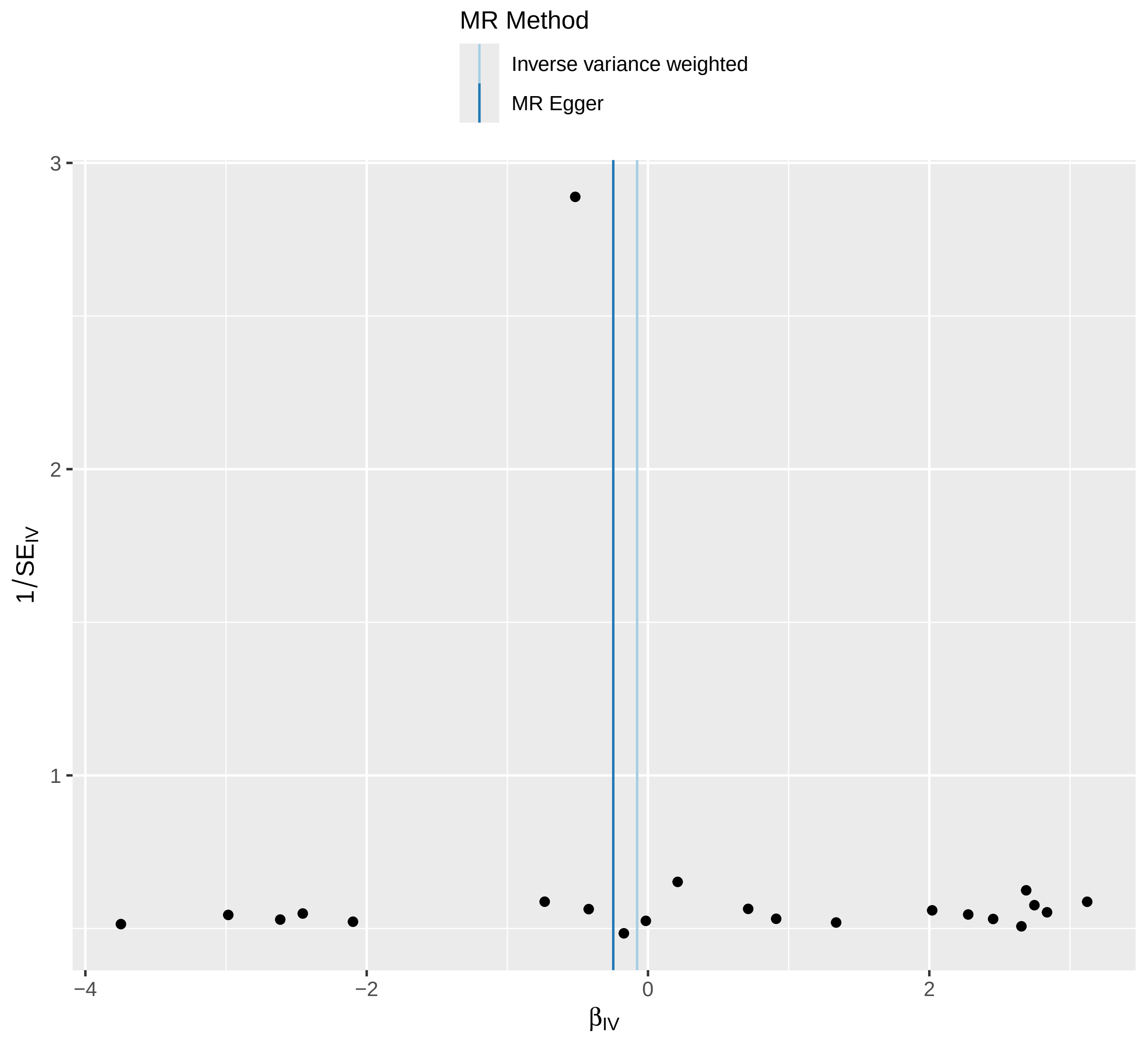
MR Method
Inverse variance weighted
MR Egger
3
2
1
1/SE_IV
−4
−2
0
2
β_IV

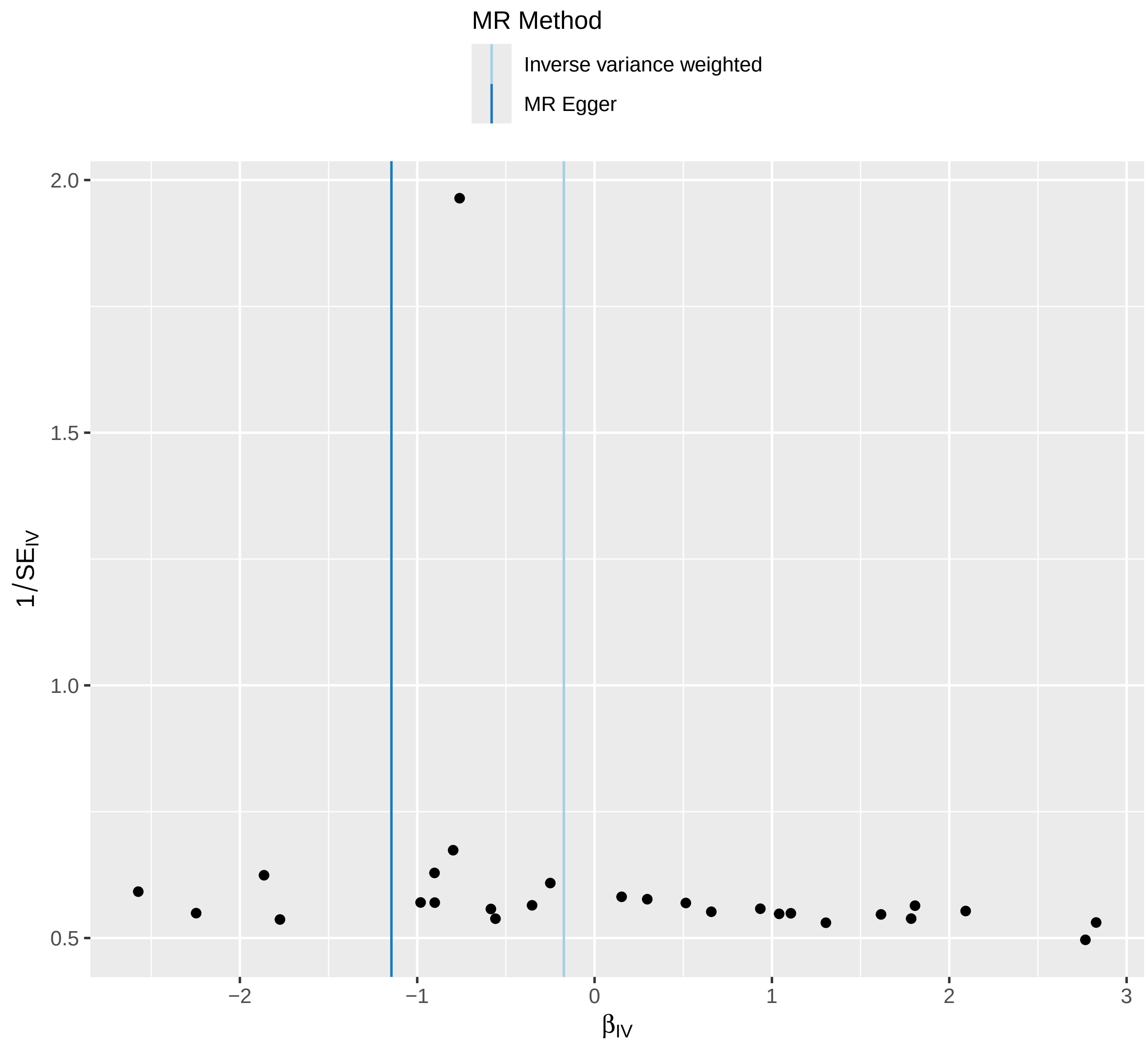

MR Method
Inverse variance weighted
MR Egger
2.0
1.5
1.0
0.5
1/SE_IV
−2
−1
0
1
2
3
β_IV

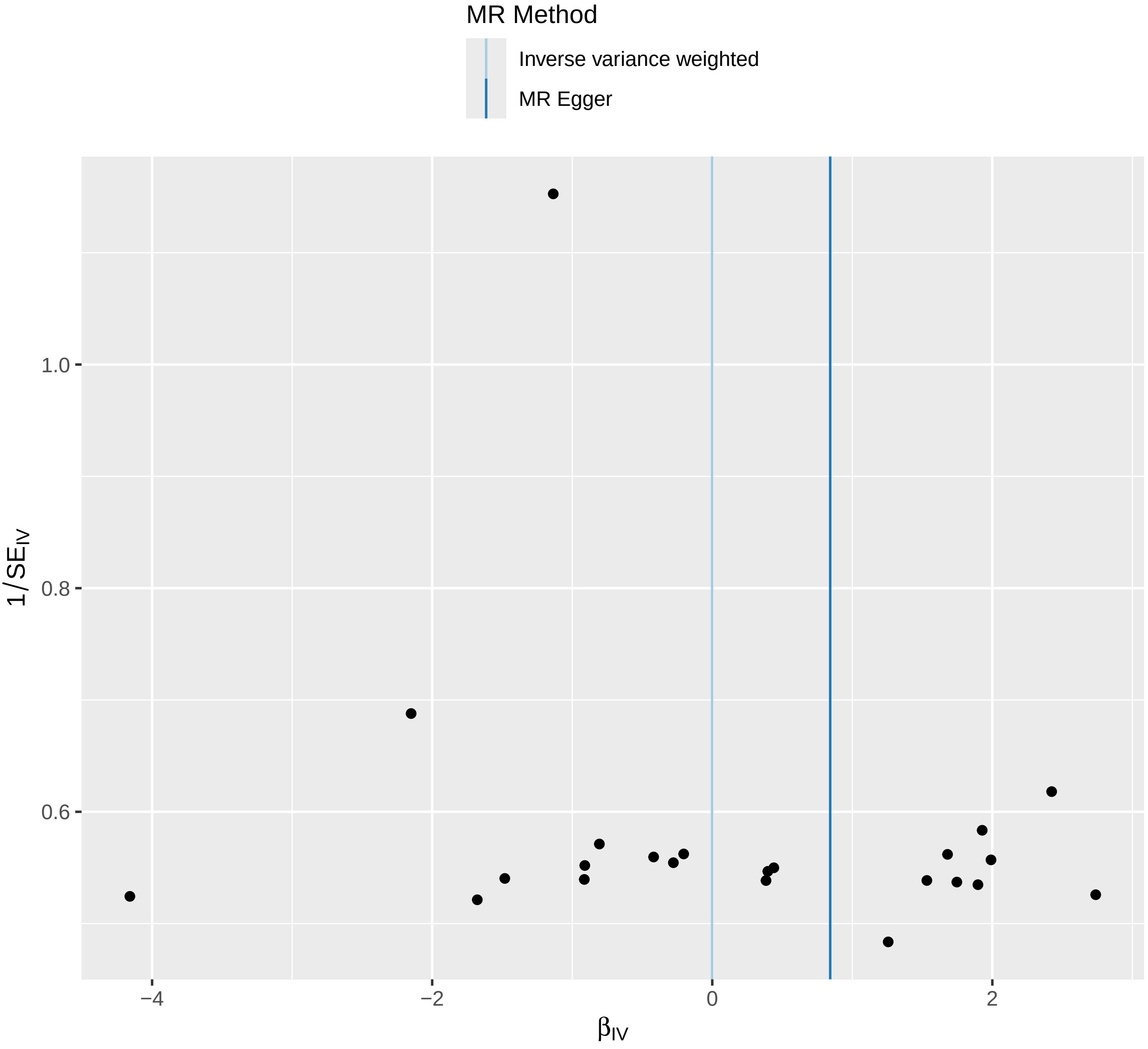
MR Method
Inverse variance weighted
MR Egger
1.0
0.8
0.6
$1/SE_{IV}$
−4
−2
0
2
β_{IV}

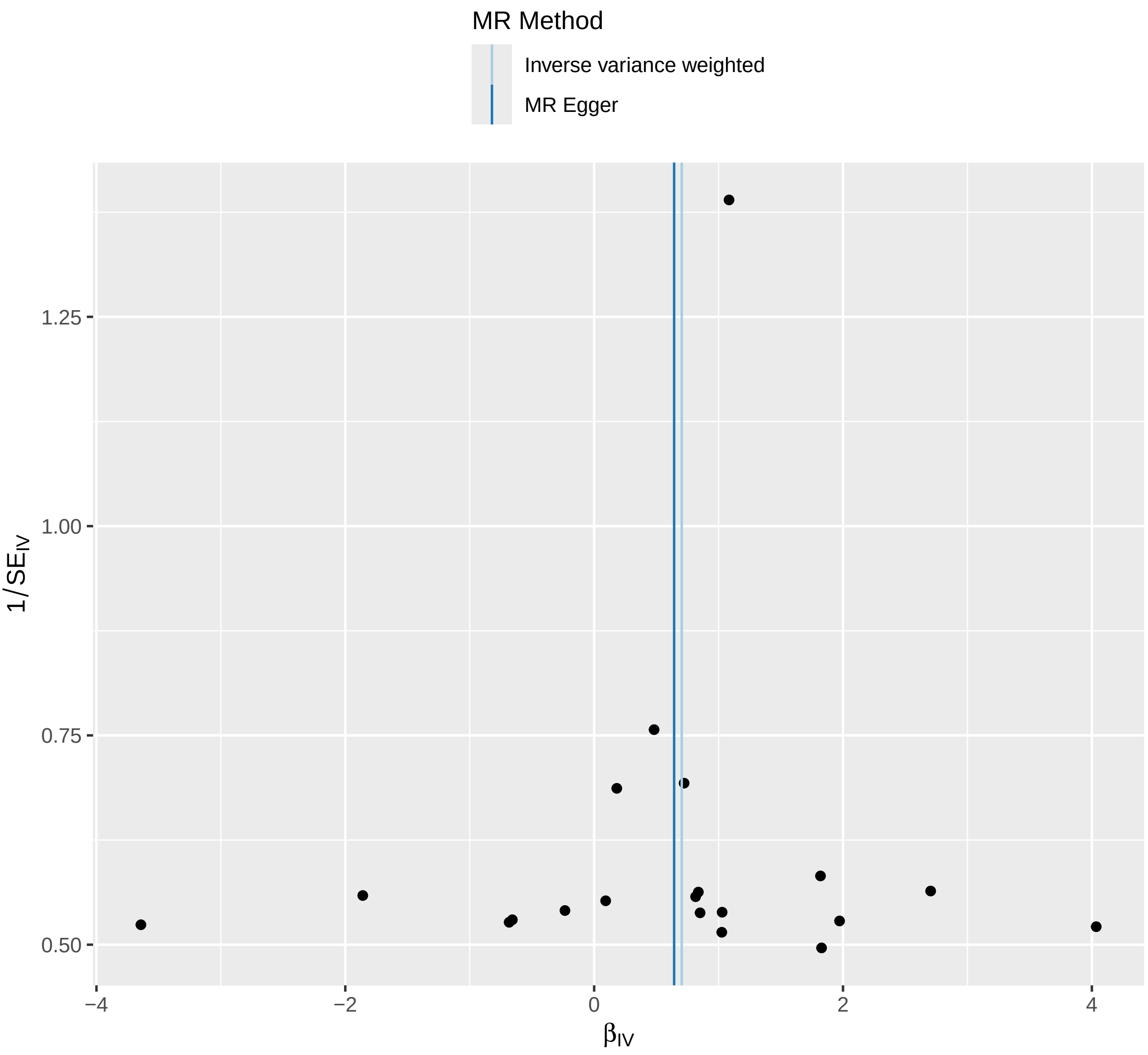
MR Method
Inverse variance weighted
MR Egger
1.25
1.00
0.75
0.50
1/SE_IV
−4
−2
0
2
4
β_IV

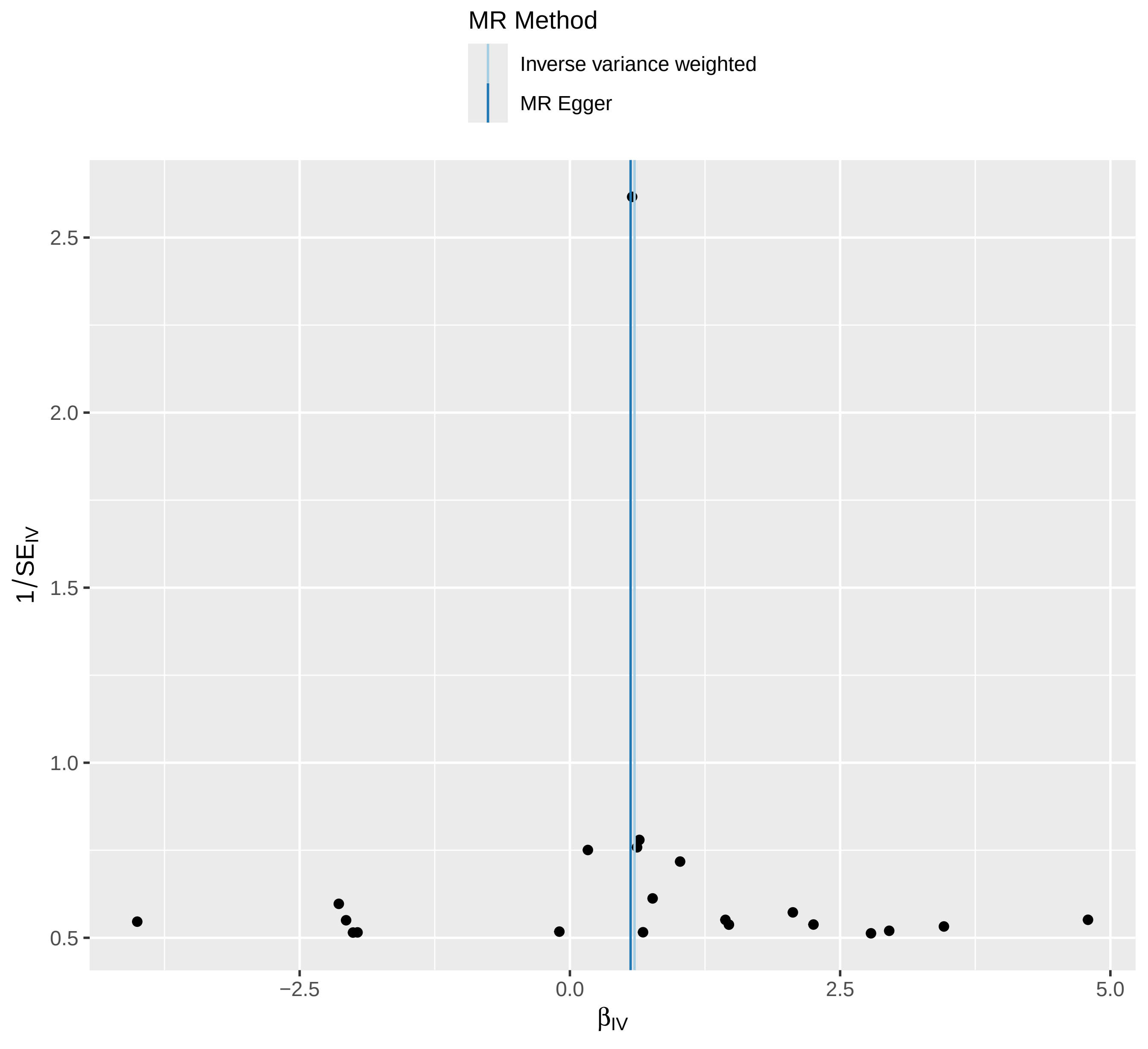

MR Method
Inverse variance weighted
MR Egger
2.5
2.0
1.5
1.0
0.5
1/SE_IV
−2.5
0.0
2.5
5.0
β_IV

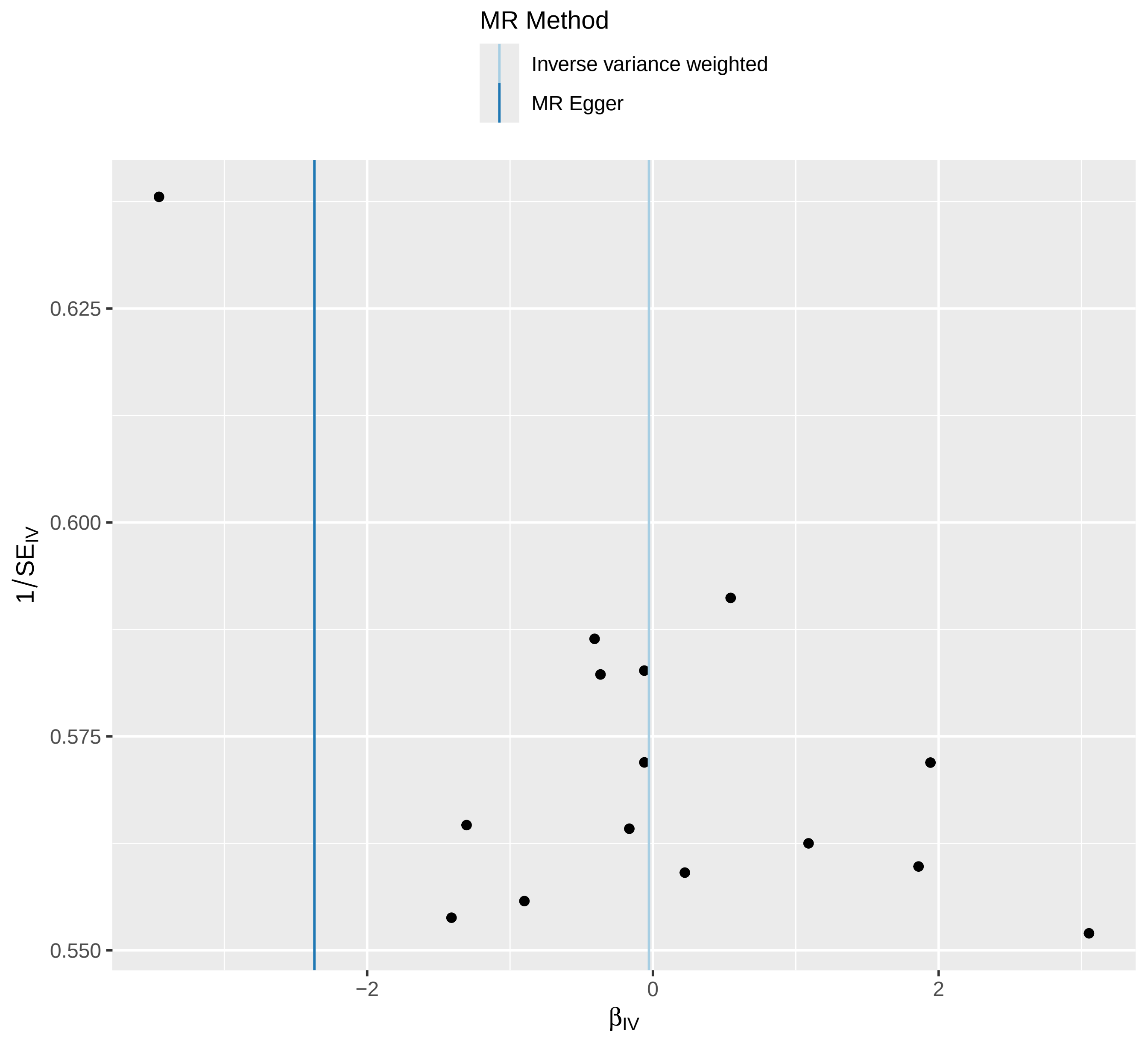
MR Method
Inverse variance weighted
MR Egger
0.625
0.600
0.575
0.550
1/SE_IV
−2
0
2
β_IV

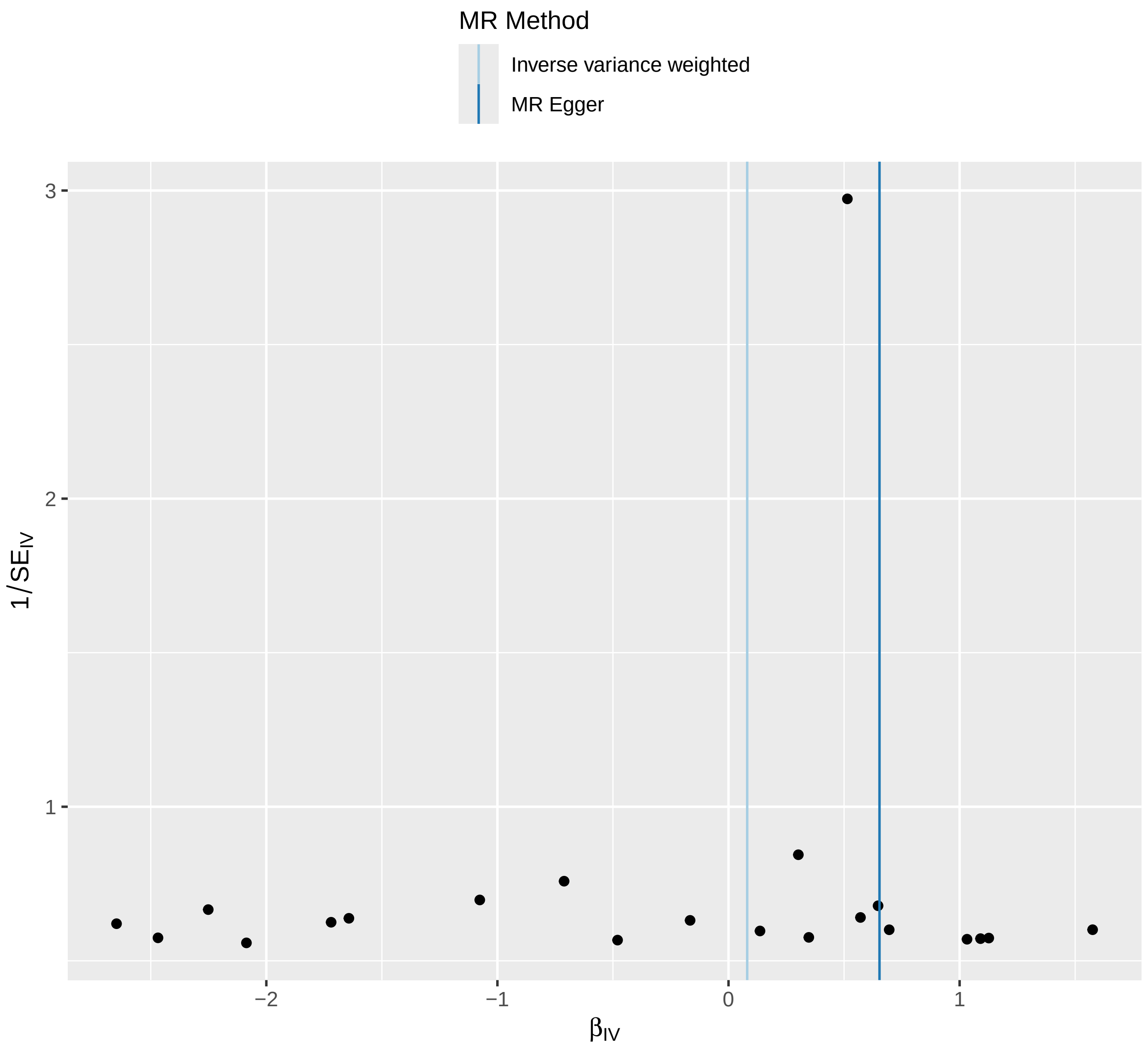

MR Method
Inverse variance weighted
MR Egger
3
2
1
1/SE_IV
−2
−1
0
1
β_IV

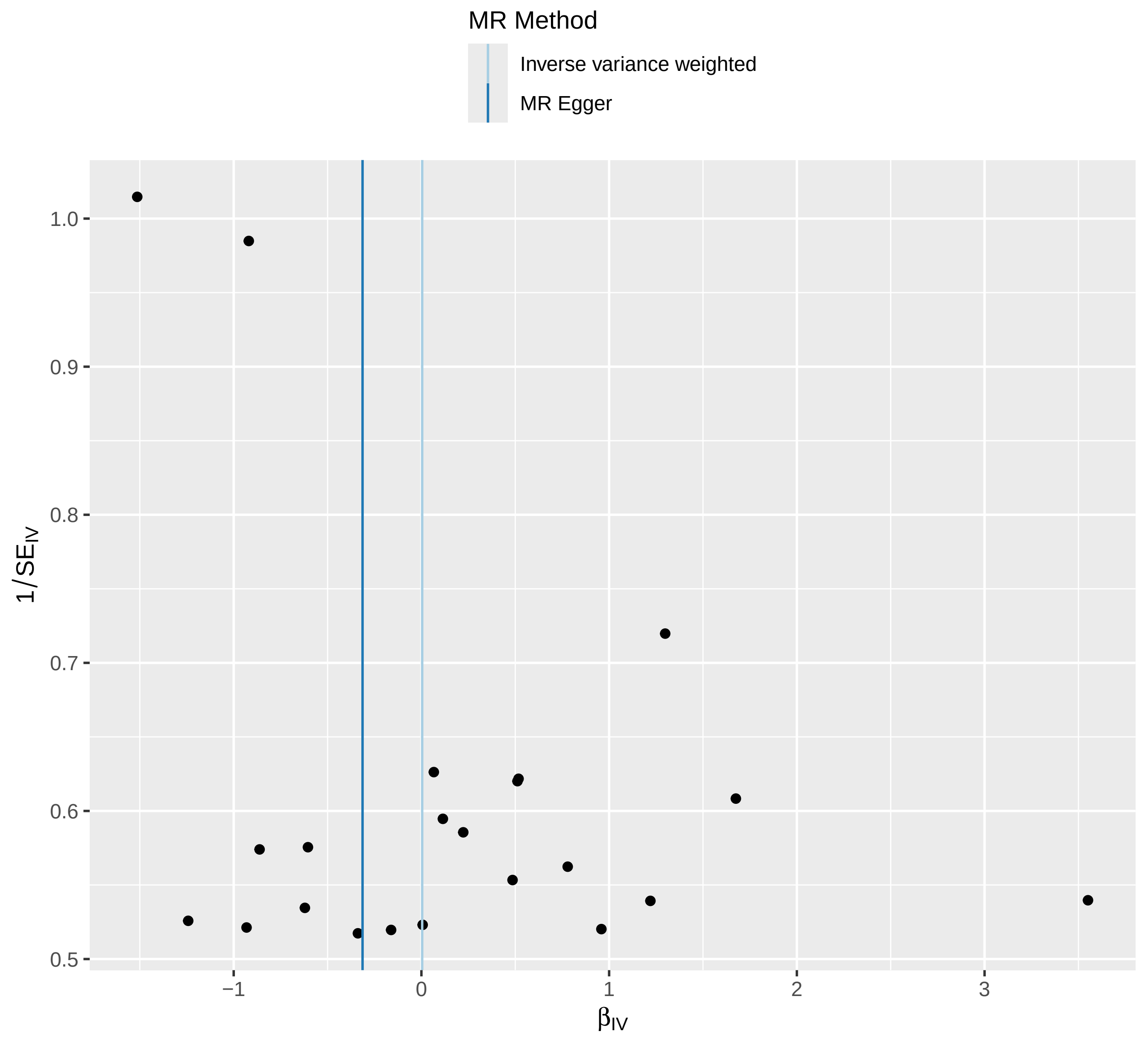
MR Method
Inverse variance weighted
MR Egger
1/SE_{IV}
1.0
0.9
0.8
0.7
0.6
0.5
−1
0
1
2
3
β_{IV}

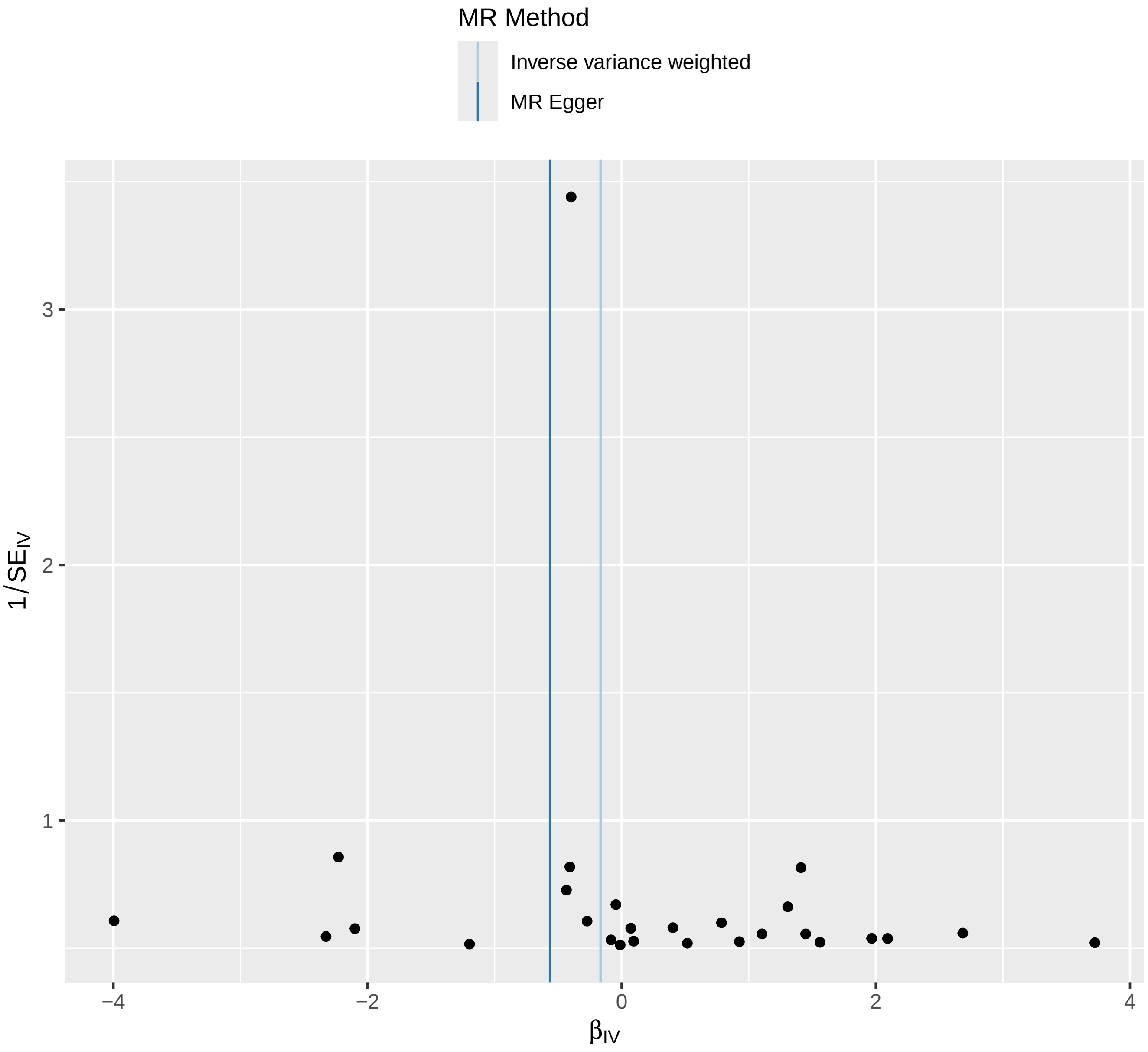

MR Method
Inverse variance weighted
MR Egger
1/SE_IV
3
2
1
−4
−2
0
2
4
β_IV

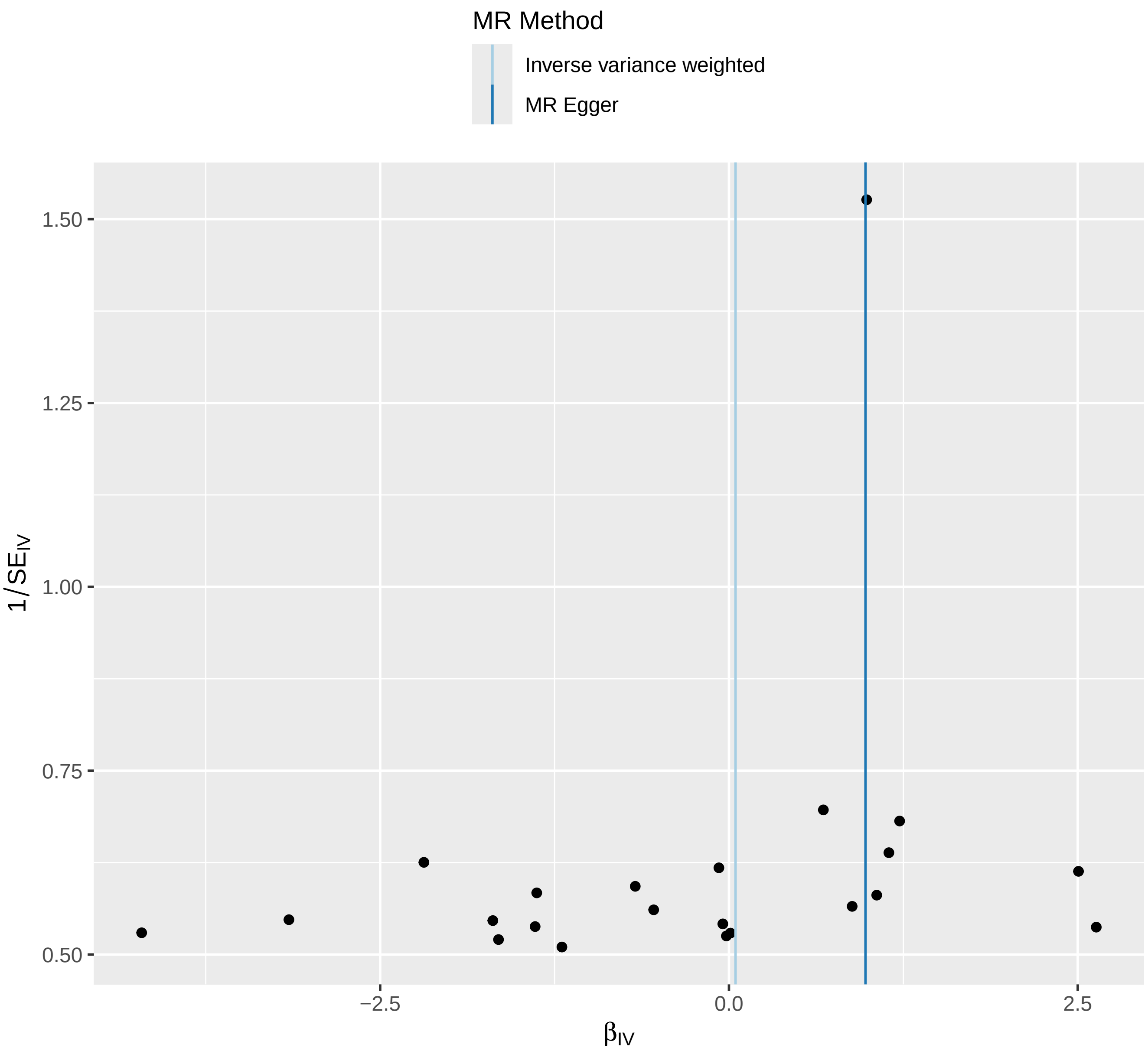
MR Method
Inverse variance weighted
MR Egger
1.50
1.25
1.00
0.75
0.50
1/SE_IV
−2.5
0.0
2.5
β_IV

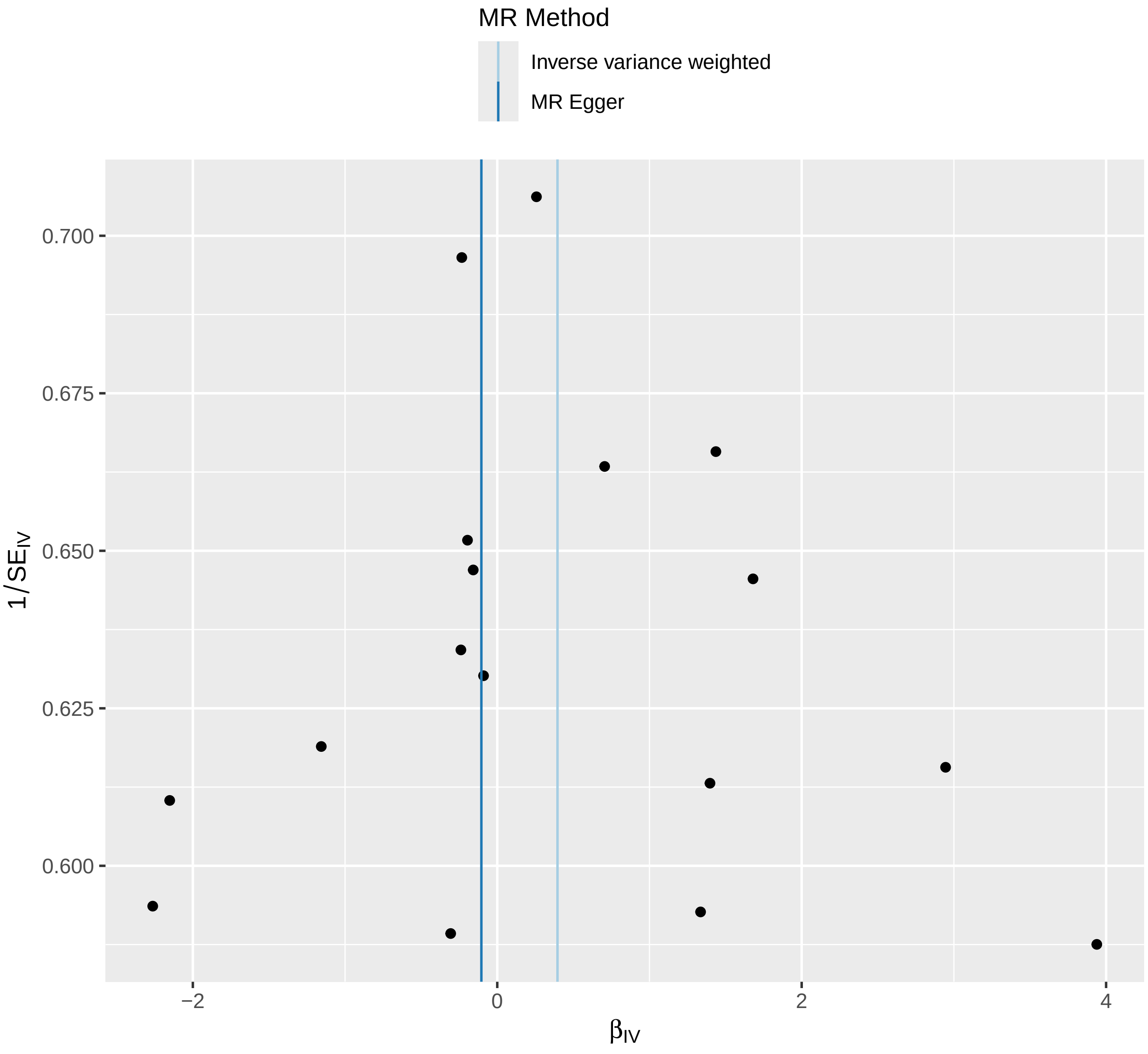
MR Method
Inverse variance weighted
MR Egger
0.700
0.675
0.650
0.625
0.600
1/SE_IV
−2
0
2
4
β_IV

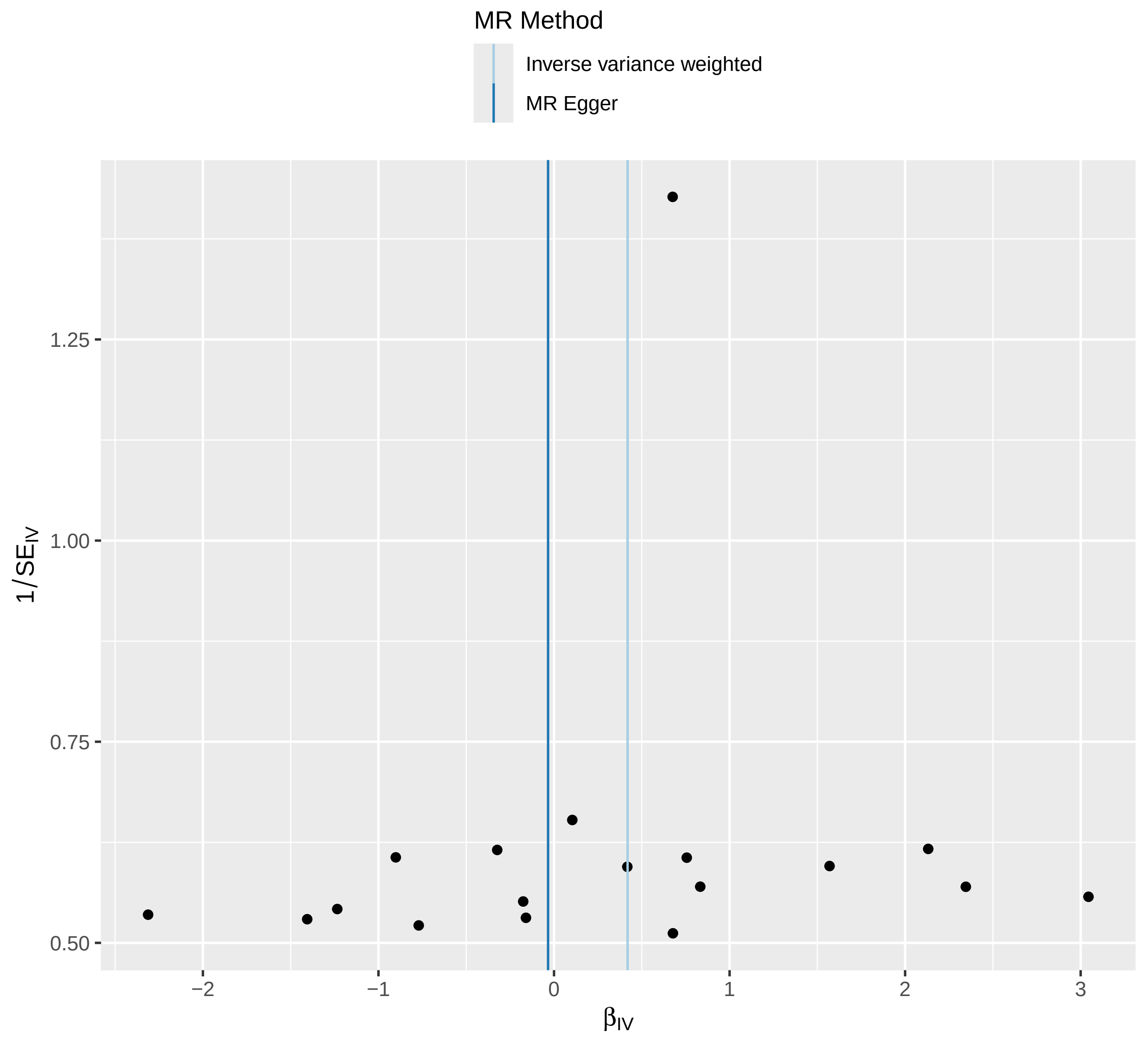
MR Method
Inverse variance weighted
MR Egger
1.25
1.00
0.75
0.50
1/SE_IV
−2
−1
0
1
2
3
β_IV

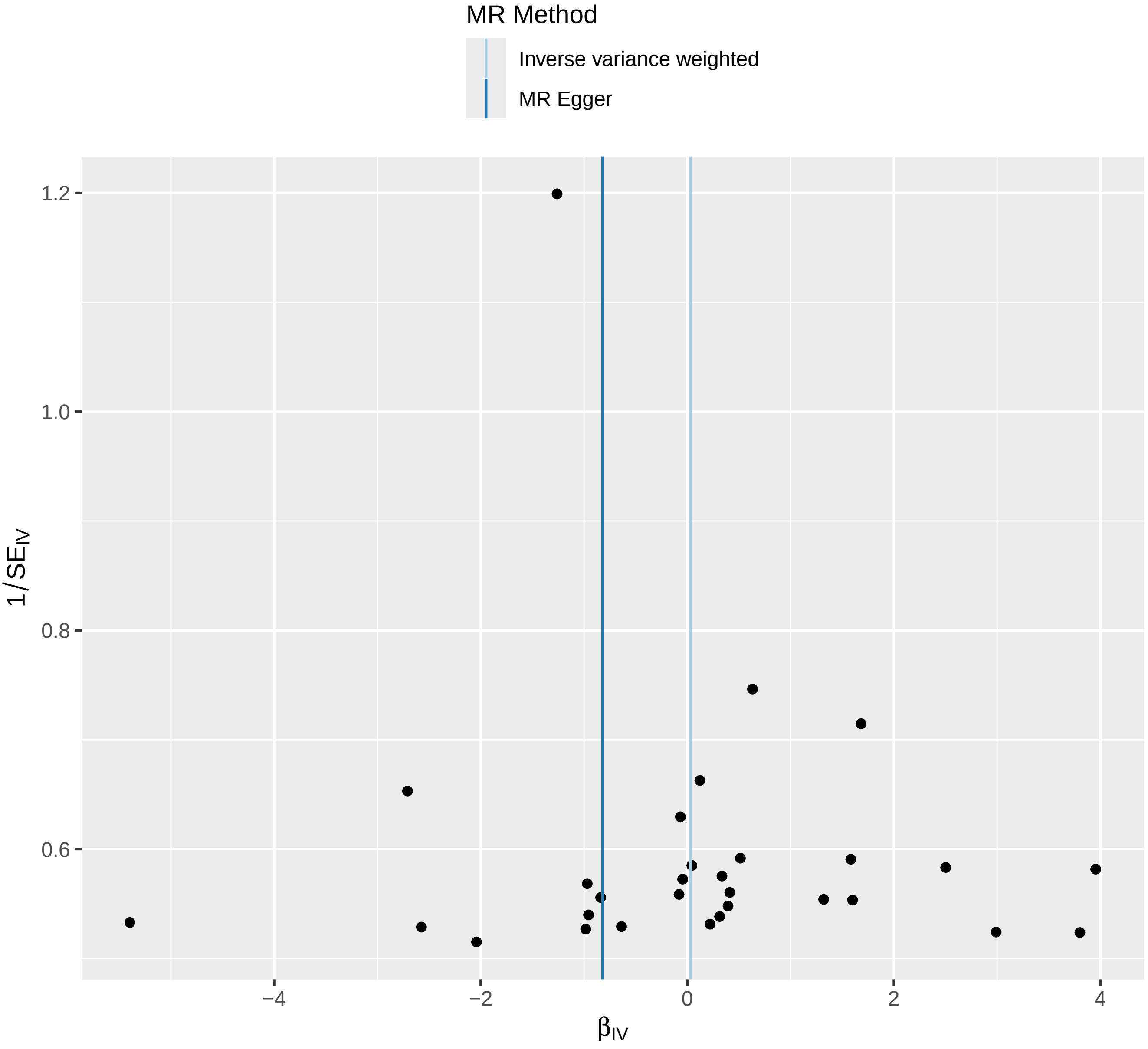
MR Method
Inverse variance weighted
MR Egger
1.2
1.0
0.8
0.6
1/SE_IV
−4
−2
0
2
4
β_IV

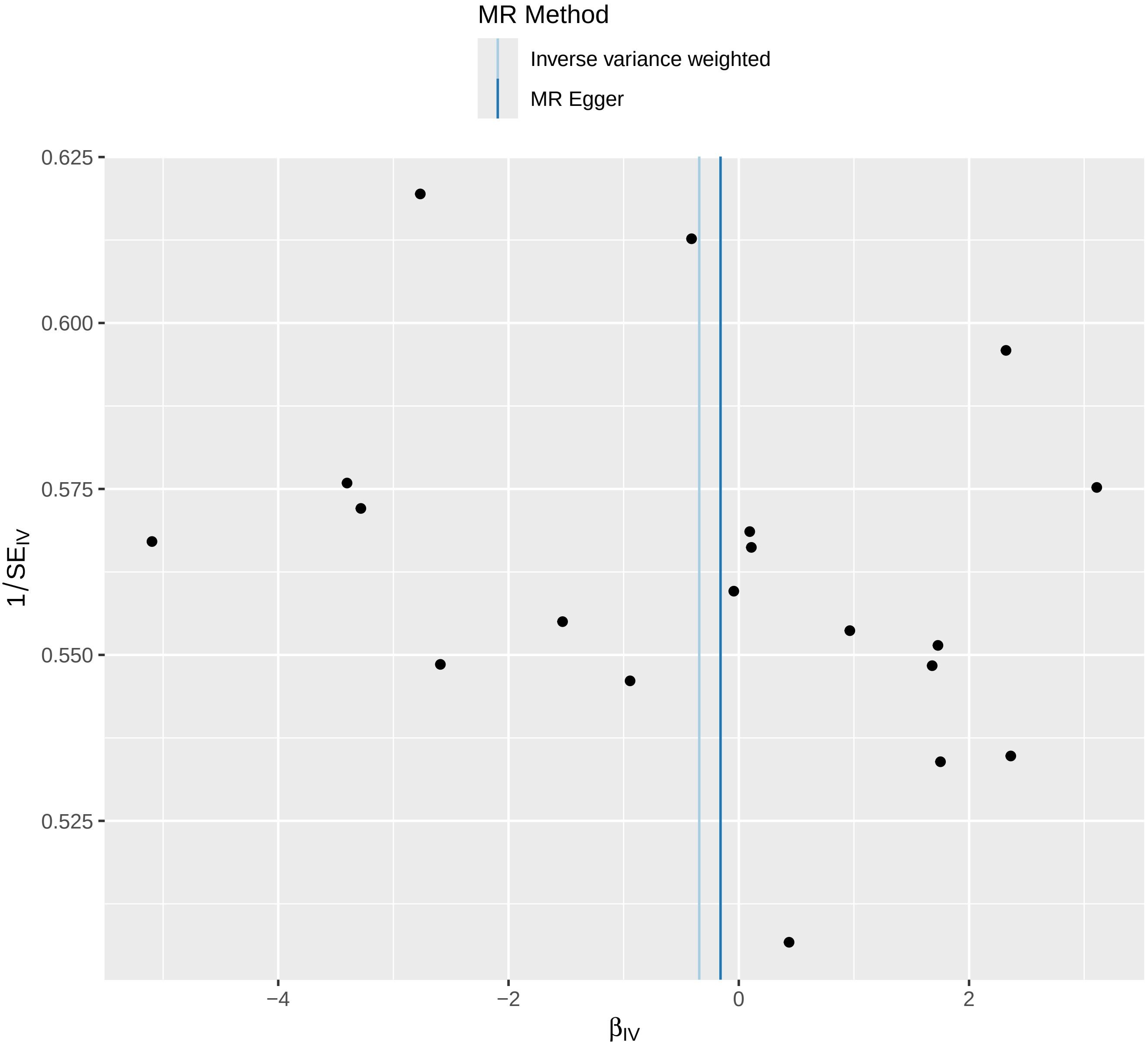
MR Method
Inverse variance weighted
MR Egger
0.625
0.600
0.575
0.550
0.525
$1/SE_{IV}$
−4
−2
0
2
β_{IV}

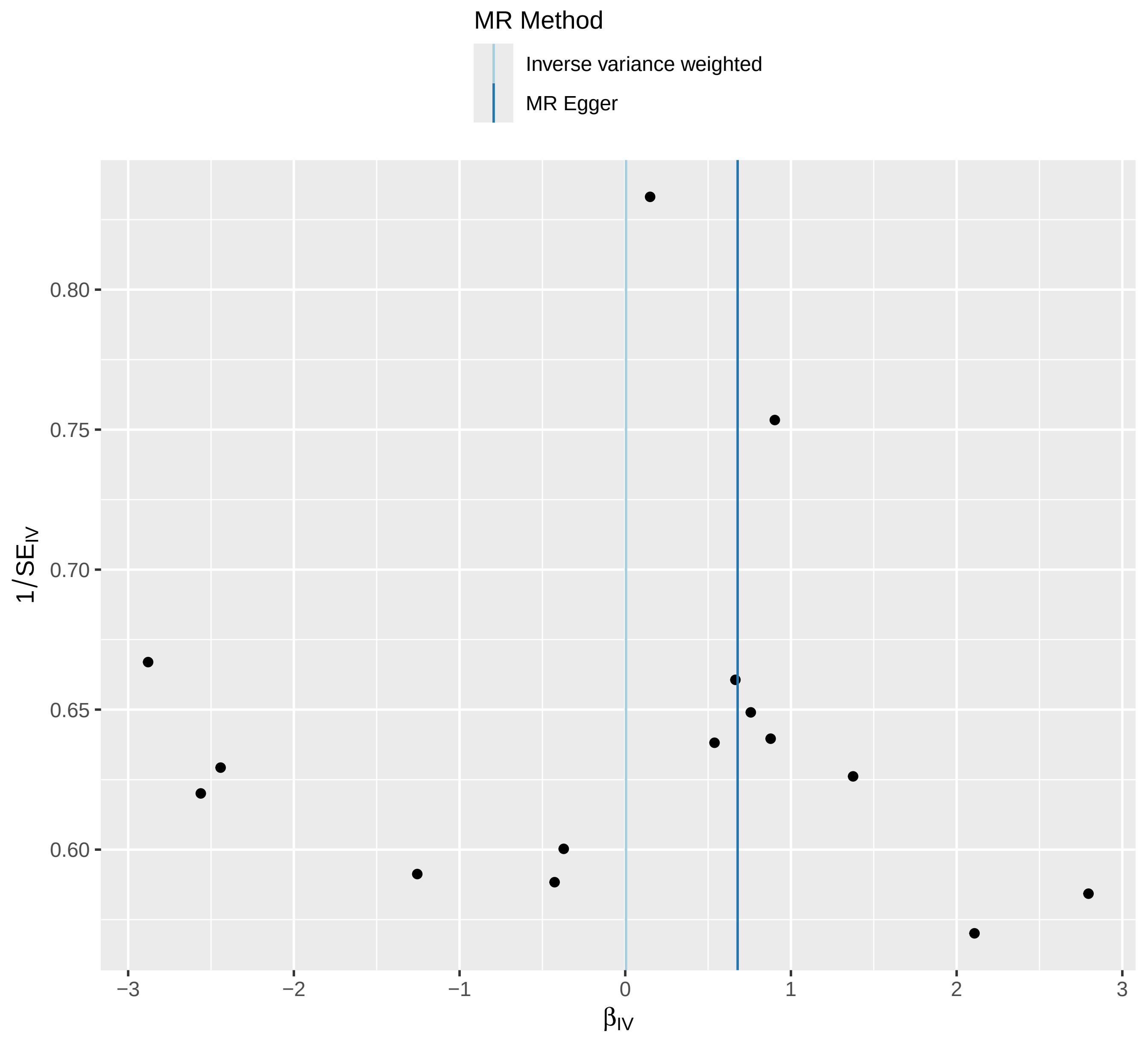
MR Method
Inverse variance weighted
MR Egger
0.80
0.75
0.70
0.65
0.60
1/SE_IV
−3
−2
−1
0
1
2
3
β_IV

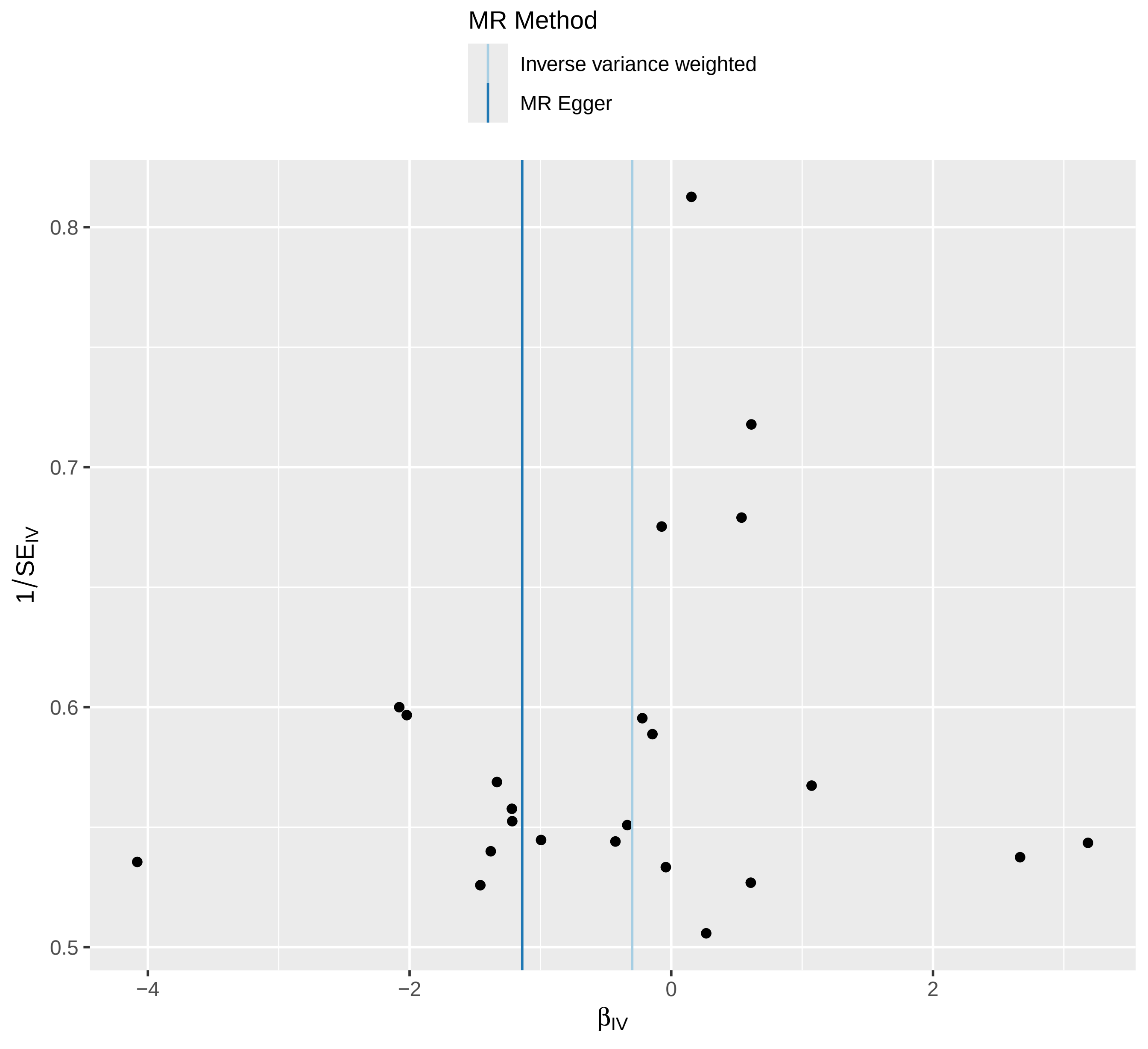
MR Method
Inverse variance weighted
MR Egger
0.8
0.7
0.6
0.5
$1/SE_{IV}$
−4
−2
0
2
β_{IV}

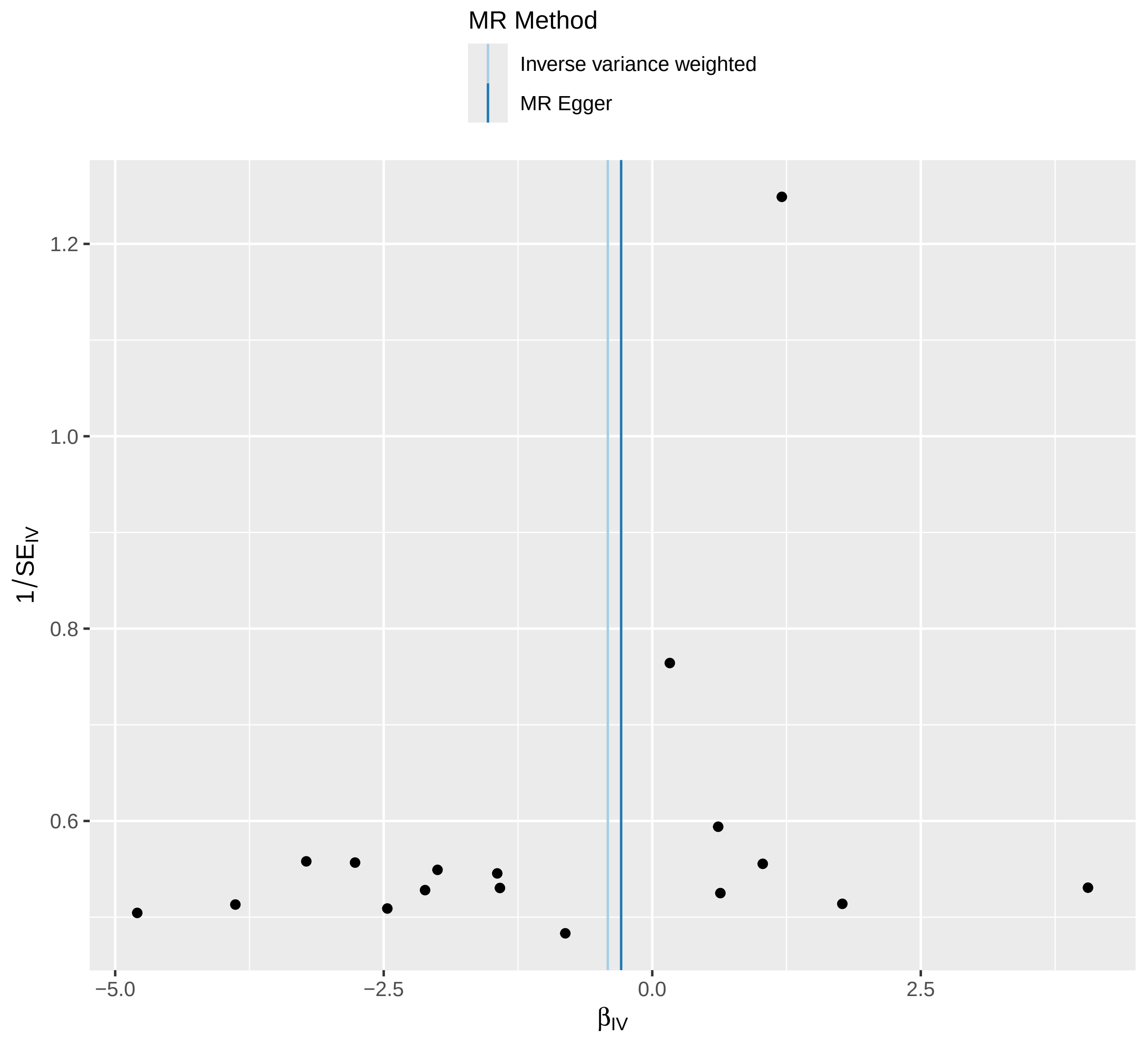
MR Method
Inverse variance weighted
MR Egger
1.2
1.0
0.8
0.6
1/SE_IV
−5.0
−2.5
0.0
2.5
β_IV

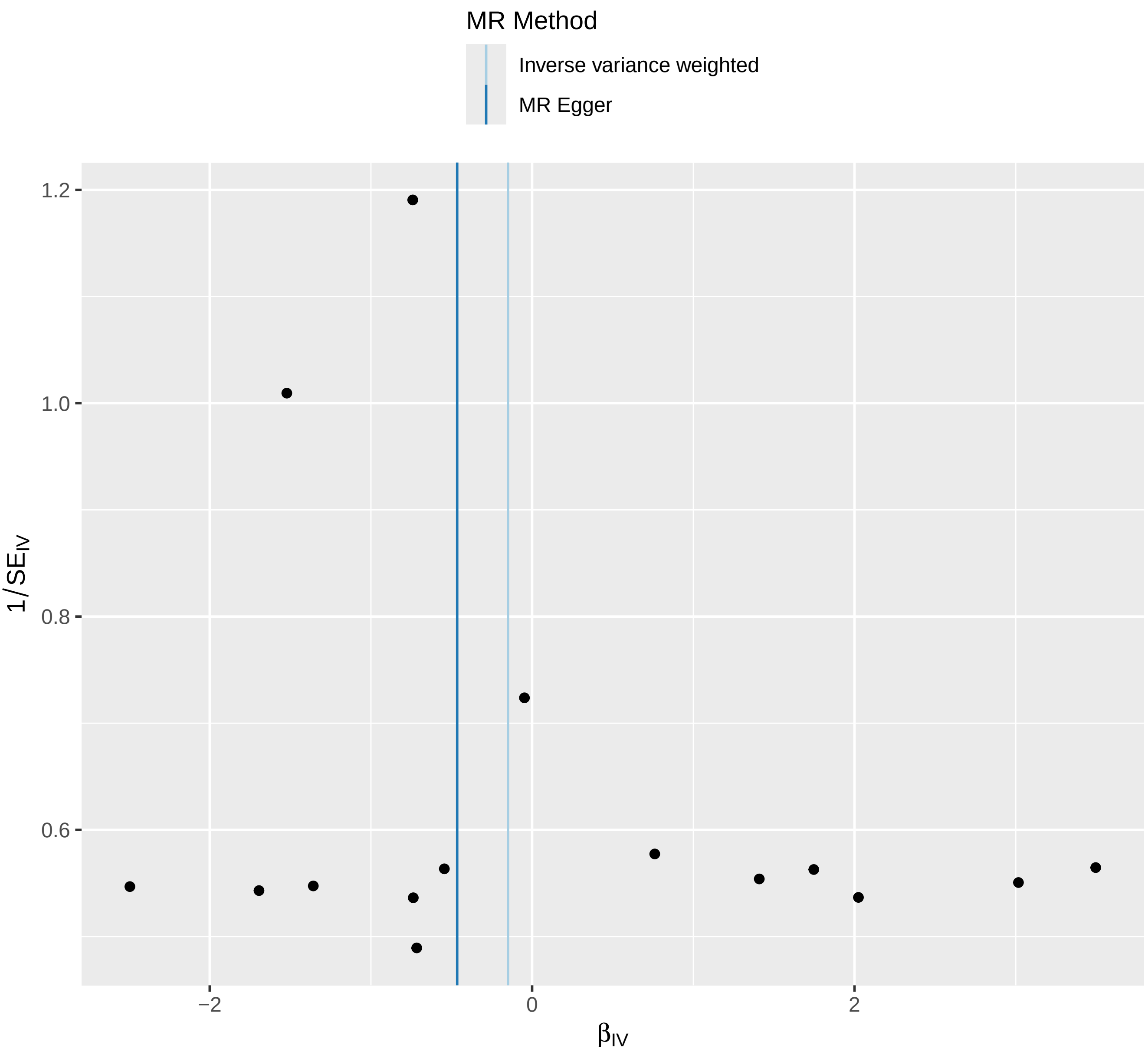
MR Method
Inverse variance weighted
MR Egger
1.2
1.0
0.8
0.6
1/SE_IV
−2
0
2
β_IV

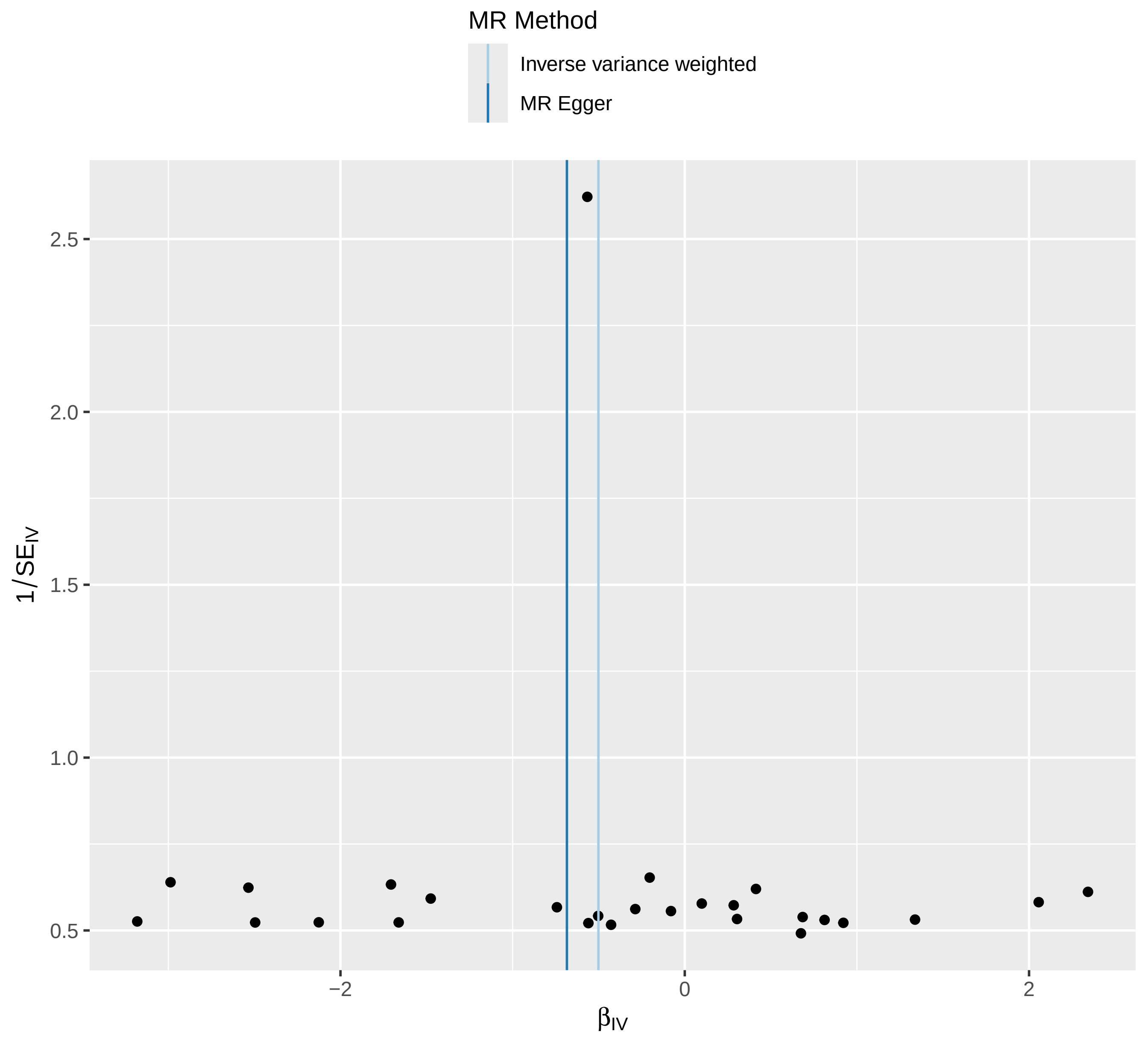

MR Method
Inverse variance weighted
MR Egger
2.5
2.0
1.5
1.0
0.5
1/SE$_{IV}$
−2
0
2
β$_{IV}$

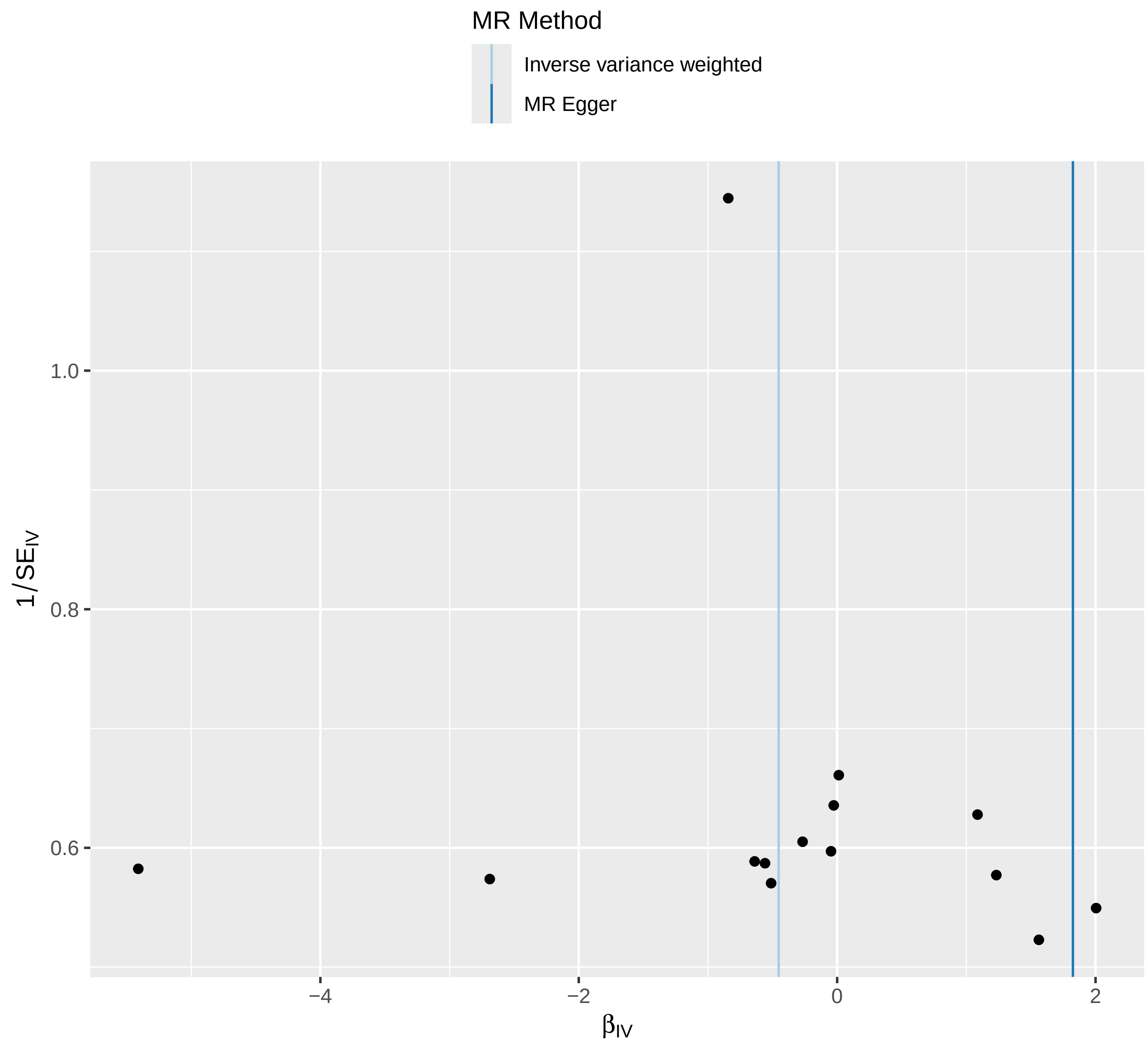
MR Method
Inverse variance weighted
MR Egger
1.0
0.8
0.6
1/SE_IV
−4
−2
0
2
β_IV

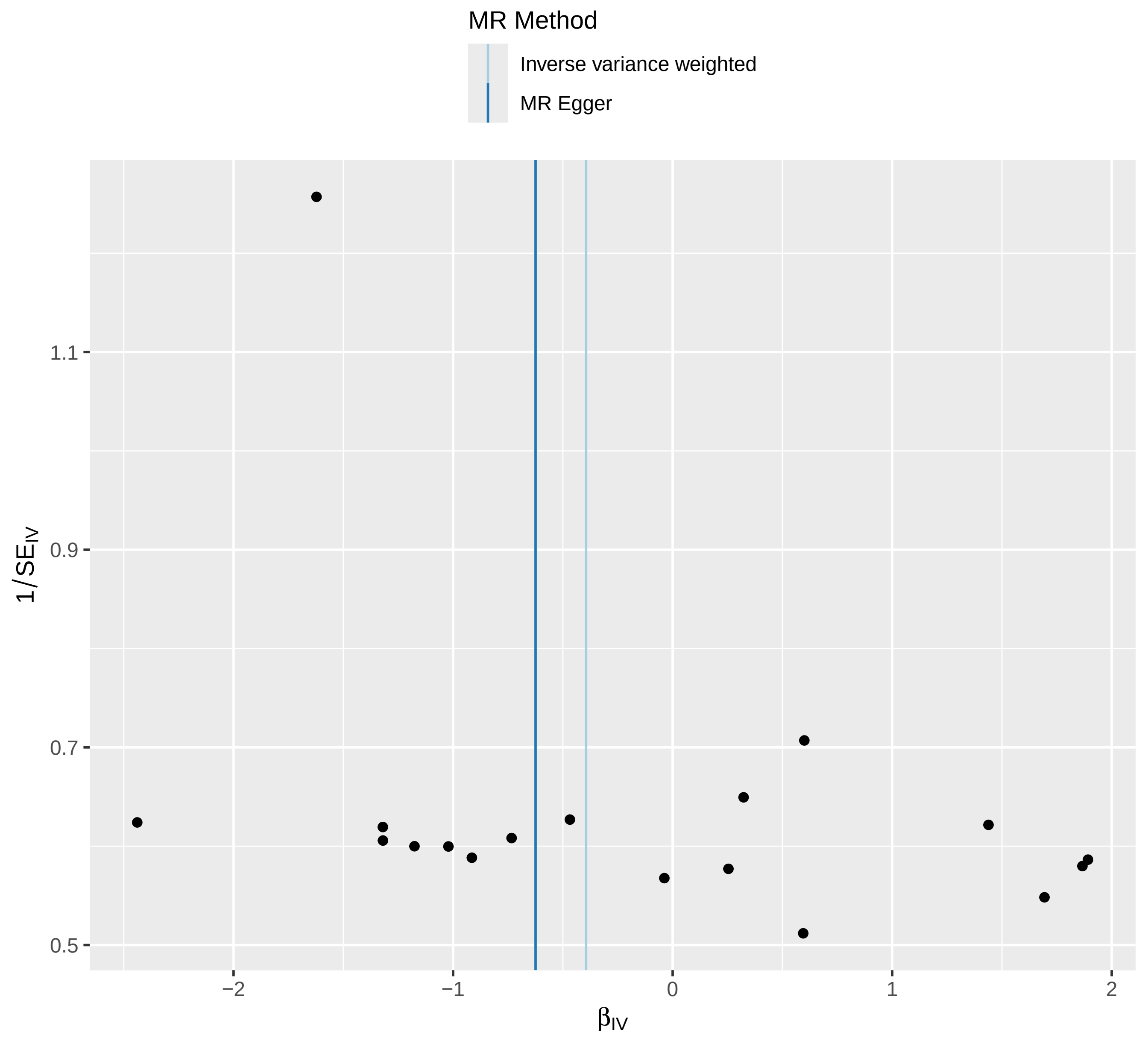
MR Method
Inverse variance weighted
MR Egger
1/SE_IV
1.1
0.9
0.7
0.5
−2
−1
0
1
2
β_IV

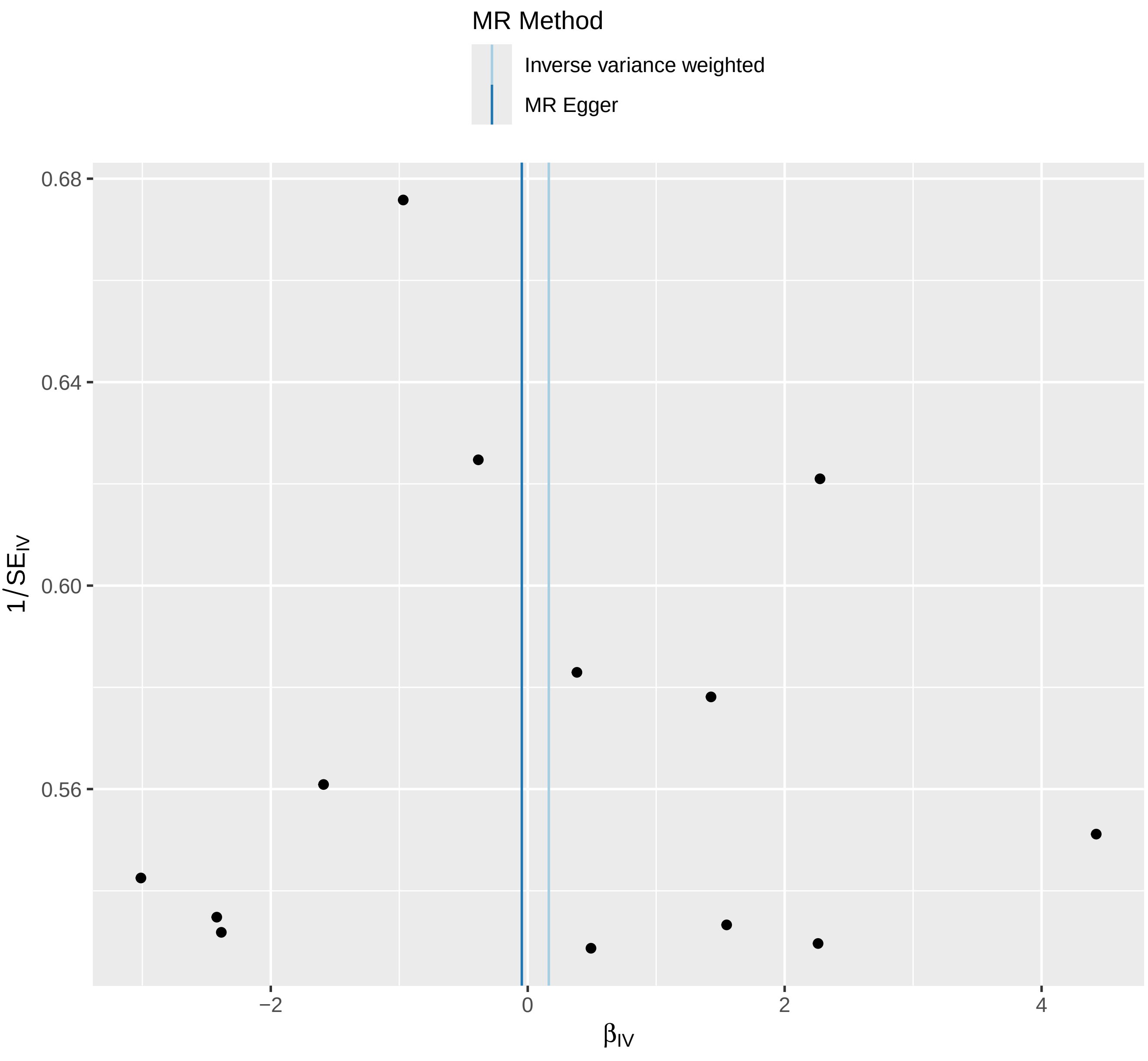
MR Method
Inverse variance weighted
MR Egger
0.68
0.64
0.60
0.56
1/SE_IV
−2
0
2
4
β_IV

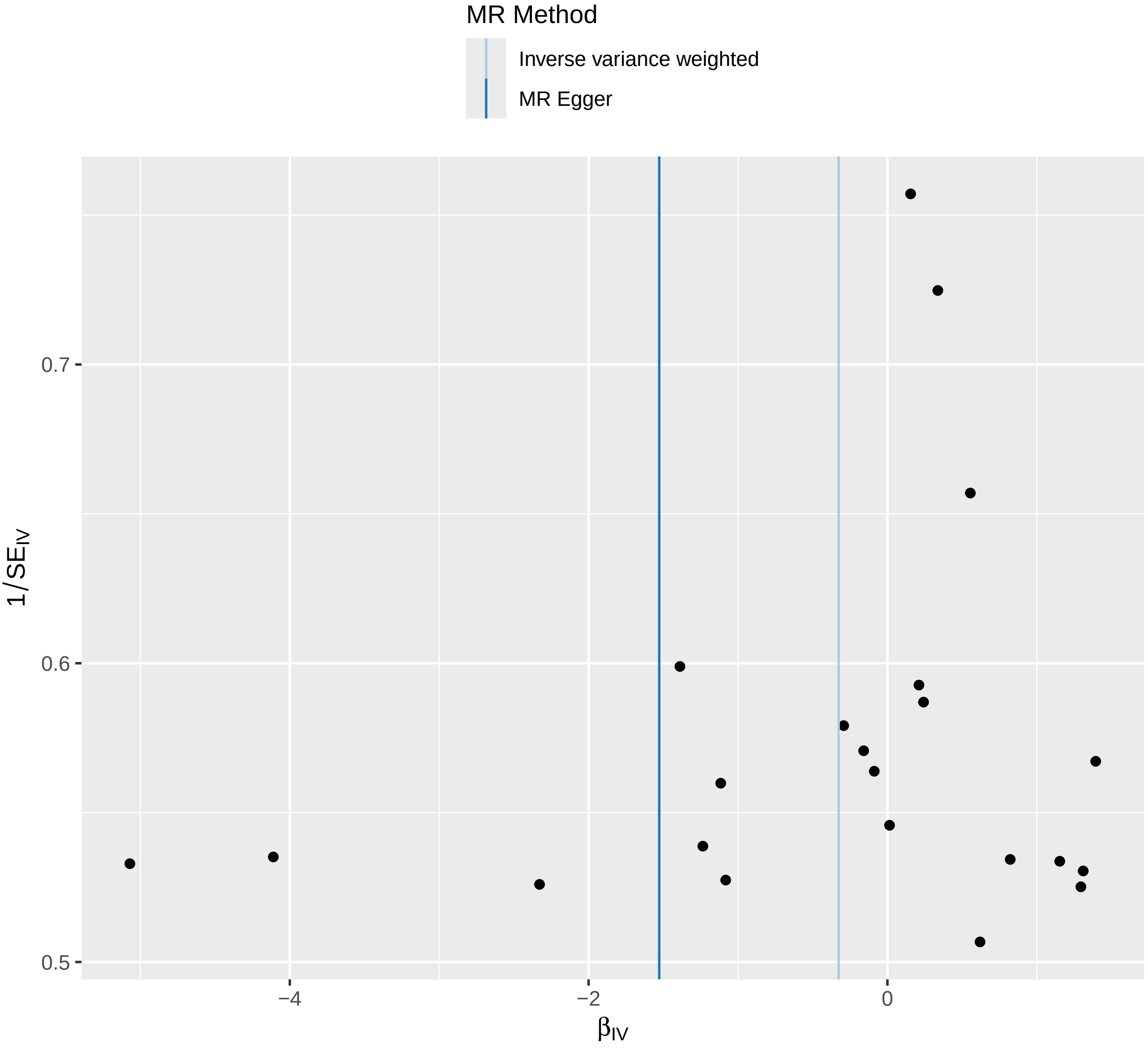

MR Method
Inverse variance weighted
MR Egger
0.7
0.6
0.5
−4
−2
0
1/SE_IV
β_IV

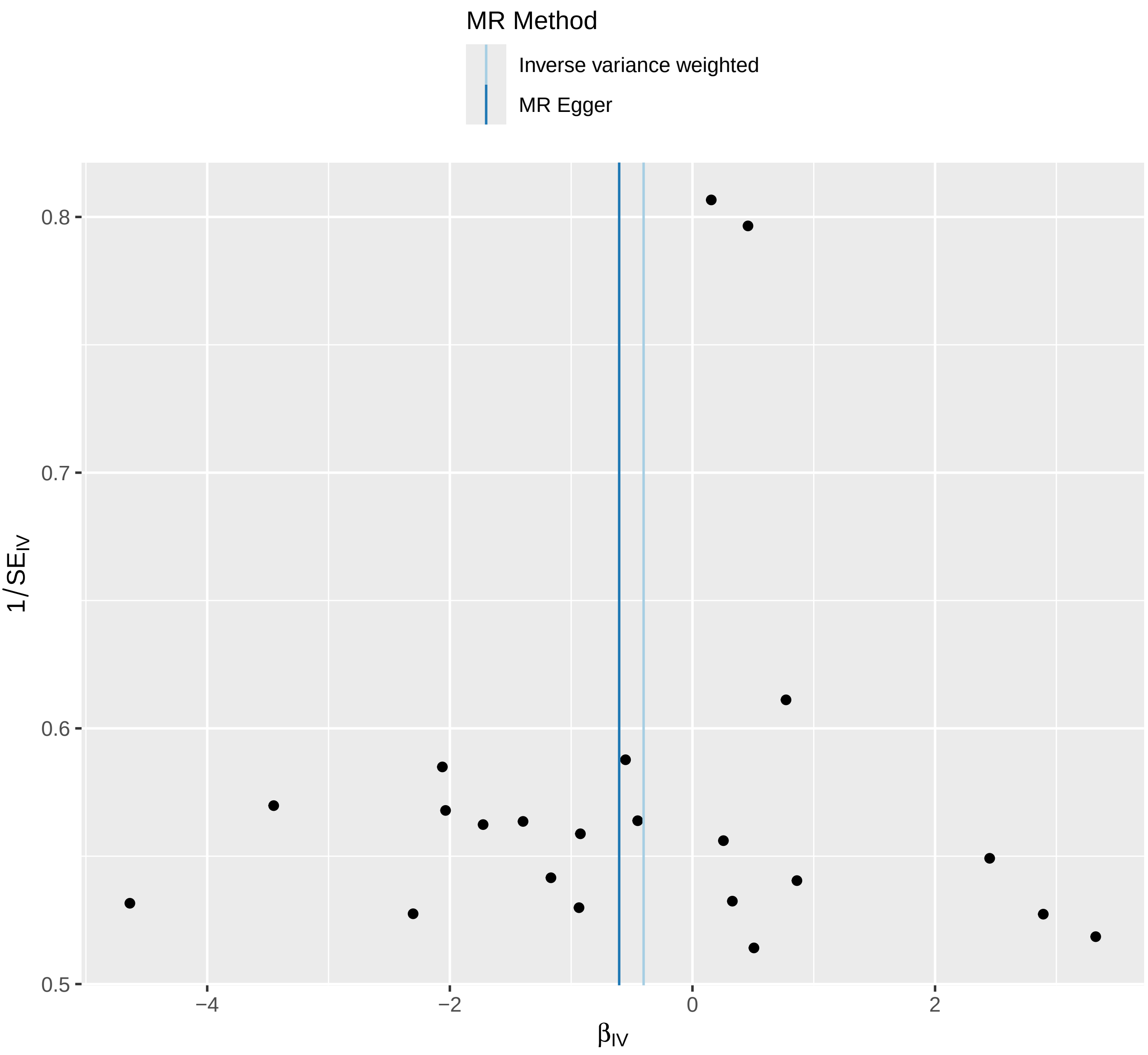
MR Method
Inverse variance weighted
MR Egger
0.8
0.7
0.6
0.5
$1/SE_{IV}$
−4
−2
0
2
β_{IV}

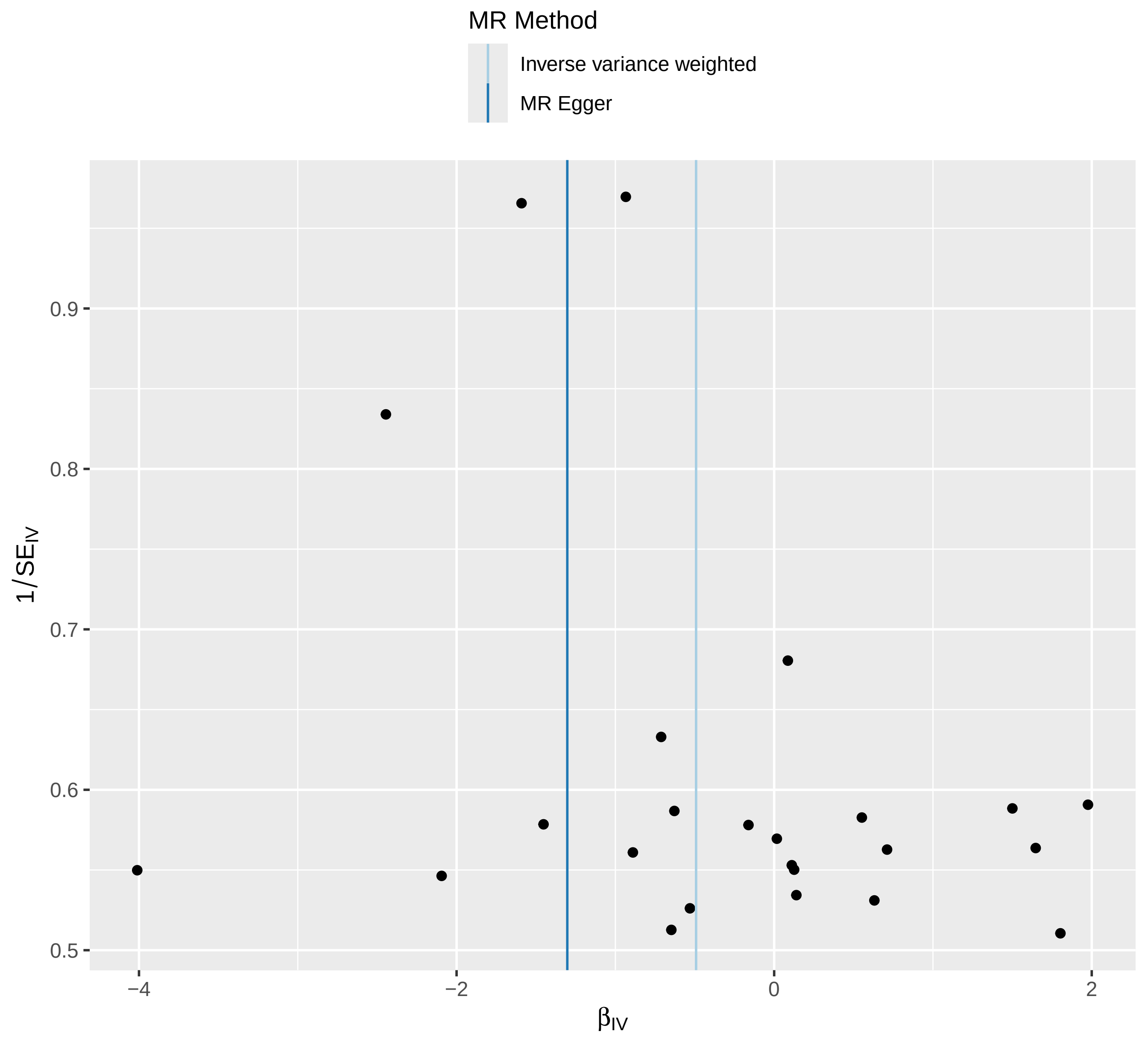
MR Method
Inverse variance weighted
MR Egger
1/SE_IV
0.5
0.6
0.7
0.8
0.9
−4
−2
0
2
β_IV

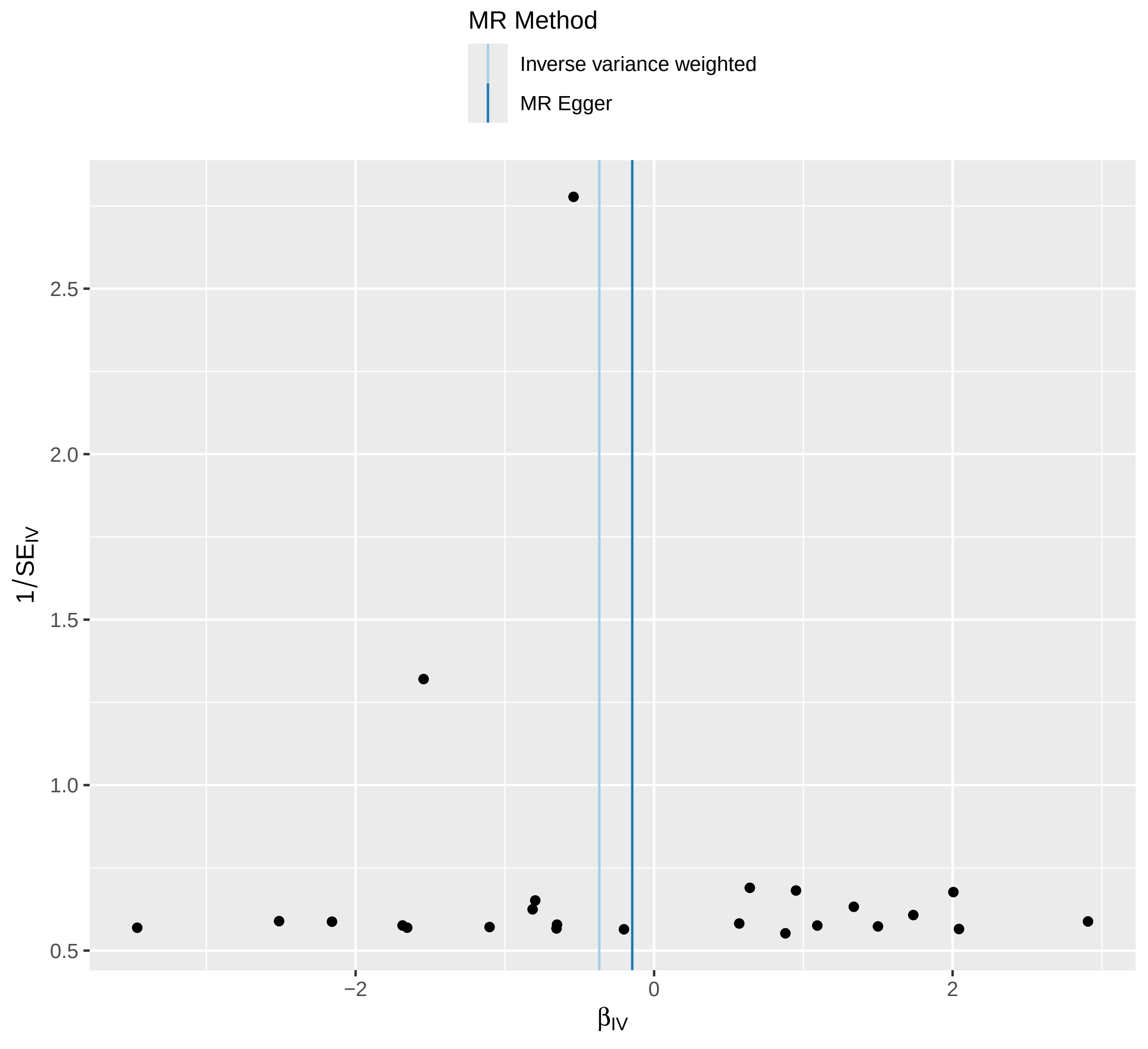

MR Method
Inverse variance weighted
MR Egger
2.5
2.0
1.5
1.0
0.5
$1/SE_{IV}$
−2
0
2
β_{IV}

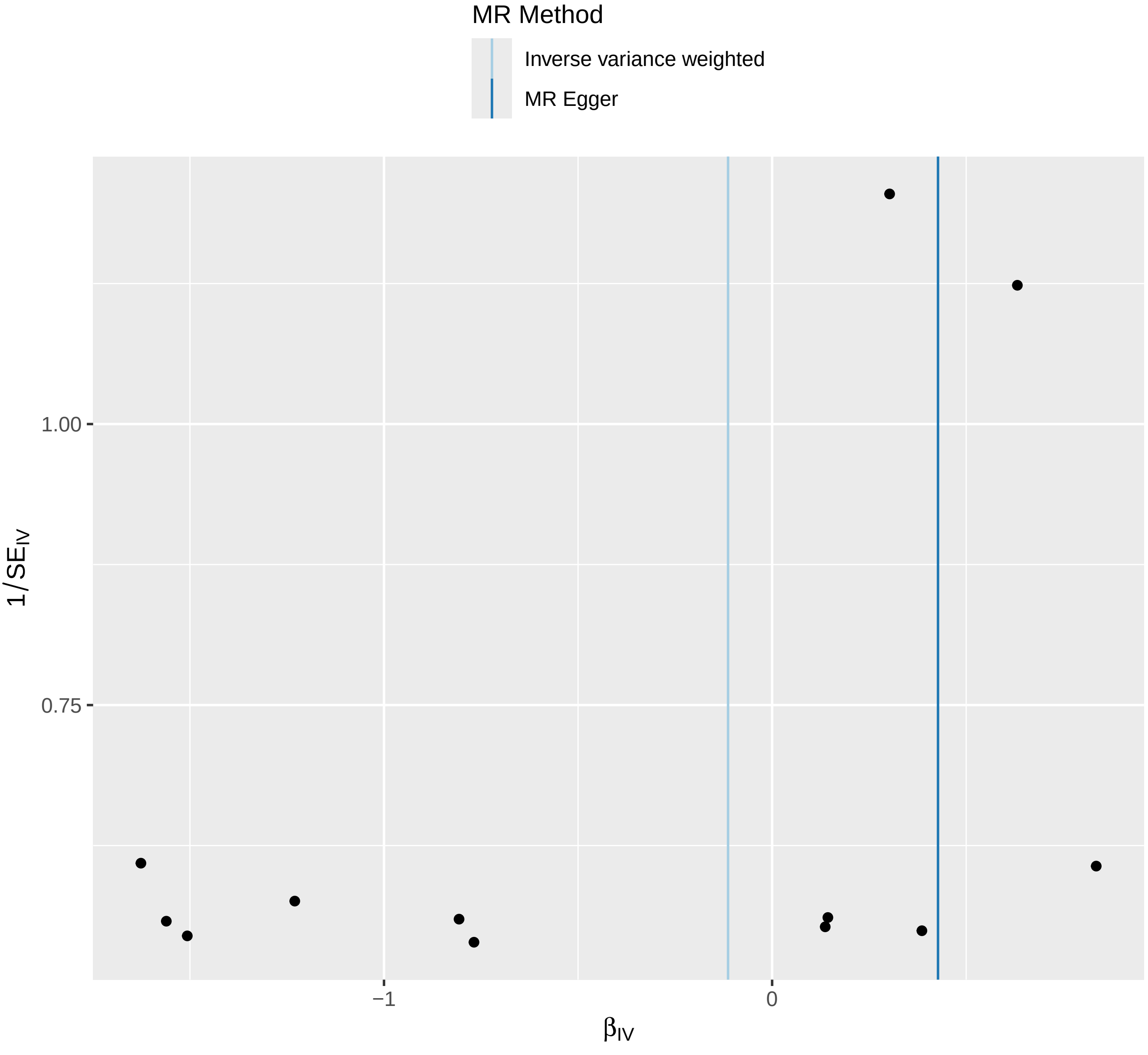

MR Method
Inverse variance weighted
MR Egger
1.00
0.75
1/SE_IV
−1
0
β_IV

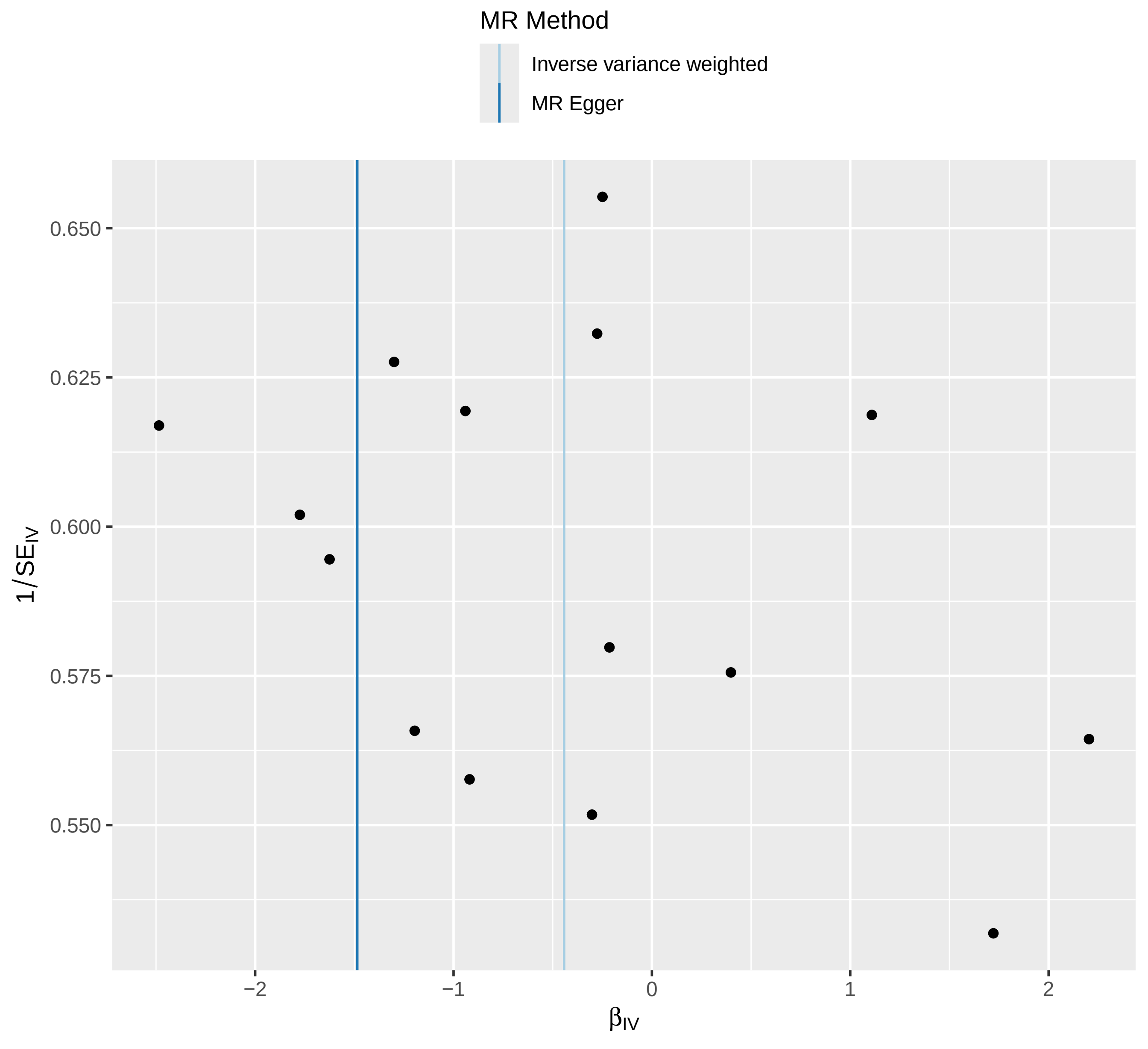
MR Method
Inverse variance weighted
MR Egger
0.650
0.625
0.600
0.575
0.550
1/SE_IV
−2
−1
0
1
2
β_IV

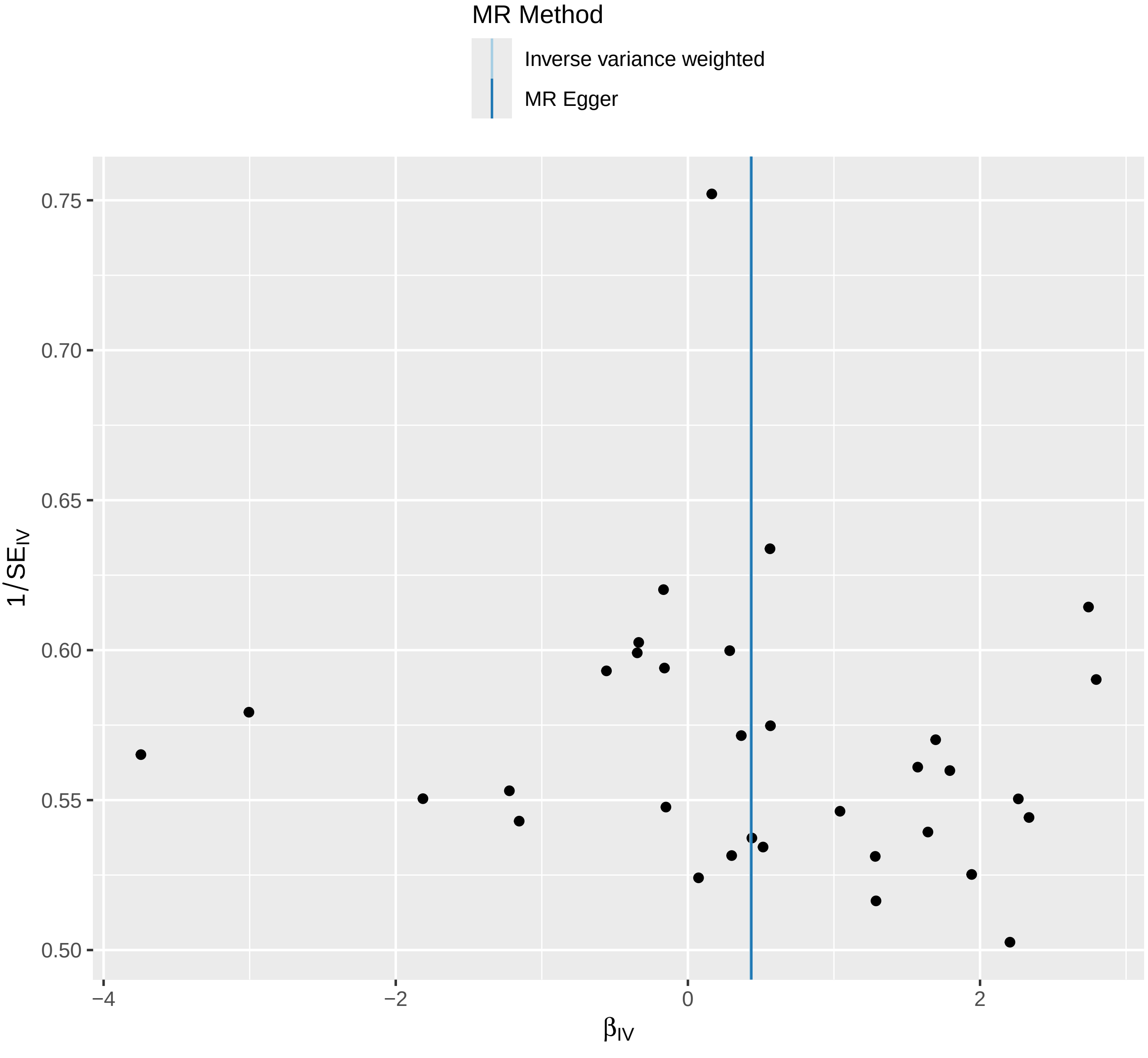
MR Method
Inverse variance weighted
MR Egger
0.75
0.70
0.65
0.60
0.55
0.50
1/SE$_{IV}$
−4
−2
0
2
β$_{IV}$

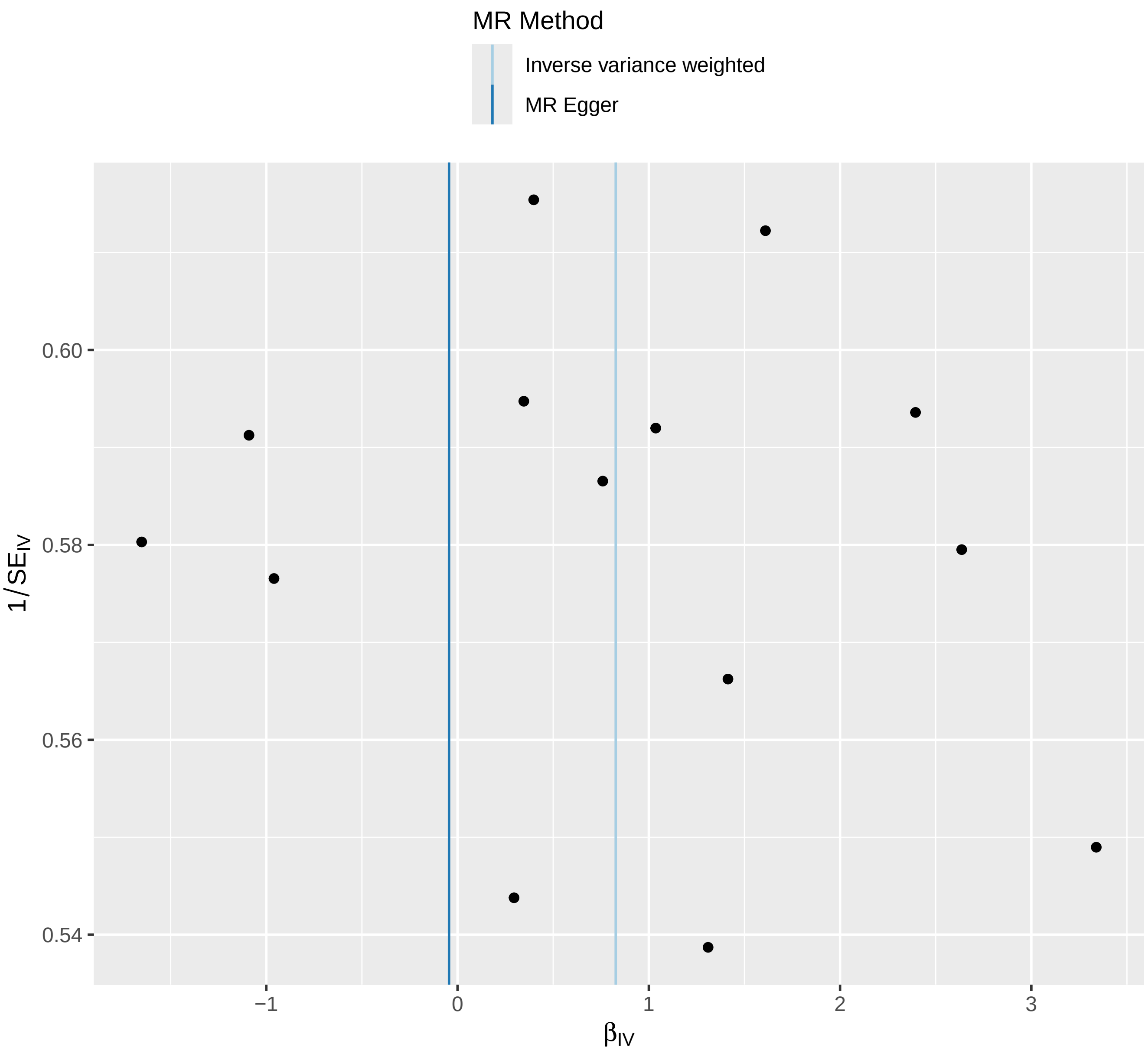
MR Method
Inverse variance weighted
MR Egger
0.60
0.58
0.56
0.54
1/SE$_{IV}$
−1
0
1
2
3
β$_{IV}$

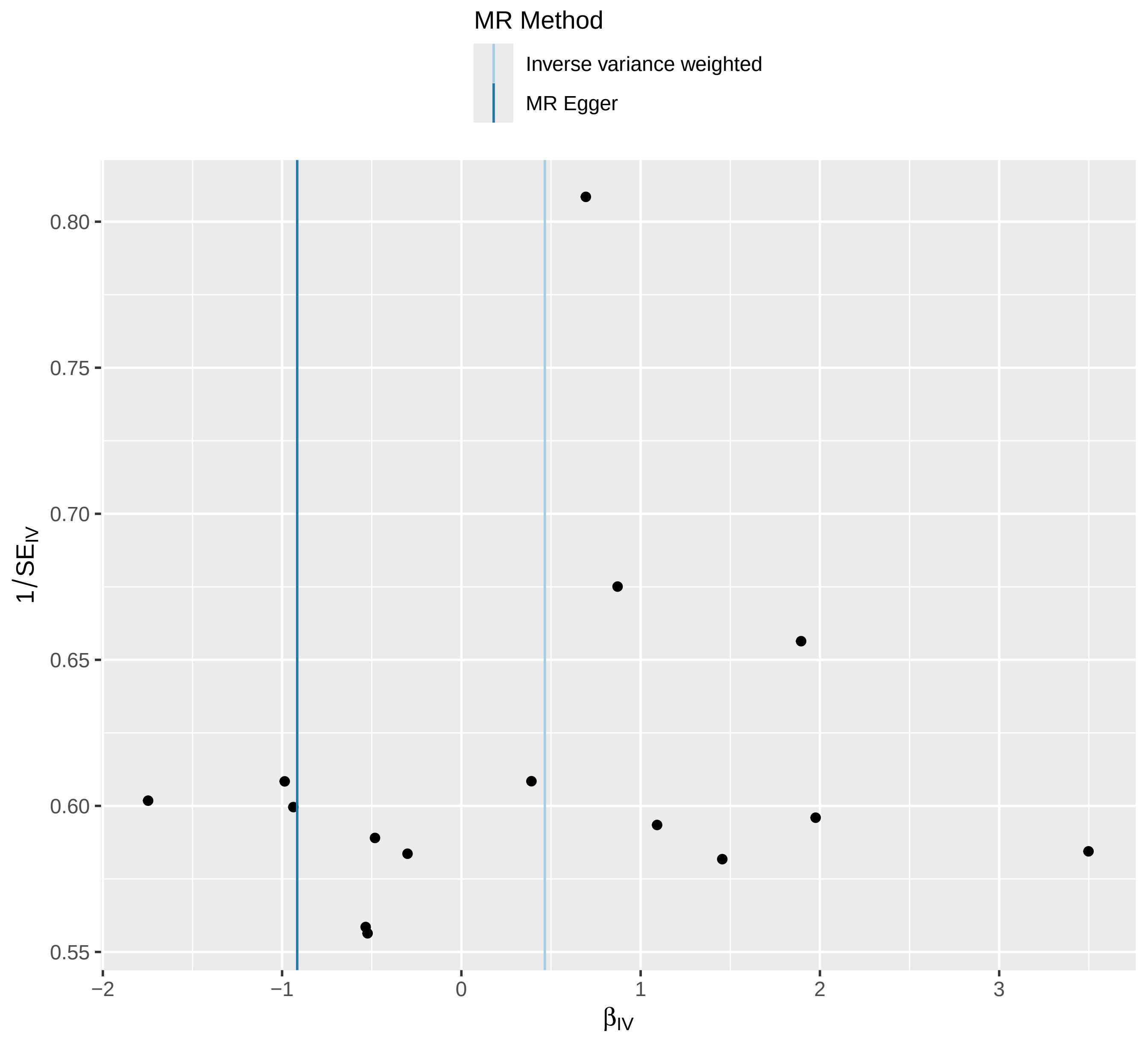
MR Method
Inverse variance weighted
MR Egger
0.80
0.75
0.70
0.65
0.60
0.55
1/SE_IV
−2
−1
0
1
2
3
β_IV

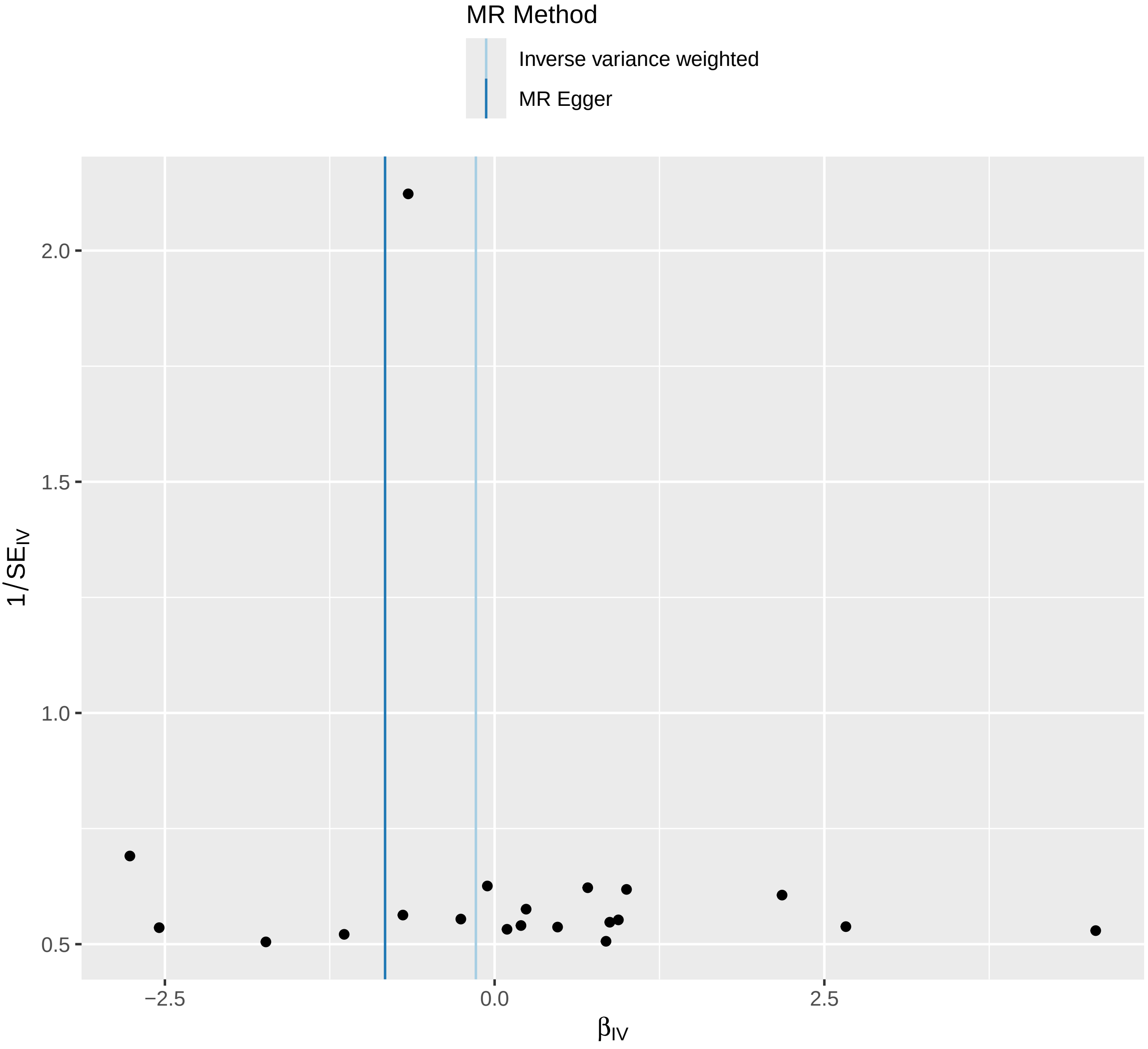
MR Method
Inverse variance weighted
MR Egger
2.0
1.5
1.0
0.5
$1/SE_{IV}$
−2.5
0.0
2.5
β_{IV}

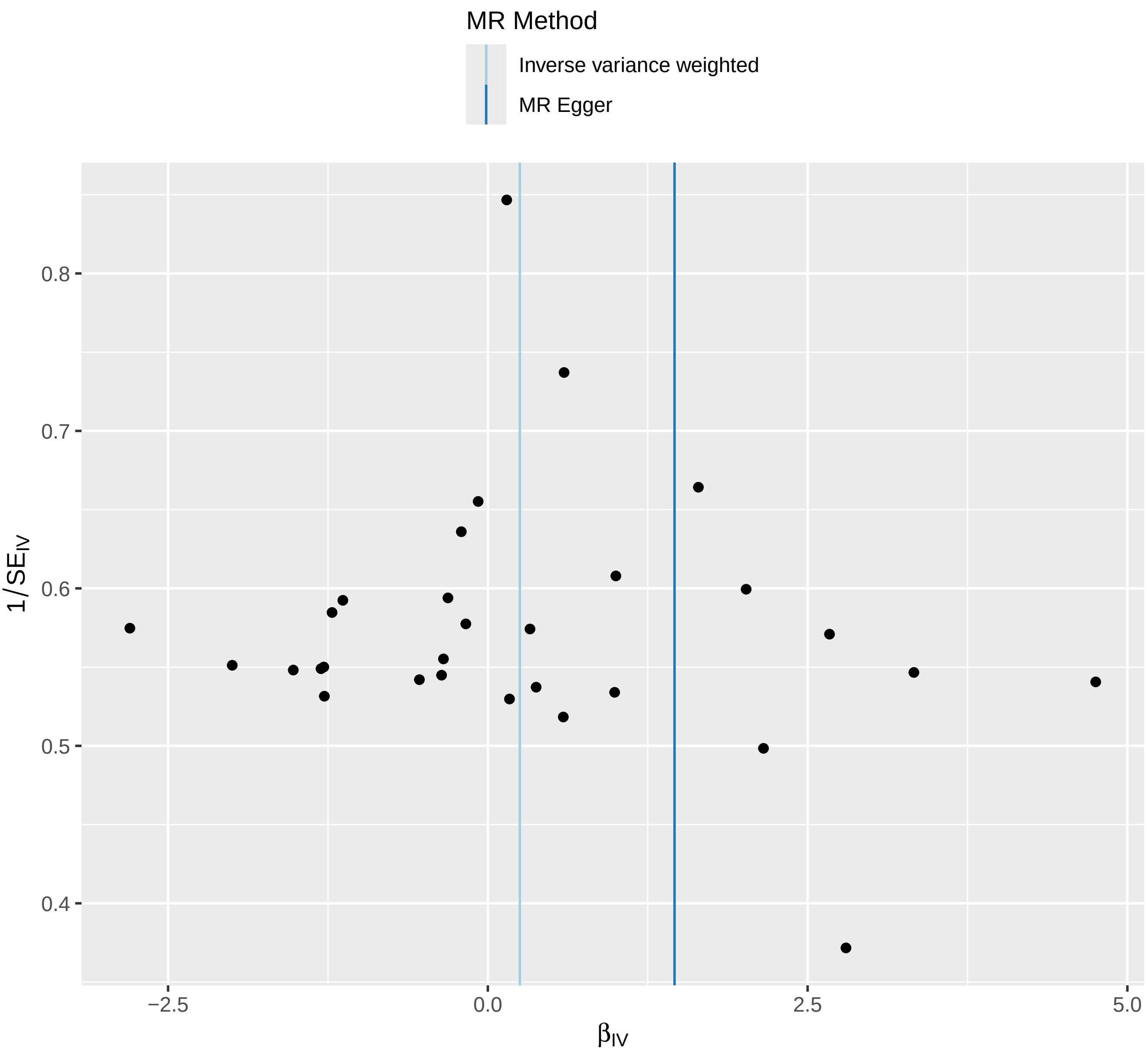
MR Method
Inverse variance weighted
MR Egger
0.8
0.7
0.6
0.5
0.4
1/SE_IV
−2.5
0.0
2.5
5.0
β_IV

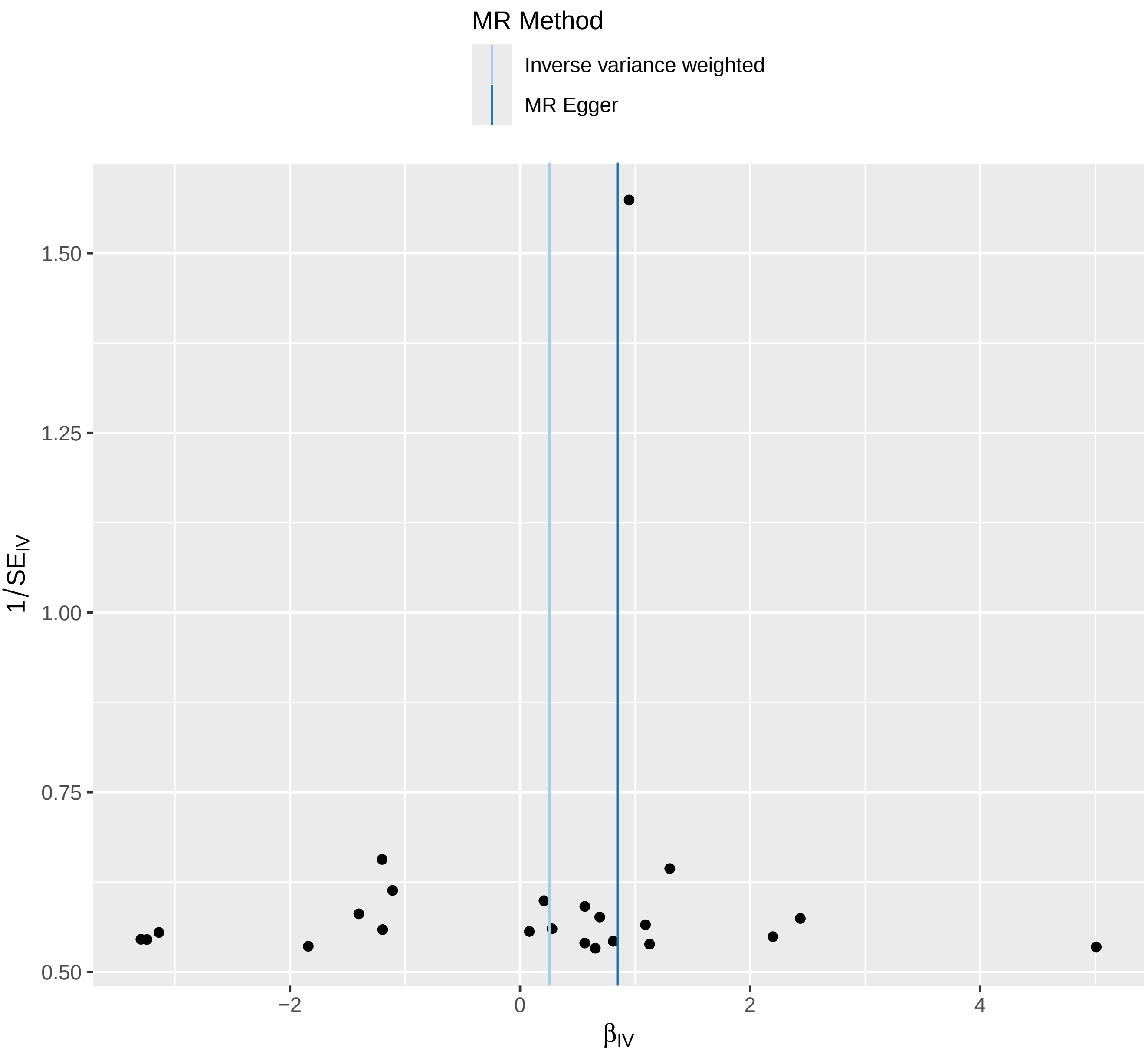
MR Method
Inverse variance weighted
MR Egger
1.50
1.25
1.00
0.75
0.50
1/SE_IV
−2
0
2
4
β_IV

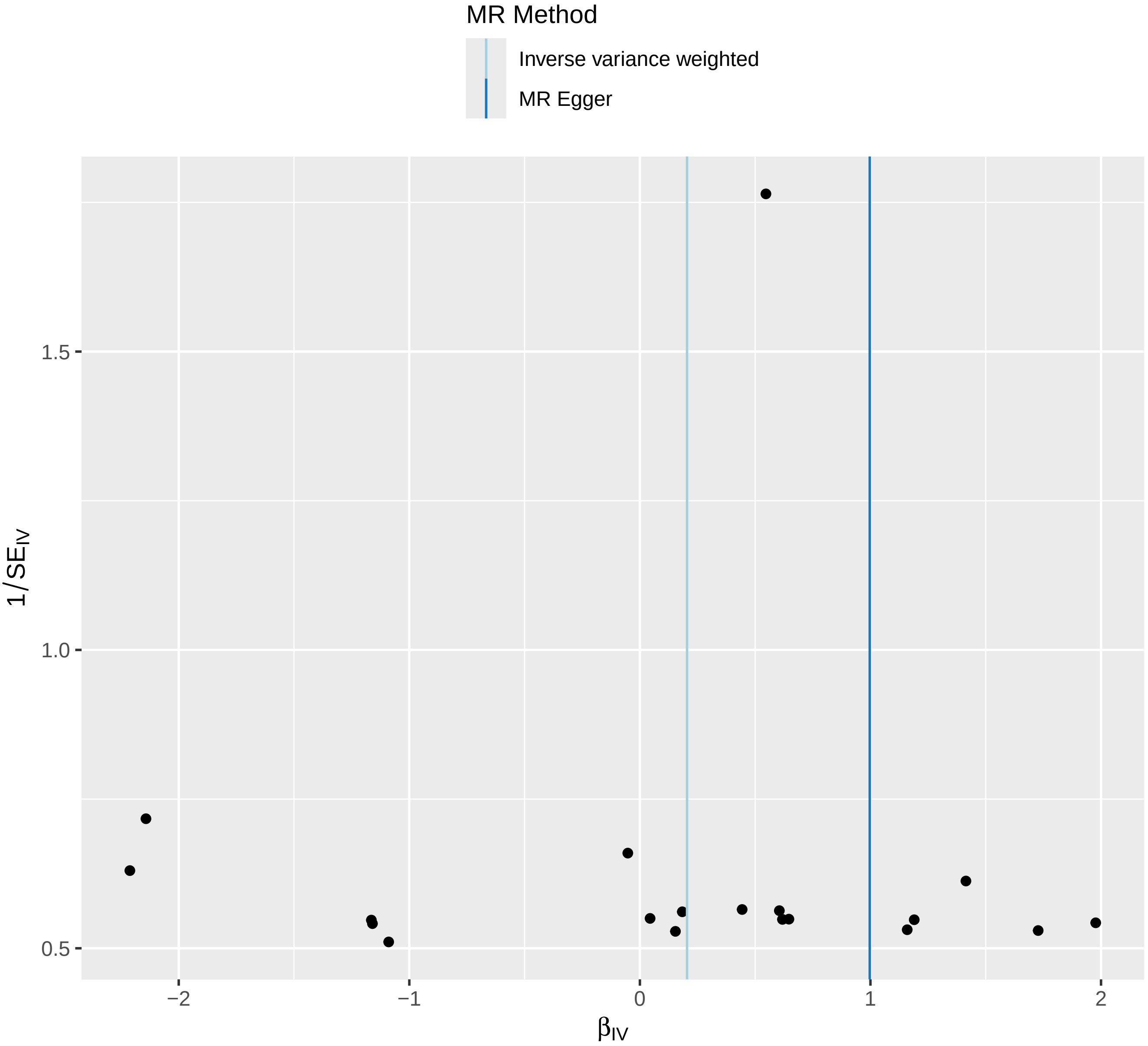
MR Method
Inverse variance weighted
MR Egger
1.5
1.0
0.5
1/SE_IV
−2
−1
0
1
2
β_IV

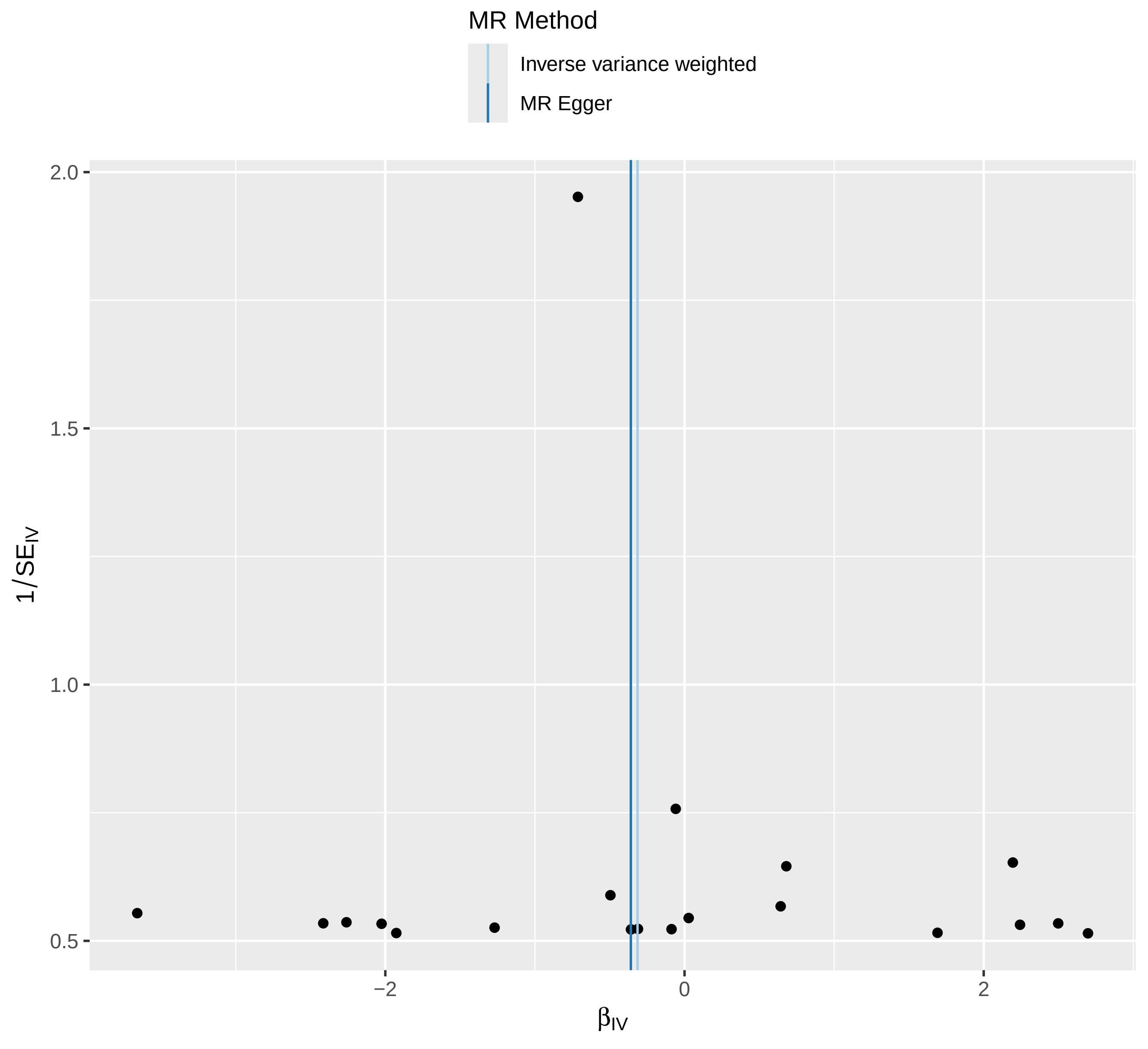

MR Method
Inverse variance weighted
MR Egger
2.0
1.5
1.0
0.5
1/SE_IV
−2
0
2
β_IV

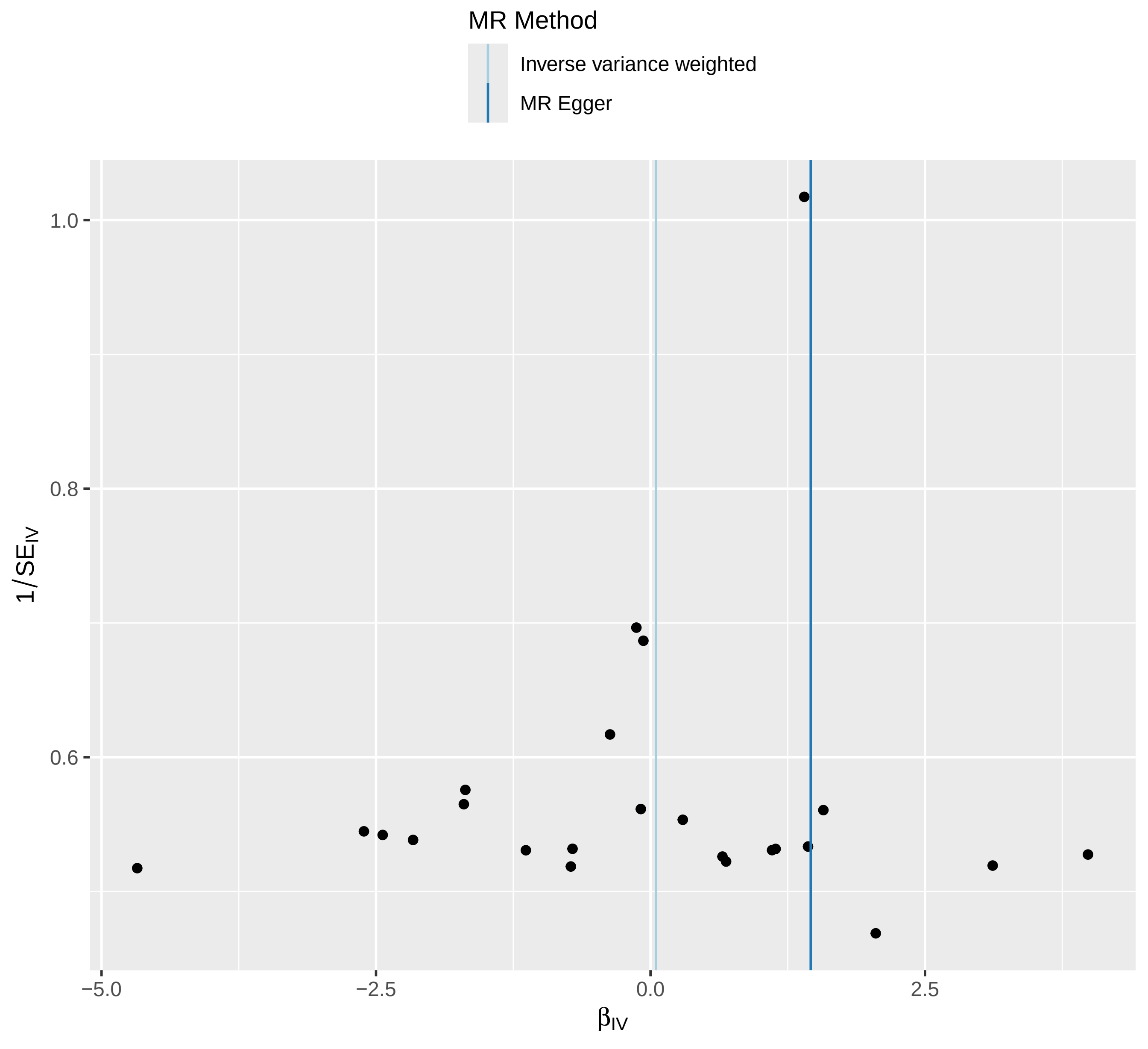
MR Method
Inverse variance weighted
MR Egger
1.0
0.8
0.6
1/SE_IV
−5.0
−2.5
0.0
2.5
β_IV

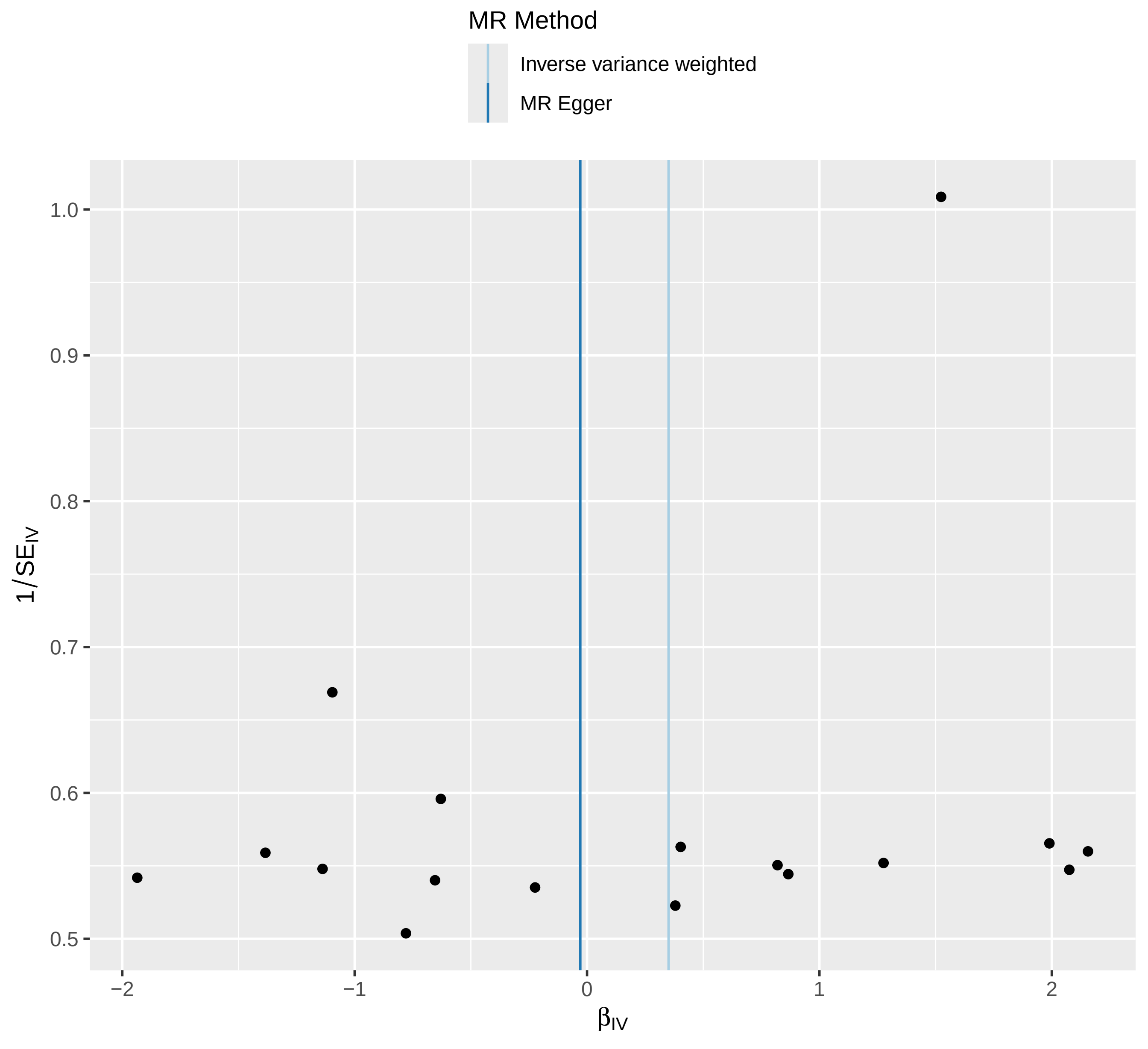
MR Method
Inverse variance weighted
MR Egger
1.0
0.9
0.8
0.7
0.6
0.5
1/SE_IV
−2
−1
0
1
2
β_IV

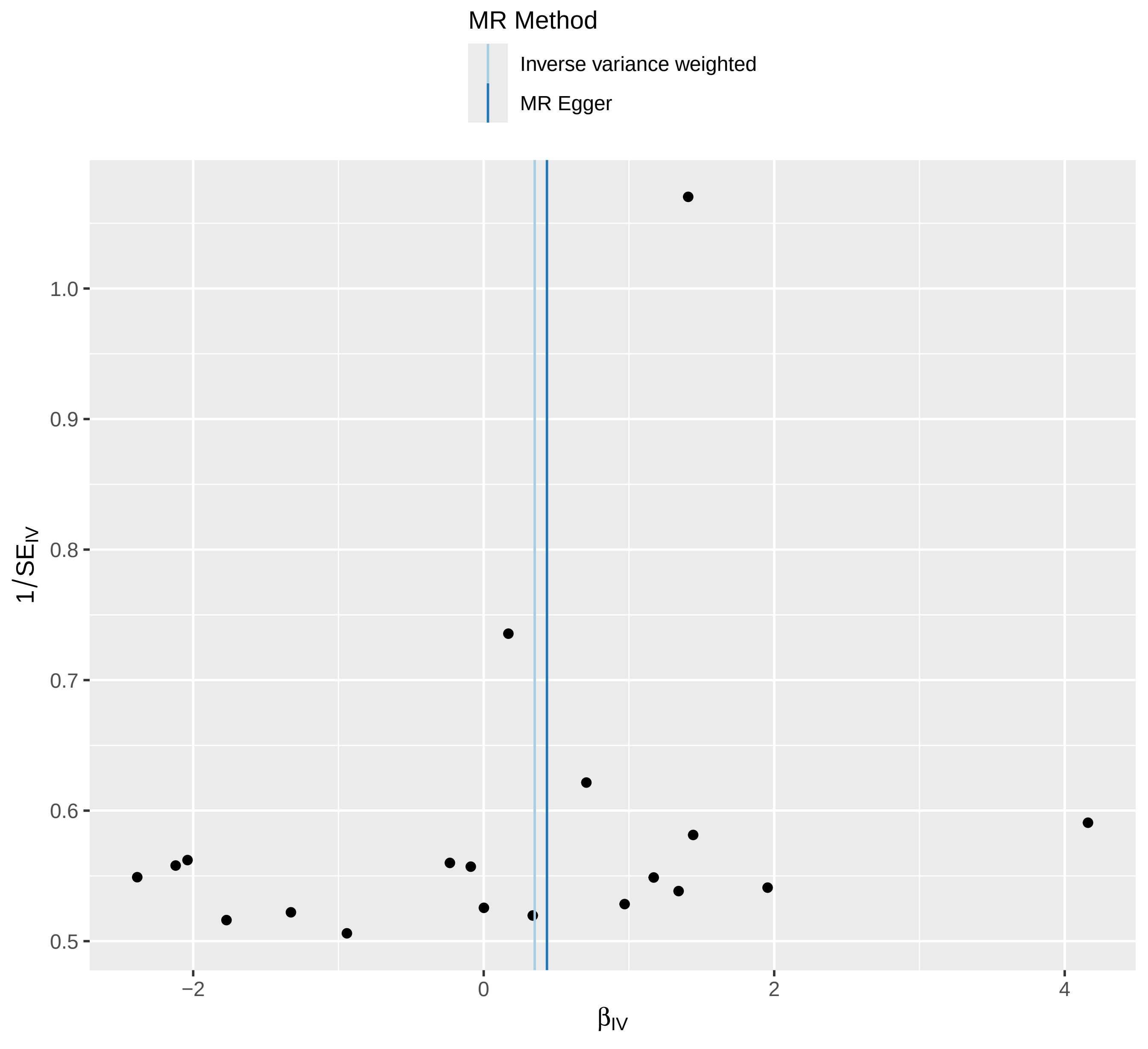
MR Method
Inverse variance weighted
MR Egger
1/SE_IV
β_IV
1.0
0.9
0.8
0.7
0.6
0.5
−2
0
2
4

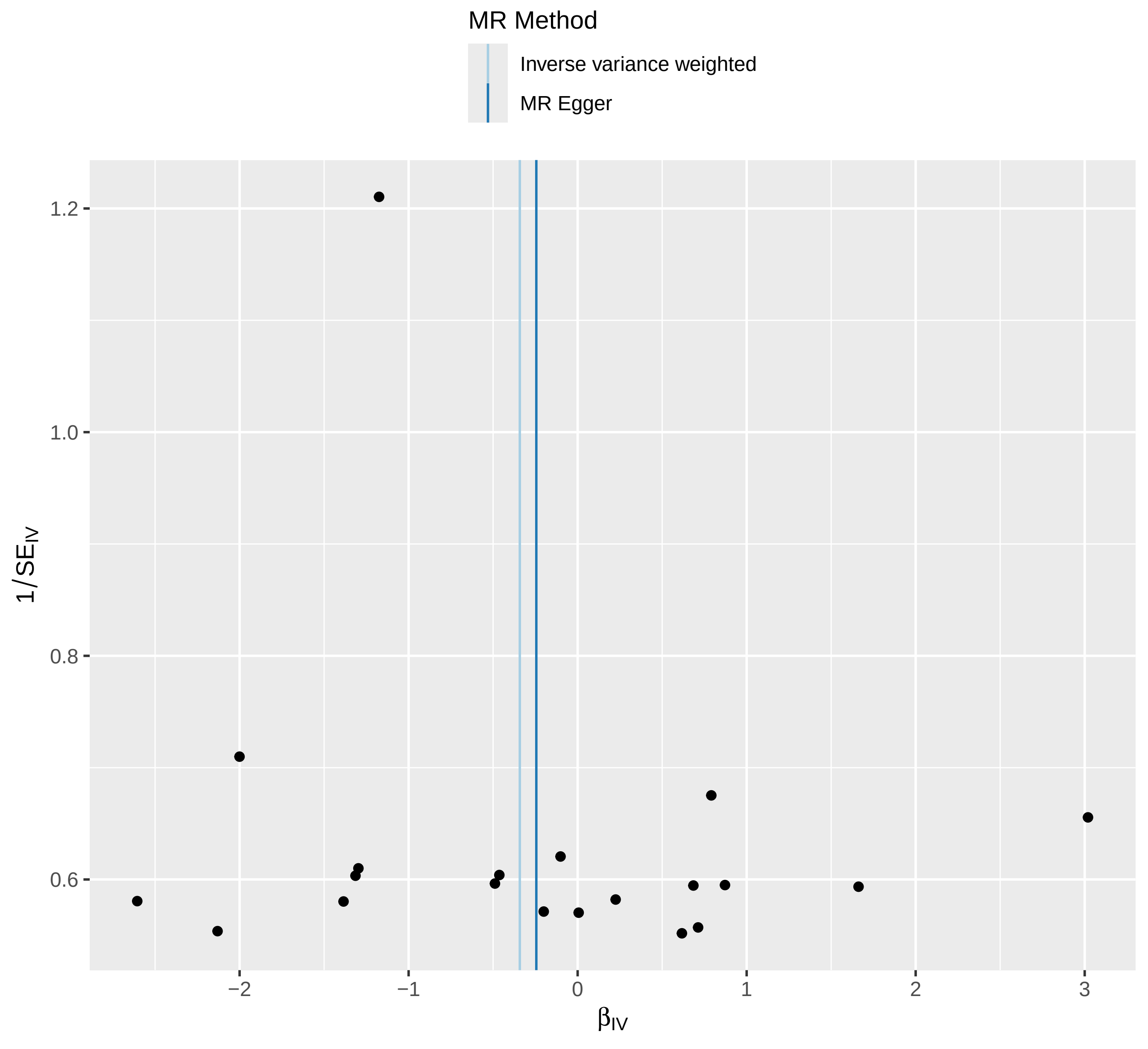
MR Method
Inverse variance weighted
MR Egger
1.2
1.0
0.8
0.6
1/SE_IV
−2
−1
0
1
2
3
β_IV

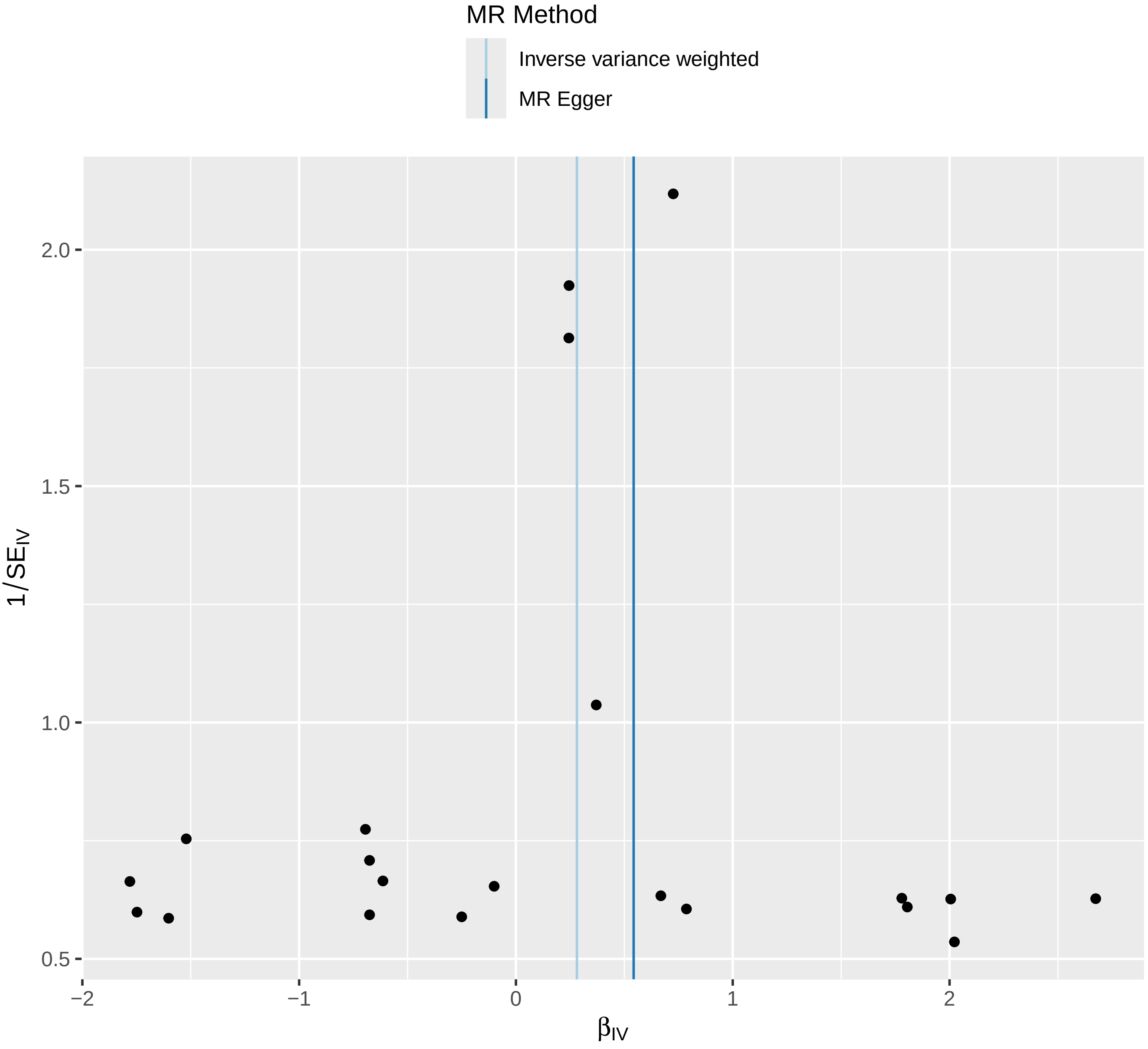
MR Method
Inverse variance weighted
MR Egger
2.0
1.5
1.0
0.5
1/SE_IV
−2
−1
0
1
2
β_IV

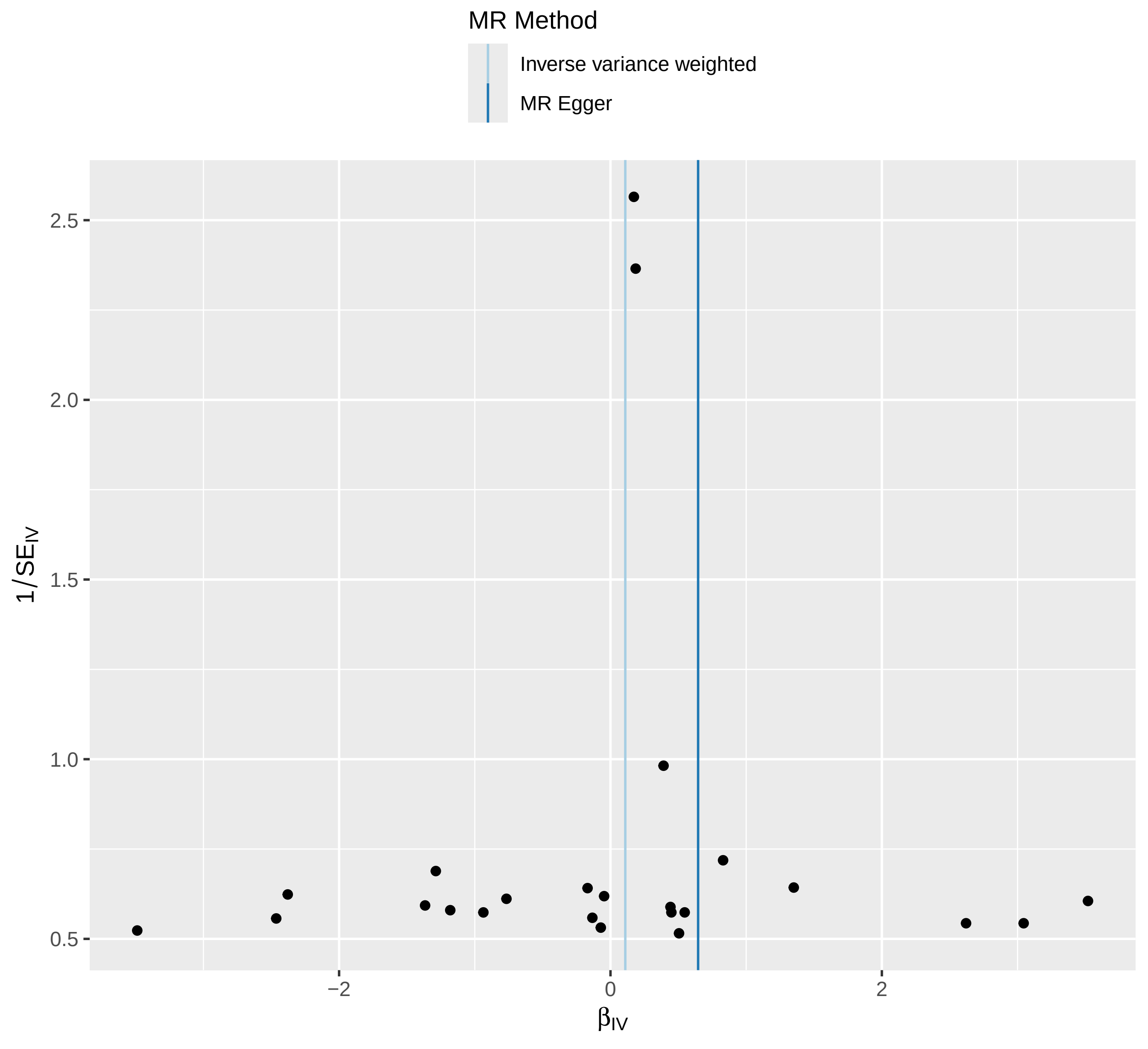

MR Method
Inverse variance weighted
MR Egger
2.5
2.0
1.5
1.0
0.5
−2
0
2
$1/SE_{IV}$
β_{IV}

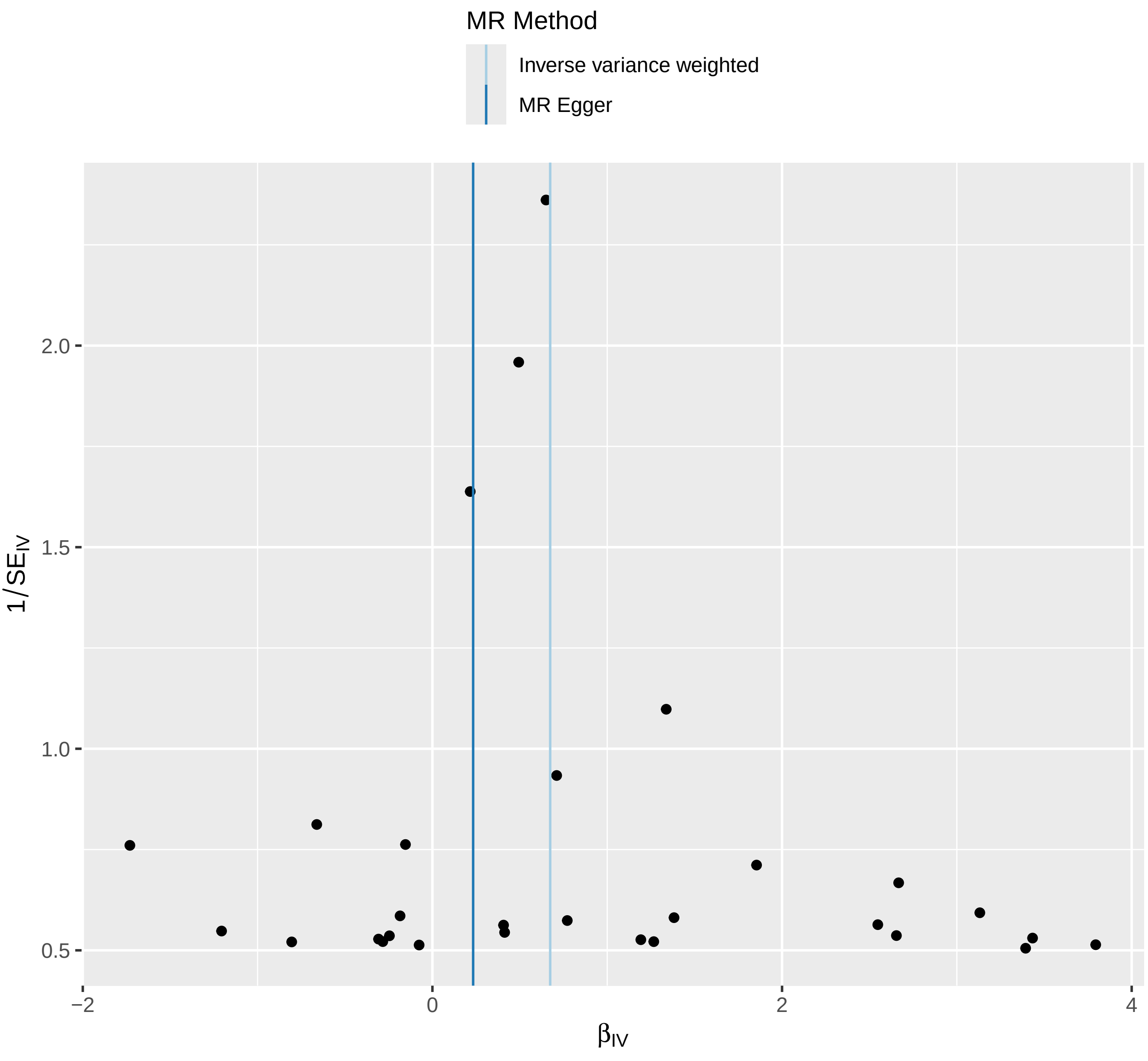
MR Method
Inverse variance weighted
MR Egger
$1/SE_{IV}$
2.0
1.5
1.0
0.5
−2
0
2
4
β_{IV}

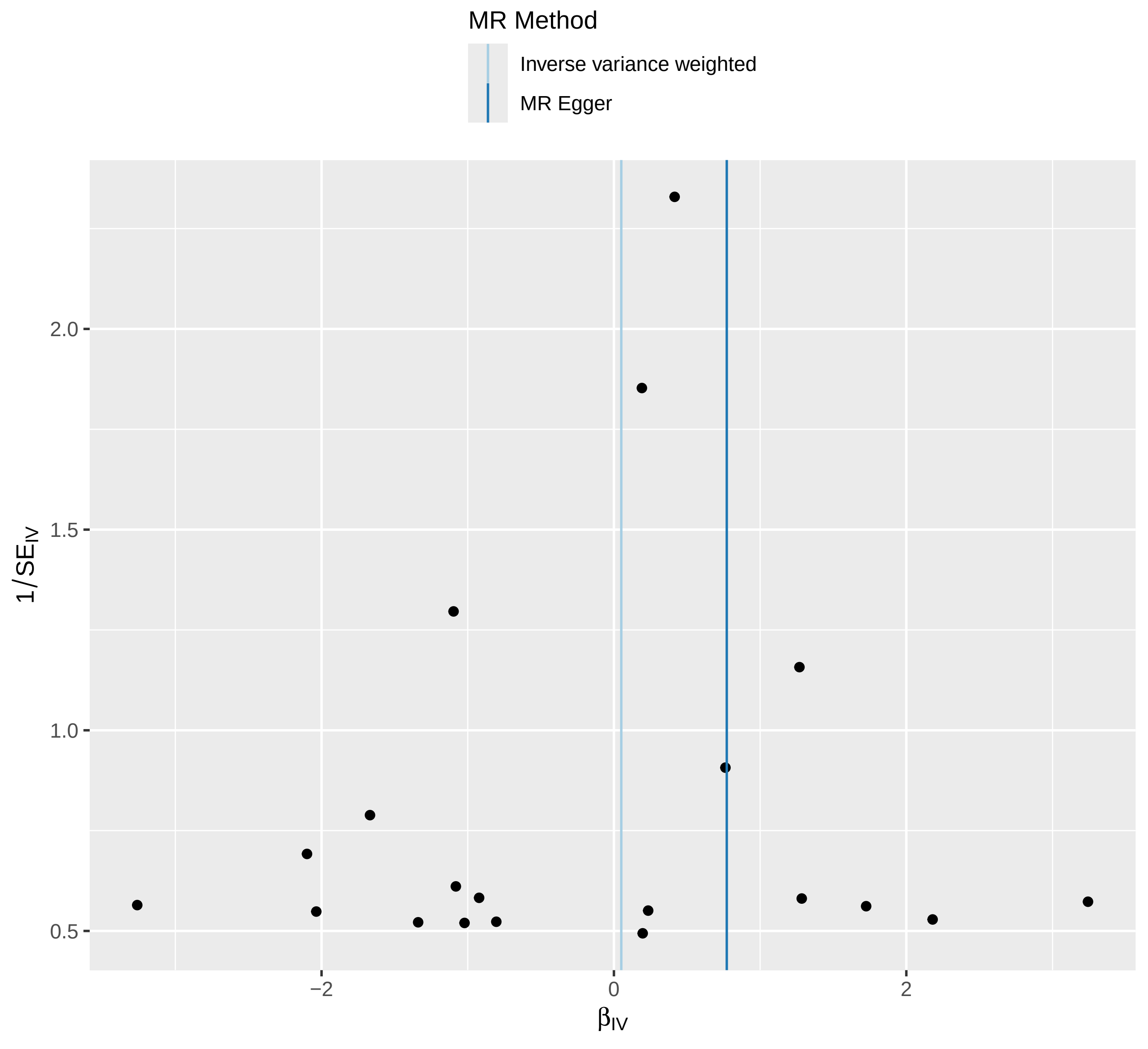
MR Method
Inverse variance weighted
MR Egger
2.0
1.5
1.0
0.5
−2
0
2
1/SE_IV
β_IV

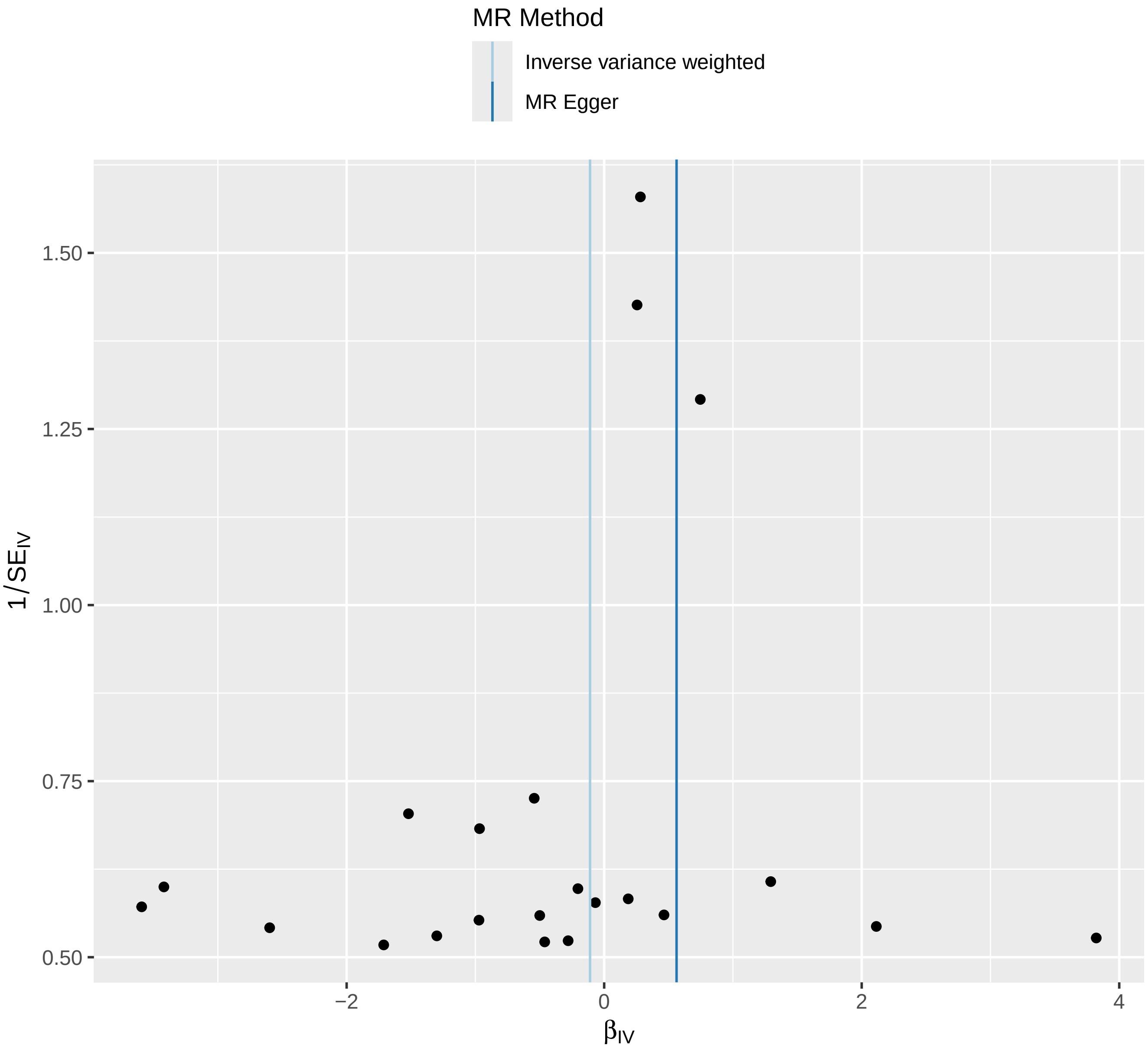
MR Method
Inverse variance weighted
MR Egger
1.50
1.25
1.00
0.75
0.50
$1/SE_{IV}$
−2
0
2
4
β_{IV}

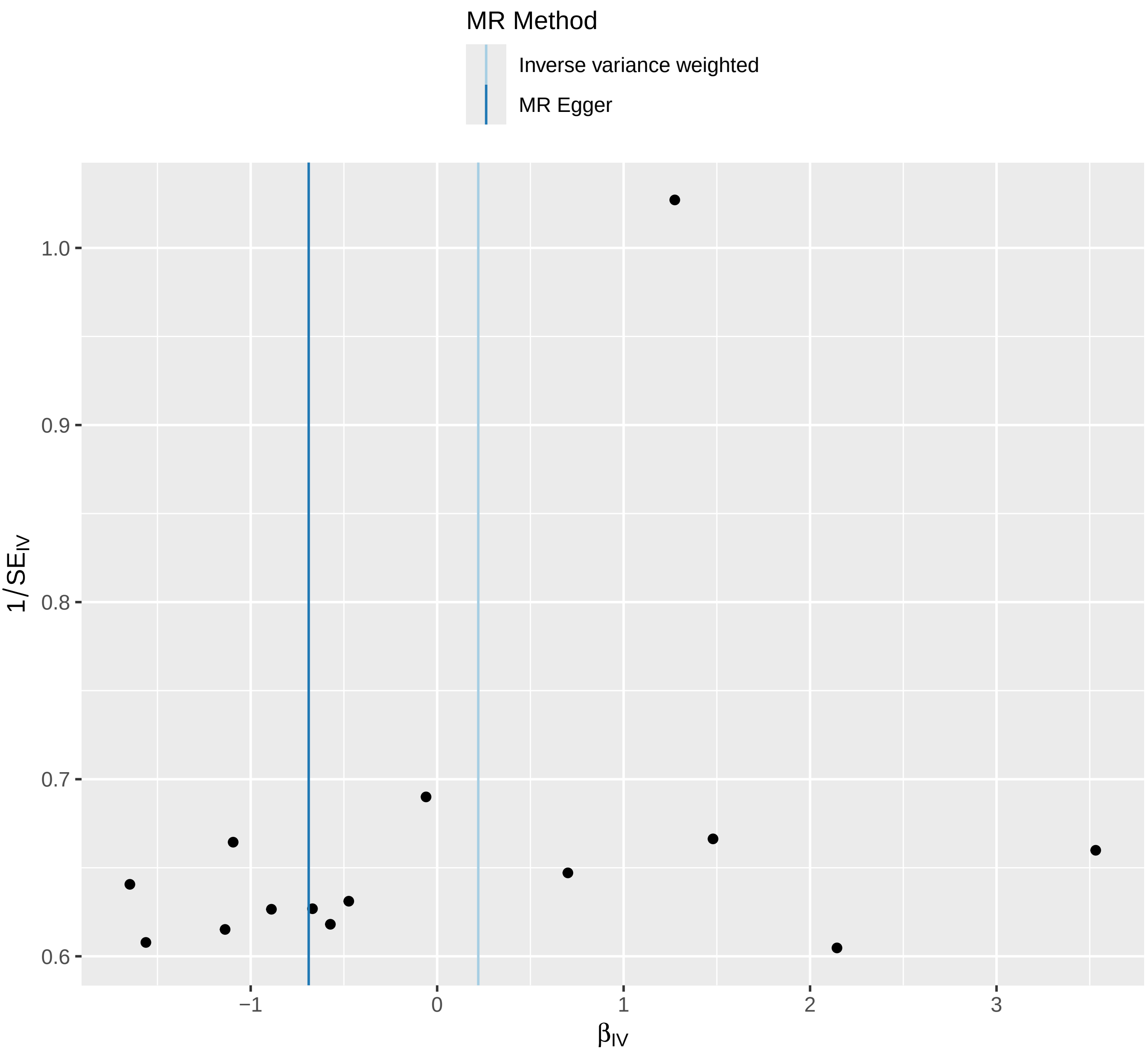
MR Method
Inverse variance weighted
MR Egger
1.0
0.9
0.8
0.7
0.6
1/SE_IV
−1
0
1
2
3
β_IV

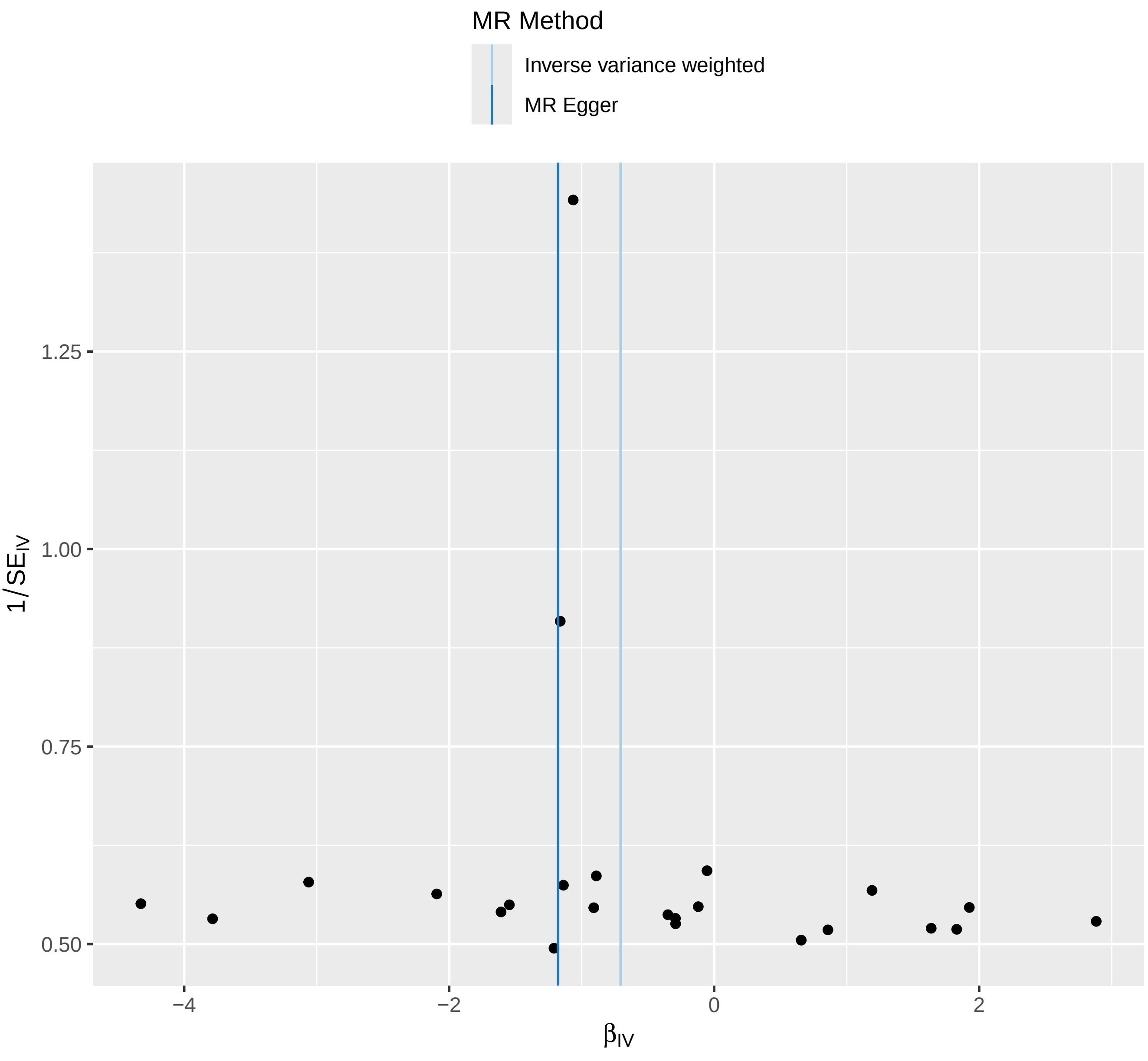

MR Method
Inverse variance weighted
MR Egger
1.25
1.00
0.75
0.50
1/SE_IV
−4
−2
0
2
β_IV

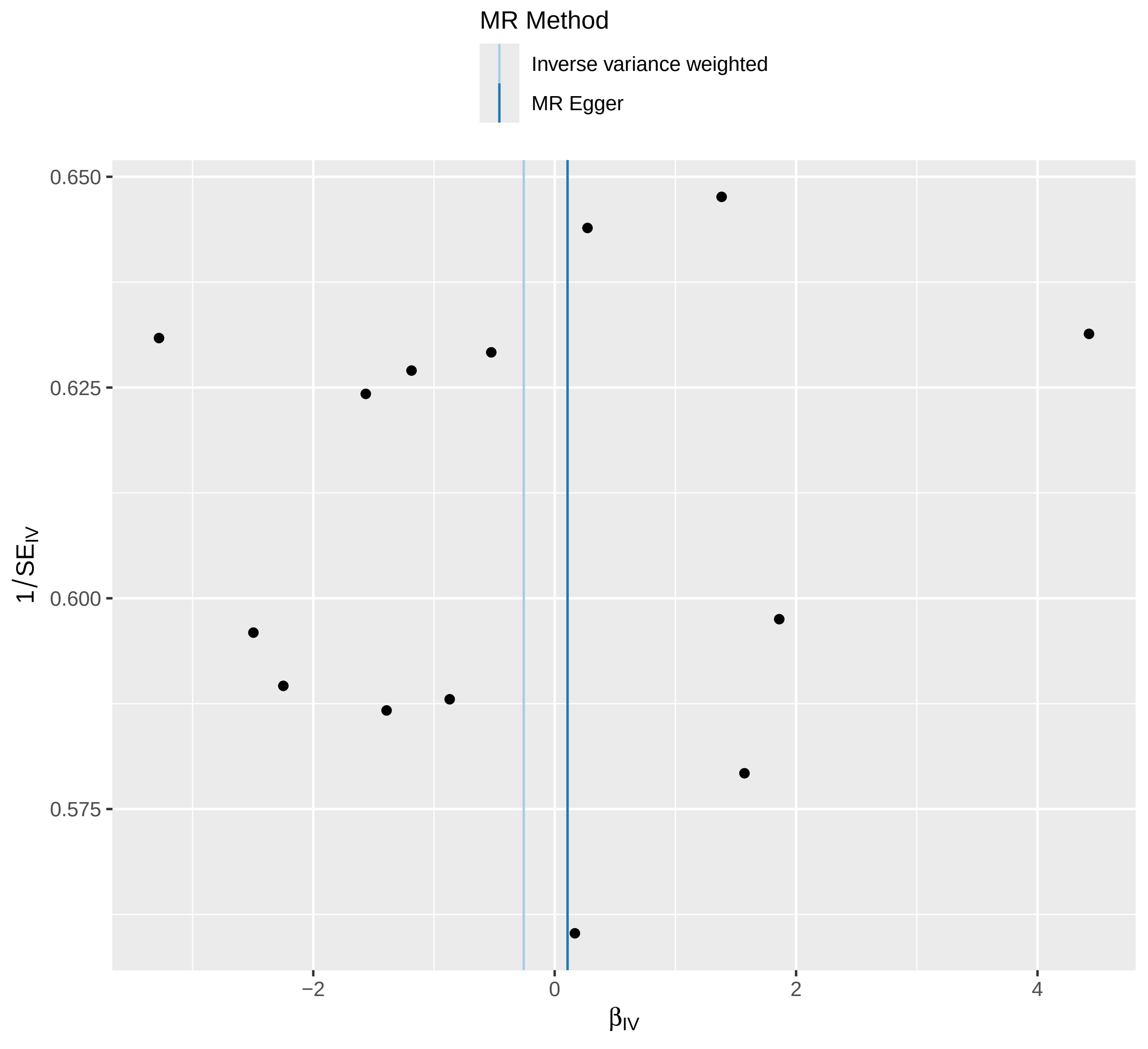

MR Method
Inverse variance weighted
MR Egger
0.650
0.625
0.600
0.575
1/SE_IV
−2
0
2
4
β_IV

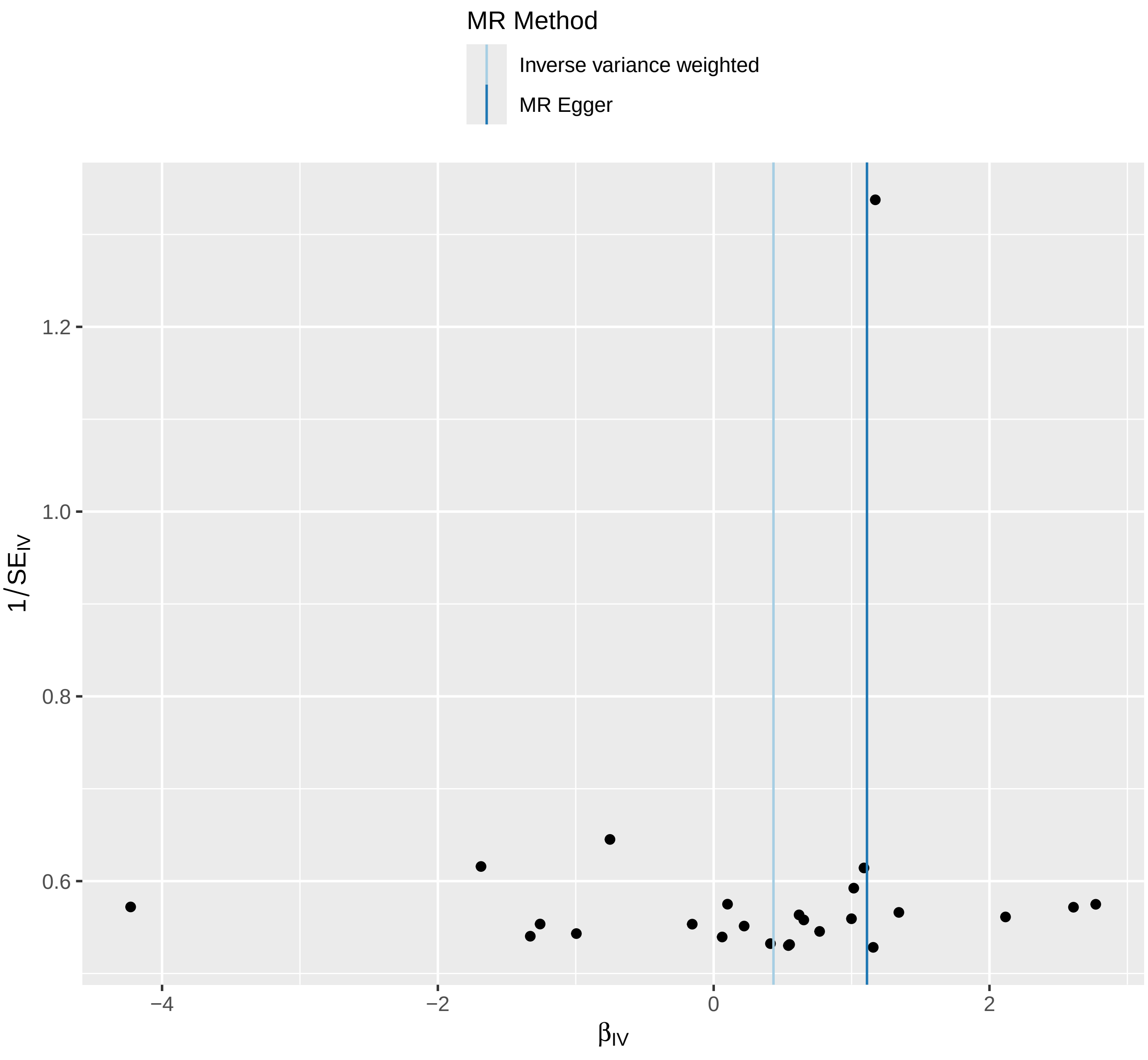
MR Method
Inverse variance weighted
MR Egger
1.2
1.0
0.8
0.6
1/SE_IV
−4
−2
0
2
β_IV

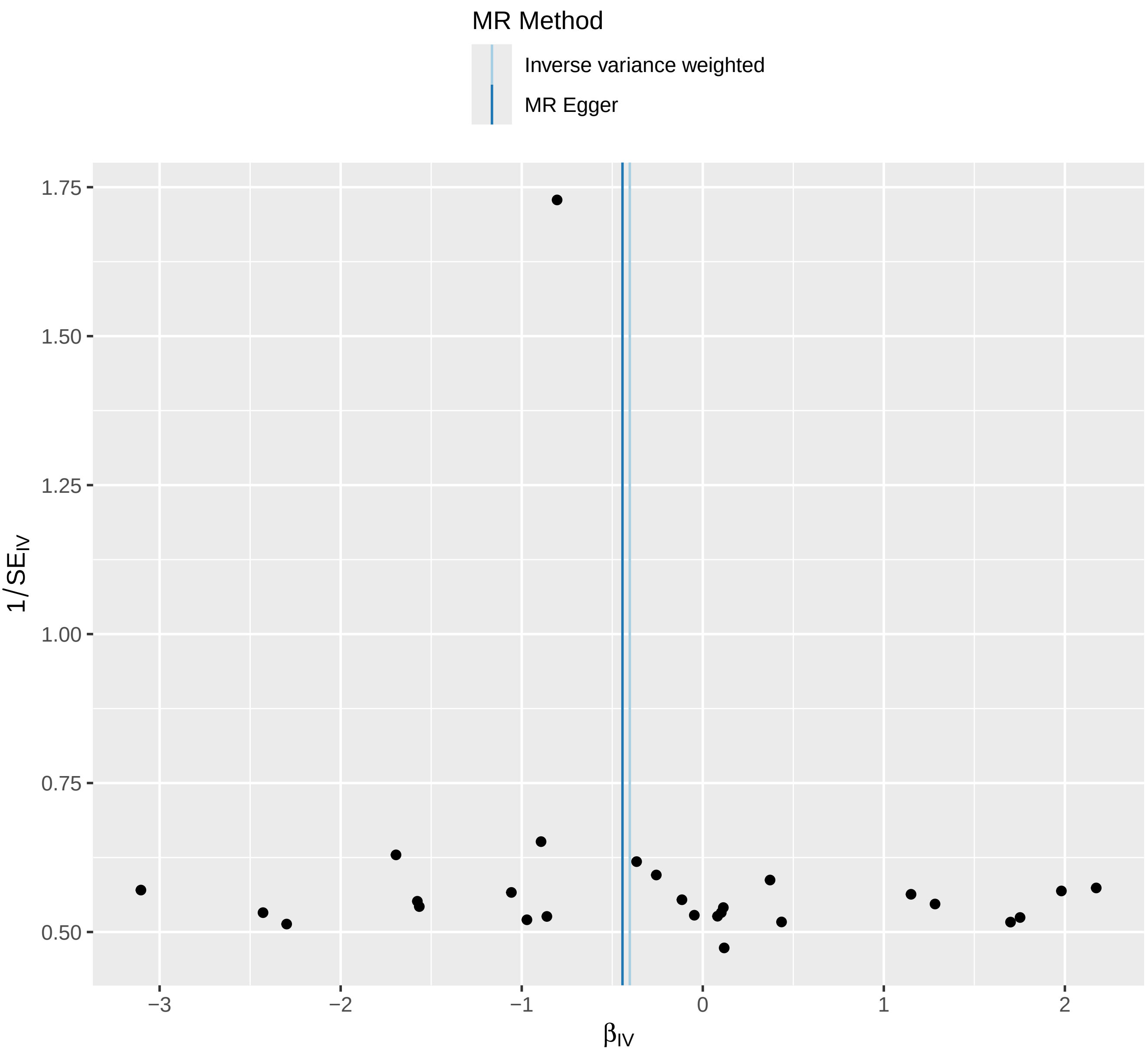
MR Method
Inverse variance weighted
MR Egger
1.75
1.50
1.25
1.00
0.75
0.50
1/SE_IV
−3
−2
−1
0
1
2
β_IV

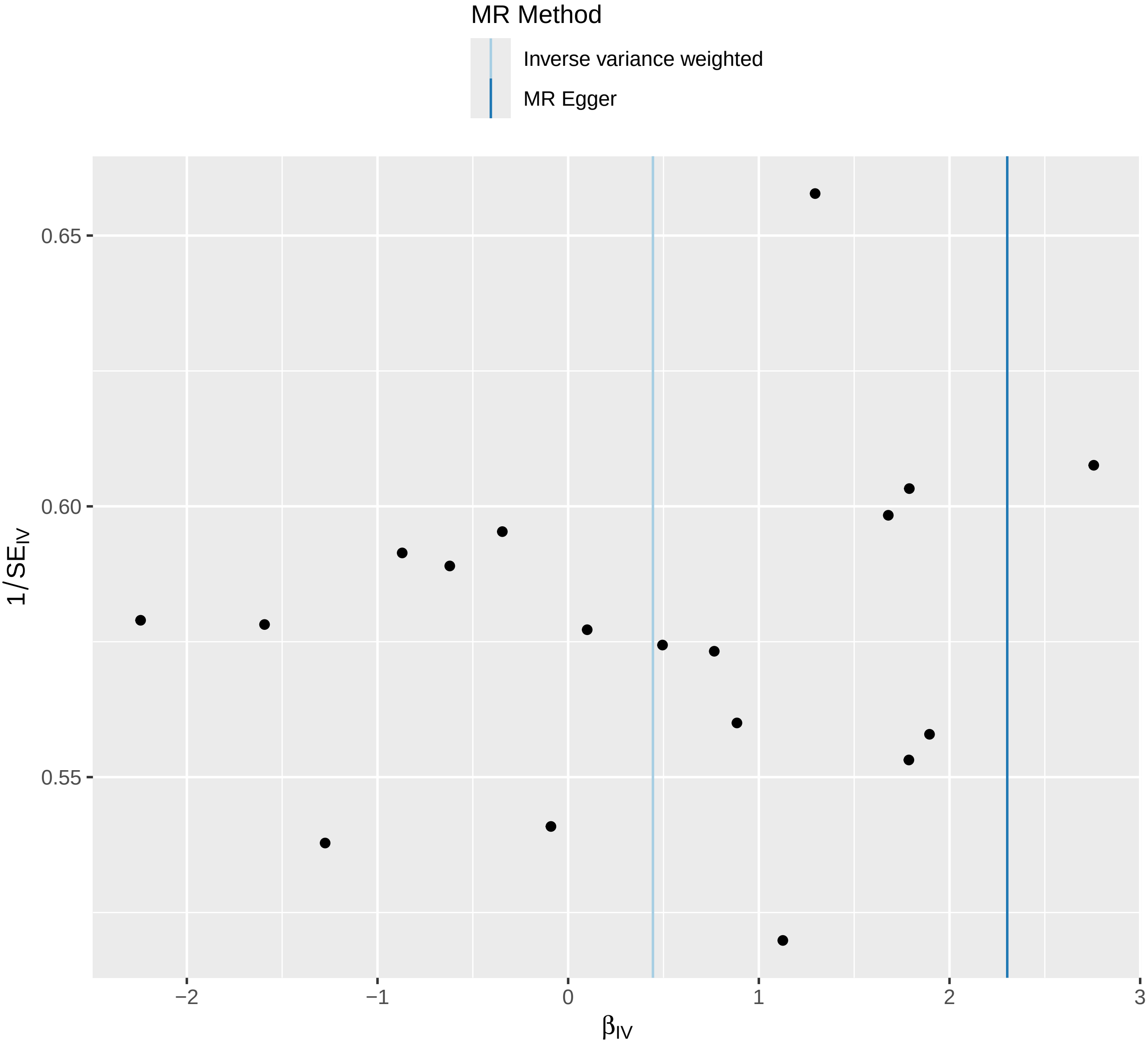
MR Method
Inverse variance weighted
MR Egger
0.65
0.60
0.55
1/SE_IV
−2
−1
0
1
2
3
β_IV

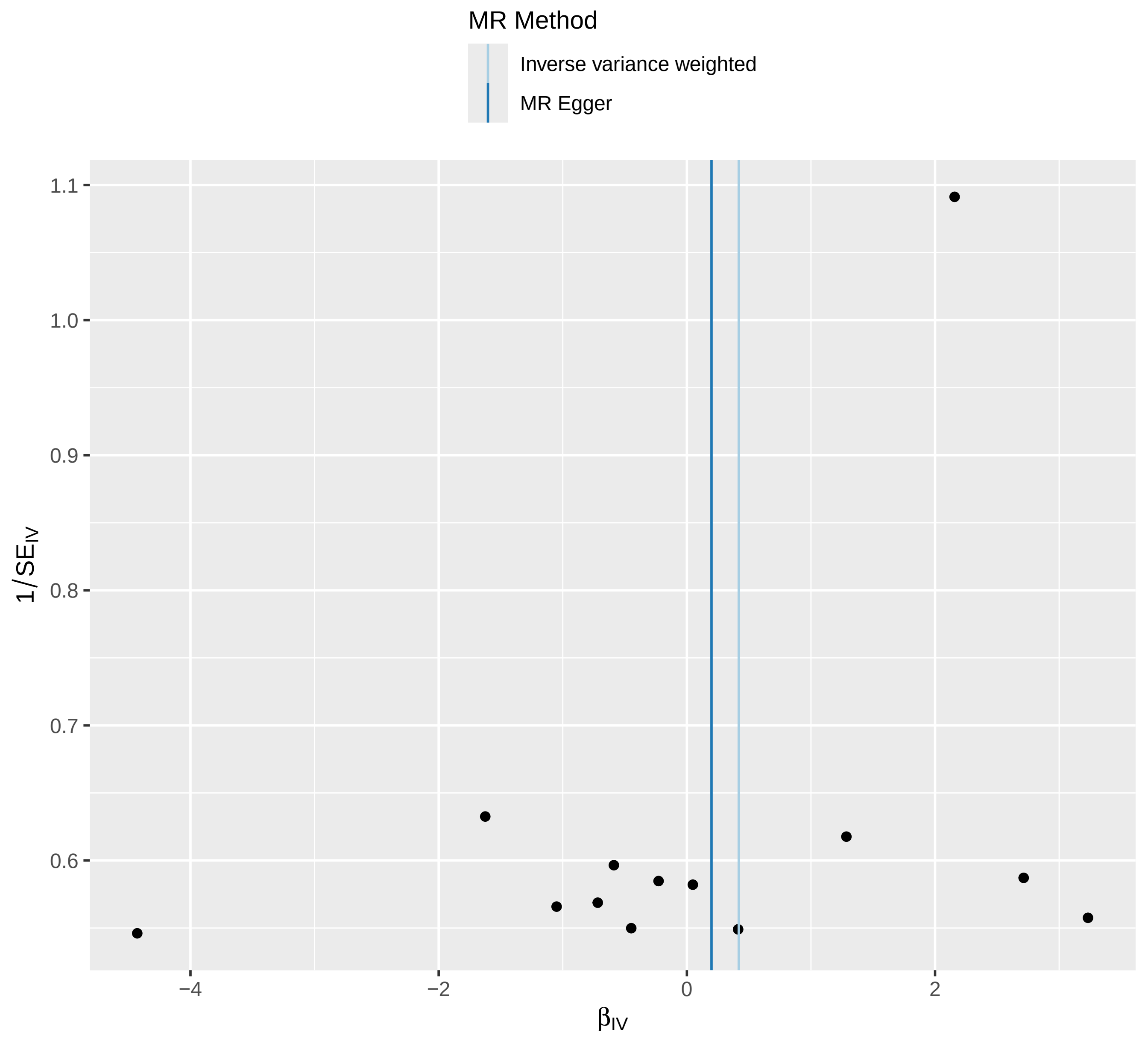
MR Method
Inverse variance weighted
MR Egger
1.1
1.0
0.9
0.8
0.7
0.6
1/SE_IV
−4
−2
0
2
β_IV

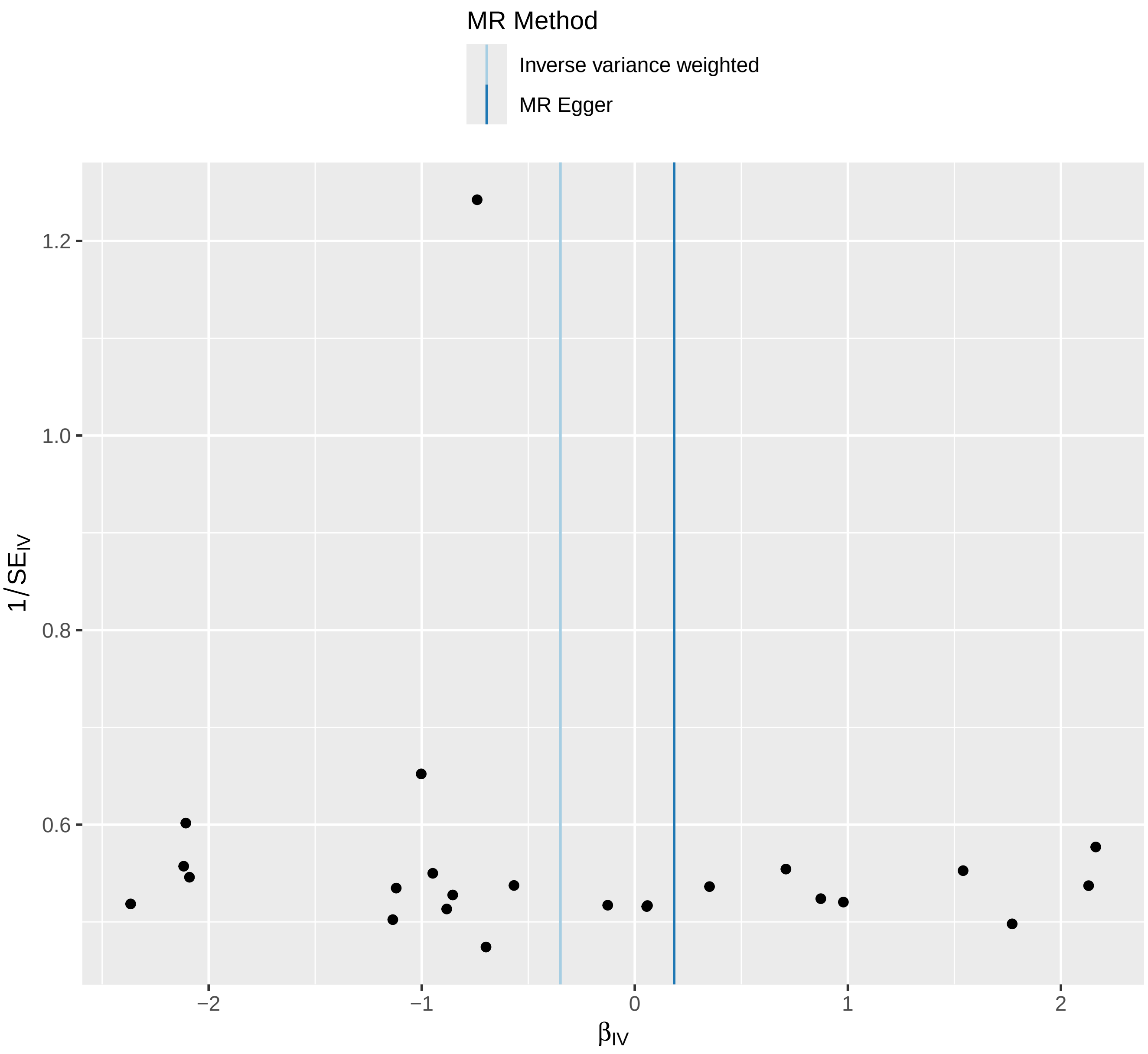
MR Method
Inverse variance weighted
MR Egger
1/SE_IV
1.2
1.0
0.8
0.6
−2
−1
0
1
2
β_IV

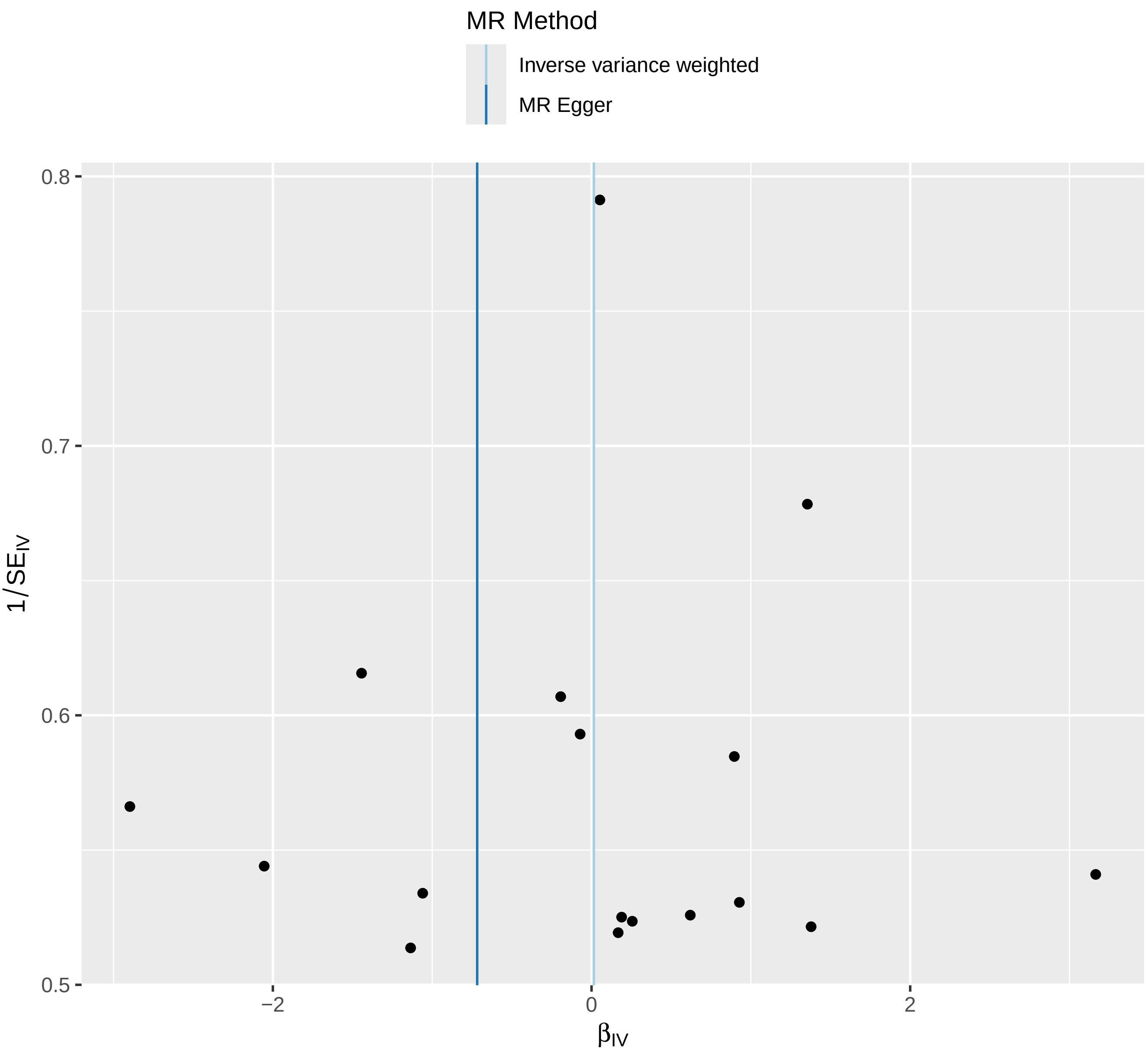
MR Method
Inverse variance weighted
MR Egger
0.8
0.7
0.6
0.5
−2
0
2
$1/SE_{IV}$
β_{IV}

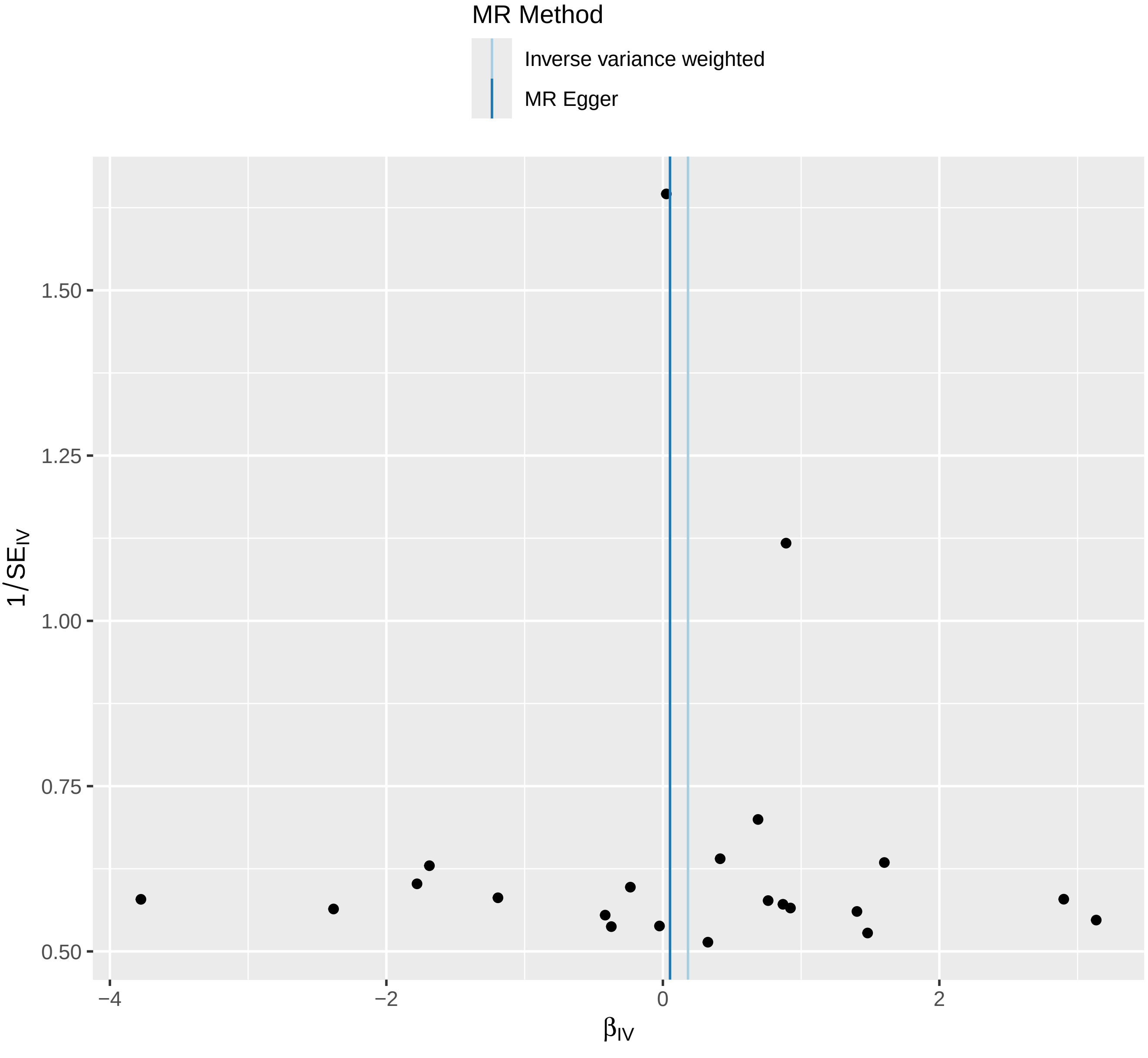

MR Method
Inverse variance weighted
MR Egger
1.50
1.25
1.00
0.75
0.50
1/SE_IV
−4
−2
0
2
β_IV

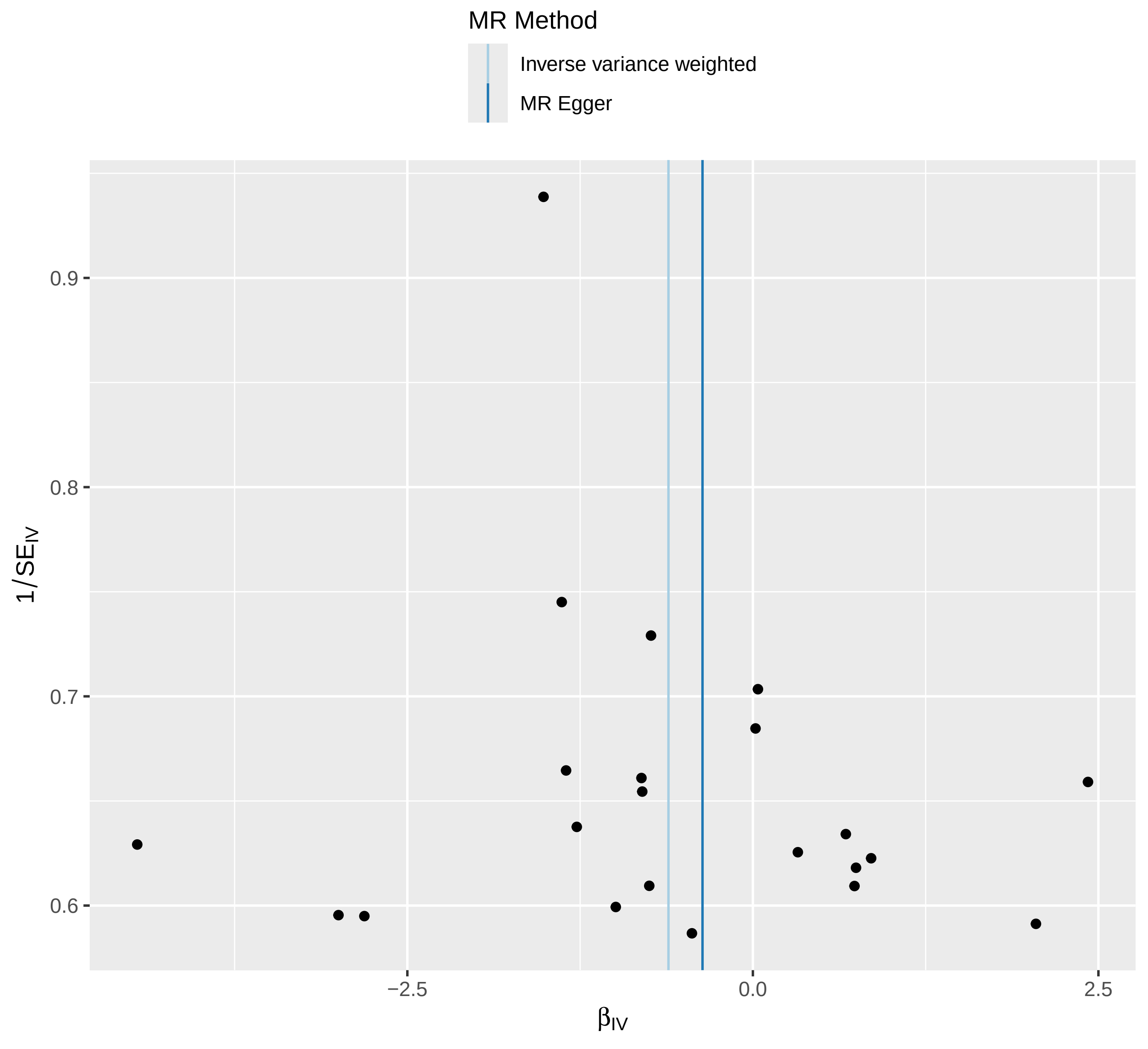
MR Method
Inverse variance weighted
MR Egger
0.9
0.8
0.7
0.6
$1/SE_{IV}$
−2.5
0.0
2.5
β_{IV}

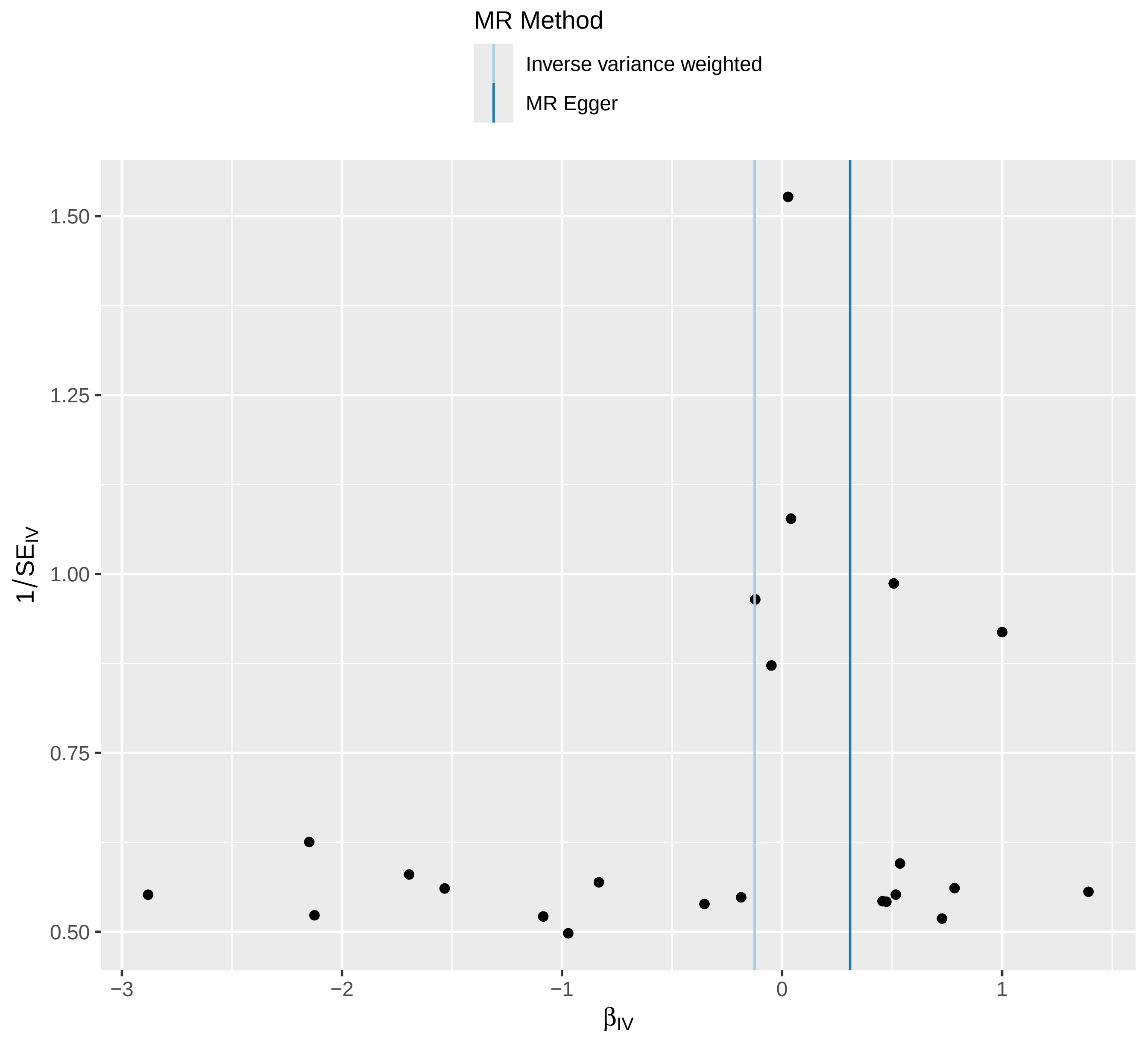

MR Method
Inverse variance weighted
MR Egger
1/SE_IV
1.50
1.25
1.00
0.75
0.50
−3
−2
−1
0
1
β_IV

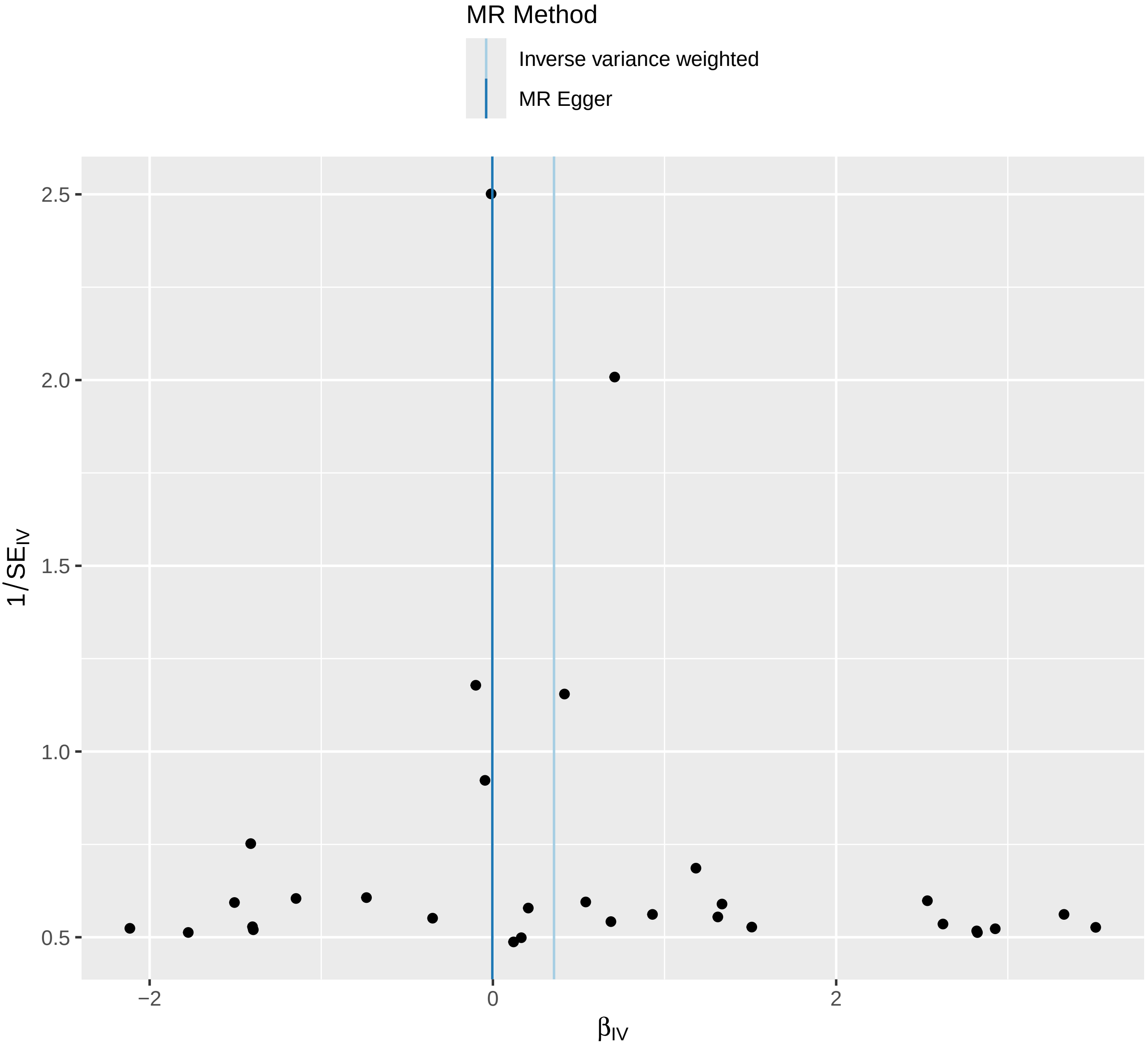
MR Method
Inverse variance weighted
MR Egger
2.5
2.0
1.5
1.0
0.5
1/SE_IV
−2
0
2
β_IV

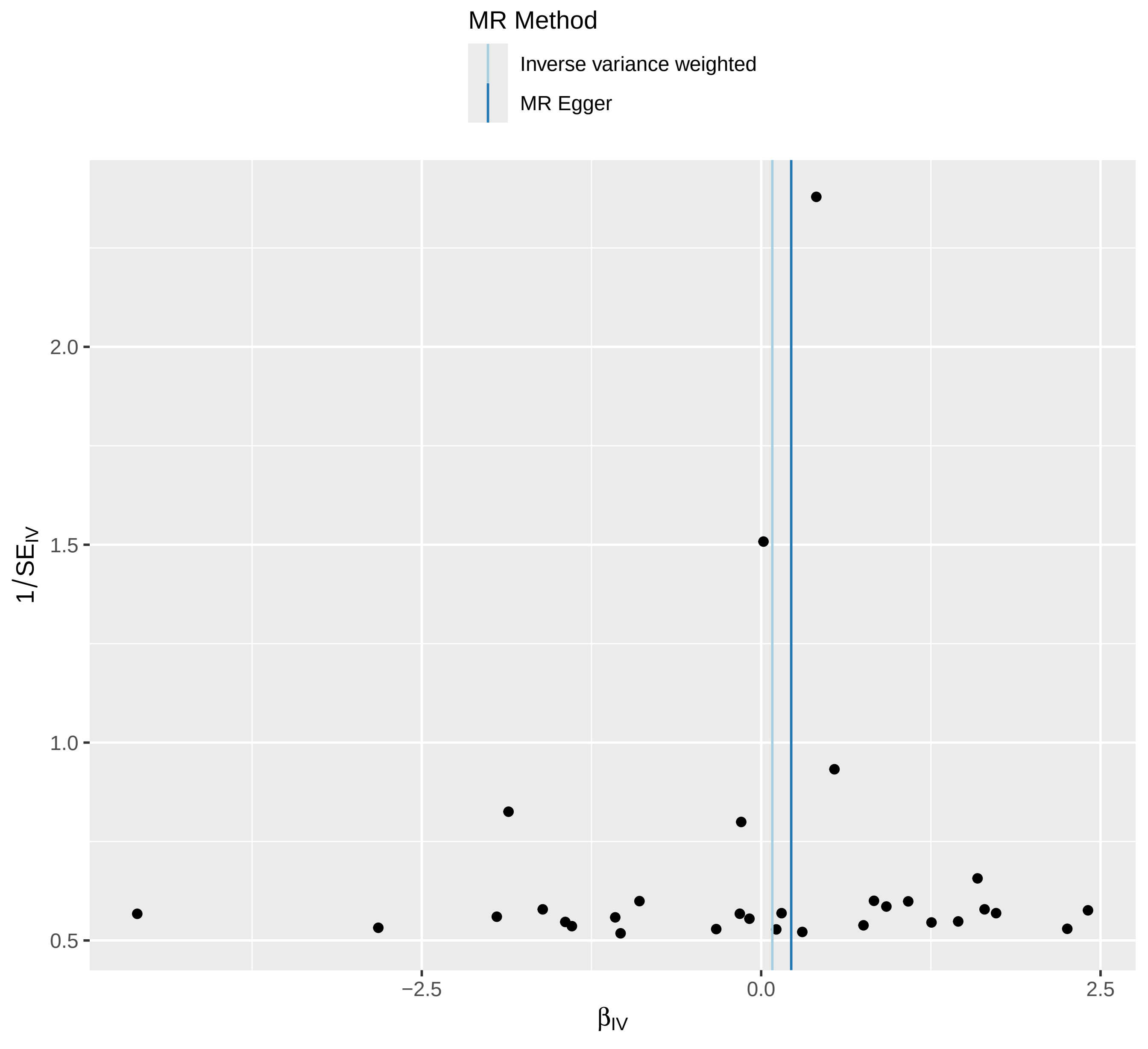

MR Method
Inverse variance weighted
MR Egger
1/SE_IV
2.0
1.5
1.0
0.5
−2.5
0.0
2.5
β_IV

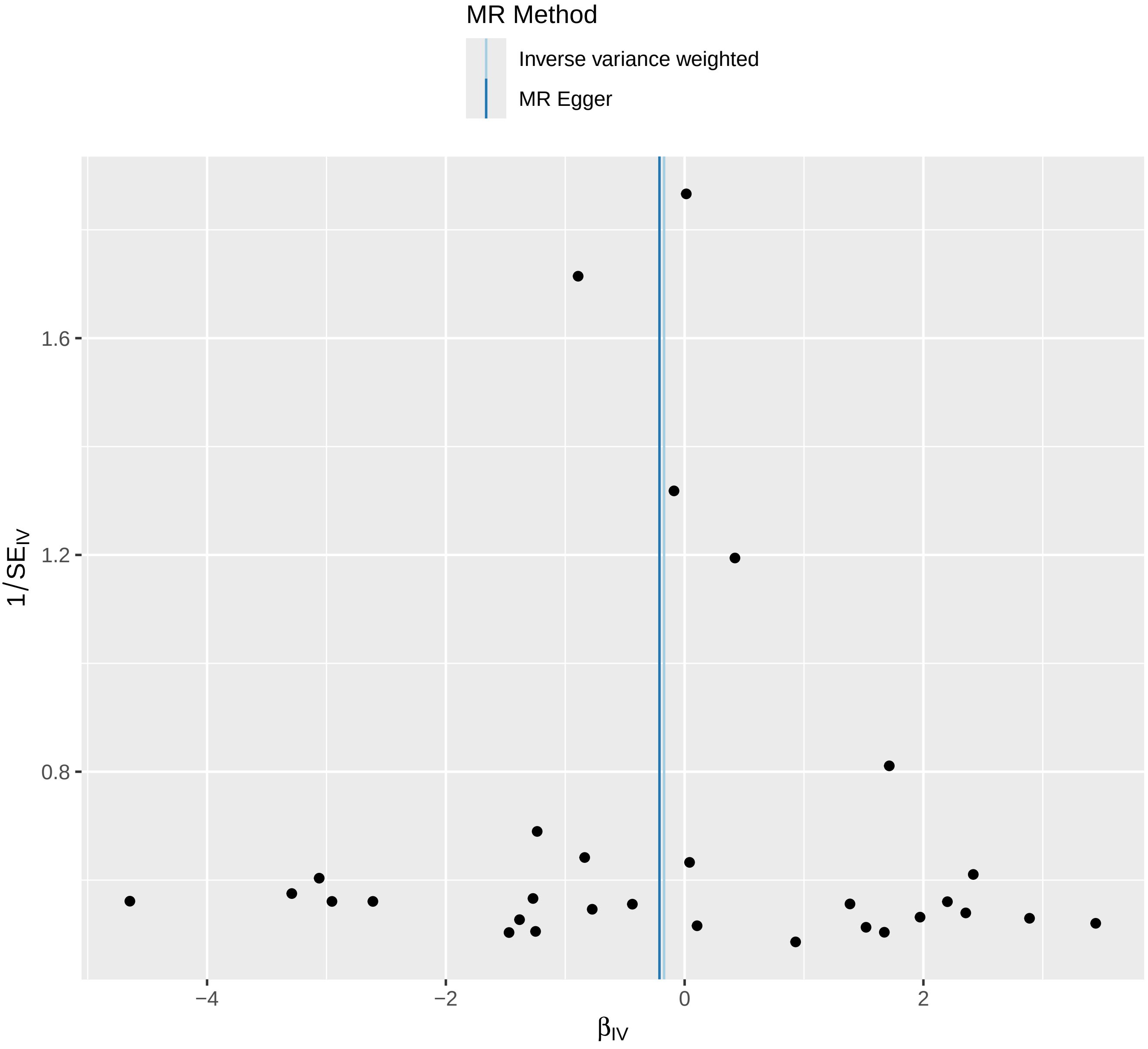
MR Method
Inverse variance weighted
MR Egger
1.6
1.2
0.8
1/SE_IV
−4
−2
0
2
β_IV

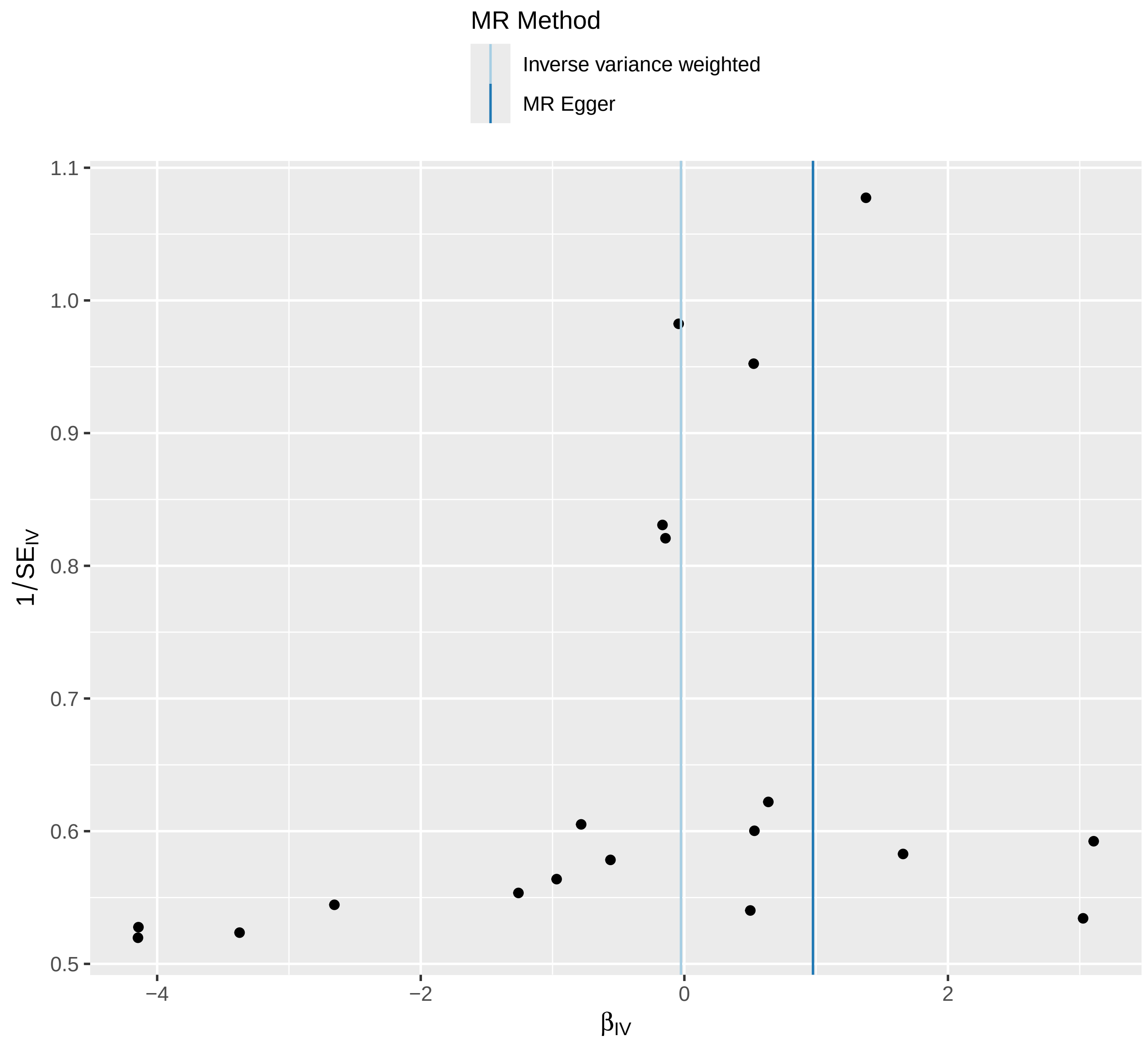
MR Method
Inverse variance weighted
MR Egger
1.1
1.0
0.9
0.8
0.7
0.6
0.5
1/SE_IV
−4
−2
0
2
β_IV

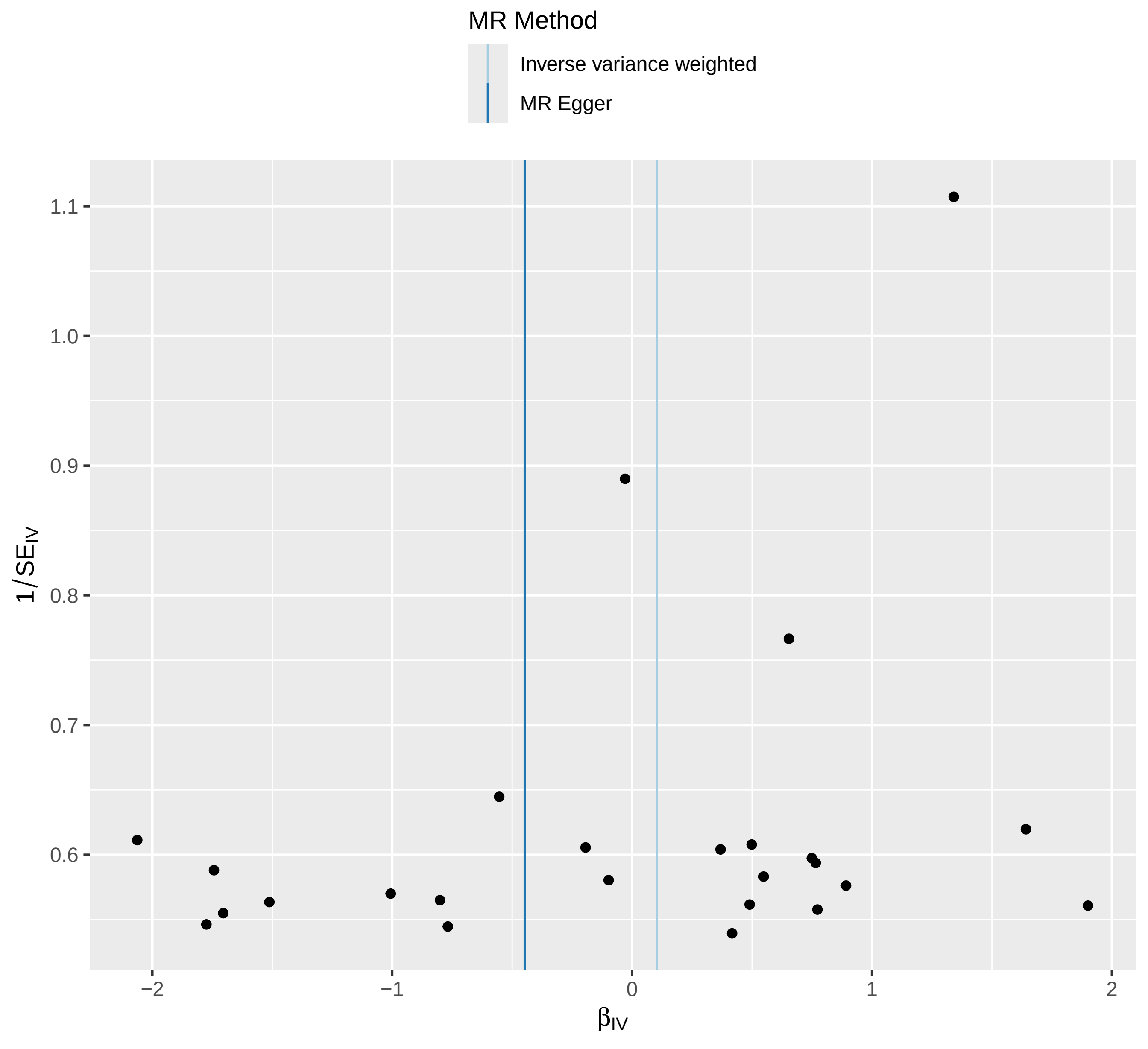
MR Method
Inverse variance weighted
MR Egger
1.1
1.0
0.9
0.8
0.7
0.6
1/SE_IV
−2
−1
0
1
2
β_IV

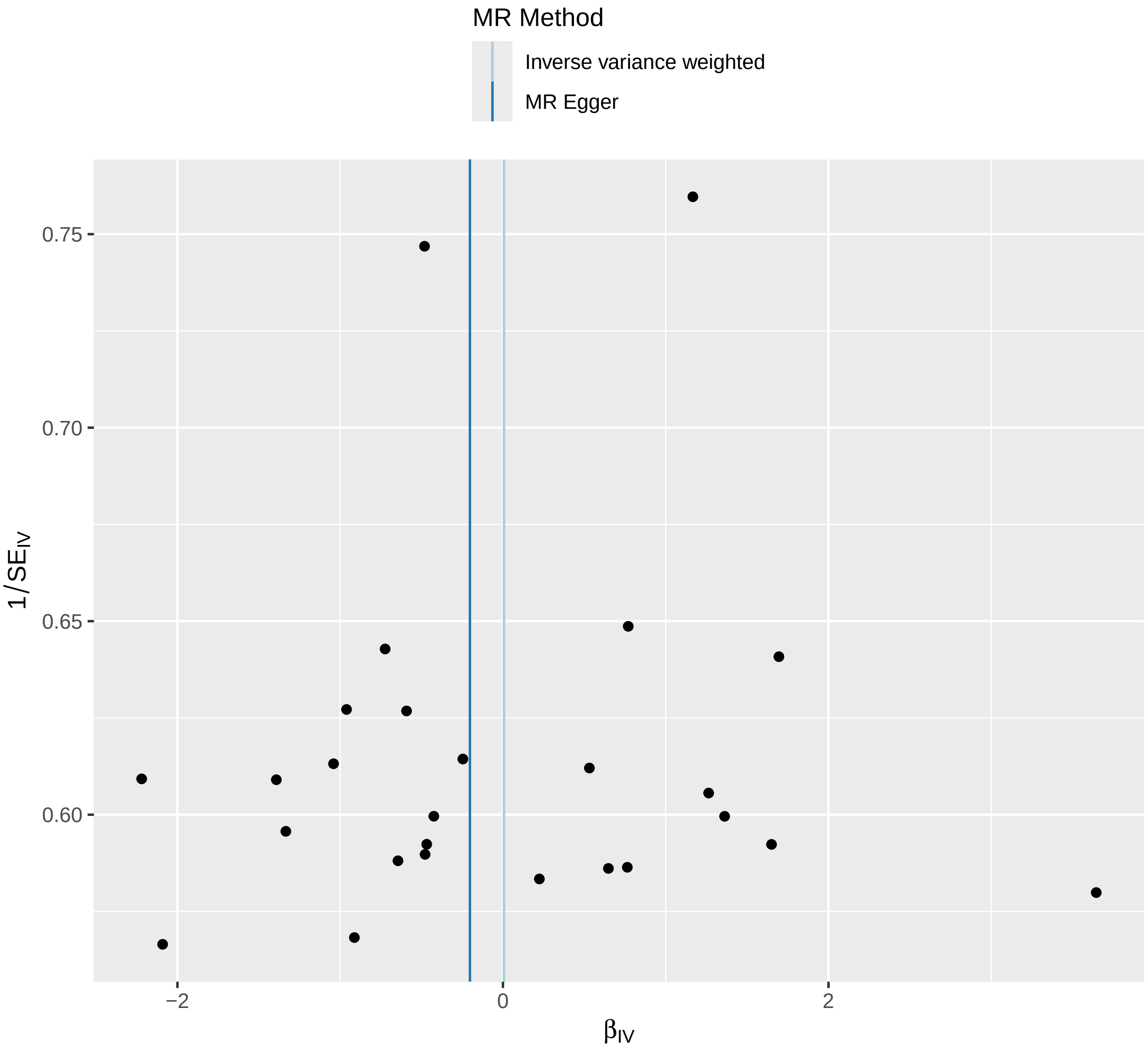

MR Method
Inverse variance weighted
MR Egger
0.75
0.70
0.65
0.60
$1/SE_{IV}$
−2
0
2
β_{IV}

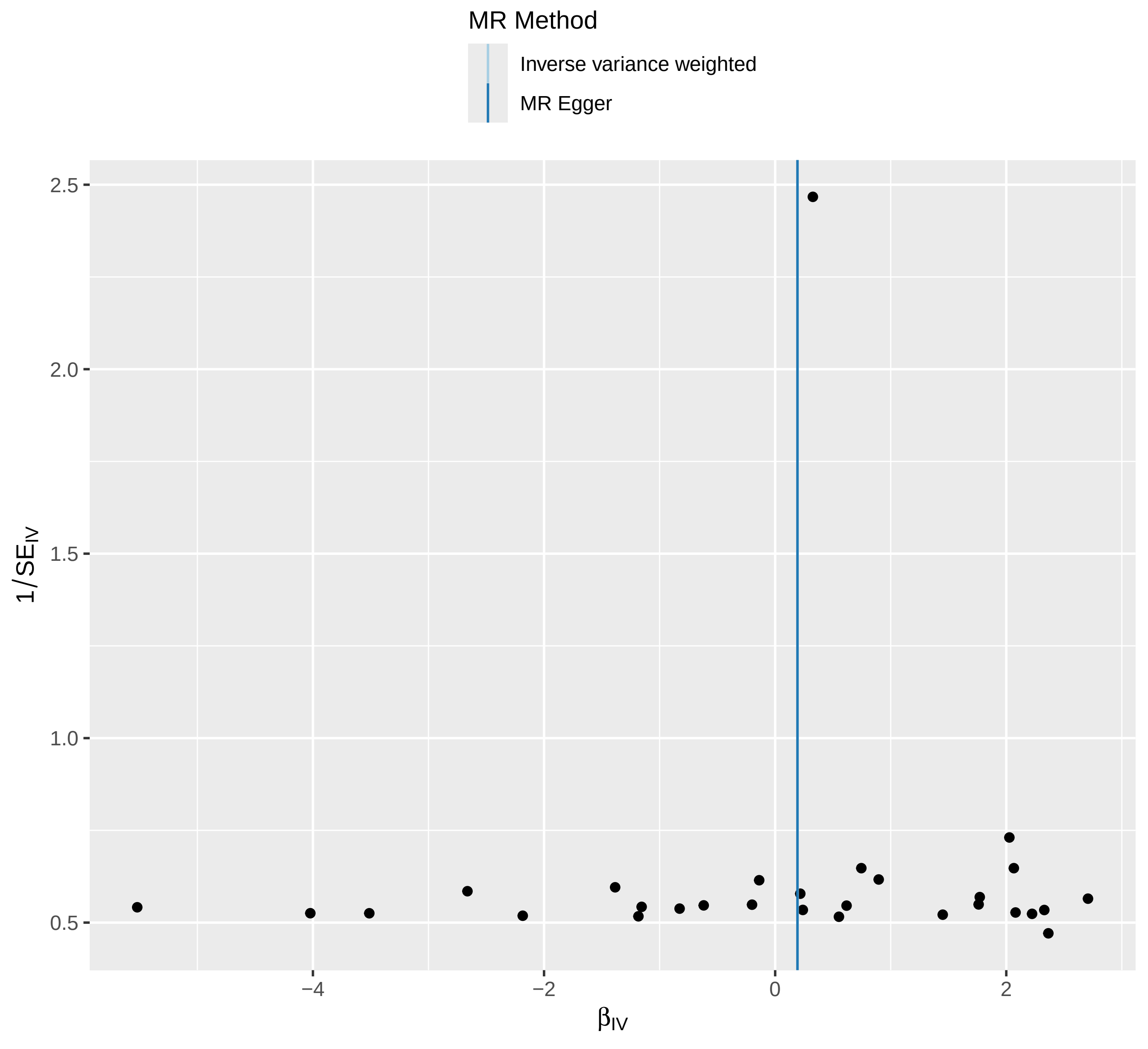
MR Method
Inverse variance weighted
MR Egger
2.5
2.0
1.5
1.0
0.5
1/SE_IV
−4
−2
0
2
β_IV

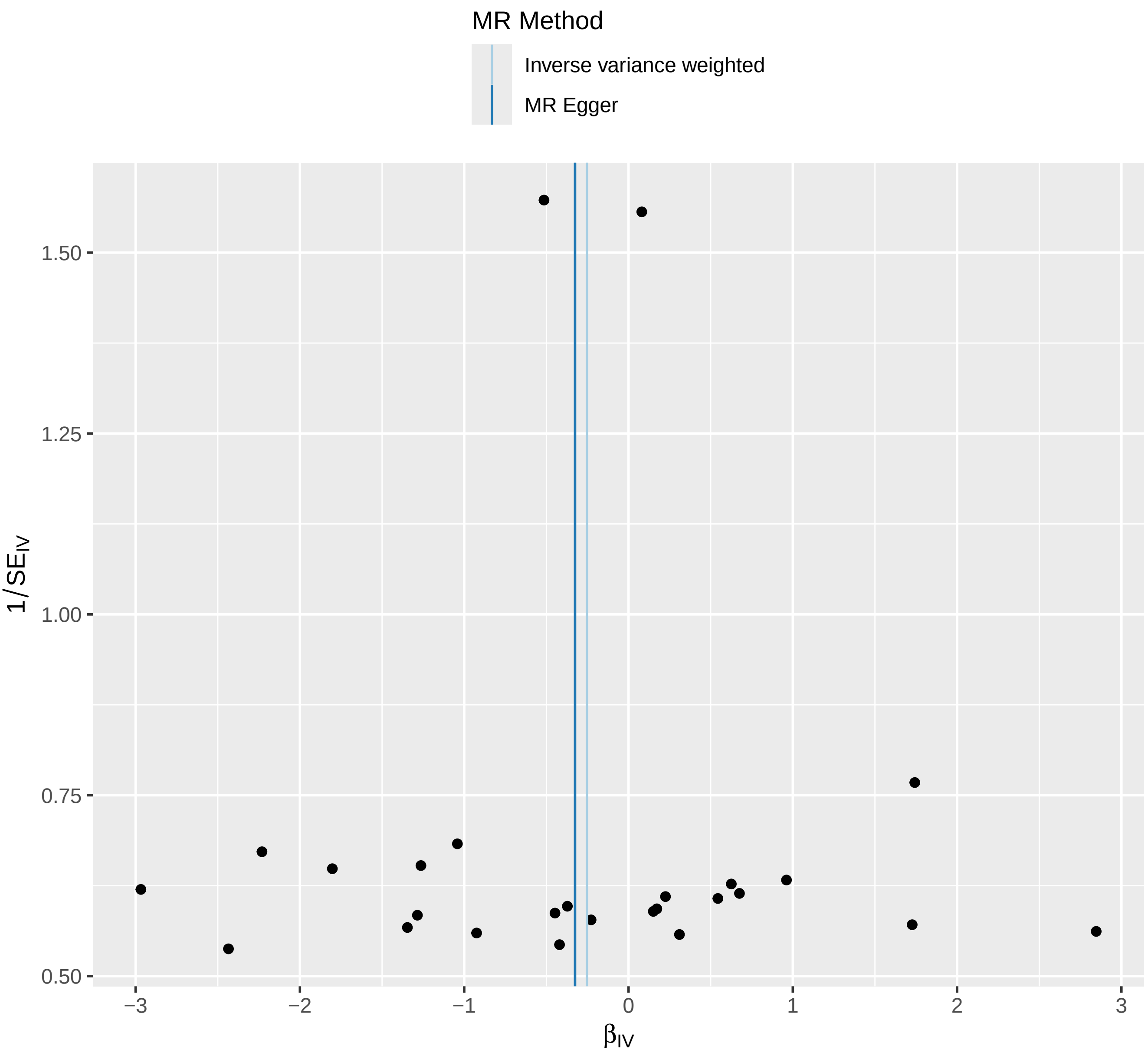
MR Method
Inverse variance weighted
MR Egger
1/SE_IV
1.50
1.25
1.00
0.75
0.50
−3
−2
−1
0
1
2
3
β_IV

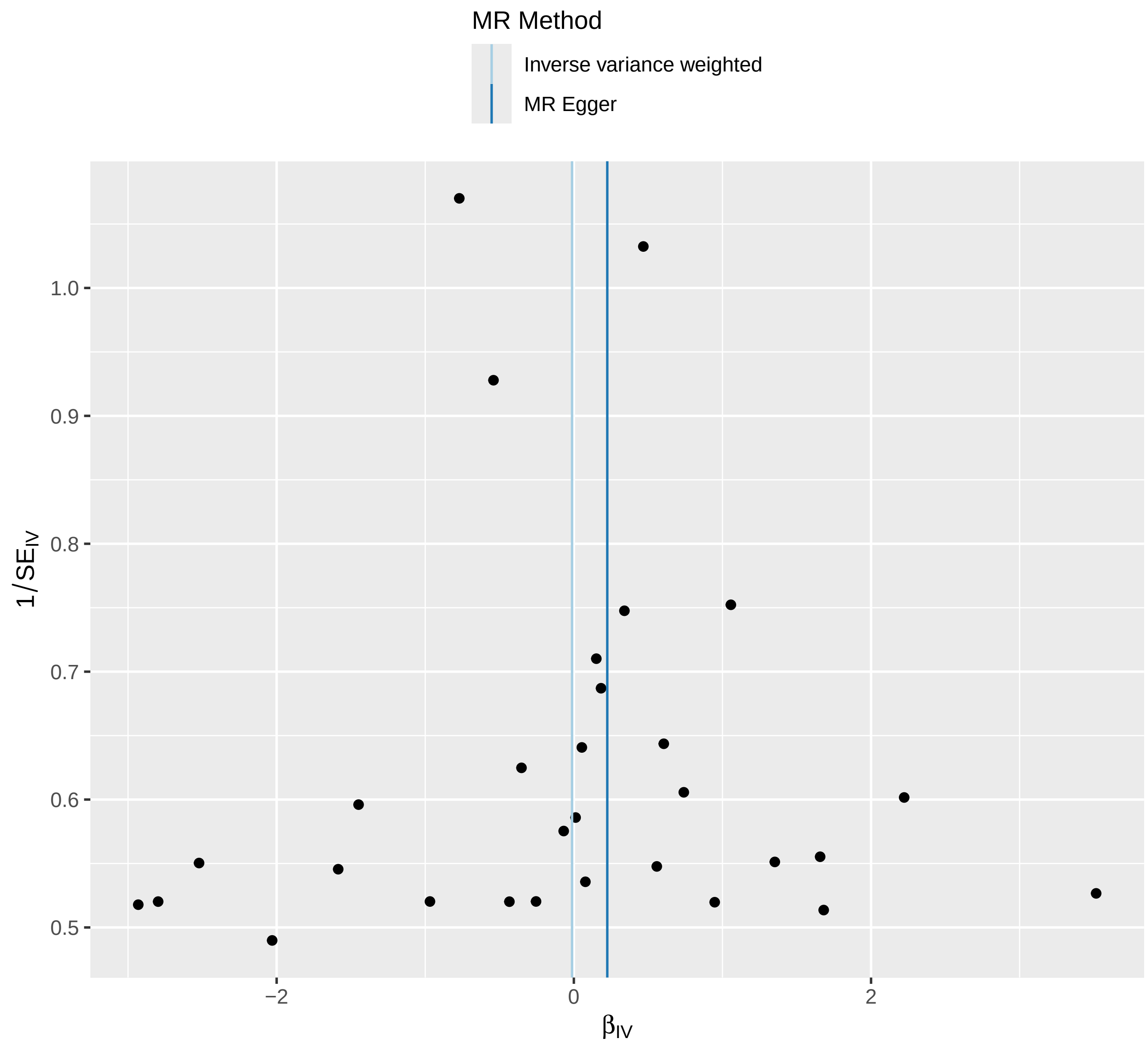

MR Method
Inverse variance weighted
MR Egger
1.0
0.9
0.8
0.7
0.6
0.5
1/SE$_{IV}$
−2
0
2
β$_{IV}$

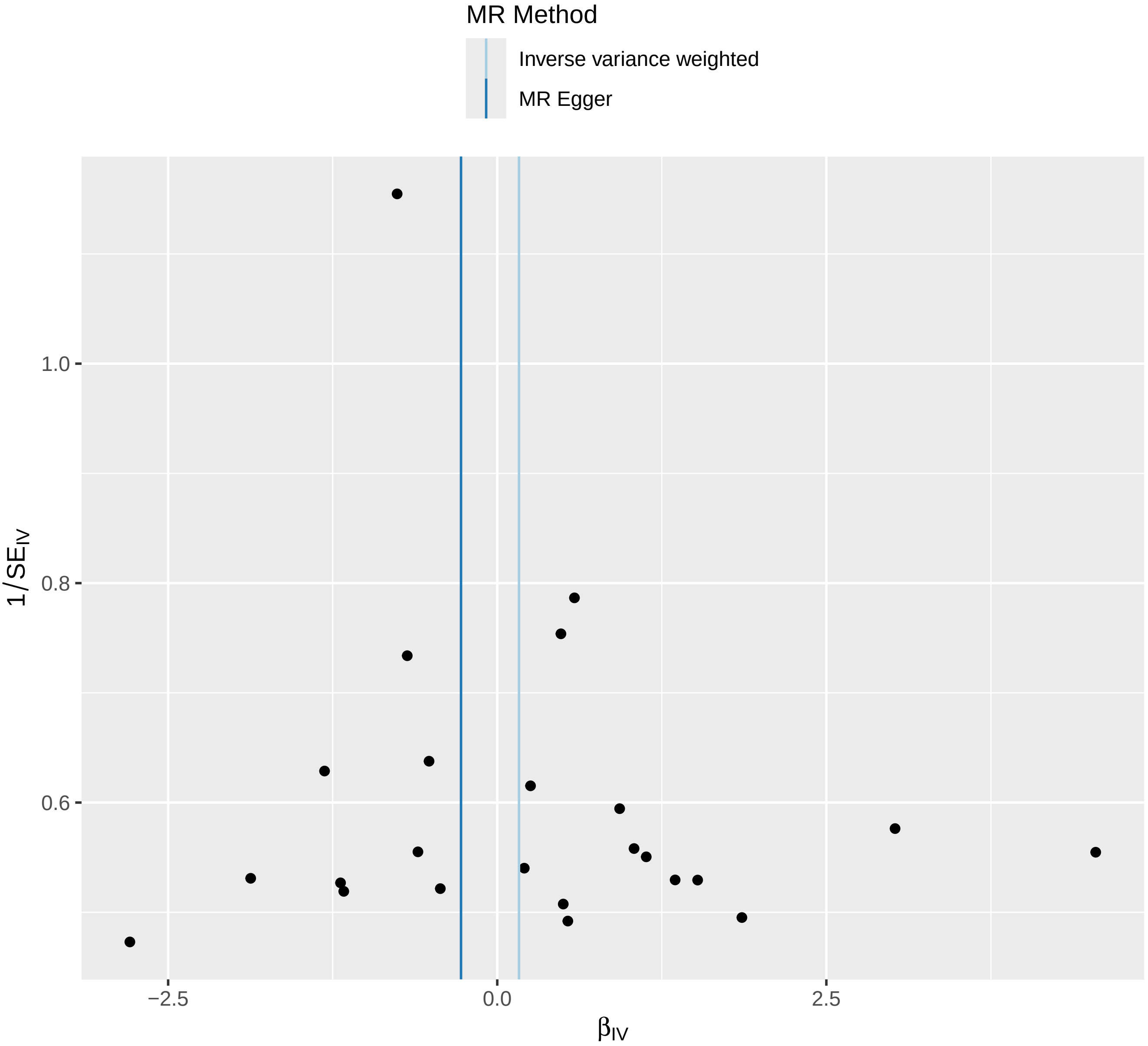

MR Method
Inverse variance weighted
MR Egger
1/SE_IV
1.0
0.8
0.6
−2.5
0.0
2.5
β_IV

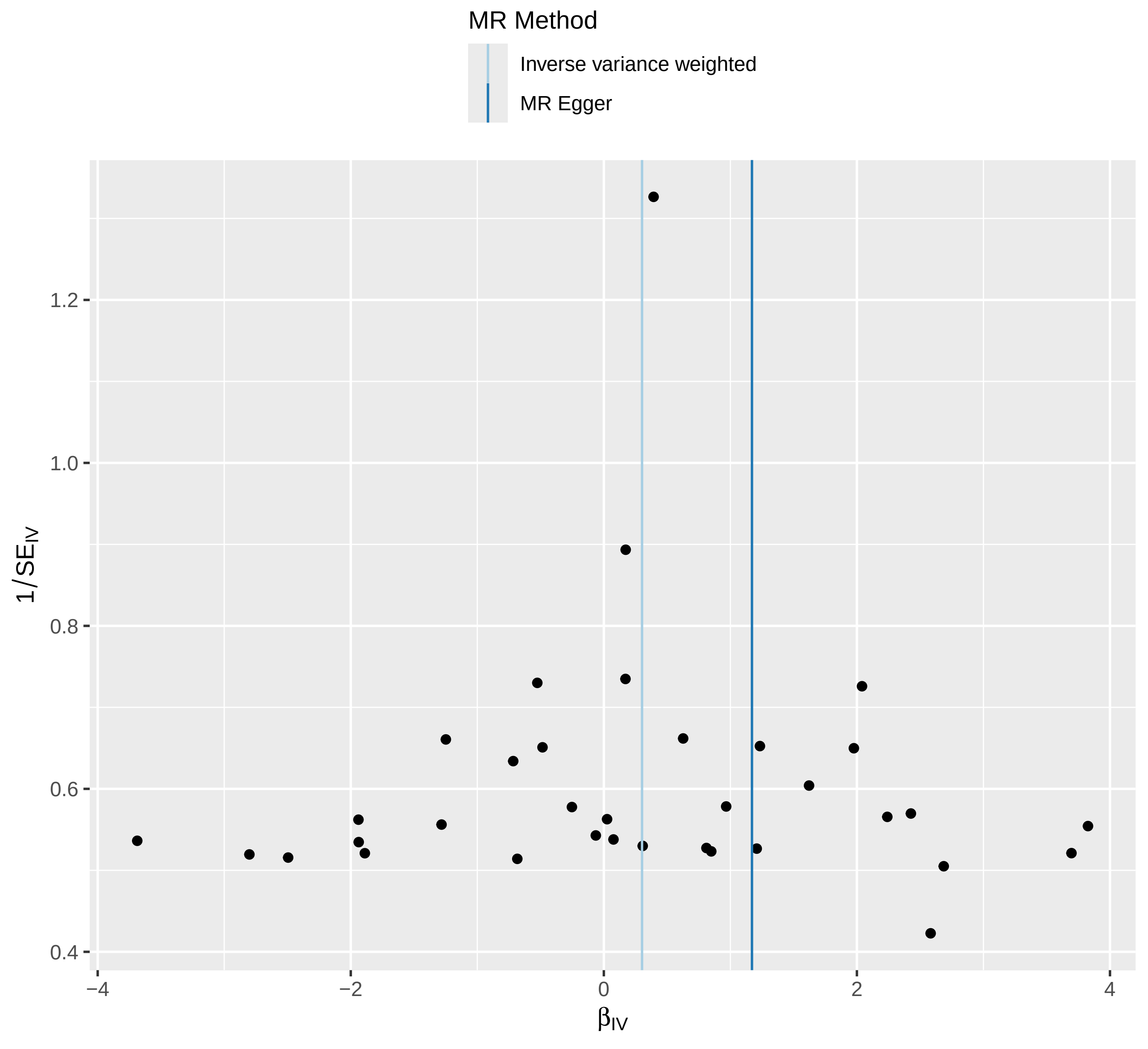
MR Method
Inverse variance weighted
MR Egger
1/SE_IV
1.2
1.0
0.8
0.6
0.4
−4
−2
0
2
4
β_IV

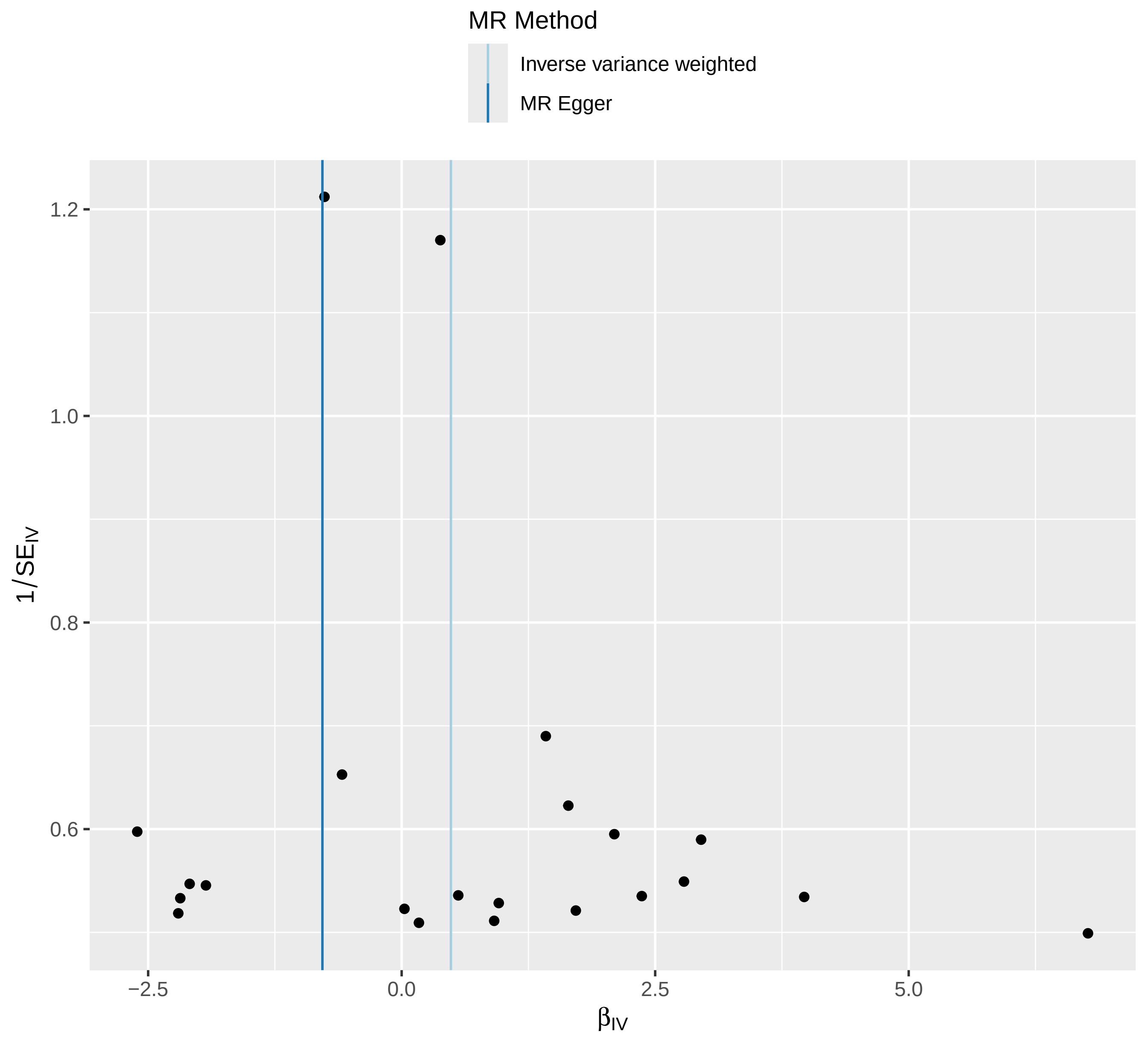
MR Method
Inverse variance weighted
MR Egger
1.2
1.0
0.8
0.6
1/SE_IV
−2.5
0.0
2.5
5.0
β_IV

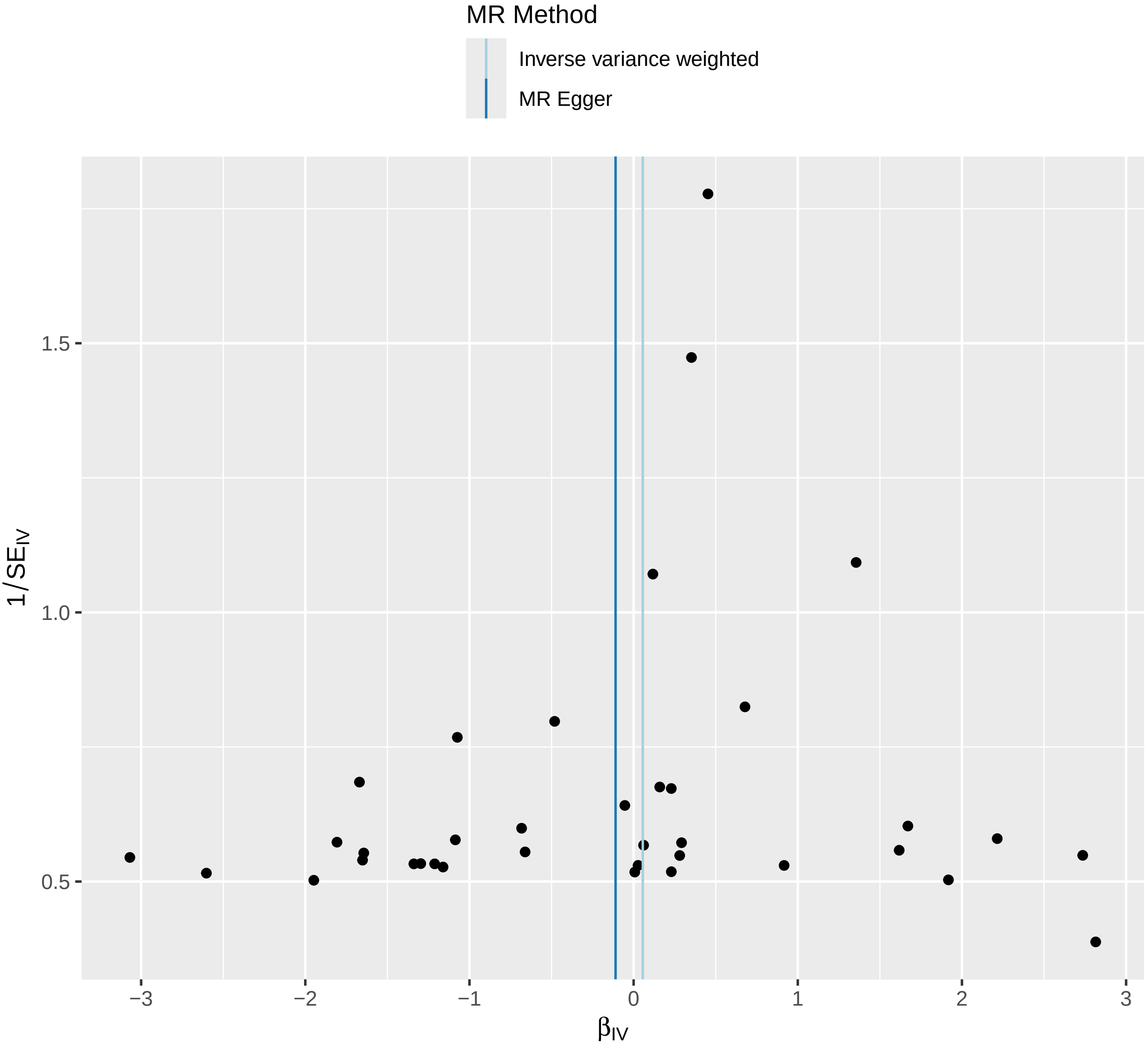
MR Method
Inverse variance weighted
MR Egger
1/SE_IV
1.5
1.0
0.5
−3
−2
−1
0
1
2
3
β_IV

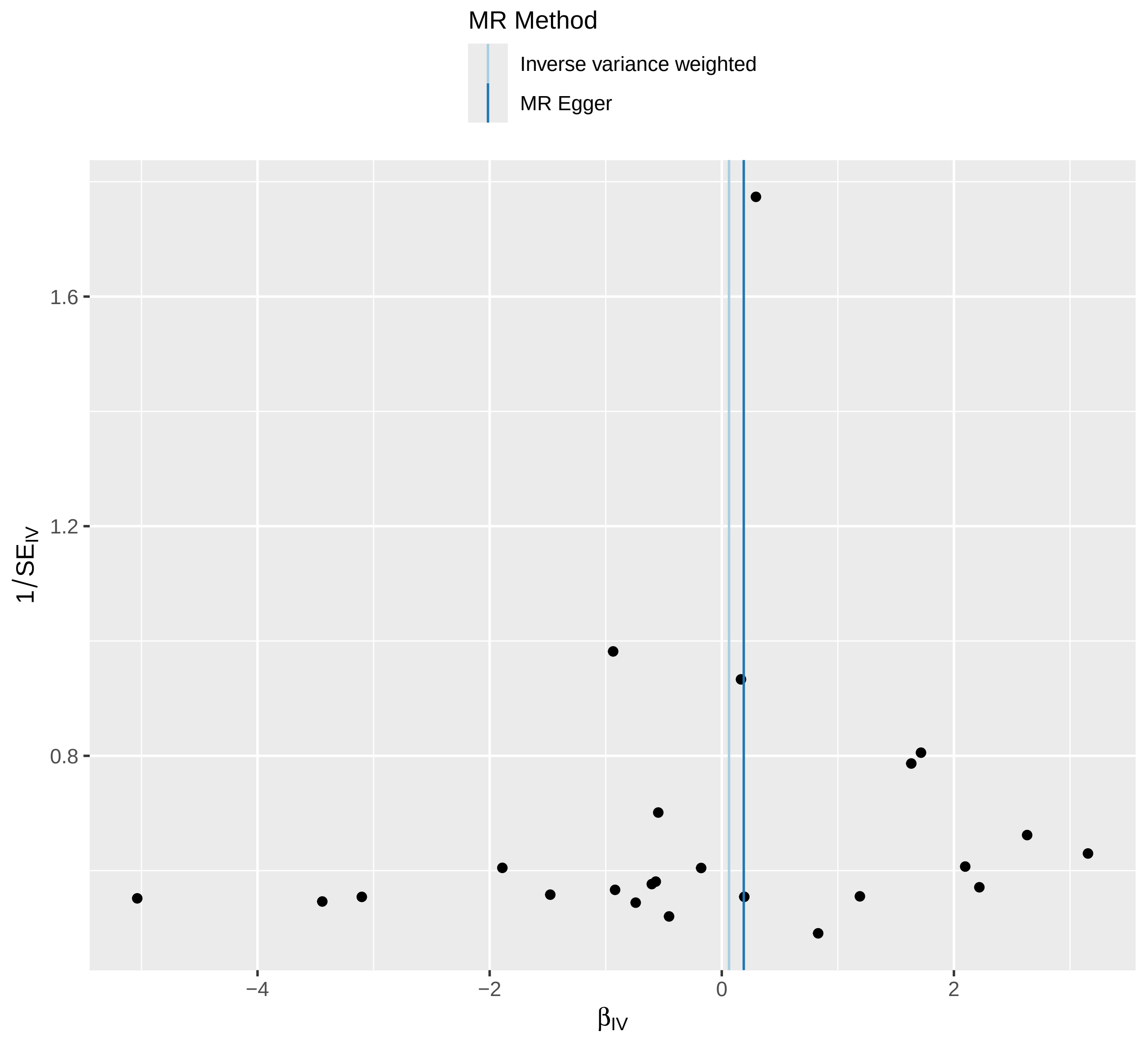
MR Method
Inverse variance weighted
MR Egger
1.6
1.2
0.8
1/SE_IV
−4
−2
0
2
β_IV

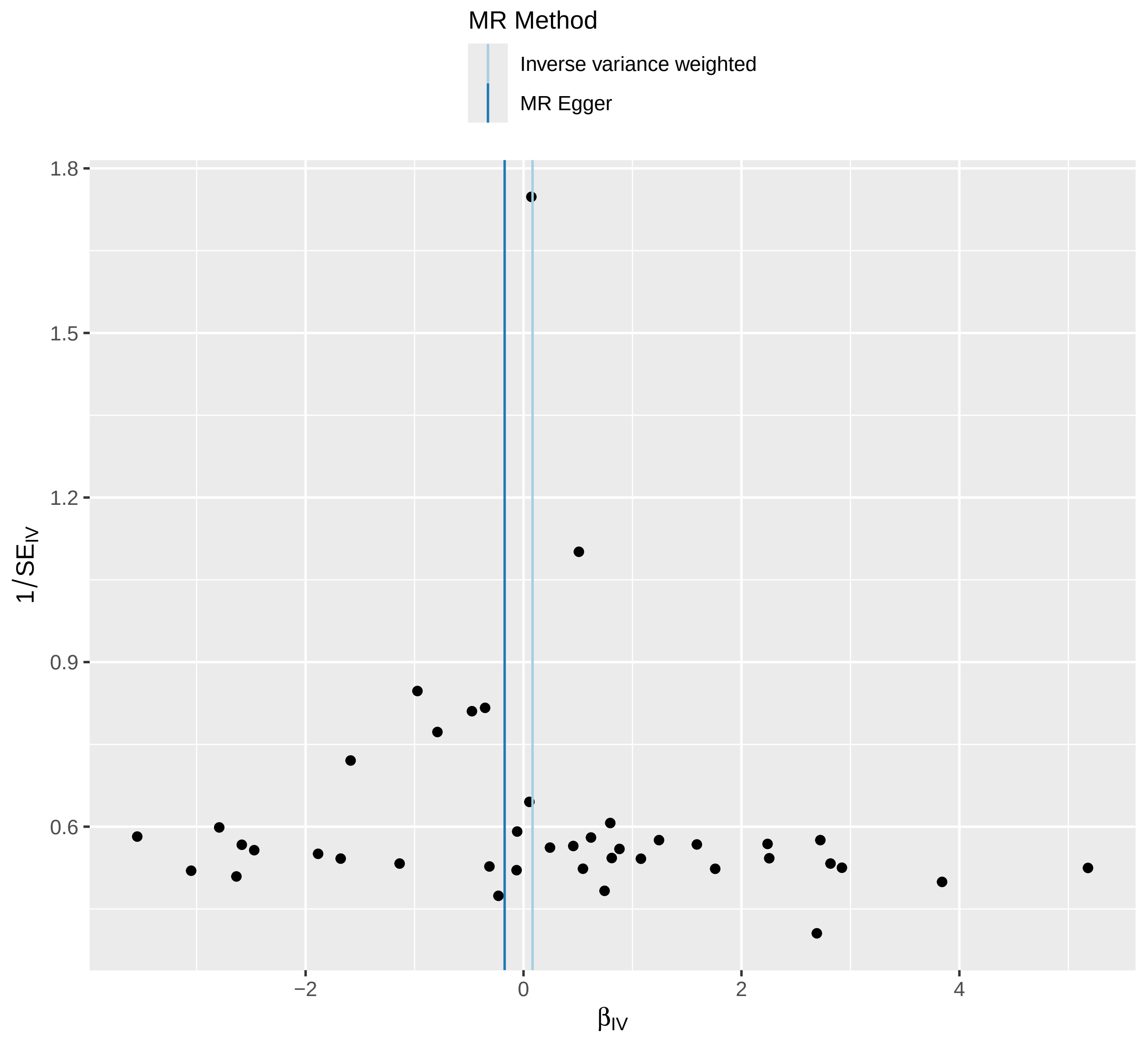
MR Method
Inverse variance weighted
MR Egger
1.8
1.5
1.2
0.9
0.6
−2
0
2
4
$1/SE_{IV}$
β_{IV}

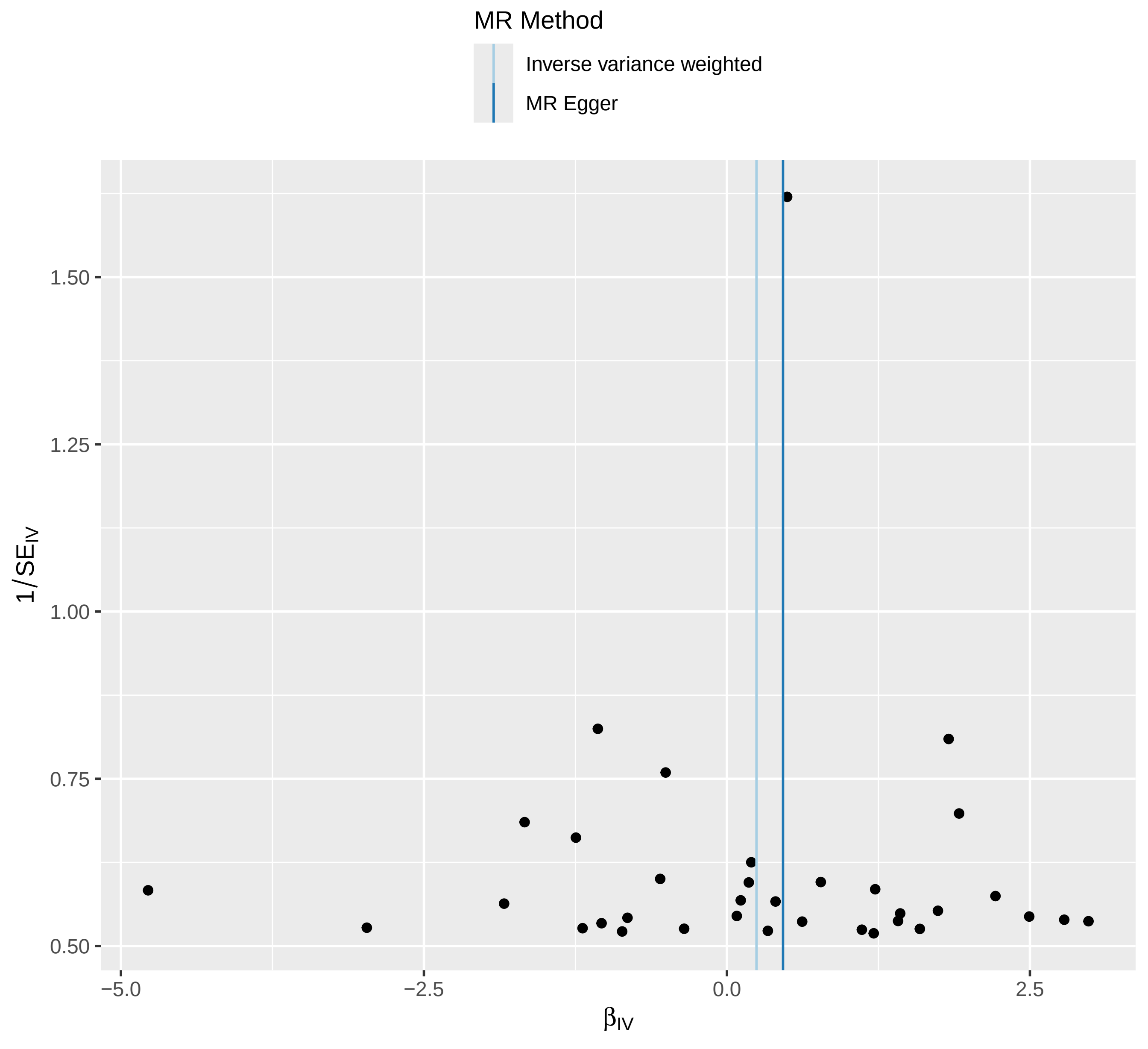
MR Method
Inverse variance weighted
MR Egger
1.50
1.25
1.00
0.75
0.50
1/SE$_{IV}$
−5.0
−2.5
0.0
2.5
β$_{IV}$

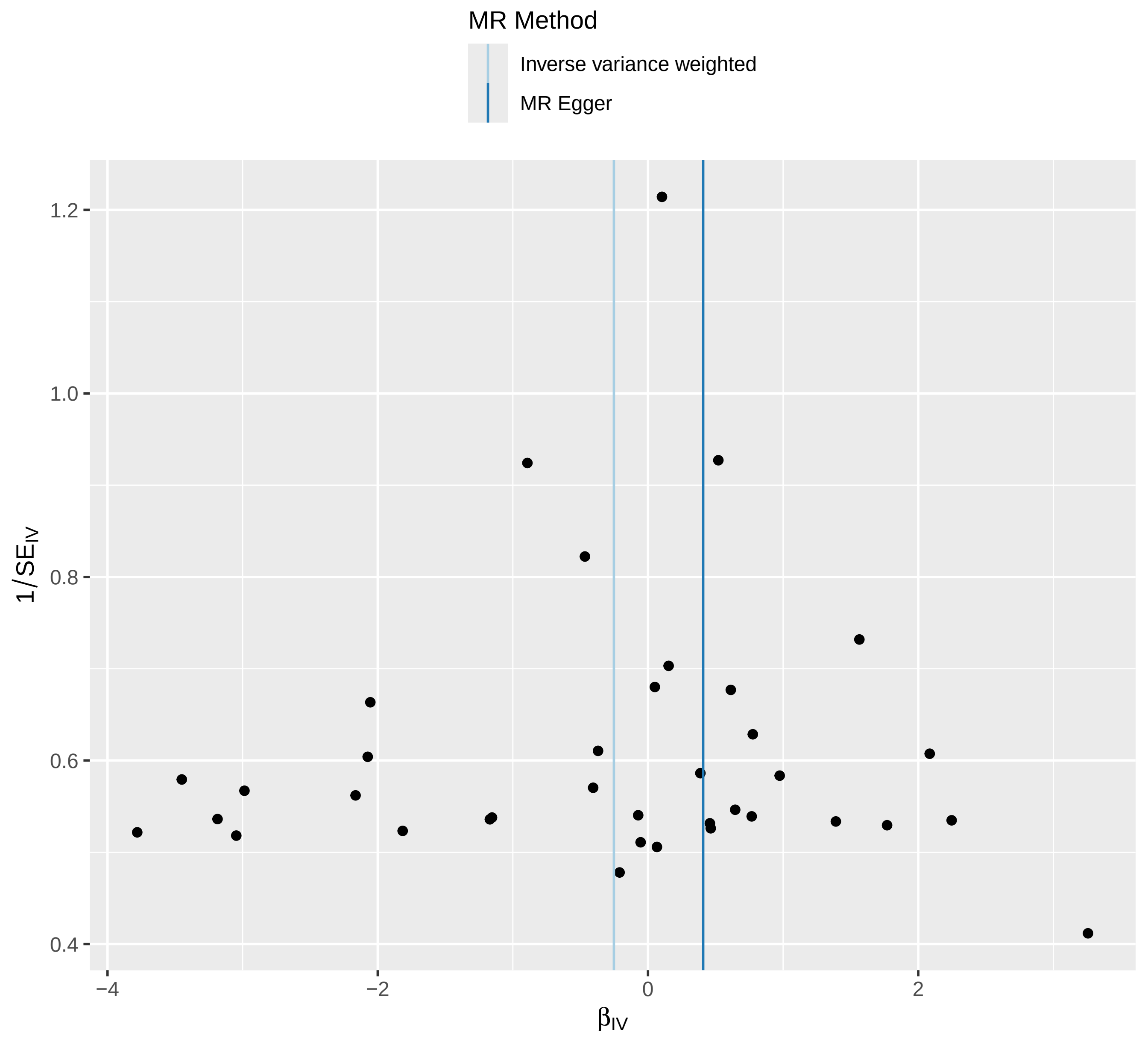

MR Method
Inverse variance weighted
MR Egger
1/SE_IV
1.2
1.0
0.8
0.6
0.4
−4
−2
0
2
β_IV

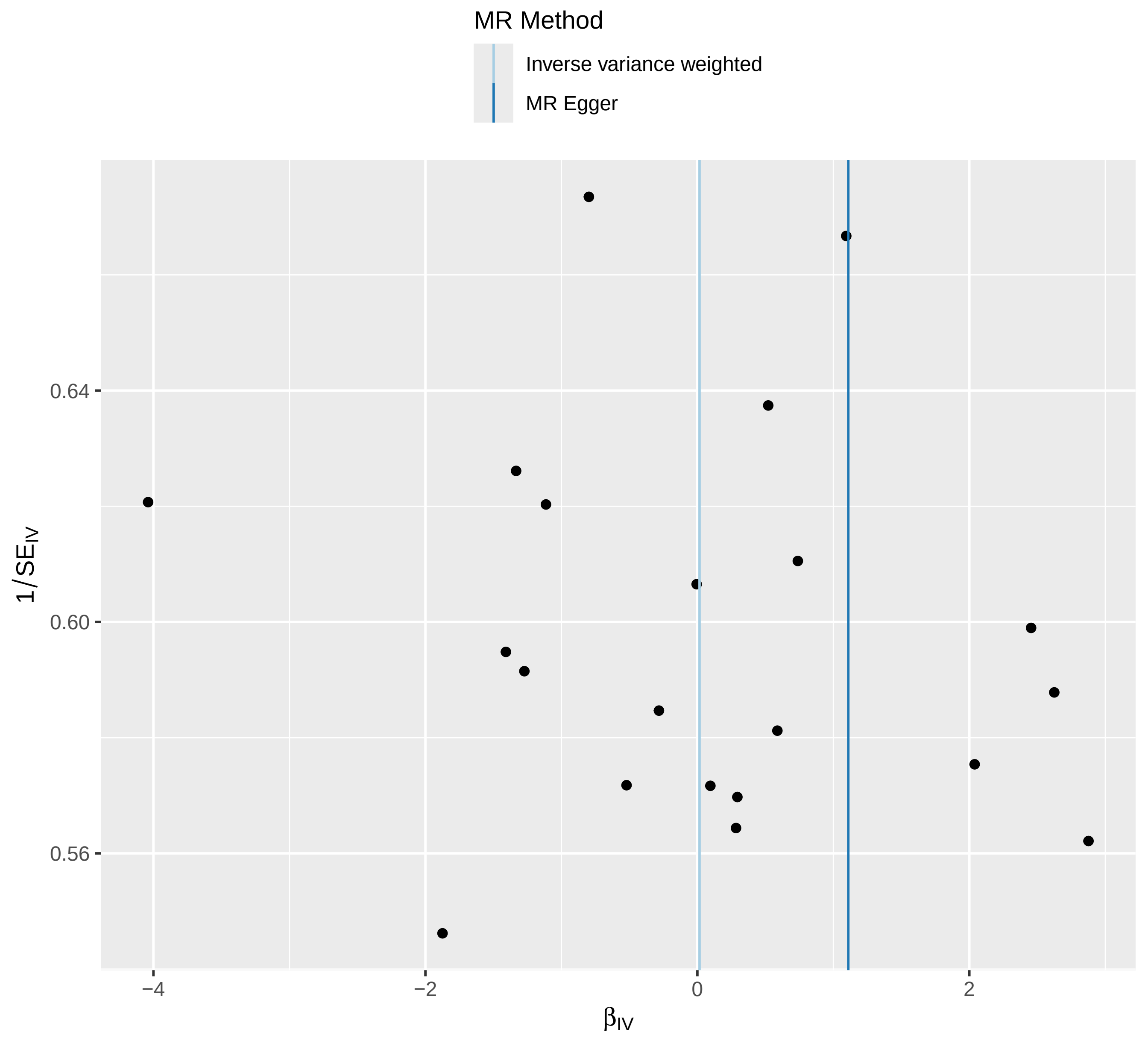

MR Method
Inverse variance weighted
MR Egger
0.64
0.60
0.56
$1/SE_{IV}$
−4
−2
0
2
β_{IV}

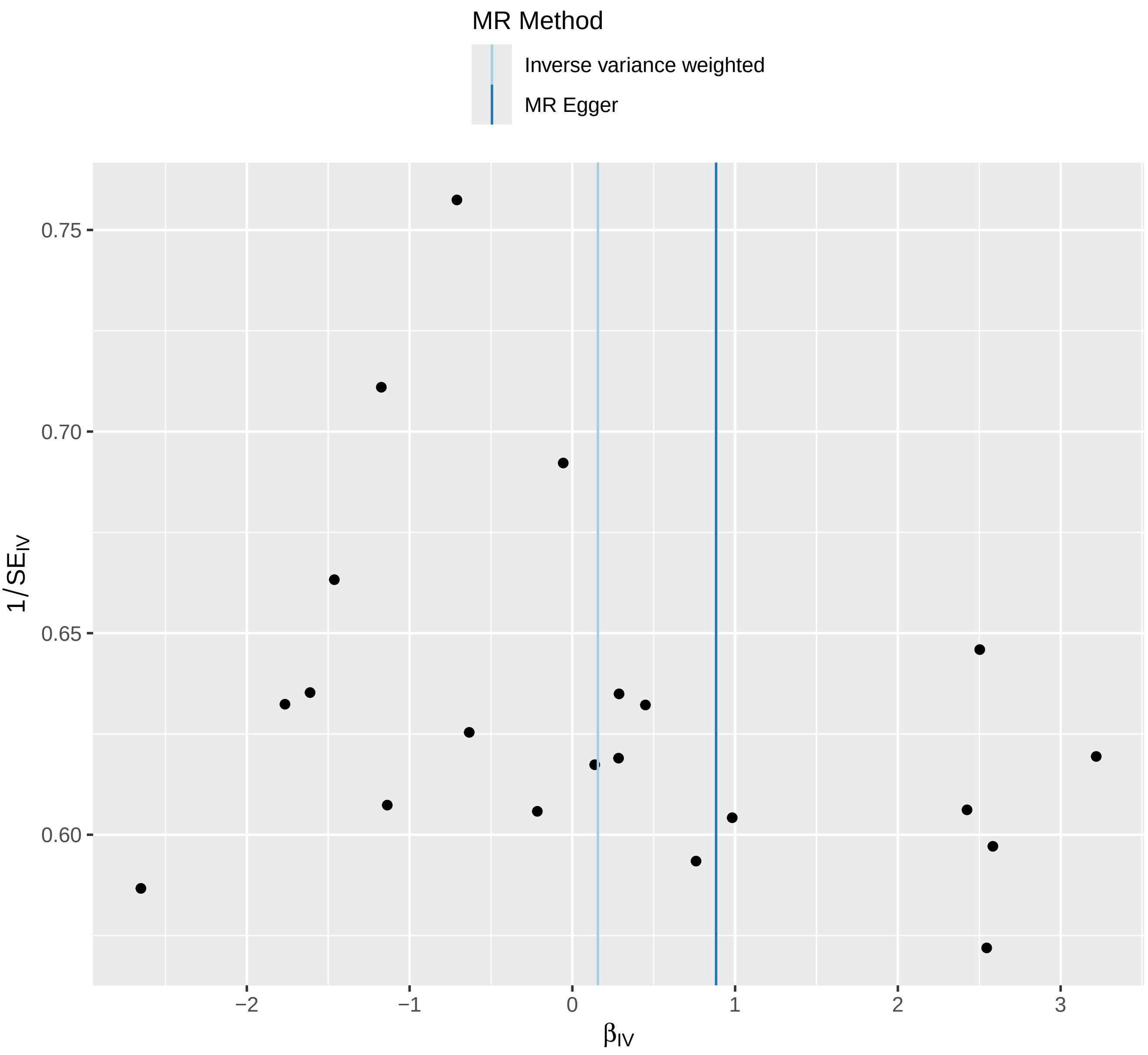
MR Method
Inverse variance weighted
MR Egger
0.75
0.70
0.65
0.60
$1/SE_{IV}$
−2
−1
0
1
2
3
β_{IV}

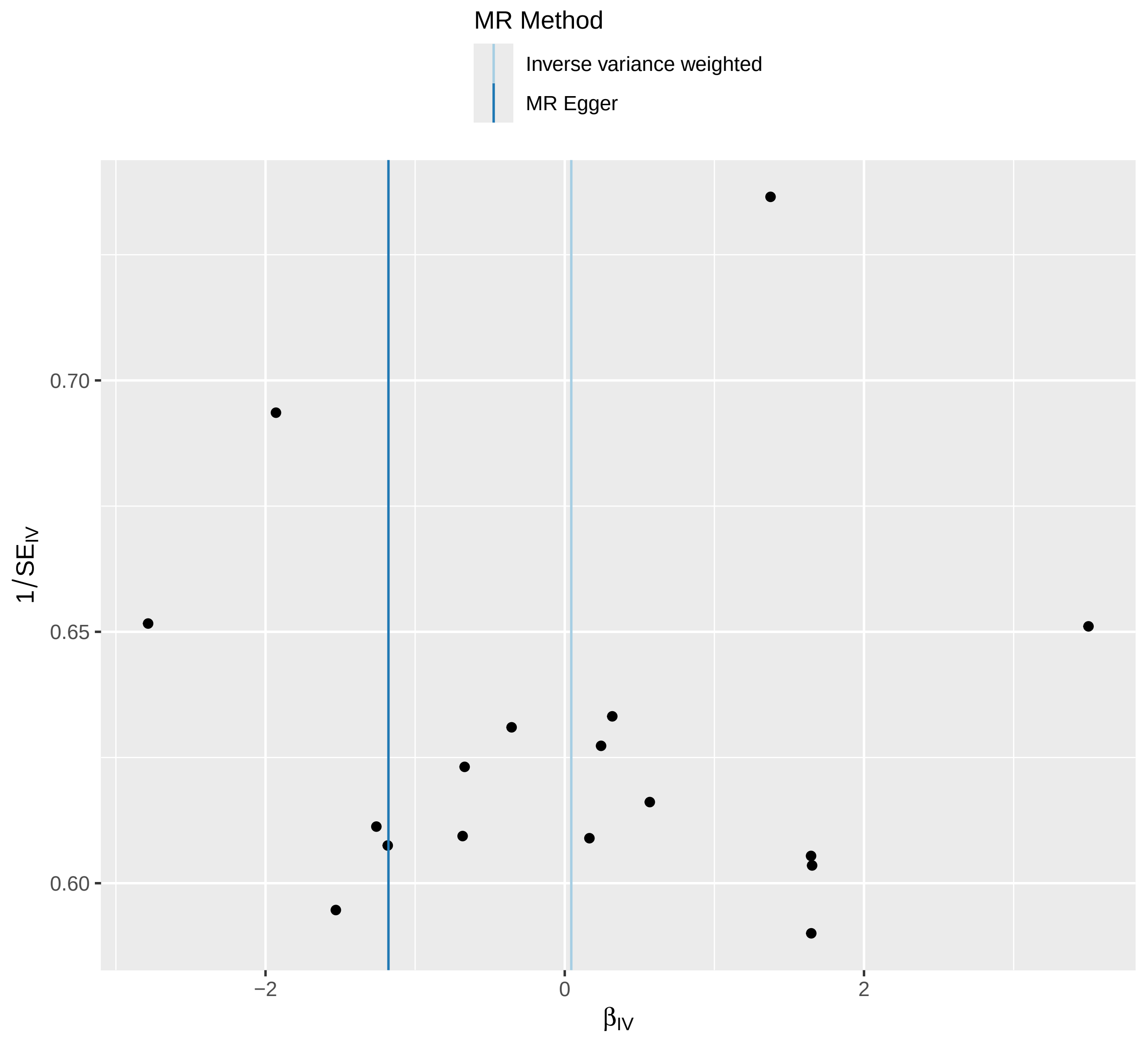
MR Method
Inverse variance weighted
MR Egger
0.70
0.65
0.60
1/SE_IV
−2
0
2
β_IV

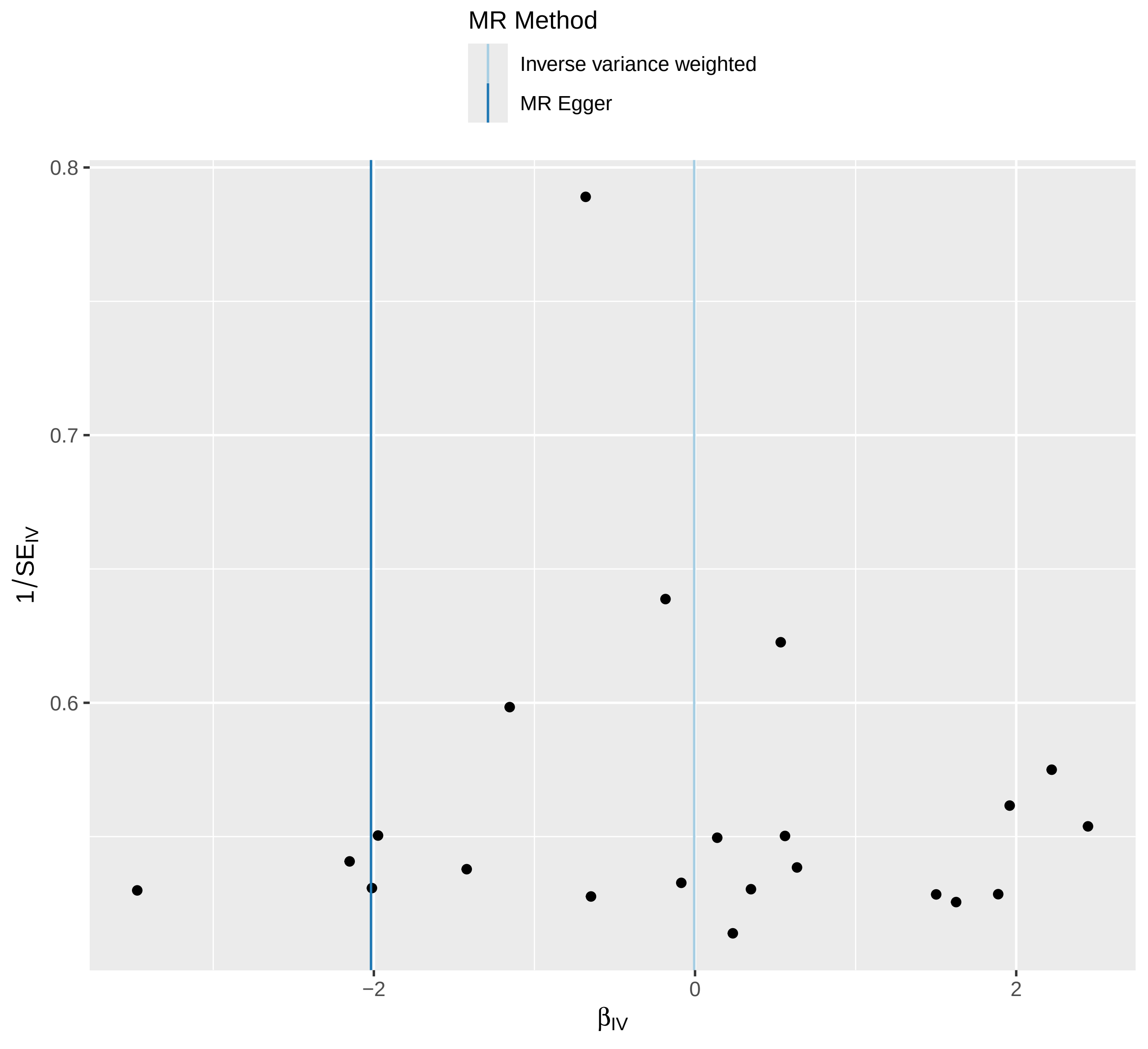
MR Method
Inverse variance weighted
MR Egger
0.8
0.7
0.6
1/SE_IV
−2
0
2
β_IV

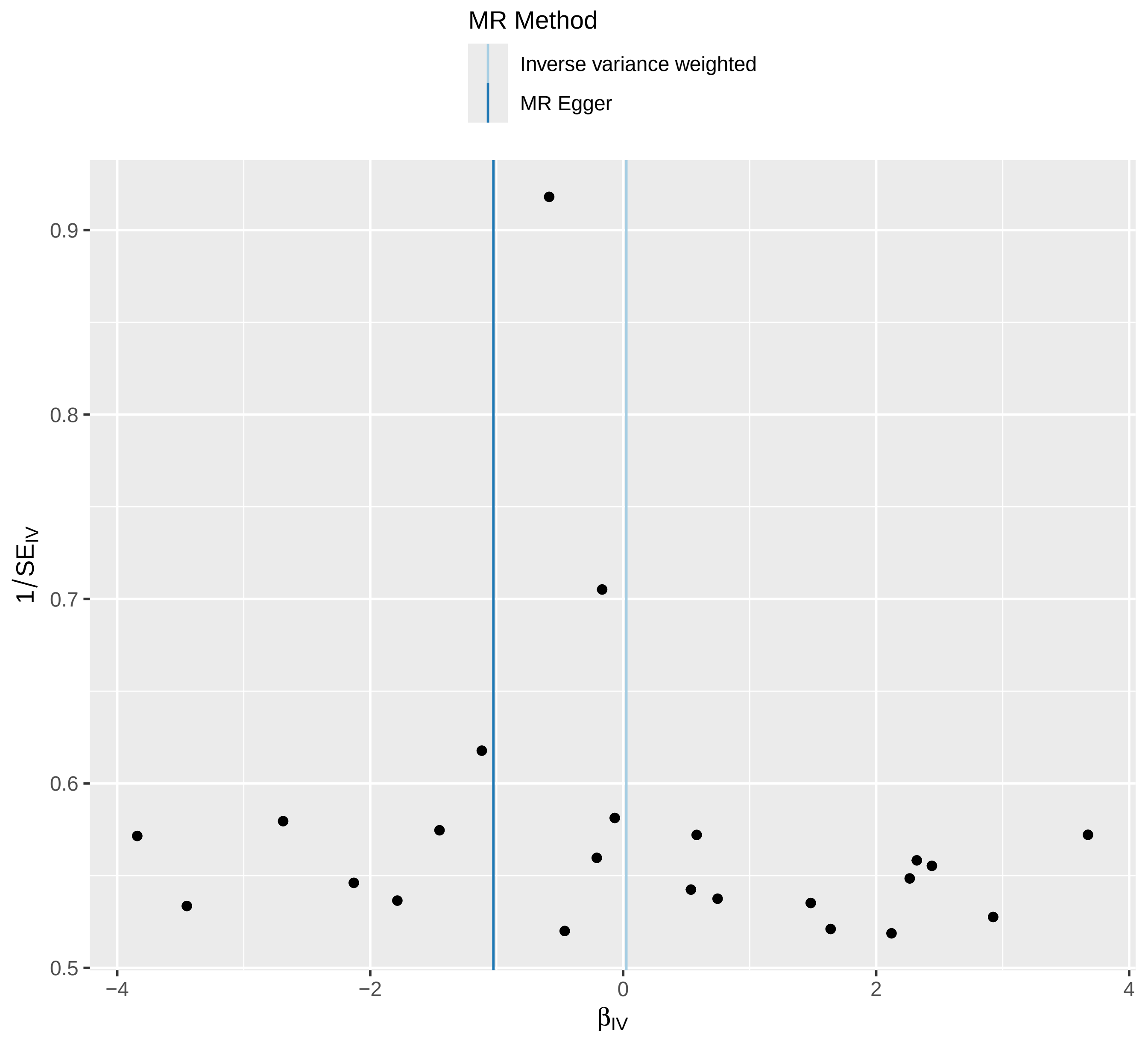
MR Method
Inverse variance weighted
MR Egger
1/SE_IV
0.9
0.8
0.7
0.6
0.5
−4
−2
0
2
4
β_IV

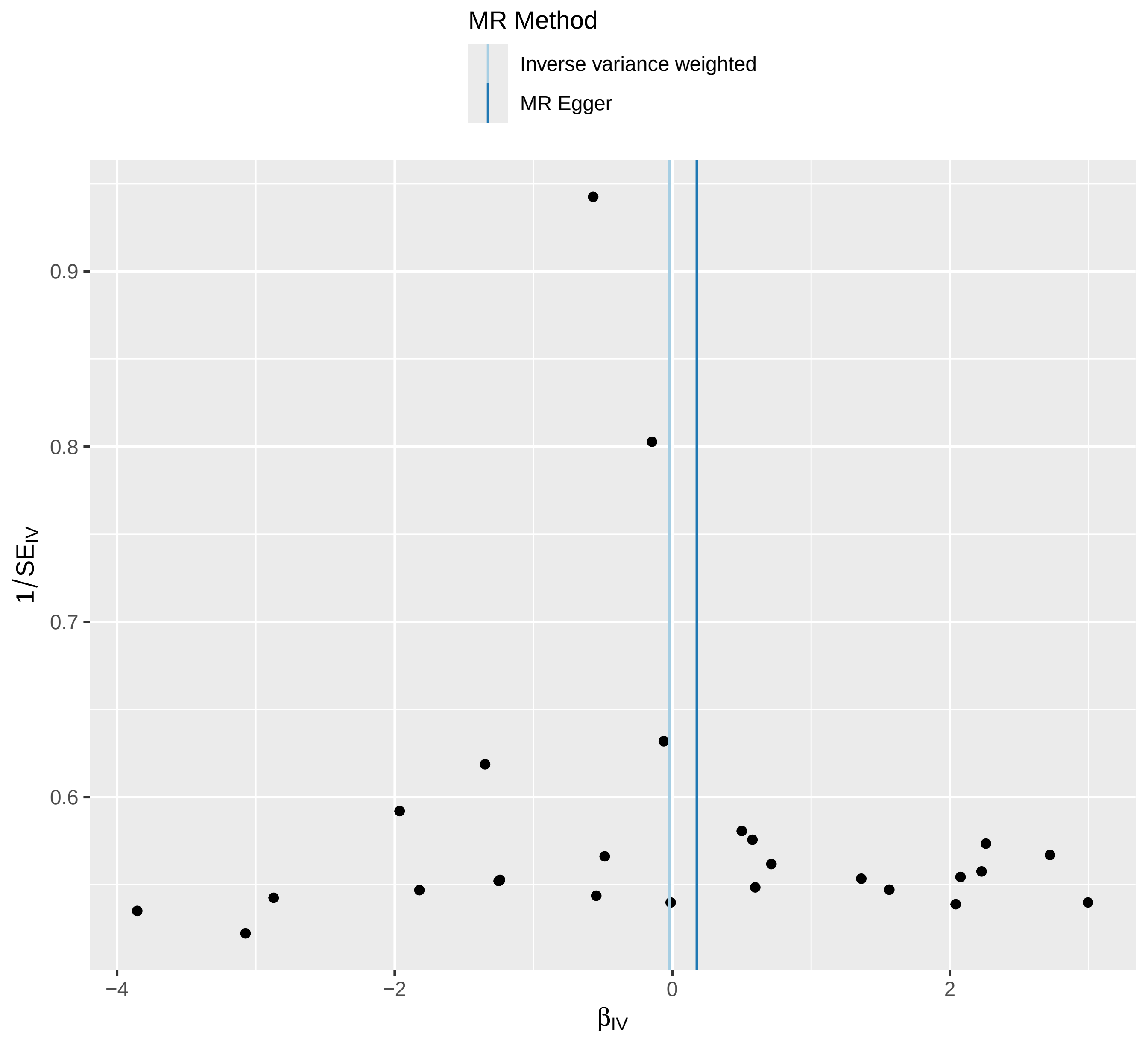
MR Method
Inverse variance weighted
MR Egger
0.9
0.8
0.7
0.6
1/SE$_{IV}$
−4
−2
0
2
β$_{IV}$

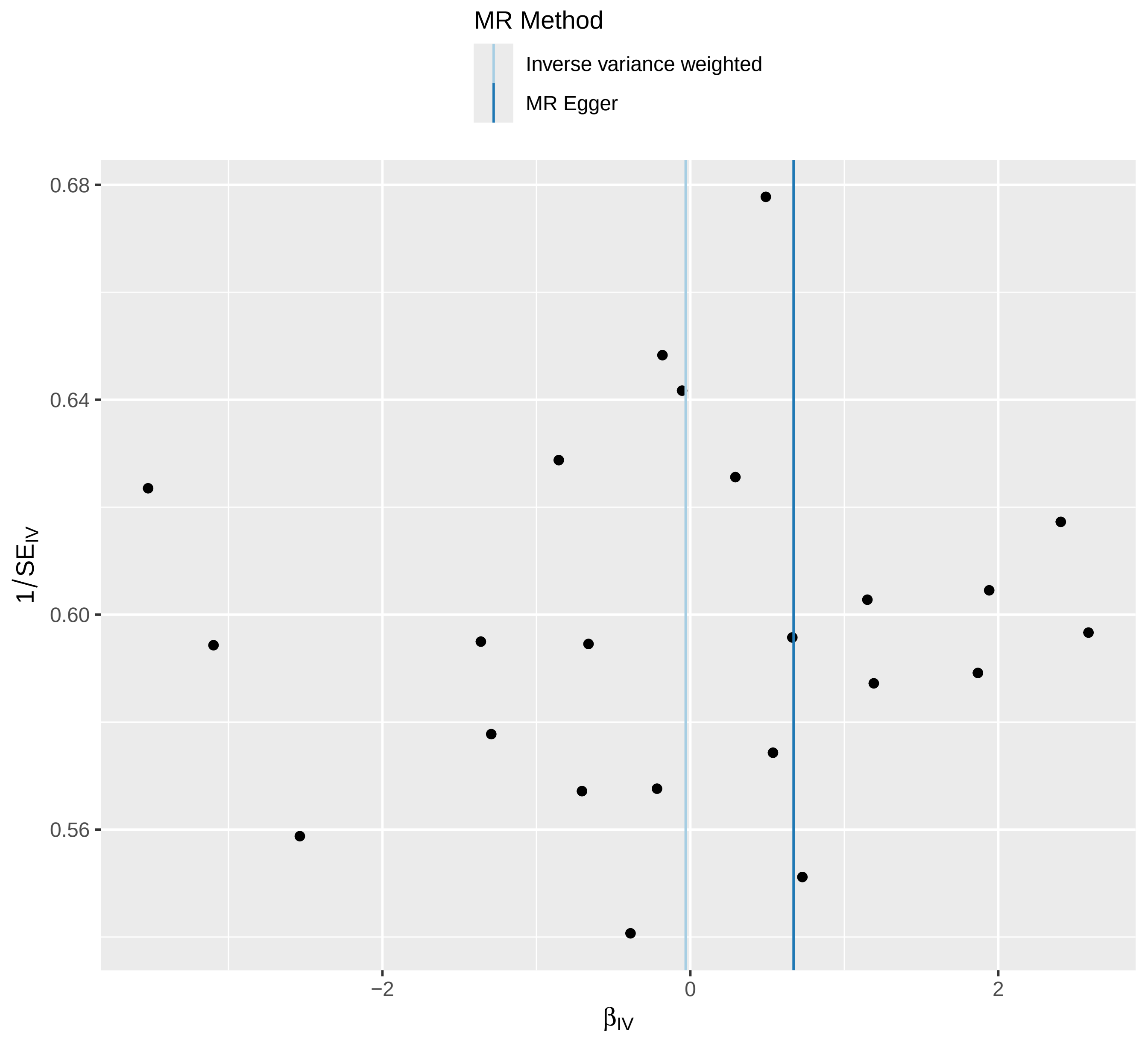
MR Method
Inverse variance weighted
MR Egger
0.68
0.64
0.60
0.56
1/SE_IV
−2
0
2
β_IV

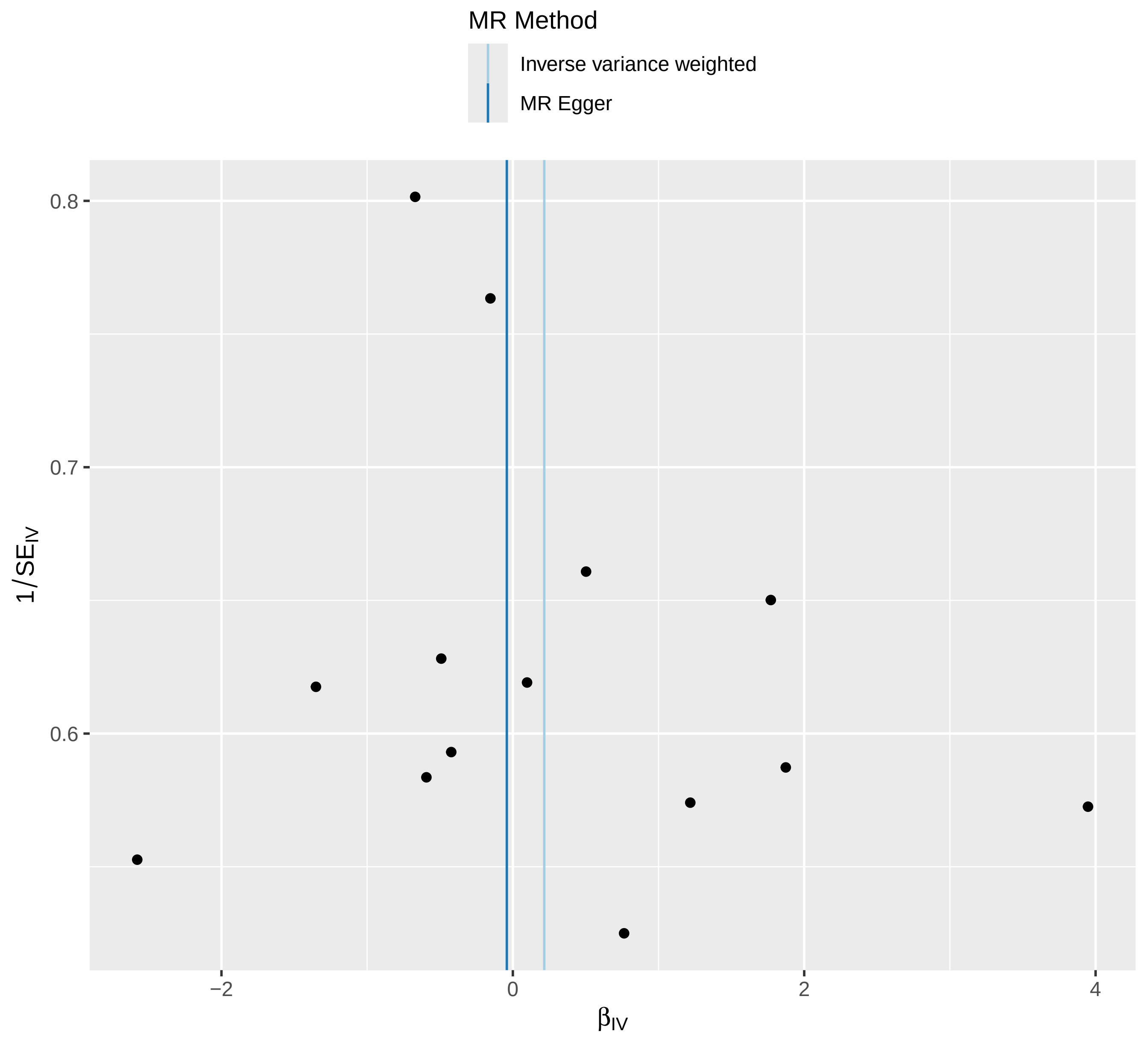

MR Method
Inverse variance weighted
MR Egger
0.8
0.7
0.6
1/SE$_{IV}$
−2
0
2
4
β$_{IV}$

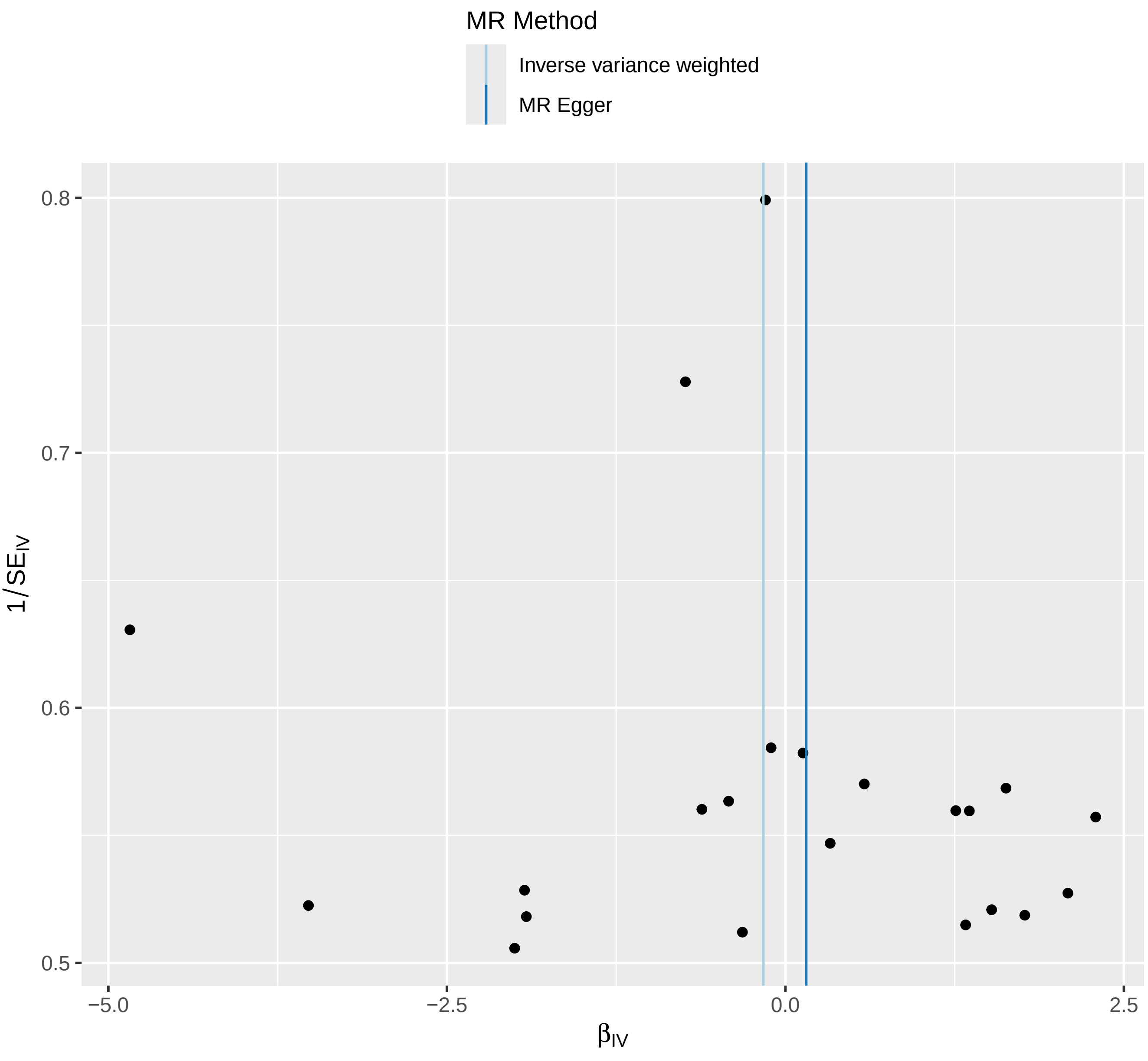

MR Method
Inverse variance weighted
MR Egger
0.8
0.7
0.6
0.5
1/SE_IV
−5.0
−2.5
0.0
2.5
β_IV

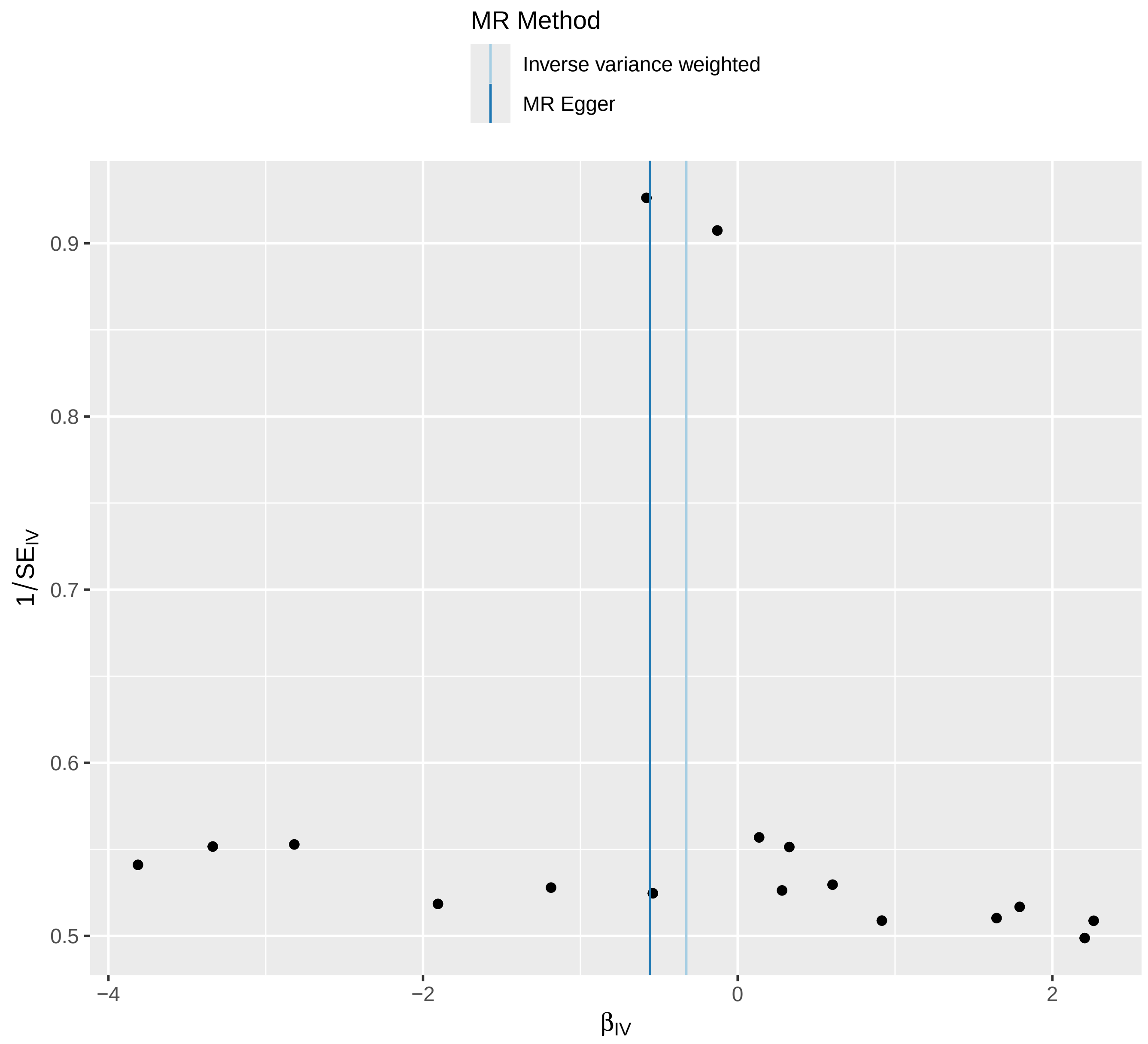

MR Method
Inverse variance weighted
MR Egger
0.9
0.8
0.7
0.6
0.5
1/SE_IV
−4
−2
0
2
β_IV

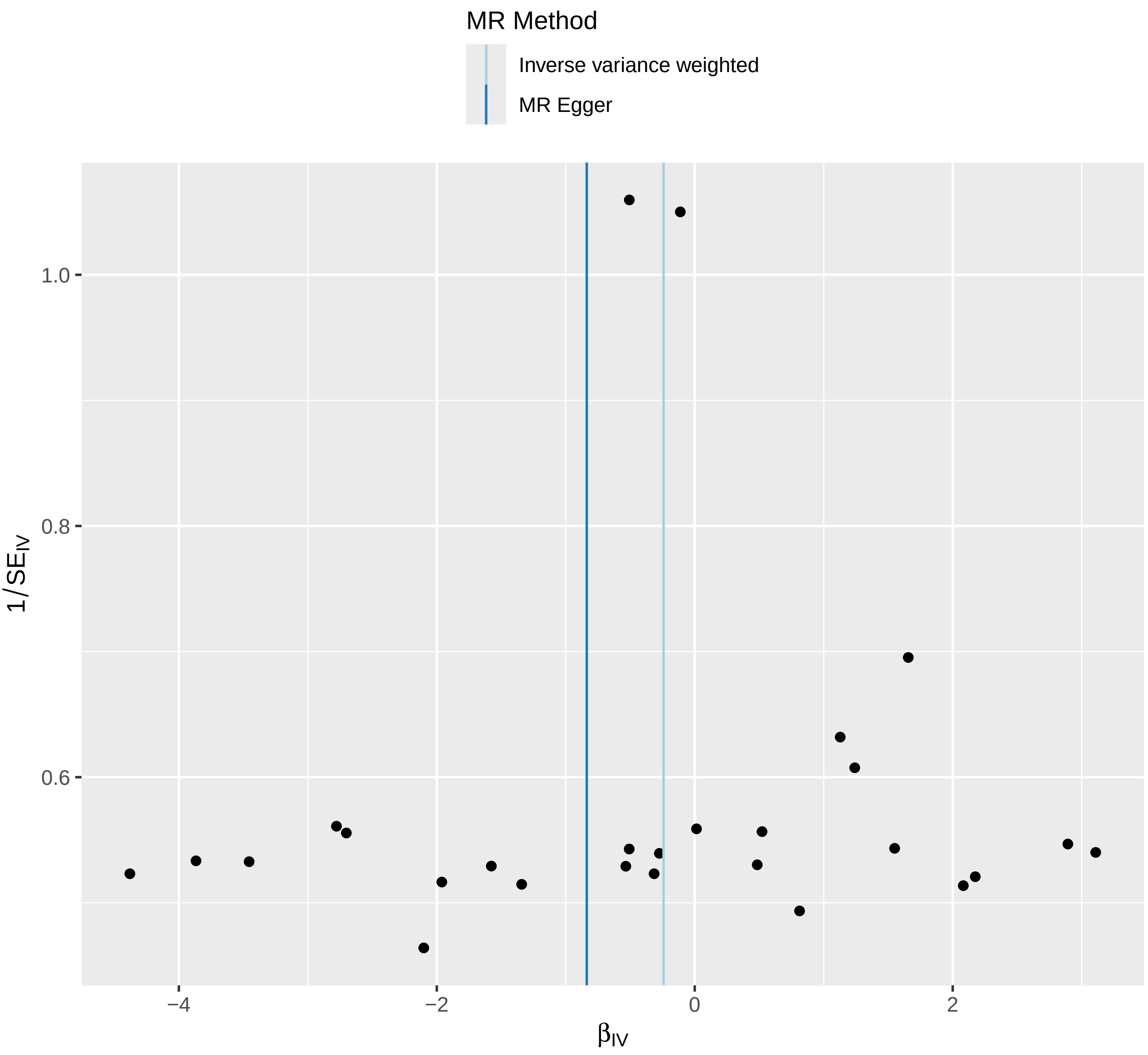
MR Method
Inverse variance weighted
MR Egger
1.0
0.8
0.6
1/SE$_{IV}$
−4
−2
0
2
β$_{IV}$

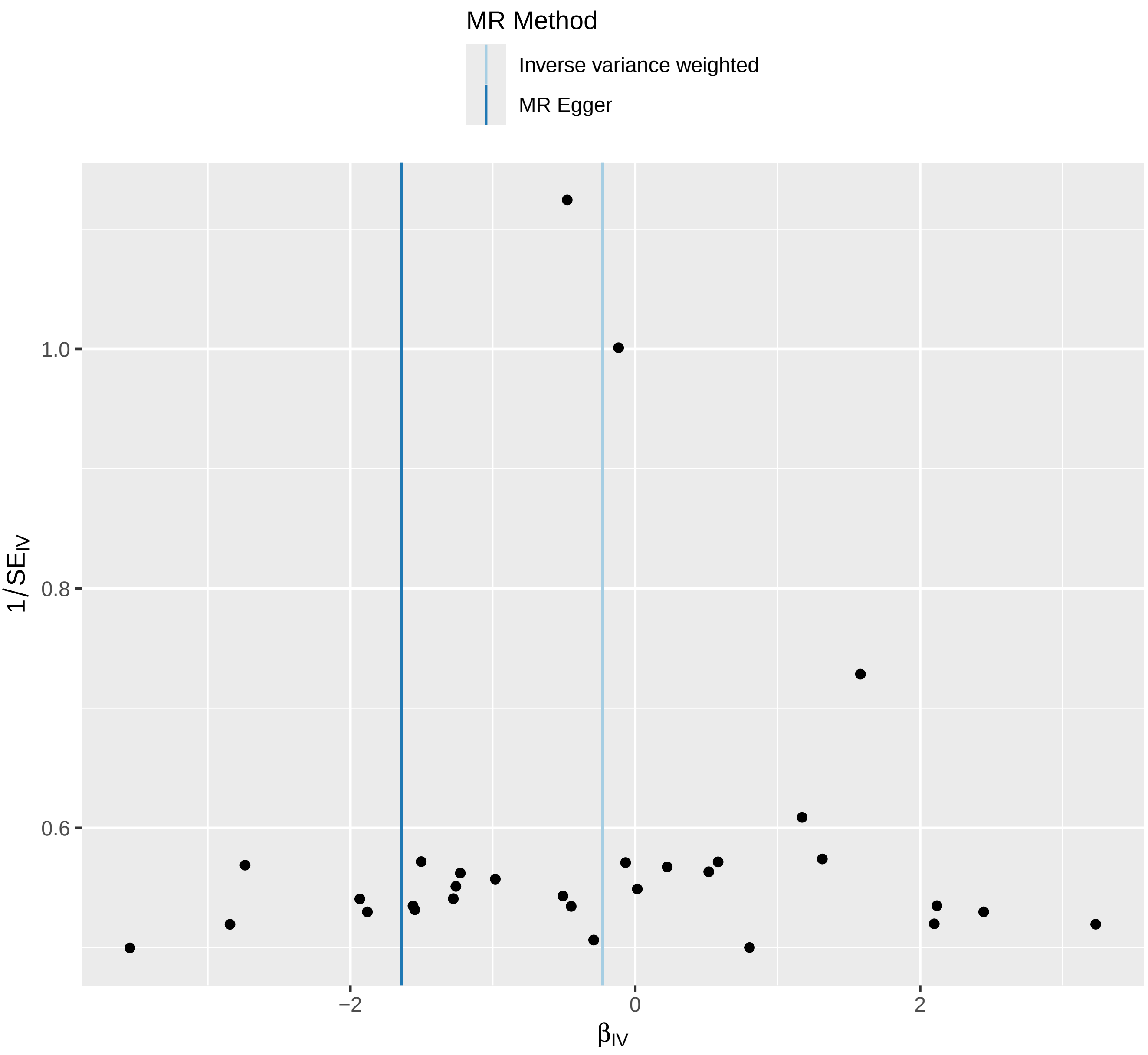
MR Method
Inverse variance weighted
MR Egger
1/SE$_{IV}$
1.0
0.8
0.6
−2
0
2
β$_{IV}$

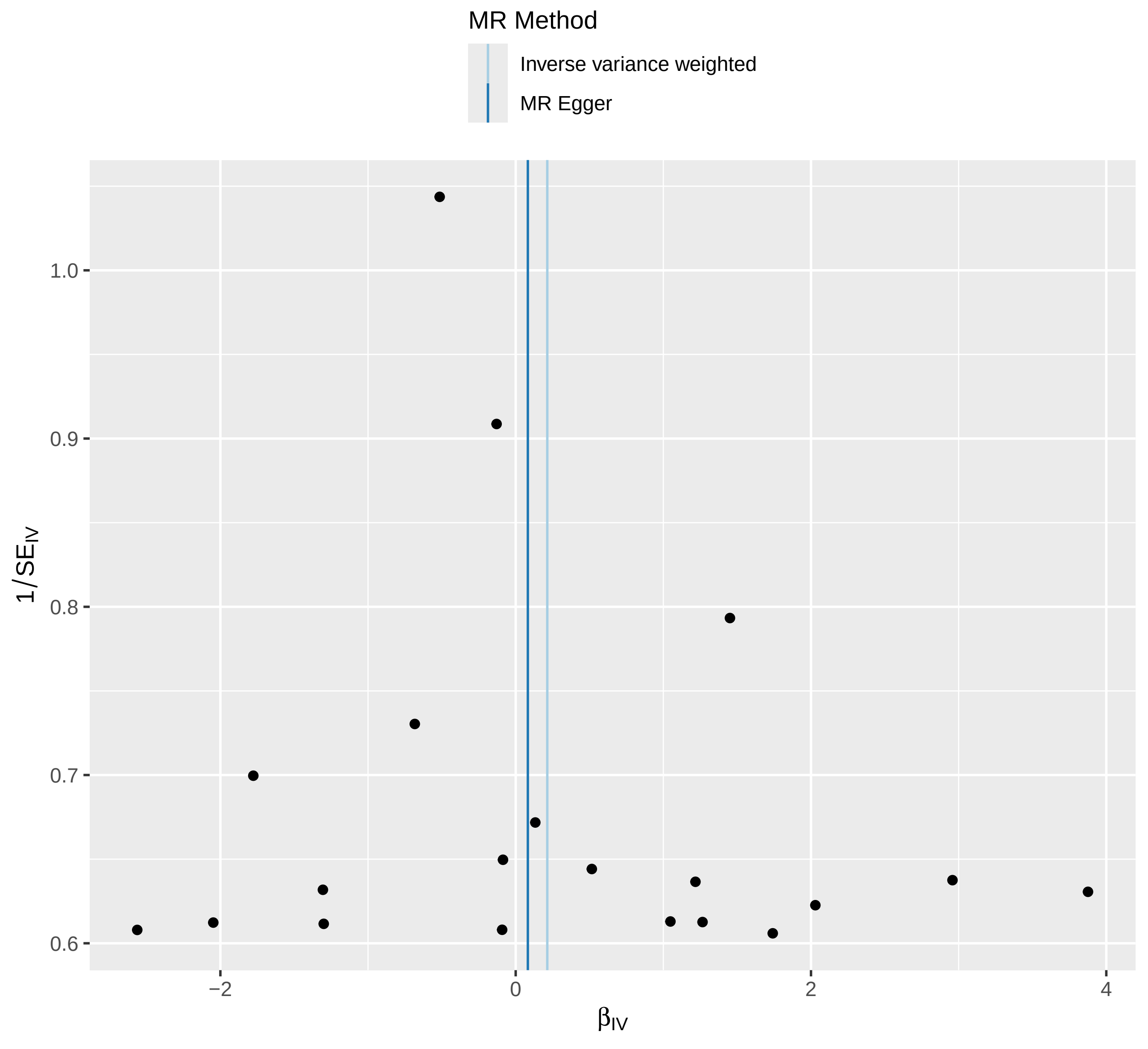
MR Method
Inverse variance weighted
MR Egger
1/SE_IV
1.0
0.9
0.8
0.7
0.6
−2
0
2
4
β_IV

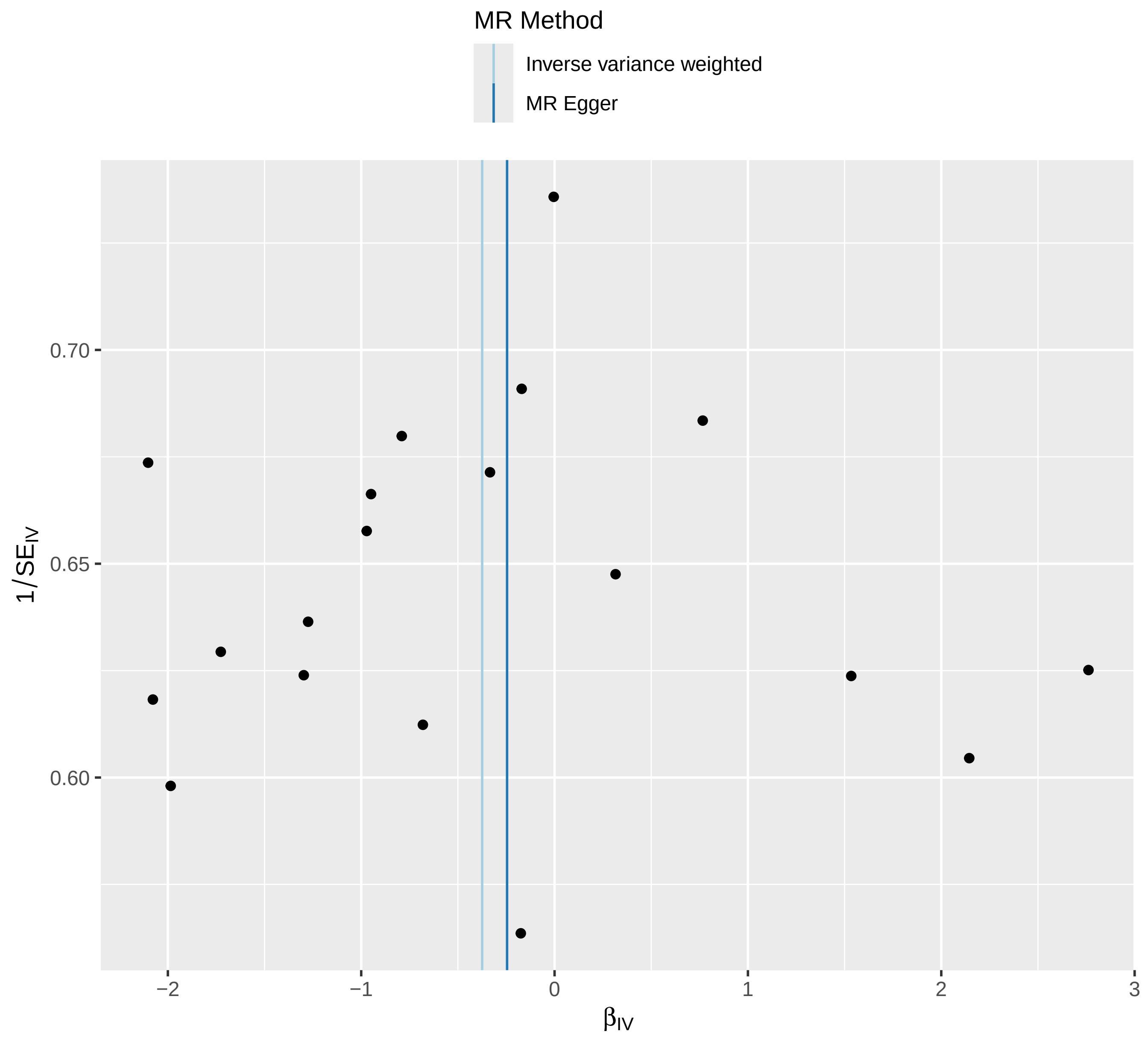
MR Method
Inverse variance weighted
MR Egger
0.70
0.65
0.60
$1/SE_{IV}$
−2
−1
0
1
2
3
β_{IV}

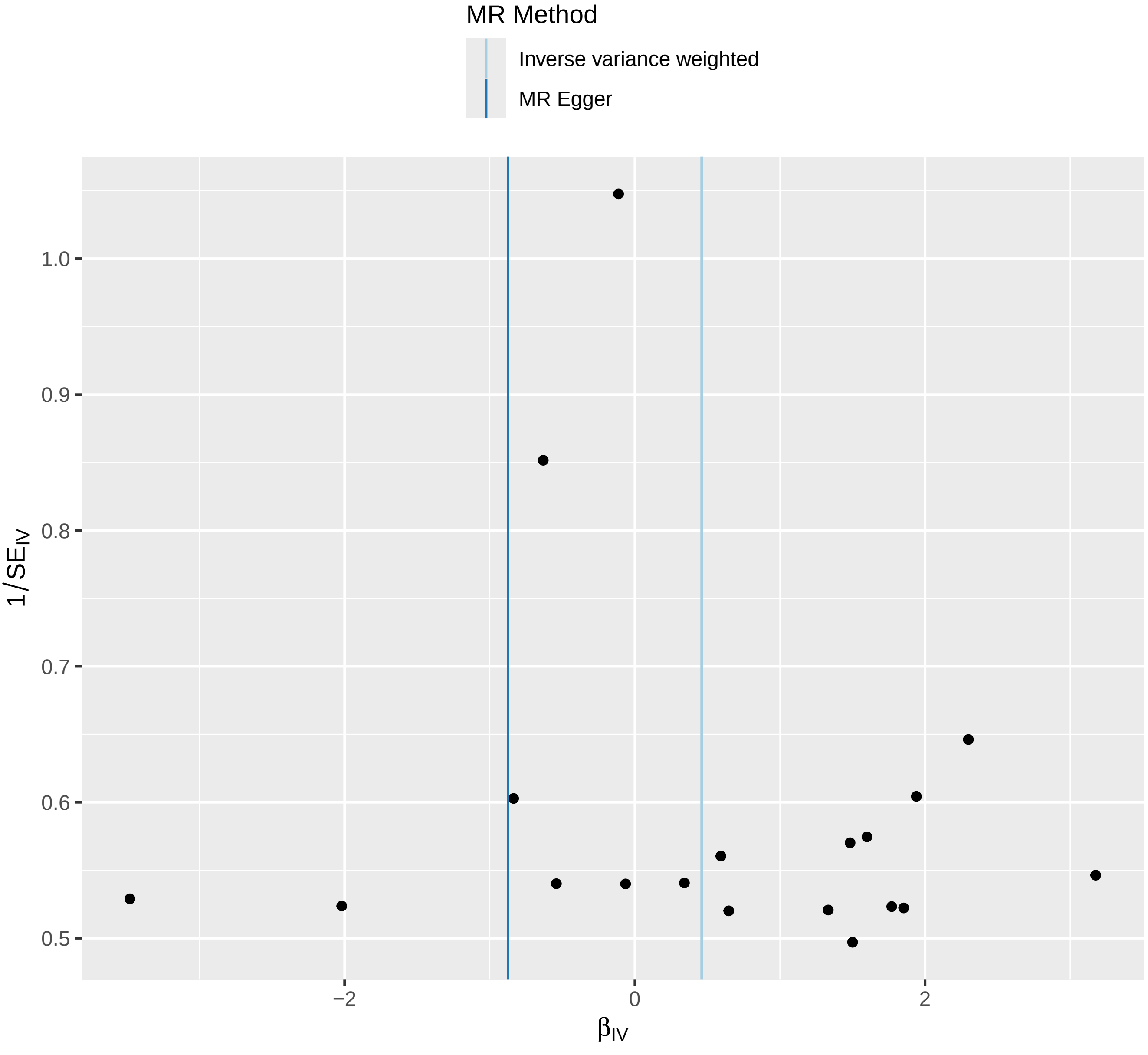
MR Method
Inverse variance weighted
MR Egger
1.0
0.9
0.8
0.7
0.6
0.5
1/SE_IV
−2
0
2
β_IV

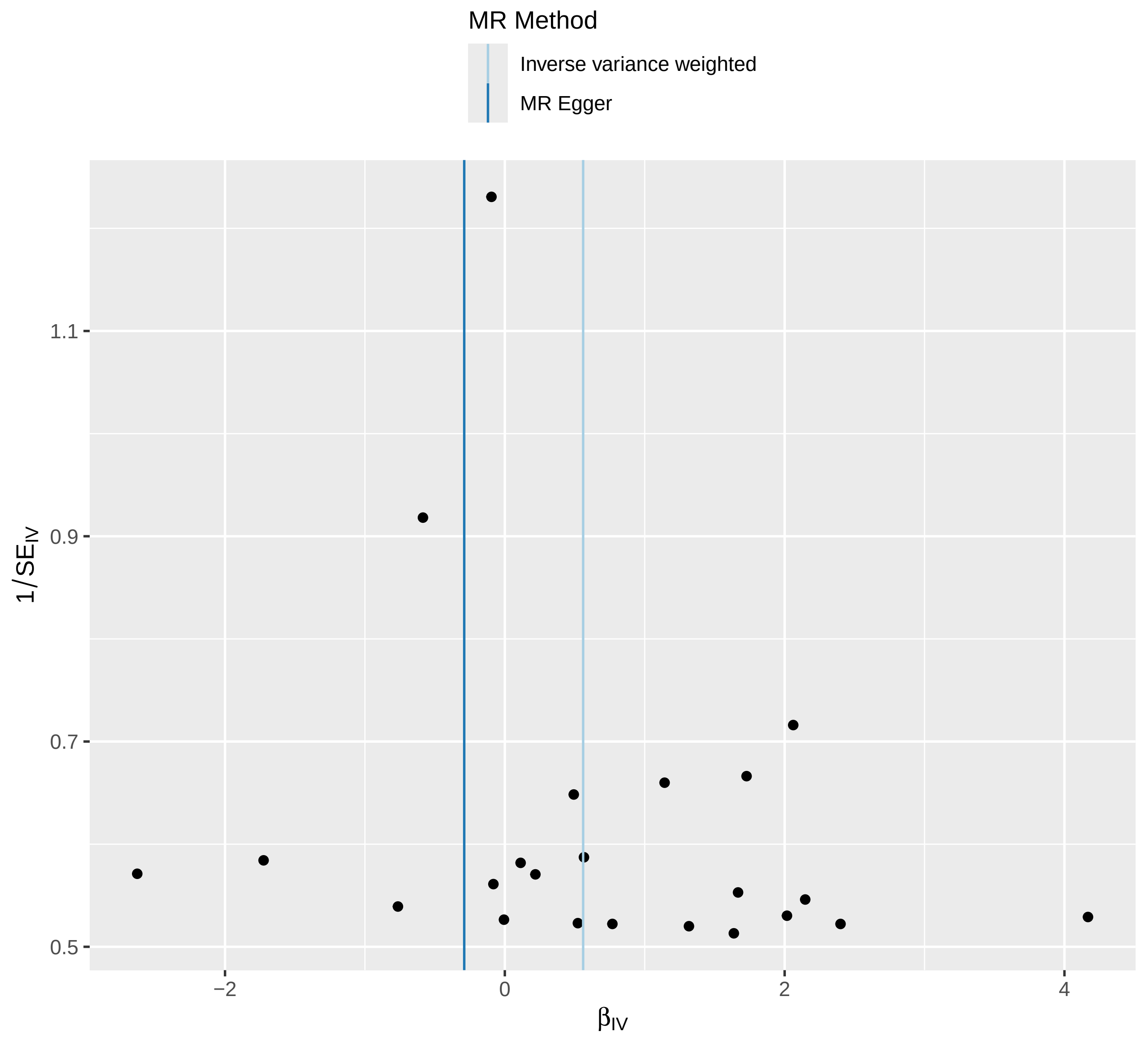
MR Method
Inverse variance weighted
MR Egger
1.1
0.9
0.7
0.5
1/SE$_{IV}$
−2
0
2
4
β$_{IV}$

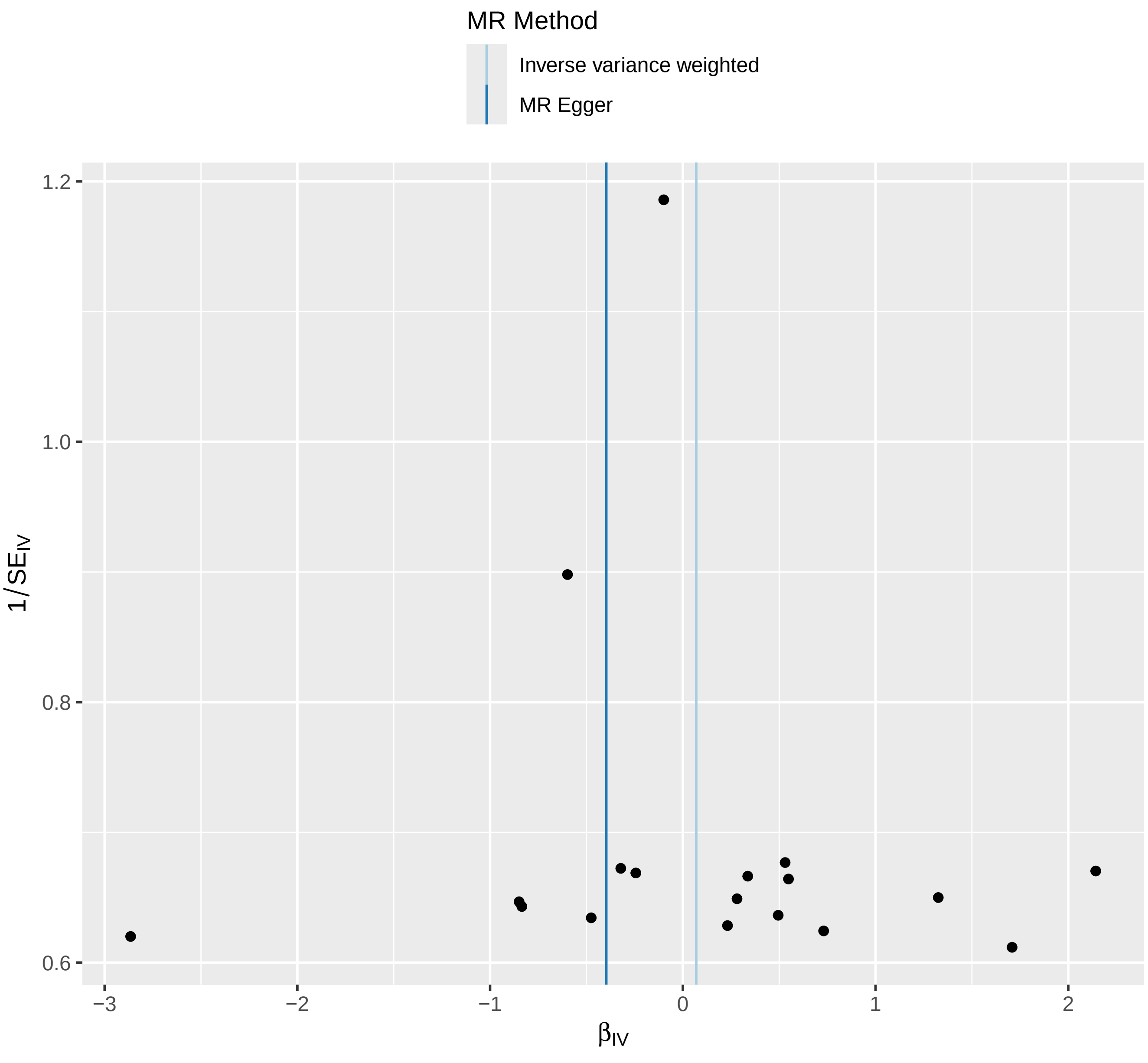
MR Method
Inverse variance weighted
MR Egger
1.2
1.0
0.8
0.6
−3
−2
−1
0
1
2
$1/SE_{IV}$
β_{IV}

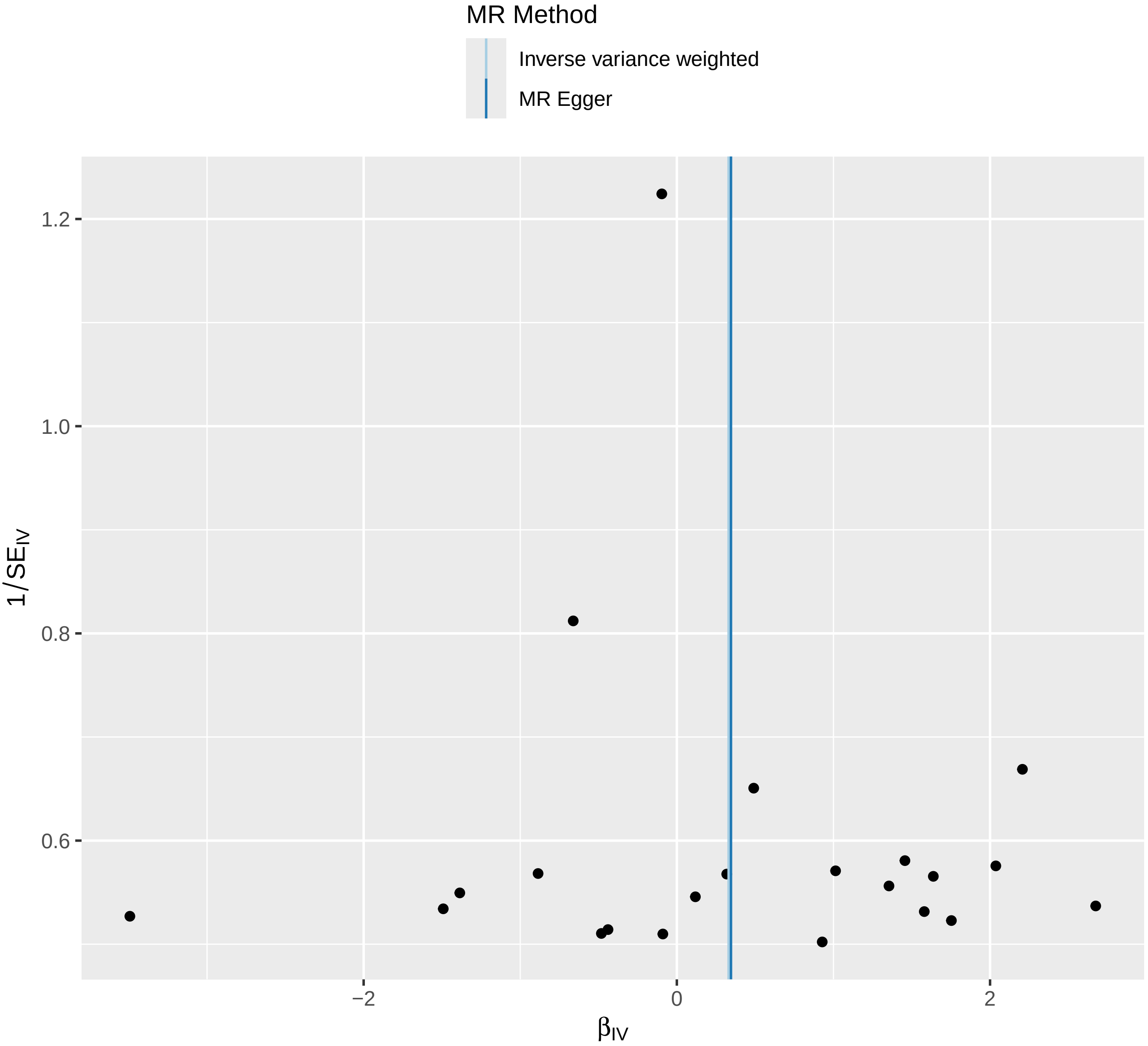
MR Method
Inverse variance weighted
MR Egger
1.2
1.0
0.8
0.6
1/SE_IV
−2
0
2
β_IV

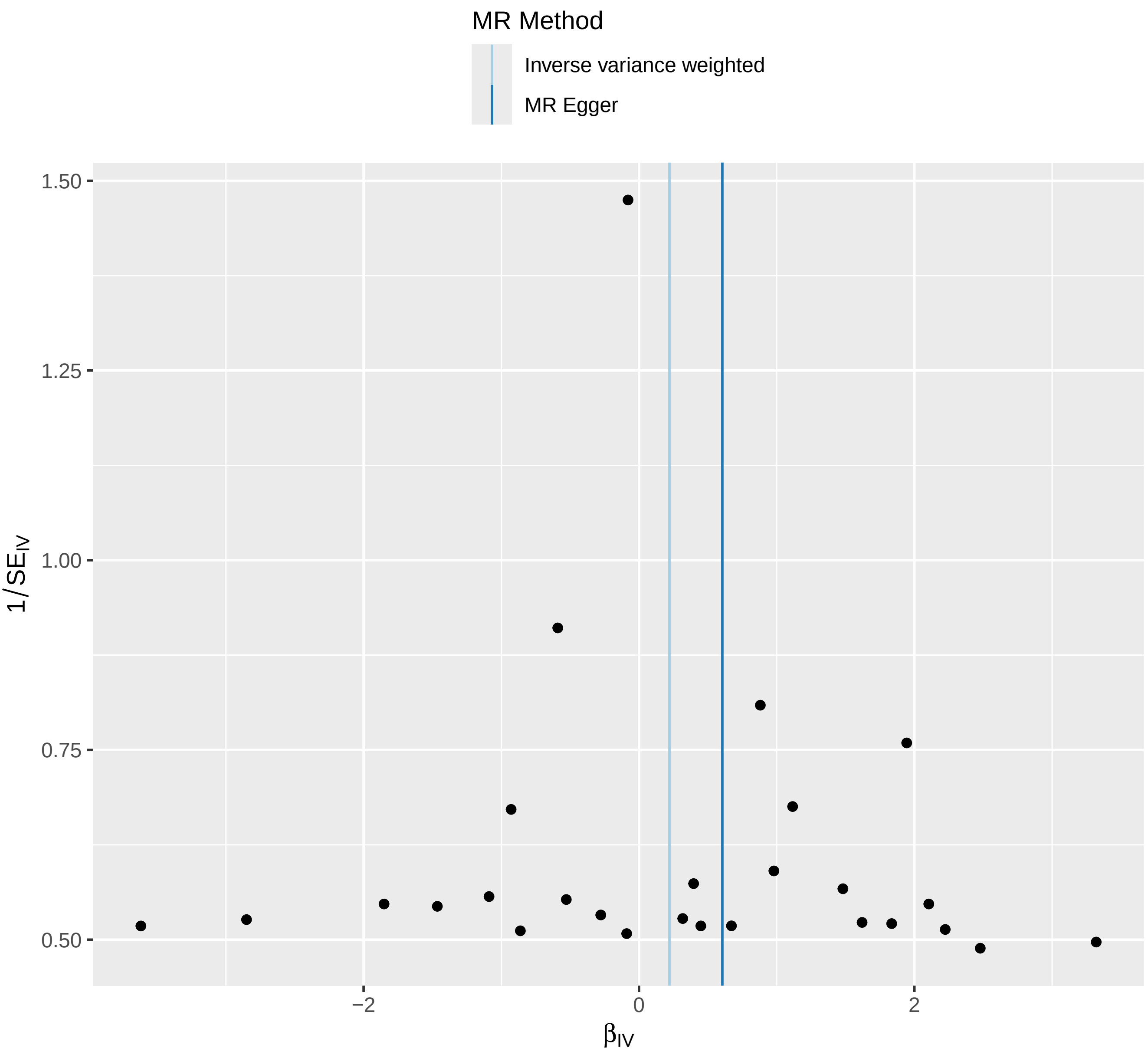
MR Method
Inverse variance weighted
MR Egger
1.50
1.25
1.00
0.75
0.50
1/SE_IV
−2
0
2
β_IV

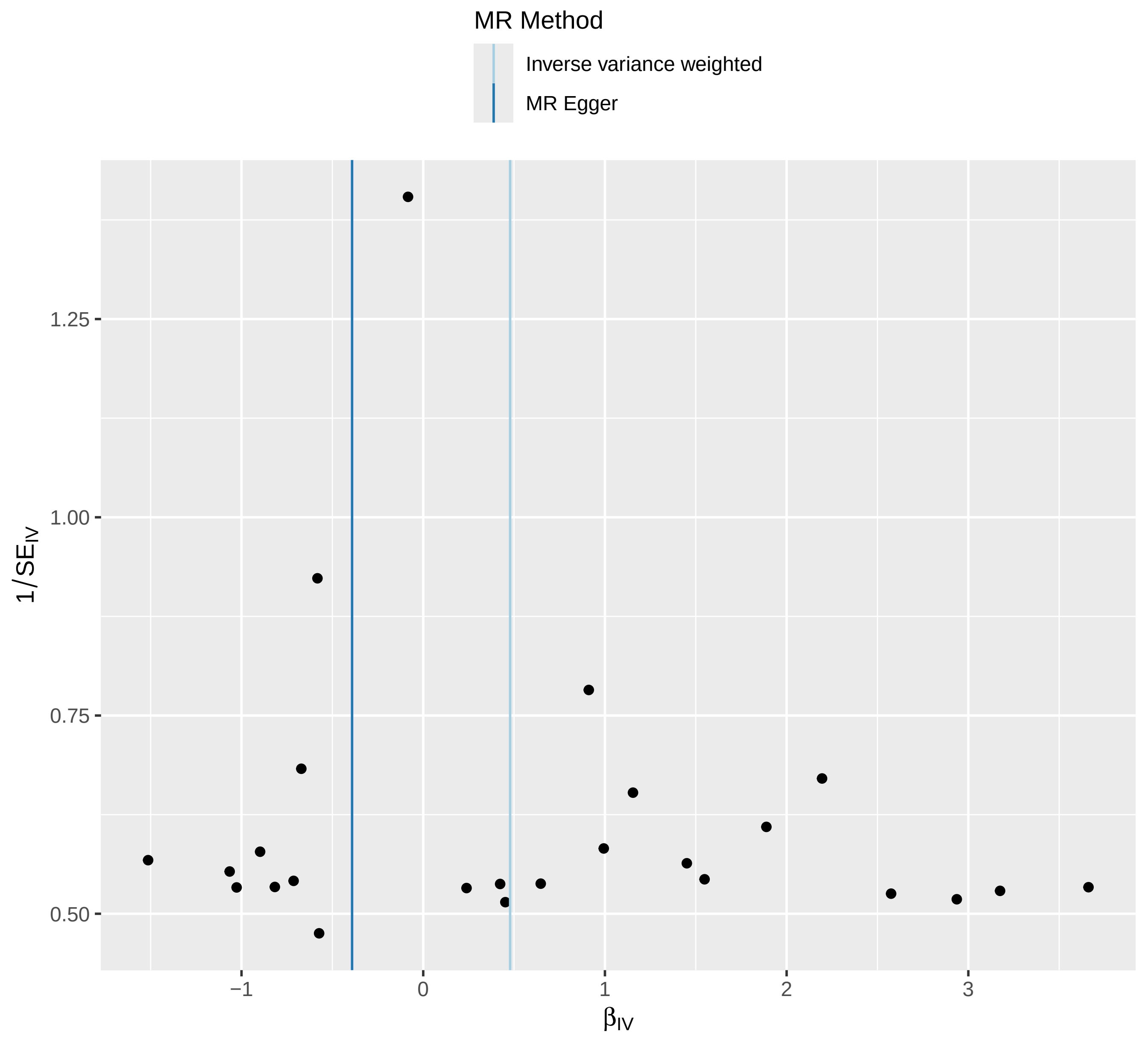
MR Method
Inverse variance weighted
MR Egger
1.25
1.00
0.75
0.50
1/SE_IV
−1
0
1
2
3
β_IV

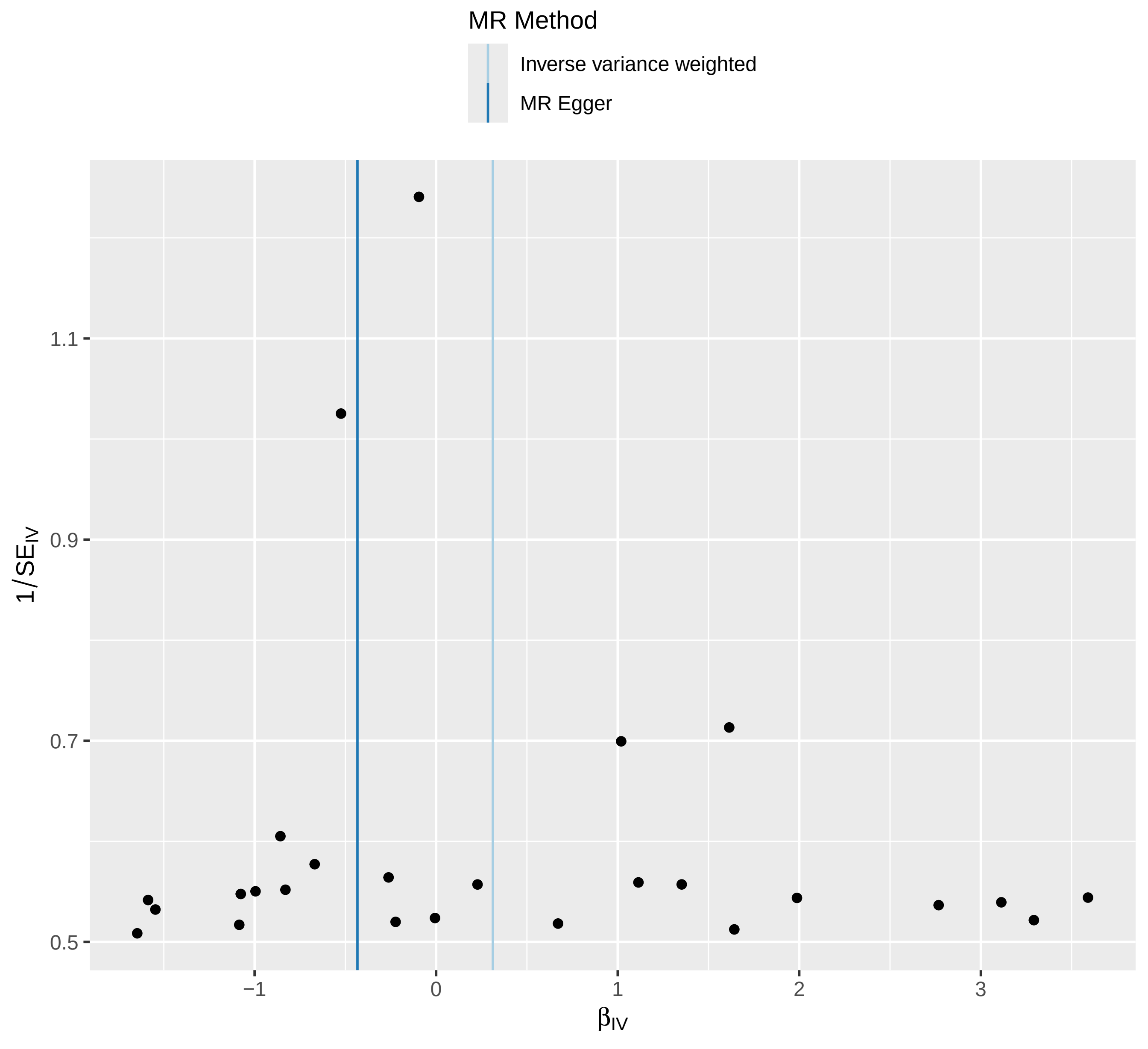
MR Method
Inverse variance weighted
MR Egger
1.1
0.9
0.7
0.5
1/SE_IV
−1
0
1
2
3
β_IV

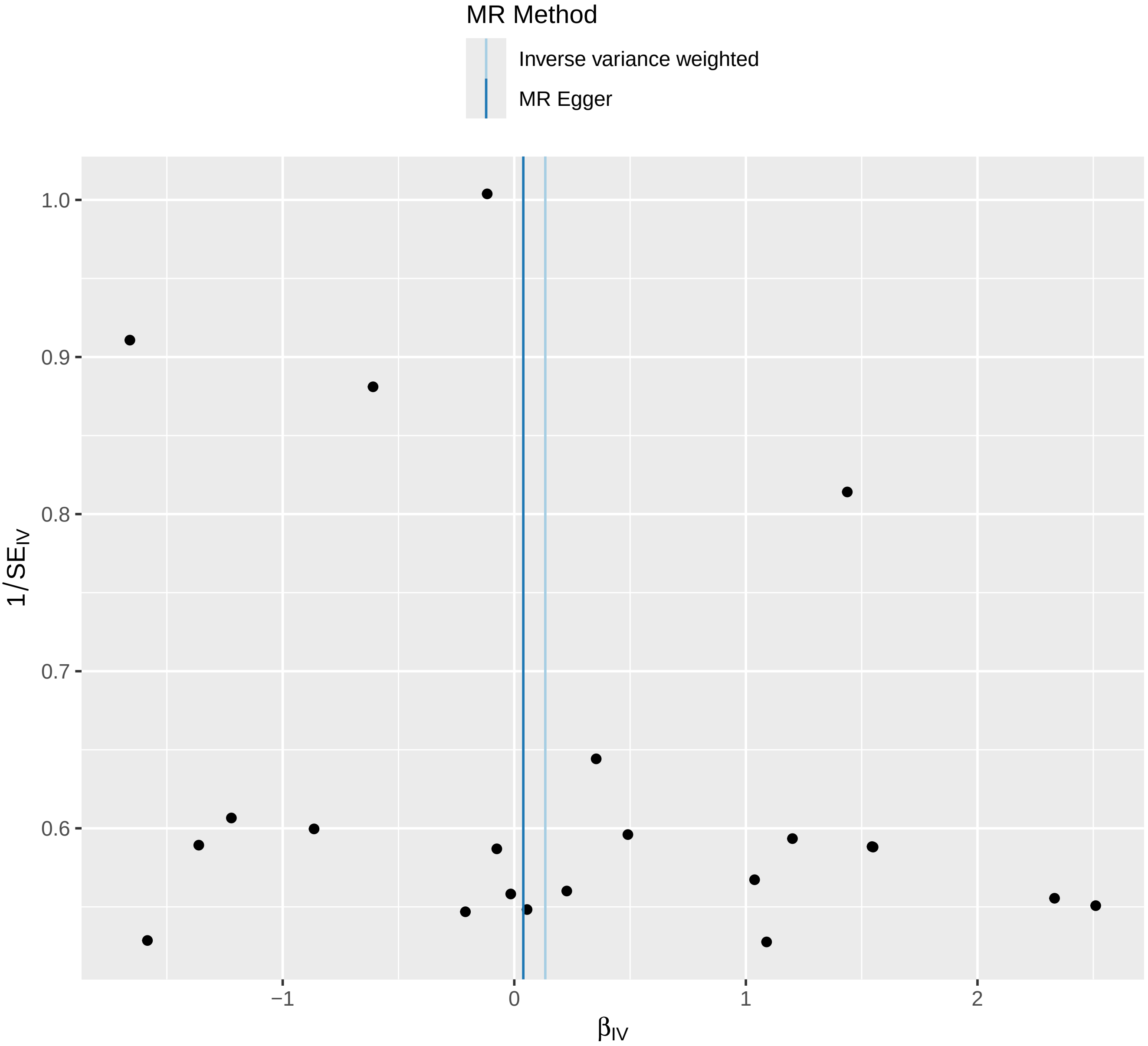
MR Method
Inverse variance weighted
MR Egger
1.0
0.9
0.8
0.7
0.6
1/SE_IV
−1
0
1
2
β_IV

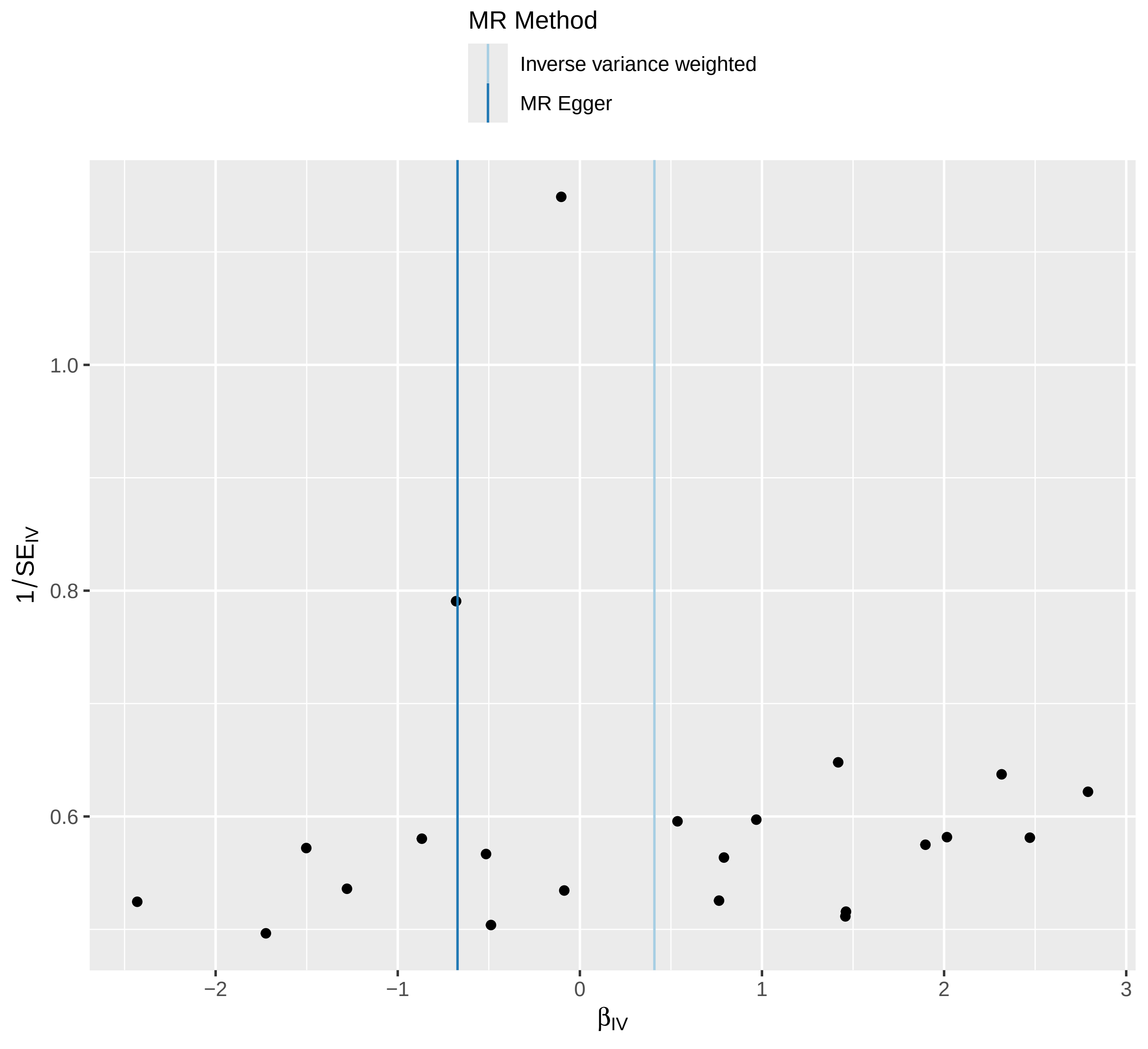

MR Method
Inverse variance weighted
MR Egger
1.0
0.8
0.6
1/SE$_{IV}$
−2
−1
0
1
2
3
β$_{IV}$

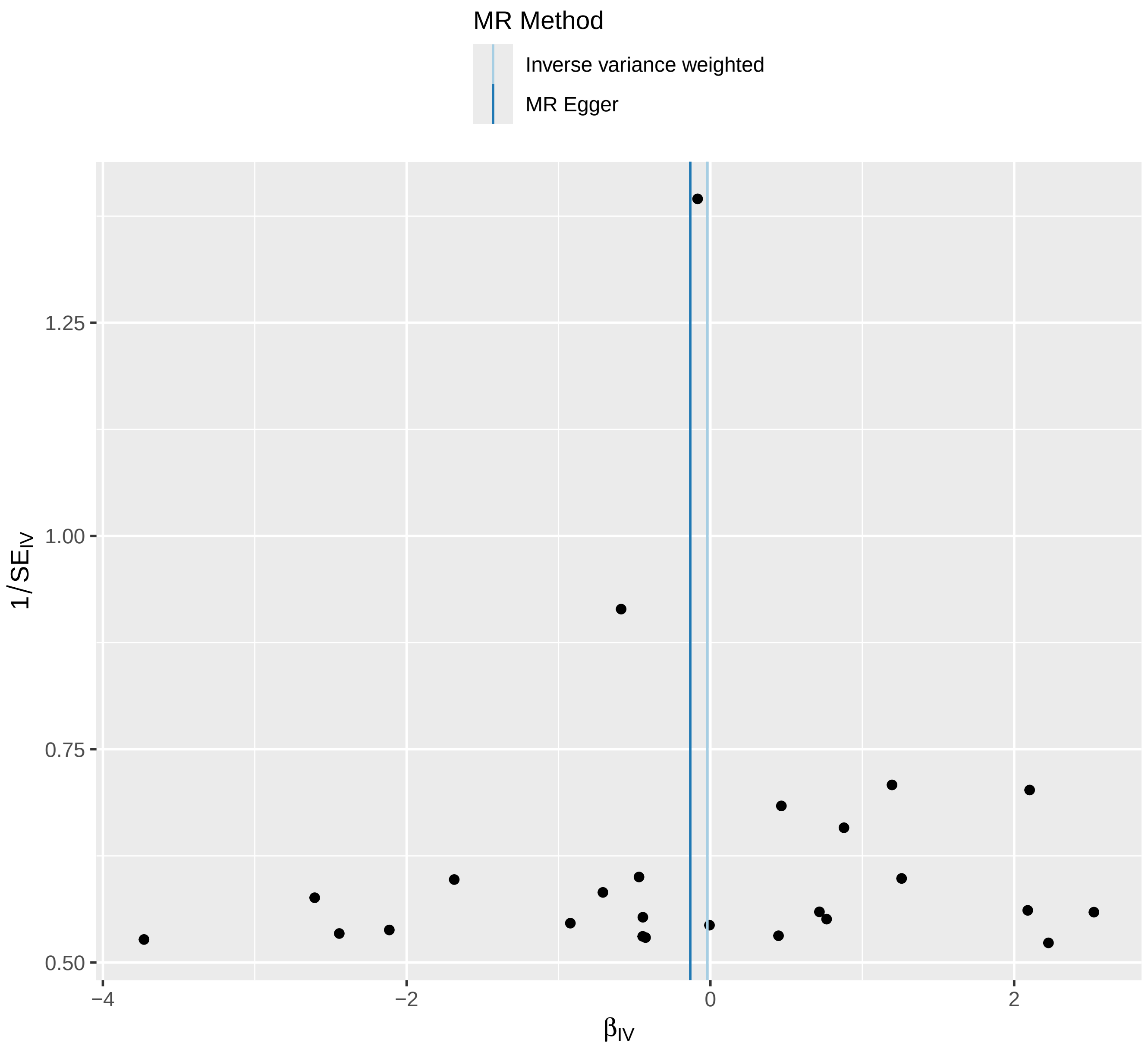
MR Method
Inverse variance weighted
MR Egger
1.25
1.00
0.75
0.50
$1/SE_{IV}$
−4
−2
0
2
β_{IV}

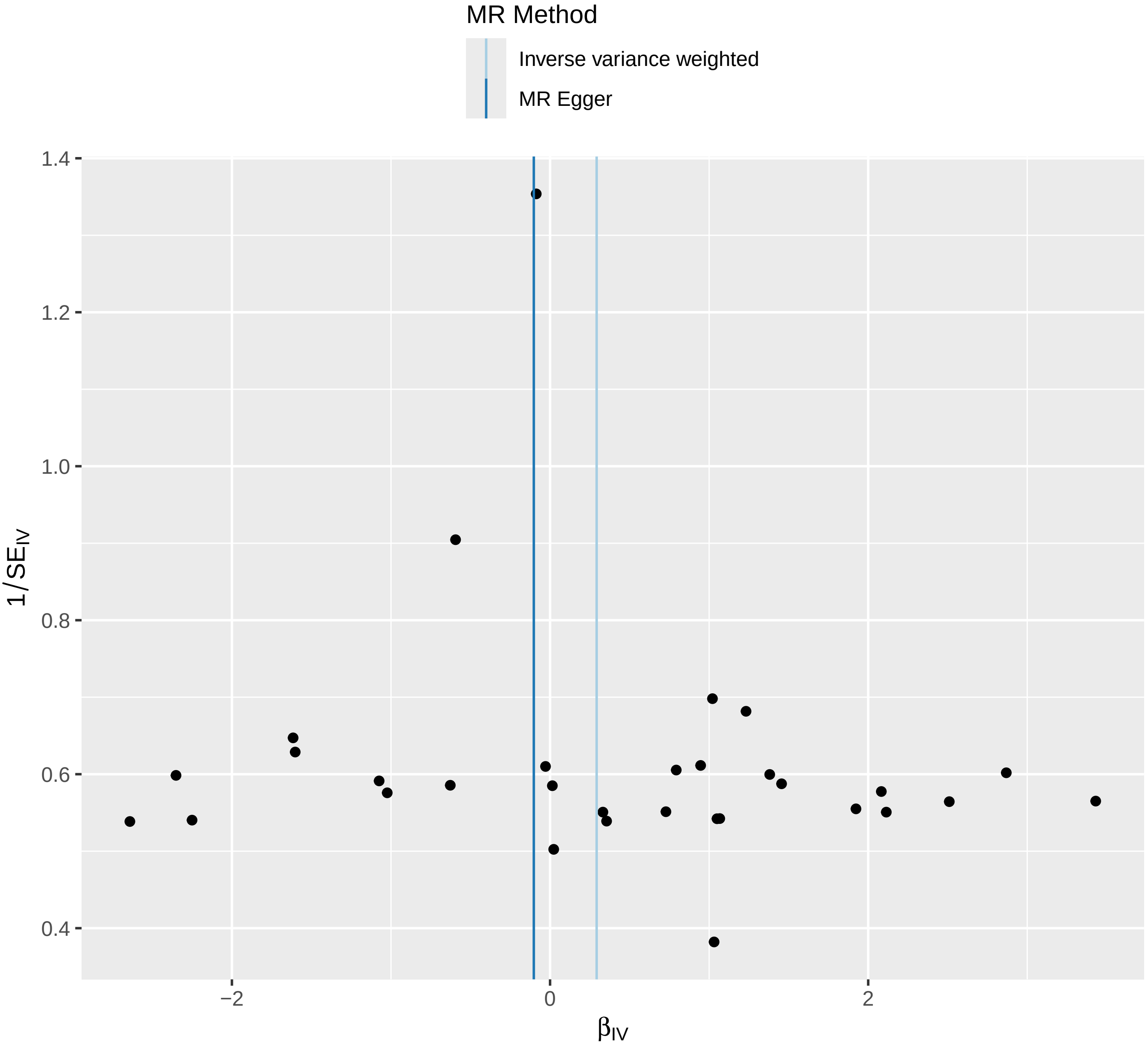
MR Method
Inverse variance weighted
MR Egger
1.4
1.2
1.0
0.8
0.6
0.4
−2
0
2
$1/SE_{IV}$
β_{IV}

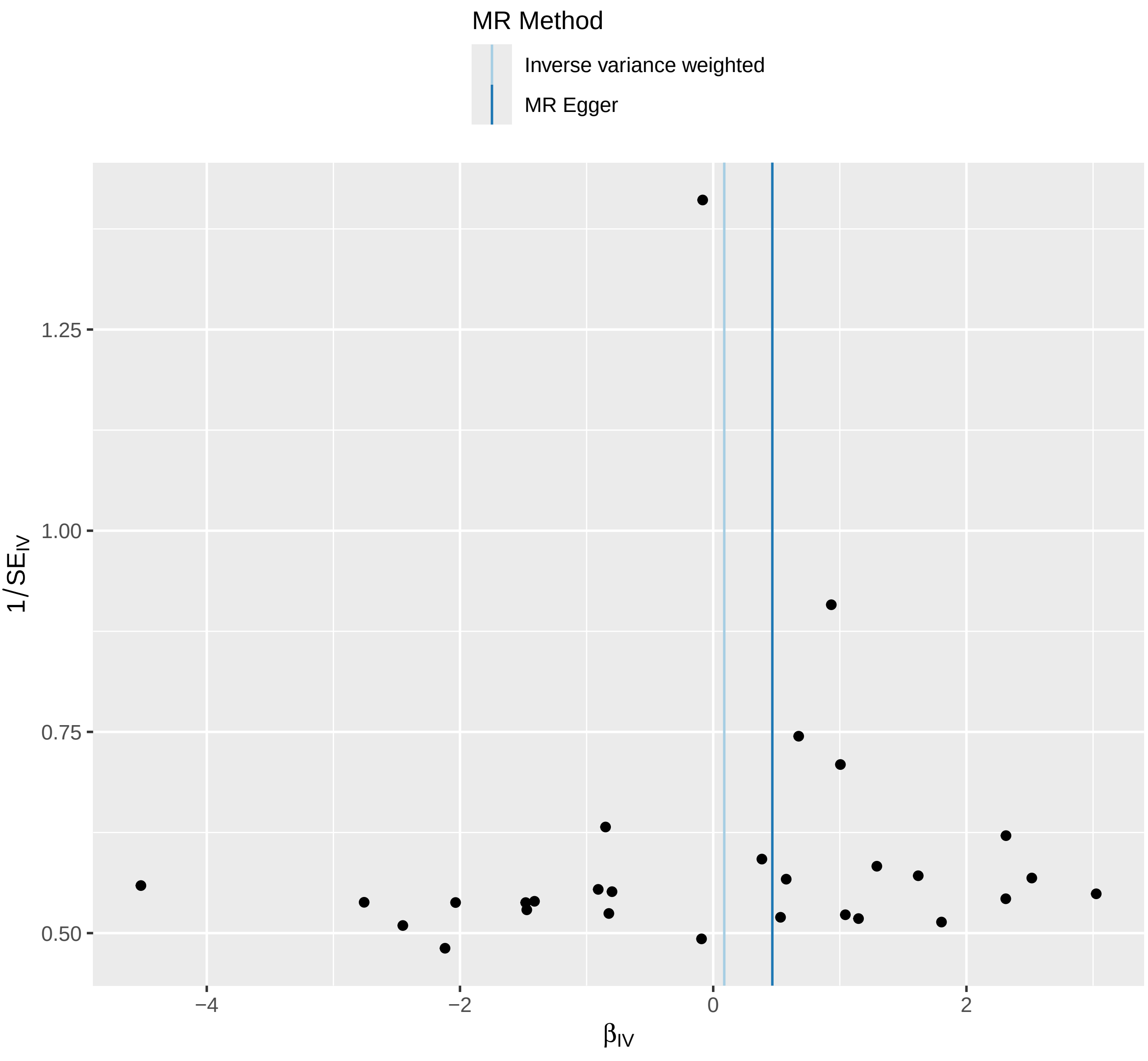
MR Method
Inverse variance weighted
MR Egger
1.25
1.00
0.75
0.50
−4
−2
0
2
$1/SE_{IV}$
β_{IV}

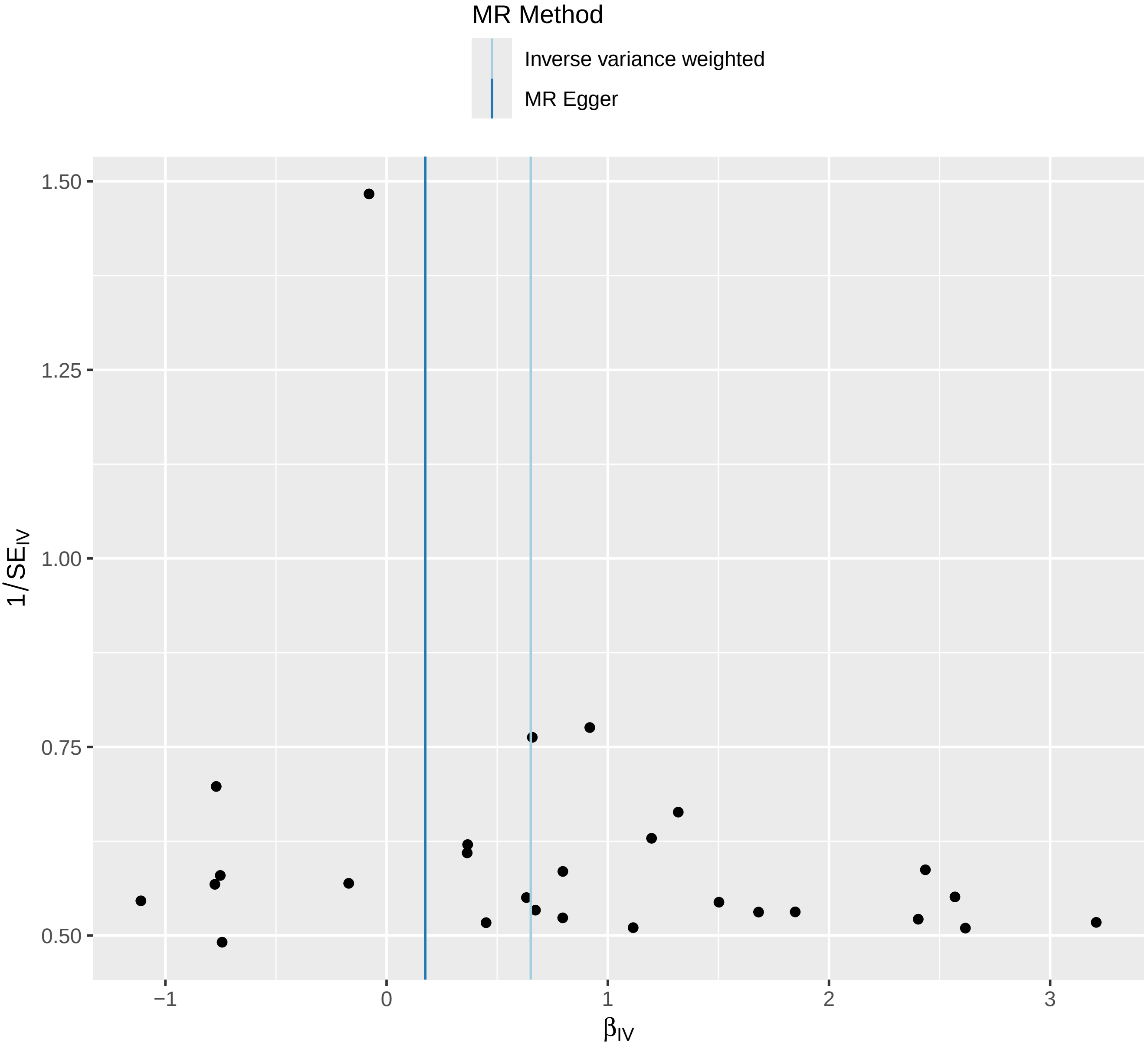
MR Method
Inverse variance weighted
MR Egger
1.50
1.25
1.00
0.75
0.50
1/SE_IV
−1
0
1
2
3
β_IV

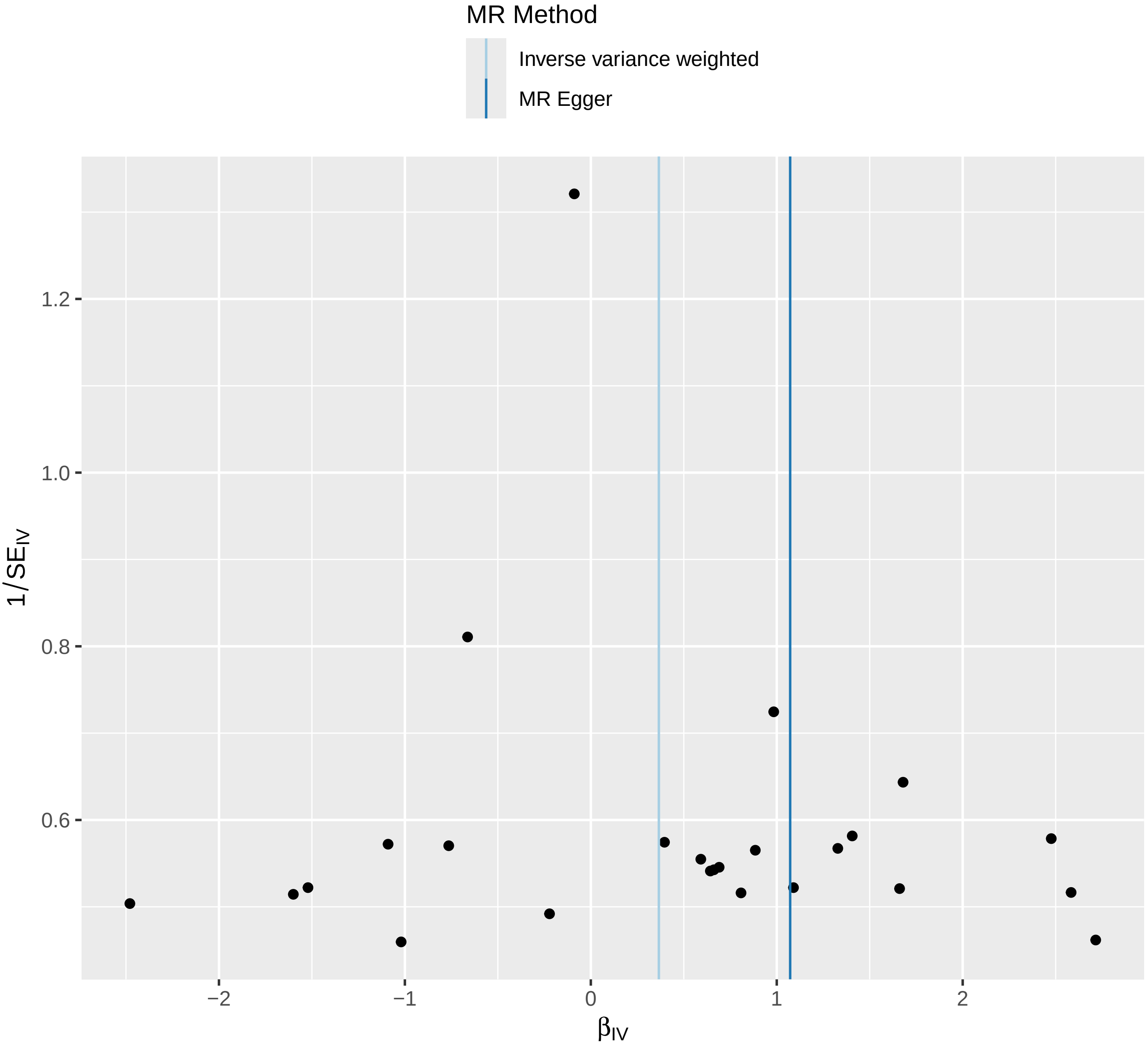
MR Method
Inverse variance weighted
MR Egger
1.2
1.0
0.8
0.6
1/SE_IV
−2
−1
0
1
2
β_IV

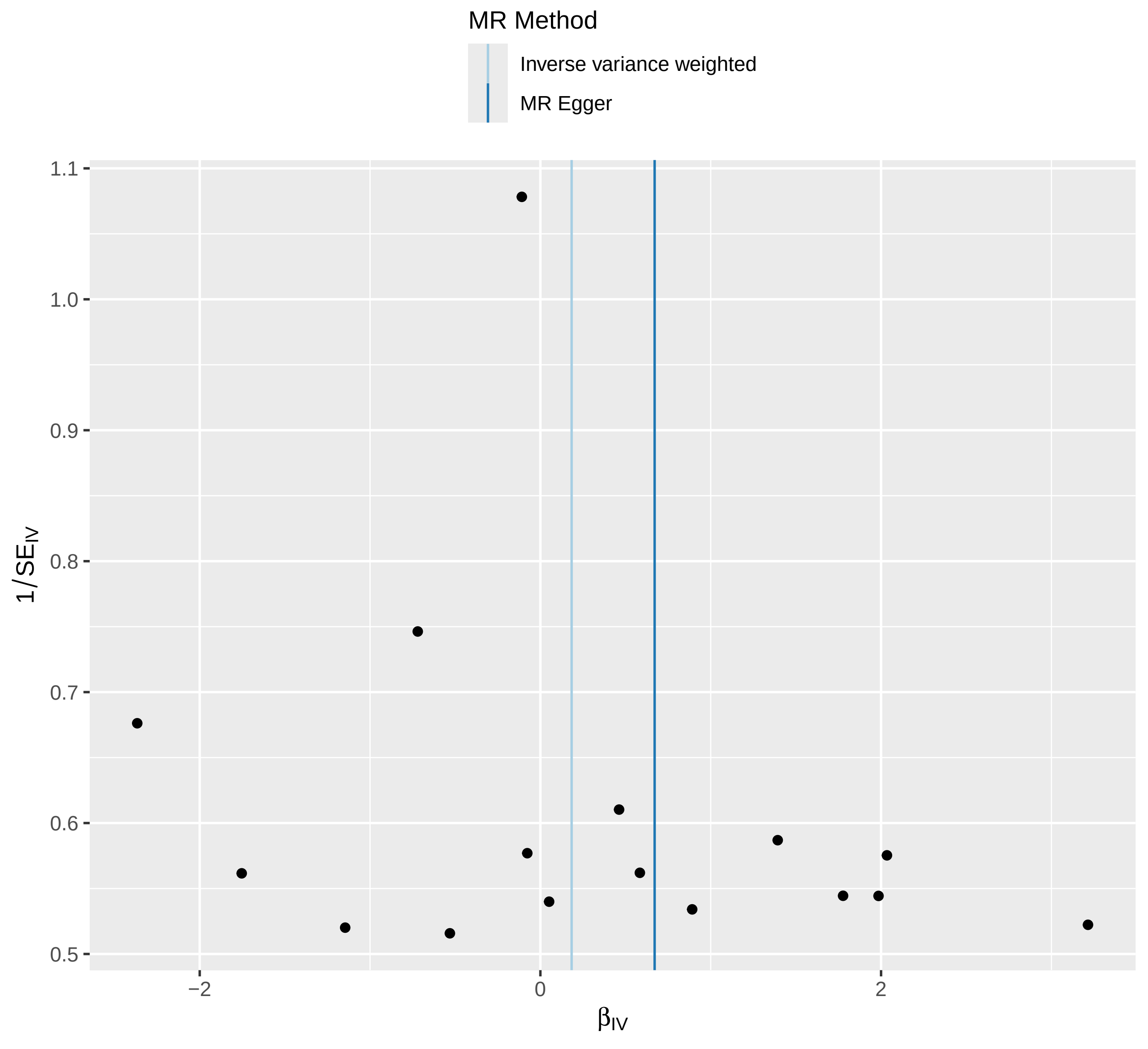
MR Method
Inverse variance weighted
MR Egger
1/SE_IV
1.1
1.0
0.9
0.8
0.7
0.6
0.5
−2
0
2
β_IV

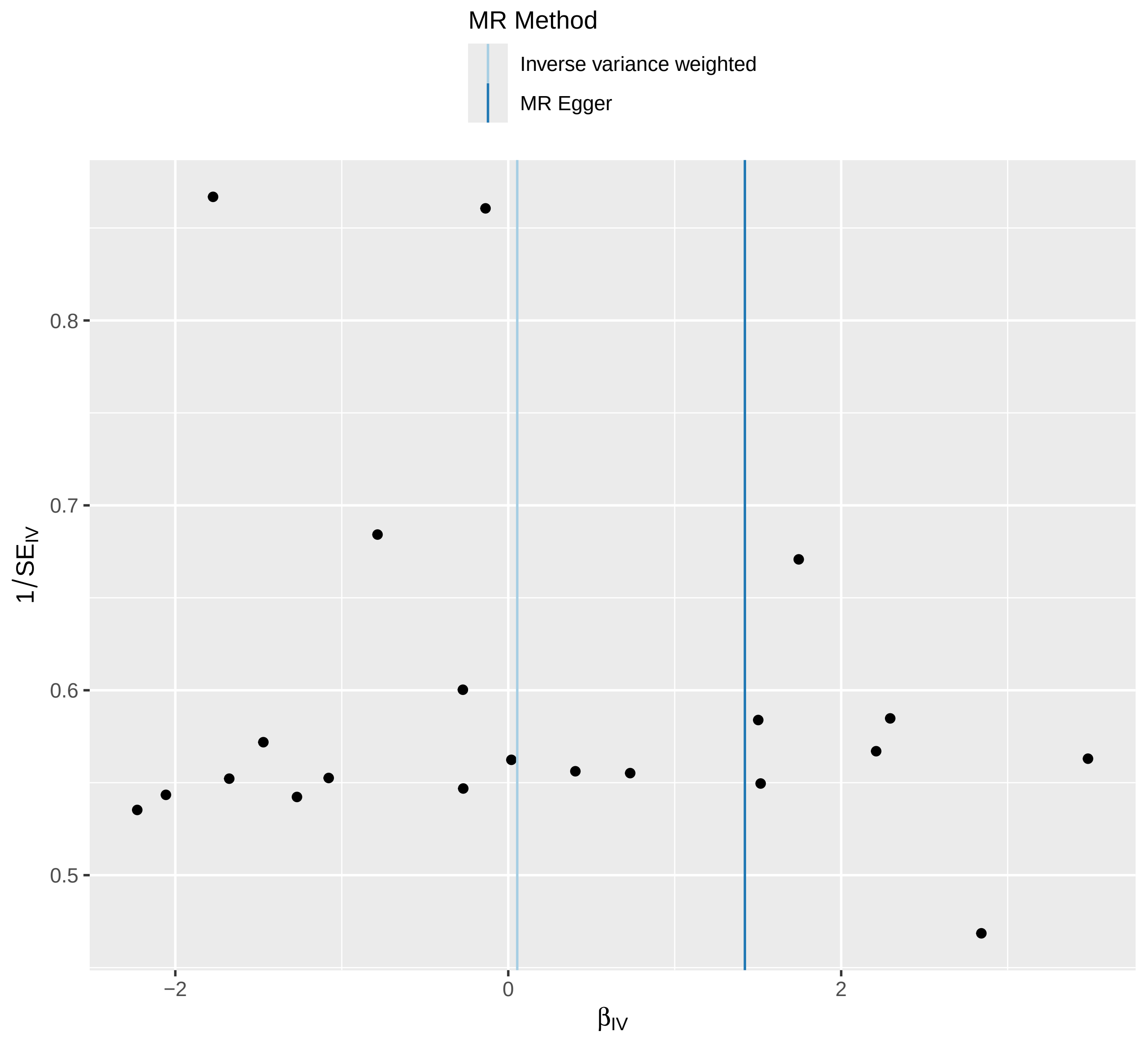

MR Method
Inverse variance weighted
MR Egger
0.8
0.7
0.6
0.5
1/SE_IV
−2
0
2
β_IV

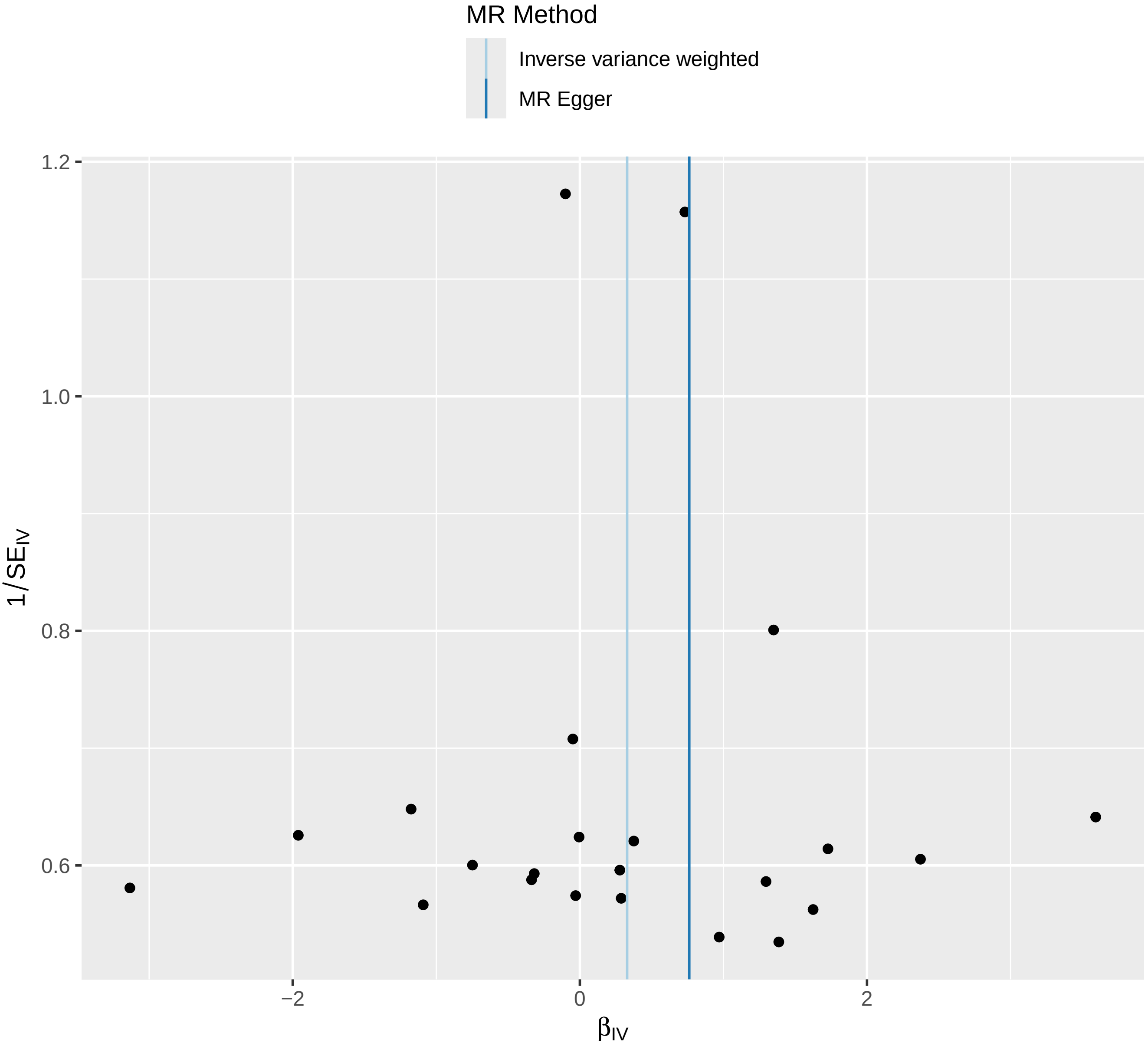

MR Method
Inverse variance weighted
MR Egger
1.2
1.0
0.8
0.6
−2
0
2
1/SE_IV
β_IV

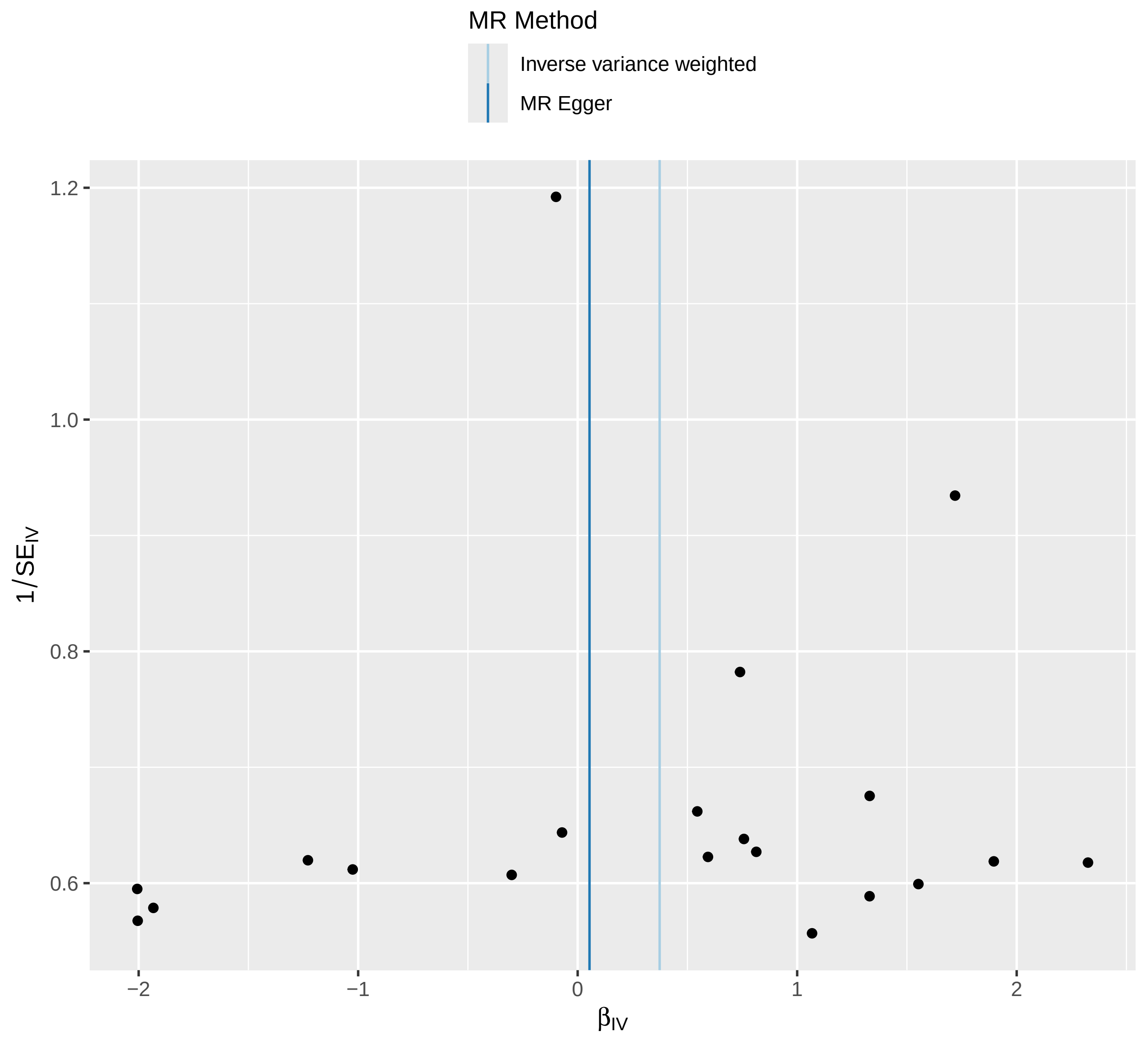
MR Method
Inverse variance weighted
MR Egger
1.2
1.0
0.8
0.6
1/SE_IV
−2
−1
0
1
2
β_IV

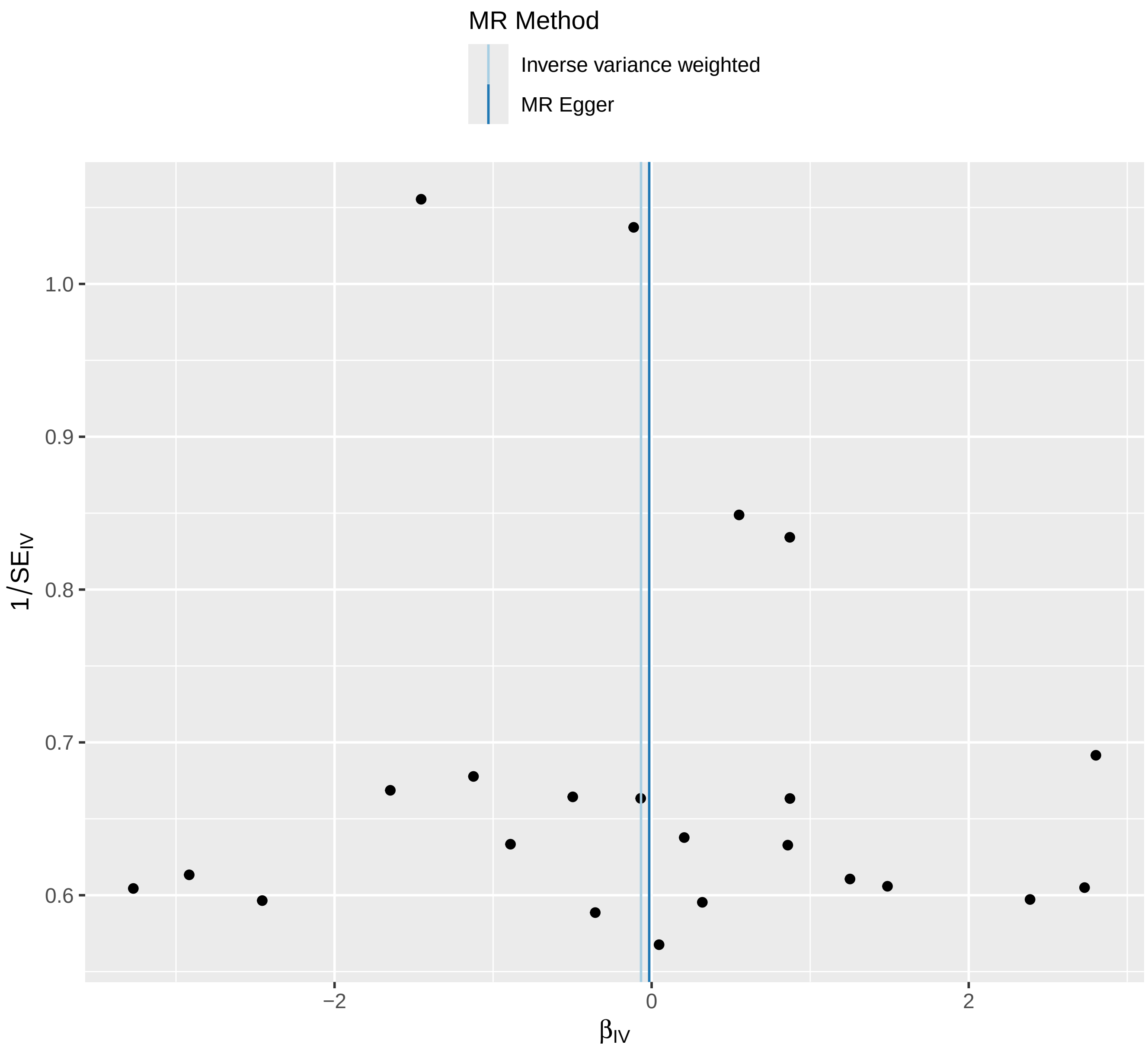
MR Method
Inverse variance weighted
MR Egger
1/SE$_{IV}$
1.0
0.9
0.8
0.7
0.6
−2
0
2
β$_{IV}$

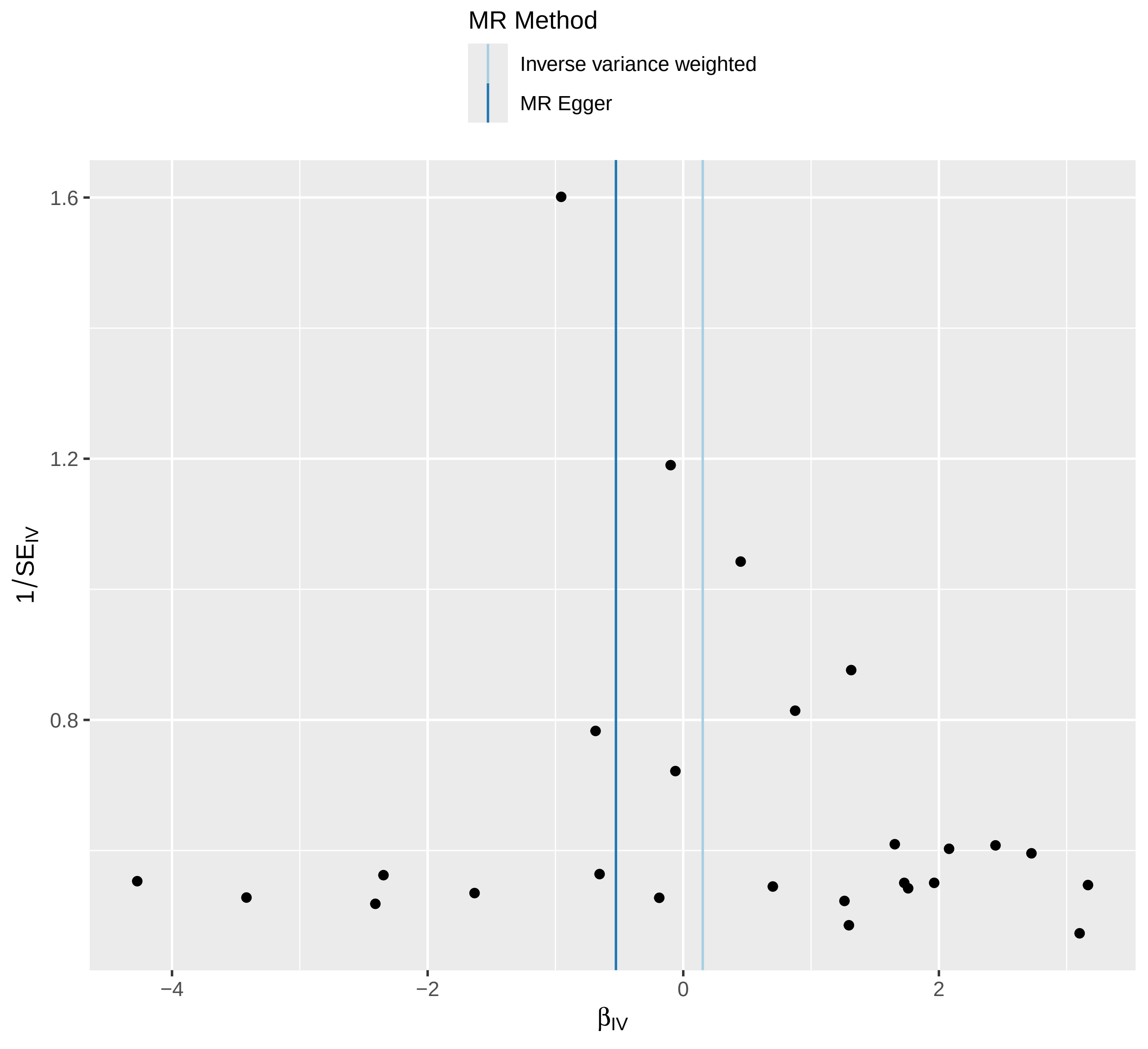
MR Method
Inverse variance weighted
MR Egger
1.6
1.2
0.8
1/SE_IV
−4
−2
0
2
β_IV

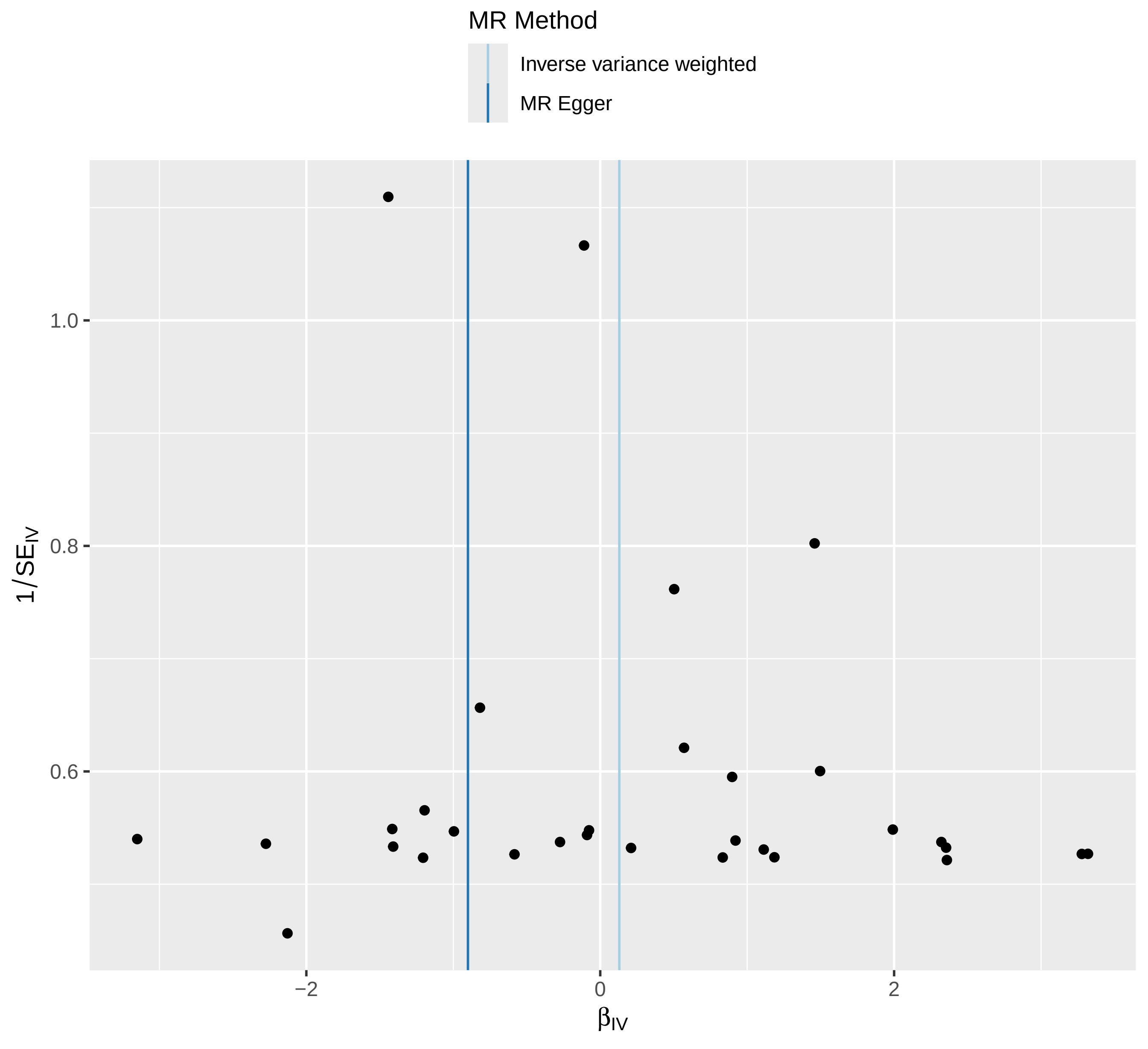

MR Method
Inverse variance weighted
MR Egger
1.0
0.8
0.6
1/SE_IV
−2
0
2
β_IV

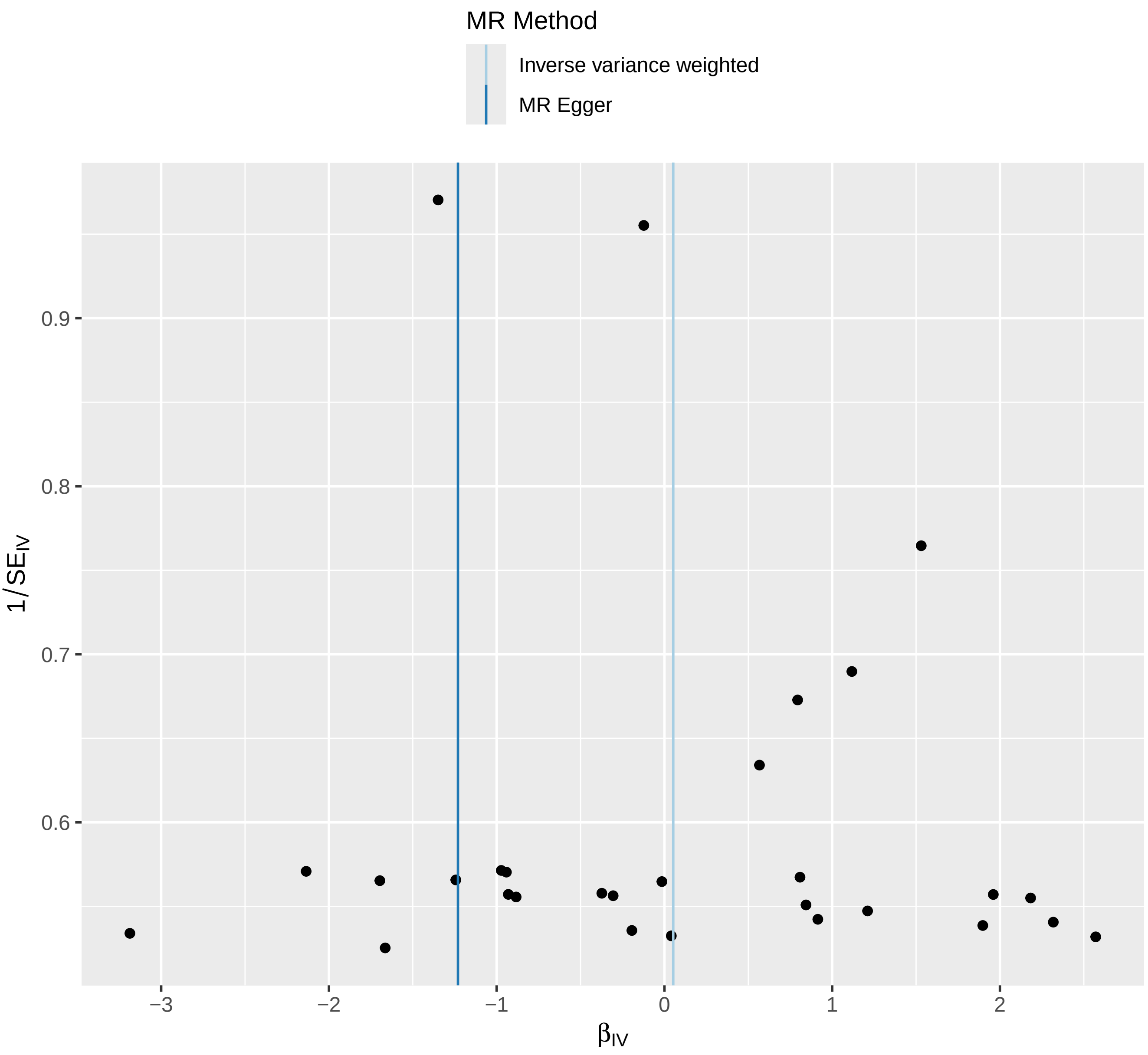
MR Method
Inverse variance weighted
MR Egger
1/SE_IV
0.9
0.8
0.7
0.6
−3
−2
−1
0
1
2
β_IV

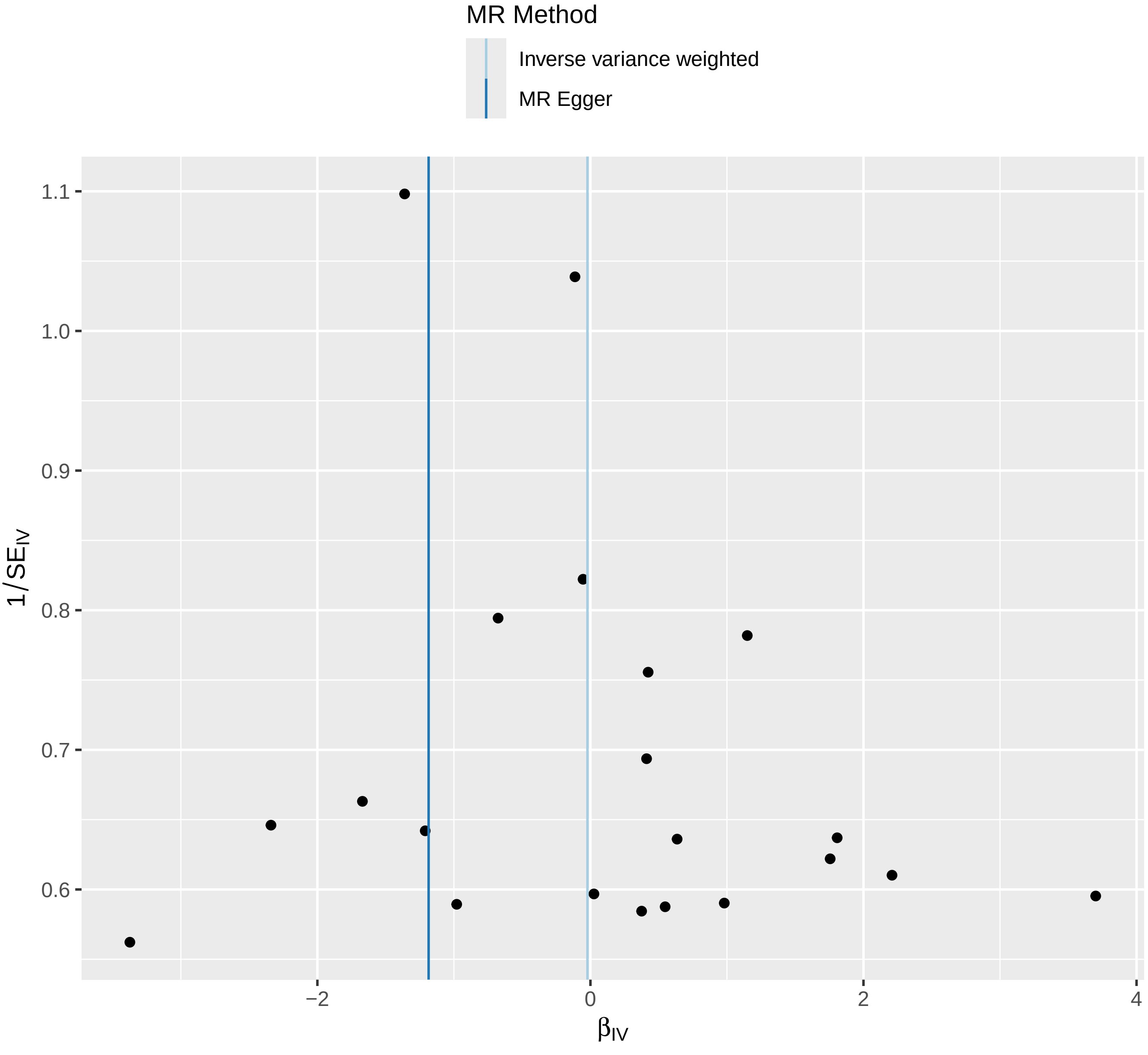
MR Method
Inverse variance weighted
MR Egger
1.1
1.0
0.9
0.8
0.7
0.6
1/SE$_{IV}$
−2
0
2
4
β$_{IV}$

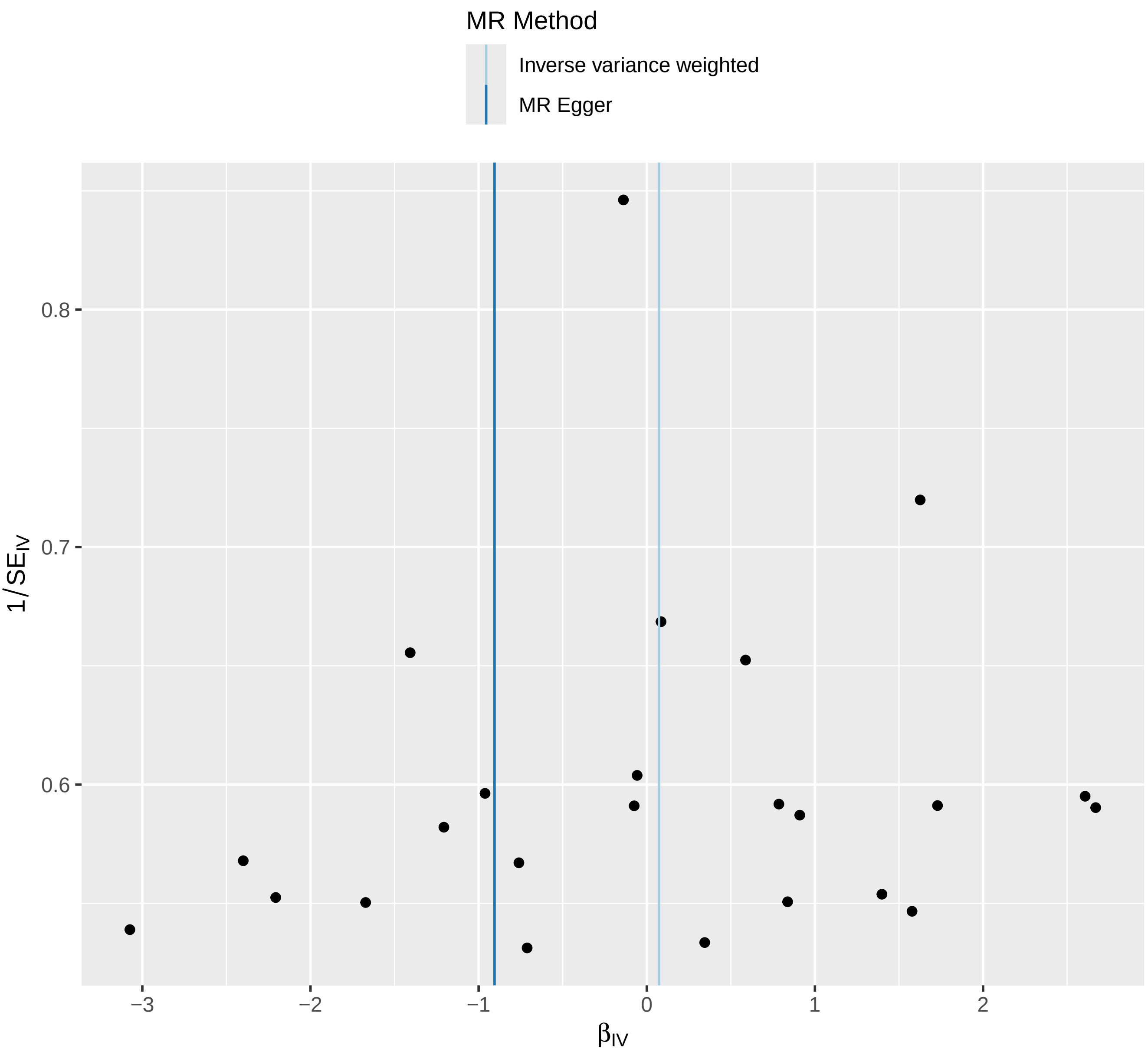

MR Method
Inverse variance weighted
MR Egger
0.8
0.7
0.6
1/SE_IV
−3
−2
−1
0
1
2
β_IV
