# Supplementary material for: Genetically predicted metabolites mediate the association between lipidome and malignant melanoma of skin
Source: Front Oncol. 2024 Sep 10;14:1430533. doi: 10.3389/fonc.2024.1430533 (PMC11419955; doi:10.3389/fonc.2024.1430533)
Supplement: Supplementary file 12 [file SupplementaryFile11.pdf]

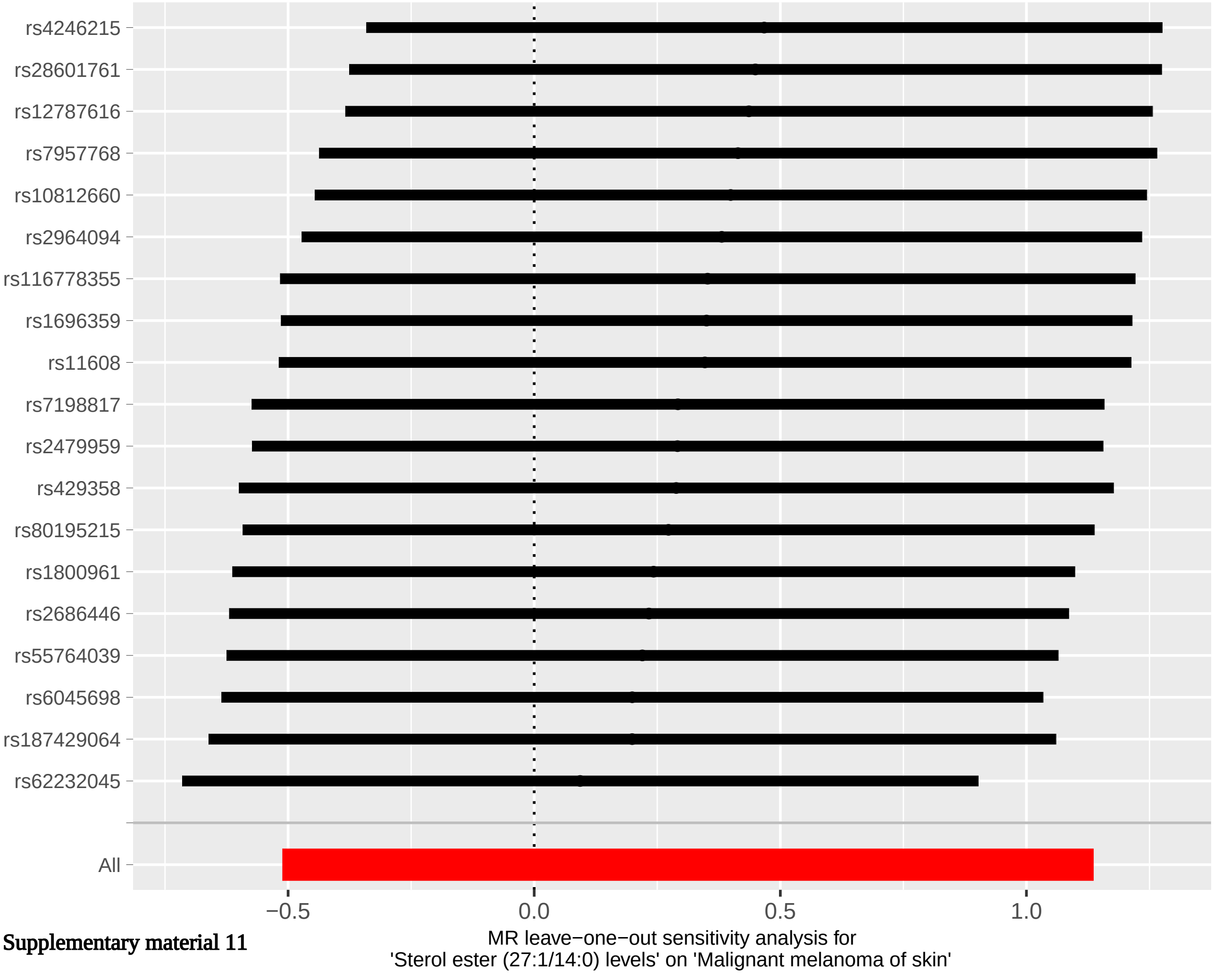

Supplementary material 11

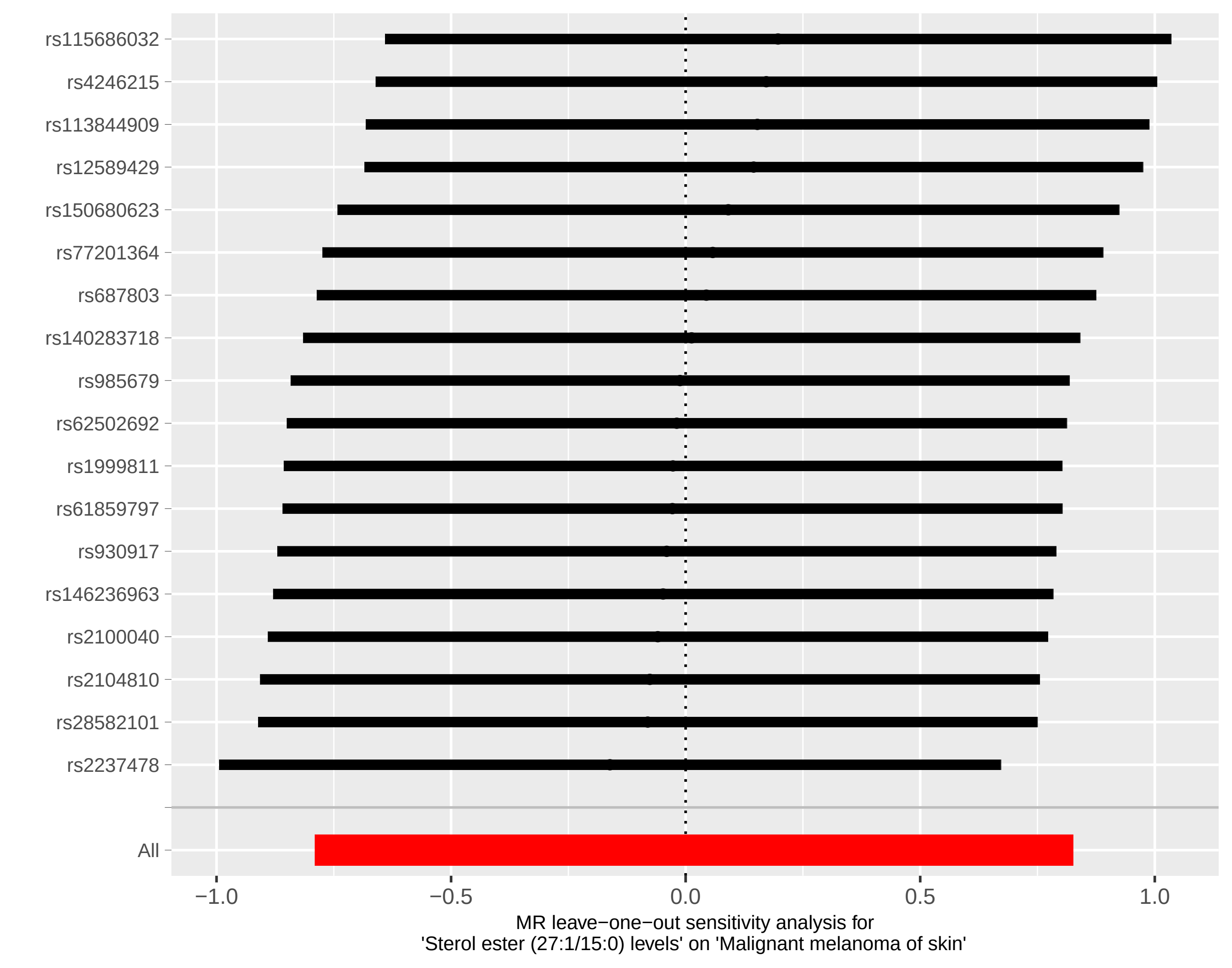
rs115686032
rs4246215
rs113844909
rs12589429
rs150680623
rs77201364
rs687803
rs140283718
rs985679
rs62502692
rs1999811
rs61859797
rs930917
rs146236963
rs2100040
rs2104810
rs28582101
rs2237478
All
−1.0
−0.5
0.0
0.5
1.0
MR leave−one−out sensitivity analysis for
'Sterol ester (27:1/15:0) levels' on 'Malignant melanoma of skin'

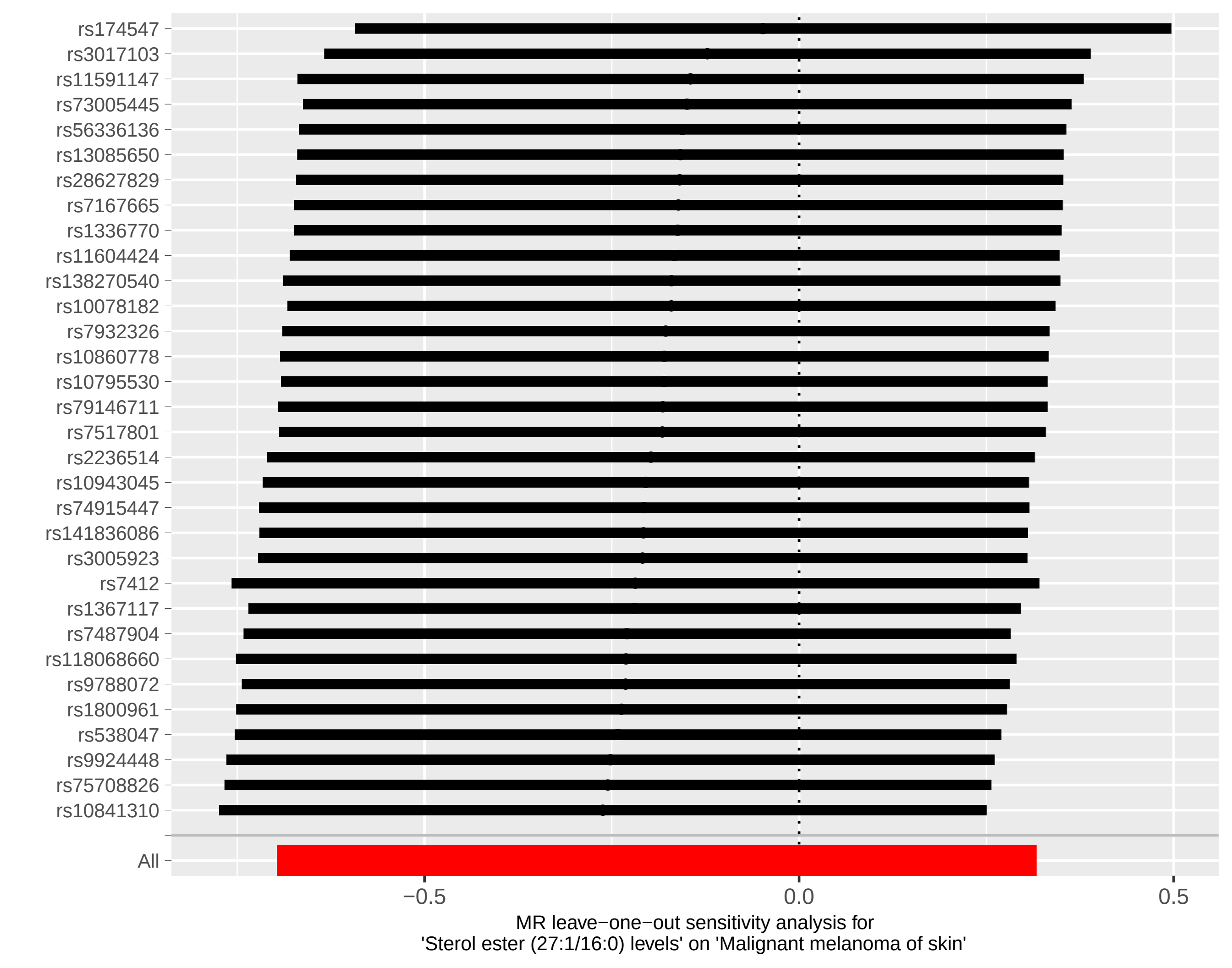
rs174547
rs3017103
rs11591147
rs73005445
rs56336136
rs13085650
rs28627829
rs7167665
rs1336770
rs11604424
rs138270540
rs10078182
rs7932326
rs10860778
rs10795530
rs79146711
rs7517801
rs2236514
rs10943045
rs74915447
rs141836086
rs3005923
rs7412
rs1367117
rs7487904
rs118068660
rs9788072
rs1800961
rs538047
rs9924448
rs75708826
rs10841310
All
−0.5
0.0
0.5
MR leave−one−out sensitivity analysis for
'Sterol ester (27:1/16:0) levels' on 'Malignant melanoma of skin'

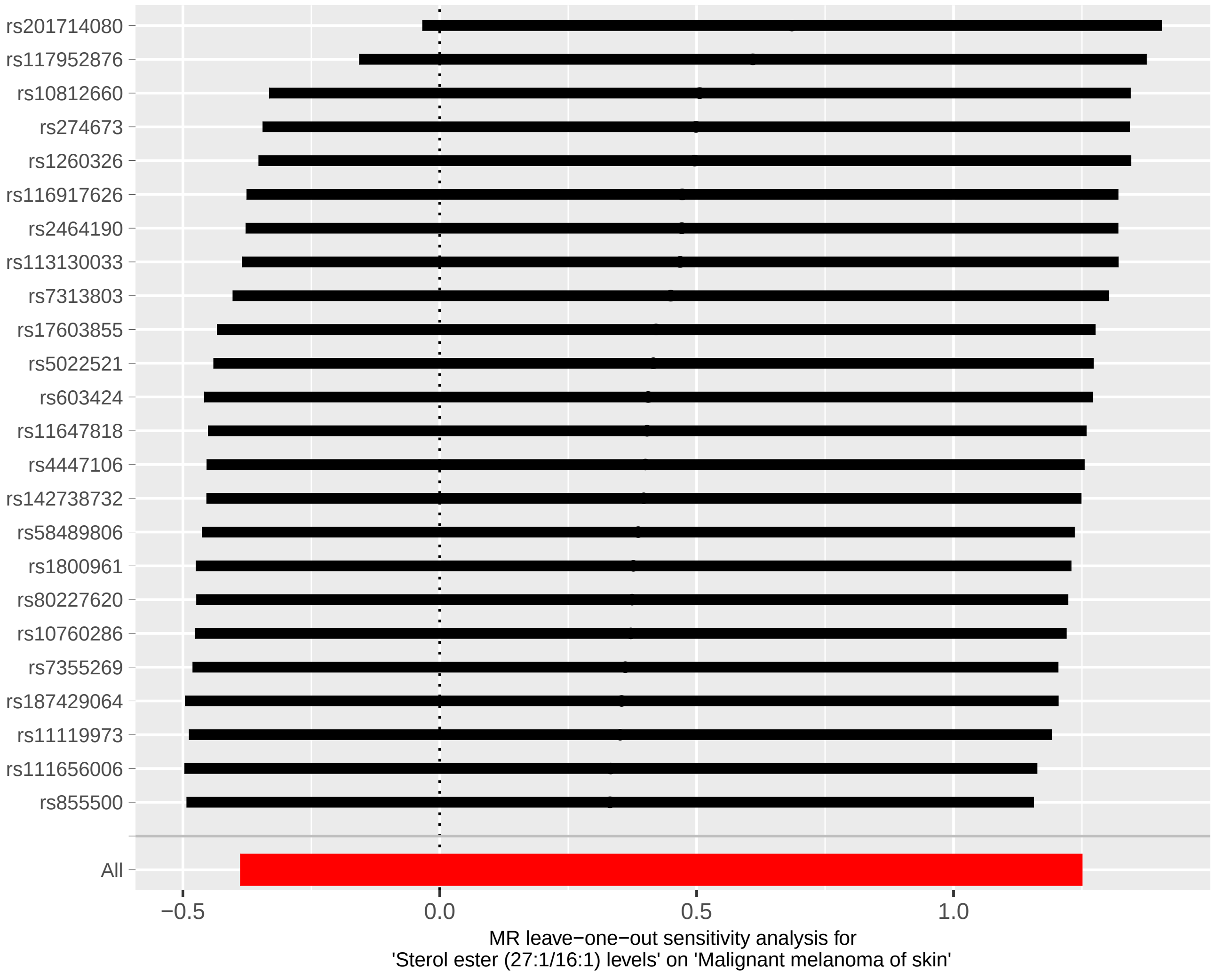
rs201714080
rs117952876
rs10812660
rs274673
rs1260326
rs116917626
rs2464190
rs113130033
rs7313803
rs17603855
rs5022521
rs603424
rs11647818
rs4447106
rs142738732
rs58489806
rs1800961
rs80227620
rs10760286
rs7355269
rs187429064
rs11119973
rs111656006
rs855500
All
−0.5
0.0
0.5
1.0
MR leave−one−out sensitivity analysis for
'Sterol ester (27:1/16:1) levels' on 'Malignant melanoma of skin'

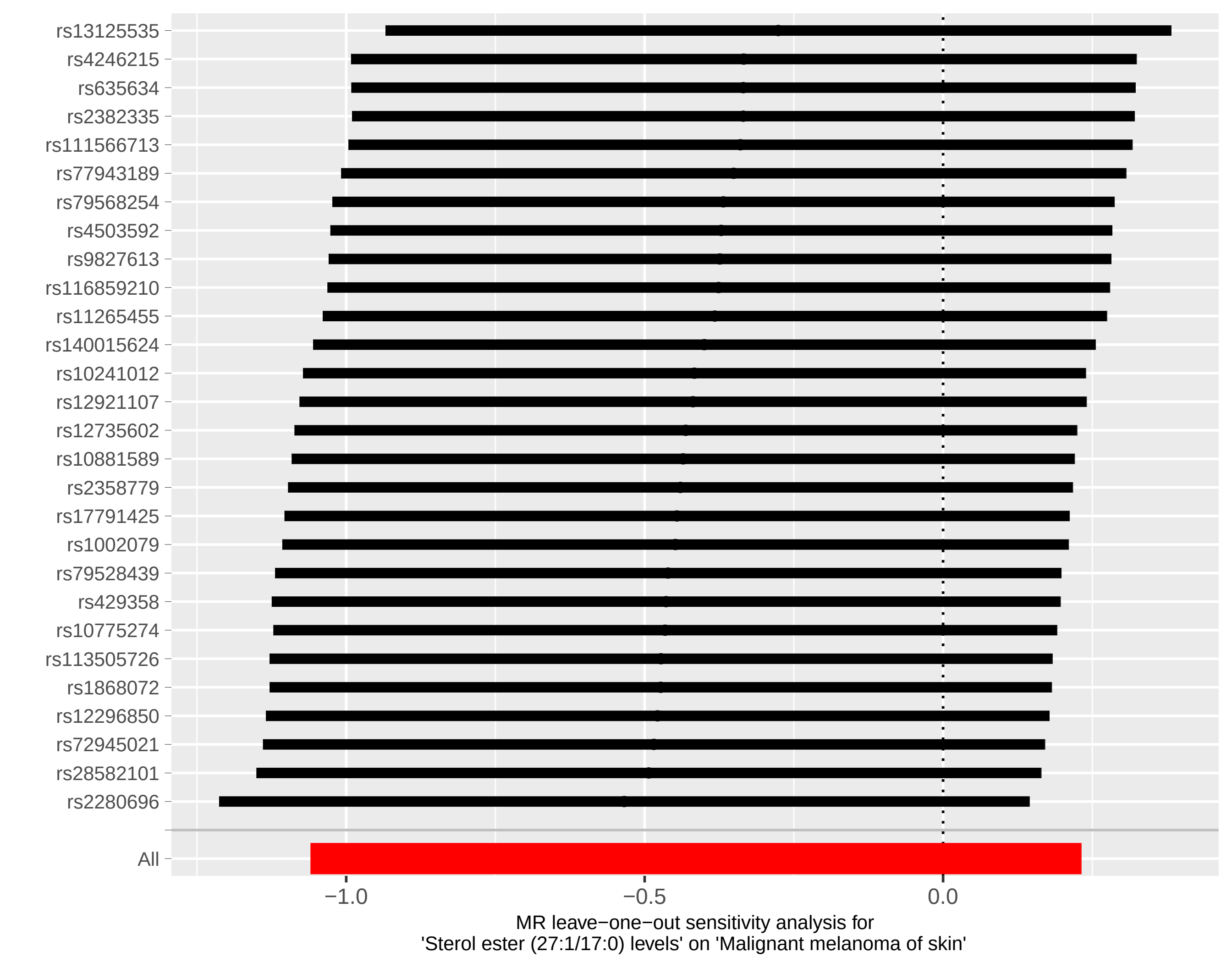
rs13125535
rs4246215
rs635634
rs2382335
rs111566713
rs77943189
rs79568254
rs4503592
rs9827613
rs116859210
rs11265455
rs140015624
rs10241012
rs12921107
rs12735602
rs10881589
rs2358779
rs17791425
rs1002079
rs79528439
rs429358
rs10775274
rs113505726
rs1868072
rs12296850
rs72945021
rs28582101
rs2280696
All
−1.0
−0.5
0.0
MR leave−one−out sensitivity analysis for
'Sterol ester (27:1/17:0) levels' on 'Malignant melanoma of skin'

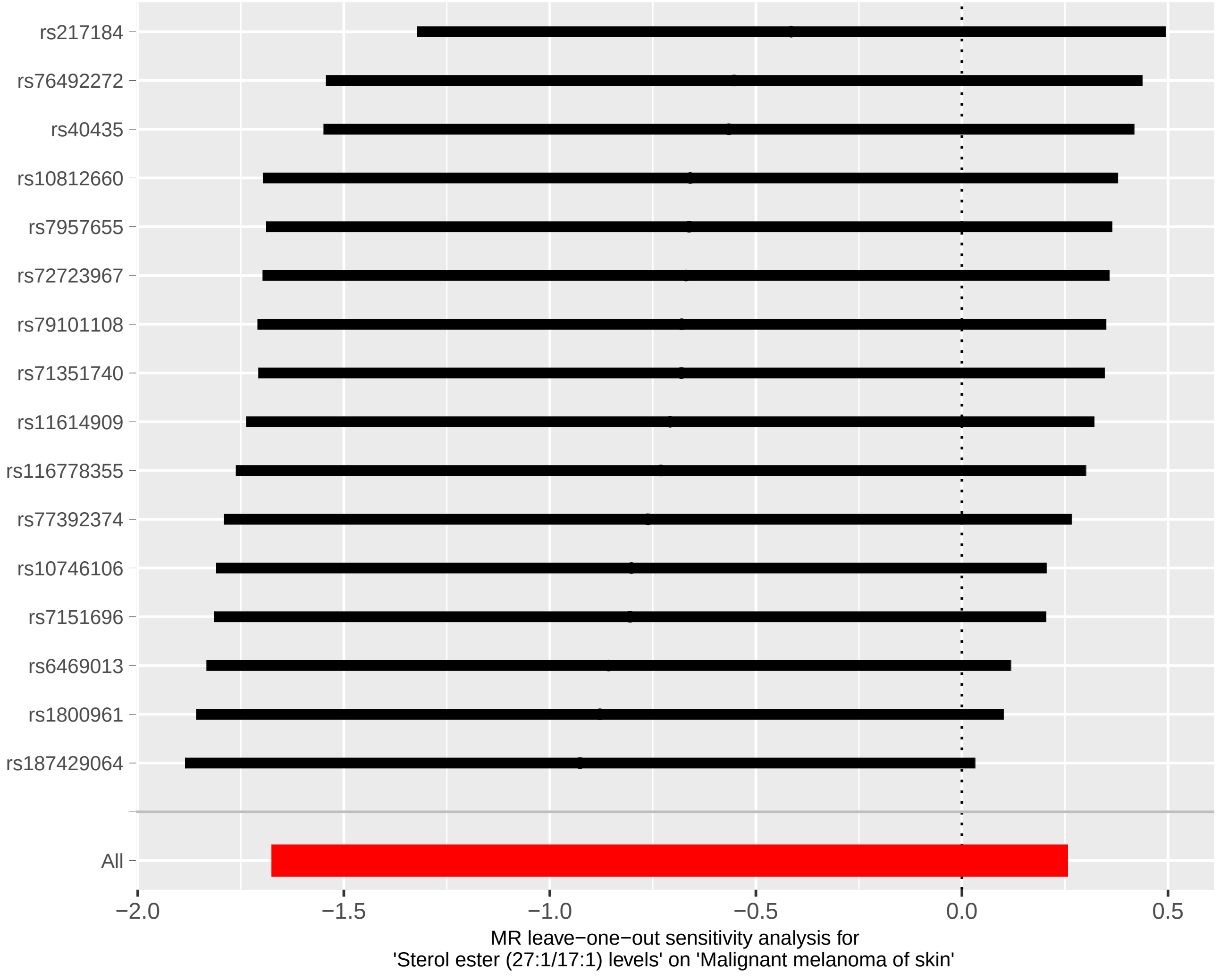
rs217184
rs76492272
rs40435
rs10812660
rs7957655
rs72723967
rs79101108
rs71351740
rs11614909
rs116778355
rs77392374
rs10746106
rs7151696
rs6469013
rs1800961
rs187429064
All
−2.0
−1.5
−1.0
−0.5
0.0
0.5
MR leave−one−out sensitivity analysis for
'Sterol ester (27:1/17:1) levels' on 'Malignant melanoma of skin'

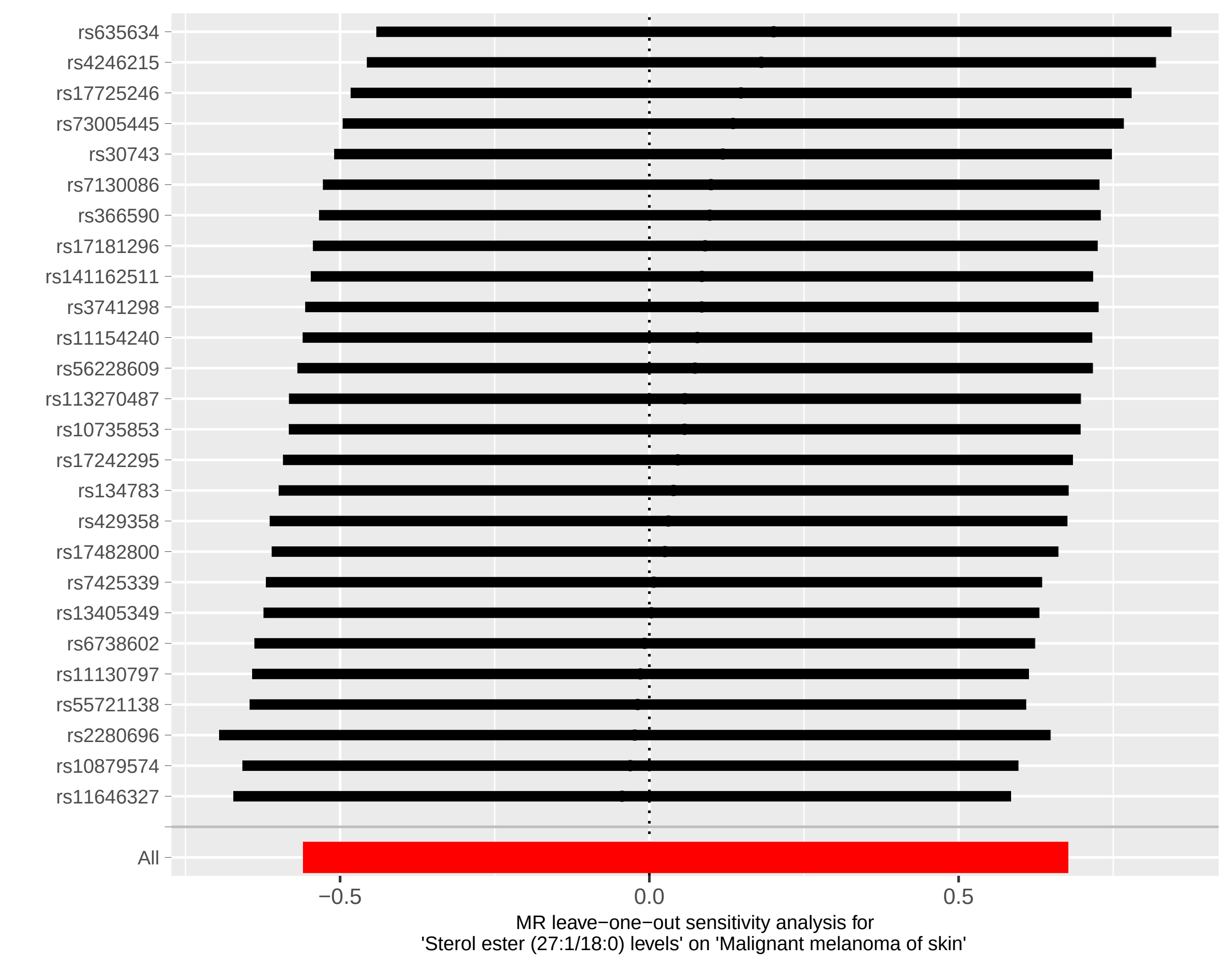
rs635634
rs4246215
rs17725246
rs73005445
rs30743
rs7130086
rs366590
rs17181296
rs141162511
rs3741298
rs11154240
rs56228609
rs113270487
rs10735853
rs17242295
rs134783
rs429358
rs17482800
rs7425339
rs13405349
rs6738602
rs11130797
rs55721138
rs2280696
rs10879574
rs11646327
All
−0.5
0.0
0.5
MR leave−one−out sensitivity analysis for
'Sterol ester (27:1/18:0) levels' on 'Malignant melanoma of skin'

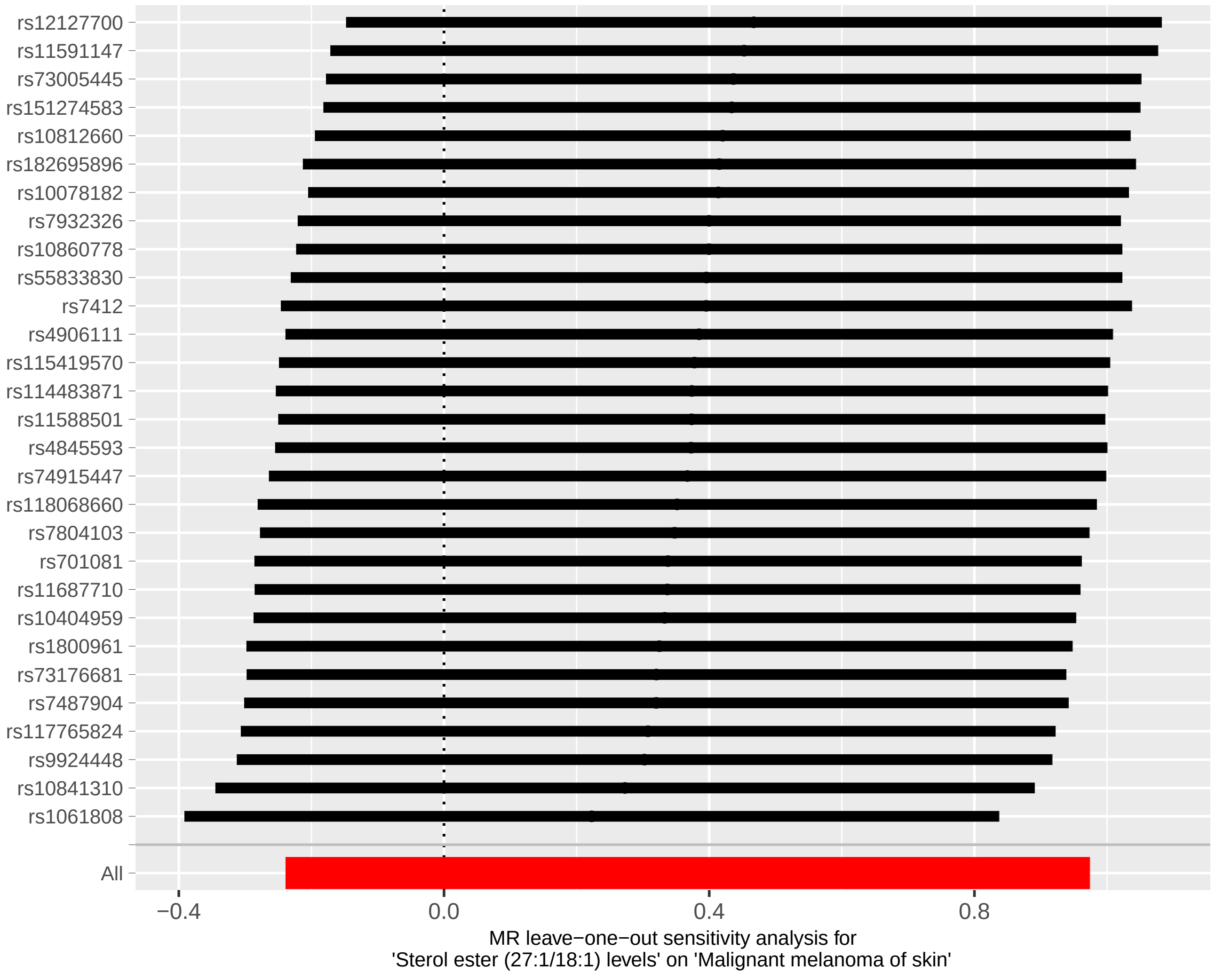
rs12127700
rs11591147
rs73005445
rs151274583
rs10812660
rs182695896
rs10078182
rs7932326
rs10860778
rs55833830
rs7412
rs4906111
rs115419570
rs114483871
rs11588501
rs4845593
rs74915447
rs118068660
rs7804103
rs701081
rs11687710
rs10404959
rs1800961
rs73176681
rs7487904
rs117765824
rs9924448
rs10841310
rs1061808
All
−0.4
0.0
0.4
0.8
MR leave−one−out sensitivity analysis for
'Sterol ester (27:1/18:1) levels' on 'Malignant melanoma of skin'

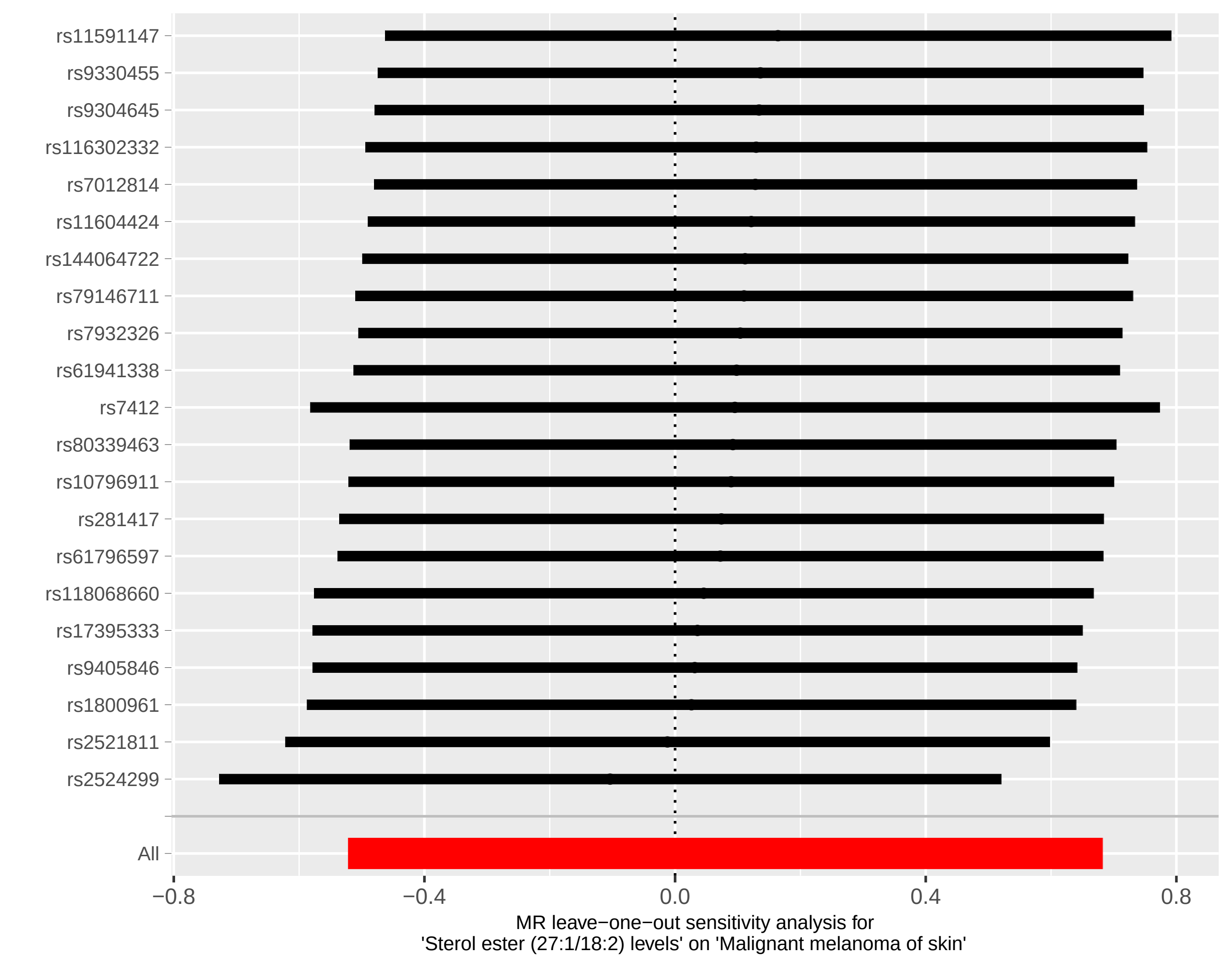
rs11591147
rs9330455
rs9304645
rs116302332
rs7012814
rs11604424
rs144064722
rs79146711
rs7932326
rs61941338
rs7412
rs80339463
rs10796911
rs281417
rs61796597
rs118068660
rs17395333
rs9405846
rs1800961
rs2521811
rs2524299
All
−0.8
−0.4
0.0
0.4
0.8
MR leave−one−out sensitivity analysis for
'Sterol ester (27:1/18:2) levels' on 'Malignant melanoma of skin'

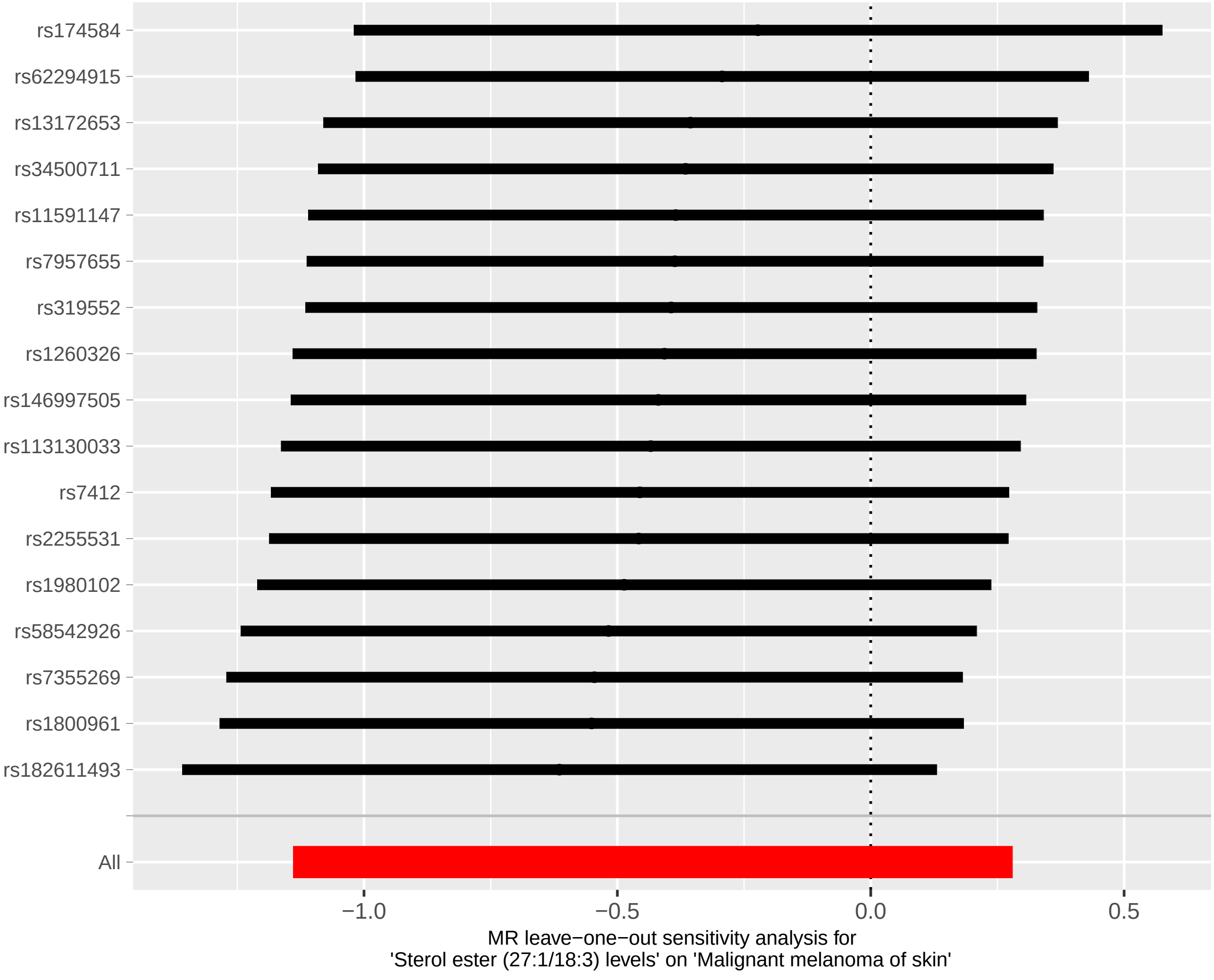

rs174584
rs62294915
rs13172653
rs34500711
rs11591147
rs7957655
rs319552
rs1260326
rs146997505
rs113130033
rs7412
rs2255531
rs1980102
rs58542926
rs7355269
rs1800961
rs182611493
All
−1.0
−0.5
0.0
0.5
MR leave−one−out sensitivity analysis for
'Sterol ester (27:1/18:3) levels' on 'Malignant melanoma of skin'

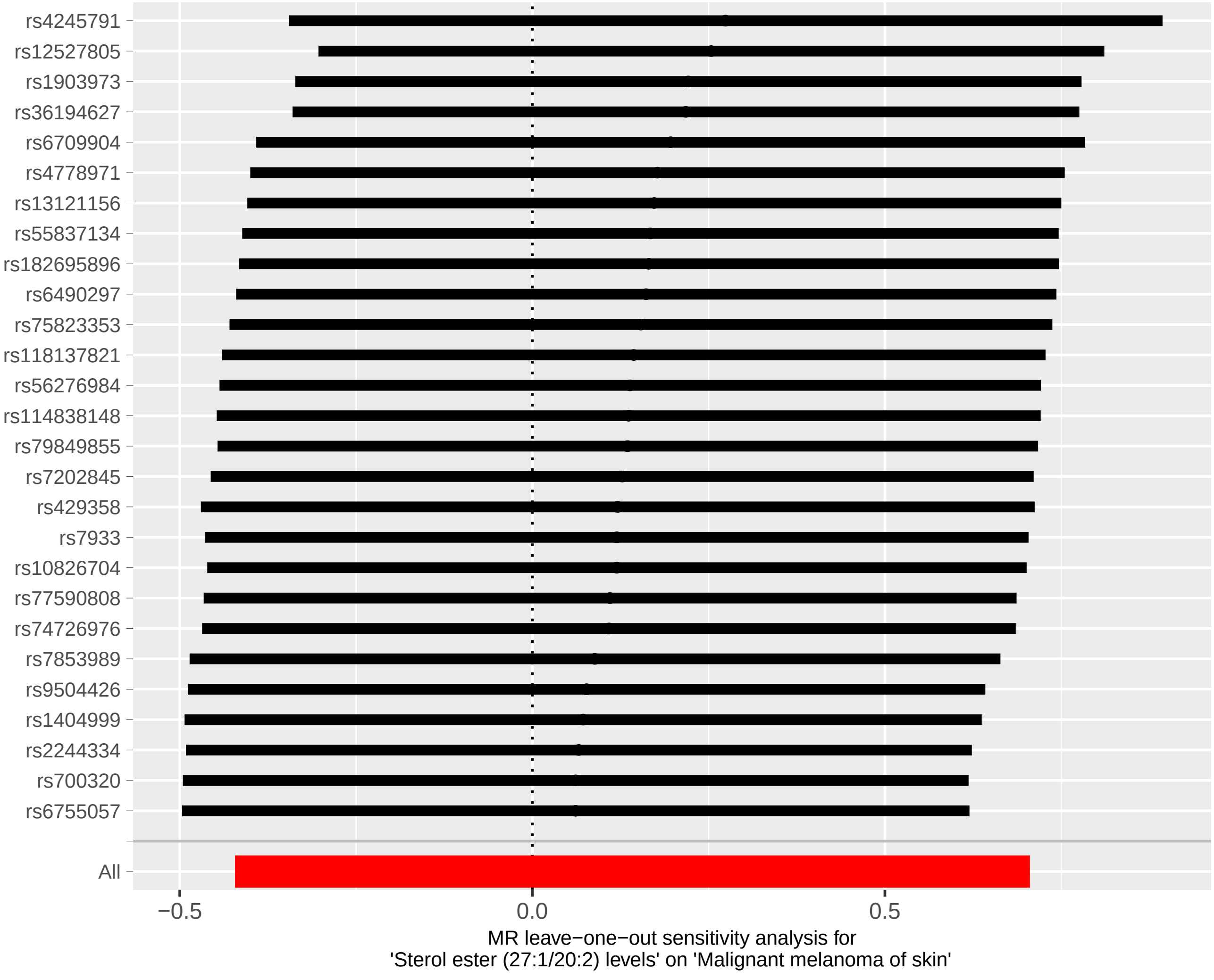
rs4245791
rs12527805
rs1903973
rs36194627
rs6709904
rs4778971
rs13121156
rs55837134
rs182695896
rs6490297
rs75823353
rs118137821
rs56276984
rs114838148
rs79849855
rs7202845
rs429358
rs7933
rs10826704
rs77590808
rs74726976
rs7853989
rs9504426
rs1404999
rs2244334
rs700320
rs6755057
All
−0.5
0.0
0.5
MR leave−one−out sensitivity analysis for
'Sterol ester (27:1/20:2) levels' on 'Malignant melanoma of skin'

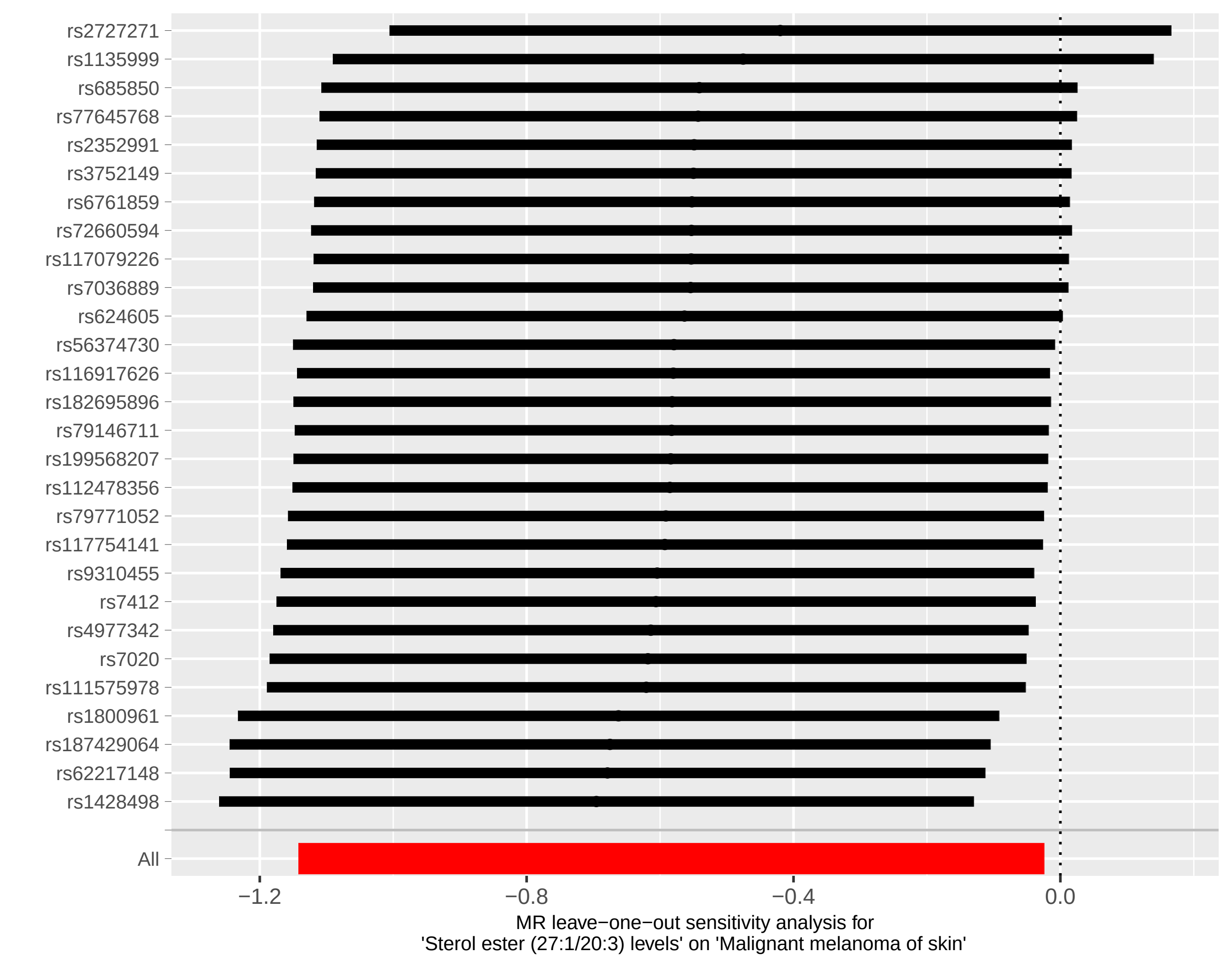
rs2727271
rs1135999
rs685850
rs77645768
rs2352991
rs3752149
rs6761859
rs72660594
rs117079226
rs7036889
rs624605
rs56374730
rs116917626
rs182695896
rs79146711
rs199568207
rs112478356
rs79771052
rs117754141
rs9310455
rs7412
rs4977342
rs7020
rs111575978
rs1800961
rs187429064
rs62217148
rs1428498
All
−1.2
−0.8
−0.4
0.0
MR leave−one−out sensitivity analysis for
'Sterol ester (27:1/20:3) levels' on 'Malignant melanoma of skin'

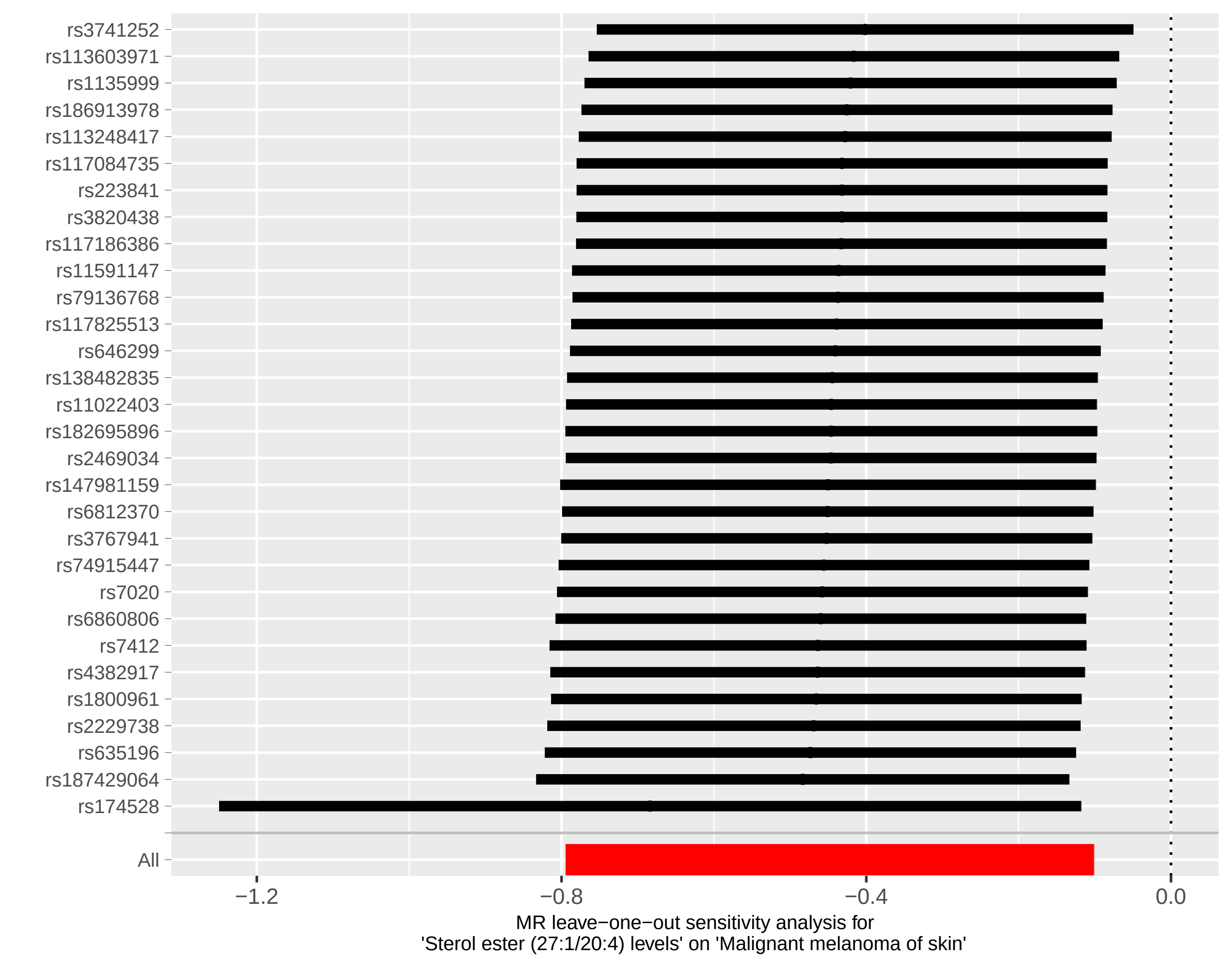
rs3741252
rs113603971
rs1135999
rs186913978
rs113248417
rs117084735
rs223841
rs3820438
rs117186386
rs11591147
rs79136768
rs117825513
rs646299
rs138482835
rs11022403
rs182695896
rs2469034
rs147981159
rs6812370
rs3767941
rs74915447
rs7020
rs6860806
rs7412
rs4382917
rs1800961
rs2229738
rs635196
rs187429064
rs174528
All
−1.2
−0.8
−0.4
0.0
MR leave−one−out sensitivity analysis for
'Sterol ester (27:1/20:4) levels' on 'Malignant melanoma of skin'

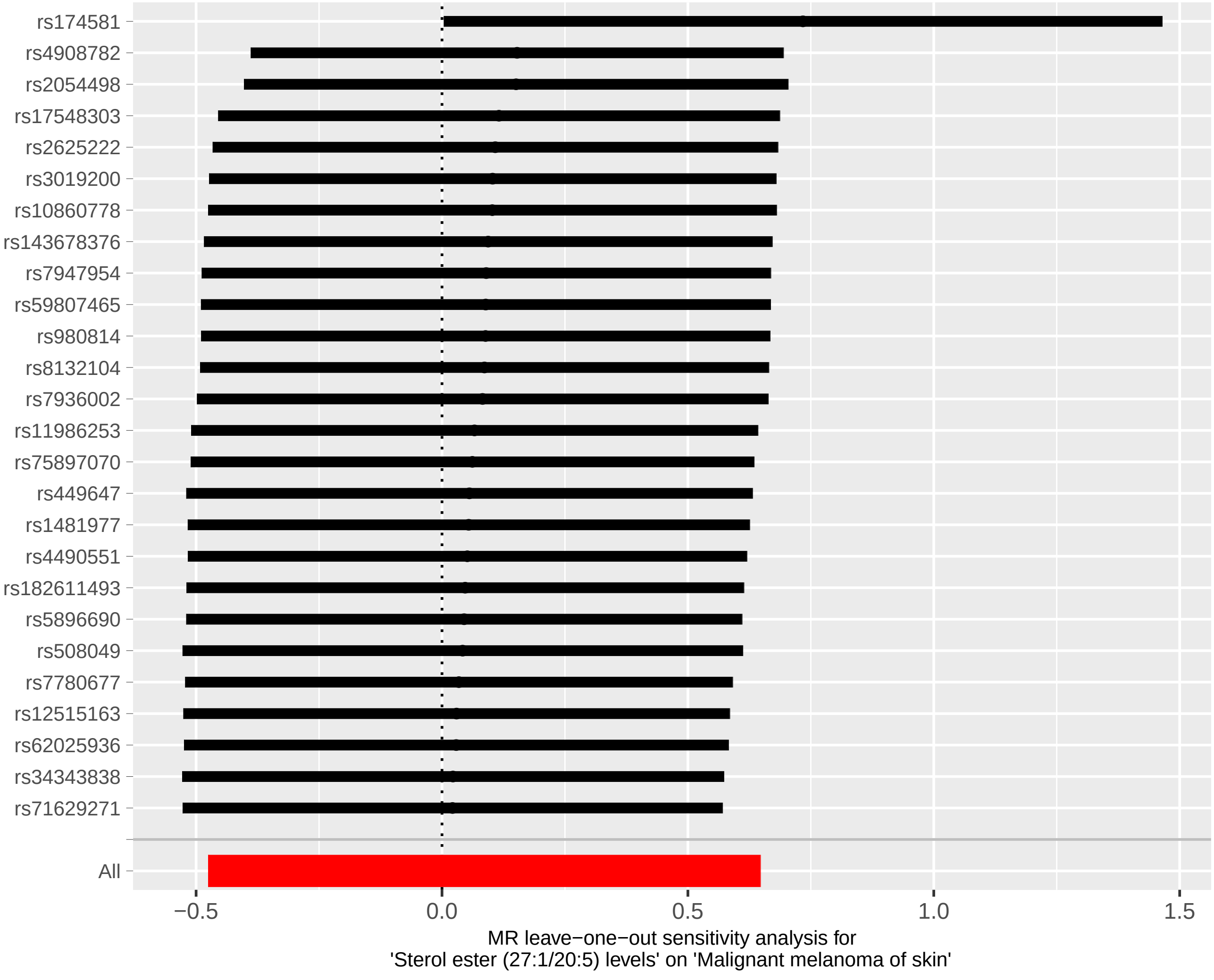
rs174581
rs4908782
rs2054498
rs17548303
rs2625222
rs3019200
rs10860778
rs143678376
rs7947954
rs59807465
rs980814
rs8132104
rs7936002
rs11986253
rs75897070
rs449647
rs1481977
rs4490551
rs182611493
rs5896690
rs508049
rs7780677
rs12515163
rs62025936
rs34343838
rs71629271
All
−0.5
0.0
0.5
1.0
1.5
MR leave−one−out sensitivity analysis for
'Sterol ester (27:1/20:5) levels' on 'Malignant melanoma of skin'

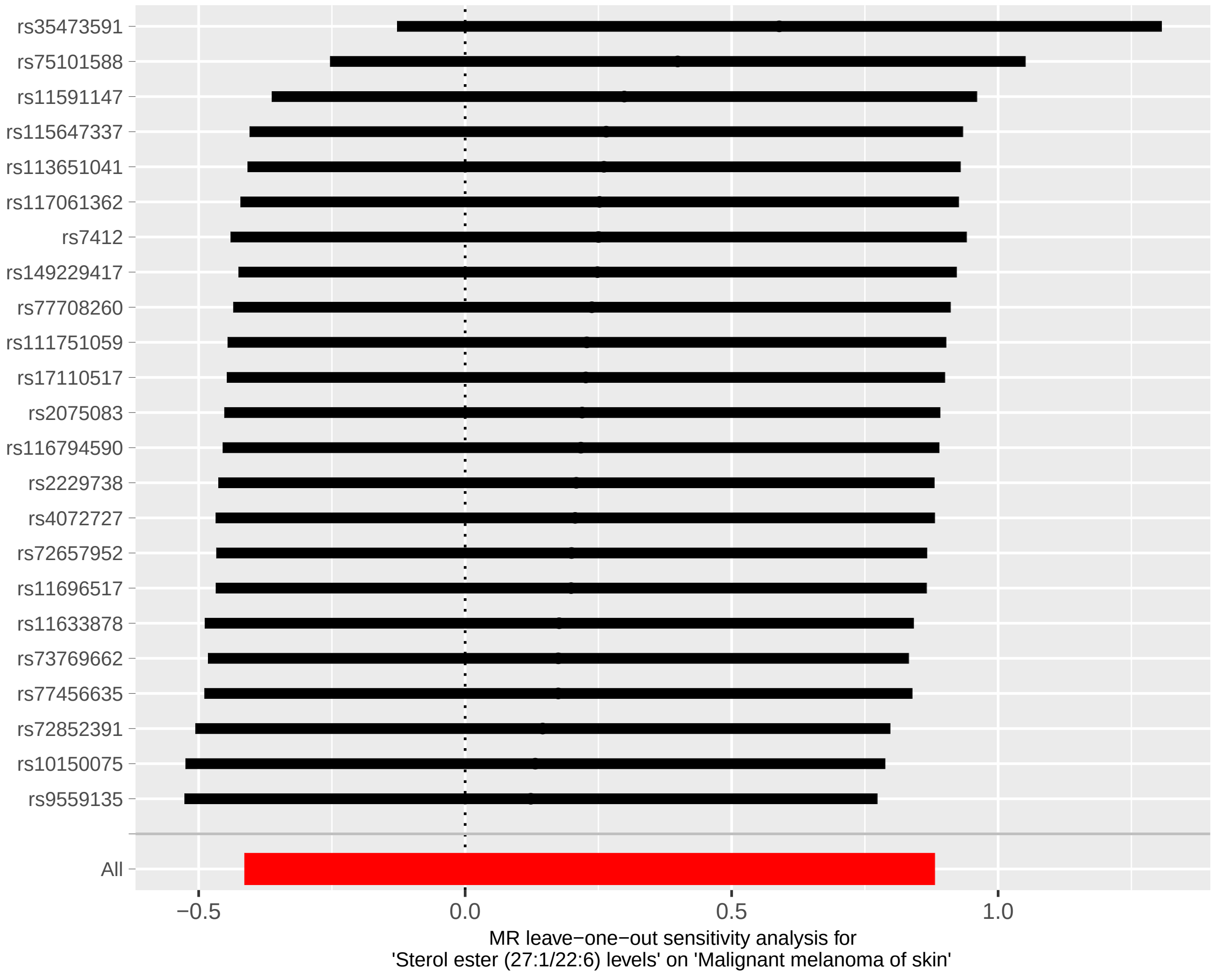
rs35473591
rs75101588
rs11591147
rs115647337
rs113651041
rs117061362
rs7412
rs149229417
rs77708260
rs111751059
rs17110517
rs2075083
rs116794590
rs2229738
rs4072727
rs72657952
rs11696517
rs11633878
rs73769662
rs77456635
rs72852391
rs10150075
rs9559135
All
−0.5
0.0
0.5
1.0
MR leave−one−out sensitivity analysis for
'Sterol ester (27:1/22:6) levels' on 'Malignant melanoma of skin'

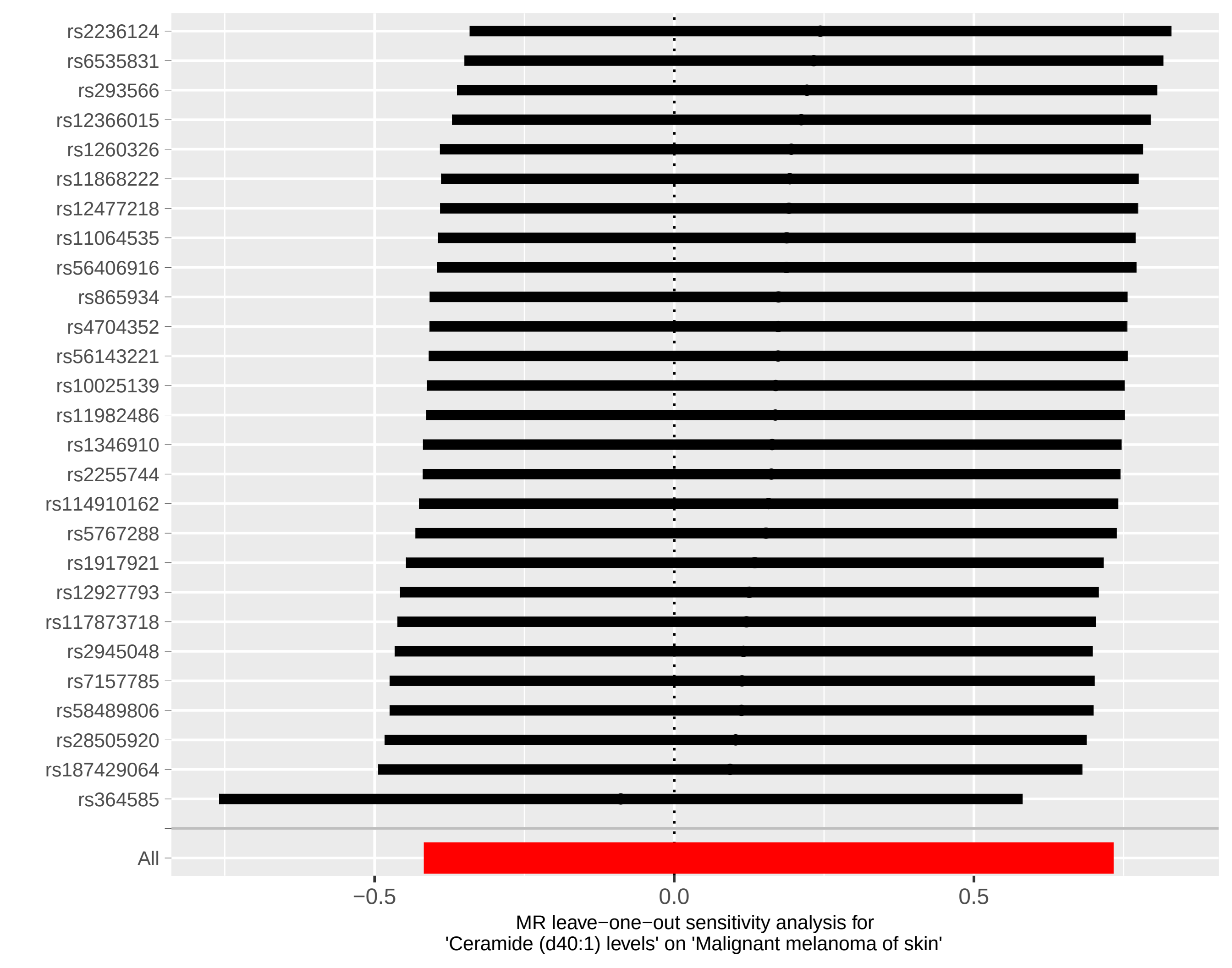
rs2236124
rs6535831
rs293566
rs12366015
rs1260326
rs11868222
rs12477218
rs11064535
rs56406916
rs865934
rs4704352
rs56143221
rs10025139
rs11982486
rs1346910
rs2255744
rs114910162
rs5767288
rs1917921
rs12927793
rs117873718
rs2945048
rs7157785
rs58489806
rs28505920
rs187429064
rs364585
All
−0.5
0.0
0.5
MR leave−one−out sensitivity analysis for
'Ceramide (d40:1) levels' on 'Malignant melanoma of skin'

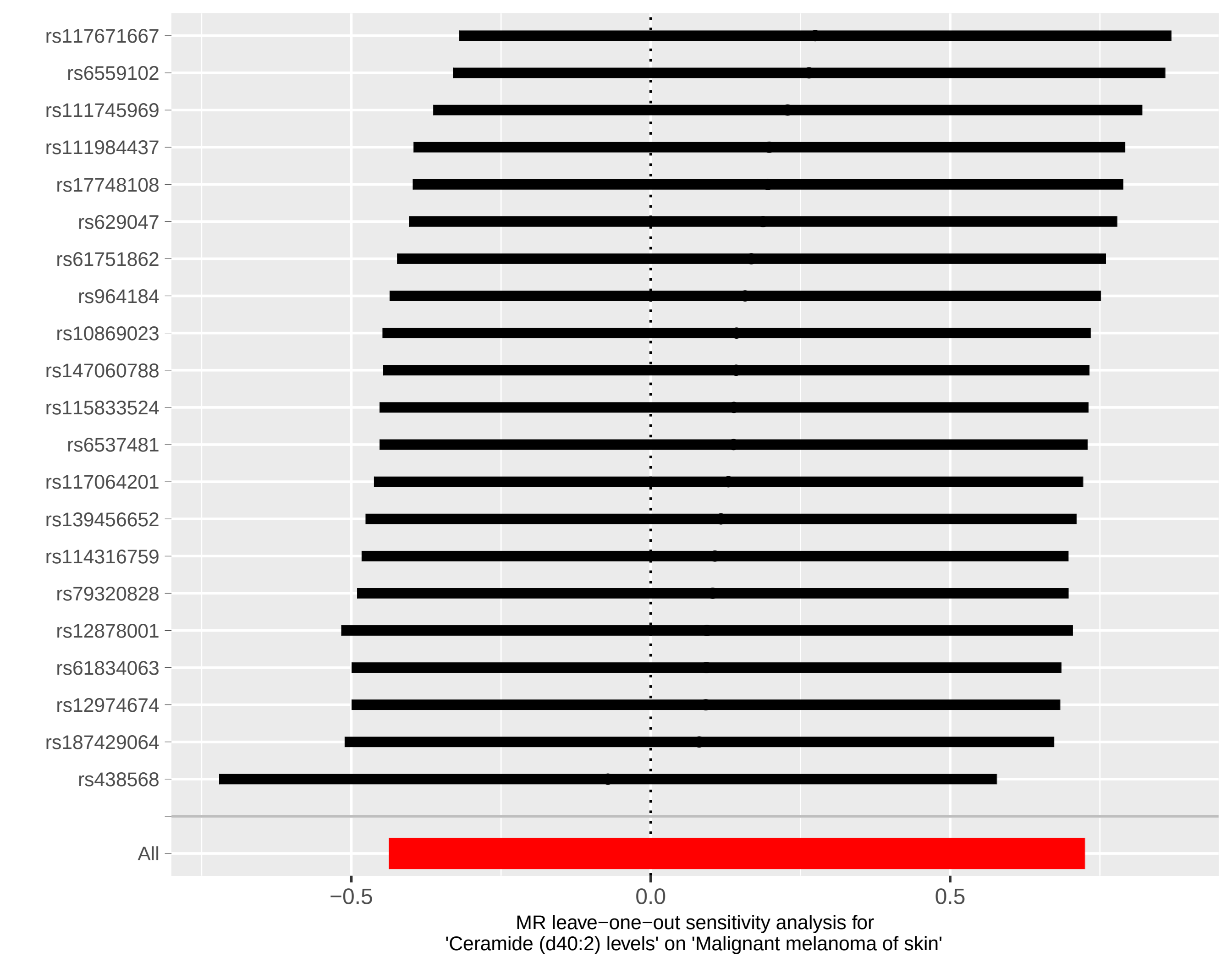
rs117671667
rs6559102
rs111745969
rs111984437
rs17748108
rs629047
rs61751862
rs964184
rs10869023
rs147060788
rs115833524
rs6537481
rs117064201
rs139456652
rs114316759
rs79320828
rs12878001
rs61834063
rs12974674
rs187429064
rs438568
All
−0.5
0.0
0.5
MR leave−one−out sensitivity analysis for
'Ceramide (d40:2) levels' on 'Malignant melanoma of skin'

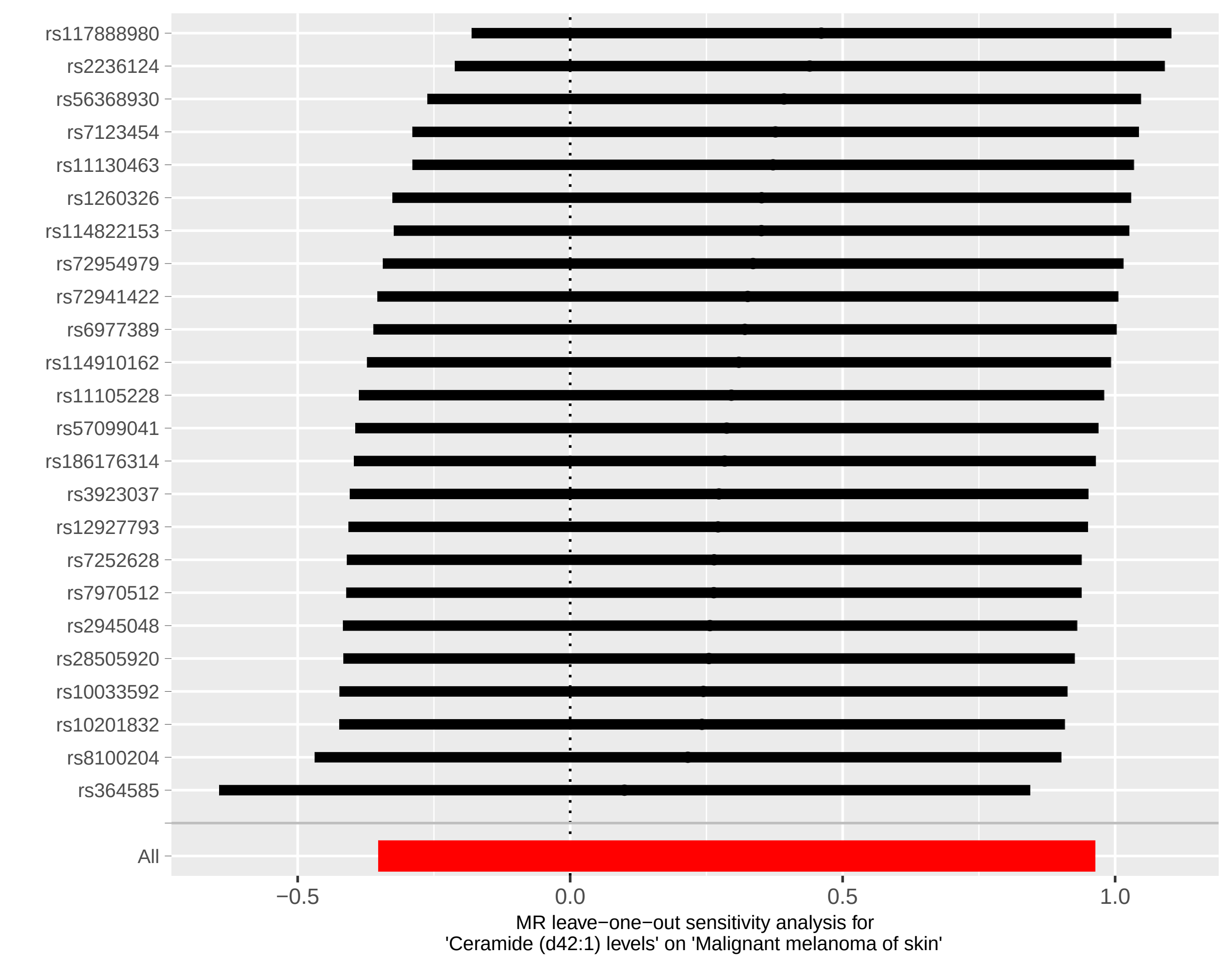

rs117888980
rs2236124
rs56368930
rs7123454
rs11130463
rs1260326
rs114822153
rs72954979
rs72941422
rs6977389
rs114910162
rs11105228
rs57099041
rs186176314
rs3923037
rs12927793
rs7252628
rs7970512
rs2945048
rs28505920
rs10033592
rs10201832
rs8100204
rs364585
All
−0.5
0.0
0.5
1.0
MR leave−one−out sensitivity analysis for
'Ceramide (d42:1) levels' on 'Malignant melanoma of skin'

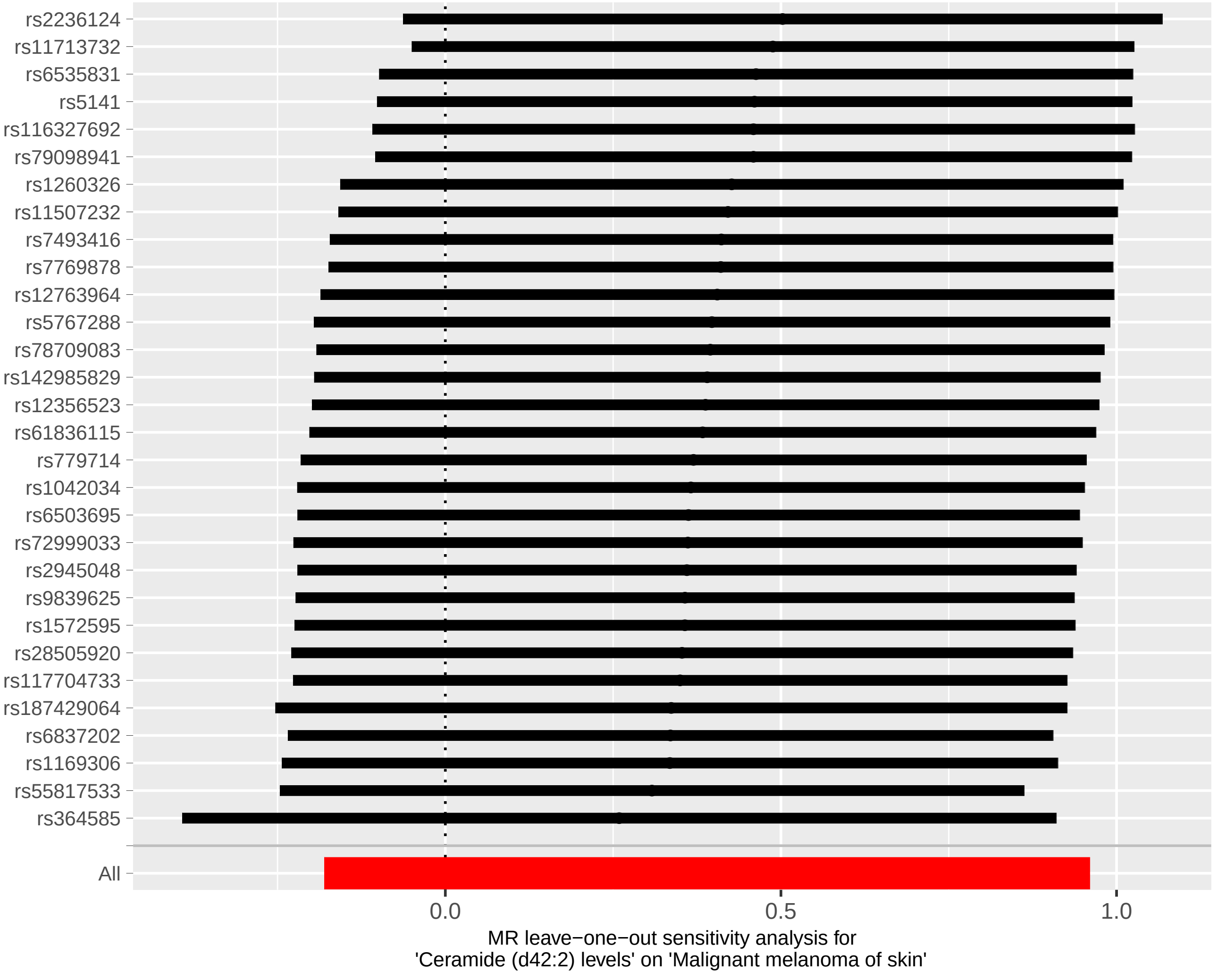
rs2236124
rs11713732
rs6535831
rs5141
rs116327692
rs79098941
rs1260326
rs11507232
rs7493416
rs7769878
rs12763964
rs5767288
rs78709083
rs142985829
rs12356523
rs61836115
rs779714
rs1042034
rs6503695
rs72999033
rs2945048
rs9839625
rs1572595
rs28505920
rs117704733
rs187429064
rs6837202
rs1169306
rs55817533
rs364585
All
0.0
0.5
1.0
MR leave−one−out sensitivity analysis for
'Ceramide (d42:2) levels' on 'Malignant melanoma of skin'

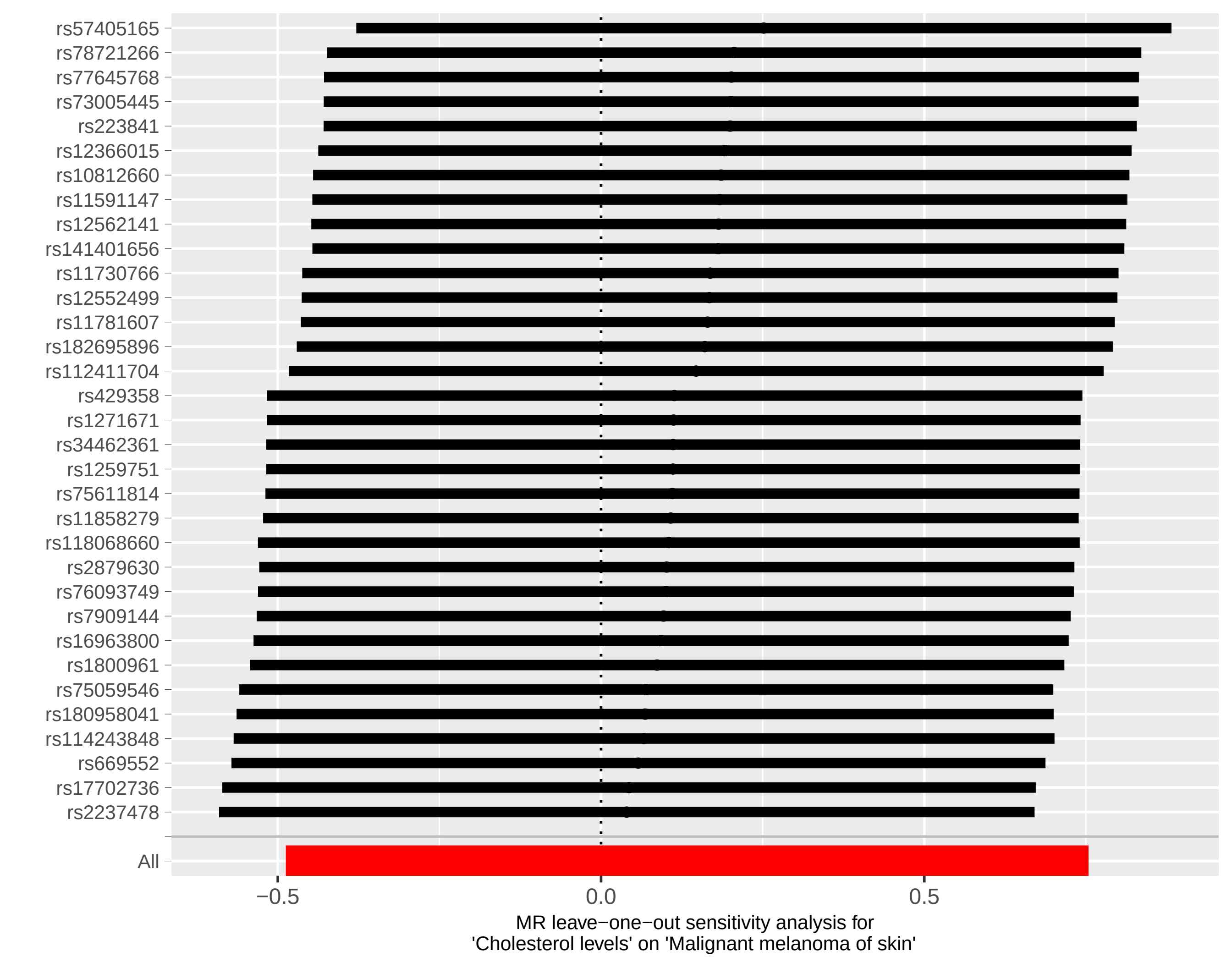
rs57405165
rs78721266
rs77645768
rs73005445
rs223841
rs12366015
rs10812660
rs11591147
rs12562141
rs141401656
rs11730766
rs12552499
rs11781607
rs182695896
rs112411704
rs429358
rs1271671
rs34462361
rs1259751
rs75611814
rs11858279
rs118068660
rs2879630
rs76093749
rs7909144
rs16963800
rs1800961
rs75059546
rs180958041
rs114243848
rs669552
rs17702736
rs2237478
All
−0.5
0.0
0.5
MR leave−one−out sensitivity analysis for
'Cholesterol levels' on 'Malignant melanoma of skin'

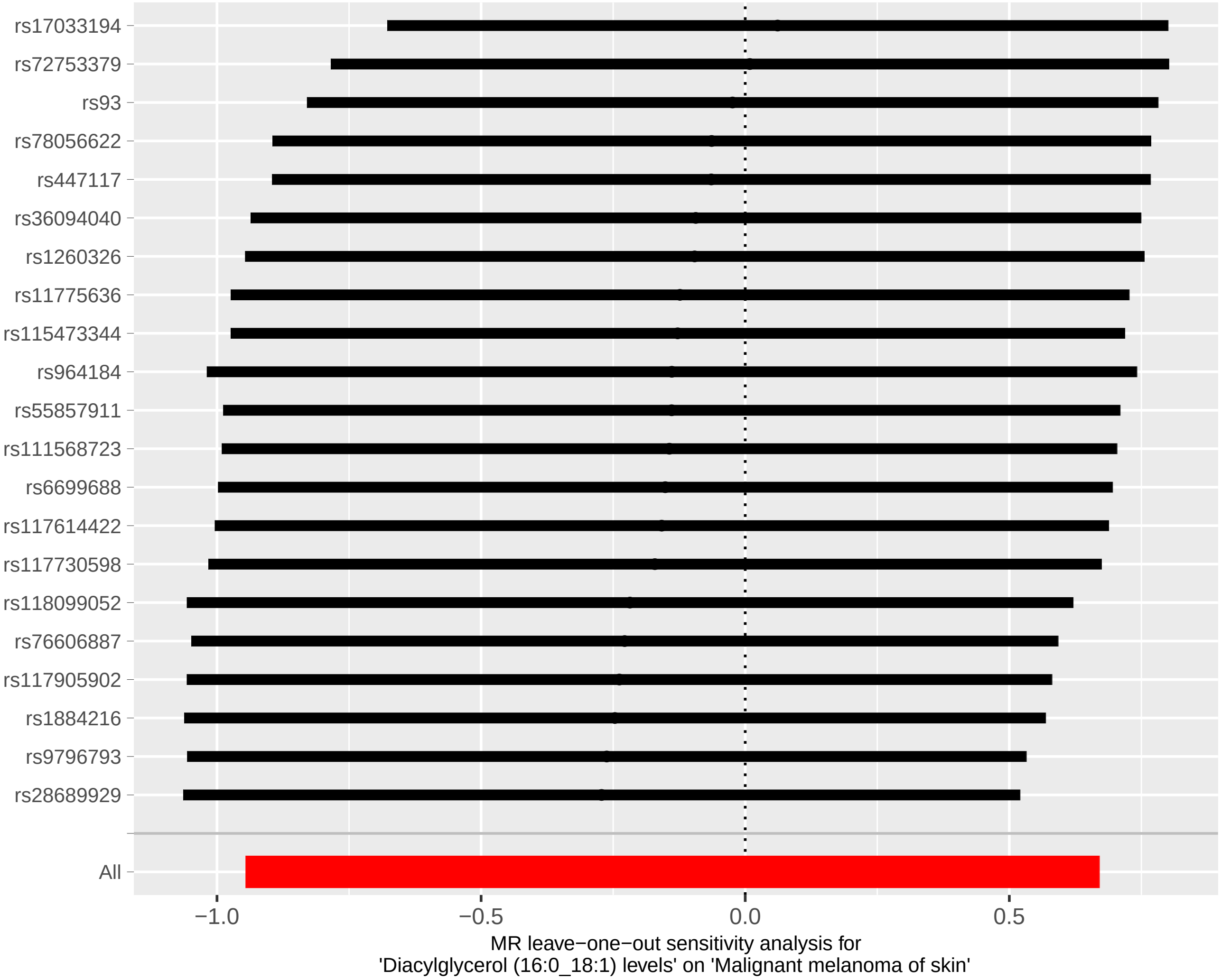
rs17033194
rs72753379
rs93
rs78056622
rs447117
rs36094040
rs1260326
rs11775636
rs115473344
rs964184
rs55857911
rs111568723
rs6699688
rs117614422
rs117730598
rs118099052
rs76606887
rs117905902
rs1884216
rs9796793
rs28689929
All
−1.0
−0.5
0.0
0.5
MR leave−one−out sensitivity analysis for
'Diacylglycerol (16:0_18:1) levels' on 'Malignant melanoma of skin'

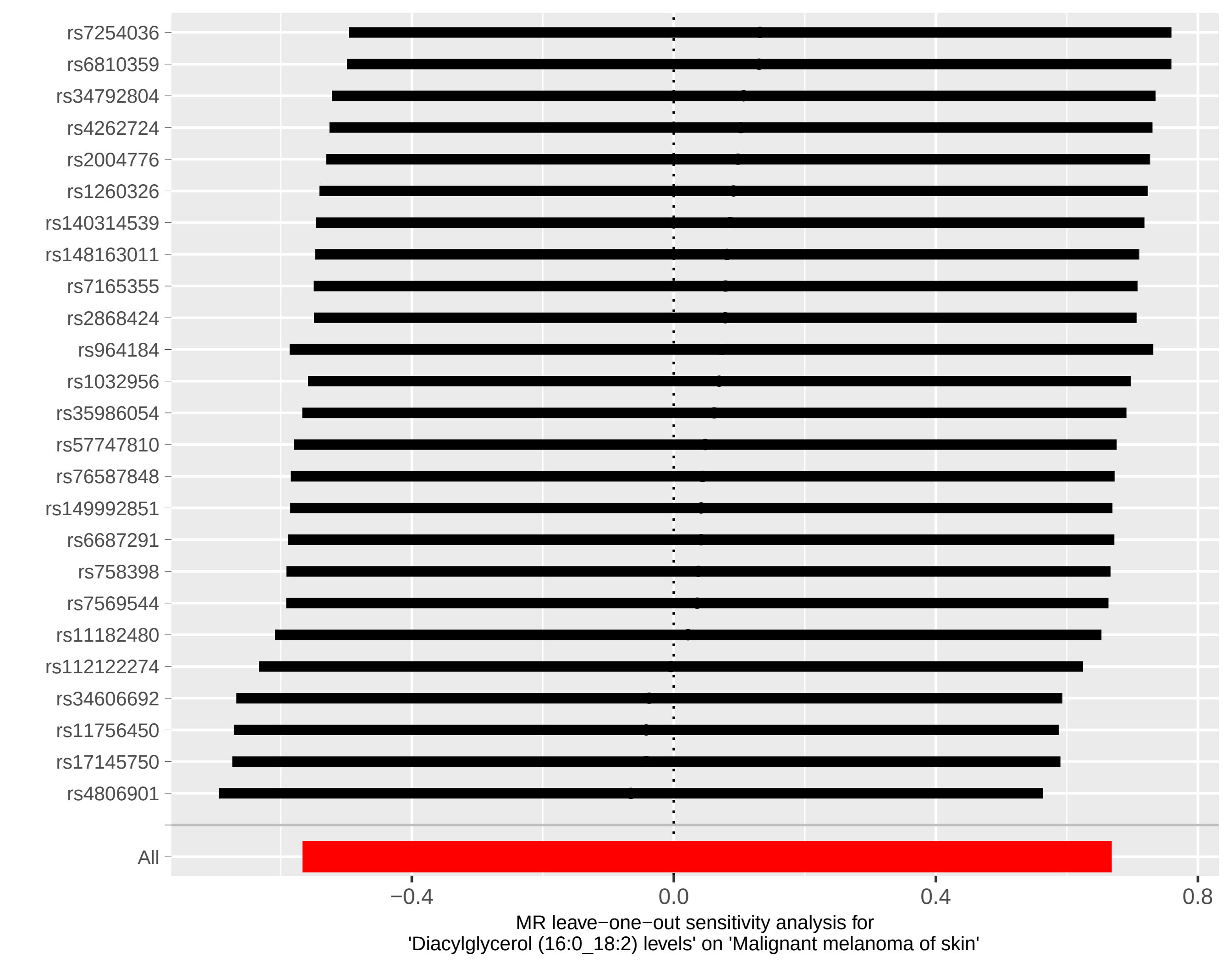
rs7254036
rs6810359
rs34792804
rs4262724
rs2004776
rs1260326
rs140314539
rs148163011
rs7165355
rs2868424
rs964184
rs1032956
rs35986054
rs57747810
rs76587848
rs149992851
rs6687291
rs758398
rs7569544
rs11182480
rs112122274
rs34606692
rs11756450
rs17145750
rs4806901
All
−0.4
0.0
0.4
0.8
MR leave−one−out sensitivity analysis for
'Diacylglycerol (16:0_18:2) levels' on 'Malignant melanoma of skin'

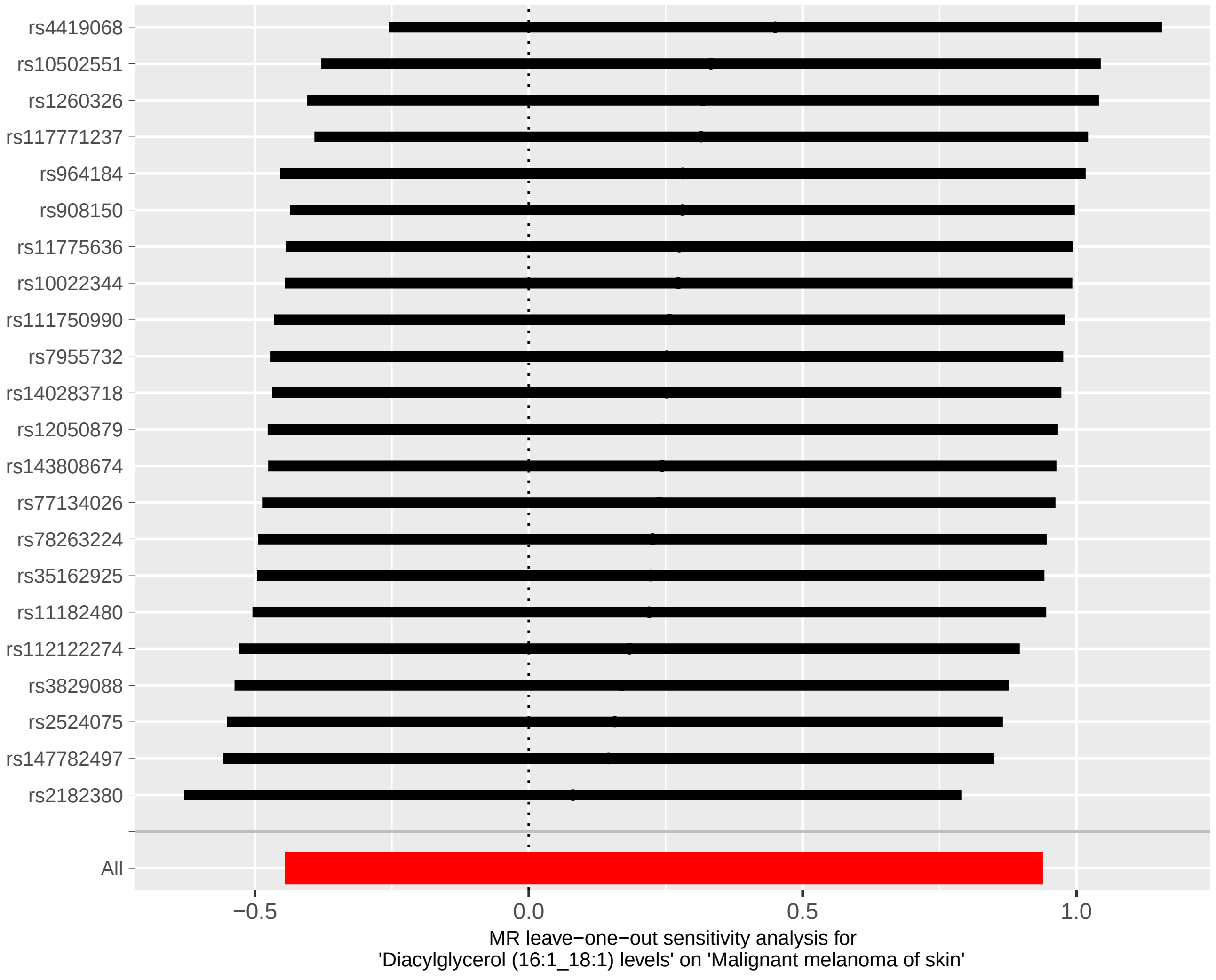
rs4419068
rs10502551
rs1260326
rs117771237
rs964184
rs908150
rs11775636
rs10022344
rs111750990
rs7955732
rs140283718
rs12050879
rs143808674
rs77134026
rs78263224
rs35162925
rs11182480
rs112122274
rs3829088
rs2524075
rs147782497
rs2182380
All
−0.5
0.0
0.5
1.0
MR leave−one−out sensitivity analysis for
'Diacylglycerol (16:1_18:1) levels' on 'Malignant melanoma of skin'

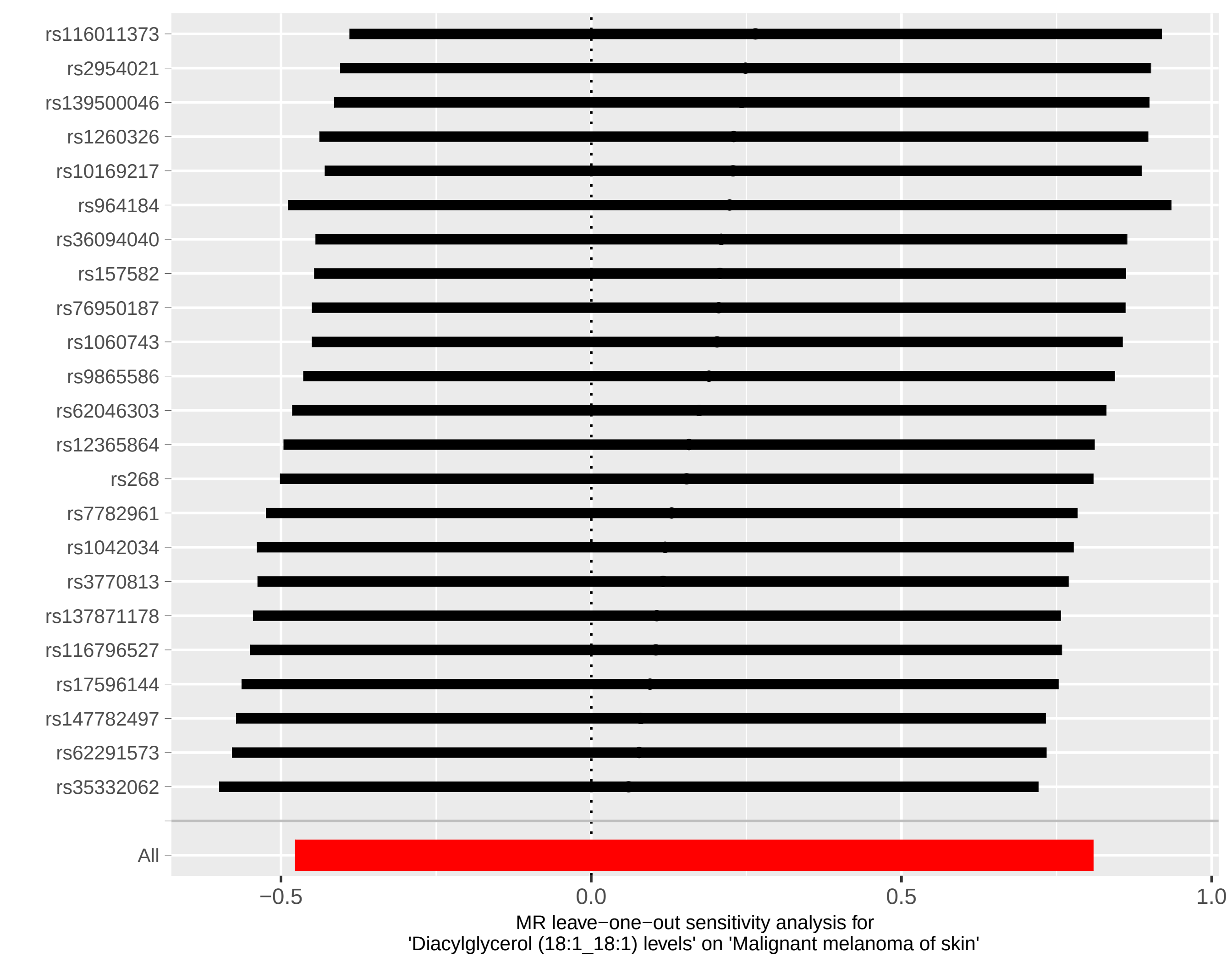

rs116011373
rs2954021
rs139500046
rs1260326
rs10169217
rs964184
rs36094040
rs157582
rs76950187
rs1060743
rs9865586
rs62046303
rs12365864
rs268
rs7782961
rs1042034
rs3770813
rs137871178
rs116796527
rs17596144
rs147782497
rs62291573
rs35332062
All
−0.5
0.0
0.5
1.0
MR leave−one−out sensitivity analysis for
'Diacylglycerol (18:1_18:1) levels' on 'Malignant melanoma of skin'

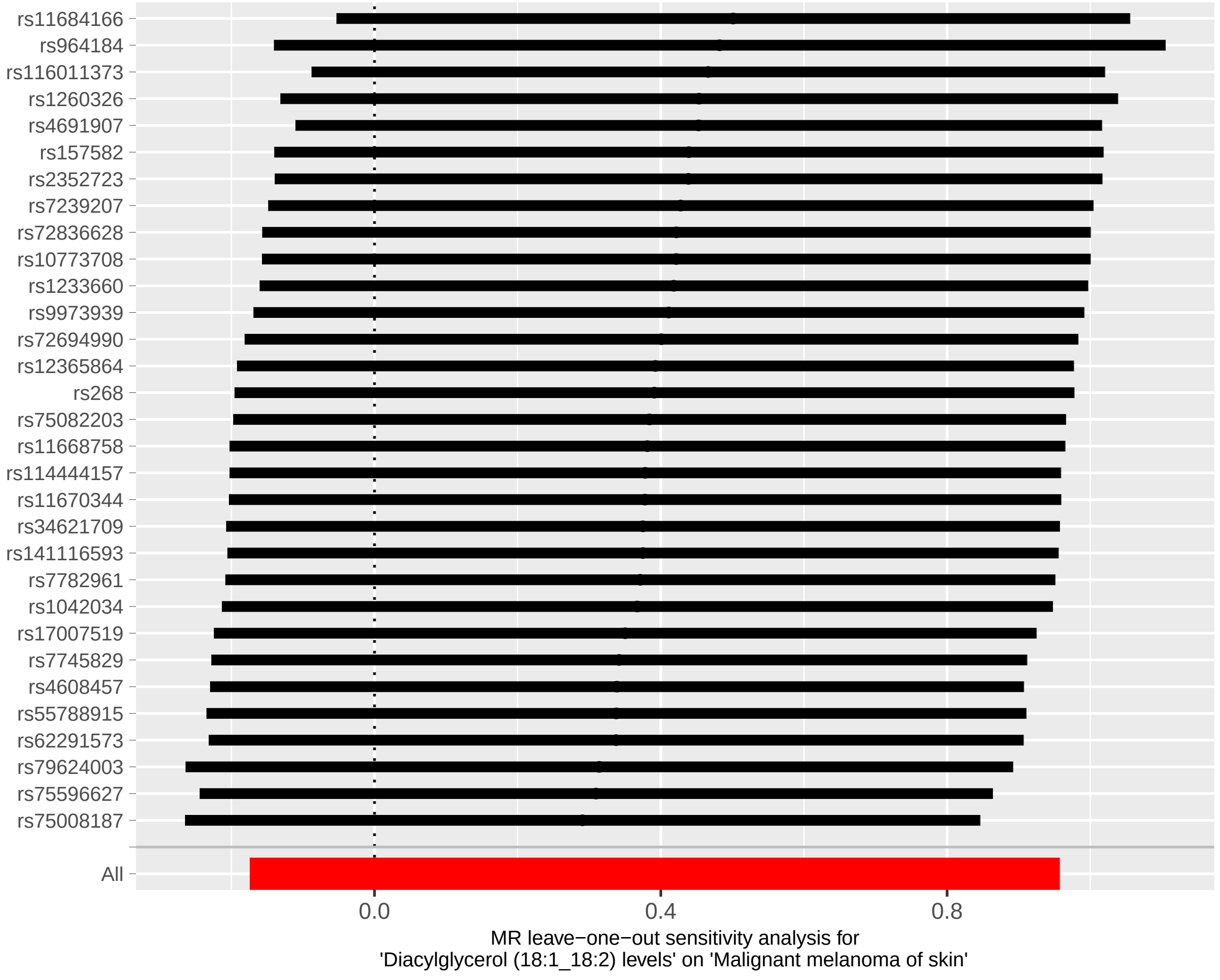
rs11684166
rs964184
rs116011373
rs1260326
rs4691907
rs157582
rs2352723
rs7239207
rs72836628
rs10773708
rs1233660
rs9973939
rs72694990
rs12365864
rs268
rs75082203
rs11668758
rs114444157
rs11670344
rs34621709
rs141116593
rs7782961
rs1042034
rs17007519
rs7745829
rs4608457
rs55788915
rs62291573
rs79624003
rs75596627
rs75008187
All
0.0
0.4
0.8
MR leave−one−out sensitivity analysis for
'Diacylglycerol (18:1_18:2) levels' on 'Malignant melanoma of skin'

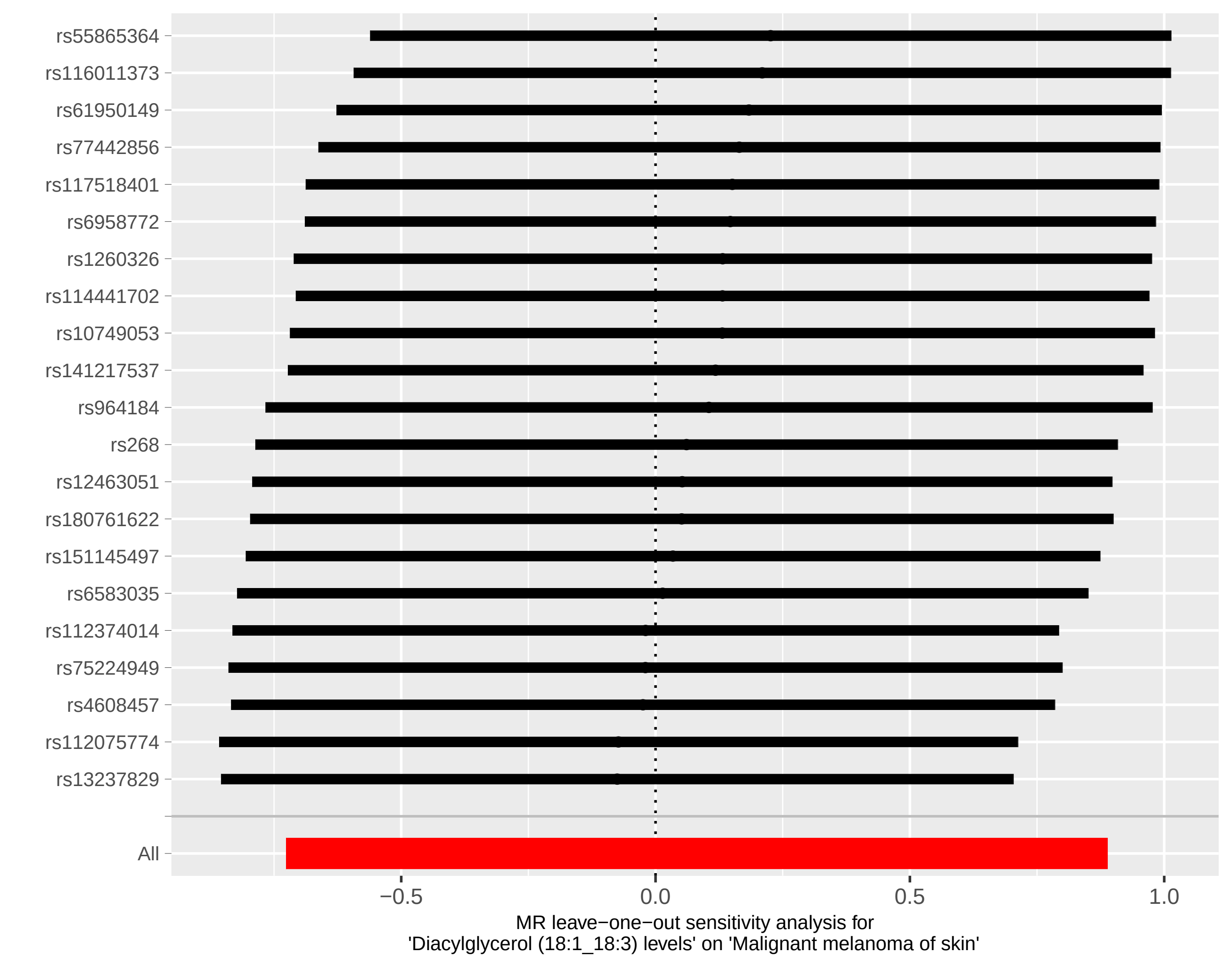
rs55865364
rs116011373
rs61950149
rs77442856
rs117518401
rs6958772
rs1260326
rs114441702
rs10749053
rs141217537
rs964184
rs268
rs12463051
rs180761622
rs151145497
rs6583035
rs112374014
rs75224949
rs4608457
rs112075774
rs13237829
All
−0.5
0.0
0.5
1.0
MR leave−one−out sensitivity analysis for
'Diacylglycerol (18:1_18:3) levels' on 'Malignant melanoma of skin'

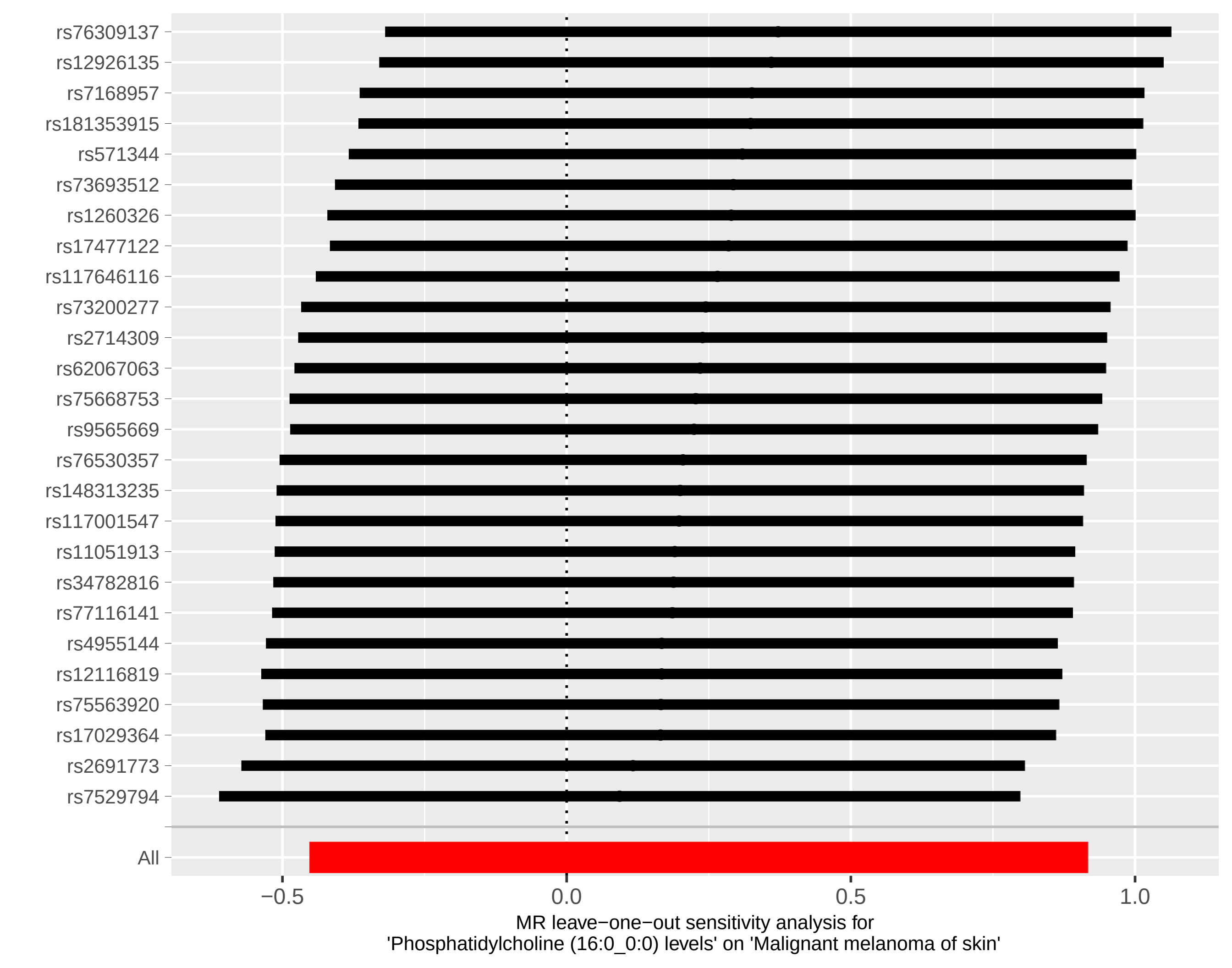

rs76309137
rs12926135
rs7168957
rs181353915
rs571344
rs73693512
rs1260326
rs17477122
rs117646116
rs73200277
rs2714309
rs62067063
rs75668753
rs9565669
rs76530357
rs148313235
rs117001547
rs11051913
rs34782816
rs77116141
rs4955144
rs12116819
rs75563920
rs17029364
rs2691773
rs7529794
All
−0.5
0.0
0.5
1.0
MR leave−one−out sensitivity analysis for
'Phosphatidylcholine (16:0_0:0) levels' on 'Malignant melanoma of skin'

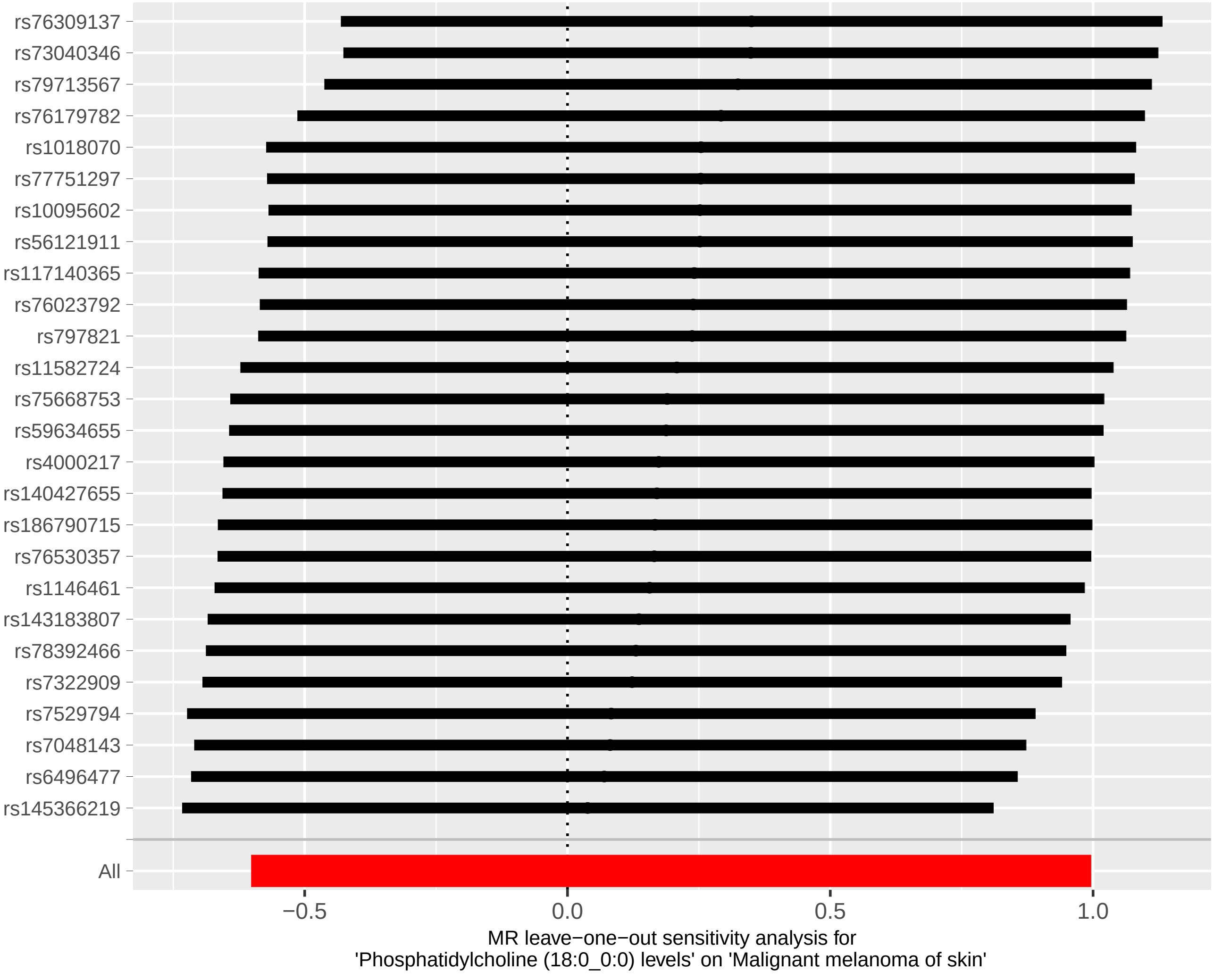
rs76309137
rs73040346
rs79713567
rs76179782
rs1018070
rs77751297
rs10095602
rs56121911
rs117140365
rs76023792
rs797821
rs11582724
rs75668753
rs59634655
rs4000217
rs140427655
rs186790715
rs76530357
rs1146461
rs143183807
rs78392466
rs7322909
rs7529794
rs7048143
rs6496477
rs145366219
All
−0.5
0.0
0.5
1.0
MR leave−one−out sensitivity analysis for
'Phosphatidylcholine (18:0_0:0) levels' on 'Malignant melanoma of skin'

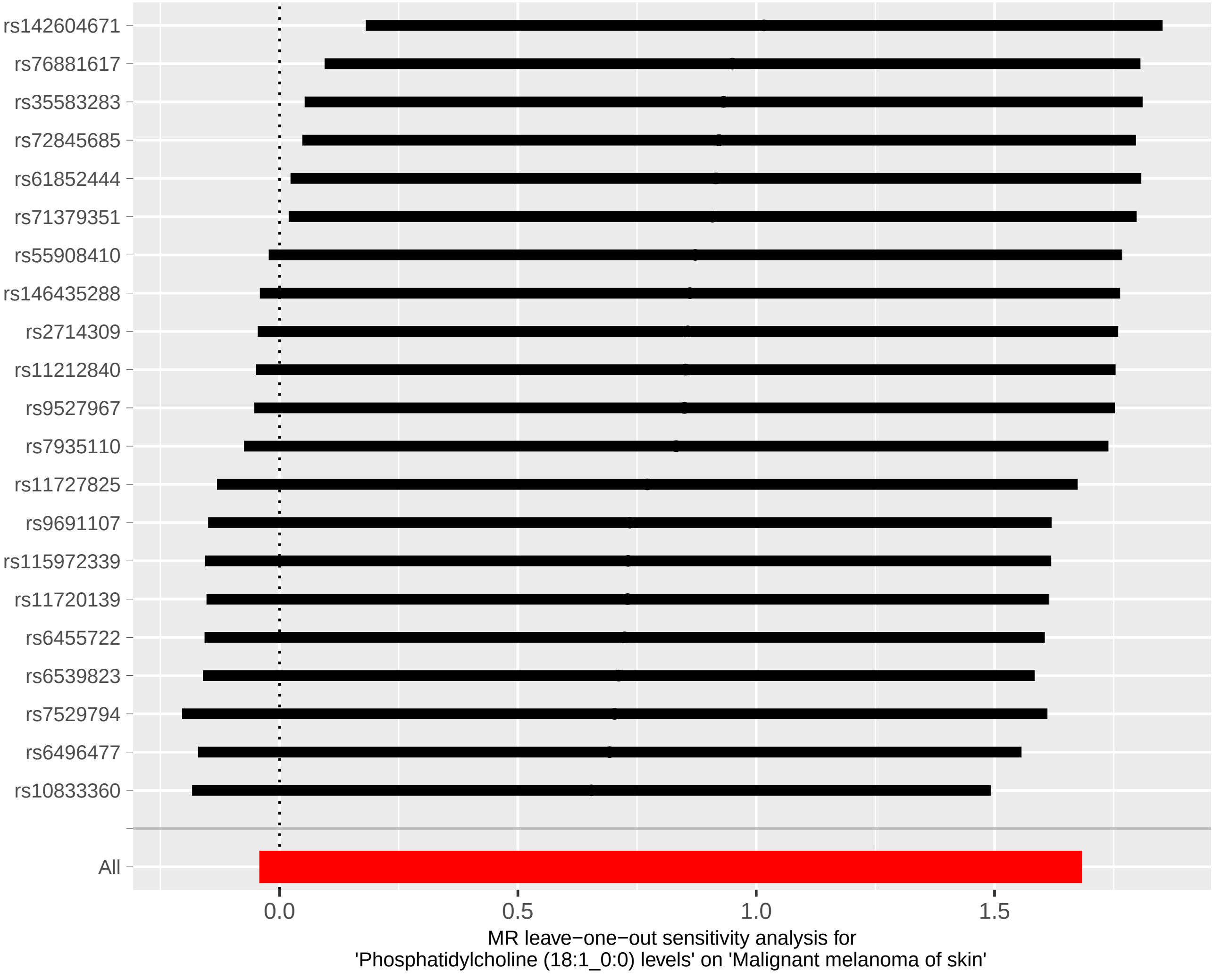
rs142604671
rs76881617
rs35583283
rs72845685
rs61852444
rs71379351
rs55908410
rs146435288
rs2714309
rs11212840
rs9527967
rs7935110
rs11727825
rs9691107
rs115972339
rs11720139
rs6455722
rs6539823
rs7529794
rs6496477
rs10833360
All
0.0
0.5
1.0
1.5
MR leave−one−out sensitivity analysis for
'Phosphatidylcholine (18:1_0:0) levels' on 'Malignant melanoma of skin'

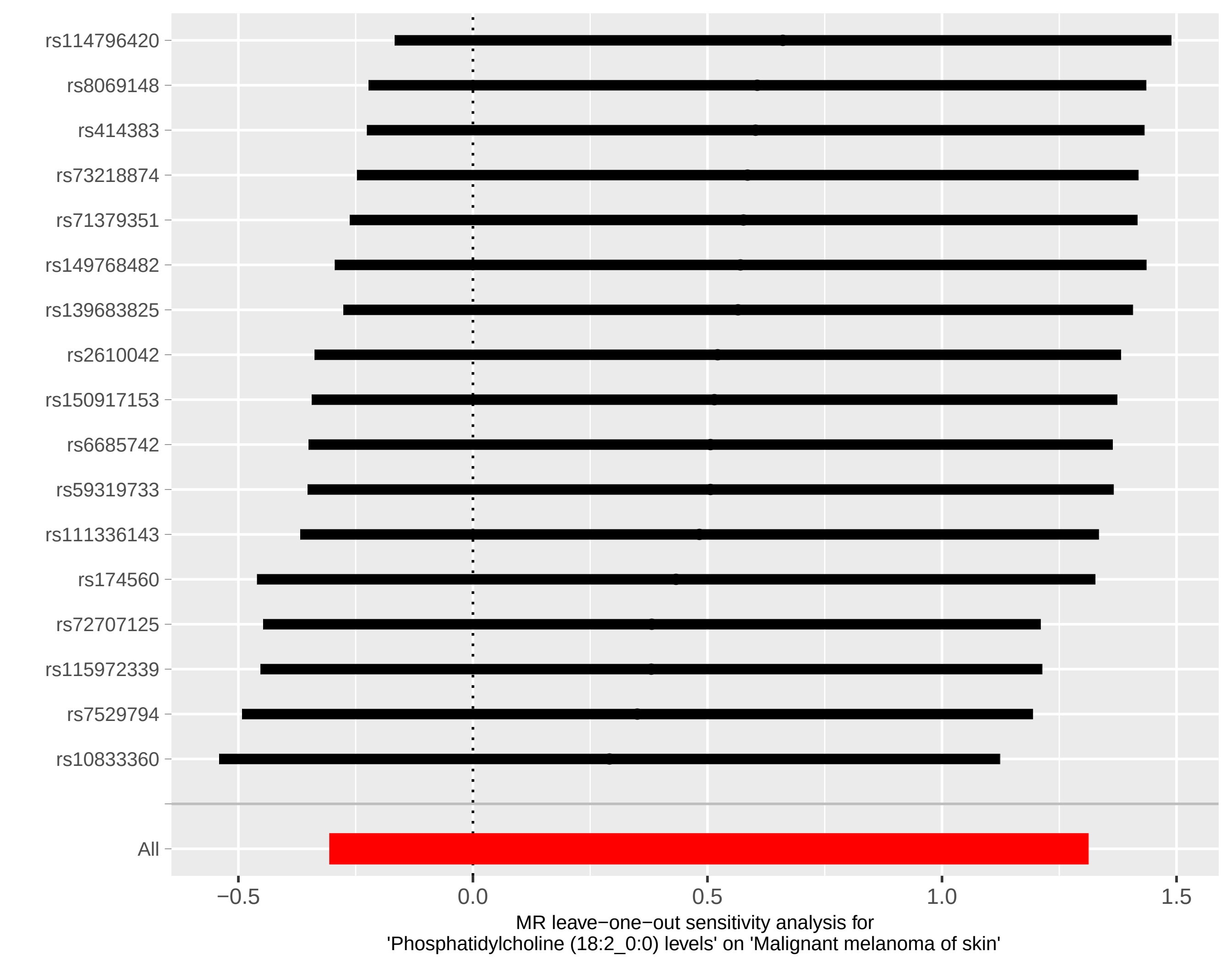
rs114796420
rs8069148
rs414383
rs73218874
rs71379351
rs149768482
rs139683825
rs2610042
rs150917153
rs6685742
rs59319733
rs111336143
rs174560
rs72707125
rs115972339
rs7529794
rs10833360
All
−0.5
0.0
0.5
1.0
1.5
MR leave−one−out sensitivity analysis for
'Phosphatidylcholine (18:2_0:0) levels' on 'Malignant melanoma of skin'

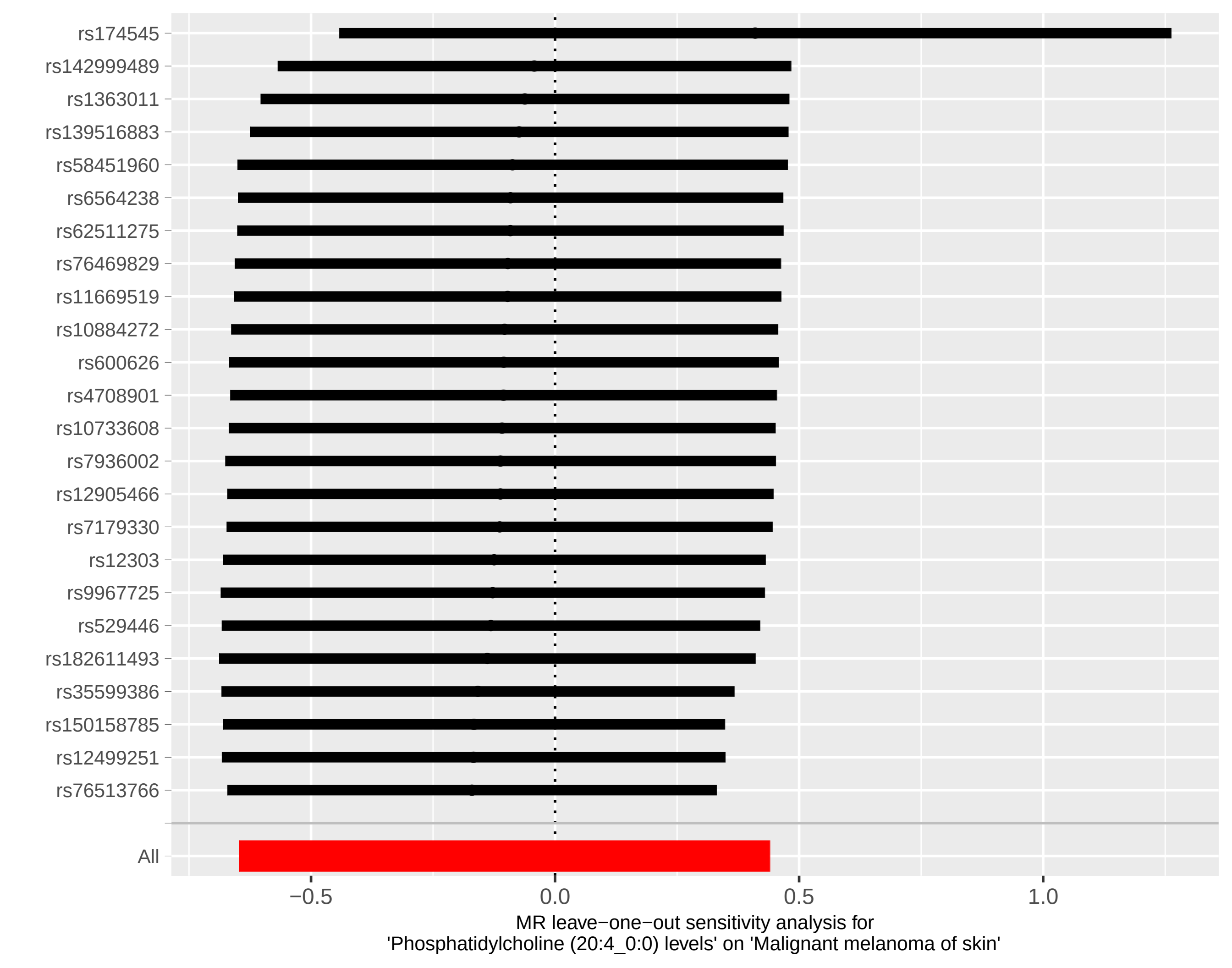

rs174545
rs142999489
rs1363011
rs139516883
rs58451960
rs6564238
rs62511275
rs76469829
rs11669519
rs10884272
rs600626
rs4708901
rs10733608
rs7936002
rs12905466
rs7179330
rs12303
rs9967725
rs529446
rs182611493
rs35599386
rs150158785
rs12499251
rs76513766
All
−0.5
0.0
0.5
1.0
MR leave−one−out sensitivity analysis for
'Phosphatidylcholine (20:4_0:0) levels' on 'Malignant melanoma of skin'

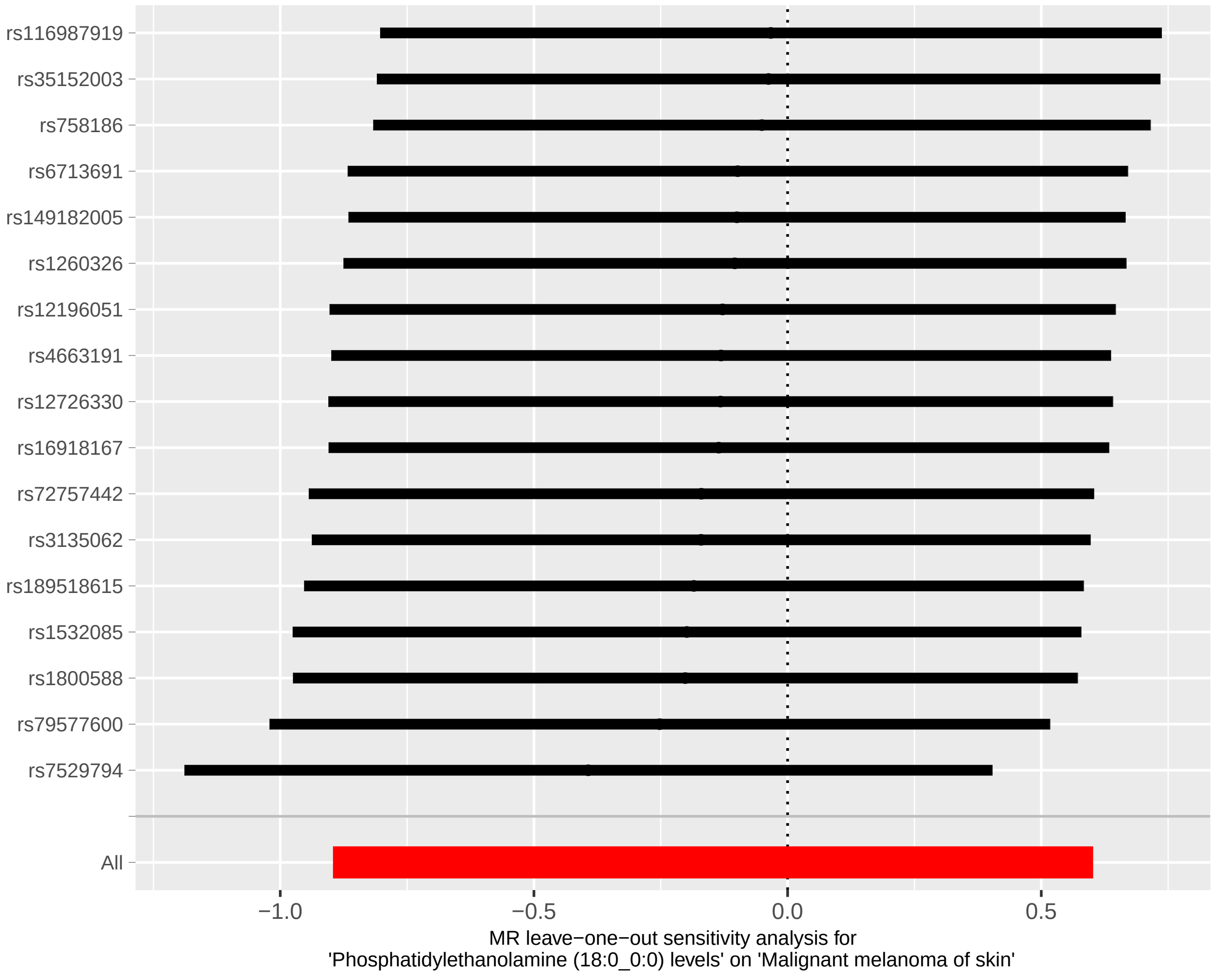

rs116987919
rs35152003
rs758186
rs6713691
rs149182005
rs1260326
rs12196051
rs4663191
rs12726330
rs16918167
rs72757442
rs3135062
rs189518615
rs1532085
rs1800588
rs79577600
rs7529794
All
−1.0
−0.5
0.0
0.5
MR leave−one−out sensitivity analysis for
'Phosphatidylethanolamine (18:0_0:0) levels' on 'Malignant melanoma of skin'

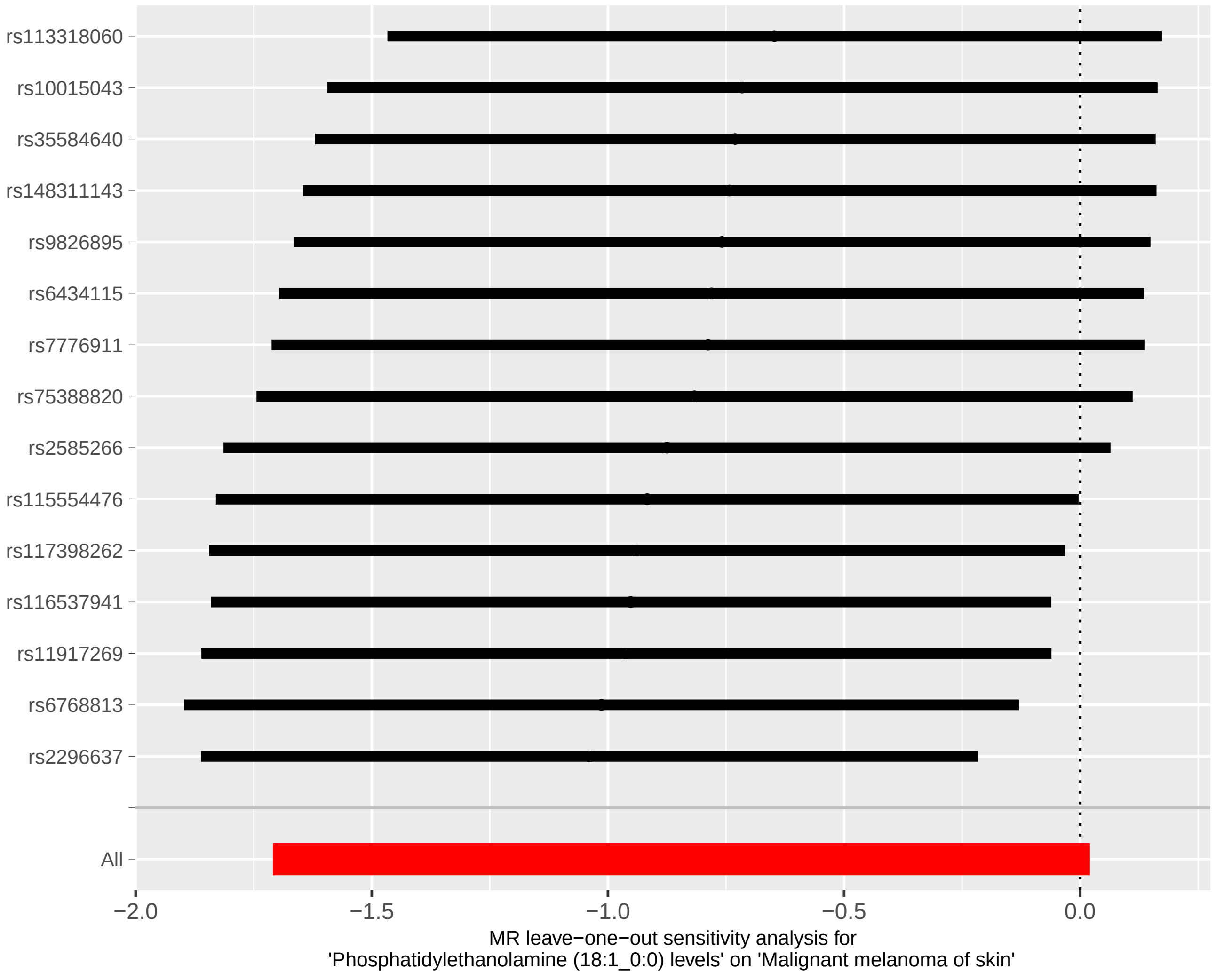
rs113318060
rs10015043
rs35584640
rs148311143
rs9826895
rs6434115
rs7776911
rs75388820
rs2585266
rs115554476
rs117398262
rs116537941
rs11917269
rs6768813
rs2296637
All
−2.0
−1.5
−1.0
−0.5
0.0
MR leave−one−out sensitivity analysis for
'Phosphatidylethanolamine (18:1_0:0) levels' on 'Malignant melanoma of skin'

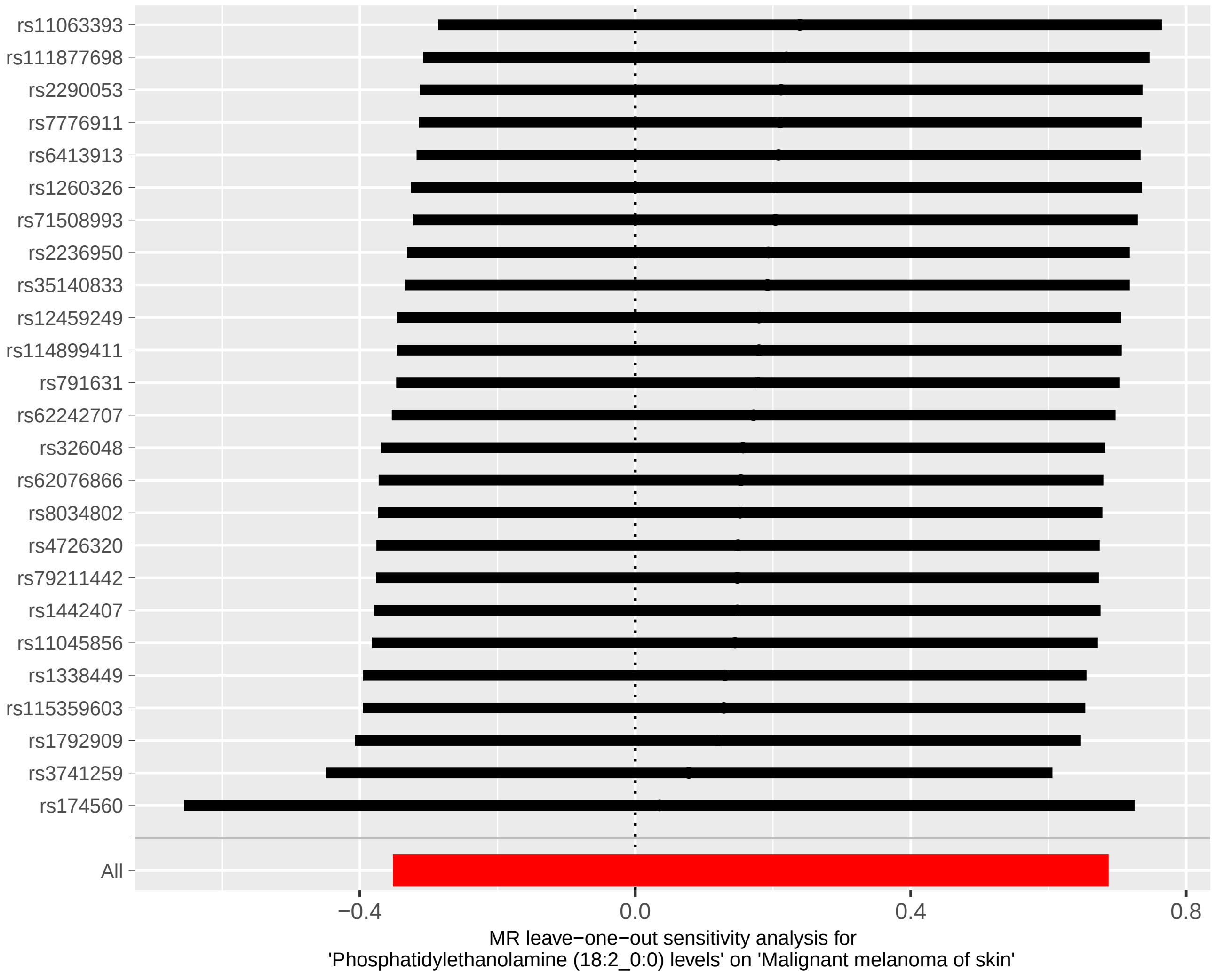
rs11063393
rs111877698
rs2290053
rs7776911
rs6413913
rs1260326
rs71508993
rs2236950
rs35140833
rs12459249
rs114899411
rs791631
rs62242707
rs326048
rs62076866
rs8034802
rs4726320
rs79211442
rs1442407
rs11045856
rs1338449
rs115359603
rs1792909
rs3741259
rs174560
All
−0.4
0.0
0.4
0.8
MR leave−one−out sensitivity analysis for
'Phosphatidylethanolamine (18:2_0:0) levels' on 'Malignant melanoma of skin'

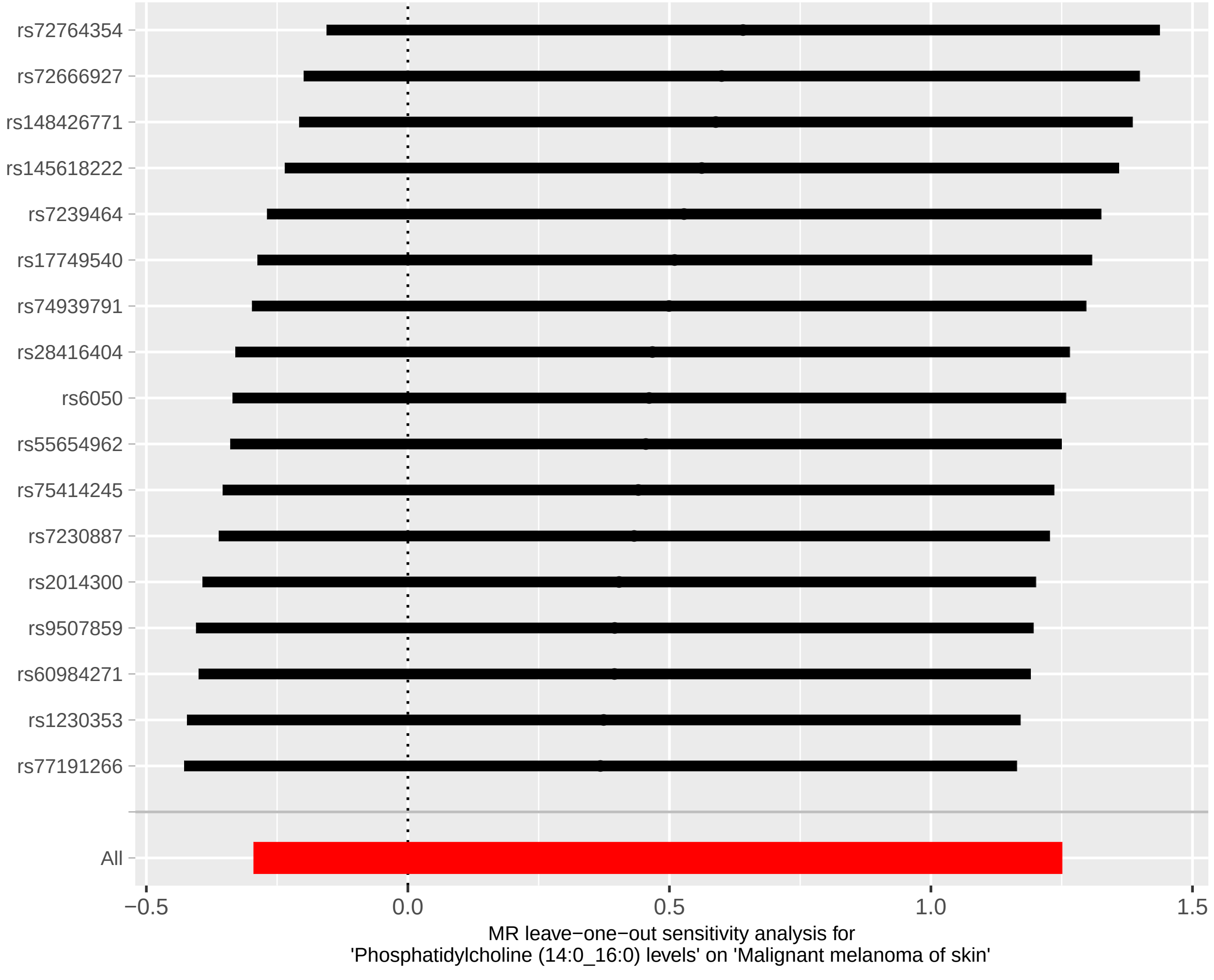

rs72764354
rs72666927
rs148426771
rs145618222
rs7239464
rs17749540
rs74939791
rs28416404
rs6050
rs55654962
rs75414245
rs7230887
rs2014300
rs9507859
rs60984271
rs1230353
rs77191266
All
−0.5
0.0
0.5
1.0
1.5
MR leave−one−out sensitivity analysis for
'Phosphatidylcholine (14:0_16:0) levels' on 'Malignant melanoma of skin'

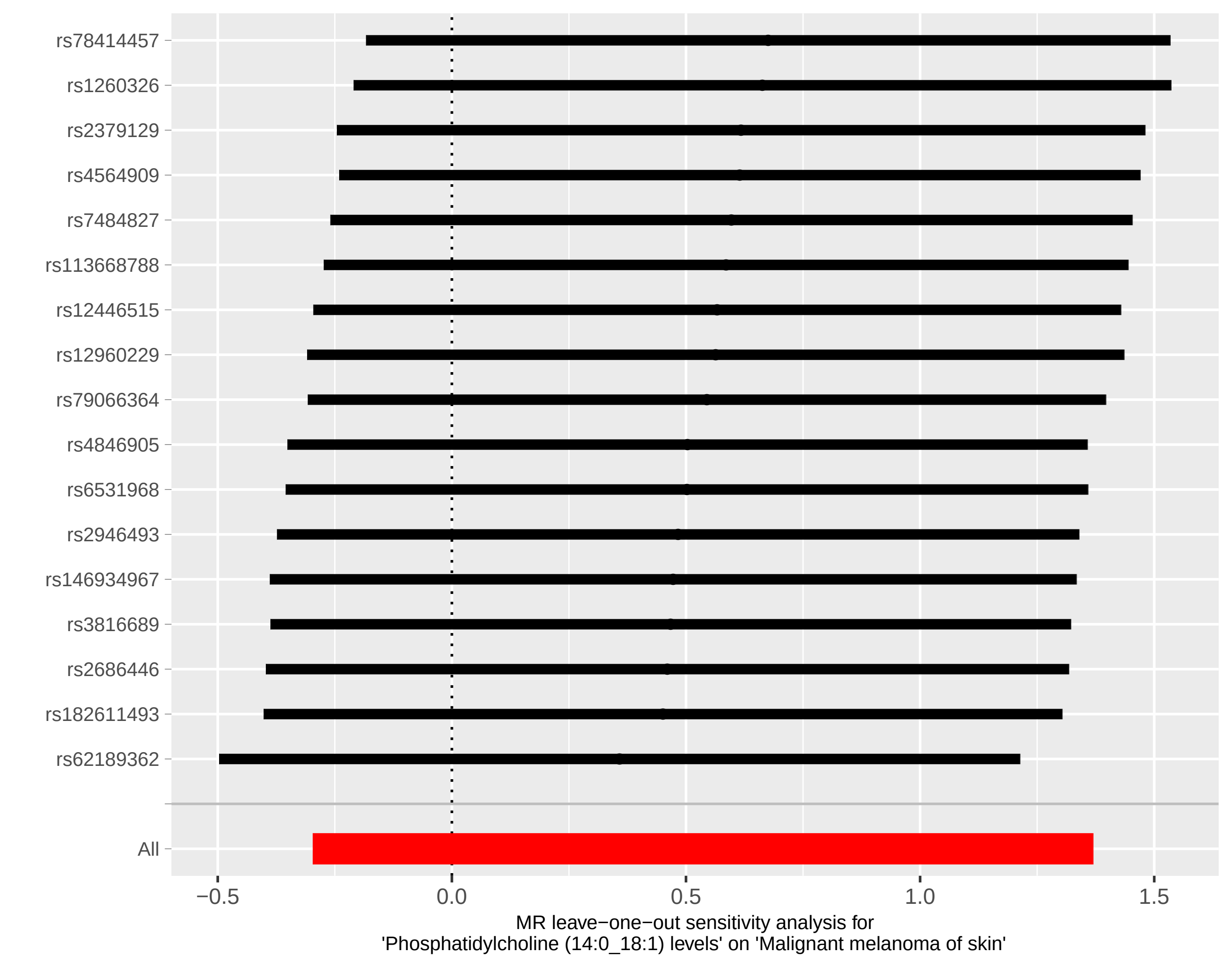
rs78414457
rs1260326
rs2379129
rs4564909
rs7484827
rs113668788
rs12446515
rs12960229
rs79066364
rs4846905
rs6531968
rs2946493
rs146934967
rs3816689
rs2686446
rs182611493
rs62189362
All
−0.5
0.0
0.5
1.0
1.5
MR leave−one−out sensitivity analysis for
'Phosphatidylcholine (14:0_18:1) levels' on 'Malignant melanoma of skin'

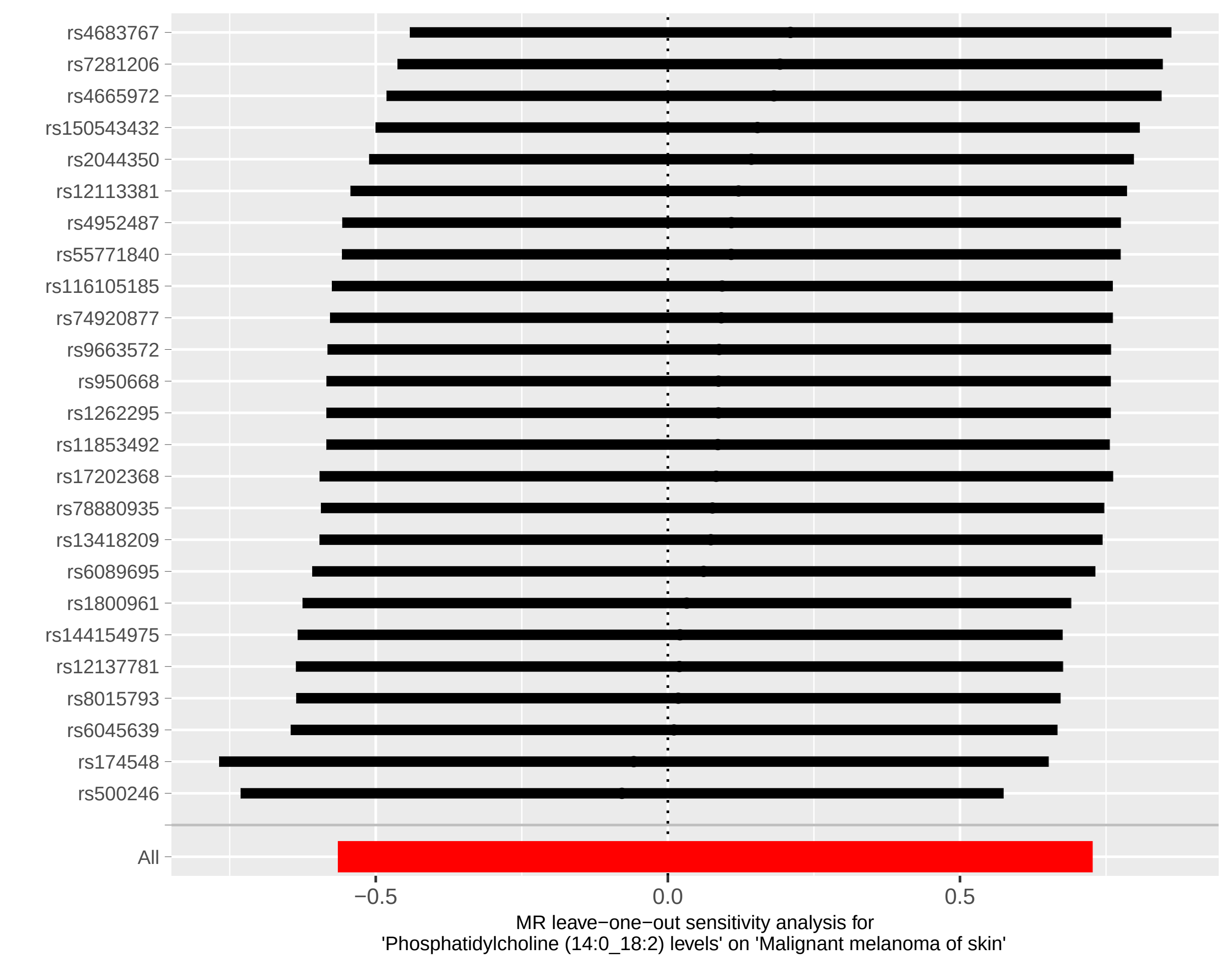

rs4683767
rs7281206
rs4665972
rs150543432
rs2044350
rs12113381
rs4952487
rs55771840
rs116105185
rs74920877
rs9663572
rs950668
rs1262295
rs11853492
rs17202368
rs78880935
rs13418209
rs6089695
rs1800961
rs144154975
rs12137781
rs8015793
rs6045639
rs174548
rs500246
All
−0.5
0.0
0.5
MR leave−one−out sensitivity analysis for
'Phosphatidylcholine (14:0_18:2) levels' on 'Malignant melanoma of skin'

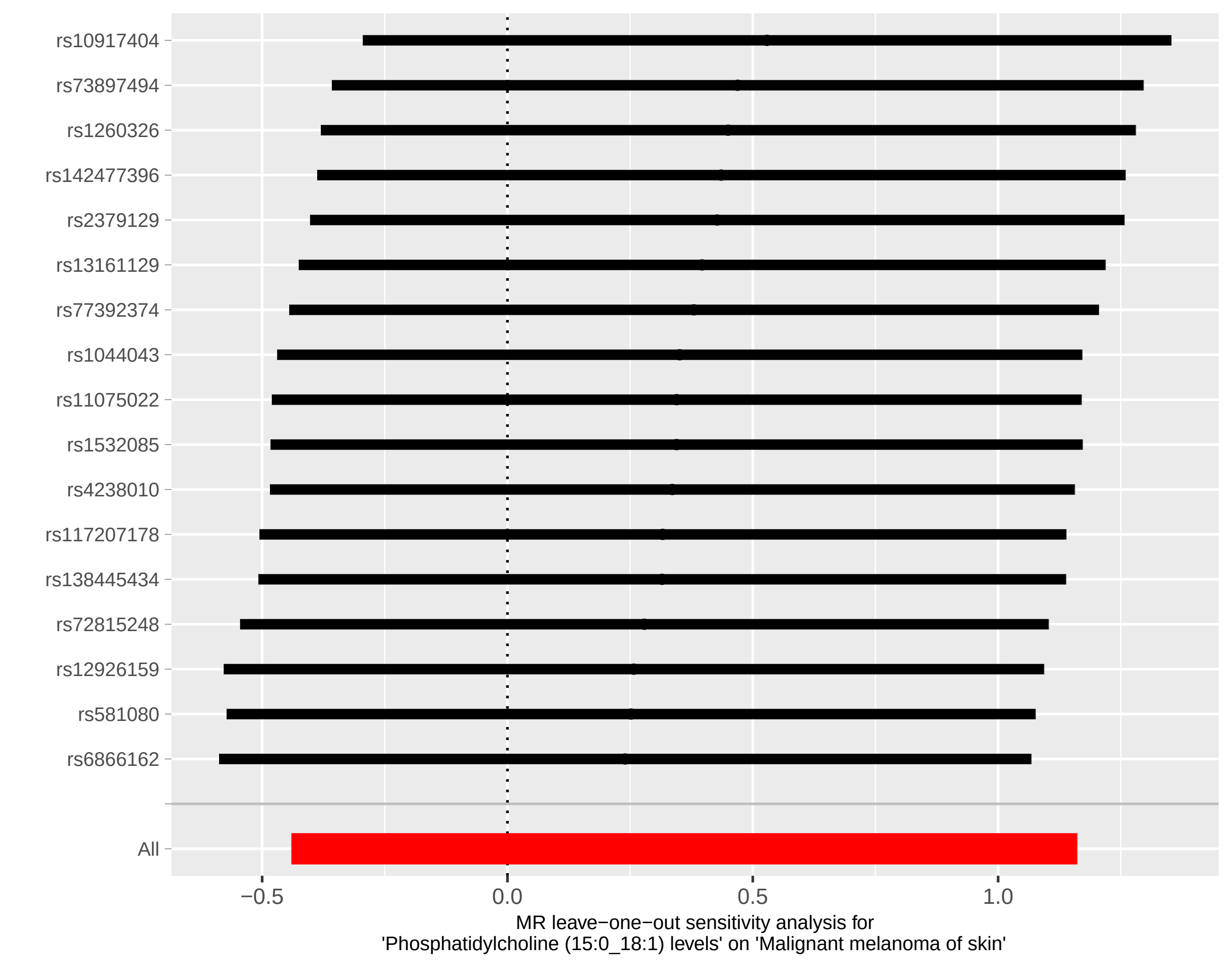
rs10917404
rs73897494
rs1260326
rs142477396
rs2379129
rs13161129
rs77392374
rs1044043
rs11075022
rs1532085
rs4238010
rs117207178
rs138445434
rs72815248
rs12926159
rs581080
rs6866162
All
−0.5
0.0
0.5
1.0
MR leave−one−out sensitivity analysis for
'Phosphatidylcholine (15:0_18:1) levels' on 'Malignant melanoma of skin'

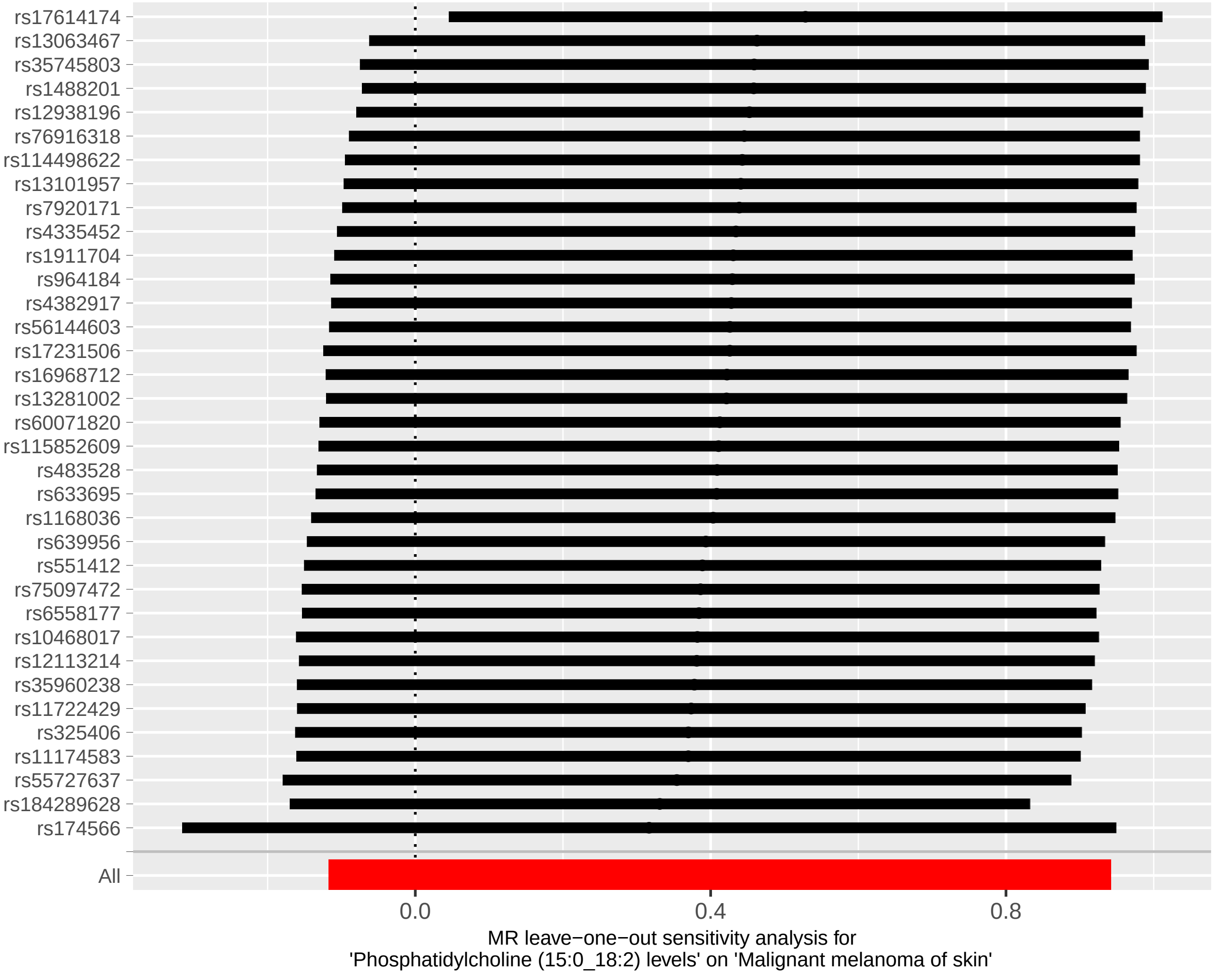
rs17614174
rs13063467
rs35745803
rs1488201
rs12938196
rs76916318
rs114498622
rs13101957
rs7920171
rs4335452
rs1911704
rs964184
rs4382917
rs56144603
rs17231506
rs16968712
rs13281002
rs60071820
rs115852609
rs483528
rs633695
rs1168036
rs639956
rs551412
rs75097472
rs6558177
rs10468017
rs12113214
rs35960238
rs11722429
rs325406
rs11174583
rs55727637
rs184289628
rs174566
All
0.0
0.4
0.8
MR leave−one−out sensitivity analysis for
'Phosphatidylcholine (15:0_18:2) levels' on 'Malignant melanoma of skin'

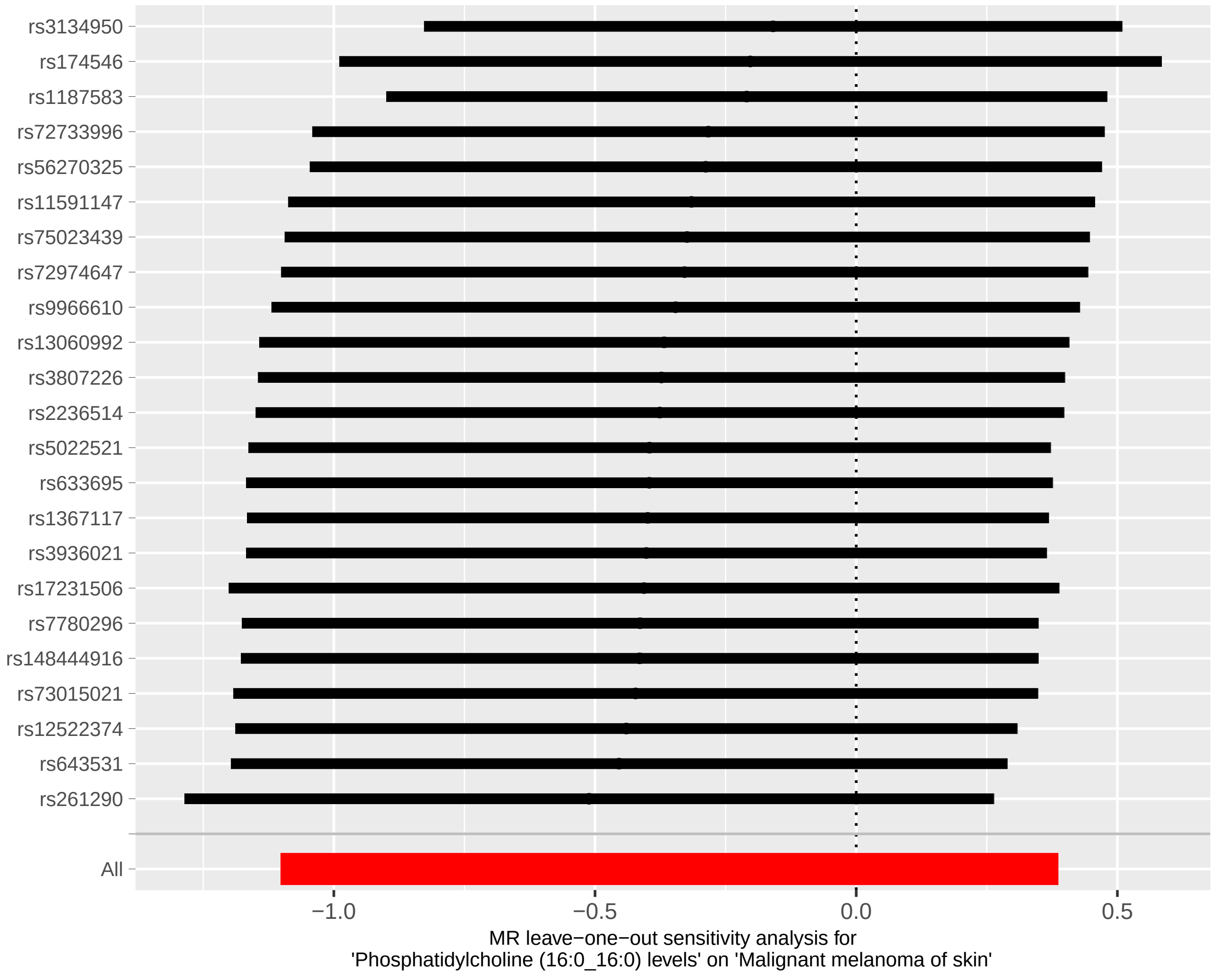
rs3134950
rs174546
rs1187583
rs72733996
rs56270325
rs11591147
rs75023439
rs72974647
rs9966610
rs13060992
rs3807226
rs2236514
rs5022521
rs633695
rs1367117
rs3936021
rs17231506
rs7780296
rs148444916
rs73015021
rs12522374
rs643531
rs261290
All
−1.0
−0.5
0.0
0.5
MR leave−one−out sensitivity analysis for
'Phosphatidylcholine (16:0_16:0) levels' on 'Malignant melanoma of skin'

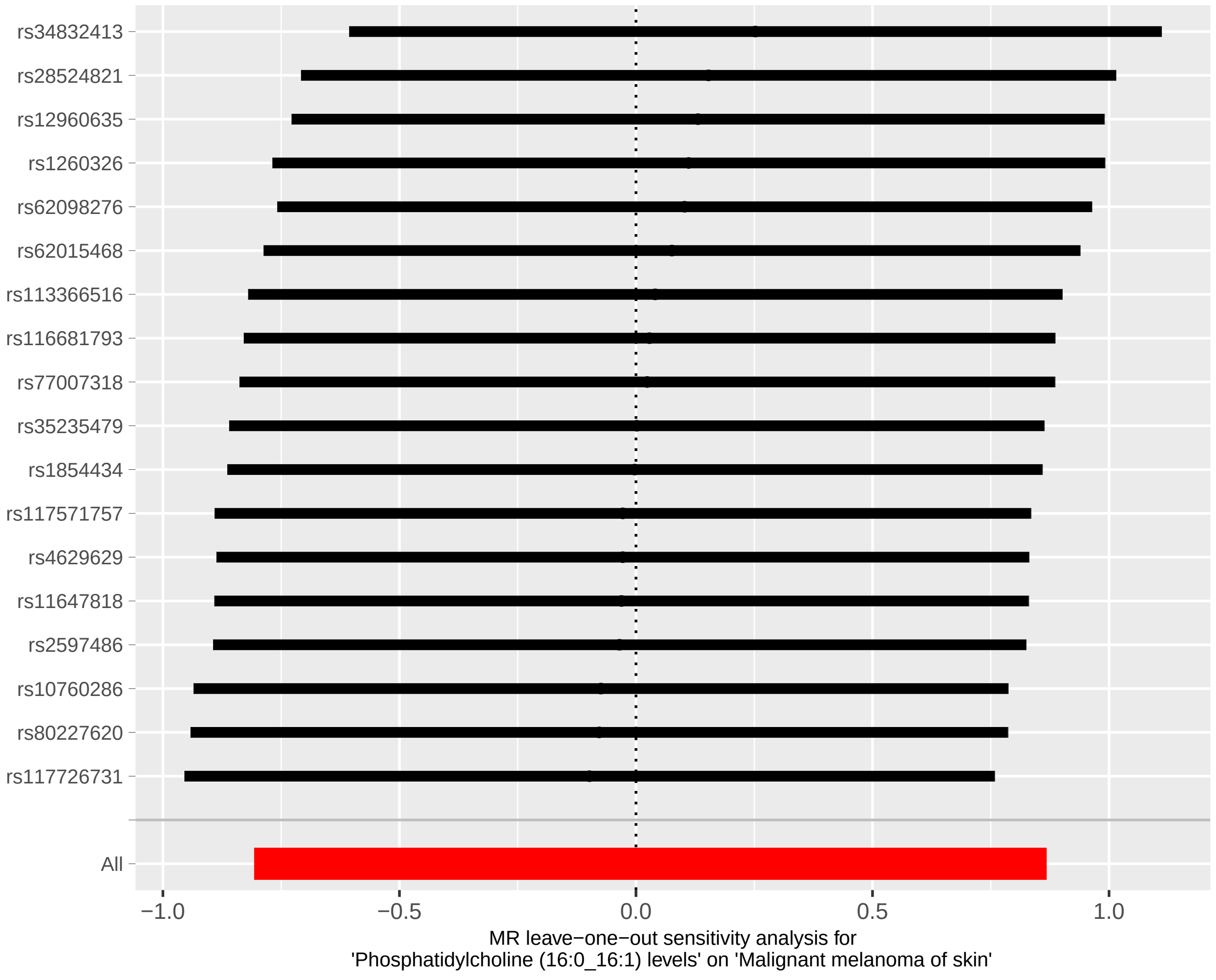

rs34832413
rs28524821
rs12960635
rs1260326
rs62098276
rs62015468
rs113366516
rs116681793
rs77007318
rs35235479
rs1854434
rs117571757
rs4629629
rs11647818
rs2597486
rs10760286
rs80227620
rs117726731
All
−1.0
−0.5
0.0
0.5
1.0
MR leave−one−out sensitivity analysis for
'Phosphatidylcholine (16:0_16:1) levels' on 'Malignant melanoma of skin'

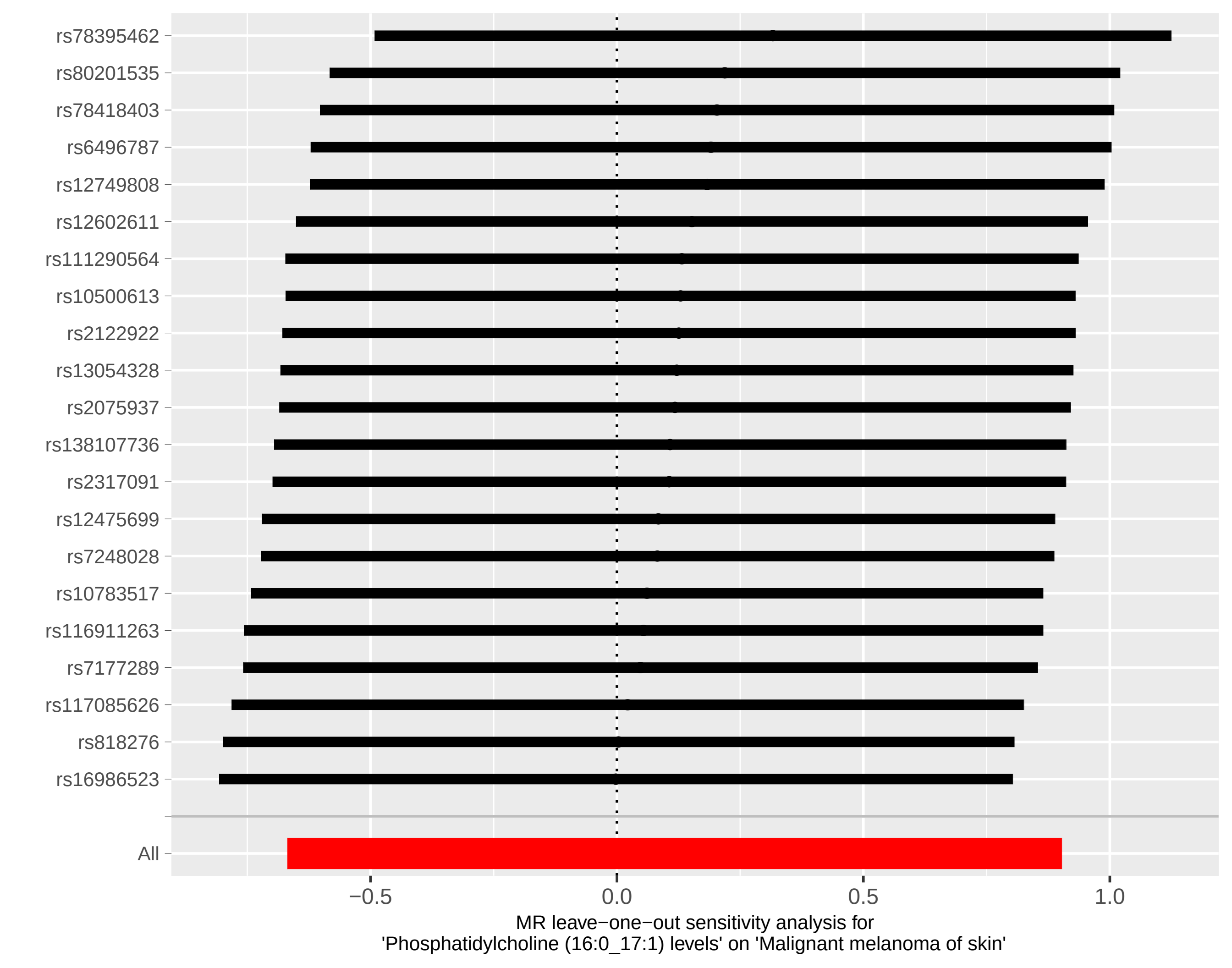
rs78395462
rs80201535
rs78418403
rs6496787
rs12749808
rs12602611
rs111290564
rs10500613
rs2122922
rs13054328
rs2075937
rs138107736
rs2317091
rs12475699
rs7248028
rs10783517
rs116911263
rs7177289
rs117085626
rs818276
rs16986523
All
−0.5
0.0
0.5
1.0
MR leave−one−out sensitivity analysis for
'Phosphatidylcholine (16:0_17:1) levels' on 'Malignant melanoma of skin'

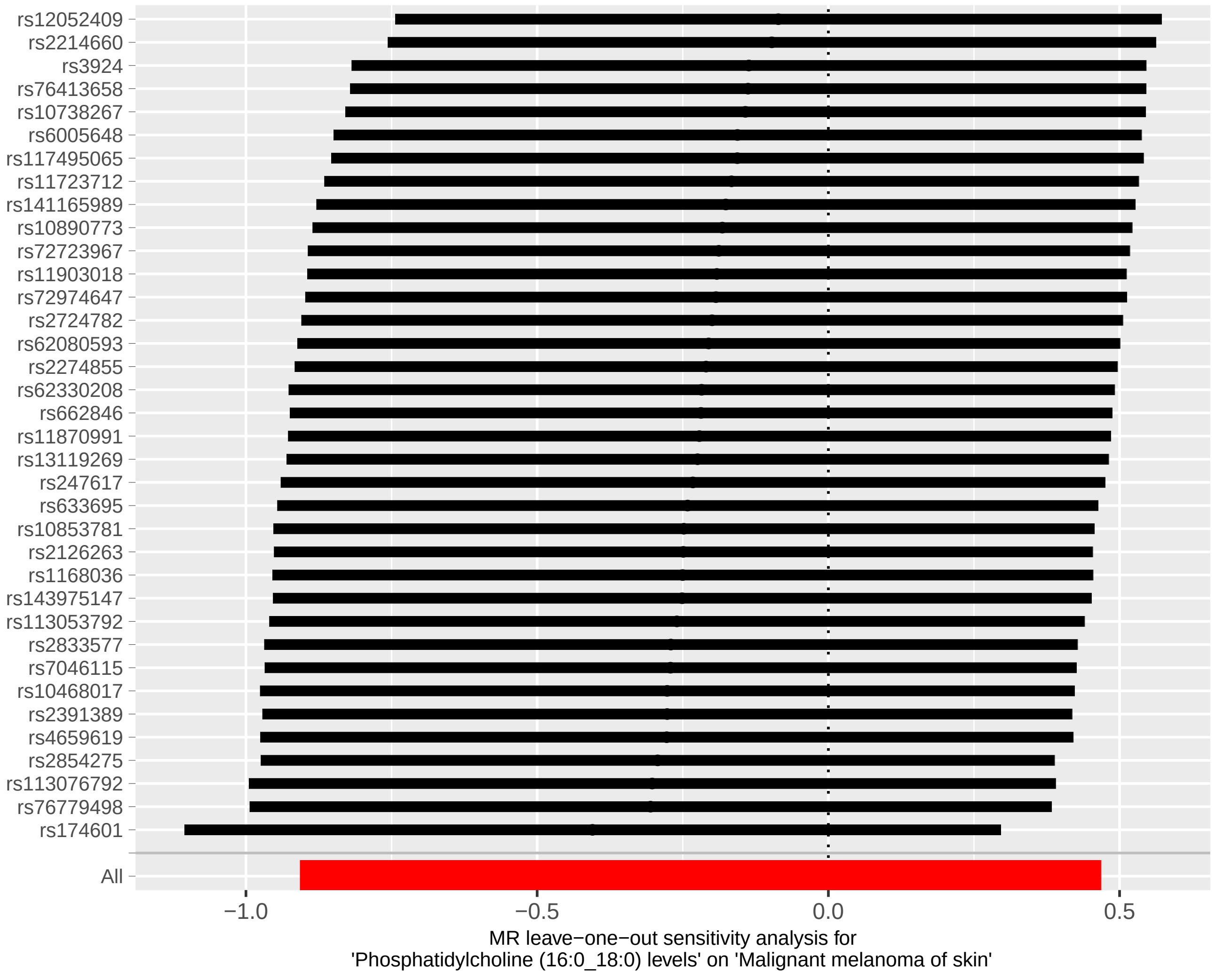
rs12052409
rs2214660
rs3924
rs76413658
rs10738267
rs6005648
rs117495065
rs11723712
rs141165989
rs10890773
rs72723967
rs11903018
rs72974647
rs2724782
rs62080593
rs2274855
rs62330208
rs662846
rs11870991
rs13119269
rs247617
rs633695
rs10853781
rs2126263
rs1168036
rs143975147
rs113053792
rs2833577
rs7046115
rs10468017
rs2391389
rs4659619
rs2854275
rs113076792
rs76779498
rs174601
All
−1.0
−0.5
0.0
0.5
MR leave−one−out sensitivity analysis for
'Phosphatidylcholine (16:0_18:0) levels' on 'Malignant melanoma of skin'

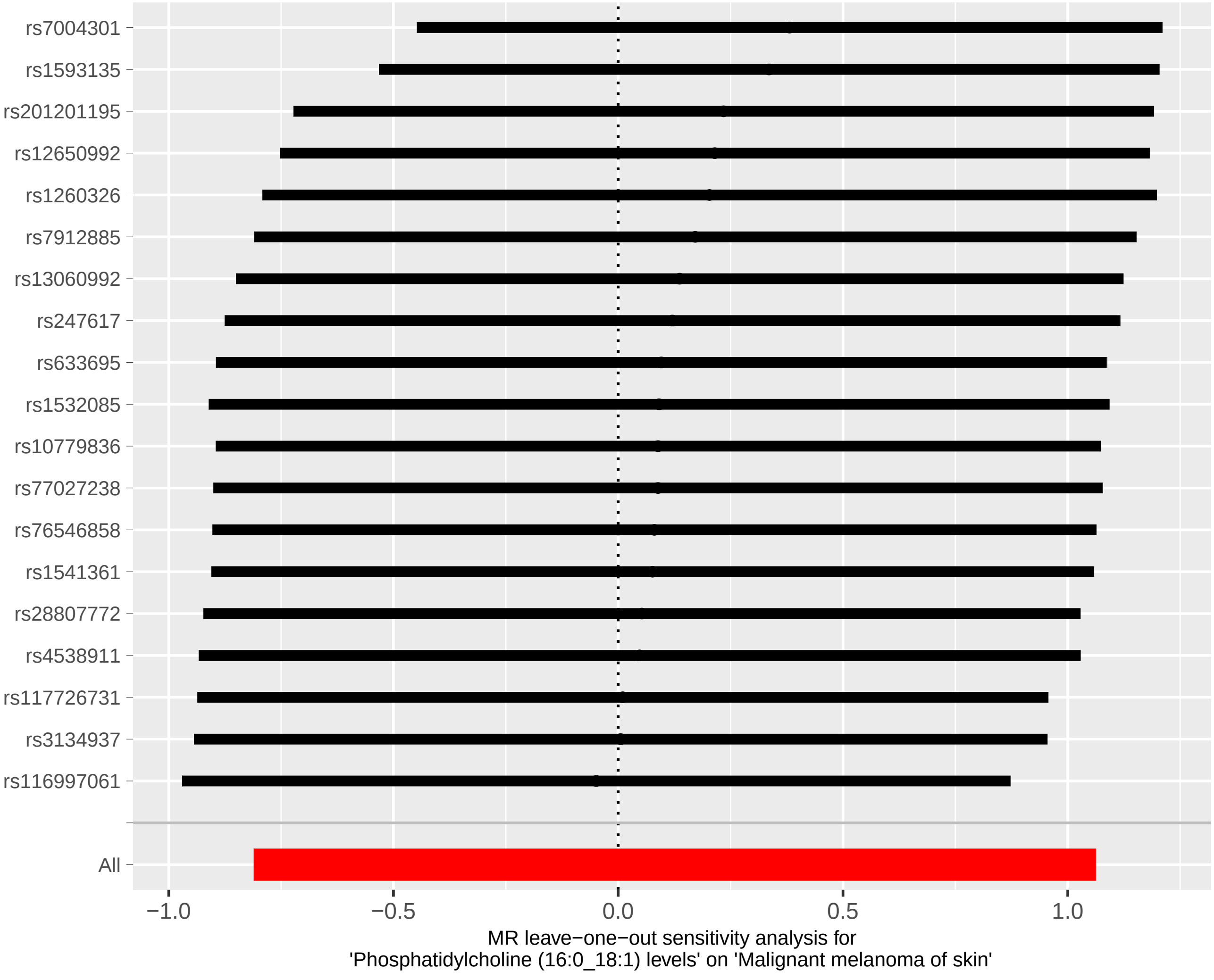

rs7004301
rs1593135
rs201201195
rs12650992
rs1260326
rs7912885
rs13060992
rs247617
rs633695
rs1532085
rs10779836
rs77027238
rs76546858
rs1541361
rs28807772
rs4538911
rs117726731
rs3134937
rs116997061
All
−1.0
−0.5
0.0
0.5
1.0
MR leave−one−out sensitivity analysis for
'Phosphatidylcholine (16:0_18:1) levels' on 'Malignant melanoma of skin'

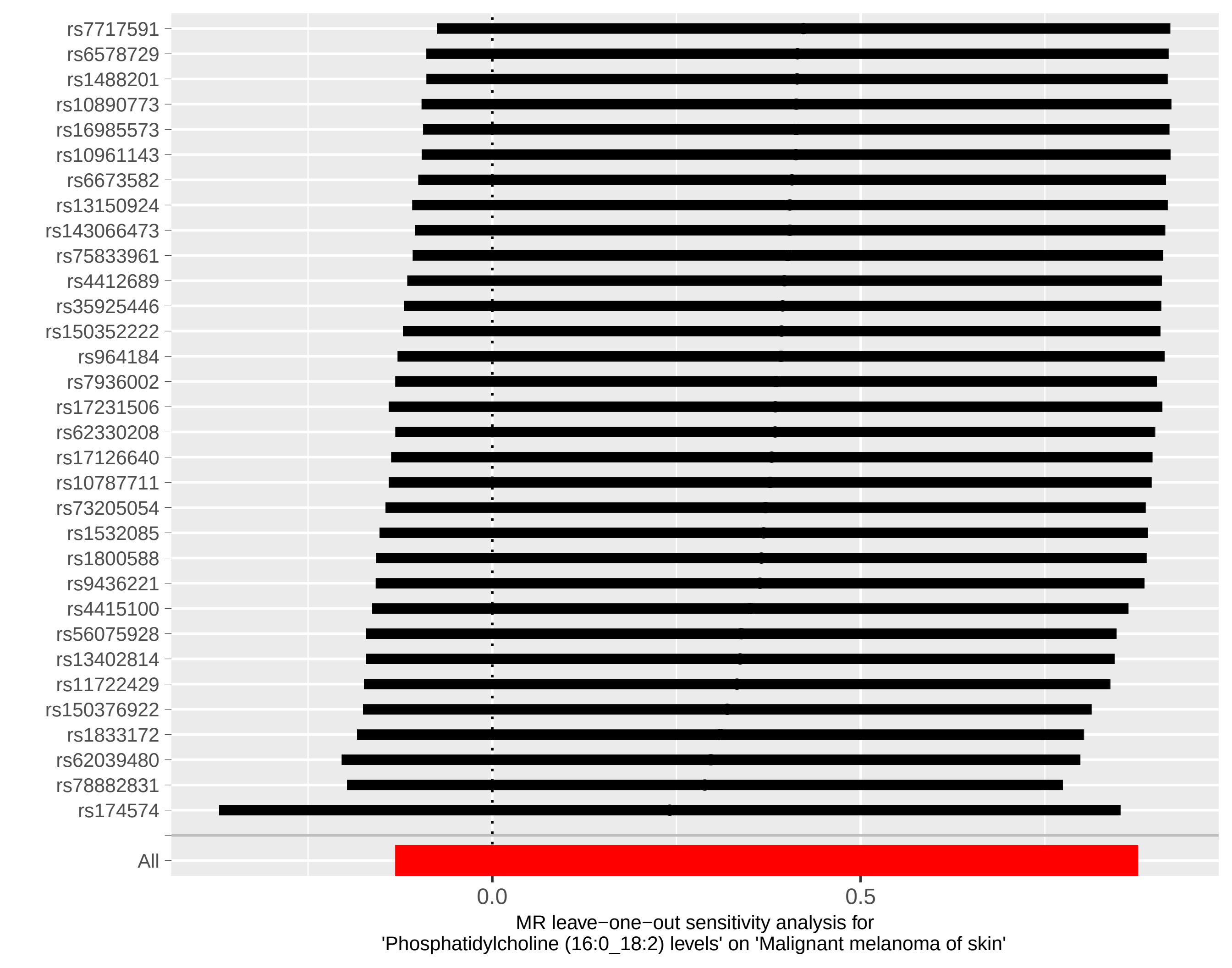
rs7717591
rs6578729
rs1488201
rs10890773
rs16985573
rs10961143
rs6673582
rs13150924
rs143066473
rs75833961
rs4412689
rs35925446
rs150352222
rs964184
rs7936002
rs17231506
rs62330208
rs17126640
rs10787711
rs73205054
rs1532085
rs1800588
rs9436221
rs4415100
rs56075928
rs13402814
rs11722429
rs150376922
rs1833172
rs62039480
rs78882831
rs174574
All
0.0
0.5
MR leave−one−out sensitivity analysis for
'Phosphatidylcholine (16:0_18:2) levels' on 'Malignant melanoma of skin'

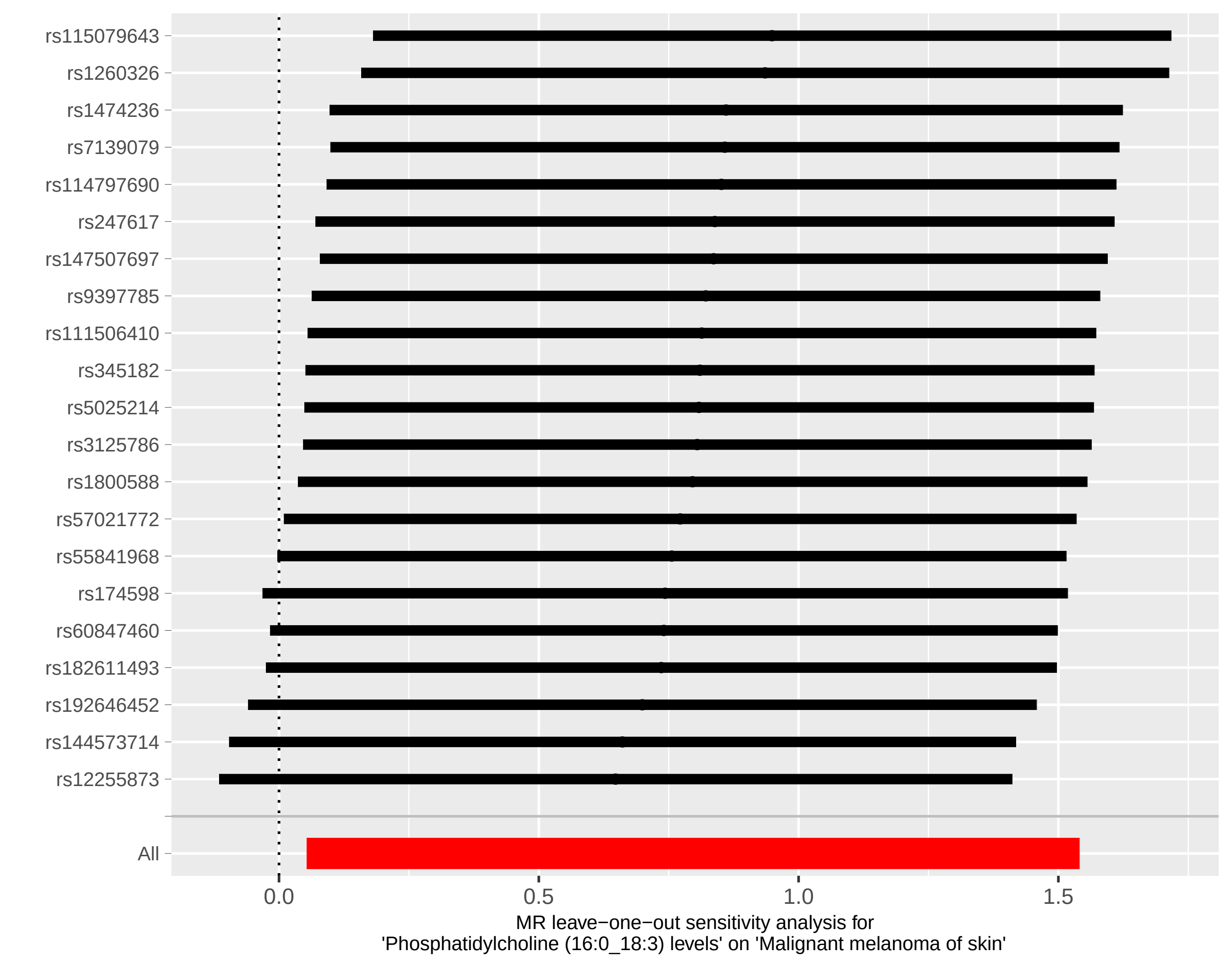
rs115079643
rs1260326
rs1474236
rs7139079
rs114797690
rs247617
rs147507697
rs9397785
rs111506410
rs345182
rs5025214
rs3125786
rs1800588
rs57021772
rs55841968
rs174598
rs60847460
rs182611493
rs192646452
rs144573714
rs12255873
All
0.0
0.5
1.0
1.5
MR leave−one−out sensitivity analysis for
'Phosphatidylcholine (16:0_18:3) levels' on 'Malignant melanoma of skin'

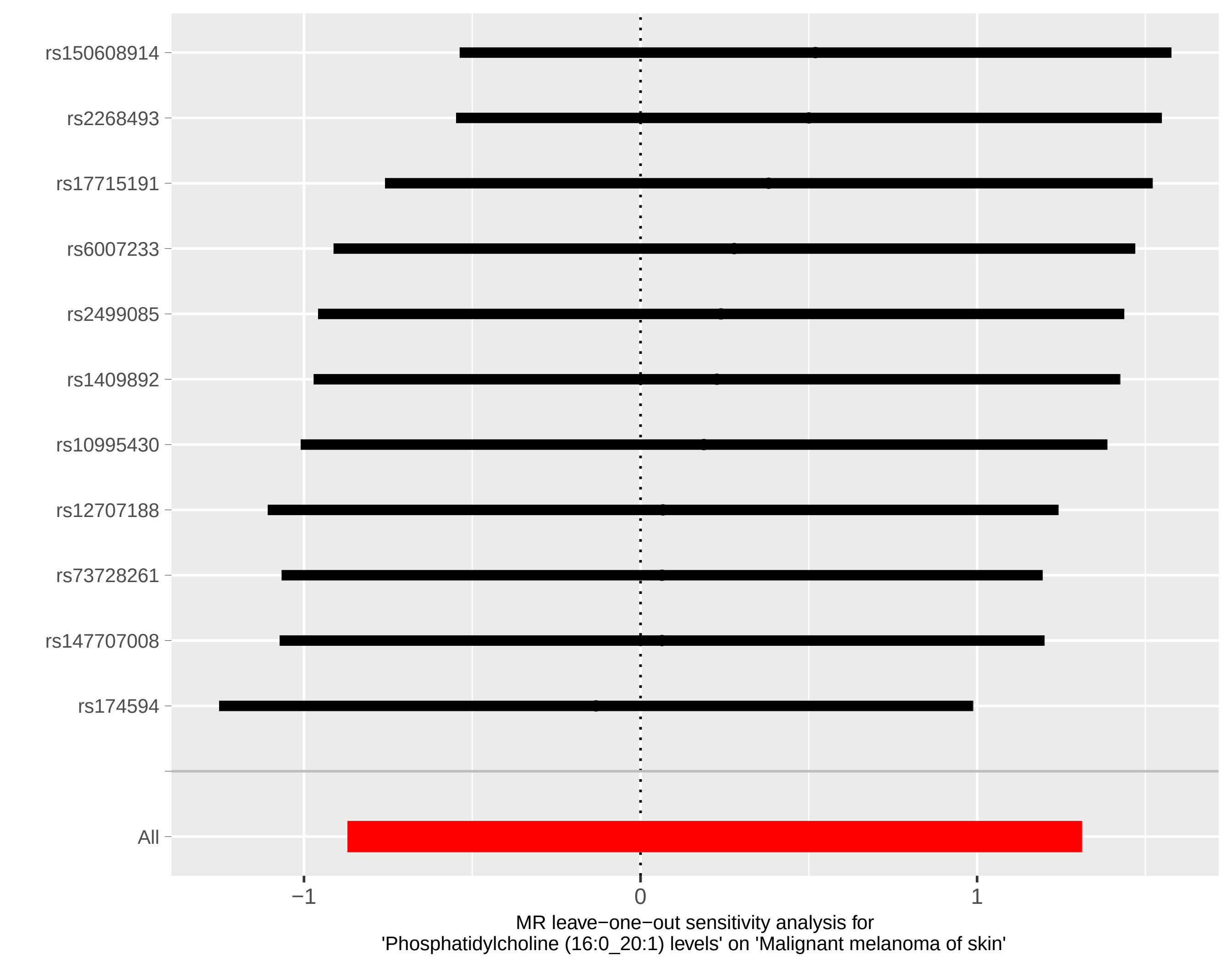
rs150608914
rs2268493
rs17715191
rs6007233
rs2499085
rs1409892
rs10995430
rs12707188
rs73728261
rs147707008
rs174594
All
−1
0
1
MR leave−one−out sensitivity analysis for
'Phosphatidylcholine (16:0_20:1) levels' on 'Malignant melanoma of skin'

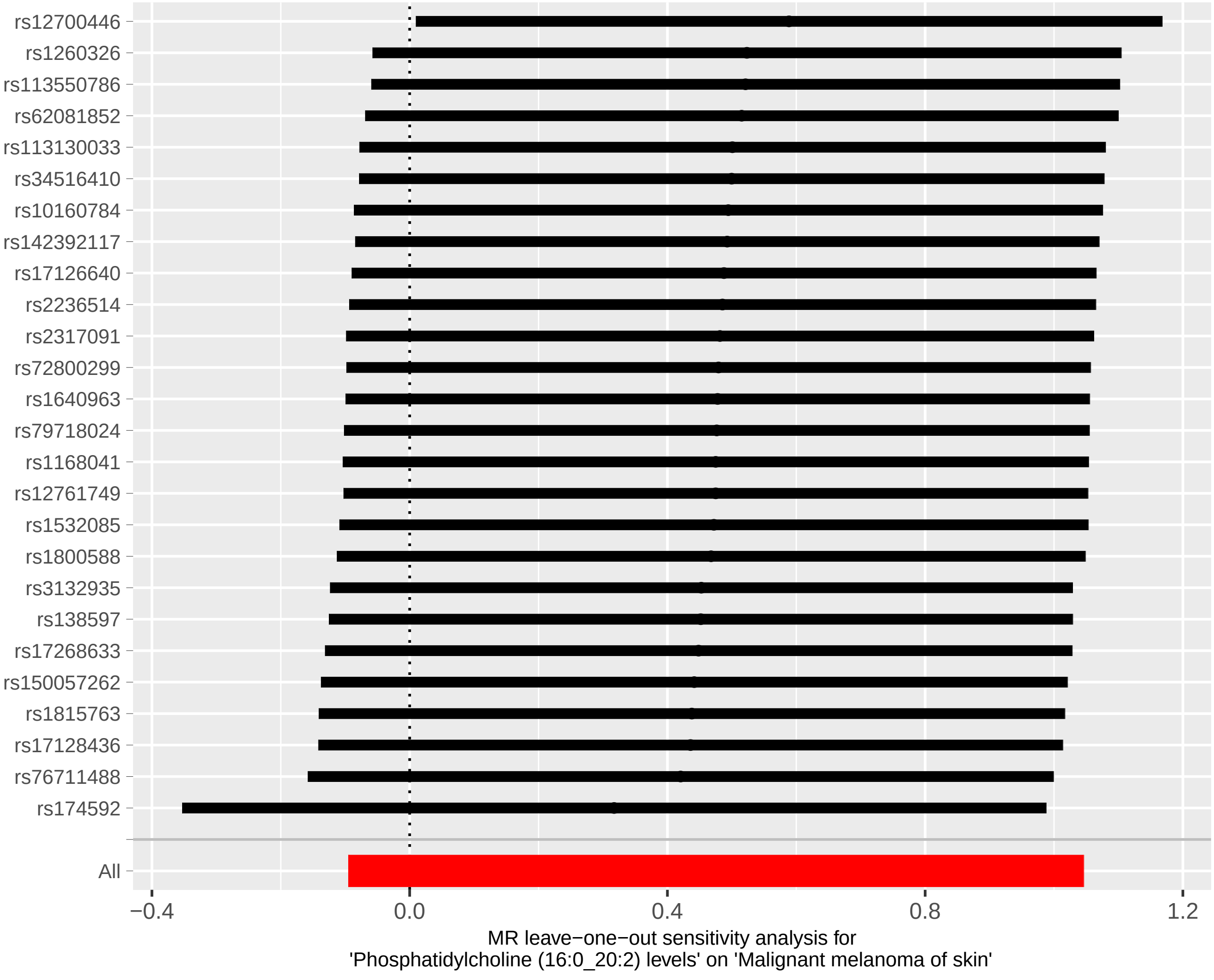
rs12700446
rs1260326
rs113550786
rs62081852
rs113130033
rs34516410
rs10160784
rs142392117
rs17126640
rs2236514
rs2317091
rs72800299
rs1640963
rs79718024
rs1168041
rs12761749
rs1532085
rs1800588
rs3132935
rs138597
rs17268633
rs150057262
rs1815763
rs17128436
rs76711488
rs174592
All
−0.4
0.0
0.4
0.8
1.2
MR leave−one−out sensitivity analysis for
'Phosphatidylcholine (16:0_20:2) levels' on 'Malignant melanoma of skin'

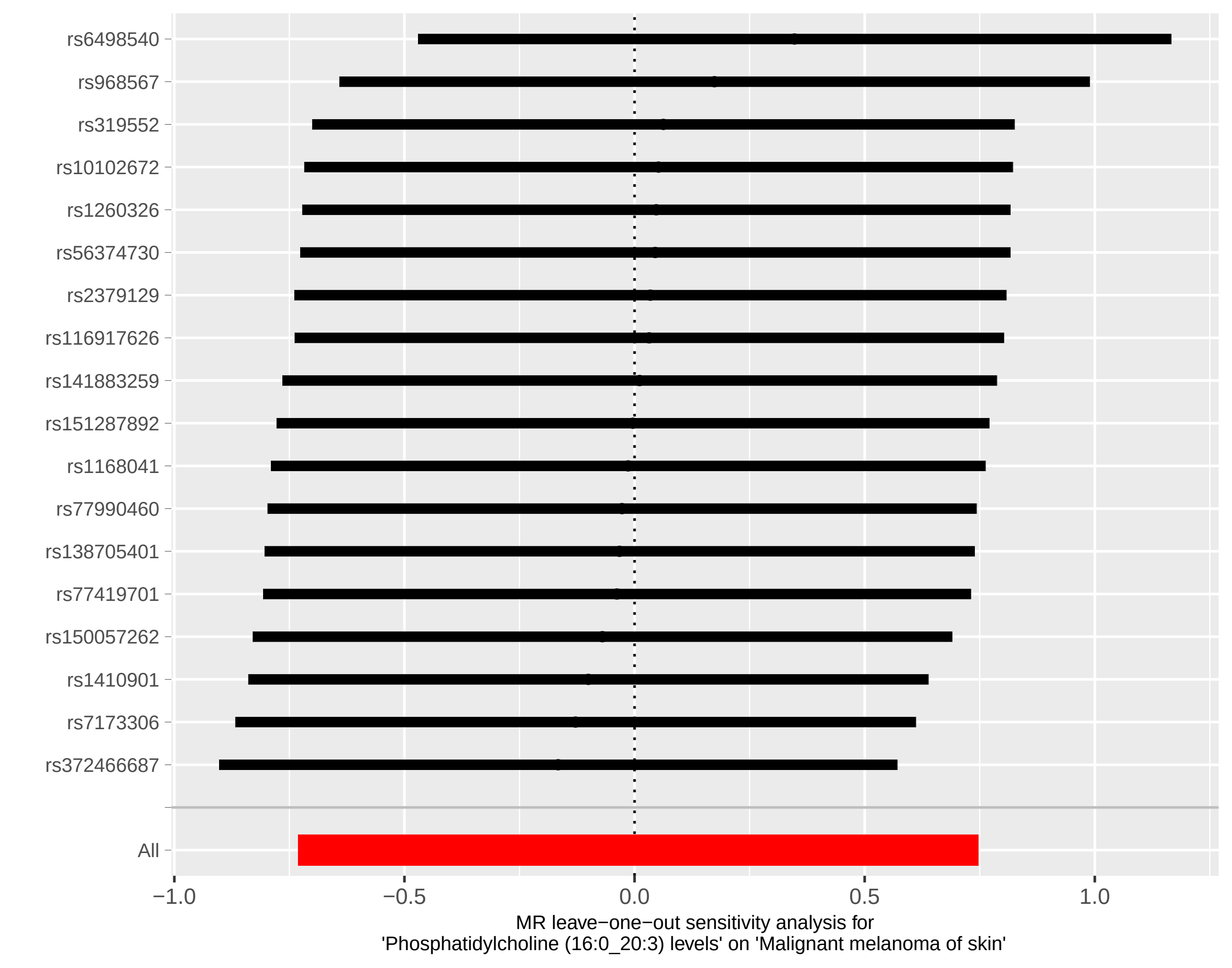
rs6498540
rs968567
rs319552
rs10102672
rs1260326
rs56374730
rs2379129
rs116917626
rs141883259
rs151287892
rs1168041
rs77990460
rs138705401
rs77419701
rs150057262
rs1410901
rs7173306
rs372466687
All
−1.0
−0.5
0.0
0.5
1.0
MR leave−one−out sensitivity analysis for
'Phosphatidylcholine (16:0_20:3) levels' on 'Malignant melanoma of skin'

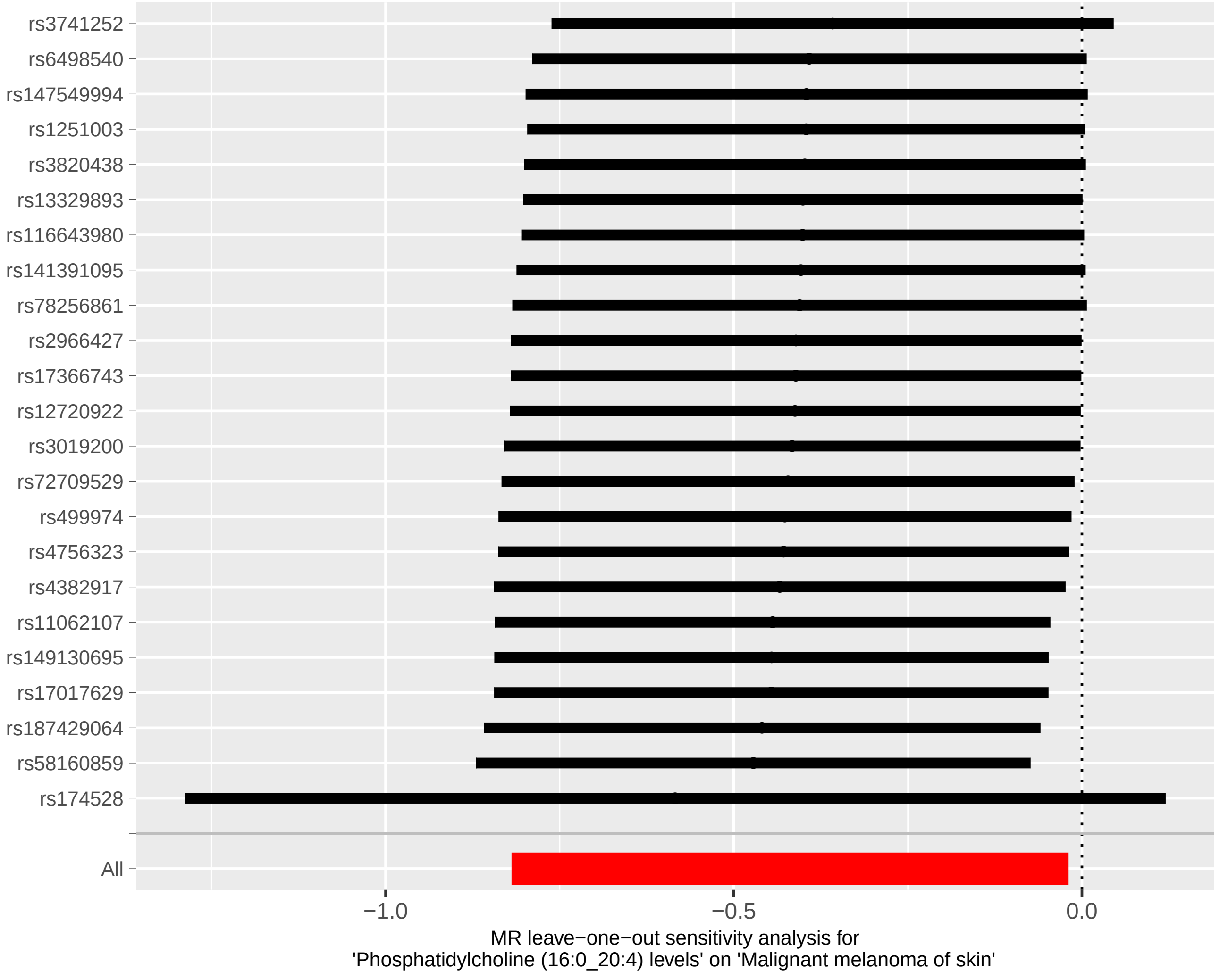
rs3741252
rs6498540
rs147549994
rs1251003
rs3820438
rs13329893
rs116643980
rs141391095
rs78256861
rs2966427
rs17366743
rs12720922
rs3019200
rs72709529
rs499974
rs4756323
rs4382917
rs11062107
rs149130695
rs17017629
rs187429064
rs58160859
rs174528
All
−1.0
−0.5
0.0
MR leave−one−out sensitivity analysis for
'Phosphatidylcholine (16:0_20:4) levels' on 'Malignant melanoma of skin'

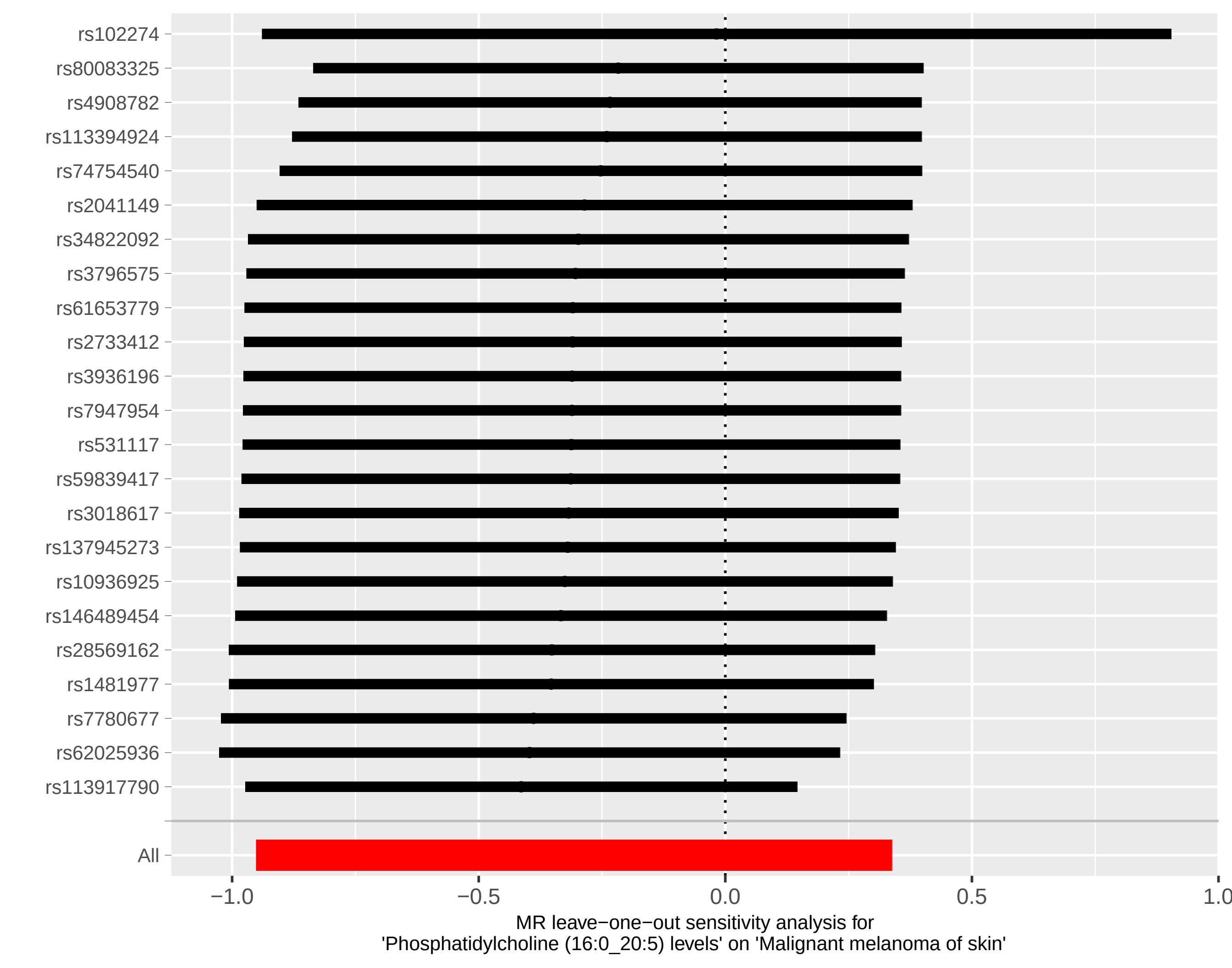
rs102274
rs80083325
rs4908782
rs113394924
rs74754540
rs2041149
rs34822092
rs3796575
rs61653779
rs2733412
rs3936196
rs7947954
rs531117
rs59839417
rs3018617
rs137945273
rs10936925
rs146489454
rs28569162
rs1481977
rs7780677
rs62025936
rs113917790
All
−1.0
−0.5
0.0
0.5
1.0
MR leave−one−out sensitivity analysis for
'Phosphatidylcholine (16:0_20:5) levels' on 'Malignant melanoma of skin'

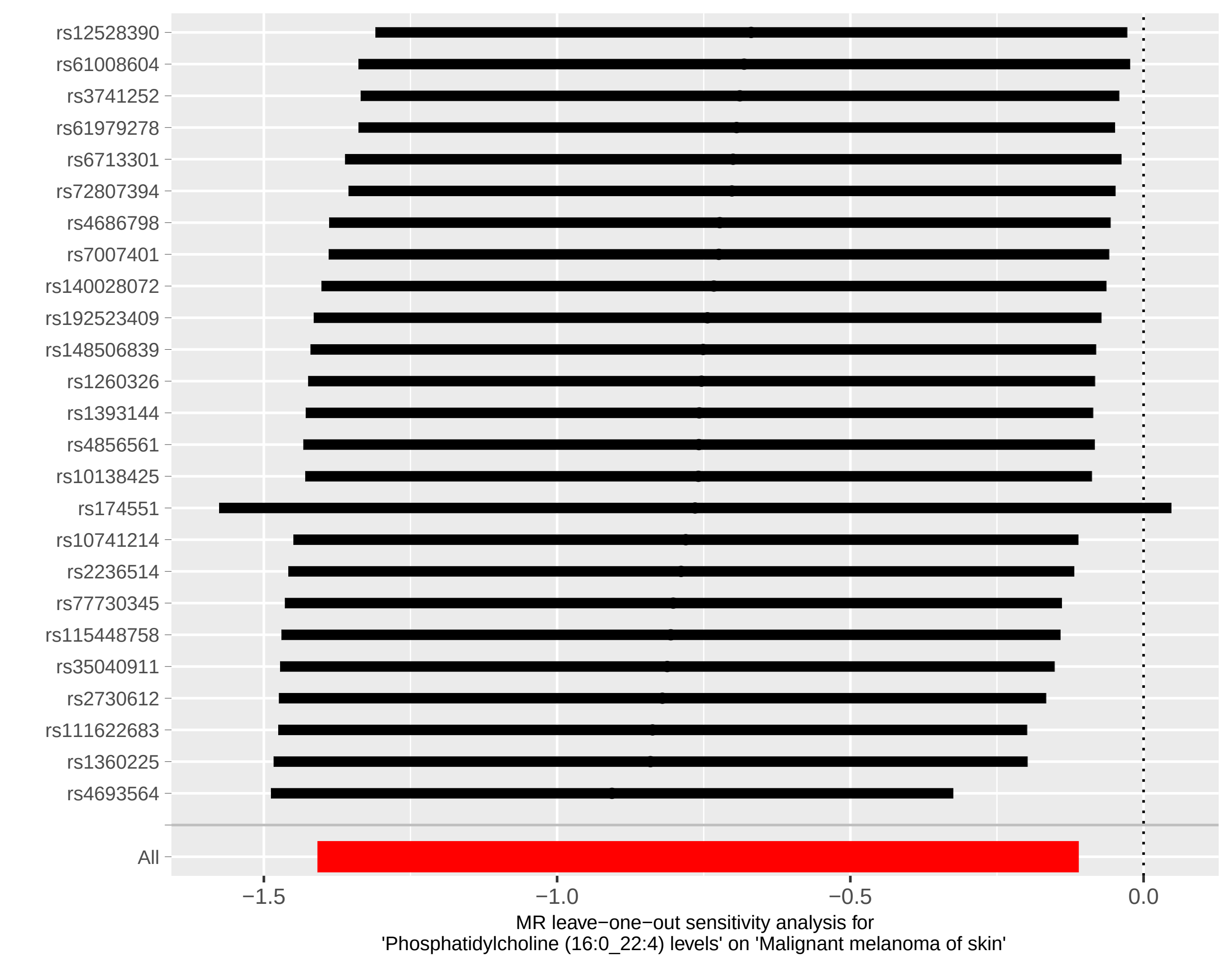
rs12528390
rs61008604
rs3741252
rs61979278
rs6713301
rs72807394
rs4686798
rs7007401
rs140028072
rs192523409
rs148506839
rs1260326
rs1393144
rs4856561
rs10138425
rs174551
rs10741214
rs2236514
rs77730345
rs115448758
rs35040911
rs2730612
rs111622683
rs1360225
rs4693564
All
−1.5
−1.0
−0.5
0.0
MR leave−one−out sensitivity analysis for
'Phosphatidylcholine (16:0_22:4) levels' on 'Malignant melanoma of skin'

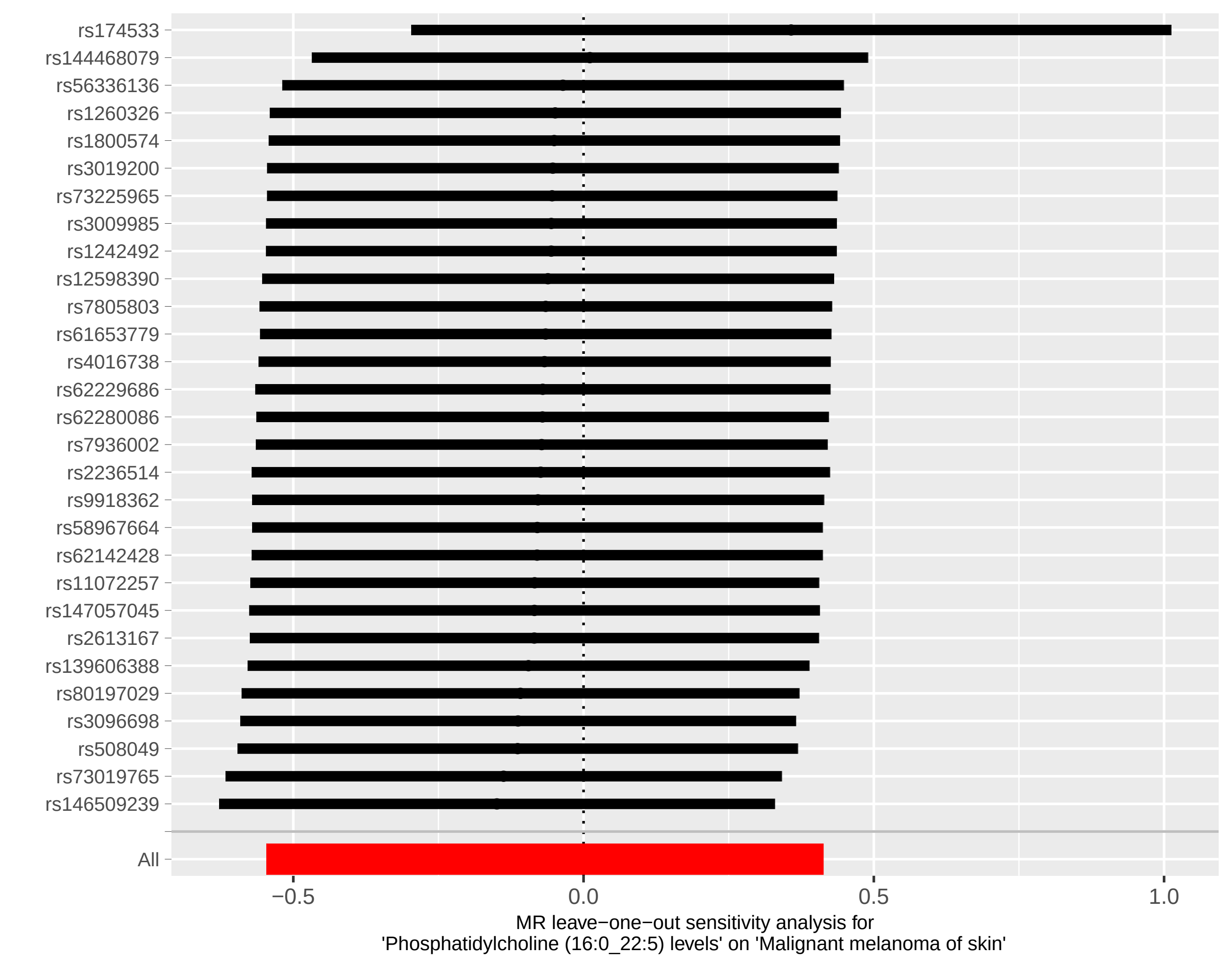
rs174533
rs144468079
rs56336136
rs1260326
rs1800574
rs3019200
rs73225965
rs3009985
rs1242492
rs12598390
rs7805803
rs61653779
rs4016738
rs62229686
rs62280086
rs7936002
rs2236514
rs9918362
rs58967664
rs62142428
rs11072257
rs147057045
rs2613167
rs139606388
rs80197029
rs3096698
rs508049
rs73019765
rs146509239
All
−0.5
0.0
0.5
1.0
MR leave−one−out sensitivity analysis for
'Phosphatidylcholine (16:0_22:5) levels' on 'Malignant melanoma of skin'

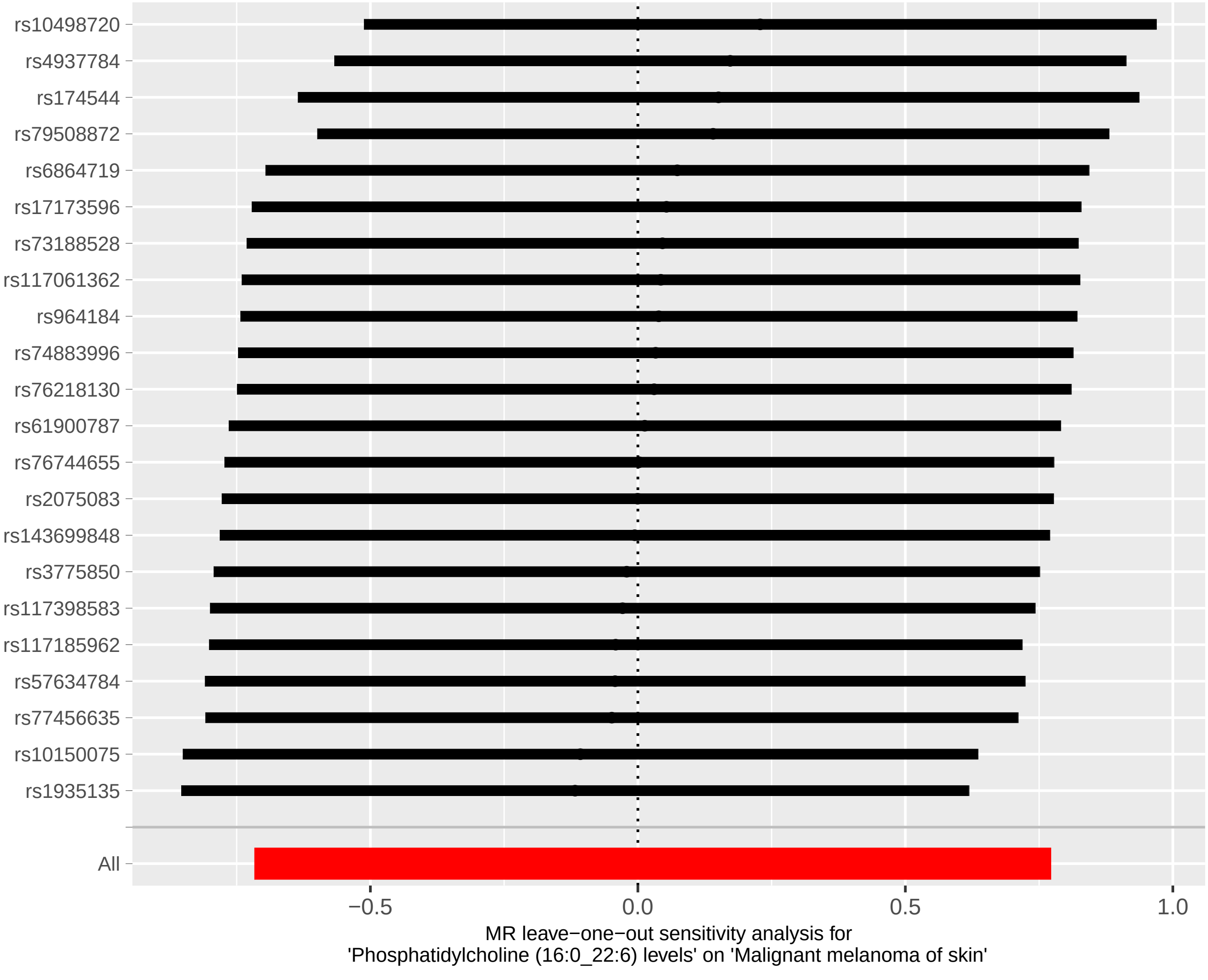
rs10498720
rs4937784
rs174544
rs79508872
rs6864719
rs17173596
rs73188528
rs117061362
rs964184
rs74883996
rs76218130
rs61900787
rs76744655
rs2075083
rs143699848
rs3775850
rs117398583
rs117185962
rs57634784
rs77456635
rs10150075
rs1935135
All
−0.5
0.0
0.5
1.0
MR leave−one−out sensitivity analysis for
'Phosphatidylcholine (16:0_22:6) levels' on 'Malignant melanoma of skin'

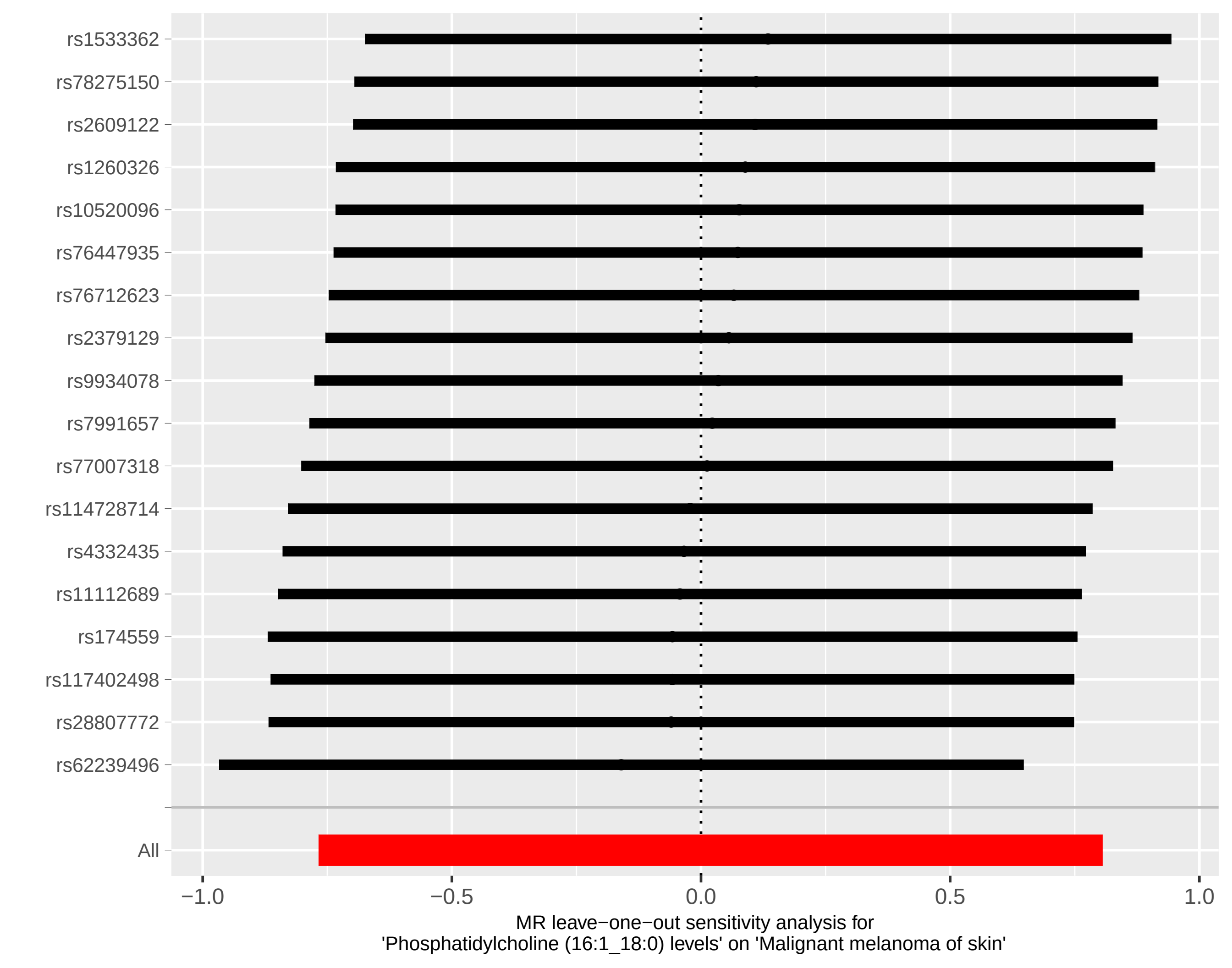
rs1533362
rs78275150
rs2609122
rs1260326
rs10520096
rs76447935
rs76712623
rs2379129
rs9934078
rs7991657
rs77007318
rs114728714
rs4332435
rs11112689
rs174559
rs117402498
rs28807772
rs62239496
All
−1.0
−0.5
0.0
0.5
1.0
MR leave−one−out sensitivity analysis for
'Phosphatidylcholine (16:1_18:0) levels' on 'Malignant melanoma of skin'

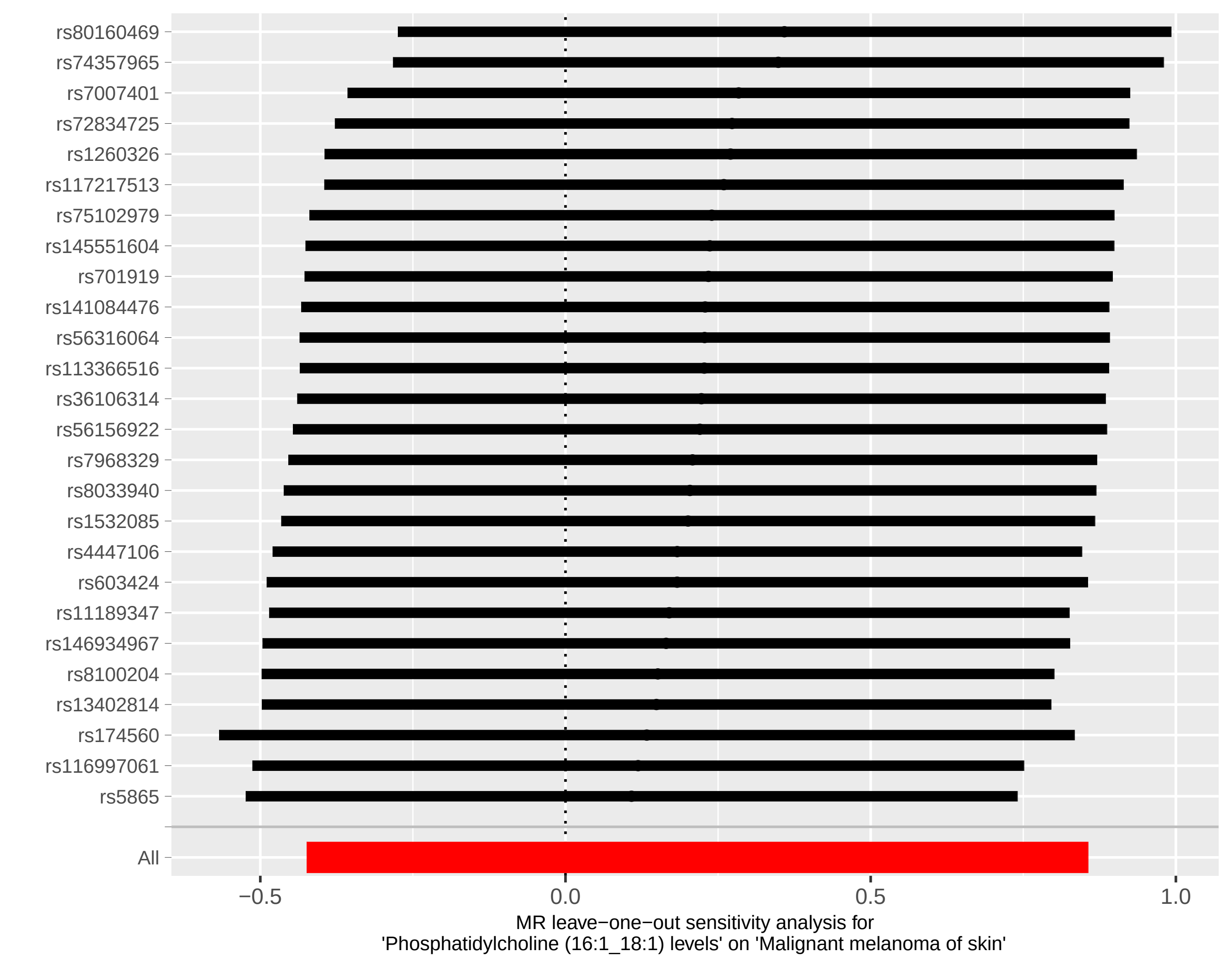

rs80160469
rs74357965
rs7007401
rs72834725
rs1260326
rs117217513
rs75102979
rs145551604
rs701919
rs141084476
rs56316064
rs113366516
rs36106314
rs56156922
rs7968329
rs8033940
rs1532085
rs4447106
rs603424
rs11189347
rs146934967
rs8100204
rs13402814
rs174560
rs116997061
rs5865
All
−0.5
0.0
0.5
1.0
MR leave−one−out sensitivity analysis for
'Phosphatidylcholine (16:1_18:1) levels' on 'Malignant melanoma of skin'

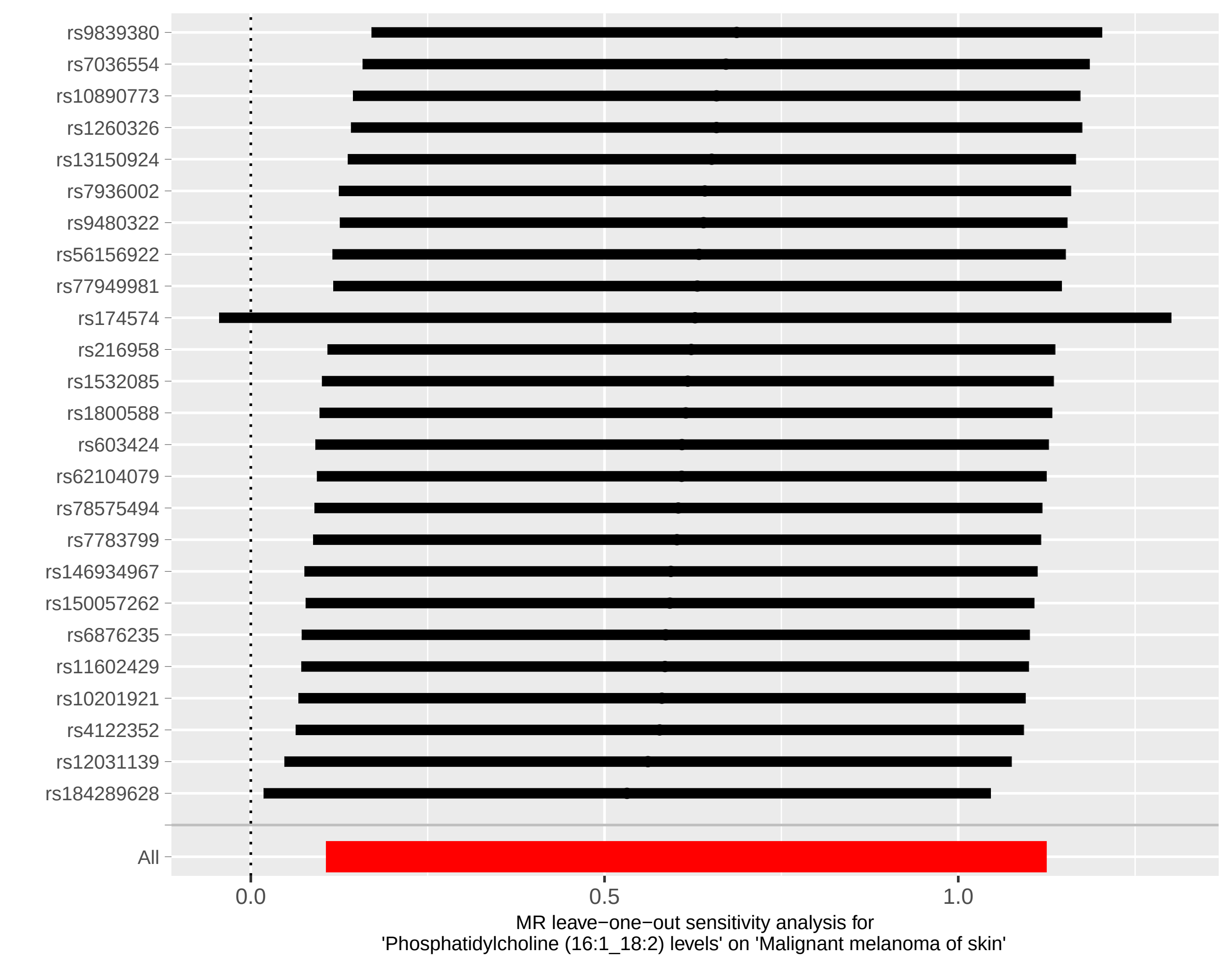

rs9839380
rs7036554
rs10890773
rs1260326
rs13150924
rs7936002
rs9480322
rs56156922
rs77949981
rs174574
rs216958
rs1532085
rs1800588
rs603424
rs62104079
rs78575494
rs7783799
rs146934967
rs150057262
rs6876235
rs11602429
rs10201921
rs4122352
rs12031139
rs184289628
All
0.0
0.5
1.0
MR leave−one−out sensitivity analysis for
'Phosphatidylcholine (16:1_18:2) levels' on 'Malignant melanoma of skin'

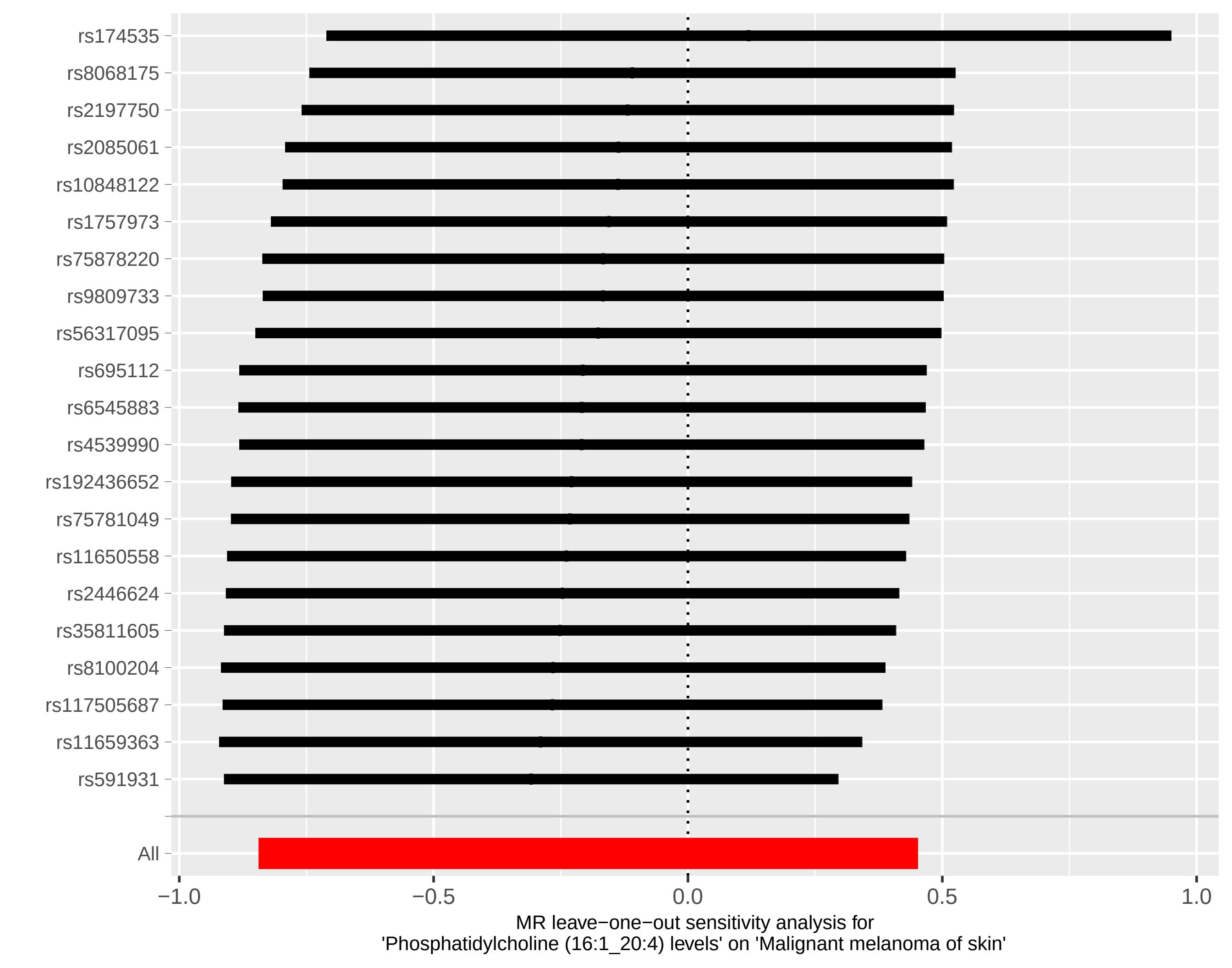

rs174535
rs8068175
rs2197750
rs2085061
rs10848122
rs1757973
rs75878220
rs9809733
rs56317095
rs695112
rs6545883
rs4539990
rs192436652
rs75781049
rs11650558
rs2446624
rs35811605
rs8100204
rs117505687
rs11659363
rs591931
All
−1.0
−0.5
0.0
0.5
1.0
MR leave−one−out sensitivity analysis for
'Phosphatidylcholine (16:1_20:4) levels' on 'Malignant melanoma of skin'

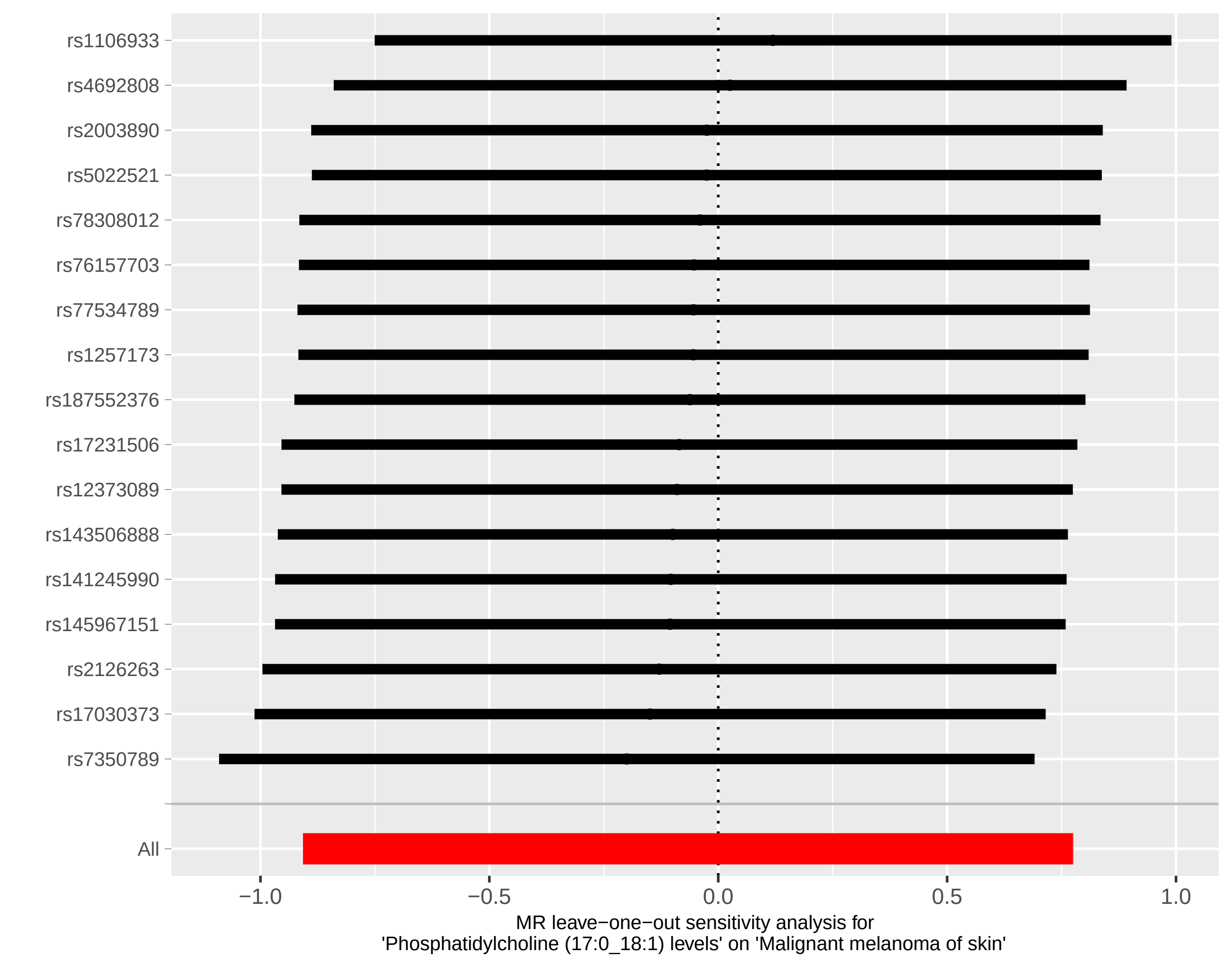
rs1106933
rs4692808
rs2003890
rs5022521
rs78308012
rs76157703
rs77534789
rs1257173
rs187552376
rs17231506
rs12373089
rs143506888
rs141245990
rs145967151
rs2126263
rs17030373
rs7350789
All
−1.0
−0.5
0.0
0.5
1.0
MR leave−one−out sensitivity analysis for
'Phosphatidylcholine (17:0_18:1) levels' on 'Malignant melanoma of skin'

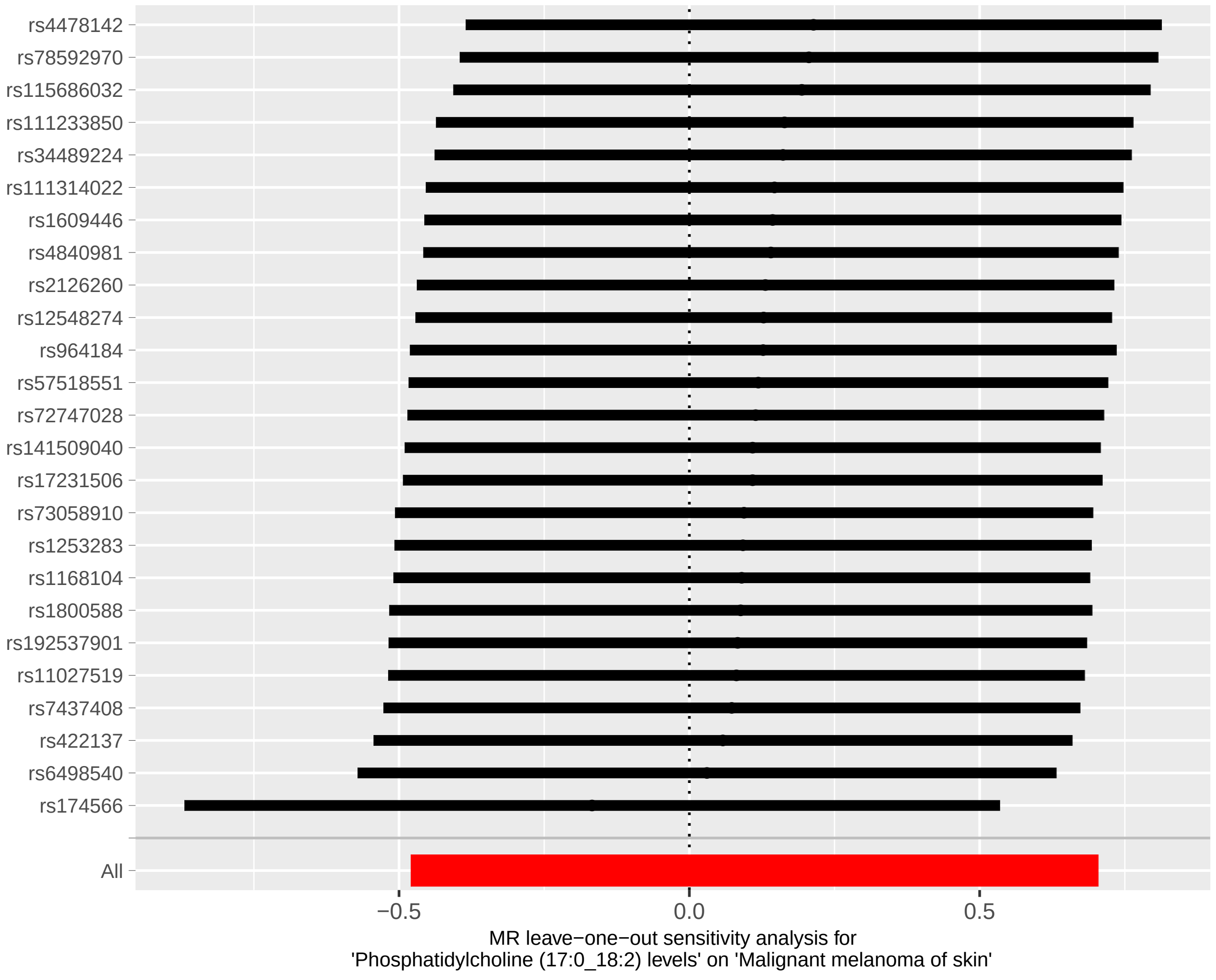

rs4478142
rs78592970
rs115686032
rs111233850
rs34489224
rs111314022
rs1609446
rs4840981
rs2126260
rs12548274
rs964184
rs57518551
rs72747028
rs141509040
rs17231506
rs73058910
rs1253283
rs1168104
rs1800588
rs192537901
rs11027519
rs7437408
rs422137
rs6498540
rs174566
All
−0.5
0.0
0.5
MR leave−one−out sensitivity analysis for
'Phosphatidylcholine (17:0_18:2) levels' on 'Malignant melanoma of skin'

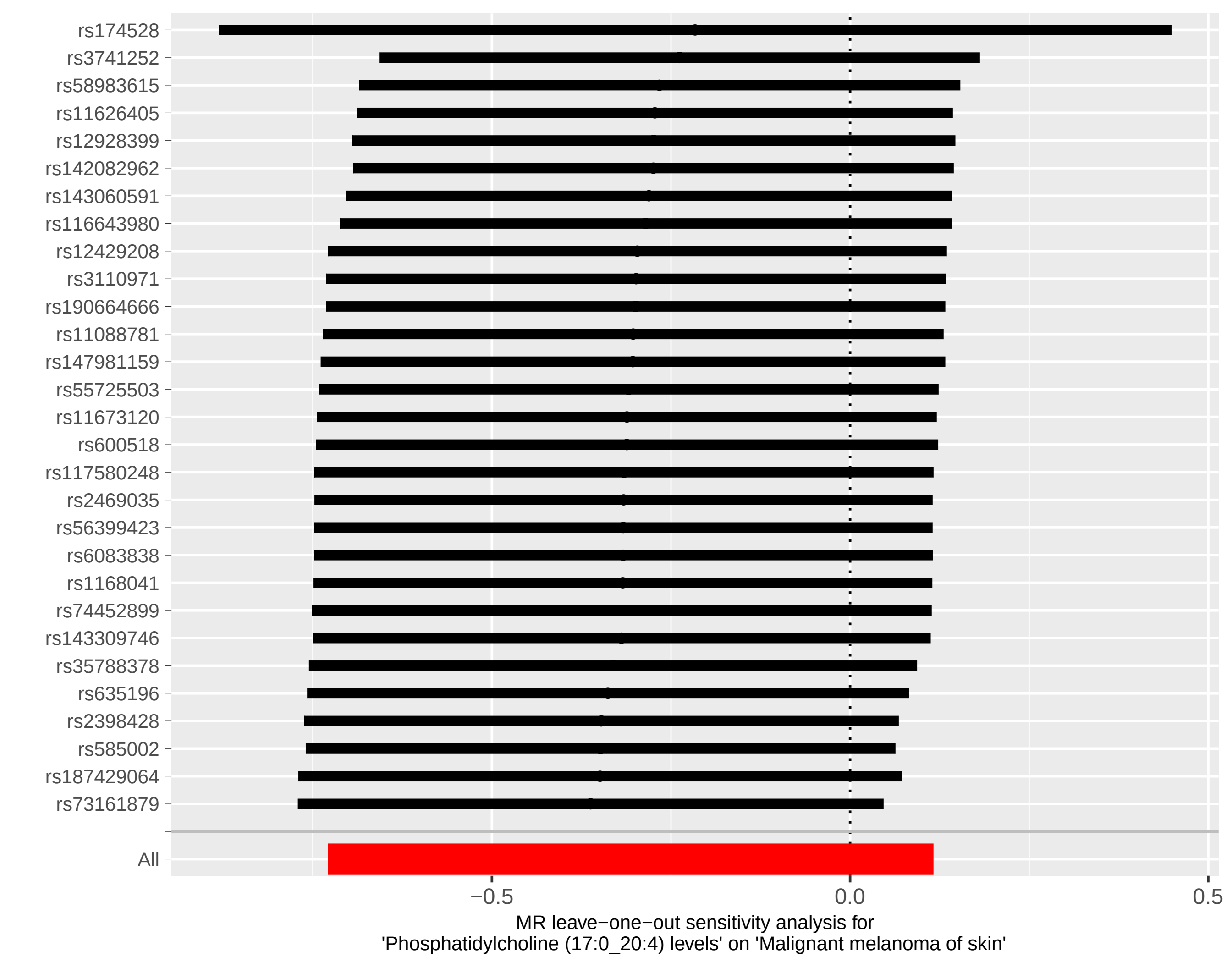
rs174528
rs3741252
rs58983615
rs11626405
rs12928399
rs142082962
rs143060591
rs116643980
rs12429208
rs3110971
rs190664666
rs11088781
rs147981159
rs55725503
rs11673120
rs600518
rs117580248
rs2469035
rs56399423
rs6083838
rs1168041
rs74452899
rs143309746
rs35788378
rs635196
rs2398428
rs585002
rs187429064
rs73161879
All
−0.5
0.0
0.5
MR leave−one−out sensitivity analysis for
'Phosphatidylcholine (17:0_20:4) levels' on 'Malignant melanoma of skin'

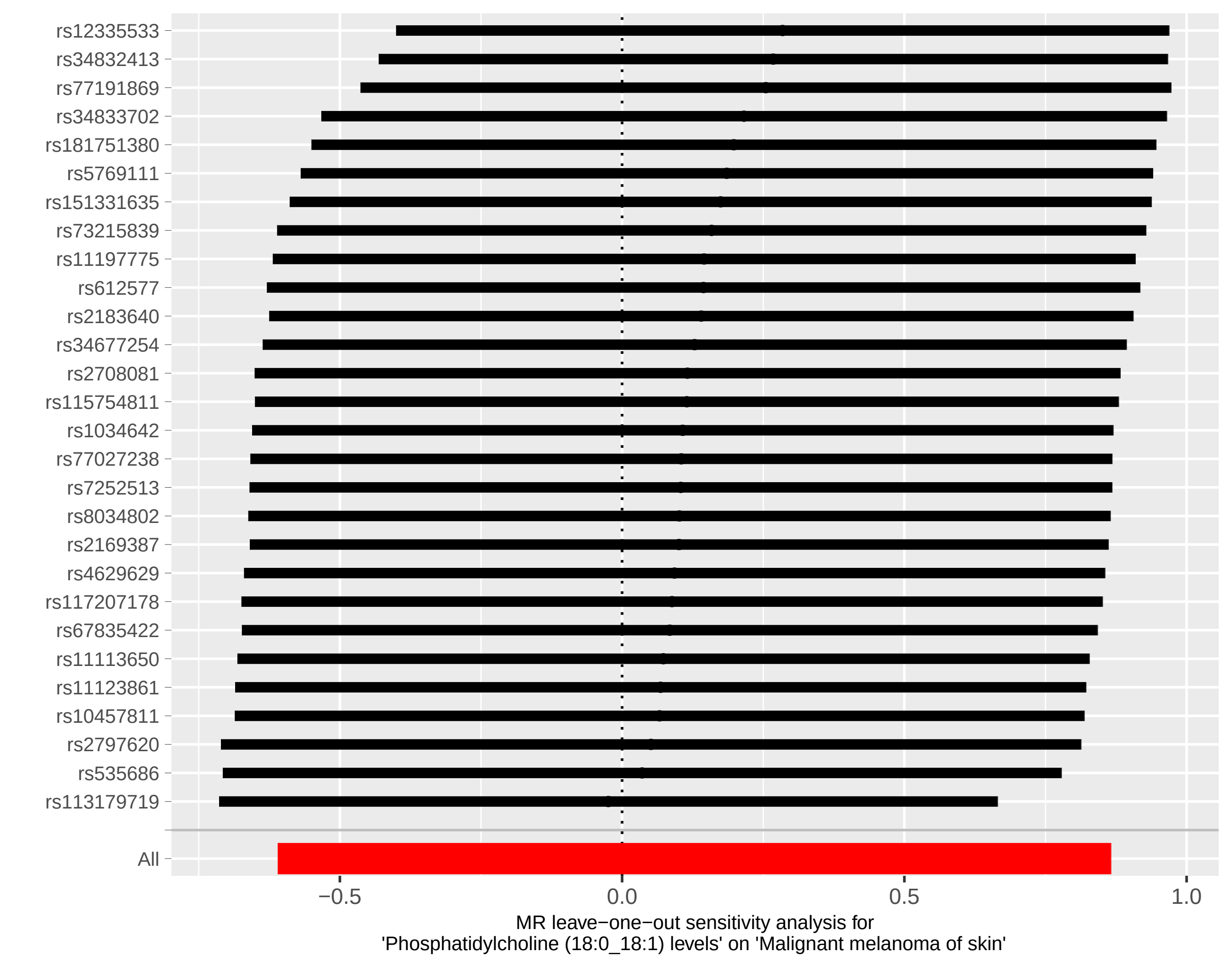
rs12335533
rs34832413
rs77191869
rs34833702
rs181751380
rs5769111
rs151331635
rs73215839
rs11197775
rs612577
rs2183640
rs34677254
rs2708081
rs115754811
rs1034642
rs77027238
rs7252513
rs8034802
rs2169387
rs4629629
rs117207178
rs67835422
rs11113650
rs11123861
rs10457811
rs2797620
rs535686
rs113179719
All
−0.5
0.0
0.5
1.0
MR leave−one−out sensitivity analysis for
'Phosphatidylcholine (18:0_18:1) levels' on 'Malignant melanoma of skin'

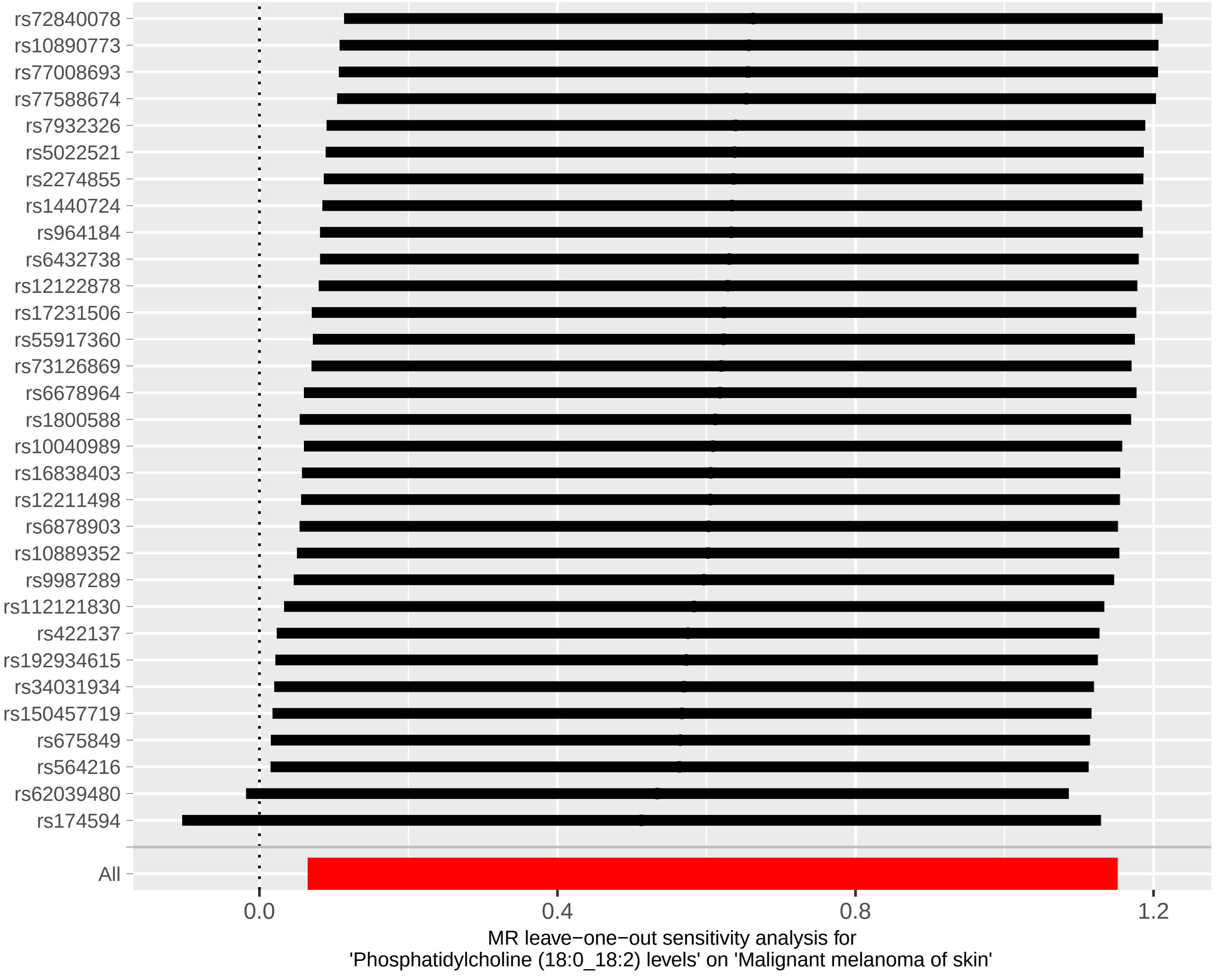
rs72840078
rs10890773
rs77008693
rs77588674
rs7932326
rs5022521
rs2274855
rs1440724
rs964184
rs6432738
rs12122878
rs17231506
rs55917360
rs73126869
rs6678964
rs1800588
rs10040989
rs16838403
rs12211498
rs6878903
rs10889352
rs9987289
rs112121830
rs422137
rs192934615
rs34031934
rs150457719
rs675849
rs564216
rs62039480
rs174594
All
0.0
0.4
0.8
1.2
MR leave−one−out sensitivity analysis for
'Phosphatidylcholine (18:0_18:2) levels' on 'Malignant melanoma of skin'

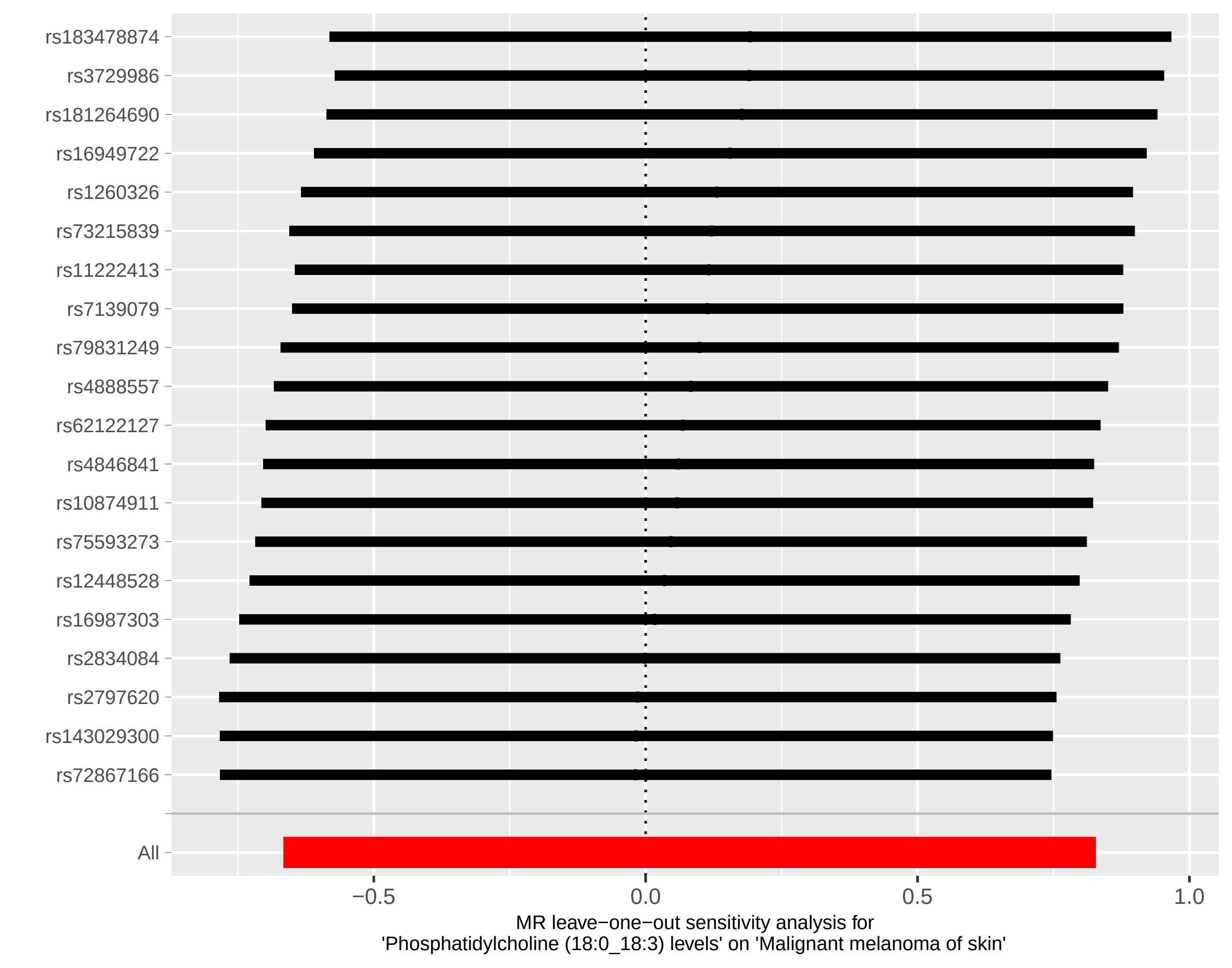
rs183478874
rs3729986
rs181264690
rs16949722
rs1260326
rs73215839
rs11222413
rs7139079
rs79831249
rs4888557
rs62122127
rs4846841
rs10874911
rs75593273
rs12448528
rs16987303
rs2834084
rs2797620
rs143029300
rs72867166
All
−0.5
0.0
0.5
1.0
MR leave−one−out sensitivity analysis for
'Phosphatidylcholine (18:0_18:3) levels' on 'Malignant melanoma of skin'

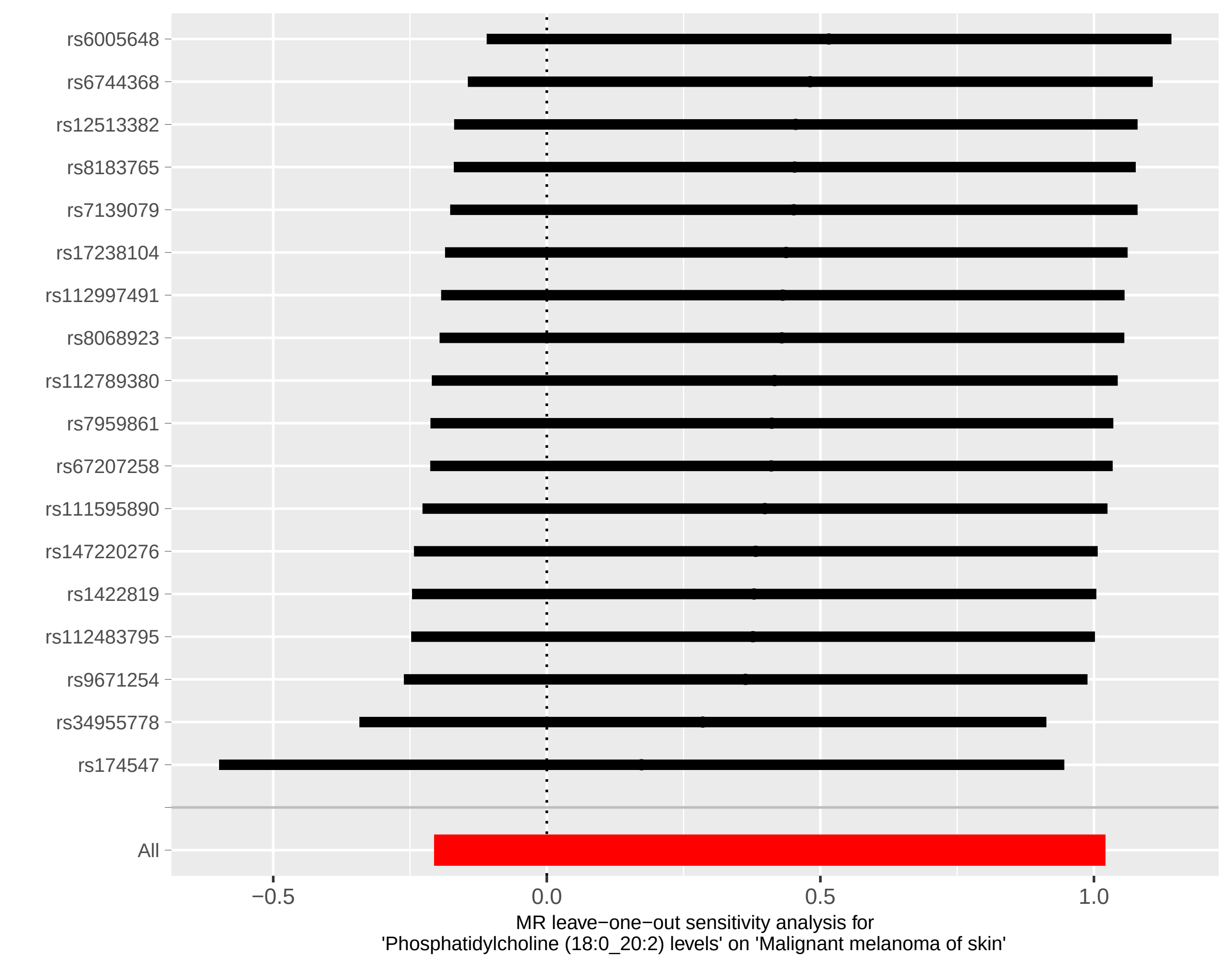
rs6005648
rs6744368
rs12513382
rs8183765
rs7139079
rs17238104
rs112997491
rs8068923
rs112789380
rs7959861
rs67207258
rs111595890
rs147220276
rs1422819
rs112483795
rs9671254
rs34955778
rs174547
All
−0.5
0.0
0.5
1.0
MR leave−one−out sensitivity analysis for
'Phosphatidylcholine (18:0_20:2) levels' on 'Malignant melanoma of skin'

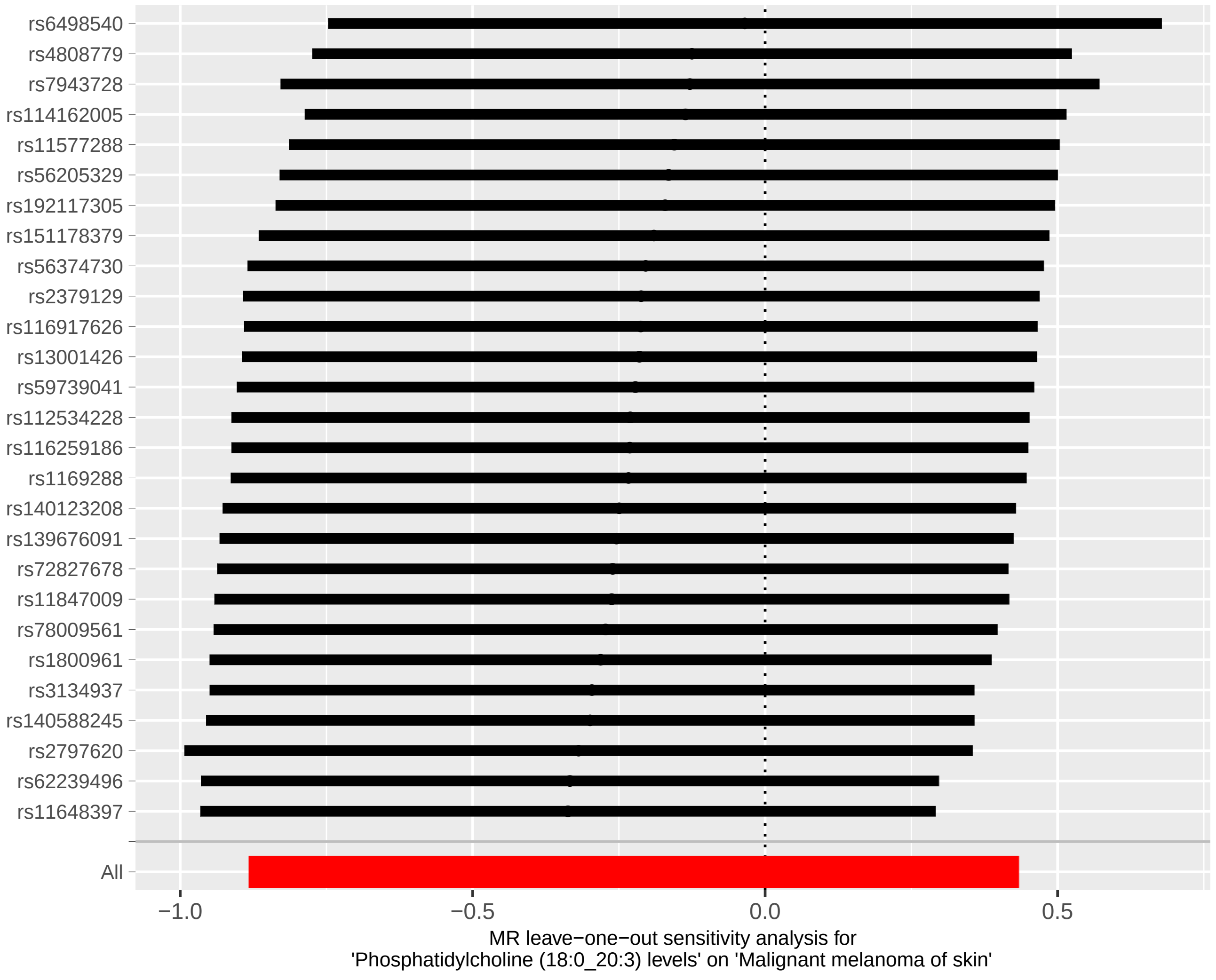
rs6498540
rs4808779
rs7943728
rs114162005
rs11577288
rs56205329
rs192117305
rs151178379
rs56374730
rs2379129
rs116917626
rs13001426
rs59739041
rs112534228
rs116259186
rs1169288
rs140123208
rs139676091
rs72827678
rs11847009
rs78009561
rs1800961
rs3134937
rs140588245
rs2797620
rs62239496
rs11648397
All
−1.0
−0.5
0.0
0.5
MR leave−one−out sensitivity analysis for
'Phosphatidylcholine (18:0_20:3) levels' on 'Malignant melanoma of skin'

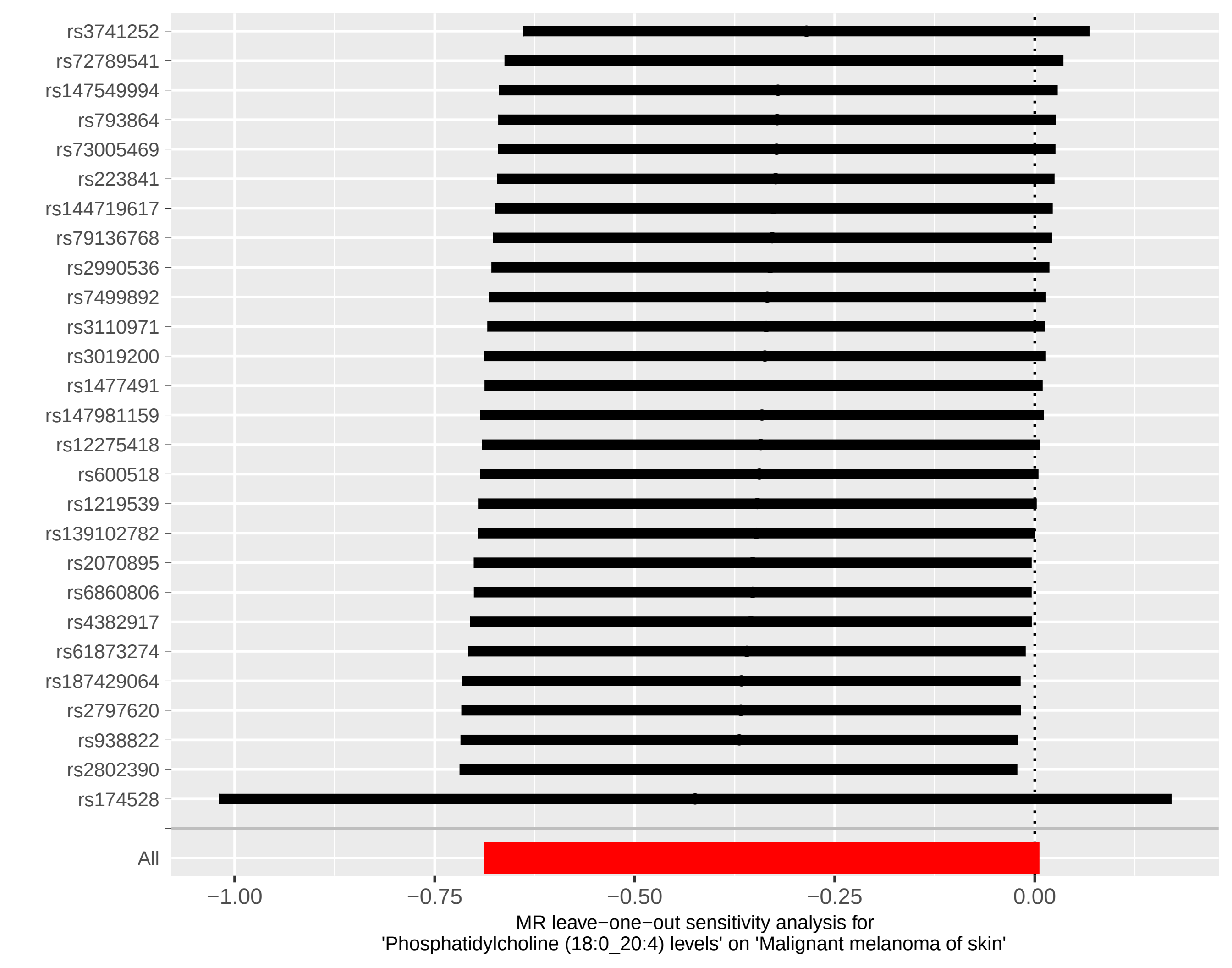
rs3741252
rs72789541
rs147549994
rs793864
rs73005469
rs223841
rs144719617
rs79136768
rs2990536
rs7499892
rs3110971
rs3019200
rs1477491
rs147981159
rs12275418
rs600518
rs1219539
rs139102782
rs2070895
rs6860806
rs4382917
rs61873274
rs187429064
rs2797620
rs938822
rs2802390
rs174528
All
−1.00
−0.75
−0.50
−0.25
0.00
MR leave−one−out sensitivity analysis for
'Phosphatidylcholine (18:0_20:4) levels' on 'Malignant melanoma of skin'

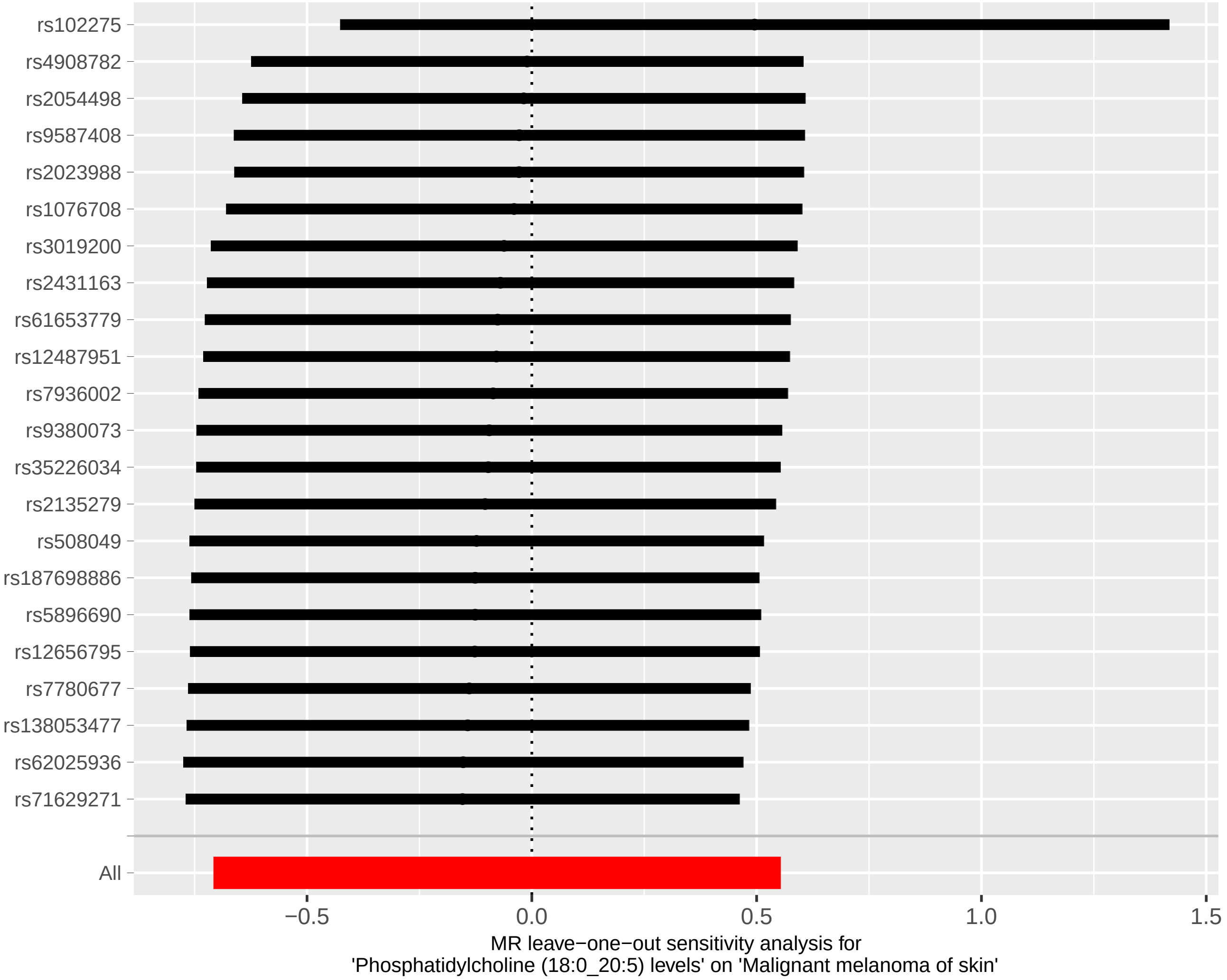
rs102275
rs4908782
rs2054498
rs9587408
rs2023988
rs1076708
rs3019200
rs2431163
rs61653779
rs12487951
rs7936002
rs9380073
rs35226034
rs2135279
rs508049
rs187698886
rs5896690
rs12656795
rs7780677
rs138053477
rs62025936
rs71629271
All
−0.5
0.0
0.5
1.0
1.5
MR leave−one−out sensitivity analysis for
'Phosphatidylcholine (18:0_20:5) levels' on 'Malignant melanoma of skin'

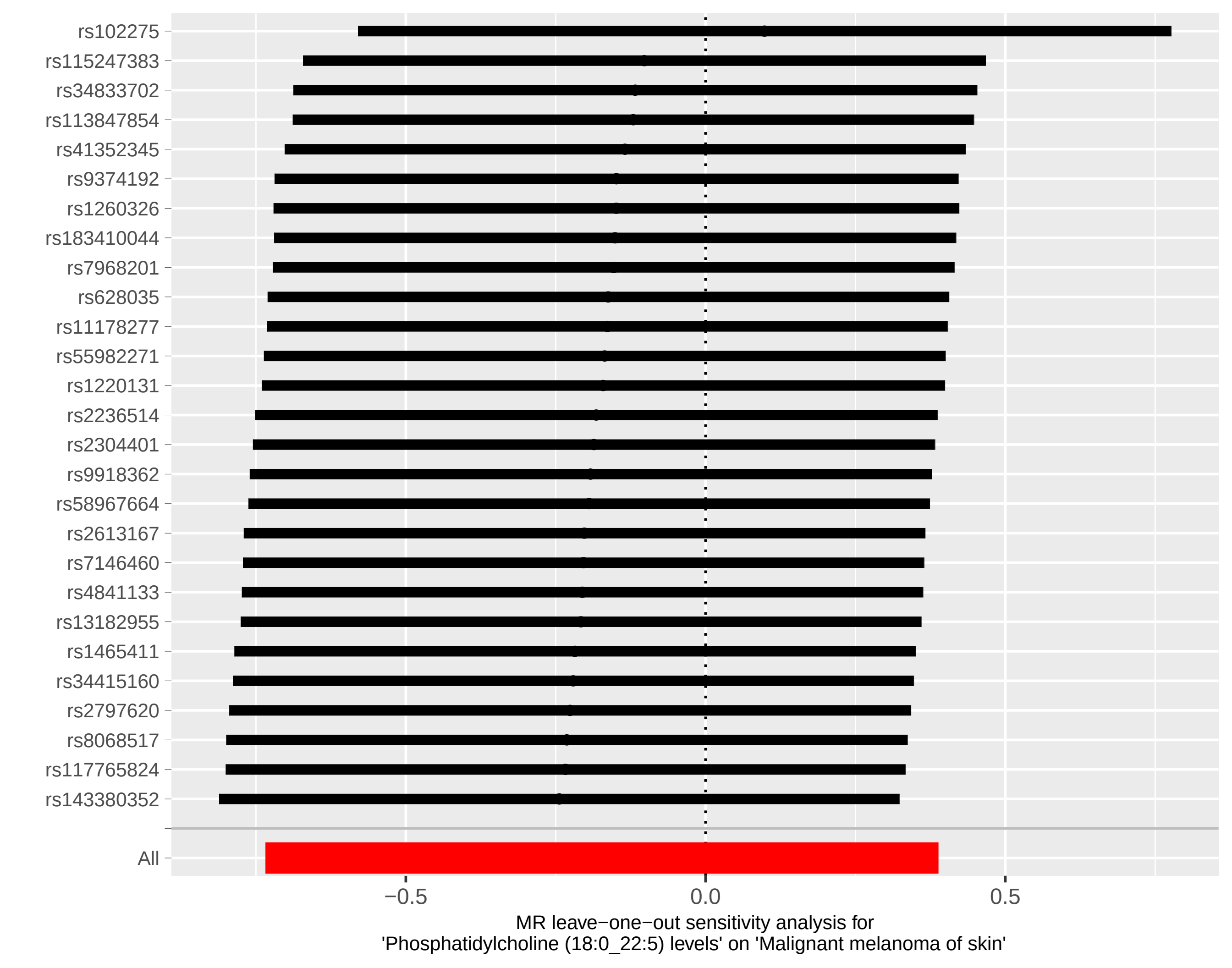
rs102275
rs115247383
rs34833702
rs113847854
rs41352345
rs9374192
rs1260326
rs183410044
rs7968201
rs628035
rs11178277
rs55982271
rs1220131
rs2236514
rs2304401
rs9918362
rs58967664
rs2613167
rs7146460
rs4841133
rs13182955
rs1465411
rs34415160
rs2797620
rs8068517
rs117765824
rs143380352
All
−0.5
0.0
0.5
MR leave−one−out sensitivity analysis for
'Phosphatidylcholine (18:0_22:5) levels' on 'Malignant melanoma of skin'

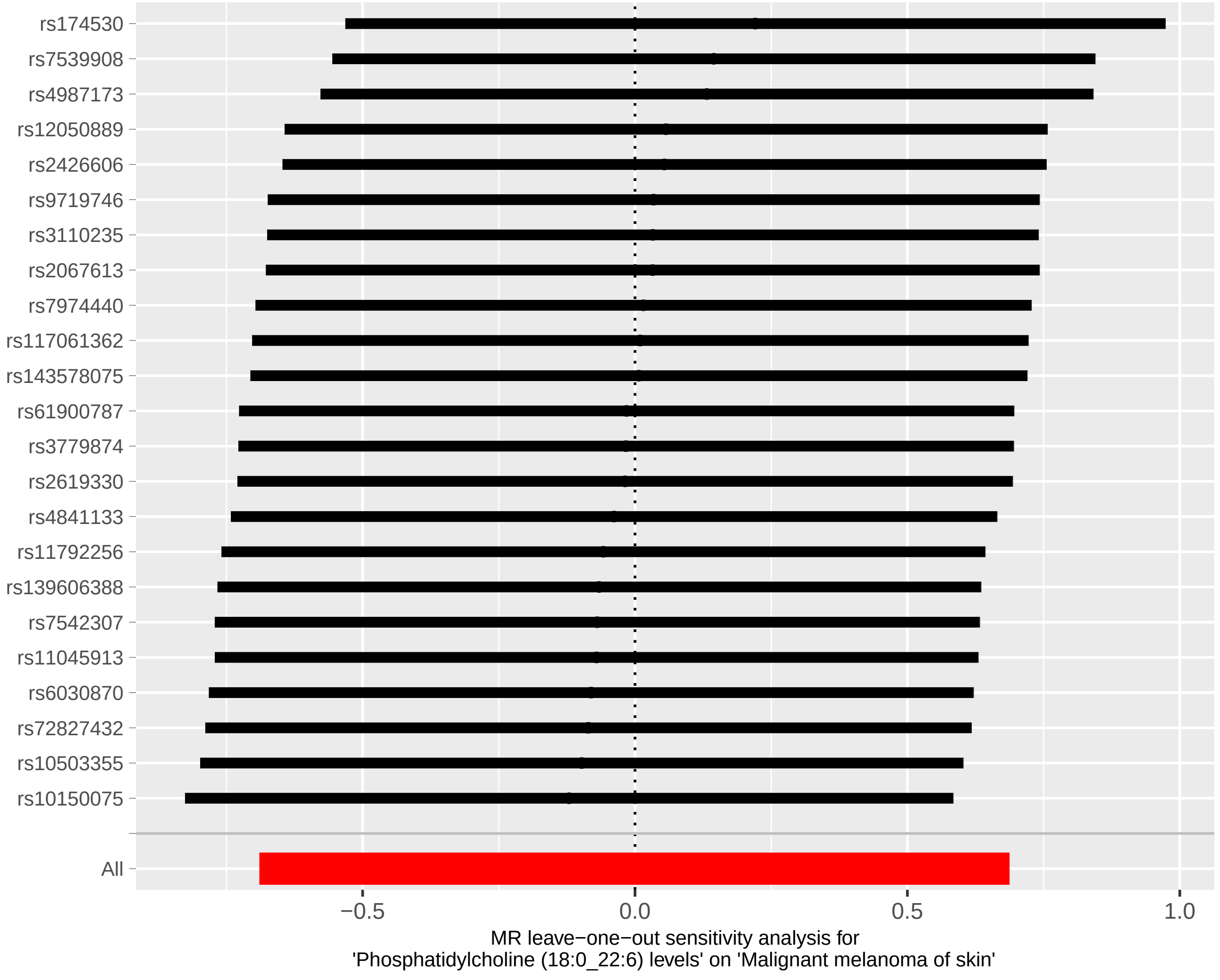
rs174530
rs7539908
rs4987173
rs12050889
rs2426606
rs9719746
rs3110235
rs2067613
rs7974440
rs117061362
rs143578075
rs61900787
rs3779874
rs2619330
rs4841133
rs11792256
rs139606388
rs7542307
rs11045913
rs6030870
rs72827432
rs10503355
rs10150075
All
−0.5
0.0
0.5
1.0
MR leave−one−out sensitivity analysis for
'Phosphatidylcholine (18:0_22:6) levels' on 'Malignant melanoma of skin'

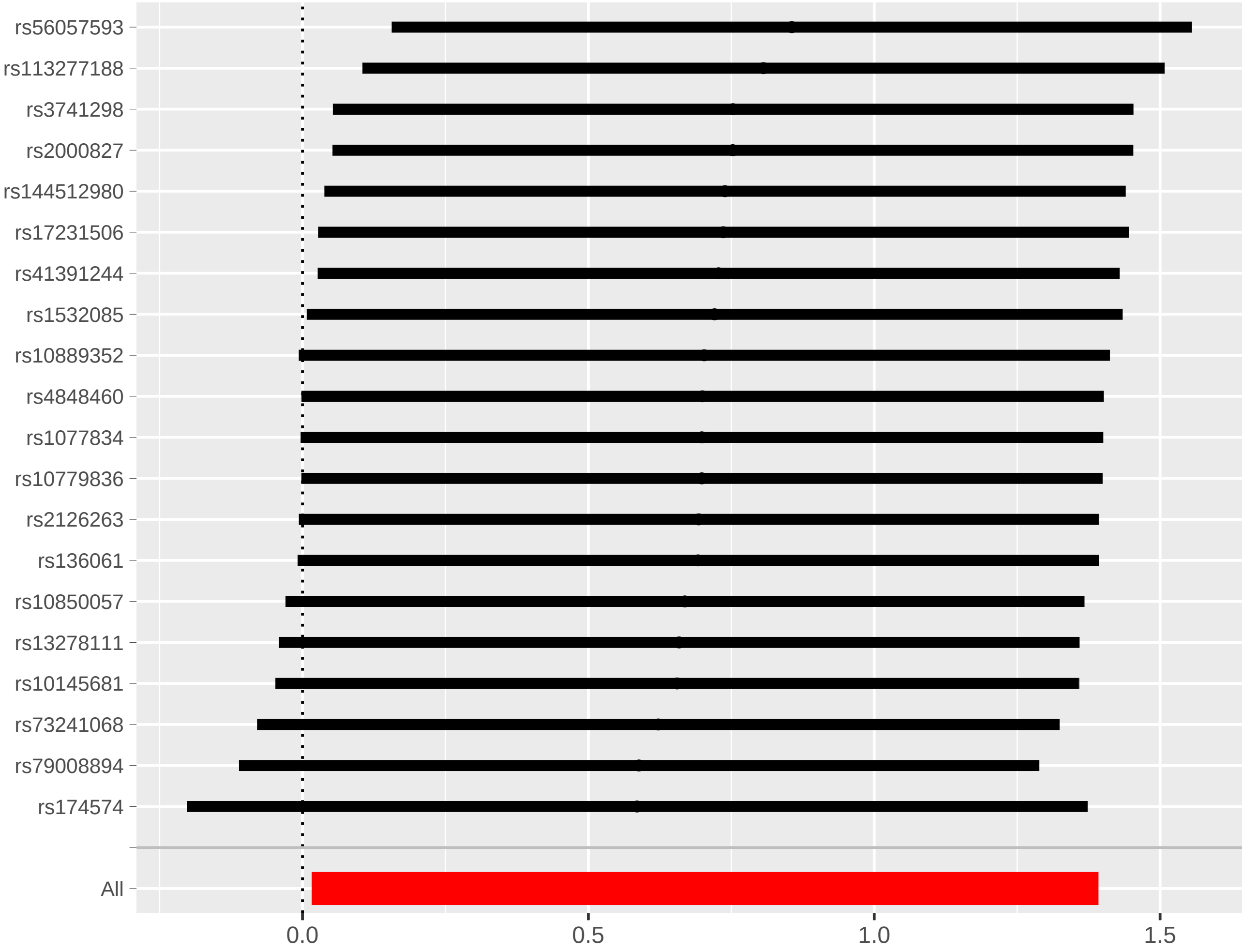

MR leave−one−out sensitivity analysis for
'Phosphatidylcholine (18:1_18:1) levels' on 'Malignant melanoma of skin'

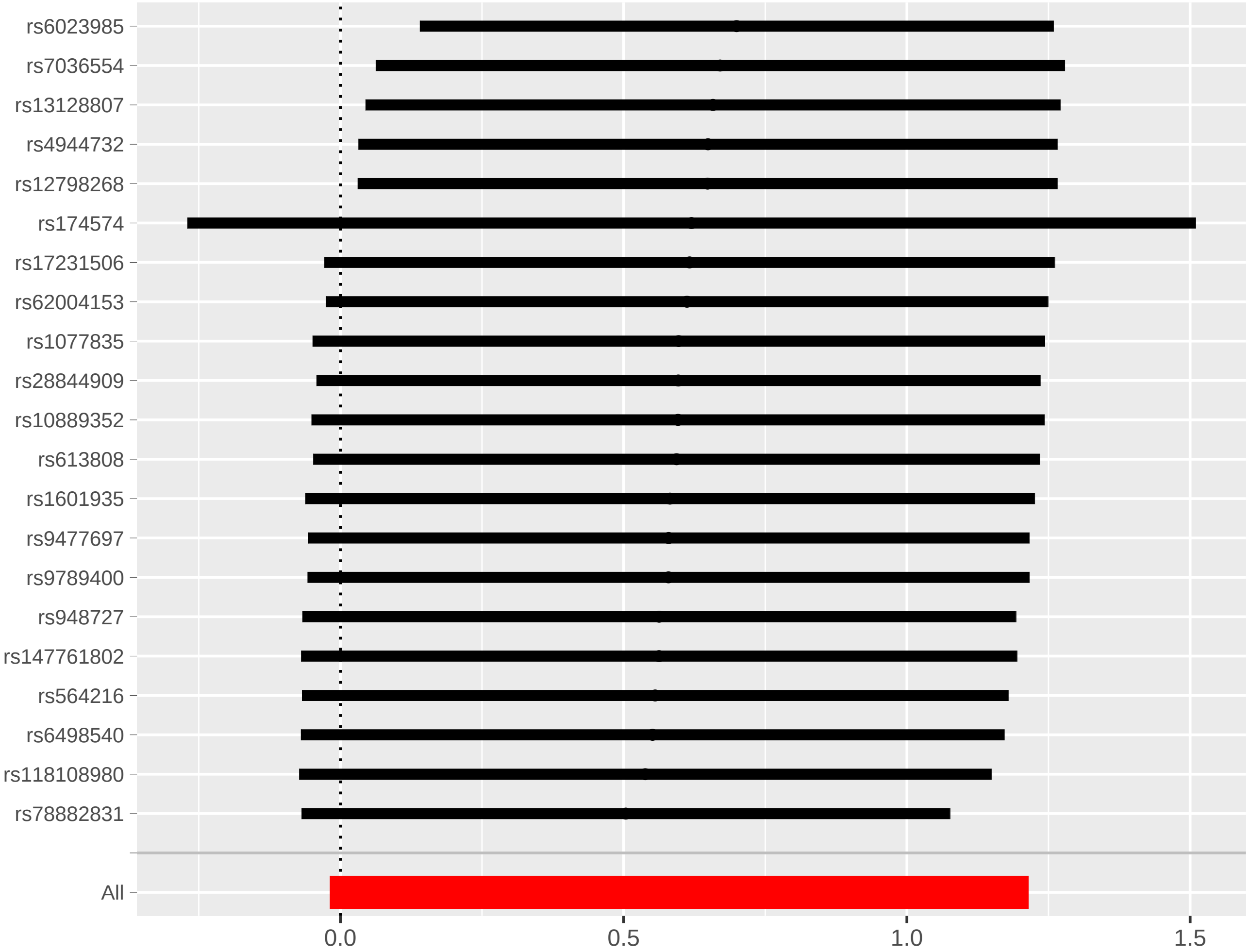

MR leave−one−out sensitivity analysis for
'Phosphatidylcholine (18:1_18:2) levels' on 'Malignant melanoma of skin'

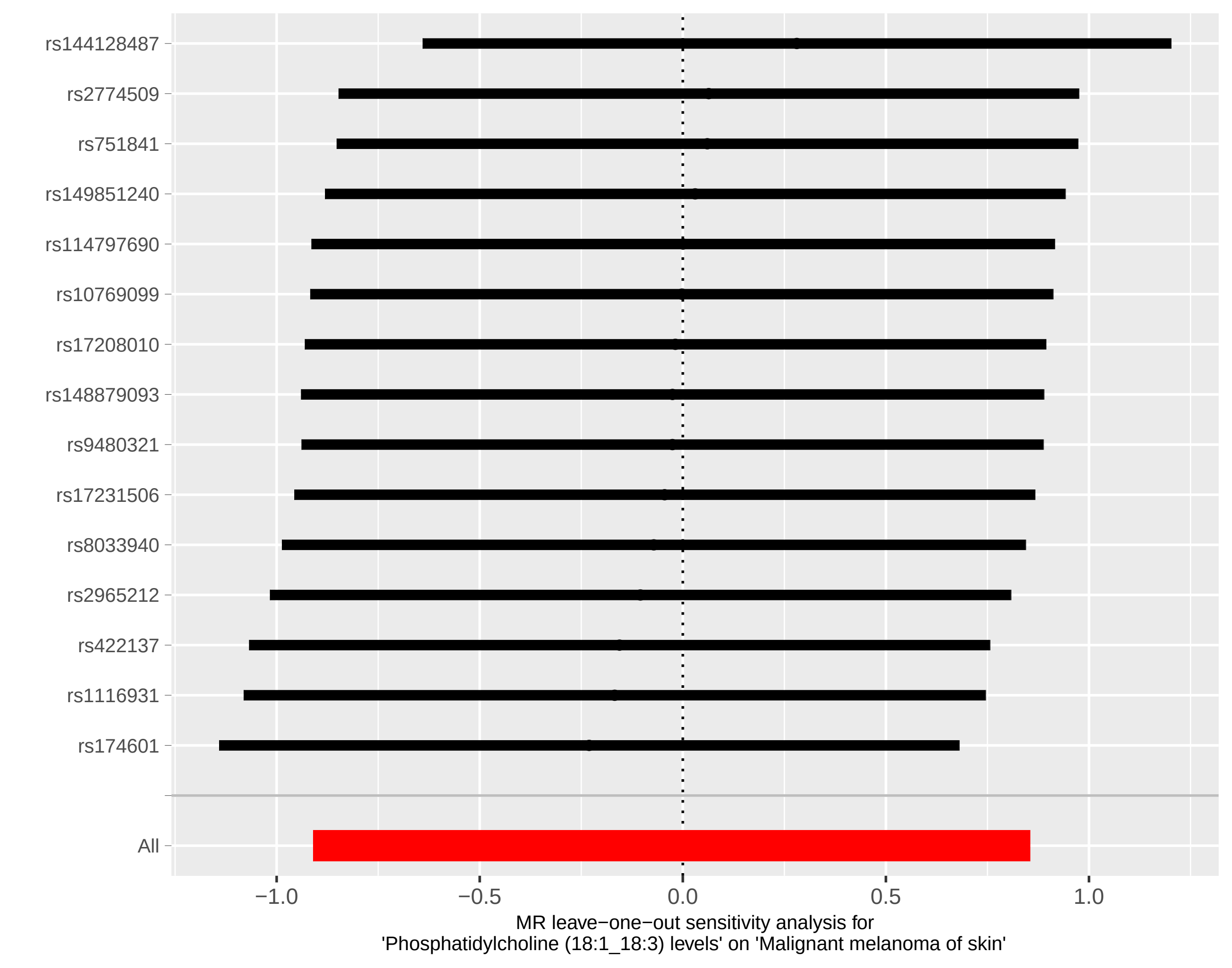
rs144128487
rs2774509
rs751841
rs149851240
rs114797690
rs10769099
rs17208010
rs148879093
rs9480321
rs17231506
rs8033940
rs2965212
rs422137
rs1116931
rs174601
All
−1.0
−0.5
0.0
0.5
1.0
MR leave−one−out sensitivity analysis for
'Phosphatidylcholine (18:1_18:3) levels' on 'Malignant melanoma of skin'

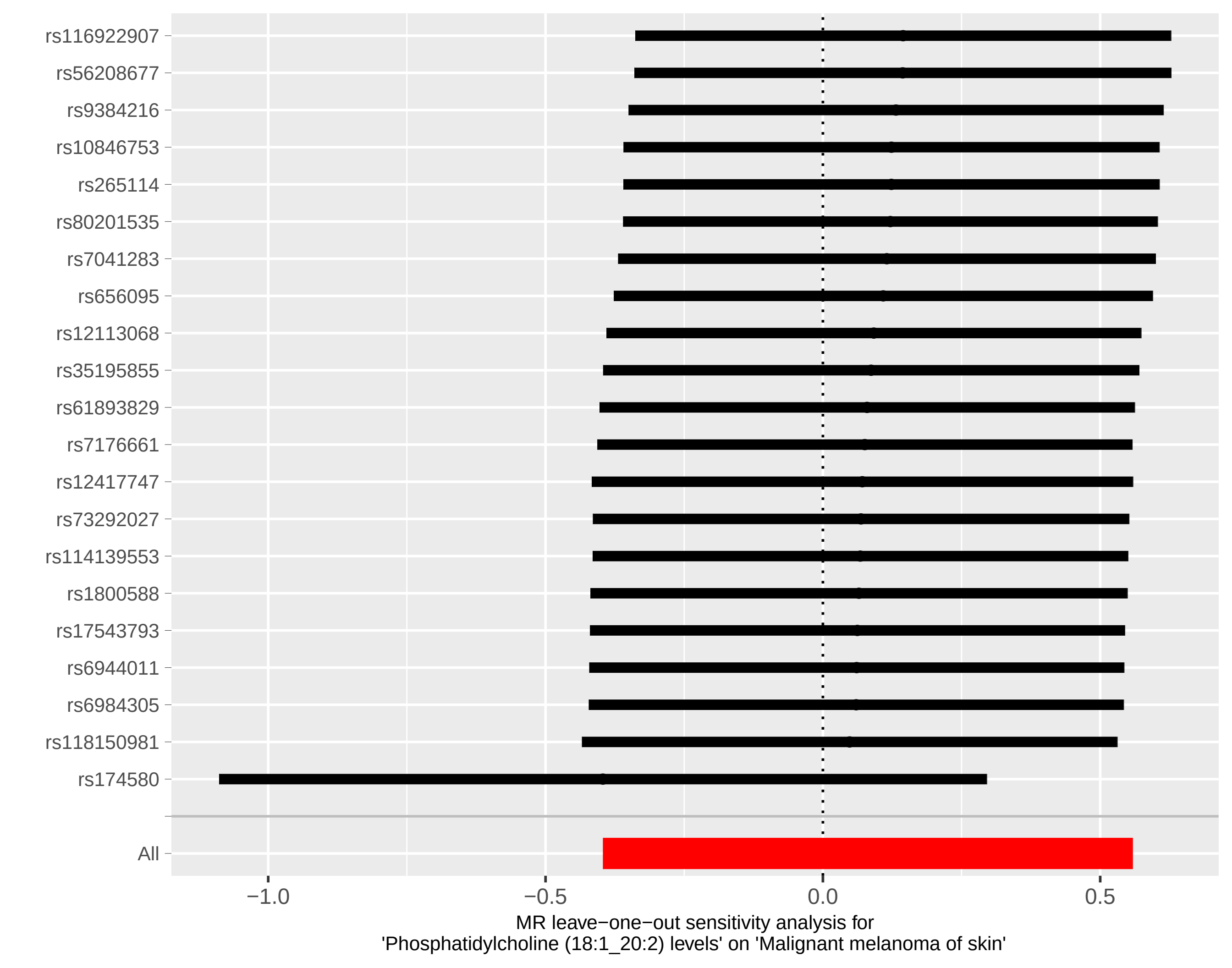
rs116922907
rs56208677
rs9384216
rs10846753
rs265114
rs80201535
rs7041283
rs656095
rs12113068
rs35195855
rs61893829
rs7176661
rs12417747
rs73292027
rs114139553
rs1800588
rs17543793
rs6944011
rs6984305
rs118150981
rs174580
All
−1.0
−0.5
0.0
0.5
MR leave−one−out sensitivity analysis for
'Phosphatidylcholine (18:1_20:2) levels' on 'Malignant melanoma of skin'

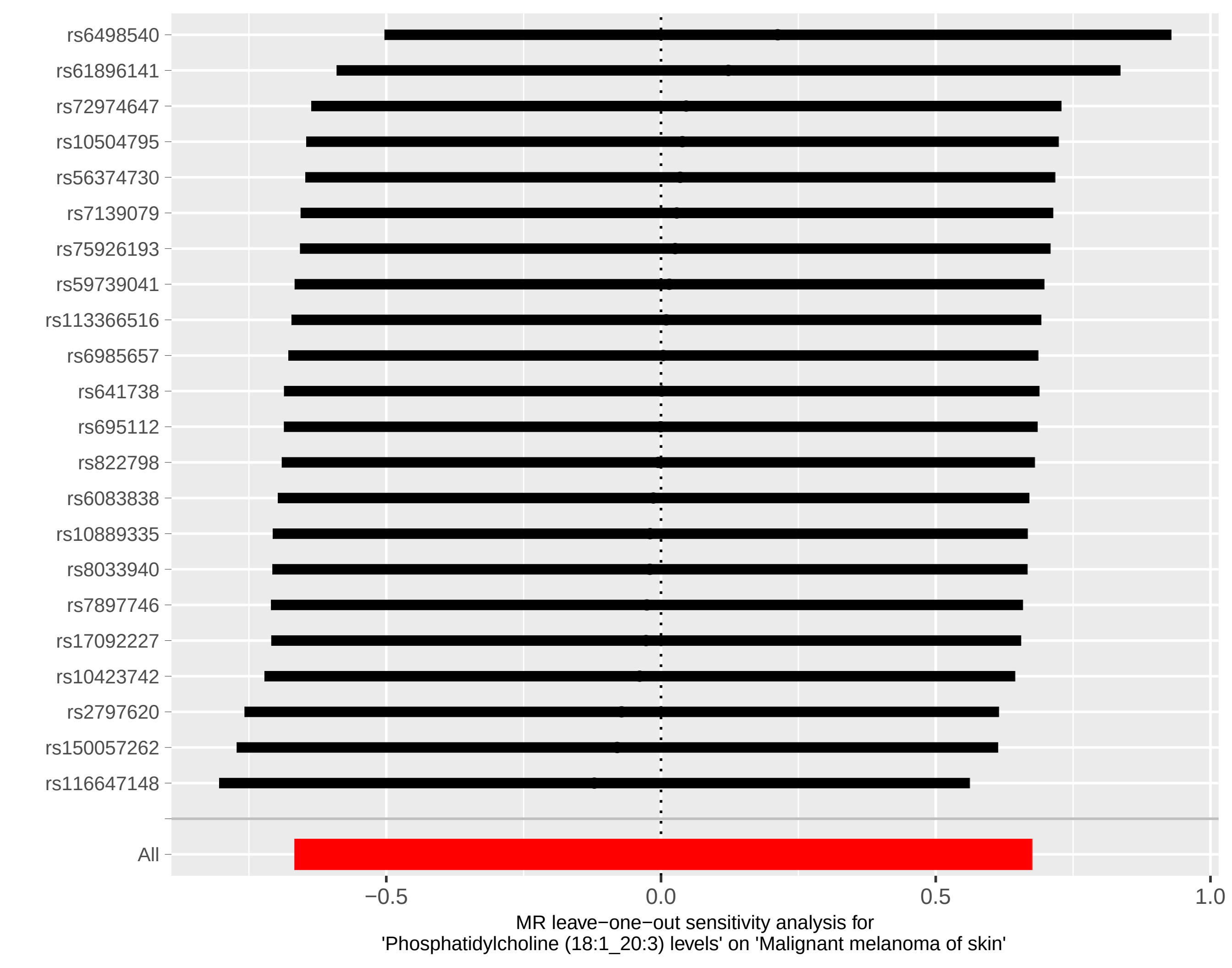
rs6498540
rs61896141
rs72974647
rs10504795
rs56374730
rs7139079
rs75926193
rs59739041
rs113366516
rs6985657
rs641738
rs695112
rs822798
rs6083838
rs10889335
rs8033940
rs7897746
rs17092227
rs10423742
rs2797620
rs150057262
rs116647148
All
−0.5
0.0
0.5
1.0
MR leave−one−out sensitivity analysis for
'Phosphatidylcholine (18:1_20:3) levels' on 'Malignant melanoma of skin'

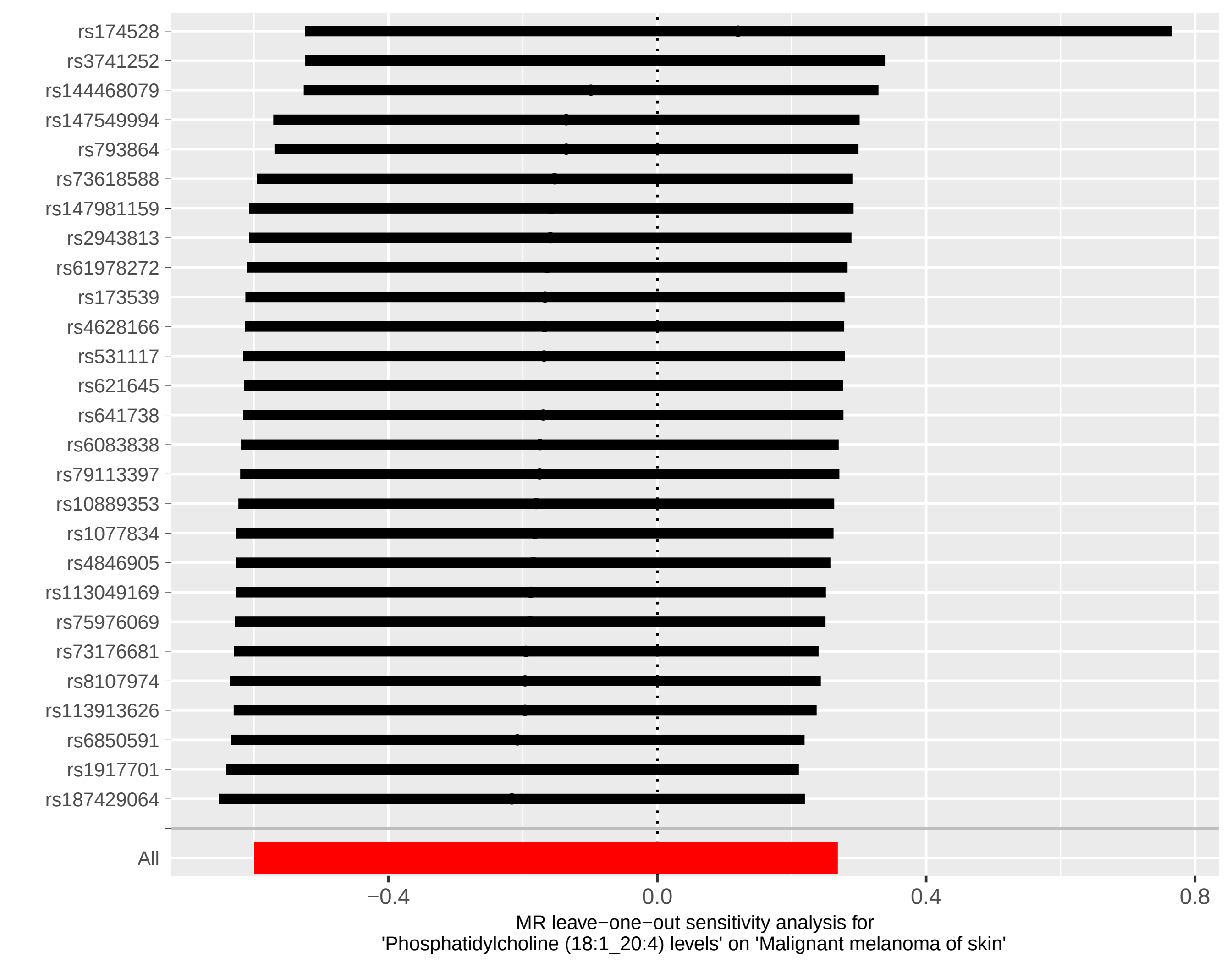

rs174528
rs3741252
rs144468079
rs147549994
rs793864
rs73618588
rs147981159
rs2943813
rs61978272
rs173539
rs4628166
rs531117
rs621645
rs641738
rs6083838
rs79113397
rs10889353
rs1077834
rs4846905
rs113049169
rs75976069
rs73176681
rs8107974
rs113913626
rs6850591
rs1917701
rs187429064
All
−0.4
0.0
0.4
0.8
MR leave−one−out sensitivity analysis for
'Phosphatidylcholine (18:1_20:4) levels' on 'Malignant melanoma of skin'

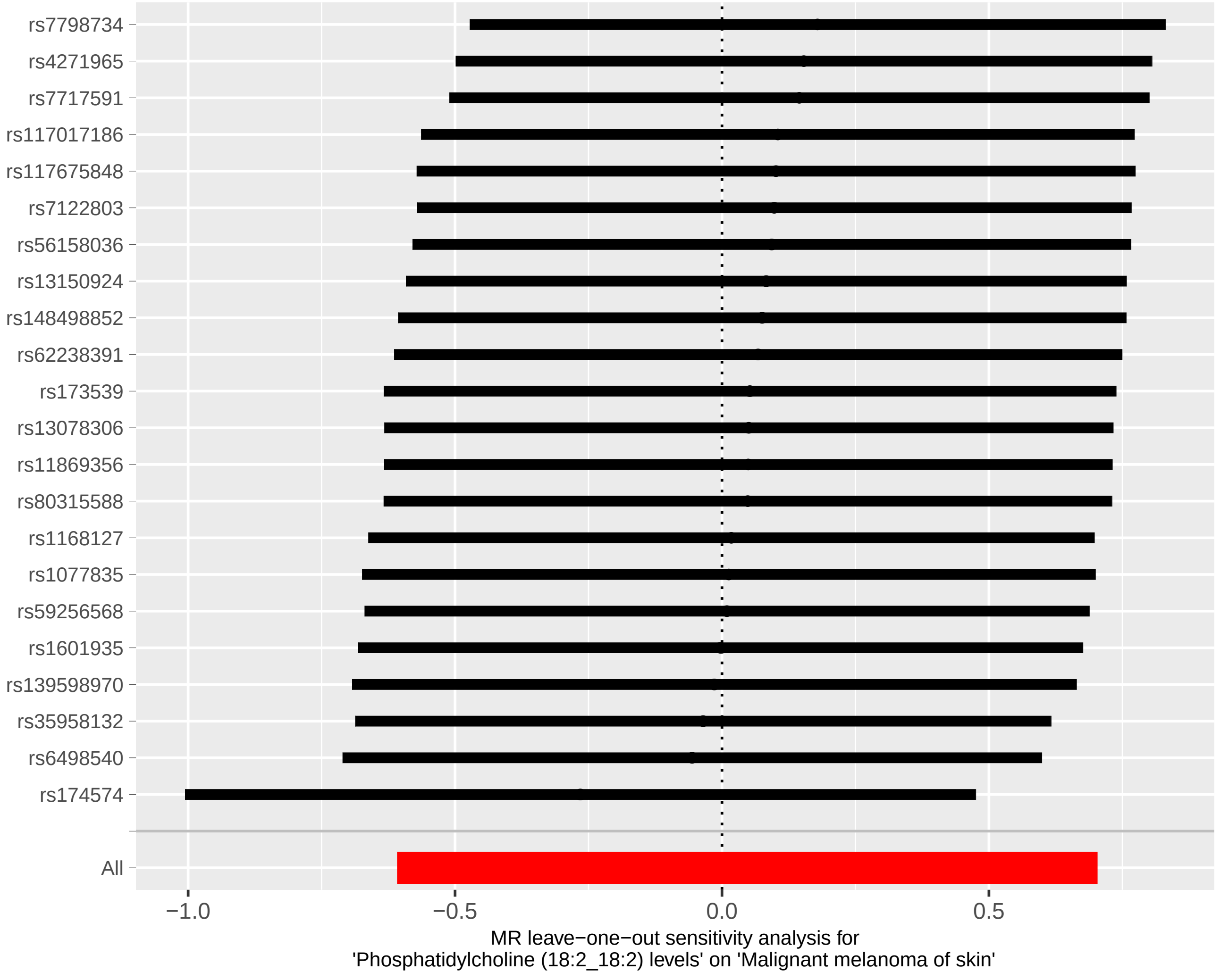
rs7798734
rs4271965
rs7717591
rs117017186
rs117675848
rs7122803
rs56158036
rs13150924
rs148498852
rs62238391
rs173539
rs13078306
rs11869356
rs80315588
rs1168127
rs1077835
rs59256568
rs1601935
rs139598970
rs35958132
rs6498540
rs174574
All
−1.0
−0.5
0.0
0.5
MR leave−one−out sensitivity analysis for
'Phosphatidylcholine (18:2_18:2) levels' on 'Malignant melanoma of skin'

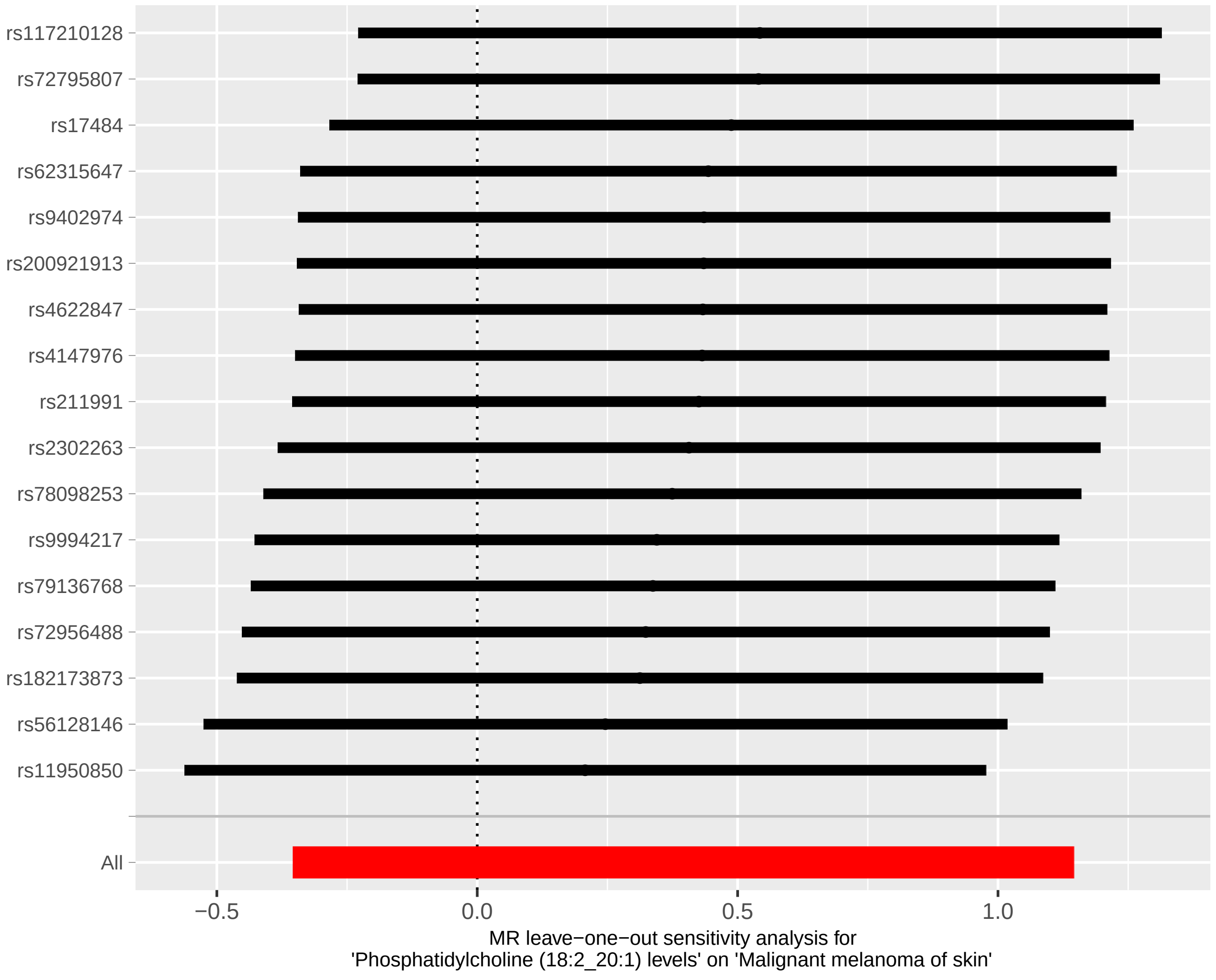

rs117210128
rs72795807
rs17484
rs62315647
rs9402974
rs200921913
rs4622847
rs4147976
rs211991
rs2302263
rs78098253
rs9994217
rs79136768
rs72956488
rs182173873
rs56128146
rs11950850
All
−0.5
0.0
0.5
1.0
MR leave−one−out sensitivity analysis for
'Phosphatidylcholine (18:2_20:1) levels' on 'Malignant melanoma of skin'

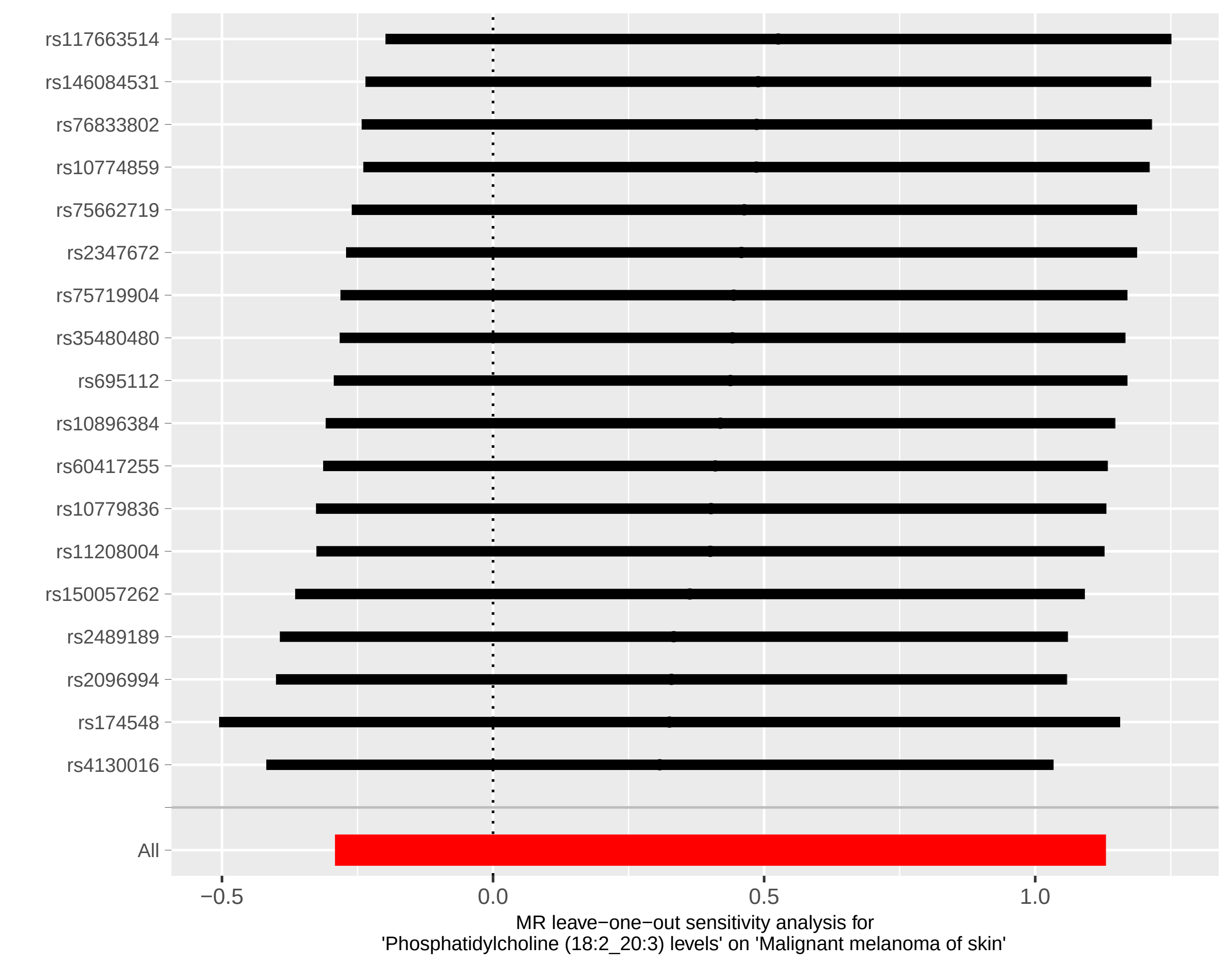
rs117663514
rs146084531
rs76833802
rs10774859
rs75662719
rs2347672
rs75719904
rs35480480
rs695112
rs10896384
rs60417255
rs10779836
rs11208004
rs150057262
rs2489189
rs2096994
rs174548
rs4130016
All
−0.5
0.0
0.5
1.0
MR leave−one−out sensitivity analysis for
'Phosphatidylcholine (18:2_20:3) levels' on 'Malignant melanoma of skin'

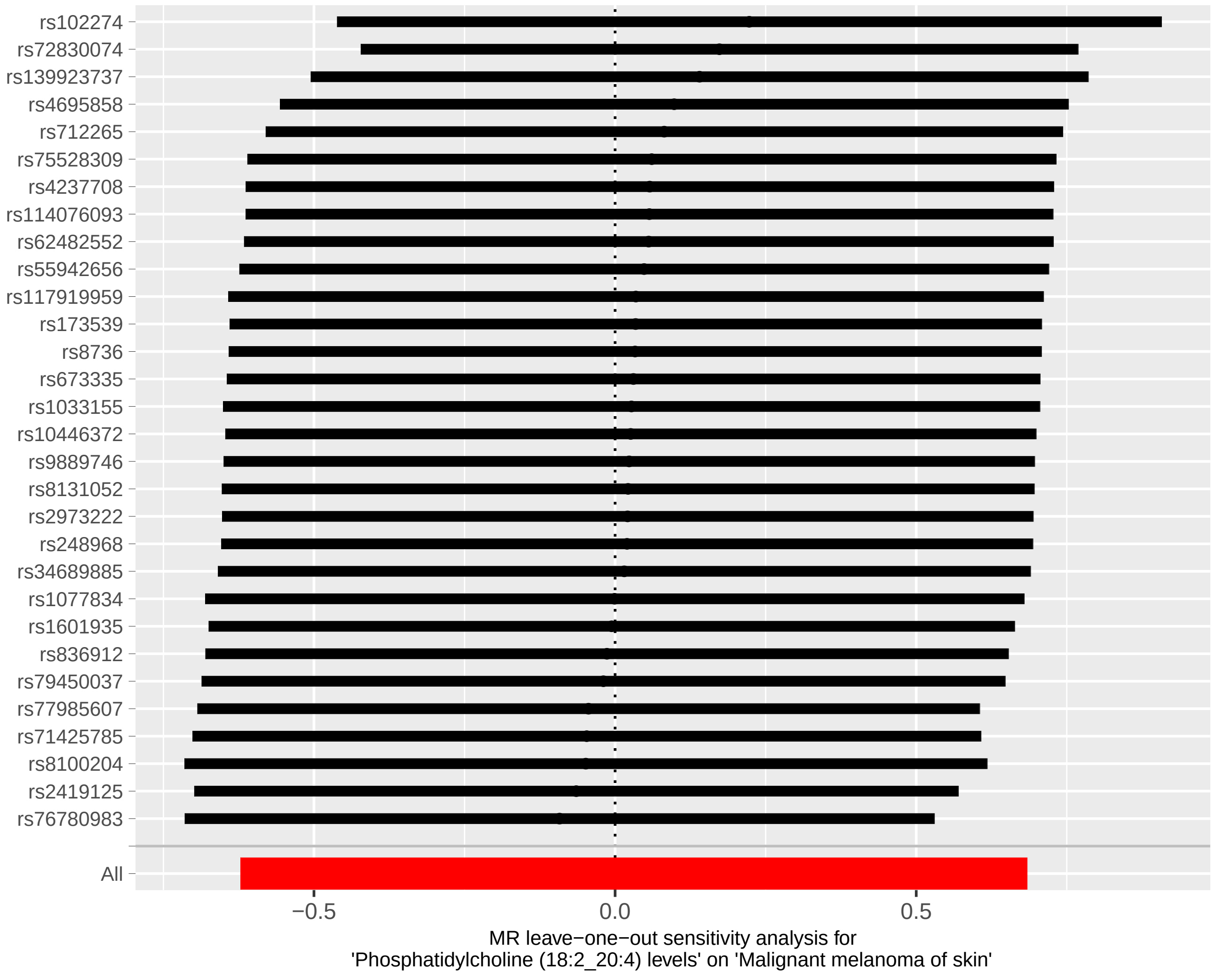
rs102274
rs72830074
rs139923737
rs4695858
rs712265
rs75528309
rs4237708
rs114076093
rs62482552
rs55942656
rs117919959
rs173539
rs8736
rs673335
rs1033155
rs10446372
rs9889746
rs8131052
rs2973222
rs248968
rs34689885
rs1077834
rs1601935
rs836912
rs79450037
rs77985607
rs71425785
rs8100204
rs2419125
rs76780983
All
−0.5
0.0
0.5
MR leave−one−out sensitivity analysis for
'Phosphatidylcholine (18:2_20:4) levels' on 'Malignant melanoma of skin'

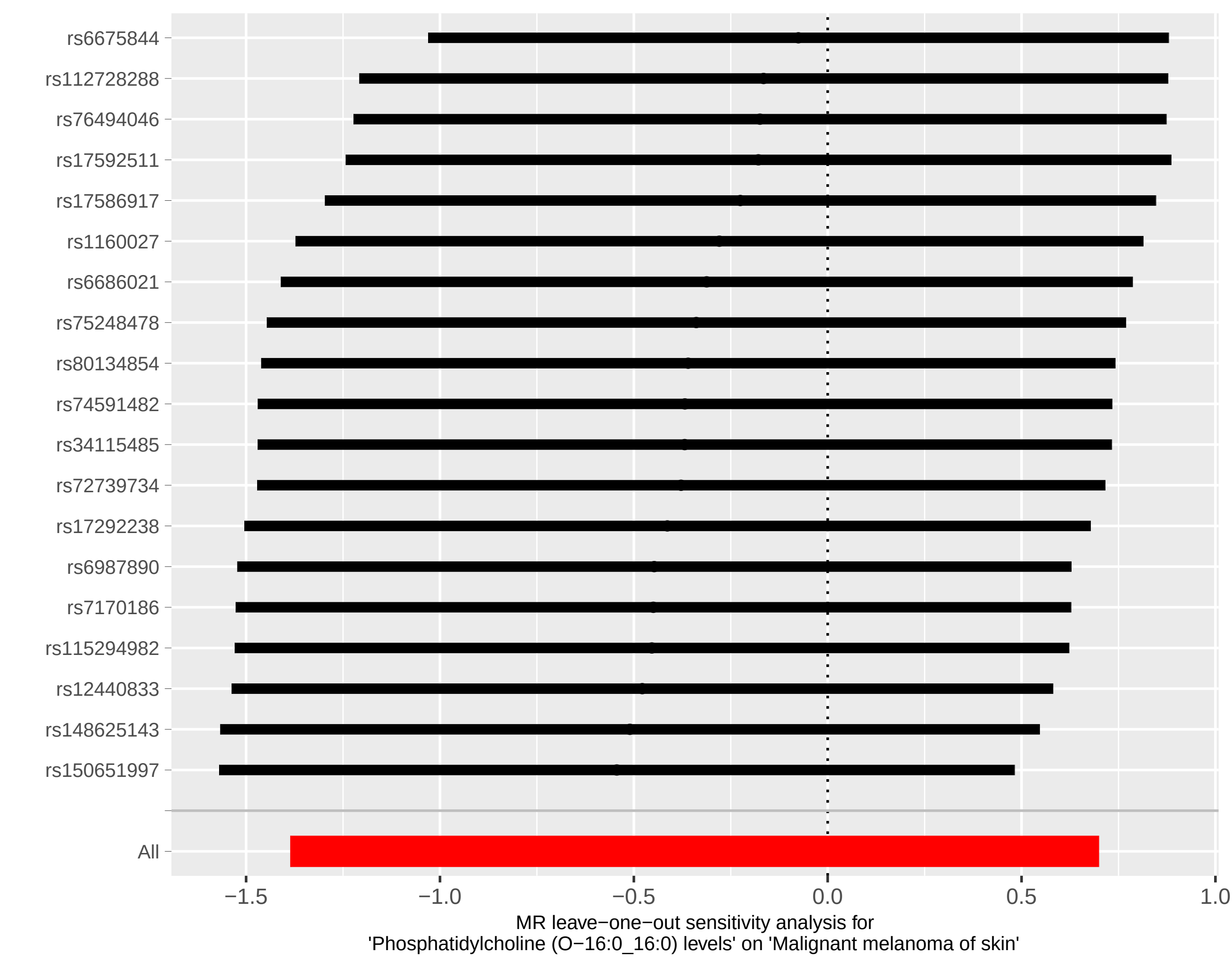

rs6675844
rs112728288
rs76494046
rs17592511
rs17586917
rs1160027
rs6686021
rs75248478
rs80134854
rs74591482
rs34115485
rs72739734
rs17292238
rs6987890
rs7170186
rs115294982
rs12440833
rs148625143
rs150651997
All
−1.5
−1.0
−0.5
0.0
0.5
1.0
MR leave−one−out sensitivity analysis for
'Phosphatidylcholine (O−16:0_16:0) levels' on 'Malignant melanoma of skin'

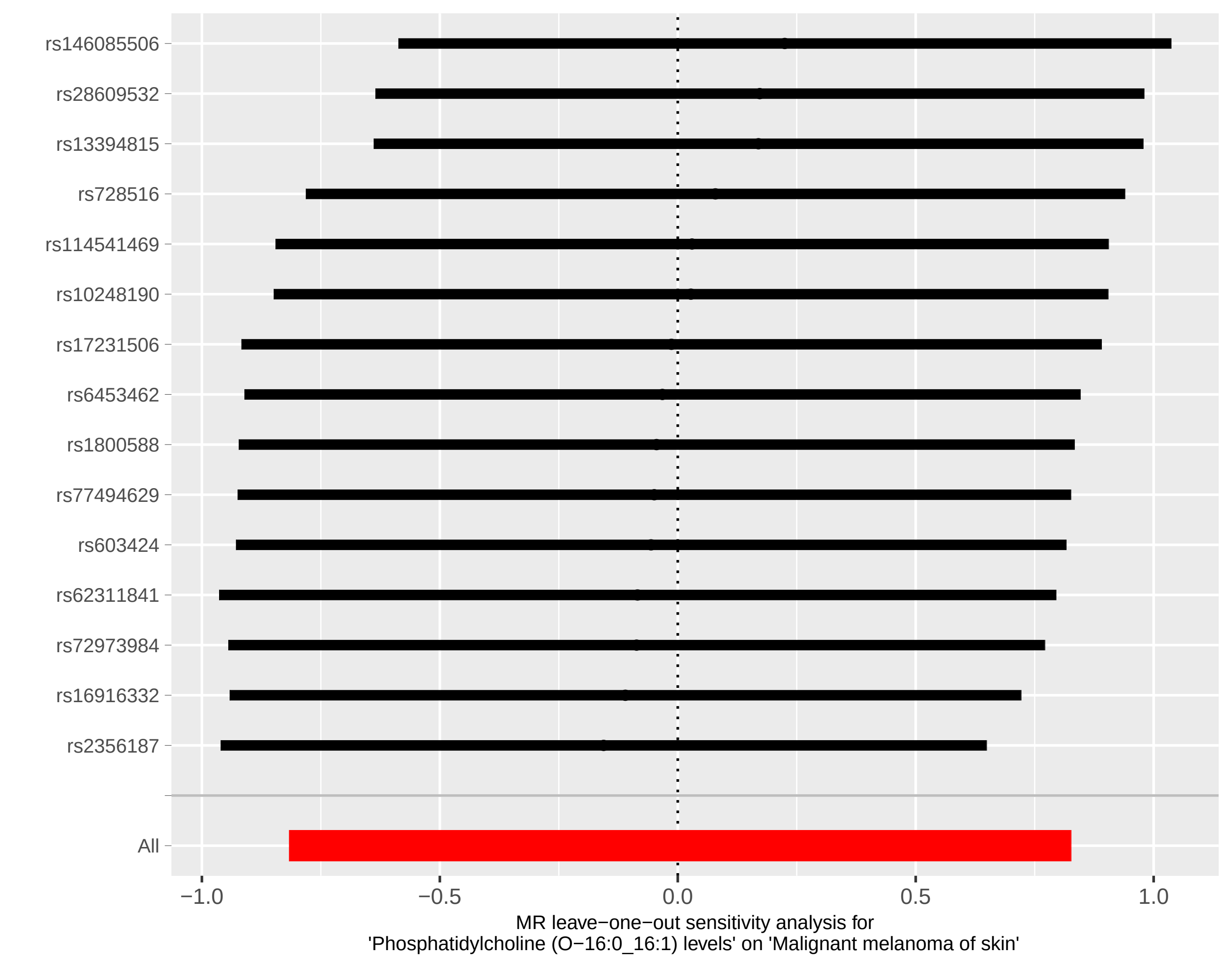
rs146085506
rs28609532
rs13394815
rs728516
rs114541469
rs10248190
rs17231506
rs6453462
rs1800588
rs77494629
rs603424
rs62311841
rs72973984
rs16916332
rs2356187
All
−1.0
−0.5
0.0
0.5
1.0
MR leave−one−out sensitivity analysis for
'Phosphatidylcholine (O−16:0_16:1) levels' on 'Malignant melanoma of skin'

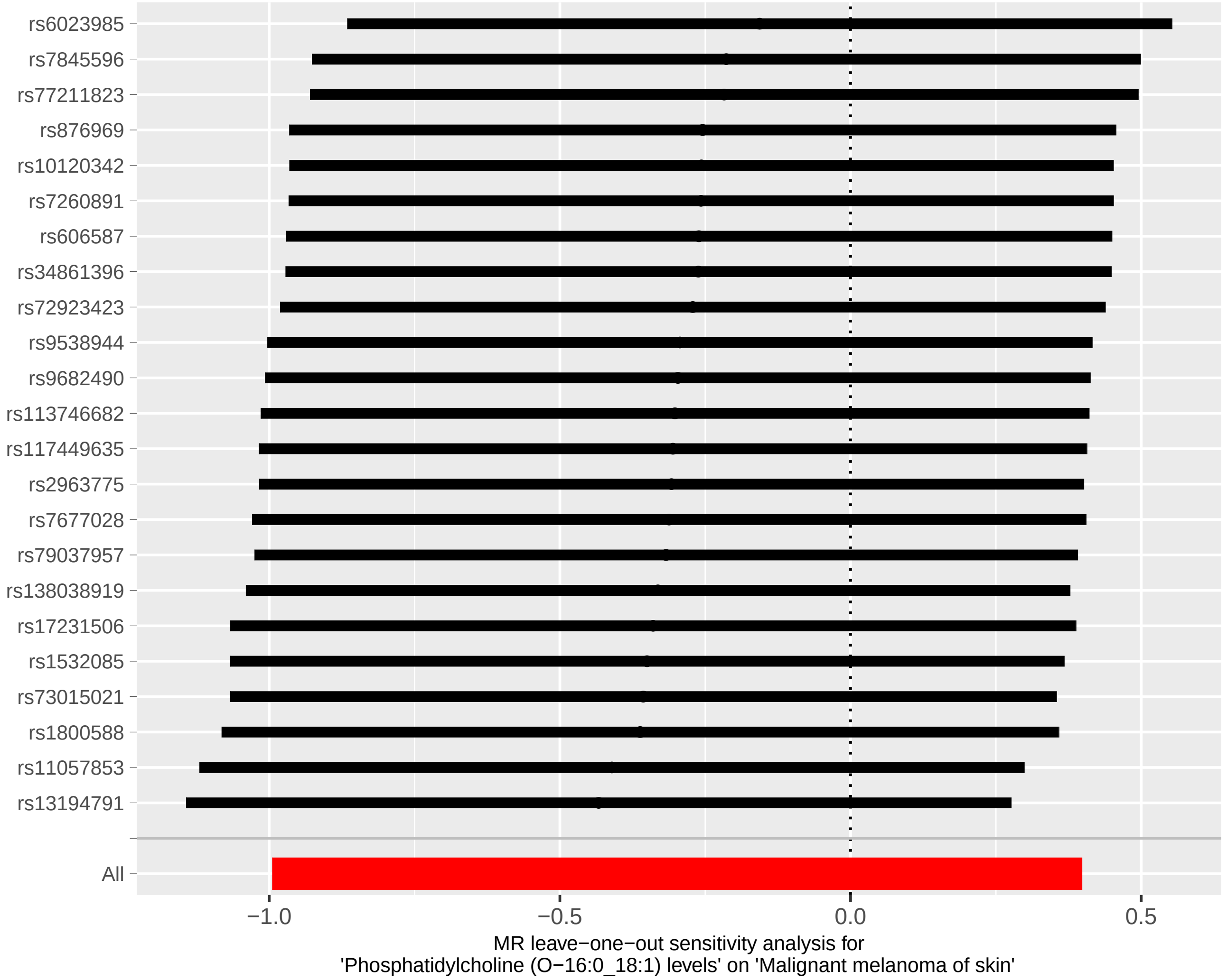

rs6023985
rs7845596
rs77211823
rs876969
rs10120342
rs7260891
rs606587
rs34861396
rs72923423
rs9538944
rs9682490
rs113746682
rs117449635
rs2963775
rs7677028
rs79037957
rs138038919
rs17231506
rs1532085
rs73015021
rs1800588
rs11057853
rs13194791
All
−1.0
−0.5
0.0
0.5
MR leave−one−out sensitivity analysis for
'Phosphatidylcholine (O−16:0_18:1) levels' on 'Malignant melanoma of skin'

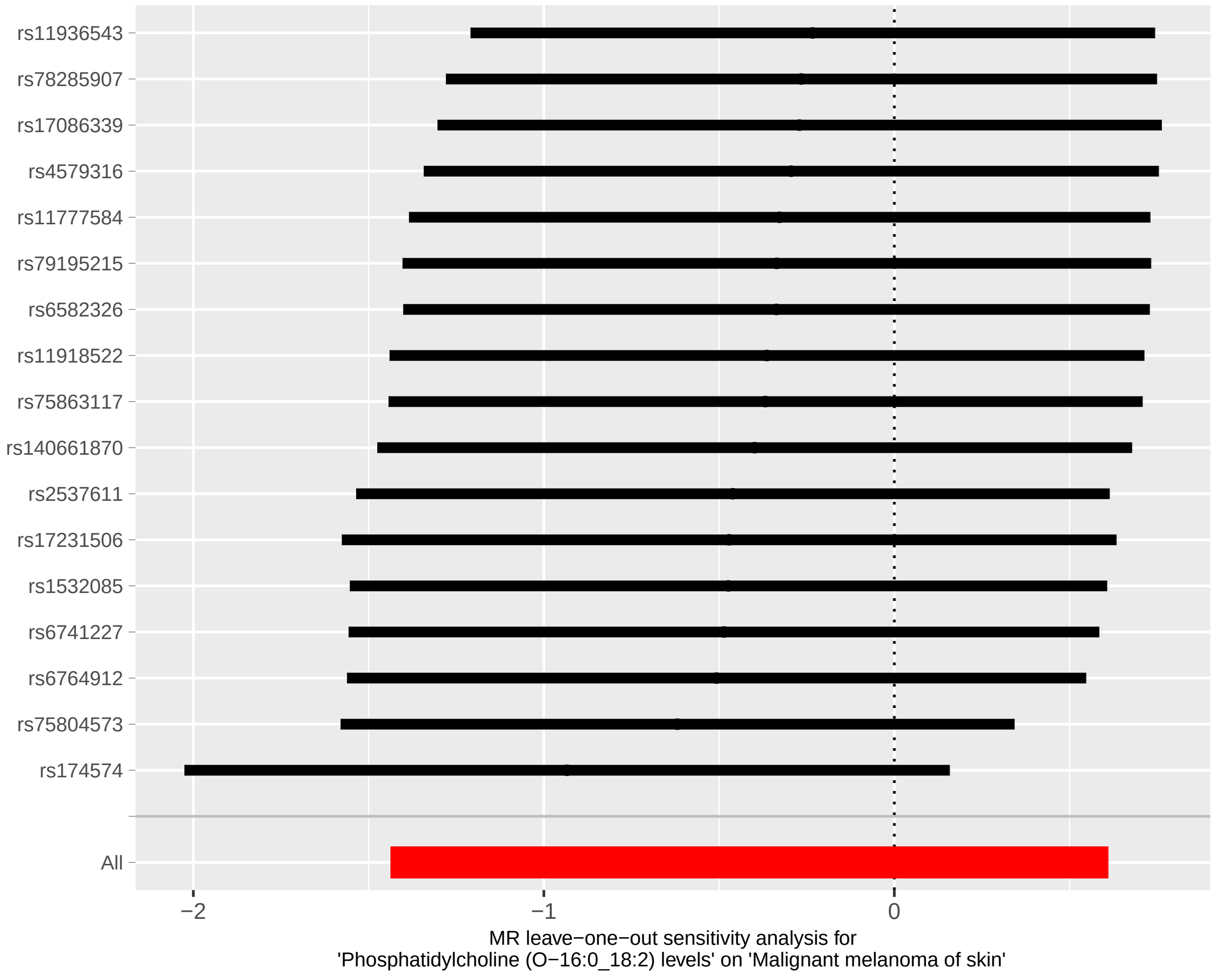
rs11936543
rs78285907
rs17086339
rs4579316
rs11777584
rs79195215
rs6582326
rs11918522
rs75863117
rs140661870
rs2537611
rs17231506
rs1532085
rs6741227
rs6764912
rs75804573
rs174574
All
−2
−1
0
MR leave−one−out sensitivity analysis for
'Phosphatidylcholine (O−16:0_18:2) levels' on 'Malignant melanoma of skin'

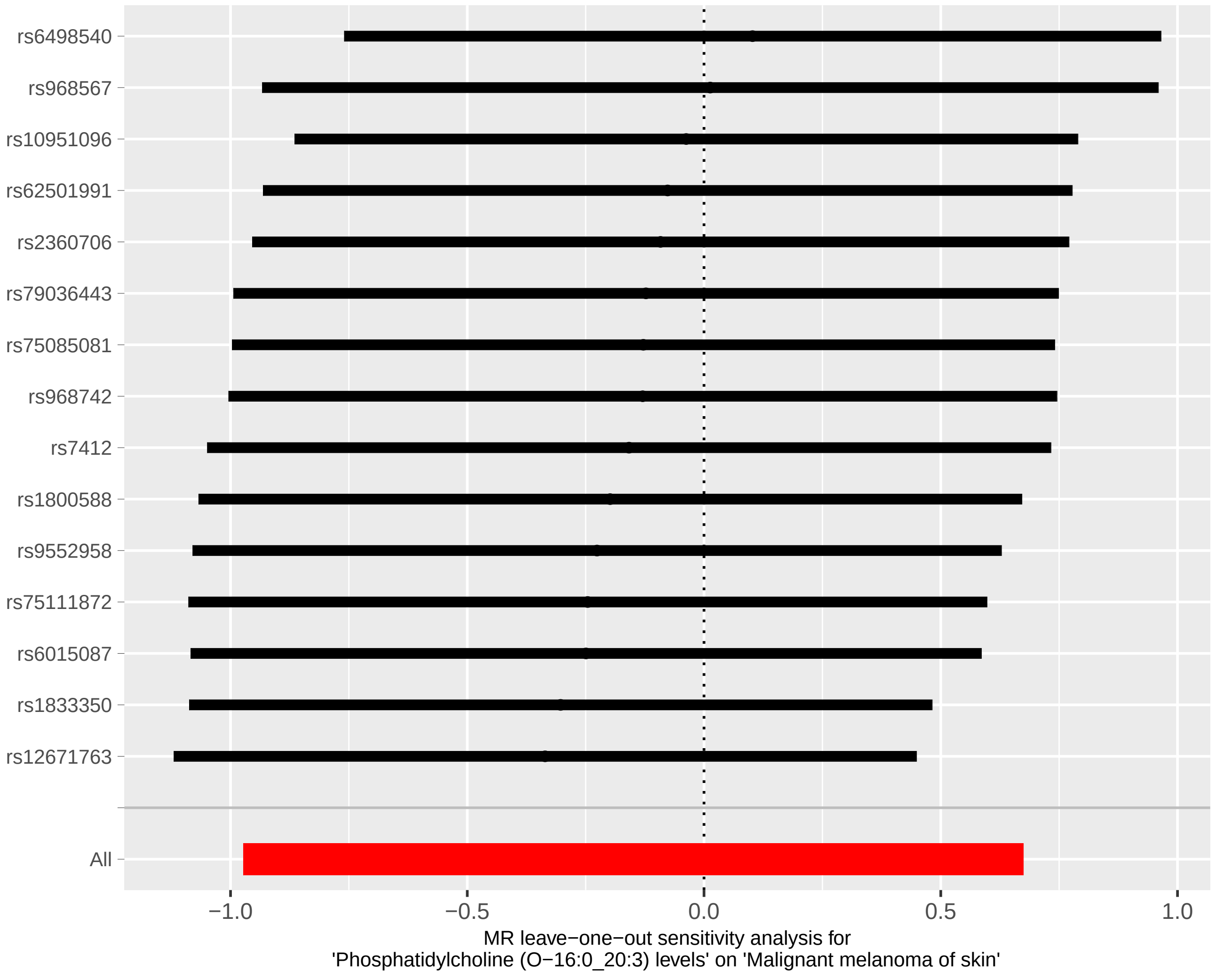

rs6498540
rs968567
rs10951096
rs62501991
rs2360706
rs79036443
rs75085081
rs968742
rs7412
rs1800588
rs9552958
rs75111872
rs6015087
rs1833350
rs12671763
All
−1.0
−0.5
0.0
0.5
1.0
MR leave−one−out sensitivity analysis for
'Phosphatidylcholine (O−16:0_20:3) levels' on 'Malignant melanoma of skin'

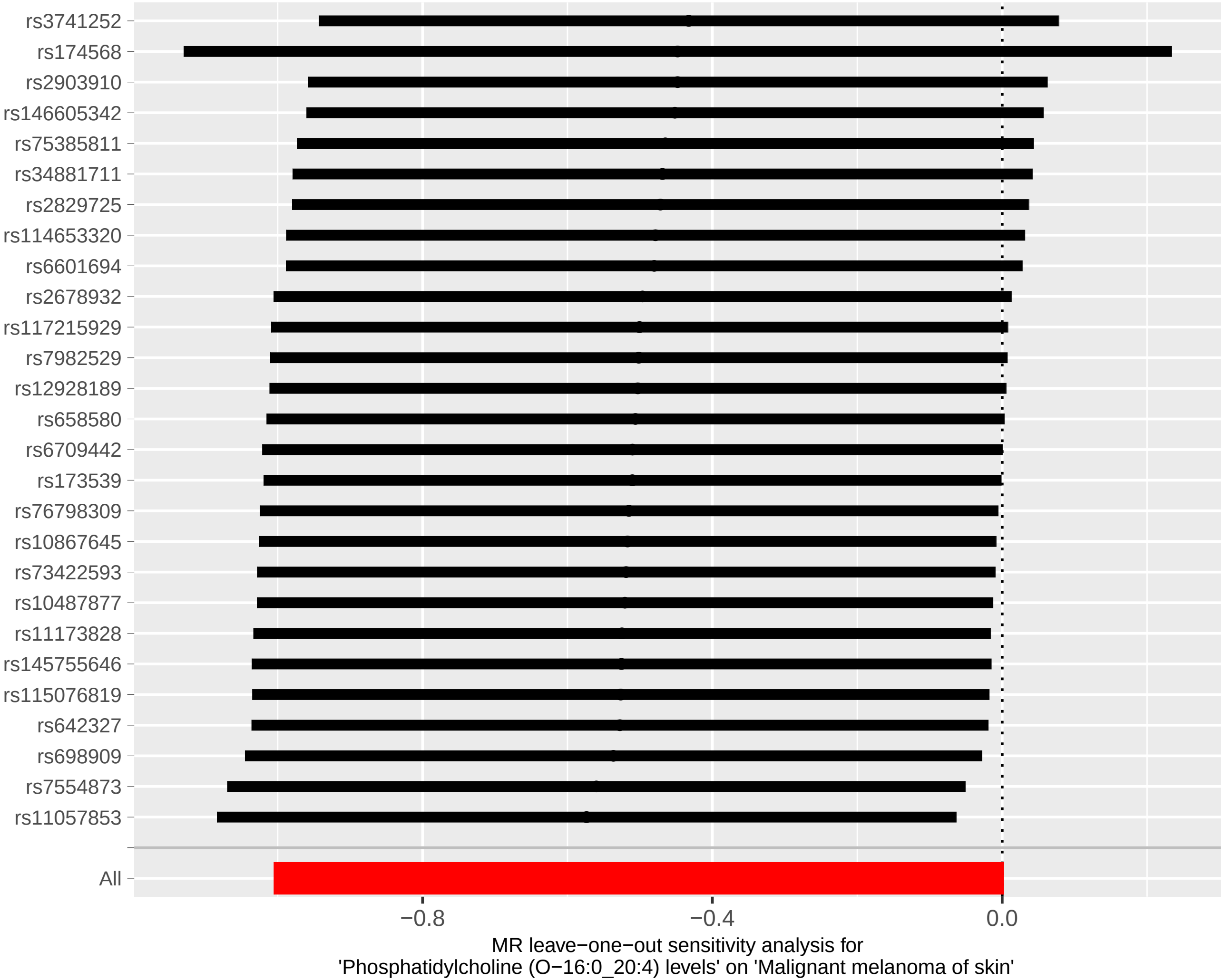
rs3741252
rs174568
rs2903910
rs146605342
rs75385811
rs34881711
rs2829725
rs114653320
rs6601694
rs2678932
rs117215929
rs7982529
rs12928189
rs658580
rs6709442
rs173539
rs76798309
rs10867645
rs73422593
rs10487877
rs11173828
rs145755646
rs115076819
rs642327
rs698909
rs7554873
rs11057853
All
−0.8
−0.4
0.0
MR leave−one−out sensitivity analysis for
'Phosphatidylcholine (O−16:0_20:4) levels' on 'Malignant melanoma of skin'

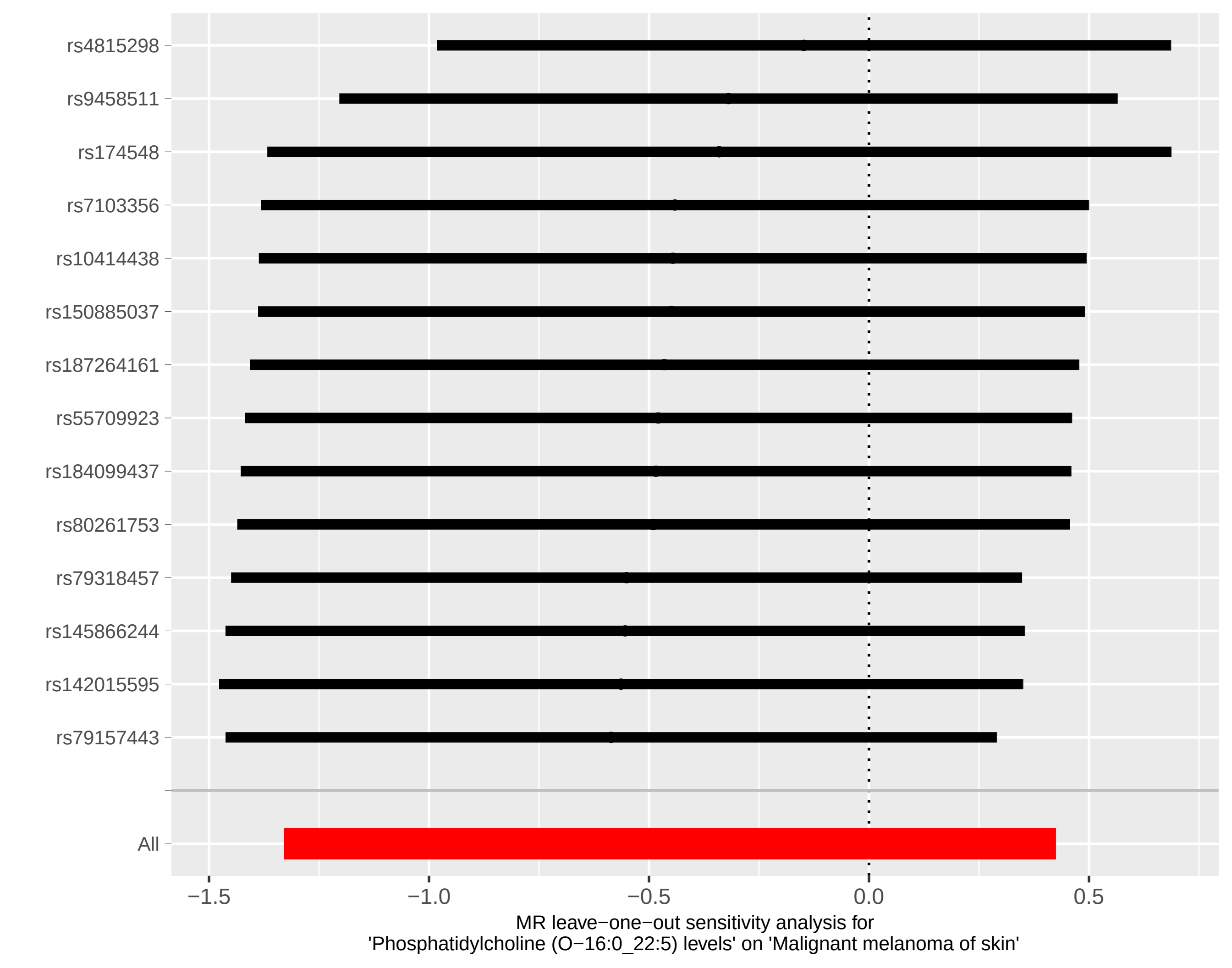
rs4815298
rs9458511
rs174548
rs7103356
rs10414438
rs150885037
rs187264161
rs55709923
rs184099437
rs80261753
rs79318457
rs145866244
rs142015595
rs79157443
All
−1.5
−1.0
−0.5
0.0
0.5
MR leave−one−out sensitivity analysis for
'Phosphatidylcholine (O−16:0_22:5) levels' on 'Malignant melanoma of skin'

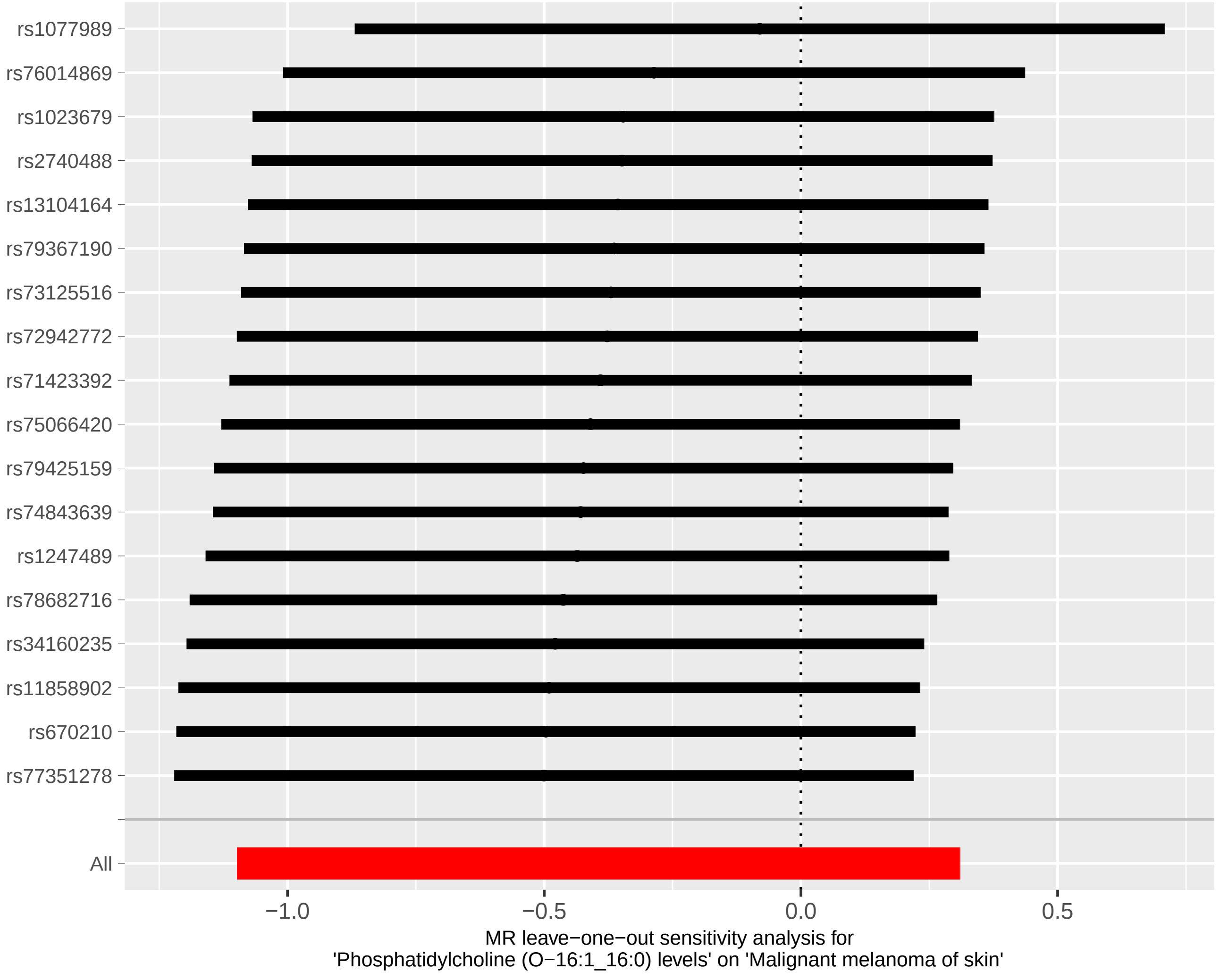

rs1077989
rs76014869
rs1023679
rs2740488
rs13104164
rs79367190
rs73125516
rs72942772
rs71423392
rs75066420
rs79425159
rs74843639
rs1247489
rs78682716
rs34160235
rs11858902
rs670210
rs77351278
All
−1.0
−0.5
0.0
0.5
MR leave−one−out sensitivity analysis for
'Phosphatidylcholine (O−16:1_16:0) levels' on 'Malignant melanoma of skin'

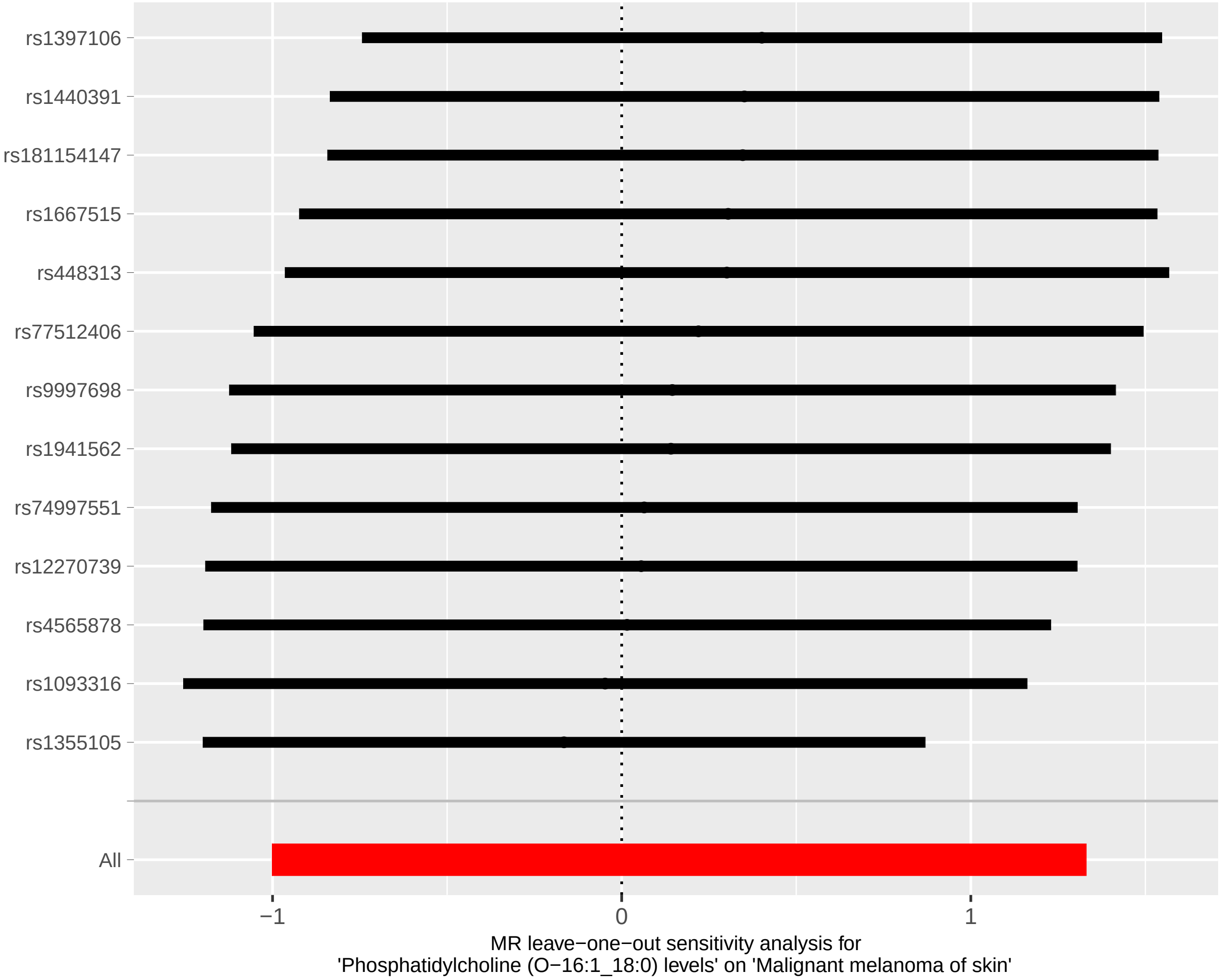
rs1397106
rs1440391
rs181154147
rs1667515
rs448313
rs77512406
rs9997698
rs1941562
rs74997551
rs12270739
rs4565878
rs1093316
rs1355105
All
−1
0
1
MR leave−one−out sensitivity analysis for
'Phosphatidylcholine (O−16:1_18:0) levels' on 'Malignant melanoma of skin'

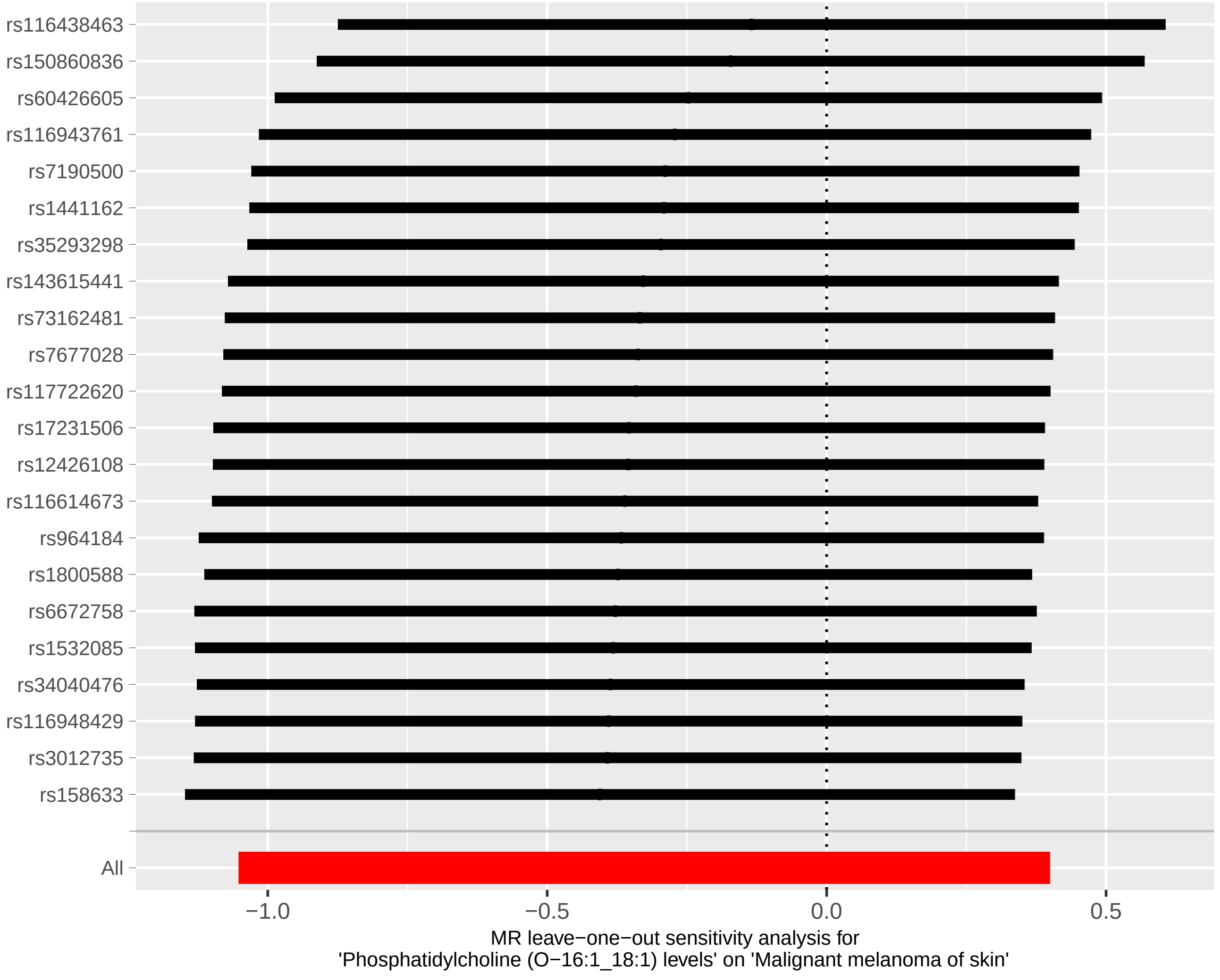
rs116438463
rs150860836
rs60426605
rs116943761
rs7190500
rs1441162
rs35293298
rs143615441
rs73162481
rs7677028
rs117722620
rs17231506
rs12426108
rs116614673
rs964184
rs1800588
rs6672758
rs1532085
rs34040476
rs116948429
rs3012735
rs158633
All
−1.0
−0.5
0.0
0.5
MR leave−one−out sensitivity analysis for
'Phosphatidylcholine (O−16:1_18:1) levels' on 'Malignant melanoma of skin'

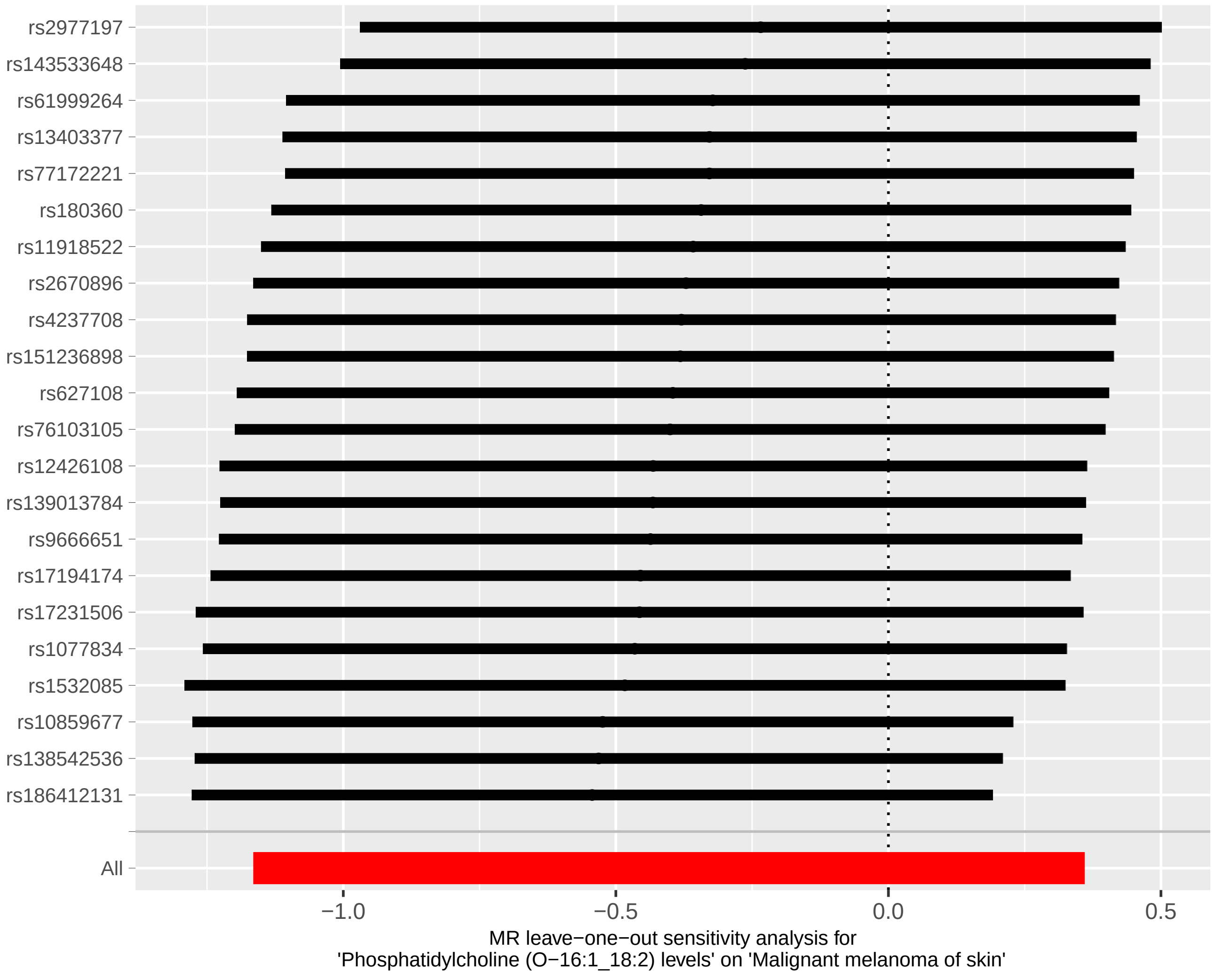
rs2977197
rs143533648
rs61999264
rs13403377
rs77172221
rs180360
rs11918522
rs2670896
rs4237708
rs151236898
rs627108
rs76103105
rs12426108
rs139013784
rs9666651
rs17194174
rs17231506
rs1077834
rs1532085
rs10859677
rs138542536
rs186412131
All
−1.0
−0.5
0.0
0.5
MR leave−one−out sensitivity analysis for
'Phosphatidylcholine (O−16:1_18:2) levels' on 'Malignant melanoma of skin'

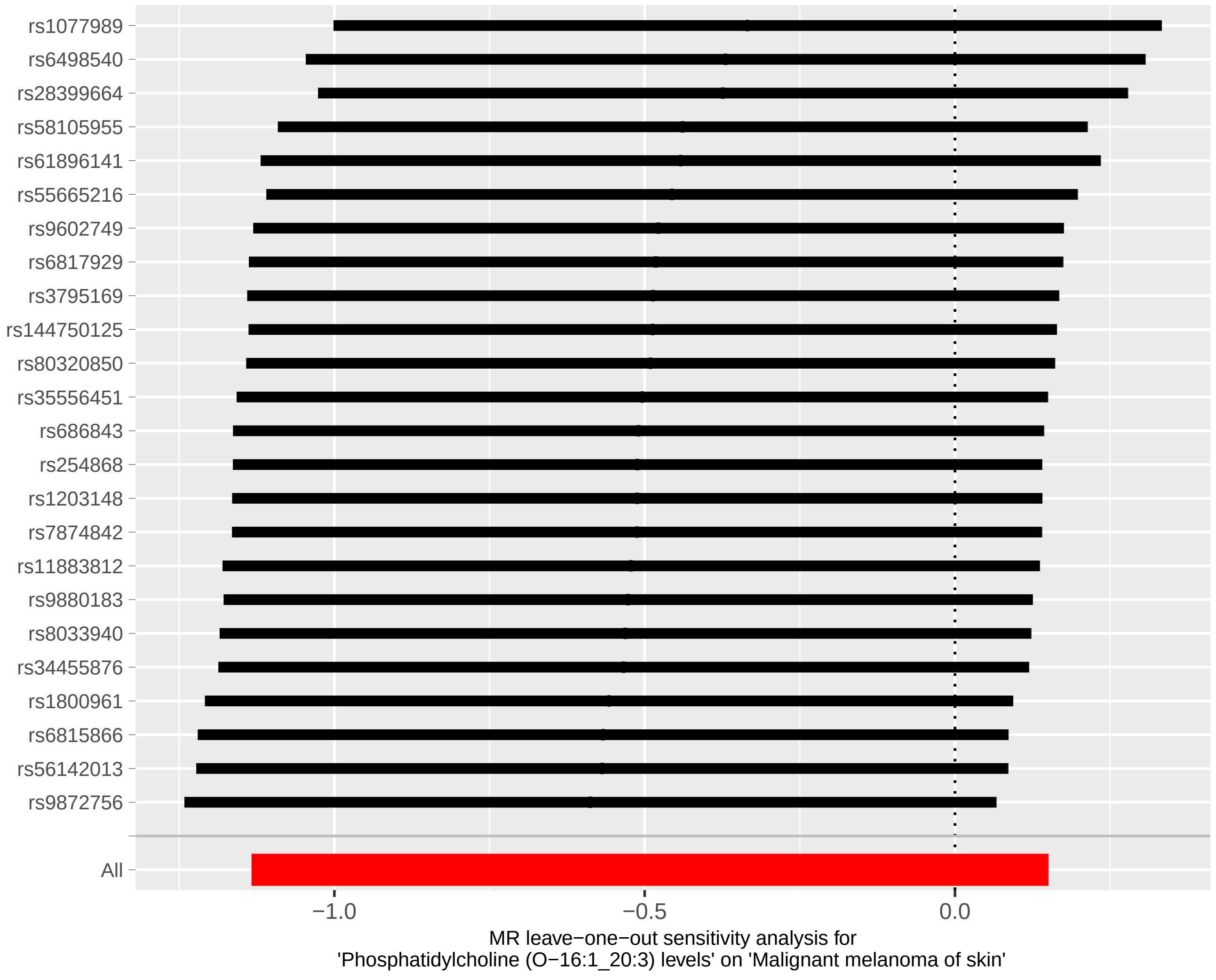

rs1077989
rs6498540
rs28399664
rs58105955
rs61896141
rs55665216
rs9602749
rs6817929
rs3795169
rs144750125
rs80320850
rs35556451
rs686843
rs254868
rs1203148
rs7874842
rs11883812
rs9880183
rs8033940
rs34455876
rs1800961
rs6815866
rs56142013
rs9872756
All
−1.0
−0.5
0.0
MR leave−one−out sensitivity analysis for
'Phosphatidylcholine (O−16:1_20:3) levels' on 'Malignant melanoma of skin'

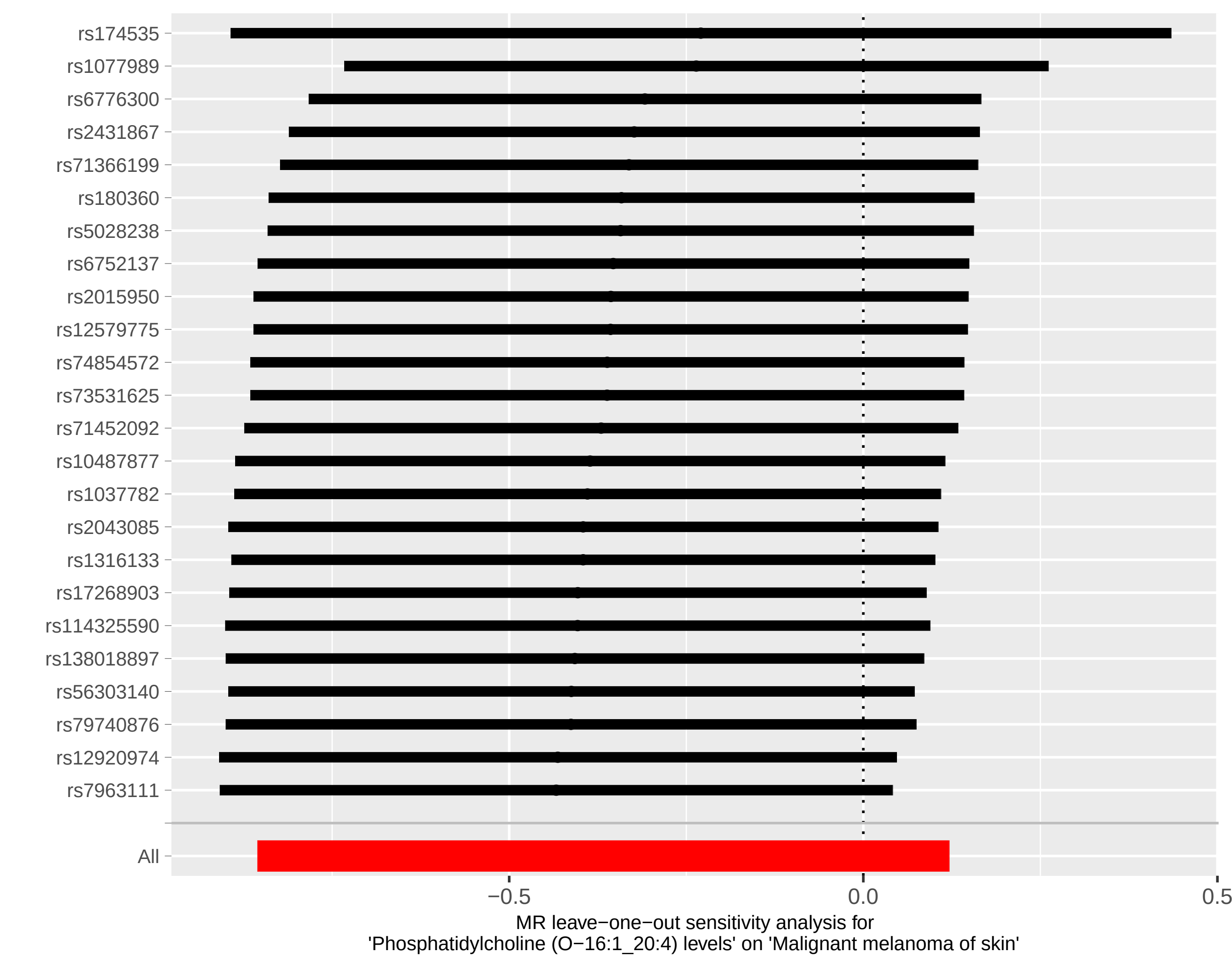

rs174535
rs1077989
rs6776300
rs2431867
rs71366199
rs180360
rs5028238
rs6752137
rs2015950
rs12579775
rs74854572
rs73531625
rs71452092
rs10487877
rs1037782
rs2043085
rs1316133
rs17268903
rs114325590
rs138018897
rs56303140
rs79740876
rs12920974
rs7963111
All
−0.5
0.0
0.5
MR leave−one−out sensitivity analysis for
'Phosphatidylcholine (O−16:1_20:4) levels' on 'Malignant melanoma of skin'

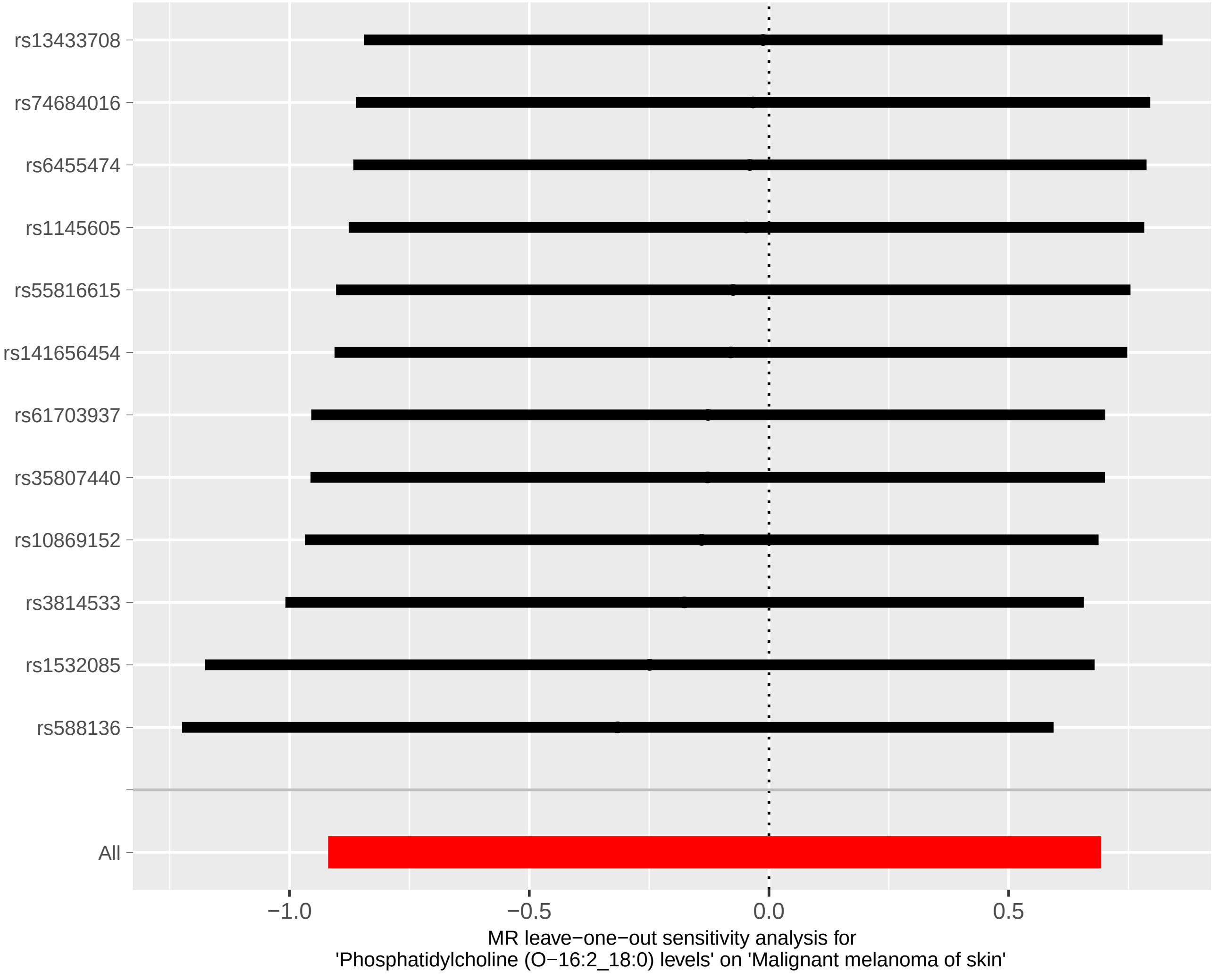
rs13433708
rs74684016
rs6455474
rs1145605
rs55816615
rs141656454
rs61703937
rs35807440
rs10869152
rs3814533
rs1532085
rs588136
All
−1.0
−0.5
0.0
0.5
MR leave−one−out sensitivity analysis for
'Phosphatidylcholine (O−16:2_18:0) levels' on 'Malignant melanoma of skin'

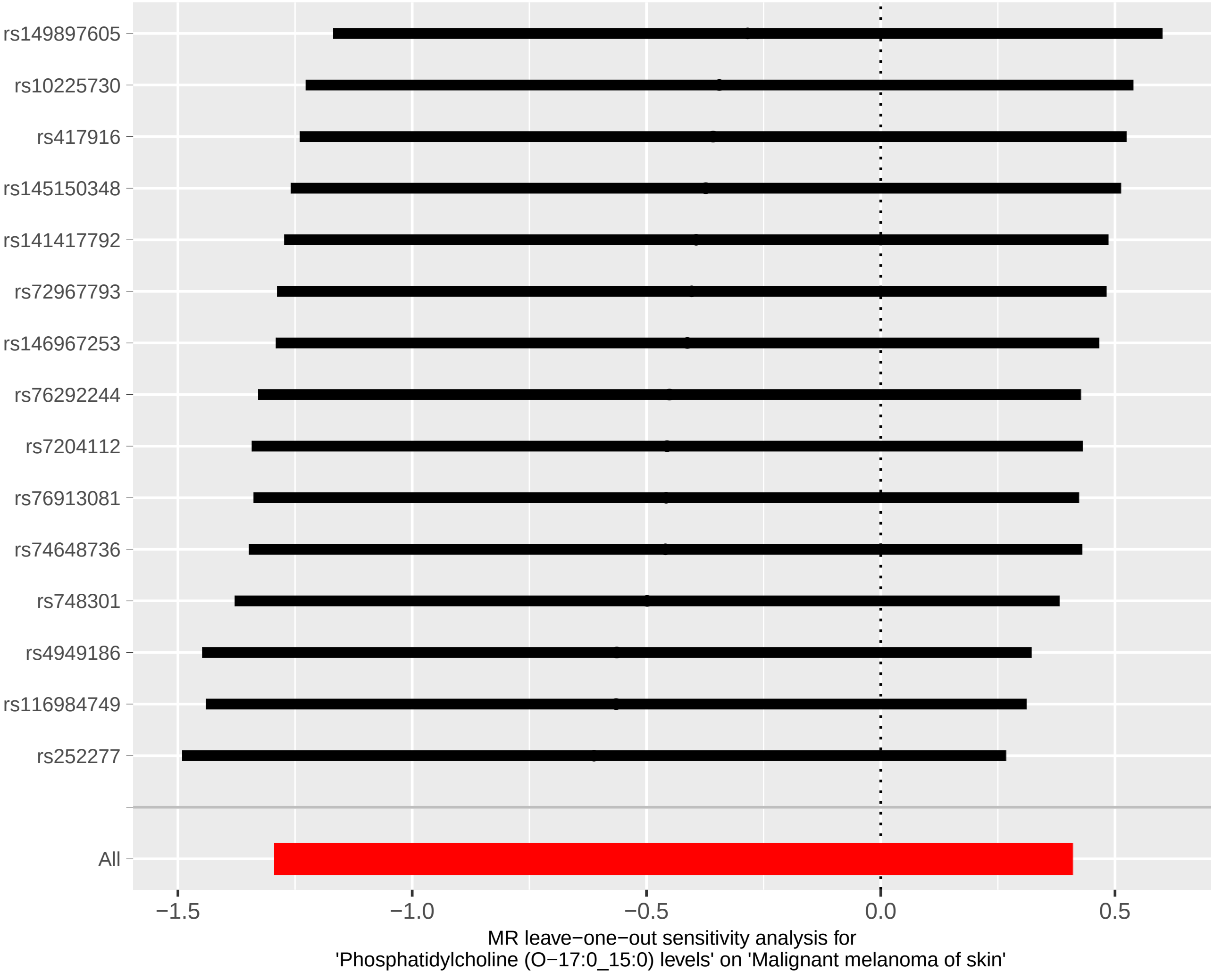
rs149897605
rs10225730
rs417916
rs145150348
rs141417792
rs72967793
rs146967253
rs76292244
rs7204112
rs76913081
rs74648736
rs748301
rs4949186
rs116984749
rs252277
All
−1.5
−1.0
−0.5
0.0
0.5
MR leave−one−out sensitivity analysis for
'Phosphatidylcholine (O−17:0_15:0) levels' on 'Malignant melanoma of skin'

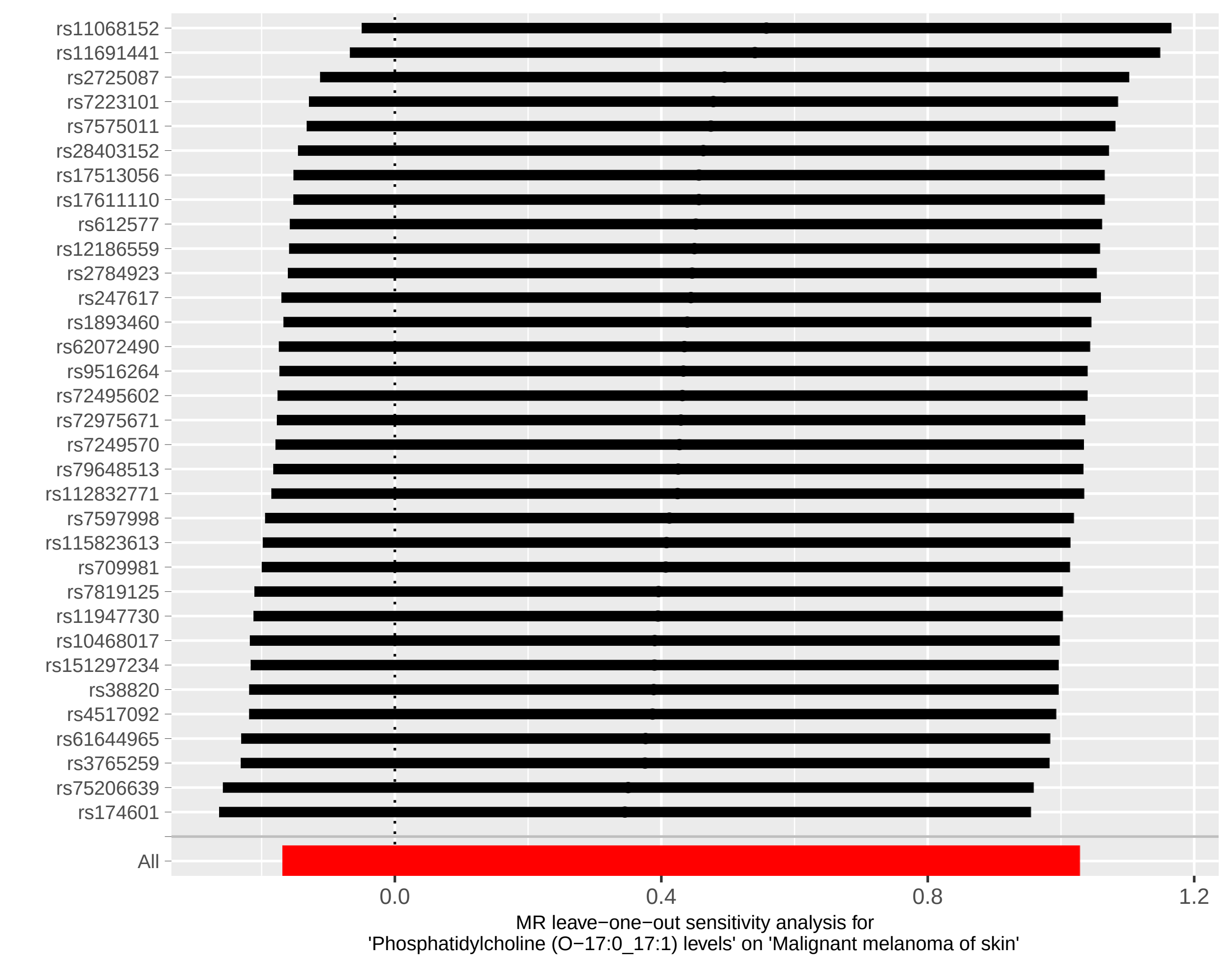
rs11068152
rs11691441
rs2725087
rs7223101
rs7575011
rs28403152
rs17513056
rs17611110
rs612577
rs12186559
rs2784923
rs247617
rs1893460
rs62072490
rs9516264
rs72495602
rs72975671
rs7249570
rs79648513
rs112832771
rs7597998
rs115823613
rs709981
rs7819125
rs11947730
rs10468017
rs151297234
rs38820
rs4517092
rs61644965
rs3765259
rs75206639
rs174601
All
0.0
0.4
0.8
1.2
MR leave−one−out sensitivity analysis for
'Phosphatidylcholine (O−17:0_17:1) levels' on 'Malignant melanoma of skin'

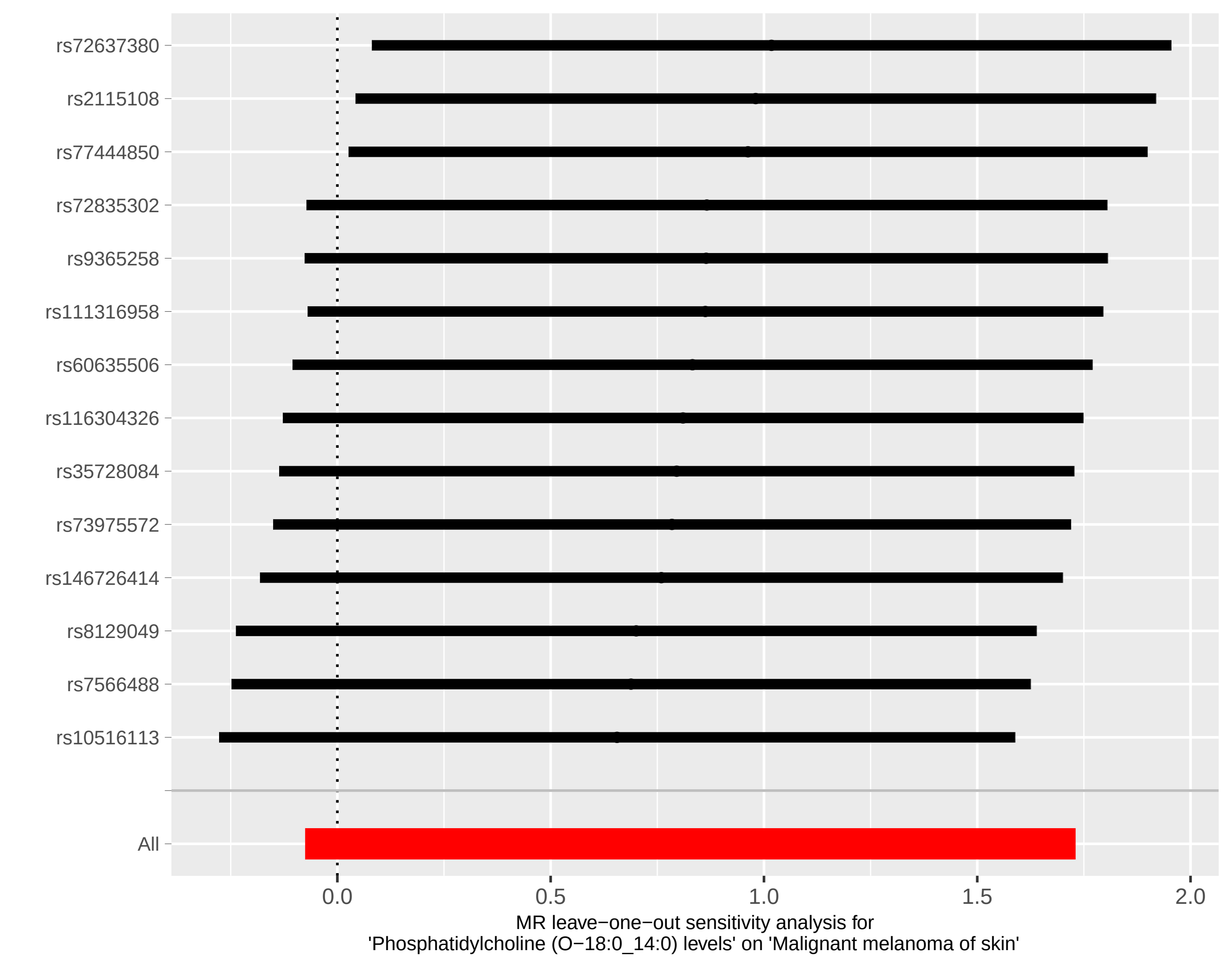
rs72637380
rs2115108
rs77444850
rs72835302
rs9365258
rs111316958
rs60635506
rs116304326
rs35728084
rs73975572
rs146726414
rs8129049
rs7566488
rs10516113
All
0.0
0.5
1.0
1.5
2.0
MR leave−one−out sensitivity analysis for
'Phosphatidylcholine (O−18:0_14:0) levels' on 'Malignant melanoma of skin'

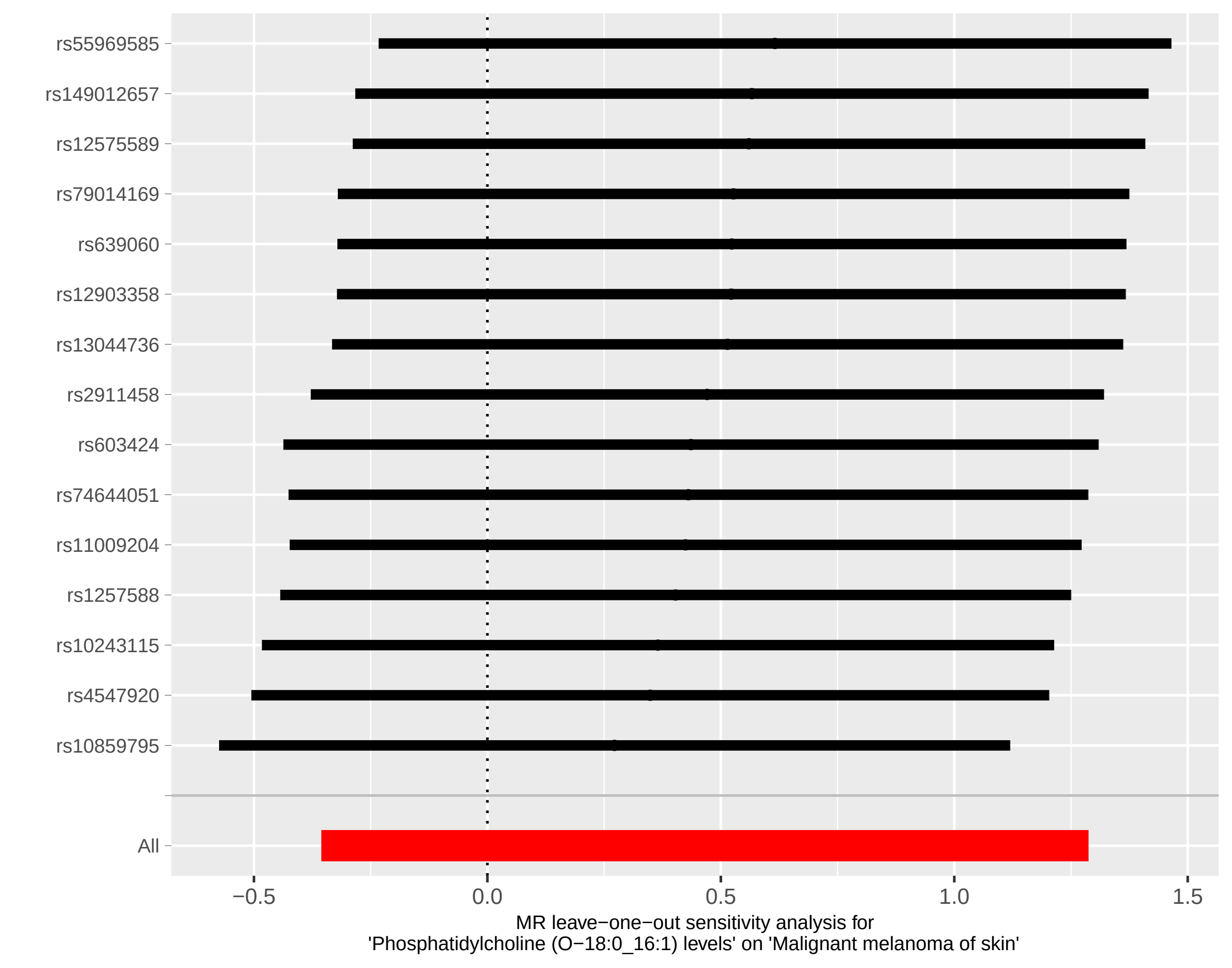

rs55969585
rs149012657
rs12575589
rs79014169
rs639060
rs12903358
rs13044736
rs2911458
rs603424
rs74644051
rs11009204
rs1257588
rs10243115
rs4547920
rs10859795
All
−0.5
0.0
0.5
1.0
1.5
MR leave−one−out sensitivity analysis for
'Phosphatidylcholine (O−18:0_16:1) levels' on 'Malignant melanoma of skin'

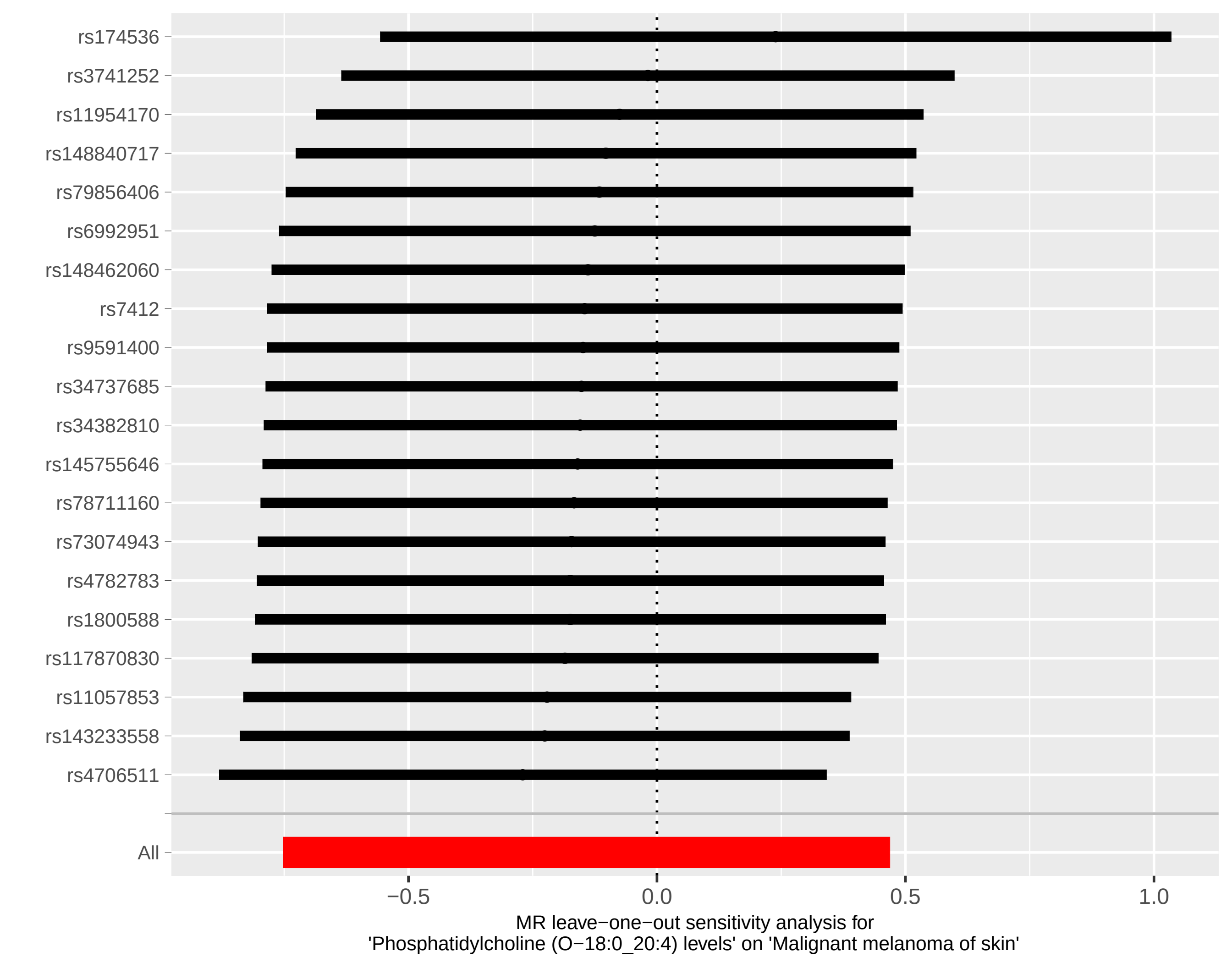
rs174536
rs3741252
rs11954170
rs148840717
rs79856406
rs6992951
rs148462060
rs7412
rs9591400
rs34737685
rs34382810
rs145755646
rs78711160
rs73074943
rs4782783
rs1800588
rs117870830
rs11057853
rs143233558
rs4706511
All
−0.5
0.0
0.5
1.0
MR leave−one−out sensitivity analysis for
'Phosphatidylcholine (O−18:0_20:4) levels' on 'Malignant melanoma of skin'

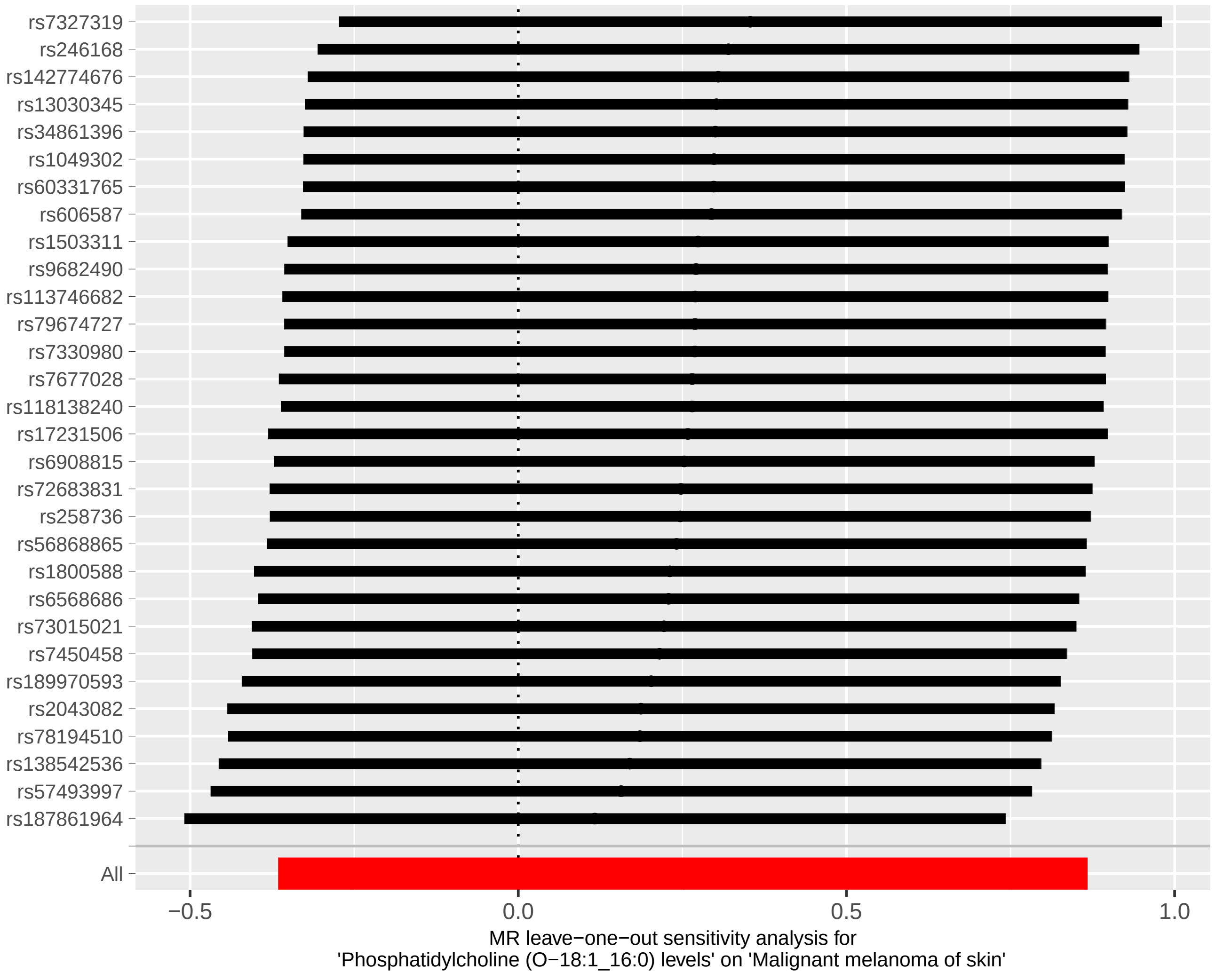
rs7327319
rs246168
rs142774676
rs13030345
rs34861396
rs1049302
rs60331765
rs606587
rs1503311
rs9682490
rs113746682
rs79674727
rs7330980
rs7677028
rs118138240
rs17231506
rs6908815
rs72683831
rs258736
rs56868865
rs1800588
rs6568686
rs73015021
rs7450458
rs189970593
rs2043082
rs78194510
rs138542536
rs57493997
rs187861964
All
−0.5
0.0
0.5
1.0
MR leave−one−out sensitivity analysis for
'Phosphatidylcholine (O−18:1_16:0) levels' on 'Malignant melanoma of skin'

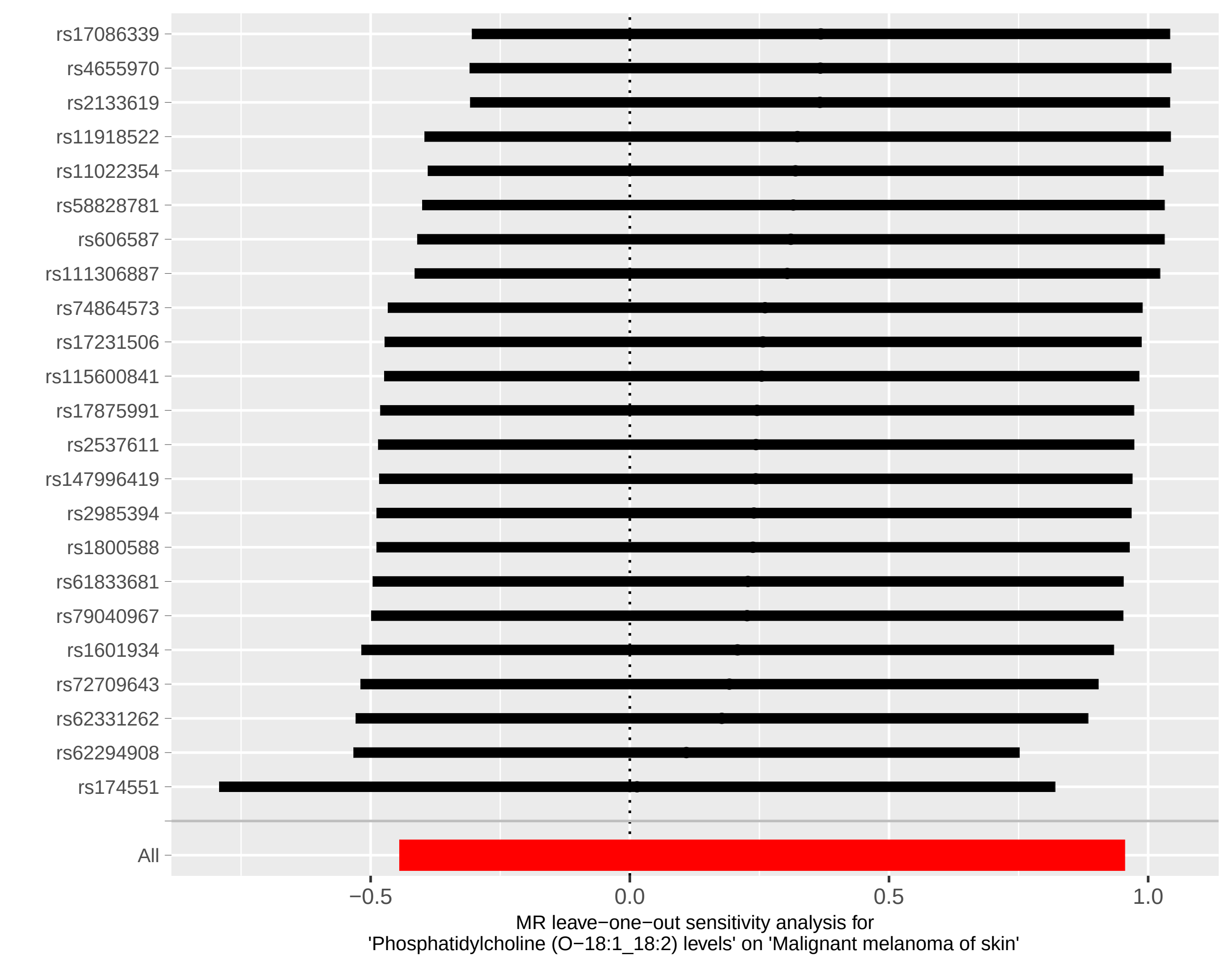

rs17086339
rs4655970
rs2133619
rs11918522
rs11022354
rs58828781
rs606587
rs111306887
rs74864573
rs17231506
rs115600841
rs17875991
rs2537611
rs147996419
rs2985394
rs1800588
rs61833681
rs79040967
rs1601934
rs72709643
rs62331262
rs62294908
rs174551
All
−0.5
0.0
0.5
1.0
MR leave−one−out sensitivity analysis for
'Phosphatidylcholine (O−18:1_18:2) levels' on 'Malignant melanoma of skin'

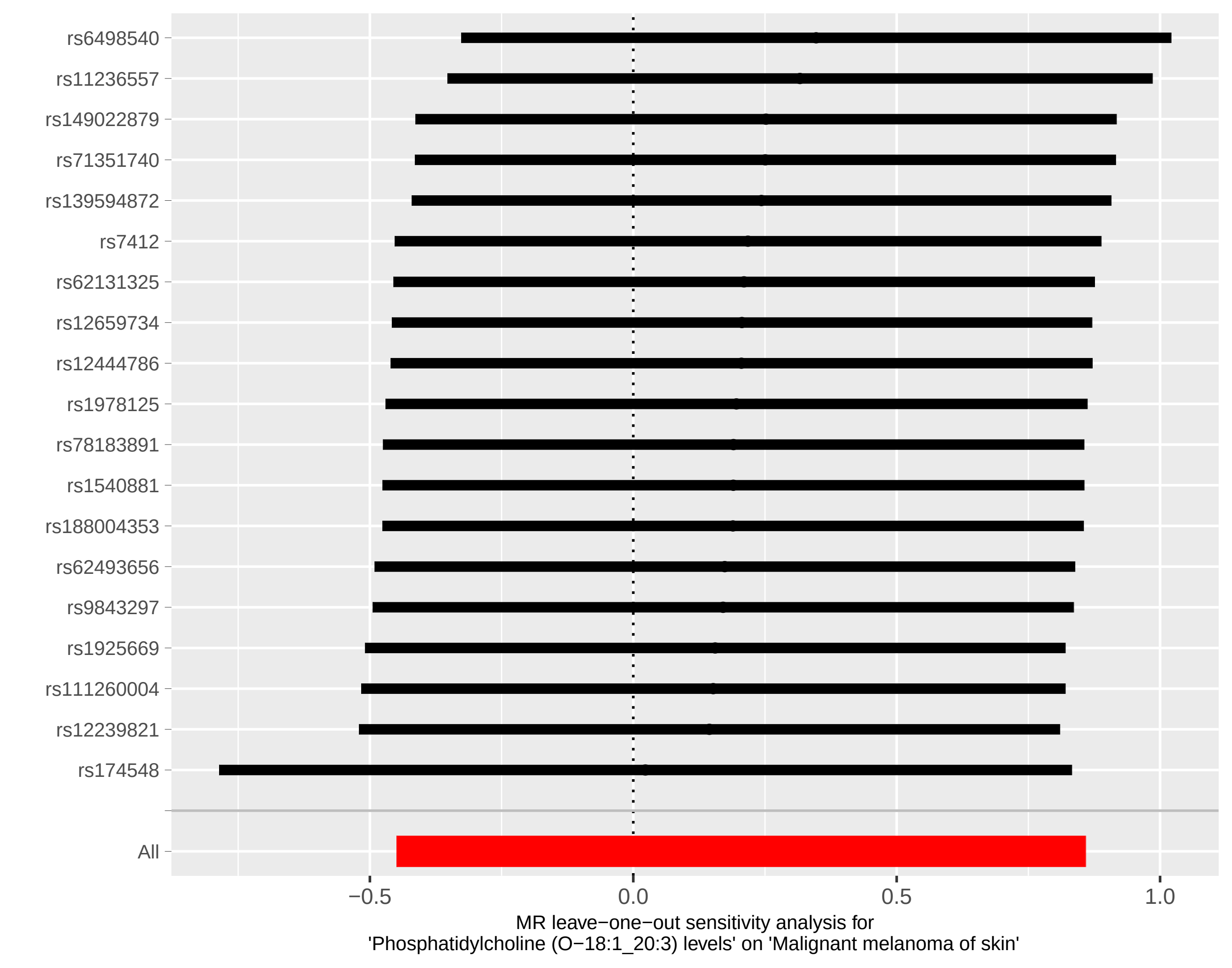
rs6498540
rs11236557
rs149022879
rs71351740
rs139594872
rs7412
rs62131325
rs12659734
rs12444786
rs1978125
rs78183891
rs1540881
rs188004353
rs62493656
rs9843297
rs1925669
rs111260004
rs12239821
rs174548
All
−0.5
0.0
0.5
1.0
MR leave−one−out sensitivity analysis for
'Phosphatidylcholine (O−18:1_20:3) levels' on 'Malignant melanoma of skin'

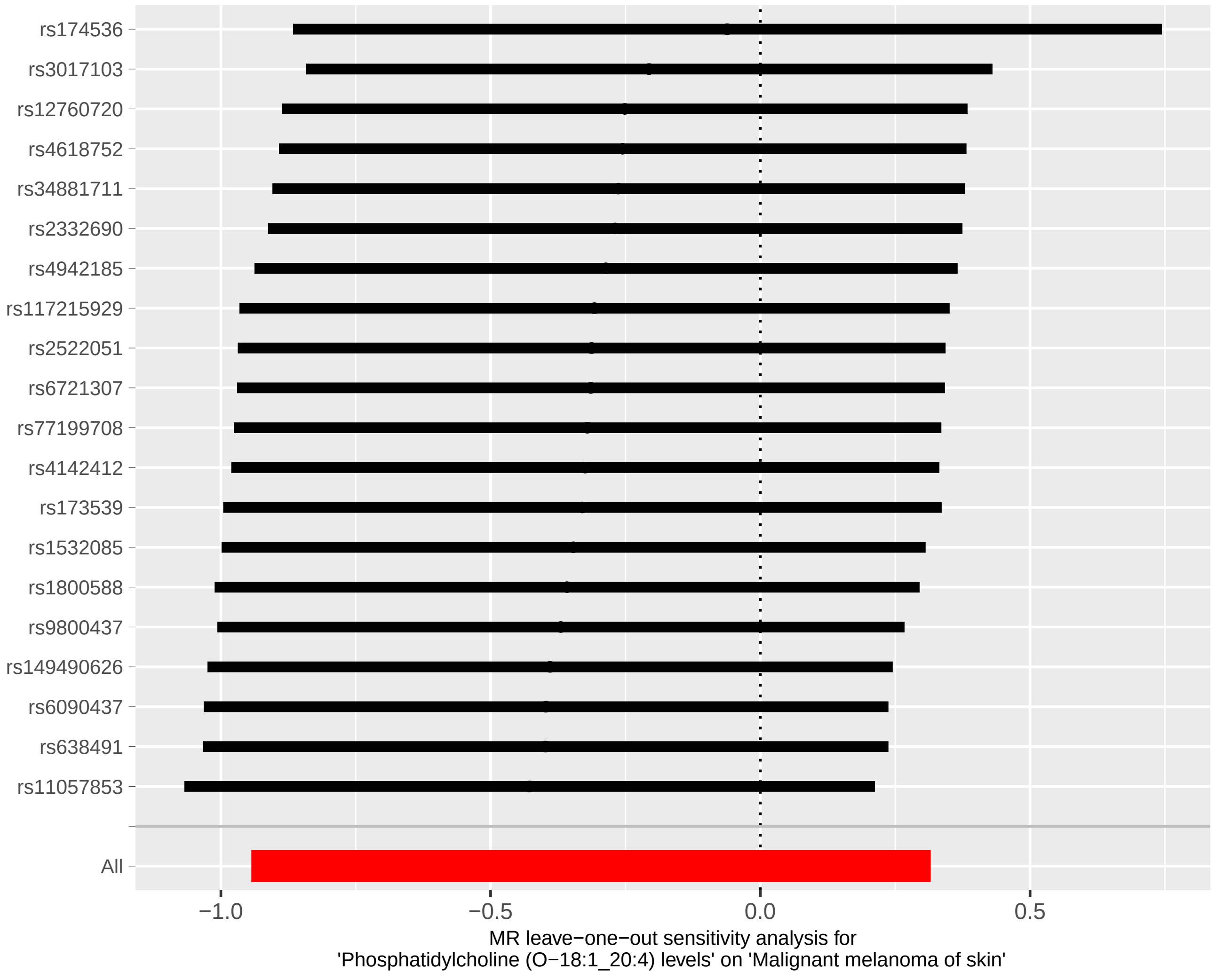
rs174536
rs3017103
rs12760720
rs4618752
rs34881711
rs2332690
rs4942185
rs117215929
rs2522051
rs6721307
rs77199708
rs4142412
rs173539
rs1532085
rs1800588
rs9800437
rs149490626
rs6090437
rs638491
rs11057853
All
−1.0
−0.5
0.0
0.5
MR leave−one−out sensitivity analysis for
'Phosphatidylcholine (O−18:1_20:4) levels' on 'Malignant melanoma of skin'

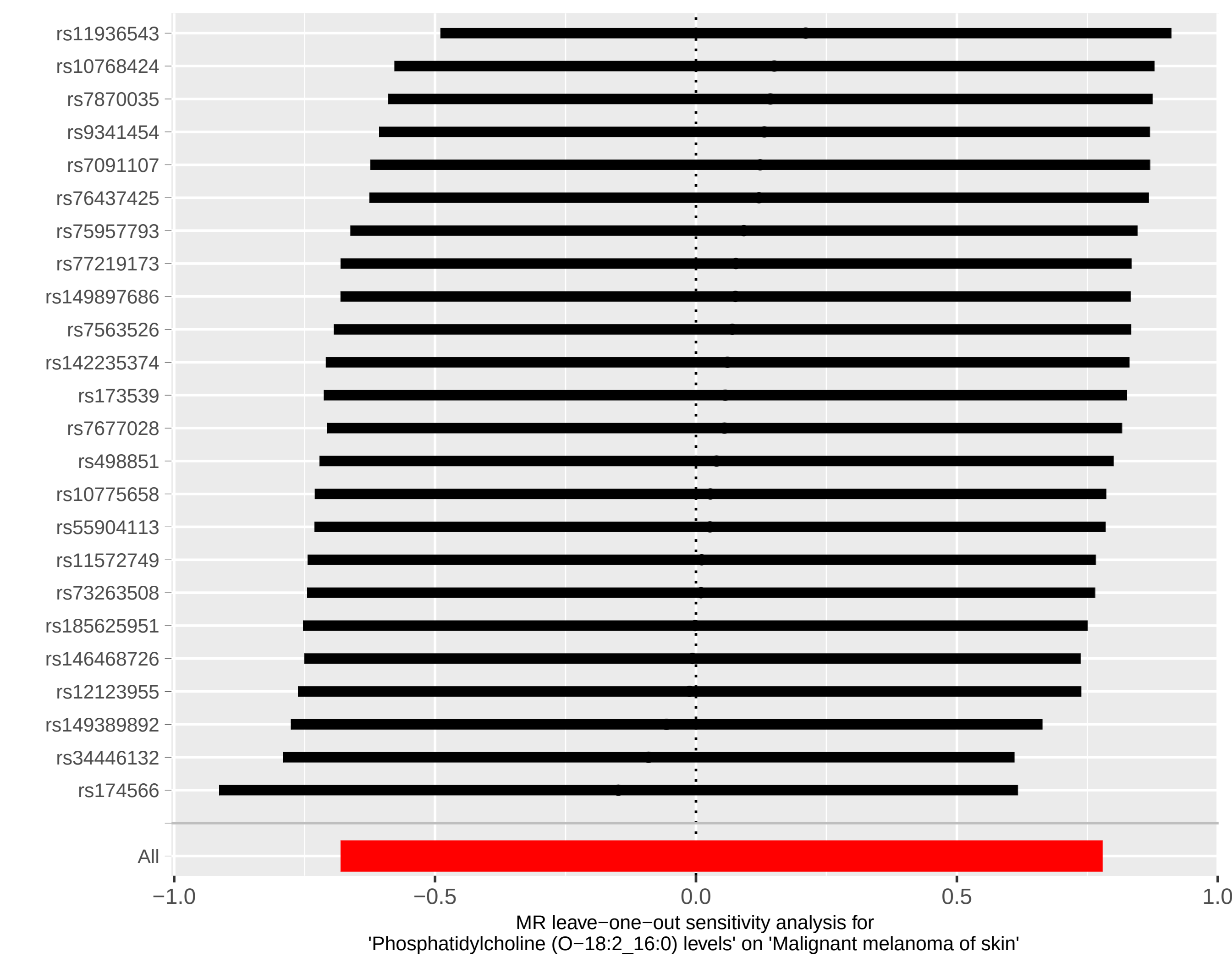
rs11936543
rs10768424
rs7870035
rs9341454
rs7091107
rs76437425
rs75957793
rs77219173
rs149897686
rs7563526
rs142235374
rs173539
rs7677028
rs498851
rs10775658
rs55904113
rs11572749
rs73263508
rs185625951
rs146468726
rs12123955
rs149389892
rs34446132
rs174566
All
−1.0
−0.5
0.0
0.5
1.0
MR leave−one−out sensitivity analysis for
'Phosphatidylcholine (O−18:2_16:0) levels' on 'Malignant melanoma of skin'

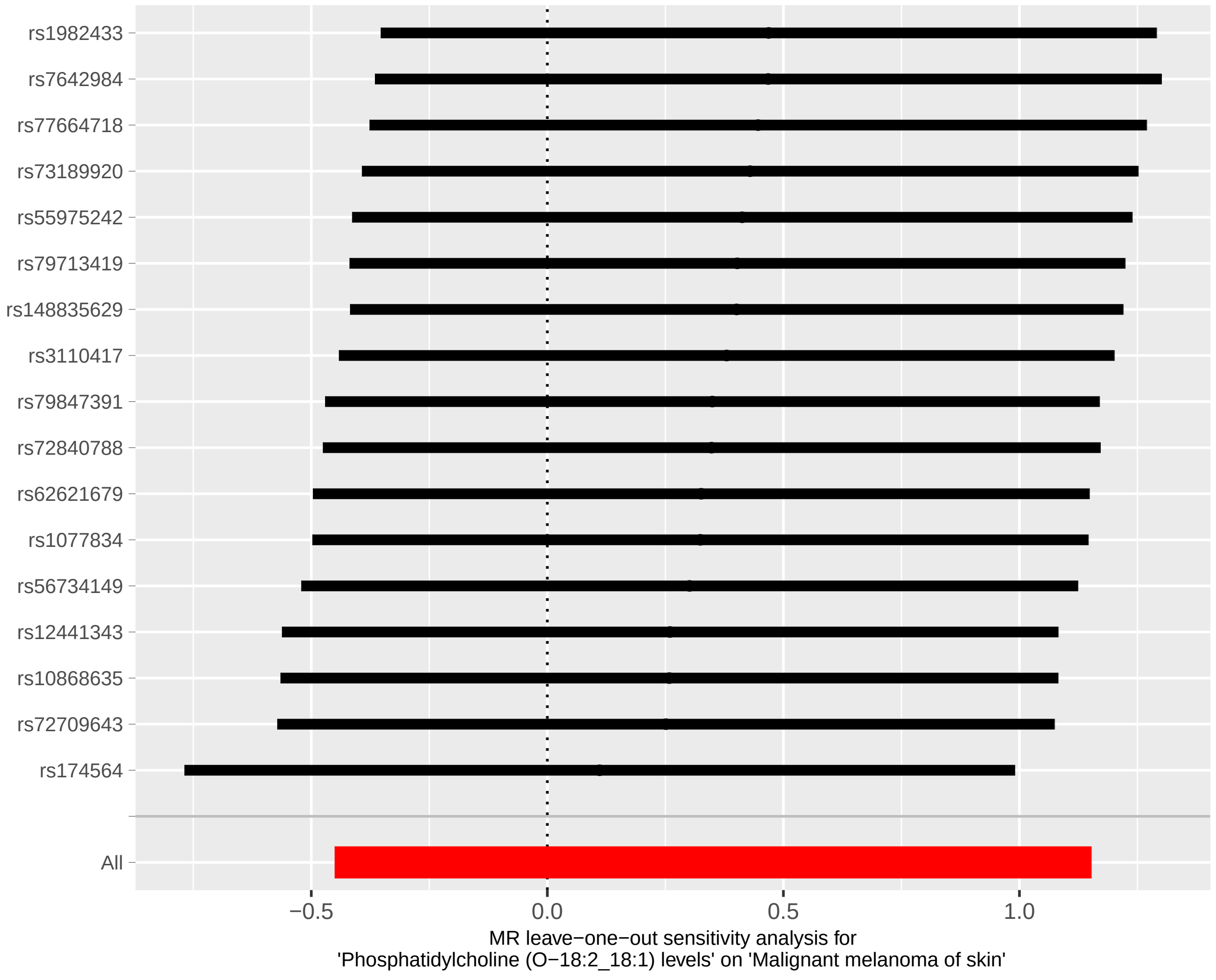

rs1982433
rs7642984
rs77664718
rs73189920
rs55975242
rs79713419
rs148835629
rs3110417
rs79847391
rs72840788
rs62621679
rs1077834
rs56734149
rs12441343
rs10868635
rs72709643
rs174564
All
−0.5
0.0
0.5
1.0
MR leave−one−out sensitivity analysis for
'Phosphatidylcholine (O−18:2_18:1) levels' on 'Malignant melanoma of skin'

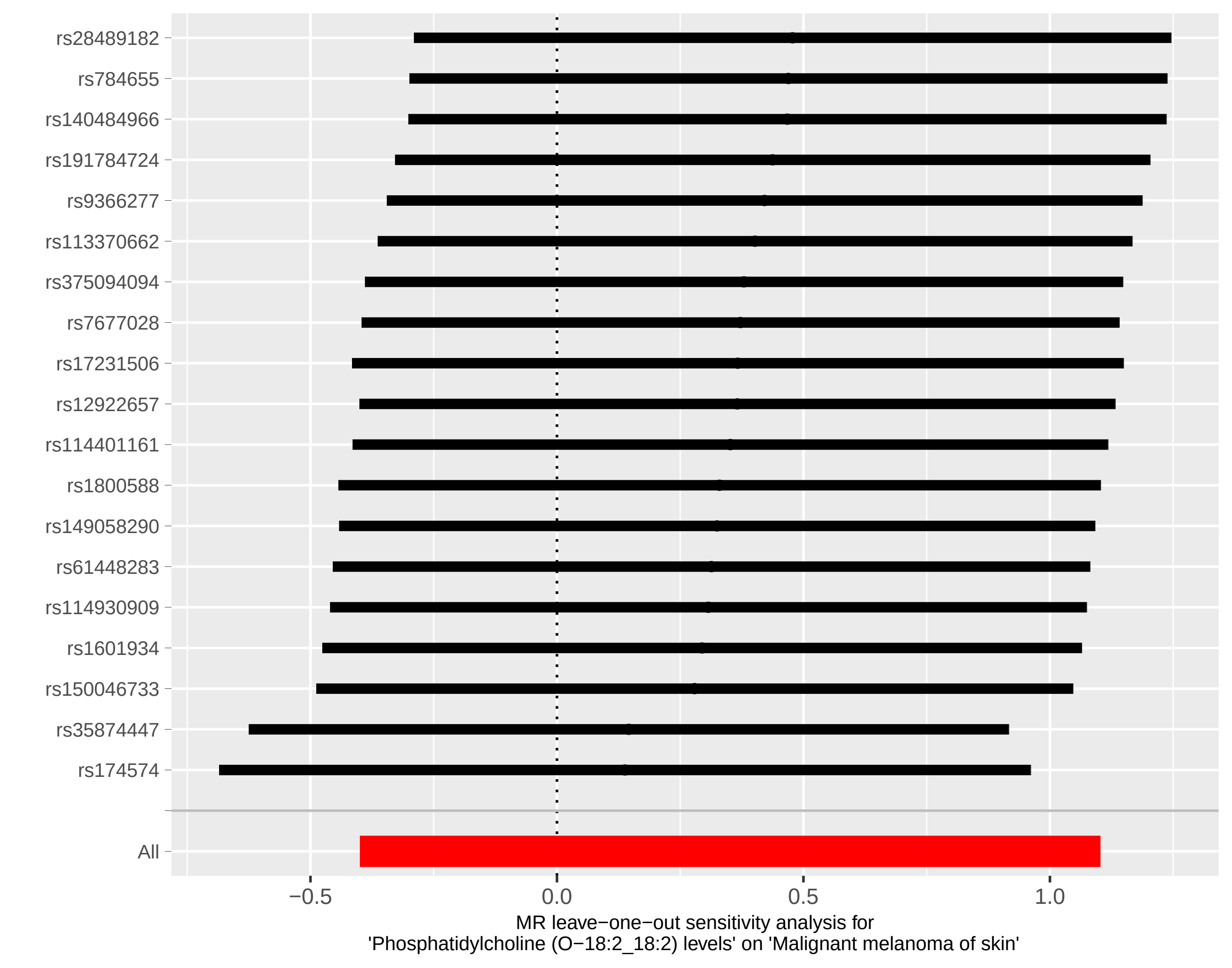

rs28489182
rs784655
rs140484966
rs191784724
rs9366277
rs113370662
rs375094094
rs7677028
rs17231506
rs12922657
rs114401161
rs1800588
rs149058290
rs61448283
rs114930909
rs1601934
rs150046733
rs35874447
rs174574
All
−0.5
0.0
0.5
1.0
MR leave−one−out sensitivity analysis for
'Phosphatidylcholine (O−18:2_18:2) levels' on 'Malignant melanoma of skin'

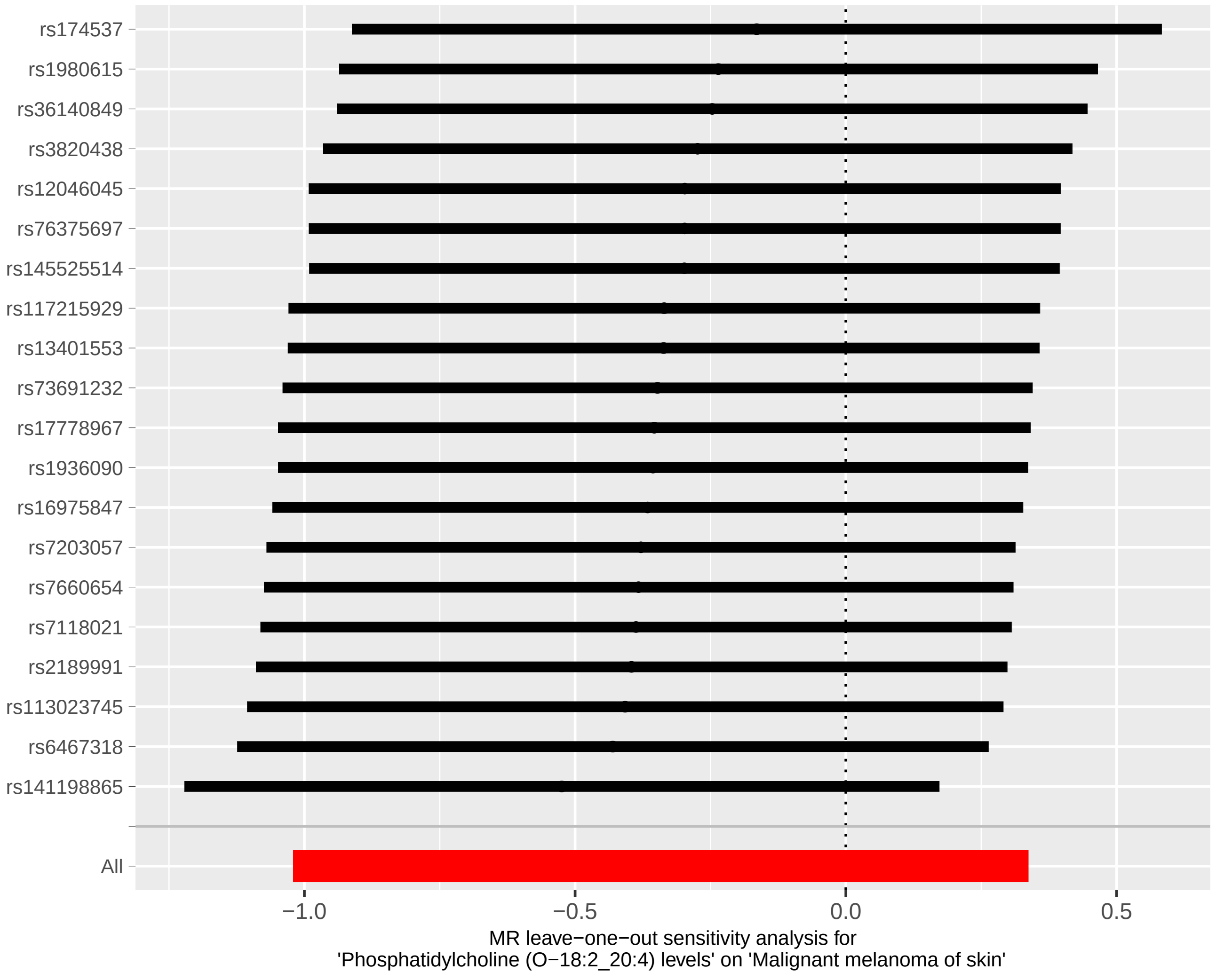
rs174537
rs1980615
rs36140849
rs3820438
rs12046045
rs76375697
rs145525514
rs117215929
rs13401553
rs73691232
rs17778967
rs1936090
rs16975847
rs7203057
rs7660654
rs7118021
rs2189991
rs113023745
rs6467318
rs141198865
All
−1.0
−0.5
0.0
0.5
MR leave−one−out sensitivity analysis for
'Phosphatidylcholine (O−18:2_20:4) levels' on 'Malignant melanoma of skin'

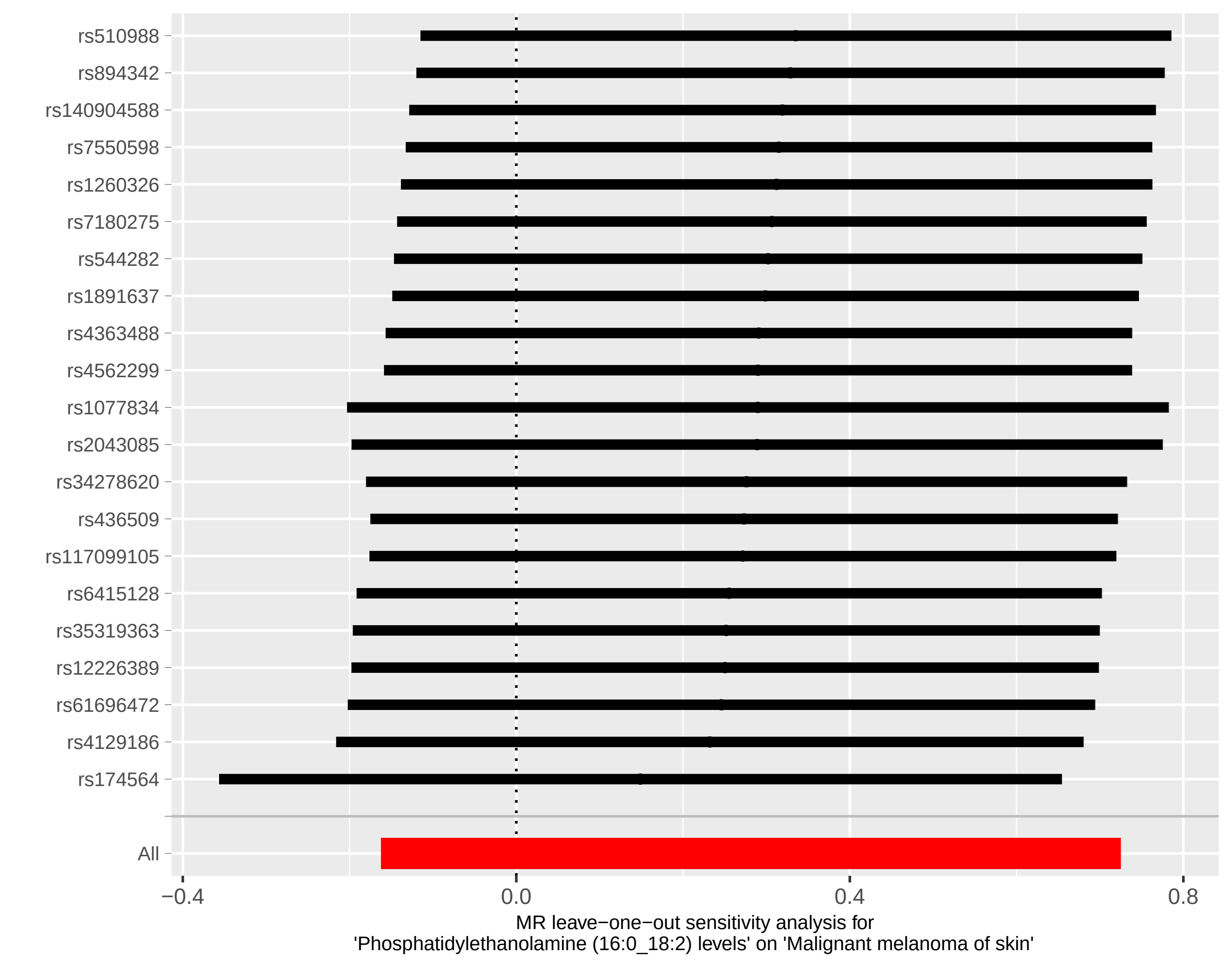
rs510988
rs894342
rs140904588
rs7550598
rs1260326
rs7180275
rs544282
rs1891637
rs4363488
rs4562299
rs1077834
rs2043085
rs34278620
rs436509
rs117099105
rs6415128
rs35319363
rs12226389
rs61696472
rs4129186
rs174564
All
−0.4
0.0
0.4
0.8
MR leave−one−out sensitivity analysis for
'Phosphatidylethanolamine (16:0_18:2) levels' on 'Malignant melanoma of skin'

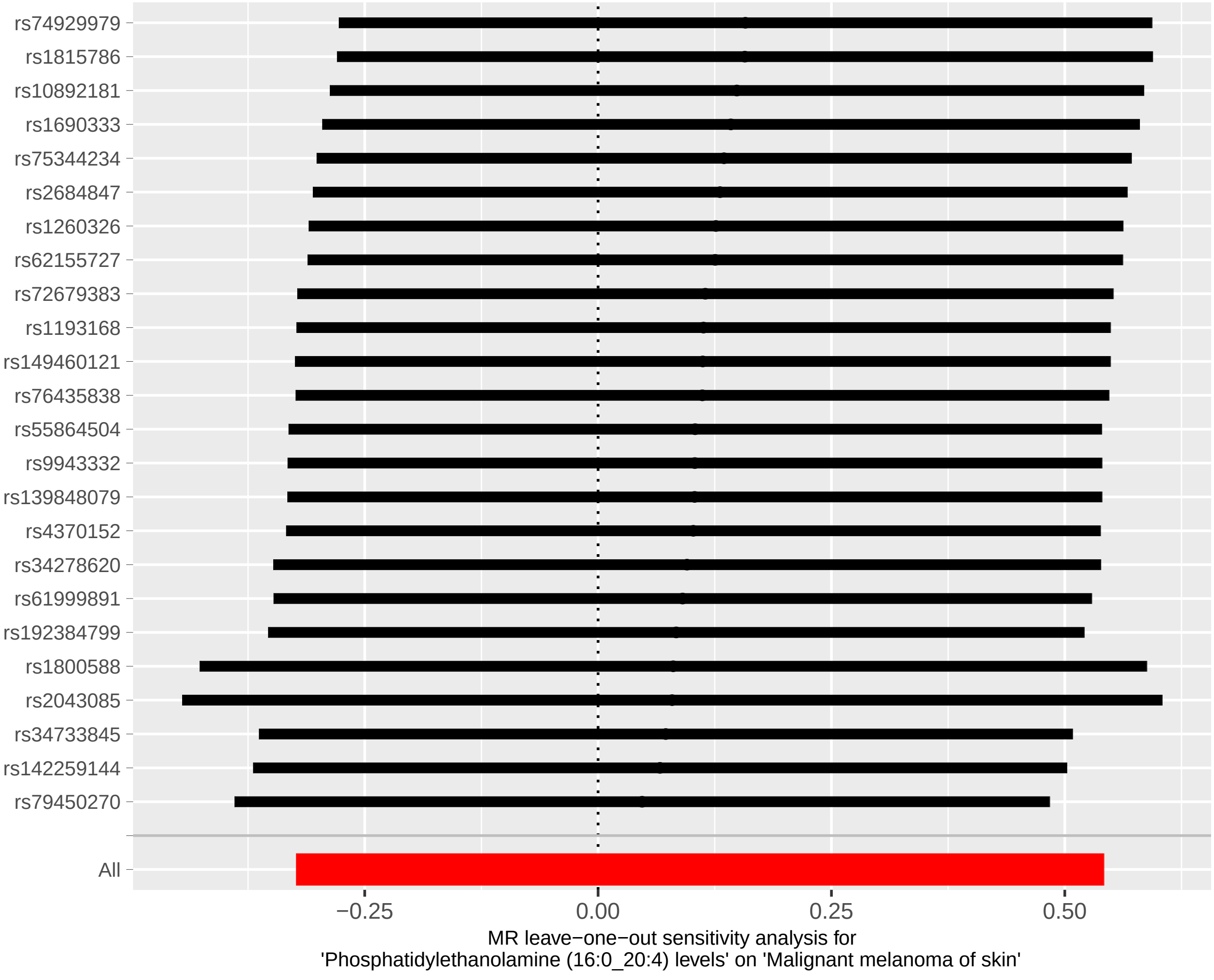
rs74929979
rs1815786
rs10892181
rs1690333
rs75344234
rs2684847
rs1260326
rs62155727
rs72679383
rs1193168
rs149460121
rs76435838
rs55864504
rs9943332
rs139848079
rs4370152
rs34278620
rs61999891
rs192384799
rs1800588
rs2043085
rs34733845
rs142259144
rs79450270
All
−0.25
0.00
0.25
0.50
MR leave−one−out sensitivity analysis for
'Phosphatidylethanolamine (16:0_20:4) levels' on 'Malignant melanoma of skin'

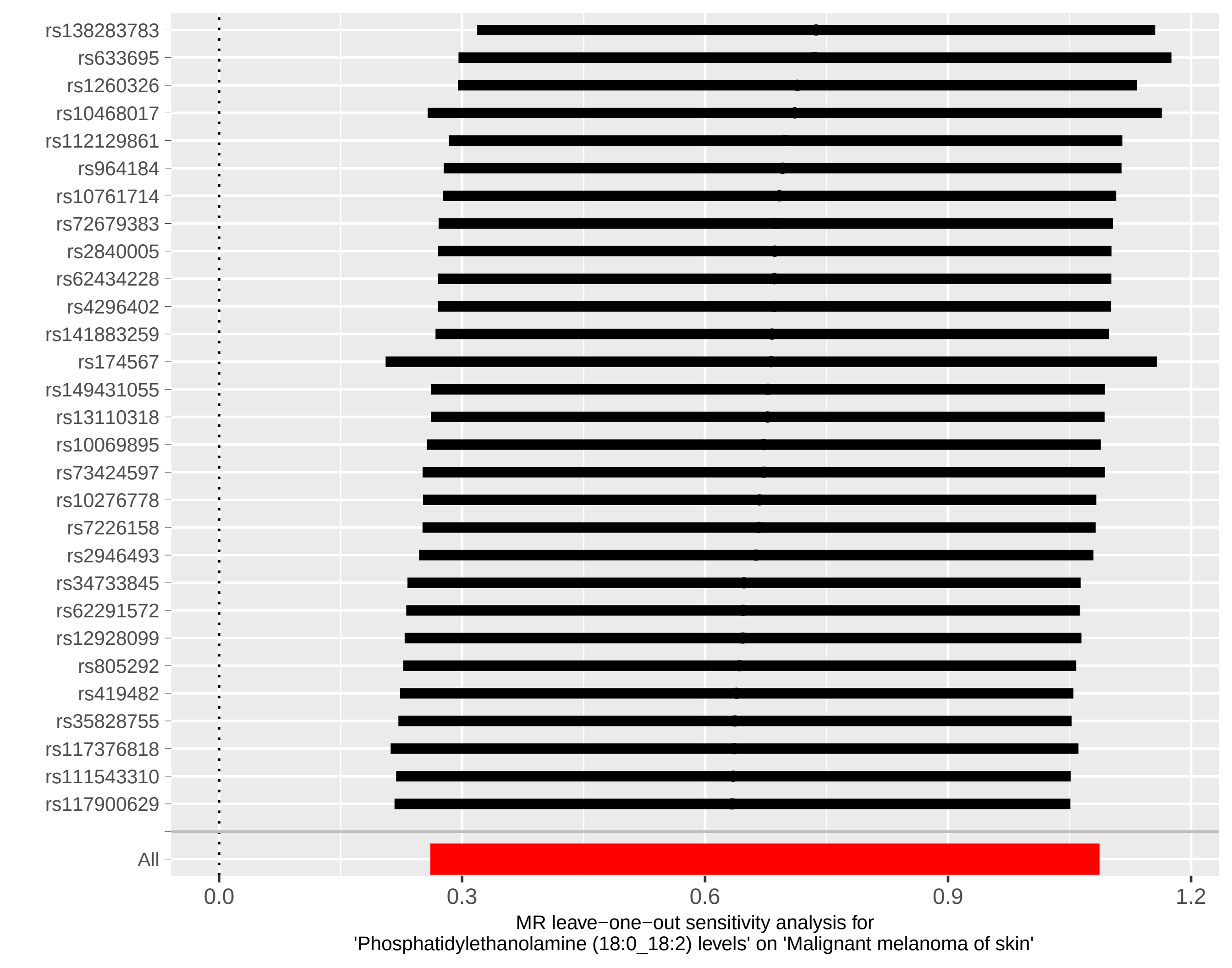
rs138283783
rs633695
rs1260326
rs10468017
rs112129861
rs964184
rs10761714
rs72679383
rs2840005
rs62434228
rs4296402
rs141883259
rs174567
rs149431055
rs13110318
rs10069895
rs73424597
rs10276778
rs7226158
rs2946493
rs34733845
rs62291572
rs12928099
rs805292
rs419482
rs35828755
rs117376818
rs111543310
rs117900629
All
0.0
0.3
0.6
0.9
1.2
MR leave−one−out sensitivity analysis for
'Phosphatidylethanolamine (18:0_18:2) levels' on 'Malignant melanoma of skin'

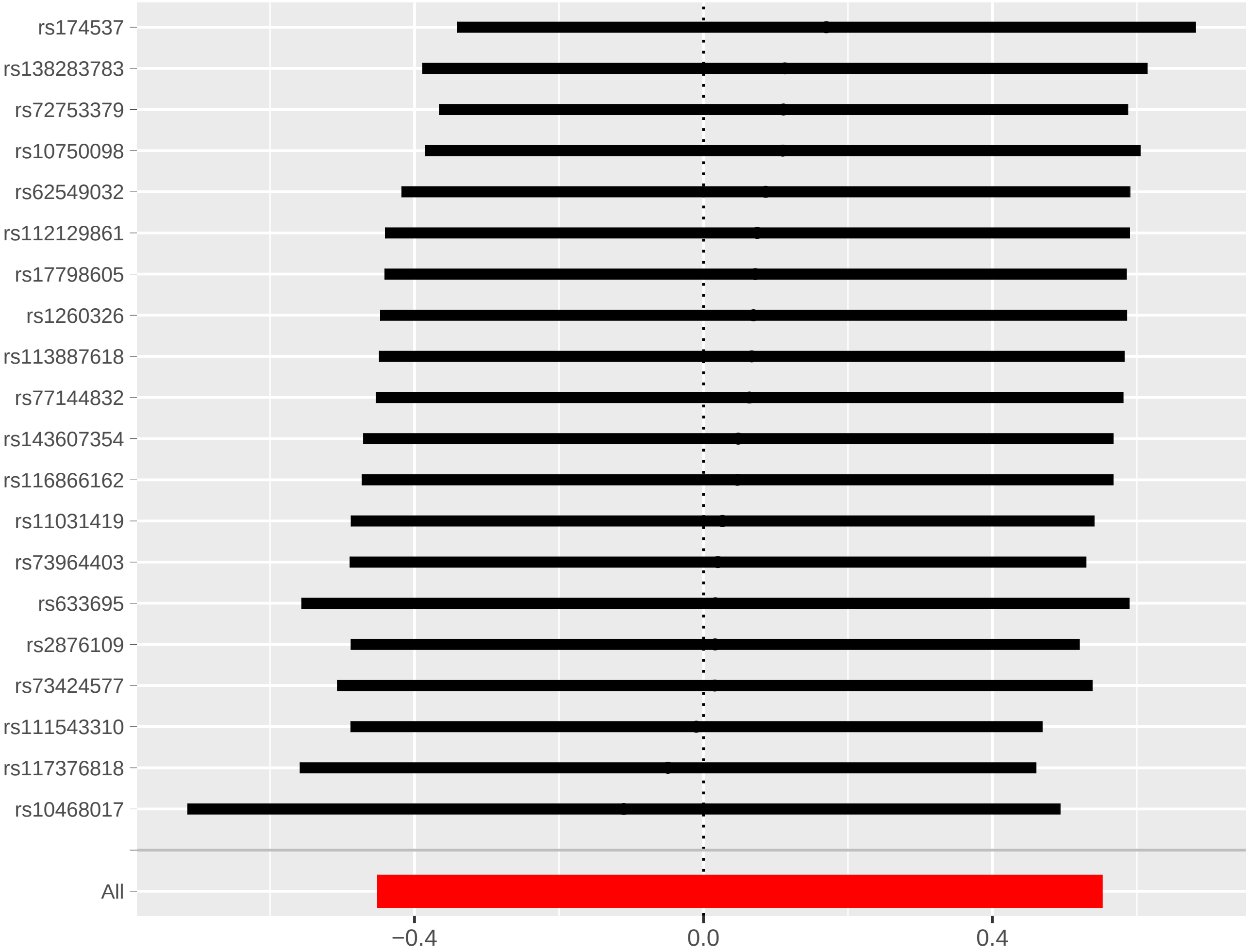

MR leave−one−out sensitivity analysis for
'Phosphatidylethanolamine (18:0_20:4) levels' on 'Malignant melanoma of skin'

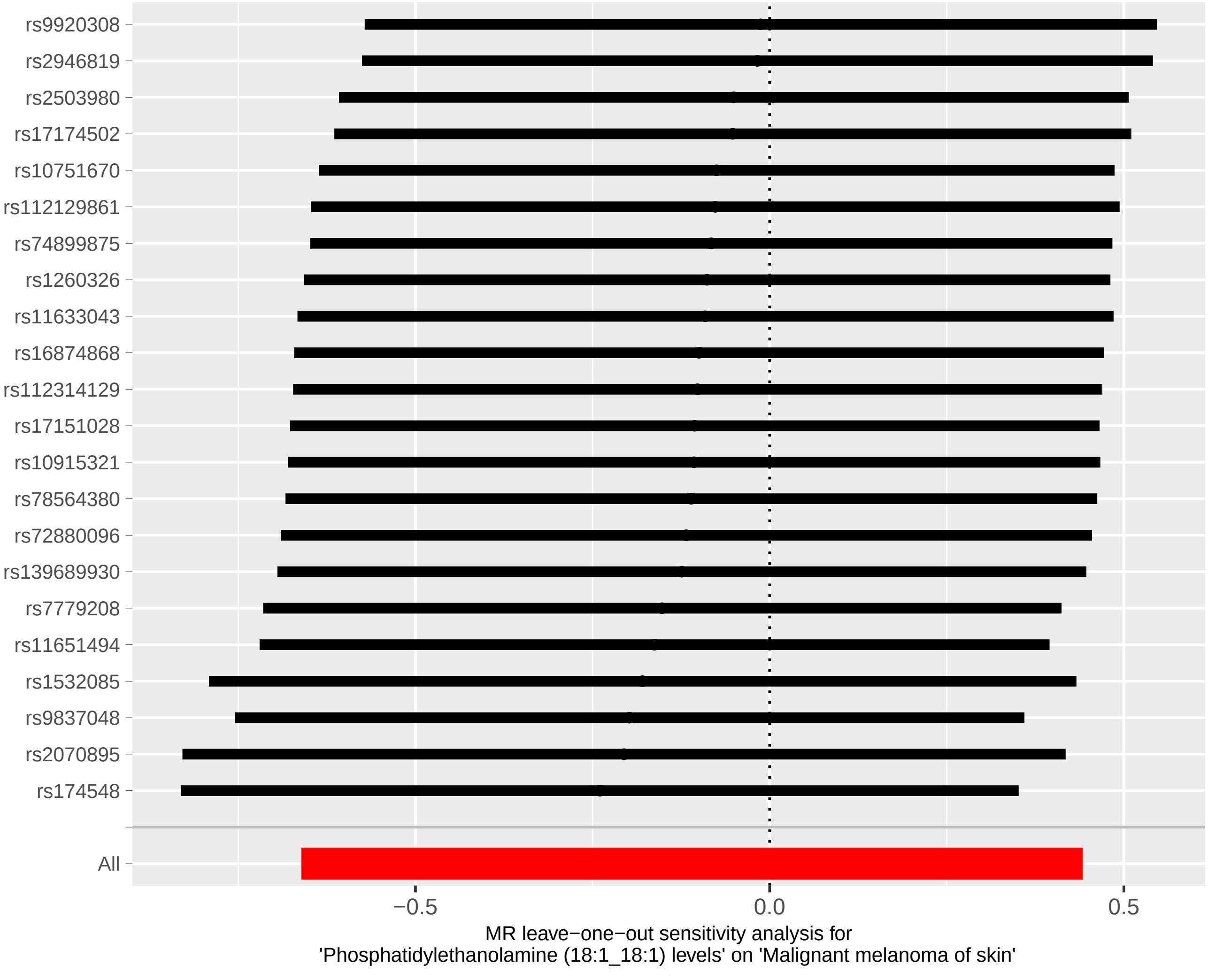

rs9920308
rs2946819
rs2503980
rs17174502
rs10751670
rs112129861
rs74899875
rs1260326
rs11633043
rs16874868
rs112314129
rs17151028
rs10915321
rs78564380
rs72880096
rs139689930
rs7779208
rs11651494
rs1532085
rs9837048
rs2070895
rs174548
All
−0.5
0.0
0.5
MR leave−one−out sensitivity analysis for
'Phosphatidylethanolamine (18:1_18:1) levels' on 'Malignant melanoma of skin'

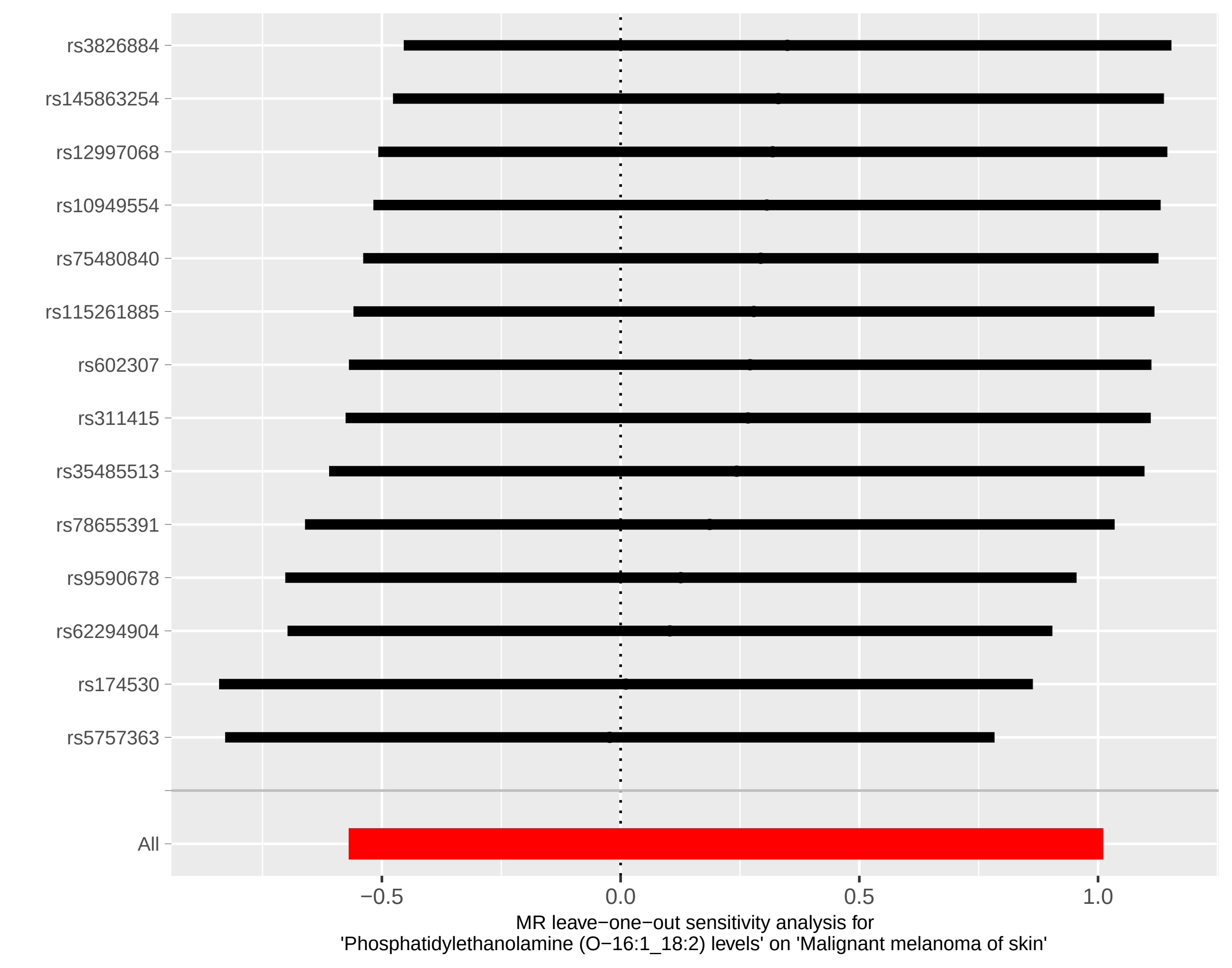

rs3826884
rs145863254
rs12997068
rs10949554
rs75480840
rs115261885
rs602307
rs311415
rs35485513
rs78655391
rs9590678
rs62294904
rs174530
rs5757363
All
−0.5
0.0
0.5
1.0
MR leave−one−out sensitivity analysis for
'Phosphatidylethanolamine (O−16:1_18:2) levels' on 'Malignant melanoma of skin'

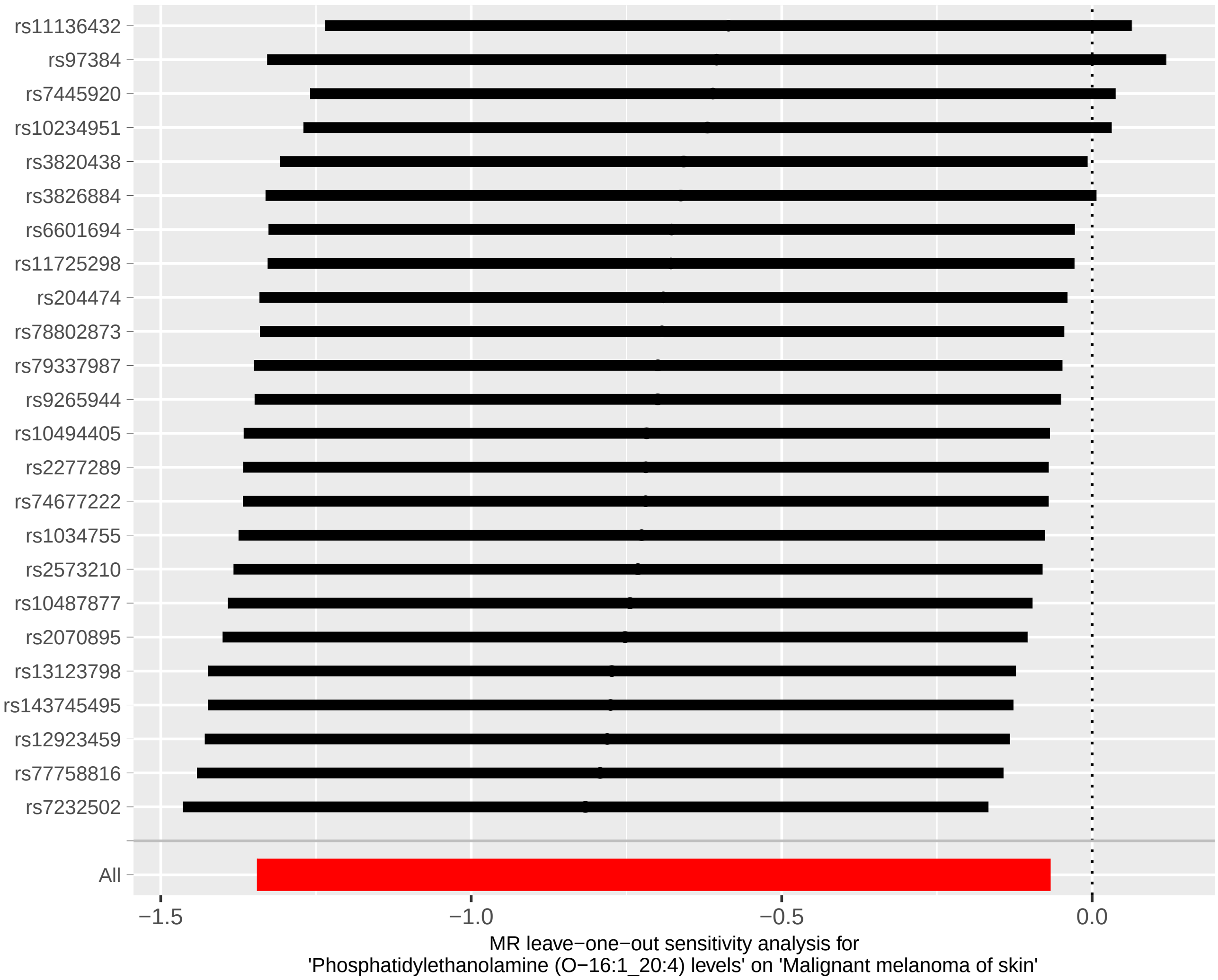
rs11136432
rs97384
rs7445920
rs10234951
rs3820438
rs3826884
rs6601694
rs11725298
rs204474
rs78802873
rs79337987
rs9265944
rs10494405
rs2277289
rs74677222
rs1034755
rs2573210
rs10487877
rs2070895
rs13123798
rs143745495
rs12923459
rs77758816
rs7232502
All
−1.5
−1.0
−0.5
0.0
MR leave−one−out sensitivity analysis for
'Phosphatidylethanolamine (O−16:1_20:4) levels' on 'Malignant melanoma of skin'

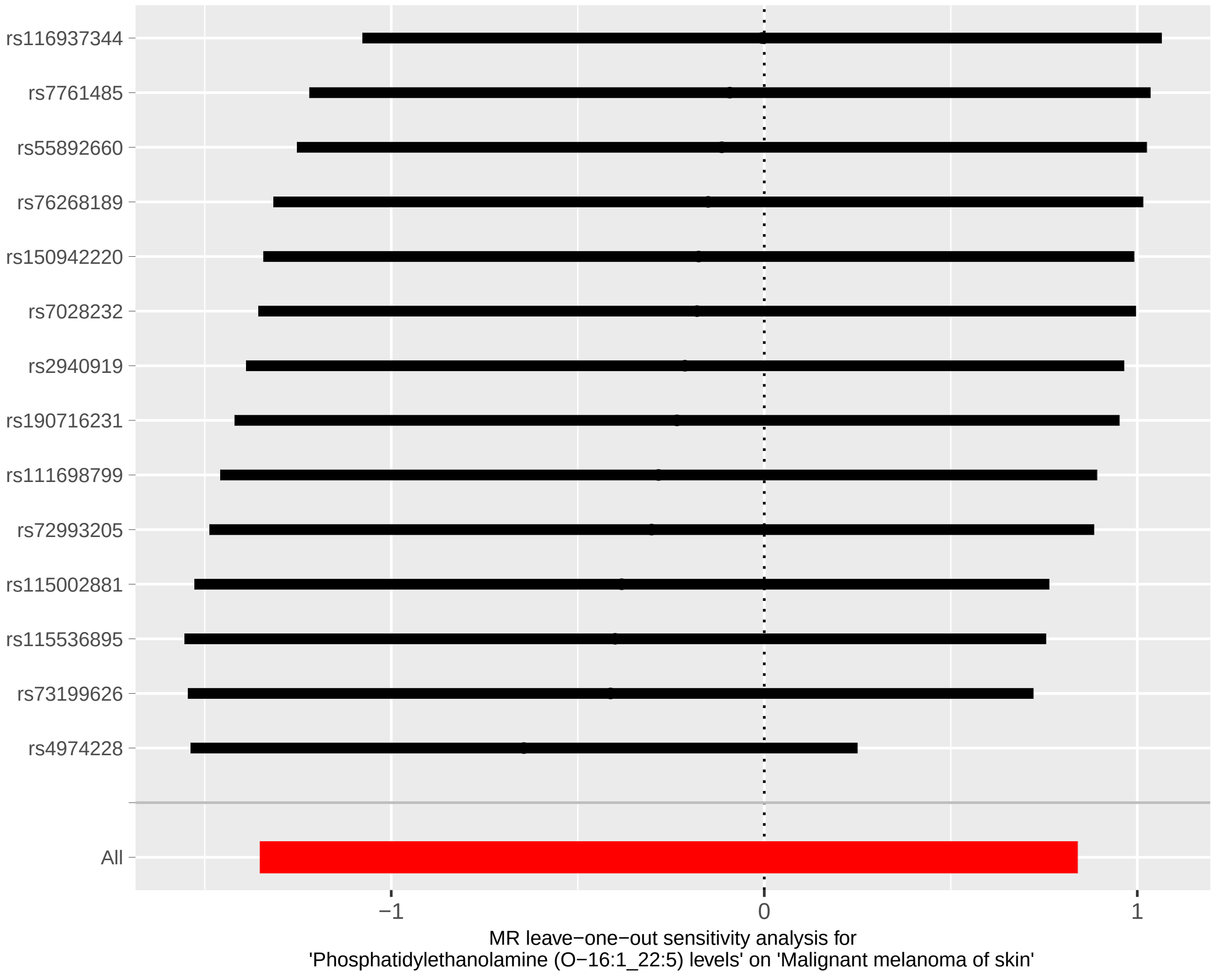
rs116937344
rs7761485
rs55892660
rs76268189
rs150942220
rs7028232
rs2940919
rs190716231
rs111698799
rs72993205
rs115002881
rs115536895
rs73199626
rs4974228
All
−1
0
1
MR leave−one−out sensitivity analysis for
'Phosphatidylethanolamine (O−16:1_22:5) levels' on 'Malignant melanoma of skin'

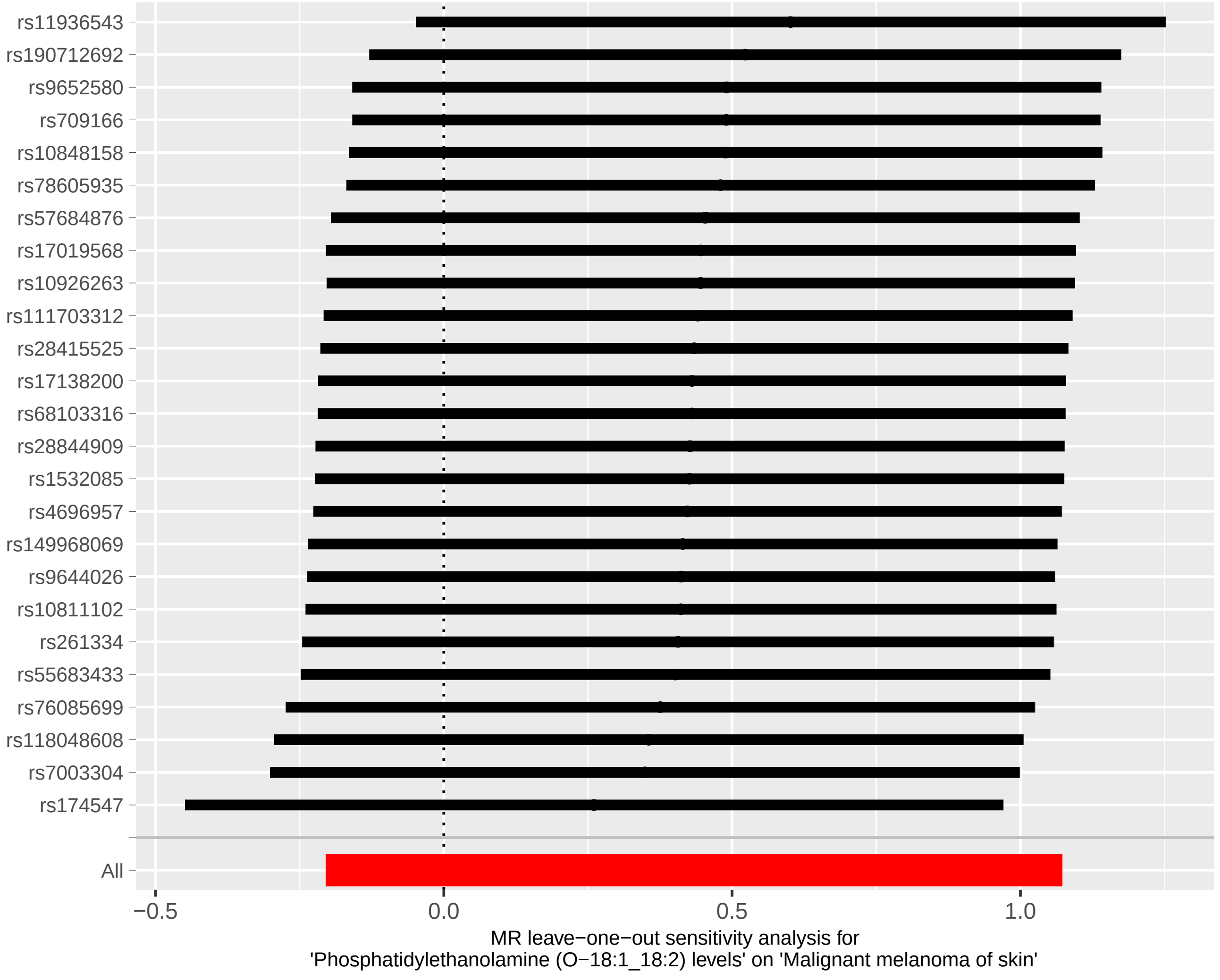
rs11936543
rs190712692
rs9652580
rs709166
rs10848158
rs78605935
rs57684876
rs17019568
rs10926263
rs111703312
rs28415525
rs17138200
rs68103316
rs28844909
rs1532085
rs4696957
rs149968069
rs9644026
rs10811102
rs261334
rs55683433
rs76085699
rs118048608
rs7003304
rs174547
All
−0.5
0.0
0.5
1.0
MR leave−one−out sensitivity analysis for
'Phosphatidylethanolamine (O−18:1_18:2) levels' on 'Malignant melanoma of skin'

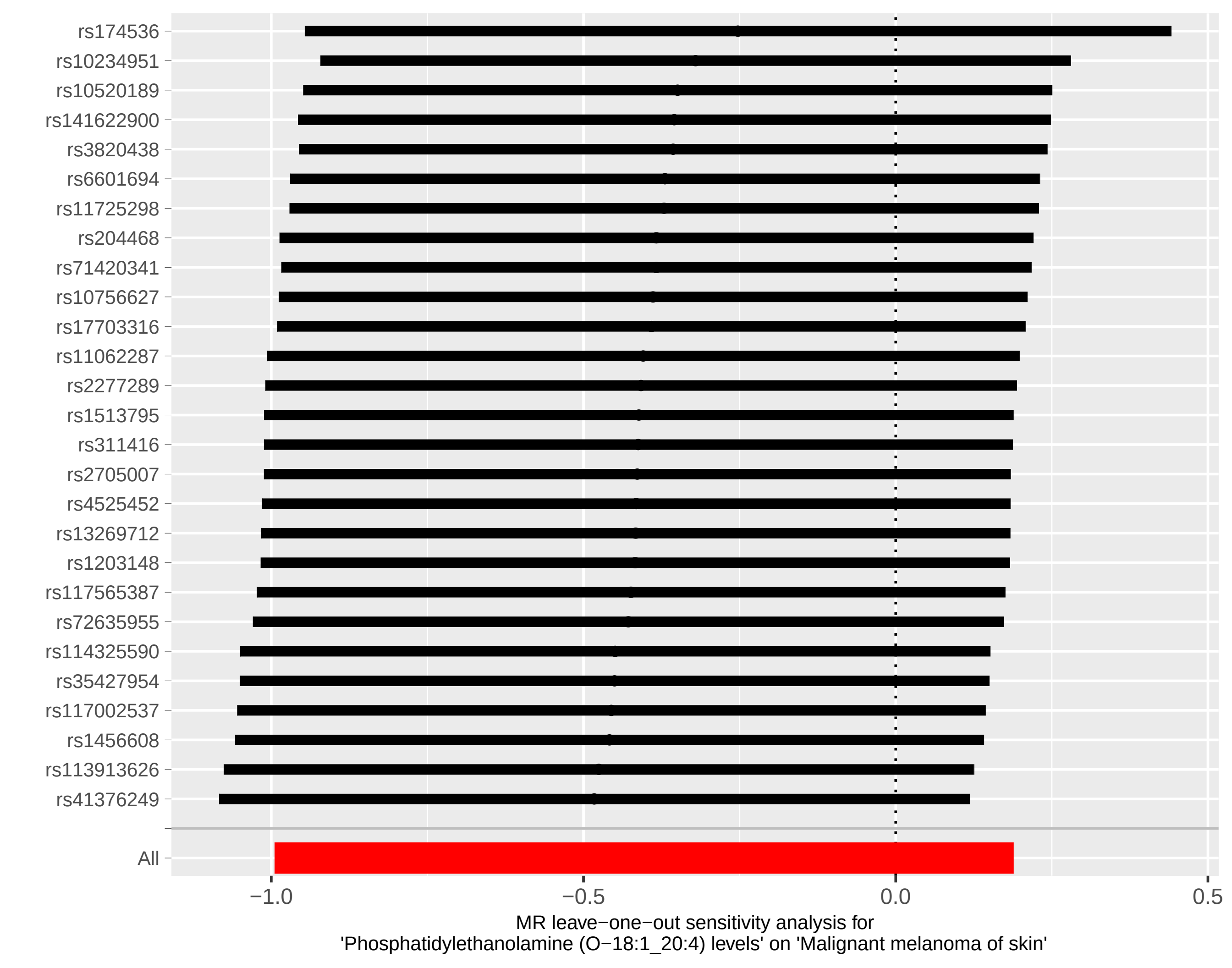
rs174536
rs10234951
rs10520189
rs141622900
rs3820438
rs6601694
rs11725298
rs204468
rs71420341
rs10756627
rs17703316
rs11062287
rs2277289
rs1513795
rs311416
rs2705007
rs4525452
rs13269712
rs1203148
rs117565387
rs72635955
rs114325590
rs35427954
rs117002537
rs1456608
rs113913626
rs41376249
All
−1.0
−0.5
0.0
0.5
MR leave−one−out sensitivity analysis for
'Phosphatidylethanolamine (O−18:1_20:4) levels' on 'Malignant melanoma of skin'

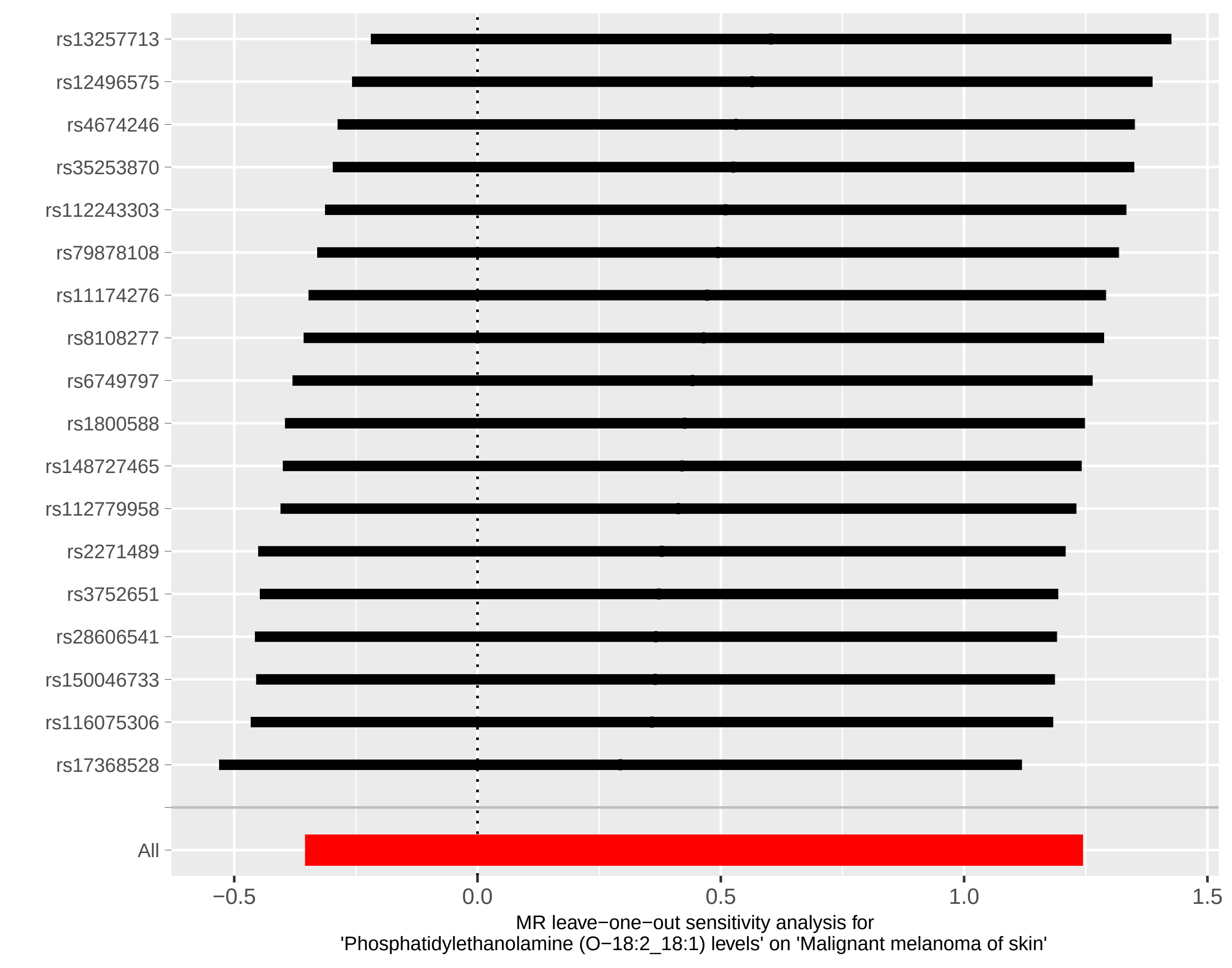

rs13257713
rs12496575
rs4674246
rs35253870
rs112243303
rs79878108
rs11174276
rs8108277
rs6749797
rs1800588
rs148727465
rs112779958
rs2271489
rs3752651
rs28606541
rs150046733
rs116075306
rs17368528
All
−0.5
0.0
0.5
1.0
1.5
MR leave−one−out sensitivity analysis for
'Phosphatidylethanolamine (O−18:2_18:1) levels' on 'Malignant melanoma of skin'

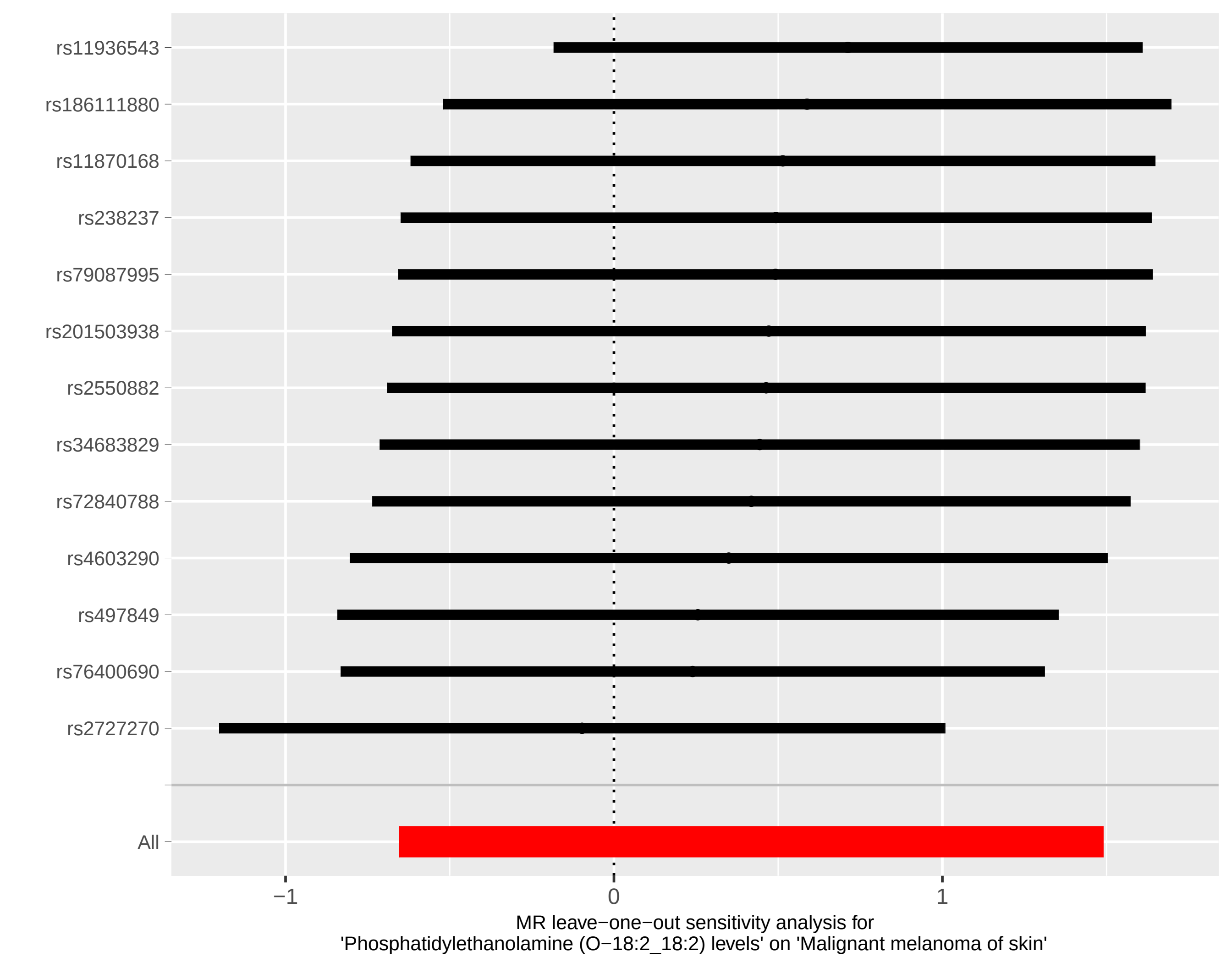
rs11936543
rs186111880
rs11870168
rs238237
rs79087995
rs201503938
rs2550882
rs34683829
rs72840788
rs4603290
rs497849
rs76400690
rs2727270
All
−1
0
1
MR leave−one−out sensitivity analysis for
'Phosphatidylethanolamine (O−18:2_18:2) levels' on 'Malignant melanoma of skin'

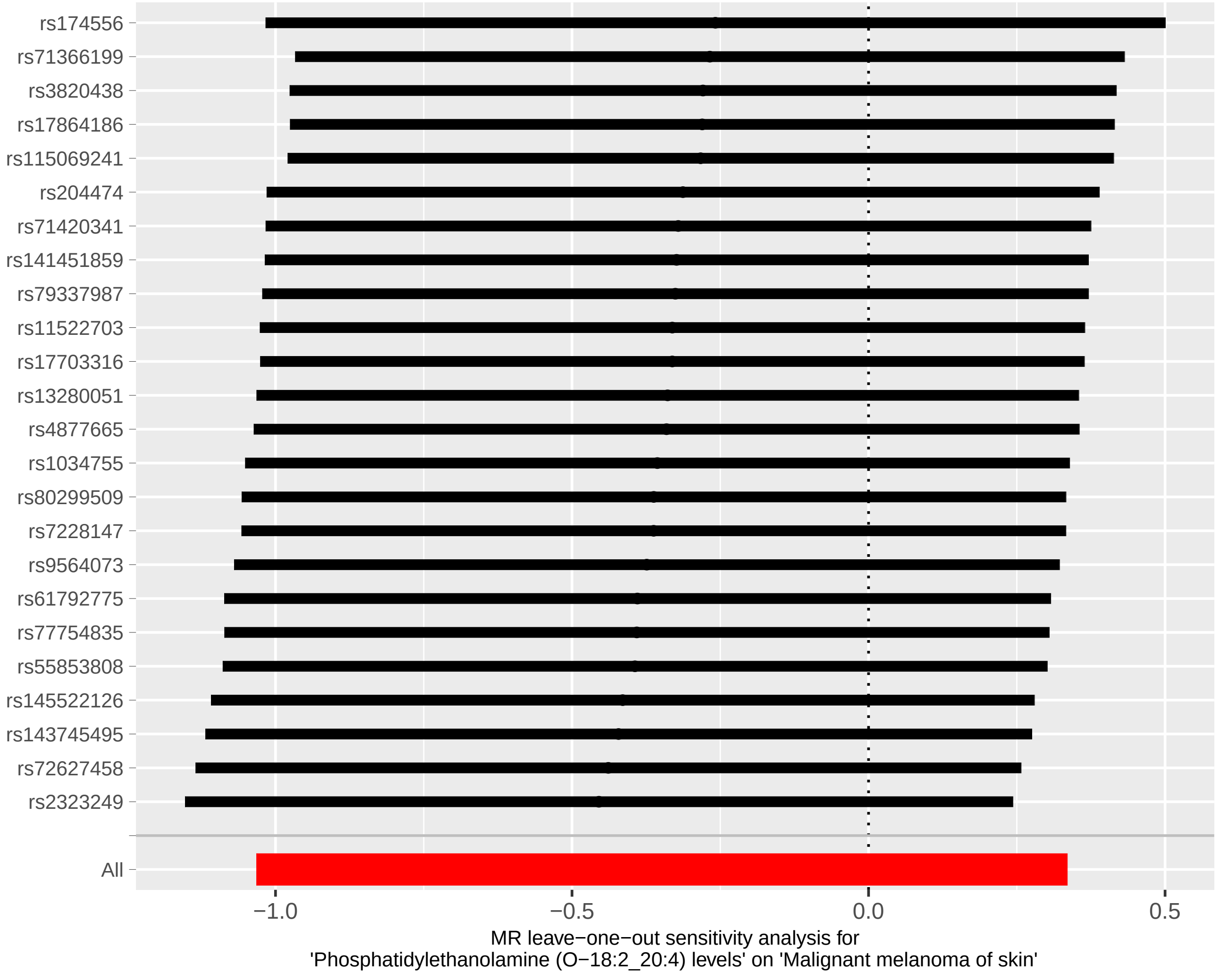
rs174556
rs71366199
rs3820438
rs17864186
rs115069241
rs204474
rs71420341
rs141451859
rs79337987
rs11522703
rs17703316
rs13280051
rs4877665
rs1034755
rs80299509
rs7228147
rs9564073
rs61792775
rs77754835
rs55853808
rs145522126
rs143745495
rs72627458
rs2323249
All
−1.0
−0.5
0.0
0.5
MR leave−one−out sensitivity analysis for
'Phosphatidylethanolamine (O−18:2_20:4) levels' on 'Malignant melanoma of skin'

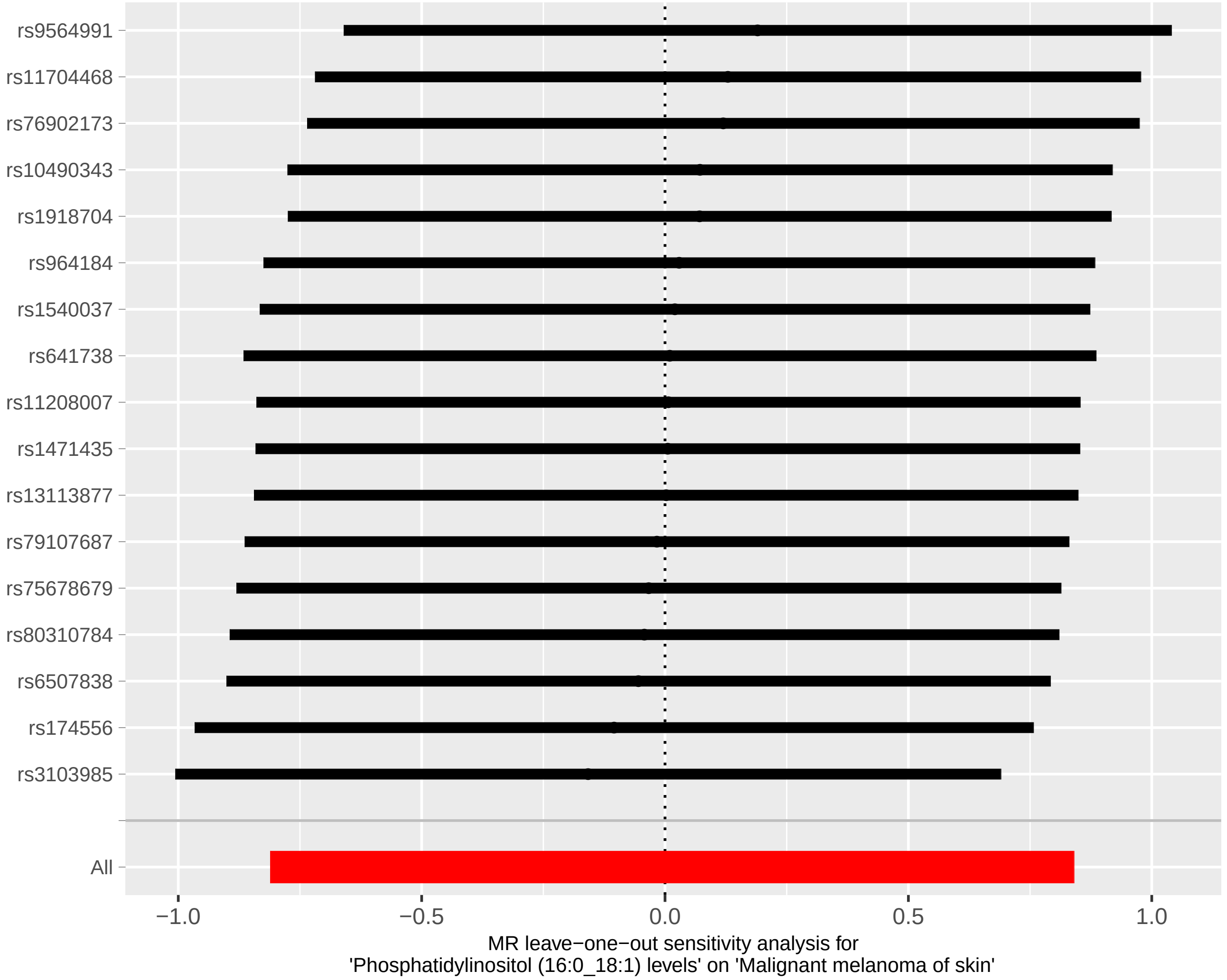
rs9564991
rs11704468
rs76902173
rs10490343
rs1918704
rs964184
rs1540037
rs641738
rs11208007
rs1471435
rs13113877
rs79107687
rs75678679
rs80310784
rs6507838
rs174556
rs3103985
All
−1.0
−0.5
0.0
0.5
1.0
MR leave−one−out sensitivity analysis for
'Phosphatidylinositol (16:0_18:1) levels' on 'Malignant melanoma of skin'

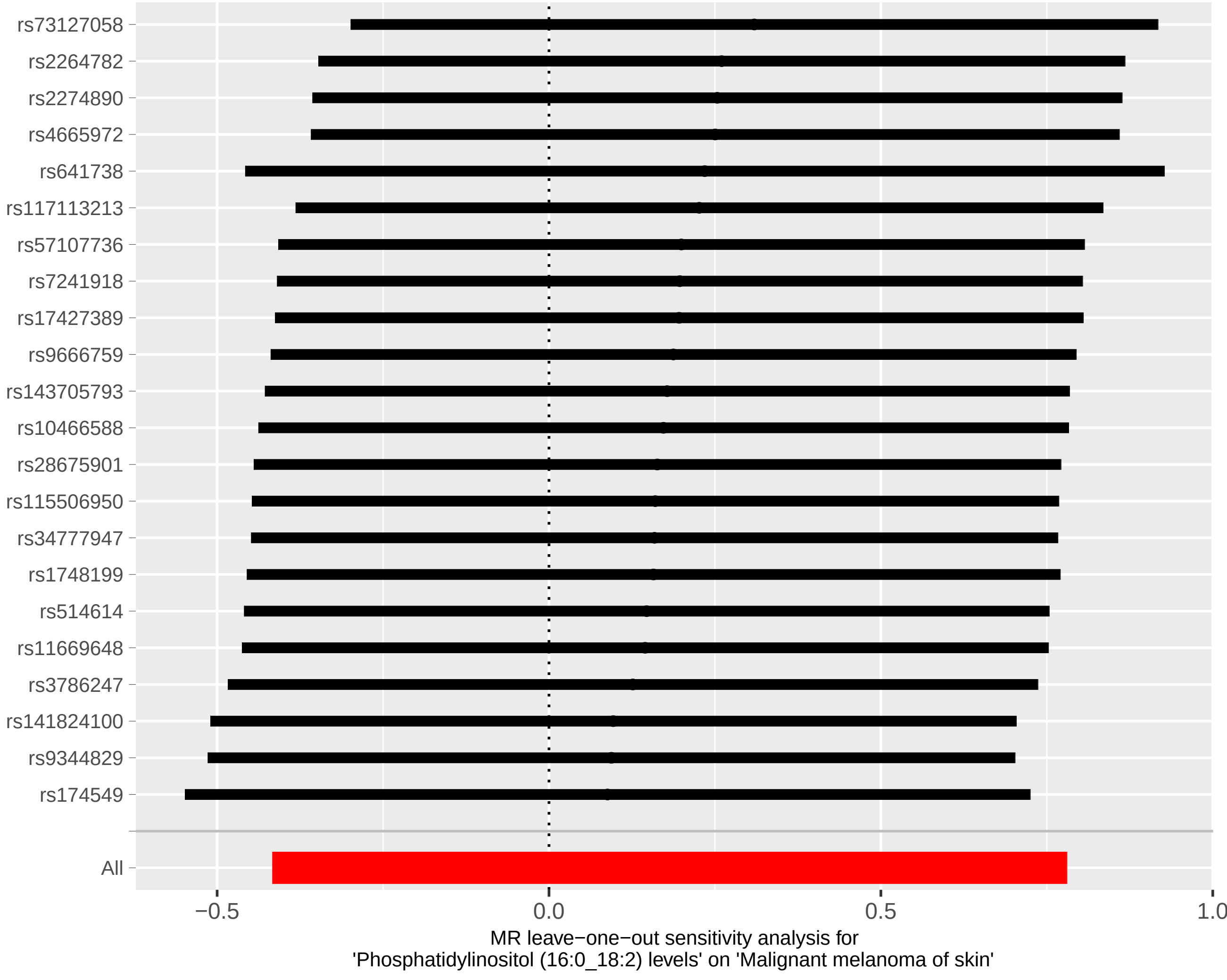
rs73127058
rs2264782
rs2274890
rs4665972
rs641738
rs117113213
rs57107736
rs7241918
rs17427389
rs9666759
rs143705793
rs10466588
rs28675901
rs115506950
rs34777947
rs1748199
rs514614
rs11669648
rs3786247
rs141824100
rs9344829
rs174549
All
−0.5
0.0
0.5
1.0
MR leave−one−out sensitivity analysis for
'Phosphatidylinositol (16:0_18:2) levels' on 'Malignant melanoma of skin'

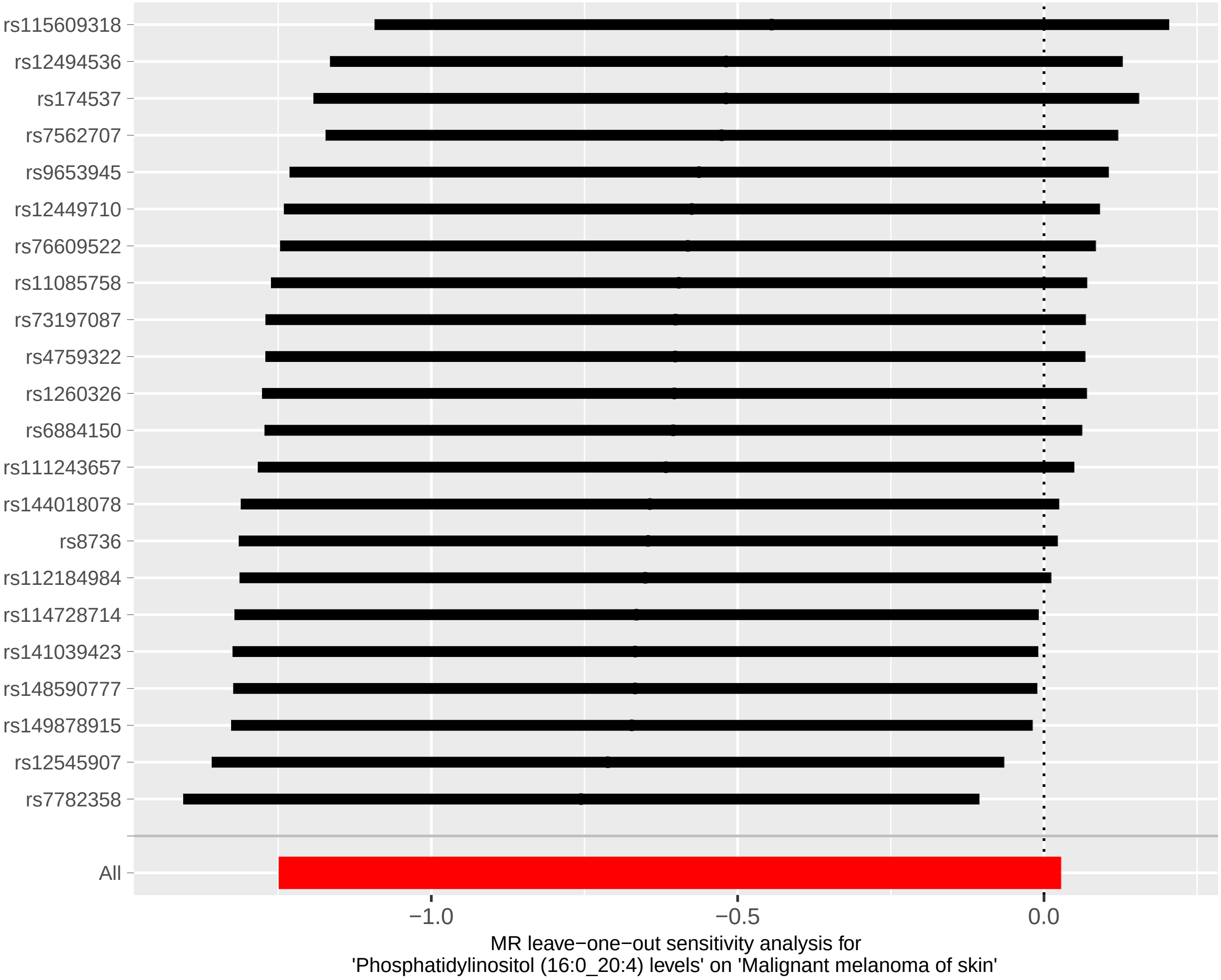

rs115609318
rs12494536
rs174537
rs7562707
rs9653945
rs12449710
rs76609522
rs11085758
rs73197087
rs4759322
rs1260326
rs6884150
rs111243657
rs144018078
rs8736
rs112184984
rs114728714
rs141039423
rs148590777
rs149878915
rs12545907
rs7782358
All
−1.0
−0.5
0.0
MR leave−one−out sensitivity analysis for
'Phosphatidylinositol (16:0_20:4) levels' on 'Malignant melanoma of skin'

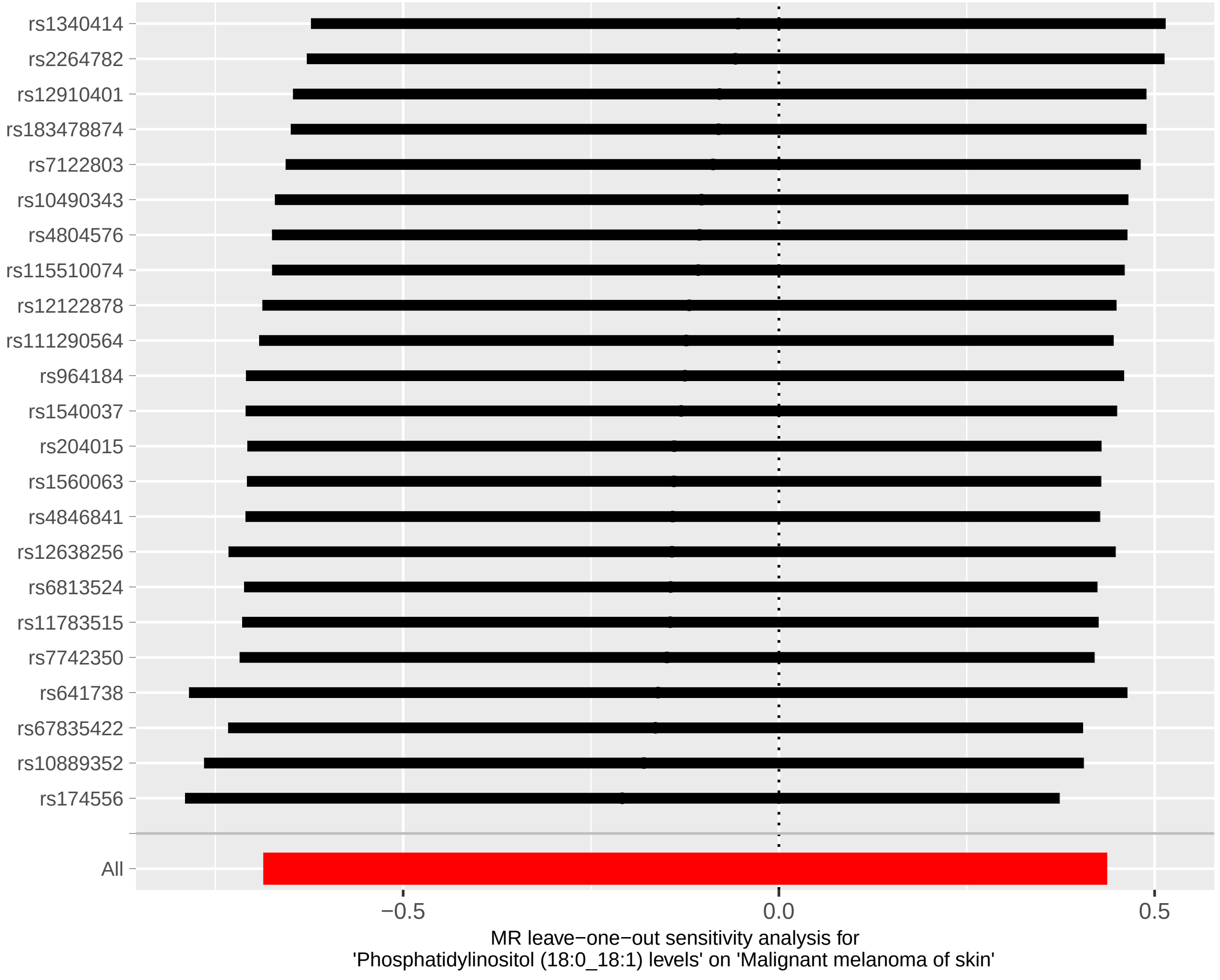
rs1340414
rs2264782
rs12910401
rs183478874
rs7122803
rs10490343
rs4804576
rs115510074
rs12122878
rs111290564
rs964184
rs1540037
rs204015
rs1560063
rs4846841
rs12638256
rs6813524
rs11783515
rs7742350
rs641738
rs67835422
rs10889352
rs174556
All
−0.5
0.0
0.5
MR leave−one−out sensitivity analysis for
'Phosphatidylinositol (18:0_18:1) levels' on 'Malignant melanoma of skin'

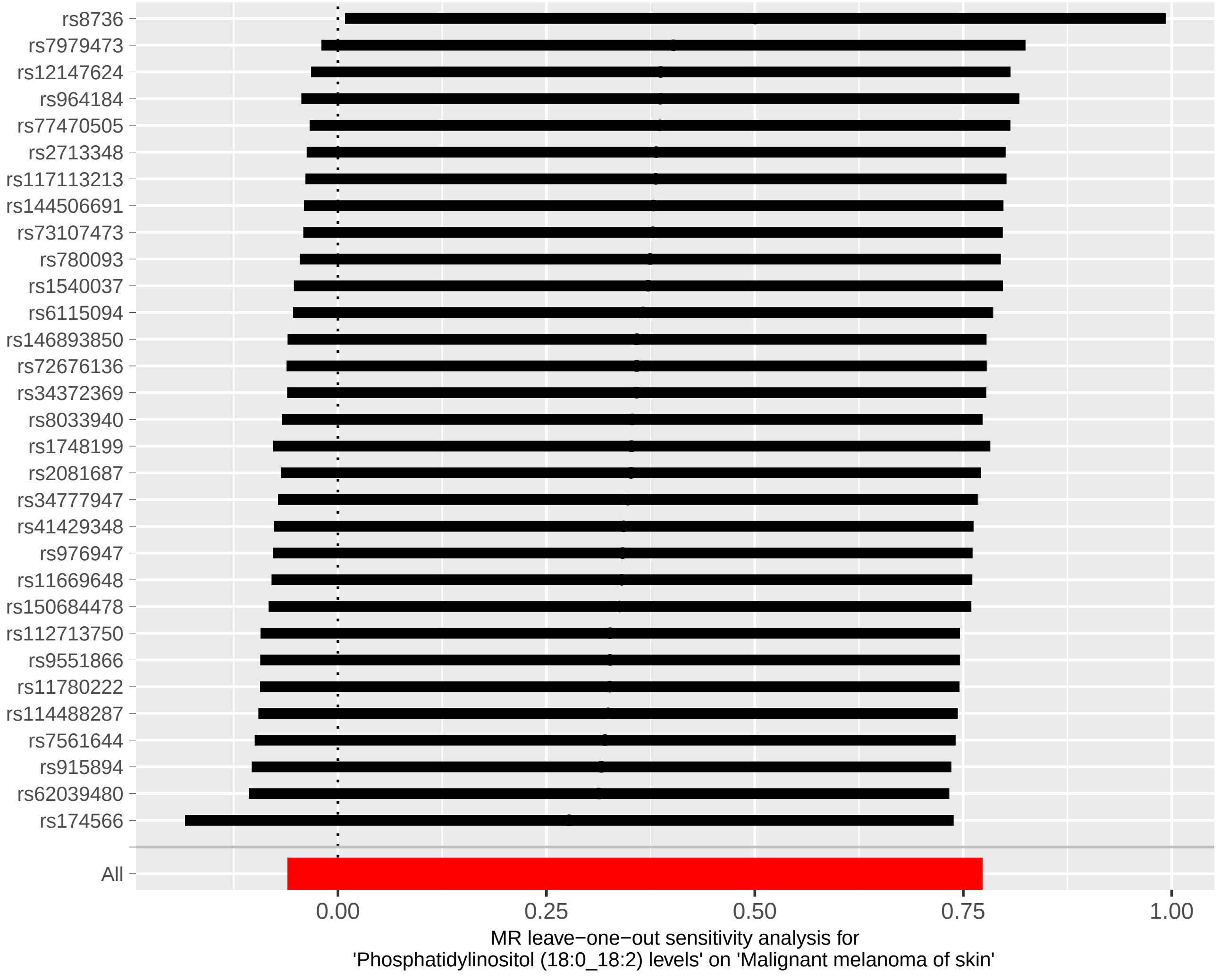
rs8736
rs7979473
rs12147624
rs964184
rs77470505
rs2713348
rs117113213
rs144506691
rs73107473
rs780093
rs1540037
rs6115094
rs146893850
rs72676136
rs34372369
rs8033940
rs1748199
rs2081687
rs34777947
rs41429348
rs976947
rs11669648
rs150684478
rs112713750
rs9551866
rs11780222
rs114488287
rs7561644
rs915894
rs62039480
rs174566
All
0.00
0.25
0.50
0.75
1.00
MR leave−one−out sensitivity analysis for
'Phosphatidylinositol (18:0_18:2) levels' on 'Malignant melanoma of skin'

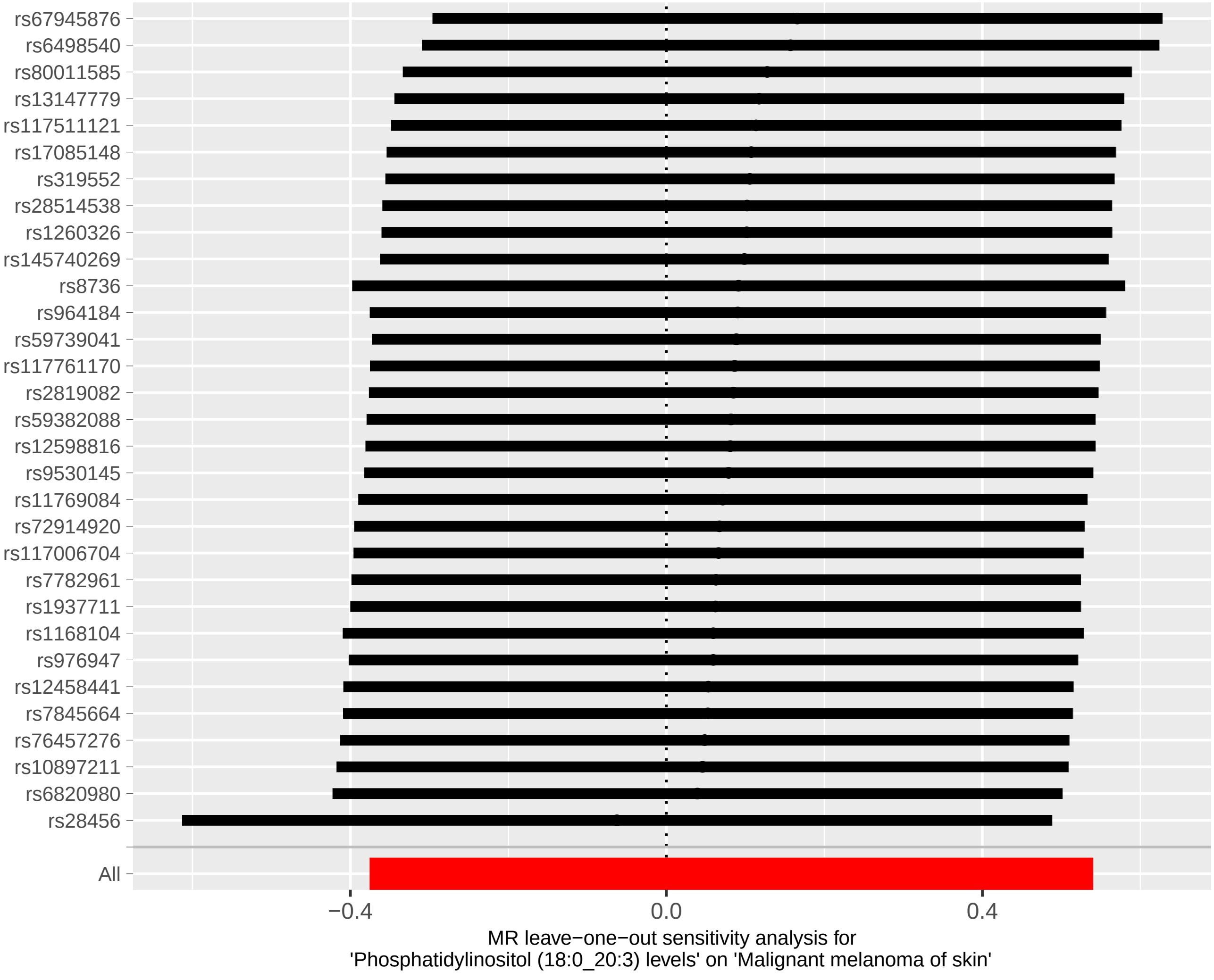
rs67945876
rs6498540
rs80011585
rs13147779
rs117511121
rs17085148
rs319552
rs28514538
rs1260326
rs145740269
rs8736
rs964184
rs59739041
rs117761170
rs2819082
rs59382088
rs12598816
rs9530145
rs11769084
rs72914920
rs117006704
rs7782961
rs1937711
rs1168104
rs976947
rs12458441
rs7845664
rs76457276
rs10897211
rs6820980
rs28456
All
−0.4
0.0
0.4
MR leave−one−out sensitivity analysis for
'Phosphatidylinositol (18:0_20:3) levels' on 'Malignant melanoma of skin'

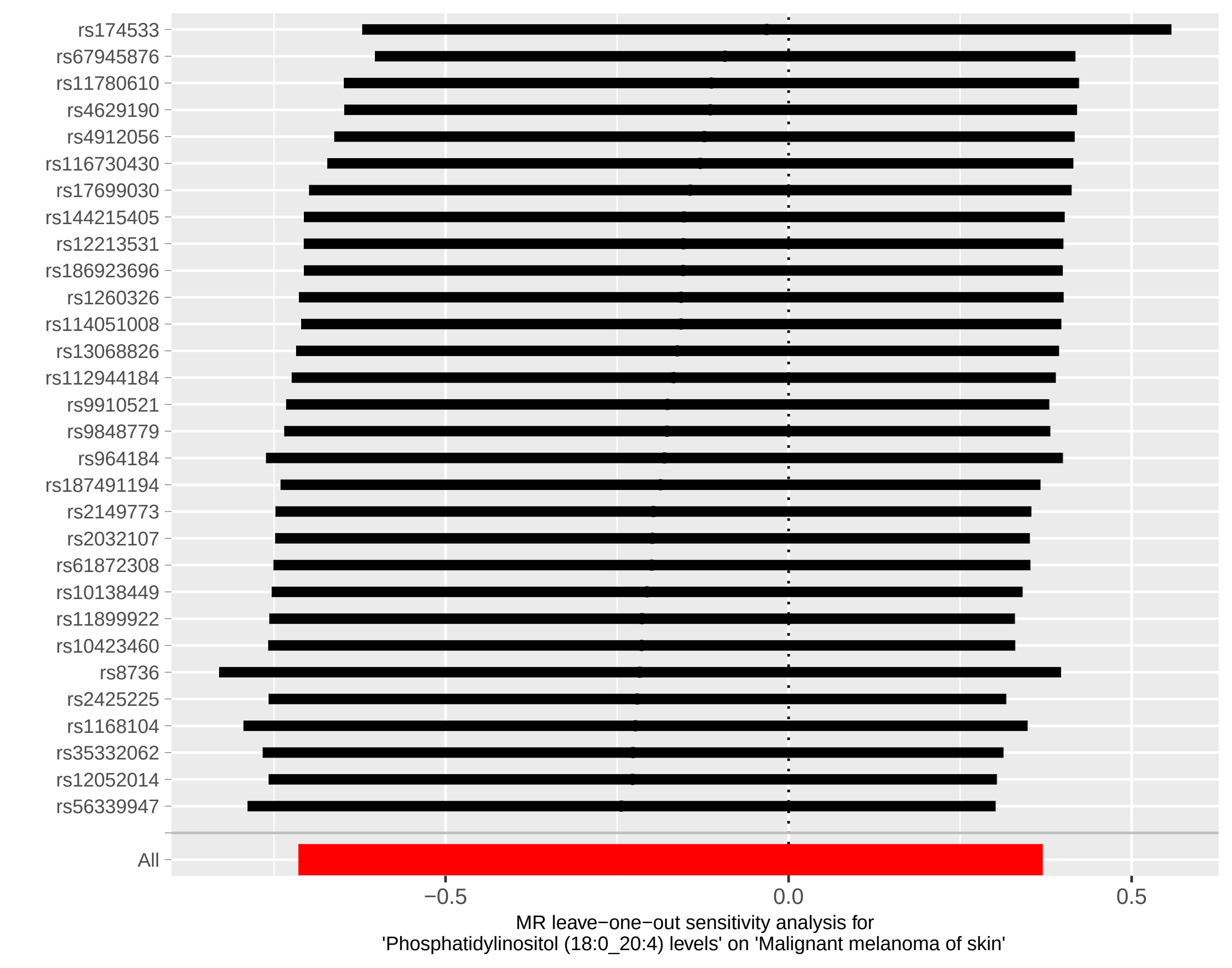
rs174533
rs67945876
rs11780610
rs4629190
rs4912056
rs116730430
rs17699030
rs144215405
rs12213531
rs186923696
rs1260326
rs114051008
rs13068826
rs112944184
rs9910521
rs9848779
rs964184
rs187491194
rs2149773
rs2032107
rs61872308
rs10138449
rs11899922
rs10423460
rs8736
rs2425225
rs1168104
rs35332062
rs12052014
rs56339947
All
−0.5
0.0
0.5
MR leave−one−out sensitivity analysis for
'Phosphatidylinositol (18:0_20:4) levels' on 'Malignant melanoma of skin'

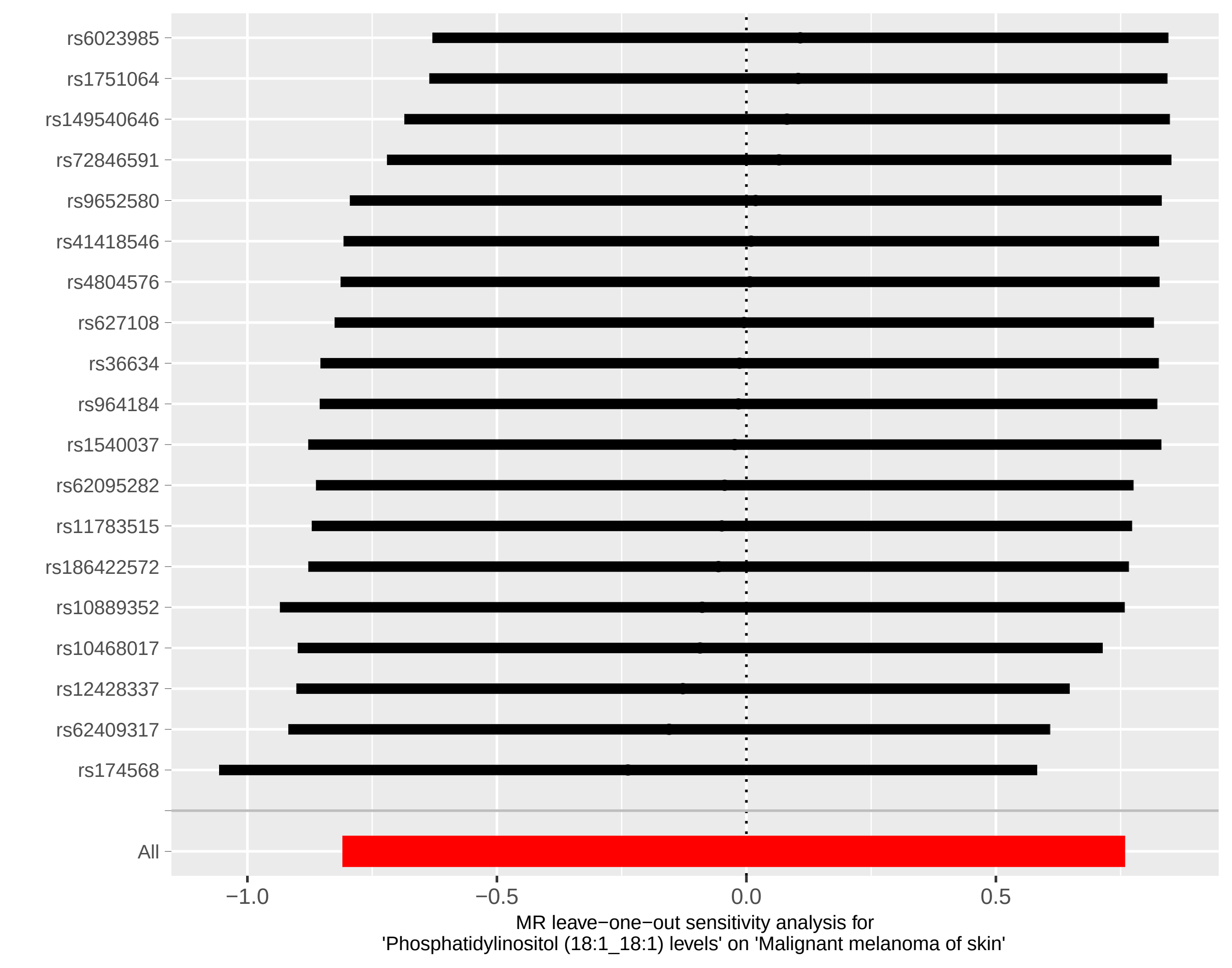
rs6023985
rs1751064
rs149540646
rs72846591
rs9652580
rs41418546
rs4804576
rs627108
rs36634
rs964184
rs1540037
rs62095282
rs11783515
rs186422572
rs10889352
rs10468017
rs12428337
rs62409317
rs174568
All
−1.0
−0.5
0.0
0.5
MR leave−one−out sensitivity analysis for
'Phosphatidylinositol (18:1_18:1) levels' on 'Malignant melanoma of skin'

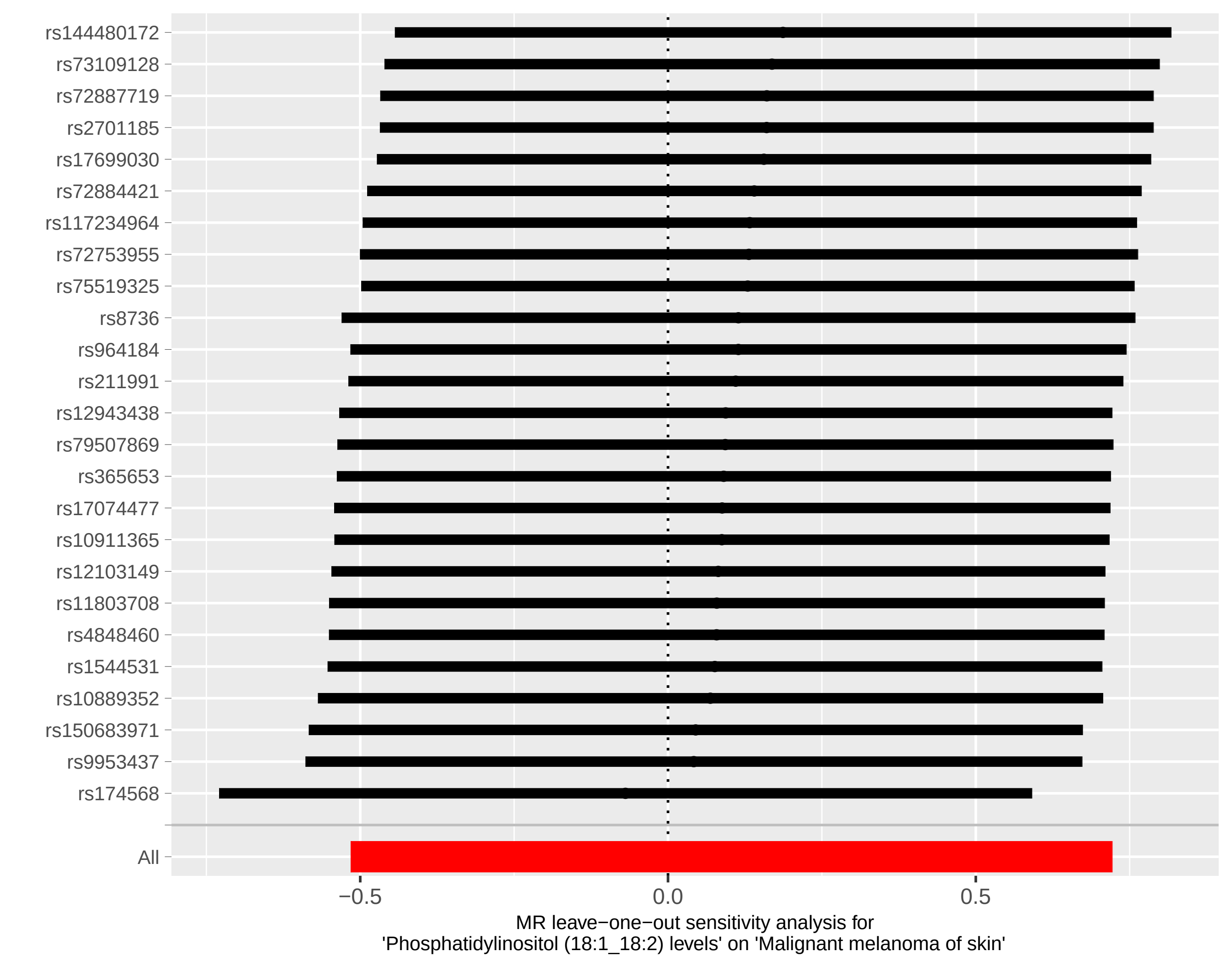

rs144480172
rs73109128
rs72887719
rs2701185
rs17699030
rs72884421
rs117234964
rs72753955
rs75519325
rs8736
rs964184
rs211991
rs12943438
rs79507869
rs365653
rs17074477
rs10911365
rs12103149
rs11803708
rs4848460
rs1544531
rs10889352
rs150683971
rs9953437
rs174568
All
−0.5
0.0
0.5
MR leave−one−out sensitivity analysis for
'Phosphatidylinositol (18:1_18:2) levels' on 'Malignant melanoma of skin'

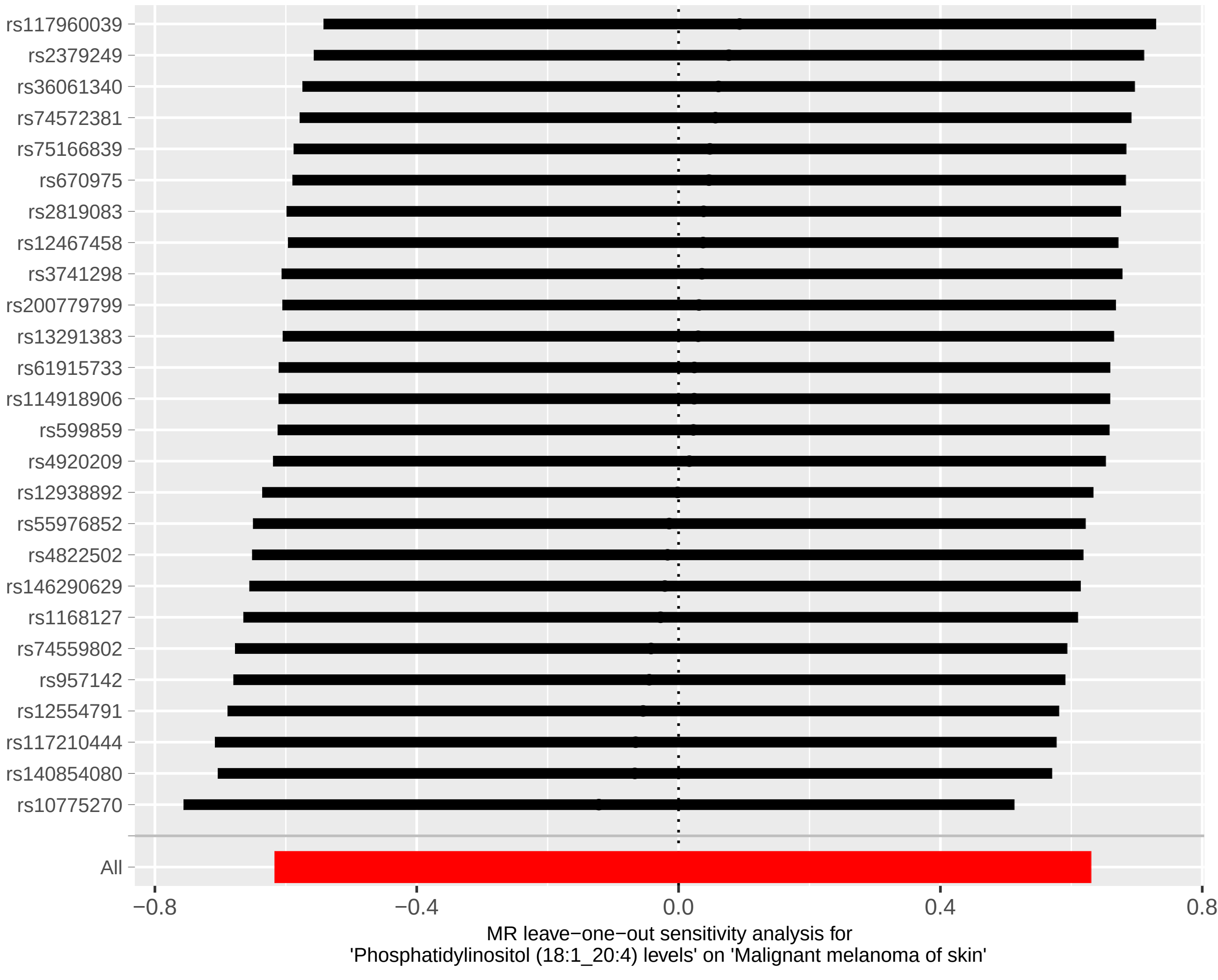

rs117960039
rs2379249
rs36061340
rs74572381
rs75166839
rs670975
rs2819083
rs12467458
rs3741298
rs200779799
rs13291383
rs61915733
rs114918906
rs599859
rs4920209
rs12938892
rs55976852
rs4822502
rs146290629
rs1168127
rs74559802
rs957142
rs12554791
rs117210444
rs140854080
rs10775270
All
−0.8
−0.4
0.0
0.4
0.8
MR leave−one−out sensitivity analysis for
'Phosphatidylinositol (18:1_20:4) levels' on 'Malignant melanoma of skin'

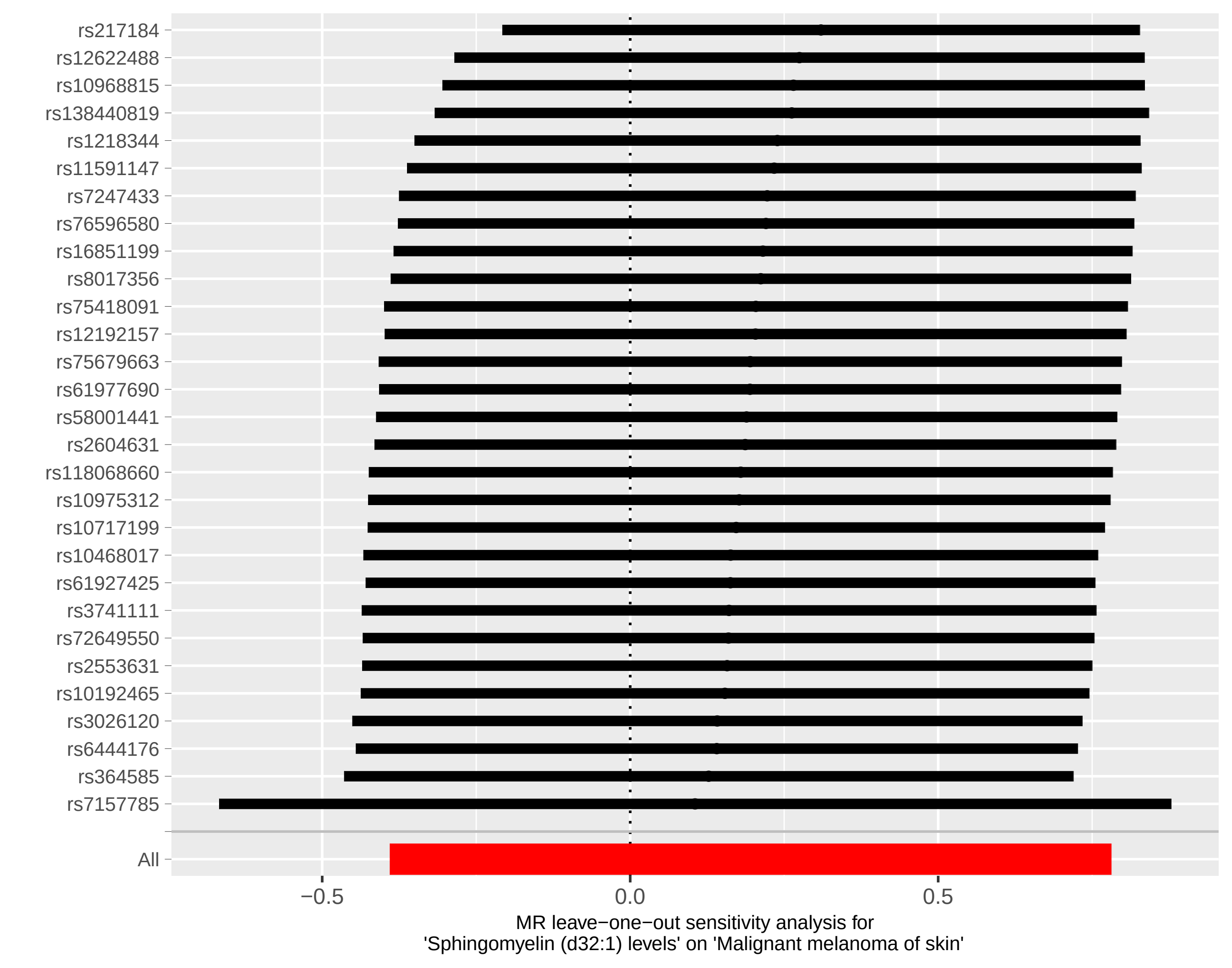
rs217184
rs12622488
rs10968815
rs138440819
rs1218344
rs11591147
rs7247433
rs76596580
rs16851199
rs8017356
rs75418091
rs12192157
rs75679663
rs61977690
rs58001441
rs2604631
rs118068660
rs10975312
rs10717199
rs10468017
rs61927425
rs3741111
rs72649550
rs2553631
rs10192465
rs3026120
rs6444176
rs364585
rs7157785
All
−0.5
0.0
0.5
MR leave−one−out sensitivity analysis for
'Sphingomyelin (d32:1) levels' on 'Malignant melanoma of skin'

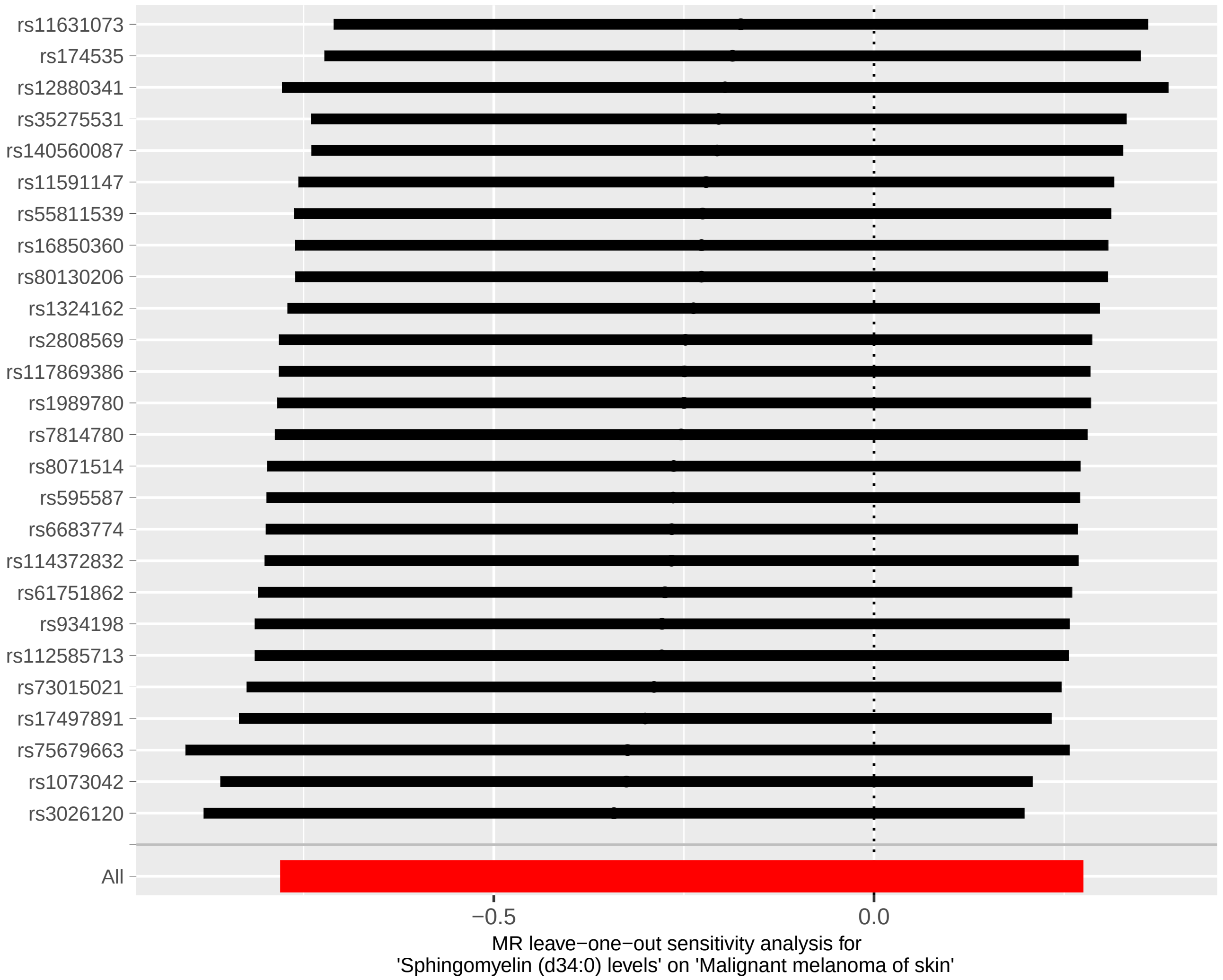
rs11631073
rs174535
rs12880341
rs35275531
rs140560087
rs11591147
rs55811539
rs16850360
rs80130206
rs1324162
rs2808569
rs117869386
rs1989780
rs7814780
rs8071514
rs595587
rs6683774
rs114372832
rs61751862
rs934198
rs112585713
rs73015021
rs17497891
rs75679663
rs1073042
rs3026120
All
−0.5
0.0
MR leave−one−out sensitivity analysis for
'Sphingomyelin (d34:0) levels' on 'Malignant melanoma of skin'

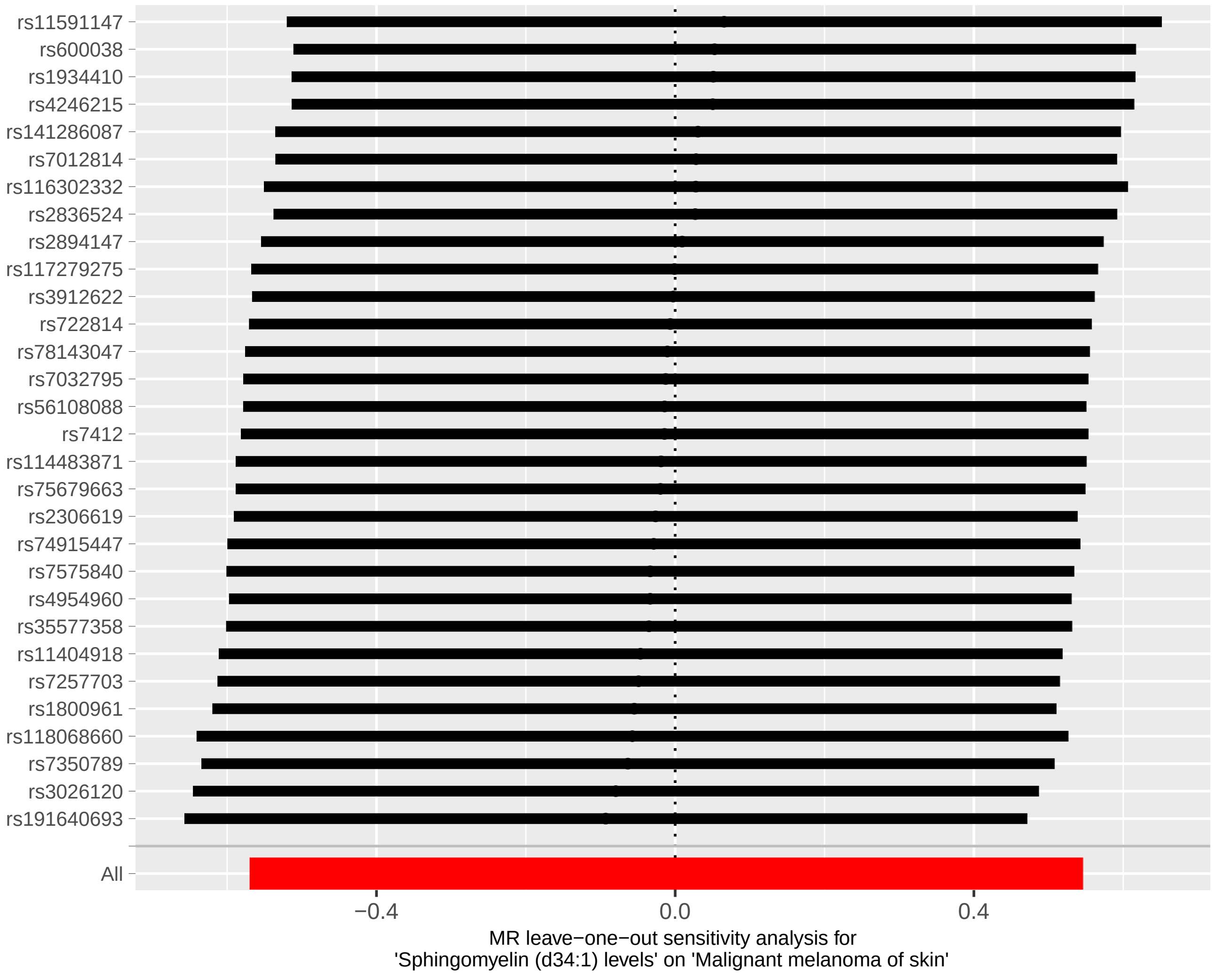
rs11591147
rs600038
rs1934410
rs4246215
rs141286087
rs7012814
rs116302332
rs2836524
rs2894147
rs117279275
rs3912622
rs722814
rs78143047
rs7032795
rs56108088
rs7412
rs114483871
rs75679663
rs2306619
rs74915447
rs7575840
rs4954960
rs35577358
rs11404918
rs7257703
rs1800961
rs118068660
rs7350789
rs3026120
rs191640693
All
−0.4
0.0
0.4
MR leave−one−out sensitivity analysis for
'Sphingomyelin (d34:1) levels' on 'Malignant melanoma of skin'

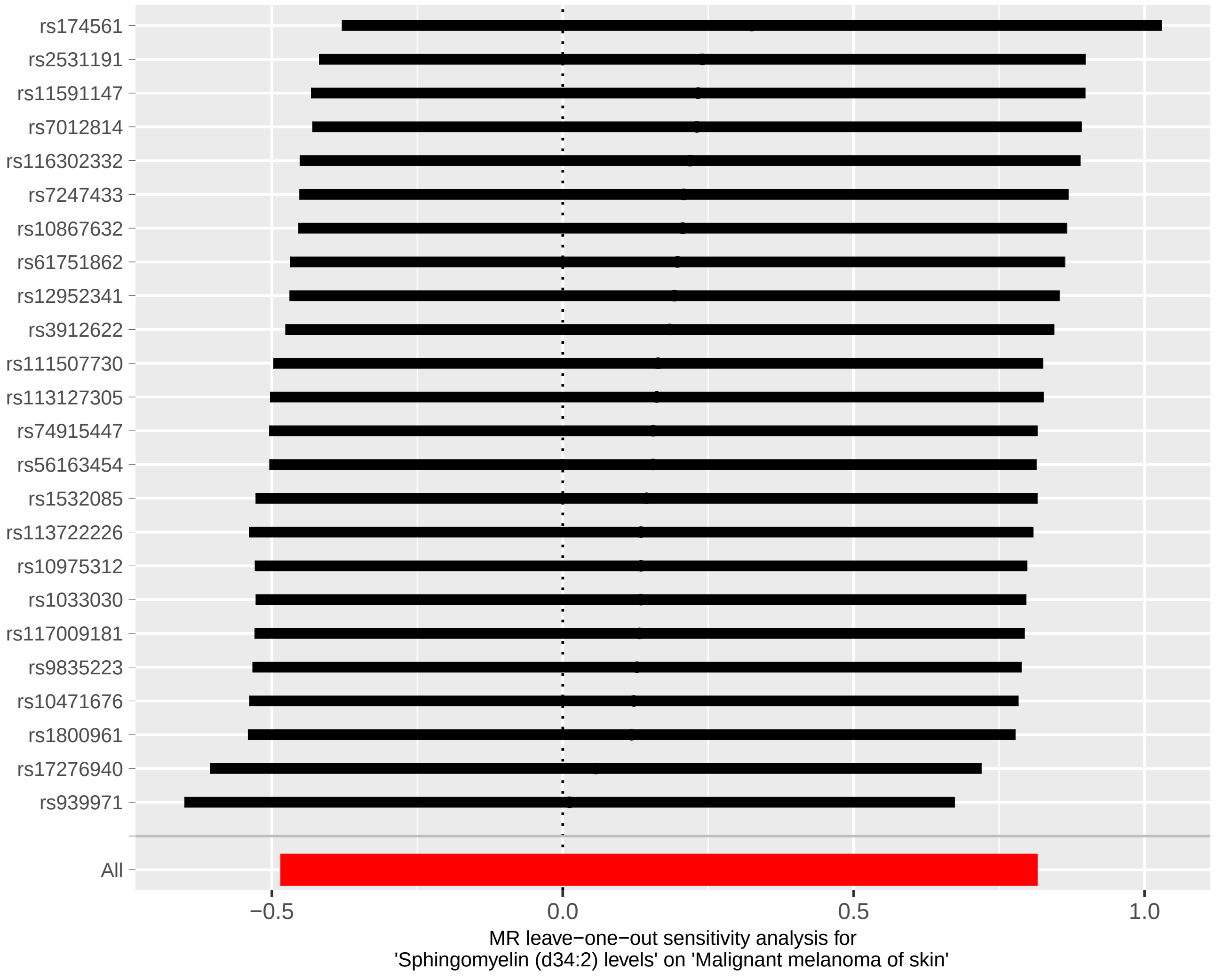

rs174561
rs2531191
rs11591147
rs7012814
rs116302332
rs7247433
rs10867632
rs61751862
rs12952341
rs3912622
rs111507730
rs113127305
rs74915447
rs56163454
rs1532085
rs113722226
rs10975312
rs1033030
rs117009181
rs9835223
rs10471676
rs1800961
rs17276940
rs939971
All
−0.5
0.0
0.5
1.0
MR leave−one−out sensitivity analysis for
'Sphingomyelin (d34:2) levels' on 'Malignant melanoma of skin'

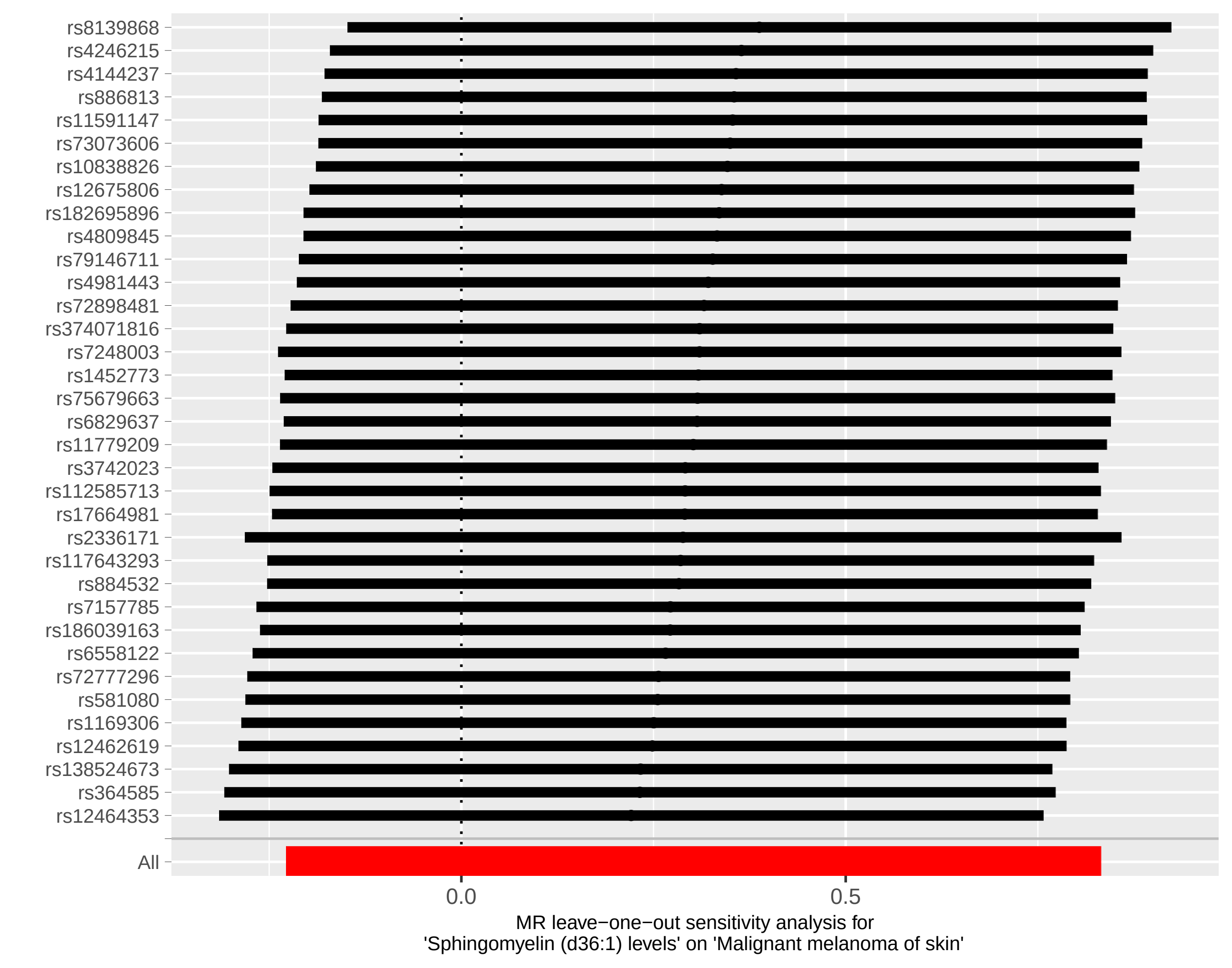
rs8139868
rs4246215
rs4144237
rs886813
rs11591147
rs73073606
rs10838826
rs12675806
rs182695896
rs4809845
rs79146711
rs4981443
rs72898481
rs374071816
rs7248003
rs1452773
rs75679663
rs6829637
rs11779209
rs3742023
rs112585713
rs17664981
rs2336171
rs117643293
rs884532
rs7157785
rs186039163
rs6558122
rs72777296
rs581080
rs1169306
rs12462619
rs138524673
rs364585
rs12464353
All
0.0
0.5
MR leave−one−out sensitivity analysis for
'Sphingomyelin (d36:1) levels' on 'Malignant melanoma of skin'

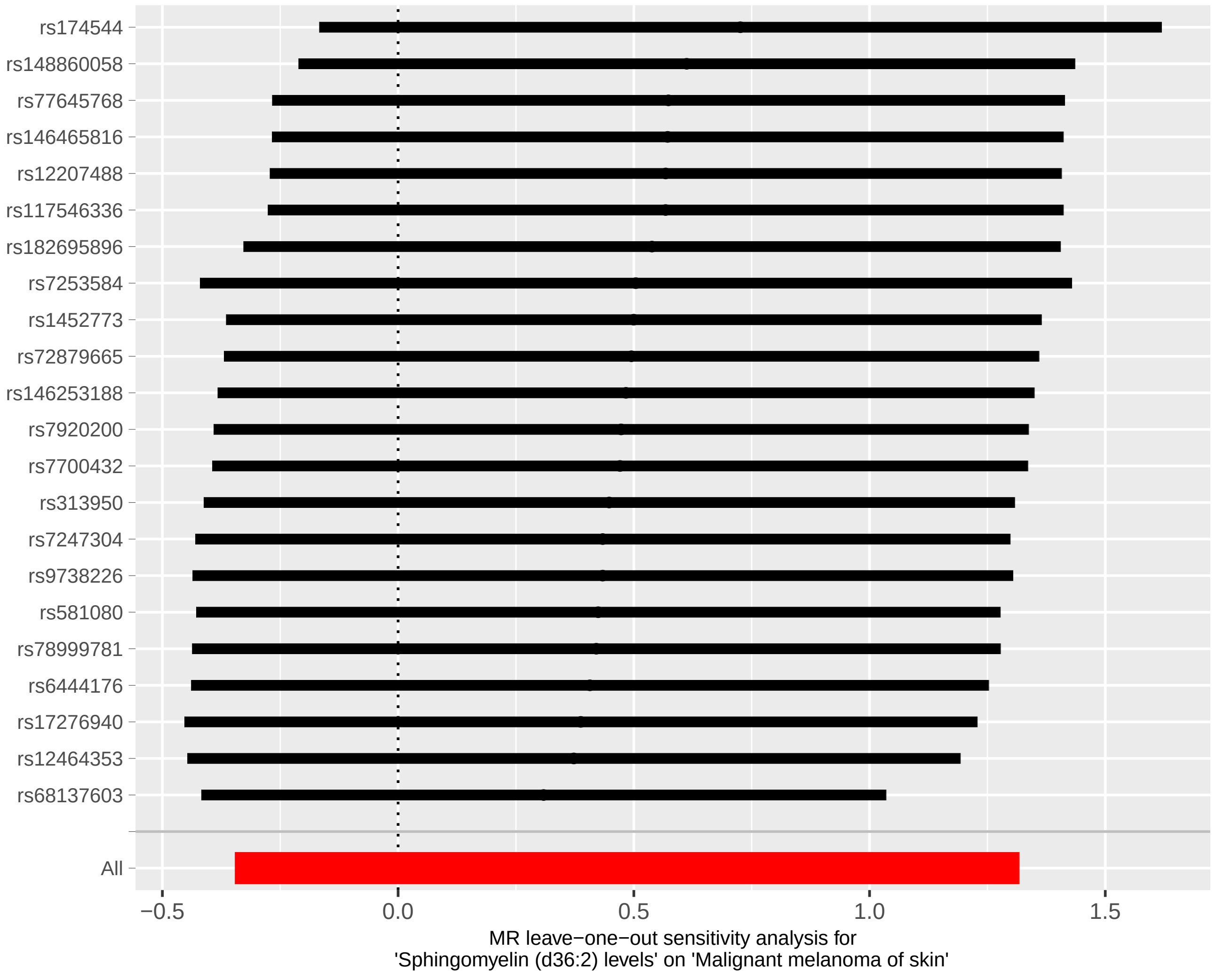
rs174544
rs148860058
rs77645768
rs146465816
rs12207488
rs117546336
rs182695896
rs7253584
rs1452773
rs72879665
rs146253188
rs7920200
rs7700432
rs313950
rs7247304
rs9738226
rs581080
rs78999781
rs6444176
rs17276940
rs12464353
rs68137603
All
−0.5
0.0
0.5
1.0
1.5
MR leave−one−out sensitivity analysis for
'Sphingomyelin (d36:2) levels' on 'Malignant melanoma of skin'

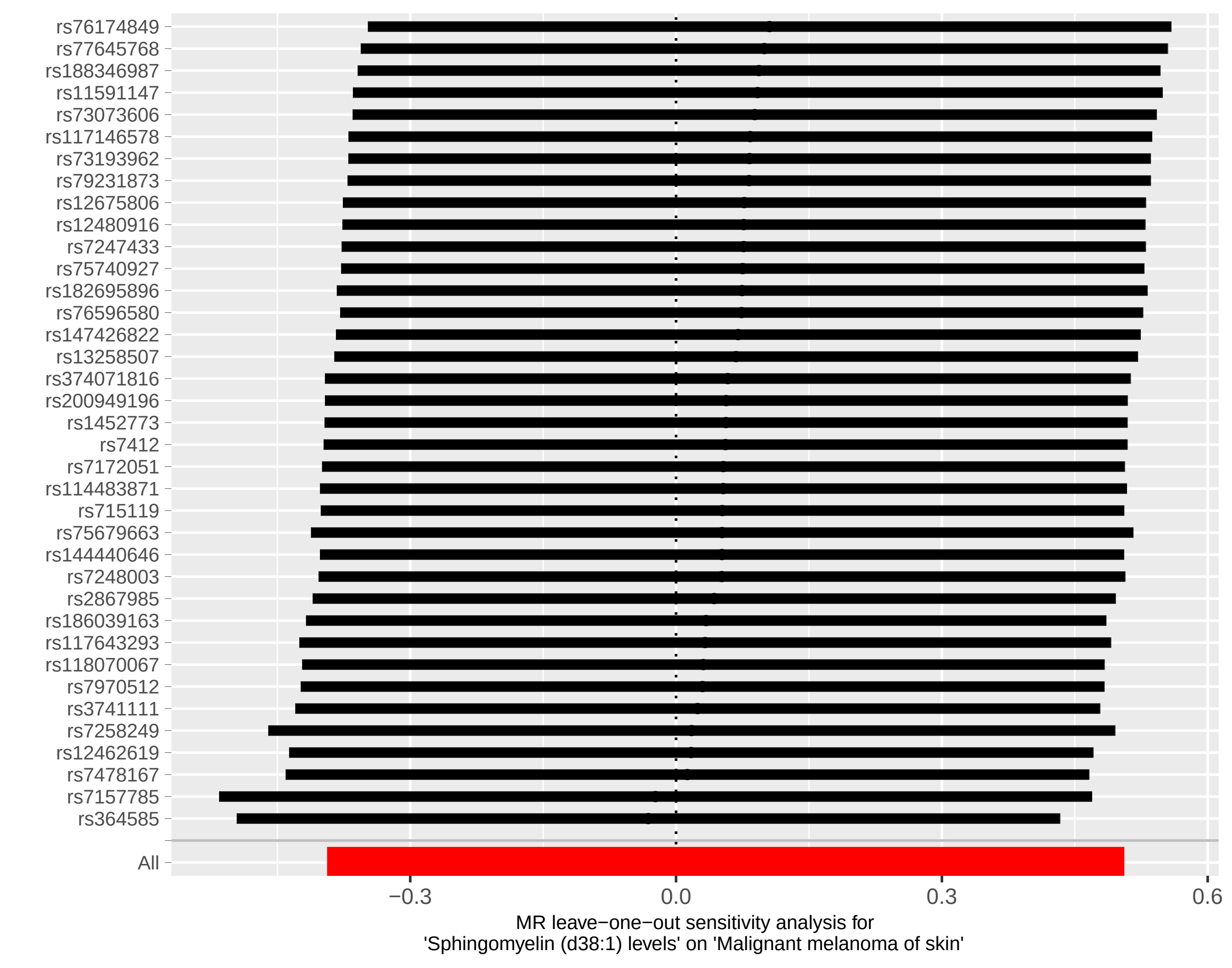
rs76174849
rs77645768
rs188346987
rs11591147
rs73073606
rs117146578
rs73193962
rs79231873
rs12675806
rs12480916
rs7247433
rs75740927
rs182695896
rs76596580
rs147426822
rs13258507
rs374071816
rs200949196
rs1452773
rs7412
rs7172051
rs114483871
rs715119
rs75679663
rs144440646
rs7248003
rs2867985
rs186039163
rs117643293
rs118070067
rs7970512
rs3741111
rs7258249
rs12462619
rs7478167
rs7157785
rs364585
All
−0.3
0.0
0.3
0.6
MR leave−one−out sensitivity analysis for
'Sphingomyelin (d38:1) levels' on 'Malignant melanoma of skin'

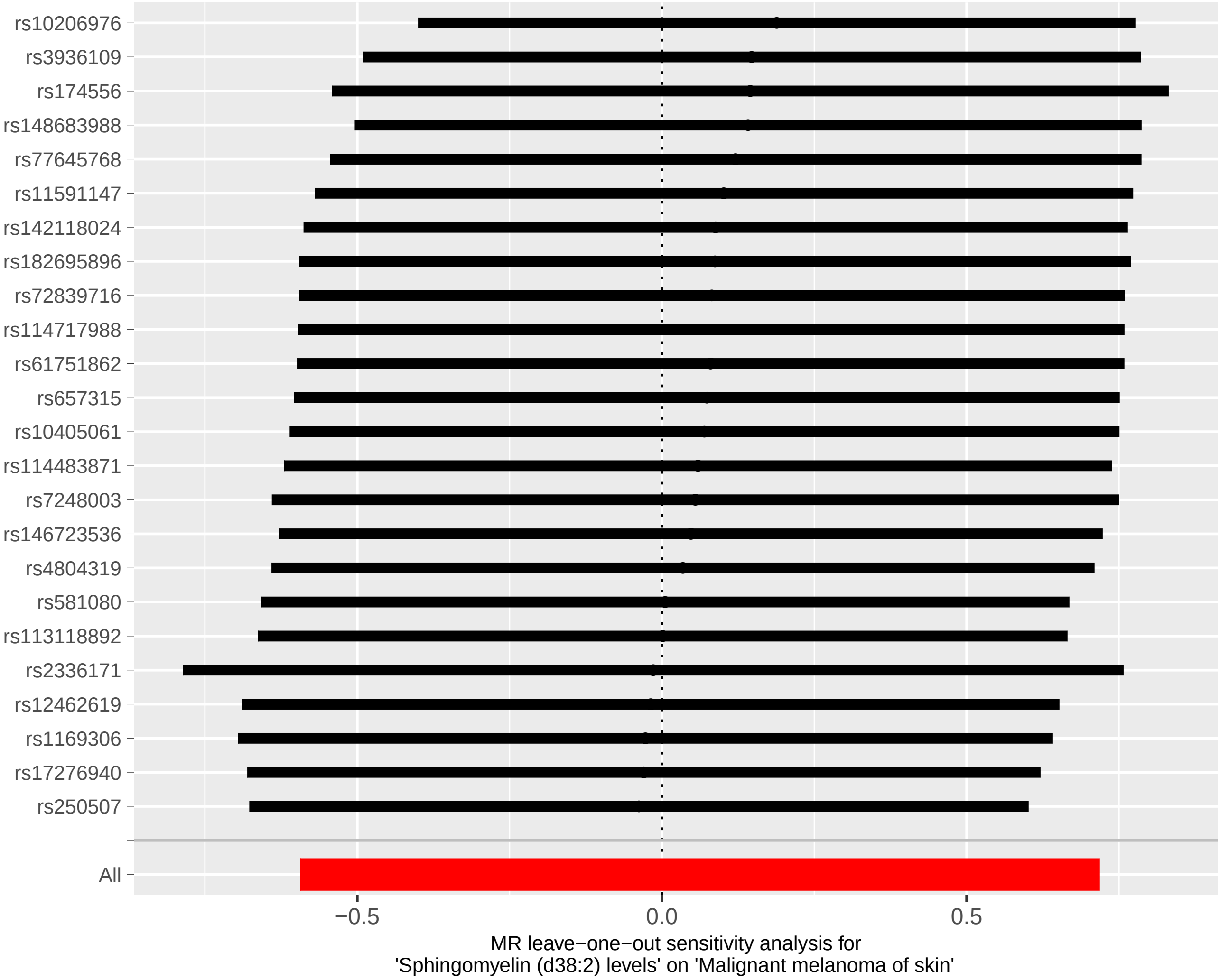
rs10206976
rs3936109
rs174556
rs148683988
rs77645768
rs11591147
rs142118024
rs182695896
rs72839716
rs114717988
rs61751862
rs657315
rs10405061
rs114483871
rs7248003
rs146723536
rs4804319
rs581080
rs113118892
rs2336171
rs12462619
rs1169306
rs17276940
rs250507
All
−0.5
0.0
0.5
MR leave−one−out sensitivity analysis for
'Sphingomyelin (d38:2) levels' on 'Malignant melanoma of skin'

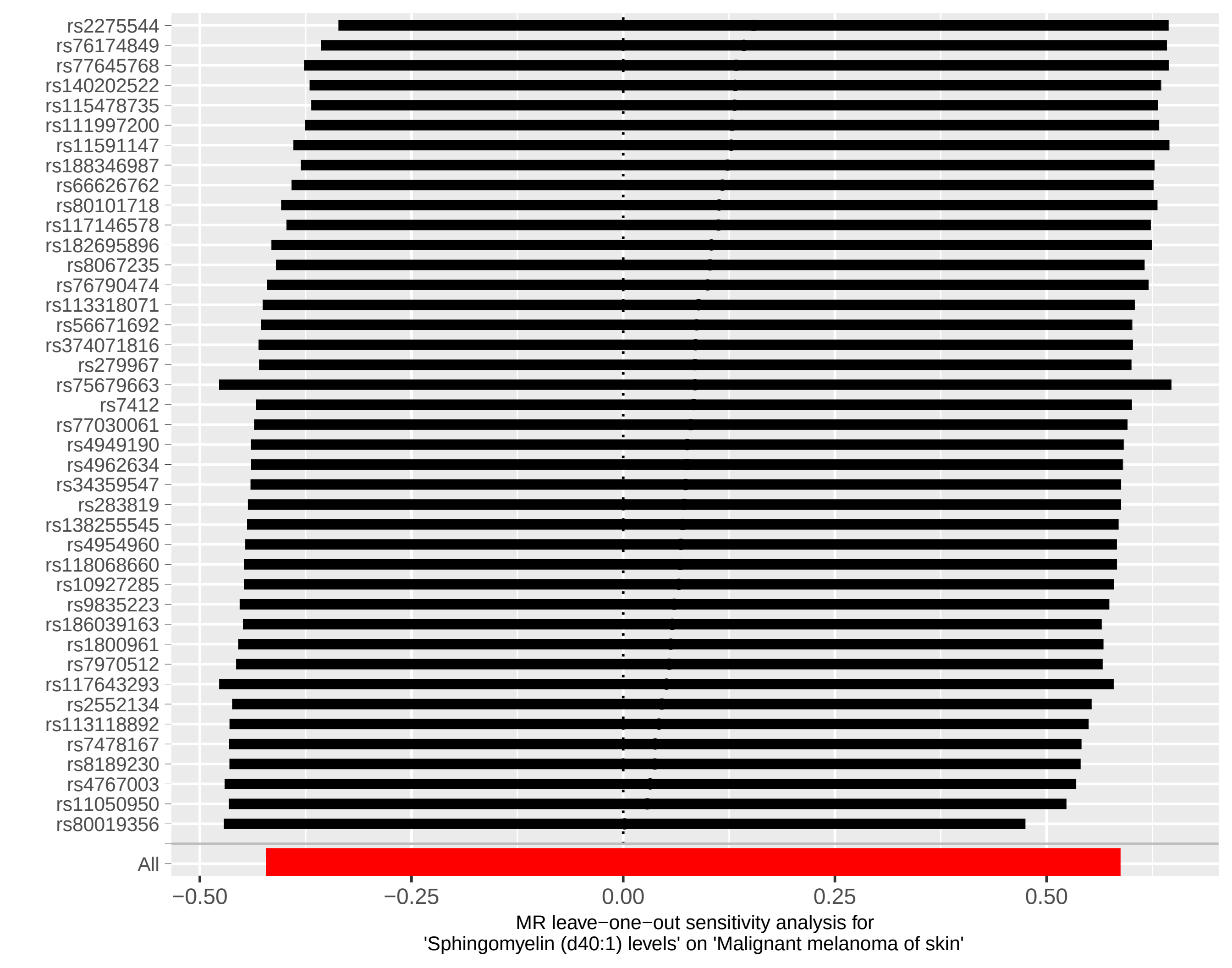
rs2275544
rs76174849
rs77645768
rs140202522
rs115478735
rs111997200
rs11591147
rs188346987
rs66626762
rs80101718
rs117146578
rs182695896
rs8067235
rs76790474
rs113318071
rs56671692
rs374071816
rs279967
rs75679663
rs7412
rs77030061
rs4949190
rs4962634
rs34359547
rs283819
rs138255545
rs4954960
rs118068660
rs10927285
rs9835223
rs186039163
rs1800961
rs7970512
rs117643293
rs2552134
rs113118892
rs7478167
rs8189230
rs4767003
rs11050950
rs80019356
All
−0.50
−0.25
0.00
0.25
0.50
MR leave−one−out sensitivity analysis for
'Sphingomyelin (d40:1) levels' on 'Malignant melanoma of skin'

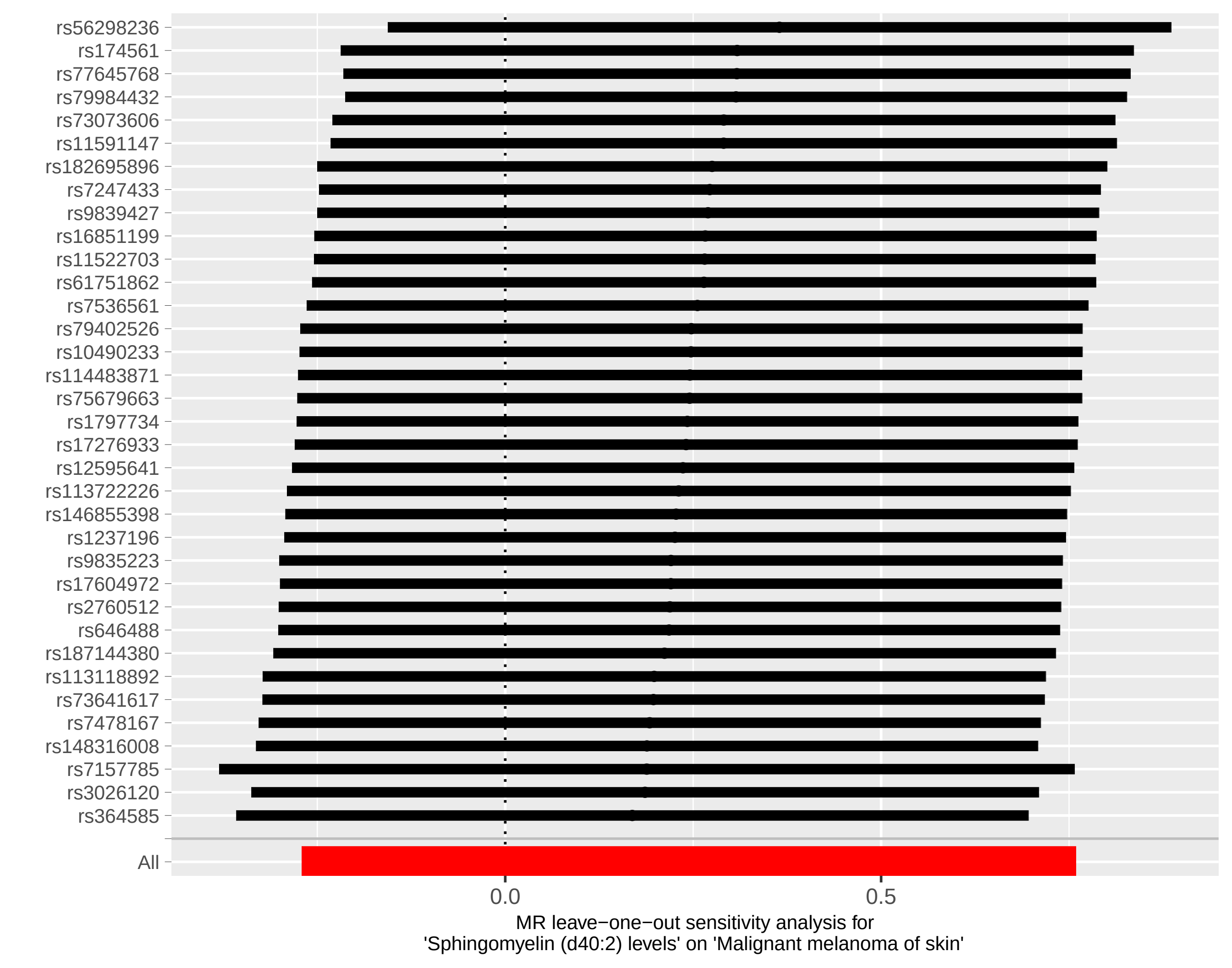
rs56298236
rs174561
rs77645768
rs79984432
rs73073606
rs11591147
rs182695896
rs7247433
rs9839427
rs16851199
rs11522703
rs61751862
rs7536561
rs79402526
rs10490233
rs114483871
rs75679663
rs1797734
rs17276933
rs12595641
rs113722226
rs146855398
rs1237196
rs9835223
rs17604972
rs2760512
rs646488
rs187144380
rs113118892
rs73641617
rs7478167
rs148316008
rs7157785
rs3026120
rs364585
All
0.0
0.5
MR leave−one−out sensitivity analysis for
'Sphingomyelin (d40:2) levels' on 'Malignant melanoma of skin'

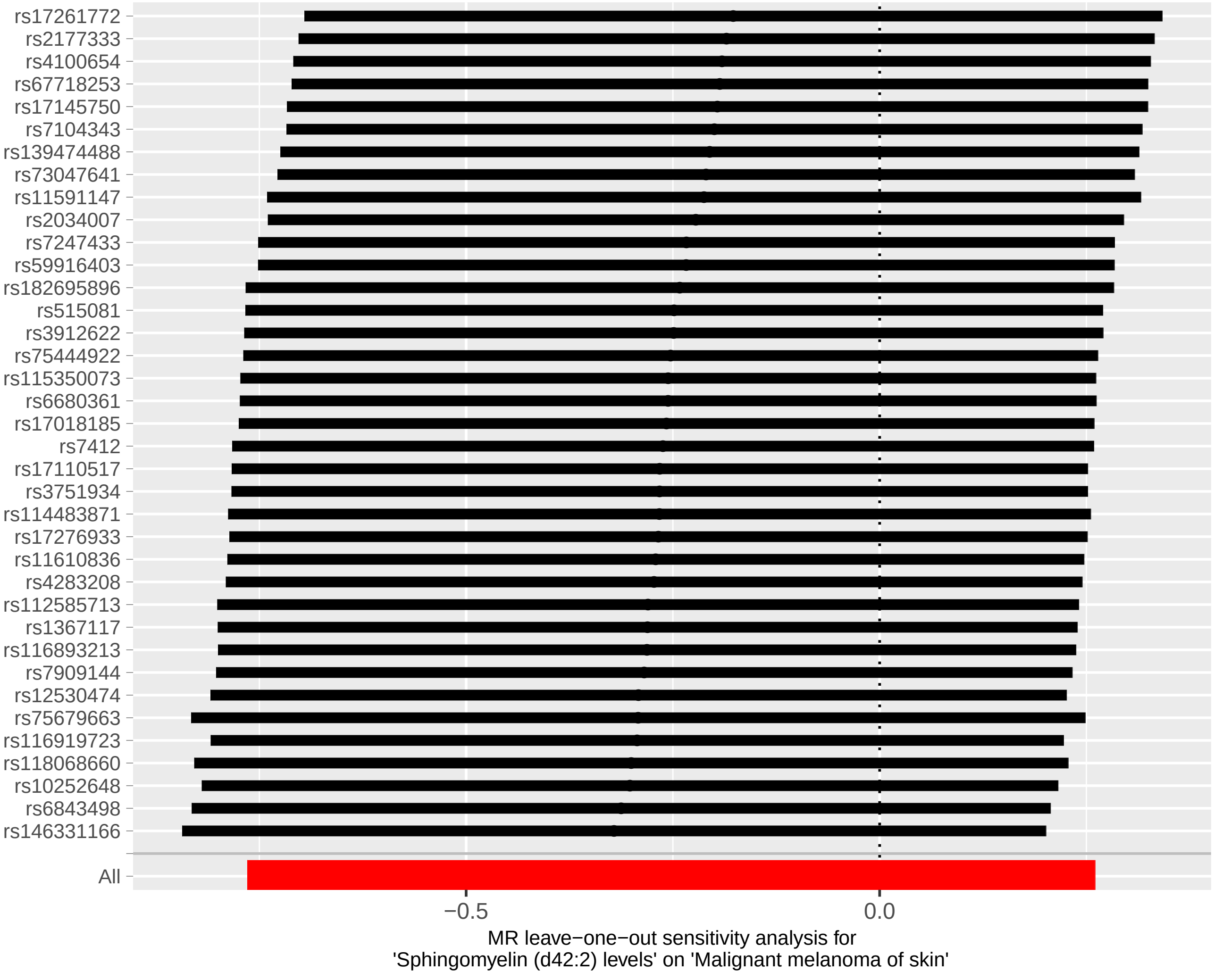

rs17261772
rs2177333
rs4100654
rs67718253
rs17145750
rs7104343
rs139474488
rs73047641
rs11591147
rs2034007
rs7247433
rs59916403
rs182695896
rs515081
rs3912622
rs75444922
rs115350073
rs6680361
rs17018185
rs7412
rs17110517
rs3751934
rs114483871
rs17276933
rs11610836
rs4283208
rs112585713
rs1367117
rs116893213
rs7909144
rs12530474
rs75679663
rs116919723
rs118068660
rs10252648
rs6843498
rs146331166
All
−0.5
0.0
MR leave−one−out sensitivity analysis for
'Sphingomyelin (d42:2) levels' on 'Malignant melanoma of skin'

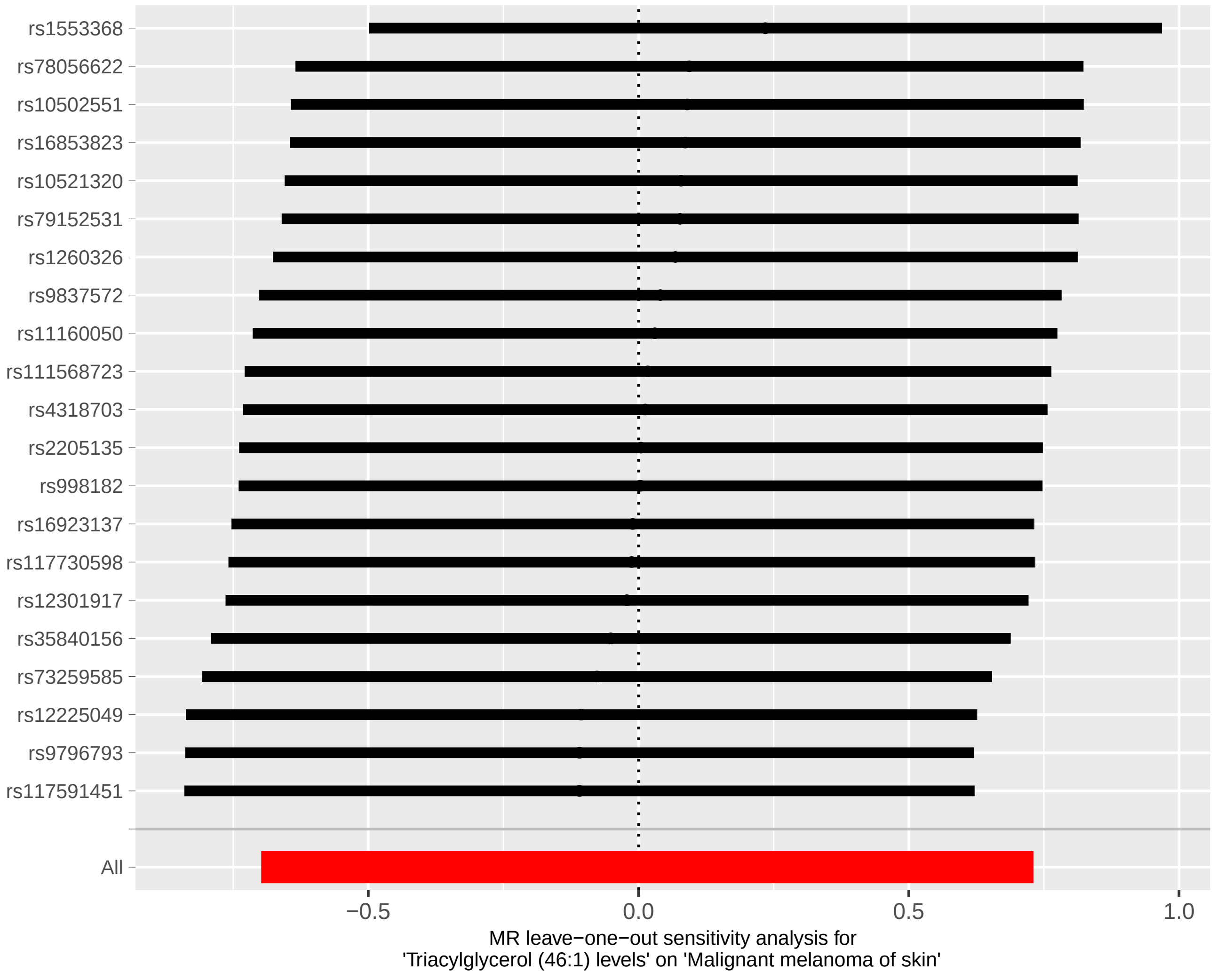
rs1553368
rs78056622
rs10502551
rs16853823
rs10521320
rs79152531
rs1260326
rs9837572
rs11160050
rs111568723
rs4318703
rs2205135
rs998182
rs16923137
rs117730598
rs12301917
rs35840156
rs73259585
rs12225049
rs9796793
rs117591451
All
−0.5
0.0
0.5
1.0
MR leave−one−out sensitivity analysis for
'Triacylglycerol (46:1) levels' on 'Malignant melanoma of skin'

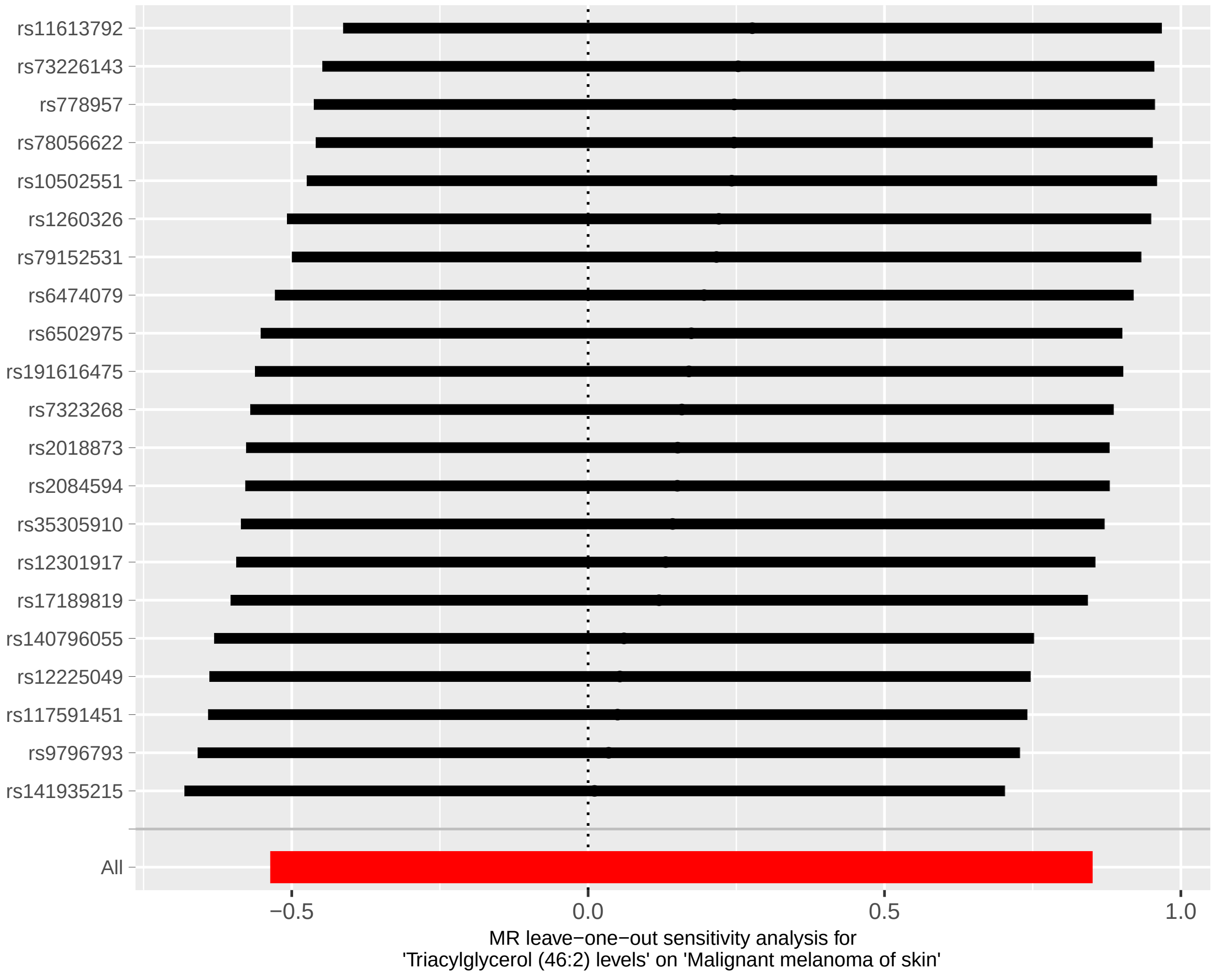

rs11613792
rs73226143
rs778957
rs78056622
rs10502551
rs1260326
rs79152531
rs6474079
rs6502975
rs191616475
rs7323268
rs2018873
rs2084594
rs35305910
rs12301917
rs17189819
rs140796055
rs12225049
rs117591451
rs9796793
rs141935215
All
−0.5
0.0
0.5
1.0
MR leave−one−out sensitivity analysis for
'Triacylglycerol (46:2) levels' on 'Malignant melanoma of skin'

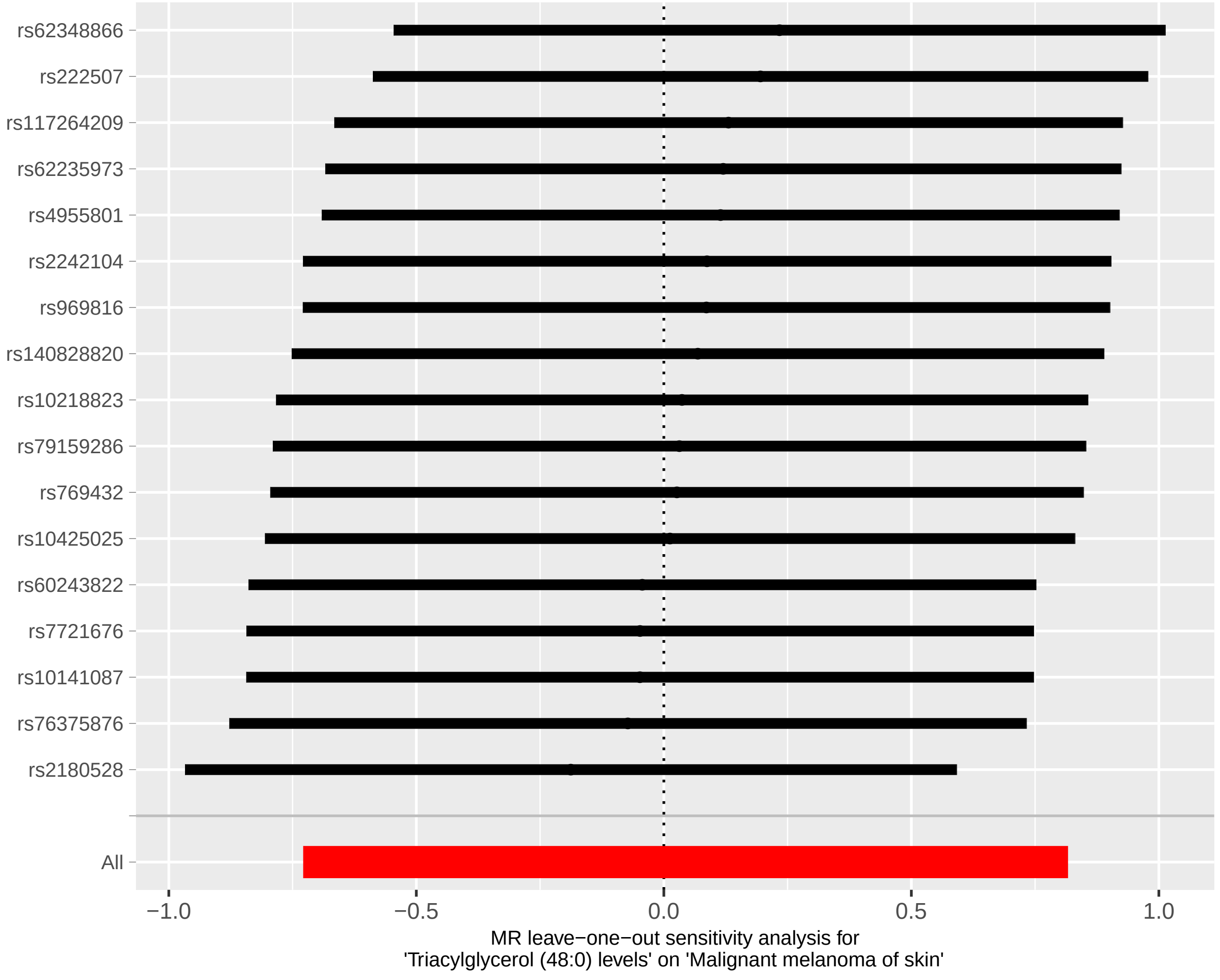
rs62348866
rs222507
rs117264209
rs62235973
rs4955801
rs2242104
rs969816
rs140828820
rs10218823
rs79159286
rs769432
rs10425025
rs60243822
rs7721676
rs10141087
rs76375876
rs2180528
All
−1.0
−0.5
0.0
0.5
1.0
MR leave−one−out sensitivity analysis for
'Triacylglycerol (48:0) levels' on 'Malignant melanoma of skin'

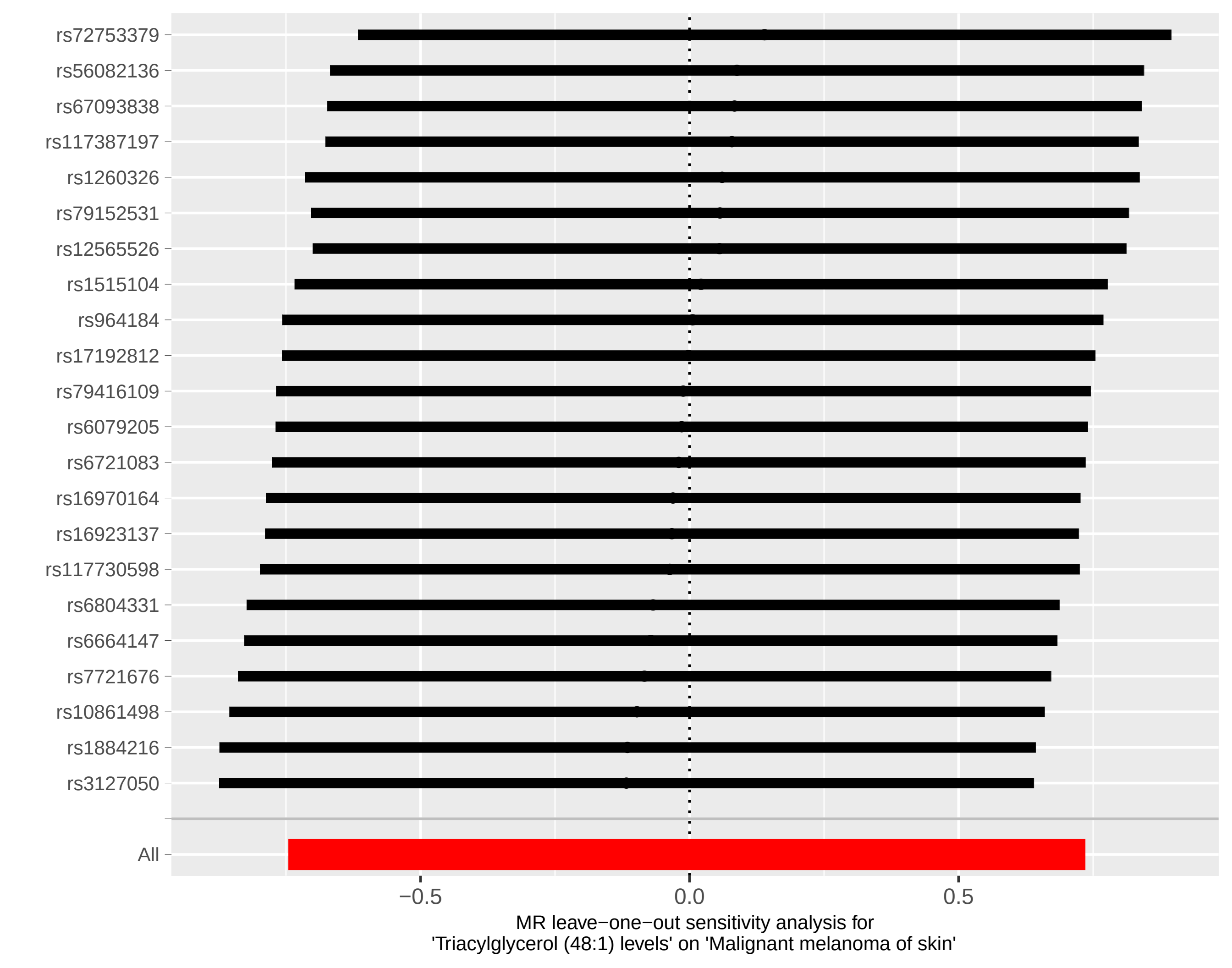
rs72753379
rs56082136
rs67093838
rs117387197
rs1260326
rs79152531
rs12565526
rs1515104
rs964184
rs17192812
rs79416109
rs6079205
rs6721083
rs16970164
rs16923137
rs117730598
rs6804331
rs6664147
rs7721676
rs10861498
rs1884216
rs3127050
All
−0.5
0.0
0.5
MR leave−one−out sensitivity analysis for
'Triacylglycerol (48:1) levels' on 'Malignant melanoma of skin'

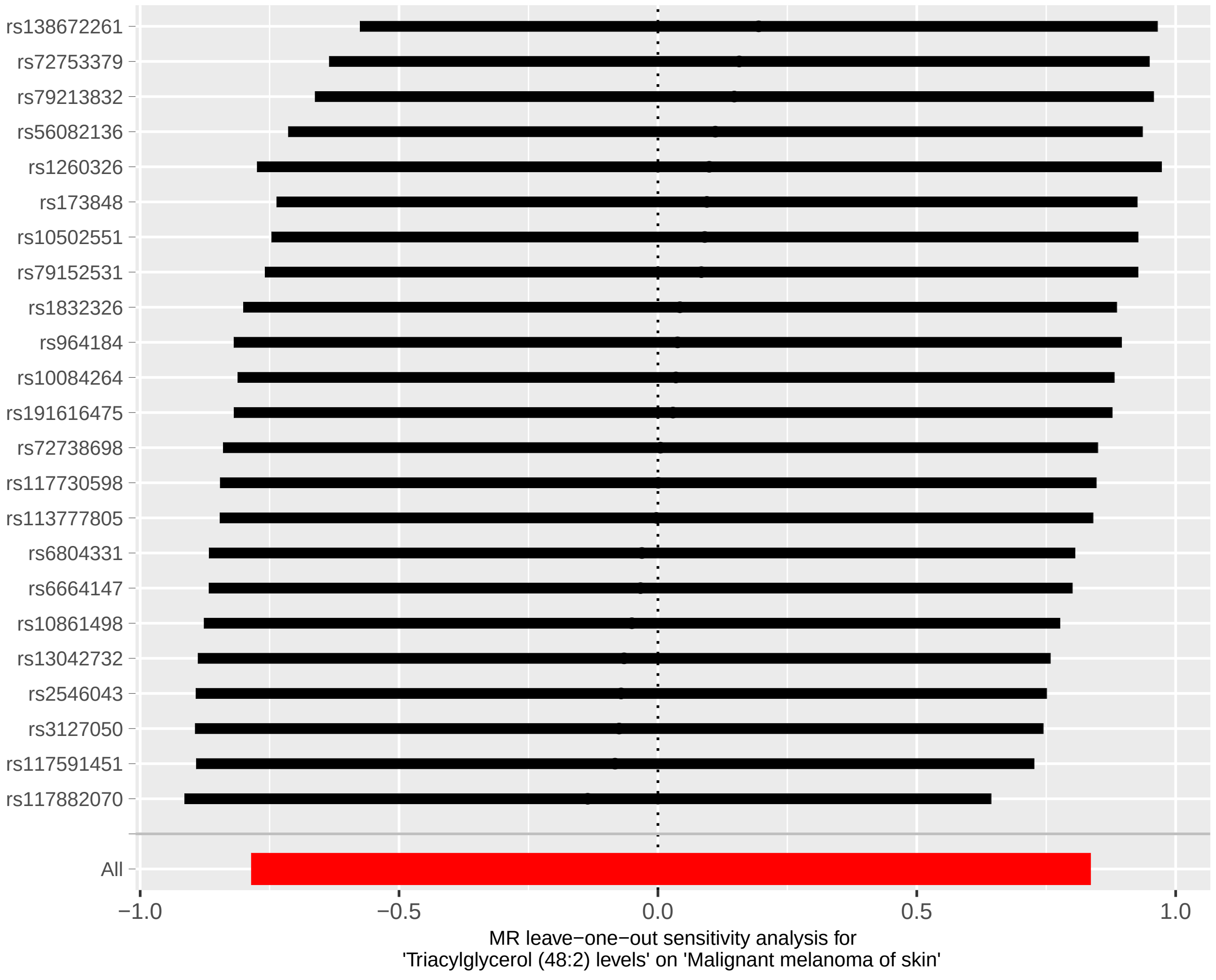
rs138672261
rs72753379
rs79213832
rs56082136
rs1260326
rs173848
rs10502551
rs79152531
rs1832326
rs964184
rs10084264
rs191616475
rs72738698
rs117730598
rs113777805
rs6804331
rs6664147
rs10861498
rs13042732
rs2546043
rs3127050
rs117591451
rs117882070
All
−1.0
−0.5
0.0
0.5
1.0
MR leave−one−out sensitivity analysis for
'Triacylglycerol (48:2) levels' on 'Malignant melanoma of skin'

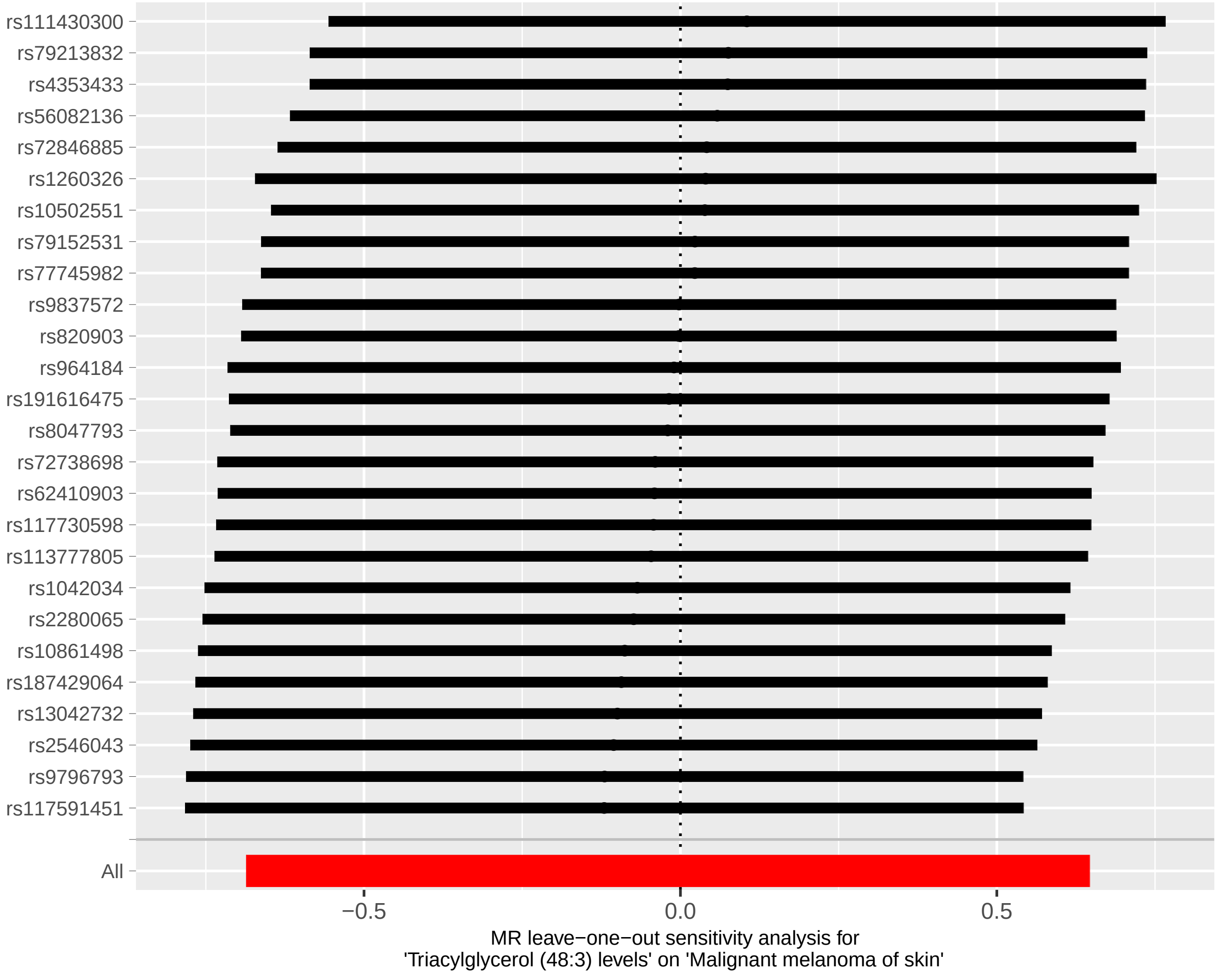
rs111430300
rs79213832
rs4353433
rs56082136
rs72846885
rs1260326
rs10502551
rs79152531
rs77745982
rs9837572
rs820903
rs964184
rs191616475
rs8047793
rs72738698
rs62410903
rs117730598
rs113777805
rs1042034
rs2280065
rs10861498
rs187429064
rs13042732
rs2546043
rs9796793
rs117591451
All
−0.5
0.0
0.5
MR leave−one−out sensitivity analysis for
'Triacylglycerol (48:3) levels' on 'Malignant melanoma of skin'

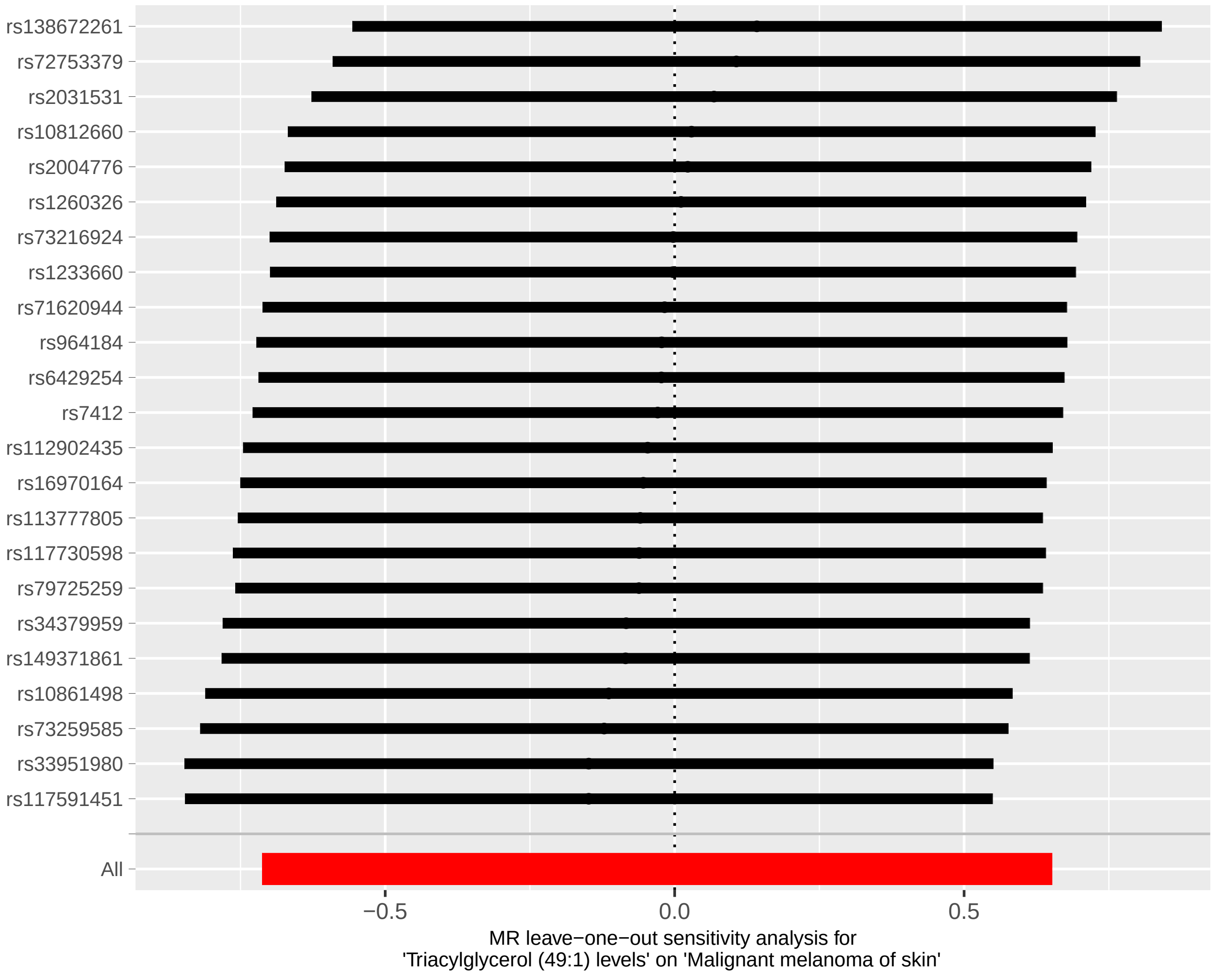
rs138672261
rs72753379
rs2031531
rs10812660
rs2004776
rs1260326
rs73216924
rs1233660
rs71620944
rs964184
rs6429254
rs7412
rs112902435
rs16970164
rs113777805
rs117730598
rs79725259
rs34379959
rs149371861
rs10861498
rs73259585
rs33951980
rs117591451
All
−0.5
0.0
0.5
MR leave−one−out sensitivity analysis for
'Triacylglycerol (49:1) levels' on 'Malignant melanoma of skin'

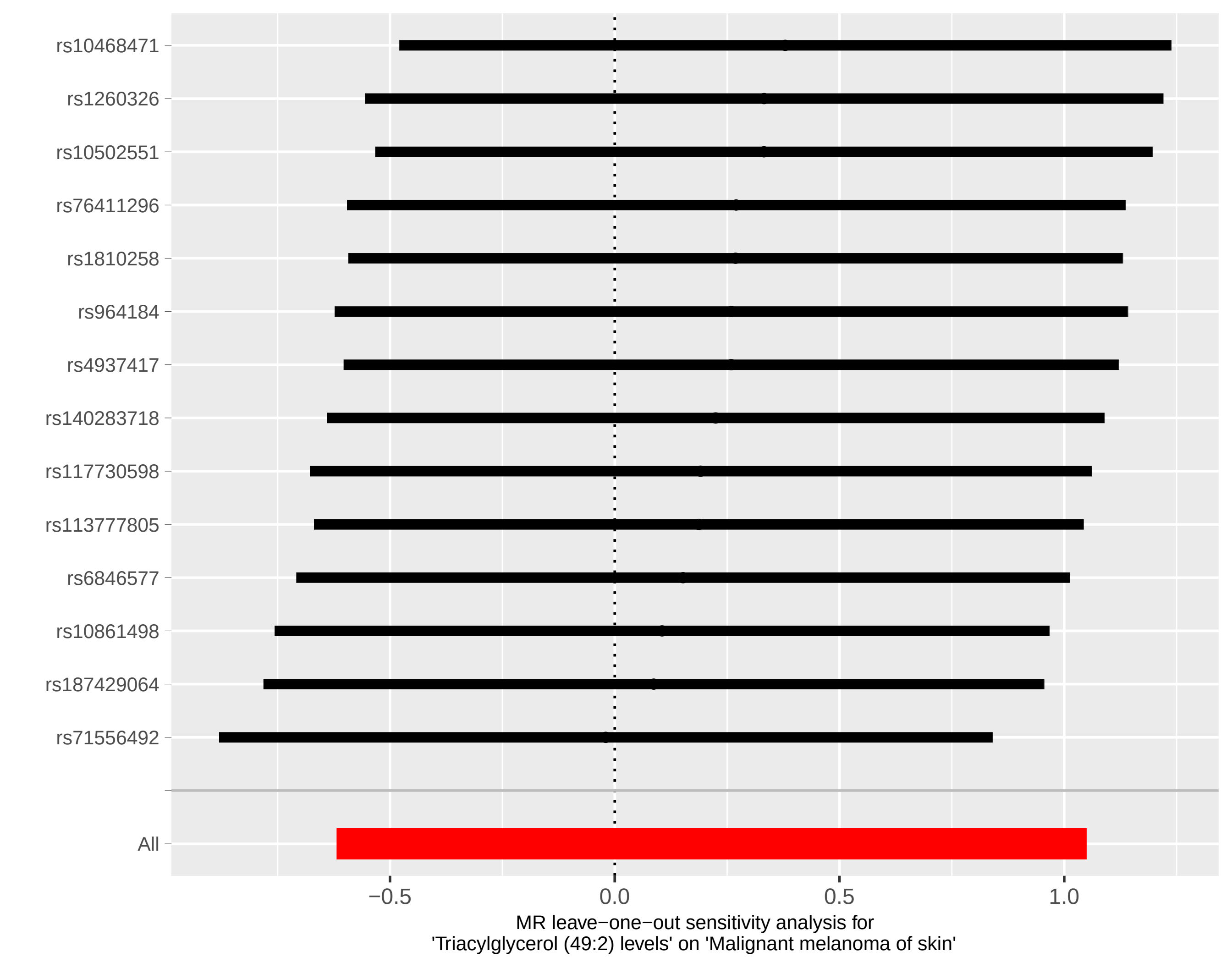

rs10468471
rs1260326
rs10502551
rs76411296
rs1810258
rs964184
rs4937417
rs140283718
rs117730598
rs113777805
rs6846577
rs10861498
rs187429064
rs71556492
All
−0.5
0.0
0.5
1.0
MR leave−one−out sensitivity analysis for
'Triacylglycerol (49:2) levels' on 'Malignant melanoma of skin'

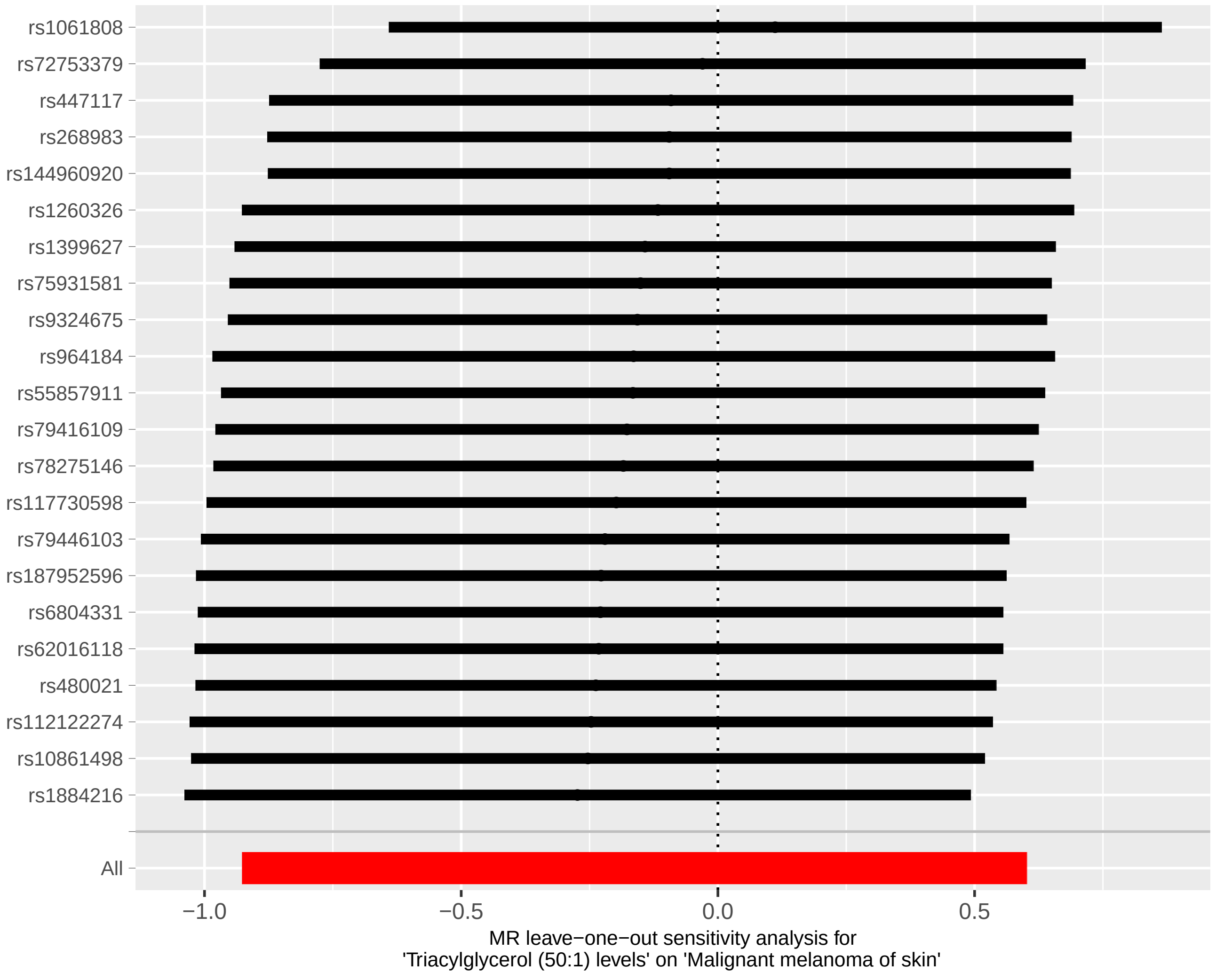
rs1061808
rs72753379
rs447117
rs268983
rs144960920
rs1260326
rs1399627
rs75931581
rs9324675
rs964184
rs55857911
rs79416109
rs78275146
rs117730598
rs79446103
rs187952596
rs6804331
rs62016118
rs480021
rs112122274
rs10861498
rs1884216
All
−1.0
−0.5
0.0
0.5
MR leave−one−out sensitivity analysis for
'Triacylglycerol (50:1) levels' on 'Malignant melanoma of skin'

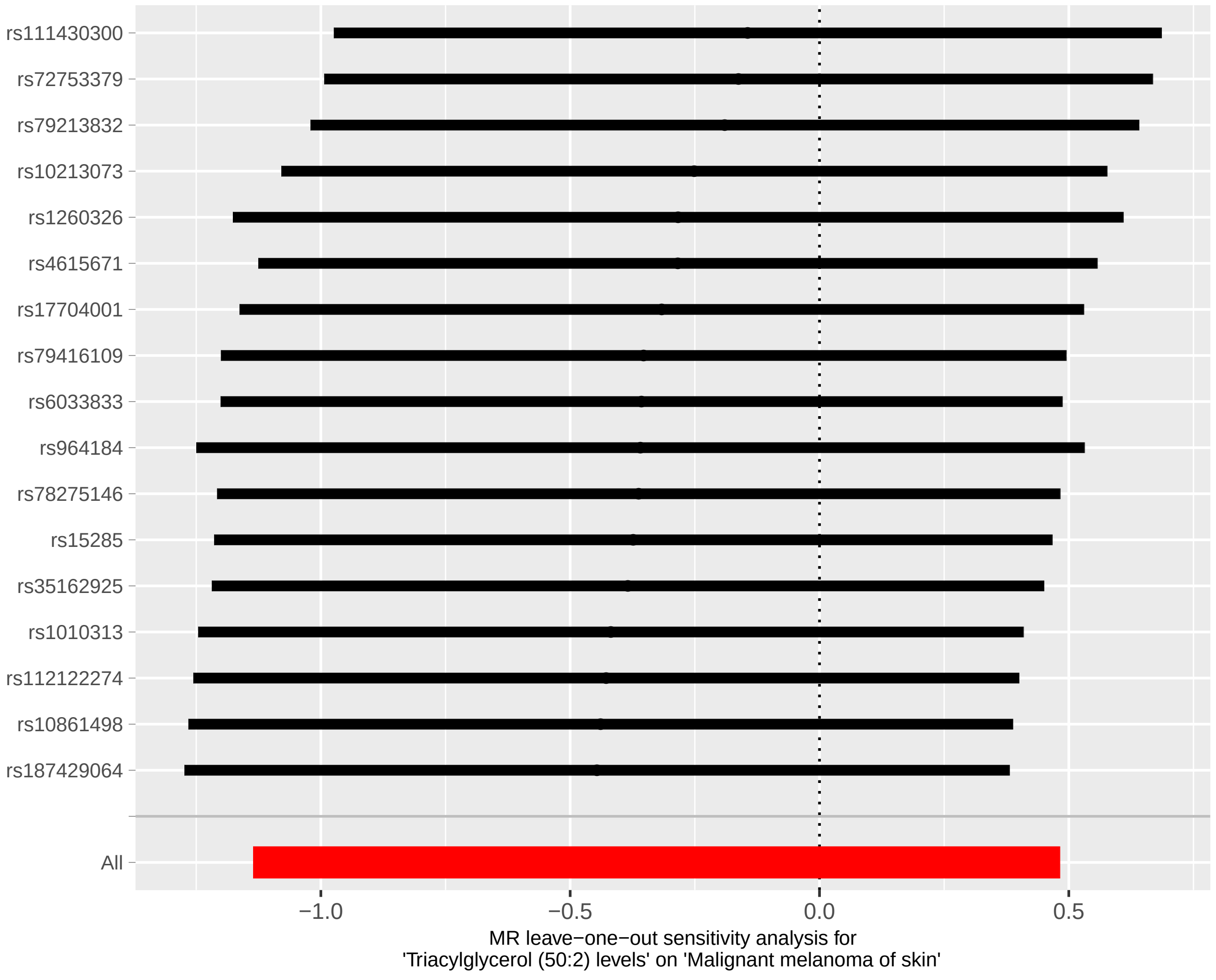
rs111430300
rs72753379
rs79213832
rs10213073
rs1260326
rs4615671
rs17704001
rs79416109
rs6033833
rs964184
rs78275146
rs15285
rs35162925
rs1010313
rs112122274
rs10861498
rs187429064
All
−1.0
−0.5
0.0
0.5
MR leave−one−out sensitivity analysis for
'Triacylglycerol (50:2) levels' on 'Malignant melanoma of skin'

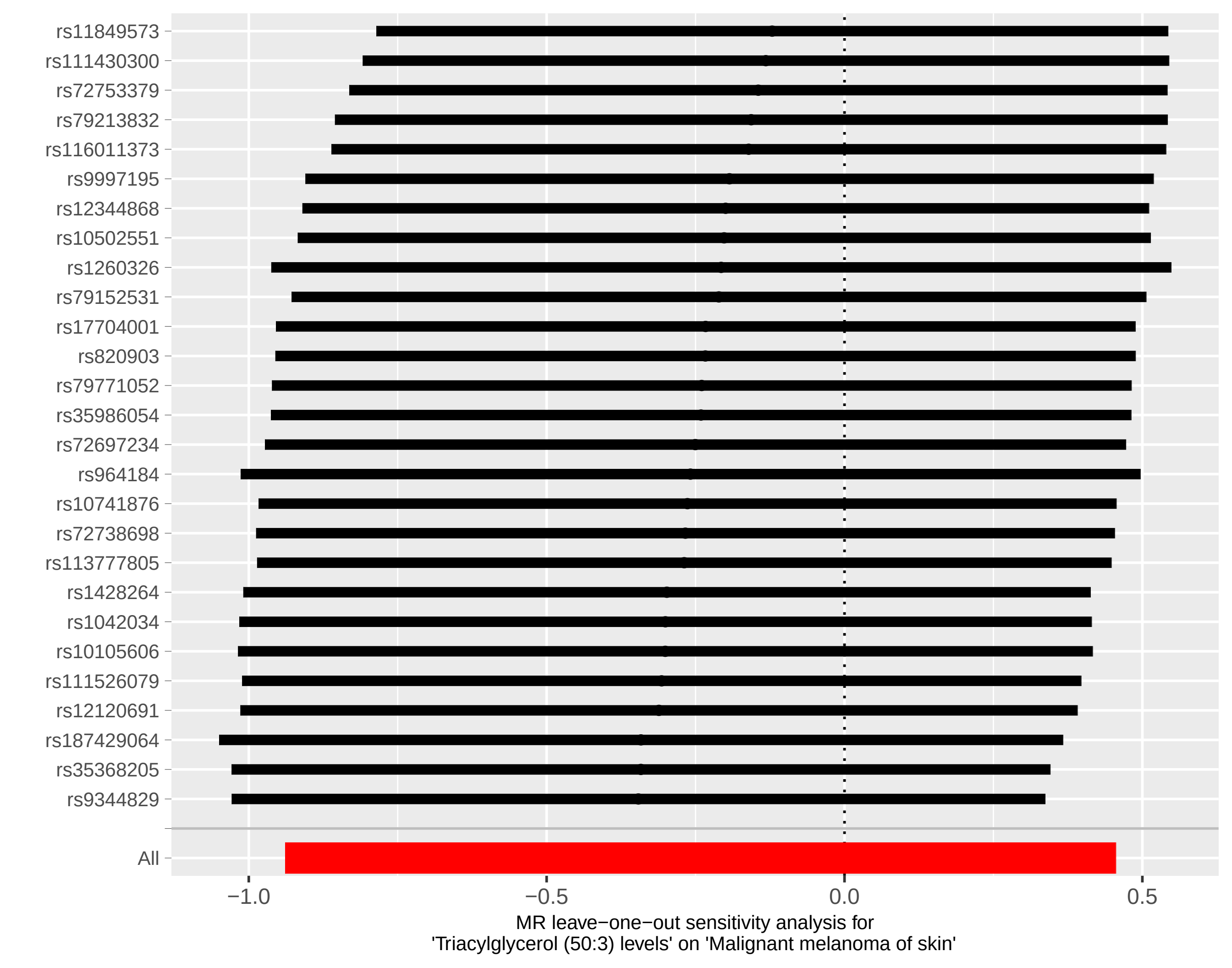
rs11849573
rs111430300
rs72753379
rs79213832
rs116011373
rs9997195
rs12344868
rs10502551
rs1260326
rs79152531
rs17704001
rs820903
rs79771052
rs35986054
rs72697234
rs964184
rs10741876
rs72738698
rs113777805
rs1428264
rs1042034
rs10105606
rs111526079
rs12120691
rs187429064
rs35368205
rs9344829
All
−1.0
−0.5
0.0
0.5
MR leave−one−out sensitivity analysis for
'Triacylglycerol (50:3) levels' on 'Malignant melanoma of skin'

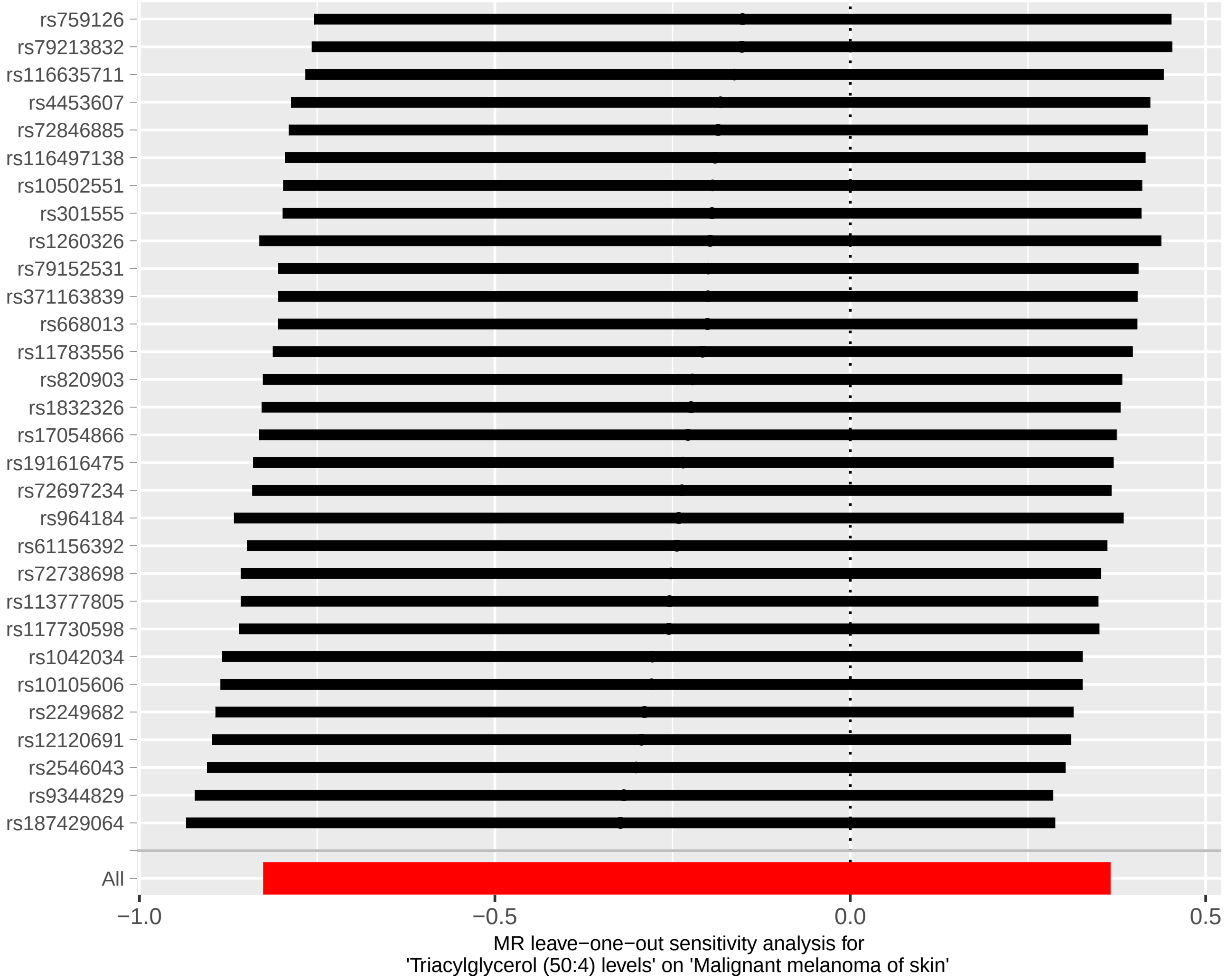

rs759126
rs79213832
rs116635711
rs4453607
rs72846885
rs116497138
rs10502551
rs301555
rs1260326
rs79152531
rs371163839
rs668013
rs11783556
rs820903
rs1832326
rs17054866
rs191616475
rs72697234
rs964184
rs61156392
rs72738698
rs113777805
rs117730598
rs1042034
rs10105606
rs2249682
rs12120691
rs2546043
rs9344829
rs187429064
All
−1.0
−0.5
0.0
0.5
MR leave−one−out sensitivity analysis for
'Triacylglycerol (50:4) levels' on 'Malignant melanoma of skin'

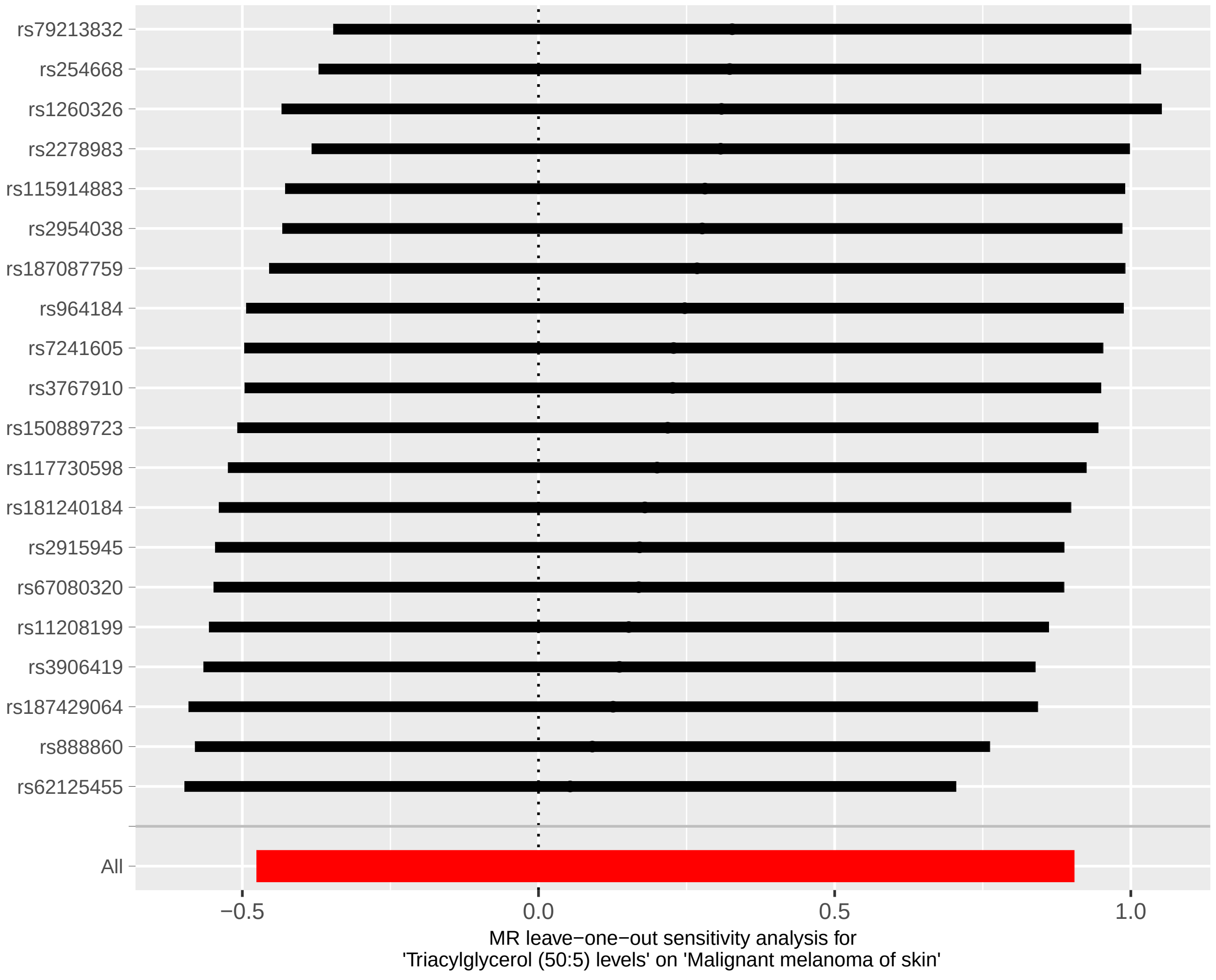
rs79213832
rs254668
rs1260326
rs2278983
rs115914883
rs2954038
rs187087759
rs964184
rs7241605
rs3767910
rs150889723
rs117730598
rs181240184
rs2915945
rs67080320
rs11208199
rs3906419
rs187429064
rs888860
rs62125455
All
−0.5
0.0
0.5
1.0
MR leave−one−out sensitivity analysis for
'Triacylglycerol (50:5) levels' on 'Malignant melanoma of skin'

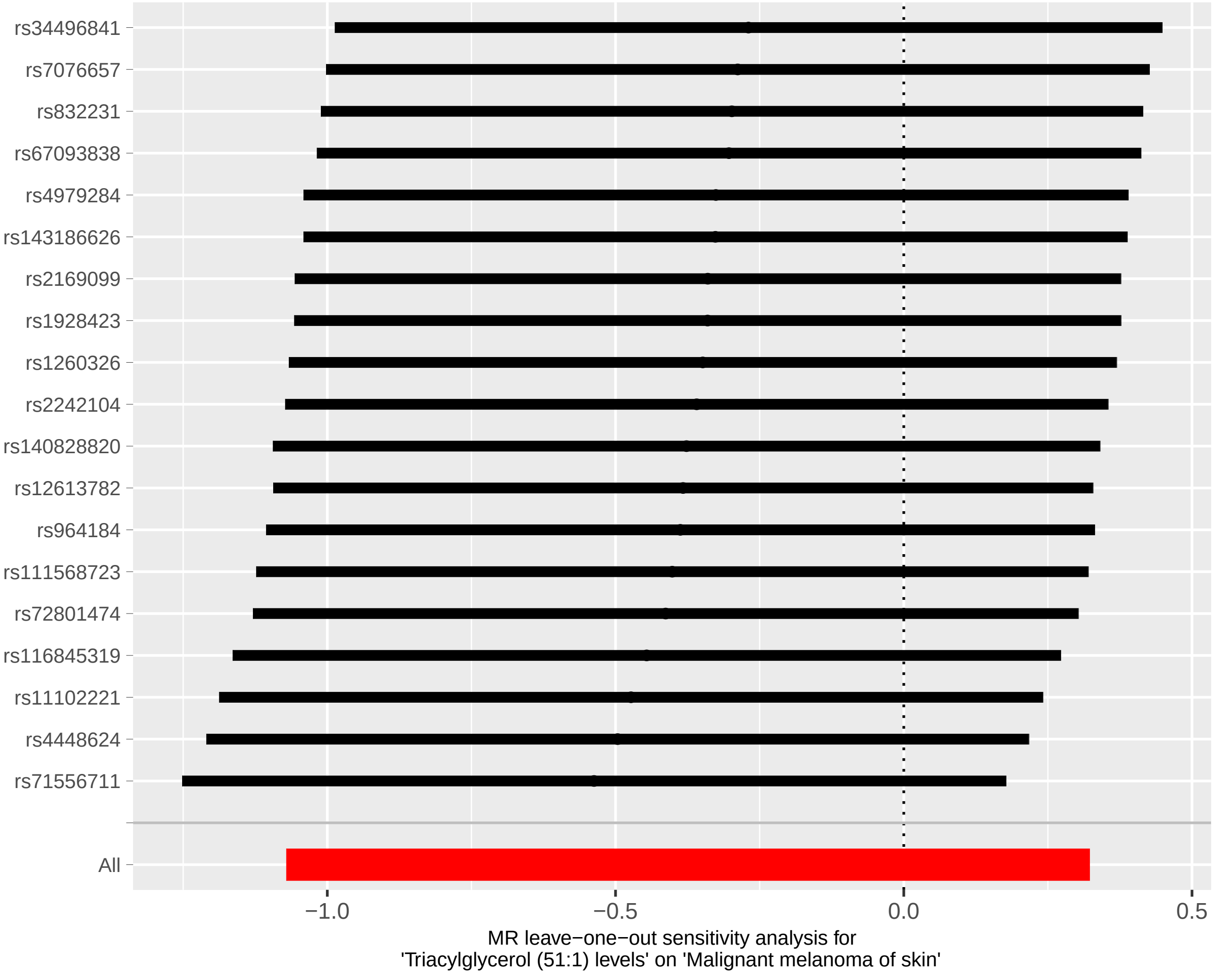
rs34496841
rs7076657
rs832231
rs67093838
rs4979284
rs143186626
rs2169099
rs1928423
rs1260326
rs2242104
rs140828820
rs12613782
rs964184
rs111568723
rs72801474
rs116845319
rs11102221
rs4448624
rs71556711
All
−1.0
−0.5
0.0
0.5
MR leave−one−out sensitivity analysis for
'Triacylglycerol (51:1) levels' on 'Malignant melanoma of skin'

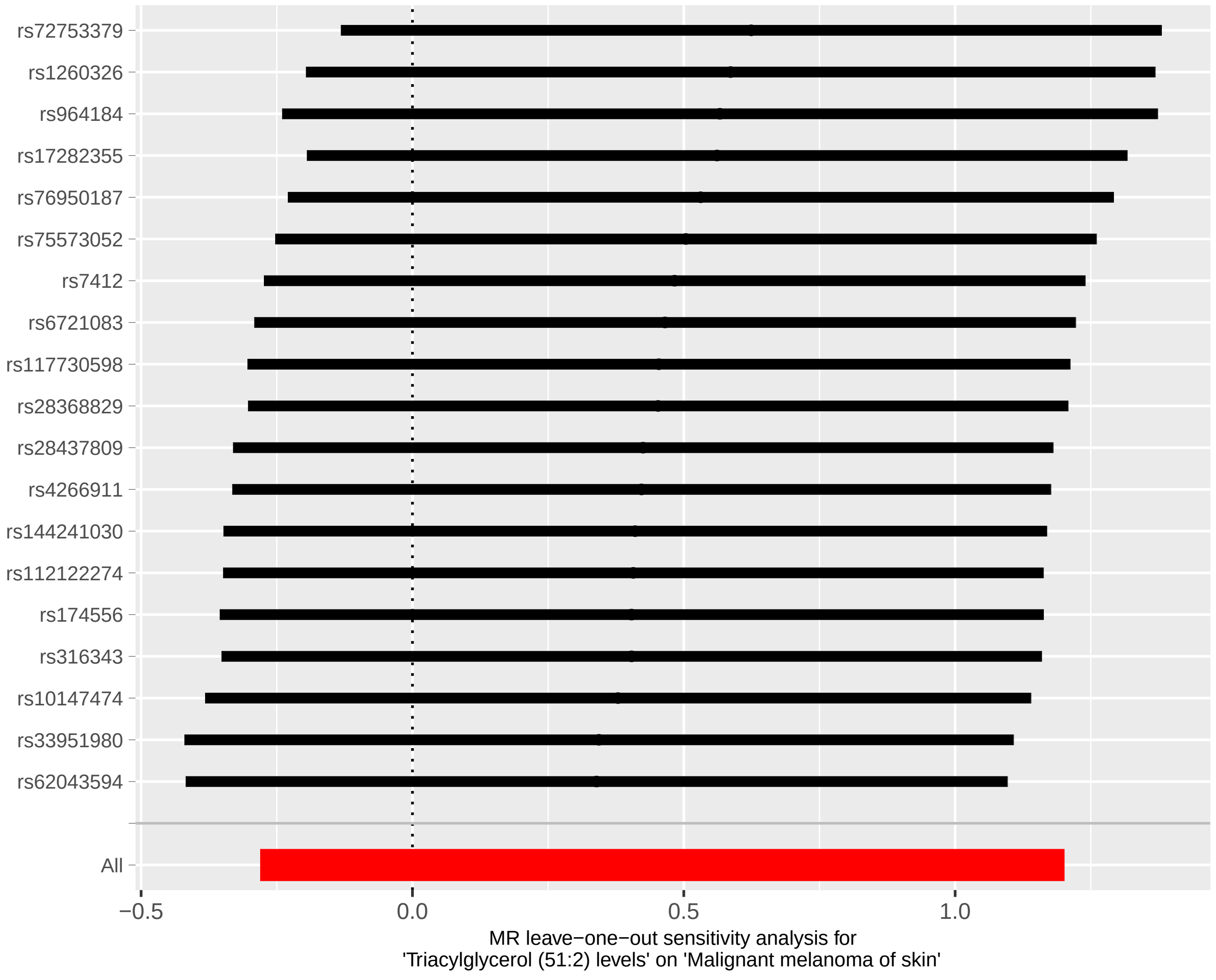
rs72753379
rs1260326
rs964184
rs17282355
rs76950187
rs75573052
rs7412
rs6721083
rs117730598
rs28368829
rs28437809
rs4266911
rs144241030
rs112122274
rs174556
rs316343
rs10147474
rs33951980
rs62043594
All
−0.5
0.0
0.5
1.0
MR leave−one−out sensitivity analysis for
'Triacylglycerol (51:2) levels' on 'Malignant melanoma of skin'

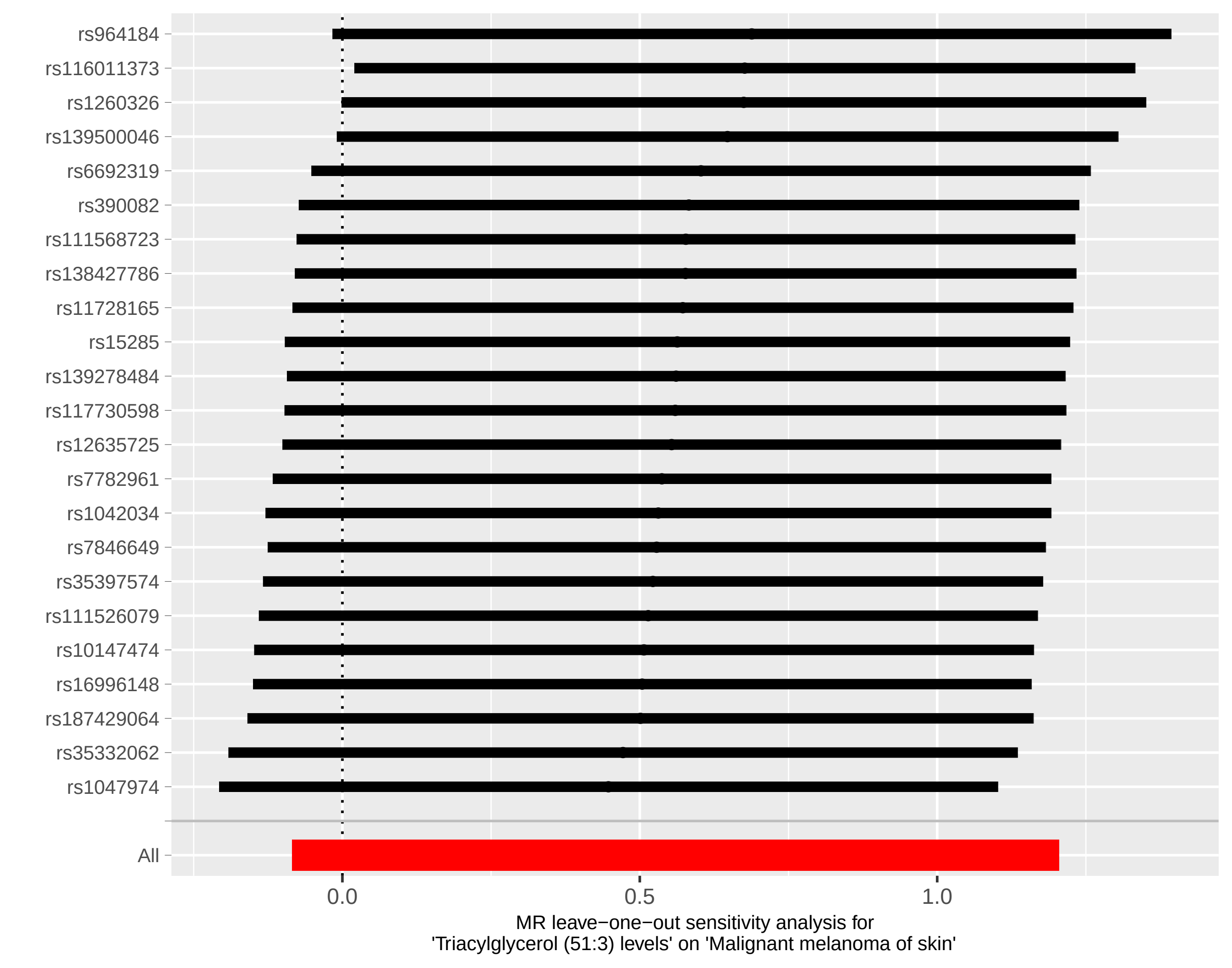
rs964184
rs116011373
rs1260326
rs139500046
rs6692319
rs390082
rs111568723
rs138427786
rs11728165
rs15285
rs139278484
rs117730598
rs12635725
rs7782961
rs1042034
rs7846649
rs35397574
rs111526079
rs10147474
rs16996148
rs187429064
rs35332062
rs1047974
All
0.0
0.5
1.0
MR leave−one−out sensitivity analysis for
'Triacylglycerol (51:3) levels' on 'Malignant melanoma of skin'

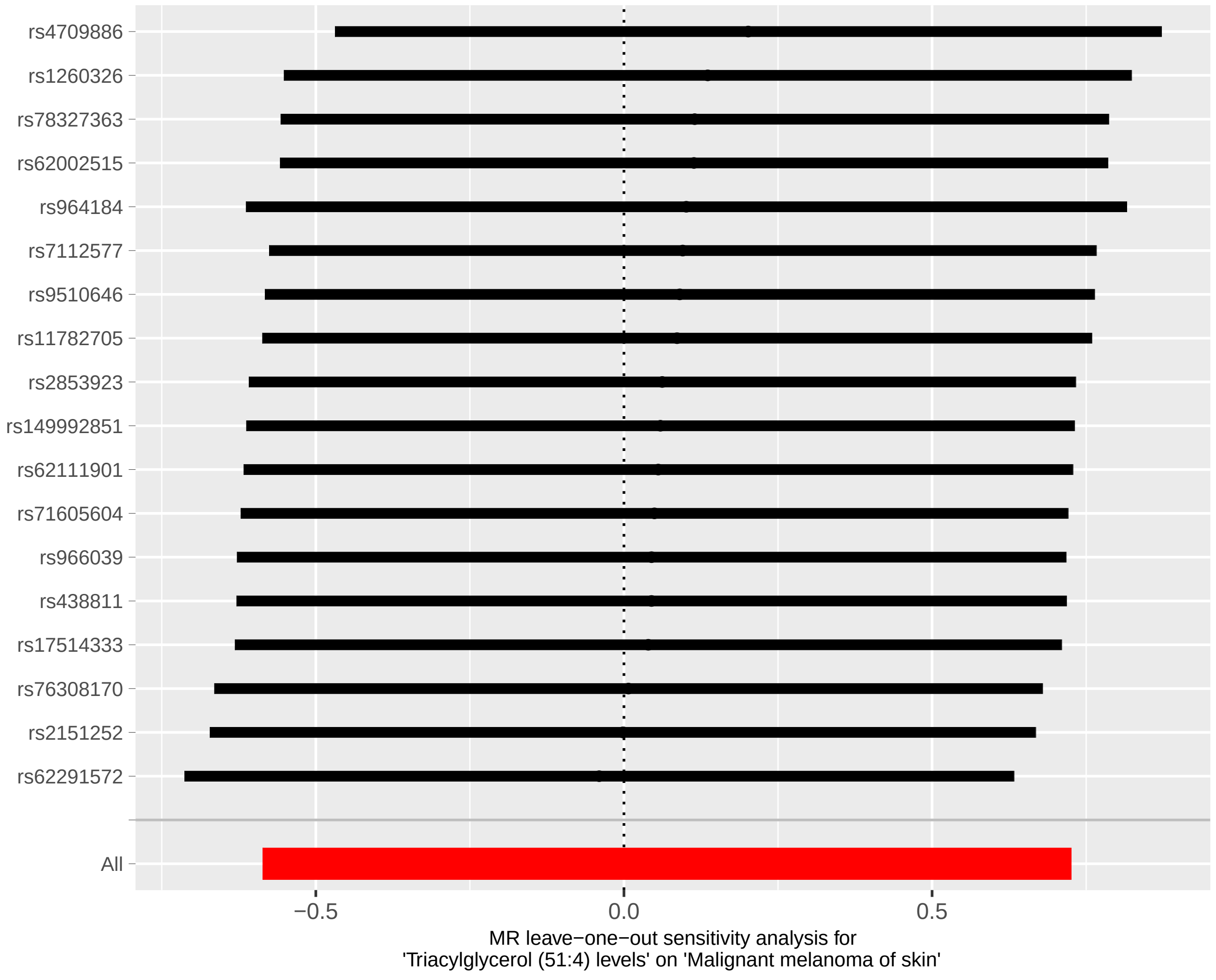

rs4709886
rs1260326
rs78327363
rs62002515
rs964184
rs7112577
rs9510646
rs11782705
rs2853923
rs149992851
rs62111901
rs71605604
rs966039
rs438811
rs17514333
rs76308170
rs2151252
rs62291572
All
−0.5
0.0
0.5
MR leave−one−out sensitivity analysis for
'Triacylglycerol (51:4) levels' on 'Malignant melanoma of skin'

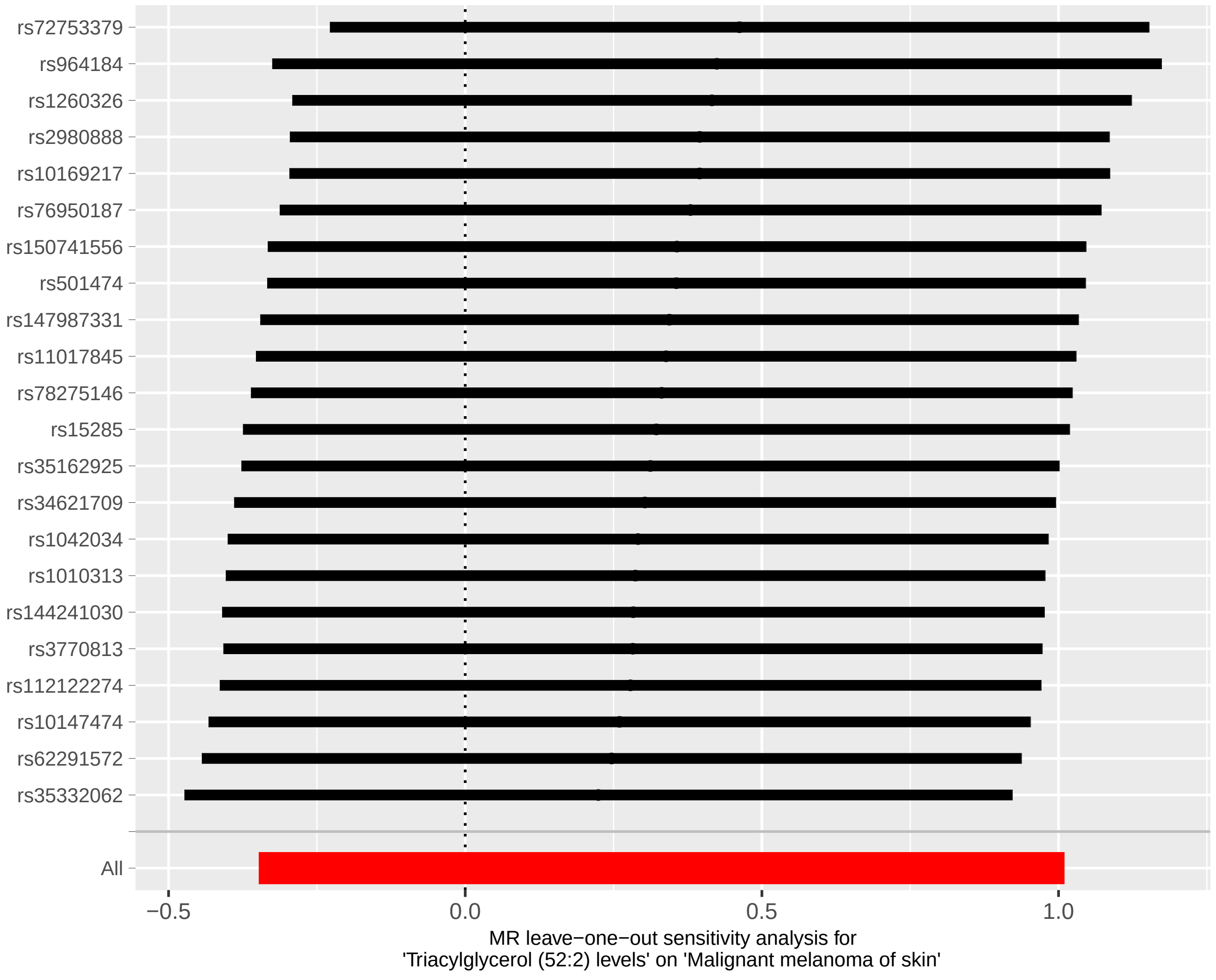
rs72753379
rs964184
rs1260326
rs2980888
rs10169217
rs76950187
rs150741556
rs501474
rs147987331
rs11017845
rs78275146
rs15285
rs35162925
rs34621709
rs1042034
rs1010313
rs144241030
rs3770813
rs112122274
rs10147474
rs62291572
rs35332062
All
−0.5
0.0
0.5
1.0
MR leave−one−out sensitivity analysis for
'Triacylglycerol (52:2) levels' on 'Malignant melanoma of skin'

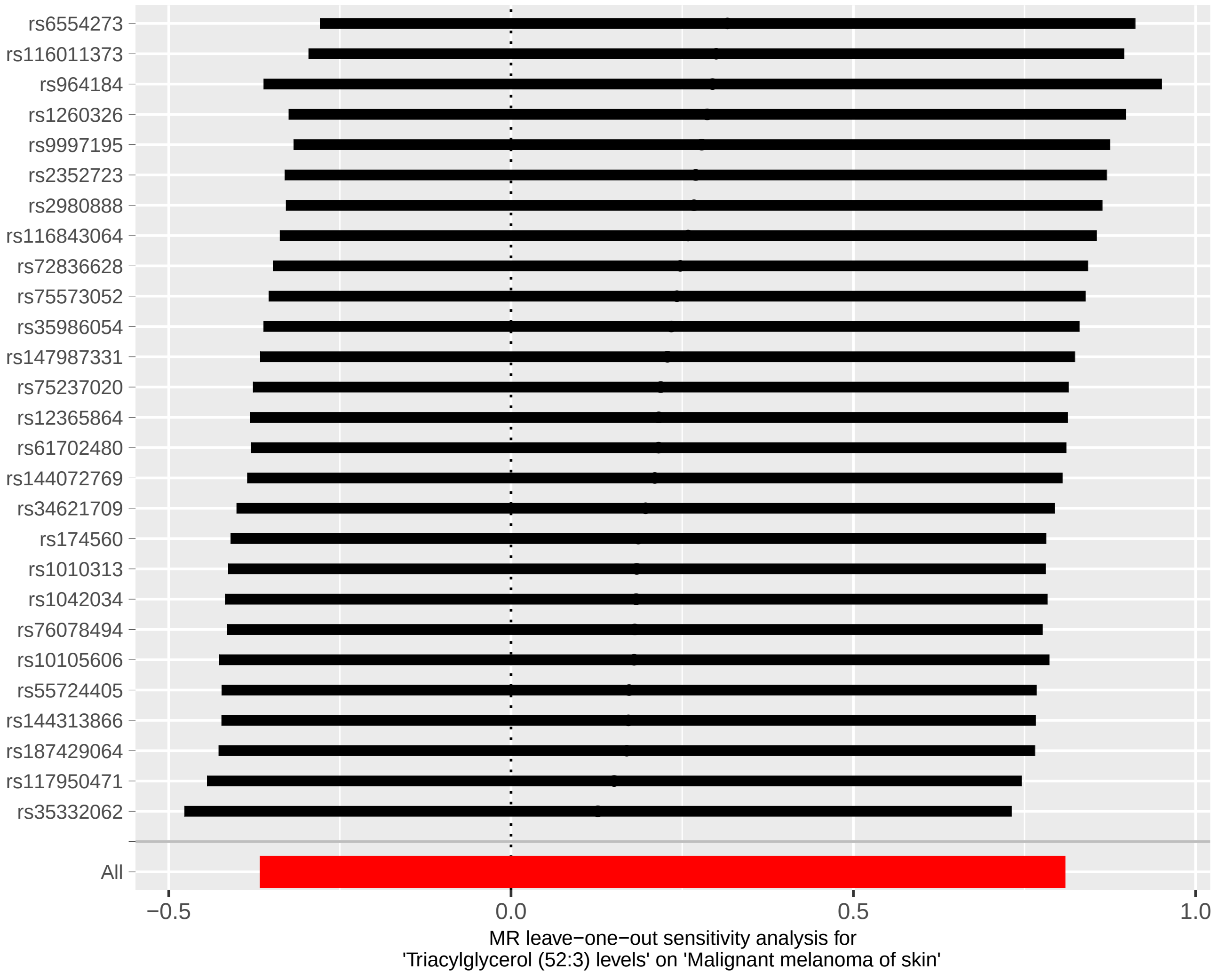
rs6554273
rs116011373
rs964184
rs1260326
rs9997195
rs2352723
rs2980888
rs116843064
rs72836628
rs75573052
rs35986054
rs147987331
rs75237020
rs12365864
rs61702480
rs144072769
rs34621709
rs174560
rs1010313
rs1042034
rs76078494
rs10105606
rs55724405
rs144313866
rs187429064
rs117950471
rs35332062
All
−0.5
0.0
0.5
1.0
MR leave−one−out sensitivity analysis for
'Triacylglycerol (52:3) levels' on 'Malignant melanoma of skin'

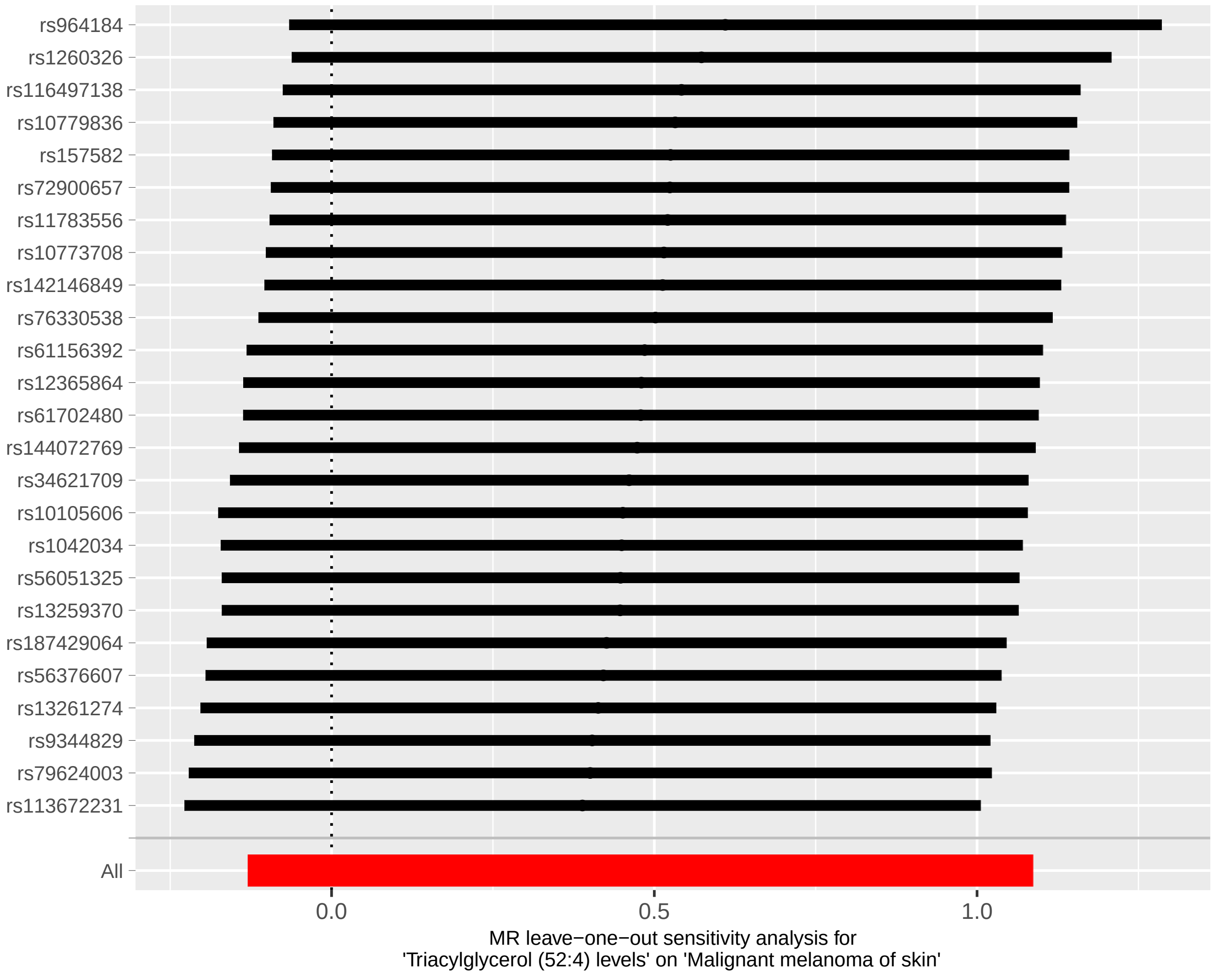
rs964184
rs1260326
rs116497138
rs10779836
rs157582
rs72900657
rs11783556
rs10773708
rs142146849
rs76330538
rs61156392
rs12365864
rs61702480
rs144072769
rs34621709
rs10105606
rs1042034
rs56051325
rs13259370
rs187429064
rs56376607
rs13261274
rs9344829
rs79624003
rs113672231
All
0.0
0.5
1.0
MR leave−one−out sensitivity analysis for
'Triacylglycerol (52:4) levels' on 'Malignant melanoma of skin'

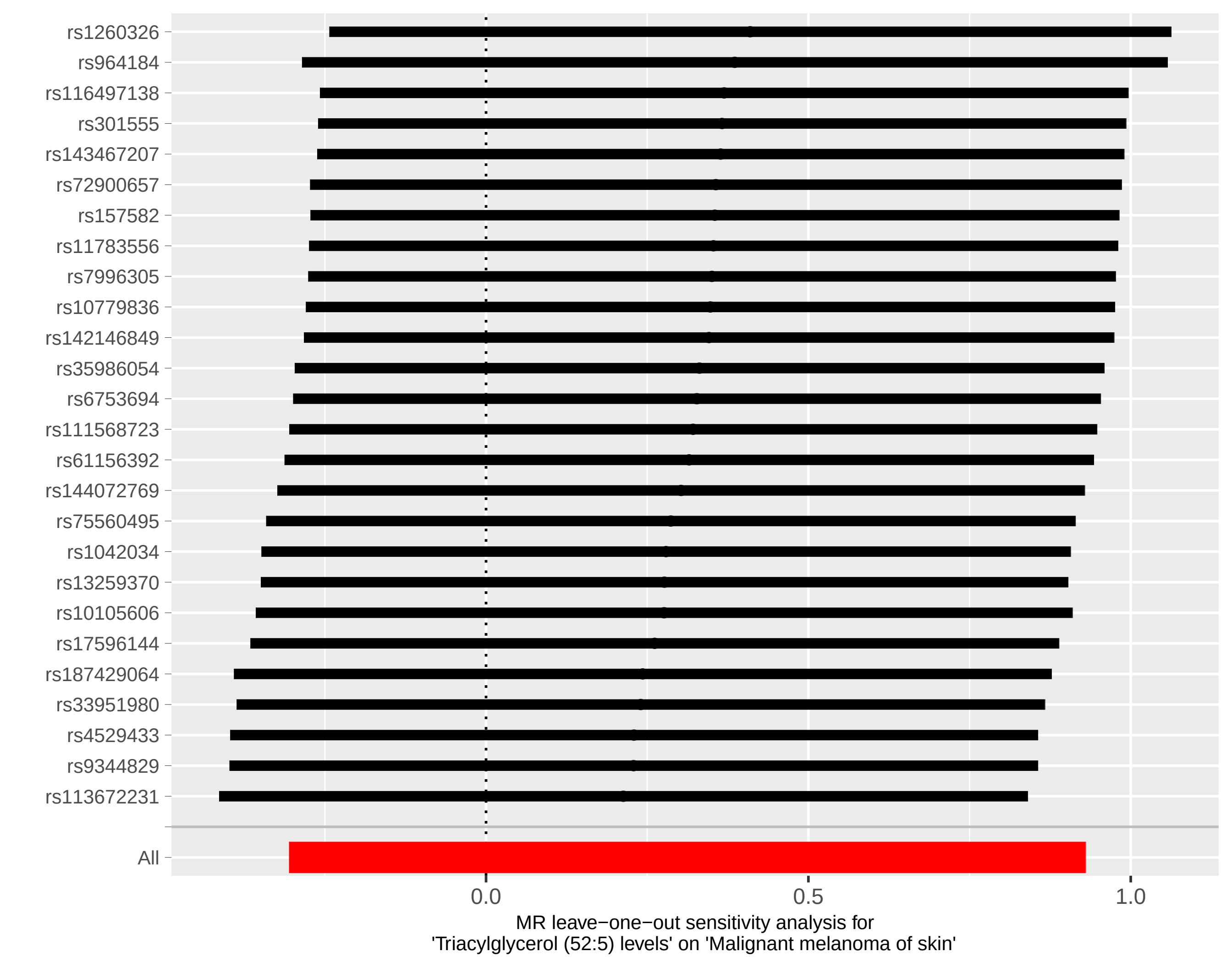

rs1260326
rs964184
rs116497138
rs301555
rs143467207
rs72900657
rs157582
rs11783556
rs7996305
rs10779836
rs142146849
rs35986054
rs6753694
rs111568723
rs61156392
rs144072769
rs75560495
rs1042034
rs13259370
rs10105606
rs17596144
rs187429064
rs33951980
rs4529433
rs9344829
rs113672231
All
0.0
0.5
1.0
MR leave−one−out sensitivity analysis for
'Triacylglycerol (52:5) levels' on 'Malignant melanoma of skin'

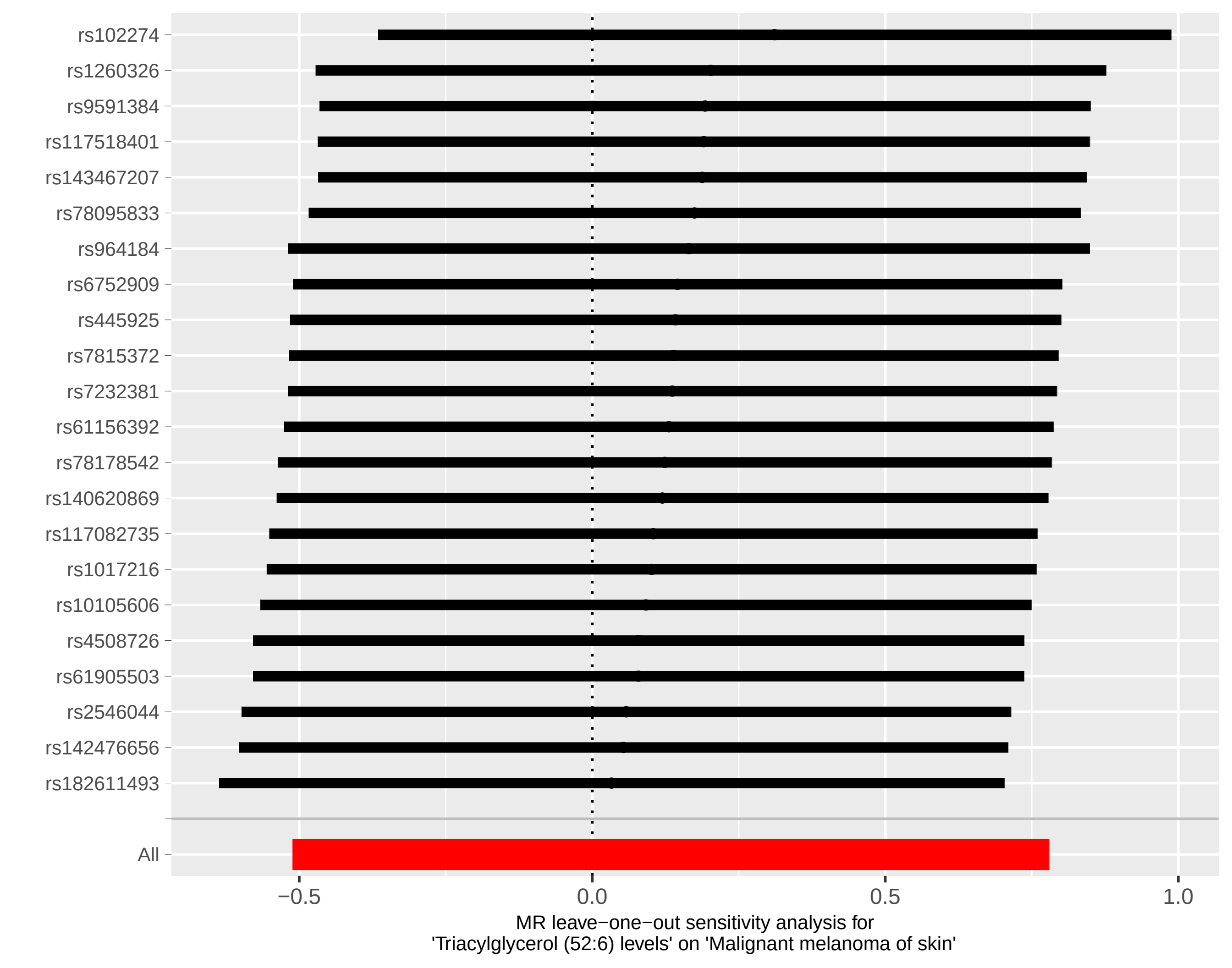
rs102274
rs1260326
rs9591384
rs117518401
rs143467207
rs78095833
rs964184
rs6752909
rs445925
rs7815372
rs7232381
rs61156392
rs78178542
rs140620869
rs117082735
rs1017216
rs10105606
rs4508726
rs61905503
rs2546044
rs142476656
rs182611493
All
−0.5
0.0
0.5
1.0
MR leave−one−out sensitivity analysis for
'Triacylglycerol (52:6) levels' on 'Malignant melanoma of skin'

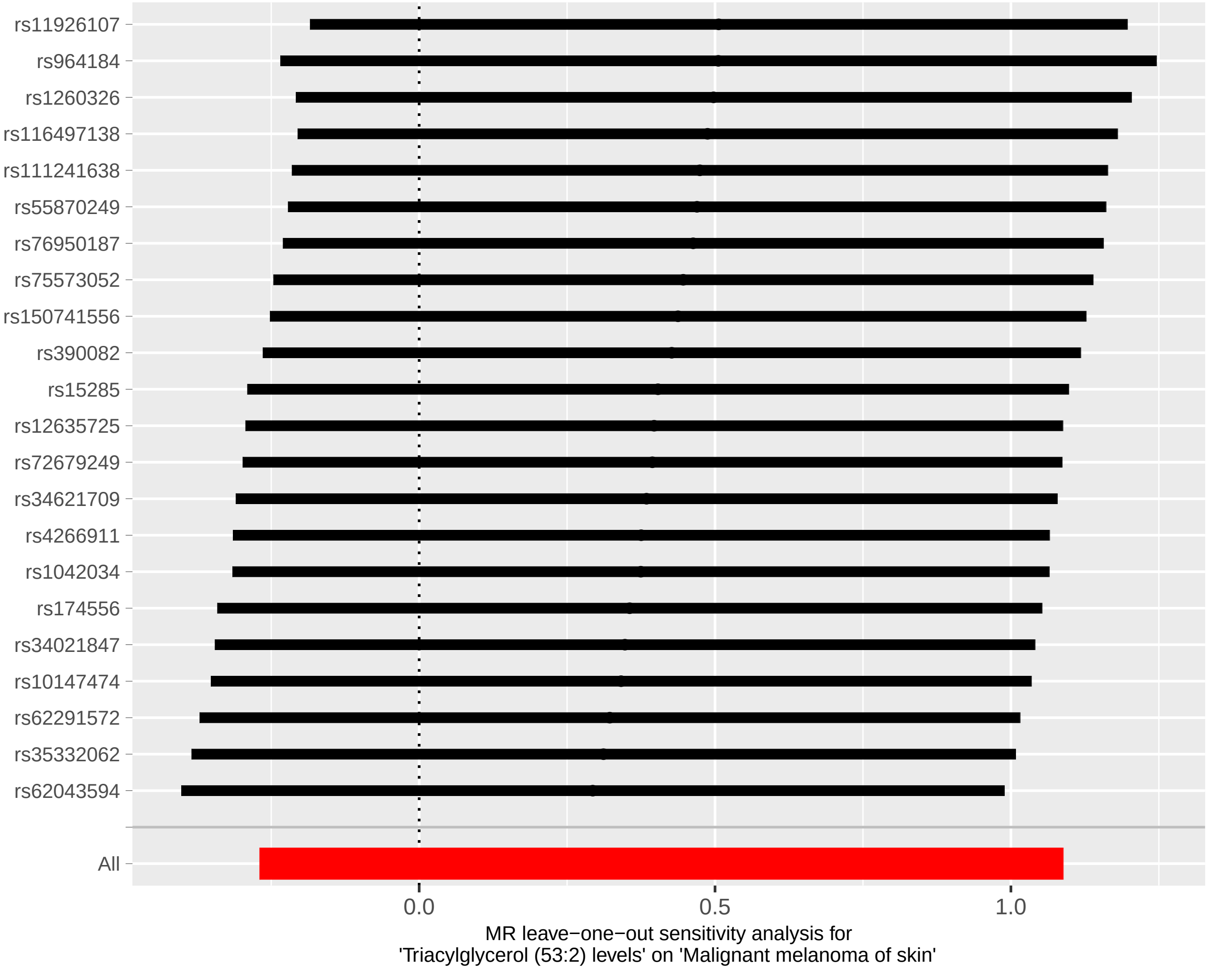
rs11926107
rs964184
rs1260326
rs116497138
rs111241638
rs55870249
rs76950187
rs75573052
rs150741556
rs390082
rs15285
rs12635725
rs72679249
rs34621709
rs4266911
rs1042034
rs174556
rs34021847
rs10147474
rs62291572
rs35332062
rs62043594
All
0.0
0.5
1.0
MR leave−one−out sensitivity analysis for
'Triacylglycerol (53:2) levels' on 'Malignant melanoma of skin'

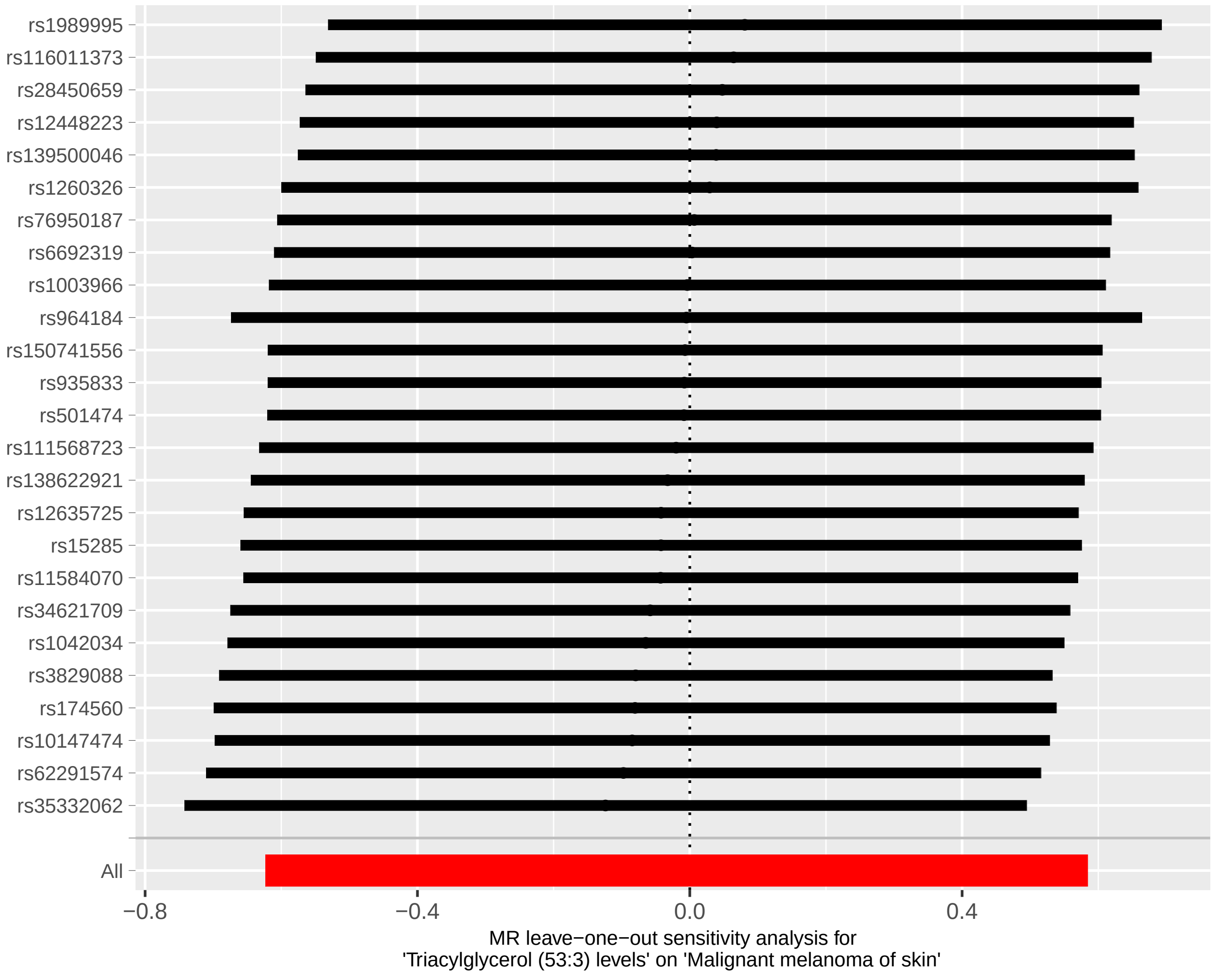
rs1989995
rs116011373
rs28450659
rs12448223
rs139500046
rs1260326
rs76950187
rs6692319
rs1003966
rs964184
rs150741556
rs935833
rs501474
rs111568723
rs138622921
rs12635725
rs15285
rs11584070
rs34621709
rs1042034
rs3829088
rs174560
rs10147474
rs62291574
rs35332062
All
−0.8
−0.4
0.0
0.4
MR leave−one−out sensitivity analysis for
'Triacylglycerol (53:3) levels' on 'Malignant melanoma of skin'

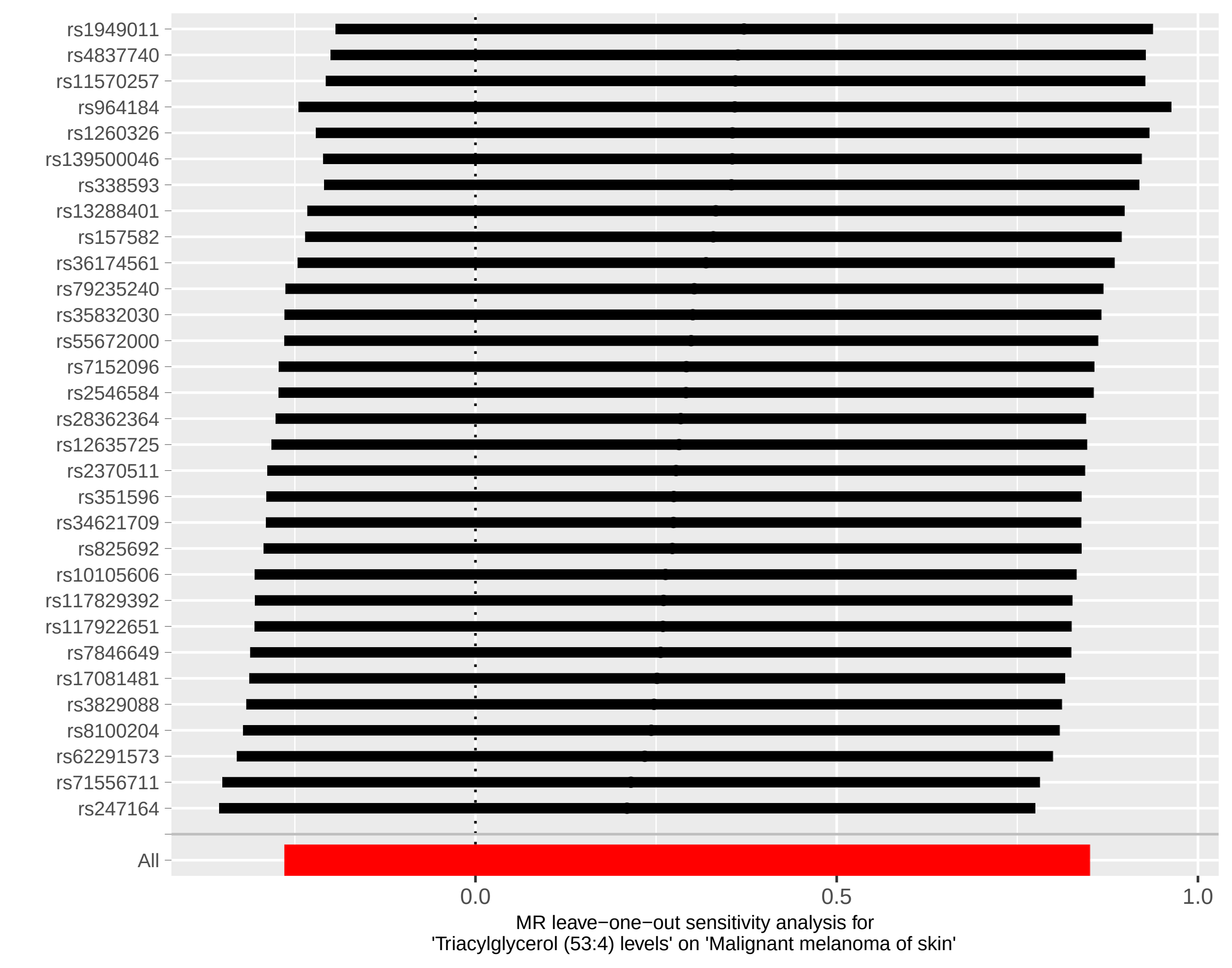
rs1949011
rs4837740
rs11570257
rs964184
rs1260326
rs139500046
rs338593
rs13288401
rs157582
rs36174561
rs79235240
rs35832030
rs55672000
rs7152096
rs2546584
rs28362364
rs12635725
rs2370511
rs351596
rs34621709
rs825692
rs10105606
rs117829392
rs117922651
rs7846649
rs17081481
rs3829088
rs8100204
rs62291573
rs71556711
rs247164
All
0.0
0.5
1.0
MR leave−one−out sensitivity analysis for
'Triacylglycerol (53:4) levels' on 'Malignant melanoma of skin'

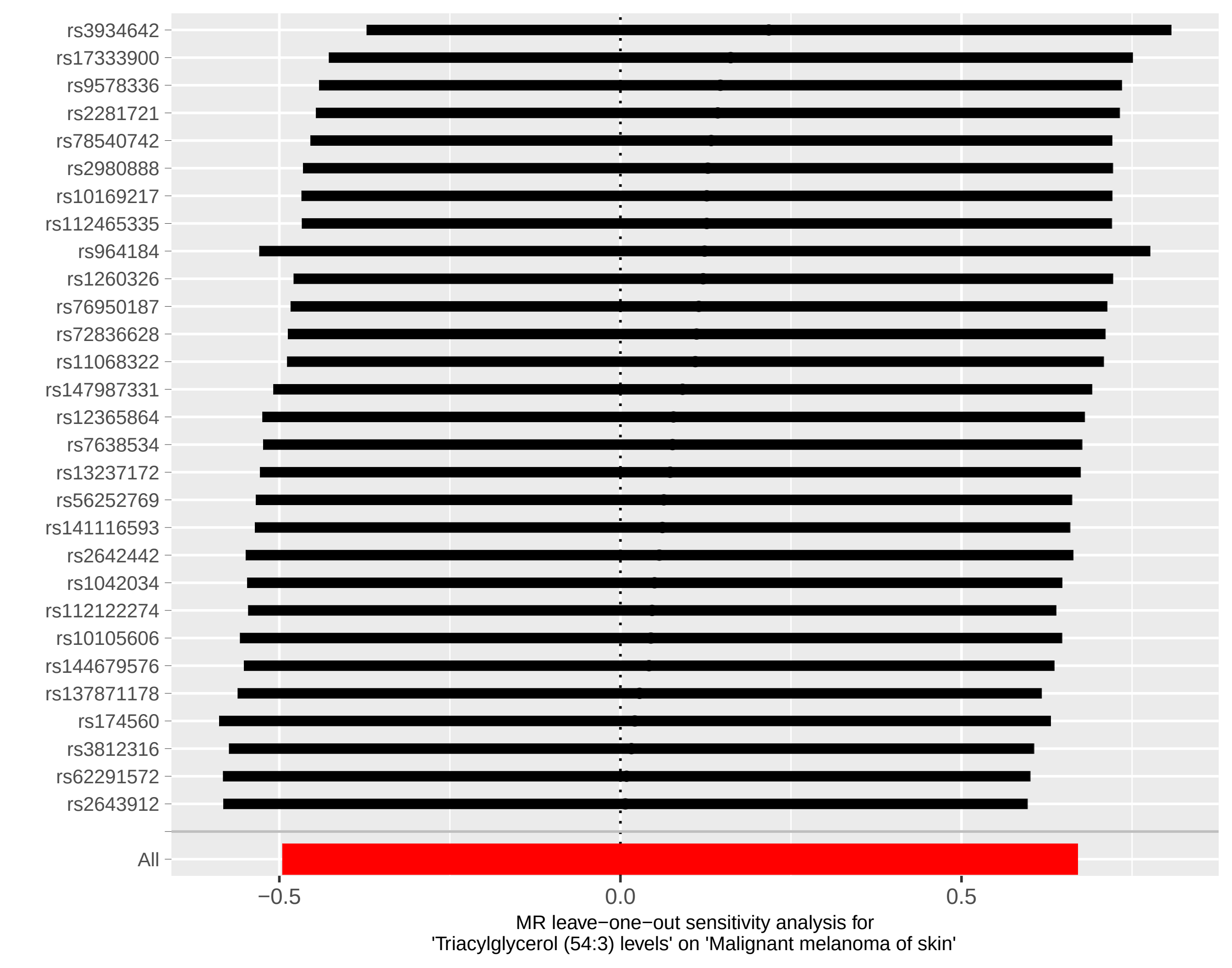

rs3934642
rs17333900
rs9578336
rs2281721
rs78540742
rs2980888
rs10169217
rs112465335
rs964184
rs1260326
rs76950187
rs72836628
rs11068322
rs147987331
rs12365864
rs7638534
rs13237172
rs56252769
rs141116593
rs2642442
rs1042034
rs112122274
rs10105606
rs144679576
rs137871178
rs174560
rs3812316
rs62291572
rs2643912
All
−0.5
0.0
0.5
MR leave−one−out sensitivity analysis for
'Triacylglycerol (54:3) levels' on 'Malignant melanoma of skin'

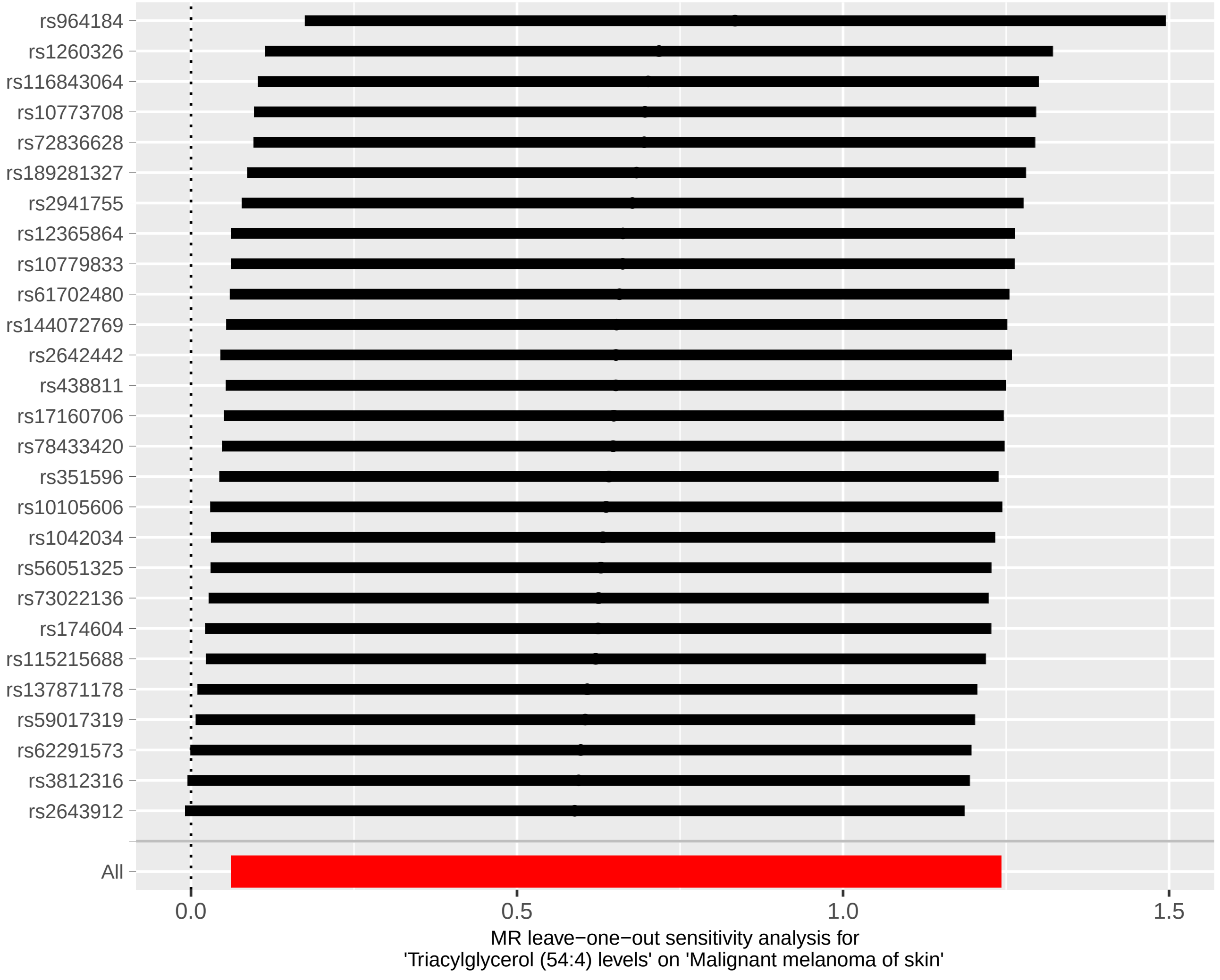
rs964184
rs1260326
rs116843064
rs10773708
rs72836628
rs189281327
rs2941755
rs12365864
rs10779833
rs61702480
rs144072769
rs2642442
rs438811
rs17160706
rs78433420
rs351596
rs10105606
rs1042034
rs56051325
rs73022136
rs174604
rs115215688
rs137871178
rs59017319
rs62291573
rs3812316
rs2643912
All
0.0
0.5
1.0
1.5
MR leave−one−out sensitivity analysis for
'Triacylglycerol (54:4) levels' on 'Malignant melanoma of skin'

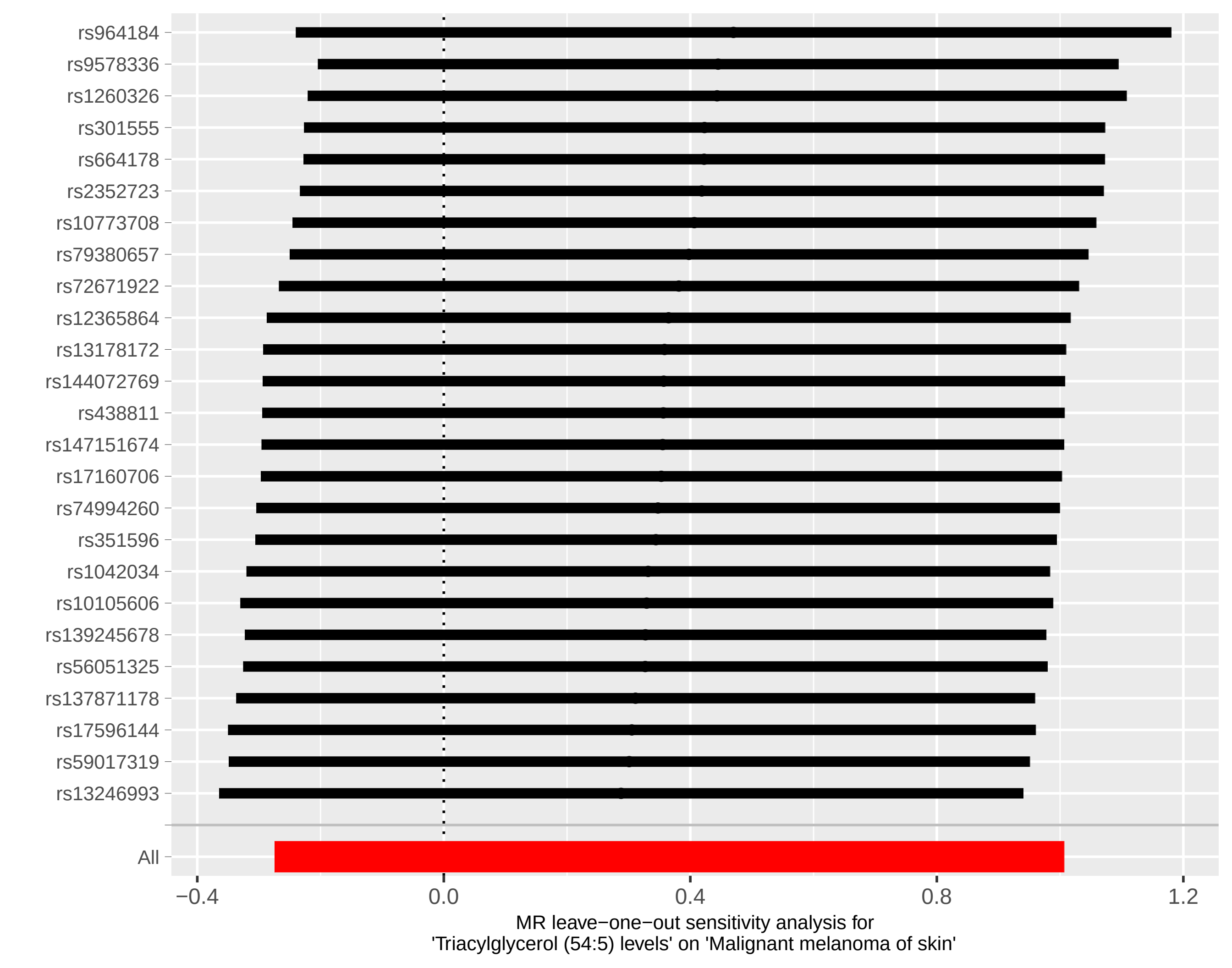
rs964184
rs9578336
rs1260326
rs301555
rs664178
rs2352723
rs10773708
rs79380657
rs72671922
rs12365864
rs13178172
rs144072769
rs438811
rs147151674
rs17160706
rs74994260
rs351596
rs1042034
rs10105606
rs139245678
rs56051325
rs137871178
rs17596144
rs59017319
rs13246993
All
−0.4
0.0
0.4
0.8
1.2
MR leave−one−out sensitivity analysis for
'Triacylglycerol (54:5) levels' on 'Malignant melanoma of skin'

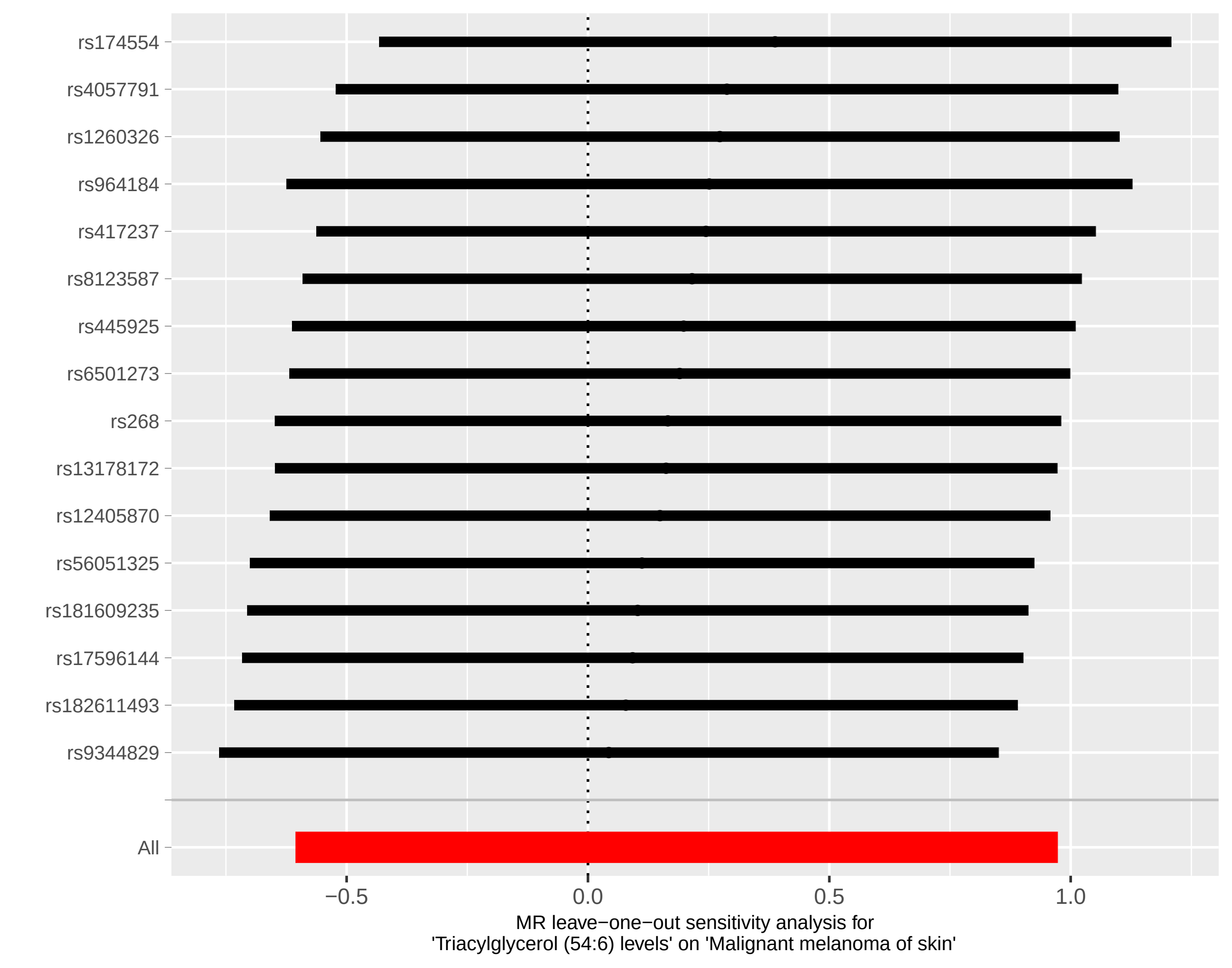

rs174554
rs4057791
rs1260326
rs964184
rs417237
rs8123587
rs445925
rs6501273
rs268
rs13178172
rs12405870
rs56051325
rs181609235
rs17596144
rs182611493
rs9344829
All
−0.5
0.0
0.5
1.0
MR leave−one−out sensitivity analysis for
'Triacylglycerol (54:6) levels' on 'Malignant melanoma of skin'

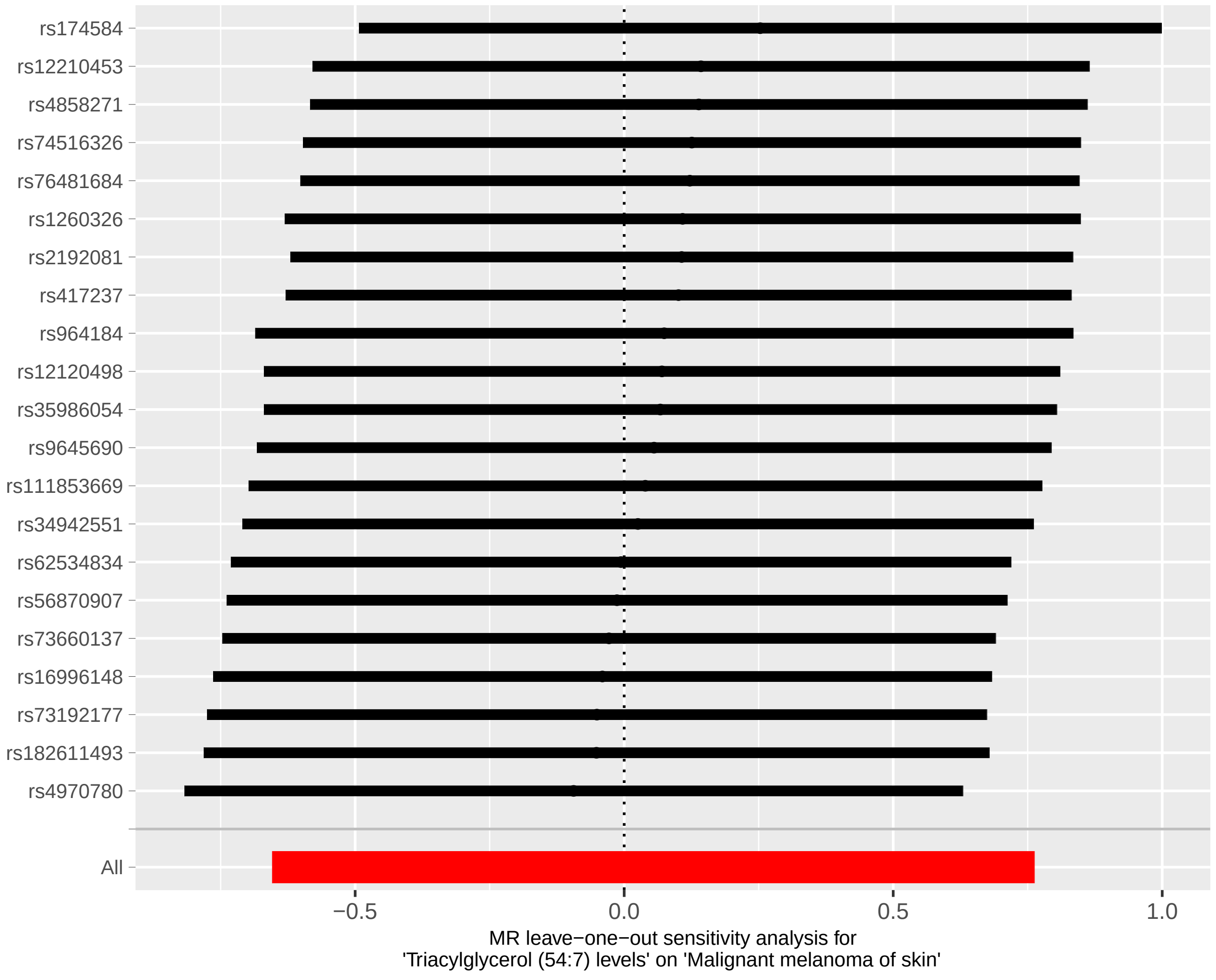
rs174584
rs12210453
rs4858271
rs74516326
rs76481684
rs1260326
rs2192081
rs417237
rs964184
rs12120498
rs35986054
rs9645690
rs111853669
rs34942551
rs62534834
rs56870907
rs73660137
rs16996148
rs73192177
rs182611493
rs4970780
All
−0.5
0.0
0.5
1.0
MR leave−one−out sensitivity analysis for
'Triacylglycerol (54:7) levels' on 'Malignant melanoma of skin'

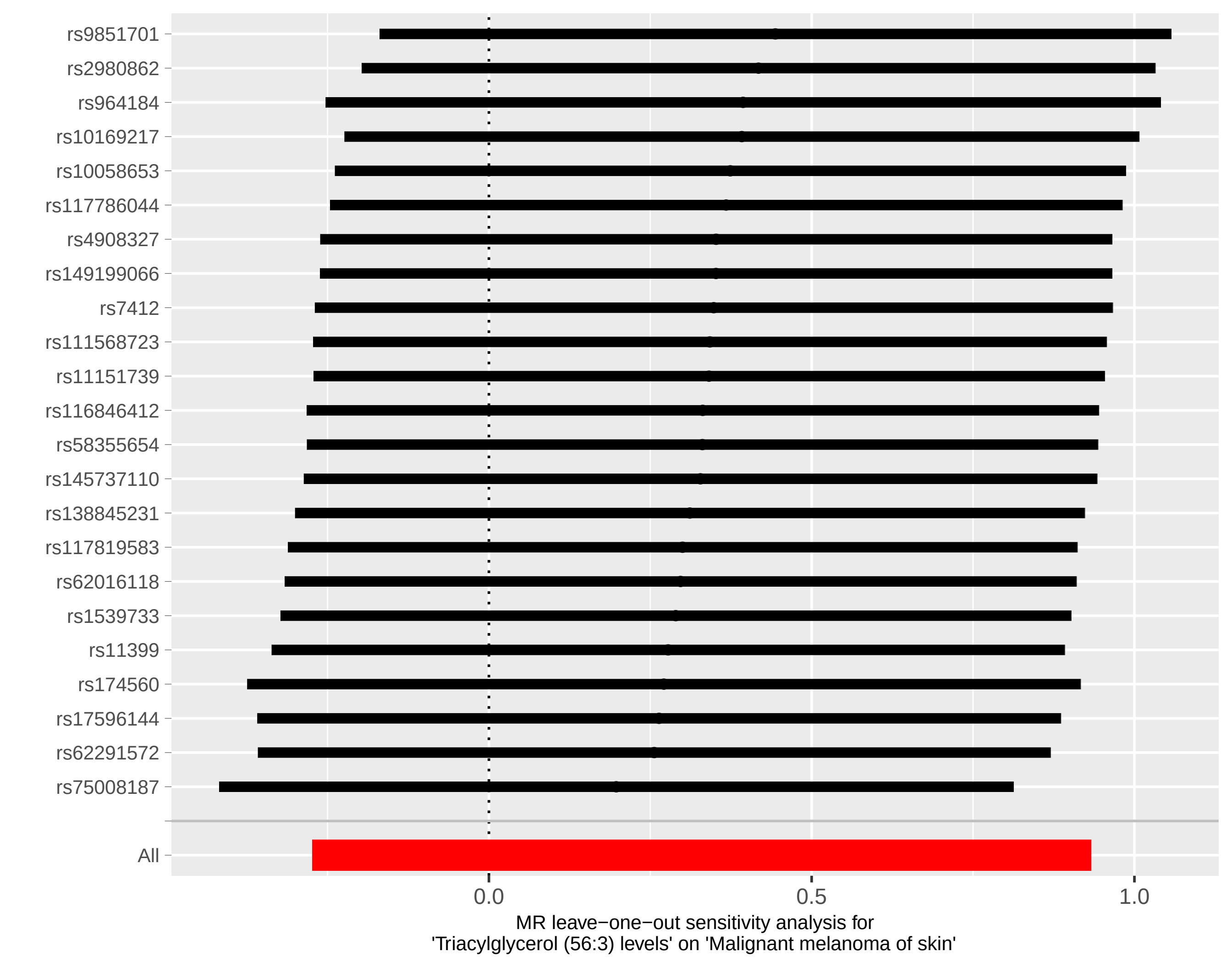
rs9851701
rs2980862
rs964184
rs10169217
rs10058653
rs117786044
rs4908327
rs149199066
rs7412
rs111568723
rs11151739
rs116846412
rs58355654
rs145737110
rs138845231
rs117819583
rs62016118
rs1539733
rs11399
rs174560
rs17596144
rs62291572
rs75008187
All
0.0
0.5
1.0
MR leave−one−out sensitivity analysis for
'Triacylglycerol (56:3) levels' on 'Malignant melanoma of skin'

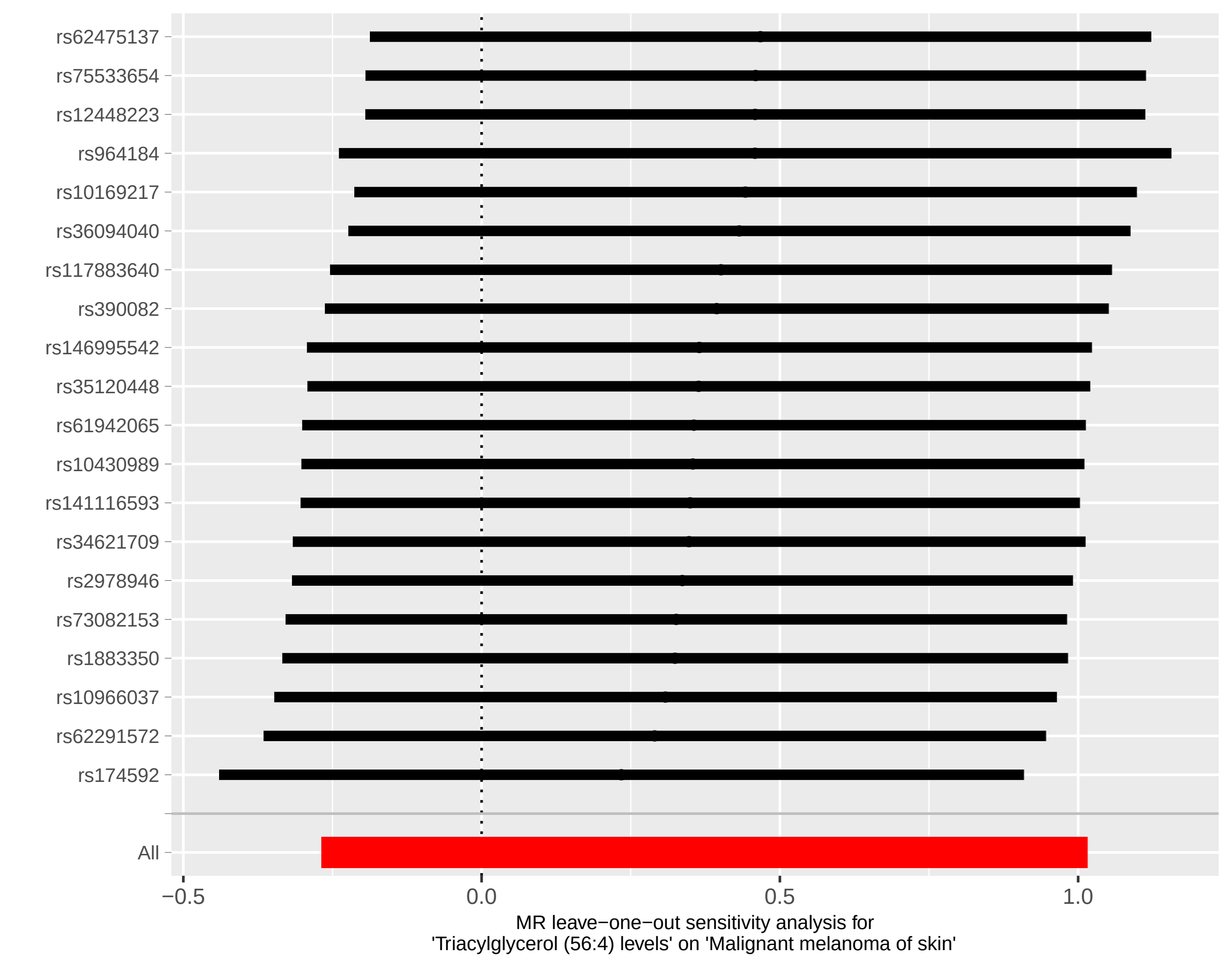
rs62475137
rs75533654
rs12448223
rs964184
rs10169217
rs36094040
rs117883640
rs390082
rs146995542
rs35120448
rs61942065
rs10430989
rs141116593
rs34621709
rs2978946
rs73082153
rs1883350
rs10966037
rs62291572
rs174592
All
−0.5
0.0
0.5
1.0
MR leave−one−out sensitivity analysis for
'Triacylglycerol (56:4) levels' on 'Malignant melanoma of skin'

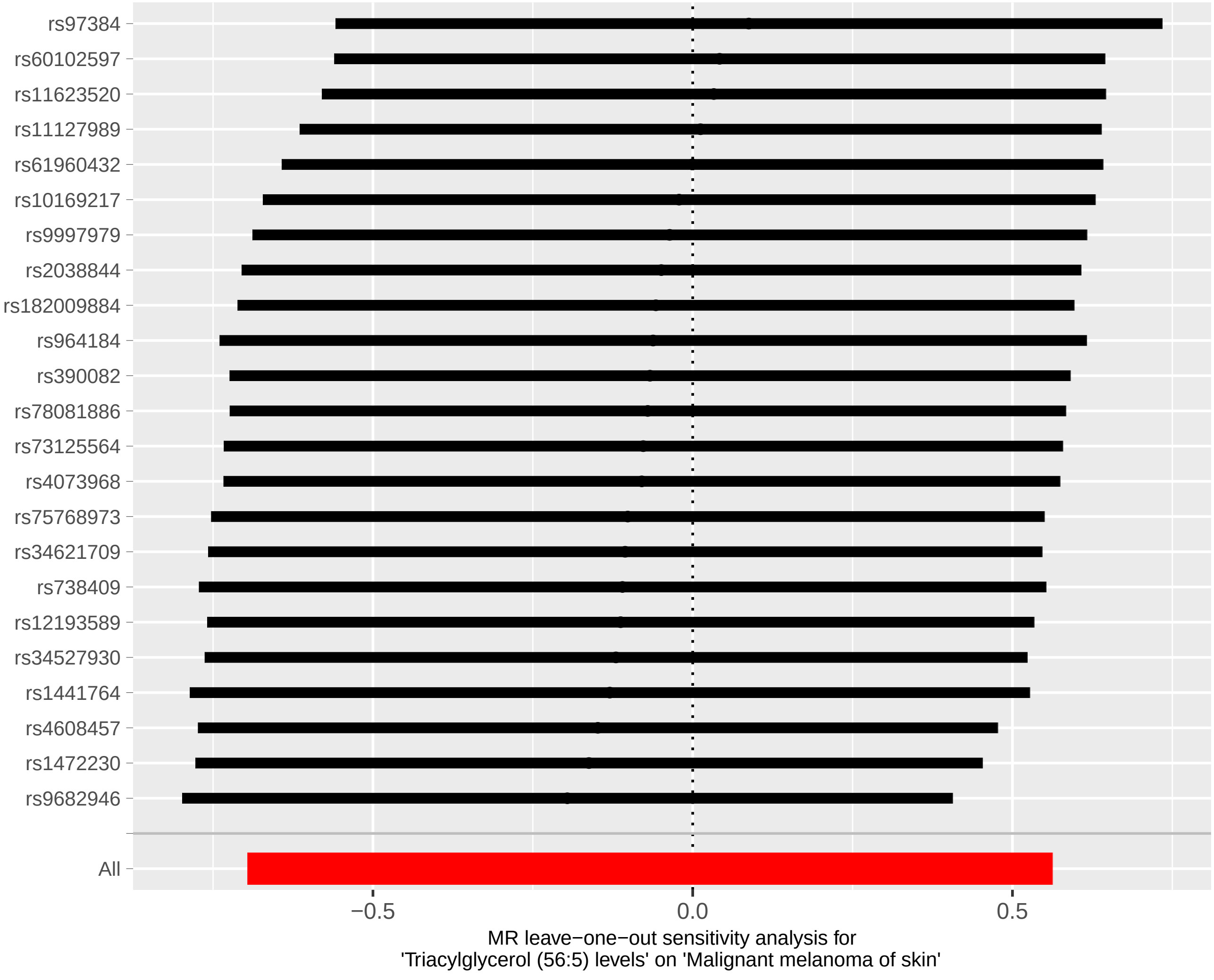
rs97384
rs60102597
rs11623520
rs11127989
rs61960432
rs10169217
rs9997979
rs2038844
rs182009884
rs964184
rs390082
rs78081886
rs73125564
rs4073968
rs75768973
rs34621709
rs738409
rs12193589
rs34527930
rs1441764
rs4608457
rs1472230
rs9682946
All
−0.5
0.0
0.5
MR leave−one−out sensitivity analysis for
'Triacylglycerol (56:5) levels' on 'Malignant melanoma of skin'

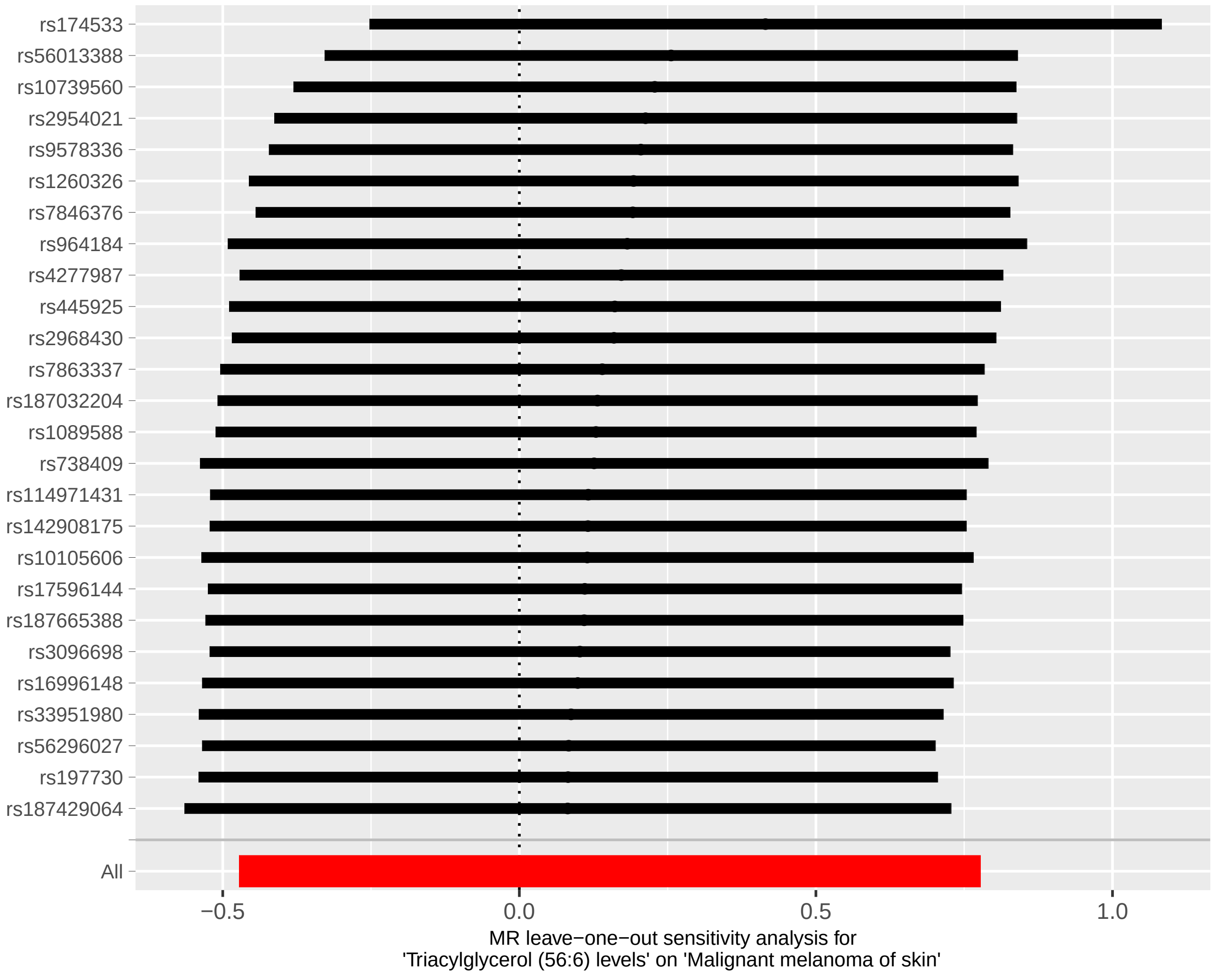

rs174533
rs56013388
rs10739560
rs2954021
rs9578336
rs1260326
rs7846376
rs964184
rs4277987
rs445925
rs2968430
rs7863337
rs187032204
rs1089588
rs738409
rs114971431
rs142908175
rs10105606
rs17596144
rs187665388
rs3096698
rs16996148
rs33951980
rs56296027
rs197730
rs187429064
All
−0.5
0.0
0.5
1.0
MR leave−one−out sensitivity analysis for
'Triacylglycerol (56:6) levels' on 'Malignant melanoma of skin'

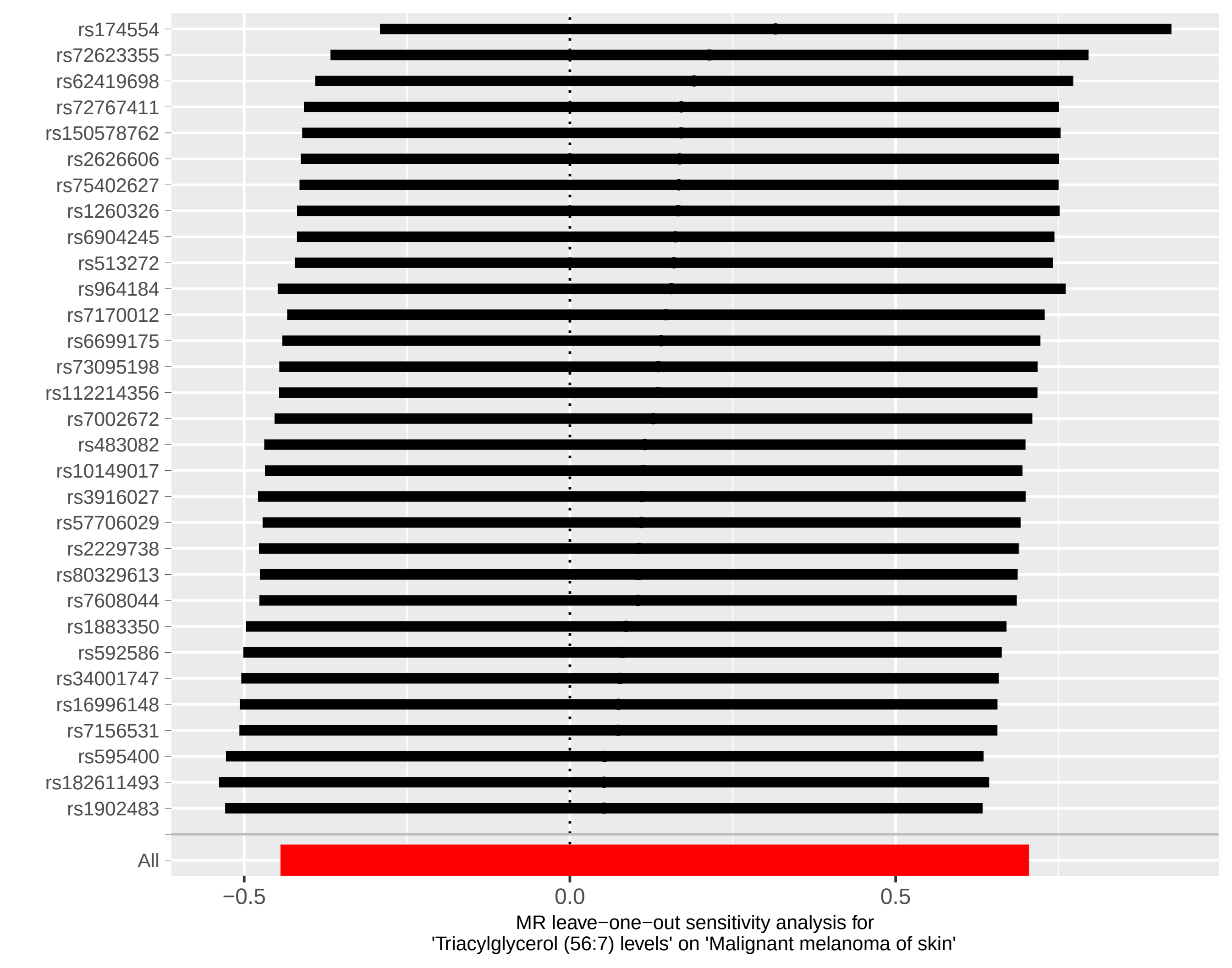

rs174554
rs72623355
rs62419698
rs72767411
rs150578762
rs2626606
rs75402627
rs1260326
rs6904245
rs513272
rs964184
rs7170012
rs6699175
rs73095198
rs112214356
rs7002672
rs483082
rs10149017
rs3916027
rs57706029
rs2229738
rs80329613
rs7608044
rs1883350
rs592586
rs34001747
rs16996148
rs7156531
rs595400
rs182611493
rs1902483
All
−0.5
0.0
0.5
MR leave−one−out sensitivity analysis for
'Triacylglycerol (56:7) levels' on 'Malignant melanoma of skin'

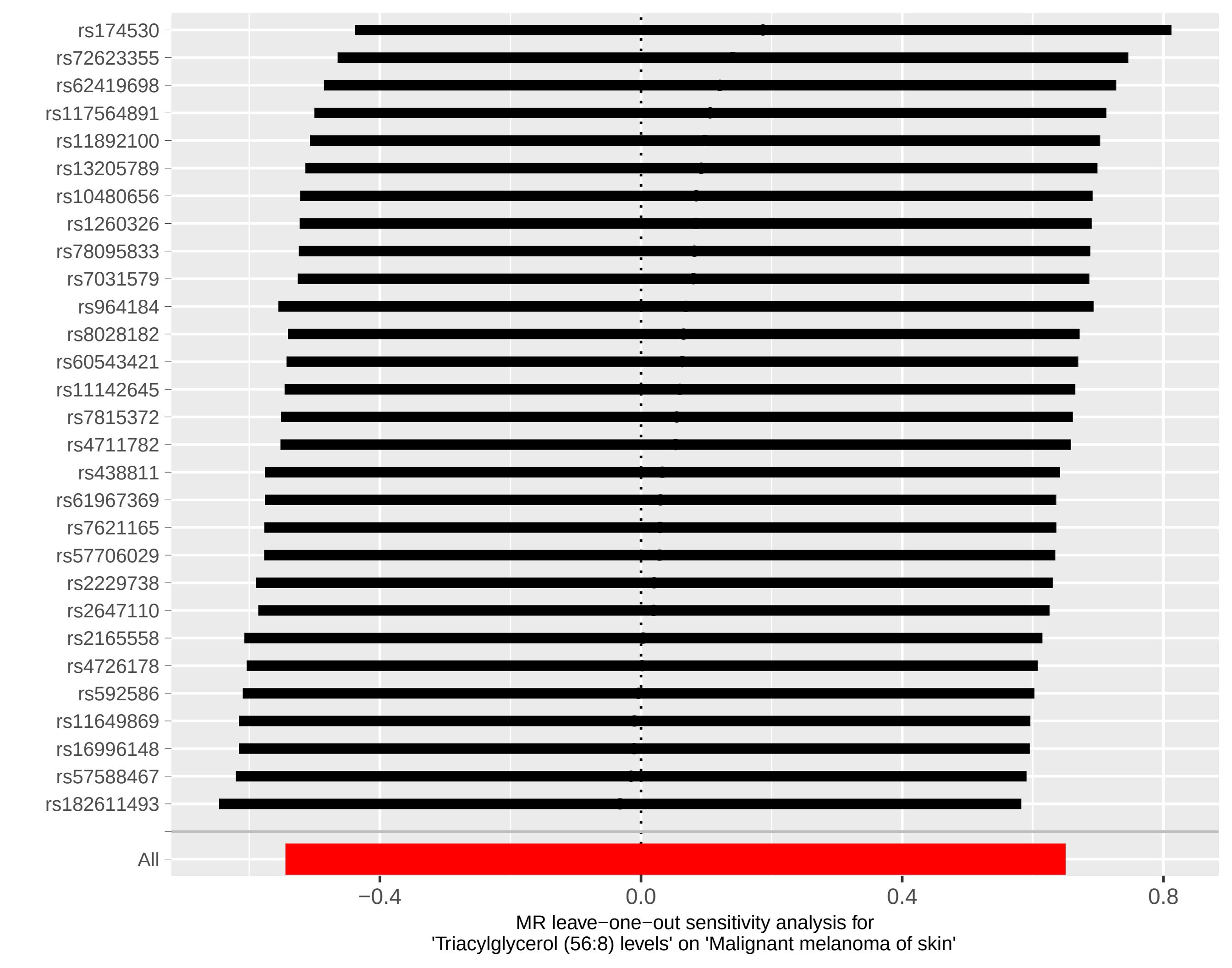
rs174530
rs72623355
rs62419698
rs117564891
rs11892100
rs13205789
rs10480656
rs1260326
rs78095833
rs7031579
rs964184
rs8028182
rs60543421
rs11142645
rs7815372
rs4711782
rs438811
rs61967369
rs7621165
rs57706029
rs2229738
rs2647110
rs2165558
rs4726178
rs592586
rs11649869
rs16996148
rs57588467
rs182611493
All
−0.4
0.0
0.4
0.8
MR leave−one−out sensitivity analysis for
'Triacylglycerol (56:8) levels' on 'Malignant melanoma of skin'

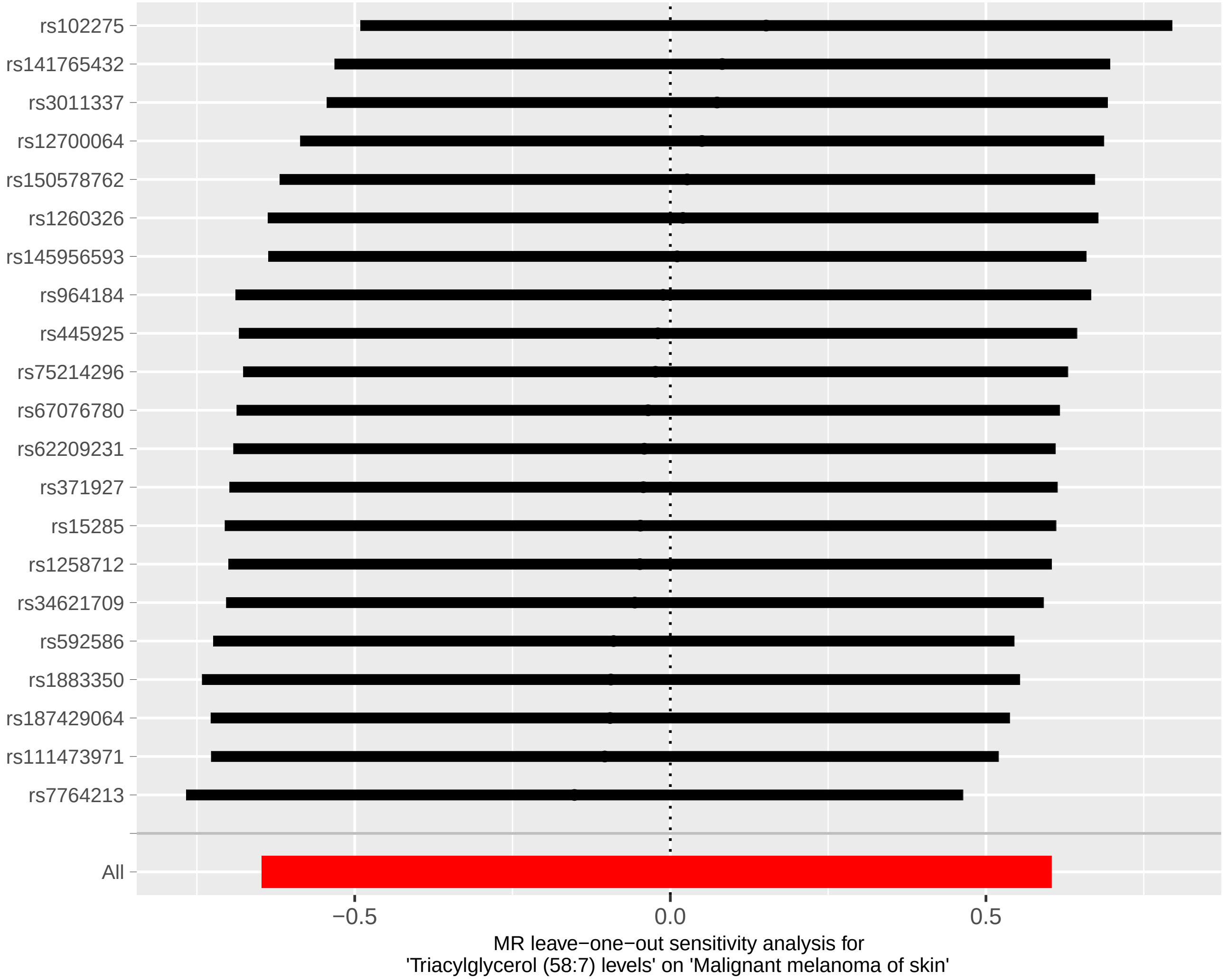
rs102275
rs141765432
rs3011337
rs12700064
rs150578762
rs1260326
rs145956593
rs964184
rs445925
rs75214296
rs67076780
rs62209231
rs371927
rs15285
rs1258712
rs34621709
rs592586
rs1883350
rs187429064
rs111473971
rs7764213
All
−0.5
0.0
0.5
MR leave−one−out sensitivity analysis for
'Triacylglycerol (58:7) levels' on 'Malignant melanoma of skin'

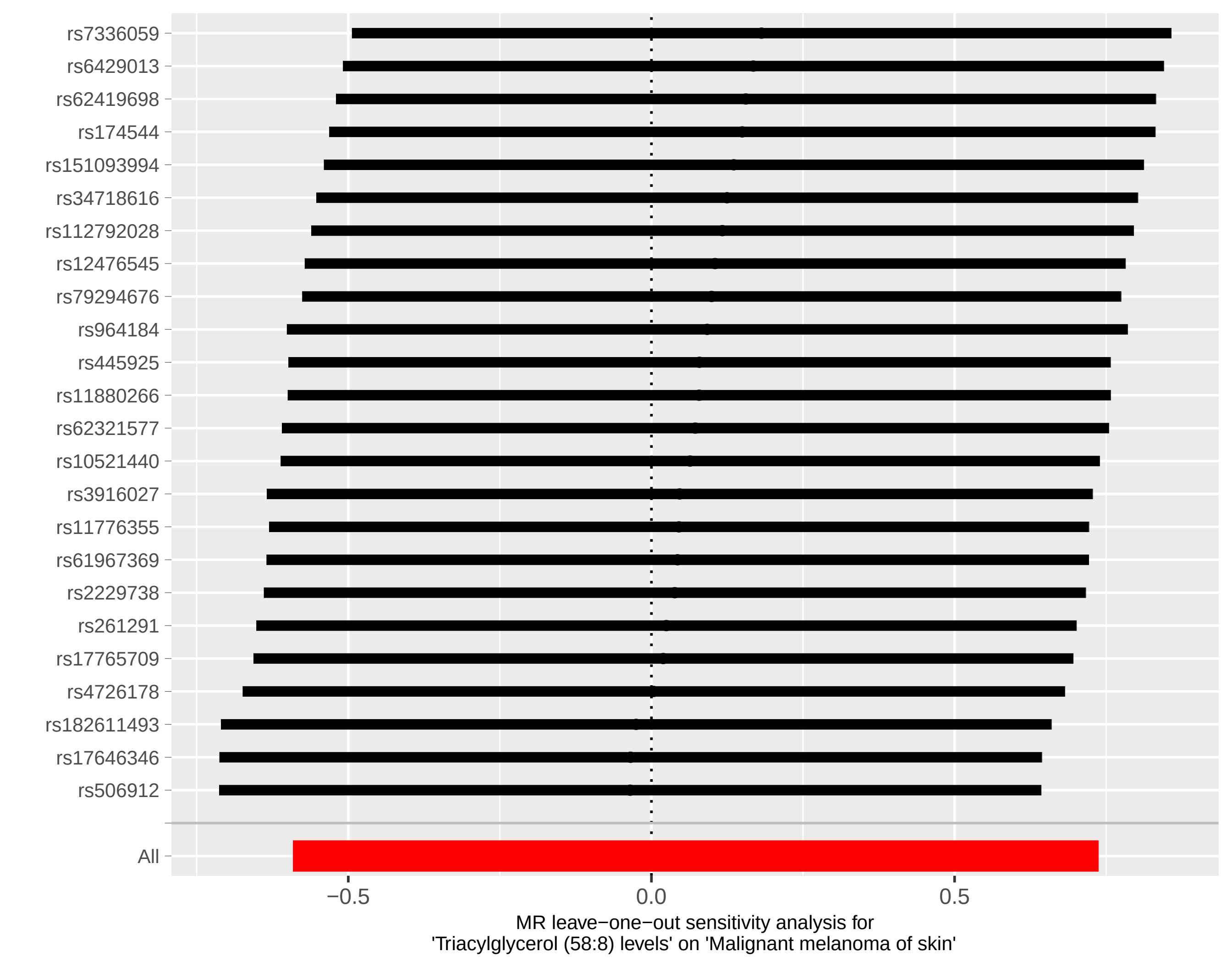
rs7336059
rs6429013
rs62419698
rs174544
rs151093994
rs34718616
rs112792028
rs12476545
rs79294676
rs964184
rs445925
rs11880266
rs62321577
rs10521440
rs3916027
rs11776355
rs61967369
rs2229738
rs261291
rs17765709
rs4726178
rs182611493
rs17646346
rs506912
All
−0.5
0.0
0.5
MR leave−one−out sensitivity analysis for
'Triacylglycerol (58:8) levels' on 'Malignant melanoma of skin'
