# Supplementary material for: Genetically predicted metabolites mediate the association between lipidome and malignant melanoma of skin
Source: Front Oncol. 2024 Sep 10;14:1430533. doi: 10.3389/fonc.2024.1430533 (PMC11419955; doi:10.3389/fonc.2024.1430533)

**Supplementary material 15**

**Visualization of the MR results(***Phosphatidylethanolamine (18:0_18:2) levels on 1-stearoyl-GPI (18:0) levels***)**

**Scatter plot Funnel plot**


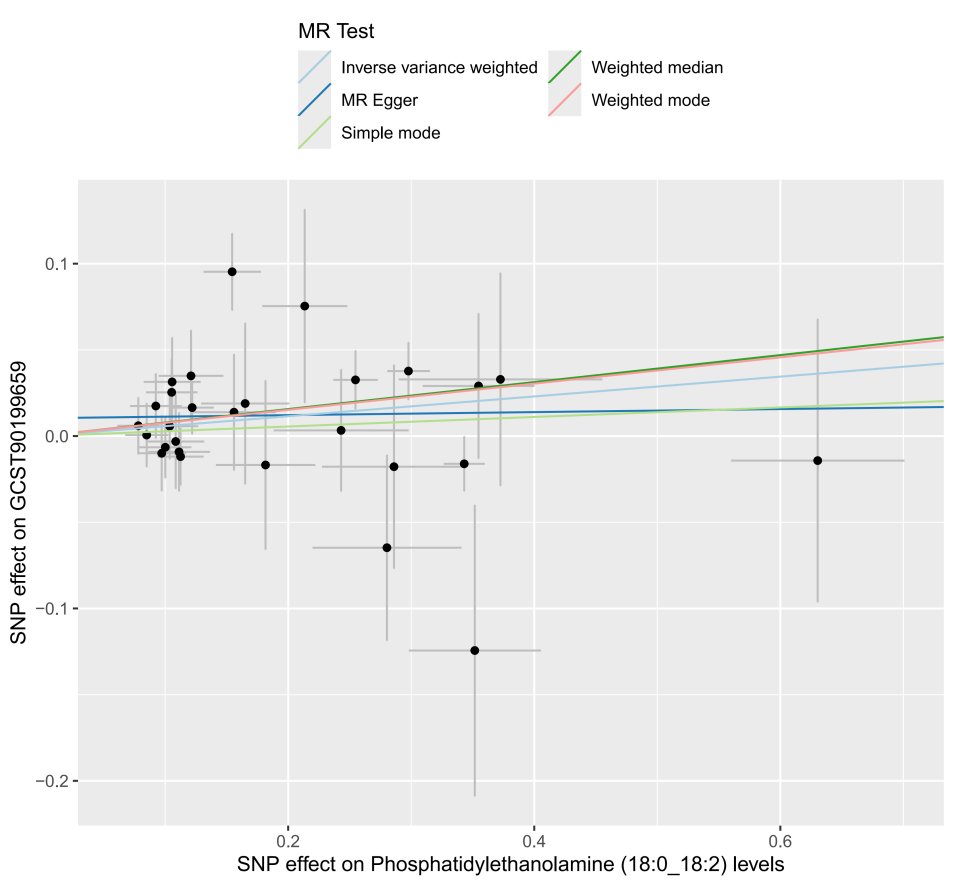

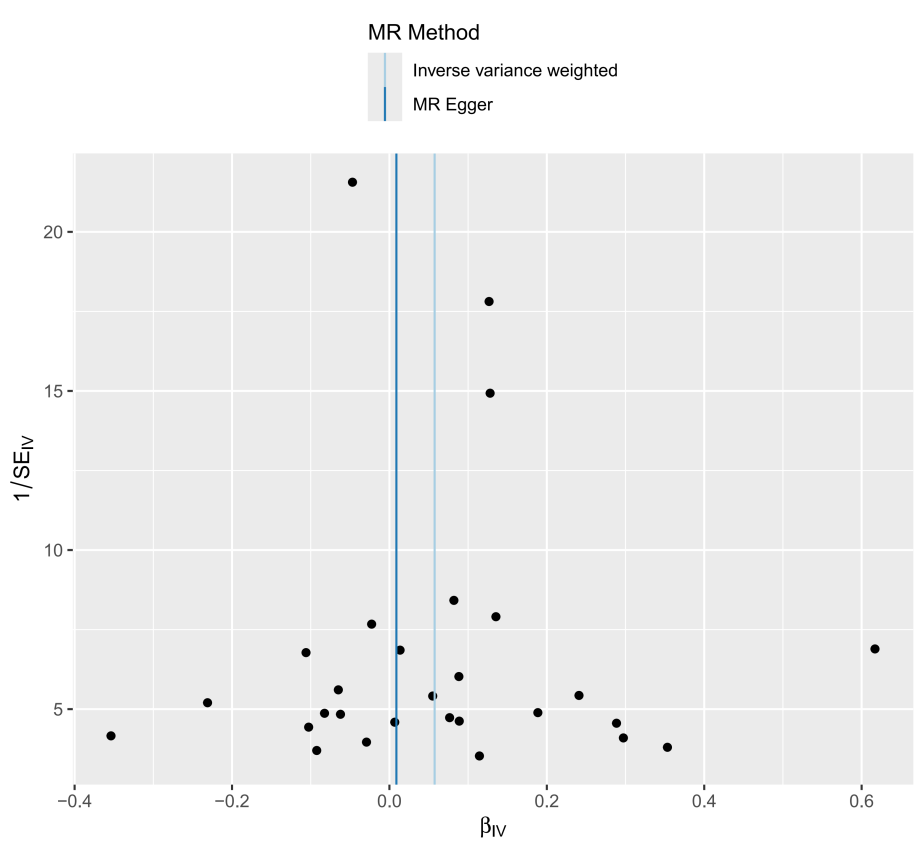


**Leave-one-out analysis Forest plot**


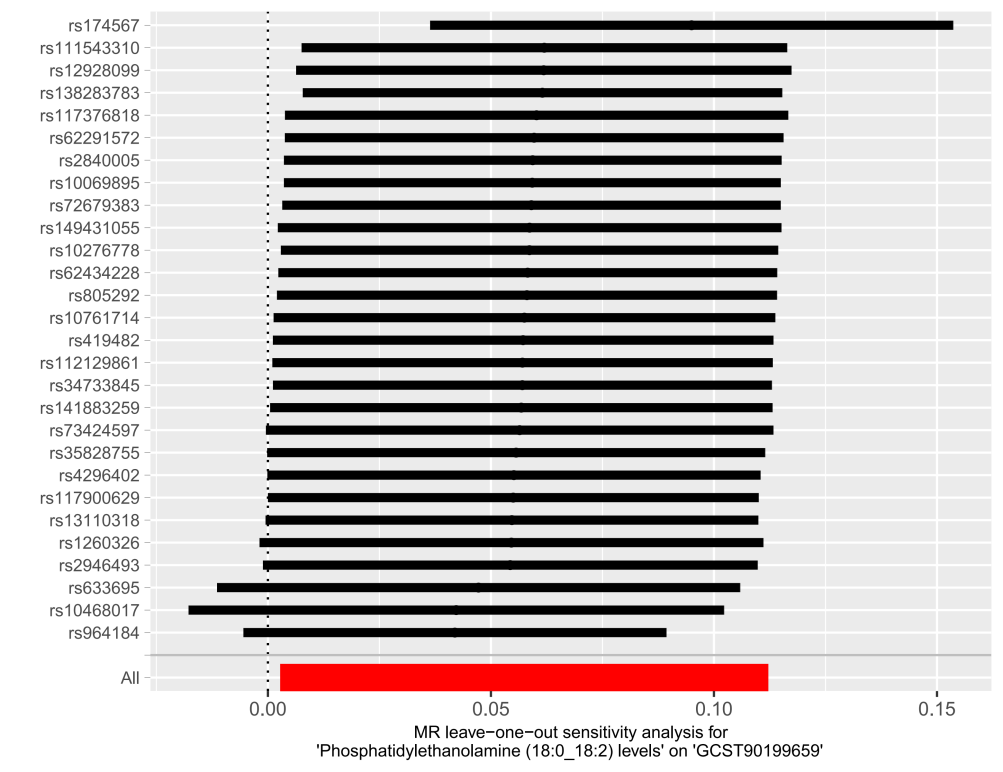

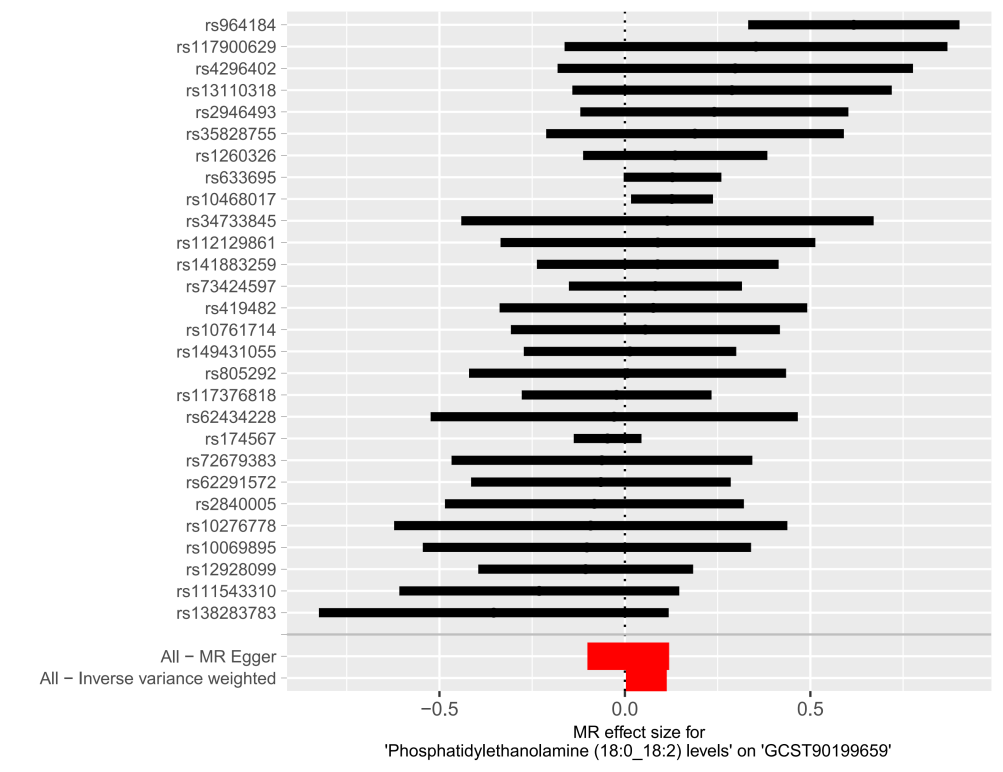

Supplement: Supplementary file 16 [file SupplementaryFile15.docx]
